# Effectiveness of Etoposide and Cisplatin vs Irinotecan and Cisplatin Therapy for Patients With Advanced Neuroendocrine Carcinoma of the Digestive System: The TOPIC-NEC Phase 3 Randomized Clinical Trial

**DOI:** 10.1001/jamaoncol.2022.3395

**Published:** 2022-08-18

**Authors:** Chigusa Morizane, Nozomu Machida, Yoshitaka Honma, Takuji Okusaka, Narikazu Boku, Ken Kato, Shogo Nomura, Nobuyoshi Hiraoka, Shigeki Sekine, Hirokazu Taniguchi, Naohiro Okano, Kensei Yamaguchi, Takuji Sato, Masafumi Ikeda, Nobumasa Mizuno, Masato Ozaka, Tomoko Kataoka, Makoto Ueno, Yuko Kitagawa, Masanori Terashima, Junji Furuse

**Affiliations:** 1 National Cancer Center Hospital, Tokyo, Japan; 2 Kanagawa Cancer Center, Yokohama, Japan; 3 Japan Clinical Oncology Group Data Center/Operations Office, National Cancer Center Hospital, Tokyo, Japan; 4 The University of Tokyo, Tokyo, Japan; 5 Kyorin University Faculty of Medicine, Mitaka, Japan; 6 Cancer Institute Hospital of Japanese Foundation for Cancer Research, Tokyo, Japan; 7 Kochi Health Sciences Center, Kochi, Japan; 8 National Cancer Center Hospital East, Chiba, Japan; 9 Aichi Cancer Center Hospital, Nagoya, Japan; 10 Keio University School of Medicine, Tokyo, Japan; 11 Shizuoka Cancer Center, Shizuoka, Japan

## Abstract

**Importance:**

Etoposide plus cisplatin (EP) and irinotecan plus cisplatin (IP) are commonly used as community standard regimens for advanced neuroendocrine carcinoma (NEC).

**Objective:**

To identify whether EP or IP is a more effective regimen in terms of overall survival (OS) in patients with advanced NEC of the digestive system.

**Design, Setting, and Participants:**

This open-label phase 3 randomized clinical trial enrolled chemotherapy-naive patients aged 20 to 75 years who had recurrent or unresectable NEC (according to the 2010 World Health Organization classification system) arising from the gastrointestinal tract, hepatobiliary system, or pancreas. Participants were enrolled across 50 institutions in Japan between August 8, 2014, and March 6, 2020.

**Interventions:**

In the EP arm, etoposide (100 mg/m^2^/d on days 1, 2, and 3) and cisplatin (80 mg/m^2^/d on day 1) were administered every 3 weeks. In the IP arm, irinotecan (60 mg/m^2^/d on days 1, 8, and 15) and cisplatin (60 mg/m^2^/d on day 1) were administered every 4 weeks.

**Main Outcomes and Measures:**

The primary end point was OS. In total, data from 170 patients were analyzed to detect a hazard ratio (HR) of 0.67 (median OS of 8 and 12 months in inferior and superior arms, respectively) with a 2-sided α of 10% and power of 80%. The pathologic findings were centrally reviewed following treatment initiation.

**Results:**

Among the 170 patients included (median [range] age, 64 [29-75] years; 117 [68.8%] male), median OS was 12.5 months in the EP arm and 10.9 months in the IP arm (HR, 1.04; 90% CI, 0.79-1.37; *P* = .80). The median progression-free survival was 5.6 (95% CI, 4.1-6.9) months in the EP arm and 5.1 (95% CI, 3.3-5.7) months in the IP arm (HR, 1.06; 95% CI, 0.78-1.45). A subgroup analysis of OS demonstrated that EP produced more favorable OS in patients with poorly differentiated NEC of pancreatic origin (HR, 4.10; 95% CI, 1.26-13.31). The common grade 3 and 4 adverse events in the EP vs IP arms were neutropenia (75 of 82 [91.5%] patients vs 44 of 82 [53.7%] patients), leukocytopenia (50 of 82 [61.0%] patients vs 25 of 82 [30.5%] patients), and febrile neutropenia (FN) (22 of 82 [26.8%] patients vs 10 of 82 [12.2%] patients). While incidence of FN was initially high in the EP arm, primary prophylactic use of granulocyte colony-stimulating factor effectively reduced the incidence of FN.

**Conclusions and Relevance:**

Results of this randomized clinical trial demonstrate that both EP and IP remain the standard first-line chemotherapy options. Although AEs were generally manageable, grade 3 and 4 AEs were more common in the EP arm.

**Trial Registration:**

Japan Registry of Clinical Trials: jRCTs031180005; UMIN Clinical Trials Registry: UMIN000014795

## Key Points

**Question:**

For patients with advanced neuroendocrine carcinoma of the digestive system, which of the 2 community standard regimens is more effective: etoposide plus cisplatin (EP) or irinotecan plus cisplatin (IP)?

**Findings:**

In this randomized clinical trial of 170 patients who were chemotherapy naive and had recurrent or unresectable neuroendocrine carcinoma of the digestive system, median overall survival was 12.5 months in the EP arm and 10.9 months in the IP arm.

**Meaning:**

Both EP and IP therapy remain standard first-line chemotherapy options.

## Teaser

This randomized clinical trial assesses whether etoposide plus cisplatin or irinotecan plus cisplatin is a more effective treatment regimen in terms of overall survival in patients with advanced neuroendocrine carcinoma of the digestive system.

## Introduction

Neuroendocrine carcinomas (NECs) are rare and high-grade neuroendocrine neoplasms (NENs) that arise from various organs, and the prognosis in advanced cases is reported to be 8 to 13 months.^1,2,3^ The 2010 World Health Organization (WHO) classification system^4^ introduced a grading scheme based on mitotic counts or Ki-67 index, classifying NENs into 3 tiers (grades 1-3). Grade 3 is composed of tumors with more than 20 mitoses per 10 high-power fields or a Ki-67 index greater than 20%. This classification used the term NEC for grade 3 tumors. Although most grade 3 tumors have been poorly differentiated NECs (PDNECs), it has been reported that some well-differentiated NENs also demonstrate a proliferation rate in the grade 3 range.^5,6,7,8^ In light of these reports, the 2017 WHO classification system^9^ and the 2019 WHO classification system^10^ proposed 3 grades (1, 2, and 3) for neuroendocrine tumors (NETs)—a new entity of NETs with grade 3 characterized by well-differentiated morphology and a Ki-67 index greater than 20%, separate from PDNECs.

Systemic chemotherapy is indicated for advanced NECs, and representative regimens for the treatment of small cell lung carcinoma have been recommended by various guidelines,^11,12,13^ given that these tumor types share pathological and clinical features. A platinum regimen, such as combined etoposide and cisplatin (EP) or irinotecan and cisplatin (IP), has been standard for treatment of small cell lung carcinoma^14^ until the immune checkpoint inhibitors demonstrate additional effects. Although there are some differences in the toxicity profiles, it is difficult to say which is less toxic of either EP or IP.^15^ Regarding NECs, reports for chemotherapies were primarily based on observational studies or small-scale clinical studies.^1,16,17,18,19,20,21,22,23,24,25,26,27,28,29,30,31^ Although we previously reported that EP and IP are commonly used for treating NEC of the digestive system in Japan,^2^ there lacks any reliable evidence regarding which of these regimens is more effective. Therefore, we conducted a phase 3 randomized clinical trial comparing overall survival (OS) between EP and IP.

## Methods

### Study Design and Patients

This study was a multicenter, randomized phase 3 trial across 50 institutions in Japan (eAppendix in Supplement 1). The eligibility criteria were as follows: (1) histologically proven NEC (by 2010 WHO classification, including PDNECs and grade 3 NETs, and harboring >70% NEC components, allowing <30% non-NEC components) diagnosed using a resected sample or having NEC components (allowing any proportions with non-NEC components) diagnosed using tissue biopsy; (2) NEC arising in the esophagus, stomach, duodenum, small intestine, appendix, colon, rectum, gallbladder, extrahepatic bile duct, ampulla of Vater, pancreas, or liver; (3) unresectable or recurrent disease; (4) patients were platinum naive (not allowed for any purpose) and received no previous chemotherapy or radiotherapy for NEC (preoperative or postoperative chemotherapy not including irinotecan or etoposide was allowed as long as it had been completed at least 8 weeks prior to registration); (5) patients aged 20 to 75 years; (6) Eastern Cooperative Oncology Group performance status of 0 or 1; and (7) adequate function of major organs, including leukocyte count 3.0 × 10^9^/L or higher, neutrophil count 1.5 × 10^9^/L or higher, hemoglobin level 9.0 g/dL or higher, number of platelets 100 × 10^9^/L or higher, aspartate aminotransferase and alanine aminotransferase concentrations 100 U/L or lower (150 U/L or lower in patients with liver metastases or liver NEC) (to convert values to μkat/L, multiply by 0.0167), total bilirubin 1.5 mg/dL or lower (to convert to μmol/L, multiply by 17.104), serum creatinine 1.3 mg/dL or lower (to convert to μmol/L, multiply by 76.25), and creatinine clearance 60 mL/min/1.73 m^2^ or higher (to convert to mL/s/m^2^, multiply by 0.0167).

The study protocol (Supplement 2) was approved by the institutional review board of each participating institution, and all enrolled patients provided written informed consent for participation. This study followed the Consolidated Standards of Reporting Trials (CONSORT) reporting guideline.

### Randomization and Masking

Investigators sent the patients’ information to the Japan Clinical Oncology Group (JCOG) Data Center using web-based systems and completed registration, and the JCOG Data Center randomly allocated (1:1) each patient to either the EP or IP arm using the minimization method with a random component to balance the institution and primary site (gastrointestinal tract vs hepatobiliary and pancreas). The trial was open-label, and investigators and patients were not masked to the treatment assignment.

### Procedures

Patients allocated to the EP arm received a regimen of etoposide (100 mg/m^2^/d on days 1, 2, and 3) and cisplatin (80 mg/m^2^/d on day 1), repeated every 3 weeks. Patients allocated to the IP arm received a regimen of irinotecan (60 mg/m^2^/d on days 1, 8, and 15) and cisplatin (60 mg/m^2^/d on day 1), repeated every 4 weeks. Treatment was terminated if disease progression was diagnosed, a serious adverse event (AE) occurred requiring delay of the next course for 21 or more days from the planned date or third or further dose reduction, or if a patient refused to continue the protocol treatment. Physical examinations and laboratory tests were conducted weekly. The AEs were evaluated according to the Common Terminology Criteria for Adverse Events, version 4.0. The JCOG Data and Safety Monitoring Committee reviewed all serious AEs. Tumor markers and enhanced (if possible) or plain computed tomography or magnetic resonance imaging were also performed every 6 weeks. Tumor responses were assessed according to the Response Evaluation Criteria in Solid Tumors, version 1.1. Pathological diagnoses were centrally reviewed by the study-specific pathology panel consisting of 6 pathologists (including N.H.). Because of the rapid proliferation of this disease, a central pathological review (CPR) was performed following registration. The CPR was held approximately once a year, and CPR panelists reviewed the morphological classification (NET grade 3, small cell carcinoma, and large cell carcinoma), Ki-67 index, and non-NEC components. The CPR results were given to the physicians, and participating centers were responsible for subsequent clinical handling.

### Outcomes

The primary end point was OS, while the secondary end points were objective response rate, progression-free survival (PFS), AEs, serious AEs, and dose intensity (DI) of cisplatin. Overall survival was defined as the interval between randomization and death (censored on the date of last contact for surviving patients), and PFS was defined as the interval between randomization and disease progression or death (censored on the last confirmation date of no progression for surviving patients). The DI of cisplatin was calculated as total dose per body-surface area divided by the number of weeks of administration.

### Statistical Analysis

This study was designed to confirm which treatment arm is superior in terms of OS on the assumption that the median OS of inferior and superior arms was 8 and 12 months, respectively (hazard ratio [HR], 0.67). To achieve a power of 70% with a 2-sided significance level of 10%, expected accrual of 6 years, and a follow-up period of 1 year after accrual completion, the required numbers of enrolled patients and deaths were originally determined as 140 and 70, respectively. Because the predetermined criteria was met, with 70 or more patients enrolled in the first 2.5 years, the required numbers of enrolled patients and deaths were revised to 170 and 150, respectively, in May 2017, and the follow-up period was extended to 2 years or until required number of events observed increased the power to 80%. One interim analysis for efficacy and futility assessment was conducted at the time point where half of the revised planned number of the patients were enrolled. The Lan-DeMets and O’Brien-Fleming α spending function was used to adjust multiplicity owing to the repeated testing for OS. The JCOG Data and Safety Monitoring Committee reviewed the interim analyses.

Statistical analyses were performed by the JCOG Data Center using SAS software, version 9.4 (SAS Institute), and a 2-sided α of 5% was used to declare statistical significance except for the primary analysis. The OS and PFS were analyzed for all randomly assigned patients (all registered patients) on an intention-to-treat basis. Subgroup analyses of sex, age, Eastern Cooperative Oncology Group performance status, primary tumor site, and pathologic findings (among CPR-eligible patients) were prespecified. Subgroup analyses of patients with non-NEC component, pancreatic PDNECs and stomach PDNECs were conducted post hoc. Other statistical analysis plans are described in the eMethods in Supplement 1 and in Supplement 3.

## Results

### Patients

**Figure 1.**
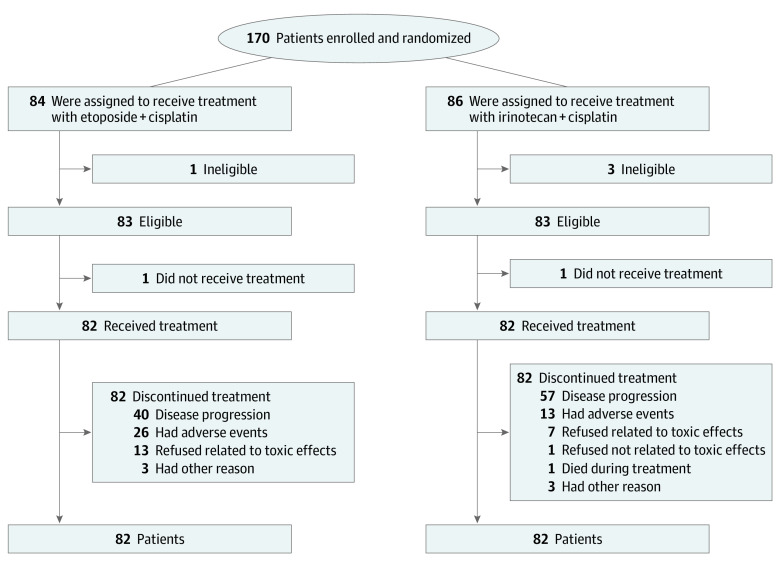
CONSORT Diagram

Between August 8, 2014, and March 6, 2020, 170 patients were enrolled from 50 institutions and randomized; 84 patients were allocated to the EP arm and 86 patients to the IP arm (Figure 1). Four patients (1 in the EP arm and 3 in the IP arm) were determined to be ineligible following enrollment. In the EP arm, 1 patient met the exclusion criteria for continuous use of insulin. In the IP arm, 1 patient was found to have acinar cell carcinoma, 1 patient had other coexisting cancer, and 1 patient did not meet the criteria for creatinine clearance. Those patients were included in the OS/PFS analysis. Baseline characteristics (Table 1) and pathological features of each primary organ (eTables 1 and 2 in Supplement 1) were well balanced between the arms.

**Table 1.** Baseline Characteristics Among the 170 Randomized Patients

Characteristic	No. (%)	
	EP arm (n = 84)	IP arm (n = 86)
Age, median (range), y	64 (38-75)	65 (29-75)
Sex		
Female	26 (31.0)	27 (31.4)
Male	58 (69.0)	59 (68.6)
ECOG PS		
0	55 (65.5)	55 (64.0)
1	29 (34.5)	31 (36.0)
Primary site		
Gastrointestinal tract	50 (59.5)	50 (58.1)
Esophagus	13 (15.5)	8 (9.3)
Stomach	24 (28.6)	31 (36.0)
Duodenum	2 (2.4)	0 (0.0)
Appendix	1 (1.2)	1 (1.2)
Colon	3 (3.6)	7 (8.1)
Rectum	7 (8.3)	3 (3.5)
Hepatobiliary and pancreas	34 (40.5)	36 (41.9)
Gallbladder	14 (16.7)	7 (8.1)
Bile duct/hilar	1 (1.2)	0
Ampulla of Vater	3 (3.6)	6 (7.0)
Pancreas	14 (16.7)	19 (22.1)
Liver	2 (2.4)	4 (4.7)
Pathological diagnosis		
Resected tumor	24 (28.6)	25 (29.1)
Biopsy^a^	60 (71.4)	61 (70.9)
Clinical stage		
Locally advanced	3 (3.6)	7 (8.1)
Metastases	62 (73.8)	62 (72.1)
Recurrence	19 (22.6)^b^	17 (19.8)
Central pathology review		
No.	84	86
Confirmed	73 (86.9)	79 (91.9)
Not confirmed	9 (10.7)	7 (8.1)
NE	2 (2.4)	0
Histological feature		
No.	73	79
Small cell carcinoma	34 (46.6)	32 (40.5)
Large cell carcinoma	37 (50.7)	42 (53.2)
Grade 3 NET	1 (1.4)	4 (5.1)
NE	1 (1.4)	1 (1.3)
Proliferative activity		
Ki-67 ≥50%	60 (82.2)	64 (81.0)
Ki-67 <50%	4 (5.5)	10 (12.7)
NE^b^	9 (12.3)	5 (6.3)
Non-NEC component		
Negative	48 (65.8)	49 (0.6)
Positive	25 (34.2)	30 (38.0)

Abbreviations: ECOG PS, Eastern Cooperative Oncology Group performance status; EP, etoposide plus cisplatin; IP, irinotecan plus cisplatin; NE, not evaluable; NEC, neuroendocrine carcinoma; NET, neuroendocrine tumor.

^a^ One gallbladder NEC (EP arm) and 1 pancreatic NEC (IP arm) were diagnosed by cell block.

^b^ Two patients in the EP arm received preoperative or postoperative chemotherapy not including irinotecan or etoposide.

### Central Pathological Review

Central pathological review was performed for all patients except for 2 patients whose specimens were not available. The pathological diagnosis was not consistent with that of the participating institutions in 16 of the 168 patients (9.5%). This inconsistency was due to the following factors: (1) insufficient proliferation index (n = 2); (2) insufficient expression of endocrine markers (n = 3); (3) NEC components less than 70% (in resected specimen; n = 2); or (4) other histological subtypes (n = 8; eFigure 1 in Supplement 1). Seven of 55 patients (12.7%) with gastric primary tumor and 4 of 31 patients (12.9%) with pancreatic primary tumor had inconsistent pathological diagnoses. Information on proliferative activity, morphological classification, and non-NEC components are summarized in eTables 1 and 2 in Supplement 1.

### Treatment Compliance

Among 166 eligible patients, 1 patient in each arm did not receive the allocated treatment. Among the remaining 164 patients, the median (range) number of cycles of chemotherapy was 4.5 (1-13) in the EP arm and 4.5 (1-17) in the IP arm. The median (range) duration for the treatment in the EP and IP arms was 3.8 (0.7-11.6) months and 4.4 (0.9-18.7) months, respectively. The dose of cisplatin was reduced in 26 of 82 patients (31.7%) in the EP arm and in 25 of 82 patients (30.5%) in the IP arm. The mean (range) DI of cisplatin was 21.9 (14.3-26.8) mg/m^2^/wk in the EP arm and 13.6 (9.9-16.3) mg/m^2^/wk in the IP arm (eTable 3 in Supplement 1), and relative DIs of cisplatin in EP and IP were 82.1% and 90.7%, respectively. The reasons for termination of the protocol treatment included disease progression (40 of 82 patients [48.8%] in the EP arm and 57 of 82 patients [69.5%] in the IP arm) and AE or patient refusal associated with AE (39 of 82 patients [47.6%] in the EP arm and 20 of 82 patients [24.4%] in the IP arm) (Figure 1). Although the number of patients with protocol termination due to AE or patient refusal associated with AE was higher in the EP arm, 12 patients were terminated due to grade 4 neutropenia occurring after second dose reduction in the EP arm vs 5 patients in IP arm. Among those 12 patients, 7 received EP therapy with additional dose reduction as subsequent chemotherapy. Of the 164 treated patients, 64 of 82 patients (78.0%) in the EP arm and 61 of 82 patients (74.4%) in the IP arm received second-line chemotherapy (eTable 7 in Supplement 1).

### Efficacy

**Figure 2.**
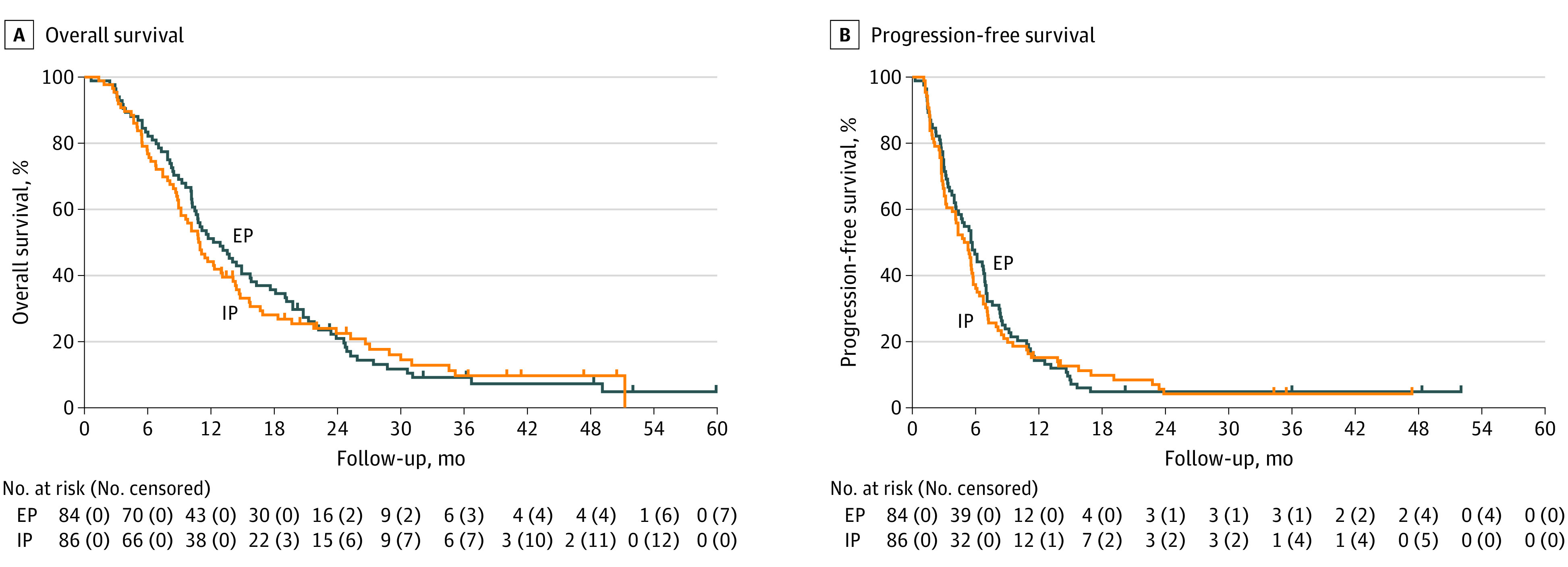
Overall and Progression-Free Survival EP indicates etoposide plus cisplatin; IP, irinotecan plus cisplatin.

**Figure 3.**
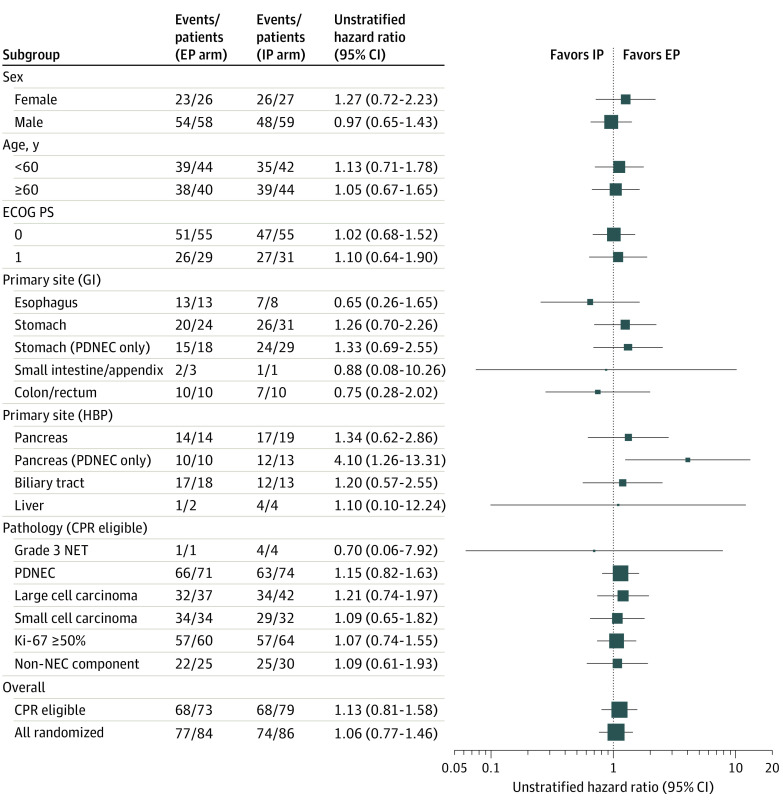
Subgroup Analyses for Overall Survival Subgroup analyses of patients with poorly differentiated neuroendocrine carcinomas (PDNECs; stomach, pancreas) and non-NEC components were completed post hoc. CPR indicates central pathological review; ECOG PS, Eastern Cooperative Oncology Group performance status; EP, etoposide plus cisplatin; GI, gastrointestinal; HBP, hepatobiliary and pancreas; IP, irinotecan plus cisplatin; NEC, neuroendocrine carcinoma; NET, neuroendocrine tumor.

At the data cutoff date of the primary analysis (March 2021), 1 year after last patient enrollment, 151 patients (88.8% of all enrolled patients) had died. The median OS was 12.5 (95% CI, 10.3-15.7) months in the EP arm vs 10.9 (95% CI, 8.9-13.1) months in the IP arm (HR, 1.04; 90% CI, 0.79-1.37; *P* = .80). The 1-year survival proportion was 52.1% (95% CI, 40.1%-62.8%) in the EP arm and 41.8% (95% CI, 30.8%-52.3%) in the IP arm (Figure 2A). No statistically significant differences were identified in the analyses of all eligible patients and of those with CPR (eFigures 2 and 3 in Supplement 1). Results of prespecified subgroup analyses are represented in Figure 3 and eTable 5 in Supplement 1. No statistically significant differences were observed for any cohort of pathological findings, such as small cell carcinoma or large cell carcinoma, or any cohort of primary organs (Figure 3). In a post hoc analysis with small sample size, EP (n = 10) produced more favorable OS than IP (n = 13) for patients with PDNEC of pancreatic origin (median OS, 18.6 [95% CI, 3.0-19.7] months vs 7.9 [95% CI, 5.0-11.0] months, respectively; HR, 4.10 [95% CI, 1.26-13.31]; eFigure 4 in Supplement 1).

The median PFS was 5.6 (95% CI, 4.1-6.9) months in the EP arm and 5.1 (95% CI, 3.3-5.7) months in the IP arm (HR, 1.06 [95% CI, 0.78-1.45]; Figure 2B). Among all eligible patients with measurable lesions, the response rates were 54.5% (42 of 77 patients; 95% CI, 42.8%-65.9%) in the EP arm and 52.5% (42 of 80 patients; 95% CI, 41.0%-63.8%) in the EP arm (*P* = .87; eTable 6 in Supplement 1).

### Adverse Events

Table 2 summarizes the AEs among all 164 treated patients. With regard to grade 3 or 4 AEs among the 82 patients each in the EP and IP arms, white blood cell count decreased in 50 (61.0%) and 25 (30.5%) patients, respectively; neutrophil count decreased in 75 (91.5%) and 44 (53.7%) patients, respectively; anemia occurred in 21 (25.6%) and 14 (17.1%) patients, respectively; platelet count decreased in 10 (12.2%) and 3 (3.7%) patients, respectively; and febrile neutropenia (FN) occurred in 22 (26.8%) and 10 (12.2%) patients, respectively. With regard to all grade AEs, diarrhea was more frequent in the IP arm (EP arm: 19 of 82 patients [23.2%] vs IP arm: 39 of 84 patients [47.6%]). One treatment-related death occurred in the IP arm (hepatic failure), while none occurred in the EP arm. Initially, prophylactic administration of granulocyte colony-stimulating factor (G-CSF) was not recommended in the protocol for either arm; however, owing to the high incidence of FN in the EP arm, the protocol was revised to recommend the primary prophylactic use of G-CSF in the EP arm. The reason for G-CSF administration (primary prophylaxis, secondary prophylaxis, or therapeutic administration) was documented in a case report form (patient No. 35, after December 2015). In the IP arm, because pegfilgrastim is not appropriate for its weekly schedule, it was not recommended.

**Table 2.** Summary of Adverse Event Data According to CTCAE, version 4.0^a^

Adverse event	No. (%)			
	EP arm (n = 82)		IP arm (n = 82)	
	All grades	Grades 3 and 4	All grades	Grades 3 and 4
White blood cell count decreased	68 (82.9)	50 (61.0)	61 (74.4)	25 (30.5)
Neutrophil count decreased	77 (93.9)	75 (91.5)	60 (73.2)	44 (53.7)
Anemia	74 (90.2)	21 (25.6)	70 (85.4)	14 (17.1)
Platelet count decreased	73 (89.0)	10 (12.2)	45 (54.9)	3 (3.7)
Febrile neutropenia	22 (26.8)	22 (26.8)	10 (12.2)	10 (12.2)
Biliary tract infection	2 (2.4)	2 (2.4)	5 (6.1)	5 (6.1)
Anorexia	70 (85.4)	11 (13.4)	60 (73.2)	13 (15.9)
Nausea	57 (69.5)	4 (4.9)	41 (50.0)	6 (7.3)
Diarrhea	19 (23.2)	1 (1.2)	39 (47.6)	5 (6.1)
Fatigue	58 (70.7)	9 (11.0)	52 (63.4)	7 (8.5)
Aspartate aminotransferase increased	49 (59.8)	5 (6.1)	54 (65.9)	3 (3.7)
Alanine transaminase increased	49 (59.8)	11 (13.4)	48 (58.5)	8 (9.8)
Hyponatremia	55 (67.1)	11 (13.4)	56 (68.3)	7 (8.5)
Hypokalemia	19 (23.2)	5 (6.1)	24 (29.3)	4 (4.9)

Abbreviations: CTCAE, Common Terminology Criteria for Adverse Events; EP, etoposide plus cisplatin; IP, irinotecan plus cisplatin.

^a^ Grades 3 and 4 adverse events were observed in at least 5% of patients in either group. One treatment-related death occurred (hepatic failure in the IP arm).

Post hoc analyses were conducted on G-CSF administration and FN incidence. Of the 32 patients who experienced FN (22 in the EP arm and 10 in the IP arm), 19 patients experienced FN during the first course, 2 patients during the second course, 3 patients during the fourth course, and 3 patients during the sixth course and later. Among patients in the EP arm enrolled after December 2015, the incidence of FN during the first course was 27.9% (12 of 43 patients) without primary prophylaxis and 9.5% (2 of 21 patients) with primary prophylaxis. When the total number of patients experiencing FN during all courses administered at initial dose level was analyzed, the incidence of FN was found to be 21.2% (14 of 66 patients) without prophylaxis and 5.1% (3 of 59 patients) with primary or secondary prophylaxis (eTable 4 in Supplement 1).

## Discussion

Evidence for treatment strategies of advanced NEC is lacking owing to the rarity of the disease. Recently, the results of a randomized phase 2 trial for NEC were reported^16^; however, enrollment was terminated early in 66 patients owing to the premature analysis, and no definitive conclusion was obtained. The present study represents the final results of a well-designed and, to our knowledge, the first phase 3 trial with high-quality pathological diagnoses and detailed information provided by a CPR.

The primary analysis of OS revealed no statistically significant difference between arms. Therefore, it is reasonable to continue using both regimens as the standard treatment for NEC. We were interested in the effect of pathological findings and primary organs on treatment efficacy. Although the results showed no statistically significant differences in any subgroups, the overall impression had a greater interaction by primary organ than by pathological findings in terms of OS and PFS (Figure 3 and eFigure 5 in Supplement 1). Because platinum regimens have been reported to be less effective in grade 3 NETs,^5,8^ and the WHO 2017 classification system^9^ and 2019 classification system^10^ formally declared grade 3 NETs as a separate entity from NECs,^9,10^ subgroup analyses of PDNECs only are of relevant clinical interest. Subgroup analysis of OS in PDNECs only indicated weak tendency of preferred results in the EP arm over the IP arm. A subgroup analysis of only PDNECs in each primary tumor indicated that the OS of the EP arm was better than that of the IP arm in the subset of pancreatic PDNEC. Although post hoc subgroup analyses should be interpreted with caution, these results have important implications. The CPR indicated that pancreatic NECs are characterized by a higher proportion of small cell carcinoma and a lower proportion of tumors with non-NEC components (eTable 2 in Supplement 1). Although these characteristics may have influenced the unique results in this subgroup, they cannot fully explain this phenomenon.

In terms of AEs, grade 3 and 4 AEs such as myelosuppression and FN were more common (>2 times) in the EP arm. Although these AEs were generally manageable, the high incidence of FN remains a point of caution. We took note of the high incidence of FN during this trial in the monitoring report; therefore, we revised the protocol to recommend primary prophylaxis with G-CSF^32,33^ after October 31, 2017. In the exploratory analysis of this study, prophylaxis with G-CSF seemed to effectively decrease the incidence of FN. However, considering higher planned/actual DI and lower relative DI in the EP arm, and higher rate of treatment termination owing to AE or patient’s refusal (third dose reduction owing to neutropenia), lower initial dose of EP therapy may be appropriate.

In addition to the outcome of the clinical trial, this study is very valuable in its provision of information on high-quality pathological diagnoses made by consistent CPR panel members throughout the entire period. Notably, the pathological diagnosis was not consistent with that of the participating institutions in nearly 10% of cases, indicating the difficulty of an accurate pathological diagnosis of NEC. Additionally, detailed information about the pathological findings was obtained for the cross-organ manner, which itself is an important asset in the field of NEC. In the forest plots of OS and PFS, the difference between EP and IP among the CPR subgroups was smaller than that of the primary organs (Figure 3 and eFigure 5 in Supplement 1).

### Limitations

This study has some limitations. Although the primary analyses revealed no statistically significant difference in OS between the 2 arms, the study was not designed to assess the equivalence of the 2 regimens. Therefore, the 2 regimens should not be recognized as equivalent, but rather as they do not differ beyond a certain level. In addition, in patients diagnosed via results of biopsy specimens, accurate information of the entire tumor could not be obtained with respect to proliferative activity or the proportion of non-NEC components. In this study, patients diagnosed by biopsy harboring any proportions of non-NEC components, as long as a harboring NEC component was allowed to be enrolled, means that theoretically a certain percentage of patients with mixed adenoneuroendocrine carcinomas were enrolled. These limitations were unavoidable considering the diagnostic procedure, and this is also a universal issue in daily practice. Additionally, because further effects of immune checkpoint inhibitors on platinum regimens has been proven in small cell lung carcinoma,^34,35^ it is the next important clinical question in NECs.

## Conclusions

Results of this phase 3 randomized clinical trial demonstrate that both EP and IP remain the standard first-line chemotherapy regimens for advanced digestive NECs. Post hoc subgroup analyses pointed to the superiority of EP in pancreatic PDNECs, and EP was safely given along with the use of primary prophylactic G-CSF.

## Notes

Supplement 1. **eAppendix.** Participating Institutions (50 institutions from north to south) **eMethods.** Statistical analysis **eTable 1.** Pathological features on CPR for each primary organ (GI/HBP) **eTable 2.** Pathological features on central pathology review for each primary organ (N > 20 primary organ) **eTable 3.** Dose intensity of Cisplatin **eTable 4.** Prophylactic Treatment with G-CSF and Febrile Neutropenia (Only initial dose level) **eTable 5.** Results of subgroup analyses of overall survival, progression free survival, and response rate for each primary organ **eTable 6.** Response rate of all eligible patients with measurable lesions and patients whose diagnosis was pathologically confirmed by central pathology review **eTable 7.** Second-line chemotherapy **eFigure 1.** CONSORT diagram (Central pathology review, CPR) **eFigure 2.** Overall survival (all eligible patients) **eFigure 3.** Overall survival (CPR confirmed patients) **eFigure 4.** Survival curves of EP arm and IP arm, subgroup analysis for PDNEC of pancreatic origin **eFigure 5.** Forest plot (progression free survival)

Supplement 2. Trial Protocol

Supplement 3. Statistical Analysis Plan

Supplement 4. **Nonauthor Collaborators.** Japan Clinical Oncology Group (JCOG) nonauthor collaborators

Supplement 5. Data Sharing Statement
