# Supplementary material for: Effectiveness of Etoposide and Cisplatin vs Irinotecan and Cisplatin Therapy for Patients With Advanced Neuroendocrine Carcinoma of the Digestive System: The TOPIC-NEC Phase 3 Randomized Clinical Trial
Source: JAMA Oncol. 2022 Aug 18;8(10):1447–55. doi: 10.1001/jamaoncol.2022.3395 (PMC9389440; doi:10.1001/jamaoncol.2022.3395)
Supplement: Supplement 1. — eAppendix. Participating Institutions (50 institutions from north to south) eMethods. Statistical analysis eTable 1. Pathological features on CPR for each primary organ (GI/HBP) eTable 2. Pathological features on central pathology review for each primary organ (N > 20 primary organ) eTable 3. Dose intensity of Cisplatin eTable 4. Prophylactic Treatment with G-CSF and Febrile Neutropenia (Only initial dose level) eTable 5. Results of subgroup analyses of overall survival, progression free survival, and response rate for each primary organ eTable 6. Response rate of all eligible patients with measurable lesions and patients whose diagnosis was pathologically confirmed by central pathology review eTable 7. Second-line chemotherapy eFigure 1. CONSORT diagram (Central pathology review, CPR) eFigure 2. Overall survival (all eligible patients) eFigure 3. Overall survival (CPR confirmed patients) eFigure 4. Survival curves of EP arm and IP arm, subgroup analysis for PDNEC of pancreatic origin eFigure 5. Forest plot (progression free survival) [file jamaoncol-e223395-s001.pdf]

Supplemental Online Content

Morizane C, Machida N, Honma Y, et al. Effectiveness of etoposide and cisplatin vs irinotecan and cisplatin therapy for patients with advanced neuroendocrine carcinoma of the digestive system: the TOPIC-NEC phase 3 randomized clinical trial. *JAMA Oncol*. Published online August 18, 2022. doi:10.1001/jamaoncol.2022.3395

eAppendix. Participating Institutions (50 institutions from north to south)
eMethods. Statistical analysis
eTable 1. Pathological features on CPR for each primary organ (GI/HBP)
eTable 2. Pathological features on central pathology review for each primary organ (N > 20 primary organ)
eTable 3. Dose intensity of Cisplatin
eTable 4. Prophylactic Treatment with G-CSF and Febrile Neutropenia (Only initial dose level)
eTable 5. Results of subgroup analyses of overall survival, progression free survival, and response rate for each primary organ
eTable 6. Response rate of all eligible patients with measurable lesions and patients whose diagnosis was pathologically confirmed by central pathology review
eTable 7. Second-line chemotherapy
eFigure 1. CONSORT diagram (Central pathology review, CPR)
eFigure 2. Overall survival (all eligible patients)
eFigure 3. Overall survival (CPR confirmed patients)
eFigure 4. Survival curves of EP arm and IP arm, subgroup analysis for PDNEC of pancreatic origin
eFigure 5. Forest plot (progression free survival)

This supplemental material has been provided by the authors to give readers additional information about their work.

© 2022 Morizane C et al. *JAMA Oncol.*

eAppendix. Participating Institutions (50 institutions from north to south)

Sapporo-Kosei General Hospital

Hokkaido University Hospital

Keiyukai Sapporo Hospital

Iwate Medical University

Tohoku University Hospital

Miyagi Cancer Center

Tochigi Cancer Center

Jichi Medical University

Saitama Cancer Center

National Cancer Center Hospital East

Chiba Cancer Center

Chiba University, Graduate School of Medicine

National Cancer Center Hospital

Kyorin University Faculty of Medicine

National Center for Global Health and Medicine (NCGM)

Cancer Institute Hospital of Japanese Foundation for Cancer Research

Toranomon Hospital

Teikyo University School of Medicine

St.Marianna University School of Medicine

Kanagawa Cancer Center

Yokohama City University Medical Center

Niigata Cancer Center Hospital

© 2022 Morizane C et al. *JAMA Oncol.*

Toyama University Hospital
Kanazawa University Hospital
Ishikawa Prefectural Central Hospital
Ogaki Municipal Hospital
Shizuoka Cancer Center
Aichi Cancer Center Hospital
Kyoto University Hospital
Osaka University Graduate School of Medicine
Kindai University Hospital
Osaka International Cancer Institute
National Hospital Organization Osaka National Hospital
Osaka General Medical Center
Osaka Medical and Pharmaceutical University
Osaka Rosai Hospital
Kobe University Graduate School of Medicine
Kansai Rosai Hospital
Hyogo College of Medicine
Hyogo Cancer Center
Itami City Hospital
Shimane University Faculty of Medicine
Hiroshima University Hospital
Fukuyama City Hospital
Tokushima Red Cross Hospital

© 2022 Morizane C et al. *JAMA Oncol.*

National Hospital Organization Shikoku Cancer Center

Kochi Health Sciences Center

National Hospital Organization Kyushu Cancer Center

Kyushu University Hospital

Oita University Hospital

© 2022 Morizane C et al. *JAMA Oncol.*

eMethods. **Statistical analysis**

OS and PFS were analyzed for all randomly assigned patients (all registered patients) on an intention-to-treat basis. Survival curves were estimated using the Kaplan-Meier method. The primary analysis was assessed by a stratified log-rank test. For OS, stratified Cox proportional-hazards model was applied to calculate hazard ratio and associated confidence intervals. The randomization adjustment factors excluding institutions were used for all stratified analyses. Except for the primary analysis for OS, unstratified log-rank test and unstratified Cox proportional-hazards model were used. We did prespecified subgroup analyses for efficacy endpoints. Response rate was analyzed among all eligible patients with measurable lesions and was compared between arms with Fisher's exact test. Safety was assessed on a per-protocol basis using all treated patients as the denominator. All P values were reported as two-sided. Definitions of analyses set were as follows

All registered patients

Among the patients enrolled according to the Procedures for Enrollment, the population excluding duplicate or mis-enrollment is considered as "all enrolled cases".

All treated patients

Of all enrolled patients, all patients for whom part or all of the protocol treatment was performed will be defined as all treated patients.

The decision to treat "non-treated patients" for whom no protocol treatment has been given and whether it is excluded from the safety analysis can be determined by the data center with the consent of the Research Office. Ineligible patients will be excluded from all treated patients. However, if there are circumstances in which ineligible patients are included in the analysis, the nature of the ineligibility will be examined and determined by the Research Secretariat in consultation with JCOG Data Centre.

© 2022 Morizane C et al. *JAMA Oncol.*

eTable 1. Pathological features on CPR for each primary organ (GI/HBP)

		GI		HBP	
		N=100		N=68*	
Central pathology review	Confirmed	90	(90%)	62	(91.2%)
	Not confirmed	10	(10%)	6	(8.8%)
		N=90		N=62	
Histological feature	Small cell carcinoma	25	(27.8%)	41	(66.1%)
	Large cell carcinoma	64	(71.1%)	15	(24.2%)
	NET G3	1	(1.1%)	4	(6.5%)
	NE	0	(0.0%)	2	(3.2%)
Proliferative activity	Ki67≥50%	75	(83.3%)	49	(79.0%)
	Ki67<50%	4	(4.4%)	10	(16.1%)
	NE**	11	(12.2%)	3	(4.8%)
non-NEC component	-	52	(57.8%)	45	(72.6%)
	+	38	(42.2%)	17	(27.4%)

*histopathological specimens were not available for CPR in 2 patients

** NE: not evaluable

© 2022 Morizane C et al. *JAMA Oncol.*

eTable 2. Pathological features on central pathology review for each primary organ (N＞20 primary organ)

		Esophagus		Stomach		Colon/Rectum		Pancreas		Biliary tract**	
Central pathology review		N=21		N=55		N=20		N=31*		N=31	
	Confirmed	20	(95.2%)	48	(87.3%)	19	(95.0%)	27	(87.1%)	30	(96.8%)
	Not confirmed	1	(4.8%)	7	(12.7%)	1	(5.0%)	4	(12.9%)	1	(3.2%)
		N=20		N=48		N=19		N=27		N=30	
Histological feature	Small cell	11	(55%)	11	(22.9%)	2	(10.5%)	15	(55.6%)	24	(80.0%)
	Large cell	9	(45%)	36	(75.0%)	17	(89.5%)	8	(29.6%)	6	(20.0%)
	NET G3	0	(0.0%)	1	(2.1%)	0	(0.0%)	3	(11.1%)	0	(0.0%)
	NE**	0	(0.0%)	0	(0.0%)	0	(0.0%)	1	(3.7%)	0	(0.0%)
Proliferative activity	Ki67≥50%	16	(80.0%)	40	(83.3%)	17	(89.5%)	21	(77.8%)	25	(83.3%)
	Ki67<50%	0	(0.0%)	3	(6.3%)	1	(5.3%)	6	(22.2%)	2	(6.7%)
	NE***	4	(20.0%)	5	(10.4%)	1	(5.3%)	0	(0.0%)	3	(10.0%)
non-NEC component	-	13	(65%)	28	(58.3%)	10	(52.6%)	20	(74.1%)	20	(66.7%)
	+	7	(35%)	20	(41.7%)	9	(47.4%)	7	(25.9%)	10	(33.3%)

*histopathological specimens were not available for CPR in 2 patients

**Gallbladder, Biliary tract-hilar, and Ampulla of Vater

*** NE: not evaluable

© 2022 Morizane C et al. *JAMA Oncol.*

eTable 3. Dose intensity of Cisplatin

		EP<br>N=82	IP<br>N=82
All	Mean DI (range)	21.9 (14.3-26.8)	13.6 (9.9-16.3)
GI	Mean DI (range)	21.8 (14.3-26.8)	13.6 (10.5-16.3)
HBP	Mean DI (range)	22.0 (16.8-26.7)	13.6 (9.9-15.2)

Dose intensity（mg/m^2/week)＝total dose/body surface area/treatment duration (week)

Treatment duration (week)

EP arm= (start day of last course - start day of 1st course + 21)/7

IP arm = (start day of last course - start day of 1st course + 28)/7

© 2022 Morizane C et al. *JAMA Oncol.*

eTable 4. Prophylactic Treatment with G-CSF and Febrile Neutropenia (Only initial dose level)

		EP arm	
		No prophylactic use	Prophylactic use
1st Cycle	FN%(n)	27.9% (12/43)	9.5% (2/21)
	[95%CI]	[15.3-43.7%]	[1.2-30.4%]
All Cycles*	FN%(n)	21.2% (14/66)	5.1% (3/59)
	[95%CI]	[12.1-33.0%]	[1.1-14.1%]

Analysis set: 131 patients (64 patients in EP arm and 67 patients in IP arm) after No.35 out of 164 all treated patients. Results of IP arm was not shown because pegfilgrastim is not appropriate because of weekly schedule.

* Proportions were calculated as the total counts up to 8 courses among the patients who started each course at initial dose level (level 0).

© 2022 Morizane C et al. *JAMA Oncol.*

eTable 5. Results of subgroup analyses of overall survival, progression free survival, and response rate for each primary organ

			ALL	GI all					HBP all			
					Esophagus	Stomach	Colon/Rectal	Small intestine /Appendix		Pancreas	Biliary tract	Liver
	N(EP/IP)		170(84/86)	100(50/50)	21(13/8)	55(24/31)	20(10/10)	4(3/1)	70(34/36)	33(14/19)	31(18/13)	6(2/4)
Overall survival	Median months [95%CI]	EP	12.5 [10.3-15.7]	11.1 [10.1-14.0]	8.4 [5.5-10.1]	13.6 [10.1-23.9]	14.0 [8.1-15.8]	10.2 [6.4-NE]	16.3 [8.9-19.7]	10.6 [3.3-19.2]	16.3 [10.4-22.2]	21.3 [NE-NE)
		IP	10.9 [8.9-13.1]	11.4 [9.1-14.8]	11.5 [3.2-18.4]	11.1 [8.7-23.9]	11.5 [1.3-NE]	28.9 [NE-NE]	9.9 [6.3-12.9]	9.8 [5.0-11.4]	12.3 [6.1-16.9]	19.2 [3.8-51.3]
Progression free survival	Median months [95%CI]	EP	5.6 [4.1-6.9]	5.6 [3.5-6.2]	3.2 [2.7-5.6]	6.0 [4.7-8.5]	4.1 [1.4-8.2]	6.9 [4.1-NE]	7.0 [2.9-9.2]	2.9 [1.5-7.6]	7.1 [4.8-11.6]	NE [4.4-NE]
		IP	5.1 [3.3-5.7]	5.1 [3.2-6.1]	3.8 [1.2-15.8]	4.8 [2.9-5.7]	6.1 [1.2-NE]	19.1 [NE-NE]	4.8 [2.8-6.0]	3.0 [1.7-5.6]	6.0 [2.8-8.5]	13.8 [1.4-23.9]
	N(EP/IP)		157(77/80)	92(45/47)	20(12/8)	50(21/29)	20(10/10)	-	65(32/33)	30(13/17)	30(17/13)	5(2/3)
Response rate	% [95%CI]	EP	54.5 [42.8-65.9]	57.8 [42.2-72.3]	41.7 [15.2-72.3]	71.4 [47.8-88.7]	40.0 [12.2-73.8]	-	50.0 [31.9-68.1]	23.1 [5.0-53.8]	76.5 [50.1-93.2]	0.0 [0.0-84.2]
		IP	52.5 [41.0-63.8]	55.3 [40.1-69.8]	62.5 [24.5-91.5]	48.3 [29.4-67.5]	70.0 [34.8-93.3]	-	48.5 [30.8-66.5]	35.3 [14.2-61.7]	69.2 [38.6-90.9]	33.3 [0.8-90.6]

NE: not estimable

© 2022 Morizane C et al. *JAMA Oncol.*

eTable 6. Response rate of all eligible patients with measurable lesions and patients whose diagnosis was pathologically confirmed by central pathology review

	EP		IP		P value†)
	All eligible patients with measurable lesions				
	N=77		N=80		
Objective response rate	54.5%		52.5%		0.87
[95% CI]	[42.8 - 65.9]		[41.0 - 63.8]		
Complete response	0	(0.0%)	1	(1.3%)	
Partial response	42	(54.5%)	41	(51.3%)	
Stable disease	24	(31.2%)	25	(31.3%)	
Progressive disease	10	(13.0%)	12	(15.0%)	
Not evaluable	1	(1.3%)	1	(1.3%)	
	Pathologically confirmed by central pathology review				
	N=69		N=77		
Objective response rate	55.1%		54.5%		1.00
[95% CI]	[42.6-67.1]		[42.8-65.9]		

© 2022 Morizane C et al. *JAMA Oncol.*

eTable 7. Second-line chemotherapy

	Arm A(EP) N=82		Arm B (IP) N=82	
Performed	69	(84.2%)	68	(82.9%)
Performed (Chemotherapy)	*64*	*(78.0%)*	*61*	*(74.4%)*
AMR	*16*	*(19.5%)*	*23*	*(28.0%)*
Irinotecan	*12*	*(14.6%)*	*3*	*(3.7%)*
IP	*7*	*(8.5%)*	*3*	*(3.7%)*
EP	*8*	*(9.8%)*	*8*	*(9.8%)*
EC	*4*	*(4.9%)*	*8*	*(9.8%)*
Everolimus	*4*	*(4.9%)*	*5*	*(6.1%)*
S-1	*2*	*(2.4%)*	*1*	*(1.2%)*
Not performed	13	(15.9%)	13	(15.9%)
Not known	0	(0.0%)	1	(1.2%)

© 2022 Morizane C et al. *JAMA Oncol.*

eFigure 1. CONSORT diagram (Central pathology review, CPR)

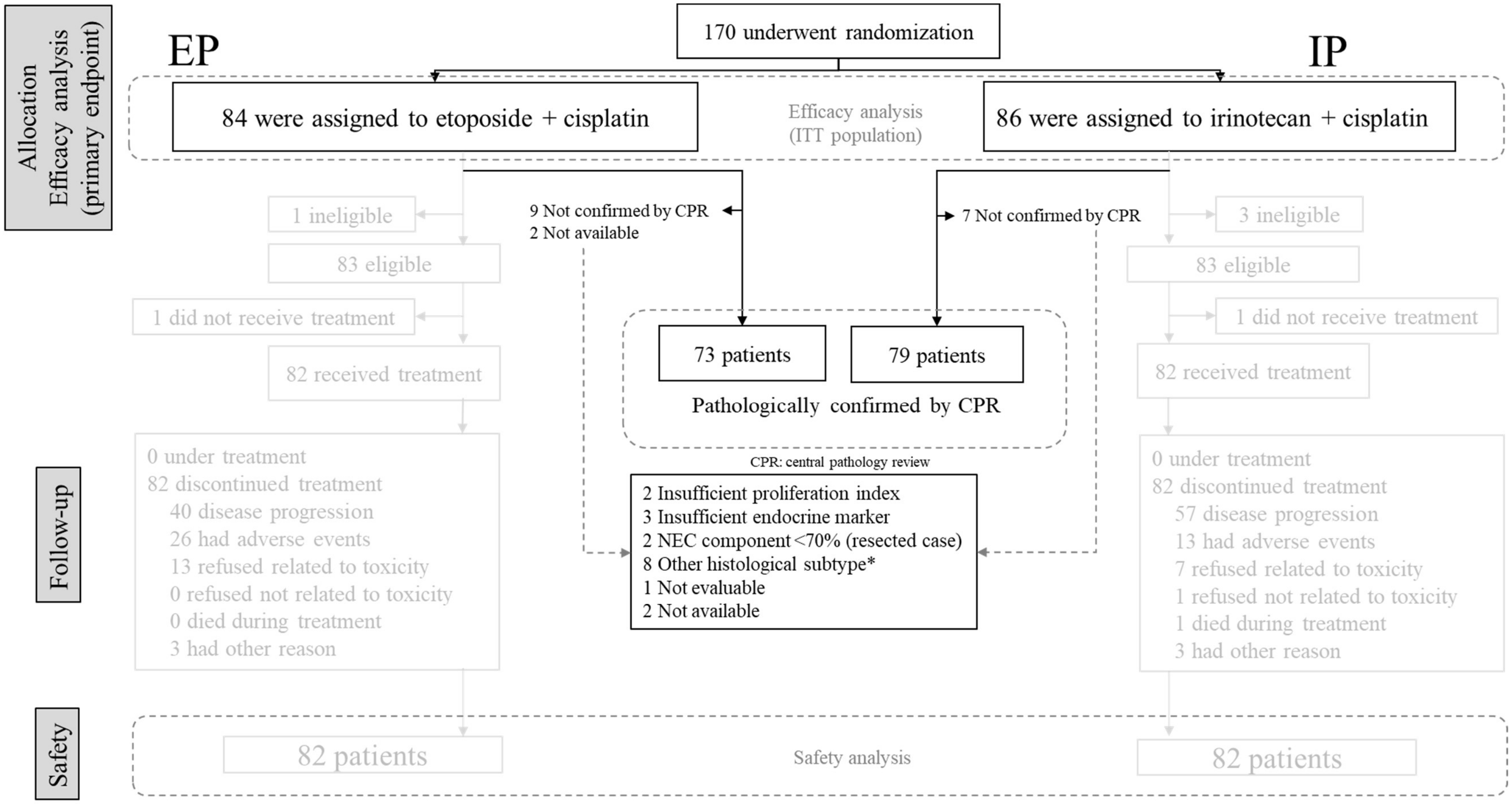

* Other histological subtype include acinar cell carcinoma, 3 (pancreas); acinar cell carcinoma or adenocarcinoma, 1 (pancreas); squamous cell carcinoma, 2 (stomach, gallbladder); goblet cell carcinoid, 1 (appendix); and sarcoma, and 1 (stomach)

© 2022 Morizane C et al. *JAMA Oncol.*

eFigure 2. Overall survival (all eligible patients)

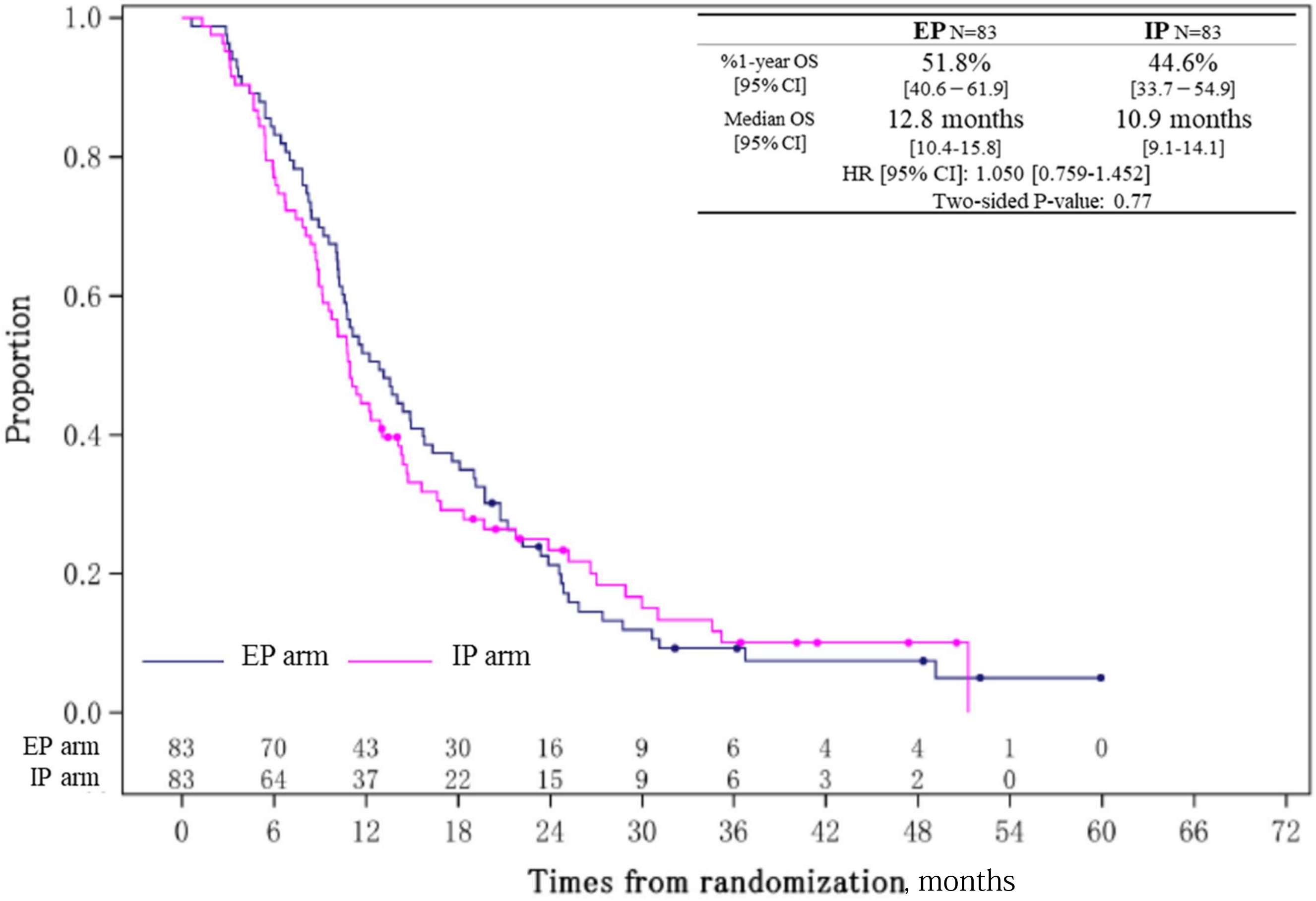

© 2022 Morizane C et al. *JAMA Oncol.*

eFigure 3. Overall survival (CPR confirmed patients)

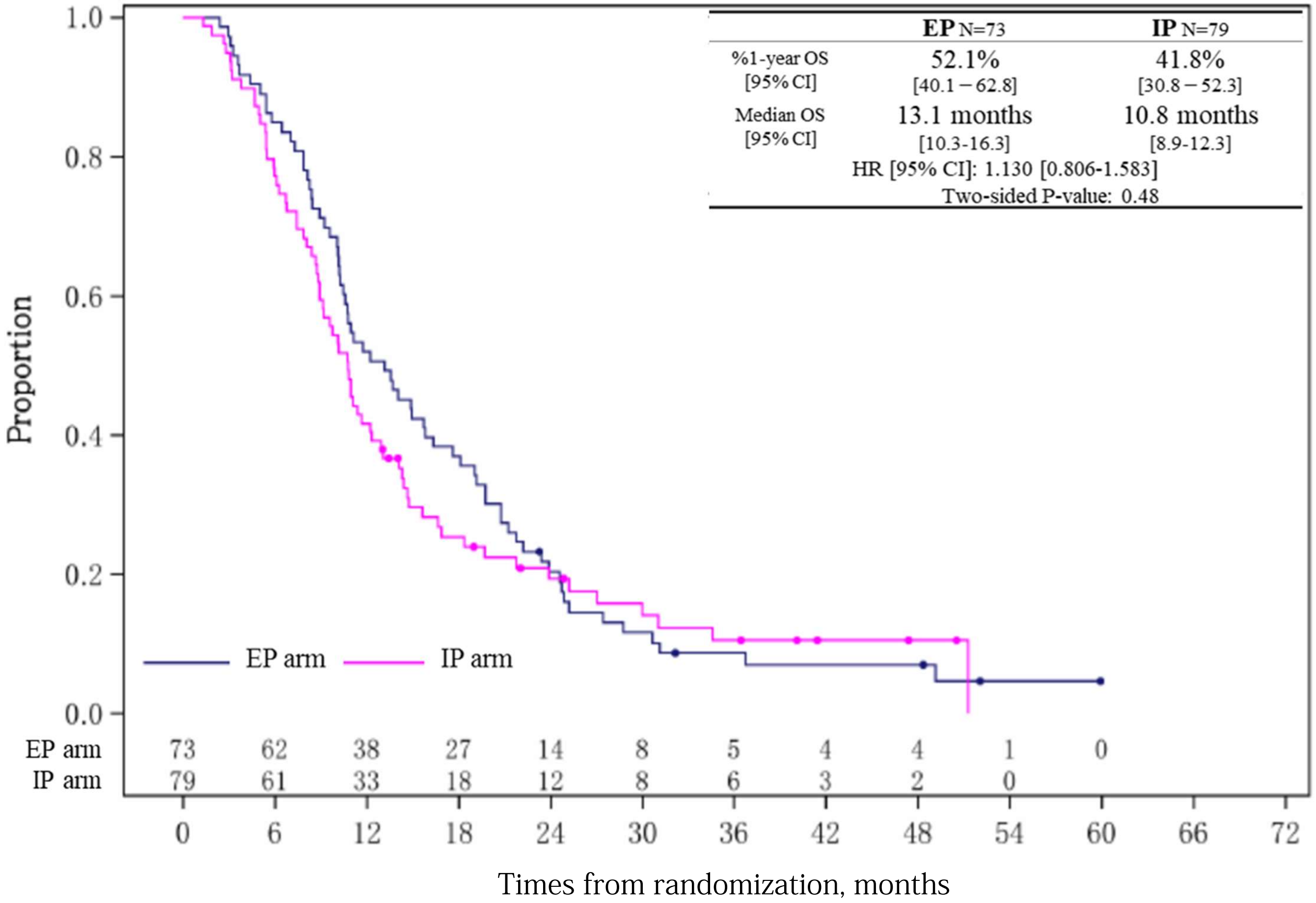

© 2022 Morizane C et al. *JAMA Oncol.*

eFigure 4. Survival curves of EP arm and IP arm, subgroup analysis for PDNEC of pancreatic origin

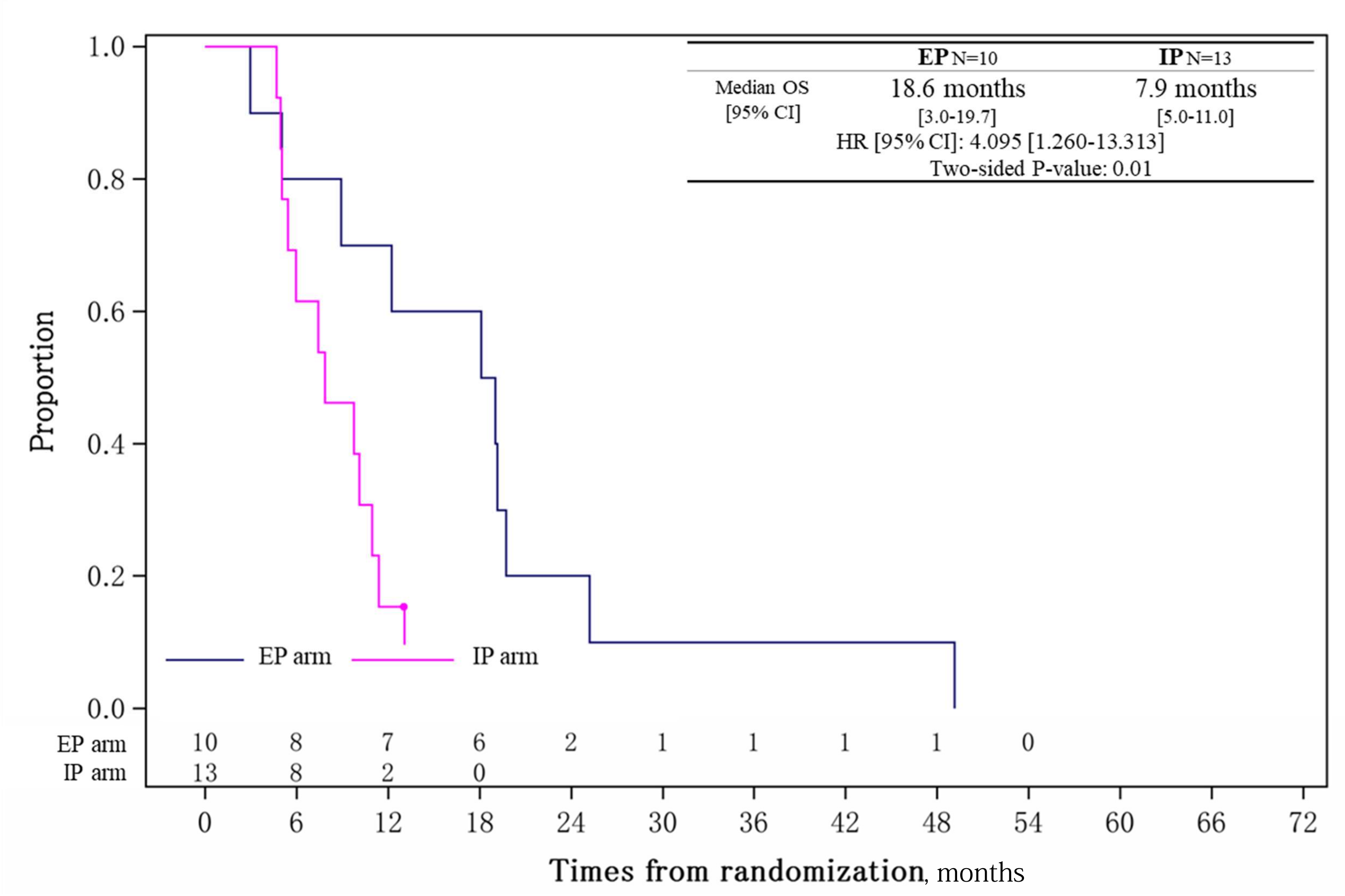

© 2022 Morizane C et al. *JAMA Oncol.*

eFigure 5. Forest plot (progression free survival)

Subgroup	Events/Patients (EP arm)	Events/Patients (IP arm)	Unstratified Hazard Ratio (95% CI)
**Sex**			
Male	56/58	55/59	1.10 (0.76− 1.60)
Female	24/26	26/27	0.98 (0.56− 1.71)
**Age**			
<60	41/44	38/42	0.96 (0.61− 1.49)
>=60	39/40	43/44	1.18 (0.76− 1.83)
**ECOG PS**			
0	52/55	50/55	1.10 (0.75− 1.63)
1	28/29	31/31	0.98 (0.59− 1.64)
**Primary site (GI)**			
Esophagus	13/13	8/8	0.56 (0.21− 1.49)
Stomach	22/24	30/31	1.51 (0.87− 2.62)
* Stomach (PDNEC only)	17/18	28/29	1.28 (0.70− 2.34)
SI/Appendix	2/3	1/1	0.88 (0.08−10.26)
Colorectal	10/10	7/10	0.58 (0.22− 1.54)
**Primary site (HBP)**			
Pancreas	14/14	19/19	1.07 (0.53− 2.18)
* Pancreas (PDNEC only)	10/10	13/13	1.85 (0.74− 4.61)
Biliary tract	18/18	12/13	1.28 (0.61− 2.67)
Liver	1/2	4/4	1.41 (0.12−15.84)
**Pathology (CPR confirmed)**			
NET G3	1/1	4/4	0.70 (0.06− 7.92)
Poorly diff NEC	69/71	69/74	1.02 (0.73− 1.42)
Large cell carcinoma	35/37	38/42	1.01 (0.64− 1.60)
Small cell carcinoma	34/34	31/32	1.00 (0.61− 1.64)
Ki67>=50%	59/60	60/64	0.97 (0.68− 1.40)
* non-NEC component +	23/25	28/30	1.02 (0.59− 1.77)
**Overall**			
CPR eligible	71/73	74/79	0.98 (0.71− 1.36)
All randomized	80/84	81/86	1.06 (0.78− 1.44)

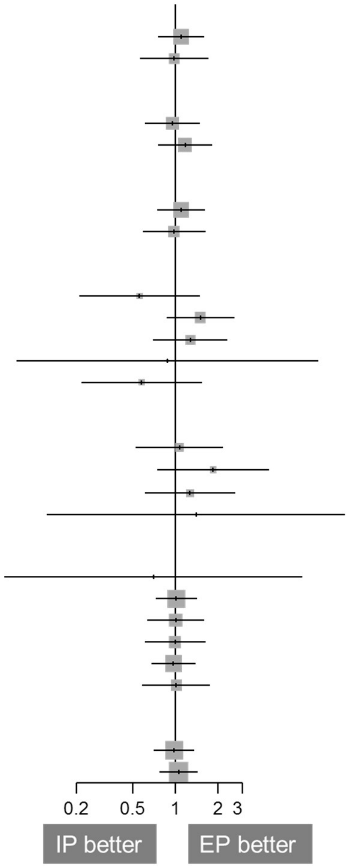

*Subgroup analyses of PDNEC only cohort (Stomach, Pancreas) and non-NEC component are post-hoc

PDNEC: poorly differentiated NEC

© 2022 Morizane C et al. *JAMA Oncol.*
