# Supplementary material for: Effectiveness of Etoposide and Cisplatin vs Irinotecan and Cisplatin Therapy for Patients With Advanced Neuroendocrine Carcinoma of the Digestive System: The TOPIC-NEC Phase 3 Randomized Clinical Trial
Source: JAMA Oncol. 2022 Aug 18;8(10):1447–55. doi: 10.1001/jamaoncol.2022.3395 (PMC9389440; doi:10.1001/jamaoncol.2022.3395)
Supplement: Supplement 2. — Trial Protocol [file jamaoncol-e223395-s002.pdf]

Japan Clinical Oncology Group
Hepatobiliary and Pancreatic Oncology Group/Stomach Cancer Study Group/Japan Esophageal Oncology Group

National Cancer Center Research and Development Fund 26-A-4

JCOG1213

Randomized phase III study of etoposide plus cisplatin combination therapy versus irinotecan plus cisplatin combination therapy in advanced neuroendocrine carcinoma of the digestive system. ver.1.0

TOPIC-NEC

HBPOG
Group Chair: Junji FURUSE
Kyorin University Faculty of Medicine

Study Chair: Takuji OKUSAKA
National Cancer Center Hospital

Study Coordinator: Chigusa MORIZANE
National Cancer Center Hospital

SCSG
Group Chair: Mitsuru SASAKO
Hyogo Medical University

Study Chair: Narikazu BOKU
St. Marianna University School of Medicine

Study Coordinator: Nozomu MACHIDA
Shizuoka Cancer Center

JEOG
Group Chair: Yuko KITAGAWA
Keio University Hospital

Study Chair: Ken KATO
National Cancer Center Hospital

Study Coordinator: Yoshitaka HONMA/Ken KATO
National Cancer Center Hospital

2013/3/16	Protocol concept approved by JCOG Executive Committee (PC1213/1214/1215)
2014/6/27	Protocol approved byJCOG Protocol Review Committee

0. Summary

This study is conducted as a "specified clinical trial" based on the Clinical Trials Act (Act No. 16 of April 14, 2017). In this protocol, the Principal Investigator is the Study Chair in the Hepatobiliary and Pancreatic Oncology Group of JCOG.

Name of study: "Randomized phase III study of etoposide plus cisplatin combination therapy versus irinotecan plus cisplatin combination therapy in advanced neuroendocrine carcinoma of the digestive system. (TOPIC-NEC) ".

Public study title: "Randomized phase III study of etoposide plus cisplatin combination therapy versus irinotecan plus cisplatin combination therapy in advanced neuroendocrine carcinoma of the digestive system. (TOPIC-NEC) "

0.1. Schema

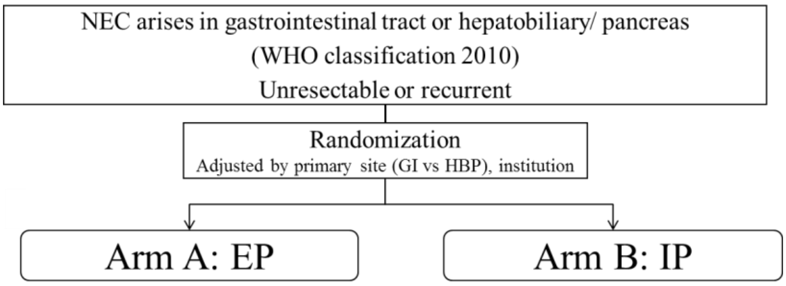

0.2. Objectives

A randomized phase III study was conducted to determine the better treatment option between etoposide/cisplatin combined therapy (EP therapy) or irinotecan/cisplatin combined therapy (IP therapy), both of which are standard treatments for non-resectable/recurrent neuroendocrine carcinoma (NEC as classified by WHO in 2010) with primary lesions in the gastrointestinal tract/hepatobiliary pancreatic organs.

Primary endpoint: Overall survival

Secondary endpoints: Response rate in case of measurable lesions, Progression-free survival (PFS), incidence rate of adverse events, dose intensity of Cisplatin, and incidence rate of serious adverse events.

0.3. Subjects

1) Any of the following is applicable based on pathological diagnosis taking findings of immunohistochemistry into consideration (see 3.1. to 3.3.).

[1] Pathologically diagnosed as neuroendocrine carcinoma (NEC*1) in the resected sample.

[2] Containing pathologically confirmed component of neuroendocrine carcinoma (NEC*1) in the biopsy sample.

1: Based on WHO 2010 classification

2) Any of the following is applicable

[1] NEC arise in esophagus, stomach, duodenum, intestine, appendix, colon, rectum, gallbladder, intrahepatic bile duct, extrahepatic bile duct, ampulla of Vater, pancreas,

[2] Liver NEC (primary liver or liver metastasis of unknown primary) *2.

*2: The tumor is only in one of the following sites after a thorough examination of the primary site by contrast CT (from the neck to pelvic) and upper/lower gastrointestinal endoscopy, FDG-PET scan, otolaryngology (head and neck) examination, urology examination (male patients only), and gynecology examination (female patients only).

a. Liver only

b. Bone, lymph nodes below the diaphragm, peritoneum, subcutaneous, muscle, spleen, and liver

3) Unresectable (see 3.6) or recurrent cancer (see 3.7). It is not essential for a pathological confirmation of the metastatic lesion or recurrent site. Cases of esophageal NEC is ineligible if corresponding to any of the following.

[1] cT4.

[2] No distant metastasis rather than supraclavicular lymph node

[3] Stenosis indicated for palliative radiotherapy

4) No previous chemotherapy or radiotherapy for NEC. Pre- or post-operative chemotherapy except irinotecan or

etoposide for NEC is allowed as long as it was completed at least 8 weeks prior to registration.
5) No previous chemotherapy using platinum agents for any malignancies.
6) Aged 20 to 75 years old.
7) ECOG performance status of 0 or 1.
8) Measurable region is not required.
9) Adequate organ functions.
[1] WBC ≥3,000/ mm^3
[2] Neutrophils ≥ 1,500/ mm^3
[3] Hemoglobin ≥ 9.0 g/dL
[4] Platelets ≥ 10×10^4/ mm^3
[5] Total bilirubin≤ 1.5 mg/dL※4
[6] AST(sGOT)≦100 IU/L※4 (for hepatic NEC and liver metastasis, ≦150 IU/L)
[7] ALT(sGPT)≦100 IU/L※4 (for hepatic NEC and liver metastasis, ≦150 IU/L)
※4: Presence or absence of biliary drainage is not relevant
[8] Serum creatinine≦ 1.3 mg/dL
[9] Creatinine clearance※5≥ 60 mL/min
※5: Creatinine clearance must have been estimated using the Cockcroft-Gault formula, and must be 60 mL/min or more. If the estimation is less than 60 mL/min, but the actual measurement is 60 mL/min or more, the patient can be deemed eligible.
Cockcroft-Gault formula
Male: Ccr ={(140−age) × body weight (kg)}/{72×serum creatinine (mg/dL)}
Female: Ccr = 0.85×{(140−age) × body weight (kg)}/{72×serum creatinine(mg/dL)}
11) Written informed consent.

0.4. Treatments

Arm A: etoposide plus cisplatin (EP) arm

The following chemotherapy is continued until the patient meets discontinuation criteria, with 3-weeks of treatments counting as one cycle.

Drug	Dosage (mg/m^2)	Dosing regimen/Dosing time	Dose day
Etoposide	100	IV/60-120 min	Day 1, 2, 3
Cisplatin	80	IV/60-120 min	Day 1

Arm B: Irinotecan plus Cisplatin (IP) arm

The following chemotherapy would be continued until the patient meets discontinuation criteria, with 4-weeks of treatments counting as one cycle.

Drug	Dose (mg/m^2)	Dosing regimen/Dosing time	Dose day
Irinotecan	60	IV/90 min	Day 1, 8, 15
Cisplatin	60	IV/60-120 min	Day 1

0.5. Planned sample size and study period

The planned sample size is 140.
Accrual period: 6 years.
Follow-up period: 1 years after accrual completion.
Total study duration 7 years.

0.6. Contact information

Eligibility criteria, treatment modification, and other issues requiring clinical decisions: Study Coordinator (front cover and 16.6.)
Enrollment procedure protocol, case report form (CRF) entries, etc.: JCOG Data Center, (16.14.)
Adverse event reporting: JCOG Data and Safety Monitoring Committee (16.11.),

Contents

0. SUMMARY 2

0.1. SCHEMA...2
0.2. OBJECTIVES 2
A RANDOMIZED PHASE III STUDY WAS CONDUCTED TO DETERMINE THE BETTER TREATMENT OPTION BETWEEN ETOPOSIDE/CISPLATIN COMBINED THERAPY (EP THERAPY) OR IRINOTECAN/CISPLATIN COMBINED THERAPY (IP THERAPY), BOTH OF WHICH ARE STANDARD TREATMENTS FOR NON-RESECTABLE/RECURRENT NEUROENDOCRINE CARCINOMA (NEC AS CLASSIFIED BY WHO IN 2010) WITH PRIMARY LESIONS IN THE GASTROINTESTINAL TRACT/HEPATOBILIARY PANCREATIC ORGANS. 2
PRIMARY ENDPOINT : OVERALL SURVIVAL 2
SECONDARY ENDPOINTS : RESPONSE RATE IN CASE OF MEASURABLE LESIONS, PROGRESSION-FREE SURVIVAL (PFS), INCIDENCE RATE OF ADVERSE EVENTS, DOSE INTENSITY OF CISPLATIN, AND INCIDENCE RATE OF SERIOUS ADVERSE EVENTS. .2
0.3. SUBJECTS...2
0.4. TREATMENTS...3
0.5. PLANNED SAMPLE SIZE AND STUDY PERIOD...3
0.6. CONTACT INFORMATION 3

1. OBJECTIVES 7

2. BACKGROUND 7

2.1. TARGET 7
2.2. STANDARD TREATMENT FOR TARGET DISEASE 15
2.3. RATIONALE FOR ESTABLISHMENT OF TREATMENT PLAN 17
2.4. STUDY DESIGN..20
2.5. SUMMARY OF EXPECTED ADVANTAGES AND DISADVANTAGES ASSOCIATED WITH STUDY PARTICIPATION.............21
2.6. SIGNIFICANCE OF THIS STUDY...22
2.7. ASSOCIATED RESEARCH (INCLUDING SAMPLE ANALYSIS RESEARCH)...22
2.8. JCOG-BIOBANK JAPAN (BBJ) COLLABORATING BIOBANK..22

3. CRITERIA/DEFINITIONS USED IN THIS STUDY 23

3.1. TISSUE CLASSIFICATION (WHO 2010 CLASSIFICATION)..23
3.2. GRADE CLASSIFICATION （ENETS [EUROPEAN NEUROENDOCRINE TUMOR SOCIETY] / WHO2010 CLASSIFICATION) 23
3.3. HISTOPATHOLOGICAL DIAGNOSIS 23
3.4. DISEASE STAGE CLASSIFICATION CRITERIA (UICC-TNM 7TH EDITION)..23
3.5. RESIDUAL TUMOR (R) CLASSIFICATION (UICC-TNM 7TH EDITION)...31
3.6. DEFINITION OF NON-RESECTABLE NEC 31
3.7. DEFINITION OF RECURRENT NEC...32
3.8. DEFINITION OF HEPATIC NEC (HEPATIC PRIMARY LESION OR LIVER METASTASIS FROM AN UNKNOWN PRIMARY LESION) 32

4. PATIENT SELECTION CRITERIA 32

4.1. INCLUSION CRITERIA (FOR ENROLLMENT)...32
4.2. EXCLUSION CRITERIA 33

5. REGISTRATION AND RANDOMIZATION...34

5.1. PROCEDURE OF REGISTRATION 34
5.2. RANDOMIZATION AND ALLOCATION ADJUSTMENT FACTOR...35

6. TREATMENT PLAN AND TREATMENT MODIFICATION CRITERIA...36

6.1. PROTOCOL TREATMENT 36
6.2. PROTOCOL TREATMENT TERMINATION/COMPLETION CRITERIA 39
6.3. TREATMENT MODIFICATION CRITERIA...40
6.4. CONCOMITANT TREATMENT AND SUPPORTIVE CARE 46
6.5. POST-STUDY TREATMENT..52

7. ANTICIPATED ADVERSE EVENTS 52

7.1. ANTICIPATED ADVERSE REACTIONS 52
7.2. EVALUATION OF ADVERSE EVENTS/REACTIONS 55

8. EXAMINATION AND EVALUATION...55

8.1. BASELINE EXAMINATION AND EVALUATION BEFORE REGISTRATION 55
8.2. EXAMINATION AND EVALUATION DURING TREATMENT 56
8.3. EXAMINATION AND EVALUATION AFTER COMPLETION OF TREATMENT...58
8.4. INFORMATION ON POST- STUDY TREATMENT...58
8.5. STUDY CALENDAR 59

9. DATA COLLECTION 63

9.1. CASE REPORT FORM (CRF) 63

10. REPORTING OF "DISEASE OR THE LIKE"(ADVERSE EVENTS) 64

10.1. SERIOUS ADVERSE EVENTS...64
10.2. INVESTIGATOR'S REPORTING REQUIREMENTS AND PROCEDURES 65
10.3. RESPONSIBILITIES OF PRINCIPAL INVESTIGATOR/STUDY COORDINATOR 67
10.4. RESPONSIBILITIES OF THE SITE INVESTIGATORS AT THE PARTICIPATING INSTITUTIONS (INCLUDING THE RELEVANT INSTITUTION)...69
10.5. RESPONSIBILITIES OF THE DATA AND SAFETY MONITORING COMMITTEE 69

11. RESPONSE EVALUATION AND ENDPOINT DEFINITION 70

11.1. RESPONSE ASSESSMENT (ONLY FOR PATIENTS WITH MEASURABLE DISEASE) 70
11.2. DEFINITIONS OF ANALYSES SET...76
11.3. DEFINITION OF ENDPOINTS...76

12. STATISTICAL CONSIDERATION...79

12.1. PRINCIPAL ANALYSIS AND DECISION CRITERIA 79
12.2. PLANNED ACCRUAL, ACCRUAL PERIOD, AND FOLLOW-UP PERIODS..80
12.3. INTERIM ANALYSIS AND EARLY TERMINATION OF THE STUDY 80
12.4. ANALYSIS OF SECONDARY ENDPOINTS 81
12.5. FINAL ANALYSIS..82
12.6. EXPLORATORY ANALYSIS 82
12.7. PREMATURE WITHDRAWAL FROM THE TRIAL 83
12.8. PROCEDURES AFTER EARLY TERMINATION OF THE STUDY 83

13. ETHICAL CONSIDERATIONS..85

13.1. PROTECTION OF HUMAN SUBJECTS...85
13.2. INFORMED CONSENT 85
13.3. PROTECTION OF PERSONAL INFORMATION AND PATIENT IDENTIFICATION 86
13.4. COMPLIANCE WITH PROTOCOL 87
13.5. APPROVAL BY MEDICAL INSTITUTION IRBS...87
13.6. PROTOCOL MODIFICATIONS 87
13.7. MANAGEMENT OF CONFLICTS OF INTEREST (COI) ON THE PART OF PERSONS INVOLVED IN JCOG STUDIES..........87

13.8. COMPENSATION 87
13.9. INTELLECTUAL PROPERTY 87

14. MONITORING AND AUDIT 89

14.1. PERIODIC MONITORING 89
14.2. SITE VISIT AUDITS 91
14.3. MANAGEMENT OF NON-COMPLIANCE 92

15. SPECIAL INSTRUCTIONS 93

15.2. JCOG BIOBANK JAPAN (BBJ) BIOREPOSITORY 93

16. ORGANIZATION 94

16.1. MAIN STUDY FUND (FUNDING SOURCE) OF THIS STUDY. 94
16.2. JAPAN CLINICAL ONCOLOGY GROUP (JCOG) 94
16.3. JCOG CHAIR 94
16.4. STUDY GROUP AND GROUP CHAIR 94
16.5. STUDY CHAIR (PRINCIPAL INVESTIGATOR) 95
16.6. STUDY COORDINATOR 95
16.7. CENTRAL PATHOLOGICAL REVIEW COORDINATOR 95
16.8. CENTRAL PATHOLOGICAL REVIEW COORDINATOR 95
16.9. PARTICIPATING SITES (PARTICIPATING INSTITUTIONS) 97
16.10. JCOG PROTOCOL REVIEW COMMITTEE 98
16.11. JCOG DATA AND SAFETY MONITORING COMMITTEE 98
16.12. JCOG AUDIT COMMITTEE 98
16.13. JCOG CONFLICT OF INTEREST COMMITTEE 98
16.14. DATA CENTER/OPERATIONS OFFICE 98
16.15. DEVELOPING A STUDY PROTOCOL 100

17. PUBLICATION OF THE STUDY RESULTS AND COMPLETION OF THE STUDY 101

17.1. PAPER AND CONFERENCE PRESENTATIONS 101
17.2. PRIMARY ENDPOINT REPORT AND CLINICAL SUMMARY REPORT 101
17.3. COMPLETION OF THE STUDY 102

18. REFERENCES 103

19. APPENDIX 103

1. Objectives

A randomized phase III study was conducted to determine the better treatment option between etoposide/cisplatin combined therapy (EP therapy) or irinotecan/cisplatin combined therapy (IP therapy), both of which are standard treatments for non-resectable/recurrent neuroendocrine carcinoma (NEC as classified by WHO in 2010) with primary lesions in the gastrointestinal tract/hepatobiliary pancreatic organs.

Primary endpoint： Overall survival

Secondary endpoints： Response rate in case of measurable lesions
Progression-free survival (PFS), incidence rate of adverse events, dose intensity of Cisplatin, and incidence rate of serious adverse events

2. Background

2.1. Target

2.1.1. Epidemiology

Neuroendocrine tumors (NETs) and neuroendocrine carcinomas (NECs) can arise in various organs in the body. Of these, NECs with primary lesions occurring in the lungs, namely small cell lung cancer and large cell lung cancer, have been collectively referred to as high-grade NECs (HGNECs). However, terms such as extrapulmonary small cell carcinoma and extrapulmonary NEC have been used to refer collectively to NECs, with primary lesions outside the lungs.

The number of newly reported cases of neuroendocrine neoplasms (NENs) annually, estimated based on the cases registered in the U.S. SEER（Surveillance, Epidemiology, and End Results）database between 1973 to 2004 is 5 in 100,000 people[1]. In Japan, the Neuroendocrine Tumor Workshop Japan （NET Work Japan）carried out a nationwide survey of pancreatic and gastrointestinal NENs, and estimated the number of new cases in 2005 at approximately 1.01 people with pancreatic primary lesion per 100,000 people, and 2.10 people with gastrointestinal primary lesion per 100,000 people[2, 3].

The report by the PRONET Study Group in France concerning their prospective observational study is a good reference for the proportion of NENs that can be classified as NECs. Out of the 778 patients diagnosed with gastrointestinal primary lesion NENs in 80 facilities from August 2010 to July 2011, 104 patients (13.4%) had NEC[4].

The frequency of incidence of NECs according to the primary lesion organ has been shown in the tables below (Table 2.1.1a and Table 2.1.1b). Despite variations between reports, gastrointestinal primary lesion NECs account for approximately 20–68% of all extrapulmonary NEC cases. The breakdown of gastrointestinal primary lesion NEC cases with primary lesion in the gastrointestinal tract (esophagus, duodenum, small and large intestines) indicated that these cases accounted for 56–84% of the total cases, while hepatobiliary pancreatic cancer cases accounted for 15–35% of all cases.

Table 2.1.1a. Frequency of extrapulmonary NECs according to the organ of primary lesion

Reported year/Reporter	N	Gastrointestinal	Gynecological organs	Urology	Head and Neck	Adrenal	Primary unknown	Other
2012/Terashima [5]	136	68%	12%	6%	NA	NA	8%	7%
2010/Brennan [6]	74	20%	35%	15%	19%	NA	9%	1%
2009/Wong [7]	1618	33%	NA	20%	11%	10%	4%	22%
2006/Haider [8]	101	20%	11%	18%	12%	9%	31%	NA
2007/Lee [9]	61	56%	20%	10%	8%	NA	6%	NA

Table 2.1.1b. Percentage breakdown of NECs as per the primary lesion organ of gastrointestinal NEC

Reported year/Reporter	N	Esophageal	Gastric	Duodenal/small intestine	Colorectal	Hepatobiliary	Pancreas	Other
2012/Machida [10]	258	33%	27%	2%	12%	12%	14%	NA
2012/Sorbye [11)]	205	6%	10%	NA	40%	NA	35%	10%
2004/Brenner [12]	544	53%	11%	0.2%	20%	11%	4%	NA

2.1.2. Clinical pathology

1) About the term of target disease

The target of this study has a complex disease concept, and since the method of classification has changed with time, various names have been assigned that could cause confusion. In this study, we use classification and nomenclature defined according to the WHO 2010 classification（WHO Classification of Tumors of the Digestive System 2010）[13].

All the tumors originating from neuroendocrine cells or differentiation into endocrine cells are called neuroendocrine neoplasms (NENs). Depending on the malignancy, NENs were classified as Grade 1 NETs (NET G1), Grade 2 NETs (NET G2), or NEC.

Furthermore, prior to the WHO 2010 classification, all disease concepts equivalent to NENs were called NETs, but in this study, we would have referred to all descriptions of the disease concept as NENs. Furthermore, according to the 2000 and 2004 WHO classifications, the term well-differentiated NEC (WDNEC) has been used for disease units equivalent to NET G2, but in this study NEC does not mean the same.

2) Classification and clinical pathology

NEN is a tumor that develops from neuroendocrine cells present in various tissues or shows a tendency of differentiation into neuroendocrine cells. Histologically these tumors express neuroendocrine markers, such as Chromogranin A, NSE (neuron-specific enolase), and synaptophysin. The WHO classifications in 2000 and 2004 combined the presence or absence of metastasis/local infiltration and cellular proliferative capacity (evaluated based on Ki-67 expression intensity and mitotic presentation), and thereby classified these tumors into well-differentiated (neuro)endocrine tumors without metastasis/local infiltration, well-differentiated (neuro)endocrine tumors with metastasis/local infiltration, and poorly differentiated (neuro)endocrine tumors with even greater cell proliferative capacity. On the other hand, European Neuroendocrine Tumor Society (ENETS) has proposed a method of classification that evaluates the malignancy as Grade 1, 2, or 3 (G1, G2, or G3), according to the cell proliferative capacity (Ki-67 index or number of mitotic presentations), which has proven to be most useful for classification of prognosis[14, 15]。

Against this backdrop, the WHO classification that targeted gastrointestinal diseases was published in 2010. The disease as a whole was considered “neuroendocrine neoplasms (NEN)”, and the disease malignancy was largely classified as NET G1 (neuroendocrine tumor Grade 1), NET G2 (Grade 2), NEC, and mixed adenoneuroendocrine carcinoma (MANEC). MANEC refers to the cancers wherein adenocarcinoma components account for more than 30% of the cancer, while those under 30% are classified as NECs. However, even though this method of classification is applicable while evaluating the entire tumor based on resected specimen, it is not possible to evaluate the proportion of each component in the entire tumor during a diagnosis based on biopsy sample.

Each of NET G1, NET G2, and NECs described in the WHO 2010 classification is equivalent to G1, G2, and G3 as per the ENETS classification. NETs are well-differentiated tumors, with relatively low atypicality and malignancy, and the clinical course is slow with a 5-year survival in the range of 62–85%[16, 17]. On the other hand, NECs are poorly differentiated tumors, where tumor cells with poor cellular constituents proliferate diffusely, and are pathologically similar to small cell lung cancers, since these show many mitotic presentations, involve necrotic lesions, and present with neuroendocrinological features during immunohistochemical staining[1]. The clinical presentations are also similar in terms of the rapid proliferation of NECs and relatively high sensitivity to anticancer drugs[18].

Similar to lung cancer, NECs also exist as small cell cancer type or large cell type (LCNEC：large cell NEC）[13]. Future challenges include studies on the frequency and differences in clinical presentation of each disease. Table 2.1.2 shows the WHO and Grade classification.

Table 2.1.2. Changes in WHO classification and grading; Shaded parts are targets of this study

WHO 2000 classification	WHO 2010 classification		ENETS Grade
Well-differentiated endocrine tumor（WDNET）<br>1.1‘Benign’ behavior<br>1.2 Uncertain behavior	Neuroendocrine tumors, NETs<br>• Well-differentiated<br>• Composed of cells similar to normal gastrointestinal	NET G1（carcinoid）	G1<br>Same as 2010 WHO classification<br>Number of mitotic presentations < 2 per 10

	endocrine cells<br>• Expression of neuroendocrine markers<br>• Hormone production<br>• Mild to moderate nuclear atypia, low proliferative capacity (Grade: G1,G2)		high power fields (HPF) and/or Ki-67 index ≤2%
Well-differentiated endocrine carcinoma (WDEC)		NET G2	G2<br>Number of mitotic presentations 2–20 per 10 HPF and/or Ki-67 index 3-20%
Poorly differentiated endocrine carcinoma/small cell carcinoma (PDEC)	Neuroendocrine carcinoma: NEC (large cell or small cell type):<br>• Poorly differentiated, highly malignant<br>• Include small cell to large cell type carcinoma<br>• Sometimes present tissue structure similar to NET<br>• Expression of neuroendocrine markers<br>• Significant nuclear atypia, multifocal necrosis, and high proliferative capacity (Grade: G3)		G3<br>Number of mitotic presentations >20 per 10 HPF and/or Ki-67 index > 20%
Mixed exocrine-endocrine carcinoma (MEEC)	Mixed adenoendocrine carcinoma (MANEC)		
Tumor-like lesions (TLL)	Hyperplastic and preneoplastic lesions		

2.1.3. Staging

Disease staging for NENs is represented by the TNM classification proposed by ENETS (hereafter, ENETS TNM[14, 15], AJCC 7th Edition TNM classification and UICC 7th Edition TNM classification published in 2009. The AJCC 7th Edition and UICC 7th Edition TNM classifications are unified, and in this study we would have used the UICC 7th Edition nomenclature).

In the UICC 7th edition, there are independent TNM classifications of NET G1 and NET G2 with primary lesions in the stomach, small intestine, appendix, and colon, but with respect to NEC, it describes "classification according to main tissue type in each organ of primary lesion (squamous cell carcinoma for esophagus, and adenocarcinoma in all other organs of primary lesion)." While there are TNM classifications for hepatocellular carcinoma and intrahepatic cholangiocarcinoma in the liver, there is no description for the classification of the tissue type for NECs with primary lesion in the liver. In this study, however, we would have used the TNM classification of intrahepatic cholangiocarcinoma, which shows similar clinical presentations. Details of each TNM classification would have been mentioned in "3.2 staging criteria".

2.1.4. Standard treatment according to disease staging and outline of prognosis

1) Standard treatment for resectable cases

Surgical resection is performed for resectable cases. The significance of postoperative adjuvant chemotherapy is not clear because a verification study has not yet been performed. Although the NCCN Guidelines[19] describe postoperative adjuvant therapy as the chemotherapy regimen for small cell lung cancers that is typically applied to advanced cases of NEC, it has relatively high toxicity and patient burden (see Table 2.2.2, 2.3.2); hence we cannot claim that it is widely used in routine practice. For NECs with gastrointestinal primary lesion with mixed adenocarcinoma, a chemotherapy regimen is suggested typically after surgery for adenocarcinoma; however, in practice, there is no consensus on postoperative adjuvant chemotherapy.

2) Standard treatment for locally advanced cases

While the NCCN Guidelines propose chemoradiotherapy for small cell lung cancer for locally advanced cases, the ENETS Guidelines provide no clear indication[20, 21] for the same. As there is no sufficient information related to radiation dose, efficacy, and safety for NECs with a multitude of primary lesion organs (scope of irradiation), radiation therapy is not widely used for pathologies other than NEC with esophageal primary lesion that is prone to stenosis, and treatment is often provided for distant metastasis.

For locally advanced cases of NEC with esophageal primary lesion, chemoradiotherapy is actively performed. Furthermore, supraclavicular lymph node metastasis in thoracic esophageal tumor is classified as Stage IV, and chemoradiotherapy is applied if metastatic lymph nodes are included in the field of irradiation.

3) Standard treatment for distal metastasis cases

Systemic chemotherapy is indicated for distal metastasis and recurrent cases regardless of the organ, and a regimen is chosen for the small cell lung cancer. With respect to NECs with esophageal primary lesion, even if there is organ metastasis, palliative chemoradiotherapy is prioritized for patients incapable of oral intake of drugs due to esophageal constriction.

The efficacy of everolimus and sunitinib has been demonstrated for NETs with pancreatic primary lesions. Furthermore, Octreotide and Lantreotide effectively inhibit proliferation of NETs that originate in the midgut and pancreatic/gastrointestinal NETs, respectively. However, these results are from clinical trials conducted in patient populations corresponding to NETs. Since the clinical presentations of NETs and NECs differ significantly, these treatments cannot be indicated for NECs during routine practice.

4) Indication of resection for distal metastasis

There is no consensus on the resection criteria specific to the NECs. Since the progression of NETs is slow, resection is actively considered even where there is distal metastasis to the liver, but as NEC progresses fast, surgical resection cannot be indicated for cases with distal metastasis[19, 21].

5) Prognosis

In a preceding study, Yamaguchi and Machida carried out a multicenter joint observational study of poorly

differentiated NEC (WHO 2010 NEC, including patients with clinical diagnosis of poor differentiation) in a total of 23 facilities, including facilities participating in JCOG Hepatobiliary and Pancreatic Oncology Group, former Gastroenterology Group, and observer facilities.

In the multicenter joint study by Yamaguchi and Machida, 89% of cases (229/258 patients) receiving systemic chemotherapy had distal metastasis, with a median survival time (MST) of 11.5 months for all patients, 11.2 months for distal metastasis cases, and 15.9 months for locally advanced cases. MST according to organ was 13.4 months for esophageal primary lesion (N = 85), 13.3 months for gastric primary lesion (N = 70), 29.7 months for small intestine/duodenal primary lesion (N = 6), 7.6 months for colonic primary lesion (N = 31), 8.5 months for pancreatic primary lesion (N = 35), and 7.9 months for hepatobiliary primary lesions (N = 31) (Table 2.1.4). While there were 9 cases of five-year survival among all the gastrointestinal cases, there were no five-year survival cases amongst those with primary lesions in hepatobiliary and pancreatic organs[10]. In a multicenter joint observational study for NECs conducted in four Northern European countries (hereafter referred to as NORDIC NEC Study), the MST for NECs with gastrointestinal primary lesion (albeit including ~30% of NECs with primary lesion sites unknown) was 11 months[11]. According to the U.S. SEER database, the MST of NEC was 10 months[3]. Based on the above information, despite differences in the primary lesion organ, the prognosis of the target population of this study is thought to have an overall MST of 10–11 months.

Table 2.1.4. Treatment results according to primary lesion organ for non-resectable/recurrent NECs that underwent systemic chemotherapy

	Esophageal	Gastric	Duodenal/small intestine	Colonic	**Gastrointestinal overall**	Pancreatic	Hepatobiliary	**Hepatobiliary and pancreatic organs overall**
N	85	70	6	31	**192**	35	31	**66**
Response rate（%）	58%	43%	50%	29%	**47%**	17%	16%	**17%**
Median PFS（month）	5.8	4.9	7.3	3.7	**5.1**	3.2	4.1	**3.7**
MST（month）	13.4	13.3	29.7	7.6	**13**	7.9	8.5	**7.9**

2.1.5. Tumor-related complications

We, here, report the complications that require attention during patient management, according to the organ of primary lesion. It is a rare disease and frequencies remain unknown.

1) Esophageal primary

Tumor bleeding/pain/esophageal stenosis/fistula formation due to primary lesion in the esophagus; hoarseness/difficulty swallowing/aspiration pneumonia caused by recurrent laryngeal nerve palsy due to lesion in lymph node metastasis; jaundice and liver failure associated with liver metastasis; respiratory failure and hemoptysis associated with lung metastasis, airway constriction, and pain due to lymph node metastasis; hypercalcemia, tracheal stenosis, tracheal obstruction, and suture failure due to disease progression.

2) Gastric primary

Chronic bleeding from primary lesion, accompanied by anemia, stomach pain, nausea, vomiting, gastric perforation, bloating, suture failure, fistula formation, cardiac/pyloric stenosis, ascites retention due to peritoneal metastasis, ileus, hydronephrosis due to ureteral stenosis, obstructive jaundice/liver failure due to bile duct stenosis, and obstructive jaundice/liver failure due to hepatic portal lymph node metastasis.

3) Small intestine/colonic primary

Tumor hemorrhage, ileus, fistula formation in the small intestine/bladder/vagina, intestinal obstruction, intestinal stenosis, intestinal perforation, and pelvic infection

4) Hepatobiliary and pancreatic primary

Cancer pain, rupture, obstructive jaundice associated with the growth of primary tumor, weight loss, fever, pancreatitis, cholangitis, liver abscess, cholecystitis, biliary hemorrhage, duodenal hemorrhage, duodenal stenosis, anastomotic ulcer/stenosis/leakage, symptoms associated with stenosis/obstruction of portal vein (liver

dysfunction/liver failure, esophageal varices and their rupture, gastritis, ascites retention, and hepatic encephalopathy/coma associated with portal hypertension).

5) Paraneoplastic syndrome

In addition to NECs, there are reports of paraneoplastic syndrome that is often associated with small cell lung carcinoma[22-24]. We report examples of paraneoplastic syndrome seen with small cell lung carcinoma below.

Hyponatremia due to inappropriate sodium secretion, psychological symptoms (changes in personality) due to ectopic ACTH syndrome, hypertension, hypokalemia, high blood sugar, Lambert-Eaton myasthenic syndrome, paraneoplastic cerebellar degeneration associated with autoantibody production (ataxia in the limbs, dysarthria, and nystagmus), paraneoplastic encephalomyelitis/sensory neuron disease (dementia, cranial nerve symptoms, dizziness, ataxia, autonomic imbalance, transverse paralysis, and sensory disorders).

Apart from these, there are thromboembolic events, aspiration (due to gastrointestinal stenosis), anemia, tumor pain, acute renal failure, myositis, and pulmonary fibrosis.

6) Complications due to metastatic lesions

Liver failure, pain, hepatobiliary infection, hemorrhage, bile duct stenosis, bile duct obstruction, respiratory failure, tracheal hemorrhage, respiratory tract infection, atelectasis, airway constriction, ascites, bloating, ileus, ureteral stenosis, ureteral obstruction, urinary retention, urinary tract infection, hiccups, pleural effusion, pleural hemorrhage, chest pain, pathological fracture, pain, hypercalcemia, meningeal carcinomatosis, ataxia, ischemia cerebrovascular, intracranial hemorrhage, nausea, vomiting, dizziness, consciousness disorder, cognitive disturbance, dysphasia, seizure, spasticity, edema limbs, intestinal obstruction/stenosis/perforation, pancreatitis, disseminated intravascular coagulation, thrombocytopenia, anorectal infection, anal hemorrhage, hoarseness, and superior vena cava syndrome

7) Others

General pain management including narcotic analgesics is performed for cancer pain.

Gastrointestinal stenting and bypass surgery are performed for esophageal stenosis, cardiac/pyloric stenosis, and duodenal stenosis.

Percutaneous transhepatic cholangial drainage (PTCD), percutaneous or endoscopic stenting, and bile duct jejunostomy is performed for obstructive jaundice. In addition, complications associated with biliary drainage and gastrointestinal stenting are listed below.

- PTCD, including internal and external fistula tube placement:
  Cholangitis, pancreatitis, cholecystitis, liver abscess, sepsis, biliary hemorrhage, PTCD tube obstruction/deviation, peritonitis, and pneumothorax/pleurisy
- Biliary stenting:
  Cholangitis, pancreatitis, cholecystitis, liver abscess, sepsis, biliary hemorrhage, peritonitis, pneumothorax/pleurisy (for percutaneous stenting), duodenal perforation, pneumonia (for endoscopic stenting), stent obstruction/deviation, duodenal ulcer, and duodenal hemorrhage
- Bile duct jejunostomy:
  Cholangitis, pancreatitis, cholecystitis, liver abscess, and sepsis
- Gastrointestinal stenting:
  Hemorrhage, perforation, pain, stent deviation, stent obstruction, foreign-body sensation, intestinal strangulation, ulcer formation, fever, sepsis, infection, diarrhea, constipation, tenesmus or uncontrollable urination/incontinence symptoms (colon), thyroid injury (esophagus), carotid artery injury (esophagus), and mediastinal abscess (esophagus).

2.1.6. Recurrent/progression

NEC is a very rare disease, and most reports of cases of surgery coincide with the case reports. According to the review by Arai et al., out of the 55 patients with NEC with gastric primary lesion who underwent surgical resection, only three patients survived for two years or more[25]. According to the report by Fischer et al., the MST of 13 patients with NEC with pancreatic primary lesion who underwent surgical resection was 11.7 months (seven of the patients showed distal metastasis)[26]. The form of recurrence among surgery cases remains unknown. The multicenter joint

study by Yamaguchi and Machida reported PFS among cases that underwent systemic chemotherapy with respect to the median as 5.8 months for esophageal primary lesions, 4.8 months for gastric primary lesions, 7.3 months for duodenal small intestine primary lesions, 3.7 months for colonic primary lesions, 3.2 months for pancreatic primary lesions, and 4.1 months for hepatic/biliary primary lesions[10]. In the NORDIC NEC study, the median PFS was 3 months for esophageal primary lesions, 5 months for gastric primary lesions, 3 months for colonic primary lesions, 4 months for rectal primary lesions, 5 months for pancreatic primary lesions, and 4 months for cases with unknown primary lesion site [11].

2.1.7. Prognostic/predictive factors

When prognostic factors were examined by multivariate analysis in the multicenter joint study by Yamaguchi and Machida, out of the various factors such as sex, age (younger or older than 60 years), PS (0 or 1 vs. 2 or more), primary lesion organ (gastrointestinal primary lesions vs. hepatobiliary or pancreatic primary lesions), LDH levels (below vs. above the upper limit of facility standard), presence or absence of liver metastasis, presence or absence of history of radical resection and treatment regimen (IP therapy vs. EP therapy); the independent prognostic factors identified were gastrointestinal primary lesion (vs. hepatobiliary or pancreatic primary lesion, hazard ratio (HR): 0.58), and LDH levels being below the upper limit of facility standard for LDH (vs. above the upper limit of facility standard for LDH, HR: 0.65).

Although IP therapy exhibited slightly better impact on overall survival as compared to EP therapy with an HR of 0.8, the *p*-value of 0.389 meant that there was no significant difference between the two therapies[10]. The NORIDIC NEC study reported poor PS, colonic primary lesions, high platelet count, and high LDH levels to be the main factors behind poor prognosis[11]. However, these prognostic/predictive factors were not obtained with a global consensus.

2.1.8. Rationale for selection of the target population

The objective of this study is to develop a primary chemotherapy regimen for non-resectable/recurrent NECs, and out of the non-resectable or postoperatively recurrent NECs with gastrointestinal primary lesions or hepatobiliary or pancreatic primary lesions (shaded parts in Table 2.1.2 of WHO 2010 classification of NEC), we established chemotherapy-naïve patients to be the target population.

In terms of whether or not to consider MANEC a target of this study, a discussion is needed from the viewpoint of standard treatment. MANEC is considered when 30% or more of the carcinoma consists of adenocarcinoma components, and it is treated as either adenocarcinoma or NEC by discretion of the attending physician, with no real consensus on standard treatment. MANEC was however, excluded from this study, as its disease concept is different from NEC in terms of the standard treatment, which has a consensus regarding the treatment by a “regimen according to small cell lung carcinoma”. In fact, a questionnaire was provided to the three groups participating in this JCOG study (response obtained from 52 facilities). The results showed that 79% of facilities considered the WHO 2010 classification of NEC as the appropriate tissue type for this study, while only 13% responded that consideration of NEC+MANEC would be appropriate. Therefore, majority of facilities considered “WHO 2010 classification of NEC as the appropriate target of this study”.

While diagnosis of NECs is generally carried out using tissue samples and biopsy samples, as mentioned in 2.1.3, there is no consensus methodology to strictly distinguish NEC and MANEC using biopsy samples. As a result of group discussions, a consensus was reached between the three groups that cases presenting with NEC components by biopsy sample-based diagnosis can be enrolled. Although the use of this method would mean that a certain percentage of patients with MANEC, who were not intended to participate in this study, would be enrolled however, currently there are no appropriate means to avoid this. Therefore, we decided to create a consensus for the future based on information obtained from this study.

1) Reason for targeting gastrointestinal/hepatobiliary or pancreatic primary lesion NEC

Various guidelines recommend treatment regimens suited for small cell lung carcinoma, regardless of the organ of primary lesion, and is the rationale showing the validity of the treatment development for NEC across many organs. Furthermore, taking into consideration the frequency of the disease, it is unrealistic to develop treatments according to each organ, and in practice the Minnie-Pearl Cancer Research Network Study (mentioned later in 2.2.2), which is a relatively large-scale clinical study of NEC, development of treatment was carried out in a cross-organ

manner[27].

On the other hand, if we take hypothesize that we do not sufficiently understand how the clinical presentations of the disease vary according to the organ, the fact that this is the first randomized study in Japan, and that the maintenance of foundation for clinical studies of this disease in Japan is inadequate at present time, the hurdle is too high to plan a study across all organs including the fields of gynecology and urology. Based on the above consideration, we have decided to target all gastrointestinal organs which have a relatively large number of common points between each other in terms of clinical presentation and types of complication.

According to the multicenter observatory study by Yamaguchi and Machida, the prognosis of NEC with hepatobiliary or pancreatic primary lesion was significantly poorer than that of NEC with gastrointestinal primary lesion (MST: 7.9 vs. 13.0 months, respectively), but we determined that it is possible to accurately evaluate the efficacy of the treatment regimen by randomizing the subjects using the primary lesion site (gastrointestinal vs. hepatobiliary or pancreatic) as an allocation adjustment factor. Therefore, we considered patients with both, gastrointestinal/hepatobiliary or pancreatic primary lesions as one target population of this study, and to examine differences between organs exploratively by subgroup analysis.

2) Reason for including hepatic NEC cases (hepatic primary lesion or liver metastasis with primary lesion site unknown)

Even among NECs, cases with hepatic primary lesions are particularly rare. There are no specific reports on the frequency of incidence and differences from the other organs and reports are limited only to case reports and their reviews. Moreover, since the liver is a major organ for metastasis of primary lesions into other organs, even when tumors have been clinically identified in the liver, in many cases the primary lesion tends to be in another organ. Therefore, liver should not be deemed the primary lesion site without sufficient examination for a primary lesion. On the other hand, although very rare, there have been reports of NEC with hepatic primary lesion[28]. Even if sufficient search for primary lesion leads to no indication of the same outside of the liver, currently it is not possible to distinguish whether it is a case of "NEC with hepatic primary lesion" or "lesion in the liver is a metastatic lesion, with the primary lesion unknown". In this study, such situations wherein "liver alone has identifiable lesions" would be henceforth, referred to as "hepatic NEC (hepatic primary lesion or liver metastasis from an unknown primary lesion site) for convenience. As the NCCN Guidelines[19] and ENETS Guidelines[20, 21] indicate the usefulness of FDG-PET for detailed examination of the primary lesion, we would also examine the primary lesion using FDG-PET. Furthermore, by referring to the diagnostic procedures for cancers with unknown primary lesion site, detailed examinations into the primary lesion would be carried out by otolaryngological (head and neck) examination and urological examination for men only, and gynecological examination for women only).

Such hepatic NEC (hepatic primary lesion or liver metastasis from an unknown primary lesion site) is anticipated even from an anatomical viewpoint, since the tumor-related complications are the same as that for other gastrointestinal primary lesion NECs, and there is no issue with treating them the same as for gastrointestinal primary lesions.

3) Reason for orienting the study for non-resectable or recurrent cases

As mentioned in 2.1.4 "Standard treatment according to disease staging and outline of prognosis", systemic chemotherapy is indicated for cases with distal metastasis or recurrent cases. Locally advanced cases are particularly prone to pancreatic or bile duct primary lesions situated in the vicinity of vital vessels, and while the NCCN Guidelines propose chemoradiotherapy according to treatment for small cell lung carcinoma[19], the ENETS Guidelines provide no clear indication[21]. In reality, for the treatment of NEC which has a variety of primary lesion organs (scope of irradiation) there is not enough information available for the chemotherapy regimen that should be combined in terms of its optimum dose, the radiation dose, efficacy, and safety. For this reason, chemoradiotherapy cannot be considered the standard treatment with consensus, and systemic chemotherapy for distal metastasis is used widely except for pathologies involving frequent stenotic symptoms when the primary lesion is in the esophagus. For this reason, this study would enroll non-resectable or recurrent cases that include locally advanced cases.

The section "3.6 Definition of non-resectable NEC" shows the definition of non-resectable cases summarized by referring to the JCOG protocol for clinical studies of systemic chemotherapy (esophagus: JCOG0807, stomach: JCOG1013 or JCOG1002, bile duct: JCOG0805, pancreas: JCOG1106). Furthermore, while radical resection has

been considered for cases of colonic primary lesion adenocarcinoma with liver or lung metastasis, as cases of NEC with distal metastasis are not indicated for resection, we defined non-resectable cases as those in “Stage IV”, and followed this definition for duodenal primary lesions, small intestine primary lesions, and appendix primary lesions. Furthermore, with respect to NEC with esophageal primary lesion, chemoradiotherapy is well-indicated for cases with supraclavicular lymph node metastasis of thoracic esophageal tumor even in Stage IV, and hence were not included in this study.

4) Treatment of draft of new classification of NEC

In recent years, proposals have been made to further differentiate the WHO 2010 classification of NECs (G3 in ENETS) to “Grade 3 proliferative tumors showing the same morphological presentations as NET” and “Grade 3 tumors with strong morphological atypicality (previously classified as poorly differentiated NECs)”[29, 30], or by Ki-67 index of 20–50% and 50% or more[11, 19]. This is yet to be confirmed; however, in this study we would have used the WHO 2010 classification. However, we would have used samples collected for central pathological diagnosis to carry out studies related to classification which exploratively adds grade classification and morphological differentiation levels.

2.2. Standard treatment for target disease

Currently, there is no standard drug treatment for non-resectable/recurrent NECs with efficacy verified by a randomized controlled trial. However, given the similarity between pathological and clinical presentations, treatments according to small cell lung carcinoma have been attempted, and there have been reports of positive treatment outcome in small-scale clinical studies and observational studies.

2.2.1. Standard treatment for small cell lung carcinoma

Cisplatin-based multidrug combined therapy is the standard first-line treatment of extensive disease (ED) small cell lung carcinoma. While cyclophosphamide/doxorubicin/vincristine (CAV therapy) was established as a standard treatment in the 1970s in Europe and U.S., the etoposide/cisplatin combined therapy (EP therapy) was introduced in the late 1980s. A comparative study of EP therapy and CAV therapy did not show superiority of EP therapy over CAV therapy in terms of survival, but the response rate (CAV: 51% vs. EP: 61%) and MST (CAV: 8.3 months vs. EP: 8.6 months) were almost the same, and the lighter toxicity level (mucositis, interstitial pneumonia, hemotoxicity) meant that EP could be used as a standard treatment[31]. Thereafter, the JCOG Lung Cancer Group carried out the “comparison between EP therapy and Irinotecan/Cisplatin combined therapy (IP therapy) for Extensive-stage Small Cell Lung Cancer (JCOG9511)”, and reported that the IP therapy provided a significantly better overall survival, with MST being 9.4 months vs. 12.8 months ($p = 0.002$) for IP therapy[32]. However, the two additional large-scale studies conducted primarily in U.S. did not show superiority of IP therapy over EP therapy, and for this reason IP therapy has not been not used as the standard treatment overseas[33, 34]. Furthermore, while the results of the “Randomized controlled trial to verify the non-inferiority of amrubicin/cisplatin combined therapy (AP therapy) over IP therapy (JCOG0509)” were reported in the 2012 conference of the American Society of Clinical Oncology (ASCO), but AP therapy was still considered significantly inferior to IP therapy[35]. Presently, the JCOG Lung Cancer Group considers IP therapy to be the standard treatment for extensive-disease small cell lung carcinoma.

2.2.2. Standard treatment for extrapulmonary NEC

While there are scattered reports relating to use of CAV therapy, EP therapy, IP therapy, and other multidrug combined therapies for extrapulmonary NEC, these reports are primarily from observational studies. Since these reports precede the unification of concept and classification methods of this disease, the nomenclature such as NEC (anaplastic type), NET (poorly differentiated type) and extrapulmonary small cell lung carcinoma have still been used. These reports, however, are presumed to be targeting almost the same disease group as NEC. There are no reports of prospective studies using EP therapy for extrapulmonary NEC. Observational studies have reported response rates between 42–67%, and MST between 15–19 months (Table 2.2.2a). On the other hand, IP therapy has reported results from observational studies and clinical studies, with response rates between 7–83% and MST of 10.1–22.6 months (Table 2.2.2b). As mentioned above, reports related to extrapulmonary NEC are limited to use of EP therapy and IP therapy in observational studies and small-scale clinical studies. The largest number of case enrollments in a clinical study of extrapulmonary NEC was seen in the Minnie-Pearl Cancer Research Network Study, which evaluated the efficacy of triple-drug combined therapy using carboplatin/etoposide/paclitaxel. The

response rate to this triple-drug combined therapy was 53%, while the MST was 14.5 months and adequate, there was intense toxicity involved, and the results were not significantly different from EP therapy, so this regimen was not considered the standard treatment[27].

The 2014 NCCN Guidelines recommend treating extrapulmonary NEC using a regimen according to small cell lung carcinoma, and the guidelines for treatment of small cell lung carcinoma mention both EP therapy and IP therapy as recommended regimens.

Therefore, the standard treatment for extrapulmonary NEC is taken as EP therapy or IP therapy.

Table 2.2.2.a. EP therapy for NEC (observational studies)

Reported year/Reporter	Target	N	Response rate	MST (in months)
1991/ Moertel[18]	Pancreatic/gastrointestinal NEC (anaplastic type)	18	67%	19
1999/ Mitry [36]	Pancreatic/gastrointestinal NEC	41	42%	15
2001/ Marie-Louise [37]	Pancreatic NET (well: 11, poorly: 4) Gastrointestinal cartinoid: 21	33	18%	19
1994/ Lo Re G [38]	Extrapulmonary SCLC	13	69%	NE
2010/ Iwasa [39]	Hepatobiliary or pancreatic NEC	21	14%	7.3
2012/ Yamaguchi [10]	Gastrointestinal NEC	12	75%	14
2012/ Yamaguchi [10]	Hepatobiliary or pancreatic NEC	34	12%	6.9
2012/ Sorbye [11]	Gastrointestinal primary lesion NEC (including primary lesion unknown)	129	31%	12

Table 2.2.2.b. IP therapy for NEC (shaded are clinical studies, others are observational studies)

Reported year/Reporter	Target	N	Response rate	MST (in months)
2003/ Hou [40]	NEC (gastrointestinal-80%)	18	43%	NE
2005/ Chin [41]	Esophageal NEC	12	83%	14
2011/ Okita [42]	Gastric NEC	12	75%	22.6
2012/ Yamaguchi [10]	Gastrointestinal NEC	142	51%	13.4
2012/ Yamaguchi [10]	Hepatobiliary or pancreatic NEC	18	39%	10.1
2006/ Kulke [43]	Pancreatic/gastrointestinal (including NET G1/G2)	15	7%	11.4
2008/ Mani [44]	Pancreatic/gastrointestinal NEC	20	58%	NE
2008/ Jin [45]	Extrapulmonary NEC	15	67%	11.4.

Table 2.2.2.c. Phase II study of carboplatin/etoposide/paclitaxel triple-drug combined therapy for NEC

Reported year/Reporter	Target (breakdown)	N	Response rate	MST (in months)
2006/ Hainsworth [27]	NEC (Colon: 9, lung: 7, skin: 4, pancreas: 3, gall bladder: 1, thyroid: 1, stomach: 1, esophagus: 1, endometrium: 1, maxillary sinus: 1, prostate: 1, primary lesion unknown: 48)	78	53%	14.5

2.2.3. Standard treatment for gastrointestinal/hepatobiliary or pancreatic primary NEC

Treatments for extrapulmonary NEC have not been developed according to any specific organ, and the standard treatment for gastrointestinal/hepatobiliary or pancreatic NEC is also both, EP therapy and IP therapy. While both treatments are considered standard treatments, since this study considers them both to be study treatments, the anticipated effect and expected adverse reactions from EP therapy and IP therapy have been described in "2.3.2 Study treatment(s) of this study".

Herewith, we describe the current status of the usage of each treatment in Japan and overseas. According to the multicenter joint study by Yamaguchi and Machida, 160 out of 258 patients (62%) who received systemic chemotherapy underwent IP therapy, the most common treatment, followed by EP therapy (46 patients, 18%). Although there is big deviation where 92% of gastrointestinal primary lesion NEC (142/154 patients) were given IP therapy, and 65% of hepatobiliary or pancreatic primary lesion NEC (34/52 patients) were given EP therapy, the present situation shows that treatments are being selected by discretion or preference of the facility/physician.

Furthermore, even with respect to the dosing schedule, the method of administration has not necessarily as per the treatment of small cell lung carcinoma. There is no unified consensus, as some facilities use methods used for treatment of gastric cancer (JCOG9912 regimen), based on the reasoning that they have familiarity with IP therapy. On the other hand, in the NORDIC NEC study the most common treatment used was the EP therapy, which was administered to 129 out of 252 patients (51%) who received systemic chemotherapy, followed by a combined therapy consisting of carboplatin and etoposide therapy (67 patients, 27%). Combined therapy of platinum and etoposide was used often, which is another choice of treatment in Japan[11].

2.3. Rationale for establishment of treatment plan

2.3.1. Drugs

1) Etoposide

Etoposide exerts an antitumor effect by inhibiting topoisomerase II, which catalyzes the untangling of supercoiled DNA strands. Main toxicities include myelosuppression, nausea/vomiting, alopecia, and stomatitis.

2) Cisplatin

It is a complex ion form of the heavy metal platinum which shows anti-tumor effect by cross-linking double-stranded DNA. Currently, it is considered one of the key drugs for NEC treatment, being a central medicine for the treatment of lung cancer due to its synergistic effect with radiation therapy and various drugs, and also due to its low bone marrow toxicity when used alone. Toxicities include nausea/vomiting, nephrotoxicity, and neurotoxicity. Sufficient infusion of cisplatin before and after drug administration is necessary to prevent nephrotoxicity.

3) Irinotecan

Irinotecan is a topoisomerase I inhibitor developed in Japan that inhibits DNA synthesis. The drug is directly converted into the active metabolite (SN-38) in human liver and various tissues by carboxyesterase. As it shows a potent antitumor effect against SCLC even when used on its own, this drug is used very often in routine clinical practice to treat NEC as well. Diarrhea and myelosuppression are observed as dose-dependent toxicities. Nausea/vomiting and interstitial pneumonia have also been noted in patients.

2.3.2. Study treatment(s) of this study

1) Etoposide/cisplatin combined therapy (EP therapy)

In the multicenter joint study performed by Yamaguchi and Machida, the response rate of gastrointestinal primary lesion NEC to EP therapy was 75% (9/12 patients), with an MST of 14 months. The response rate of hepatobiliary and pancreatic primary lesion NEC to EP therapy was 12% (4/34 patients), and the MST was 6.9 months. With respect to safety, the multicenter joint study by Yamaguchi and Machida showed no treatment-related deaths associated with EP therapy (N = 46) administered as first-line treatment for gastrointestinal/hepatobiliary and pancreatic primary lesion NEC, and toxicity-related study discontinuation was observed in 6.5% of cases. Details of toxicities remain unknown as they were not investigated. The observational study of EP therapy for hepatobiliary and pancreatic primary lesion NEC (N = 21) carried out by Iwasa et al. at the National Cancer Center Hospital showed major Grade 3/4 adverse events to be neutropenia (90%), nausea (33%), and anorexia (24%). Grade 3 febrile neutropenia was observed in 8 patients (38%)[39]. Table 2.3.2 shows the toxicity profile of EP therapy from the JCOG9511 study which treated small cell lung carcinoma (Grade 3 or higher as per JCOG toxicity criteria).

Table 2.3.2. Toxicity of EP therapy and IP therapy in studies treating small cell lung carcinoma※
※Report by Iwasa et al., JCOG0509：CTCAE v3.0 Grade 3 or higher, JCOG9511：JCOG toxicity criteria Grade 3 or higher

	EP therapy		IP therapy	
	Report by Iwasa et al.[34]	JCOG9511[27]	JCOG9511[27]	JCOG0509[30]
Neutropenia	90%	92.2%	65.3%	58.5%
Leukopenia	71%	51.9%	26.7%	22.5%
Decreased hemoglobin	29%	29.9%	26.7%	23.2%
Thrombocytopenia	24%	18.2%	5.3%	2.1%
Diarrhea	0%	0%	16%	7.7%

Nausea	33%	6.5%	13.3%	6.3%
Aspartate aminotransferase increased	19%	2.6%	0%	-
Alanine aminotransferase increased	24%	3.9%	4%	-
Blood bilirubin increased	19%	0%	0%	-
Creatinine increased	0%	0%	0%	-
Peripheral motor neuropathy	0%	0%	0%	-
Febrile neutropenia	38%	0%	0%	1.4%

There are several reports concerning the specific dosage schedule/administered dose for EP therapy, and four dosing methods have been described in the NCCN Guidelines for SCLC as well. However, it is not clear as to which of the dosing methods is the best, and the dosing method for NEC varies between reports. In Japan, the dosing for EP therapy according to JCOG9511 is also often used for NEC. For this reason, this study would also follow the same method of administration. In terms of the number of cycles, a comparison of 4 courses vs. 8 courses of combined chemotherapy including cyclophosphamide for SCLC showed poor efficacy even when the treatment continued for long-term[46], and treatment of 4 cycles has also been specified in JCOG9511. There have been no such comparative studies for gastrointestinal/hepatobiliary and pancreatic primary lesion NEC, rather, there is no evidence to suggest that first-line chemotherapy should be discontinued if the chemotherapy is efficacious and toxicity is within the permitted scope. Unlike SCLC, NEC does not respond well to chemotherapy and tends to re-exacerbation to the underlying disease soon after the end of chemotherapy. For this reason, continuation of chemotherapy is very likely to be beneficial for patients with NEC as long as the treatment is effective.

While peripheral motor neuropathy, hearing impairment, and renal disorder are known accumulation toxicities of cisplatin, if sufficiently safe, it is ideal for treatments to continue as much as possible, considering the very few treatment options available for NEC. Based on the above rationale, we decided to ensure safety by establishing strict protocol treatment discontinuation criteria and chose not to limit the total dose or prescribe the number of treatment cycles.

2）Irinotecan/Cisplatin combined therapy (IP therapy)

In the multicenter joint study by Yamaguchi and Machida, the response rate of gastrointestinal primary lesion NEC to IP therapy was 51% (73/142 patients), with an MST of 13.4 months. The response rate of hepatobiliary and pancreatic primary lesion NEC to IP therapy was 39% (7/18 patients), and the MST was 10.1 months. There were no treatment-related deaths among 160 patients, and discontinuation due to toxicity in first-line chemotherapy was seen in 11.2% of cases. Details about toxicity were not collected. As a reference, Table 2.3.2 shows the toxicity profile of IP therapy from the JCOG9511 and JCOG0509 studies which treated small cell lung carcinoma.

There are several reports concerning specific dosing methods for IP therapy, and two dosing methods have been described in the NCCN Guidelines for SCLC as well. It is not known which of the dosing methods is the best, and the dosing method has also been variable in the NEC. In Japan, the dosing method of IP therapy, according to JCOG9511 is considered the standard treatment for SCLC and the same method has also been used in this study.

The total dose and number of cycles would have not been prescribed for the same reason as EP therapy, and safety has been ensured by establishing strict protocol treatment discontinuation criteria.

3）Other study treatment candidates

Presently, there are no drugs or treatment methods, including molecular-targeted drugs, which surpass the EP and IP therapy. There are also no planned or ongoing large-scale clinical trials globally, related to first-line treatment of NEC.

As the second-line treatment, a phase II study of Everolimus is underway for pancreatic primary lesion NEC resistant or unresponsive to platinum preparations. It is a multicenter study being carried out in 31 facilities in Japan, led by the National Cancer Center East Hospital, through the cancer research and development fund (UMIN000012752）.

2.3.3. Summary of risk/benefit balance of the standard treatment and study treatment

In the multicenter joint study by Yamaguchi and Machida, IP therapy had better prognosis compared to EP therapy (MST: 13.0 months vs. 7.3 months; $p < 0.0001$). However, as shown in Table 2.3.3, since IP therapy was more frequently chosen for gastrointestinal primary lesions (142/154 patients) and EP therapy for hepatobiliary and

pancreatic primary lesions (34/52 patients), it has not been possible to determine whether this difference is due to difference in efficacy between the regimens, or due to differences in the primary lesion organ. Primary lesion organ remained a significant prognostic factor after multivariate analysis, and while IP therapy turned out to be a slightly better regimen than EP therapy with HR = 0.8 (95% CI. 0.48–1.33), $p = 0.389$ indicated that there was no significant difference between the two (see “2.1.5 Prognostic/predictive factors”). Taking into account the number of EP therapy cases (46 patients), the fact that it was an observational study, and including other unknown bias, the data interpretation is fairly limited. Therefore, based on these results we are unable to deem either of IP or EP therapies to be more efficacious than the other.

With regard to toxicity, while myelosuppression such as neutropenia is milder in IP therapy than EP therapy, frequencies of events such as diarrhea and nausea are high. While the two treatments have different toxicity profiles, it is difficult to conclude that one has clearly worse toxicity than the other. The risk of bile duct obstruction is high in biliary and pancreatic primary lesion NECs and the use of irinotecan (which undergoes biliary excretion) in patients exhibiting biliary excretion disorder runs the risk of increased toxicity, hence care must be taken while administering irinotecan. We believe that this risk can be avoided by appropriate pre-treatment drainage and proper monitoring during treatment. Based on the above, it is difficult to assign superiority to IP therapy or EP therapy over the other in terms of risk/benefit balance, and both the regimens can be considered suitable standard treatments.

Table 2.3.3. Summary of results from multicenter study by Yamaguchi and Machida

		IP	EP	*p*-value*
Total	N	160	46	
	Response rate (%)	50	27	＜0.001
	Median PFS (in months)	5.2	4.0	0.033
	Median OS (in months)	13.0	7.3	＜0.0001
Gastrointestinal primary lesion NEC	N	142	12	
	Response rate (%)	51	75	0.14
	Median PFS (in months)	5.4	4.9	0.585
	Median OS (in months)	13.4	14.0	0.976
Hepatobiliary and pancreatic primary lesion NEC	N	18	34	
	Response rate (%)	39%	12%	0.034
	Median PFS (in months)	4.4	3.7	0.056
	Median OS (in months)	10.1	6.9	0.05

*Response rate by chi-squared test, PFS and OS by log-rank test

2.3.4. Post-treatment(s)

In the multicenter joint study by Yamaguchi and Machida, 56% (116/206 patients) of patients with NEC who received IP therapy or EP therapy were administered chemotherapy as the second-line of treatment. The most common second-line chemotherapy after IP therapy was amurcibin (22/88 patients), while irinotecan was the most common second-line treatment after EP therapy (13/28 patients), and we expected a similar pattern for the second-line treatments in this study as well. The overall performance of second-line chemotherapy was poor with a response rate of 11%, and PFS of 2.1 months. As such, the significance of the second-line treatment has not been indicated, and hence, no standard treatment has been established.

2.4. Study design

2.4.1. Rationale for establishing endpoints

For NECs, an alternative endpoint for overall survival, such as PFS has not been established as an indicator for comparing the usefulness of the treatment regimens. Therefore, comparison of overall survival was considered appropriate when examining the therapeutic effects. For the same, overall survival was examined as the primary endpoint. The secondary endpoints to evaluate the efficacy and safety were the response rate (only for patients with a measurable lesion), progression-free survival, incidence rate of adverse events, and dose intensity of cisplatin.

2.4.2. Clinical hypothesis and rationale for setting the number of enrollments

This study compares the two standard treatments, both of which are difficult to deem superior than the other in terms of efficacy and safety. We have, therefore, adopted a study design using bilateral testing. The main clinical hypothesis of this study is that either IP therapy or EP therapy is likely to provide better overall survival than the other treatment group, and should this hypothesis be validated, the superior treatment with statistical significance would be deemed the better treatment, and would therefore be positioned as the standard treatment in future. If the study fails to validate this hypothesis, it would indicate that there was no clinically significant difference in overall survival between the two groups, and if there are no large differences in terms of toxicity, we would conclude that both treatments are viable options as the standard treatment. However, if unlike our initial hypothesis, one treatment shows clearly more toxicity than the other, we would reconsider the study design before carrying out the main analysis.

Furthermore, as mentioned in “section 2.1.1 Epidemiology”, gastroenterological primary lesion NEC, which is the primary target of this study, is a very rare disease with an annual prevalence of around 3 in 100,000 people, hence, the significance level of testing has been placed at 10% bilaterally, instead of 5% bilaterally. In the multicenter joint study conducted by Yamaguchi and Machida, the MST of gastrointestinal/hepatobiliary or pancreatic primary lesion NEC was 11.5 months. To determine if one treatment is superior to the other, we expected a difference of four months in terms of MST. If we expect the more inferior treatment to have an MST of 8.0 months and the superior treatment to have an MST of 12.0 months, the number of enrollments are calculated using the aforementioned parameters and based on the discussion mentioned later (see “12.2 Expected number of enrollments/Enrollment period/Follow-up period”). This provided a research period of 7- years, consisting of 6-years of enrollment period and one year of follow-up period, and with $\alpha = 0.1$ bilaterally and detection power of 70% to detect differences between the two groups, 63 subjects were required per group. Taking into consideration some subjects who could be lost post follow-up, we aimed to enroll 70 subjects per group, and a total of 140 subjects between two groups.

If enrollment proceeds better than expected than prior to the start of the study (i.e. if the number of enrolled subjects reach 70 in less than 2.5 years from the start of enrollment), the number of enrollments is likely to be re-established by changing the detection power from 70% to 80% during the study, with the aim of obtaining more accurate results.

2.4.3. Expected patient enrollment

In the multicenter joint study by Yamaguchi and Machida, 258 cases of gastrointestinal/hepatobiliary or pancreatic primary lesion NECs were reported from 23 sites during the 11 years between 2000 and 2011, but between 2000 and 2006, information could not be obtained because of old cases. When limited to the most recent five years (2006–2011), when sufficient information was obtained from each facility, the number of enrollments was placed at162 subjects.

This is a joint study between three groups, namely the JCOG Hepatobiliary and Pancreatic Oncology Group, JCOG Gastric Cancer Group, and the JCOG Esophageal Cancer Group. Therefore, the total number of facilities, excluding overlaps, amounted to 82 facilities. Compared to the multicenter joint study by Yamaguchi and Machida, we expect an increase in the number of patients enrolled, and simple calculations lead us to extrapolate 115 subjects to be enrolled annually. On the other hand, if we take into account the possibility of patient enrollments being biased to some high volume centers, it would be difficult to estimate the effect of increasing facility count to proceed. In addition, considering that the study is a randomized, we estimate the annual number of patients enrolled to be between 30–50 people. Taking into account ineligible cases, we provided 6-years for patient enrollment period.

2.4.4. Rationale for setting allocation adjustment factors

1) Facility

It is widely known that background, treatment, efficacy evaluation, and safety evaluation of enrolled patients vary depending on the facility, and JCOG standards have been used to make adjustments between facilities.

2) Primary lesion organ (gastrointestinal tract vs. hepatobiliary or pancreatic organ)

In the multicenter joint study by Yamaguchi and Machida, multivariate analysis using the Cox proportional hazard model was carried out with 183 subjects, which indicated primary lesion organ (gastrointestinal tract vs. hepatobiliary or pancreatic organ) to be a significant prognostic factor.

2.4.5. Centralized pathological diagnosis

In this study, a centralized pathological diagnosis would be performed for analysis of NEC, despite patients being diagnosed by a third party. The operation of the centralized pathological diagnosis is described in section 15.1, while details of the operation have been described in the Centralized Pathological Diagnosis Procedure Manual. Furthermore, an analysis of endpoints based on the results of centralized pathological diagnosis would be provided as a reference.

2.5. Summary of expected advantages and disadvantages associated with study participation

2.5.1. Expected advantages

Drugs used for both groups of this study are treatments used in routine medical practice. As mentioned in the next section, although it would be necessary to use some drugs for which insurance coverage does not apply, since insurance claims for these treatments are made in a similar manner to general medical care however, in practice they do not receive insurance assessments.

Moreover, since the medical fees of study participants during the study, which includes drug fees, are paid in principle by the patients themselves and their insurance coverage, so the patients would receive no special medical or financial benefits from participation in this study.

2.5.2. Expected risks and disadvantages

Both treatment arms A and B would receive chemotherapy regimen used in routine medical practice, so they would be unlikely to be exposed to special risks or disadvantages not observed in routine medical practice. The descriptions in “2.3.3 Risk/benefit balance of standard treatment and study treatments” outline the expected risks and disadvantages for each treatment arm.

To minimize the risk of adverse events and disadvantages, the “ Patient selection criteria (Section 4)”, “ Criteria to change treatment (Section 6.3)” and “Concomitant/Supportive therapies (Section 6.4)” have been carefully considered for the three groups. Furthermore, JCOG clinical trials require periodic monitoring twice a year after the start of the trial and the Institutional Review Board would monitor if the adverse events are within the scope of expectation, while any serious adverse events or unexpected adverse events would be carefully examined and reviewed according to the provisions related to “JCOG Guidelines for Handling Clinical Safety Information”, and a system has been provided to take any necessary countermeasures.

Precautions related to etoposide, irinotecan, and cisplatin

As of December 2013, etoposide therapy, which is the intended to be used in this study had not received insurance approval for the treatment of gastrointestinal or hepatobiliary and pancreatic primary lesion cancers. Irinotecan has also not received insurance approval for treatment of cancers with primary lesions in organs other than the stomach, colon, and rectum. Furthermore, as cisplatin is only indicated for esophageal, gastric cancer, and for biliary tract cancers when combined with gemcitabine (25 mg/m^2), the dosage and administration used in this study have not been approved previously.

As each facility in this study may carry out the insurance claims in the same manner as routine general medical practice, the treatment may receive insurance assessment after-the-fact. However, if a facility incurs losses, the loss must be borne by the relevant facility (medical institution), as there is no system of compensation arranged by the JCOG Research Organization. If actual losses occur, the continued participation in the study would then be carefully discussed between the facility supervisor and the principal investigator/clinical trial secretariat. The facility

supervisor would be expected to gain approval from the facility IRB and the head of the medical institutions.

2.6. Significance of this study

While both, EP therapy and IP therapy are standard treatments, the efficiency of either treatment being better than the other is not yet clear, and currently in routine medical practice, the choice of treatment is being made on the discretion or preference of the facility/attending physician.

If this study is able to clearly indicate the efficiency of EP therapy or IP therapy over the other, we expect that the diminished use of the relatively inferior treatment would contribute to improvement in patient prognosis. If the study indicates no clinically meaningful differences between the two treatments, the results would provide evidence that there is no significant issue with the treatments being chosen by discretion or preference of the facility/attending physician, which has been done traditionally without any data that directly compare the two treatments, although that would not indicate that the effects of the two treatments are equivalent. We also expect that the establishment of a highly reliable standard treatment in this study which would provide a foundation for therapeutic development when promising treatment regimen in the future.

As NEC is a rare disease, and since each clinician is not likely to have adequate experience, we believe that work associated with multicenter studies such as confirmation of diagnosis by centralized diagnosis/results feedback and sharing results of treatments by unified treatment regimen would lead to improvement of quality of care for NEC in Japan.

2.7. Associated research (including sample analysis research)

No such studies have been planned or carried out at the time of preparation of the protocol.

2.8. JCOG-Biobank Japan (BBJ) collaborating biobank

omit

3. Criteria/definitions used in this study

Tissue classification would be performed according to WHO 2010 classification and ENETS (European Neuroendocrine Tumor Society) classification[14, 15], while disease staging would be done according to "UICC-TNM 7th Edition".

3.1. Tissue classification (WHO 2010 classification)

The shaded parts are the targets of this study

Neuroendocrine neoplasms

1) Neuroendocrine tumor: NET Grade 1 (NETG1)
2) Neuroendocrine tumor: NET Grade 2 (NETG2)
3) Neuroendocrine carcinoma: NEC (large cell or small cell type)
4) Mixed adenoendocrine carcinoma (MANEC)
5) Hyperplastic and preneoplastic lesions

3.2. Grade classification (ENETS [European Neuroendocrine Tumor Society] / WHO2010 classification)

Grade 1 (G1)	Number of mitotic presentations < 2 per 10 high power fields (HPF) and/or Ki-67 index ≤2%*
Grade 2 (G2)	Number of mitotic presentations 2–20 per 10 HPF and/or Ki-67 index 3-20%
Grade 3 (G3)	Number of mitotic presentations > 20 per 10 HPF and/or Ki-67 index >20%

*The description of number of mitotic presentations in WHO 2010 classification is ≤2, but 2–3% are classified to G1[47].

3.3. Histopathological diagnosis

- Immunostaining (Chromogranin A and synaptophysin) is essential for a pathological diagnosis of NEC.
- Either1. or 2., or both are studied to determine proliferative activity. Number of mitotic presentations and Ki-67 index would adopt a high grade evaluation.
  1. Number of mitotic presentations (to evaluate 50HPF)
  2. Ki-67 index (500–2000 tumor cells*)

  *(Only in this study, if the number of tumor cells in the collected sample is less than 500, the number of tumor cells measured is also listed. A minimum of 100 tumor cells is required.)
- If sufficient amount of biopsy sample cannot be collected for a pathological diagnosis, a cell block prepared using material obtained from EUS-FNA, brushing or needle biopsy may be used. However, the use of a cell block prepared from ascites or pleural effusion is not permitted.
- During a histological diagnosis using a resected tumor sample, a slide with a representative section shall be used to evaluate the proportion of NEC components (differential diagnosis with mixed adenoendocrine carcinoma). For a comprehensive examination, the entire tumor must be evaluated.

For further details NEC Pathological Diagnosis Handbook would be referred (posted on JCOG website).

3.4. Disease stage classification criteria (UICC-TNM 7th Edition)

Special notice regarding disease stage classification: Although there is an independent TNM classification for NET G1 and NET G2 with primary lesions in the stomach, small intestines, and colon; since this study concerns NEC, the disease would be classified according to the classification method of major tissue type in each primary lesion organ (squamous cell carcinoma for esophagus, and adenocarcinoma for all other primary lesion organs). In this study, the classification of intrahepatic cholangiocarcinoma is used for hepatic NEC, due to their similar clinical presentation.

3.4.1. Esophagus

T –Primary tumor

TX: Primary tumor cannot be evaluated
T0: Primary tumor unidentified
Tis: Epithelial carcinoma/highly dysplastic
T1: Tumor invading mucosal lamina propria, muscularis mucosae, or submucosa
  T1a: Tumor invading the mucosal lamina propria or muscularis mucosae
  T1b: Tumor invading the submucosa

T2 : Tumor invading the muscularis propia

T3 : Tumor invading the adventitia

T4 : Tumor invading the surrounding tissue

T4a : Tumor invading the pleura, pericardium, and diaphragm

T4b : Tumor invading other surrounding tissues such as the aorta, centrum, trachea

N –Regional lymph nodes

NX : Regional lymph node metastasis cannot be evaluated

N0 : No regional lymph node metastasis

N1 : 1–2 foci of regional lymph node metastases

N2 : 3–6 regional foci of lymph node metastases

N3 : 7 or more foci of regional lymph node metastases

M –Distal metastasis

M0 : No distal metastasis

M1 : Distal metastasis present

Stage	T	N	M
**0**	Tis	N0	M0
**IA**	T1	N0	M0
**IB**	T2	N0	M0
**IIA**	T3	N0	M0
**IIB**	T1, T2	N1	M0
**IIIA**	T4a	N0	M0
	T3	N1	M0
	T1, T2	N2	M0
**IIIB**	T3	N2	M0
**IIIC**	T4a	N1、N2	M0
	T4b	Unrelated to N	M0
	Any T	N3	M0
**IV**	Any T	Any N	M1

3.4.2. Stomach

T –Primary tumor

TX : Primary tumor cannot be evaluated

T0 : Primary tumor unidentified

Tis : Epithelial carcinoma: Epithelial carcinoma/highly dysplastic carcinoma not invading the lamina propia mucosa

T1 : Tumor invading mucosal lamina propria, muscularis mucosae, or submucosa

T1a : Tumor invading the mucosal lamina propria or muscularis mucosae

T1b : Tumor invading the submucosa

T2 : Tumor invading the muscularis propia

T3 : Tumor invading the subserosa

T4 : Tumor perforating the serosa, or invading adjacent structures[1,2,3]

T4a : Tumor perforating the serosa

T4b : Tumor invading adjacent structures[1,2,3]

1. Adjacent organs of the stomach are spleen, transverse colon, liver, diaphragm, pancreas, abdominal wall, adrenal glands, kidneys, small intestine, and retroperitoneum.
2. If the invasion has spread from the stomach to the duodenum or esophagus, classification is made in terms of depth.
3. A tumor that advances into the gastrocolic ligament, into the hepatogastric ligament, or into the greater or lesser omentum, and is classified as T3, when there is no perforation of the visceral peritoneum.

N –Regional lymph nodes

NX : Regional lymph node metastasis cannot be evaluated
N0 : No regional lymph node metastasis
N1 : 1–2 regional foci of lymph node metastases
N2 : 3–6 regional foci of lymph node metastases
N3 : 7 or more regional foci of lymph node metastases
N3a : 7–15 regional foci of lymph node metastases
N3b : 16 or more regional foci of lymph node metastases

M –Distal metastasis
M0 : No distal metastasis
M1 : Distal metastasis present

Stage	T	N	M
**0**	Tis	N0	M0
**IA**	T1	N0	M0
**IB**	T2	N0	M0
	T1	N1	M0
**IIA**	T3	N0	M0
	T2	N1	M0
	T1	N2	M0
**IIB**	T4a	N0	M0
	T3	N1	M0
	T2	N2	M0
	T1	N3	M0
**IIIA**	T4a	N1	M0
	T3	N2	M0
	T2	N3	M0
**IIIB**	T4b	N0, N1	M0
	T4a	N2	M0
	T3	N3	M0
**IIIC**	T4a	N3	M0
	T4b	N2, N3	M0
**IV**	Any T	Any N	M1

3.4.3. Small intestines (including duodenum)

T –Primary tumor
TX : Primary tumor cannot be evaluated
T0 : Primary tumor unidentified
Tis : Epithelial carcinoma
T1 : Tumor invading mucosal lamina propria, muscularis mucosae, or submucosa
T1a : Tumor infiltrating the mucosal lamina propria or muscularis mucosae
T1b : Tumor infiltrating the submucosa
T2 : Tumor infiltrating the muscularis propia
T3 : Tumor invading the subserosa, or tumor invading within 2 cm of surrounding tissue of muscularis externa without peritoneal cover (mesenterium, retroperitoneum)*
*Surrounding tissue of muscularis externa without peritoneal cover refers to the mesenterium in the jejunum and ileum, and the retroperitoneum in the duodenum without serosa.
T4 : Tumor penetrating the visceral peritoneum, or tumor directly invading another organ or tissue (invasion of other loops of the small intestine, invasion by 2 cm or more into the mesenterium and retroperitoneum, including invasion into the abdominal wall through the serosa; invasion to the pancreas only for duodenum)

N –Regional lymph nodes
NX : Regional lymph node metastasis cannot be evaluated
N0 : No regional lymph node metastasis

N1 : 1–3 regional foci of lymph node metastases

N2 : 4 or more regional foci of lymph node metastases

M –Distal metastasis

M0 : No distal metastasis

M1 : Distal metastasis present

Stage	T	N	M
**0**	Tis	N0	M0
**I**	T1, T2	N0	M0
**IIA**	T3	N0	M0
**IIB**	T4	N0	M0
**IIIA**	Any T	N1	M0
**IIIB**	Any T	N2	M0
**IV**	Any T	Any N	M1

3.4.4. Appendix (partial revision of UICC-TNM 7th Edition)

T –Primary tumor

TX : Primary tumor cannot be evaluated

T0 : Primary tumor unidentified

Tis : Epithelial carcinoma: Tumor invading epithelium or lamina propia mucosae

T1 : Tumor invading the submucosa

T2 : Tumor invading the muscularis propia

T3 : Tumor invading the subserosa or mesoappendix

T4 : Tumor penetrating the visceral peritoneum, tumor including a peritoneal mucosal tumor in the lower right abdomen and/or tumor directly invading other organs or tissue

T4a : Tumor penetrating the visceral peritoneum, or peritoneal mucosal tumor in the lower right abdomen

T4b : Tumor directly invading other organs or tissues

N –Regional lymph nodes

NX : Regional lymph node metastasis cannot be evaluated

N0 : No regional lymph node metastasis

N1 : 1–3 regional foci of lymph node metastases

N2 : 4 or more foci of regional lymph node metastases

M –Distal metastasis

M0 : No distal metastasis

M1 : Distal metastasis present

M1a : Peritoneal metastasis beyond the lower right abdomen, including pseudomyxoma peritonei

M1b : Distal metastasis other than peritoneal metastasis

Stage	T	N	M
**0**	Tis	N0	M0
**I**	T1, T2	N0	M0
**IIA**	T3	N0	M0
**IIB**	T4a	N0	M0
**IIC**	T4b	N0	M0
**IIIA**	T1, T2	N1	M0
**IIIB**	T3, T4	N1	M0
**IIIC**	Any T	N2	M0
**IVA**	Any T	N0	M1a
**IVB**	Any T	N0	M1a
	Any T	N1, N2	M1a
**IVC**	Any T	Any N	M1b

3.4.5. Colon and rectum

T –Primary tumor

TX : Primary tumor cannot be evaluated

T0 : Primary tumor unidentified

Tis : Carcinoma in situ: Tumor invading epithelium or lamina propia mucosae

T1 : Tumor invading the submucosa

T2 : Tumor invading the muscularis propia

T3 : Tumor invading the subserosa, or tissue surrounding the colon or rectum without peritoneal covering

T4 : Tumor penetrating the visceral peritoneum, and/or directly invading another organ or tissues

T4a : Tumor penetrating the visceral peritoneum

T4b : Tumor directly invading another organ or tissues

N –Regional lymph nodes

NX : Regional lymph node metastasis cannot be evaluate

N0 : No regional lymph node metastasis

N1 : 1–3 regional foci of lymph node metastases

N1a : 1 regional lymph node metastasis

N1b : 2–3 regional foci of lymph node metastases

N1c : Presence of tumor deposits, that is, satellite nodes in the subserosa or in the soft tissue surrounding colon or rectum without peritoneal covering, but with no regional lymph node metastasis

N2 : 4 or more regional lymph node metastases

N2a : 4–6 regional foci of lymph node metastases

N2b : 7 or more regional foci of lymph node metastases

M –Distal metastasis

M0 : No distal metastasis

M1 : Distal metastasis present

M1a : Local metastasis to one organ (liver, lungs, ovaries, or lymph nodes other than regional lymph nodes)

M1b : 2 or more organs, or peritoneal metastasis

Stage	T	N	M
**0**	Tis	N0	M0
**I**	T1, T2	N0	M0
**II**	T3, T4	N0	M0
**IIA**	T3	N0	M0
**IIB**	T4a	N0	M0
**IIC**	T4b	N0	M0
**III**	Any T	N1, N2	M0
**IIIA**	T1, T2	N1	M0
	T1	N2a	M0
**IIIB**	T3, T4	N1	M0
	T2, T3	N2a	M0
	T1, T2	N2b	M0
**IIIC**	T4a	N2a	M0
	T3, T4a	N2b	M0
	T4b	N1, N2	M0
**IVA**	Any T	Any N	M1a
**IVB**	Any T	Any N	M1b

3.4.6. Applies to hepatic NEC (hepatic primary lesion or liver metastasis from unknown primary lesion)

T –Primary tumor

TX : Primary tumor cannot be evaluated

T0 : Primary tumor unidentified

Tis: Carcinoma *in situ*

T1: Isolated tumor without vascular invasion

T2a: Isolated tumor with vascular invasion

T2b: Multifocal tumor regardless of vascular invasion

T3: Tumor penetrating visceral peritoneum or directly invading adjacent extrahepatic structures

T4: Tumor with bile duct invasion (bile duct proliferative type)

N –Regional lymph nodes

NX: Regional lymph node metastasis cannot be evaluated

N0: No regional lymph node metastasis

N1: Regional lymph node metastasis present

M –Distal metastasis

M0: No distal metastasis

M1: Distal metastasis present

Stage	T	N	M
**I**	T1	N0	M0
**II**	T2	N0	M0
**III**	T3	N0	M0
**IVA**	T4	N0	M0
	Any T	N1	M0
**IVB**	Any T	Any N	M1

3.4.7. Gall bladder

T –Primary tumor

TX: Primary tumor cannot be evaluated

T0: Primary tumor unidentified

Tis: Carcinoma *in situ*

T1: Tumor invading mucosal lamina propria or muscularis externa

T1a: Tumor invading mucosal lamina propia

T1b: Tumor invading muscularis externa

T2: Tumor invading the connective tissue around the muscularis externa, but shows no progression beyond the serosa or to the liver

T3: Tumor perforating the serosa (visceral peritoneum), tumor directly advancing to the liver and/or an adjacent organ that is not the liver (stomach, duodenum, colon, pancreas, omentum, or extrahepatic bile duct)

T4: Tumor invading the main trunk of the portal vein or the hepatic artery, or tumor advancing to two or more adjacent organs which are not the liver

N –Regional lymph nodes

NX: Regional lymph node metastasis cannot be evaluated

N0: No regional lymph node metastasis

N1: Regional lymph node metastasis present (cystic duct, common bile duct, proper hepatic artery, including lymph nodes along the portal vein)

M –Distal metastasis

M0: No distal metastasis

M1: Distal metastasis present

Stage	T	N	M
**0**	Tis	N0	M0
**I**	T1	N0	M0
**II**	T2	N0	M0
**IIIA**	T3	N0	M0
**IIIB**	T1, T2, T3	N1	M0
**IVA**	T4	Any N	M0

**IVB**	Any T	Any N	M1

3.4.8. Extrahepatic bile duct-hepatic portal region

T –Primary tumor

TX: Primary tumor cannot be evaluated

T0: Primary tumor unidentified

Tis: Carcinoma *in situ*

T1: Tumor localized to the bile duct which advances until muscularis externa or fibrous tissue

T2a: Tumor invading beyond the bile duct wall and into the surrounding adipose tissue

T2b: Tumor invading the adjacent liver parenchyma

T3: Tumor invading the branch of one side of portal vein or hepatic artery

T4: Tumor invades the main trunk of portal vein, and branches on both sides of the portal vein, proper hepatic artery or the secondary branches of the bile duct on both left and right side, or to the secondary bile duct branches on one side and portal vein or hepatic artery on the other side

N –Regional lymph nodes

NX: Regional lymph node metastasis cannot be evaluated

N0: No regional lymph node metastasis

N1: Presence of regional lymph node metastasis in the cystic duct, common bile duct, proper hepatic artery, and lymph nodes along portal vein

M –Distal metastasis

M0: No distal metastasis

M1: Distal metastasis present

Stage	T	N	M
**0**	Tis	N0	M0
**I**	T1	N0	M0
**II**	T2a, T2b	N0	M0
**IIIA**	T3	N0	M0
**IIIB**	T1,T2, T3	N1	M0
**IVA**	T4	N0, N1	M0
**IVB**	Any T	Any N	M1

3.4.9. Extrahepatic bile duct-distal

T –Primary tumor

TX: Primary tumor cannot be evaluated

T0: Primary tumor unidentified

Tis: Carcinoma *in situ*

T1: Tumor localized in the bile duct wall

T2: Tumor invading beyond the bile duct wall

T3: Tumor invading the gallbladder, liver, pancreas, duodenum, or other adjacent organs

T4: Tumor invading the celiac axis or superior mesenteric artery

N –Regional lymph nodes

NX: Regional lymph node metastasis cannot be evaluated

N0: No regional lymph node metastasis

N1: Regional lymph node metastasis present

M –Distal metastasis

M0: No distal metastasis

M1: Distal metastasis present

Stage	T	N	M
**0**	Tis	N0	M0
**IA**	T1	N0	M0
**IB**	T2	N0	M0

**IIA**	T3	N0	M0
**IIB**	T1, T2, T3	N1	M0
**III**	T4	Any N	M0
**IV**	Any T	Any N	M1

3.4.10. Ampulla of Vater

T –Primary tumor

TX: Primary tumor cannot be evaluated

T0: Primary tumor unidentified

Tis: Carcinoma *in situ*

T1: Tumor localized to the ampulla of Vater, or sphincter of Oddi

T2: Tumor invading the duodenal wall

T3: Tumor invading the pancreas

T4: Tumor invading the soft tissue around the pancreas or other adjacent organs

N –Regional lymph nodes

NX: Regional lymph node metastasis cannot be evaluated

N0: No regional lymph node metastasis

N1: Regional lymph node metastasis present

M –Distal metastasis

M0: No distal metastasis

M1: Distal metastasis present

Stage	T	N	M
**0**	Tis	N0	M0
**IA**	T1	N0	M0
**IB**	T2	N0	M0
**IIA**	T3	N0	M0
**IIB**	T1, T2, T3	N1	M0
**III**	T4	Any N	M0
**IV**	Any T	Any N	M1

3.4.11. Pancreas

T –Primary tumor

TX: Primary tumor cannot be evaluated

T0: Primary tumor unidentified

Tis: Carcinoma *in situ*

T1: Tumor localized within the pancreas, with a maximum size of < 2 cm

T2: Tumor localized within the pancreas, with a maximum size > cm

T3: Tumor advancing outside the pancreas, but with no invasion to the celiac axis or superior mesenteric artery

T4: Tumor invading the celiac axis or superior mesenteric artery

N –Regional lymph nodes

NX: Regional lymph node metastasis cannot be evaluated

N0: No regional lymph node metastasis

N1: Regional lymph node metastasis present

M –Distal metastasis

M0: No distal metastasis

M1: Distal metastasis present

Stage	T	N	M
**0**	Tis	N0	M0
**IA**	T1	N0	M0
**IB**	T2	N0	M0
**IIA**	T3	N0	M0

**IIB**	T1, T2, T3	N1	M0
**III**	T4	Any N	M0
**IV**	Any T	Any N	M1

3.5. Residual tumor (R) classification (UICC-TNM 7th Edition)

RX : Presence of residual tumor cannot be evaluated

R0 : No residual tumor

R1 : Presence of residual tumor by microscopic examination

R2 : Presence of residual tumor by macroscopic examination

3.6. Definition of non-resectable NEC

Unoperated cases for which surgery as a curative process was not indicated based on clinical findings, including image-based diagnosis, or operated cases that underwent resection (including experimental laparotomy) but ultimately ended as R2 resection are considered non-resectable.

The details by organs are shown below. One or more criterion must be fulfilled for each organ.

<Esopghagus>

- Diagnose as Stage IV (UICC 7th edition) based on clinical findings.

<Stomach>

- Diagnose as Stage IV (UICC 7th edition) based on clinical or surgical findings. However, this does not include situations where Stage IV diagnosis is provided based only on peritoneal lavage cytology (CY1).
- Bulky lymph node* metastasis is identified.

  *Bulky lymph node: Two or more lymph nodes with a major axis of 1.5 cm or more, that are present in contact with each other around the celiac artery, the common hepatic artery, the splenic artery, and the proper hepatic artery, or on the front surface of the superior mesenteric vein, where single or multiple large and small lymph nodes have formed an aggregate with the overall major axis length of 3.0 cm or more.

<Duodenum (excluding the ampulla of Vater), small intestines, appendix, and colon>

- Diagnosed as Stage IV (UICC 7th edition) based on the clinical findings.

<Extrahepatic bile duct, ampulla of Vater, gallbladder>

- Distant metastasis is identified
- Para-aortic lymph node metastasis is identified
- Bulky metastasis identified in the hepatoduodenal mesentery and lymph nodes surrounding the head of the pancreas
- Invasion in the proper hepatic artery, common hepatic artery, celiac artery, or the superior mesenteric artery identified
- Invasion in the hepatic artery branches on both the left and right side identified
- Extensive invasion or occlusion of the main trunk of portal vein, or invasion in the portal vein branches on both the left and right side identified
- Invasion in the blood vessels on the lobe on one side of the liver (portal vein or artery) or atrophy of lobe on one side of liver identified, and advancement into the bile duct on the other side is identified up to the secondary branch level
- Advancement into the bile ducts on both sides identified up to the secondary branch level

<Pancreas>

- Diagnosed as Stage III or Stage IV (UICC 7th edition) based on clinical findings.

＜Hepatic NEC (hepatic primary lesion or liver metastasis from unknown primary lesion)>

- Distal metastasis* other than intrahepatic metastasis identified.

  *Limited to organs that cannot be the primary lesion site (bone, lymph nodes below the diaphragm,

peritoneum, subcutaneous, muscle, and spleen) (see 3.8)

- Para-aortic lymph node metastasis is identified
- Bulky metastasis identified in the hepatoduodenal mesentery and lymph nodes surrounding the head of the pancreas
- Multiple masses identified in the liver.
- Invasion in one of the proper hepatic artery, common hepatic artery, celiac artery, or the superior mesenteric artery identified.
- Invasion to hepatic artery branches on both the left and right side identified
- Extensive invasion or occlusion of the main trunk of portal vein, or invasion in portal vein branches on both the left and right side identified
- Invasion in blood vessels on lobe on one side of the liver (portal vein or artery) or atrophy of lobe on one side of liver identified, and advancement into the bile duct on the other side is identified up to the secondary branch level
- Advancement into the bile ducts on both sides identified up to the secondary branch level

3.7. Definition of recurrent NEC

If surgery (R0 resection or R1 resection) was performed for gastrointestinal/hepatobiliary or pancreatic primary lesion, malignant tumor and the condition was diagnosed as NEC based on pathological samples taken from surgery, and were deemed as a clinical relapse thereafter.

If there are past histological samples from the primary lesion, no histological biopsy of the relapse lesion is necessary.

3.8. Definition of hepatic NEC (hepatic primary lesion or liver metastasis from an unknown primary lesion)

After a detailed investigation into the primary lesion by cervical-pelvic contrast CT, gastrointestinal endoscopy, FDG-PET, otolaryngology (head and neck examination), urology examination (for men only) and gynecological examination (for women only), if the tumor is in one of the following sites, the case is defined as hepatic primary lesion (or primary lesion unknown).

- Tumor is present only in the liver
- Tumor is present in an organ that cannot be a primary lesion site (bone, lymph nodes below the diaphragm, peritoneum, subcutaneous, muscle, and spleen), and in the liver.

4. Patient selection criteria

Patients that fulfill all of the following inclusion criteria and do not correspond to any of the exclusion criteria are eligible for enrollment.

4.1. Inclusion criteria (for enrollment)

1) Any of the following is applicable based on pathological diagnosis taking findings of immunohistochemistry into consideration (see 3.1. to 3.3.).

[1] Pathologically diagnosed as neuroendocrine carcinoma (NEC*1) in the resected sample.

[2] Containing pathologically confirmed component of neuroendocrine carcinoma (NEC*1) in the biopsy sample.

1: Based on WHO 2010 classification

2) Any of the following is applicable

[1] NEC arise in esophagus, stomach, duodenum, intestine, appendix, colon, rectum, gallbladder, intrahepatic bile duct, extrahepatic bile duct, ampulla of Vater, pancreas,

[2] Liver NEC (primary liver or liver metastasis of unknown primary) *2.

*2: The tumor is only in one of the following sites after a thorough examination of the primary site by contrast CT (from the neck to pelvic) and upper/lower gastrointestinal endoscopy, FDG-PET scan, otolaryngology (head and neck) examination, urology examination (male patients only), and gynecology examination (female patients only).

a. Liver only

b. Bone, lymph nodes below the diaphragm, peritoneum, subcutaneous, muscle, spleen, and liver

3) Unresectable (see 3.6) or recurrent cancer (see 3.7). It is not essential for a pathological confirmation of the metastatic lesion or recurrent site. Cases of esophageal NEC is ineligible if corresponding to any of the following.

[1] cT4.

[2] No distant metastasis rather than supraclavicular lymph node

[3] Stenosis indicated for palliative radiotherapy

4) No previous chemotherapy or radiotherapy for NEC. Pre- or post-operative chemotherapy except irinotecan or etoposide for NEC is allowed as long as it was completed at least 8 weeks prior to registration.

5) No previous chemotherapy using platinum agents for any malignancies.

6) Aged 20 to 75 years old.

7) ECOG performance status of 0 or 1.

8) Measurable region is not required.

9) Adequate organ functions.

[1] WBC ≥3,000/ mm^3

[2] Neutrophils ≥ 1,500/ mm^3

[3] Hemoglobin ≥ 9.0 g/dL

[4] Platelets ≥ 10×10^4/ mm^3

[5] Total bilirubin≤ 1.5 mg/dL※4

[6] AST(sGOT)≦100 IU/L※4（for hepatic NEC and liver metastasis, ≦150 IU/L）

[7] ALT(sGPT)≦100 IU/L※4（for hepatic NEC and liver metastasis, ≦150 IU/L）

※4：Presence or absence of biliary drainage is not relevant

[8] Serum creatinine≦ 1.3 mg/dL

[9] Creatinine clearance※5≥ 60 mL/min

※5： Creatinine clearance must have been estimated using the Cockcroft-Gault formula, and must be 60 mL/min or more. If the estimation is less than 60 mL/min, but the actual measurement is 60 mL/min or more, the patient can be deemed eligible.

Cockcroft-Gault formula

Male：Ccr ={(140－age) × body weight (kg)}/{72×serum creatinine (mg/dL)}

Female：Ccr = 0.85×{(140－age) × body weight (kg)}/{72×serum creatinine(mg/dL)}

10) Written informed consent.

4.2. Exclusion criteria

1) Synchronous or metachronous (within 5 years) malignancies except carcinoma in situ or intramucosal tumor curatively treated with local therapy.

2) Active infection requiring systemic therapy.

3) Fever of 38 degrees Celsius or higher.

4) Pregnant or lactating women, women of childbearing potential, or women within 28 days after delivery.

5) Psychiatric disease.

6) Patients requiring systemic steroids medication.

7) Interstitial pneumonia, pulmonary fibrosis.

8) Serious co-existing illness.

9) Unstable angina pectoris within 3 weeks, or with a history of myocardial infarction within 6 months.

10) Impossible to use both iodine and gadolinium due to being allergic to contrast agent.

11) Uncontrolled diabetes mellitus or routine administration of insulin.

5. Registration and randomization

5.1. Procedure of registration

Ensure that a patient to be registered meets all eligibility criteria and does not meet any of exclusion criteria and register the patient by using JCOG Web Entry System. JCOG Web System Personal Account and password are required for web registration. If unknown, contact JCOG Data Center.

Patient registration JCOG Web Entry System
URL:https://secure.jcog.jp/dc/
(Web registration can be used for 24 hours.)

Contact information for patient registration and JCOG Web Entry System

JCOG Data Center
TEL: 03-3542-3373
Weekdays 9:00-17:00 (not available in holidays, Saturdays and Sundays, New Year's holidays)
E-mail:JCOGdata@ml.jcog.jp

Contact information on Patients Selection Criteria

Study Coordinator Contact : Chigusa Morizane
Hepato-Biliary Pancreatic Group: Futomi Mori (Main Research Secretariat)
Department of Hepatobiliary and Pancreatic Medicine, National Cancer Center
TEL:03-3542-2511
FAX:03-3542-3815
E-mail:cmorizan@ncc.go.jp

Gastric Cancer Group: Yoshinori Machida
Shizuoka Cancer Center
〒411-8777 1007 Shimonagakubo, Nagaizumi-cho, Sunto-gun, Shizuoka Prefecture
TEL:+81-55-989-5222
FAX:+81-55-989-5631
E-mail: no.machida@scchr.jp

Esophageal Cancer Group: Ken Kato/Yushi Homma
Department of Gastrointestinal Oncology, National Cancer Center
TEL: 03-3542-2511
FAX: 03-3542-3815
E-mail: kenkato@ncc.go.jp / yohonma@ncc.go.jp

5.1.1. Precautions for patient registration

① Registration after initiation of protocol treatment is unacceptable.
② Registration is performed by accessing the URL in '5.1. Procedure of registration'.
③ Eligibility checks are performed on the screen of Registration Form, so it is not necessary to send a Registration Form to Data Center by mail or fax.
④ If input data are insufficient, registration is not accepted until all are met.
⑤ The registration number is issued after the confirmation of eligibility on the registration screen, then the registration is completed.
⑥ Once registered, patients will not be retracted (retracted from the database) unless there is withdrawal of consent, including refusal to use the data for research. For duplicate registration, the information at the initial registration (registration number, allocated arm) are used in any case.
⑦ When misregistration or duplicate registration is found, contact Data Center immediately.
⑧ Body surface area and drug dose calculations are institutional responsibilities, and the body surface area

and drug dose displayed on Web Entry System at registration are only for double-checking. Those should always be calculated and checked at the institution. When the body surface area calculation formula adopted in the hospital information system of the institution differs from calculation formula adopted by JCOG (Dubois formula: Body surface area (m^2) = Body weight (kg) $^{0.425}$ x Height (cm) $^{0.725}$ 84 ÷ 10,000), there can be a difference in the dose by the hospital information system of the institution and the dose by the calculation formula adopted by JCOG, but in that case, either dosage used is decided by the site investigator.

5.2. Randomization and allocation adjustment factor

During enrollment, the treatment arm for a patient is allocated randomly by the data center.

Randomization would use a method of minimization using the 1) institutions, 2) Primary lesion organ (gastrointestinal tract [esophagus, stomach, duodenum, small intestines, appendix, colon, and rectum] vs. hepatobiliary and pancreatic organ [hepatic NEC, gallbladder, extrahepatic bile duct, ampulla of Vater, or pancreas]) as the adjustment factors, so that there are no large discrepancies between them. Researchers at participating facilities would not be informed of the detailed procedures of randomization.

6. Treatment Plan and Treatment Modification Criteria

Unless patient safety is threatened, treatment and treatment modifications is done in compliance with the specifications in this chapter.

If it is considered that the protocol specification may cause medically dangerous situation of the patient, treatment modifications should be made according to the medical judgment of the investigators/sub-investigators. Such protocol deviation is considered to be "clinically relevant deviation" if considered medically appropriate (see 14.1.4.Protocol deviation/violation). Deviations that occur with intentions other than safety, such as increasing efficacy, are not considered clinically relevant deviations.

6.1. Protocol treatment

Protocol treatment is initiated within 7 days of enrollment.

If treatment initiation occurs after 8 days from registration for any reason, the reason should be documented on the Treatment Course Form. If it is determined that treatment cannot be initiated, describe the details in the Off-treatment Form as Protocol Treatment Termination.

When laboratory parameters worsen and eligibility criteria are no longer met by the start of treatment after registration, the investigator/sub-investigator is allowed to decide whether initiate or terminate protocol treatment at their own discretion.

「6.3. The course initiation criteria is not applied at treatment changes.

Drugs used

- Etoposide ※
- Cisplatin ※
- Irinotecan ※
- Entecavir, tenofovir disoproxil fumarate, tenofovir alafenamide fumarate

The use of generic drugs is not restricted.

※ The company that manufactures or distributes these drugs, or intends to manufacture or sell these drugs, requires conflicts of interest control in the Clinical Trials Act as a company involved in this study (see 13.8.).

6.1.1. Arm A: Etoposide plus cisplatin (EP) therapy

The following chemotherapy courses will be given once a week for 3 weeks and treatment will be continued until the patient meets the discontinuation criteria.

Drug	Dosage(mg/m^2)	Dosing regimen/Dosing time	Dose day
Etoposide	100	IV/60-120 min	Day 1、2、3
Cisplatin	80	IV/60-120 min	Day 1

1) Calculation of the dosage

① Body surface area is calculated by determining drug dose to the third decimal point.

② For both etoposide and cisplatin, the calculated dose is determined by truncating the decimal point. For drug doses, ±10% is the acceptable range.

③ Dose recalculation due to body weight change after treatment initiation is not performed.

2) Administration of anticancer drugs

Etoposide and cisplatin are given in any order.

Examples of administration in the package insert are described below.

(i) Before administration: Before administration of anticancer drugs, 1,000-2,000 mL of hydration is done to achieve adequate diuresis.

(ii) Etoposide: Etoposide is mixed with infusion solutions such as isotonic sodium chloride solution of 250-500mL, and infused intravenously in about 60-120 minutes. DEHP (2-ethylhexyl) phthalate: di-(2-ethylhexyl) phthalate), which is a plasticizer, elutes from polyvinyl chloride infusion sets and catheters. Avoid the use of polyvinyl chloride infusion sets and catheters.

(iii) Cisplatin administration: Cisplatin is mixed with stock solution or 500 mL of saline (or glucose-saline) and given intravenously in 60-120 min. During the administration, caution should be exercised in ensuring urine volume, and diuretics such as mannitol and furosemide should be administered as

necessary.

(iv) After administration: After administration of cisplatin, 1,000-2,000 mL of hydration should be done so that adequate diuresis can be achieved.

In addition, short hydration administration at the discretion of each institution is permitted. (see Table 6.1.1. for treatment cases).

Table 6.1.1. Example of short hydration

	Dose	Time
5HT3 antagonist<br>Dexamethasone<br>Physiological saline	 9.9 mg<br>50 mL	 15<br>min
Etoposide<br>Physiological saline	100 mg/m^2<br>250 mL	60<br>min
No. 1 solution<br>Potassium chloride<br>Magnesium sulfate	500 mL<br>10 mEq<br>8 mEq	 60<br>min
20% mannitol	200 mL	30<br>min
Cisplatin<br>Physiological saline	80 mg/m^2<br>250 mL	60<br>min
No. 1 solution<br>Potassium chloride	500 mL<br>10 mEq	60<br>min

(Referred from Horinouchi H, et al., Japan Society of Clinical Oncology 2012

3) Precautions for treatment

JCOG9511, an upfront trial in small-cell lung cancer, suggested an association between treatment-related deaths and first-course neutropenia. Therefore, caution should be exercised when neutropenia is strongly observed from the first course.

6.1.2. Arm B: Irinotecan plus cisplatin (IP) therapy

One 4-week course of the following chemotherapy will be continued until the discontinuation criteria are met. However, if day 15 irinotecan is skipped, 3 weeks should be used as one course, and the next course should be started with day 22.

Drug	Dosage(mg/m^2)	Dosing regimen/Dosing time	Dose day
Irinotecan	60	IV/90 min	Day 1、8、15
Cisplatin	60	IV/60-120 min	Day 1

1) Calculation of the dosage

(i) Body surface area is determined by determining the dose of drug administered until the third decimal point.

(ii) The dose is calculated for both irinotecan and cisplatin by truncating the decimal point. For drug doses, ±10% is the acceptable range.

(iii) Dose recalculation due to body weight change after treatment initiation is not performed.

2) Administration of anticancer drugs

Irinotecan and cisplatin are given in any order.

Examples of administration in the package insert are described below.

(i) Before administration: Before administration of anticancer drugs, 1,000-2,000 mL of hydration is done to achieve adequate diuresis.

(ii) Irinotecan: Irinotecan is mixed with 500 mL of isotonic sodium chloride solution, glucose solution, or electrolyte maintenance solution and given by intravenous drip infusion at about 90 minutes.

(iii) Cisplatin administration: Cisplatin is mixed with stock solution or 500 mL of saline (or glucose-saline) and given intravenously in 60-120 min. During the administration, caution should be exercised in ensuring urine volume, and diuretics such as mannitol and furosemide should be administered as necessary.

(iv) After administration: After administration of cisplatin, 1,000-2,000 mL of hydration should be done so that adequate diuresis can be achieved.

In addition, short hydration administration at the discretion of each institution is permitted. (See Table 6.1.1.

3) Precautions for treatment

JCOG9511, an upfront trial in small-cell lung cancer, suggested an association between treatment-related deaths and first-course neutropenia. Therefore, caution should be exercised when neutropenia is strongly observed from the first course.

6.2. Protocol Treatment Termination/Completion Criteria

6.2.1. Definition of protocol treatment completion

Protocol treatment is continued in this study unless the protocol treatment discontinuation criteria are met, so no definition of protocol treatment completion is provided.

6.2.2. Criteria for termination of protocol treatment

Protocol treatment is terminated in any of the following cases:

1) Judged as protocol treatment is ineffective
   - When a definite tumor exacerbation is confirmed by imaging or clinically
     ※ Protocol treatment should not be discontinued if the clinical judgement of PD based on the assessment of response based on imaging indicates that continuation of protocol treatment is appropriate, and protocol treatment should be continued.
2) Protocol treatment cannot be continued due to adverse events
   ① If Grade 4 non-hematological toxicity is observed (with the exception of the following adverse events) 'hypernatremia', 'hyponatremia', 'hyperkalemia', 'hypokalemia', 'hyperglycemia', 'hypoglycemia', 'alkaline phosphatase increase', 'alanine aminotransferase increase', 'aspartate aminotransferase increase', 'blood bilirubin increase','serum amylase increase', 'lipase increase', 'GGT increase'.
   (*Adverse events other than "anaemia," "myeloid cytopenia," "lymphocyte count decreased," "neutrophil count decreased," "white blood cell count decreased," "platelet count decreased," "CD4 lymphopenia" in CTCAE v4 0)
   ② If the next course cannot be initiated beyond 21 days from the expected start date of the course due to an adverse event
   ③ When the criteria for terminating protocol treatment in the treatment modification criteria (6.3.) are met.
   ④ Adverse events other than the treatment modification criteria that the investigator/sub-investigator judges to require termination of protocol treatment
3) If the patient offers termination of protocol treatment for reasons not denied to be associated with the adverse event
   - This category should be used if an association with an adverse event cannot be ruled out.
4) When the patient offers termination of protocol treatment because of reasons for denial of association with adverse events
   - Patient refusal after enrollment and before initiation of protocol treatment
   - When the association with an adverse event can first be denied, such as the relocation of the person or household member during protocol treatment.
5) Death during protocol treatment
   - Death before deciding to terminate protocol treatment for other reasons
6) Palliative surgery for pathogenic adverse events or surgery for patients with complete response to chemotherapy (see Section 6.3.9).
7) In addition, exacerbations before the start of treatment after enrollment (protocol treatment could not be initiated due to rapid exacerbation), protocol violations were found, ineligibility was determined due to modifications in pathological diagnosis after enrollment, etc., treatment was changed, and it was judged that it was difficult to continue protocol treatment due to social reasons and safety management problems, etc.

The date of discontinuation of protocol treatment is defined as the date of death in 6.2.2.5), the date of surgery in 6.2.2.6), and otherwise, the date on which the treating physician judges that protocol treatment is discontinued.

In this study, non-NEC may be diagnosed by central pathological diagnosis during protocol treatment, because central pathological diagnosis is performed. If the patient is continuing on protocol treatment when the results of the central pathology diagnosis are reported to the registry, the attending physician and the institutional pathologist will review the results and take a clinically appropriate response. Protocol treatment is discontinued if discontinuation of protocol treatment is judged to be appropriate, and the reason for discontinuation is other.

6.3. Treatment modification criteria

The following terms shall be used for the treatment modification.

Delay: Delay the start of the course or administration of treatment from the planned date.

Termination: Discontinuation of a part of or all of the treatment without restarting.

Suspending: temporary interruptions or withdrawals that may be resumed if conditions are met

Skip: Do not administer one or more drugs and proceed to the next schedule.

Categories of infection (CTCAEv4.0) used in this study are as follows

Infection: CTCAEv4.0 infections and infestations

Bronchial infection; pulmonary infection; upper respiratory tract infection; mediastinal infection; pleural infection; catheter-related infection; Biliary tract infection; Gallbladder infection; urinary tract infection

6.3.1. Arm A (EP-therapy): Dose level

1) Etoposide

Drug	Dose level	Dosing schedule	Dose day
Etoposide	Level 0	100 mg/m^2 div	Day 1、2、3
	Level -1	80 mg/m^2 div	Day 1、2、3
	Level -2	60 mg/m^2 div	Day 1、2、3

2) Cisplatin

Drug	Dose level	Dosing schedule	Dose day
Cisplatin	Level 0	80 mg/m^2 div	Day 1
	Level -1	60 mg/m^2 div	Day 1
	Level -2	40 mg/m^2 div	Day 1

6.3.2. A arm (EP therapy): Course initiation criteria

- Initiate the course after confirming that all of the following "Course Initiation Criteria" are met on the scheduled start date of the course or the day before the plan start date of the course.
- If any one is not met, the initiation of the course is delayed.
- If the course cannot be initiated within 21 days of the expected start date of the course (if the start date of the previous course was day 1 and the course could not be initiated by day 42), discontinue the protocol treatment.
- The course initiation criteria is not applied at the start of the first course.

Course initiation criteria

① Neutrophil count Grade 0-1 (≧1500 per mm^3).

② Platelet count ≧10×10^4 /mm^3

③ AST≦100 IU/L (in the presence of hepatic metastases vs. hepatic NECs) was ≦200 IU/L).

④ ALT≦100 IU/L (≦200 IU/L for liver metastases vs liver NECs).

⑤ Total bilirubin <= 2.0 mg/dL

⑥ Serum creatinine <= 1.5 mg/dL

⑦ Fever Grade 0 (axillary temperature, no antipyretic)

⑧ Constipation, fatigue, phlebitis, oral mucositis, and infection ※1 are all Grade 2 or less.

Infected ※1: bronchial infection, pulmonary infection, upper respiratory tract infection, mediastinal infection, pleural infection, catheter-related infection, biliary tract infection, gallbladder infection, urinary tract infection

⑨ Anorexia, nausea, and emesis are all Grade 0-1.

⑩ Diarrhoeal Grade 0

6.3.3. Arm A (EP therapy): Dose reduction criteria

If any of the following toxicities are identified during the course, dose reduction should be performed in accordance with the dose reduction criteria (Table 6.3.3.) from the following course (no dose reduction in the course).

However, even if two or more items are met, the dose reduction for each drug is only one step. Re-escalation after dose reduction is not performed. Protocol treatment is terminated if the dose reduction criteria are met again after the dose reduction to Level-2.

Table 6.3.3. Arm A (EP therapy): Dose reduction criteria

Item	Etoposide	Cisplatin
Neutrophil count Grade 4 (<500 per mm^3).	Reduce the level by 1	No change
Platelet count Grade 4 (<$2.5\times10^4/mm^3$)	Reduce the level by 1	No change
1.5 <serum creatinine ≦2.0 mg/dL.	No change	Reduce the level by 1
Serum creatinine <= >2.0 mg/dL	Termination of protocol treatment	Termination of protocol treatment
Grade 3<br>Infected ※1	Reduce the level by 1	Reduce the level by 1
Grade 2<br>Peripheral sensory neuropathy<br>Peripheral motor neuropathy,<br>Myalgia; arthralgia; tinnitus; hearing impairment	No change	Reduce the level by 1
Grade 3<br>Peripheral sensory neuropathy<br>Peripheral motor neuropathy,<br>Myalgia; arthralgia; tinnitus; hearing impairment	Termination of protocol treatment	Termination of protocol treatment
Non-hematologic toxicities of Grade 3 other than those listed above in ※2 that are causally related to EP-therapy (excluding hyponatremia, hypokalemia, hypomagnesemia, hypocalcemia, hyperglycemia, and weight loss)	Reduce the level by 1	Reduce the level by 1

※1:※1 of infection: bronchial infection, pulmonary infection, upper respiratory tract infection, mediastinal infection, pleural infection, catheter-related infection,
Biliary tract infection; Gallbladder infection; Urinary tract infection

※2:Causal relationship is judged as either of possible, probable, definite

6.3.4. Arm A (EP therapy): Within-course pause, skipping criteria

Following initiation of treatment with each course after the course initiation criteria are met, if any of the following adverse events are observed, day 2, day 3 etoposide will be suspended.

- Fever (axillary temperature) Grade 1-3
- Grade 3 of infection (bronchial infection, pulmonary infection, upper respiratory tract infection, mediastinal infection, pleural infection, catheter-related infection,
  Biliary tract infection, gallbladder infection, urinary tract infection)

Resting etoposide should be resumed after confirming that all initiation criteria are met until day 7. However, it should not be administered after day 8.

That is, if etoposide could not be administered by day 7, the remaining etoposide should be skipped.

If the above suspension and/or skip occur, the next course of etoposide should be started day 22 (after 3 weeks) counting from day 1 of the previous course if the ininiation criteria are met.

6.3.5. Arm B (IP therapy): Dose-level

1) Irinotecan

Drug	Dose level	Dosing schedule	Dose day
Irinotecan	Level 0	60 mg/m^2 div	Day 1、8、15
	Level -1	50 mg/m^2 div	Day 1、8、15
	Level -2	40 mg/m^2 div	Day 1、8、15

2) Cisplatin

Drug	Dose level	Dosing schedule	Dose day
Cisplatin	Level 0	60 mg/m^2 div	Day 1
	Level -1	50 mg/m^2 div	Day 1
	Level -2	40 mg/m^2 div	Day 1

6.3.6. Arm B (IP therapy): Course initiation criteria

- On the day of the initiation of the course or the day before the expected start of the course, start the course after confirming that all of the following "Course Initiation Criteria" are met.
- If any one is not met, the initiation of the course is delayed.
- If the course cannot be initiated within 21 days of the expected start date of the course (if the start date of the previous course was day 1 and the course could not be initiated by day 49), discontinue the protocol treatment.
- However, if day 15 irinotecan is skipped in the previous course, day 22 of the previous course is set as the scheduled start date of the next course regarded as one course per 3 weeks.
- The course initiation criteria is not applied at the start of the first course.

Course initiation criteria

① Neutrophil count Grade 0-1 (≧1500/mm^3).
② Platelet count ≧10×10^4 /mm^3
③ AST≦100 IU/L (≦200 IU/L for liver metastases versus liver NECs)).
④ ALT≦100 IU/L (≦200 IU/L for liver metastases vs liver NECs).
⑤ Total bilirubin <= 2.0 mg/dL
⑥ Serum creatinine <= 1.5 mg/dL
⑦ Fever Grade 0 (measured by axillary temperature, temperature <38°C without antipyretic use)
⑧ Constipation, fatigue, phlebitis, oral mucositis, and infection ※1 are all Grade 2 or less.
Infected ※1: bronchial infection, pulmonary infection, upper respiratory tract infection, mediastinal infection, pleural infection, catheter-related infection, biliary tract infection, gallbladder infection, urinary tract infection
⑨ Anorexia, nausea, and emesis are all Grade 0-1.
⑩ Diarrhoea Grade 0

6.3.7. Arm B (IP-therapy): Dosing criteria for day 8, day 15

After confirming that all of the following ①-③ are met, the second (day 8) or third (day 15) dose of irinotecan is administered. If day 8, day 15 dosing criteria are not met, skip day 8, day 15 dosing.

① All of the following are met with the most recent laboratory data on the scheduled day of administration or the day before the scheduled day of administration.
i) Neutrophil count Grade 0-2 (≧1000 per mm^3).
ii) Platelet count ≧10×10^4 /mm^3
iii) AST ≦ 100 IU/L (≦200 IU/L for liver metastases vs liver NECs).
iv) ALT ≦ 100 IU/L (≦200 IU/L in the presence of hepatic metastases versus hepatic NECs).
v) Total bilirubin <= 2.0 mg/dL
vi) Serum creatinine <= 2.0 mg/dL

② All of the following are met on the scheduled day of administration:
i) Fever Grade 0 (measured by axillary temperature, temperature <38°C without antipyretic use)

ii） Diarrhoeal Grade 0

③ Constipation, anorexia, nausea, emesis, fatigue, phlebitis, oral mucositis, and infection ※1 are all Grade 2 or less.

Infected ※1: bronchial infection, pulmonary infection, upper respiratory tract infection, mediastinal infection, pleural infection, catheter-related infection, biliary tract infection, gallbladder infection, urinary tract infection

6.3.8. Arm B (IP therapy): Dose reduction criteria

If any of the following toxicities are identified during the course, dose reduction should be performed in accordance with the dose reduction criteria (Table 6.3.8.) from the following course (no dose reduction in the course).

However, even if two or more items are met, the dose reduction for each drug is only one step. Re-escalation after dose reduction is not performed. Protocol treatment is terminated if the dose reduction criteria are met again after the dose reduction to Level-2.

Table 6.3.8. Arm B (IP therapy): Dose reduction criteria

Item	Irinotecan	Cisplatin
Neutrophil count Grade 4 (<500 per mm^3).	Reduce the level by 1	No change
Platelet count Grade 4 (< $2.5\times10^4/mm^3$)	Reduce the level by 1	No change
1.5 <serum creatinine ≦2.0 mg/dL.	No change	Reduce the level by 1
Serum creatinine <= >2.0 mg/dL	Termination of protocol treatment	Termination of protocol treatment
Grade 3<br>Infected ※1	Reduce the level by 1	Reduce the level by 1
Grade2<br>Peripheral sensory neuropathy<br>Peripheral motor neuropathy,<br>Myalgia; arthralgia; tinnitus; hearing impairment	No change	Reduce the level by 1
Grade 3<br>Peripheral sensory neuropathy<br>Peripheral motor neuropathy,<br>Myalgia; arthralgia; tinnitus; hearing impairment	Termination of protocol treatment	Termination of protocol treatment
Non-hematologic toxicities of Grade 3 other than those listed above in ※2 that are causally related to IP-therapy (excluding hyponatremia, hypokalemia, hypomagnesemia, hypocalcemia, hyperglycemia, and weight loss)	Reduce the level by 1	Reduce the level by 1

※1:※1 of infection: bronchial infection, pulmonary infection, upper respiratory tract infection, mediastinal infection, pleural infection, catheter-related infection,

Biliary tract infection; Gallbladder infection; Urinary tract infection

※2:Causal relationship is judged as either of possible, probable, definite

6.3.9. Surgical of after end of chemotherapy

1) Surgery for Adverse Events Associated with Pathogenesis

Surgery may be performed if it is judged clinically desirable to perform surgery for newly appearing symptoms such as hemorrhage or stenosis. Protocol treatment is discontinued if surgery is performed, regardless of the content. In this case, the date of discontinuation of protocol treatment is the date of surgery.

2) Surgery for patients with complete response to chemotherapy

- When chemotherapy is highly effective, all metastases present at the time of enrollment on imaging studies disappear, and curative resection (R0 resection) is considered possible, resection including primary and metastatic disease may be performed.
- Surgical procedures are not specifically specified, but the Research Office will collect information on the details of the surgery performed individually in order to be reviewed by the research representative/research office.
- In all surgical cases, the group group conference shall confirm the validity of the judgment that surgery is indicated.

- When surgery is performed, protocol treatment is discontinued, regardless of whether curative resection was performed or not. In this case, the date of discontinuation of protocol treatment is the date of surgery.

6.3.10. Consultation on treatment modification

If there are any questions about treatment modification, contact "16.6. Study Coordinator".

Study Coordinator Contact : Chigusa Morizane

Hepato-Biliary Pancreatic Group: Futomi Mori (Main Research Secretariat)
Department of Hepatobiliary and Pancreatic Medicine, National Cancer Center
TEL:03-3542-2511
FAX:03-3542-3815
E-mail:cmorizan@ncc.go.jp

Gastric Cancer Group: Yoshinori Machida
Shizuoka Cancer Center
〒411-8777　1007 Shimonagakubo, Nagaizumi-cho, Sunto-gun, Shizuoka Prefecture
TEL:+81-55-989-5222
FAX:+81-55-989-5631
E-mail：no.machida@scchr.jp

Esophageal Cancer Group: Ken Kato/Yushi Homma
Department of Gastrointestinal Oncology, National Cancer Center
TEL:03-3542-2511
FAX:03-3542-3815
E-mail: kenkato@ncc.go.jp / yohonma@ncc.go.jp

6.4. Concomitant treatment and supportive care

6.4.1. Required concomitant treatment/supportive care

1) Testing and Supportive Care for HBsAg-Positive Cases.

In HBsAg-positive cases, steroids and chemotherapy can lead to rapid hepatitis B virus (HBV) expansion (reactivation: reactivation) and potentially fatal severe hepatitis. Therefore, the following tests and supportive care are performed based on the "Guidelines for the Treatment of Hepatitis B, 1.2 Edition (Japanese Society of Hepatology)." It is advisable to consult a hepatologist at the time prior to initiation of a nucleic acid analogue (entecavir).

①Testing prior to initiation of chemotherapy: HBV-DNA quantitation

HBV-DNA quantitative analysis should be performed at least once prior to initiation of chemotherapy.

HBV-DNA assays are performed by PCR or real-time PCRs.

HBeAg and HBe antibodies should also be measured in accordance with the Guidelines for the Treatment of Hepatitis B, Third Edition (Japanese Society of Hepatology).

②Dosing schedule for supportive care (nucleic acid analogues prophylaxis)

- **Drugs used :**
  - **Entecavir (Bristol-Myers: Baraclude Tablets 0.5 mg)**

The following dosage regimen should be followed, starting at least 1 week before the start of chemotherapy, and continuing for at least 12 months after the end of chemotherapy. Nucleic acid analogues may be discontinued after 12 months after completion of chemotherapy if conditions *1 and 2 for discontinuation of NAs are met. However, if the administration of a nucleic acid analogue is discontinued, consultation with a hepatologist is always obtained, and the administration is discontinued only if the hepatologist deems it appropriate.

※1 Requirements for discontinuation of nucleic acid analogues (entecavir, tenofovir disoproxil fumarate, tenofovir alafenamide fumarate): all of the following

1. The patient has been on NA for more than 2 years.
2. HBV-DNA assays are not sensitive to detect
3. Be negative for HBeAg

※2 Patient background requirements: All of the following

1. Both the treating physician and the patient have sufficiently understood that the hepatitis exacerbation is frequently observed after the nucleic acid analog withdrawal, and that there is a danger of becoming serious in the time.
2. Follow-up is possible after treatment cessation, and appropriate treatment is possible even if hepatitis recurs
3. It is judged that the liver fibrillation is slight and the hepatic reserve is good, and it is difficult to become serious even if the hepatitis is exacerbated.

(Adapted from Guidelines for the Treatment of Hepatitis B, the 3rd edition (Japanese Society of Hepatology))

- **Dosage regimen: Take this medicine on an empty stomach (2 hours after meals and more than 2 hours before the next meal).**
- **Dosage :**

Creatinine clearance (mL/min).	Dosage
50 or more	0.5 mg once daily
30 Beyond 50	0.5 mg once every 2 days
10 Beyond 30	0.5 mg once every 3 days
10 Less than	0.5 mg once every 7 days

- **Adverse drug reactions (incidence of all grades): nucleoside analog-naïve patients**

Diarrhea (6.0%), nausea (4.5%), constipation (3.7%), upper abdominal pain (3.0%), malaise (1.5%), nasopharyngitis (3.0%), muscle stiffness (2.2%), headache (3.0%), rash (incidence unknown), laboratory tests: elevated AST (3.7%), increased blood bilirubin (6.0%), increased blood amylase (10.4%), and increased lipase (10.4%).Blood glucose increased (6.0%), blood lactate increased (6.7%), urine occult blood positive (4.5%), white blood cell count decreased (8.2%), and eosinophil count increased (0.7%). [Significant adverse reactions (incidence unknown)] Hepatitis worsened after completion of treatment, anaphylactoid symptoms, lactic acidosis, and severe hepatomegaly due to fatty liver

③Monitoring: Quantitative analysis of HBV-DNA (during and after administration of nucleic acid analogues)

During entecavir administration:

They are monitored every 4 weeks by both HBV-DNA quantitative analysis and liver function (ASTs, ALTs). However, if HBV-DNA level is less than 20 IU/mL (1.3 log IU/mL) during administration of nucleic acid analogues, it is acceptable to perform tests every 4 to 12 weeks.

After discontinuation of entecavir administration:

Bearing in mind that reactivation may occur even after discontinuation of administration of a entecavir, the patient should be consulted with a hepatologist, and the patient should be monitored for HBV-DNA determination and hepatic function (AST/ALT) every 4 weeks for at least 1 year after discontinuation of administration of a entecavir. Subsequent follow-up will be decided after consulting a hepatologist.

2) Laboratory Teste and Supportive Care for HBsAg-Negative and HBc Antibody-Positive and/or HBs Antibody-Positive Cases.

i) HBV-DNA ≧ 20 IU/mL (1. 3 log IU/mL) prior to initiation of chemotherapy

It has been clarified that HBV-DNA replicates persist at low levels in the livers and peripheral blood mononuclear cells when HBc or HBs are positive, even if they are HBs-Ag negative. It has been reported that reactivation of HBV and development of severe hepatitis are caused by the use of potent immunosuppressive agents even in such patients with previous infections.

If HBV-DNA ≧ 20 IU/mL (1.3 log IU/mL), the risk of HBV reactivation is judged to be as high as in HBsAg-positive cases, and prophylactic administration of entecavir is administered. The following laboratory tests and supportive care are performed in accordance with the "Guideline for the Treatment of Hepatitis B, 3rd edition (Japanese Society of Hepatology)" with reference to the following for examination, dosage, and monitoring of supportive care before the start of chemotherapy.

However, these are not applicable if the HBs antibody alone is positive and the HBV vaccination history is obvious.

①Dosing schedule for supportive care (nucleic acid analogues prophylaxis)

According to the dosage and administration of nucleic acid analogues (entecavir, tenofovir disoproxil fumarate, tenofovir alafenamide fumarate) in "1) Test and supportive care for HBsAg positive cases". Same conditions for NA discontinuation.

② Monitoring: Quantitative analysis of HBV-DNA (during and after administration of nucleic acid analogues)

The intervals for monitoring during and after discontinuation of NA are in accordance with the provision of "1) Testing and supportive care for HBsAg-positive patients".

ii) HBV-DNA less than 20 IU/mL (1.3 log IU/mL) prior to initiation of chemotherapy

HBV-DNA quantitative analysis and hepatic function (AST, ALT) will be monitored, and nucleic acid analogues (entecavir, tenofovir disoproxil fumarate, tenofovir alafenamide fumarate) will be started when ≧20 IU/mL (1.3 log IU/mL) is achieved.

The Guidelines for the Treatment of Hepatitis B, the 3rd edition (Japanese Society of Hepatology) recommends monitoring with HBV-DNA quantitative analysis or high-sensitivity HBs antibodies during and after chemotherapy, depending on the risks of revitalization.

①Monitor:HBV-DNA quantitative analysis

HBV-DNA quantitative analysis should be performed every 4-12 weeks from the start of chemotherapy until at least 12 months after the end of chemotherapy.

If HBV-DNA level is more than 20 IU/mL (1.3 log IU/mL), administration of nucleic acid analogues should be started immediately in accordance with the Guidelines for the Treatment of Hepatitis B, the 3rd edition (Japanese Society of Hepatology). If HBsAg monitoring is positive for < 1 IU/mL (low positive), nucleic acid analogues should be administered after additional HBV DNA determinations of ≧ 20 IU/mL (1.3 log IU/mL). It is advisable to consult a hepatologist at a time prior to initiation of NAs.

②**Supportive care in reactivation**

Nucleic acid analogues should be administered according to the supportive care described in i) When HBV-DNA prior to the initiation of chemotherapy is 20 IU/mL (1.3 log IU/mL) or more in 6.4.1.2). Once administration of nucleic acid analogues is started, nucleic acid analogues should be discontinued only if appropriate by the hepatologist.

6.4.2. Recommended/not recommended concomitant treatment/supportive care

1) G-CSF

Prophylactic G-CSF administration is not recommended.

Timing of initiation	1) Fever (≥ 38 °C in principle) with neutrophil count < 1,000/ mm^3<br>2) Neutrophil count = 500 /mm^3<br>3) 1) or 2) in the previous cycle followed by neutrophil count < 1,000 /mm^3 after the same chemotherapy
Dosage and administration	1) Filgrastim: subcutaneous injection at a dose of 50 µg/ m^2 once daily<br>2) Nartograstim: subcutaneous injection at a dose of 1 µg/ kg once daily<br>3) Lenograstim: subcutaneous injection at a dose of 2 µg/ kg once daily
Timing of discontinuation	1) Discontinue treatment when the neutrophil count increases from the minimum to ≥ 5,000/ mm^3.<br>2) Consider discontinuation of treatment or dose reduction when patient safety is supported by recovery of neutrophil count to ≥ 2,000/ mm^3 and no infection symptoms.

2) Treatment of fever during neutropenia

(1) When the neutrophil count is less than 500 /mm3 or less than 1,000 /mm3 and is expected to decrease to less than 500 /mm3 within 48 hours, and the axillary temperature is 37.5°C or higher (oral temperature 38°C or higher), a risk assessment should be performed immediately and antimicrobial therapy should be started according to risk.

(2) Risk assessment should be performed with reference to the Multinational Association for Supportive Care in Cancer (MASCC) scoring system*1.

(3) For initial assessment, a complete blood count including white blood cell fraction and platelet count, renal function (BUN, creatinine), electrolytes, and liver function (transaminases, total bilirubin, alkaline phosphatase) tests, at least two sets of venous blood culture tests before starting antimicrobial therapy, and if a central venous catheter is implanted If a central venous catheter is placed, one set of culture tests from the lumen of the catheter and one set from a peripheral vein, culture tests at sites where infection is suspected, and chest radiographs if respiratory symptoms or signs are present.

(4) In high-risk patients, beta-lactams with anti-Pseudomonas aeruginosa activity should be administered intravenously as a single agent. However, other antimicrobial agents (aminoglycosides, fluoroquinolones, and/or vancomycin) may be added to the single agent in the initial regimen in patients with unstable or comorbid conditions or when drug-resistant organisms are strongly suspected. In low-risk patients, antimicrobials may be administered orally or intravenously, with hospitalization or outpatient therapy when appropriate after thorough evaluation.

(5) Reevaluate after 3-4 days of antimicrobial initiation to discuss continuation or modification of antimicrobials, and as a rule, continue antimicrobials until the neutrophil count recovers to 500 /mm3 or higher.

(6) In high-risk patients who do not respond to broad-spectrum antimicrobial therapy for 4-7 days, empiric antifungal therapy is recommended.

(7) Fluoroquinolone prophylaxis is recommended in high-risk patients who are expected to have neutrophil counts below 100 /mm3 for longer than 7 days.

(8) Prophylactic administration of G-CSF is recommended in patients with a 20% or greater risk of developing febrile neutropenia and in patients with a 10-20% risk of developing febrile neutropenia who are i) older than 65 years, ii) in the advanced stage of disease, iii) not receiving prophylactic antibacterial therapy, or iv) have a history of febrile neutropenia. prophylactic administration of G-CSF is recommended. In patients at less than 10% risk of developing the disease, it is recommended only if a severe course is anticipated.

(9) If febrile neutropenia (FN) develops in a patient with a central venous catheter inserted, blood cultures from the catheter and peripheral blood should be performed and considered a catheter-related infection if there is a time

difference of 120 minutes or more between the two positive results. Catheter removal should be performed if there is no improvement after 72 hours of appropriate antimicrobial therapy. In case of infection caused by Staphylococcus aureus, Pseudomonas aeruginosa, Bacillus, fungi, or acid-fast bacilli, remove the catheter and administer appropriate antimicrobial therapy based on culture results.

(10) Implement infection prevention measures by performing hand hygiene, standard precautions for infection of healthcare workers, and isolation precautions for patient pathogens.

2) Nausea and vomiting

Regarding nausea and vomiting, antiemetics are positively administered according to Clinical Practice Guidelines for Antiemesis in Oncology [48], and fluid and electrolyte repletion are performed when oral intake is severely reduced.

3) Anorexia

If oral intake drops markedly, fluid and electrolyte supplements should be given as needed. Especially, in the cases with diabetes mellitus, the abnormality of blood sugar level and electrolyte is noticed.

4) Anemia, thrombocytopenia

If anaemia (haemoglobin < 8.0 g/dL) or thrombocytopenia (platelet count < $2 \times 10^4/mm^3$) is observed, blood should be transfused as appropriate at the discretion of the treating physician.

5) Diarrhea

Severe diarrhoea occasionally occurs with arm B (IP therapy arm) and is extremely dangerous when complicated by febrile neutropenia. Patients should be fully informed about toxicities and their management, and should be instructed to measure body temperature, especially when neutrophils counts are most decreasing, and to contact a physician or nurse immediately during fever and diarrhea. Nonsteroidal anti-inflammatory drugs may not cause fever to become overt, so unnecessary anti-inflammatory drugs are not given.

If irinotecan-induced diarrhea occurs, the following supportive measures are recommended:

High-dose loperamide hydrochloride therapy

① Loperamide hydrochloride was started after signs of diarrhea were observed.
② Initial dose of 4 mg followed by 2 mg/2 hours (4 mg/4 hours at night)
③ It is administered until watery stool does not appear for more than 12 hours.
④ Doses should not be given for more than 48 hours.

6) Precautions on the day of cisplatin administration

Aminoglycoside antibiotics, vancomycin, and nonsteroidal anti-inflammatory drugs are not administered on the day of cisplatin administration or are used with caution.

6.4.3. Acceptable concomitant treatment and supportive care

The following concomitant treatment and supportive care may be used as needed.

Concomitant use of drugs for the treatment of complications such as hypertension and diabetes mellitus and symptomatic drugs such as morphine may be performed, but this drug should be administered with caution when furosemide, piretanide and phenytoin are used. Oral antibiotics for febrile neutropenia prophylaxis may be given at the discretion of the investigator/sub-investigator. Bisphosphonate denosumab may be used in combination for bone metastases.

6.4.4. Unacceptable concomitant treatment and supportive care

None of the following treatments will be given during protocol treatment:

① Administration of anticancer drugs other than protocol treatment
② Radiation therapy

6.4.5. Granulocyte colony-stimulating factor (granulocyte-colony stimulating factor:G-CSF

※This study permits the use of G-CSF biogenerics (biosimilars).

1)※ of primary prophylaxis with G-CSF

※Primary prophylaxis: G-CSF administration before developing febrile neutropenia or prolonged neutropenia to prevent them during anticancer therapy.

Since the risk of developing FN in both groups is less than 10%, primary prophylactic administration of G-CSF is not recommended according to the "Guidelines for the Appropriate Use of G-CSF 2013" and "JSMO Practice Guidelines for Febrile Neutropenia (FN)".

However, there is information that in IP therapy (group B), the incidence of FN was 10.6% in the JCOG0509 study (advanced small cell lung cancer), and in EP therapy (group A), the incidence of FN was 38% in a study report of 21 patients with hepatobiliary-pancreatic primary NEC by Iwasa et al, although it was an observational study. In addition, the package insert allows primary prophylactic administration of G-CSF for small cell lung cancer, and the treatment regimen for NEC is similar to that for small cell lung cancer. Therefore, primary prophylactic administration of G-CSF is acceptable in the presence of infection, which is a risk factor for the development of FN (including high-risk conditions for infection such as biliary stents) and in elderly patients (>65 years).

Since the safety of pegfilgrastim administered 14 days prior to the initiation of cancer chemotherapy and within 24 hours after completion of administration has not been established, the primary prophylactic administration of pegfilgrastim is not performed in group B where day 8 is administered the drug.

Table 6.4.5. Primary prophylactic administration of G-CSF should be performed according to the approved dosage and administration shown in the table below.

Drug	・・ Pegfilgrastim (arm A only)<br>・・ Filgrastim<br>・・ Naltograstim<br>・・ Lenograstim
Time of initiation	Twenty-four hours after completion of chemotherapy
Dosage Dosing regimen	・Pegfilgrastim (genetical recombination) at a dose of 3.6 mg subcutaneously once per chemotherapy course (group A only)<br>・Filgrastim: 50 µg per m^2 SC once daily or 100 µg per m^2 IV once daily<br>・Naltograstim: 1 µg/kg SC once daily or 2µg/kg IV once daily<br>・Lenograstim: 2 µg/kg SC once daily or 5µg/kg IV once daily
Timing of discontinuation (other than pegfilgrastim)	・If the neutrophil count reaches 5000 per mm^3 or more after the course, administration should be discontinued.<br>・If the neutrophil count recovers to ≧2000 cells per mm^3, there are no suspected infections, and the patient's safety is judged to be ensured by the responsiveness to the drug used, the drug should be discontinued or reduced.

2)Secondary prophylactic※ of G-CSF

※ Secondary prophylaxis: G-CSF prophylactic administration after once occurrence of febrile neutropenia or prolonged neutropenia to prevent febrile neutropenia or prolonged neutropenia from occurring again during anticancer therapy.

If febrile neutropenia or Grade 4 neutropenia lasting more than 7 days occurred in the previous course, secondary prophylactic administration of G-CSF is not recommended because the dose of anticancer agents will be reduced according to the protocol in the next and subsequent courses. However, in the presence of infection, which is a risk factor for the development of FN (including high-risk conditions for infection such as biliary stents) or in elderly patients (65 years and older), secondary prophylactic administration of G-CSF is acceptable after reduction of anticancer agents according to the protocol.

However, the safety of pegfilgrastim administered 14 days prior to the start of cancer chemotherapy and 24 hours after the end of treatment has not been established, so secondary prophylactic pegfilgrastim administration is not performed in group B, where day 8 is given the drug.

Secondary prophylactic administration of G-CSF should be performed according to the approved dosage and administration shown in the table below.

Drug	･･ Pegfilgrastim (arm A only)<br>･･ Filgrastim<br>･･ Naltograstim<br>･･ Lenograstim
Time of initiation	･･ Pegfilgrastim (arm A only)<br>Twenty-four hours after completion of chemotherapy<br>･･ Filgrastim, nartograstim, and lenograstim<br>When neutrophil counts <1000 per mm^3 are observed
Dosage Dosing regimen	･Pegfilgrastim (genetical recombination) at a dose of 3.6 mg subcutaneously once per chemotherapy course (group A only)<br>･Filgrastim: 50 µg per m^2 SC once daily or 100 µg per m^2 IV once daily<br>･Naltograstim: 1 µg/kg SC once daily or 2µg/kg IV once daily<br>･Lenograstim: 2 µg/kg SC once daily or 5µg/kg IV once daily
Timing of discontinuation (other than pegfilgrastim)	･If the neutrophil count reaches 5000 per mm^3 or more after the course, administration should be discontinued.<br>･If the neutrophil count recovers to ≧2000 cells per mm^3, there are no suspected infections, and the patient's safety is judged to be ensured by the responsiveness to the drug used, the drug should be discontinued or reduced.

3) Therapeutic administration of G-CSF

Therapeutic administration of G-CSF should be performed according to the approved dosage and administration shown in the table below.

Time of initiation	･When the neutrophil count is less than 1000 per mm^3 and fever (in principle, > 38.0°C) is observed<br>･When neutrophil counts <500 per mm^3 are observed
Dosage Dosing regimen	･Filgrastim: 50 µg per m^2 SC once daily or 100 µg per m^2 IV once daily<br>･Naltograstim: 1 µg/kg SC once daily or 2µg/kg IV once daily<br>･Lenograstim: 2 µg/kg SC once daily or 5µg/kg IV once daily
Timing of discontinuation	･If the neutrophil count reaches 5000 per mm^3 or more after the course, administration should be discontinued.<br>･If the neutrophil count recovers to ≧2000 cells per mm^3, there are no suspected infections, and the patient's safety is judged to be ensured by the responsiveness to the drug used, the drug should be discontinued or reduced.

6.5. Post-study treatment

Treatment after discontinuation of protocol treatment and treatment after progression or recurrence after completion are not specified.

Treatment (cross over) with drugs included in treatment regimens in the unassigned groups may be used, but if the total dose of cisplatin is greater than 500 mg/m^2, careful attention should be given to accumulating toxicities such as peripheral sensory/motor neuropathy, hearing loss, and renal impairment. Patients should be carefully monitored with adequate risk explanation only if the benefits are apparently outweighed by the risks, such as those with persistent sensitivity to cisplatin and mild cumulative toxicity.

7. Anticipated Adverse Events

7.1. Anticipated adverse reactions

Anticipated adverse reactions in this study are as follows:

7.1.1. Anticipated Adverse Drug Reactions with Drugs

Adverse drug reactions anticipated with protocol treatments and drugs used in protocol-specified tests are referred

to the latest version of the drug package insert.

7.1.2. Anticipated adverse reactions in the standard treatment arm (arm A)

Table 7.1.2. Adverse events in the EP-therapy group at JCOG9511 (excerpt from the final analysis report)

Examination Items	Grade 0	Grade 1	Grade 2	Grade 3	Grade 4	Grade 3,4(%)	Grade4(%)	Total
White blood cells	2	5	30	35	5	51.9%	6.5%	77
Neutrophils	1	0	5	21	50	92.2%	64.9%	77
Hemoglobin	2	9	43	23	-	29.9%	-	77
Platelet	31	19	13	14	0	18.2%	0%	77
Total bilirubin	57	-	20	0	0	0%	0%	77
GOT	49	24	2	1	1	2.6%	1.3%	77
GPT	40	28	6	2	1	3.9%	1.3%	77
Creatinine	56	16	5	0	0	0%	0%	77
Oxygen tension	13	27	9	2	1	5.8%	1.9%	52
Nausea and vomiting	13	36	23	5	-	6.5%	-	77
Diarrhea	64	8	5	0	0	0%	0%	77
Oral cavity (stomatitis)	68	6	2	1	0	1.3%	0%	77
Infection	42	23	9	1	2	3.9%	2.6%	77
Hair loss (hair)	9	46	19	-	-	-	-	74
Fever (uninfected)	45	14	16	2	0	2.6%	0%	77
Perception (peripheral nerve)	66	10	1	0	-	0%	-	77
Rash	74	2	1	0	0	0%	0%	77

※: Use JCOG Toxicity Criteria.

7.1.3. Anticipated adverse reactions in the study treatment arm (Arm B)

Table 7.1.3. Adverse Events in the IP-Therapy Group in a JCOG9511 (Extracted from the Final Analysis Report)

Examination Items	Grade 0	Grade 1	Grade 2	Grade 3	Grade 4	Grade 3,4(%)	Grade 4(%)	Total
White blood cells	1	16	38	17	3	26.7%	4.0%	75
Neutrophils	1	8	17	30	19	65.3%	25.3%	75
Hemoglobin	7	10	38	20	-	26.7%	-	75
Platelet	56	6	9	1	3	5.3%	4.0%	75
Total bilirubin	59	-	16	0	0	0%	0%	75
GOT	40	30	5	0	0	0%	0%	75
GPT	35	30	7	3	0	4.0%	0%	75
Creatinine	56	15	4	0	0	0%	0%	75
Oxygen tension	13	20	5	1	1	5.0%	2.5	40
Nausea and vomiting	11	26	28	10	-	13.3%	-	75
Diarrhea	23	19	21	8	4	16.0%	5.3%	75
Oral cavity (stomatitis)	66	9	0	0	0	0%	0%	75
Infection	45	17	9	3	1	5.3%	1.3%	75
Hair loss (hair)	17	44	13	-	-	-	-	74
Fever (uninfected)	45	12	17	1	0	1.3%	0%	75
Perception (peripheral nerve)	71	4	0	0	-	0%	-	75
Rash	70	5	0	0	0	0%	0%	75

※: Use JCOG Toxicity Criteria.

Table 7.1.3. Adverse events in the IP-therapy group in b JCOG0509 (abstracted from the main analysis report)

Examination Items	Grade 0	Grade 1	Grade 2	Grade 3	Grade 4	Grade 3,4(%)	Grade 4(%)	Total
White blood cells	16	36	58	29	3	22.5	2.1	142
Neutrophils	6	10	43	51	32	58.5	22.5	142
Hemoglobin	20	24	65	24	9	23.2	6.3	142
Platelet	125	6	8	2	1	2.1	0.7	142

Total bilirubin	116	15	10	1	0	0.7	0	142
GOT	98	37	5	2	0	1.4	0	142
GPT	80	56	4	2	0	1.4	0	142
Creatinine	97	37	8	0	0	0	0	142
Anorexia	22	67	33	19	1	14.1	0.7	142
Nausea	30	67	36	9	0	6.3	0	142
Vomiting	89	38	10	5	0	3.5	0	142
Diarrhea	52	51	28	11	0	7.7	0	142
Mucositis (examination findings)-oral cavity	126	15	0	0	1	0.7	0.7	142
Hair loss	64	66	12	-	-	-	-	142
Onset of febrile neutropenia	127	-	-	14	1	10.6	0.7	142
Infection with Grade3-4 neutropenia -Bronchus	142	-	0	0	0	0	0	142
-Lung (pneumonia)	137	-	0	4	1	3.5	0.7	142
-Pharynx	142	-	0	0	0	0	0	142
-Upper respiratory tract-unclassifiable	141	-	1	0	0	0	0	142
-Bladder	142	-	0	0	0	0	0	142
-Kidney	142	-	0	0	0	0	0	142
-Urinary tract-subclassification impossible	142	-	0	0	0	0	0	142
Neuropathy: Sensory	127	13	2	0	0	0	0	142

Table 7.1.3.c Adverse events from a pilot trial of postoperative adjuvant chemotherapy with irinotecan plus cisplatin for high-grade neuroendocrine lung cancer (excerpt)

Examination Items	Grade 2	Grade 3	Grade 4	Grade 3,4(%)	Grade 4(%)	Total
White blood cells	17	7	0	17.5%	0%	40
Neutrophils	12	15	4	47.5%	10.0%	40
Hemoglobin	14	6	4	25.0%	10.0%	40
Platelet	2	0	0	0%	0%	40
Total bilirubin	0	0	0	0%	0%	40
GOT	0	0	0	0%	0%	40
GPT	1	0	0	0%	0%	40
Creatinine	0	0	0	0%	0%	40
Hyponatremia	0	5	0	12.5%	0%	40
Endotoxemia	3	1	0	2.5%	0%	40
Hypokalemia	0	4	0	10.0%	0%	40
Nausea	8	4	-	10.0%	-	40
Vomiting	4	2	0	5.0%	0%	40
Anorexia	2	0	-	0%	0%	40
Diarrhea	11	2	0	5.0%	0%	40
Fatigue	10	5	-	12.5%	0%	40
Infection	2	0	0	0%	0%	40

7.2. Evaluation of Adverse Events/Reactions

The Common Terminology Criteria for Adverse Events v4.0 Japanese Translated JCOG Version (Japanese translation of NCI-Common Terminology Criteria for Adverse Events v4.0 (CTCAE v4.0)) (CTCAE v4.0-JCOG) will be used to assess adverse events/reactions. For CTCAE v4 0-JCOG in which Grade is defined by laboratory reference values at the institutional reference value, the "JCOG sharing reference range" will be used instead of the institutional reference value at each medical institution. For more information on JCOG sharing reference ranges, see JCOG website (see http://www.jcog.jp/doctor/tool/kijun.html)).

In grading of adverse events, each grading is closest to the definitions of Grade 0 4 (nearest match). Grading to a higher Grade when the definition of more than one Grade is comparable and when it is difficult to decide on either (highest grade).

Grading should also be given to Grade if specific actions are described, due to their clinical need. For example, patients may refuse oxygen inhalation or chest drainage, even when the patient's pleural effusion is increasing and oxygen inhalation or chest drainage is indicated. In such cases, grading is based on the medical judgment of what should have been done (what should be done) rather than on whether the treatment was actually given (what was actually done).

In the event of treatment-related deaths, original NCI-CTCAE states that the causative adverse event should be Grade 5, but the outcome of the serious adverse event is reported in the SAE report and reviewed in detail. Therefore, Grade 5 of the institutional physician's judgment is not likely to be changed, and whether or not the serious adverse event will result in death will be significantly affected by other factors than the event is not appropriate, so it is not appropriate to compare the frequency of the adverse event by Grade (%Grade 4 and%Grade 5, respectively) between treatment groups or between studies. Because of the poor significance of distinguishing between Grade 4 and Grade 5 in the tabulation, Grade 4 is not considered "Grade 5" in the recording form of this study. A discussion of the causal relationship between adverse events observed in treatment-related deaths and deaths should be included in the "Situation at Death" section of the treatment completion report form and follow-up form, and an urgent report should be made. For the adverse event items specified in "8.2. Testing and Assessment during Treatment" and "8.3. Testing and Endpoints after Treatment Completion" that are determined to be Grade 5 in the post-hoc review including the emergency report, Grade and the date of the first occurrence of the event should be included in the relevant record form (Treatment Course Record Form). If Grade 3 or greater is observed for any other adverse event, or if Grade 3/2/1 adverse event and treatment requires at least 24 hours of hospital stay or prolongation of hospital stay (see 10.1.1.3.)), the AE and Grade and the date of first occurrence should be included in the free form of the treatment course record.

Any Grade on the record form should be recorded in the medical record. Confirmed during site visit audit.

8. Examination and Evaluation

8.1. Baseline examination and evaluation before registration

8.1.1. Test conducted before registration (regardless of time before registration)

1) Histopathology (immunostaining requires chromogranin A and synaptophysin) (see Section 3.3)
2) HBs antigen; HBc antibody ※1; HBs antibody ※1, HBV-DNA※2

※1: For HBsAg positive, HBc and HBs antibodies are not required, and HBV-DNA, HBeAg, and HBe antibodies are measured.

※2: Positive results for at least one of the HBc and HBs antibodies also indicate HBV-DNA prior to initiation of therapy (see Section 6.4.1).

8.1.2. Test performed within 56 days before enrollment (liver primary (or unknown primary))

If the liver is primary (or of unknown primary), the following tests should be performed (all allowing for tests performed in other hospitals):

1) Upper gastrointestinal endoscope
2) Lower gastrointestinal endoscope

3) FDG-PET test

4) Otolaryngology (head and neck) examination

5) Urology consultation (male only)

6) Gynecologic exam (female only)

8.1.3. Tests performed within 28 days before enrollment

1) Contrast-enhanced CT※1 (slice thickness of 5 mm or less, if the patient is allergic to iodine), both contrast-enhanced MRI of the abdomen and plain computed tomography (CT) of the imaging range, which is considered to be indispensable below, are performed. All tests are not performed in other hospitals.

※The following areas are indispensable for each primary organ, and if there is another site suspected of metastasis, the radiographic extent is added accordingly.

Primary Organ	Essential radiographic area
Esophagus	Cervical, chest, or abdominal regions
Stomach, duodenum, small intestine, colon, appendix, rectum,<br>Extrahepatic bile duct, Vater ampulla, gallbladder, and pancreas	Chest, abdomen, and pelvis
Hepatic NEC<br>(liver primary or liver metastasis of unknown primary)	Neck, chest, abdomen, and pelvis

2) Endoscopic ※2

Primary Organ	Mandatory test ranges
Esophagus, stomach, duodenum, and ampulla of Vater	Upper gastrointestinal tract (no examination performed at another hospital)
Small intestine, extrahepatic bile ducts, gallbladder, and pancreas	Be not mandatory
Colon, appendix, and rectum	Lower gastrointestinal tract (no examination performed at another hospital)
Hepatic NEC<br>(liver primary or liver metastasis of unknown primary)	8.1.2. Refer to the test performed within 56 days before registration.

※2 Unnecessary in recurrent case

3) 12-lead, resting electrocardiography

8.1.4. Tests performed within 14 days before enrollment

1) General condition: PS (ECOG), body weight

2) Physical findings

3) Peripheral blood count: white blood cell count, neutrophil count (ANC: rod + segmented karyocyte), hemoglobin, platelet count

4) Blood biochemistry: total protein, albumin, total bilirubin, AST (GOT), ALT (GPT), BUN, creatinine, LDH, ALP, sodium, potassium, calcium, magnesium, CRP, FBS (fasting blood glucose)

5) Creatinine clearance (CCr): CCr estimates by Cockcroft-Gault equation

Cockcroft-Gault formula

Male: Ccr = {(140-Age) × Body Weight (kg)}/{72 × Serum Creatinine Level (mg/dL)}

Women: Ccr = 0.85 × {(140-Age) × Body Weight (kg)}/{72 × Serum Creatinine Level (mg/dL)}

6) Tumour markers: NSEs, ProGRP, CEAs, SCCs (esophagus primary only), CA19-9 (non-esophageal primary only)

7) Chest X-P (frontal) (substitutable if contrast-enhanced and plain chest CT is obtained)

8.2. Examination and evaluation during treatment

The following safety examination and evaluation are minimal in frequency: Performing examinations more frequently at the discretion of the treating physician is not prohibited.

However, the examination for efficacy evaluation should be performed at specified frequencies, unless progression is suspected, because dense frequency may lead to bias in the efficacy evaluation.

8.2.1. Safety endpoint assessed weekly (CTCAE v4 0 Japanese translation)

The following 1)-3) tests or evaluations should be performed at least weekly. In addition, all of the following items should be checked and evaluated on the scheduled date or the day before anticancer drug administration. However, at least weekly examinations or evaluations should be performed in the same manner until day 28 using the starting date of the last course as day 1, even when the treatment is completed or terminated.

1） Peripheral blood count: white blood cell count, neutrophil count (rod + segmented count), hemoglobin, and platelet count
2） Biochemical tests: albumin, total bilirubin, AST (GOT), ALT (GPT), creatinine, sodium, potassium, calcium, magnesium, CRP
3） Subjective and objective findings (described according to CTCAE v4.0JAPANESE TRANSLATION)
   - General disorders and administration site conditions: fever, fatigue
   - Ear and labyrinth disorders: Tinnitus, hearing impairment
   - Skin and subcutaneous tissue disorders: alopecia
   - Gastrointestinal disorders: Constipation, diarrhea, nausea, vomiting, oral mucositis
   - Metabolism and nutrition disorders: anorexia, dehydration
   - Nervous system disorders: dysgeusia, peripheral sensory neuropathy, peripheral motor neuropathy
   - Musculoskeletal and connective tissue disorders: myalgia, arthralgia
   - Infections and parasites: bronchial infection, pulmonary infection, upper respiratory tract infection, mediastinal infection, pleural infection, catheter-related infection, biliary tract infection, gallbladder infection, and urinary tract infection
   - Blood and lymphatic system disorders: Febrile Neutropenia
   - Vascular disorders: Phlebitis
   - Respiratory, thoracic and mediastinal disorders: dyspnea, hypoxia, pneumonitis

8.2.2. Safety examination and evaluation for each course

1) General condition: Body weight
2) Blood chemistry: LDH, ALP FBS (fasting glucose)

8.2.3. Safety examination and evaluation to be performed as necessary

1） When dyspnea is observed
   - Chest X-P, percutaneous oxygen saturation: SpO2, arterial blood gases: PaO_2
2） If an arrhythmia is observed
   - 12-lead, resting electrocardiography
3） When HBs antigen, HBs antibody, or HBc antibody is positive
   - HBV-DNA (see 6.4.1.).

8.2.4. Efficacy end point

The following tests will be performed every 6 weeks during protocol treatment (±1 week allowed: 6 ± 1 week, 12 ± 1 week, and 18 ± 1 week after the start date of protocol treatment). Tumor response will be assessed according to "11.1. Response Evaluation". Evaluation of the response will be performed using the same test conditions and test methods as the baseline evaluation.

If CT is allergic to contrast material, it is evaluated with plain CT and/or contrast-enhanced MRI. Allergy to contrast media on MRI is assessed by plain CT or plain MRI. When the use of CT contrast medium and MRI contrast medium becomes difficult due to renal dysfunction, it is evaluated by simple CT or simple MRI.

1) Tumour markers: NSEs, ProGRP, CEAs (≧all cases), SCCs (esophagus primary only), CA19-9 (non-esophageal primary only)
2) Contrast-enhanced CT: In principle, the extent of imaging is as follows. () Areas in can be omitted if there is no lesion

Primary Organ	Essential radiographic area
Esophagus	Cervical, chest, or abdominal regions
Stomach, duodenum, small intestine, colon, appendix, rectum,<br>Extrahepatic bile duct, Vater ampulla, gallbladder, and pancreas	(chest), abdomen, and pelvis
Liver NEC (liver primary or liver metastasis of unknown primary)	(neck), (chest), abdomen, and pelvis

8.3. Examination and evaluation after completion of treatment

8.3.1. Efficacy evaluation after completion of treatment

After completion of the protocol treatment, examination and evaluation are made at the following timing:
The following tests should be performed at least every 6 weeks until progressions are confirmed or death.
If progressions are observed, only observation of the outcome will be continued.

1) Tumour markers: NSEs, ProGRP, CEAs (≧all cases), SCCs (esophagus primary only), CA19-9 (non-esophageal primary only)
2) Contrast-enhanced CT: In principle, the extent of imaging is as follows. Areas in parentheses can be omitted if there is no lesion

Primary Organ	Essential radiographic area
Esophagus	Cervical, chest, or abdominal regions
Stomach, duodenum, small intestine, colon, appendix, rectum,<br>Extrahepatic bile duct, Vater ampulla, gallbladder, and pancreas	(chest), abdomen, and pelvis
Liver NEC (liver primary or liver metastasis of unknown primary)	(neck), (chest), abdomen, and pelvis

8.4. Information on post- study treatment

After completion/termination of protocol treatment, the following items will be recorded on the Follow-up Form at each follow-up survey:

1) Content of post-study treatment (if post-study treatment is performed)
2) After protocol treatment termination, the initiation date of the first post-study treatment ((if post-study treatment is performed)
3) PS at the start of post-study treatment

8.5. Study calendar

If the primary site is the esophagus, stomach, duodenum, small intestine, colon, appendix, rectum, extrahepatic bile duct, ampulla of Vater, gallbladder, or pancreas

	Before registratikon Record Pre	After end of chemotherapy		From the date of protocol treatment discontinuation Within 28 days	Until progression after completion of protocol treatment	
		Course Before initiation of the course	During the course		Only at the start of post-treatment	Every 6 weeks
Physical findings						
Body weight	○^{14}	○				
PS	○^{14}				■	
Physical findings	○^{14}	○			■	
Laboratory tests						
WBC, differential (neutrophil) Hb, platelets	○^{14}	○	●	●		
Alb, T-Bil, AST, ALT, Cr, Na, K, Ca, Mg, CRP	○^{14}	○	●	●		
LDH, ALP, FBS	○^{14}	○				
Total protein, BUN, Ca	○^{14}					
NSE, ProGRP、CEA	○^{14}		△			
SCC (esophagus primary only), CA19-9 (other than primary esophageal)	○^{14}		△			
HBs antigen, HBc antibody, and HBs antibody	○^{前}					
Chest X-P (can be substituted if CT is taken)	○^{14}					
12-lead, resting electrocardiography	○^{28}					
Upper gastrointestinal endoscope (Primary: esophagus, stomach, duodenum)	○^{28}					
Lower gastrointestinal endoscope (Primary: colonic, appendix, rectum)	○^{28}					
Efficacy evaluation						
Contrast-Enhanced CT*	○^{28}		△			△
Toxicity evaluation						
Subjective symptom check		○	●	●		
Objective symptom check		○	●	●		
Submission of CRFs						
Pre-treatment Form		□				
Treatment Form			□			
Off-treatment Form				□		
Follow-up Form					2 times/year	

○^{前}: Conduct before registration, ○^{28}: Perform within 28 days before registration, ○^{14}: Perform within 14 days before registration

○:Conduct, ♀: Implementation at least once a week

△: Every 6 weeks (see 8.2.4.), ∞: Only once at the beginning of aftertreatment, □: Submitted.

*See 8.1.3 for the shooting range. Contrast-induced allergy or renal dysfunction is assessed by plain CT or contrast-

enhanced MRI.

※Follow-up Forms will be sent up to 1 years after completion of accrual and will be submitted after 1 years of registration in the individual patient according to the closing date of registration.

For liver NEC (liver primary or liver metastasis of unknown primary)

	Before registratiko n Record Pre	After end of chemotherapy		From the date of protocol treatment discontinuation Within 28 days	Until progression after completion of protocol treatment	
		Course Before initiation of the course	During the course		Only at the start of post-treatment	Every 6 weeks
Physical findings						
Body weight	○^{14}	○				
PS	○^{14}				■	
Physical findings	○^{14}	○			■	
Laboratory tests						
WBC, differential (neutrophil) Hb, platelets	○^{14}	○	●	●		
Alb, T-Bil, AST, ALT, Cr, Na,K, Ca, Mg, CRP	○^{14}	○	●	●		
LDH, ALP, FBS	○^{14}	○				
Total protein, BUN, Ca	○^{14}					
NSE, ProGRP、CEA、CA19-9	○^{14}		△			
HBs antigen, HBc antibody, and HBs antibody	○^{前}					
Chest X-P (can be substituted if CT is taken)	○^{14}					
12-lead, resting electrocardiography	○^{28}					
Upper gastrointestinal endoscope	○^{56}					
Lower gastrointestinal endoscope	○^{56}					
FDG-PET	○^{56}					
Otolaryngologic examination	○^{56}					
Urology consultation (male only)	○^{56}					
Gynecologic exam (female only)	○^{56}					
Efficacy evaluation						
Contrast-Enhanced CT*	○^{28}		△			△
Toxicity evaluation						
Subjective symptom check		○	●	●		
Objective symptom check		○	●	●		
Submission of CRFs						
Pre-treatment Form		□				
Treatment Form			□			
Off-treatment Form				□		
Follow-up Form					□ 2 times/year	

○^{前}: Conduct before registration, ○^{56}: Perform within 56 days before registration, ○^{28}: Perform within 28 days before registration,

○^{14}: Implemented within 14 days prior to enrollment

○:Conduct, ♀: Implementation at least once a week

△: Every 6 weeks (see 8.2.4.), ∞: Only once at the commencement of post-treatment, □: Submitted.

*See 8.1.3 for the shooting range. Contrast-induced allergy and renal dysfunction are evaluated by plain CT or contrast-enhanced MRI.

※Follow-up Forms will be sent up to 1 years after completion of accrual and will be submitted after 1 years of

registration in the individual patient according to the closing date of registration.

9. Data collection

9.1. Case Report Form (CRF)

9.1.1. Types of CRF and submission deadlines

The case report forms (CRF) used in this study and their submission deadlines are as follows:

1) Pre-treatment report (blue)- Less than 2 weeks after enrollment
2) Treatment course records- Every 2 cycles/less than 2 weeks after end of protocol treatment
   3)-1 Treatment (yellow)
   3)-2 Test (yellow)
   3)-3 Adverse events (yellow)
3) Tumor shrinkage report (green) - Less than 2 weeks after judgment of effect
4) End of treatment report (red) - Less than 2 weeks after discontinuation/end of protocol treatment
5) Follow-up investigation (white)- By the deadline indicated in the Follow-up Investigation Form

- For "1) Pre-treatment report to 4) End of treatment report", CRFs with basic patient information (enrollment code, facility name) pre-printed on them would be sent by post from the data center. If the CRFs do not arrive within one week of enrollment, or if the CRFs have been lost/damaged, the data center should be contacted by telephone, and a request should be made for them to be re-issued.
- "5) Follow-up investigation" would be sent by post from the data center at the time of follow-up investigations that are conducted at the same time as monitoring and interim/final analyses at the data center.

9.1.2. Storage CRF

- Completed CRFs must all be archived at the facility as photocopies or in electronic form.
- Copies of CRFs should be kept archived until the final analysis report is issued for reference while filling other CRFs, or for review while retrieving information from the data center.

9.1.3. Method of sending CRF

- All CRF must be sent by post or handed over in person at the data center. They must not be sent by FAX.
- To avoid the risk of personal patient information being leaked, the patient enrollment code should be used, instead of using the patient medical chart number at the facility, when contacting the data center for request of CRF dispatch.

9.1.4. Correction of the contents of CRFs

If any data necessary for the CRF are found to be missing or there are inappropriate category classifications after the start of the study, the CRFs may be corrected with the agreement of the head of the data center and the clinical trial secretariat, in a manner not exceeding the scope of data collected as prescribed in "8. Evaluation item/Clinical laboratory tests/Evaluation schedule", and within the scope deemed not to increase medical and financial burden on the enrolled patient from the CRF correction. Modification of CRFs that do not require the main body of the protocol to be revised is not considered a protocol revision by JCOG. Reports to the head of the medical institution related to CRF correction and the request of application for revision should follow the rules of the facility.

10. Reporting of "disease or the like"(adverse events)

Site investigator should report to Study Coordinator/Principal Investigator (Study Chair) if a serious adverse event ("disease or the like" on Clinical Trials Act) occurs in accordance with the regulations of Clinical Trials Act (Law No. 16, 2017), Enforcement Regulations of Clinical Trials Act (MHLW Notification No. 17, 2018) and the regulations in this chapter based on the relevant notifications.

The most recent version of the report is available on the MHLW website [1] and on the JCOG website [2]. Use the most recent version of the report.

Serious adverse events occurring after the initiation of protocol treatment (after the date of registration if death) by the date of final follow-up will be subjects.

1) http://www.mhlw.go.jp/stf/seisakunitsuite/bunya/0000163417.html
2) http://www.jcog.jp/doctor/todo/researcher/harmfulness.html
3) http://www.pmda.go.jp/safety/reports/hcp/pmd-act/0002.html

10.1. Serious Adverse Events

Serious adverse events are defined as any of the following:
(These are classified as "disease or the like" on Clinical Trials Act.)

1) Death
2) Diseases that may lead to death
3) "Disease or the like" requiring hospitalization or prolongation of hospital stay for treatment.
4) Disability
5) "Disease or the like" that may lead to disability
6) Serious "disease or the like" according to 1) to 5)
7) Congenital disease or abnormality in later generations

1) Death

(i) All deaths that occur after registration and before the start of protocol treatment
(ii) All deaths (with or without causality to protocol treatment) that occur during protocol treatment or within 30 days of the last treatment day
(iii) Death that occur after 31 days from the last treatment date that are causally related to protocol treatment (definite, probable, possible)

2) "Disease or the like" that may lead to death

(i) Grade 4 adverse events that occur during protocol treatment or within 30 days of the last treatment day (excluding events in Table 10.1)
(ii) Grade 4 adverse events that occur after 31 days from the last treatment date (excluding events in Table 10.1) that are causally related to the protocol treatment (definite, probable, possible)

3) Hospitalization or prolongation of hospital※1 stay for treatment

(i) Grade 3/2/1 adverse events that occur during or within 30 days of protocol treatment and requiring at least 24 hours of hospitalization or prolongation of hospital stay※1 to treat the adverse event (excluding the event in Table 10.1).
(ii) Grade 3/2/1 adverse events that occur 31 days after the last treatment day and requires 24-hour or longer hospitalization or prolongation of hospital stay※1 for treatment and causally related to protocol treatment (definite, probable, possible) (excluding the events in Table 10.1)

※ 1 "Hospitalization or prolongation of hospital stay" refers only to those for which hospitalization of at least 24 hours or prolongation of hospital stay is medically required for the treatment of an adverse event. The followings are not subjects for reporting:

- Hospitalization or prolongation of hospital stay performed for follow-up of adverse event that has disappeared or improved
- Hospitalization or prolongation of hospital stay for reducing patient burden, e.g. patients from distant areas.

- Hospitalization or prolongation of hospital stay for other medically unnecessary situation

4) Disability, 5) Disease that may lead to disability

Permanent or marked disability/dysfunction (excluding myelodysplastic syndromes, secondary cancers, etc.) or possible medical situation

6) Serious disease similar to 1) to 5)

7) Congenital disorders or abnormalities in later generations

Table 10.1. Adverse events excluded from the subjects of Expedited Reporting

SOC※ (CTCAE ver4.0)	AE term
Blood and lymphocyte disorders	Anemia, bone marrow hypocellular
Gastrointestinal disorders	Constipation
General disorders and local symptoms	Fever
Infections and infestations	Viral hepatitis
Clinical laboratory test	ALP increased, CD4 lymphocytes decreased, high cholesterol, GGT increased, lipase increased, lymphocytes decreased, neutrophils decreased, platelet count decreased, serum amylase increased, WBC decreased, hyponatremia, hypokalemia, hyperglycemia, and hypoglycemia
Metabolism and nutritional disorders	Obesity, anorexia, hyperuricemia, and hypoalbuminemia
Musculoskeletal and connective tissue disorders	Fibrosis deep connective tissue and superficial soft tissue fibrosis
Renal and urinary disorders	Chronic kidney disease
Respiratory, thoracic, and mediastinal disorders	Sinus disorder and sleep apnea
Skin and subcutaneous tissue disorders	Hypohidrosis

※ SOC:System Organ Class

10.2. Investigator's reporting requirements and procedures

10.2.1. Expedited Reporting

In the event of a serious adverse event, the Subinvestigator must promptly inform the Investigator. If the Investigator cannot be contacted, the Site Coordinator or Subinvestigator must take over the responsibility of the Investigator. The Investigator must report adverse events according to the following procedures.

Attention should be paid not to include the patient's name and medical record number when sent.

Serious adverse events that occur after the initiation of protocol treatment (after the date of registration if death) by the date of final follow-up are subjects of Expedited Reporting.

1) "Disease or the like" that may lead to death or death specified in 10.1 1) and 2).

Primary reporting:

The Subinvestigator who is aware of the occurrence of adverse events will promptly notify the Investigator. The Investigator who receives the notice should fill out JCOG Adverse Event Report Form(for institution) and the "Disease or the like" Report Form to Certified Review Board specified in Clinical Trials Act Enforcement Regulations as far as possible and contact Principal Investigator/Study Coordinator via e-mail within 72 hours of knowledge of the occurrence of the adverse event.

Secondary reporting:

The Investigator should add detailed information on adverse events to JCOG Adverse Event Report Form(for institution) and the "Disease or the like" Report Form to Certified Review Board specified in Clinical Trials Act Enforcement Regulations within 7 days of knowledge of the occurrence of adverse events and send them to the Principal Investigator/Study Coordinator via e-mail. If necessary, attach copies of laboratory data, images, autopsy report, etc.

2) 10.1. 3) Disease or other medically important condition requiring hospitalization or prolongation of hospital stay for treatment. Adverse events are judged to be either of 10.1. 4)-7)

The Subinvestigator who is aware of the occurrence of adverse events will promptly notify the Investigator. The Investigator who receives notice must fill out JCOG Adverse Event Report Form (for institution) and the "Disease or the like" Report Form to Certified Review Board specified in Clinical Trials Act Enforcement Regulations within 10 days of knowledge of the occurrence of an adverse event and send them to the Principal Investigator/Study Coordinator via e-mail. If necessary, attach copies of laboratory data, images, autopsy report, etc.

3) Additional reporting

If new information is obtained after conducting the above reporting, the Investigator must add information to JCOG Adverse Event Report Form (for institution) and the "Disease or the like" Report Form to Certified Review Board specified in Clinical Trials Act Enforcement Regulations and report it as needed.

Table 10.2.1. Summary of Adverse Events which are subjects for Expedited Reporting and the deadline of reporting to Principal Investigator/Study Coordinator

Causal relationship	The patient was hospitalized due to Grade 1-3 AE. Other medically important conditions		Grade 4		Death	
	Expected	Not expected	Expected	Not expected	Expected	Not expected
Present	Primary reporting: within 10 days Additional reporting: as needed	Primary reporting: within 10 days Additional reporting: as needed	Primary reporting: within 72 hours Secondary reporting: within 7 days Additional reporting: as needed			
None	<Only on-treatment or within 30 days of last protocol treatment day>					
	Primary reporting: within 10 days Additional reporting: as needed	Primary reporting: within 10 days Additional reporting: as needed	Primary reporting: within 72 hours Secondary reporting: within 7 days Additional reporting: as needed			

* 4) Disability, 5) "Disease or the like" that may lead to disability, and 6) "Disease or the like" that are serious similar to 1) to 5) in 10.1., 7) Congenital disorders or abnormalities in later generations

※ "Unexpected" refers to those not listed in "7. Expected Adverse Events"

Table 10.2.1. Summary of Adverse Events which are subjects for Expedited Reporting and the deadline of reporting to Principal Investigator/Study Coordinator

Causal relationship	The patient was hospitalized due to Grade 1-3 AE. *Other medically important conditions		Grade 4		Death	
	Expected	Not expected	Expected	Not expected	Expected	Not expected
Present	Primary reporting: within 10 days Additional reporting: as needed	Primary reporting: within 10 days Additional reporting: as needed	Primary reporting: within 72 hours Secondary reporting: within 7 days Additional reporting: as needed			
None	<Only on-treatment or within 30 days of last protocol treatment day>					
	Primary reporting: within 10 days Additional reporting: as needed	Primary reporting: within 10 days Additional reporting: as needed	Primary reporting: within 72 hours Secondary reporting: within 7 days Additional reporting: as needed			

* 4) Disability, 5) "Disease or the like" that may lead to disability, and 6) "Disease or the like" that are serious similar to 1) to 5) in 10.1., 7) Congenital disorders or abnormalities in later generations

※ "Unexpected" refers to those not listed in "7. Expected Adverse Events"

10.2.2. Reporting to the Administrator of participating medical organizations

If an Adverse Event which is subjects for Expedited Reporting occurs and is assessed as causal after reporting to Principal Investigator and reported to Certified Review Board, the Investigator must report it to the Administrator of the relevant medical institution in accordance with the requirements of the medical institution.

10.3. Responsibilities of Principal Investigator/Study Coordinator

10.3.1. Determination of necessity of suspension of registration and emergency notification to institutions

Principal Investigator/Study Coordinator who received the report from the Site Investigator should report to Group Chair and determine the urgency, significance, and impact of the report. If needed, take measures such as suspending registration (contacting JCOG Data Center and all participating institutions) and urgently communicating information to participating institutions. Telephone calls can be made to Data Center and institutions as urgent, but they should also be promptly contacted by document (e-mail).

10.3.2. Reporting to JCOG Operations Office and Certified Review Board and MHLW

1) Reporting from the Principal Investigator/Study Coordinator to JCOG Operations Office

Principal Investigator/Study Coordinator should consult with Group Chair and report to JCOG Operations Office (Safety Contact) by e-mail within 72 hours of knowledge of the occurrence of the adverse event, if reported AE is considered to meet the adverse events specified in 10.1.1 1)~7). In doing so, to the extent feasible, Principal Investigator/Study Coordinator should send "JCOG Adverse Event Report Form (for institution)" sent from the institution, "Disease or the like Report Form" addressed to Certified Review Board as stipulated in Clinical Trials Act Enforcement Regulations, and attach "JCOG Adverse Event Report (for Study Coordinator)" with Study Coordinator/Principal Investigator's view (including judgments of causality and expectation, and judgments of continuation/discontinuation of the study)". For the expected adverse events of 10.1.1 1)~7)., include a discussion not only of the individual patient's course but also of whether the frequency of appearance is within the expected range.

2) Reporting to Certified Review Board

JCOG Operations Office (Safety Contact) reviews the appropriateness of the judgement of causality and expectation of adverse events reported in the above procedures and can ask Principal Investigator/Study Coordinator to reconsider them if there is any doubt. Adverse events considered by Principal Investigator/Study Coordinator and JCOG Operations Office to be related to the protocol treatment and to be the subject of reporting in the following tables should reported to Certified Review Board through JCOG Operations Office.

If there is a disagreement between Principal Investigator/Study Coordinator and JCOG Operations Office, report it to Data and Safety Monitoring Committee and seek final judgment from the Chair of Data and Safety Monitoring Committee. However, if the reporting may exceed deadlines of reporting, the report can be tentatively reported as "causal" to Certified Review Board.

Reporting subjects and reporting deadline

Principal Investigator/Study Coordinator must report to Certified Review Board through JCOG Operations Office within the following time periods after knowledge of the occurrence of adverse events.

Causal relation ship	The patient was hospitalized due to Grade 1-3 AE. Other medically important condition		Grade 4		Death	
	Expected	Not expected	Expected	Not expected	Expected	Not expected
Present	No need of reporting	Within 15 days	Within 15 days	Within 7 days	Within 15 days	Within 7 days
None	No need of reporting	No need of reporting	No need of reporting	No need of reporting	No need of reporting	No need of reporting

※ "Unexpected" refers to those not listed in "7. Expected Adverse Events"

Principal Investigator/Study Coordinator must report adverse events to Certified Review Board through JCOG

Operations Office within the following deadlines after knowledge of the occurrence of adverse events.

Causal relationship	The patient was hospitalized due to Grade 1-3 AE. Other medically important condition		Grade 4		Death	
	Expected	Not expected	Expected	Not expected	Expected	Not expected
Present	Within 30 days	Within 15 days	Within 30 days	Within 15 days	Within 15 days	Within 15 days
None	No need of reporting	No need of reporting	No need of reporting	No need of reporting	No need of reporting	No need of reporting

※ "Unexpected" refers to those not listed in "7. Expected Adverse Events"

3) Reporting to MHLW

Principal Investigator/Study Coordinator of the study with unapproved or off-label health care should report "Disease or the like" Report Form specified in Clinical Trials Act Enforcement Regulations to MHLW through JCOG Operations Office, if an adverse event is considered to be unexpected and to have a causal relationship to the protocol treatment (See 10.5. Responsibilities of the Data and Safety Monitoring Committee).

Reporting subjects and reporting deadlines

Principal Investigator/Study Coordinator should report to the MHLW through JCOG Operations Office within the following deadlines after knowledge of the occurrence of adverse events.

※ Pharmaceuticals and Medical Devices Agency Safety Division I (trk-shippeitouhokoku@pmda.go.jp)

Causal relationship	The patient was hospitalized due to Grade 1-3 AE. Other medically important condition		Grade 4		Death	
	Expected	Not expected	Expected	Not expected	Expected	Not expected
Present	No need of reporting	Within 15 days	No need of reporting	Within 7 days	No need of reporting	Within 7 days
None	No need of reporting	No need of reporting	No need of reporting	No need of reporting	No need of reporting	No need of reporting

※ "Unexpected" refers to those not listed in "7. Expected Adverse Events"

4) Additional reporting

Following receipt of secondary or additional reports from the Investigator, Principal Investigator/Study Coordinator must add additional information from the primary report and their views to JCOG Adverse Event Report Form (for the institution) and the "Disease or the like" Report Form to Certified Review Board set out in Clinical Trials Act Enforcement Regulations, and promptly contact JCOG Operations Office (Safety Contact) by e-mail. If the report was sent to Certified Review Board and the MHLW in the primary reporting,, the secondary reporting and additional reporting must be made in the same manner.

10.3.3. Notification to the Site Investigators

When reported to Certified Review Board, Principal Investigator/Study Coordinator should inform the Investigators of all participating institutions of the review results and recommendations by documents (e-mail is allowed). Principal Investigator/Study Coordinator must inform the Investigators without waiting for Certified Review Board review if there is any urgent information to be disseminated. In the event that reported to the MHLW, Principal Investigator/Study Coordinator should notify the Investigators of all participating institutions.

In addition, even if no reporting is made to Certified Review Board, Principal Investigator/Study Coordinator must inform the Investigator of the reporting institution of the decision of Principal Investigator/Study Coordinator by documents (e-mail is allowed).

10.3.4. Assessment of Adverse Events in Periodic Monitoring

During Periodic Monitoring, Principal investigator/Study Coordinator should carefully review the adverse events

in the Monitoring Reports issued by the Data Center and ensure that there are no missed reporting from the participating institutions. It should also be confirmed that all reported adverse events are listed in the Monitoring Reports. The presence or absence of a missed reporting should be indicated in the column of the results of Group review on the Periodic Monitoring Report.

10.4. Responsibilities of the Site Investigators at the participating institutions (including the relevant institution)

In accordance with the instructions of Principal Investigator/Study Coordinator, the Site Investigator at the participating institution should report to the administrator of the relevant institution if the adverse event is subjects of reporting of "disease or the like" to Certified Review Board in accordance with the regulations of the relevant institution.

10.5. Responsibilities of the Data and Safety Monitoring Committee

JCOG Operations Office (Safety Contact) should check the details of the adverse event reports received from the Principal Investigator/Study Coordinator according to the procedures described in 10.3.2. and should report them to Certified Review Board and the MHLW according to the procedures described below, with the presence or absence of causality or expectation.

In addition, Principal Investigator and Director of Data Center can hear the opinions of JCOG Data and Safety Monitoring Committee according to the reported adverse events. If a review request is issued, Data and Safety Monitoring Committee can review the appropriateness of the institutional response to adverse events and the propriety of continuation of the study in a consensus or written form.

In addition, the submitted information (JCOG Adverse Event Report (for institution), JCOG Adverse Event Report (for Study Coordinator), "Disease or the like" Report, etc.) will be stored semi-permanently in JCOG Operations Office.

Subjects, destination and deadlines for reporting after the knowledge by Investigator/Study Coordinator are as described in 10.3.2.

11. Response Evaluation and Endpoint Definition

11.1. Response assessment (only for patients with measurable disease)

Tumour response assessment will be performed according to the following steps according to the [49] of version 1. 1-Japanese translational JCOG version-Revised RECIST guideline (version 1. 1) Revised new guidelines for the assessment of treatment response in solid tumours (RECIST guidelines). RECISTv1. The 0 original article stipulates that "the use of this guideline for the purpose of determining continuation of treatment is not the subject of this guideline." Similar statements continue to be included in RECISTv1 1 as follows.

> "Many oncologists make decisions about whether to continue treatment based on both objective imaging criteria and symptom-based criteria for follow-up of patients with malignancies in their daily clinical practice, but these revised guidelines are not intended to be used to make decisions about whether or not to continue treatment in these individual patients, unless the treating oncologist determines that it is appropriate."

Therefore, the "overall effect" as determined by RECIST Guideline-based response assessment should be used to determine whether a drug or regimen shows encouraging results that merit continued developmental studies. In other words, judgment of whether or not to continue treatment in individual patients should not be based on CR/PR/SD/PD of overall efficacy, but rather on "clinical judgment" based on comprehensive consideration of symptoms, physical findings, and various laboratory data, in addition to imaging findings.

Therefore, it may be clinically appropriate to continue protocolized treatment, even when PD (Progressive Disease: progression) is judged as an overall response based on the assessment of response based on imaging. In this case, the pros and cons of continuing protocol treatment should be determined based on clinical judgment, regardless of response assessment, but the date of the event for progression-free survival, which is judged to be an overall effect of PD, should be used. This is due to three reasons: (i) it may be possible to decide whether protocol treatment should be continued for each group; (ii) RECIST is a criterion intended to standardize not only response rates but also progression-free survival; and (iii) the standard definition of US Cooperative Group is that PD is the event of progression-free survival for any reason if the overall response is PD.

On the other hand, if a physician judges "clinical progression" based on clinical and comprehensive judgment not based on diagnostic imaging, even if PD is not met by the response criteria based on diagnostic imaging, protocol treatment should be discontinued in accordance with "6.2.2. Criteria for discontinuation of protocol treatment". If "clinical exacerbation" is judged, even if "PD" is not judged by the response evaluation, the day of "clinical exacerbation" is considered as an event of progression-free survival. This is because imaging is often not performed as planned after a patient is judged to have a "clinical progression" and therefore the risk of overestimating progression-free survival is greater if "clinical progression" is not an event for progression-free survival. It is also statistically incorrect (informative censoring) to treat "clinical progression" as "censoring" progression-free survival, as it would censoring patients at increased risk of progression or death.

In RECISTv1 1, the original article described "definite progression (unequivocal progression") in the PD criteria for non-target lesions as "marked progression of non-target lesions that deserves discontinuation of treatment as an increase in total tumor burden" and therefore described "marked progression of non-target lesions" as "judgement of whether or not to continue treatment in individual patients" in some of the PD criteria for non-target lesions, which is confusing. It should be noted that this "unequivocal progression" is a criterion of judgment restricted to "PD of non-target lesions".

The relation between the events of 'PD', 'clinical progression', 'progression', and progression-free survival in JCOG is as in the lower panel.

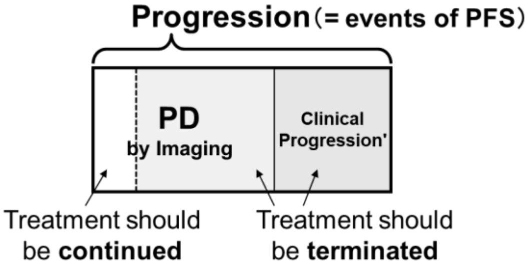

Figure 11.1. Relationship Between Exacerbations, PD on Imaging, and Clinical Exacerbations.

11.1.1. Baseline Evaluation

「8.1. According to "Pre-registration Evaluation Items" contrast enhanced-enhanced computed tomography (CT) with a range of indispensable for the primary organ is used to identify neoplastic lesions before enrollment, and each lesion is classified into "measurable lesions" and "unmeasurable lesions". If CT is allergic to contrast medium, both contrast-enhanced MRI and plain CT should be performed.

Tumor diameters are measured by CT or MRI in cross-sectional views, and sagittal and coronal measurements by three-dimensional reconstructed images are not used. Baseline assessment will be done using the latest imaging studies within 28 days prior to enrollment. If the imaging test is re-examined after enrollment and before the start of treatment, the latest imaging test with re-examination should be used.

11.1.2. Definition of measurable lesions

Lesions that fall under any of the following conditions are considered measurable lesions (measurable lesion):

1) Non-nodal disease (non-nodal disease) of 10 mm or greater in greatest dimension on CT or MRI with a slice thickness of 5 mm or less
2) CT or MRI of 5 mm or less slice thickness showing lymph node lesion of 15 mm or more in short diameter (Nodal lesions with short diameters between 10 mm and less than 15 mm are defined as non-target lesions, and those with short diameters less than 10 mm are not)

All other lesions will be non-measurable (non-measurable lesion).

Caution should be exercised because the following lesions are not measurable regardless of the examination method or the size of the lesion.

- Bone lesions (excluding osteolytic lesions with measurable soft tissue components)
- Cystic lesion
- Leptomeningeal lesions
- Ascites, pleural effusion, and pericardial effusion
- Lymphangiosis of the skin and lungs
- Palpable but not measurable abdominal mass or enlargement of abdominal organs
- Superficial skin lesions

11.1.3. Target Lesion Selection and Baseline Recording

Up to five measurable lesions, in descending order of diameter (non-lymph node lesions are long diameters and nodal lesions are short diameters), up to two lesions per organ are selected to be target lesions (target lesion) among measurable lesions at enrollment. Selection should take into account the universal inclusion of as many organs with measurable disease as possible and the reproducibility or ease of measurement (reproducible repeated measurement) of repeated measurements (avoiding lesions that are not measurable even if they are large in diameter).

For selected target lesions, in order from cranial to caudal, site (code), test method, test date, long diameter of non-lymph node target lesion, short diameter of nodal target lesion, and sum of all target lesion diameters (hereafter, sum of diameters) will be recorded in Pretreatment Report 3.

11.1.4. Baseline recording of non-target lesions

For lesions not selected as target lesions, all measurable or non-target lesions (non-target lesion) should be recorded as site of lesion (code), method of examination, and date of examination in Pretreatment Report 3. Multiple non-target lesions within the same organ may be recorded as a single lesion (e.g., multiple enlarged pelvic lymph nodes, multiple liver metastases).

11.1.5. Determining tumor response

Evaluation of target and non-target lesions will be performed every 6 weeks according to "8.2 Testing and Evaluation during Treatment" in the same manner as at enrollment. Target lesion diameter, non-target lesion disappearance or progression will be recorded in the "Tumor Reduction Effect Report".

11.1.6. Response Evaluation Criteria for Target Lesions

・CR(Complete Response): Complete response

When all non-lymph node target lesions disappear and all nodal target lesions have a short diameter of less than 10 mm. If a nodal target lesion is selected at baseline, the effect of the target lesion may be CR

even if the sum of diameters is not 0 mm.

・PR(Partial Response): Partial response

30% or more reduction in target diameter sum compared to baseline diameter sum

・PD(Progressive Disease): Progress

Compared to the minimum diameters (when the baseline is the minimum value during the passage, this is the minimum sum of diameters), the sum of the target disease increases by more than 20%, and the sum of diameters increases by more than 5 mm even in absolute value.

・SD(Stable Disease): Stability

There is no reduction corresponding to PR and no increase corresponding to PD compared to the smallest sum of diameters during the course

・Lack of study; Not all Evaluated

If the test cannot be performed for any reason or if neither CR, PR, PD, or SD can be determined

Pre-treatment sum of diameters-sum of diameters at study
Percentage of reduction of the diameter sum =---------------× x 100%
Pre-treatment diameter sum

Sum of diameters at study-minimum sum of diameters
Increasing Percentage of Diagram =---------------× x 100%
Minimum sum of diameters

※ Measured target lesion diameters are recorded whenever measurable (e.g., <5 mm). If the target lesion diameter is judged to be too small to be measured (too small to measure), the diameter should be 0 mm if the tumor lesion is judged not to be retained, and 5 mm if the tumor lesion is judged to be retained, regardless of the CT slice thickness.

※ PD is defined when the reduction ratio meets the condition of PR and the concomitant increase ratio meets the condition of PD.

※ When one lesion is separated during treatment, each diameter is added to the sum of diameters.

※ If more than one lesion fuses and the boundary cannot be distinguished during treatment, the diameter of the fused lesion is added to the sum of the diameters. The diameter of each lesion is added to the sum of diameters when the boundary of the lesion is identifiable, even if the lesion is in contact with each other.

11.1.7. Response Evaluation Criteria for Non-Target Lesions

・CR(Complete Response): Complete response

When all non-lymph node non-target lesions disappear, the short diameter of all nodal non-target lesions becomes less than 10 mm, and all tumour markers (NSEs, ProGRP※) are below the upper shared reference limits.

Because ※ProGRP is not included in the shared baseline range, the baseline range is 6.5-46.0 pg/mL.

・Non-CR/non-PD: non-CR/non-PD

Residual one or more non-target lesions (including residual nodal non-target lesions ≧10 mm in short diameter) and/or tumour markers (NSEs, ProGRP※) exceeding the shared upper reference limits.

・PD(Progressive Disease): Progress

'Apparent exacerbation' (including relapse) of pre-existing non-target lesions.

For measurable disease: A marked progression of a non-target lesion that deserves discontinuation of treatment as an increase in overall tumor burden must be observed if the effect of the target lesion is SD or PR but is judged to be "clear progression" based on the change in the non-target lesion. If the effect of the target lesion is SD or PR, then an increase in the tumor burden of the non-target lesion to a degree that far exceeds the decrease in tumor burden is considered "obvious progression" and otherwise Non-CR/non-PD.

If only unmeasurable disease is present, the increase in non-target disease, as judged to clearly exceed the tumor burden corresponding to a 20% increase in diameter and a 73% increase in tumor volume, is

considered "definite progression".

・NE(Not all Evaluated): Lack of study

If the test could not be done for any reason or if neither CR, Non-CR/non-PD nor PD could be determined.

11.1.8. Presence or absence of new lesions

If a lesion that was not present at baseline was observed after the start of treatment, it is considered "new lesion" to be present.

However, a "new lesion" requires that it is not an imaging change due to a difference in the imaging method from the baseline assessment or a change in the imaging modality, nor is it an imaging change due to a condition other than the tumor. For example, a cystic lesion arising within a lesion due to necrosis of a liver metastatic lesion is not a new lesion. New lesions will be defined as new lesions by examination of sites that were not mandatory at baseline (pre-enrollment study).

If a lesion disappears and later reappears, measurement is continued. However, the effect at the time the lesion reappears depends on the status of the other lesion. When the overall effect reappears after CR, the lesion is judged as PD at the time of reappearance. When the overall effect is PR or SD, on the other hand, once the disappeared lesion reappears, the diameter of the lesion will be added to the sum of the diameters of the remaining lesions to calculate the effect. That is, in the presence of many residual lesions, even if one lesion reappears after an apparent disappearance, it is not judged as PD by itself, and it is judged as PD when the sum of the diameters of all lesions meets the criteria for PD. This is because of the perception that the majority of lesions do not truly 'disappear' and are not only depicted by the limits of resolution of the imaging modalities used.

If there is a possibility of a new lesion but it cannot be determined, it should not be a new lesion, and imaging should be reexamined at a clinically relevant time. If a new lesion is confirmed by repeat imaging, the new lesion will appear based on the date of imaging at which the new lesion is confirmed.

11.1.9. Overall efficacy (Overall Response)

The overall response (Overall response) will be determined by combining the effects of target lesions, non-target lesions, and the presence or absence of new lesions every 6 weeks according to Table 11.1.9.a below. The overall effect in the absence of a non-target lesion at baseline will be determined by the effect of the target lesion and the presence or absence of a new lesion, and the overall effect in the absence of a target lesion at baseline will be determined according to the effect of a non-target lesion and the presence or absence of a new lesion according to Table 11.1.9.b.

Table 11.1.9.a. Overall efficacy at each time point for target lesions (with or without non-target lesions)

Target lesion	Nontarget lesions	New lesions	Overall effect
CR	CR	None	CR
CR	Non-CR/non-PD	None	PR
CR	Lack of study	None	PR
PR	Lack of Non-PD or study	None	PR
SD	Lack of Non-PD or study	None	SD
Lack of study	Non-PD	None	NE
PD (obvious progression)	Irrespective of	With or without	PD
Irrespective of	PD	With or without	PD
Irrespective of	Irrespective of	Present	PD

Table 11.1.9.b. Overall efficacy at each time point for patients with non-target lesions only

Nontarget lesions	New lesions	Overall effect
CR	None	CR
Non-CR/non-PD	None	Non-CR/non-PD
Lack of study	None	NE
PD (obvious progression)	With or without	PD
Irrespective of	Present	PD

11.1.10. Best overall effectiveness (Best Overall Response)

CR > PR > SD > PD > NE is considered good, and the best overall effect is the best overall effect throughout the entire course.

PD is defined when imaging cannot be determined due to exacerbation of obvious disease or death before the first response assessment. In addition, NE is defined if it cannot be determined by imaging due to discontinuation of toxicity before the first response assessment or patient refusal.

11.2. Definitions of analyses set

The analysis sets used in periodic central monitoring, interim analysis, and final analysis are defined as follows: The flow diagram below shows the analysis sets.

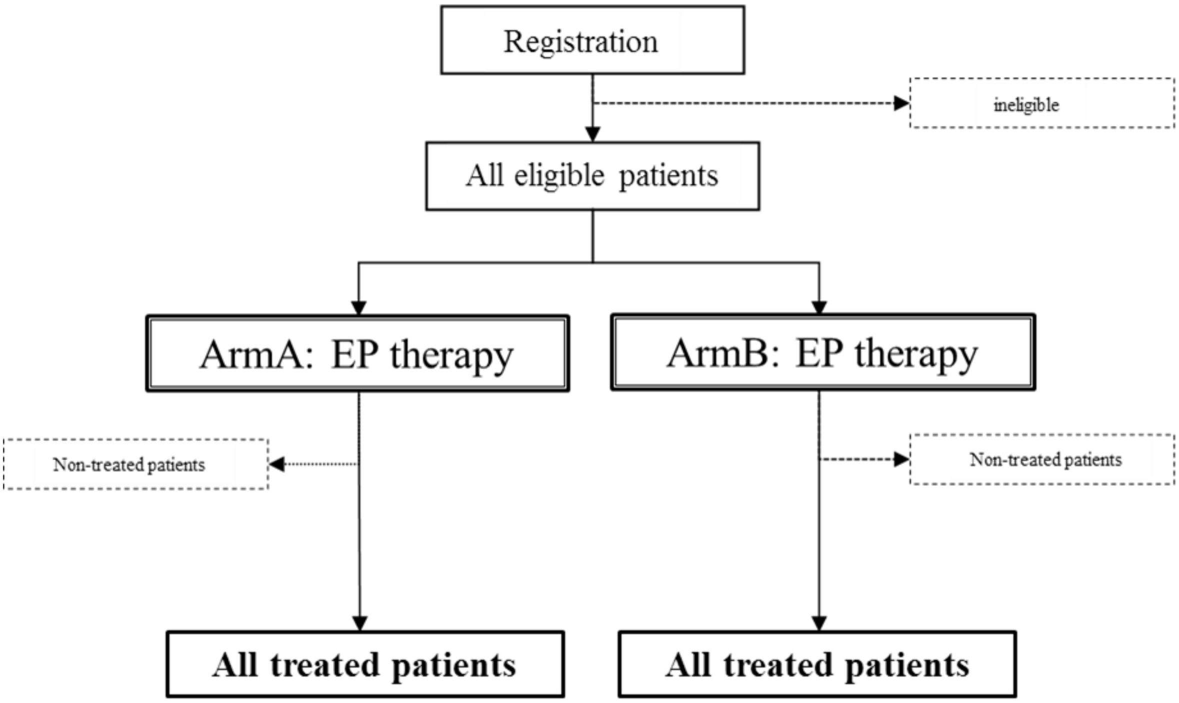

11.2.1. All registered patients

「5.1. Among the patients enrolled according to the Procedures for Enrollment, the population excluding duplicate or mis-enrollment is considered as "all enrolled cases".

11.2.2. All eligible patients

The group excluding "ineligible cases (post hoc ineligibles, de facto ineligibles, violation of registration)" determined by group review from all registered patients is regarded as all eligible patients. Ineligible cases as judged by the investigator or sub-investigator alone are included in all eligible patients. Only those judged not eligible by the central pathological diagnosis are ineligible and not included in all eligible patients.

11.2.3. All treated patients

Of all enrolled patients, all patients for whom part or all of the protocol treatment was performed will be defined as all treated patients.

The decision to treat "non-treated patients" for whom no protocol treatment has been given and whether it is excluded from the safety analysis can be determined by the data center with the consent of the Research Office. Ineligible patients will be excluded from all treated patients. However, if there are circumstances in which ineligible patients are included in the analysis, the nature of the ineligibility will be examined and determined by the Research Secretariat in consultation with JCOG Data Centre.

11.2.4. Patients eligible for central pathology diagnosis

Of all eligible patients, the population excluding ineligible patients with a central pathological diagnosis will be considered as eligible patients with a central pathological diagnosis.

11.3. Definition of endpoints

Endpoint	Events (whichever is earlier)		Censoring date
Overall survival time<br>Overall survival(OS)	All deaths	-	Date of final survival confirmation
Progression-free survival time<br>Progression-free survival(PFS)	All deaths	Progression/recurrence	Final date of clinically confirmed freedom from exacerbation

11.3.1. Overall survival

The duration from the date of registration to the date of death from any cause.

- Survivors are censored at the date of final survival confirmation (survival confirmation by telephone contact is also permitted, but the fact that survival confirmation was performed should be recorded in the medical record).
- Patients lost to follow-up are censored at the last date of survival confirmation before lost to follow-up.

11.3.2. Progression-free survival (PFS: Progression-free survival).

The duration from the date of registration to the date of the judgement of exacerbation or death from any cause, whichever comes first.

- The exacerbation (progression) includes both imaging-based PD (progression) and exacerbation of pathogenic disease (clinical exacerbation) that cannot be confirmed by imaging studies in Section 11.1.9. Global Effectiveness. If an exacerbation is judged based on diagnostic imaging, the exacerbation date is the test date on which the imaging was performed, and in the case of clinical exacerbation, the date of clinical judgment is the exacerbation date. PD may occur in accordance with the Response Evaluation Criteria, even if the tumor diameter is very small, but the disease is clinically judged to be "not obviously aggravated" in accordance with the Response Evaluation Criteria Criteria (in this case, clinical judgment should be prioritized for continued treatment with the protocol). In addition, even if PD is not observed according to the response evaluation criteria, the clinical judgment is prioritized for progression if it is judged to be clinically obvious exacerbation.
- In survivors who are not judged to have progression, clinically confirmed progression is censored (date of final progression-free survival confirmation) (confirmation of progression-free by imaging or specimen examination is not mandatory and clinical progression-free by outpatient examination etc. is not permitted. Telephone contact alone is not permitted. If information on progression or progression-free is obtained at a medical institution or referral center, receive and retain a medical information form describing the rationale for diagnosis. In this case, telephone contact alone is not permitted).
- Events and censoring are treated similarly, if chemotherapy is terminated for reasons such as toxicity or patient refusal, and if other therapies are added as post-study treatment. i.e., it is not censored at the time of treatment termination or at the date of initiation of post-study treatment.
- When the diagnosis of exacerbation is based on imaging, the relapse is not regarded as an event at the test date of imaging with "suspicious diagnosis of relapse", but regarded as an event at the later test date of imaging with "definite diagnosis of relapse"" If the event is judged to be clinically aggravated based on diagnostic imaging, the event is considered to be the day of the progression.
- If the definitive diagnosis of relapse or new lesion is based on biopsy pathology, the date of clinical diagnosis is defined as the date of clinical diagnosis when the diagnosis of recurrence or new lesion is made clinically, and the date of biopsy is defined as the date of event when the diagnosis of recurrence is made based on biopsy pathology diagnosis without clinical diagnosis of recurrence.
- The incidence of a second cancer (metachronous double cancer) is not censored or event, and progression-free survival is defined until other events are observed.

11.3.3. Response rate (response rate) Response proportion (Response rate)

Among all enrolled patients with measurable disease, the response rate is defined as the proportion of patients with "11.1.10. Best Global Effect" that is either CR or PR.

11.3.4. Incidence of adverse events (adverse reactions)

Using all treated patients as the denominator, the frequency of the worst Grade during the entire course by CTCAE v4 0 Japanese translation JCOG version for each of the following adverse events (toxicities) will be determined by group:

- Laboratory tests: hemoglobin decreased, white blood cell decreased, neutrophil count decreased, platelet count decreased,
  Increased blood bilirubin, aspartate aminotransferase increased (AST or GOT),
  Alanine aminotransferase increased (ALT or GPT), creatinine increased,
  Hypernatremia, hyponatremia, hyperkalemia, hypokalemia, hypercalcemia, hypocalcemia,

hypermagnesemia, hypomagnesemia

- General disorders and administration site conditions: fever, fatigue
- Ear and labyrinth disorders: Tinnitus, hearing impairment
- Skin and subcutaneous tissue disorders: alopecia
- Gastrointestinal disorders: constipation, diarrhea, nausea, vomiting, oral mucositis
- Metabolism and nutrition disorders: anorexia, dehydration
- Nervous system disorders: dysgeusia, peripheral sensory neuropathy, peripheral motor neuropathy
- Musculoskeletal and connective tissue disorders: myalgia, arthralgia
- Infections and parasites: bronchial infection, pulmonary infection, upper respiratory tract infection, catheter-related infection, biliary tract infection,
  Gallbladder infection; Mediastinal infection; Pleural infection; Urinary tract infection
- Blood and lymphatic system disorders: Febrile Neutropenia
- Musculoskeletal and connective tissue disorders: arthralgia, Vascular disorders: phlebitis
- Respiratory, thoracic and mediastinal disorders: dyspnea, hypoxia, pneumonitis

In the other adverse event (toxicitiy) than the above, the proportion of occurrence are not calculated unless a large number of specific adverse events are observed, since only Grade 3 or more non-hematological toxicity ※ is reported in the Treatment Form.

※※ Non-hematological toxicity refers to adverse events other than those listed below in CTCAE v4.0-JCJCOG.
Anemia, decreased bone marrow cells, decreased lymphocyte count, decreased neutrophil count, decreased white blood cell count, and decreased platelet count.
CD4 lymphocytopenia

11.3.5. Dose intensity of cisplatin

Dose intensity of cisplatin per patient (DOOC.) will be calculated for all treated patients to assess treatment compliance with cisplatin. The summary statistics (minimum, 25% point, median, 75% point, maximum, mean, and standard deviation) will be calculated for each group.

- ・Active dose D.I. (mg/m2/week) = total drug dosebody surface areatreatment duration (weeks)
- Body surface area: BSA is calculated by height at enrollment and body weight calculated by Data Center.
- ・ Treatment period (weeks)
  Arm A = (start date of last course-start date of 1st course + 21)/7
  Arm B = (start date of last course-start date of 1st course + 28)/ 7

11.3.6. Incidence of serious adverse events (adverse reactions)

1) Grade 4 non-hematologic toxicities, early deaths, and treatment

Using all treatments as denominators, the percentage of patients with one or more Grade 4 non-hematologic toxic ※ that are considered to be related (either to definite, probable, possible) to the protocol treatment among the adverse events listed in the free text of the CRFs in addition to the stereotyped items in Section 11.3.4 is used as the numerator.

※※ Non-hematological toxicity refers to adverse events other than those listed below in CTCAE v4.0-JCJCOG.
Anemia, decreased bone marrow cells, decreased lymphocyte count, decreased neutrophil count, decreased white blood cell count, and decreased platelet count.
CD4 lymphocytopenia

2) Early mortality rate

Proportion of all deaths during the protocol treatment or within 30 days from the last protocol treatment day among all treated patients. Causes of death irrespective of causality with protocol treatment. However, if premature death occurs in patients excluded from all treated patients, the details are provided separately.

3) Proportion of treatment-related death (TRD incidence)

Proportion of all deaths judged as causally related (either definite, probable, possible) to the protocol treatment among all treated patients However, if TRD occurs in patients who have been removed from all treated patients, the details are presented separately.

12. Statistical consideration

Methods for statistical analysis are as follows: In addition, the details required for conducting specific analyses are specified in the statistical analysis plan prepared separately prior to the analysis, and in documents that clarify the endpoint definition, etc. If substantial changes occur in statistical analyses as described below, follow the policy set out in "13.6. Protocol Changes." Facilities are contacted by "14.1. Periodic Monitoring" for missing or abnormal results, and data collection or exclusion is determined by review by the Research Secretariat based on the results of inquiries to the institution in accordance with the policies stipulated in "14.1.2. eligible (eligibility/ineligibility)" and "14.1.3. Protocol Deviations/Violations". 「11. Handling of missing values and abnormal data that cannot be addressed by the definition and analysis method for each endpoint, as specified in "Definition of Response Evaluation and Endpoints" and "12. Statistical Items" below, is specified in the above statistical analysis plan.

12.1. Principal Analysis and Decision Criteria

The primary analysis of this study will be the final analysis.

The purpose analysis of this trial is to test whether one of the two community standard-of-care arm A (EP-therapy) and B (IP-therapy) outperforms primary endpoint overall survival. The null hypothesis of equal overall survival in the two groups in the main analysis will be tested by stratified log-rank test stratified by non-institutional allocation adjustment factors (primary organ [gastrointestinal vs. hepatobiliary-pancreatic]) in all patient patients. However, if it is assumed that a stratified log-rank test cannot be performed appropriately, such as when the number of subjects and events in each stratum is small, the allocation adjustment factors will be addressed in the analysis plan prepared without information related to the comparison between groups before performing a confirmatory analysis with comparison between groups. Sensitivity analysis will also be performed in all eligible patients and in patients eligible for central pathology diagnosis.

Due to interest in which treatment group is superior, the test will be two-tailed. The study-wise significance level for the study is set at both sides 10%. In the main analysis, two-sided 90% confidence intervals corresponding to a two-sided 10% significance level will be calculated, and in other analyses, two-sided 95% confidence intervals will be calculated for descriptive purposes.

We conclude that EP therapy is a more useful treatment when the two-sided p-value is less than 10% and the survival curve of EP therapy exceeds that of IP therapy. Meanwhile, we conclude that IP therapy is a more useful treatment when the two-sided p-value is less than 10% and the survival curve of IP therapy exceeds that of EP therapy. If the difference was not significant at a two-sided significance level of 10%, one of the two modalities cannot be judged to be useful. In this case, we conclude that both modalities continue to be the standard of care, as there is no evidence to actively recommend either one of them.

However, if there are major differences in the toxicity profiles of EP and IP therapies, contrary to prior assumptions, during the course of the study, and if the toxic therapy is not superior to other therapies, it is judged that it will not be of significance to be used in clinical practice, the following measures should be taken. In other words, the clinical hypothesis will be changed from two-sided to one-sided in an analysis plan with no information on group comparisons before a confirmatory analysis with group comparisons will be conducted, and the superiority of the toxicity treatment over other treatment modalities will be verified at a one-sided significance level of 5% (in this case, the policy of change for the interim analysis will be described in Section 12.3.2).

Estimates such as cumulative survival curves, median survival times, and annual survival rates are performed using Kaplan-Meier method. Brookmeyer and Crowley methods are used to obtain 95% confidence intervals for median survival. Greenwood's formula is used to obtain 95% confidence intervals for annual survival rates. Hazard ratios and their confidence intervals for treatment effects between groups are obtained using stratified Cox proportional hazards models with the same factors as the test of the primary analysis as estimates of treatment effects. Cox regression adjusted by imbalance background factors in addition to adjustment factors will be performed as needed.

The main analysis results will be summarized as the "Main Analysis Report" by the Data Center one year after the completion of enrollment and submitted to the Research Secretariat, Research Representatives, Group Representatives, Group Secretariats, Efficacy and Safety Assessment Committee, and JCOG Representatives.

The principal investigator/study coordinator summarizes the content of the main analysis report, prepares a

"Clinical Study Report" summarizing the conclusions, problems, interpretations and discussion of the results, and future policies of the entire study, and submits it to the Data and Safety Monitoring Committee and JCOG chair with approval from the Group chair and the Head of JCOG Data Center.

Approval of the clinical study report by the Data and Safety Monitoring Committee shall be considered as "end of the study."

12.2. Planned accrual, accrual period, and follow-up periods

「2.4.2. Based on the background presented in Clinical Hypothesis and Rationale for Number of Enrollment, we assume a median survival of 8 and 12 months (HR=0.67) for the inferior and superior treatment groups, respectively, among the two treatment groups. When a superiority trial design is used, 62 patients per group and 124 patients in both groups (114 events required) will be included in the required analysis using Schoenfeld & Richter methodological [50] at 6 years of enrollment, 1 year of follow-up, α = 10% (two-sided), and 70% power. The number of required Inferior treatment (number of required events) when the median survival time in A is different from the assumption as shown in Table 12.2.1 below.

Table 12.2.1. Number of Analyses Required (Number of Events Required)

	Power			
Median Survival (mo)	65%	70%	75%	80%
7.0 vs. 10.5	108(100)	124(114)	142(131)	162(150)
8.0 vs. 12.0	112(100)	126(114)	144(131)	166(150)
9.0 vs. 13.5	114(100)	130(114)	148(131)	170(150)

※※ Number of required events in parentheses

Based on these findings, the following will be established in view of some cases of loss to follow-up.

Planned enrollment: 70 patients in each group and 140 patients in both groups

Enrollment period: 6 years, follow-up period: 1 year after completion of enrollment

Consider redesigning the sample size if the prognosis is obviously better than assumed, or if it reaches 70 people within 2.5 years of enrollment initiation. Clinically meaningful differences will then be reviewed and redesigned in a blinded fashion prior to the conduct of the analysis.

12.3. Interim Analysis and Early Termination of the Study

12.3.1. Purpose and Timing of the Interim Analysis

Once interim analyses will be conducted to determine if the primary objective of the study has been achieved during the study period. Interim analyses will be conducted to determine if it is reasonable to continue enrollment during enrollment. If the primary objective of the study is determined to be achieved, the study will be discontinued and the study results will be published promptly at the conference and in the article.

Interim analyses will be conducted using data from the initial periodic monitoring that will be queried after the time enrollment of half of the planned enrollment was obtained. Based on the information in the periodic monitoring report, the group will submit the presence or absence of changes in clinical hypotheses and, if any, details of changes to the Efficacy and Safety Assessment Committee by the time of the interim analysis.

During the first interim, patient accrual is continued in principle. If the progress of the study progressed as planned, the expected number of events at the time of the interim analysis under the assumptions presented in 12.2 is expected to be 44 if the interim analysis is conducted at 3 years after the start of enrollment.

12.3.2. Method of interim analysis

Interim analyses will be conducted by the JCOG Data Center. To keep the study-wise alpha errors at 10%, the multiplicity of the interim and final analyses is adjusted using Lan & DeMets's alpha spending functions, and statistical significance is examined for differences in primary endpoint between arms. As α spending functions, we use O'Brien & Fleming types.[51]

For details of the interim analysis, the statistical staff in charge of the group at the Data Center will prepare the statistical analysis plan by the time of the interim analysis. Actual interim analyses will be performed by statistical staff who are not in charge of the group and an interim analysis report will be prepared.

In the interim analysis, if the survival of one treatment group exceeds that of the period, and the p-value of the stratified log-rank test falls below the level specified by the above method, it is judged statistically significant and the trial is discontinued as a rule. The ineffective discontinuation is not planned at a stage where no statistically significant differences are observed unless prior assumptions regarding toxicity are changed. However, the clinical hypothesis may be changed for the reasons described in 12.1, and the primary analysis may be performed by one-sided rather than two-sided testing, which may result in deviations from prior assumptions. In such cases, the primary hypothesis change should be specified in the analysis plan to be prepared without information on group comparisons before a confirmatory analysis with group comparisons. If the overall survival curve in the highly toxic treatment group is below the other, the consideration of ineffective discontinuation should not be judged by a test and considered comprehensively.

12.3.3. Interim analysis Reporting and review of the results

The results of the interim analysis will be submitted to the Data and Safety Monitoring Committee by the Data Center as an Interim Analysis Report and reviewed for the acceptability of continuation of the study and for publication of the results. The Data and Safety Monitoring Committee considers whether to continue the study at the meeting and recommends whether to continue the study and whether to publish the results to principal physician or group chair based on the results of the review.

Members of the Data and Safety Monitoring Committee of the relevant group are not included in the review. Unless the results of the interim analysis make recommendations for discontinuation of the study from the Efficacy and Safety Assessment Committee, the research representative, research office, participating institution researchers, group representatives, and group secretaries of the study will not be able to know the results of the interim analysis until the final follow-up is completed.

When the Interim Analysis Report has been reviewed by the Data and Safety Monitoring Committee to recommend termination or change of all or part of the study, the principle investigator and group chair review the recommendations and decide whether to discontinue or change some of the study.

If the study is discontinued or part of the study is changed, the principal investigator and group chair shall submit in written form a request to the Data and Safety Monitoring Committee for permission to discontinue the study or a request to revise the protocol. Following approval by the Data and Safety Monitoring Committee, the principal investigator may discontinue the study or change part of the study.

The Study Chair and Group Chair can disagree with the recommendations of the Data and Safety Monitoring Committee, but if they fail to coordinate their opinions with the Data and Safety Monitoring Committee, they will ultimately follow the instructions of JCOG Chair.

If the study is terminated, the subsequent follow-up period will be 1 years from last registration per study.

If the interim analysis resulted in study termination, the interim analysis will be the primary analysis of the study. The Data Center, in cooperation with the Research Representative Physicians and Research Secretariat, will conduct the analysis required to complement the incomplete data and publish the results, focusing on the results of the interim analysis, and promptly prepare the Major Analysis Report and submit it to the Group and the Efficacy and Safety Assessment Committee.

12.4. Analysis of Secondary endpoints

Secondary endpoints analyses will be conducted to provide a supplementary discussion of the primary analysis results of the study. Because the analysis of secondary endpoint is exploratory, no multiplicity adjustments are made. Comparisons between arms are made where appropriate, note that when the results of the group comparisons are not significant, they do not mean that there is no difference between the two arms.

12.4.1. Analysis of safety secondary endpoints

Among Secondary endpoints, the safety endpoints are the incidence of adverse events and the incidence of serious adverse events, which are in principle the items of periodic monitoring (14.1. Periodic monitoring).

The incidence rate of adverse events will be summarized as well as the incidence rate of Grade3 or higher. For adverse events other than laboratory data, the incidence of Grade 2 or higher is also calculated.. Non-hematological toxicity incidence, early mortality, and treatment-related mortality rates of Grade4, which are serious adverse events, are reported in periodic monitoring reports with registration numbers and details. The rates of non-hematologic

toxicity, early mortality, and treatment-related mortality for Grade4 will be calculated at the time of the interim analysis and the main analysis. When interval estimation of proportions is performed, accurate confidence intervals based on binomial distributions are used. Comparisons between arms will be made using Fisher's exact test where appropriate.

12.4.2. Analysis of efficacy secondary endpoints

Among Secondary endpoints, efficacy endpoints are response rate, progression-free survival, which will only be analyzed in the interim and primary analyses.

Secondary endpoints analyses do not adjust for multiplicity.

Progression-free survival will be included in all enrolled patients, but a comparison of all eligible patients, excluding ineligible patients, as determined by group study, will also be performed as a sensitivity analysis.

The response rate will include all enrolled patients with measurable disease, but a comparison of all eligible patients excluding ineligible cases determined after group review will also be performed as a sensitivity analysis.

Fisher's exact test will be used to compare response rates between groups, and binomial distribution-based exact confidence intervals will be used for interval estimation. Estimates, including progression-free survival curves, median progression-free survival, and time-point progression-free survival, will be performed using Kaplan-Meier method, Brookmeyer and Crowley methods will be used to obtain confidence intervals for median progression-free survival, and Greenwood formulas will be used to obtain confidence intervals for progression-free survival. Log-rank test is used for comparison between arms. Hazard ratios and their 95% confidence intervals for treatment effects between arms will be calculated using Cox's proportional hazards model as an estimate of treatment effect. Cox regression adjusted by imbalance background factors in addition to adjustment factors will be performed as needed.

12.5. Final analysis

The primary analysis will be the final analysis unless this study is withdrawn from the interim analysis.

If the interim analysis is withdrawn from the study, then after the end of the 1-year follow-up period, the final analysis will be performed after the final survey confirms the data and then analyses will be performed for all endpoints.

Except for the interim analyses and the final analysis, analyses with between-arms comparisons for the primary and secondary endpoints for efficacy are not performed unless approved by the Data and Safety Monitoring Committee.

If the final analysis is performed after the main analysis, the final analysis will be summarized by the Data Center as the "Final Analysis Report" and submitted to the Research Secretariat, Research Representatives, Group Representatives, Group Secretariats, Efficacy and Safety Assessment Committee, and JCOG Representatives.

The Study Representative Physician/Research Secretariat summarizes the content of the final analysis report, prepares the "Clinical Study Report" summarizing the conclusions, issues, interpretations and discussion of the results, future policies, etc. mainly from the clinical point of view (if the "Clinical Study Report" is prepared in the previous analysis report, it will be the "Clinical Study Report (Supplementary Version)) with additional updates), and submits it to the Study Representatives and JCOG Representatives with approval from the Group Representatives and the Head of JCOG Data Center.

Approval of the clinical study report by the Data and Safety Monitoring Committee shall be considered as "end of the study."

12.6. Exploratory analysis

To investigate the interaction between treatment effect and the subpopulation, subgroup analyses will be conducted exploratory with respect to the following factors: Because these analyses are not adequately powered and do not adjust for multiplicity, the results of each subgroup analysis should be interpreted as exploratory.

- PS0/1
- Age 65 years or older/<
- Gender (male/female)
- Primary organ (gastrointestinal tract/hepatobiliary pancreas)
- Organ of origin (esophagus/stomach/small intestine/large intestine/pancreas/biliary tract/liver NEC (liver

primary or liver metastasis of unknown primary)

- Organ of origin (pancreas/non-pancreas)
- Extent of extension of the primary lesion (locally advanced/distant metastasis or recurrence)
- Extent of extension of the primary lesion (locally advanced/distant metastasis/recurrence)
- Prior radical resection of the primary lesion (none/present)
- Pathological diagnosis was biopsy/resection specimen
- Grade 3 tumour with morphologically similar features of NETs but Grade 3 proliferative activity/morphologically more atypical (previously classified as poorly differentiated endocrine carcinoma) on histopathology with central pathology
- Histopathological examination by central pathological diagnosis, including Small cell carcinoma/Large cell carcinoma /
- Histopathologically diagnosed by central pathology, Ki67 50% or higher/less than 50%

12.7. Premature withdrawal from the trial

In this study, early termination of the study may occur in the following cases:

1) Early termination due to discontinuation of interim analysis
2) Early termination due to adverse events
3) Early termination due to poor enrollment
4) Early termination due to other reasons

12.7.1. Early termination by interim analysis

In this study, based on the criteria described in 12.3, early termination recommendations may be made at the interim analysis review by the Efficacy and Safety Assessment Committee. If the Data and Safety Monitoring Committee provides recommendations for early termination of the study, the principle investigator and group chair will review the recommendations and decide whether to terminate the study early.

12.7.2. Early termination due to adverse events

In JCOG9511 for small-cell lung cancer, 1/77 (1.3%) treatment-related deaths were reported with EP therapy and 3/77 (3.9%) with IP therapy; in JCOG0509, a successor study for the same subject, 1/142 (0.7%) treatment-related deaths were reported with IP therapy and 2/142 (1.4%) with amrubicin plus cisplatin therapy. Using these as reference, we believe that the treatment-related mortality rate should not exceed 3% in this study. Since it is clear that the final point estimate will be at least 3% at the time of 3 treatment-related deaths in either group, immediate entry should be suspended to consider whether or not to withdraw from the study. At this point, the subsequent treatment of the patient being treated will be reviewed. At the time of 3 or fewer treatment-related deaths in each group, each patient will be reported to the Efficacy and Safety Assessment Committee for adjudication. Enrollment will be continued until the results are obtained in principle.

12.7.3. Early termination due to poor enrollment

If the patient enrollment pace is significantly worse than at the time of planning, early termination of the study may be advised by the Data and Safety Monitoring Committee. If early termination recommendations are issued by the Data and Safety Monitoring Committee due to poor enrollment, the principle investigator and group chair will review the recommendations and decide whether to terminate the study early.

12.7.4. Early termination due to other reasons

12.7.1.～12.7.3. If it is judged difficult to continue the study for other reasons, the research representative physician shall submit a request for early termination of the study to the Efficacy and Safety Assessment Committee. If the Data and Safety Monitoring Committee recommends early termination of the study based on the submitted data, the procedure for early termination of the study will be progressed.

12.8. Procedures after Early termination of the Study

If the Study Chair accepts the recommendations made by the Data and Safety Monitoring Committee based on Section 12.7, he/she will promptly submit a notification to the Data and Safety Monitoring Committee that early termination of the study will be performed.

The Study Chair will submit a termination notification to the Certified Review Board within 10 days of the date

they decide to terminate the study early. If the study falls into a specified clinical trials under the Clinical Trials Act, the Study Chair shall submit a termination notification to the Certified Review Board within 10 days of the date on which the study was decided to be prematurely discontinued, as well as submit a specified clinical trials termination notification to the MHLW.

The Study Chair promptly informs the investigator of the decision to terminate the study early in writing, and the investigator who has received a report of early termination of the study will report in writing that the study was prematurely terminated to the institution's manager without delay.

If the study is terminated early, JCOG Data Center will promptly initiate the development of the primary analysis report or final analysis report. The subsequent follow-up period will be 1 year from the final enrollment.

13. Ethical Considerations

13.1. Protection of Human Subjects

All researchers involved in this study will conduct this trial in accordance with the "Helsinki Declaration" [1] and "Clinical Trials Act" (2017 Law No. 16) [2] "Clinical Trials Act Enforcement Regulations" (2018 Ministry of Health, Labour and Welfare Order No. 17) and related notices.

1) http://www.med.or.jp/wma/helsinki08_j.html
2) http://www.mhlw.go.jp/stf/seisakunitsuite/bunya/0000163417.html

Prior to commencing this study, the site investigator is required to obtain input from Certified Review Board※1 regarding the conduct of the study, and to obtain approval from the Administrator of the participating institution, and submit the Implementation Plan ※2 to the Ministry of Health, Labour and Welfare.

※ 1 JCOG study will be submitted to the following Certified Review Board.
National Cancer Center Hospital Certified Review Board (accreditation number CRB3180008)

※ 2 "Implementation Plan" means "Documented plan formatted according to the Form No.1 (Form No.1 of the Ministerial Ordinance) specified in Article 39 of the Clinical Trials Act Enforcement Regulations"

13.2. Informed consent

13.2.1. Explanation to the patient

Prior to patient registration, the investigator or subinvestigator will provide the patient with written informed consent form approved by Certified Review Board and explain the following details verbally.

Descriptions

1) Disease names, Stages, and expected prognosis
2) That this study is a clinical trial and is conducted by JCOG
   Name of Certified Review Board and contact information for receiving complaints and inquiries to the committee
3) Design and rationale of the study
4) Protocol treatment content
5) Effects expected by protocol treatment
6) Expected adverse events, complications, and sequelae and how to deal with them
   Explanation of the extent and frequency of expected adverse events, including complications, sequelae, and treatment-related deaths, and how to deal with them when they occur. In addition to these explanation, obtain the most recent version of the drug package insert and deliver it to patients (PMDA Prescription Pharmaceutical Information Search http ://www.pmda.go. jp/PmdaSearch/iyakuSearch/)
7) Cost burden and compensations
   Explanation of the cost of treatment, compensation that can be received in the event of a health hazard (equivalent to measures taken in general practice, etc.)
8) Alternative treatment
   Explanation of treatments that can be received if not participating in this study
9) Anticipated benefits and possible disadvantages
   Explanation of anticipated benefits and possible disadvantages by participating in this study
10) Direct access to the medical records
   Explanations on acceptance of site visit audits, such as "direct access to medical records etc. by healthcare professionals at other medical institutions for quality control with permission from the administrator of the participating institution."
11) Refusal of consent and withdrawal of consent
   Refusal to consent prior to participation in the study is free, and withdrawal after having given consent is free, thereby not causing undue medical disadvantage.
12) Protecting human rights
   Every effort should be made to ensure that personal information, such as names, is kept confidential.
13) Secondary use of data
   The possibility of secondary use of data obtained from this study in Japan and overseas (ancillary studies, meta-

analyses, etc.) only when approved by either committee in JCOG

14) Freedom of questions
Explanation that investigators, written contact information for consultations on study details, on the Principal Investigator and the Study Coordinator, and freely asking questions about study and treatment

15) Explanation of the use and burden of medicinal products not covered by insurance

16) Central pathological review

17) Central image review for response

18) Sample collection for ancillary studies

13.2.2. Consent

Patients are requested to participate in the study after it has been explained to them and they have been given sufficient time for consideration. They must confirm that they fully understand the nature of the study. If the patient consents to study participation, written informed consent is obtained using the attached Consent Form or another document provided by the medical institution concerned and which the patient has signed. Investigators must confirm that the name of the doctor who provided the explanation, the date of the explanation, the name of the patient who consented after receiving the explanation, and the date of consent are all indicated on the Consent Form.

Two copies of the Consent Form are made. One is given to the patient and the other is kept by the Site Coordinator. The original Consent Form is kept in Medical Records or at a storage location stipulated by the medical institution.

13.3. Protection of personal information and patient identification

13.3.1. Policies, legislation, and regulations complied with by JCOG

When it conducts studies, JCOG complies with the legislation and regulations listed below in addition to all other policies not appearing in the following list.

- Protection of Personal Information Act (No. 57 of May 30, 2003; Latest version: Amendment of Act No. 65 of 2015
- WMA Declaration of Helsinki
- Ethical Guidelines for Clinical Research

13.3.2. Purpose of personal information collection, items used, and method of use

<u>1) Purpose of use</u>

In line with JCOG's guiding philosophy of "providing more patients with the best methods of treatment," personal information will be used to identify individual patients during treatment and over the long term after the end of the protocol. In this way, accurate results can be obtained from the clinical studies and the data can be appropriately managed.

<u>2) Items used</u>

The following items are regarded by JCOG as the minimum required to identify and refer to patients: patient ID (medical record number), date of birth, initials, and pathological sample number (when required)

<u>3) Method of use</u>

Patient personal and medical data are noted in the various CRFs by investigators at medical institutions. They are collected when the CRFs are submitted to the Data Center either by mail or hand delivery. Telephone or fax may also be used but only for patient registration which must be transmitted immediately.

13.3.3. Secondary use of data

Data obtained in this study may be used for secondary purposes such as meta-analyses. However, the data must be presented in such a way that it is not linked to patient identification. Moreover, its use for these purposes must first be reviewed and approved by the JCOG committee concerned (example: the Protocol Review Committee).

13.3.4. System of Responsibility for Safety Management

Privacy Protection Managers and Privacy Protection Officers will be delegated. Appropriate safety management measures will be implemented to minimize the risk of leakage of personal information.

13.3.5. Disclosure of patient information

Requests from patients for the disclosure of privacy-related data kept by JCOG will be handled by study personnel

(Site Principal Investigator, Site Coordinator, or investigators) at the medical institutions where the patients are being treated.

13.3.6. Contact details for general inquiries and complaints

General inquiries and complaints about the privacy policy can be submitted by mail, e-mail, or fax using the contact information provided below:

Inquiries: JCOG Data Center Privacy Protection Officer

Address: 5-1-1 Tsukiji, Chuo-ku, Tokyo 104-0045, Japan
National Cancer Center Hospital Clinical Research Support Office
E-mail: JCOG_privacy@ml.JCOG.jp
Fax: 03-3542-3374

13.4. Compliance with protocol

Research personnel participating in this study will comply with this protocol as long as it does not violate patient safety or human rights.

13.5. Approval by medical institution IRBs

13.5.1. Approval at the start of study participation

Medical institutions participating in this study must approve this protocol and the fact that the study is performed with written patient consent.

13.5.2. Review and approval of the state of progress of the study and study continuation

Regular reporting of the state of progress of the study, the occurrence of adverse events, and the procedures for reviewing study continuation are carried out in accordance with the regulations of the medical institution concerned.

13.6. Protocol modifications

13.6.1. Categories of protocol modifications

An Application for Protocol Revision must first be submitted to and approved by the Data and Safety Evaluation Committee before protocol modifications can go into effect. However, this protocol revision procedure is not required when the registration period is to be extended for < 6 months.

1) Protcol amendment
2) Protocol revision
3) Memorandum

13.6.2. Approval of Protcol amendment/revision in institutions

13.6.3. Correction of CRF: omit

13.7. Management of conflicts of interest (COI) on the part of persons involved in JCOG studies

COI on the part of the personnel involved in or supporting JCOG studies are managed as described below.

- COIs on the part of Site Principal Investigators, Site Coordinators, and other persons involved in JCOG studies and engaged in medical care at participating institutions will be managed according to the regulations of those institutions.
- COIs on the part of persons playing a central role in JCOG studies, such as the Study Chair, the Study Coordinator, the Group Chair, or the Group Coordinator are managed by the JCOG COI Committee. COIs on the part of members of JCOG committees such as the Data and Safety Evaluation Committee and JCOG Data Center/Executive Secretariat staff are managed in the same way.

13.8. Compensation

With respect to health damage resulting from participation in this study, treatment appropriate to the condition is provided under health insurance in the same way as regular clinical treatment. Patients are responsible for their own copayments. No financial compensation in the form of condolence payments or subsidies will be provided.

13.9. Intellectual property

The results, data, and intellectual property derived from this study shall be submitted to the following four persons/bodies: the Study Chair, the Study Coordinator, the Group Chair, and the National Cancer Center.

Disposition of these instruments will be decided by consensus. Distribution of the intellectual property will be determined in accordance with the arrangements made by the medical institutions concerned.

14. Monitoring and audit

14.1. Periodic monitoring

In this study, monitoring is performed in order to ensure that the study is conducted safely and in accordance with this protocol and that data are collected accurately. Periodic monitoring should be performed twice a year in principle, using the entered data on the CRFs collected in the Data Center. Specific procedures for periodic monitoring are provided separately in the Monitoring Plan.

The Data Center submits a "Monitoring Report" summarizing the results of central monitoring to Principal Investigator, Study Coordinator and investigators. Together, they are submitted to Group Chair, Data and Safety Monitoring Committee, and JCOG Chair.

The Monitoring Report is a material for periodic reports to be made every year from the date of submission of the Implementation Plan.

14.1.1. Monitoring items

① Registration status: number of registration - cumulative/by month, by arm/site
② Eligibility: ineligibles/potentially ineligible cases: by arm/site
③ Pre-treatment baseline factors: by arm
④ On/off-treatment, reason for treatment termination: arm/site
⑤ Protocol deviation: arm/site
⑥ Serious Adverse Events: arm/site
⑦ Adverse reaction/event: arm
⑧ Overall survival, progression-free survival (or relapse-free survival, etc.): all registered patients
⑨ Other issues related to study progress and safety (studies in accordance with Clinical Trials Act: status of occurrence of non-compliance and subsequent response, number of subjects for compensation, number of disease or the like reports in accordance with Article 13 of Clinical Trials Act)

14.1.2. Eigibility (Eligible/Ineligible)

For all registered patients, eligibility will be classified according to the following definitions as either: In monitoring, Data Center lists potentially ineligible cases in the "Evaluation of Eligibility" section of the monitoring report, and after review by Study Coordinator, confirms them to be either 1), 2), 9), or 99) with Group Chair approval prior to performing primary analysis.

Only 1) eligible shall be "eligible case", 2) post hoc ineligible,9) de facto ineligible and 99) violation of registration shall be "ineligible case". This is a category established from the perspective of analysis set setting.

In the study in accordance with Clinical Trials Act, "99) violation of registration" will be regarded as "major non-compliance" in Clinical Trials Act, and Principal Investigator will promptly report the situation to Certified Review Board as soon as possible. See 14.3. for management of non-compliance.

9) de facto ineligibles corresponds to "non-compliance" on Clinical Trials Act and is reported to the administrator of participating medical organizations with the submission of the monitoring report on which they were described (twice a year).

2) Post hoc ineligibles is not treated as "non-compliance" on Clinical Trials Act because it does not correspond to non-compliance with the study protocol, as discussed below.

1) Eligible

All information generated prior to registration meets all o the Patients Selection Criteria according to the methods and criteria specified in the study protocol.

2) Post hoc ineligible

The information generated after registration does not meet either Patients Selection Criteria, or the information generated prior to registration does not meet either Patients Selection Criteria by methods or criteria other than those specified in the protocol.

Examples)

(i) In the study for Stage II-III, bone scintigraphy performed immediately after registration revealed bone

metastases, and the patient was diagnosed as Stage IV. The protocol treatment was terminated.

(ii) In the study for early gastric cancer, bloody stools is seen after registration, and colonoscopy revealed advanced colorectal cancer (synchronous double cancer). Colectomy was performed after termination of the protocol treatment.

(iii) In the study for gastric cancer (adenocarcinoma), the institution's pathological diagnosis was changed to malignant lymphoma after registration.

9) De facto ineligible

Information generated prior to registration according to protocol-specified methods (performed in all cases) and criteria does not meet either Patients Selection Criteria. This includes cases where it is determined after registration that the information that occurred before registration had been incorrect.

Example: When the supervising physician reviews the CT images performed before registration as specified, there is obvious liver metastasis (if it is a mistake by the attending physician and it is considered that there is no future).

99) Violation of registration

Deliberately (falsely) enroll while knowing that Patients Selection Criteria is not met. Corresponds to a misconduct and treats it as a serious problem.

14.1.3. Protocol Deviations/Violations

Protocol deviations are defined as those in which treatment, such as drug administration, radiotherapy, or surgical resection, as well as laboratory tests and evaluation of toxicity and efficacy, etc. were not performed according to the protocols.

In monitoring, deviations that exceed a certain acceptable range limit for each study decided by the Data Center and Principal Investigator/Study Coordinator prior to or after the initiation of the study are listed in the monitoring report as "possible deviations" and are classified into one of the following categories after consideration by Study Coordinator and study groups: Except for those described in the protocol and monitoring report, the acceptable deviations agreed between the Data Center and Principal Investigator/Study Coordinator may be changed through periodic monitoring during the study, so they should be described in the supplemental material rather than in the text of the Monitoring Plan, and the Monitoring Plan should be described as "defining the acceptable ranges separately".

1) Violation

Any deviation from the protocol that is clinically inappropriate and caused by the treating physician/institution and that meets two or more following criteria shall be classified as a violation. [When conducted in accordance with Clinical Trials Act, the following shall be added. [In the study according to Clinical Trials Act, the violation shall be treated as "major non-compliance" and Principal Investigator should report the situation to Certified Review Board as soon as possible].

① Have a substantial impact on the assessment of study endpoints

② Intentional or systematic

③ Dangerous or remarkable deviation

For "violations", in principle, the content of each violation should be described in a paper when publishing.

2) Deviation

Deviations that do not fall into 1) violation or 3) acceptable deviation. If same kind of deviations are frequent, they should preferably be included in the publication of the article. They are classified as either of the following at the time of monitoring report review:

Because deviations correspond to "non-compliance" in Clinical Trials Act, they are reported (biannually) to the administrators of the institution with the submission of the monitoring report. Not to be "major non-compliance".

(i) Deviations - Undesirable and to be reduced

(ii) Deviations (unavoidable) - things that are not proactively reducing (e.g. delay by the New Year period, equipment breakdown, etc.)

(iii) Deviations (clinically relevant) - Those in which the decision of the treating physician/institution are positively affirmed (if a similar situation again arises similar deviations are considered desirable).

※ Deviations do not always mean that the treating physician at the institution is problematic. Since patient safety is a primary priority in clinical trials, deviations should rather be made by the medical judgment of the treating physician if the condition of the individual patient are considered to be dangerous when following the protocols. If the deviation is judged to be clinically relevant for the safety of the patient, it is recorded as ③ Deviation (clinically relevant). Clinically relevant deviations in a small number of patients are not required to be particularly problematic; however, protocol revision should be considered when multiple deviations occur because protocol specification is likely to be inadequate. However, deviations that are not intended to be safety (e.g., increased doses of anticancer drugs in the hope of increasing efficacy, shortened treatment periods not specified in the protocol) are not considered clinically relevant deviations.

3) Acceptable deviation

Deviations from protocols within acceptable range agreed by the entire JCOG, study groups, or Study Chair/Study Coordinator and Data Center, pre- or post-study initiation, on a trial-by-trial basis. When conducted according to Clinical Trials Act, the following are added. Not considered to be "non-compliance" in Clinical Trials Act. Deviations within the pre-specified acceptable ranges are not included in the monitoring report.

14.2. Site visit audits

In this study, site visit audits will be conducted to ensure the reliability of clinical research and the reliability of data and information collected by clinical research from the perspective of protecting human subjects in clinical research.

Site visit audits are conducted by auditors appointed by Principal Investigator by visiting a medical institution to confirm the approval documents of the medical institution, check the list of subinvestigators in the research institution, confirm the informed consent documents, and verify CRF entry data with medical records (direct access to source documents). Specific procedures for site visit audits are provided separately in the operating procedures.

The auditor shall report the audit report summarizing the audit results to Principal Investigator/Study Coordinator, site investigators. Together, the report will be submitted to Group Chair, Director of JCOG Data Center, Director of JCOG Operations Office, and JCOG Chair. Reports should also be submitted to the site investigators of the relevant groups and JCOG Executive Committee as appropriate.

14.2.1. Items to be audited

In the site visit audits, the following items are checked by direct access to source documents:

<Confirmation Items by Study>

- Approval documents (including initial approval forms, revision approval forms, and annual report approvals/reports) from administrators of medical institutions
- Management status of the protocol
- Contents of explanatory documents and informed consent forms

<Confirmation Items by Patient>

- Patient consent (presence or absence of consent form, signature, and date of consent)
- Implementation of pre-registration mandatory tests, eligibility for registration (inclusion criteria and exclusion criteria)
- Accuracy of reported data
  - Pre-treatment evaluation, course of treatment (protocol treatment)
  - Various test results (including diagnostic imaging reports and pathology reports)
  - Accuracy of test date, response evaluation, adverse event, survival or death information, and other reported data

<Other items>

- Presence or absence of study misconduct (possibility of false reporting, fabrication, or falsification)
- Improvement status of the items pointed out in the previous audit

14.2.2. Reporting of major non-compliance found in audits to Certified Review Board

Principal Investigator/Study Coordinator should report to Certified Review Board immediately when finding possible "major non-compliance (See 14.3.2.)" as a result of site visit audits.

14.3. Management of non-compliance

14.3.1. Non-compliance.

Non-compliance in Clinical Trials Act refers to the condition in which clinical research is not compliant with Clinical Trials Act Enforcement Regulations or study protocols. In the Clinical Trials Act Enforcement Regulations, etc. (February 28, 2018), non-compliance with regulations, study protocols, operation procedures, etc., and fabrication of falsification of research data, etc. are listed as examples.

If the site investigator knows that there is non-compliance, the site investigator should report to the administrator of the medical institution and inform Principal Investigator/Study Coordinator.

If site investigator find non-compliance (regardless of major non-compliance or not) prior to the implementation of central monitoring or site visit audits, the site investigator will promptly report to Principal Investigator/Study Coordinator and JCOG Data Center.

"14.1.3.2) Deviations" correspond to "non-compliance" in Clinical Trials Act. As described in 14.1.3.2), these deviations are reported by submitting monitoring reports or their excerpts or summaries issued twice a year to the administrators of the medical institution.

Changes in protocols and implementation plans associated with investigator transfer require Certified Review Board review and notification of implementation plans to the MHLW, therefore, require a certain time to complete the sequence of procedures. In addition, it is often difficult to complete the change procedure before the transfer because the transfer may not be open until just before the transfer. Therefore, even if the site investigator is absent for a certain period of time due to the transfer, the study does not fall into "non-compliance" in this study if the research management system is maintained by the subinvestigator and the medical care system of the enrolled patients who are surviving is ensured.

14.3.2. Major non-compliance

"Major non-compliance" is that affect the human rights and safety of subjects of clinical research, the study progress and the reliability of study results. Examples of "major non-compliance" in JCOG study are provided in. If major non-compliance is likely, Principal Investigator/Study Coordinator will report the situation to Certified Review Board immediately.

1) Major non-compliance with respect to eligibility

Violation of registration

- Enrolled intentionally (falsely) while knowing that eligibility criteria was not met
- Patient enrollment was performed without necessary informed consent, and protocol treatment was performed
- The source documents for the determination of eligibility cannot be identified (including the loss of the consent form).

2) Protocol violation

Violations that affect the increased risk of enrolled patients or that affect the reliability of the study results

- Significant violation in inclusion criteria or exclusion criteria
- Violation threatening patient safety in off-treatment criteria
- Serious violation of prohibited concomitant drug, prohibited concomitant treatment, etc.
- e.g. intentional or systematic non-compliance with protocol regulations

3) Other major non-compliance

- Study was conducted prior to Certified Review Board approval or prior to approval of site administrator
- The study was continued without providing information to the enrolled patient that could affect the willingness to continue to participate the study.
- Those judged to be research misconduct (fabrication or falsification of data, etc.)

- Any leakage of personal information or violation of human rights that may have a significant impact on the enrolled patient.

15. Special Instructions

15.1 Central pathology diagnosis and related matters

15.1.1 Central pathological diagnosis

In this study, the pathological tissues of enrolled patients will be collected and the central pathological diagnosis will be determined after the fact. Since the central pathological diagnosis is not performed in real time for each individual patient enrollment, the main analysis target and the decision on treatment strategy will be based solely on the pathological diagnosis at the institution. The details of the central pathological diagnosis procedure will be specified separately in the Central Pathological Diagnosis Procedure Manual.

Timing: Once a year throughout the study period.

Subjects: All registered patients

Methods: Pathological specimens (or duplicates made from the same paraffin block) used for eligibility criteria determination at the registered facilities will be collected, and after necessary staining (chromogranin A, synaptophysin) is added, pathological eligibility will be re-determined by two or more pathology judges (16.8 Pathology judges) appointed by the group representative. (16.8 Central Pathological Review Comittie).

Management of the collected specimens: The Hepatobiliary and Pancreatic Group Study Office will be responsible for the management of the collected specimens.

Staining: If additional staining is required, it will be performed by the Central Pathology Coordinator (16.7).

Notification of the central judgment to each facility:

After the results of the central pathological diagnosis are fixed, the research secretariat will notify the results of the judgment to the registered facility (facility coordinator) of each patient. At that time, the rationale for the decision should be attached in writing. If there is a difference between the institutional diagnosis and the central diagnosis, the facility principal investigator/facility coordinator reports the decision results to the facility pathologist and discusses it with the facility pathologist, and carefully decides on the final pathological diagnosis at the facility (whether to change the diagnosis or not) and what to do if the patient is under treatment (whether to change the treatment or not).

15.1.2 Providing information to the institutional pathologist

In view of the high level of difficulty in NEC pathological diagnosis and the unique nature of this study, which covers multiple target organs, the following information will be provided to institutional pathologists.

(1) Pre-registration pathological diagnosis consultation

Since it is anticipated that there will be cases of confusion in diagnosis at facilities, pre-enrollment pathology consultation will be available as needed, with the pathology judgment committee members of this study serving as consultants. Details of the consultation operation are described in the Central Pathology Procedures.

(2) Holding of pathology-related meetings

In this study, the research secretariat and the pathology secretariat first held a pathology-related meeting (attended by pathologists from participating institutions) on February 11, 2014, during which a lecture was given to form a consensus on diagnostic criteria according to the WHO 2010 classification. The slides used at the meeting were revised as necessary based on the discussion at the meeting and posted on the JCOG website for reference by pathologists at each facility.

15.2. JCOG BioBank Japan (BBJ) Biorepository

This study will participate in the banking of blood samples (DNA/plasma) in JCOG BBJ Biorepository based on a common protocol for all JCOG studies (hereafter referred to as common banking).

Subjects:

Among patients who agreed to participate in this study, patients whose consent to shared banking was obtained.

Sample:

1) Whole blood

Blood sampling is performed before the start of the protocol treatment in this study in principle. However, blood sampling after initiation of protocol treatment is allowed. Blood samples of 7 mL×2 (total 14 mL) of

venous blood are collected using a blood collection tube (for blood counting) with a EDTA Na dedicated to the shared banking of JCOG-BBJ Biorepository, and stored at 4°C at the respective institutions until they are handed over to the sample transport/processing company (See "JCOG-Biobank Japan Biorepository protocol" for details).

2) Pathological specimens

Archived pathological tissues in daily clinical practice such as surgery, biopsy and laboratory tests can also be used in future translational researches, but the type of pathology specimen, preparation method and tissue quantity required by the studies are varied and there is no consensus that it is efficient to bank pathological tissues in a certain way prospectively. In addition, there is the opinion that the sample deteriorates (DNA fragmentation) when the thin-section sample from pathological tissue is stored for a long time.

Consent on the use of archival pathological tissue after medical care should therefore be obtained at the time of consent to banking, but actual collection should be initiated on an individual basis by creating a protocol and defining the most appropriate procedure for the study content in the protocol.

16. Organization

Changes to this chapter are considered to be revision rather than amendment.

16.1. Main study fund (funding source) of this study.

Practical Research for Innovative Cancer Control from Japan Agency for Medical Research and Development “Establishment of standard treatments for neuroendocrine carcinoma of the digestive system” JP15ck0106138, JP16ck0106138, JP17ck0106355, JP18ck0106355, JP19ck0106355, JP20ck0106618

National Cancer Center Research and Development Fund (23-A-22, 26-A-4, 29-A-3, 2020-J-3)

“Scientific research on multi-institutional trials to establish new standard treatment of solid tumors in adults”

16.2. Japan Clinical Oncology Group (JCOG)

JCOG is a multi-institutional clinical research group consisting of research teams funded by public research grants mainly on National Cancer Center Research and Development Fund and Japan Agency for Medical Research and Development research costs that receive direct support for research by the Clinical Research Support Office of the National Cancer Center Hospital in accordance with JCOG Policy (http://www.jcog.jp/).

This study is conducted using JCOG research organisations and in accordance with the regulations set out by JCOG Executive Committee.

16.3. JCOG Chair

Yuichiro Ohe National Cancer Center Hospital

16.4. Study group and Group Chair

JCOG Hepatobiliary and Pancreatic Oncology Group

Group Chair: Junji FURUSE

Kyorin University Faculty of Medicine

Group Secretary: Hirohi Ishii

Clinical Research Center, Chiba Cancer Center

JCOG Stomach Cancer Study Group:

Group Chair: Mitsuru SASAKO

Hyogo Medical University

Group Secretary: Narikazu Boku

St. Marianna University School of Medicine

JCOG Japan Esophageal Oncology Group
Group Chair: Yuko Kitagawa
Keio University Hospital

Group Secretary: Ken Kato
National Cancer Center Hospital

16.5. Study Chair (Principal Investigator)

JCOG Hepatobiliary and Pancreatic Oncology Group
Takuji Okusaka
National Cancer Center Hospital

JCOG Stomach Cancer Study Group:
Narikazu Boku
St. Marianna University School of Medicine
JCOG Japan Esophageal Oncology Group
Ken Kato
National Cancer Center Hospital

16.6. Study Coordinator

JCOG Hepatobiliary and Pancreatic Oncology Group
Chigusa Morizane
National Cancer Center Hospital

JCOG Stomach Cancer Study Group:
Nozomu Machida
Shizuoka Cancer Center

JCOG Japan Esophageal Oncology Group
Ken Kato
National Cancer Center Hospital

Yoshitaka Honma
National Cancer Center Hospital

16.7. Central Pathological Review Coordinator

Nobuyoshi Hiraoka
National Cancer Center Hospital

Hirokazu Taniguchi
National Cancer Center

16.8. Central Pathological Review Coordinator

Nobuyoshi Hiraoka (National Cancer Center Hospital)
Noriyoshi Fukushima (Jichi Medical University)
Nobuyuki Ohike (Showa University Fujigaoka Hospital)

Ryoji Kushima (Shiga University of Medical Science)
Mitsuya Iwafuchi (Scool of Health Sciences Faculty of Medicine, Niigata University)
Tetsuo Ushiku (The University of Tokyo)

16.9. Participating sites (participating institutions)

HBP	SCSG	JEOG	Participating institutions
○			Sapporo-Kosei General Hospital
○		○	Hokkaido University Hospital
	○		Keiyukai Sapporo Hospital
	○	○	Iwate Medical University
		○	Tohoku University Hospital
	○		Miyagi Cancer Center
○	○	○	Tochigi Cancer Center
○			Jichi Medical University
○	○	○	Saitama Cancer Center
○	○	○	National Cancer Center Hospital East
○		○	Chiba Cancer Center
○		○	Chiba University, Graduate School of Medicine
○	○	○	National Cancer Center Hospital
○			Kyorin University Faculty of Medicine
○			National Center for Global Health and Medicine (NCGM)
○	○	○	Cancer Institute Hospital of Japanese Foundation for Cancer Research
	○	○	Toranomon Hospital
○			Teikyo University School of Medicine
○			St.Marianna University School of Medicine
○	○	○	Kanagawa Cancer Center
○	○		Yokohama City University Medical Center
○	○		Niigata Cancer Center Hospital
○			Toyama University Hospital
○			Kanazawa University School of Medicine
	○		Ishikawa Prefectural Central Hospital
	○		Ogaki Municipal Hospital
○	○	○	Shizuoka Cancer Center
○	○	○	Aichi Cancer Center Hospital
		○	Kyoto University Hospital
	○	○	Osaka University Graduate School of Medicine
○	○		Kindai University Hospital
○	○	○	Osaka International Cancer Institute
○	○	○	National Hospital Organization Osaka National Hospital
	○	○	Osaka General Medical Center
	○	○	Osaka Medical and Pharmaceutical University
	○		Osaka Rosai Hospital
○	○	○	Kobe University Graduate School of Medicine
	○	○	Kansai Rosai Hospital
○	○		Hyogo College of Medicine
○	○	○	Hyogo Cancer Center
	○		Itami City Hospital
	○		Shimane University Faculty of Medicine
	○	○	Hiroshima University Hospital
	○		Fukuyama City Hospital
	○		Tokushima Red Cross Hospital
○	○	○	National Hospital Organization Shikoku Cancer Center
○		○	Kochi Health Sciences Center
○		○	National Kyushu Cancer Center
○		○	Kyushu University Hospital

	○	○	Oita University Faculty of Medicine

HBP: Hepatobiliary and Pancreatic Oncology Group
SCSG: Stomach Cancer Study Group:
JEOG: Japan Esophageal Oncology Group

16.10. JCOG Protocol Review Committee

This protocol was approved by JCOG Protocol Review Committee prior to submit to Certified Review Board.

(For membership, see website http://www.jcog.jp/basic/org/committee/protocol.html)

Contact: Protocol Review Committee Office

JCOG Operations Office/Clinical Research Support Office, National Cancer Center Hospital
〒104-0045 5-1-1, Tsukiji, Chuo-ku, Tokyo
TEL: 03-3542-2511 (ext. 2302)
FAX:03-3542-7006
E-mail: jcogoffice@ml.jcog.jp

16.11. JCOG Data and Safety Monitoring Committee

During study period, the study will be monitored by Data and Safety Monitoring Committee (e.g., adverse event reports, interim analysis reviews, monitoring report reviews, protocol revision reviews). However, the committee members from the study group conducting this study do not participate directly in the review of this study.

(For membership, see website http://www.jcog.jp/basic/org/committee/jury.html)

Contact: JCOG Data and Safety Monitoring Committee Office

JCOG Operations Office/Clinical Research Support Office, National Cancer Center Hospital
〒104-0045 5-1-1, Tsukiji, Chuo-ku, Tokyo
TEL:03-3542-2511 (ext. 2403)
FAX:03-3542-7006
E-mail: jcogoffice@ml.jcog.jp

16.12. JCOG Audit Committee

Site-visit audits by Audit Committee will be conducted during study period.

(For membership, see website http://www.jcog.jp/basic/org/committee/audit.html)

Contact: JCOG Auditing Committee Office

JCOG Operations Office/Clinical Research Support Office, National Cancer Center Hospital
〒104-0045 5-1-1, Tsukiji, Chuo-ku, Tokyo
TEL:03-3542-2511 (ext. 2403)
FAX:03-3542-7006
E-mail: jcogoffice@ml.jcog.jp

16.13. JCOG Conflict of Interest Committee

During study period, JCOG investigators involved in this study will be managed by the Conflict of Interest Committee.

(For membership, see website http://www.jcog.jp/basic/org/committee/coi.html)

Contact: JCOG Conflict of Interest Committee Office

JCOG Operations Office/Clinical Research Support Office, National Cancer Center Hospital
〒104-0045 5-1-1, Tsukiji, Chuo-ku, Tokyo
TEL:03-3542-2511 (ext. 2404)
FAX:03-3547-1002
E-mail: jcogoffice@ml.jcog.jp

16.14. Data Center/Operations Office

JCOG Data Center

Director of Data Center: Haruhiko Fukuda
Clinical Research Support Office, National Cancer Center Hospital

〒104-0045 5-1-1, Tsukiji, Chuo-ku, Tokyo
TEL:03-3542-3373
FAX:03-3542-3374
E-mail: jcogdata@ml.jcog.jp

JCOG Operations Office
Director of Operations Office: Kenichi Nakamura
Clinical Research Support Office, National Cancer Center Hospital
〒104-0045 5-1-1, Tsukiji, Chuo-ku, Tokyo
TEL:03-3547-1002
FAX:03-3547-1002
E-mail: jcogoffice@ml.jcog.jp

Official website: http://www.jcog.jp/

16.14.1. Data management administrator

Data management organization		JCOG Data Center
Data management administrator	Name	Harumi Kaba
	e-Rad number	40543442
	Affiliation	Clinical Research Support Office, National Cancer Center Hospital
	Title	Head of Multi-institutional Data Management Section

16.14.2. Monitoring administrator

Monitoring organization		JCOG Data Center
Monitoring administrator	Name	Haruhiko Fukuda
	e-Rad number	70263390
	Affiliation	Clinical Research Support Office, National Cancer Center Hospital
	Title	Chief of Data Management Division

16.14.3. Site-visit audit administrator

Auditing organization		JCOG Operations Office
Site-visit audit administrator	Name	Kenichi Nakamura
	e-Rad number	40543533
	Affiliation	Clinical Research Support Office, National Cancer Center Hospital
	Title	Director of Research Management Division

16.14.4. Statistical analysis administrator

Statistical analysis organization		JCOG Data Center
Statistical analysis Administrator	Name	Junki Mizusawa
	e-Rad number	60706646
	Affiliation	Biostatistics Division, Center for Research Administration and Support, National Cancer Center/<br>Biostatistics Section, Research Management Division, Clinical Research Support Office, National Cancer Center Hospital
	Title	Biostatistics Section Head

16.14.5. Research and development plan support personnel

Organizations in charge of supporting research and development plans		JCOG Operations Office
Research and development plan support personnel	Name	Tomoko Kataoka
	e-Rad number	70569863
	Affiliation	Clinical Research Support Office, National Cancer Center Hospital
	Title	Medical officer

16.14.6. Coordination management practitioner

Organizations in charge of coordination and management		JCOG Operations Office
Coordinating and managing practitioners	Name	Junko Eba
	e-Rad number	80754085
	Affiliation	Clinical Research Support Office, National Cancer Center Hospital
	Title	Medical officer

16.14.7. Personnel who oversees the study other than Principal Investigator and site investigators

Personnel who oversees the study other than Principal Investigator and site investigators	Name	Not applicable	
	e-Rad number		
	Affiliation		
	Title		
	Relevance of Secondary Sponsor	□ Applicable	□ Not applicable

16.14.8. Study group personnel

JCOG Data Center
Statistics Section Gakuto OGAWA
Data Management Section Kyoko HASEGAWA
JCOG Operations Office
Science Section Tomoko KATAOKA/Tadayoshi HASHIMOTO

In addition, JCOG Data Center/Operations Office commissioned some of theire research support activities (such as support for the preparation of study protocols, data management, and site-visit audits) to other corporations. The commissioned duties are supervised by the National Cancer Center through routine work, as well as by receiving work reports from the institution and providing supervisory guidance. The current consignee is as follows

Clinical Oncology Research and Education, a specified non-profit organization
〒104-0061 DJ Ginza Building 7F, 8-18-3, Ginza, Chuo-ku, Tokyo
Official website http://www.core.or.jp/

16.15. Developing a study protocol

Chigusa MORIZANE, National Cancer Center Hospital
Nozomu MACHIDA, Shizuoka Cancer Center
Yoshitaka HONMA/Ken KATO, National Cancer Center Hospital

Support for protocol development
JCOG Data Center
Statistics Section (in charge of study design) Junki MIZUSAWA
Data Management Section (CRF preparation) Harumi KABA
JCOG Operations Office
Protocol development Hiroshi KATAYAMA/Kozo KATAOKA/Aya MIURA
Person in charge of IC documents Aya KIMURA/Noriko TSUJI

17. Publication of the study results and completion of the study

17.1. Paper and conference presentations

Primary publication will be published in English journals.

Paper publication including review article and conference presentation of Introduction of the study, by Principal Investigator or Study Coordinator, which does not include the analytical results of the endpoint of the study, are allowed when Group Chair and JCOG Data Center Director agree to them. Publication of the distribution of baseline factors or the safety data after the end of accrual are also allowed. No publication other than primary analysis and final analysis will be performed unless previously approved by Data and Safety Monitoring Committee.

In principle, the authors of the main published papers on the results of the study (the first publication of the results of primary endpoint) shall be the first Study Coordinator (HBPOG), followed by Study Coordinator (JEOG or SCSG), Study Coordinator (JEOG or SCSG), Principal Investigator (HBPOG), Principal Investigator (JEOG or SCSG), Principal Investigator (JEOG or SCSG), the statistical staff of Data Center (one statistician in charge at the time of the analysis for publication), Centralized Pathological diagnosis Coordinator (HBP), Centralized Pathological diagnosis Coordinator (GI). In accordance with the limitations imposed by the article's posting provisions, researchers who contributed in descending order of their number of patients registrations were selected for each institution as co-authors, and the last author was Group Chair (or Study Chair). The inclusion of staffs of JCOG Operations Office as co-authors will be determined by Group Chair depending on their contributions. Authors of articles other than the primary publication (e.g., Secondary endpoints articles, secondary analysis articles) will be determined by Study Chair with Group Chair approval.

All co-authors will review the article contents prior to submission for publication and only those who agree to the article contents. If there is no consensus on the contents, principle investigator may not include the investigator in the co-authorship with the approval of Group Chair. If there is no consensus between groups and JCOG Data Center/Operations Office, ultimately follow JCOG Chair instructions.

17.2. Primary Endpoint Report and Clinical Summary Report

The procedures are specified from the preparation of the primary endpoint report and clinical summary report. If primary analysis is the final analysis, the primary endpoint report will not be prepared and the clinical summary report will be prepared.

17.2.1. Clinical Summary Report

Based on the final analysis report, Principal Investigator will prepare a "Abstract of the Clinical Summary Report" containing subjects background information (age, sex, etc.), study design and study progress, results of analyses for each endpoint, conclusions of the entire study, interpretations and discussion of the results, etc. within 6 months from the issue date of the final analysis report, submit it to the Data Center, and undergo review. In addition, the issue date of the final analysis report shall be "the date when the period for collecting data on all endpoints is completed" as specified in the Enforcement Regulations.

After obtaining approval from Group Chair and the Director of the Data Center, abstract of the clinical summary report will be submitted to Certified Review Board review as a "clinical summary report" with the final analysis report within 1 year of the issue date of the final analysis report.

Principal Investigator/Study Coordinator will disclose abstract of the clinical summary report (Notification of Completion of Form 1 of Article 24 of Regulation) to jRCT within 1 month after obtaining Certified Review Board approval (if the report is unpublished, abstract will not be released in jRCT and will be published immediately after publication).

The approved abstract of the clinical summary report will be submitted to the administrators of each participating institution through the site investigators at each institution and to JCOG Chair. Abstract of the clinical summary report is available on JCOG website (http://www.JCOG.jp/).

The timing of distribution of the final analysis report to participating institutions will be determined by Principal Investigator/Study Coordinator considering the timing of publication of the final analysis results, and the final analysis report will be distributed to the researchers at participating institutions by themselves or through the Data Center.

17.3. Completion of the study

On the date that abstract of the clinical summary report was released to iRCT, the study is completed.

In institutions where patients were not enrolled, the institution may be withdrawn from the institution list by submitting a request for change (Unified Form 3 for Clinical Research) and a Notification of Changes in Protocol (Form 2) stating that Principal Investigator withdraws the institution from the institution list in question to Certified Review Board, and after obtaining approval, notifying the MHLW (Local Health Bureau responsible for the location of Certified Review Board). When a protocol change notification is received by the Local Health Service, the investigator at the institution shall report the withdrawal to the Administrator of the institution.

18. References

omit

19. Appendix

- Informed consent form
- Body surface area table
- Toxicity Criteria (CTCAE v5.0-JCOG)
- CRF samples* (CRF draft attached for the first review submission)
- JCOG-Biobank Japan Biorepository Protocol
- JCOG-Biobank Japan Biorepository Informed Consent Form

Japan Clinical Oncology Group
Hepatobiliary and Pancreatic Oncology Group/Stomach Cancer Study Group/Japan Esophageal Oncology Group

Practical Research for Innovative Cancer Control from Japan Agency for Medical Research and Development
“Establishment of standard treatments for neuroendocrine carcinoma of the digestive system”
National Cancer Center Research and Development Fund 29-A-3

JCOG1213

Randomized phase III study of etoposide plus cisplatin combination therapy versus irinotecan plus cisplatin combination therapy in advanced neuroendocrine carcinoma of the digestive system. ver.1.1
TOPIC-NEC

HBPOG
Group Chair: Junji FURUSE
Department of Medical Oncology, Kyorin University Faculty of Medicine

Study Chair: Takuji OKUSAKA
Department of Hepatobiliary and Pancreatic Oncology, National Cancer Center Hospital 5-1-1 Tsukiji, Chuo-ku, Tokyo 104-0045 Japan
Tel: +81-3-3542-2511
E -mail : tokusaka@ncc.go.jp

Study Coordinator: Chigusa MORIZANE
Department of Hepatobiliary and Pancreatic Oncology, National Cancer Center Hospital 5-1-1 Tsukiji, Chuo-ku, Tokyo 104-0045 Japan
Tel: +81-3-3542-2511
E -mail : cmorizan@ncc.go.jp

SCSG
Group Chair: Masanori TERASHIMA
Division of Gastric Surgery, Shizuoka Cancer Center Hospital

Study Chair: Narikazu BOKU
Department of Gastrointestinal Oncology, National Cancer Center Hospital
5-1-1 Tsukiji, Chuo-ku, Tokyo 104-0045 Japan
Tel: +81-3-3542-2511
E -mail : nboku@ncc.go.jp

Study Coordinator: Nozomu MACHIDA
Department of Hepatobiliary and Pancreatic Oncology, Shizuoka Cancer Center
1007 Shimonagakubo, Nagaizumi-cho, Sunto-gun, Shizuoka Prefecture 411-8777 Japan
Tel: +81-55-989-5222
E -mail : no.machida@scchr.jp

JEOG
Group Chair: Yuko KITAGAWA
General and Gastroenterological Surgery, Keio University Hospital

Study Chair: Ken KATO
Department of Gastrointestinal Oncology, National Cancer Center Hospital 5-1-1 Tsukiji, Chuo-ku, Tokyo 104-0045 Japan
Tel: +81-3-3542-2511
E -mail : kenkato@ncc.go.jp

Study Coordinator: Yoshitaka HONMA/Ken KATO
Department of Gastrointestinal Oncology, National Cancer Center Hospital 5-1-1 Tsukiji, Chuo-ku, Tokyo 104-0045 Japan
Tel: +81-3-3542-2511
E-mail:yohonma@ncc.go.jp
/kenkato@ncc.go.jp

2013/3/16 Protocol concept approved by JCOG Executive Committee (PC1213/1214/1215)
2014/6/27 Protocol approved byJCOG Protocol Review Committee
2017/5/23 Revision ver. 1.1 approved by JCOG Data and Safety Monitring Committee

0. Summary

This study is conducted as a "specified clinical trial" based on the Clinical Trials Act (Act No. 16 of April 14, 2017). In this protocol, the Principal Investigator is the Study Chair in the Hepatobiliary and Pancreatic Oncology Group of JCOG.

Name of study: "Randomized phase III study of etoposide plus cisplatin combination therapy versus irinotecan plus cisplatin combination therapy in advanced neuroendocrine carcinoma of the digestive system. (TOPIC-NEC) ".

Public study title: "Randomized phase III study of etoposide plus cisplatin combination therapy versus irinotecan plus cisplatin combination therapy in advanced neuroendocrine carcinoma of the digestive system. (TOPIC-NEC) "

0.1. Schema

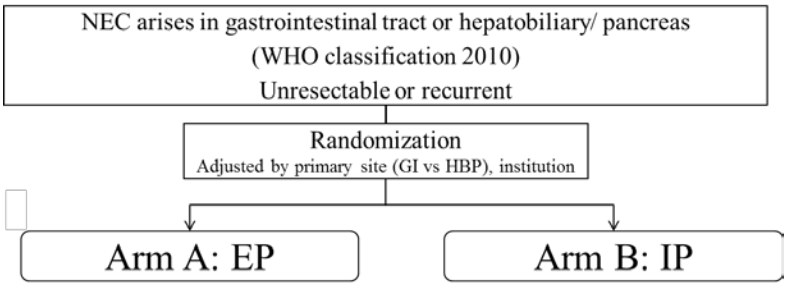

0.2. Objectives

A randomized phase III study was conducted to determine the better treatment option between etoposide/cisplatin combined therapy (EP therapy) or irinotecan/cisplatin combined therapy (IP therapy), both of which are standard treatments for non-resectable/recurrent neuroendocrine carcinoma (NEC as classified by WHO in 2010) with primary lesions in the gastrointestinal tract/hepatobiliary pancreatic organs.

Primary endpoint: Overall survival

Secondary endpoints: Response rate in case of measurable lesions, Progression-free survival (PFS), incidence rate of adverse events, dose intensity of Cisplatin, and incidence rate of serious adverse events.

0.3. Subjects

1) Any of the following is applicable based on pathological diagnosis taking findings of immunohistochemistry into consideration (see 3.1. to 3.3.).

[1] Pathologically diagnosed as neuroendocrine carcinoma (NEC*1) in the resected sample.

[2] Containing pathologically confirmed component of neuroendocrine carcinoma (NEC*1) in the biopsy sample.

1: Based on WHO 2010 classification

2) Any of the following is applicable

[1] NEC arise in esophagus, stomach, duodenum, intestine, appendix, colon, rectum, gallbladder, intrahepatic bile duct, extrahepatic bile duct, ampulla of Vater, pancreas,

[2] Liver NEC (primary liver or liver metastasis of unknown primary) *2.

*2: The tumor is only in one of the following sites after a thorough examination of the primary site by contrast CT (from the neck to pelvic) and upper/lower gastrointestinal endoscopy, FDG-PET scan, otolaryngology (head and neck) examination, urology examination (male patients only), and gynecology examination (female patients only).

a. Liver only

b. Bone, lymph nodes below the diaphragm, peritoneum, subcutaneous, muscle, spleen, and liver

3) Unresectable (see 3.6) or recurrent cancer (see 3.7). It is not essential for a pathological confirmation of the metastatic lesion or recurrent site. Cases of esophageal NEC is ineligible if corresponding to any of the following.

[1] cT4.

[2] No distant metastasis rather than supraclavicular lymph node

[3] Stenosis indicated for palliative radiotherapy

4) No previous chemotherapy or radiotherapy for NEC. Pre- or post-operative chemotherapy except irinotecan or

etoposide for NEC is allowed as long as it was completed at least 8 weeks prior to registration.

5) No previous chemotherapy using platinum agents for any malignancies.

6) Aged 20 to 75 years old.

7) ECOG performance status of 0 or 1.

8) Measurable region is not required.

9) Adequate organ functions.

[1] WBC ≥3,000/ mm^3

[2] Neutrophils ≥ 1,500/ mm^3

[3] Hemoglobin ≥ 9.0 g/dL

[4] Platelets ≥ 10×10^4/ mm^3

[5] Total bilirubin≤ 1.5 mg/dL※4

[6] AST(sGOT)≦100 IU/L※4（for hepatic NEC and liver metastasis, ≦150 IU/L）

[7] ALT(sGPT)≦100 IU/L※4（for hepatic NEC and liver metastasis, ≦150 IU/L）

※4：Presence or absence of biliary drainage is not relevant

[8] Serum creatinine≦ 1.3 mg/dL

[9] Creatinine clearance※5≥ 60 mL/min

※5： Creatinine clearance must have been estimated using the Cockcroft-Gault formula, and must be 60 mL/min or more. If the estimation is less than 60 mL/min, but the actual measurement is 60 mL/min or more, the patient can be deemed eligible.

Cockcroft-Gault formula

Male：Ccr ={(140－age) × body weight (kg)}/{72×serum creatinine (mg/dL)}

Female：Ccr = 0.85×{(140－age) × body weight (kg)}/{72×serum creatinine(mg/dL)}

11) Written informed consent.

0.4. Treatments

Arm A: etoposide plus cisplatin (EP) arm

The following chemotherapy is continued until the patient meets discontinuation criteria, with 3-weeks of treatments counting as one cycle.

Drug	Dosage (mg/m^2)	Dosing regimen/Dosing time	Dose day
Etoposide	100	IV/60-120 min	Day 1, 2, 3
Cisplatin	80	IV/60-120 min	Day 1

Arm B: Irinotecan plus Cisplatin (IP) arm

The following chemotherapy would be continued until the patient meets discontinuation criteria, with 4-weeks of treatments counting as one cycle.

Drug	Dose (mg/m^2)	Dosing regimen/Dosing time	Dose day
Irinotecan	60	IV/90 min	Day 1, 8, 15
Cisplatin	60	IV/60-120 min	Day 1

0.5. Planned sample size and study period

The planned sample size is 170.

Accrual period: 6 years.

Follow-up period: 1 years after accrual completion.

Analysis period: 1 year.

Total study duration 8 years.

Scheduled start date of the study Aug 8, 2014

Expected completion date of the study Aug 8, 2028

0.6. Contact information

Eligibility criteria, treatment modification, and other issues requiring clinical decisions: Study Coordinator (front cover and 16.6.)

Enrollment procedure protocol, case report form (CRF) entries, etc.: JCOG Data Center, (16.14.)

Adverse event reporting: JCOG Data and Safety Monitoring Committee (16.11.),

Contents

0. SUMMARY 2

0.1. SCHEMA 2
0.2. OBJECTIVES 2
0.3. SUBJECTS 2
0.4. TREATMENTS 3
0.5. PLANNED SAMPLE SIZE AND STUDY PERIOD 3
0.6. CONTACT INFORMATION 3

1. OBJECTIVES 8

2. BACKGROUND 8

2.1. TARGET 8
2.2. STANDARD TREATMENT FOR TARGET DISEASE 16
2.3. RATIONALE FOR ESTABLISHMENT OF TREATMENT PLAN 18
2.4. STUDY DESIGN 21
2.5. SUMMARY OF EXPECTED ADVANTAGES AND DISADVANTAGES ASSOCIATED WITH STUDY PARTICIPATION 22
2.6. SIGNIFICANCE OF THIS STUDY 23
2.7. ASSOCIATED RESEARCH (INCLUDING SAMPLE ANALYSIS RESEARCH) 23
2.8. JCOG-BIOBANK JAPAN (BBJ) COLLABORATING BIOBANK 23

3. CRITERIA/DEFINITIONS USED IN THIS STUDY 25

3.1. TISSUE CLASSIFICATION (WHO 2010 CLASSIFICATION) 25
3.2. GRADE CLASSIFICATION（ENETS [EUROPEAN NEUROENDOCRINE TUMOR SOCIETY] / WHO2010 CLASSIFICATION） 25
3.3. HISTOPATHOLOGICAL DIAGNOSIS 25
3.4. DISEASE STAGE CLASSIFICATION CRITERIA (UICC-TNM 7TH EDITION) 25
3.5. RESIDUAL TUMOR (R) CLASSIFICATION (UICC-TNM 7TH EDITION) 33
3.6. DEFINITION OF NON-RESECTABLE NEC 33
3.7. DEFINITION OF RECURRENT NEC 34
3.8. DEFINITION OF HEPATIC NEC (HEPATIC PRIMARY LESION OR LIVER METASTASIS FROM AN UNKNOWN PRIMARY LESION) 34

4. PATIENT SELECTION CRITERIA 34

4.1. INCLUSION CRITERIA (FOR ENROLLMENT) 34
4.2. EXCLUSION CRITERIA 35

5. REGISTRATION AND RANDOMIZATION 36

5.1. PROCEDURE OF REGISTRATION 36
5.2. RANDOMIZATION AND ALLOCATION ADJUSTMENT FACTOR 37

6. TREATMENT PLAN AND TREATMENT MODIFICATION CRITERIA 38

6.1. PROTOCOL TREATMENT 38
6.2. PROTOCOL TREATMENT TERMINATION/COMPLETION CRITERIA 41
6.3. TREATMENT MODIFICATION CRITERIA 42
6.4. CONCOMITANT TREATMENT AND SUPPORTIVE CARE 48
6.5. POST-STUDY TREATMENT 55

7. ANTICIPATED ADVERSE EVENTS 56

7.1. ANTICIPATED ADVERSE REACTIONS 56

7.2. ANTICIPATED ADVERSE EVENTS DUE TO PATHOGENESIS 59
7.3. EVALUATION OF ADVERSE EVENTS/REACTIONS 60

8. EXAMINATION AND EVALUATION 62

8.1. BASELINE EXAMINATION AND EVALUATION BEFORE REGISTRATION 62
8.2. EXAMINATION AND EVALUATION DURING TREATMENT 63
8.3. EXAMINATION AND EVALUATION AFTER COMPLETION OF TREATMENT 64
8.4. INFORMATION ON POST- STUDY TREATMENT 64
8.5. STUDY CALENDAR 65

9. DATA COLLECTION 69

9.1. CASE REPORT FORM (CRF) 69

10. REPORTING OF "DISEASE OR THE LIKE"(ADVERSE EVENTS) 70

10.1. SERIOUS ADVERSE EVENTS AND SUBJECTS OF EXPEDITED REPORTING. 70
10.2. INVESTIGATOR'S REPORTING REQUIREMENTS AND PROCEDURES 71
10.3. RESPONSIBILITIES OF PRINCIPAL INVESTIGATOR/STUDY COORDINATOR 73
10.4. RESPONSIBILITIES OF THE SITE INVESTIGATORS AT THE PARTICIPATING INSTITUTIONS (INCLUDING THE RELEVANT INSTITUTION) 75
10.5. RESPONSIBILITIES OF THE DATA AND SAFETY MONITORING COMMITTEE 75

11. RESPONSE EVALUATION AND ENDPOINT DEFINITION 76

11.1. RESPONSE ASSESSMENT (ONLY FOR PATIENTS WITH MEASURABLE DISEASE) 76
11.2. DEFINITIONS OF ANALYSES SET 82
11.3. DEFINITION OF ENDPOINTS 82

12. STATISTICAL CONSIDERATION 85

12.1. PRINCIPAL ANALYSIS AND DECISION CRITERIA 85
12.2. PLANNED ACCRUAL, ACCRUAL PERIOD, AND FOLLOW-UP PERIODS 86
12.3. INTERIM ANALYSIS AND EARLY TERMINATION OF THE STUDY 86
12.4. ANALYSIS OF SECONDARY ENDPOINTS 88
12.5. FINAL ANALYSIS 88
12.6. EXPLORATORY ANALYSIS 89
12.7. PREMATURE WITHDRAWAL FROM THE TRIAL 89
12.8. PROCEDURES AFTER EARLY TERMINATION OF THE STUDY 90

13. ETHICAL CONSIDERATIONS 92

13.1. PROTECTION OF HUMAN SUBJECTS 92
13.2. INFORMED CONSENT 92
13.3. PROTECTION OF PERSONAL INFORMATION AND PATIENT IDENTIFICATION 94
13.4. ADHERENCE TO THE PROTOCOL 96
13.5. APPLICATION TO CERTIFIED REVIEW BOARD AND NOTIFICATION OF IMPLEMENTATION PLANS 96
13.6. PROTOCOL REVISION/AMENDMENT 100
13.7. CONFLICTS OF INTEREST (COIS) INVOLVED IN THIS STUDY 102
13.8. COMPENSATION 104
13.9. INTELLECTUAL PROPERTY 105
13.10. DISCLOSURE OF INFORMATION ON THIS STUDY 105

14. MONITORING AND AUDIT 106

14.1. PERIODIC MONITORING 106
14.2. SITE VISIT AUDITS 108

14.3. MANAGEMENT OF NON-COMPLIANCE 109

15. SPECIAL INSTRUCTIONS 110

15.2. JCOG BIOBANK JAPAN (BBJ) BIOREPOSITORY 111

16. ORGANIZATION 111

16.1. MAIN STUDY FUND (FUNDING SOURCE) OF THIS STUDY. 111
16.2. JAPAN CLINICAL ONCOLOGY GROUP (JCOG) 111
16.3. JCOG CHAIR 112
16.4. STUDY GROUP AND GROUP CHAIR 112
16.5. STUDY CHAIR (PRINCIPAL INVESTIGATOR) 113
16.6. STUDY COORDINATOR 113
16.7. CENTRAL PATHOLOGICAL REVIEW COORDINATOR 114
16.8. CENTRAL PATHOLOGICAL REVIEW COORDINATOR 114
16.9. PARTICIPATING SITES (PARTICIPATING INSTITUTIONS) 115
16.10. JCOG PROTOCOL REVIEW COMMITTEE 116
16.11. JCOG DATA AND SAFETY MONITORING COMMITTEE 116
16.12. JCOG AUDIT COMMITTEE 116
16.13. JCOG CONFLICT OF INTEREST COMMITTEE 116
16.14. DATA CENTER/OPERATIONS OFFICE 116
16.15. DEVELOPING A STUDY PROTOCOL 118

17. PUBLICATION OF THE STUDY RESULTS AND COMPLETION OF THE STUDY 119

17.1. PAPER AND CONFERENCE PRESENTATIONS 119
17.2. PRIMARY ENDPOINT REPORT AND CLINICAL SUMMARY REPORT 119
17.3. COMPLETION OF THE STUDY 120

18. REFERENCES 121

19. APPENDIX 121

1. Objectives

A randomized phase III study was conducted to determine the better treatment option between etoposide/cisplatin combined therapy (EP therapy) or irinotecan/cisplatin combined therapy (IP therapy), both of which are standard treatments for non-resectable/recurrent neuroendocrine carcinoma (NEC as classified by WHO in 2010) with primary lesions in the gastrointestinal tract/hepatobiliary pancreatic organs.

Primary endpoint： Overall survival

Secondary endpoints： Response rate in case of measurable lesions
Progression-free survival (PFS), incidence rate of adverse events, dose intensity of Cisplatin, and incidence rate of serious adverse events

2. Background

2.1. Target

2.1.1. Epidemiology

Neuroendocrine tumors (NETs) and neuroendocrine carcinomas (NECs) can arise in various organs in the body. Of these, NECs with primary lesions occurring in the lungs, namely small cell lung cancer and large cell lung cancer, have been collectively referred to as high-grade NECs (HGNECs). However, terms such as extrapulmonary small cell carcinoma and extrapulmonary NEC have been used to refer collectively to NECs, with primary lesions outside the lungs.

The number of newly reported cases of neuroendocrine neoplasms (NENs) annually, estimated based on the cases registered in the U.S. SEER（Surveillance, Epidemiology, and End Results）database between 1973 to 2004 is 5 in 100,000 people[1]. In Japan, the Neuroendocrine Tumor Workshop Japan （NET Work Japan）carried out a nationwide survey of pancreatic and gastrointestinal NENs, and estimated the number of new cases in 2005 at approximately 1.01 people with pancreatic primary lesion per 100,000 people, and 2.10 people with gastrointestinal primary lesion per 100,000 people[2, 3].

The report by the PRONET Study Group in France concerning their prospective observational study is a good reference for the proportion of NENs that can be classified as NECs. Out of the 778 patients diagnosed with gastrointestinal primary lesion NENs in 80 facilities from August 2010 to July 2011, 104 patients (13.4%) had NEC[4].

The frequency of incidence of NECs according to the primary lesion organ has been shown in the tables below (Table 2.1.1a and Table 2.1.1b). Despite variations between reports, gastrointestinal primary lesion NECs account for approximately 20–68% of all extrapulmonary NEC cases. The breakdown of gastrointestinal primary lesion NEC cases with primary lesion in the gastrointestinal tract (esophagus, duodenum, small and large intestines) indicated that these cases accounted for 56–84% of the total cases, while hepatobiliary pancreatic cancer cases accounted for 15–35% of all cases.

Table 2.1.1a. Frequency of extrapulmonary NECs according to the organ of primary lesion

Reported year/Reporter	N	Gastrointestinal	Gynecological organs	Urology	Head and Neck	Adrenal	Primary unknown	Other
2012/Terashima [5]	136	68%	12%	6%	NA	NA	8%	7%
2010/Brennan [6]	74	20%	35%	15%	19%	NA	9%	1%
2009/Wong [7]	1618	33%	NA	20%	11%	10%	4%	22%
2006/Haider [8]	101	20%	11%	18%	12%	9%	31%	NA
2007/Lee [9]	61	56%	20%	10%	8%	NA	6%	NA

Table 2.1.1b. Percentage breakdown of NECs as per the primary lesion organ of gastrointestinal NEC

Reported year/Reporter	N	Esophageal	Gastric	Duodenal/small intestine	Colorectal	Hepatobiliary	Pancreas	Other
2012/Machida [10]	258	33%	27%	2%	12%	12%	14%	NA
2012/Sorbye [11)]	205	6%	10%	NA	40%	NA	35%	10%
2004/Brenner [12]	544	53%	11%	0.2%	20%	11%	4%	NA

2.1.2. Clinical pathology

1) About the term of target disease

The target of this study has a complex disease concept, and since the method of classification has changed with time, various names have been assigned that could cause confusion. In this study, we use classification and nomenclature defined according to the WHO 2010 classification（WHO Classification of Tumors of the Digestive System 2010）[13].

All the tumors originating from neuroendocrine cells or differentiation into endocrine cells are called neuroendocrine neoplasms (NENs). Depending on the malignancy, NENs were classified as Grade 1 NETs (NET G1), Grade 2 NETs (NET G2), or NEC.

Furthermore, prior to the WHO 2010 classification, all disease concepts equivalent to NENs were called NETs, but in this study, we would have referred to all descriptions of the disease concept as NENs. Furthermore, according to the 2000 and 2004 WHO classifications, the term well-differentiated NEC (WDNEC) has been used for disease units equivalent to NET G2, but in this study NEC does not mean the same.

2) Classification and clinical pathology

NEN is a tumor that develops from neuroendocrine cells present in various tissues or shows a tendency of differentiation into neuroendocrine cells. Histologically these tumors express neuroendocrine markers, such as Chromogranin A, NSE (neuron-specific enolase), and synaptophysin. The WHO classifications in 2000 and 2004 combined the presence or absence of metastasis/local infiltration and cellular proliferative capacity (evaluated based on Ki-67 expression intensity and mitotic presentation), and thereby classified these tumors into well-differentiated (neuro)endocrine tumors without metastasis/local infiltration, well-differentiated (neuro)endocrine tumors with metastasis/local infiltration, and poorly differentiated (neuro)endocrine tumors with even greater cell proliferative capacity. On the other hand, European Neuroendocrine Tumor Society (ENETS) has proposed a method of classification that evaluates the malignancy as Grade 1, 2, or 3 (G1, G2, or G3), according to the cell proliferative capacity (Ki-67 index or number of mitotic presentations), which has proven to be most useful for classification of prognosis[14, 15]。

Against this backdrop, the WHO classification that targeted gastrointestinal diseases was published in 2010. The disease as a whole was considered “neuroendocrine neoplasms (NEN)”, and the disease malignancy was largely classified as NET G1 (neuroendocrine tumor Grade 1), NET G2 (Grade 2), NEC, and mixed adenoneuroendocrine carcinoma (MANEC). MANEC refers to the cancers wherein adenocarcinoma components account for more than 30% of the cancer, while those under 30% are classified as NECs. However, even though this method of classification is applicable while evaluating the entire tumor based on resected specimen, it is not possible to evaluate the proportion of each component in the entire tumor during a diagnosis based on biopsy sample.

Each of NET G1, NET G2, and NECs described in the WHO 2010 classification is equivalent to G1, G2, and G3 as per the ENETS classification. NETs are well-differentiated tumors, with relatively low atypicality and malignancy, and the clinical course is slow with a 5-year survival in the range of 62–85%[16, 17]. On the other hand, NECs are poorly differentiated tumors, where tumor cells with poor cellular constituents proliferate diffusely, and are pathologically similar to small cell lung cancers, since these show many mitotic presentations, involve necrotic lesions, and present with neuroendocrinological features during immunohistochemical staining[1]. The clinical presentations are also similar in terms of the rapid proliferation of NECs and relatively high sensitivity to anticancer drugs[18].

Similar to lung cancer, NECs also exist as small cell cancer type or large cell type (LCNEC：large cell NEC）[13]. Future challenges include studies on the frequency and differences in clinical presentation of each disease. Table 2.1.2 shows the WHO and Grade classification.

Table 2.1.2. Changes in WHO classification and grading; Shaded parts are targets of this study

WHO 2000 classification	WHO 2010 classification		ENETS Grade
Well-differentiated endocrine tumor（WDNET）<br>1.1‘Benign’ behavior<br>1.2 Uncertain behavior	Neuroendocrine tumors, NETs<br>• Well-differentiated<br>• Composed of cells similar to normal gastrointestinal	NET G1（carcinoid）	G1<br>Same as 2010 WHO classification<br>Number of mitotic presentations < 2 per 10

	endocrine cells<br>• Expression of neuroendocrine markers<br>• Hormone production<br>• Mild to moderate nuclear atypia, low proliferative capacity（Grade：G1,G2）		high power fields（HPF） and/or Ki-67 index ≤2%
Well-differentiated endocrine carcinoma（WDEC）		NET G2	G2<br>Number of mitotic presentations 2–20 per 10 HPF and/or Ki-67 index 3-20%
Poorly differentiated endocrine carcinoma/small cell carcinoma（PDEC）	Neuroendocrine carcinoma：NEC（large cell or small cell type）:<br>• Poorly differentiated, highly malignant<br>• Include small cell to large cell type carcinoma<br>• Sometimes present tissue structure similar to NET<br>• Expression of neuroendocrine markers<br>• Significant nuclear atypia, multifocal necrosis, and high proliferative capacity (Grade: G3)		G3<br>Number of mitotic presentations >20 per 10 HPF and/or Ki-67 index > 20%
Mixed exocrine-endocrine carcinoma（MEEC）	Mixed adenoendocrine carcinoma（MANEC）		
Tumor-like lesions（TLL）	Hyperplastic and preneoplastic lesions		

2.1.3. Staging

Disease staging for NENs is represented by the TNM classification proposed by ENETS (hereafter, ENETS TNM[14, 15], AJCC 7th Edition TNM classification and UICC 7th Edition TNM classification published in 2009. The AJCC 7th Edition and UICC 7th Edition TNM classifications are unified, and in this study we would have used the UICC 7th Edition nomenclature).

In the UICC 7th edition, there are independent TNM classifications of NET G1 and NET G2 with primary lesions in the stomach, small intestine, appendix, and colon, but with respect to NEC, it describes "classification according to main tissue type in each organ of primary lesion (squamous cell carcinoma for esophagus, and adenocarcinoma in all other organs of primary lesion)." While there are TNM classifications for hepatocellular carcinoma and intrahepatic cholangiocarcinoma in the liver, there is no description for the classification of the tissue type for NECs with primary lesion in the liver. In this study, however, we would have used the TNM classification of intrahepatic cholangiocarcinoma, which shows similar clinical presentations. Details of each TNM classification would have been mentioned in “3.2 staging criteria”.

2.1.4. Standard treatment according to disease staging and outline of prognosis

1) Standard treatment for resectable cases

Surgical resection is performed for resectable cases. The significance of postoperative adjuvant chemotherapy is not clear because a verification study has not yet been performed. Although the NCCN Guidelines[19] describe postoperative adjuvant therapy as the chemotherapy regimen for small cell lung cancers that is typically applied to advanced cases of NEC, it has relatively high toxicity and patient burden (see Table 2.2.2, 2.3.2); hence we cannot claim that it is widely used in routine practice. For NECs with gastrointestinal primary lesion with mixed adenocarcinoma, a chemotherapy regimen is suggested typically after surgery for adenocarcinoma; however, in practice, there is no consensus on postoperative adjuvant chemotherapy.

2) Standard treatment for locally advanced cases

While the NCCN Guidelines propose chemoradiotherapy for small cell lung cancer for locally advanced cases, the ENETS Guidelines provide no clear indication[20, 21] for the same. As there is no sufficient information related to radiation dose, efficacy, and safety for NECs with a multitude of primary lesion organs (scope of irradiation), radiation therapy is not widely used for pathologies other than NEC with esophageal primary lesion that is prone to stenosis, and treatment is often provided for distant metastasis.

For locally advanced cases of NEC with esophageal primary lesion, chemoradiotherapy is actively performed. Furthermore, supraclavicular lymph node metastasis in thoracic esophageal tumor is classified as Stage IV, and chemoradiotherapy is applied if metastatic lymph nodes are included in the field of irradiation.

3) Standard treatment for distal metastasis cases

Systemic chemotherapy is indicated for distal metastasis and recurrent cases regardless of the organ, and a regimen is chosen for the small cell lung cancer. With respect to NECs with esophageal primary lesion, even if there is organ metastasis, palliative chemoradiotherapy is prioritized for patients incapable of oral intake of drugs due to esophageal constriction.

The efficacy of everolimus and sunitinib has been demonstrated for NETs with pancreatic primary lesions. Furthermore, Octreotide and Lantreotide effectively inhibit proliferation of NETs that originate in the midgut and pancreatic/gastrointestinal NETs, respectively. However, these results are from clinical trials conducted in patient populations corresponding to NETs. Since the clinical presentations of NETs and NECs differ significantly, these treatments cannot be indicated for NECs during routine practice.

4) Indication of resection for distal metastasis

There is no consensus on the resection criteria specific to the NECs. Since the progression of NETs is slow, resection is actively considered even where there is distal metastasis to the liver, but as NEC progresses fast, surgical resection cannot be indicated for cases with distal metastasis[19, 21].

5) Prognosis

In a preceding study, Yamaguchi and Machida carried out a multicenter joint observational study of poorly

differentiated NEC (WHO 2010 NEC, including patients with clinical diagnosis of poor differentiation) in a total of 23 facilities, including facilities participating in JCOG Hepatobiliary and Pancreatic Oncology Group, former Gastroenterology Group, and observer facilities.

In the multicenter joint study by Yamaguchi and Machida, 89% of cases (229/258 patients) receiving systemic chemotherapy had distal metastasis, with a median survival time (MST) of 11.5 months for all patients, 11.2 months for distal metastasis cases, and 15.9 months for locally advanced cases. MST according to organ was 13.4 months for esophageal primary lesion (N = 85), 13.3 months for gastric primary lesion (N = 70), 29.7 months for small intestine/duodenal primary lesion (N = 6), 7.6 months for colonic primary lesion (N = 31), 8.5 months for pancreatic primary lesion (N = 35), and 7.9 months for hepatobiliary primary lesions (N = 31) (Table 2.1.4). While there were 9 cases of five-year survival among all the gastrointestinal cases, there were no five-year survival cases amongst those with primary lesions in hepatobiliary and pancreatic organs[10]. In a multicenter joint observational study for NECs conducted in four Northern European countries (hereafter referred to as NORDIC NEC Study), the MST for NECs with gastrointestinal primary lesion (albeit including ~30% of NECs with primary lesion sites unknown) was 11 months[11]. According to the U.S. SEER database, the MST of NEC was 10 months[3]. Based on the above information, despite differences in the primary lesion organ, the prognosis of the target population of this study is thought to have an overall MST of 10–11 months.

Table 2.1.4. Treatment results according to primary lesion organ for non-resectable/recurrent NECs that underwent systemic chemotherapy

	Esophageal	Gastric	Duodenal/small intestine	Colonic	**Gastrointestinal overall**	Pancreatic	Hepatobiliary	**Hepatobiliary and pancreatic organs overall**
N	85	70	6	31	**192**	35	31	**66**
Response rate（%）	58%	43%	50%	29%	**47%**	17%	16%	**17%**
Median PFS（month）	5.8	4.9	7.3	3.7	**5.1**	3.2	4.1	**3.7**
MST（month）	13.4	13.3	29.7	7.6	**13**	7.9	8.5	**7.9**

2.1.5. Tumor-related complications

We, here, report the complications that require attention during patient management, according to the organ of primary lesion. It is a rare disease and frequencies remain unknown.

1) Esophageal primary

Tumor bleeding/pain/esophageal stenosis/fistula formation due to primary lesion in the esophagus; hoarseness/difficulty swallowing/aspiration pneumonia caused by recurrent laryngeal nerve palsy due to lesion in lymph node metastasis; jaundice and liver failure associated with liver metastasis; respiratory failure and hemoptysis associated with lung metastasis, airway constriction, and pain due to lymph node metastasis; hypercalcemia, tracheal stenosis, tracheal obstruction, and suture failure due to disease progression.

2) Gastric primary

Chronic bleeding from primary lesion, accompanied by anemia, stomach pain, nausea, vomiting, gastric perforation, bloating, suture failure, fistula formation, cardiac/pyloric stenosis, ascites retention due to peritoneal metastasis, ileus, hydronephrosis due to ureteral stenosis, obstructive jaundice/liver failure due to bile duct stenosis, and obstructive jaundice/liver failure due to hepatic portal lymph node metastasis.

3) Small intestine/colonic primary

Tumor hemorrhage, ileus, fistula formation in the small intestine/bladder/vagina, intestinal obstruction, intestinal stenosis, intestinal perforation, and pelvic infection

4) Hepatobiliary and pancreatic primary

Cancer pain, rupture, obstructive jaundice associated with the growth of primary tumor, weight loss, fever, pancreatitis, cholangitis, liver abscess, cholecystitis, biliary hemorrhage, duodenal hemorrhage, duodenal stenosis, anastomotic ulcer/stenosis/leakage, symptoms associated with stenosis/obstruction of portal vein (liver

dysfunction/liver failure, esophageal varices and their rupture, gastritis, ascites retention, and hepatic encephalopathy/coma associated with portal hypertension).

5) Paraneoplastic syndrome

In addition to NECs, there are reports of paraneoplastic syndrome that is often associated with small cell lung carcinoma[22-24]. We report examples of paraneoplastic syndrome seen with small cell lung carcinoma below.

Hyponatremia due to inappropriate sodium secretion, psychological symptoms (changes in personality) due to ectopic ACTH syndrome, hypertension, hypokalemia, high blood sugar, Lambert-Eaton myasthenic syndrome, paraneoplastic cerebellar degeneration associated with autoantibody production (ataxia in the limbs, dysarthria, and nystagmus), paraneoplastic encephalomyelitis/sensory neuron disease (dementia, cranial nerve symptoms, dizziness, ataxia, autonomic imbalance, transverse paralysis, and sensory disorders).

Apart from these, there are thromboembolic events, aspiration (due to gastrointestinal stenosis), anemia, tumor pain, acute renal failure, myositis, and pulmonary fibrosis.

6) Complications due to metastatic lesions

Liver failure, pain, hepatobiliary infection, hemorrhage, bile duct stenosis, bile duct obstruction, respiratory failure, tracheal hemorrhage, respiratory tract infection, atelectasis, airway constriction, ascites, bloating, ileus, ureteral stenosis, ureteral obstruction, urinary retention, urinary tract infection, hiccups, pleural effusion, pleural hemorrhage, chest pain, pathological fracture, pain, hypercalcemia, meningeal carcinomatosis, ataxia, ischemia cerebrovascular, intracranial hemorrhage, nausea, vomiting, dizziness, consciousness disorder, cognitive disturbance, dysphasia, seizure, spasticity, edema limbs, intestinal obstruction/stenosis/perforation, pancreatitis, disseminated intravascular coagulation, thrombocytopenia, anorectal infection, anal hemorrhage, hoarseness, and superior vena cava syndrome

7) Others

General pain management including narcotic analgesics is performed for cancer pain.

Gastrointestinal stenting and bypass surgery are performed for esophageal stenosis, cardiac/pyloric stenosis, and duodenal stenosis.

Percutaneous transhepatic cholangial drainage (PTCD), percutaneous or endoscopic stenting, and bile duct jejunostomy is performed for obstructive jaundice. In addition, complications associated with biliary drainage and gastrointestinal stenting are listed below.

- PTCD, including internal and external fistula tube placement:
  Cholangitis, pancreatitis, cholecystitis, liver abscess, sepsis, biliary hemorrhage, PTCD tube obstruction/deviation, peritonitis, and pneumothorax/pleurisy
- Biliary stenting:
  Cholangitis, pancreatitis, cholecystitis, liver abscess, sepsis, biliary hemorrhage, peritonitis, pneumothorax/pleurisy (for percutaneous stenting), duodenal perforation, pneumonia (for endoscopic stenting), stent obstruction/deviation, duodenal ulcer, and duodenal hemorrhage
- Bile duct jejunostomy:
  Cholangitis, pancreatitis, cholecystitis, liver abscess, and sepsis
- Gastrointestinal stenting:
  Hemorrhage, perforation, pain, stent deviation, stent obstruction, foreign-body sensation, intestinal strangulation, ulcer formation, fever, sepsis, infection, diarrhea, constipation, tenesmus or uncontrollable urination/incontinence symptoms (colon), thyroid injury (esophagus), carotid artery injury (esophagus), and mediastinal abscess (esophagus).

2.1.6. Recurrent/progression

NEC is a very rare disease, and most reports of cases of surgery coincide with the case reports. According to the review by Arai et al., out of the 55 patients with NEC with gastric primary lesion who underwent surgical resection, only three patients survived for two years or more[25]. According to the report by Fischer et al., the MST of 13 patients with NEC with pancreatic primary lesion who underwent surgical resection was 11.7 months (seven of the patients showed distal metastasis)[26]. The form of recurrence among surgery cases remains unknown. The multicenter joint

study by Yamaguchi and Machida reported PFS among cases that underwent systemic chemotherapy with respect to the median as 5.8 months for esophageal primary lesions, 4.8 months for gastric primary lesions, 7.3 months for duodenal small intestine primary lesions, 3.7 months for colonic primary lesions, 3.2 months for pancreatic primary lesions, and 4.1 months for hepatic/biliary primary lesions[10]. In the NORDIC NEC study, the median PFS was 3 months for esophageal primary lesions, 5 months for gastric primary lesions, 3 months for colonic primary lesions, 4 months for rectal primary lesions, 5 months for pancreatic primary lesions, and 4 months for cases with unknown primary lesion site [11].

2.1.7. Prognostic/predictive factors

When prognostic factors were examined by multivariate analysis in the multicenter joint study by Yamaguchi and Machida, out of the various factors such as sex, age (younger or older than 60 years), PS (0 or 1 vs. 2 or more), primary lesion organ (gastrointestinal primary lesions vs. hepatobiliary or pancreatic primary lesions), LDH levels (below vs. above the upper limit of facility standard), presence or absence of liver metastasis, presence or absence of history of radical resection and treatment regimen (IP therapy vs. EP therapy); the independent prognostic factors identified were gastrointestinal primary lesion (vs. hepatobiliary or pancreatic primary lesion, hazard ratio (HR): 0.58), and LDH levels being below the upper limit of facility standard for LDH (vs. above the upper limit of facility standard for LDH, HR: 0.65).

Although IP therapy exhibited slightly better impact on overall survival as compared to EP therapy with an HR of 0.8, the *p*-value of 0.389 meant that there was no significant difference between the two therapies[10]. The NORIDIC NEC study reported poor PS, colonic primary lesions, high platelet count, and high LDH levels to be the main factors behind poor prognosis[11]. However, these prognostic/predictive factors were not obtained with a global consensus.

2.1.8. Rationale for selection of the target population

The objective of this study is to develop a primary chemotherapy regimen for non-resectable/recurrent NECs, and out of the non-resectable or postoperatively recurrent NECs with gastrointestinal primary lesions or hepatobiliary or pancreatic primary lesions (shaded parts in Table 2.1.2 of WHO 2010 classification of NEC), we established chemotherapy-naïve patients to be the target population.

In terms of whether or not to consider MANEC a target of this study, a discussion is needed from the viewpoint of standard treatment. MANEC is considered when 30% or more of the carcinoma consists of adenocarcinoma components, and it is treated as either adenocarcinoma or NEC by discretion of the attending physician, with no real consensus on standard treatment. MANEC was however, excluded from this study, as its disease concept is different from NEC in terms of the standard treatment, which has a consensus regarding the treatment by a “regimen according to small cell lung carcinoma”. In fact, a questionnaire was provided to the three groups participating in this JCOG study (response obtained from 52 facilities). The results showed that 79% of facilities considered the WHO 2010 classification of NEC as the appropriate tissue type for this study, while only 13% responded that consideration of NEC+MANEC would be appropriate. Therefore, majority of facilities considered “WHO 2010 classification of NEC as the appropriate target of this study”.

While diagnosis of NECs is generally carried out using tissue samples and biopsy samples, as mentioned in 2.1.3, there is no consensus methodology to strictly distinguish NEC and MANEC using biopsy samples. As a result of group discussions, a consensus was reached between the three groups that cases presenting with NEC components by biopsy sample-based diagnosis can be enrolled. Although the use of this method would mean that a certain percentage of patients with MANEC, who were not intended to participate in this study, would be enrolled however, currently there are no appropriate means to avoid this. Therefore, we decided to create a consensus for the future based on information obtained from this study.

1）**<u>Reason for targeting gastrointestinal/hepatobiliary or pancreatic primary lesion NEC</u>**

Various guidelines recommend treatment regimens suited for small cell lung carcinoma, regardless of the organ of primary lesion, and is the rationale showing the validity of the treatment development for NEC across many organs. Furthermore, taking into consideration the frequency of the disease, it is unrealistic to develop treatments according to each organ, and in practice the Minnie-Pearl Cancer Research Network Study (mentioned later in 2.2.2), which is a relatively large-scale clinical study of NEC, development of treatment was carried out in a cross-organ

manner[27].

On the other hand, if we take hypothesize that we do not sufficiently understand how the clinical presentations of the disease vary according to the organ, the fact that this is the first randomized study in Japan, and that the maintenance of foundation for clinical studies of this disease in Japan is inadequate at present time, the hurdle is too high to plan a study across all organs including the fields of gynecology and urology. Based on the above consideration, we have decided to target all gastrointestinal organs which have a relatively large number of common points between each other in terms of clinical presentation and types of complication.

According to the multicenter observatory study by Yamaguchi and Machida, the prognosis of NEC with hepatobiliary or pancreatic primary lesion was significantly poorer than that of NEC with gastrointestinal primary lesion (MST: 7.9 vs. 13.0 months, respectively), but we determined that it is possible to accurately evaluate the efficacy of the treatment regimen by randomizing the subjects using the primary lesion site (gastrointestinal vs. hepatobiliary or pancreatic) as an allocation adjustment factor. Therefore, we considered patients with both, gastrointestinal/hepatobiliary or pancreatic primary lesions as one target population of this study, and to examine differences between organs exploratively by subgroup analysis.

2) Reason for including hepatic NEC cases (hepatic primary lesion or liver metastasis with primary lesion site unknown)

Even among NECs, cases with hepatic primary lesions are particularly rare. There are no specific reports on the frequency of incidence and differences from the other organs and reports are limited only to case reports and their reviews. Moreover, since the liver is a major organ for metastasis of primary lesions into other organs, even when tumors have been clinically identified in the liver, in many cases the primary lesion tends to be in another organ. Therefore, liver should not be deemed the primary lesion site without sufficient examination for a primary lesion. On the other hand, although very rare, there have been reports of NEC with hepatic primary lesion[28]. Even if sufficient search for primary lesion leads to no indication of the same outside of the liver, currently it is not possible to distinguish whether it is a case of “NEC with hepatic primary lesion” or “lesion in the liver is a metastatic lesion, with the primary lesion unknown”. In this study, such situations wherein “liver alone has identifiable lesions” would be henceforth, referred to as “hepatic NEC (hepatic primary lesion or liver metastasis from an unknown primary lesion site) for convenience. As the NCCN Guidelines[19] and ENETS Guidelines[20, 21] indicate the usefulness of FDG-PET for detailed examination of the primary lesion, we would also examine the primary lesion using FDG-PET. Furthermore, by referring to the diagnostic procedures for cancers with unknown primary lesion site, detailed examinations into the primary lesion would be carried out by otolaryngological (head and neck) examination and urological examination for men only, and gynecological examination for women only).

Such hepatic NEC (hepatic primary lesion or liver metastasis from an unknown primary lesion site) is anticipated even from an anatomical viewpoint, since the tumor-related complications are the same as that for other gastrointestinal primary lesion NECs, and there is no issue with treating them the same as for gastrointestinal primary lesions.

3) Reason for orienting the study for non-resectable or recurrent cases

As mentioned in 2.1.4 “Standard treatment according to disease staging and outline of prognosis”, systemic chemotherapy is indicated for cases with distal metastasis or recurrent cases. Locally advanced cases are particularly prone to pancreatic or bile duct primary lesions situated in the vicinity of vital vessels, and while the NCCN Guidelines propose chemoradiotherapy according to treatment for small cell lung carcinoma[19], the ENETS Guidelines provide no clear indication[21]. In reality, for the treatment of NEC which has a variety of primary lesion organs (scope of irradiation) there is not enough information available for the chemotherapy regimen that should be combined in terms of its optimum dose, the radiation dose, efficacy, and safety. For this reason, chemoradiotherapy cannot be considered the standard treatment with consensus, and systemic chemotherapy for distal metastasis is used widely except for pathologies involving frequent stenotic symptoms when the primary lesion is in the esophagus. For this reason, this study would enroll non-resectable or recurrent cases that include locally advanced cases.

The section “3.6 Definition of non-resectable NEC” shows the definition of non-resectable cases summarized by referring to the JCOG protocol for clinical studies of systemic chemotherapy (esophagus: JCOG0807, stomach: JCOG1013 or JCOG1002, bile duct: JCOG0805, pancreas: JCOG1106). Furthermore, while radical resection has

been considered for cases of colonic primary lesion adenocarcinoma with liver or lung metastasis, as cases of NEC with distal metastasis are not indicated for resection, we defined non-resectable cases as those in "Stage IV", and followed this definition for duodenal primary lesions, small intestine primary lesions, and appendix primary lesions. Furthermore, with respect to NEC with esophageal primary lesion, chemoradiotherapy is well-indicated for cases with supraclavicular lymph node metastasis of thoracic esophageal tumor even in Stage IV, and hence were not included in this study.

4) Treatment of draft of new classification of NEC

In recent years, proposals have been made to further differentiate the WHO 2010 classification of NECs (G3 in ENETS) to "Grade 3 proliferative tumors showing the same morphological presentations as NET" and "Grade 3 tumors with strong morphological atypicality (previously classified as poorly differentiated NECs)"[29, 30], or by Ki-67 index of 20–50% and 50% or more[11, 19]. This is yet to be confirmed; however, in this study we would have used the WHO 2010 classification. However, we would have used samples collected for central pathological diagnosis to carry out studies related to classification which exploratively adds grade classification and morphological differentiation levels.

2.2. Standard treatment for target disease

Currently, there is no standard drug treatment for non-resectable/recurrent NECs with efficacy verified by a randomized controlled trial. However, given the similarity between pathological and clinical presentations, treatments according to small cell lung carcinoma have been attempted, and there have been reports of positive treatment outcome in small-scale clinical studies and observational studies.

2.2.1. Standard treatment for small cell lung carcinoma

Cisplatin-based multidrug combined therapy is the standard first-line treatment of extensive disease (ED) small cell lung carcinoma. While cyclophosphamide/doxorubicin/vincristine (CAV therapy) was established as a standard treatment in the 1970s in Europe and U.S., the etoposide/cisplatin combined therapy (EP therapy) was introduced in the late 1980s. A comparative study of EP therapy and CAV therapy did not show superiority of EP therapy over CAV therapy in terms of survival, but the response rate (CAV: 51% vs. EP: 61%) and MST (CAV: 8.3 months vs. EP: 8.6 months) were almost the same, and the lighter toxicity level (mucositis, interstitial pneumonia, hemotoxicity) meant that EP could be used as a standard treatment[31]. Thereafter, the JCOG Lung Cancer Group carried out the "comparison between EP therapy and Irinotecan/Cisplatin combined therapy (IP therapy) for Extensive-stage Small Cell Lung Cancer (JCOG9511)", and reported that the IP therapy provided a significantly better overall survival, with MST being 9.4 months vs. 12.8 months ($p = 0.002$) for IP therapy[32]. However, the two additional large-scale studies conducted primarily in U.S. did not show superiority of IP therapy over EP therapy, and for this reason IP therapy has not been not used as the standard treatment overseas[33, 34]. Furthermore, while the results of the "Randomized controlled trial to verify the non-inferiority of amrubicin/cisplatin combined therapy (AP therapy) over IP therapy (JCOG0509)" were reported in the 2012 conference of the American Society of Clinical Oncology (ASCO), but AP therapy was still considered significantly inferior to IP therapy[35]. Presently, the JCOG Lung Cancer Group considers IP therapy to be the standard treatment for extensive-disease small cell lung carcinoma.

2.2.2. Standard treatment for extrapulmonary NEC

While there are scattered reports relating to use of CAV therapy, EP therapy, IP therapy, and other multidrug combined therapies for extrapulmonary NEC, these reports are primarily from observational studies. Since these reports precede the unification of concept and classification methods of this disease, the nomenclature such as NEC (anaplastic type), NET (poorly differentiated type) and extrapulmonary small cell lung carcinoma have still been used. These reports, however, are presumed to be targeting almost the same disease group as NEC. There are no reports of prospective studies using EP therapy for extrapulmonary NEC. Observational studies have reported response rates between 42–67%, and MST between 15–19 months (Table 2.2.2a). On the other hand, IP therapy has reported results from observational studies and clinical studies, with response rates between 7–83% and MST of 10.1–22.6 months (Table 2.2.2b). As mentioned above, reports related to extrapulmonary NEC are limited to use of EP therapy and IP therapy in observational studies and small-scale clinical studies. The largest number of case enrollments in a clinical study of extrapulmonary NEC was seen in the Minnie-Pearl Cancer Research Network Study, which evaluated the efficacy of triple-drug combined therapy using carboplatin/etoposide/paclitaxel. The

response rate to this triple-drug combined therapy was 53%, while the MST was 14.5 months and adequate, there was intense toxicity involved, and the results were not significantly different from EP therapy, so this regimen was not considered the standard treatment[27].

The 2014 NCCN Guidelines recommend treating extrapulmonary NEC using a regimen according to small cell lung carcinoma, and the guidelines for treatment of small cell lung carcinoma mention both EP therapy and IP therapy as recommended regimens.

Therefore, the standard treatment for extrapulmonary NEC is taken as EP therapy or IP therapy.

Table 2.2.2.a. EP therapy for NEC (observational studies)

Reported year/Reporter	Target	N	Response rate	MST (in months)
1991/ Moertel[18]	Pancreatic/gastrointestinal NEC (anaplastic type)	18	67%	19
1999/ Mitry [36]	Pancreatic/gastrointestinal NEC	41	42%	15
2001/ Marie-Louise [37]	Pancreatic NET (well: 11, poorly: 4) Gastrointestinal cartinoid: 21	33	18%	19
1994/ Lo Re G [38]	Extrapulmonary SCLC	13	69%	NE
2010/ Iwasa [39]	Hepatobiliary or pancreatic NEC	21	14%	7.3
2012/ Yamaguchi [10]	Gastrointestinal NEC	12	75%	14
2012/ Yamaguchi [10]	Hepatobiliary or pancreatic NEC	34	12%	6.9
2012/ Sorbye [11]	Gastrointestinal primary lesion NEC (including primary lesion unknown)	129	31%	12

Table 2.2.2.b. IP therapy for NEC (shaded are clinical studies, others are observational studies)

Reported year/Reporter	Target	N	Response rate	MST (in months)
2003/ Hou [40]	NEC (gastrointestinal-80%)	18	43%	NE
2005/ Chin [41]	Esophageal NEC	12	83%	14
2011/ Okita [42]	Gastric NEC	12	75%	22.6
2012/ Yamaguchi [10]	Gastrointestinal NEC	142	51%	13.4
2012/ Yamaguchi [10]	Hepatobiliary or pancreatic NEC	18	39%	10.1
2006/ Kulke [43]	Pancreatic/gastrointestinal (including NET G1/G2)	15	7%	11.4
2008/ Mani [44]	Pancreatic/gastrointestinal NEC	20	58%	NE
2008/ Jin [45]	Extrapulmonary NEC	15	67%	11.4.

Table 2.2.2.c. Phase II study of carboplatin/etoposide/paclitaxel triple-drug combined therapy for NEC

Reported year/Reporter	Target (breakdown)	N	Response rate	MST (in months)
2006/ Hainsworth [27]	NEC (Colon：9, lung: 7, skin: 4, pancreas：3, gall bladder：1, thyroid:1, stomach: 1, esophagus: 1, endometrium:1, maxillary sinus: 1, prostate: 1, primary lesion unknown: 48)	78	53%	14.5

2.2.3. Standard treatment for gastrointestinal/hepatobiliary or pancreatic primary NEC

Treatments for extrapulmonary NEC have not been developed according to any specific organ, and the standard treatment for gastrointestinal/hepatobiliary or pancreatic NEC is also both, EP therapy and IP therapy. While both treatments are considered standard treatments, since this study considers them both to be study treatments, the anticipated effect and expected adverse reactions from EP therapy and IP therapy have been described in "2.3.2 Study treatment(s) of this study".

Herewith, we describe the current status of the usage of each treatment in Japan and overseas. According to the multicenter joint study by Yamaguchi and Machida, 160 out of 258 patients (62%) who received systemic chemotherapy underwent IP therapy, the most common treatment, followed by EP therapy (46 patients, 18%). Although there is big deviation where 92% of gastrointestinal primary lesion NEC (142/154 patients) were given IP therapy, and 65% of hepatobiliary or pancreatic primary lesion NEC (34/52 patients) were given EP therapy, the present situation shows that treatments are being selected by discretion or preference of the facility/physician.

Furthermore, even with respect to the dosing schedule, the method of administration has not necessarily as per the treatment of small cell lung carcinoma. There is no unified consensus, as some facilities use methods used for treatment of gastric cancer (JCOG9912 regimen), based on the reasoning that they have familiarity with IP therapy. On the other hand, in the NORDIC NEC study the most common treatment used was the EP therapy, which was administered to 129 out of 252 patients (51%) who received systemic chemotherapy, followed by a combined therapy consisting of carboplatin and etoposide therapy (67 patients, 27%). Combined therapy of platinum and etoposide was used often, which is another choice of treatment in Japan[11].

2.3. Rationale for establishment of treatment plan

2.3.1. Drugs

1) Etoposide

Etoposide exerts an antitumor effect by inhibiting topoisomerase II, which catalyzes the untangling of supercoiled DNA strands. Main toxicities include myelosuppression, nausea/vomiting, alopecia, and stomatitis.

2) Cisplatin

It is a complex ion form of the heavy metal platinum which shows anti-tumor effect by cross-linking double-stranded DNA. Currently, it is considered one of the key drugs for NEC treatment, being a central medicine for the treatment of lung cancer due to its synergistic effect with radiation therapy and various drugs, and also due to its low bone marrow toxicity when used alone. Toxicities include nausea/vomiting, nephrotoxicity, and neurotoxicity. Sufficient infusion of cisplatin before and after drug administration is necessary to prevent nephrotoxicity.

3) Irinotecan

Irinotecan is a topoisomerase I inhibitor developed in Japan that inhibits DNA synthesis. The drug is directly converted into the active metabolite (SN-38) in human liver and various tissues by carboxyesterase. As it shows a potent antitumor effect against SCLC even when used on its own, this drug is used very often in routine clinical practice to treat NEC as well. Diarrhea and myelosuppression are observed as dose-dependent toxicities. Nausea/vomiting and interstitial pneumonia have also been noted in patients.

2.3.2. Study treatment(s) of this study

1) Etoposide/cisplatin combined therapy (EP therapy)

In the multicenter joint study performed by Yamaguchi and Machida, the response rate of gastrointestinal primary lesion NEC to EP therapy was 75% (9/12 patients), with an MST of 14 months. The response rate of hepatobiliary and pancreatic primary lesion NEC to EP therapy was 12% (4/34 patients), and the MST was 6.9 months. With respect to safety, the multicenter joint study by Yamaguchi and Machida showed no treatment-related deaths associated with EP therapy (N = 46) administered as first-line treatment for gastrointestinal/hepatobiliary and pancreatic primary lesion NEC, and toxicity-related study discontinuation was observed in 6.5% of cases. Details of toxicities remain unknown as they were not investigated. The observational study of EP therapy for hepatobiliary and pancreatic primary lesion NEC (N = 21) carried out by Iwasa et al. at the National Cancer Center Hospital showed major Grade 3/4 adverse events to be neutropenia (90%), nausea (33%), and anorexia (24%). Grade 3 febrile neutropenia was observed in 8 patients (38%)[39]. Table 2.3.2 shows the toxicity profile of EP therapy from the JCOG9511 study which treated small cell lung carcinoma (Grade 3 or higher as per JCOG toxicity criteria).

Table 2.3.2. Toxicity of EP therapy and IP therapy in studies treating small cell lung carcinoma※
※Report by Iwasa et al., JCOG0509：CTCAE v3.0 Grade 3 or higher, JCOG9511：JCOG toxicity criteria Grade 3 or higher

	EP therapy		IP therapy	
	Report by Iwasa et al.[34]	JCOG9511[27]	JCOG9511[27]	JCOG0509[30]
Neutropenia	90%	92.2%	65.3%	58.5%
Leukopenia	71%	51.9%	26.7%	22.5%
Decreased hemoglobin	29%	29.9%	26.7%	23.2%
Thrombocytopenia	24%	18.2%	5.3%	2.1%
Diarrhea	0%	0%	16%	7.7%

Nausea	33%	6.5%	13.3%	6.3%
Aspartate aminotransferase increased	19%	2.6%	0%	-
Alanine aminotransferase increased	24%	3.9%	4%	-
Blood bilirubin increased	19%	0%	0%	-
Creatinine increased	0%	0%	0%	-
Peripheral motor neuropathy	0%	0%	0%	-
Febrile neutropenia	38%	0%	0%	1.4%

There are several reports concerning the specific dosage schedule/administered dose for EP therapy, and four dosing methods have been described in the NCCN Guidelines for SCLC as well. However, it is not clear as to which of the dosing methods is the best, and the dosing method for NEC varies between reports. In Japan, the dosing for EP therapy according to JCOG9511 is also often used for NEC. For this reason, this study would also follow the same method of administration. In terms of the number of cycles, a comparison of 4 courses vs. 8 courses of combined chemotherapy including cyclophosphamide for SCLC showed poor efficacy even when the treatment continued for long-term[46], and treatment of 4 cycles has also been specified in JCOG9511. There have been no such comparative studies for gastrointestinal/hepatobiliary and pancreatic primary lesion NEC, rather, there is no evidence to suggest that first-line chemotherapy should be discontinued if the chemotherapy is efficacious and toxicity is within the permitted scope. Unlike SCLC, NEC does not respond well to chemotherapy and tends to re-exacerbation to the underlying disease soon after the end of chemotherapy. For this reason, continuation of chemotherapy is very likely to be beneficial for patients with NEC as long as the treatment is effective.

While peripheral motor neuropathy, hearing impairment, and renal disorder are known accumulation toxicities of cisplatin, if sufficiently safe, it is ideal for treatments to continue as much as possible, considering the very few treatment options available for NEC. Based on the above rationale, we decided to ensure safety by establishing strict protocol treatment discontinuation criteria and chose not to limit the total dose or prescribe the number of treatment cycles.

2）Irinotecan/Cisplatin combined therapy (IP therapy)

In the multicenter joint study by Yamaguchi and Machida, the response rate of gastrointestinal primary lesion NEC to IP therapy was 51% (73/142 patients), with an MST of 13.4 months. The response rate of hepatobiliary and pancreatic primary lesion NEC to IP therapy was 39% (7/18 patients), and the MST was 10.1 months. There were no treatment-related deaths among 160 patients, and discontinuation due to toxicity in first-line chemotherapy was seen in 11.2% of cases. Details about toxicity were not collected. As a reference, Table 2.3.2 shows the toxicity profile of IP therapy from the JCOG9511 and JCOG0509 studies which treated small cell lung carcinoma.

There are several reports concerning specific dosing methods for IP therapy, and two dosing methods have been described in the NCCN Guidelines for SCLC as well. It is not known which of the dosing methods is the best, and the dosing method has also been variable in the NEC. In Japan, the dosing method of IP therapy, according to JCOG9511 is considered the standard treatment for SCLC and the same method has also been used in this study.

The total dose and number of cycles would have not been prescribed for the same reason as EP therapy, and safety has been ensured by establishing strict protocol treatment discontinuation criteria.

3）Other study treatment candidates

Presently, there are no drugs or treatment methods, including molecular-targeted drugs, which surpass the EP and IP therapy. There are also no planned or ongoing large-scale clinical trials globally, related to first-line treatment of NEC.

As the second-line treatment, a phase II study of Everolimus is underway for pancreatic primary lesion NEC resistant or unresponsive to platinum preparations. It is a multicenter study being carried out in 31 facilities in Japan, led by the National Cancer Center East Hospital, through the cancer research and development fund (UMIN000012752）.

2.3.3. Summary of risk/benefit balance of the standard treatment and study treatment

In the multicenter joint study by Yamaguchi and Machida, IP therapy had better prognosis compared to EP therapy (MST: 13.0 months vs. 7.3 months; $p < 0.0001$). However, as shown in Table 2.3.3, since IP therapy was more frequently chosen for gastrointestinal primary lesions (142/154 patients) and EP therapy for hepatobiliary and

pancreatic primary lesions (34/52 patients), it has not been possible to determine whether this difference is due to difference in efficacy between the regimens, or due to differences in the primary lesion organ. Primary lesion organ remained a significant prognostic factor after multivariate analysis, and while IP therapy turned out to be a slightly better regimen than EP therapy with HR = 0.8 (95% CI. 0.48–1.33), $p = 0.389$ indicated that there was no significant difference between the two (see “2.1.5 Prognostic/predictive factors”). Taking into account the number of EP therapy cases (46 patients), the fact that it was an observational study, and including other unknown bias, the data interpretation is fairly limited. Therefore, based on these results we are unable to deem either of IP or EP therapies to be more efficacious than the other.

With regard to toxicity, while myelosuppression such as neutropenia is milder in IP therapy than EP therapy, frequencies of events such as diarrhea and nausea are high. While the two treatments have different toxicity profiles, it is difficult to conclude that one has clearly worse toxicity than the other. The risk of bile duct obstruction is high in biliary and pancreatic primary lesion NECs and the use of irinotecan (which undergoes biliary excretion) in patients exhibiting biliary excretion disorder runs the risk of increased toxicity, hence care must be taken while administering irinotecan. We believe that this risk can be avoided by appropriate pre-treatment drainage and proper monitoring during treatment. Based on the above, it is difficult to assign superiority to IP therapy or EP therapy over the other in terms of risk/benefit balance, and both the regimens can be considered suitable standard treatments.

Table 2.3.3. Summary of results from multicenter study by Yamaguchi and Machida

		IP	EP	*p*-value*
Total	N	160	46	
	Response rate（%）	50	27	＜0.001
	Median PFS (in months)	5.2	4.0	0.033
	Median OS (in months)	13.0	7.3	＜0.0001
Gastrointestinal primary lesion NEC	N	142	12	
	Response rate（%）	51	75	0.14
	Median PFS (in months)	5.4	4.9	0.585
	Median OS (in months)	13.4	14.0	0.976
Hepatobiliary and pancreatic primary lesion NEC	N	18	34	
	Response rate（%）	39%	12%	0.034
	Median PFS (in months)	4.4	3.7	0.056
	Median OS (in months)	10.1	6.9	0.05

*Response rate by chi-squared test, PFS and OS by log-rank test

2.3.4. Post-treatment(s)

In the multicenter joint study by Yamaguchi and Machida, 56% (116/206 patients) of patients with NEC who received IP therapy or EP therapy were administered chemotherapy as the second-line of treatment. The most common second-line chemotherapy after IP therapy was amurcibin (22/88 patients), while irinotecan was the most common second-line treatment after EP therapy (13/28 patients), and we expected a similar pattern for the second-line treatments in this study as well. The overall performance of second-line chemotherapy was poor with a response rate of 11%, and PFS of 2.1 months. As such, the significance of the second-line treatment has not been indicated, and hence, no standard treatment has been established.

2.4. Study design

2.4.1. Rationale for establishing endpoints

For NECs, an alternative endpoint for overall survival, such as PFS has not been established as an indicator for comparing the usefulness of the treatment regimens. Therefore, comparison of overall survival was considered appropriate when examining the therapeutic effects. For the same, overall survival was examined as the primary endpoint. The secondary endpoints to evaluate the efficacy and safety were the response rate (only for patients with a measurable lesion), progression-free survival, incidence rate of adverse events, and dose intensity of cisplatin.

2.4.2. Clinical hypothesis and rationale for setting the number of enrollments

This study compares the two standard treatments, both of which are difficult to deem superior than the other in terms of efficacy and safety. We have, therefore, adopted a study design using bilateral testing. The main clinical hypothesis of this study is that either IP therapy or EP therapy is likely to provide better overall survival than the other treatment group, and should this hypothesis be validated, the superior treatment with statistical significance would be deemed the better treatment, and would therefore be positioned as the standard treatment in future. If the study fails to validate this hypothesis, it would indicate that there was no clinically significant difference in overall survival between the two groups, and if there are no large differences in terms of toxicity, we would conclude that both treatments are viable options as the standard treatment. However, if unlike our initial hypothesis, one treatment shows clearly more toxicity than the other, we would reconsider the study design before carrying out the main analysis.

Furthermore, as mentioned in "section 2.1.1 Epidemiology", gastroenterological primary lesion NEC, which is the primary target of this study, is a very rare disease with an annual prevalence of around 3 in 100,000 people, hence, the significance level of testing has been placed at 10% bilaterally, instead of 5% bilaterally. In the multicenter joint study conducted by Yamaguchi and Machida, the MST of gastrointestinal/hepatobiliary or pancreatic primary lesion NEC was 11.5 months. To determine if one treatment is superior to the other, we expected a difference of four months in terms of MST. If we expect the more inferior treatment to have an MST of 8.0 months and the superior treatment to have an MST of 12.0 months, the number of enrollments are calculated using the aforementioned parameters and based on the discussion mentioned later (see "12.2 Expected number of enrollments/Enrollment period/Follow-up period"). This provided a research period of 7- years, consisting of 6-years of enrollment period and one year of follow-up period, and with $\alpha = 0.1$ bilaterally and detection power of 70% to detect differences between the two groups, 63 subjects were required per group. Taking into consideration some subjects who could be lost post follow-up, we aimed to enroll 70 subjects per group, and a total of 140 subjects between two groups.

If enrollment proceeds better than expected than prior to the start of the study (i.e. if the number of enrolled subjects reach 70 in less than 2.5 years from the start of enrollment), the number of enrollments is likely to be re-established by changing the detection power from 70% to 80% during the study, with the aim of obtaining more accurate results.

＜Addition to ver.1.1＞

The pace of enrollment after the start of enrollment period exceeded expectations, and the number of enrollments reached 70 subjects in October 2016, which was 2 years and 2 months after the start. As this exceeded the criteria of 70 subjects in less than 2.5 years, since the start as provided above, we obtained the approval of the Hepatobiliary and Pancreatic Oncology Group Meeting on October 29, 2016, Esophageal Cancer Group Meeting on November 19, 2016, and Gastric Cancer Group Meeting on January 7, 2017 to change the detection power to 80% according to provisions at the time of study planning. Consequently, the number of intended enrollments was changed to 170 subjects.

2.4.3. Expected patient enrollment

In the multicenter joint study by Yamaguchi and Machida, 258 cases of gastrointestinal/hepatobiliary or pancreatic primary lesion NECs were reported from 23 sites during the 11 years between 2000 and 2011, but between 2000 and 2006, information could not be obtained because of old cases. When limited to the most recent five years (2006–2011), when sufficient information was obtained from each facility, the number of enrollments was placed at162 subjects.

This is a joint study between three groups, namely the JCOG Hepatobiliary and Pancreatic Oncology Group,

JCOG Gastric Cancer Group, and the JCOG Esophageal Cancer Group. Therefore, the total number of facilities, excluding overlaps, amounted to 82 facilities. Compared to the multicenter joint study by Yamaguchi and Machida, we expect an increase in the number of patients enrolled, and simple calculations lead us to extrapolate 115 subjects to be enrolled annually. On the other hand, if we take into account the possibility of patient enrollments being biased to some high volume centers, it would be difficult to estimate the effect of increasing facility count to proceed. In addition, considering that the study is a randomized, we estimate the annual number of patients enrolled to be between 30–50 people. Taking into account ineligible cases, we provided 6-years for patient enrollment period.

2.4.4. Rationale for setting allocation adjustment factors

1) Facility

It is widely known that background, treatment, efficacy evaluation, and safety evaluation of enrolled patients vary depending on the facility, and JCOG standards have been used to make adjustments between facilities.

2) Primary lesion organ (gastrointestinal tract vs. hepatobiliary or pancreatic organ)

In the multicenter joint study by Yamaguchi and Machida, multivariate analysis using the Cox proportional hazard model was carried out with 183 subjects, which indicated primary lesion organ (gastrointestinal tract vs. hepatobiliary or pancreatic organ) to be a significant prognostic factor.

2.4.5. Centralized pathological diagnosis

In this study, a centralized pathological diagnosis would be performed for analysis of NEC, despite patients being diagnosed by a third party. The operation of the centralized pathological diagnosis is described in section 15.1, while details of the operation have been described in the Centralized Pathological Diagnosis Procedure Manual. Furthermore, an analysis of endpoints based on the results of centralized pathological diagnosis would be provided as a reference.

2.5. Summary of expected advantages and disadvantages associated with study participation

2.5.1. Expected advantages

Drugs used for both groups of this study are treatments used in routine medical practice. As mentioned in the next section, although it would be necessary to use some drugs for which insurance coverage does not apply, since insurance claims for these treatments are made in a similar manner to general medical care however, in practice they do not receive insurance assessments.

Moreover, since the medical fees of study participants during the study, which includes drug fees, are paid in principle by the patients themselves and their insurance coverage, so the patients would receive no special medical or financial benefits from participation in this study.

2.5.2. Expected risks and disadvantages

Both treatment arms A and B would receive chemotherapy regimen used in routine medical practice, so they would be unlikely to be exposed to special risks or disadvantages not observed in routine medical practice. The descriptions in “2.3.3 Risk/benefit balance of standard treatment and study treatments” outline the expected risks and disadvantages for each treatment arm.

To minimize the risk of adverse events and disadvantages, the “ Patient selection criteria (Section 4)”, “ Criteria to change treatment (Section 6.3)” and “Concomitant/Supportive therapies (Section 6.4)” have been carefully considered for the three groups. Furthermore, the Institutional Review Board would monitor if the adverse events are within the scope of expectation, while any serious adverse events or unexpected adverse events would be carefully examined and reviewed according to the provisions related to “JCTN-Adverse Events Report Guideline” and “JCOG Guidelines for Handling Clinical Safety Information”, and a system has been provided to take any necessary countermeasures.

Precautions related to etoposide, irinotecan, and cisplatin

As of December 2013, etoposide therapy, which is the intended to be used in this study had not received insurance approval for the treatment of gastrointestinal or hepatobiliary and pancreatic primary lesion cancers. Irinotecan has also not received insurance approval for treatment of cancers with primary lesions in organs other than the stomach, colon, and rectum. Furthermore, as cisplatin is only indicated for esophageal, gastric cancer, and for biliary tract

cancers when combined with gemcitabine (25 mg/m^2), the dosage and administration used in this study have not been approved previously.

As each facility in this study may carry out the insurance claims in the same manner as routine general medical practice, the treatment may receive insurance assessment after-the-fact. However, if a facility incurs losses, the loss must be borne by the relevant facility (medical institution), as there is no system of compensation arranged by the JCOG Research Organization. If actual losses occur, the continued participation in the study would then be carefully discussed between the facility supervisor and the principal investigator/clinical trial secretariat. The facility supervisor would be expected to gain approval from the facility IRB and the head of the medical institutions.

2.6. Significance of this study

While both, EP therapy and IP therapy are standard treatments, the efficiency of either treatment being better than the other is not yet clear, and currently in routine medical practice, the choice of treatment is being made on the discretion or preference of the facility/attending physician.

If this study is able to clearly indicate the efficiency of EP therapy or IP therapy over the other, we expect that the diminished use of the relatively inferior treatment would contribute to improvement in patient prognosis. If the study indicates no clinically meaningful differences between the two treatments, the results would provide evidence that there is no significant issue with the treatments being chosen by discretion or preference of the facility/attending physician, which has been done traditionally without any data that directly compare the two treatments, although that would not indicate that the effects of the two treatments are equivalent. We also expect that the establishment of a highly reliable standard treatment in this study which would provide a foundation for therapeutic development when promising treatment regimen in the future.

As NEC is a rare disease, and since each clinician is not likely to have adequate experience, we believe that work associated with multicenter studies such as confirmation of diagnosis by centralized diagnosis/results feedback and sharing results of treatments by unified treatment regimen would lead to improvement of quality of care for NEC in Japan.

2.7. Associated research (including sample analysis research)

No such studies have been planned or carried out at the time of preparation of the protocol.

2.8. JCOG-Biobank Japan (BBJ) collaborating biobank

This study would participate in the banking of blood samples (DNA/plasma) through the JCOG-BBJ collaboration biobank based on the protocol common to all JCOG studies (hereafter termed as “common banking”).

Common banking by JCOG-BBJ collaborating biobank would collect and store the samples of patients enrolled in clinical studies conducted by JCOG regardless of presence/absence of pre-planned sample analysis research, would also provide samples for future analysis research and diagnostic information obtained through the main research.

The target of such biobanking are those patients who provided consent to participate in this study and gave consent to provide samples to the JCOG-BBJ collaborating biobank for their use in future sample analysis research (hereafter termed as consent for banking).

Samples collected during common banking include whole blood and preserved pathological tissue samples from routine clinical practice. Plasma and DNA separated/isolated from blood would be stored in the JCOG-BBJ collaborating biobank and would be provided for sample analysis research in the future. Preserved pathological tissue samples from routine medical practices such as surgery, and biopsy/clinical laboratory tests would also likely to be used for future sample analysis research. Although the type, sample preparation methods, and tissue quantity required would vary from study to study, there is no definite consensus that banking pathological tissue in a certain method is more efficient than others. Furthermore, it has been suggested that long-term preservation of a sectioned specimen from preserved pathological tissue may lead to sample deterioration (DNA fragmentation). The discussions between the of JCOG and BBJ personnel about these problems, led to the conclusion that preserved pathological tissue taken after medical examination shall require only patients’ consent for future use, and actual collection must be started after standardizing a separate protocol and specifying in it the procedures best suited for the details of the research.

The detailed procedures for sample collection, storage, and method of sample provision for future sample analysis

research in common banking have been stipulated in the “JCOG-BioBank Japan Collaborating Biobank Protocols” that apply to all the JCOG studies. To participate in the common banking carried out by JCOG-BBJ collaborating biobanks, the subject matter must be reviewed and approved by the ethics committees of each participating facility.

Furthermore, to conduct sample analysis research in future using samples stored in the JCOG-BBJ collaborating biobank, it would be necessary to prepare a “Sample Analysis Research Protocol” and have it reviewed and approved by the JCOG Protocol Review Committee and the ethics committee of facilities involved in the sample analysis.

3. Criteria/definitions used in this study

Tissue classification would be performed according to WHO 2010 classification and ENETS（European Neuroendocrine Tumor Society） classification[14, 15], while disease staging would be done according to “UICC-TNM 7th Edition”.

3.1. Tissue classification (WHO 2010 classification)

The shaded parts are the targets of this study

Neuroendocrine neoplasms

1) Neuroendocrine tumor：NET Grade 1（NETG1）
2) Neuroendocrine tumor：NET Grade 2（NETG2）
3) Neuroendocrine carcinoma：NEC（large cell or small cell type）
4) Mixed adenoendocrine carcinoma（MANEC）
5) Hyperplastic and preneoplastic lesions

3.2. Grade classification （ENETS [European Neuroendocrine Tumor Society] / WHO2010 classification）

Grade 1（G1）	Number of mitotic presentations < 2 per 10 high power fields（HPF）and/or Ki-67 index ≤2%＊
Grade 2（G2）	Number of mitotic presentations 2–20 per 10 HPF and/or Ki-67 index 3-20%
Grade 3（G3）	Number of mitotic presentations > 20 per 10 HPF and/or Ki-67 index >20%

＊The description of number of mitotic presentations in WHO 2010 classification is ≤2, but 2–3% are classified to G1[47].

3.3. Histopathological diagnosis

- Immunostaining (Chromogranin A and synaptophysin) is essential for a pathological diagnosis of NEC.
- Either1. or 2., or both are studied to determine proliferative activity. Number of mitotic presentations and Ki-67 index would adopt a high grade evaluation.
  1. Number of mitotic presentations (to evaluate 50HPF)
  2. Ki-67 index（500–2000 tumor cells*）

  *（Only in this study, if the number of tumor cells in the collected sample is less than 500, the number of tumor cells measured is also listed. A minimum of 100 tumor cells is required.）
- If sufficient amount of biopsy sample cannot be collected for a pathological diagnosis, a cell block prepared using material obtained from EUS-FNA, brushing or needle biopsy may be used. However, the use of a cell block prepared from ascites or pleural effusion is not permitted.
- During a histological diagnosis using a resected tumor sample, a slide with a representative section shall be used to evaluate the proportion of NEC components (differential diagnosis with mixed adenoendocrine carcinoma). For a comprehensive examination, the entire tumor must be evaluated.

For further details NEC Pathological Diagnosis Handbook would be referred (posted on JCOG website).

3.4. Disease stage classification criteria (UICC-TNM 7th Edition)

Special notice regarding disease stage classification: Although there is an independent TNM classification for NET G1 and NET G2 with primary lesions in the stomach, small intestines, and colon; since this study concerns NEC, the disease would be classified according to the classification method of major tissue type in each primary lesion organ (squamous cell carcinoma for esophagus, and adenocarcinoma for all other primary lesion organs). In this study, the classification of intrahepatic cholangiocarcinoma is used for hepatic NEC, due to their similar clinical presentation.

3.4.1. Esophagus

T –Primary tumor

TX： Primary tumor cannot be evaluated
T0： Primary tumor unidentified
Tis： Epithelial carcinoma/highly dysplastic
T1： Tumor invading mucosal lamina propria, muscularis mucosae, or submucosa
T1a：Tumor invading the mucosal lamina propria or muscularis mucosae
T1b： Tumor invading the submucosa

T2 : Tumor invading the muscularis propia
T3 : Tumor invading the adventitia
T4 : Tumor invading the surrounding tissue
T4a : Tumor invading the pleura, pericardium, and diaphragm
T4b : Tumor invading other surrounding tissues such as the aorta, centrum, trachea

N –Regional lymph nodes
NX : Regional lymph node metastasis cannot be evaluated
N0 : No regional lymph node metastasis
N1 : 1–2 foci of regional lymph node metastases
N2 : 3–6 regional foci of lymph node metastases
N3 : 7 or more foci of regional lymph node metastases

M –Distal metastasis
M0 : No distal metastasis
M1 : Distal metastasis present

Stage	T	N	M
**0**	Tis	N0	M0
**IA**	T1	N0	M0
**IB**	T2	N0	M0
**IIA**	T3	N0	M0
**IIB**	T1, T2	N1	M0
**IIIA**	T4a	N0	M0
	T3	N1	M0
	T1, T2	N2	M0
**IIIB**	T3	N2	M0
**IIIC**	T4a	N1、N2	M0
	T4b	Unrelated to N	M0
	Any T	N3	M0
**IV**	Any T	Any N	M1

3.4.2. Stomach

T –Primary tumor
TX : Primary tumor cannot be evaluated
T0 : Primary tumor unidentified
Tis : Epithelial carcinoma: Epithelial carcinoma/highly dysplastic carcinoma not invading the lamina propia mucosa
T1 : Tumor invading mucosal lamina propria, muscularis mucosae, or submucosa
T1a : Tumor invading the mucosal lamina propria or muscularis mucosae
T1b : Tumor invading the submucosa
T2 : Tumor invading the muscularis propia
T3 : Tumor invading the subserosa
T4 : Tumor perforating the serosa, or invading adjacent structures[1,2,3]
T4a : Tumor perforating the serosa
T4b : Tumor invading adjacent structures[1,2,3]

1. Adjacent organs of the stomach are spleen, transverse colon, liver, diaphragm, pancreas, abdominal wall, adrenal glands, kidneys, small intestine, and retroperitoneum.
2. If the invasion has spread from the stomach to the duodenum or esophagus, classification is made in terms of depth.
3. A tumor that advances into the gastrocolic ligament, into the hepatogastric ligament, or into the greater or lesser omentum, and is classified as T3, when there is no perforation of the visceral peritoneum.

N –Regional lymph nodes

NX : Regional lymph node metastasis cannot be evaluated

N0 : No regional lymph node metastasis

N1 : 1–2 regional foci of lymph node metastases

N2 : 3–6 regional foci of lymph node metastases

N3 : 7 or more regional foci of lymph node metastases

N3a : 7–15 regional foci of lymph node metastases

N3b : 16 or more regional foci of lymph node metastases

M –Distal metastasis

M0 : No distal metastasis

M1 : Distal metastasis present

Stage	T	N	M
**0**	Tis	N0	M0
**IA**	T1	N0	M0
**IB**	T2	N0	M0
	T1	N1	M0
**IIA**	T3	N0	M0
	T2	N1	M0
	T1	N2	M0
**IIB**	T4a	N0	M0
	T3	N1	M0
	T2	N2	M0
	T1	N3	M0
**IIIA**	T4a	N1	M0
	T3	N2	M0
	T2	N3	M0
**IIIB**	T4b	N0, N1	M0
	T4a	N2	M0
	T3	N3	M0
**IIIC**	T4a	N3	M0
	T4b	N2, N3	M0
**IV**	Any T	Any N	M1

3.4.3. Small intestines (including duodenum)

T –Primary tumor

TX : Primary tumor cannot be evaluated

T0 : Primary tumor unidentified

Tis : Epithelial carcinoma

T1 : Tumor invading mucosal lamina propria, muscularis mucosae, or submucosa

T1a : Tumor infiltrating the mucosal lamina propria or muscularis mucosae

T1b : Tumor infiltrating the submucosa

T2 : Tumor infiltrating the muscularis propia

T3 : Tumor invading the subserosa, or tumor invading within 2 cm of surrounding tissue of muscularis externa without peritoneal cover (mesenterium, retroperitoneum)*

*Surrounding tissue of muscularis externa without peritoneal cover refers to the mesenterium in the jejunum and ileum, and the retroperitoneum in the duodenum without serosa.

T4 : Tumor penetrating the visceral peritoneum, or tumor directly invading another organ or tissue (invasion of other loops of the small intestine, invasion by 2 cm or more into the mesenterium and retroperitoneum, including invasion into the abdominal wall through the serosa; invasion to the pancreas only for duodenum)

N –Regional lymph nodes

NX : Regional lymph node metastasis cannot be evaluated

N0 : No regional lymph node metastasis

N1 : 1–3 regional foci of lymph node metastases

N2 : 4 or more regional foci of lymph node metastases

M –Distal metastasis

M0 : No distal metastasis

M1 : Distal metastasis present

Stage	T	N	M
**0**	Tis	N0	M0
**I**	T1, T2	N0	M0
**IIA**	T3	N0	M0
**IIB**	T4	N0	M0
**IIIA**	Any T	N1	M0
**IIIB**	Any T	N2	M0
**IV**	Any T	Any N	M1

3.4.4. Appendix (partial revision of UICC-TNM 7th Edition)

T –Primary tumor

TX : Primary tumor cannot be evaluated

T0 : Primary tumor unidentified

Tis : Epithelial carcinoma: Tumor invading epithelium or lamina propia mucosae

T1 : Tumor invading the submucosa

T2 : Tumor invading the muscularis propia

T3 : Tumor invading the subserosa or mesoappendix

T4 : Tumor penetrating the visceral peritoneum, tumor including a peritoneal mucosal tumor in the lower right abdomen and/or tumor directly invading other organs or tissue

T4a : Tumor penetrating the visceral peritoneum, or peritoneal mucosal tumor in the lower right abdomen

T4b : Tumor directly invading other organs or tissues

N –Regional lymph nodes

NX : Regional lymph node metastasis cannot be evaluated

N0 : No regional lymph node metastasis

N1 : 1–3 regional foci of lymph node metastases

N2 : 4 or more foci of regional lymph node metastases

M –Distal metastasis

M0 : No distal metastasis

M1 : Distal metastasis present

M1a : Peritoneal metastasis beyond the lower right abdomen, including pseudomyxoma peritonei

M1b : Distal metastasis other than peritoneal metastasis

Stage	T	N	M
**0**	Tis	N0	M0
**I**	T1, T2	N0	M0
**IIA**	T3	N0	M0
**IIB**	T4a	N0	M0
**IIC**	T4b	N0	M0
**IIIA**	T1, T2	N1	M0
**IIIB**	T3, T4	N1	M0
**IIIC**	Any T	N2	M0
**IVA**	Any T	N0	M1a
**IVB**	Any T	N0	M1a
	Any T	N1, N2	M1a
**IVC**	Any T	Any N	M1b

3.4.5. Colon and rectum

T –Primary tumor

TX: Primary tumor cannot be evaluated

T0: Primary tumor unidentified

Tis: Carcinoma in situ: Tumor invading epithelium or lamina propia mucosae

T1: Tumor invading the submucosa

T2: Tumor invading the muscularis propia

T3: Tumor invading the subserosa, or tissue surrounding the colon or rectum without peritoneal covering

T4: Tumor penetrating the visceral peritoneum, and/or directly invading another organ or tissues

T4a: Tumor penetrating the visceral peritoneum

T4b: Tumor directly invading another organ or tissues

N –Regional lymph nodes

NX: Regional lymph node metastasis cannot be evaluate

N0: No regional lymph node metastasis

N1: 1–3 regional foci of lymph node metastases

N1a: 1 regional lymph node metastasis

N1b: 2–3 regional foci of lymph node metastases

N1c: Presence of tumor deposits, that is, satellite nodes in the subserosa or in the soft tissue surrounding colon or rectum without peritoneal covering, but with no regional lymph node metastasis

N2: 4 or more regional lymph node metastases

N2a: 4–6 regional foci of lymph node metastases

N2b: 7 or more regional foci of lymph node metastases

M –Distal metastasis

M0: No distal metastasis

M1: Distal metastasis present

M1a: Local metastasis to one organ (liver, lungs, ovaries, or lymph nodes other than regional lymph nodes)

M1b: 2 or more organs, or peritoneal metastasis

Stage	T	N	M
**0**	Tis	N0	M0
**I**	T1, T2	N0	M0
**II**	T3, T4	N0	M0
**IIA**	T3	N0	M0
**IIB**	T4a	N0	M0
**IIC**	T4b	N0	M0
**III**	Any T	N1, N2	M0
**IIIA**	T1, T2	N1	M0
	T1	N2a	M0
**IIIB**	T3, T4	N1	M0
	T2, T3	N2a	M0
	T1, T2	N2b	M0
**IIIC**	T4a	N2a	M0
	T3, T4a	N2b	M0
	T4b	N1, N2	M0
**IVA**	Any T	Any N	M1a
**IVB**	Any T	Any N	M1b

3.4.6. Applies to hepatic NEC (hepatic primary lesion or liver metastasis from unknown primary lesion)

T –Primary tumor

TX: Primary tumor cannot be evaluated

T0: Primary tumor unidentified

Tis : Carcinoma *in situ*

T1 : Isolated tumor without vascular invasion

T2a : Isolated tumor with vascular invasion

T2b : Multifocal tumor regardless of vascular invasion

T3 : Tumor penetrating visceral peritoneum or directly invading adjacent extrahepatic structures

T4 : Tumor with bile duct invasion (bile duct proliferative type)

N –Regional lymph nodes

NX : Regional lymph node metastasis cannot be evaluated

N0 : No regional lymph node metastasis

N1 : Regional lymph node metastasis present

M –Distal metastasis

M0 : No distal metastasis

M1 : Distal metastasis present

Stage	T	N	M
**I**	T1	N0	M0
**II**	T2	N0	M0
**III**	T3	N0	M0
**IVA**	T4	N0	M0
	Any T	N1	M0
**IVB**	Any T	Any N	M1

3.4.7. Gall bladder

T –Primary tumor

TX : Primary tumor cannot be evaluated

T0 : Primary tumor unidentified

Tis : Carcinoma *in situ*

T1 : Tumor invading mucosal lamina propria or muscularis externa

T1a : Tumor invading mucosal lamina propria

T1b : Tumor invading muscularis externa

T2 : Tumor invading the connective tissue around the muscularis externa, but shows no progression beyond the serosa or to the liver

T3 : Tumor perforating the serosa (visceral peritoneum), tumor directly advancing to the liver and/or an adjacent organ that is not the liver (stomach, duodenum, colon, pancreas, omentum, or extrahepatic bile duct)

T4 : Tumor invading the main trunk of the portal vein or the hepatic artery, or tumor advancing to two or more adjacent organs which are not the liver

N –Regional lymph nodes

NX : Regional lymph node metastasis cannot be evaluated

N0 : No regional lymph node metastasis

N1 : Regional lymph node metastasis present (cystic duct, common bile duct, proper hepatic artery, including lymph nodes along the portal vein)

M –Distal metastasis

M0 : No distal metastasis

M1 : Distal metastasis present

Stage	T	N	M
**0**	Tis	N0	M0
**I**	T1	N0	M0
**II**	T2	N0	M0
**IIIA**	T3	N0	M0
**IIIB**	T1, T2, T3	N1	M0
**IVA**	T4	Any N	M0

**IVB**	Any T	Any N	M1

3.4.8. Extrahepatic bile duct-hepatic portal region

T –Primary tumor

TX: Primary tumor cannot be evaluated

T0: Primary tumor unidentified

Tis: Carcinoma *in situ*

T1: Tumor localized to the bile duct which advances until muscularis externa or fibrous tissue

T2a: Tumor invading beyond the bile duct wall and into the surrounding adipose tissue

T2b: Tumor invading the adjacent liver parenchyma

T3: Tumor invading the branch of one side of portal vein or hepatic artery

T4: Tumor invades the main trunk of portal vein, and branches on both sides of the portal vein, proper hepatic artery or the secondary branches of the bile duct on both left and right side, or to the secondary bile duct branches on one side and portal vein or hepatic artery on the other side

N –Regional lymph nodes

NX: Regional lymph node metastasis cannot be evaluated

N0: No regional lymph node metastasis

N1: Presence of regional lymph node metastasis in the cystic duct, common bile duct, proper hepatic artery, and lymph nodes along portal vein

M –Distal metastasis

M0: No distal metastasis

M1: Distal metastasis present

Stage	T	N	M
**0**	Tis	N0	M0
**I**	T1	N0	M0
**II**	T2a, T2b	N0	M0
**IIIA**	T3	N0	M0
**IIIB**	T1,T2, T3	N1	M0
**IVA**	T4	N0, N1	M0
**IVB**	Any T	Any N	M1

3.4.9. Extrahepatic bile duct-distal

T –Primary tumor

TX: Primary tumor cannot be evaluated

T0: Primary tumor unidentified

Tis: Carcinoma *in situ*

T1: Tumor localized in the bile duct wall

T2: Tumor invading beyond the bile duct wall

T3: Tumor invading the gallbladder, liver, pancreas, duodenum, or other adjacent organs

T4: Tumor invading the celiac axis or superior mesenteric artery

N –Regional lymph nodes

NX: Regional lymph node metastasis cannot be evaluated

N0: No regional lymph node metastasis

N1: Regional lymph node metastasis present

M –Distal metastasis

M0: No distal metastasis

M1: Distal metastasis present

Stage	T	N	M
**0**	Tis	N0	M0
**IA**	T1	N0	M0
**IB**	T2	N0	M0

**IIA**	T3	N0	M0
**IIB**	T1, T2, T3	N1	M0
**III**	T4	Any N	M0
**IV**	Any T	Any N	M1

3.4.10. Ampulla of Vater

T –Primary tumor

TX: Primary tumor cannot be evaluated

T0: Primary tumor unidentified

Tis: Carcinoma *in situ*

T1: Tumor localized to the ampulla of Vater, or sphincter of Oddi

T2: Tumor invading the duodenal wall

T3: Tumor invading the pancreas

T4: Tumor invading the soft tissue around the pancreas or other adjacent organs

N –Regional lymph nodes

NX: Regional lymph node metastasis cannot be evaluated

N0: No regional lymph node metastasis

N1: Regional lymph node metastasis present

M –Distal metastasis

M0: No distal metastasis

M1: Distal metastasis present

Stage	T	N	M
**0**	Tis	N0	M0
**IA**	T1	N0	M0
**IB**	T2	N0	M0
**IIA**	T3	N0	M0
**IIB**	T1, T2, T3	N1	M0
**III**	T4	Any N	M0
**IV**	Any T	Any N	M1

3.4.11. Pancreas

T –Primary tumor

TX: Primary tumor cannot be evaluated

T0: Primary tumor unidentified

Tis: Carcinoma *in situ*

T1: Tumor localized within the pancreas, with a maximum size of < 2 cm

T2: Tumor localized within the pancreas, with a maximum size > cm

T3: Tumor advancing outside the pancreas, but with no invasion to the celiac axis or superior mesenteric artery

T4: Tumor invading the celiac axis or superior mesenteric artery

N –Regional lymph nodes

NX: Regional lymph node metastasis cannot be evaluated

N0: No regional lymph node metastasis

N1: Regional lymph node metastasis present

M –Distal metastasis

M0: No distal metastasis

M1: Distal metastasis present

Stage	T	N	M
**0**	Tis	N0	M0
**IA**	T1	N0	M0
**IB**	T2	N0	M0
**IIA**	T3	N0	M0

**IIB**	T1, T2, T3	N1	M0
**III**	T4	Any N	M0
**IV**	Any T	Any N	M1

3.5. Residual tumor (R) classification (UICC-TNM 7th Edition)

RX : Presence of residual tumor cannot be evaluated

R0 : No residual tumor

R1 : Presence of residual tumor by microscopic examination

R2 : Presence of residual tumor by macroscopic examination

3.6. Definition of non-resectable NEC

Unoperated cases for which surgery as a curative process was not indicated based on clinical findings, including image-based diagnosis, or operated cases that underwent resection (including experimental laparotomy) but ultimately ended as R2 resection are considered non-resectable.

The details by organs are shown below. One or more criterion must be fulfilled for each organ.

<Esopghagus>

- Diagnose as Stage IV (UICC 7th edition) based on clinical findings.

<Stomach>

- Diagnose as Stage IV (UICC 7th edition) based on clinical or surgical findings. However, this does not include situations where Stage IV diagnosis is provided based only on peritoneal lavage cytology (CY1).
- Bulky lymph node* metastasis is identified.

  *Bulky lymph node: Two or more lymph nodes with a major axis of 1.5 cm or more, that are present in contact with each other around the celiac artery, the common hepatic artery, the splenic artery, and the proper hepatic artery, or on the front surface of the superior mesenteric vein, where single or multiple large and small lymph nodes have formed an aggregate with the overall major axis length of 3.0 cm or more.

<Duodenum (excluding the ampulla of Vater), small intestines, appendix, and colon>

- Diagnosed as Stage IV (UICC 7th edition) based on the clinical findings.

<Extrahepatic bile duct, ampulla of Vater, gallbladder>

- Distant metastasis is identified
- Para-aortic lymph node metastasis is identified
- Bulky metastasis identified in the hepatoduodenal mesentery and lymph nodes surrounding the head of the pancreas
- Invasion in the proper hepatic artery, common hepatic artery, celiac artery, or the superior mesenteric artery identified
- Invasion in the hepatic artery branches on both the left and right side identified
- Extensive invasion or occlusion of the main trunk of portal vein, or invasion in the portal vein branches on both the left and right side identified
- Invasion in the blood vessels on the lobe on one side of the liver (portal vein or artery) or atrophy of lobe on one side of liver identified, and advancement into the bile duct on the other side is identified up to the secondary branch level
- Advancement into the bile ducts on both sides identified up to the secondary branch level

<Pancreas>

- Diagnosed as Stage III or Stage IV (UICC 7th edition) based on clinical findings.

＜Hepatic NEC (hepatic primary lesion or liver metastasis from unknown primary lesion)>

- Distal metastasis* other than intrahepatic metastasis identified.

  *Limited to organs that cannot be the primary lesion site (bone, lymph nodes below the diaphragm,

peritoneum, subcutaneous, muscle, and spleen) (see 3.8)

- Para-aortic lymph node metastasis is identified
- Bulky metastasis identified in the hepatoduodenal mesentery and lymph nodes surrounding the head of the pancreas
- Multiple masses identified in the liver.
- Invasion in one of the proper hepatic artery, common hepatic artery, celiac artery, or the superior mesenteric artery identified.
- Invasion to hepatic artery branches on both the left and right side identified
- Extensive invasion or occlusion of the main trunk of portal vein, or invasion in portal vein branches on both the left and right side identified
- Invasion in blood vessels on lobe on one side of the liver (portal vein or artery) or atrophy of lobe on one side of liver identified, and advancement into the bile duct on the other side is identified up to the secondary branch level
- Advancement into the bile ducts on both sides identified up to the secondary branch level

3.7. Definition of recurrent NEC

If surgery (R0 resection or R1 resection) was performed for gastrointestinal/hepatobiliary or pancreatic primary lesion, malignant tumor and the condition was diagnosed as NEC based on pathological samples taken from surgery, and were deemed as a clinical relapse thereafter.

If there are past histological samples from the primary lesion, no histological biopsy of the relapse lesion is necessary.

3.8. Definition of hepatic NEC (hepatic primary lesion or liver metastasis from an unknown primary lesion)

After a detailed investigation into the primary lesion by cervical-pelvic contrast CT, gastrointestinal endoscopy, FDG-PET, otolaryngology (head and neck examination), urology examination (for men only) and gynecological examination (for women only), if the tumor is in one of the following sites, the case is defined as hepatic primary lesion (or primary lesion unknown).

- Tumor is present only in the liver
- Tumor is present in an organ that cannot be a primary lesion site (bone, lymph nodes below the diaphragm, peritoneum, subcutaneous, muscle, and spleen), and in the liver.

4. Patient selection criteria

Patients that fulfill all of the following inclusion criteria and do not correspond to any of the exclusion criteria are eligible for enrollment.

4.1. Inclusion criteria (for enrollment)

1) Any of the following is applicable based on pathological diagnosis taking findings of immunohistochemistry into consideration (see 3.1. to 3.3.).

[1] Pathologically diagnosed as neuroendocrine carcinoma (NEC*1) in the resected sample.

[2] Containing pathologically confirmed component of neuroendocrine carcinoma (NEC*1) in the biopsy sample.

1: Based on WHO 2010 classification

2) Any of the following is applicable

[1] NEC arise in esophagus, stomach, duodenum, intestine, appendix, colon, rectum, gallbladder, intrahepatic bile duct, extrahepatic bile duct, ampulla of Vater, pancreas,

[2] Liver NEC (primary liver or liver metastasis of unknown primary) *2.

*2: The tumor is only in one of the following sites after a thorough examination of the primary site by contrast CT (from the neck to pelvic) and upper/lower gastrointestinal endoscopy, FDG-PET scan, otolaryngology (head and neck) examination, urology examination (male patients only), and gynecology examination (female patients only).

a. Liver only

b. Bone, lymph nodes below the diaphragm, peritoneum, subcutaneous, muscle, spleen, and liver

3) Unresectable (see 3.6) or recurrent cancer (see 3.7). It is not essential for a pathological confirmation of the metastatic lesion or recurrent site. Cases of esophageal NEC is ineligible if corresponding to any of the following.

[1] cT4.

[2] No distant metastasis rather than supraclavicular lymph node

[3] Stenosis indicated for palliative radiotherapy

4) No previous chemotherapy or radiotherapy for NEC. Pre- or post-operative chemotherapy except irinotecan or etoposide for NEC is allowed as long as it was completed at least 8 weeks prior to registration.

5) No previous chemotherapy using platinum agents for any malignancies.

6) Aged 20 to 75 years old.

7) ECOG performance status of 0 or 1.

8) Measurable region is not required.

9) Adequate organ functions.

[1] WBC ≥3,000/ mm^3

[2] Neutrophils ≥ 1,500/ mm^3

[3] Hemoglobin ≥ 9.0 g/dL

[4] Platelets ≥ 10×10^4/ mm^3

[5] Total bilirubin≤ 1.5 mg/dL※4

[6] AST(sGOT)≦100 IU/L※4（for hepatic NEC and liver metastasis, ≦150 IU/L）

[7] ALT(sGPT)≦100 IU/L※4（for hepatic NEC and liver metastasis, ≦150 IU/L）

※4：Presence or absence of biliary drainage is not relevant

[8] Serum creatinine≦ 1.3 mg/dL

[9] Creatinine clearance※5≥ 60 mL/min

※5： Creatinine clearance must have been estimated using the Cockcroft-Gault formula, and must be 60 mL/min or more. If the estimation is less than 60 mL/min, but the actual measurement is 60 mL/min or more, the patient can be deemed eligible.

Cockcroft-Gault formula

Male：Ccr ={(140－age) × body weight (kg)}/{72×serum creatinine (mg/dL)}

Female：Ccr = 0.85×{(140－age) × body weight (kg)}/{72×serum creatinine(mg/dL)}

10) Written informed consent.

4.2. Exclusion criteria

1) Synchronous or metachronous (within 5 years) malignancies except carcinoma in situ or intramucosal tumor curatively treated with local therapy.

2) Active infection requiring systemic therapy.

3) Fever of 38 degrees Celsius or higher.

4) Pregnant or lactating women, women of childbearing potential, or women within 28 days after delivery.

5) Psychiatric disease.

6) Patients requiring systemic steroids medication.

7) Interstitial pneumonia, pulmonary fibrosis.

8) Serious co-existing illness.

9) Unstable angina pectoris within 3 weeks, or with a history of myocardial infarction within 6 months.

10) Impossible to use both iodine and gadolinium due to being allergic to contrast agent.

11) Uncontrolled diabetes mellitus or routine administration of insulin.

5. Registration and randomization

5.1. Procedure of registration

Ensure that a patient to be registered meets all eligibility criteria and does not meet any of exclusion criteria and register the patient by using JCOG Web Entry System. JCOG Web System Personal Account and password are required for web registration. If unknown, contact JCOG Data Center.

Patient registration JCOG Web Entry System
URL:https://secure.jcog.jp/dc/
(Web registration can be used for 24 hours.)

Contact information for patient registration and JCOG Web Entry System

JCOG Data Center
TEL: 03-3542-3373
Weekdays 9:00-17:00 (not available in holidays, Saturdays and Sundays, New Year's holidays)
E-mail:JCOGdata@ml.jcog.jp

Contact information on Patients Selection Criteria

Study Coordinator Contact : Chigusa Morizane
Hepato-Biliary Pancreatic Group: Futomi Mori (Main Research Secretariat)
Department of Hepatobiliary and Pancreatic Medicine, National Cancer Center
TEL:03-3542-2511
FAX:03-3542-3815
E-mail:cmorizan@ncc.go.jp

Gastric Cancer Group: Yoshinori Machida
Shizuoka Cancer Center
〒411-8777 1007 Shimonagakubo, Nagaizumi-cho, Sunto-gun, Shizuoka Prefecture
TEL:+81-55-989-5222
FAX:+81-55-989-5631
E-mail：no.machida@scchr.jp

Esophageal Cancer Group: Ken Kato/Yushi Homma
Department of Gastrointestinal Oncology, National Cancer Center
TEL: 03-3542-2511
FAX: 03-3542-3815
E-mail: kenkato@ncc.go.jp / yohonma@ncc.go.jp

5.1.1. Precautions for patient registration

① Registration after initiation of protocol treatment is unacceptable.
② Registration is performed by accessing the URL in '5.1. Procedure of registration'.
③ Eligibility checks are performed on the screen of Registration Form, so it is not necessary to send a Registration Form to Data Center by mail or fax.
④ If input data are insufficient, registration is not accepted until all are met.
⑤ The registration number is issued after the confirmation of eligibility on the registration screen, then the registration is completed.
⑥ Once registered, patients will not be retracted (retracted from the database) unless there is withdrawal of consent, including refusal to use the data for research. For duplicate registration, the information at the initial registration (registration number, allocated arm) are used in any case.
⑦ When misregistration or duplicate registration is found, contact Data Center immediately.
⑧ Body surface area and drug dose calculations are institutional responsibilities, and the body surface area

and drug dose displayed on Web Entry System at registration are only for double-checking. Those should always be calculated and checked at the institution. When the body surface area calculation formula adopted in the hospital information system of the institution differs from calculation formula adopted by JCOG (Dubois formula: Body surface area (m^2) = Body weight (kg) $^{0.425}$ x Height (cm) $^{0.725}$ 84 ÷ 10,000), there can be a difference in the dose by the hospital information system of the institution and the dose by the calculation formula adopted by JCOG, but in that case, either dosage used is decided by the site investigator.

5.2. Randomization and allocation adjustment factor

During enrollment, the treatment arm for a patient is allocated randomly by the data center.

Randomization would use a method of minimization using the 1) institutions, 2) Primary lesion organ (gastrointestinal tract [esophagus, stomach, duodenum, small intestines, appendix, colon, and rectum] vs. hepatobiliary and pancreatic organ [hepatic NEC, gallbladder, extrahepatic bile duct, ampulla of Vater, or pancreas]) as the adjustment factors, so that there are no large discrepancies between them. Researchers at participating facilities would not be informed of the detailed procedures of randomization.

6. Treatment Plan and Treatment Modification Criteria

Unless patient safety is threatened, treatment and treatment modifications is done in compliance with the specifications in this chapter.

If it is considered that the protocol specification may cause medically dangerous situation of the patient, treatment modifications should be made according to the medical judgment of the investigators/sub-investigators. Such protocol deviation is considered to be "clinically relevant deviation" if considered medically appropriate (see 14.1.4.Protocol deviation/violation). Deviations that occur with intentions other than safety, such as increasing efficacy, are not considered clinically relevant deviations.

6.1. Protocol treatment

Protocol treatment is initiated within 7 days of enrollment.

If treatment initiation occurs after 8 days from registration for any reason, the reason should be documented on the Treatment Course Form. If it is determined that treatment cannot be initiated, describe the details in the Off-treatment Form as Protocol Treatment Termination.

When laboratory parameters worsen and eligibility criteria are no longer met by the start of treatment after registration, the investigator/sub-investigator is allowed to decide whether initiate or terminate protocol treatment at their own discretion.

「6.3. The course initiation criteria is not applied at treatment changes.

Drugs used

- Etoposide ※
- Cisplatin ※
- Irinotecan ※
- Entecavir, tenofovir disoproxil fumarate, tenofovir alafenamide fumarate

The use of generic drugs is not restricted.

※ The company that manufactures or distributes these drugs, or intends to manufacture or sell these drugs, requires conflicts of interest control in the Clinical Trials Act as a company involved in this study (see 13.8.).

6.1.1. Arm A: Etoposide plus cisplatin (EP) therapy

The following chemotherapy courses will be given once a week for 3 weeks and treatment will be continued until the patient meets the discontinuation criteria.

Drug	Dosage(mg/m^2)	Dosing regimen/Dosing time	Dose day
Etoposide	100	IV/60-120 min	Day 1、2、3
Cisplatin	80	IV/60-120 min	Day 1

1) Calculation of the dosage

① Body surface area is calculated by determining drug dose to the third decimal point.

② For both etoposide and cisplatin, the calculated dose is determined by truncating the decimal point. For drug doses, ±10% is the acceptable range.

③ Dose recalculation due to body weight change after treatment initiation is not performed.

2) Administration of anticancer drugs

Etoposide and cisplatin are given in any order.

Examples of administration in the package insert are described below.

(i) Before administration: Before administration of anticancer drugs, 1,000-2,000 mL of hydration is done to achieve adequate diuresis.

(ii) Etoposide: Etoposide is mixed with infusion solutions such as isotonic sodium chloride solution of 250 mL or more, and infused intravenously in about 60-120 minutes. DEHP (2-ethylhexyl) phthalate: di-(2-ethylhexyl) phthalate), which is a plasticizer, elutes from polyvinyl chloride infusion sets and catheters. Avoid the use of polyvinyl chloride infusion sets and catheters.

(iii) Cisplatin administration: Cisplatin is mixed with stock solution or 500 mL of saline (or glucose-saline) and given intravenously in 60-120 min. During the administration, caution should be exercised in ensuring urine volume, and diuretics such as mannitol and furosemide should be administered as

necessary.

(iv) After administration: After administration of cisplatin, 1,000-2,000 mL of hydration should be done so that adequate diuresis can be achieved.

In addition, short hydration administration at the discretion of each institution is permitted. (see Table 6.1.1. for treatment cases).

Table 6.1.1. Example of short hydration

	Dose	Time
5HT3 antagonist<br>Dexamethasone<br>Physiological saline	9.9 mg<br>50 mL	15<br>min
Etoposide<br>Physiological saline	100 mg/m^2<br>250 mL	60<br>min
No. 1 solution<br>Potassium chloride<br>Magnesium sulfate	500 mL<br>10 mEq<br>8 mEq	60<br>min
20% mannitol	200 mL	30<br>min
Cisplatin<br>Physiological saline	80 mg/m^2<br>250 mL	60<br>min
No. 1 solution<br>Potassium chloride	500 mL<br>10 mEq	60<br>min

(Referred from Horinouchi H, et al., Japan Society of Clinical Oncology 2012

3) Precautions for treatment

JCOG9511, an upfront trial in small-cell lung cancer, suggested an association between treatment-related deaths and first-course neutropenia. Therefore, caution should be exercised when neutropenia is strongly observed from the first course.

6.1.2. Arm B: Irinotecan plus cisplatin (IP) therapy

One 4-week course of the following chemotherapy will be continued until the discontinuation criteria are met. However, if day 15 irinotecan is skipped, 3 weeks should be used as one course, and the next course should be started with day 22.

Drug	Dosage(mg/m^2)	Dosing regimen/Dosing time	Dose day
Irinotecan	60	IV/90 min	Day 1、8、15
Cisplatin	60	IV/60-120 min	Day 1

1) Calculation of the dosage

(i) Body surface area is determined by determining the dose of drug administered until the third decimal point.

(ii) The dose is calculated for both irinotecan and cisplatin by truncating the decimal point. For drug doses, ±10% is the acceptable range.

(iii) Dose recalculation due to body weight change after treatment initiation is not performed.

2) Administration of anticancer drugs

Irinotecan and cisplatin are given in any order.

Examples of administration in the package insert are described below.

(i) Before administration: Before administration of anticancer drugs, 1,000-2,000 mL of hydration is done to achieve adequate diuresis.

(ii) Irinotecan: Irinotecan is mixed with 500 mL or more of isotonic sodium chloride solution, glucose solution, or electrolyte maintenance solution and given by intravenous drip infusion at about 90 minutes.

(iii) Cisplatin administration: Cisplatin is mixed with stock solution or 500 mL of saline (or glucose-saline) and given intravenously in 60-120 min. During the administration, caution should be exercised in ensuring urine volume, and diuretics such as mannitol and furosemide should be administered as necessary.

(iv) After administration: After administration of cisplatin, 1,000-2,000 mL of hydration should be done so that adequate diuresis can be achieved.

In addition, short hydration administration at the discretion of each institution is permitted. (See Table 6.1.1.

3) Precautions for treatment

JCOG9511, an upfront trial in small-cell lung cancer, suggested an association between treatment-related deaths and first-course neutropenia. Therefore, caution should be exercised when neutropenia is strongly observed from the first course.

6.2. Protocol Treatment Termination/Completion Criteria

6.2.1. Definition of protocol treatment completion

Protocol treatment is continued in this study unless the protocol treatment discontinuation criteria are met, so no definition of protocol treatment completion is provided.

6.2.2. Criteria for termination of protocol treatment

Protocol treatment is terminated in any of the following cases:

1) Judged as protocol treatment is ineffective
   - When a definite tumor exacerbation is confirmed by imaging or clinically
     ※ Protocol treatment should not be discontinued if the clinical judgement of PD based on the assessment of response based on imaging indicates that continuation of protocol treatment is appropriate, and protocol treatment should be continued.
2) Protocol treatment cannot be continued due to adverse events
   ① If Grade 4 non-hematological toxicity is observed (with the exception of the following adverse events) 'hypernatremia', 'hyponatremia', 'hyperkalemia', 'hypokalemia', 'hyperglycemia', 'hypoglycemia', 'alkaline phosphatase increase', 'alanine aminotransferase increase', 'aspartate aminotransferase increase', 'blood bilirubin increase','serum amylase increase', 'lipase increase', 'GGT increase'.
   (*Adverse events other than "anaemia," "myeloid cytopenia," "lymphocyte count decreased," "neutrophil count decreased," "white blood cell count decreased," "platelet count decreased," "CD4 lymphopenia" in CTCAE v4 0)
   ② If the next course cannot be initiated beyond 21 days from the expected start date of the course due to an adverse event
   ③ When the criteria for terminating protocol treatment in the treatment modification criteria (6.3.) are met.
   ④ Adverse events other than the treatment modification criteria that the investigator/sub-investigator judges to require termination of protocol treatment
3) If the patient offers termination of protocol treatment for reasons not denied to be associated with the adverse event
   - This category should be used if an association with an adverse event cannot be ruled out.
4) When the patient offers termination of protocol treatment because of reasons for denial of association with adverse events
   - Patient refusal after enrollment and before initiation of protocol treatment
   - When the association with an adverse event can first be denied, such as the relocation of the person or household member during protocol treatment.
5) Death during protocol treatment
   - Death before deciding to terminate protocol treatment for other reasons
6) Palliative surgery for pathogenic adverse events or surgery for patients with complete response to chemotherapy (see Section 6.3.9).
7) In addition, exacerbations before the start of treatment after enrollment (protocol treatment could not be initiated due to rapid exacerbation), protocol violations were found, ineligibility was determined due to modifications in pathological diagnosis after enrollment, etc., treatment was changed, and it was judged that it was difficult to continue protocol treatment due to social reasons and safety management problems, etc.

The date of discontinuation of protocol treatment is defined as the date of death in 6.2.2.5), the date of surgery in 6.2.2.6), and otherwise, the date on which the treating physician judges that protocol treatment is discontinued.

In this study, non-NEC may be diagnosed by central pathological diagnosis during protocol treatment, because central pathological diagnosis is performed. If the patient is continuing on protocol treatment when the results of the central pathology diagnosis are reported to the registry, the attending physician and the institutional pathologist will review the results and take a clinically appropriate response. Protocol treatment is discontinued if discontinuation of protocol treatment is judged to be appropriate, and the reason for discontinuation is other.

6.3. Treatment modification criteria

The following terms shall be used for the treatment modification.

Delay: Delay the start of the course or administration of treatment from the planned date.

Termination: Discontinuation of a part of or all of the treatment without restarting.

Suspending: temporary interruptions or withdrawals that may be resumed if conditions are met

Skip: Do not administer one or more drugs and proceed to the next schedule.

Categories of infection (CTCAEv4.0) used in this study are as follows

Infection: CTCAEv4.0 infections and infestations

Bronchial infection; pulmonary infection; upper respiratory tract infection; mediastinal infection; pleural infection; catheter-related infection; Biliary tract infection; Gallbladder infection; urinary tract infection

6.3.1. Arm A (EP-therapy): Dose level

1) Etoposide

Drug	Dose level	Dosing schedule	Dose day
Etoposide	Level 0	100 mg/m^2 div	Day 1、2、3
	Level -1	80 mg/m^2 div	Day 1、2、3
	Level -2	60 mg/m^2 div	Day 1、2、3

2) Cisplatin

Drug	Dose level	Dosing schedule	Dose day
Cisplatin	Level 0	80 mg/m^2 div	Day 1
	Level -1	60 mg/m^2 div	Day 1
	Level -2	40 mg/m^2 div	Day 1

6.3.2. A arm (EP therapy): Course initiation criteria

- Initiate the course after confirming that all of the following "Course Initiation Criteria" are met on the scheduled start date of the course or the day before the plan start date of the course.
- If any one is not met, the initiation of the course is delayed.
- If the course cannot be initiated within 21 days of the expected start date of the course (if the start date of the previous course was day 1 and the course could not be initiated by day 42), discontinue the protocol treatment.
- The course initiation criteria is not applied at the start of the first course.

Course initiation criteria

① Neutrophil count Grade 0-1 (≧1500 per mm^3).

② Platelet count ≧10×10^4 /mm^3

③ AST≦100 IU/L (in the presence of hepatic metastases vs. hepatic NECs) was ≦200 IU/L).

④ ALT≦100 IU/L (≦200 IU/L for liver metastases vs liver NECs).

⑤ Total bilirubin <= 2.0 mg/dL

⑥ Serum creatinine <= 1.5 mg/dL

⑦ Fever Grade 0 (axillary temperature, no antipyretic)

⑧ Constipation, fatigue, phlebitis, oral mucositis, and infection ※1 are all Grade 2 or less.
Infected ※1: bronchial infection, pulmonary infection, upper respiratory tract infection, mediastinal infection, pleural infection, catheter-related infection, biliary tract infection, gallbladder infection, urinary tract infection

⑨ Anorexia, nausea, and emesis are all Grade 0-1.

⑩ Diarrhoeal Grade 0

6.3.3. Arm A (EP therapy): Dose reduction criteria

If any of the following toxicities are identified during the course, dose reduction should be performed in accordance with the dose reduction criteria (Table 6.3.3.) from the following course (no dose reduction in the course).

However, even if two or more items are met, the dose reduction for each drug is only one step. Re-escalation after dose reduction is not performed. Protocol treatment is terminated if the dose reduction criteria are met again after the dose reduction to Level-2.

Table 6.3.3. Arm A (EP therapy): Dose reduction criteria

Item	Etoposide	Cisplatin
Neutrophil count Grade 4 (<500 per mm^3).	Reduce the level by 1	No change
Platelet count Grade 4 (<2.5×10^4/mm^3)	Reduce the level by 1	No change
1.5 <serum creatinine ≦2.0 mg/dL.	No change	Reduce the level by 1
Serum creatinine <= >2.0 mg/dL	Termination of protocol treatment	Termination of protocol treatment
Grade 3<br>Infected ※1	Reduce the level by 1	Reduce the level by 1
Grade 2<br>Peripheral sensory neuropathy<br>Peripheral motor neuropathy,<br>Myalgia; arthralgia; tinnitus; hearing impairment	No change	Reduce the level by 1
Grade 3<br>Peripheral sensory neuropathy<br>Peripheral motor neuropathy,<br>Myalgia; arthralgia; tinnitus; hearing impairment	Termination of protocol treatment	Termination of protocol treatment
Non-hematologic toxicities of Grade 3 other than those listed above in ※2 that are causally related to EP-therapy (excluding hyponatremia, hypokalemia, hypomagnesemia, hypocalcemia, hyperglycemia, and weight loss)	Reduce the level by 1	Reduce the level by 1

※1:※1 of infection: bronchial infection, pulmonary infection, upper respiratory tract infection, mediastinal infection, pleural infection, catheter-related infection,
Biliary tract infection; Gallbladder infection; Urinary tract infection

※2:Causal relationship is judged as either of possible, probable, definite

6.3.4. Arm A (EP therapy): Within-course pause, skipping criteria

Following initiation of treatment with each course after the course initiation criteria are met, if any of the following adverse events are observed, day 2, day 3 etoposide will be suspended.

- Fever (axillary temperature) Grade 1-3
- Grade 3 of infection (bronchial infection, pulmonary infection, upper respiratory tract infection, mediastinal infection, pleural infection, catheter-related infection,
  Biliary tract infection, gallbladder infection, urinary tract infection)

Resting etoposide should be resumed after confirming that all initiation criteria are met until day 7. However, it should not be administered after day 8.

That is, if etoposide could not be administered by day 7, the remaining etoposide should be skipped.

If the above suspension and/or skip occur, the next course of etoposide should be started day 22 (after 3 weeks) counting from day 1 of the previous course if the ininiation criteria are met.

6.3.5. Arm B (IP therapy): Dose-level

1) Irinotecan

Drug	Dose level	Dosing schedule	Dose day
Irinotecan	Level 0	60 mg/m^2 div	Day 1、8、15
	Level -1	50 mg/m^2 div	Day 1、8、15
	Level -2	40 mg/m^2 div	Day 1、8、15

2) Cisplatin

Drug	Dose level	Dosing schedule	Dose day
Cisplatin	Level 0	60 mg/m^2 div	Day 1
	Level -1	50 mg/m^2 div	Day 1
	Level -2	40 mg/m^2 div	Day 1

6.3.6. Arm B (IP therapy): Course initiation criteria

- On the day of the initiation of the course or the day before the expected start of the course, start the course after confirming that all of the following "Course Initiation Criteria" are met.
- If any one is not met, the initiation of the course is delayed.
- If the course cannot be initiated within 21 days of the expected start date of the course (if the start date of the previous course was day 1 and the course could not be initiated by day 49), discontinue the protocol treatment.
- However, if day 15 irinotecan is skipped in the previous course, day 22 of the previous course is set as the scheduled start date of the next course regarded as one course per 3 weeks.
- The course initiation criteria is not applied at the start of the first course.

Course initiation criteria

① Neutrophil count Grade 0-1 (≧1500/mm^3).
② Platelet count ≧10×10^4 /mm^3
③ AST≦100 IU/L (≦200 IU/L for liver metastases versus liver NECs)).
④ ALT≦100 IU/L (≦200 IU/L for liver metastases vs liver NECs).
⑤ Total bilirubin <= 2.0 mg/dL
⑥ Serum creatinine <= 1.5 mg/dL
⑦ Fever Grade 0 (measured by axillary temperature, temperature <38°C without antipyretic use)
⑧ Constipation, fatigue, phlebitis, oral mucositis, and infection ※1 are all Grade 2 or less.
Infected ※1: bronchial infection, pulmonary infection, upper respiratory tract infection, mediastinal infection, pleural infection, catheter-related infection, biliary tract infection, gallbladder infection, urinary tract infection
⑨ Anorexia, nausea, and emesis are all Grade 0-1.
⑩ Diarrhoea Grade 0

6.3.7. Arm B (IP-therapy): Dosing criteria for day 8, day 15

After confirming that all of the following ①-③ are met, the second (day 8) or third (day 15) dose of irinotecan is administered. If day 8, day 15 dosing criteria are not met, skip day 8, day 15 dosing.

① All of the following are met with the most recent laboratory data on the scheduled day of administration or the day before the scheduled day of administration.
i) Neutrophil count Grade 0-2 (≧1000 per mm^3).
ii) Platelet count ≧10×10^4 /mm^3
iii) AST ≦ 100 IU/L (≦200 IU/L for liver metastases vs liver NECs).
iv) ALT ≦ 100 IU/L (≦200 IU/L in the presence of hepatic metastases versus hepatic NECs).
v) Total bilirubin <= 2.0 mg/dL
vi) Serum creatinine <= 2.0 mg/dL

② All of the following are met on the scheduled day of administration:
i) Fever Grade 0 (measured by axillary temperature, temperature <38°C without antipyretic use)

ii） Diarrhoeal Grade 0

③ Constipation, anorexia, nausea, emesis, fatigue, phlebitis, oral mucositis, and infection ※1 are all Grade 2 or less.

Infected ※1: bronchial infection, pulmonary infection, upper respiratory tract infection, mediastinal infection, pleural infection, catheter-related infection, biliary tract infection, gallbladder infection, urinary tract infection

6.3.8. Arm B (IP therapy): Dose reduction criteria

If any of the following toxicities are identified during the course, dose reduction should be performed in accordance with the dose reduction criteria (Table 6.3.8.) from the following course (no dose reduction in the course).

However, even if two or more items are met, the dose reduction for each drug is only one step. Re-escalation after dose reduction is not performed. Protocol treatment is terminated if the dose reduction criteria are met again after the dose reduction to Level-2.

Table 6.3.8. Arm B (IP therapy): Dose reduction criteria

Item	Irinotecan	Cisplatin
Neutrophil count Grade 4 (<500 per mm^3).	Reduce the level by 1	No change
Platelet count Grade 4 (< 2.5×10^4/mm^3)	Reduce the level by 1	No change
1.5 <serum creatinine ≦2.0 mg/dL.	No change	Reduce the level by 1
Serum creatinine <= >2.0 mg/dL	Termination of protocol treatment	Termination of protocol treatment
Grade 3<br>Infected ※1	Reduce the level by 1	Reduce the level by 1
Grade2<br>Peripheral sensory neuropathy<br>Peripheral motor neuropathy,<br>Myalgia; arthralgia; tinnitus; hearing impairment	No change	Reduce the level by 1
Grade 3<br>Peripheral sensory neuropathy<br>Peripheral motor neuropathy,<br>Myalgia; arthralgia; tinnitus; hearing impairment	Termination of protocol treatment	Termination of protocol treatment
Non-hematologic toxicities of Grade 3 other than those listed above in ※2 that are causally related to IP-therapy (excluding hyponatremia, hypokalemia, hypomagnesemia, hypocalcemia, hyperglycemia, and weight loss)	Reduce the level by 1	Reduce the level by 1

※1:※1 of infection: bronchial infection, pulmonary infection, upper respiratory tract infection, mediastinal infection, pleural infection, catheter-related infection,

Biliary tract infection; Gallbladder infection; Urinary tract infection

※2:Causal relationship is judged as either of possible, probable, definite

6.3.9. Surgical of after end of chemotherapy

1) Surgery for Adverse Events Associated with Pathogenesis

Surgery may be performed if it is judged clinically desirable to perform surgery for newly appearing symptoms such as hemorrhage or stenosis. Protocol treatment is discontinued if surgery is performed, regardless of the content. In this case, the date of discontinuation of protocol treatment is the date of surgery.

2) Surgery for patients with complete response to chemotherapy

- When chemotherapy is highly effective, all metastases present at the time of enrollment on imaging studies disappear, and curative resection (R0 resection) is considered possible, resection including primary and metastatic disease may be performed.
- Surgical procedures are not specifically specified, but the Research Office will collect information on the details of the surgery performed individually in order to be reviewed by the research representative/research office.
- In all surgical cases, the group group conference shall confirm the validity of the judgment that surgery is indicated.

- When surgery is performed, protocol treatment is discontinued, regardless of whether curative resection was performed or not. In this case, the date of discontinuation of protocol treatment is the date of surgery.

6.3.10. Consultation on treatment modification

If there are any questions about treatment modification, contact "16.6. Study Coordinator".

Study Coordinator Contact : Chigusa Morizane

Hepato-Biliary Pancreatic Group: Futomi Mori (Main Research Secretariat)
Department of Hepatobiliary and Pancreatic Medicine, National Cancer Center
TEL:03-3542-2511
FAX:03-3542-3815
E-mail:cmorizan@ncc.go.jp

Gastric Cancer Group: Yoshinori Machida
Shizuoka Cancer Center
〒411-8777 1007 Shimonagakubo, Nagaizumi-cho, Sunto-gun, Shizuoka Prefecture
TEL:+81-55-989-5222
FAX:+81-55-989-5631
E-mail：no.machida@scchr.jp

Esophageal Cancer Group: Ken Kato/Yushi Homma
Department of Gastrointestinal Oncology, National Cancer Center
TEL:03-3542-2511
FAX:03-3542-3815
E-mail: kenkato@ncc.go.jp / yohonma@ncc.go.jp

6.4. Concomitant treatment and supportive care

6.4.1. Required concomitant treatment/supportive care

1) Testing and Supportive Care for HBsAg-Positive Cases.

In HBsAg-positive cases, steroids and chemotherapy can lead to rapid hepatitis B virus (HBV) expansion (reactivation: reactivation) and potentially fatal severe hepatitis. Therefore, the following tests and supportive care are performed based on the "Guidelines for the Treatment of Hepatitis B, Third Edition (Japanese Society of Hepatology)." It is advisable to consult a hepatologist at the time prior to initiation of a nucleic acid analogue (entecavir, tenofovir disoproxil fumarate, and tenofovir alafenamide fumarate).

①Testing prior to initiation of chemotherapy: HBV-DNA quantitation

HBV-DNA quantitative analysis should be performed at least once prior to initiation of chemotherapy.

HBV-DNA assays are performed by real-time PCRs.

HBeAg and HBe antibodies should also be measured in accordance with the Guidelines for the Treatment of Hepatitis B, Third Edition (Japanese Society of Hepatology).

②Dosing schedule for supportive care (nucleic acid analogues prophylaxis)

- **Drugs used :**
  - **Entecavir (Bristol-Myers: Baraclude Tablets 0.5 mg)**
  - **Tenofovir disoproxil fumarate (GlaxoSmithKline: Tenozet Tablets 300 mg)**
  - **Tenofovir alafenamide fumarate (Giliado: Vemuridi Tablets 25 mg)**

The following dosage regimen should be followed, starting at least 1 week before the start of chemotherapy (as soon as possible), and continuing for at least 12 months after the end of chemotherapy. However, fulminant hepatitis has been reported in HBsAg-positive patients with high viral load, even during NA prophylaxis, and it is desirable to reduce the viral load before starting immunosuppression/chemotherapy. Nucleic acid analogues may be discontinued after 12 months after completion of chemotherapy if conditions *1 and 2 for discontinuation of NAs are met. However, if the administration of a nucleic acid analogue is discontinued, consultation with a hepatologist is always obtained, and the administration is discontinued only if the hepatologist deems it appropriate.

※1 Requirements for discontinuation of nucleic acid analogues (entecavir, tenofovir disoproxil fumarate, tenofovir alafenamide fumarate): all of the following

1. The patient has been on NA for more than 2 years.
2. HBV-DNA assays are not sensitive to detect
3. Be negative for HBeAg

※2 Patient background requirements: All of the following

1. Both the treating physician and the patient have sufficiently understood that the hepatitis exacerbation is frequently observed after the nucleic acid analog withdrawal, and that there is a danger of becoming serious in the time.
2. Follow-up is possible after treatment cessation, and appropriate treatment is possible even if hepatitis recurs
3. It is judged that the liver fibrillation is slight and the hepatic reserve is good, and it is difficult to become serious even if the hepatitis is exacerbated.

(Adapted from Guidelines for the Treatment of Hepatitis B, the 3rd edition (Japanese Society of Hepatology))

Entecavir

- **Dosage regimen: Take this medicine on an empty stomach (2 hours after meals and more than 2 hours before the next meal).**
- **Dosage :**

Creatinine clearance (mL/min).	Dosage
50 or more	0.5 mg once daily
30 Beyond 50	0.5 mg once every 2 days
10 Beyond 30	0.5 mg once every 3 days
10 Less than	0.5 mg once every 7 days

- **Adverse drug reactions (incidence of all grades): nucleoside analog-naïve patients**

Diarrhea (6.0%), nausea (4.5%), constipation (3.7%), upper abdominal pain (3.0%), malaise (1.5%), nasopharyngitis (3.0%), muscle stiffness (2.2%), headache (3.0%), rash (incidence unknown), laboratory tests: elevated AST (3.7%), increased blood bilirubin (6.0%), increased blood amylase (10.4%), and increased lipase

(10.4%).Blood glucose increased (6.0%), blood lactate increased (6.7%), urine occult blood positive (4.5%), white blood cell count decreased (8.2%), and eosinophil count increased (0.7%). [Significant adverse reactions (incidence unknown)] Hepatitis worsened after completion of treatment, anaphylactoid symptoms, lactic acidosis, and severe hepatomegaly due to fatty liver

Tenofovir disoproxil fumarate

- **Dosage and administration: 300 mg is orally administered once daily.**
- **Dosage :**

Creatinine clearance (mL/min).	Dosage
50 or more	300 mg once daily
30 Beyond 50	300 mg once every 2 days
10 Beyond 30	300 mg once every 3 to 4 days
Hemodialysis	注) of 300 mg once every 7 days<br>Or 300 mg after completion of cumulative approximately 12 hours of dialysis<br>NOTE) After hemodialysis was performed. The pharmacokinetics in patients with creatinine clearance < 10 mL/min and not on hemodialysis have not been investigated.

- **Dosing Precautions:**

In the long-term administration of tenofovir disoproxil fumarate, attention should be paid to renal dysfunction, hypophosphatemia (including Fanconi syndrome), and decrease in bone mineral density. It is recommended that renal function and serum phosphorus should be measured regularly during tenofovir disoproxil fumarate administration.

- **Adverse reactions (incidence of all grades):**

Abnormal liver function tests (AST, ALT and γ-GTP increased, etc.) in 7 patients (4.9%), increased creatinine in 4 patients (2.8%), increased amylase, increased lipase and nausea in 3 patients each (2.1%), abdominal pain in 2 patients (1.4%), [major adverse reactions (incidence unknown)] renal dysfunction, renal failure, acute renal failure, proximal renal tubular dysfunction, Fanconi syndrome, severe renal dysfunction such as acute renal tubular necrosis, nephrogenic diabetes insipidus or nephritis, severe hepatomegaly due to lactic acidosis and fatty deposition (steatohepatitis), pancreatitis

Tenofovir alafenamide fumarate

- **Dosage and administration: 25 mg is orally administered once daily.**
- **Dosage :**

Creatinine clearance (mL/min).	Dosage
15 or more	25 mg once daily
Less than 15	Consider discontinuation

- **Dosing Precautions:**

In the long-term administration of tenofovir alafenamide fumarate, attention should be paid to renal dysfunction, hypophosphatemia (including Fanconi syndrome), and decrease in bone density. It is recommended that renal function and serum phosphorus should be measured periodically during tenofovir alafenamide fumarate administration.

- **Adverse reactions (incidence of all grades):**

Nausea and abdominal distension, headache, fatigue (≧1%), dyspepsia and diarrhea, flatus, upper abdominal pain, constipation, ALT increased, arthralgia, dizziness, insomnia, pruritus, rash (≧0.5% to <1%), [significant adverse reactions (incidence unknown)] renal dysfunction, renal failure, acute renal failure, proximal renal tubular dysfunction, severe renal impairment such as Fanconi syndrome, acute renal tubular necrosis, renal diabetes insipidus or nephritis, severe hepatomegaly due to lactic acidosis and fatty deposits (fatty liver)

③Monitoring: Quantitative analysis of HBV-DNA (during and after administration of nucleic acid analogues)

During nucleic acid analogue administration:

They are monitored every 4 weeks by both HBV-DNA quantitative analysis and liver function (ASTs, ALTs). However, if HBV-DNA level is less than 20 IU/mL (1.3 log IU/mL) during administration of nucleic acid analogues, it is acceptable to perform tests every 4 to 12 weeks.

After discontinuation of nucleic acid analogue administration:

Bearing in mind that reactivation may occur even after discontinuation of administration of a nucleic acid analogues, the patient should be consulted with a hepatologist, and the patient should be monitored for HBV-DNA determination and hepatic function (AST/ALT) every 4 weeks for at least 1 year after discontinuation of administration of a nucleic acid analogues. Subsequent follow-up will be decided after consulting a hepatologist.

2) Laboratory Teste and Supportive Care for HBsAg-Negative and HBc Antibody-Positive and/or HBs Antibody-Positive Cases.

HBV-DNA quantitative analysis should be performed at least once prior to initiation of chemotherapy. HBV-DNA assays are performed by real-time PCRs.

i) HBV-DNA ≧ 20 IU/mL (1. 3 log IU/mL) prior to initiation of chemotherapy

It has been clarified that HBV-DNA replicates persist at low levels in the livers and peripheral blood mononuclear cells when HBc or HBs are positive, even if they are HBs-Ag negative. It has been reported that reactivation of HBV and development of severe hepatitis are caused by the use of potent immunosuppressive agents even in such patients with previous infections.

If HBV-DNA ≧ 20 IU/mL (1.3 log IU/mL), the risk of HBV reactivation is judged to be as high as in HBsAg-positive cases, and prophylactic administration of nucleic acid analogues (entecavir or tenofovir disoproxil fumarate, tenofovir alafenamide fumarate) is administered. The following laboratory tests and supportive care are performed in accordance with the "Guideline for the Treatment of Hepatitis B, 3rd edition (Japanese Society of Hepatology)" with reference to the following for examination, dosage, and monitoring of supportive care before the start of chemotherapy.

However, these are not applicable if the HBs antibody alone is positive and the HBV vaccination history is obvious.

①Dosing schedule for supportive care (nucleic acid analogues prophylaxis)

According to the dosage and administration of nucleic acid analogues (entecavir, tenofovir disoproxil fumarate, tenofovir alafenamide fumarate) in "1) Test and supportive care for HBsAg positive cases". Same conditions for NA discontinuation.

② Monitoring: Quantitative analysis of HBV-DNA (during and after administration of nucleic acid analogues)

The intervals for monitoring during and after discontinuation of NA are in accordance with the provision of "1) Testing and supportive care for HBsAg-positive patients".

ii) HBV-DNA less than 20 IU/mL (1.3 log IU/mL) prior to initiation of chemotherapy

HBV-DNA quantitative analysis and hepatic function (AST, ALT) will be monitored, and nucleic acid analogues (entecavir, tenofovir disoproxil fumarate, tenofovir alafenamide fumarate) will be started when ≧20 IU/mL (1.3 log IU/mL) is achieved.

The Guidelines for the Treatment of Hepatitis B, the 3rd edition (Japanese Society of Hepatology) recommends monitoring with HBV-DNA quantitative analysis or high-sensitivity HBs antibodies during and after chemotherapy, depending on the risks of revitalization.

①Monitor:HBV-DNA quantitative analysis

HBV-DNA quantitative analysis should be performed every 4-12 weeks from the start of chemotherapy until at least 12 months after the end of chemotherapy.

If HBV-DNA level is more than 20 IU/mL (1.3 log IU/mL), administration of nucleic acid analogues should be started immediately in accordance with the Guidelines for the Treatment of Hepatitis B, the 3rd edition (Japanese Society of Hepatology). If HBsAg monitoring is positive for < 1 IU/mL (low positive), nucleic acid analogues should be administered after additional HBV DNA determinations of ≧ 20 IU/mL (1.3 log IU/mL).

It is advisable to consult a hepatologist at a time prior to initiation of NAs.

②Supportive care in reactivation

Nucleic acid analogues should be administered according to the supportive care described in i) When HBV-DNA prior to the initiation of chemotherapy is 20 IU/mL (1.3 log IU/mL) or more in 6.4.1.2). Once administration of nucleic acid analogues is started, nucleic acid analogues should be discontinued only if appropriate by the hepatologist.

6.4.2. Recommended/not recommended concomitant treatment/supportive care

The following concomitant treatment and supportive care are recommended. Even if it is not carried out, it is not regarded as protocol deviation,

1) Addressing Febrile Neutropenia.

① Assessment at onset of febrile neutropenia (FN).

a) If the count is less than 500 per mm^3, or is less than 1000 per mm^3 and is expected to be less than 500 per mm^3 within 48 hours, and if the axillary temperature is 37.5°C or higher (mouth temperature is 38.0°C or higher), a severity-risk assessment should be performed promptly and anti-virus treatment initiated as appropriate.

b) Severity risk assessment is performed with reference to Multinational Association for Supportive Care in Cancer(MASCC) scoring system [※1].

c) For initial evaluation, complete blood count including differential WHITE BLOOD CELL and platelet count, renal function (BUN, creatinine), electrolytes, liver function (transaminases, total bilirubin, alkaline phosphatase) tests, ≧2 sets of venous blood cultures prior to initiation of antimicrobials, one set of cultures from the catheter lumen and one set from the peripheral vein if a central venous catheter is in place, cultures of suspected infection areas, and chest x-rays if respiratory symptoms and signs are present.

d) When febrile neutropenia (FN) develops in a patient with a central venous catheter, blood cultures from the catheter and peripheral blood are performed, and catheter-related infections are considered if there is a time difference of more than 120 minutes in the positivity of both. If appropriate antimicrobial therapy does not improve after more than 72 hours, catheter should be removed. For infections caused by Staphylococcus aureus, Pseudomonas aeruginosa, Bacillus, fungi, and acid-fast bacilli, the catheter should be removed and appropriate antimicrobial therapy based on culture results should be performed.

② Antibiotic use

a) In high-risk patients, β-lactams with anti-Pseudomonas aeruginosa activity are administered intravenously as a single agent. However, other antimicrobials (aminoglycosides, fluoroquinolones, and/or vancomycin) may be added to a single agent in the initial regimen in patients with unstable or complicated conditions or when drug-resistant organisms are strongly suspected. Low-risk patients may be treated with antibiotics orally or intravenously, hospitalized, or with adequate evaluation, if appropriate, as outpatients.

b) Re-evaluation will be performed 3-4 days after initiation of antibiotics to investigate the continuation or change of antibiotics. In principle, antibiotics will be continued until the neutrophil count recovers to 500 cells per mm^3 or more.

c) Empiric antifungal therapy is recommended in high-risk patients who do not respond to 4-7 days of broad-spectrum antibiotics.

d) Fluoroquinolone prophylaxis is recommended in high-risk individuals with an expected neutrophil count of 100 cells per mm^3 or less lasting >7 days.

③ Therapeutic administration of G-CSF

For therapeutic administration of G-CSF during the incidence of FNs, refer to "6.4.5. 3) Therapeutic administration of G-CSF".

※1 Multinational Association for Supportive Care in Cancer (MASCC) scoring system.
(Adapted in part from the Practice Guideline for Febrile Neutropenia (FN) [Japanese Society of Medical Oncology]. *2)

Item	Score
Clinical manifestations (select one of the following * mark 3 sections)<br>*No symptoms<br>*Mild symptoms<br>*Moderate symptoms	 5<br>5<br>3
No decrease in blood pressure	5
No chronic obstructive pulmonary disease	4
Solid tumors, or hematopoietic tumors without a history of fungal infection	4
No dehydration symptoms	3
Patients with fever during outpatient management	3
Age < 60	2

The total score is up to 26 points. Twenty-one points or more are considered low risk and 20 points or less are considered high risk

※2 Since patients aged 20 years or older are subjects in this study, we deleted "Not applicable to patients younger than 16 years old" from the original edition of the Practice Guideline for Febrile Neutropenia (FN) [Japanese Society of Medical Oncology].

2) Nausea and vomiting

Regarding nausea and vomiting, antiemetics are positively administered according to Clinical Practice Guidelines for Antiemesis in Oncology [48], and fluid and electrolyte repletion are performed when oral intake is severely reduced.

3) Anorexia

If oral intake drops markedly, fluid and electrolyte supplements should be given as needed. Especially, in the cases with diabetes mellitus, the abnormality of blood sugar level and electrolyte is noticed.

4) Anemia, thrombocytopenia

If anaemia (haemoglobin < 8.0 g/dL) or thrombocytopenia (platelet count < $2\times10^4/mm^3$) is observed, blood should be transfused as appropriate at the discretion of the treating physician.

5) Diarrhea

Severe diarrhoea occasionally occurs with arm B (IP therapy arm) and is extremely dangerous when complicated by febrile neutropenia. Patients should be fully informed about toxicities and their management, and should be instructed to measure body temperature, especially when neutrophils counts are most decreasing, and to contact a physician or nurse immediately during fever and diarrhea. Nonsteroidal anti-inflammatory drugs may not cause fever to become overt, so unnecessary anti-inflammatory drugs are not given.

If irinotecan-induced diarrhea occurs, the following supportive measures are recommended:

High-dose loperamide hydrochloride therapy

① Loperamide hydrochloride was started after signs of diarrhea were observed.

② Initial dose of 4 mg followed by 2 mg/2 hours (4 mg/4 hours at night)

③ It is administered until watery stool does not appear for more than 12 hours.

④ Doses should not be given for more than 48 hours.

6) Precautions on the day of cisplatin administration

Aminoglycoside antibiotics, vancomycin, and nonsteroidal anti-inflammatory drugs are not administered on the day of cisplatin administration or are used with caution.

6.4.3. Acceptable concomitant treatment and supportive care

The following concomitant treatment and supportive care may be used as needed.

Concomitant use of drugs for the treatment of complications such as hypertension and diabetes mellitus and symptomatic drugs such as morphine may be performed, but this drug should be administered with caution when furosemide, piretanide and phenytoin are used. Oral antibiotics for febrile neutropenia prophylaxis may be given at the discretion of the investigator/sub-investigator. Bisphosphonate denosumab may be used in combination for bone metastases.

6.4.4. Unacceptable concomitant treatment and supportive care

None of the following treatments will be given during protocol treatment:

① Administration of anticancer drugs other than protocol treatment

② Radiation therapy

6.4.5. Granulocyte colony-stimulating factor (granulocyte-colony stimulating factor:G-CSF

※This study permits the use of G-CSF biogenerics (biosimilars).

1)※ of primary prophylaxis with G-CSF

※Primary prophylaxis: G-CSF administration before developing febrile neutropenia or prolonged neutropenia to prevent them during anticancer therapy.

Since the risk of developing FN in both groups is less than 10%, primary prophylactic administration of G-CSF is not recommended according to the "Guidelines for the Appropriate Use of G-CSF 2013" and "JSMO Practice Guidelines for Febrile Neutropenia (FN)".

However, there is information that in IP therapy (group B), the incidence of FN was 10.6% in the JCOG0509 study (advanced small cell lung cancer), and in EP therapy (group A), the incidence of FN was 38% in a study report of 21 patients with hepatobiliary-pancreatic primary NEC by Iwasa et al, although it was an observational study. In addition, the package insert allows primary prophylactic administration of G-CSF for small cell lung cancer, and the treatment regimen for NEC is similar to that for small cell lung cancer. Therefore, primary prophylactic administration of G-CSF is acceptable in the presence of infection, which is a risk factor for the development of FN (including high-risk conditions for infection such as biliary stents) and in elderly patients (>65 years).

Since the safety of pegfilgrastim administered 14 days prior to the initiation of cancer chemotherapy and within 24 hours after completion of administration has not been established, the primary prophylactic administration of pegfilgrastim is not performed in group B where day 8 is administered the drug.

Table 6.4.5. Primary prophylactic administration of G-CSF should be performed according to the approved dosage and administration shown in the table below.

Drug	･･ Pegfilgrastim (arm A only)<br>･･ Filgrastim<br>･･ Naltograstim<br>･･ Lenograstim
Time of initiation	Twenty-four hours after completion of chemotherapy
Dosage Dosing regimen	･Pegfilgrastim (genetical recombination) at a dose of 3.6 mg subcutaneously once per chemotherapy course (group A only)<br>･Filgrastim: 50 µg per m^2 SC once daily or 100 µg per m^2 IV once daily<br>･Naltograstim: 1 µg/kg SC once daily or 2µg/kg IV once daily<br>･Lenograstim: 2 µg/kg SC once daily or 5µg/kg IV once daily
Timing of discontinuation (other than pegfilgrastim)	･If the neutrophil count reaches 5000 per mm^3 or more after the course, administration should be discontinued.<br>･If the neutrophil count recovers to ≧2000 cells per mm^3, there are no suspected infections, and the patient's safety is judged to be ensured by the responsiveness to the drug used, the drug should be discontinued or reduced.

2)Secondary prophylactic※ of G-CSF

※ Secondary prophylaxis: G-CSF prophylactic administration after once occurrence of febrile neutropenia or prolonged neutropenia to prevent febrile neutropenia or prolonged neutropenia from occurring again during anticancer therapy.

If febrile neutropenia or Grade 4 neutropenia lasting more than 7 days occurred in the previous course, secondary prophylactic administration of G-CSF is not recommended because the dose of anticancer agents will be reduced according to the protocol in the next and subsequent courses. However, in the presence of infection, which is a risk factor for the development of FN (including high-risk conditions for infection such as biliary stents) or in elderly patients (65 years and older), secondary prophylactic administration of G-CSF is acceptable after reduction of anticancer agents according to the protocol.

However, the safety of pegfilgrastim administered 14 days prior to the start of cancer chemotherapy and 24 hours after the end of treatment has not been established, so secondary prophylactic pegfilgrastim administration is not performed in group B, where day 8 is given the drug.

Secondary prophylactic administration of G-CSF should be performed according to the approved dosage and administration shown in the table below.

Drug	・・ Pegfilgrastim (arm A only)<br>・・ Filgrastim<br>・・ Naltograstim<br>・・ Lenograstim
Time of initiation	・・ Pegfilgrastim (arm A only)<br>Twenty-four hours after completion of chemotherapy<br>・・ Filgrastim, nartograstim, and lenograstim<br>When neutrophil counts <1000 per mm^3 are observed
Dosage Dosing regimen	・Pegfilgrastim (genetical recombination) at a dose of 3.6 mg subcutaneously once per chemotherapy course (group A only)<br>・Filgrastim: 50 μg per m^2 SC once daily or 100 μg per m^2 IV once daily<br>・Naltograstim: 1 μg/kg SC once daily or 2μg/kg IV once daily<br>・Lenograstim: 2 μg/kg SC once daily or 5μg/kg IV once daily
Timing of discontinuation (other than pegfilgrastim)	・If the neutrophil count reaches 5000 per mm^3 or more after the course, administration should be discontinued.<br>・If the neutrophil count recovers to ≧2000 cells per mm^3, there are no suspected infections, and the patient's safety is judged to be ensured by the responsiveness to the drug used, the drug should be discontinued or reduced.

3) Therapeutic administration of G-CSF

Therapeutic administration of G-CSF should be performed according to the approved dosage and administration shown in the table below.

Time of initiation	・When the neutrophil count is less than 1000 per mm^3 and fever (in principle, > 38.0°C) is observed<br>・When neutrophil counts <500 per mm^3 are observed
Dosage Dosing regimen	・Filgrastim: 50 μg per m^2 SC once daily or 100 μg per m^2 IV once daily<br>・Naltograstim: 1 μg/kg SC once daily or 2μg/kg IV once daily<br>・Lenograstim: 2 μg/kg SC once daily or 5μg/kg IV once daily
Timing of discontinuation	・If the neutrophil count reaches 5000 per mm^3 or more after the course, administration should be discontinued.<br>・If the neutrophil count recovers to ≧2000 cells per mm^3, there are no suspected infections, and the patient's safety is judged to be ensured by the responsiveness to the drug used, the drug should be discontinued or reduced.

6.5. Post-study treatment

Treatment after discontinuation of protocol treatment and treatment after progression or recurrence after completion are not specified.

Treatment (cross over) with drugs included in treatment regimens in the unassigned groups may be used, but if the total dose of cisplatin is greater than 500 mg/m^2, careful attention should be given to accumulating toxicities such as peripheral sensory/motor neuropathy, hearing loss, and renal impairment. Patients should be carefully monitored with adequate risk explanation only if the benefits are apparently outweighed by the risks, such as those with persistent sensitivity to cisplatin and mild cumulative toxicity.

If primary analysis or interim analysis reveals the primary conclusions of the trial, the results of the study will be explained to patients enrolled in this study as needed, and the best treatment will be provided, taking into account the course of treatment of individual patients.

In addition, if the protocol treatment discontinuation criteria apply but clinically "protocol treatment continuation" is judged to be appropriate, consult the research office through the institutional research director or institutional coordinator rather than at the physician level as a general rule (except when time is not allowed). In agreement between the Research Secretariat and the Investigator/Institution Coordinator, decide whether to treat as a → after discontinuation of protocol treatment or to deviate and continue protocol treatment. The details of the consultation

with the Research Secretariat and the decision-making process should be provided in the comment column for the patient's end-of-treatment report and progress record. If continuing the protocol treatment with protocol deviation occurs frequently, the Study Coordinator should consider revising protocol treatment termination criteria using group meetings and group mailing lists, because it is considered that the protocol treatment termination criteria is clinically inappropriate in such situation.

7. Anticipated Adverse Events

7.1. Anticipated adverse reactions

Anticipated adverse reactions in this study are as follows:

7.1.1. Anticipated Adverse Drug Reactions with Drugs

Adverse drug reactions anticipated with protocol treatments and drugs used in protocol-specified tests are referred to the latest version of the drug package insert.

7.1.2. Anticipated adverse reactions in the standard treatment arm (arm A)

Table 7.1.2. Adverse events in the EP-therapy group at JCOG9511 (excerpt from the final analysis report)

Examination Items	Grade 0	Grade 1	Grade 2	Grade 3	Grade 4	Grade 3,4(%)	Grade4(%)	Total
White blood cells	2	5	30	35	5	51.9%	6.5%	77
Neutrophils	1	0	5	21	50	92.2%	64.9%	77
Hemoglobin	2	9	43	23	-	29.9%	-	77
Platelet	31	19	13	14	0	18.2%	0%	77
Total bilirubin	57	-	20	0	0	0%	0%	77
GOT	49	24	2	1	1	2.6%	1.3%	77
GPT	40	28	6	2	1	3.9%	1.3%	77
Creatinine	56	16	5	0	0	0%	0%	77
Oxygen tension	13	27	9	2	1	5.8%	1.9%	52
Nausea and vomiting	13	36	23	5	-	6.5%	-	77
Diarrhea	64	8	5	0	0	0%	0%	77
Oral cavity (stomatitis)	68	6	2	1	0	1.3%	0%	77
Infection	42	23	9	1	2	3.9%	2.6%	77
Hair loss (hair)	9	46	19	-	-	-	-	74
Fever (uninfected)	45	14	16	2	0	2.6%	0%	77
Perception (peripheral nerve)	66	10	1	0	-	0%	-	77
Rash	74	2	1	0	0	0%	0%	77

※: Use JCOG Toxicity Criteria.

7.1.3. Anticipated adverse reactions in the study treatment arm (Arm B)

Table 7.1.3. Adverse Events in the IP-Therapy Group in a JCOG9511 (Extracted from the Final Analysis Report)

Examination Items	Grade 0	Grade 1	Grade 2	Grade 3	Grade 4	Grade 3,4(%)	Grade 4(%)	Total
White blood cells	1	16	38	17	3	26.7%	4.0%	75
Neutrophils	1	8	17	30	19	65.3%	25.3%	75
Hemoglobin	7	10	38	20	-	26.7%	-	75
Platelet	56	6	9	1	3	5.3%	4.0%	75
Total bilirubin	59	-	16	0	0	0%	0%	75
GOT	40	30	5	0	0	0%	0%	75
GPT	35	30	7	3	0	4.0%	0%	75
Creatinine	56	15	4	0	0	0%	0%	75
Oxygen tension	13	20	5	1	1	5.0%	2.5	40
Nausea and vomiting	11	26	28	10	-	13.3%	-	75
Diarrhea	23	19	21	8	4	16.0%	5.3%	75
Oral cavity (stomatitis)	66	9	0	0	0	0%	0%	75

Infection	45	17	9	3	1	5.3%	1.3%	75
Hair loss (hair)	17	44	13	-	-	-	-	74
Fever (uninfected)	45	12	17	1	0	1.3%	0%	75
Perception (peripheral nerve)	71	4	0	0	-	0%	-	75
Rash	70	5	0	0	0	0%	0%	75

※: Use JCOG Toxicity Criteria.

Table 7.1.3. Adverse events in the IP-therapy group in b JCOG0509 (abstracted from the main analysis report)

Examination Items	Grade 0	Grade 1	Grade 2	Grade 3	Grade 4	Grade 3,4(%)	Grade 4(%)	Total
White blood cells	16	36	58	29	3	22.5	2.1	142
Neutrophils	6	10	43	51	32	58.5	22.5	142
Hemoglobin	20	24	65	24	9	23.2	6.3	142
Platelet	125	6	8	2	1	2.1	0.7	142
Total bilirubin	116	15	10	1	0	0.7	0	142
GOT	98	37	5	2	0	1.4	0	142
GPT	80	56	4	2	0	1.4	0	142
Creatinine	97	37	8	0	0	0	0	142
Anorexia	22	67	33	19	1	14.1	0.7	142
Nausea	30	67	36	9	0	6.3	0	142
Vomiting	89	38	10	5	0	3.5	0	142
Diarrhea	52	51	28	11	0	7.7	0	142
Mucositis (examination findings)-oral cavity	126	15	0	0	1	0.7	0.7	142
Hair loss	64	66	12	-	-	-	-	142
Onset of febrile neutropenia	127	-	-	14	1	10.6	0.7	142
Infection with Grade3-4 neutropenia -Bronchus	142	-	0	0	0	0	0	142
-Lung (pneumonia)	137	-	0	4	1	3.5	0.7	142
-Pharynx	142	-	0	0	0	0	0	142
-Upper respiratory tract-unclassifiable	141	-	1	0	0	0	0	142
-Bladder	142	-	0	0	0	0	0	142
-Kidney	142	-	0	0	0	0	0	142
-Urinary tract-subclassification impossible	142	-	0	0	0	0	0	142
Neuropathy: Sensory	127	13	2	0	0	0	0	142

Table 7.1.3.c Adverse events from a pilot trial of postoperative adjuvant chemotherapy with irinotecan plus cisplatin for high-grade neuroendocrine lung cancer (excerpt)

Examination Items	Grade 2	Grade 3	Grade 4	Grade 3,4(%)	Grade 4(%)	Total
White blood cells	17	7	0	17.5%	0%	40
Neutrophils	12	15	4	47.5%	10.0%	40
Hemoglobin	14	6	4	25.0%	10.0%	40
Platelet	2	0	0	0%	0%	40
Total bilirubin	0	0	0	0%	0%	40
GOT	0	0	0	0%	0%	40
GPT	1	0	0	0%	0%	40
Creatinine	0	0	0	0%	0%	40
Hyponatremia	0	5	0	12.5%	0%	40
Endotoxemia	3	1	0	2.5%	0%	40
Hypokalemia	0	4	0	10.0%	0%	40
Nausea	8	4	-	10.0%	-	40
Vomiting	4	2	0	5.0%	0%	40
Anorexia	2	0	-	0%	0%	40

Diarrhea	11	2	0	5.0%	0%	40
Fatigue	10	5	-	12.5%	0%	40
Infection	2	0	0	0%	0%	40

7.2. Anticipated Adverse Events Due to Pathogenesis

1） Esophageal primary

Esophageal bleeding; Esophageal pain; Esophageal stenosis; Esophageal obstruction; Esophageal perforation; Esophageal ulcer; Esophageal fistula; Hoarseness; Pharyngolaryngeal dysesthesia; Hypercalcemia of advanced disease; Tracheal stenosis; Tracheal obstruction; Esophageal anastomotic leakage; Recurrent laryngeal nerve palsy

2） Gastric primary

Gastric bleeding, upper gastrointestinal bleeding, gastric pain, gastrointestinal pain, back pain, nausea, vomiting, dyspepsia, gastroparesis, abdominal fullness, gastric stenosis, gastric obstruction, gastric perforation, duodenal bleeding, duodenal fistula, duodenal perforation, duodenal perforation, duodenal stenosis, gastrostomy, gastrointestinal fistula, gastrointestinal fistula, ileus, gastric anastomotic leak, gastrointestinal anastomotic leak

3） Small intestin/Colon and rectum

Bleeding from tumor; Ileus; Duodenal bleeding; Duodenal obstruction; Duodenal perforation; Duodenal stenosis; Small bowel obstruction; Small bowel stenosis; Small bowel ulcer; Small bowel perforation; Small bowel fistula; Vaginal fistula; Colonic fistula; Colonic stenosis; Colonic obstruction; Colonic perforation; Rectal stenosis; Rectal obstruction; Rectal fistula; Rectal perforation; Rectal fistula; Jejunal perforation; Jejunal perforation; Ileostomy; Ileovesical fistula; Intestinal fistula; Anal pain; Small bowel anastomotic leakage; Large bowel anastomotic leakage; Rectal anastomotic leakage; Pelvic infection

4） Pancreas, biliary tract, and liver

Body weight loss; Fever; Pancreatitis; Cholecystitis; Cholecystic obstruction; Gallbladder pain; Liver failure; Biliary tract infection; Hemorrhage from biliary tract; Duodenal hemorrhage; Duodenal fistula; Duodenal obstruction; Duodenal perforation; Duodenal stricture; Anastomotic ulcer; Biliary anastomosis leak; Pancreatic anastomosis leak; Anastomotic hemorrhage; Anastomotic stricture; Hemorrhage from tumor; Hemorrhage from portal vein stenosis/obstruction; Symptoms associated with portal vein stenosis/obstruction (Portal hypertension, Hepatic failure; Esophageal variceal hemorrhage; Gastritis; Ascites); Back pain; Abdominal pain

5） Anticipated Adverse Events Due to Metastasis

Liver failure, hepatic pain, fever, hepatic infection, respiratory failure, pulmonary infection, superior vena cava syndrome, abdominal distention, abdominal distention, ileus, esophageal obstruction, reverse smoking, gastric obstruction, duodenal obstruction, small bowel obstruction, colonic obstruction, rectal obstruction, rectal stenosis, anal bleeding, anorectal infection, nausea, diarrhea, constipation, ureteral obstruction, and urinary retention,urethral infection, urinary tract infection, bile duct obstruction, biliary obstruction, cholecystitis, biliary hemorrhage, gallbladder pain, pancreatitis, pain, narrowing of luminal organs near metastatic sites (tracheal/gastrointestinal tract, etc.), tracheal obstruction, bronchial stenosis, penetration with adjacent organs, perforation, hoarseness, pleural effusion, chest wall pain, pleural pain, atelectasis, hypercalcemia, disseminated intravascular coagulation, fracture, ataxia, cerebrovascular ischemia, intracranial hemorrhage,headache, dizziness, decreased level of consciousness, aphasia, seizures, spasticity

6） Paraneoplastic syndrome, etc.

(due to incompatible secretory syndrome) hyponatremia, (due to ectopic ACTH syndrome) personality changes, hypertension, hypokalemia, hyperglycemia, Lambert-Eaton myasthenic syndrome, subacute cerebellar degeneration associated with autoantibody production (including ataxia, dysarthria, and nystagmus of limbs), paraneoplastic encephalomyelitis, sensory neuropathy (including dementia, cranial nerve symptoms, dizziness, ataxia, autonomic ataxia, transverse paralysis, and sensory disturbance), thromboembolism, (due to gastrointestinal stenosis) aspiration, anaemia, tumor pain, acute renal failure, myositis, and pulmonary fibrosis

Complications associated with etiolation and gastrointestinal stent insertion are shown below.

- Percutaneous transhepatic cholangiodrainage (PTCD) procedures, including internal-external fistula tube placement:

  Pancreatitis, bile duct stricture, cholecystitis, gallbladder obstruction, gallbladder pain, liver failure, biliary

tract infection, liver infection, cholecystitis, septicemia, hemorrhage, PTCD tubing obstruction/deviation, peritoneal infection, pneumothorax, pleural pain, pleural effusion, intrapleural hemorrhage

- Biliary stenting:
  Pancreatitis, biliary stricture, cholecystitis, gallbladder obstruction, gallbladder pain, liver failure, biliary tract infection, liver infection, cholecystitis, sepsis, hemorrhage, peritoneum infection, pneumothorax, pleural pain, pleural effusion, intrapleural hemorrhage (in case of percutaneous placement), duodenal perforation, pneumonitis (in case of transendoscopic placement), stent obstruction/deviation, duodenal ulcer, and duodenal hemorrhage
- Choledochojejunostomy:
  Biliary tract infection; Pancreatitis; Cholecystitis; Liver infection; Sepsis
- Gastrointestinal stent insertion:
  Bleeding, perforation, pain, stent deviation, stent obstruction, ulceration, fever, sepsis, infection, diarrhea, constipation, urgency (colorectal), thyroid injury (esophagus), jugular arteriovenous injury (esophagus), and mediastinal abscess (esophagus).

7.3. Evaluation of Adverse Events/Reactions

The Common Terminology Criteria for Adverse Events v4.0 Japanese Translated JCOG Version (Japanese translation of NCI-Common Terminology Criteria for Adverse Events v4.0 (CTCAE v4.0)) (CTCAE v4.0-JCOG) will be used to assess adverse events/reactions. For CTCAE v4 0-JCOG in which Grade is defined by laboratory reference values at the institutional reference value, the "JCOG sharing reference range" will be used instead of the institutional reference value at each medical institution. For more information on JCOG sharing reference ranges, see JCOG website (see http://www.jcog.jp/doctor/tool/kijun.html)).

7.3.1. Grading of s adverse events

In grading of adverse events, each grading is closest to the definitions of Grade 0 4 (nearest match). Grading to a higher Grade when the definition of more than one Grade is comparable and when it is difficult to decide on either (highest grade).

Grading should also be given to Grade if specific actions are described, due to their clinical need. For example, patients may refuse oxygen inhalation or chest drainage, even when the patient's pleural effusion is increasing and oxygen inhalation or chest drainage is indicated. In such cases, grading is based on the medical judgment of what should have been done (what should be done) rather than on whether the treatment was actually given (what was actually done).

In the event of treatment-related deaths, original NCI-CTCAE states that the causative adverse event should be Grade 5, but the outcome of the serious adverse event is reported in the SAE report and reviewed in detail. Therefore, Grade 5 of the institutional physician's judgment is not likely to be changed, and whether or not the serious adverse event will result in death will be significantly affected by other factors than the event is not appropriate, so it is not appropriate to compare the frequency of the adverse event by Grade (%Grade 4 and%Grade 5, respectively) between treatment groups or between studies. Because of the poor significance of distinguishing between Grade 4 and Grade 5 in the tabulation, Grade 4 is not considered "Grade 5" in the recording form of this study. A discussion of the causal relationship between adverse events observed in treatment-related deaths and deaths should be included in the "Situation at Death" section of the treatment completion report form and follow-up form, and an urgent report should be made. For the adverse event items specified in "8.2. Testing and Assessment during Treatment" and "8.3. Testing and Endpoints after Treatment Completion" that are determined to be Grade 5 in the post-hoc review including the emergency report, Grade and the date of the first occurrence of the event should be included in the relevant record form (Treatment Course Record Form). If Grade 3 or greater is observed for any other adverse event, or if Grade 3/2/1 adverse event and treatment requires at least 24 hours of hospital stay or prolongation of hospital stay (see 10.1.1.3.)), the AE and Grade and the date of first occurrence should be included in the free form of the treatment course record.

Any Grade on the record form should be recorded in the medical record. Confirmed during site visit audit.

7.3.2. Determination of the causal relationship between adverse events and treatment

In determining the causal relationship between adverse events and treatment, patients are classified into 5 categories of "definite, probable, possible, unlikely, unrelated". Each "causality" is defined as "causality" when judged to be either "definite, probable, possible" and "no causality" when judged to be either "unlikely, unrelated" (see TABLE 7.3.2.

According to Grade of adverse events, serious adverse events requiring expedited reporting as specified in "10.1. Serious Adverse Events and Expedited Reporting" should be reported to the Research Secretariat in accordance with "10.2. Mandatory Reporting and Reporting Procedures of the Investigator".

Table 7.3.2. Criteria for a Causal Relationship Between Adverse Events and Treatment

	Determination	Approach to determination
Cause Fruit Concern Associate Ah Fistula	Definite	The AE is clearly related to the intervention<br>Adverse events are apparently caused/aggravated by protocol treatment and are unlikely to be due to exacerbation of the etiology or other factors (comorbidities, other medications/treatments, or incidents).
	Probable	The AE is likely related to the intervention<br>It is unlikely that the adverse event was caused/aggravated by progression of the underlying pathology or other factors (comorbidity, other drugs/treatments, incident) and is likely to be due to protocol treatment.
	Possible	The AE may be related to the intervention<br>It is plausible (plausible) that an adverse event is considered to have occurred/become more severe with protocol treatment, and unlikely to be due to exacerbation of the etiology or other factors (comorbidities, other medications/treatments, or incidents).
Cause Fruit Concern Associate In Pouch	Unlikely	The AE is doubtfully related to the intervention<br>It is considered plausible (plausible) that the adverse event is due to exacerbation of the pathogenic disease or other factors (comorbidity, other drugs/treatments, incident) rather than to the protocol treatment that it is caused/aggravated.
	Unrelated	The AE is clearly NOT related to the intervention<br>It is judged that the adverse event was caused/aggravated by aggravation of the pathogenic disease or other factors (comorbidity, other drugs/treatments, incident) and is unlikely to be caused by protocol treatment.

8. Examination and Evaluation

8.1. Baseline examination and evaluation before registration

8.1.1. Test conducted before registration (regardless of time before registration)

1） Histopathology (immunostaining requires chromogranin A and synaptophysin) (see Section 3.3)
2） HBs antigen; HBc antibody ※1; HBs antibody ※1, HBV-DNA※2

※1: For HBsAg positive, HBc and HBs antibodies are not required, and HBV-DNA, HBeAg, and HBe antibodies are measured.

※2: Positive results for at least one of the HBc and HBs antibodies also indicate HBV-DNA prior to initiation of therapy (see Section 6.4.1).

8.1.2. Test performed within 56 days before enrollment (liver primary (or unknown primary))

If the liver is primary (or of unknown primary), the following tests should be performed (all allowing for tests performed in other hospitals):

1） Upper gastrointestinal endoscope
2） Lower gastrointestinal endoscope
3） FDG-PET test
4） Otolaryngology (head and neck) examination
5） Urology consultation (male only)
6） Gynecologic exam (female only)

8.1.3. Tests performed within 28 days before enrollment

1) Contrast-enhanced CT※1 (slice thickness of 5 mm or less, if the patient is allergic to iodine), both contrast-enhanced MRI of the abdomen and plain computed tomography (CT) of the imaging range, which is considered to be indispensable below, are performed. All tests are not performed in other hospitals.

※The following areas are indispensable for each primary organ, and if there is another site suspected of metastasis, the radiographic extent is added accordingly.

Primary Organ	Essential radiographic area
Esophagus	Cervical, chest, or abdominal regions
Stomach, duodenum, small intestine, colon, appendix, rectum,<br>Extrahepatic bile duct, Vater ampulla, gallbladder, and pancreas	Chest, abdomen, and pelvis
Hepatic NEC<br>(liver primary or liver metastasis of unknown primary)	Neck, chest, abdomen, and pelvis

2) Endoscopic ※2

Primary Organ	Mandatory test ranges
Esophagus, stomach, duodenum, and ampulla of Vater	Upper gastrointestinal tract (no examination performed at another hospital)
Small intestine, extrahepatic bile ducts, gallbladder, and pancreas	Be not mandatory
Colon, appendix, and rectum	Lower gastrointestinal tract (no examination performed at another hospital)
Hepatic NEC<br>(liver primary or liver metastasis of unknown primary)	8.1.2. Refer to the test performed within 56 days before registration.

※2 Unnecessary if the primary lesion has been resected

3) 12-lead, resting electrocardiography

8.1.4. Tests performed within 14 days before enrollment

1) General condition: PS (ECOG), body weight
2) Physical findings
3) Peripheral blood count: white blood cell count, neutrophil count (ANC: rod + segmented karyocyte), hemoglobin, platelet count

4) Blood biochemistry: total protein, albumin, total bilirubin, AST (GOT), ALT (GPT), BUN, creatinine, LDH, ALP, sodium, potassium, calcium, magnesium, CRP, FBS (fasting blood glucose)
5) Creatinine clearance (CCr): CCr estimates by Cockcroft-Gault equation

   Cockcroft-Gault formula

   Male: Ccr = {(140-Age) × Body Weight (kg)}/{72 × Serum Creatinine Level (mg/dL)}

   Women: Ccr = 0.85 × {(140-Age) × Body Weight (kg)}/{72 × Serum Creatinine Level (mg/dL)}
6) Tumour markers: NSEs, ProGRP, CEAs, SCCs (esophagus primary only), CA19-9 (non-esophageal primary only)
7) Chest X-P (frontal) (substitutable if contrast-enhanced and plain chest CT is obtained)

8.2. Examination and evaluation during treatment

The following safety examination and evaluation are minimal in frequency: Performing examinations more frequently at the discretion of the treating physician is not prohibited.

However, the examination for efficacy evaluation should be performed at specified frequencies, unless progression is suspected, because dense frequency may lead to bias in the efficacy evaluation.

8.2.1. Safety endpoint assessed weekly (CTCAE v4 0 Japanese translation)

The following 1)-3) tests or evaluations should be performed at least weekly. In addition, all of the following items should be checked and evaluated on the scheduled date or the day before anticancer drug administration. However, at least weekly examinations or evaluations should be performed in the same manner until day 28 using the starting date of the last course as day 1, even when the treatment is completed or terminated.

1) Peripheral blood count: white blood cell count, neutrophil count (rod + segmented count), hemoglobin, and platelet count
2) Biochemical tests: albumin, total bilirubin, AST (GOT), ALT (GPT), creatinine, sodium, potassium, calcium, magnesium, CRP
3) Subjective and objective findings (described according to CTCAE v4.0JAPANESE TRANSLATION)
   - General disorders and administration site conditions: fever, fatigue
   - Ear and labyrinth disorders: Tinnitus, hearing impairment
   - Skin and subcutaneous tissue disorders: alopecia
   - Gastrointestinal disorders: Constipation, diarrhea, nausea, vomiting, oral mucositis
   - Metabolism and nutrition disorders: anorexia, dehydration
   - Nervous system disorders: dysgeusia, peripheral sensory neuropathy, peripheral motor neuropathy
   - Musculoskeletal and connective tissue disorders: myalgia, arthralgia
   - Infections and parasites: bronchial infection, pulmonary infection, upper respiratory tract infection, mediastinal infection, pleural infection, catheter-related infection, biliary tract infection, gallbladder infection, and urinary tract infection
   - Blood and lymphatic system disorders: Febrile Neutropenia
   - Vascular disorders: Phlebitis
   - Respiratory, thoracic and mediastinal disorders: dyspnea, hypoxia, pneumonitis

8.2.2. Safety examination and evaluation for each course

1) General condition: Body weight
2) Blood chemistry: LDH, ALP FBS (fasting glucose)

8.2.3. Safety examination and evaluation to be performed as necessary

1) When dyspnea is observed
   - Chest X-P, percutaneous oxygen saturation: SpO2, arterial blood gases: PaO_2
2) If an arrhythmia is observed
   - 12-lead, resting electrocardiography
3) When HBs antigen, HBs antibody, or HBc antibody is positive
   - HBV-DNA (see 6.4.1.).

8.2.4. Efficacy end point

The following tests will be performed every 6 weeks during protocol treatment (±1 week allowed: 6 ± 1 week, 12 ± 1 week, and 18 ± 1 week after the start date of protocol treatment). Tumor response will be assessed according to "11.1. Response Evaluation". Evaluation of the response will be performed using the same test conditions and test methods as the baseline evaluation.

If CT is allergic to contrast material, it is evaluated with plain CT and/or contrast-enhanced MRI. Allergy to contrast media on MRI is assessed by plain CT or plain MRI. When the use of CT contrast medium and MRI contrast medium becomes difficult due to renal dysfunction, it is evaluated by simple CT or simple MRI.

1） Tumour markers: NSEs, ProGRP, CEAs (≧all cases), SCCs (esophagus primary only), CA19-9 (non-esophageal primary only)

2） Contrast-enhanced CT: In principle, the extent of imaging is as follows. () Areas in can be omitted if there is no lesion

Primary Organ	Essential radiographic area
Esophagus	Cervical, chest, or abdominal regions
Stomach, duodenum, small intestine, colon, appendix, rectum,<br>Extrahepatic bile duct, Vater ampulla, gallbladder, and pancreas	(chest), abdomen, and pelvis
Liver NEC (liver primary or liver metastasis of unknown primary)	(neck), (chest), abdomen, and pelvis

8.3. Examination and evaluation after completion of treatment

8.3.1. Efficacy evaluation after completion of treatment

After completion of the protocol treatment, examination and evaluation are made at the following timing:

The following tests should be performed at least every 6 weeks until progressions are confirmed or death.

If progressions are observed, only observation of the outcome will be continued.

1） Tumour markers: NSEs, ProGRP, CEAs (≧all cases), SCCs (esophagus primary only), CA19-9 (non-esophageal primary only)

2） Contrast-enhanced CT: In principle, the extent of imaging is as follows. Areas in parentheses can be omitted if there is no lesion

Primary Organ	Essential radiographic area
Esophagus	Cervical, chest, or abdominal regions
Stomach, duodenum, small intestine, colon, appendix, rectum,<br>Extrahepatic bile duct, Vater ampulla, gallbladder, and pancreas	(chest), abdomen, and pelvis
Liver NEC (liver primary or liver metastasis of unknown primary)	(neck), (chest), abdomen, and pelvis

8.4. Information on post- study treatment

After completion/termination of protocol treatment, the following items will be recorded on the Follow-up Form at each follow-up survey:

1） Content of post-study treatment (if post-study treatment is performed)

2） After protocol treatment termination, the initiation date of the first post-study treatment ((if post-study treatment is performed)

3） PS at the start of post-study treatment

8.5. Study calendar

If the primary site is the esophagus, stomach, duodenum, small intestine, colon, appendix, rectum, extrahepatic bile duct, ampulla of Vater, gallbladder, or pancreas

	Before registratikon Record Pre	After end of chemotherapy		From the date of protocol treatment discontinuation Within 28 days	Until progression after completion of protocol treatment	
		Course Before initiation of the course	During the course		Only at the start of post-treatment	Every 6 weeks
Physical findings						
Body weight	○[14]	○				
PS	○[14]				■	
Physical findings	○[14]	○			■	
Laboratory tests						
WBC, differential (neutrophil) Hb, platelets	○[14]	○	●	●		
Alb, T-Bil, AST, ALT, Cr, Na, K, Ca, Mg, CRP	○[14]	○	●	●		
LDH, ALP, FBS	○[14]	○				
Total protein, BUN, Ca	○[14]					
NSE, ProGRP、CEA	○[14]		△			
SCC (esophagus primary only), CA19-9 (other than primary esophageal)	○[14]		△			
HBs antigen, HBc antibody, and HBs antibody	○[前]					
Chest X-P (can be substituted if CT is taken)	○[14]					
12-lead, resting electrocardiography	○[28]					
Upper gastrointestinal endoscope (Primary: esophagus, stomach, duodenum)	○[28]					
Lower gastrointestinal endoscope (Primary: colonic, appendix, rectum)	○[28]					
Efficacy evaluation						
Contrast-Enhanced CT*	○[28]		△			△
Toxicity evaluation						
Subjective symptom check		○	●	●		
Objective symptom check		○	●	●		
Submission of CRFs						
Pre-treatment Form		□				
Treatment Form			□			
Off-treatment Form				□		
Follow-up Form					2 times/year	

○[前]: Conduct before registration, ○[28]: Perform within 28 days before registration, ○[14]: Perform within 14 days before registration

○:Conduct, ♀: Implementation at least once a week

△: Every 6 weeks (see 8.2.4.), ∞: Only once at the beginning of aftertreatment, □: Submitted.

*See 8.1.3 for the shooting range. Contrast-induced allergy or renal dysfunction is assessed by plain CT or contrast-

enhanced MRI.

※Follow-up Forms will be sent up to 1 years after completion of accrual and will be submitted after 1 years of registration in the individual patient according to the closing date of registration.

For liver NEC (liver primary or liver metastasis of unknown primary)

	Before registratikon Record Pre	After end of chemotherapy<br>Course Before initiation of the course	 During the course	From the date of protocol treatment discontinuation Within 28 days	Until progression after completion of protocol treatment<br>Only at the start of post-treatment	 Every 6 weeks
Physical findings						
Body weight	○[14]	○				
PS	○[14]				■	
Physical findings	○[14]	○			■	
Laboratory tests						
WBC, differential (neutrophil) Hb, platelets	○[14]	○	●	●		
Alb, T-Bil, AST, ALT, Cr, Na,K, Ca, Mg, CRP	○[14]	○	●	●		
LDH, ALP, FBS	○[14]	○				
Total protein, BUN, Ca	○[14]					
NSE, ProGRP、CEA、CA19-9	○[14]		△			
HBs antigen, HBc antibody, and HBs antibody	○[前]					
Chest X-P (can be substituted if CT is taken)	○[14]					
12-lead, resting electrocardiography	○[28]					
Upper gastrointestinal endoscope	○[56]					
Lower gastrointestinal endoscope	○[56]					
FDG-PET	○[56]					
Otolaryngologic examination	○[56]					
Urology consultation (male only)	○[56]					
Gynecologic exam (female only)	○[56]					
Efficacy evaluation						
Contrast-Enhanced CT*	○[28]		△			△
Toxicity evaluation						
Subjective symptom check		○	●	●		
Objective symptom check		○	●	●		
Submission of CRFs						
Pre-treatment Form		□				
Treatment Form			□			
Off-treatment Form				□		
Follow-up Form					□ 2 times/year	

○[前]: Conduct before registration, ○[56]: Perform within 56 days before registration, ○[28]: Perform within 28 days before registration,

○[14]: Implemented within 14 days prior to enrollment

○:Conduct, ♀: Implementation at least once a week

△: Every 6 weeks (see 8.2.4.), ∞: Only once at the commencement of post-treatment, □: Submitted.

*See 8.1.3 for the shooting range. Contrast-induced allergy and renal dysfunction are evaluated by plain CT or contrast-enhanced MRI.

※Follow-up Forms will be sent up to 1 years after completion of accrual and will be submitted after 1 years of

registration in the individual patient according to the closing date of registration.

9. Data collection

9.1. Case Report Form (CRF）

9.1.1. Types of CRF and submission deadlines

The case report forms (CRF) used in this study and their submission deadlines are as follows:

1) Pre-treatment report (blue)- Less than 2 weeks after enrollment
2) Treatment course records- Every 2 cycles/less than 2 weeks after end of protocol treatment
   3）-1 Treatment (yellow)
   3）-2 Test (yellow)
   3）-3 Adverse events (yellow)
3) Tumor shrinkage report (green) - Less than 2 weeks after judgment of effect
4) End of treatment report (red) - Less than 2 weeks after discontinuation/end of protocol treatment
5) Follow-up investigation (white)- By the deadline indicated in the Follow-up Investigation Form

- For “1) Pre-treatment report to 4) End of treatment report”, CRFs with basic patient information (enrollment code, facility name) pre-printed on them would be sent by post from the data center. If the CRFs do not arrive within one week of enrollment, or if the CRFs have been lost/damaged, the data center should be contacted by telephone, and a request should be made for them to be re-issued.
- “5) Follow-up investigation” would be sent by post from the data center at the time of follow-up investigations that are conducted at the same time as monitoring and interim/final analyses at the data center.

9.1.2. Storage CRF

- Completed CRFs must all be archived at the facility as photocopies or in electronic form.
- Copies of CRFs should be kept archived until the final analysis report is issued for reference while filling other CRFs, or for review while retrieving information from the data center.

9.1.3. Method of sending CRF

- All CRF must be sent by post or handed over in person at the data center. They must not be sent by FAX.
- To avoid the risk of personal patient information being leaked, the patient enrollment code should be used, instead of using the <u>patient medical chart number at the facility</u>, when contacting the data center for request of CRF dispatch.

9.1.4. Correction of the contents of CRFs

If any data necessary for the CRF are found to be missing or there are inappropriate category classifications after the start of the study, the CRFs may be corrected with the agreement of the head of the data center and the clinical trial secretariat, in a manner not exceeding the scope of data collected as prescribed in “8. Evaluation item/Clinical laboratory tests/Evaluation schedule”, and within the scope deemed not to increase medical and financial burden on the enrolled patient from the CRF correction. <u>Modification of CRFs that do not require the main body of the protocol to be revised is not considered a protocol revision by JCOG</u>. Reports to the head of the medical institution related to CRF correction and the request of application for revision should follow the rules of the facility.

10. Reporting of "disease or the like"(adverse events)

Site investigator should report to Study Coordinator/Principal Investigator (Study Chair) if a serious adverse event ("disease or the like" on Clinical Trials Act) occurs in accordance with the regulations of Clinical Trials Act (Law No. 16, 2017), Enforcement Regulations of Clinical Trials Act (MHLW Notification No. 17, 2018) and the regulations in this chapter based on the relevant notifications.

The most recent version of the report is available on the MHLW website [1)] and on the JCOG website [2)]. Use the most recent version of the report.

Serious adverse events occurring after the initiation of protocol treatment (after the date of registration if death) by the date of final follow-up will be subjects.

1) http://www.mhlw.go.jp/stf/seisakunitsuite/bunya/0000163417.html
2) http://www.jcog.jp/doctor/todo/researcher/harmfulness.html
3) http://www.pmda.go.jp/safety/reports/hcp/pmd-act/0002.html

10.1. Serious Adverse Events

Serious adverse events are defined as any of the following:
(These are classified as "disease or the like" on Clinical Trials Act.)

1) Death
2) Diseases that may lead to death
3) "Disease or the like" requiring hospitalization or prolongation of hospital stay for treatment.
4) Disability
5) "Disease or the like" that may lead to disability
6) Serious "disease or the like" according to 1) to 5)
7) Congenital disease or abnormality in later generations

1) Death

(i) All deaths that occur after registration and before the start of protocol treatment
(ii) All deaths (with or without causality to protocol treatment) that occur during protocol treatment or within 30 days of the last treatment day
(iii) Death that occur after 31 days from the last treatment date that are causally related to protocol treatment (definite, probable, possible)

2) "Disease or the like" that may lead to death

(i) Grade 4 adverse events that occur during protocol treatment or within 30 days of the last treatment day (excluding events in Table 10.1)
(ii) Grade 4 adverse events that occur after 31 days from the last treatment date (excluding events in Table 10.1) that are causally related to the protocol treatment (definite, probable, possible)

3) Hospitalization or prolongation of hospital※1 stay for treatment

(i) Grade 3/2/1 adverse events that occur during or within 30 days of protocol treatment and requiring at least 24 hours of hospitalization or prolongation of hospital stay※1 to treat the adverse event (excluding the event in Table 10.1).
(ii) Grade 3/2/1 adverse events that occur 31 days after the last treatment day and requires 24-hour or longer hospitalization or prolongation of hospital stay※1 for treatment and causally related to protocol treatment (definite, probable, possible) (excluding the events in Table 10.1)

※ 1 "Hospitalization or prolongation of hospital stay" refers only to those for which hospitalization of at least 24 hours or prolongation of hospital stay is medically required for the treatment of an adverse event. The followings are not subjects for reporting:

- Hospitalization or prolongation of hospital stay performed for follow-up of adverse event that has disappeared or improved
- Hospitalization or prolongation of hospital stay for reducing patient burden, e.g. patients from distant areas.

- Hospitalization or prolongation of hospital stay for other medically unnecessary situation

4) Disability, 5) Disease that may lead to disability

Permanent or marked disability/dysfunction (excluding myelodysplastic syndromes, secondary cancers, etc.) or possible medical situation

6) Serious disease similar to 1) to 5)

7) Congenital disorders or abnormalities in later generations

Table 10.1. Adverse events excluded from the subjects of Expedited Reporting

SOC※(CTCAE ver4.0)	AE term
Blood and lymphocyte disorders	Anemia, bone marrow hypocellular
Gastrointestinal disorders	Constipation
General disorders and local symptoms	Fever
Infections and infestations	Viral hepatitis
Clinical laboratory test	ALP increased, CD4 lymphocytes decreased, high cholesterol, GGT increased, lipase increased, lymphocytes decreased, neutrophils decreased, platelet count decreased, serum amylase increased, WBC decreased, hyponatremia, hypokalemia, hyperglycemia, and hypoglycemia
Metabolism and nutritional disorders	Obesity, anorexia, hyperuricemia, and hypoalbuminemia
Musculoskeletal and connective tissue disorders	Fibrosis deep connective tissue and superficial soft tissue fibrosis
Renal and urinary disorders	Chronic kidney disease
Respiratory, thoracic, and mediastinal disorders	Sinus disorder and sleep apnea
Skin and subcutaneous tissue disorders	Hypohidrosis

※ SOC:System Organ Class

10.2. Investigator's reporting requirements and procedures

10.2.1. Expedited Reporting

In the event of a serious adverse event, the Subinvestigator must promptly inform the Investigator. If the Investigator cannot be contacted, the Site Coordinator or Subinvestigator must take over the responsibility of the Investigator. The Investigator must report adverse events according to the following procedures.

Attention should be paid not to include the patient's name and medical record number when sent.

Serious adverse events that occur after the initiation of protocol treatment (after the date of registration if death) by the date of final follow-up are subjects of Expedited Reporting.

1) "Disease or the like" that may lead to death or death specified in 10.1 1) and 2).

Primary reporting:

The Subinvestigator who is aware of the occurrence of adverse events will promptly notify the Investigator. The Investigator who receives the notice should fill out JCOG Adverse Event Report Form(for institution) and the "Disease or the like" Report Form to Certified Review Board specified in Clinical Trials Act Enforcement Regulations as far as possible and contact Principal Investigator/Study Coordinator via e-mail within 72 hours of knowledge of the occurrence of the adverse event.

Secondary reporting:

The Investigator should add detailed information on adverse events to JCOG Adverse Event Report Form(for institution) and the "Disease or the like" Report Form to Certified Review Board specified in Clinical Trials Act Enforcement Regulations within 7 days of knowledge of the occurrence of adverse events and send them to the Principal Investigator/Study Coordinator via e-mail. If necessary, attach copies of laboratory data, images, autopsy report, etc.

2) 10.1. 3) Disease or other medically important condition requiring hospitalization or prolongation of hospital stay for treatment. Adverse events are judged to be either of 10.1. 4)-7)

The Subinvestigator who is aware of the occurrence of adverse events will promptly notify the Investigator. The Investigator who receives notice must fill out JCOG Adverse Event Report Form (for institution) and the "Disease or the like" Report Form to Certified Review Board specified in Clinical Trials Act Enforcement Regulations within 10 days of knowledge of the occurrence of an adverse event and send them to the Principal Investigator/Study Coordinator via e-mail. If necessary, attach copies of laboratory data, images, autopsy report, etc.

3) Additional reporting

If new information is obtained after conducting the above reporting, the Investigator must add information to JCOG Adverse Event Report Form (for institution) and the "Disease or the like" Report Form to Certified Review Board specified in Clinical Trials Act Enforcement Regulations and report it as needed.

Table 10.2.1. Summary of Adverse Events which are subjects for Expedited Reporting and the deadline of reporting to Principal Investigator/Study Coordinator

Causal relationship	The patient was hospitalized due to Grade 1-3 AE. Other medically important conditions		Grade 4		Death	
	Expected	Not expected	Expected	Not expected	Expected	Not expected
Present	Primary reporting: within 10 days Additional reporting: as needed	Primary reporting: within 10 days Additional reporting: as needed	Primary reporting: within 72 hours Secondary reporting: within 7 days Additional reporting: as needed			
None	<Only on-treatment or within 30 days of last protocol treatment day>					
	Primary reporting: within 10 days Additional reporting: as needed	Primary reporting: within 10 days Additional reporting: as needed	Primary reporting: within 72 hours Secondary reporting: within 7 days Additional reporting: as needed			

* 4) Disability, 5) "Disease or the like" that may lead to disability, and 6) "Disease or the like" that are serious similar to 1) to 5) in 10.1., 7) Congenital disorders or abnormalities in later generations

※ "Unexpected" refers to those not listed in "7. Expected Adverse Events"

Table 10.2.1. Summary of Adverse Events which are subjects for Expedited Reporting and the deadline of reporting to Principal Investigator/Study Coordinator

Causal relationship	The patient was hospitalized due to Grade 1-3 AE. *Other medically important conditions		Grade 4		Death	
	Expected	Not expected	Expected	Not expected	Expected	Not expected
Present	Primary reporting: within 10 days Additional reporting: as needed	Primary reporting: within 10 days Additional reporting: as needed	Primary reporting: within 72 hours Secondary reporting: within 7 days Additional reporting: as needed			
None	<Only on-treatment or within 30 days of last protocol treatment day>					
	Primary reporting: within 10 days Additional reporting: as needed	Primary reporting: within 10 days Additional reporting: as needed	Primary reporting: within 72 hours Secondary reporting: within 7 days Additional reporting: as needed			

* 4) Disability, 5) "Disease or the like" that may lead to disability, and 6) "Disease or the like" that are serious similar to 1) to 5) in 10.1., 7) Congenital disorders or abnormalities in later generations

※ "Unexpected" refers to those not listed in "7. Expected Adverse Events"

10.2.2. Reporting to the Administrator of participating medical organizations

If an Adverse Event which is subjects for Expedited Reporting occurs and is assessed as causal after reporting to Principal Investigator and reported to Certified Review Board, the Investigator must report it to the Administrator of the relevant medical institution in accordance with the requirements of the medical institution.

10.3. Responsibilities of Principal Investigator/Study Coordinator

10.3.1. Determination of necessity of suspension of registration and emergency notification to institutions

Principal Investigator/Study Coordinator who received the report from the Site Investigator should report to Group Chair and determine the urgency, significance, and impact of the report. If needed, take measures such as suspending registration (contacting JCOG Data Center and all participating institutions) and urgently communicating information to participating institutions. Telephone calls can be made to Data Center and institutions as urgent, but they should also be promptly contacted by document (e-mail).

10.3.2. Reporting to JCOG Operations Office and Certified Review Board and MHLW

1) Reporting from the Principal Investigator/Study Coordinator to JCOG Operations Office

Principal Investigator/Study Coordinator should consult with Group Chair and report to JCOG Operations Office (Safety Contact) by e-mail within 72 hours of knowledge of the occurrence of the adverse event, if reported AE is considered to meet the adverse events specified in 10.1.1 1)~7). In doing so, to the extent feasible, Principal Investigator/Study Coordinator should send "JCOG Adverse Event Report Form (for institution)" sent from the institution, "Disease or the like Report Form" addressed to Certified Review Board as stipulated in Clinical Trials Act Enforcement Regulations, and attach "JCOG Adverse Event Report (for Study Coordinator)" with Study Coordinator/Principal Investigator's view (including judgments of causality and expectation, and judgments of continuation/discontinuation of the study)". For the expected adverse events of 10.1.1 1)~7)., include a discussion not only of the individual patient's course but also of whether the frequency of appearance is within the expected range.

2) Reporting to Certified Review Board

JCOG Operations Office (Safety Contact) reviews the appropriateness of the judgement of causality and expectation of adverse events reported in the above procedures and can ask Principal Investigator/Study Coordinator to reconsider them if there is any doubt. Adverse events considered by Principal Investigator/Study Coordinator and JCOG Operations Office to be related to the protocol treatment and to be the subject of reporting in the following tables should reported to Certified Review Board through JCOG Operations Office.

If there is a disagreement between Principal Investigator/Study Coordinator and JCOG Operations Office, report it to Data and Safety Monitoring Committee and seek final judgment from the Chair of Data and Safety Monitoring Committee. However, if the reporting may exceed deadlines of reporting, the report can be tentatively reported as "causal" to Certified Review Board.

Reporting subjects and reporting deadline

Principal Investigator/Study Coordinator must report to Certified Review Board through JCOG Operations Office within the following time periods after knowledge of the occurrence of adverse events.

Causal relation ship	The patient was hospitalized due to Grade 1-3 AE. Other medically important condition		Grade 4		Death	
	Expected	Not expected	Expected	Not expected	Expected	Not expected
Present	No need of reporting	Within 15 days	Within 15 days	Within 7 days	Within 15 days	Within 7 days
None	No need of reporting	No need of reporting	No need of reporting	No need of reporting	No need of reporting	No need of reporting

※ "Unexpected" refers to those not listed in "7. Expected Adverse Events"

Principal Investigator/Study Coordinator must report adverse events to Certified Review Board through JCOG

Operations Office within the following deadlines after knowledge of the occurrence of adverse events.

Causal relationship	The patient was hospitalized due to Grade 1-3 AE. Other medically important condition		Grade 4		Death	
	Expected	Not expected	Expected	Not expected	Expected	Not expected
Present	Within 30 days	Within 15 days	Within 30 days	Within 15 days	Within 15 days	Within 15 days
None	No need of reporting	No need of reporting	No need of reporting	No need of reporting	No need of reporting	No need of reporting

※ "Unexpected" refers to those not listed in "7. Expected Adverse Events"

3) Reporting to MHLW

Principal Investigator/Study Coordinator of the study with unapproved or off-label health care should report "Disease or the like" Report Form specified in Clinical Trials Act Enforcement Regulations to MHLW through JCOG Operations Office, if an adverse event is considered to be unexpected and to have a causal relationship to the protocol treatment (See 10.5. Responsibilities of the Data and Safety Monitoring Committee).

Reporting subjects and reporting deadlines

Principal Investigator/Study Coordinator should report to the MHLW through JCOG Operations Office within the following deadlines after knowledge of the occurrence of adverse events.

※ Pharmaceuticals and Medical Devices Agency Safety Division I (trk-shippeitouhokoku@pmda.go.jp)

Causal relationship	The patient was hospitalized due to Grade 1-3 AE. Other medically important condition		Grade 4		Death	
	Expected	Not expected	Expected	Not expected	Expected	Not expected
Present	No need of reporting	Within 15 days	No need of reporting	Within 7 days	No need of reporting	Within 7 days
None	No need of reporting	No need of reporting	No need of reporting	No need of reporting	No need of reporting	No need of reporting

※ "Unexpected" refers to those not listed in "7. Expected Adverse Events"

4) Additional reporting

Following receipt of secondary or additional reports from the Investigator, Principal Investigator/Study Coordinator must add additional information from the primary report and their views to JCOG Adverse Event Report Form (for the institution) and the "Disease or the like" Report Form to Certified Review Board set out in Clinical Trials Act Enforcement Regulations, and promptly contact JCOG Operations Office (Safety Contact) by e-mail. If the report was sent to Certified Review Board and the MHLW in the primary reporting,, the secondary reporting and additional reporting must be made in the same manner.

10.3.3. Notification to the Site Investigators

When reported to Certified Review Board, Principal Investigator/Study Coordinator should inform the Investigators of all participating institutions of the review results and recommendations by documents (e-mail is allowed). Principal Investigator/Study Coordinator must inform the Investigators without waiting for Certified Review Board review if there is any urgent information to be disseminated. In the event that reported to the MHLW, Principal Investigator/Study Coordinator should notify the Investigators of all participating institutions.

In addition, even if no reporting is made to Certified Review Board, Principal Investigator/Study Coordinator must inform the Investigator of the reporting institution of the decision of Principal Investigator/Study Coordinator by documents (e-mail is allowed).

10.3.4. Assessment of Adverse Events in Periodic Monitoring

During Periodic Monitoring, Principal investigator/Study Coordinator should carefully review the adverse events

in the Monitoring Reports issued by the Data Center and ensure that there are no missed reporting from the participating institutions. It should also be confirmed that all reported adverse events are listed in the Monitoring Reports. The presence or absence of a missed reporting should be indicated in the column of the results of Group review on the Periodic Monitoring Report.

10.4. Responsibilities of the Site Investigators at the participating institutions (including the relevant institution)

In accordance with the instructions of Principal Investigator/Study Coordinator, the Site Investigator at the participating institution should report to the administrator of the relevant institution if the adverse event is subjects of reporting of "disease or the like" to Certified Review Board in accordance with the regulations of the relevant institution.

10.5. Responsibilities of the Data and Safety Monitoring Committee

JCOG Operations Office (Safety Contact) should check the details of the adverse event reports received from the Principal Investigator/Study Coordinator according to the procedures described in 10.3.2. and should report them to Certified Review Board and the MHLW according to the procedures described below, with the presence or absence of causality or expectation.

In addition, Principal Investigator and Director of Data Center can hear the opinions of JCOG Data and Safety Monitoring Committee according to the reported adverse events. If a review request is issued, Data and Safety Monitoring Committee can review the appropriateness of the institutional response to adverse events and the propriety of continuation of the study in a consensus or written form.

In addition, the submitted information (JCOG Adverse Event Report (for institution), JCOG Adverse Event Report (for Study Coordinator), "Disease or the like" Report, etc.) will be stored semi-permanently in JCOG Operations Office.

Subjects, destination and deadlines for reporting after the knowledge by Investigator/Study Coordinator are as described in 10.3.2.

11. Response Evaluation and Endpoint Definition

11.1. Response assessment (only for patients with measurable disease)

Tumour response assessment will be performed according to the following steps according to the [49] of version 1. 1-Japanese translational JCOG version-Revised RECIST guideline (version 1. 1) Revised new guidelines for the assessment of treatment response in solid tumours (RECIST guidelines). RECISTv1. The 0 original article stipulates that "the use of this guideline for the purpose of determining continuation of treatment is not the subject of this guideline." Similar statements continue to be included in RECISTv1 1 as follows.

> "Many oncologists make decisions about whether to continue treatment based on both objective imaging criteria and symptom-based criteria for follow-up of patients with malignancies in their daily clinical practice, but these revised guidelines are not intended to be used to make decisions about whether or not to continue treatment in these individual patients, unless the treating oncologist determines that it is appropriate."

Therefore, the "overall effect" as determined by RECIST Guideline-based response assessment should be used to determine whether a drug or regimen shows encouraging results that merit continued developmental studies. In other words, judgment of whether or not to continue treatment in individual patients should not be based on CR/PR/SD/PD of overall efficacy, but rather on "clinical judgment" based on comprehensive consideration of symptoms, physical findings, and various laboratory data, in addition to imaging findings.

Therefore, it may be clinically appropriate to continue protocolized treatment, even when PD (Progressive Disease: progression) is judged as an overall response based on the assessment of response based on imaging. In this case, the pros and cons of continuing protocol treatment should be determined based on clinical judgment, regardless of response assessment, but the date of the event for progression-free survival, which is judged to be an overall effect of PD, should be used. This is due to three reasons: (i) it may be possible to decide whether protocol treatment should be continued for each group; (ii) RECIST is a criterion intended to standardize not only response rates but also progression-free survival; and (iii) the standard definition of US Cooperative Group is that PD is the event of progression-free survival for any reason if the overall response is PD.

On the other hand, if a physician judges "clinical progression" based on clinical and comprehensive judgment not based on diagnostic imaging, even if PD is not met by the response criteria based on diagnostic imaging, protocol treatment should be discontinued in accordance with "6.2.2. Criteria for discontinuation of protocol treatment". If "clinical exacerbation" is judged, even if "PD" is not judged by the response evaluation, the day of "clinical exacerbation" is considered as an event of progression-free survival. This is because imaging is often not performed as planned after a patient is judged to have a "clinical progression" and therefore the risk of overestimating progression-free survival is greater if "clinical progression" is not an event for progression-free survival. It is also statistically incorrect (informative censoring) to treat "clinical progression" as "censoring" progression-free survival, as it would censoring patients at increased risk of progression or death.

In RECISTv1 1, the original article described "definite progression (unequivocal progression") in the PD criteria for non-target lesions as "marked progression of non-target lesions that deserves discontinuation of treatment as an increase in total tumor burden" and therefore described "marked progression of non-target lesions" as "judgement of whether or not to continue treatment in individual patients" in some of the PD criteria for non-target lesions, which is confusing. It should be noted that this "unequivocal progression" is a criterion of judgment restricted to "PD of non-target lesions".

The relation between the events of 'PD', 'clinical progression', 'progression', and progression-free survival in JCOG is as in the lower panel.

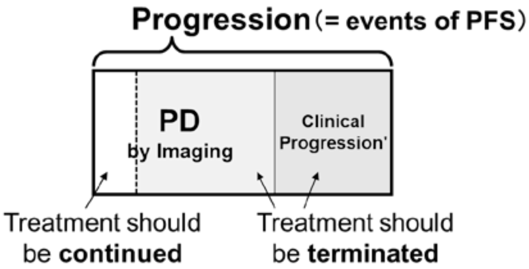

Figure 11.1. Relationship Between Exacerbations, PD on Imaging, and Clinical Exacerbations.

11.1.1. Baseline Evaluation

「8.1. According to "Pre-registration Evaluation Items" contrast enhanced-enhanced computed tomography (CT) with a range of indispensable for the primary organ is used to identify neoplastic lesions before enrollment, and each lesion is classified into "measurable lesions" and "unmeasurable lesions". If CT is allergic to contrast medium, both contrast-enhanced MRI and plain CT should be performed.

Tumor diameters are measured by CT or MRI in cross-sectional views, and sagittal and coronal measurements by three-dimensional reconstructed images are not used. Baseline assessment will be done using the latest imaging studies within 28 days prior to enrollment. If the imaging test is re-examined after enrollment and before the start of treatment, the latest imaging test with re-examination should be used.

11.1.2. Definition of measurable lesions

Lesions that fall under any of the following conditions are considered measurable lesions (measurable lesion):

1) Non-nodal disease (non-nodal disease) of 10 mm or greater in greatest dimension on CT or MRI with a slice thickness of 5 mm or less
2) CT or MRI of 5 mm or less slice thickness showing lymph node lesion of 15 mm or more in short diameter (Nodal lesions with short diameters between 10 mm and less than 15 mm are defined as non-target lesions, and those with short diameters less than 10 mm are not)

All other lesions will be non-measurable (non-measurable lesion).

Caution should be exercised because the following lesions are not measurable regardless of the examination method or the size of the lesion.

- Bone lesions (excluding osteolytic lesions with measurable soft tissue components)
- Cystic lesion
- Leptomeningeal lesions
- Ascites, pleural effusion, and pericardial effusion
- Lymphangiosis of the skin and lungs
- Palpable but not measurable abdominal mass or enlargement of abdominal organs
- Superficial skin lesions

11.1.3. Target Lesion Selection and Baseline Recording

Up to five measurable lesions, in descending order of diameter (non-lymph node lesions are long diameters and nodal lesions are short diameters), up to two lesions per organ are selected to be target lesions (target lesion) among measurable lesions at enrollment. Selection should take into account the universal inclusion of as many organs with measurable disease as possible and the reproducibility or ease of measurement (reproducible repeated measurement) of repeated measurements (avoiding lesions that are not measurable even if they are large in diameter).

For selected target lesions, in order from cranial to caudal, site (code), test method, test date, long diameter of non-lymph node target lesion, short diameter of nodal target lesion, and sum of all target lesion diameters (hereafter, sum of diameters) will be recorded in Pretreatment Report 3.

11.1.4. Baseline recording of non-target lesions

For lesions not selected as target lesions, all measurable or non-target lesions (non-target lesion) should be recorded as site of lesion (code), method of examination, and date of examination in Pretreatment Report 3. Multiple non-target lesions within the same organ may be recorded as a single lesion (e.g., multiple enlarged pelvic lymph nodes, multiple liver metastases).

11.1.5. Determining tumor response

Evaluation of target and non-target lesions will be performed every 6 weeks according to "8.2 Testing and Evaluation during Treatment" in the same manner as at enrollment. Target lesion diameter, non-target lesion disappearance or progression will be recorded in the "Tumor Reduction Effect Report".

11.1.6. Response Evaluation Criteria for Target Lesions

・CR(Complete Response): Complete response

When all non-lymph node target lesions disappear and all nodal target lesions have a short diameter of less than 10 mm. If a nodal target lesion is selected at baseline, the effect of the target lesion may be CR

even if the sum of diameters is not 0 mm.

・PR(Partial Response): Partial response

30% or more reduction in target diameter sum compared to baseline diameter sum

・PD(Progressive Disease): Progress

Compared to the minimum diameters (when the baseline is the minimum value during the passage, this is the minimum sum of diameters), the sum of the target disease increases by more than 20%, and the sum of diameters increases by more than 5 mm even in absolute value.

・SD(Stable Disease): Stability

There is no reduction corresponding to PR and no increase corresponding to PD compared to the smallest sum of diameters during the course

・Lack of study; Not all Evaluated

If the test cannot be performed for any reason or if neither CR, PR, PD, or SD can be determined

Pre-treatment sum of diameters-sum of diameters at study
Percentage of reduction of the diameter sum =---------------× x 100%
Pre-treatment diameter sum

Sum of diameters at study-minimum sum of diameters
Increasing Percentage of Diagram =---------------× x 100%
Minimum sum of diameters

※ Measured target lesion diameters are recorded whenever measurable (e.g., <5 mm). If the target lesion diameter is judged to be too small to be measured (too small to measure), the diameter should be 0 mm if the tumor lesion is judged not to be retained, and 5 mm if the tumor lesion is judged to be retained, regardless of the CT slice thickness.

※ PD is defined when the reduction ratio meets the condition of PR and the concomitant increase ratio meets the condition of PD.

※ When one lesion is separated during treatment, each diameter is added to the sum of diameters.

※ If more than one lesion fuses and the boundary cannot be distinguished during treatment, the diameter of the fused lesion is added to the sum of the diameters. The diameter of each lesion is added to the sum of diameters when the boundary of the lesion is identifiable, even if the lesion is in contact with each other.

11.1.7. Response Evaluation Criteria for Non-Target Lesions

・CR(Complete Response): Complete response

When all non-lymph node non-target lesions disappear, the short diameter of all nodal non-target lesions becomes less than 10 mm, and all tumour markers (NSEs, ProGRP※) are below the upper shared reference limits.

Because ※ProGRP is not included in the shared baseline range, the baseline range is 6.5-46.0 pg/mL.

・Non-CR/non-PD: non-CR/non-PD

Residual one or more non-target lesions (including residual nodal non-target lesions ≧10 mm in short diameter) and/or tumour markers (NSEs, ProGRP※) exceeding the shared upper reference limits.

・PD(Progressive Disease): Progress

'Apparent exacerbation' (including relapse) of pre-existing non-target lesions.

For measurable disease: A marked progression of a non-target lesion that deserves discontinuation of treatment as an increase in overall tumor burden must be observed if the effect of the target lesion is SD or PR but is judged to be "clear progression" based on the change in the non-target lesion. If the effect of the target lesion is SD or PR, then an increase in the tumor burden of the non-target lesion to a degree that far exceeds the decrease in tumor burden is considered "obvious progression" and otherwise Non-CR/non-PD.

If only unmeasurable disease is present, the increase in non-target disease, as judged to clearly exceed the tumor burden corresponding to a 20% increase in diameter and a 73% increase in tumor volume, is

considered "definite progression".

・NE(Not all Evaluated): Lack of study

If the test could not be done for any reason or if neither CR, Non-CR/non-PD nor PD could be determined.

11.1.8. Presence or absence of new lesions

If a lesion that was not present at baseline was observed after the start of treatment, it is considered "new lesion" to be present.

However, a "new lesion" requires that it is not an imaging change due to a difference in the imaging method from the baseline assessment or a change in the imaging modality, nor is it an imaging change due to a condition other than the tumor. For example, a cystic lesion arising within a lesion due to necrosis of a liver metastatic lesion is not a new lesion. New lesions will be defined as new lesions by examination of sites that were not mandatory at baseline (pre-enrollment study).

If a lesion disappears and later reappears, measurement is continued. However, the effect at the time the lesion reappears depends on the status of the other lesion. When the overall effect reappears after CR, the lesion is judged as PD at the time of reappearance. When the overall effect is PR or SD, on the other hand, once the disappeared lesion reappears, the diameter of the lesion will be added to the sum of the diameters of the remaining lesions to calculate the effect. That is, in the presence of many residual lesions, even if one lesion reappears after an apparent disappearance, it is not judged as PD by itself, and it is judged as PD when the sum of the diameters of all lesions meets the criteria for PD. This is because of the perception that the majority of lesions do not truly 'disappear' and are not only depicted by the limits of resolution of the imaging modalities used.

If there is a possibility of a new lesion but it cannot be determined, it should not be a new lesion, and imaging should be reexamined at a clinically relevant time. If a new lesion is confirmed by repeat imaging, the new lesion will appear based on the date of imaging at which the new lesion is confirmed.

11.1.9. Overall efficacy (Overall Response)

The overall response (Overall response) will be determined by combining the effects of target lesions, non-target lesions, and the presence or absence of new lesions every 6 weeks according to Table 11.1.9.a below. The overall effect in the absence of a non-target lesion at baseline will be determined by the effect of the target lesion and the presence or absence of a new lesion, and the overall effect in the absence of a target lesion at baseline will be determined according to the effect of a non-target lesion and the presence or absence of a new lesion according to Table 11.1.9.b.

Table 11.1.9.a. Overall efficacy at each time point for target lesions (with or without non-target lesions)

Target lesion	Nontarget lesions	New lesions	Overall effect
CR	CR	None	CR
CR	Non-CR/non-PD	None	PR
CR	Lack of study	None	PR
PR	Lack of Non-PD or study	None	PR
SD	Lack of Non-PD or study	None	SD
Lack of study	Non-PD	None	NE
PD (obvious progression)	Irrespective of	With or without	PD
Irrespective of	PD	With or without	PD
Irrespective of	Irrespective of	Present	PD

Table 11.1.9.b. Overall efficacy at each time point for patients with non-target lesions only

Nontarget lesions	New lesions	Overall effect
CR	None	CR
Non-CR/non-PD	None	Non-CR/non-PD
Lack of study	None	NE
PD (obvious progression)	With or without	PD
Irrespective of	Present	PD

11.1.10. Best overall effectiveness (Best Overall Response)

CR > PR > SD > PD > NE is considered good, and the best overall effect is the best overall effect throughout the entire course.

PD is defined when imaging cannot be determined due to exacerbation of obvious disease or death before the first response assessment. In addition, NE is defined if it cannot be determined by imaging due to discontinuation of toxicity before the first response assessment or patient refusal.

11.2. Definitions of analyses set

The analysis sets used in periodic central monitoring, interim analysis, and final analysis are defined as follows: The flow diagram below shows the analysis sets.

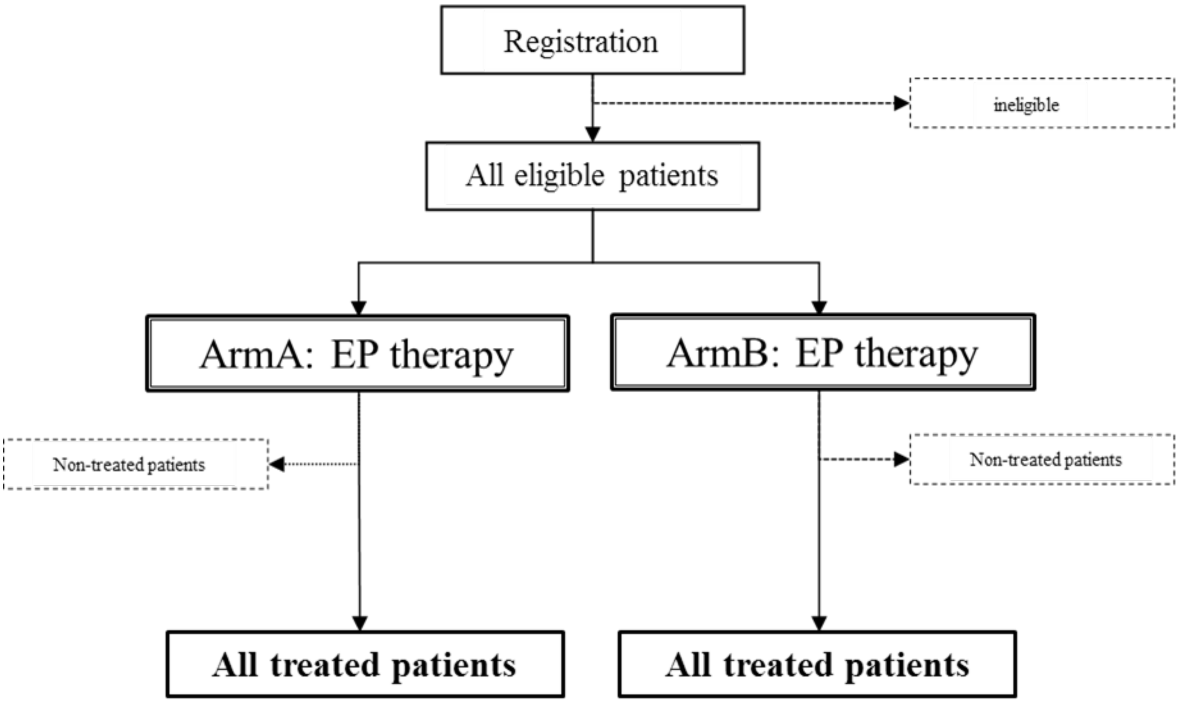

11.2.1. All registered patients

「5.1. Among the patients enrolled according to the Procedures for Enrollment, the population excluding duplicate or mis-enrollment is considered as "all enrolled cases".

11.2.2. All eligible patients

The group excluding "ineligible cases (post hoc ineligibles, de facto ineligibles, violation of registration)" determined by group review from all registered patients is regarded as all eligible patients. Ineligible cases as judged by the investigator or sub-investigator alone are included in all eligible patients. Only those judged not eligible by the central pathological diagnosis are ineligible and not included in all eligible patients.

11.2.3. All treated patients

Of all enrolled patients, all patients for whom part or all of the protocol treatment was performed will be defined as all treated patients.

The decision to treat "non-treated patients" for whom no protocol treatment has been given and whether it is excluded from the safety analysis can be determined by the data center with the consent of the Research Office. Ineligible patients will be excluded from all treated patients. However, if there are circumstances in which ineligible patients are included in the analysis, the nature of the ineligibility will be examined and determined by the Research Secretariat in consultation with JCOG Data Centre.

11.2.4. Patients eligible for central pathology diagnosis

Of all eligible patients, the population excluding ineligible patients with a central pathological diagnosis will be considered as eligible patients with a central pathological diagnosis.

11.3. Definition of endpoints

Endpoint	Events (whichever is earlier)		Censoring date
Overall survival time<br>Overall survival(OS)	All deaths	-	Date of final survival confirmation
Progression-free survival time<br>Progression-free survival(PFS)	All deaths	Progression/r ecurrence	Final date of clinically confirmed freedom from exacerbation

11.3.1. Overall survival

The duration from the date of registration to the date of death from any cause.

- Survivors are censored at the date of final survival confirmation (survival confirmation by telephone contact is also permitted, but the fact that survival confirmation was performed should be recorded in the medical record).
- Patients lost to follow-up are censored at the last date of survival confirmation before lost to follow-up.

11.3.2. Progression-free survival (PFS: Progression-free survival).

The duration from the date of registration to the date of the judgement of exacerbation or death from any cause, whichever comes first.

- The exacerbation (progression) includes both imaging-based PD (progression) and exacerbation of pathogenic disease (clinical exacerbation) that cannot be confirmed by imaging studies in Section 11.1.9. Global Effectiveness. If an exacerbation is judged based on diagnostic imaging, the exacerbation date is the test date on which the imaging was performed, and in the case of clinical exacerbation, the date of clinical judgment is the exacerbation date. PD may occur in accordance with the Response Evaluation Criteria, even if the tumor diameter is very small, but the disease is clinically judged to be "not obviously aggravated" in accordance with the Response Evaluation Criteria Criteria (in this case, clinical judgment should be prioritized for continued treatment with the protocol). In addition, even if PD is not observed according to the response evaluation criteria, the clinical judgment is prioritized for progression if it is judged to be clinically obvious exacerbation.
- In survivors who are not judged to have progression, clinically confirmed progression is censored (date of final progression-free survival confirmation) (confirmation of progression-free by imaging or specimen examination is not mandatory and clinical progression-free by outpatient examination etc. is not permitted. Telephone contact alone is not permitted. If information on progression or progression-free is obtained at a medical institution or referral center, receive and retain a medical information form describing the rationale for diagnosis. In this case, telephone contact alone is not permitted).
- Events and censoring are treated similarly, if chemotherapy is terminated for reasons such as toxicity or patient refusal, and if other therapies are added as post-study treatment. i.e., it is not censored at the time of treatment termination or at the date of initiation of post-study treatment.
- When the diagnosis of exacerbation is based on imaging, the relapse is not regarded as an event at the test date of imaging with "suspicious diagnosis of relapse", but regarded as an event at the later test date of imaging with "definite diagnosis of relapse"" If the event is judged to be clinically aggravated based on diagnostic imaging, the event is considered to be the day of the progression.
- If the definitive diagnosis of relapse or new lesion is based on biopsy pathology, the date of clinical diagnosis is defined as the date of clinical diagnosis when the diagnosis of recurrence or new lesion is made clinically, and the date of biopsy is defined as the date of event when the diagnosis of recurrence is made based on biopsy pathology diagnosis without clinical diagnosis of recurrence.
- The incidence of a second cancer (metachronous double cancer) is not censored or event, and progression-free survival is defined until other events are observed.

11.3.3. Response rate (response rate) Response proportion (Response rate)

Among all enrolled patients with measurable disease, the response rate is defined as the proportion of patients with "11.1.10. Best Global Effect" that is either CR or PR.

11.3.4. Incidence of adverse events (adverse reactions)

Using all treated patients as the denominator, the frequency of the worst Grade during the entire course by CTCAE v4 0 Japanese translation JCOG version for each of the following adverse events (toxicities) will be determined by group:

- Laboratory tests: hemoglobin decreased, white blood cell decreased, neutrophil count decreased, platelet count decreased,

  Increased blood bilirubin, aspartate aminotransferase increased (AST or GOT),
  Alanine aminotransferase increased (ALT or GPT), creatinine increased,
  Hypernatremia, hyponatremia, hyperkalemia, hypokalemia, hypercalcemia, hypocalcemia,

hypermagnesemia, hypomagnesemia

- General disorders and administration site conditions: fever, fatigue
- Ear and labyrinth disorders: Tinnitus, hearing impairment
- Skin and subcutaneous tissue disorders: alopecia
- Gastrointestinal disorders: constipation, diarrhea, nausea, vomiting, oral mucositis
- Metabolism and nutrition disorders: anorexia, dehydration
- Nervous system disorders: dysgeusia, peripheral sensory neuropathy, peripheral motor neuropathy
- Musculoskeletal and connective tissue disorders: myalgia, arthralgia
- Infections and parasites: bronchial infection, pulmonary infection, upper respiratory tract infection, catheter-related infection, biliary tract infection,
  Gallbladder infection; Mediastinal infection; Pleural infection; Urinary tract infection
- Blood and lymphatic system disorders: Febrile Neutropenia
- Musculoskeletal and connective tissue disorders: arthralgia, Vascular disorders: phlebitis
- Respiratory, thoracic and mediastinal disorders: dyspnea, hypoxia, pneumonitis

In the other adverse event (toxicitiy) than the above, the proportion of occurrence are not calculated unless a large number of specific adverse events are observed, since only Grade 3 or more non-hematological toxicity ※ is reported in the Treatment Form.

※※ Non-hematological toxicity refers to adverse events other than those listed below in CTCAE v4.0-JCJCOG.
Anemia, decreased bone marrow cells, decreased lymphocyte count, decreased neutrophil count, decreased white blood cell count, and decreased platelet count.
CD4 lymphocytopenia

11.3.5. Dose intensity of cisplatin

Dose intensity of cisplatin per patient (DOOC.) will be calculated for all treated patients to assess treatment compliance with cisplatin. The summary statistics (minimum, 25% point, median, 75% point, maximum, mean, and standard deviation) will be calculated for each group.

- ・Active dose D.I. (mg/m2/week) = total drug dosebody surface areatreatment duration (weeks)
- Body surface area: BSA is calculated by height at enrollment and body weight calculated by Data Center.
- ・ Treatment period (weeks)
  Arm A = (start date of last course-start date of 1st course + 21)/7
  Arm B = (start date of last course-start date of 1st course + 28)/ 7

11.3.6. Incidence of serious adverse events (adverse reactions)

1) Grade 4 non-hematologic toxicities, early deaths, and treatment

Using all treatments as denominators, the percentage of patients with one or more Grade 4 non-hematologic toxic ※ that are considered to be related (either to definite, probable, possible) to the protocol treatment among the adverse events listed in the free text of the CRFs in addition to the stereotyped items in Section 11.3.4 is used as the numerator.

※※ Non-hematological toxicity refers to adverse events other than those listed below in CTCAE v4.0-JCJCOG.
Anemia, decreased bone marrow cells, decreased lymphocyte count, decreased neutrophil count, decreased white blood cell count, and decreased platelet count.
CD4 lymphocytopenia

2) Early mortality rate

Proportion of all deaths during the protocol treatment or within 30 days from the last protocol treatment day among all treated patients. Causes of death irrespective of causality with protocol treatment. However, if premature death occurs in patients excluded from all treated patients, the details are provided separately.

3) Proportion of treatment-related death (TRD incidence)

Proportion of all deaths judged as causally related (either definite, probable, possible) to the protocol treatment among all treated patients However, if TRD occurs in patients who have been removed from all treated patients, the details are presented separately.

12. Statistical consideration

Methods for statistical analysis are as follows: In addition, the details required for conducting specific analyses are specified in the statistical analysis plan prepared separately prior to the analysis, and in documents that clarify the endpoint definition, etc. If substantial changes occur in statistical analyses as described below, follow the policy set out in "13.6. Protocol Changes." Facilities are contacted by "14.1. Periodic Monitoring" for missing or abnormal results, and data collection or exclusion is determined by review by the Research Secretariat based on the results of inquiries to the institution in accordance with the policies stipulated in "14.1.2. eligible (eligibility/ineligibility)" and "14.1.3. Protocol Deviations/Violations". 「11. Handling of missing values and abnormal data that cannot be addressed by the definition and analysis method for each endpoint, as specified in "Definition of Response Evaluation and Endpoints" and "12. Statistical Items" below, is specified in the above statistical analysis plan.

12.1. Principal Analysis and Decision Criteria

The primary analysis of this study will be the final analysis.

The purpose analysis of this trial is to test whether one of the two community standard-of-care arm A (EP-therapy) and B (IP-therapy) outperforms primary endpoint overall survival. The null hypothesis of equal overall survival in the two groups in the main analysis will be tested by stratified log-rank test stratified by non-institutional allocation adjustment factors (primary organ [gastrointestinal vs. hepatobiliary-pancreatic]) in all patient patients. However, if it is assumed that a stratified log-rank test cannot be performed appropriately, such as when the number of subjects and events in each stratum is small, the allocation adjustment factors will be addressed in the analysis plan prepared without information related to the comparison between groups before performing a confirmatory analysis with comparison between groups. Sensitivity analysis will also be performed in all eligible patients and in patients eligible for central pathology diagnosis.

Due to interest in which treatment group is superior, the test will be two-tailed. The study-wise significance level for the study is set at both sides 10%. In the main analysis, two-sided 90% confidence intervals corresponding to a two-sided 10% significance level will be calculated, and in other analyses, two-sided 95% confidence intervals will be calculated for descriptive purposes.

We conclude that EP therapy is a more useful treatment when the two-sided p-value is less than 10% and the survival curve of EP therapy exceeds that of IP therapy. Meanwhile, we conclude that IP therapy is a more useful treatment when the two-sided p-value is less than 10% and the survival curve of IP therapy exceeds that of EP therapy. If the difference was not significant at a two-sided significance level of 10%, one of the two modalities cannot be judged to be useful. In this case, we conclude that both modalities continue to be the standard of care, as there is no evidence to actively recommend either one of them.

However, if there are major differences in the toxicity profiles of EP and IP therapies, contrary to prior assumptions, during the course of the study, and if the toxic therapy is not superior to other therapies, it is judged that it will not be of significance to be used in clinical practice, the following measures should be taken. In other words, the clinical hypothesis will be changed from two-sided to one-sided in an analysis plan with no information on group comparisons before a confirmatory analysis with group comparisons will be conducted, and the superiority of the toxicity treatment over other treatment modalities will be verified at a one-sided significance level of 5% (in this case, the policy of change for the interim analysis will be described in Section 12.3.2).

Estimates such as cumulative survival curves, median survival times, and annual survival rates are performed using Kaplan-Meier method. Brookmeyer and Crowley methods are used to obtain 95% confidence intervals for median survival. Greenwood's formula is used to obtain 95% confidence intervals for annual survival rates. Hazard ratios and their confidence intervals for treatment effects between groups are obtained using stratified Cox proportional hazards models with the same factors as the test of the primary analysis as estimates of treatment effects. Cox regression adjusted by imbalance background factors in addition to adjustment factors will be performed as needed.

The main analysis results will be summarized as the "Main Analysis Report" by the Data Center one year after the completion of enrollment and submitted to the Research Secretariat, Research Representatives, Group Representatives, Group Secretariats, Efficacy and Safety Assessment Committee, and JCOG Representatives.

The principal investigator/study coordinator summarizes the content of the main analysis report, prepares a

"Clinical Study Report" summarizing the conclusions, problems, interpretations and discussion of the results, and future policies of the entire study, and submits it to the Data and Safety Monitoring Committee and JCOG chair with approval from the Group chair and the Head of JCOG Data Center.

Approval of the clinical study report by the Data and Safety Monitoring Committee shall be considered as "end of the study."

12.2. Planned accrual, accrual period, and follow-up periods

「2.4.2. Based on the background presented in Clinical Hypothesis and Rationale for Number of Enrollment, we assume a median survival of 8 and 12 months (HR=0.67) for the inferior and superior treatment groups, respectively, among the two treatment groups. When a superiority trial design is used, 63 patients per group and 126 patients in both groups (114 events required) will be included in the required analysis using Schoenfeld & Richter methodological [50] at 6 years of enrollment, 1 year of follow-up, α = 10% (two-sided), and 70% power. The number of required Inferior treatment (number of required events) when the median survival time in A is different from the assumption as shown in Table 12.2.1 below.

Table 12.2.1. Number of Analyses Required (Number of Events Required)

	Power			
Median Survival (mo)	65%	70%	75%	80%
7.0 vs. 10.5	108(100)	124(114)	142(131)	162(150)
8.0 vs. 12.0	112(100)	126(114)	144(131)	166(150)
9.0 vs. 13.5	114(100)	130(114)	148(131)	170(150)

※※ Number of required events in parentheses

Based on these findings, the following will be established in view of some cases of loss to follow-up.

Planned enrollment: 70 patients in each group and 140 patients in both groups

Enrollment period: 6 years, follow-up period: 1 year after completion of enrollment

Consider redesigning the sample size if the prognosis is obviously better than assumed, or if it reaches 70 people within 2.5 years of enrollment initiation. Clinically meaningful differences will then be reviewed and redesigned in a blinded fashion prior to the conduct of the analysis.

<Additions in ver. 1.1>

The registration pace after the enrollment start was smooth and exceeded the plan, and 70 cases were reached in October, 2016, when 2 years and 2 months passed from the enrollment start. Since it exceeded the standard of 70 patients within 2.5 years from the initiation of enrollment specified above, acceptance was obtained at the meeting of the Hepatobiliary and Pancreatic Group on October 29, 2016, the group meeting of the Esophageal Cancer Group on November 19, 2016, and the group meeting of the Gastric Cancer Group on January 7, 2017 with respect to changing the power to 80% according to the rules at the time of the study plan. Therefore, the planned enrollment number was changed to 170. In addition, the "1-year analysis period" was added to the study period, and the following changes were made.

Planned enrollment: 85 patients in each group and 170 patients in both groups

Enrollment period: 6 years; Follow-up period: 1 year after completion of enrollment; Analysis period: 1 year; Total study period: 8 years

12.3. Interim Analysis and Early Termination of the Study

12.3.1. Purpose and Timing of the Interim Analysis

Once interim analyses will be conducted to determine if the primary objective of the study has been achieved during the study period. Interim analyses will be conducted to determine if it is reasonable to continue enrollment during enrollment. If the primary objective of the study is determined to be achieved, the study will be discontinued and the study results will be published promptly at the conference and in the article.

Interim analyses will be conducted using data from the initial periodic monitoring that will be queried after the time enrollment of half of the planned enrollment was obtained. Based on the information in the periodic monitoring report, the group will submit the presence or absence of changes in clinical hypotheses and, if any, details of changes to the Efficacy and Safety Assessment Committee by the time of the interim analysis.

During the first interim, patient accrual is continued in principle. If the progress of the study progressed as planned, the expected number of events at the time of the interim analysis under the assumptions presented in 12.2 is expected to be 44 if the interim analysis is conducted at 3 years after the start of enrollment.

12.3.2. Method of interim analysis

Interim analyses will be conducted by the JCOG Data Center. To keep the study-wise alpha errors at 10%, the multiplicity of the interim and final analyses is adjusted using Lan & DeMets's alpha spending functions, and statistical significance is examined for differences in primary endpoint between arms. As α spending functions, we use O'Brien & Fleming types.[51]

For details of the interim analysis, the statistical staff in charge of the group at the Data Center will prepare the statistical analysis plan by the time of the interim analysis. Actual interim analyses will be performed by statistical staff who are not in charge of the group and an interim analysis report will be prepared.

In the interim analysis, if the survival of one treatment group exceeds that of the period, and the p-value of the stratified log-rank test falls below the level specified by the above method, it is judged statistically significant and the trial is discontinued as a rule. The ineffective discontinuation is not planned at a stage where no statistically significant differences are observed unless prior assumptions regarding toxicity are changed. However, the clinical hypothesis may be changed for the reasons described in 12.1, and the primary analysis may be performed by one-sided rather than two-sided testing, which may result in deviations from prior assumptions. In such cases, the primary hypothesis change should be specified in the analysis plan to be prepared without information on group comparisons before a confirmatory analysis with group comparisons. If the overall survival curve in the highly toxic treatment group is below the other, the consideration of ineffective discontinuation should not be judged by a test and considered comprehensively.

12.3.3. Interim analysis Reporting and review of the results

The results of the interim analysis will be submitted to the Data and Safety Monitoring Committee by the Data Center as an Interim Analysis Report and reviewed for the acceptability of continuation of the study and for publication of the results. The Data and Safety Monitoring Committee considers whether to continue the study at the meeting and recommends whether to continue the study and whether to publish the results to principal physician or group chair based on the results of the review.

Members of the Data and Safety Monitoring Committee of the relevant group are not included in the review. Unless the results of the interim analysis make recommendations for discontinuation of the study from the Efficacy and Safety Assessment Committee, the research representative, research office, participating institution researchers, group representatives, and group secretaries of the study will not be able to know the results of the interim analysis until the final follow-up is completed.

When the Interim Analysis Report has been reviewed by the Data and Safety Monitoring Committee to recommend termination or change of all or part of the study, the principle investigator and group chair review the recommendations and decide whether to discontinue or change some of the study.

If the study is discontinued or part of the study is changed, the principal investigator and group chair shall submit in written form a request to the Data and Safety Monitoring Committee for permission to discontinue the study or a request to revise the protocol. Following approval by the Data and Safety Monitoring Committee, the principal investigator may discontinue the study or change part of the study.

The Study Chair and Group Chair can disagree with the recommendations of the Data and Safety Monitoring Committee, but if they fail to coordinate their opinions with the Data and Safety Monitoring Committee, they will ultimately follow the instructions of JCOG Chair.

If the study is terminated, the subsequent follow-up period will be 1 years from last registration per study.

If the interim analysis resulted in study termination, the interim analysis will be the primary analysis of the study. The Data Center, in cooperation with the Research Representative Physicians and Research Secretariat, will conduct the analysis required to complement the incomplete data and publish the results, focusing on the results of the interim analysis, and promptly prepare the Major Analysis Report and submit it to the Group and the Efficacy and Safety Assessment Committee.

12.4. Analysis of Secondary endpoints

Secondary endpoints analyses will be conducted to provide a supplementary discussion of the primary analysis results of the study. Because the analysis of secondary endpoint is exploratory, no multiplicity adjustments are made. Comparisons between arms are made where appropriate, note that when the results of the group comparisons are not significant, they do not mean that there is no difference between the two arms.

12.4.1. Analysis of safety secondary endpoints

Among Secondary endpoints, the safety endpoints are the incidence of adverse events and the incidence of serious adverse events, which are in principle the items of periodic monitoring (14.1. Periodic monitoring).

The incidence rate of adverse events will be summarized as well as the incidence rate of Grade3 or higher. For adverse events other than laboratory data, the incidence of Grade 2 or higher is also calculated.. Non-hematological toxicity incidence, early mortality, and treatment-related mortality rates of Grade4, which are serious adverse events, are reported in periodic monitoring reports with registration numbers and details. The rates of non-hematologic toxicity, early mortality, and treatment-related mortality for Grade4 will be calculated at the time of the interim analysis and the main analysis. When interval estimation of proportions is performed, accurate confidence intervals based on binomial distributions are used. Comparisons between arms will be made using Fisher's exact test where appropriate.

12.4.2. Analysis of efficacy secondary endpoints

Among Secondary endpoints, efficacy endpoints are response rate, progression-free survival, which will only be analyzed in the interim and primary analyses.

Secondary endpoints analyses do not adjust for multiplicity.

Progression-free survival will be included in all enrolled patients, but a comparison of all eligible patients, excluding ineligible patients, as determined by group study, will also be performed as a sensitivity analysis.

The response rate will include all enrolled patients with measurable disease, but a comparison of all eligible patients excluding ineligible cases determined after group review will also be performed as a sensitivity analysis.

Fisher's exact test will be used to compare response rates between groups, and binomial distribution-based exact confidence intervals will be used for interval estimation. Estimates, including progression-free survival curves, median progression-free survival, and time-point progression-free survival, will be performed using Kaplan-Meier method, Brookmeyer and Crowley methods will be used to obtain confidence intervals for median progression-free survival, and Greenwood formulas will be used to obtain confidence intervals for progression-free survival. Log-rank test is used for comparison between arms. Hazard ratios and their 95% confidence intervals for treatment effects between arms will be calculated using Cox's proportional hazards model as an estimate of treatment effect. Cox regression adjusted by imbalance background factors in addition to adjustment factors will be performed as needed.

12.5. Final analysis

The primary analysis will be the final analysis unless this study is withdrawn from the interim analysis.

If the interim analysis is withdrawn from the study, then after the end of the 1-year follow-up period, the final analysis will be performed after the final survey confirms the data and then analyses will be performed for all endpoints.

Except for the interim analyses and the final analysis, analyses with between-arms comparisons for the primary and secondary endpoints for efficacy are not performed unless approved by the Data and Safety Monitoring Committee.

If the final analysis is performed after the main analysis, the final analysis will be summarized by the Data Center as the "Final Analysis Report" and submitted to the Research Secretariat, Research Representatives, Group Representatives, Group Secretariats, Efficacy and Safety Assessment Committee, and JCOG Representatives.

The Study Representative Physician/Research Secretariat summarizes the content of the final analysis report, prepares the "Clinical Study Report" summarizing the conclusions, issues, interpretations and discussion of the results, future policies, etc. mainly from the clinical point of view (if the "Clinical Study Report" is prepared in the previous analysis report, it will be the "Clinical Study Report (Supplementary Version)) with additional updates), and submits it to the Study Representatives and JCOG Representatives with approval from the Group Representatives and the Head of JCOG Data Center.

Approval of the clinical study report by the Data and Safety Monitoring Committee shall be considered as "end of the study."

12.6. Exploratory analysis

To investigate the interaction between treatment effect and the subpopulation, subgroup analyses will be conducted exploratory with respect to the following factors: Because these analyses are not adequately powered and do not adjust for multiplicity, the results of each subgroup analysis should be interpreted as exploratory.

- PS0/1
- Age 65 years or older/<
- Gender (male/female)
- Primary organ (gastrointestinal tract/hepatobiliary pancreas)
- Organ of origin (esophagus/stomach/small intestine/large intestine/pancreas/biliary tract/liver NEC (liver primary or liver metastasis of unknown primary)
- Organ of origin (pancreas/non-pancreas)
- Extent of extension of the primary lesion (locally advanced/distant metastasis or recurrence)
- Extent of extension of the primary lesion (locally advanced/distant metastasis/recurrence)
- Prior radical resection of the primary lesion (none/present)
- Pathological diagnosis was biopsy/resection specimen
- Grade 3 tumour with morphologically similar features of NETs but Grade 3 proliferative activity/morphologically more atypical (previously classified as poorly differentiated endocrine carcinoma) on histopathology with central pathology
- Histopathological examination by central pathological diagnosis, including Small cell carcinoma/Large cell carcinoma /
- Histopathologically diagnosed by central pathology, Ki67 50% or higher/less than 50%

12.7. Premature withdrawal from the trial

In this study, early termination of the study may occur in the following cases:

1) Early termination due to discontinuation of interim analysis
2) Early termination due to adverse events
3) Early termination due to poor enrollment
4) Early termination due to other reasons

12.7.1. Early termination by interim analysis

In this study, based on the criteria described in 12.3, early termination recommendations may be made at the interim analysis review by the Efficacy and Safety Assessment Committee. If the Data and Safety Monitoring Committee provides recommendations for early termination of the study, the principle investigator and group chair will review the recommendations and decide whether to terminate the study early.

12.7.2. Early termination due to adverse events

In JCOG9511 for small-cell lung cancer, 1/77 (1.3%) treatment-related deaths were reported with EP therapy and 3/77 (3.9%) with IP therapy; in JCOG0509, a successor study for the same subject, 1/142 (0.7%) treatment-related deaths were reported with IP therapy and 2/142 (1.4%) with amrubicin plus cisplatin therapy. Using these as reference, we believe that the treatment-related mortality rate should not exceed 3% in this study. Since it is clear that the final point estimate will be at least 3% at the time of 3 treatment-related deaths in either group, immediate entry should be suspended to consider whether or not to withdraw from the study. At this point, the subsequent treatment of the patient being treated will be reviewed. At the time of 3 or fewer treatment-related deaths in each group, each patient will be reported to the Efficacy and Safety Assessment Committee for adjudication. Enrollment will be continued until the results are obtained in principle.

12.7.3. Early termination due to poor enrollment

If the patient enrollment pace is significantly worse than at the time of planning, early termination of the study may be advised by the Data and Safety Monitoring Committee. If early termination recommendations are issued by

the Data and Safety Monitoring Committee due to poor enrollment, the principle investigator and group chair will review the recommendations and decide whether to terminate the study early.

12.7.4. Early termination due to other reasons

12.7.1.～12.7.3. If it is judged difficult to continue the study for other reasons, the research representative physician shall submit a request for early termination of the study to the Efficacy and Safety Assessment Committee. If the Data and Safety Monitoring Committee recommends early termination of the study based on the submitted data, the procedure for early termination of the study will be progressed.

12.8. Procedures after Early termination of the Study

If the Study Chair accepts the recommendations made by the Data and Safety Monitoring Committee based on Section 12.7, he/she will promptly submit a notification to the Data and Safety Monitoring Committee that early termination of the study will be performed.

The Study Chair will submit a termination notification to the Certified Review Board within 10 days of the date they decide to terminate the study early. If the study falls into a specified clinical trials under the Clinical Trials Act, the Study Chair shall submit a termination notification to the Certified Review Board within 10 days of the date on which the study was decided to be prematurely discontinued, as well as submit a specified clinical trials termination notification to the MHLW.

The Study Chair promptly informs the investigator of the decision to terminate the study early in writing, and the investigator who has received a report of early termination of the study will report in writing that the study was prematurely terminated to the institution's manager without delay.

If the study is terminated early, JCOG Data Center will promptly initiate the development of the primary analysis report or final analysis report. The subsequent follow-up period will be 1 year from the final enrollment.

13. Ethical Considerations

13.1. Protection of Human Subjects

All researchers involved in this study will conduct this trial in accordance with the "Helsinki Declaration" [1] and "Clinical Trials Act" (2017 Law No. 16) [2] "Clinical Trials Act Enforcement Regulations" (2018 Ministry of Health, Labour and Welfare Order No. 17) and related notices.

1） http://dl.med.or.jp/dl-med/wma/helsinki2013j.pdf

2） http://www.mhlw.go.jp/stf/seisakunitsuite/bunya/0000163417.html

Prior to commencing this study, the site investigator is required to obtain input from Certified Review Board[※1] regarding the conduct of the study, and to obtain approval from the Administrator of the participating institution, and submit the Implementation Plan [※2] to the Ministry of Health, Labour and Welfare.

※ 1 JCOG study will be submitted to the following Certified Review Board.
National Cancer Center Hospital Certified Review Board (accreditation number CRB3180008)

※ 2 "Implementation Plan" means "Documented plan formatted according to the Form No.1 (Form No.1 of the Ministerial Ordinance) specified in Article 39 of the Clinical Trials Act Enforcement Regulations"

13.2. Informed consent

13.2.1. Explanation to the patient

Prior to patient registration, the investigator or subinvestigator will provide the patient with written informed consent form approved by Certified Review Board and explain the following details verbally.

Descriptions

1） Disease names, Stages, and expected prognosis

2） That this study is a clinical trial and is conducted by JCOG
Name of Certified Review Board and contact information for receiving complaints and inquiries to the committee

3） Design and rationale of the study

4） Protocol treatment content

5） Effects expected by protocol treatment

6） Expected adverse events, complications, and sequelae and how to deal with them
Explanation of the extent and frequency of expected adverse events, including complications, sequelae, and treatment-related deaths, and how to deal with them when they occur. In addition to these explanation, obtain the most recent version of the drug package insert and deliver it to patients (PMDA Prescription Pharmaceutical Information Search http ://www.pmda.go. jp/PmdaSearch/iyakuSearch/)

7） Post-study treatment after end of protocol treatment should also be performed appropriately

8） Cost burden and compensations
Explanation of the cost of treatment, compensation that can be received in the event of a health hazard (equivalent to measures taken in general practice, etc.)

9） Alternative treatment
Explanation of treatments that can be received if not participating in this study

10） Anticipated benefits and possible disadvantages
Explanation of anticipated benefits and possible disadvantages by participating in this study

11） Direct access to the medical records
Explanations on acceptance of site visit audits, such as "direct access to medical records etc. by healthcare professionals at other medical institutions for quality control with permission from the administrator of the participating institution."

12） Refusal of consent and withdrawal of consent
Refusal to consent prior to participation in the study is free, and withdrawal after having given consent is free, thereby not causing undue medical disadvantage.

13） Protecting human rights
Every effort should be made to ensure that personal information, such as names, is kept confidential.

14） Secondary use of data

The possibility of secondary use of data obtained from this study in Japan and overseas (ancillary studies, meta-analyses, etc.) only when approved by either committee in JCOG

15） Method of disclosure of information on the study
The study is registered and published in jRCT※. In addition, the results of clinical studies should also be published in jRCT (＊ Databases (Japan Registry of Clinical Trials) https://jrct.niph.go.jp/ prepared by the MHLW as stipulated in Paragraph 1 in Article 24 of Clinical Trials Act Enforcement Regulations)

16） Freedom of questions
Explanation that investigators, written contact information for consultations on study details, on the Principal Investigator and the Study Coordinator, and freely asking questions about study and treatment

17） Explanation of the use and burden of medicinal products not covered by insurance

18） Central pathological review

19） Central image review for response

20） Sample collection for ancillary studies

13.2.2. Consent

Explain the study, give sufficient time to think, confirm that the patient understood the study well, and ask for participation in the study. If the patient agrees to participate in the study, the written informed consent form in the appendix will be used to obtain the patient's own signature. The site investigator or the subinvestigator confirms that the study consent form contains the name of the physician who provided the explanation and the date of explanation, the name of the patient who gave informed consent, and the date of informed consent.

In addition, when it is not possible to read the documents due to visual impairment, etc., but the details can be understood by verbal explanation, or the documents can not be signed due to limb disorders, etc., but the documents can be read and understood, signatures may be obtained from the proxy author under the consent by the patient. However, the signature of the proxy author should be based on this study's consent, and should be described as "Signing by the proxy author" and "Relationship with the patient" so that the person can be found to be the proxy author.

Two copies of the consent form will be provided, one will be handed over to the patient, and one will be stored by the site coordinator. Original copies will be stored in the medical records or in the archives specified by the participating institution.

13.2.3. Response to inquiries, consultations, etc. after consent

In principle, the investigator or subinvestigator of the relevant patient's participating institution responds to any consultation related to the study by the patient or his/her family after registration. If it is unclear how to respond, respond in consultation with Principal Investigator, Study Coordinator, the Group Secretariat, Group Chair, JCOG Data Center/Operations Office, etc. in accordance with the content of the consultation.

13.2.4. Withdrawal of consent

After obtaining informed consent for participation in the study, consent will be withdrawn if the patient expressed withdrawal of consent.

Withdrawal of consent refers to withdrawal of consent to participate in research and is distinguished from refusal to continue protocol treatment (below (i)). If the withdrawal of consent is expressed, clarify whether (ii) or (iii) below and promptly notify JCOG Data Center. If consent is withdrawn, record it in the medical record as well as (ii) or (iii).

Data Center discontinues subsequent follow-up requests according to the protocol in case of (ii) withdrawal of consent, In the case of (iii) full withdrawal of consent, the data of the patient are removed from the database when it is confirmed that the patient has full withdrawn of consent.

The procedures for discontinuation of the patient's follow-up request and removal of patient data will be specified separately in the procedural manual, and the completion of each task will be reported to Principal Investigator and Study Coordinator.

(i) Patient refusal: Refusal to continue subsequent protocol treatment (follow-up continues).

(ii) Withdrawal of consent: Withdrawal of consent to participate in the study and termination of all subsequent treatment and follow-up in accordance with the study protocol. Research use of data prior to withdrawal of consent is permitted.

(iii) Full withdrawal of consent: Withdrawal of consent to participate in the study and unavailability of all data from the time of patient registration, including information at registration.

In addition, some medical institutions may request that a "withdrawal of consent" form be prepared as a written document. However, in the event of withdrawal of consent, written expressions of willingness are required to increase the psychological barriers to withdrawal of consent (i.e., it is difficult to withdraw consent), and it is considered unwanted from the viewpoint of protecting human subjects. Therefore, in JCOG, written expressions of willingness are not mandatory for withdrawal of consent, verbal withdrawal of consent is valid, and the "withdrawal of consent" form is not prepared. If "withdrawal of consent" form is required by the participating institution, it should be prepared by the institution.

- This should be used in studies conducted in accordance with the Ethical Guidelines for Medical and Health Research Involving Human Subjects.

The procedures for discontinuation of the patient's follow-up request and removal of patient data will be specified separately in the procedural manual, and the completion of each task will be reported to Study Chair and Study Coordinator.

(i) Patient refusal: Refusal to continue subsequent protocol treatment (follow-up continues).

(ii) Withdrawal of consent: Withdrawal of consent to participate in the study and termination of all subsequent treatment and follow-up in accordance with the study protocol. Research use of data prior to withdrawal of consent is permitted.

(iii) Full withdrawal of consent: Withdrawal of consent to participate in the study and unavailability of all data from the time of patient registration, including information at registration.

In addition, some medical institutions may request that a "withdrawal of consent" form be prepared as a written document. However, in the event of withdrawal of consent, written expressions of willingness are required to increase the psychological barriers to withdrawal of consent (i.e., it is difficult to withdraw consent), and it is considered unwanted from the viewpoint of protecting human subjects. Therefore, in JCOG, written expressions of willingness are not mandatory for withdrawal of consent, verbal withdrawal of consent is valid, and the "withdrawal of consent" form is not prepared. If "withdrawal of consent" form is required by the participating institution, it should be prepared by the institution.

13.3. Protection of personal Information and patient identification

JCOG recognizes that information on privacy, such as personal information and medical information, should be protected and handled carefully, based on the principle of respecting the personality of individuals, and has formulated JCOG Privacy Policy, and will take all possible measures to protect privacy. For more information, see JCOG website (http://www.JCOG.jp/).

13.3.1. Policies, legislation, and norms followed by JCOG

In conducting JCOG study, JCOG follows, in principle, JCOG Privacy Policy as well as the following laws and norms depending on the content of the research. If other laws, norms, and policies are applicable, they should be followed.

- Clinical Trials Act (Law No. 16, 2017).
- Act on the Protection of Personal Information (Law No. 57, 2003, Final Amendment: Law No. 65, September 9, 2015).
- Helsinki Declaration (Translation by the Japan Medical Association)
- Ethical Guidelines for Medical and Health Research Involving Human Subjects (No.1 of Notice of the Ministry of Education, Culture, Sports, Science and Technology and the Ministry of Health, Labour and Welfare, 2017).

13.3.2. Use of personal information objective, items to be used, and methods of use

1) Objective of use

In accordance with the basic philosophy "Providing the best treatment to more patients," JCOG uses personal information, etc. of patients for objective of "Identifying patients and conducting surveys not only during treatment but also for a long period after end of treatment in order to obtain the correct results of clinical studies, and appropriately managing the acquired information."

2) Items to be used

Information to identify individuals who will be used by JCOG as minimally require for identification and inquiry of patients is as follows.

Medical record number, date of birth, initials, and registration number, Pathology specimen number

In other words, information that can identify individuals other than those listed above, such as the patient's name, is not informed to Data Center by participating institutions, and if they are falsely informed, they should be destroyed without using the recording medium or stored after performing appropriate processing, such as masking, which is incapable of reading.

3) Method of use

Personal information of patients used by JCOG will be collected by entering the CRFs etc. by researchers at participating institutions and submitting them to Data Center either by JCOG Web Entry System, mailing, or handover as a rule. However, telephone calls will be used only for patient registration where prompt contact is necessary.

In addition, in order to confirm the accuracy of the collected information, inquiries regarding various types of CRFs, including personal information, between Data Center and researchers at medical institutions are limited to either JCOG Web Entry System, mailing, or handover. Only the more anonymous registration number should be used when interacting with e-mail inquiries, and medical record numbers and initials should not be used.

13.3.3. Preparation of records for provision of samples and information, etc. at participating institutions

The investigator at each site will prepare records for this study of enrolled patients. The matters prescribed by the Ordinance of the Ministry of Health, Labour and Welfare (Article 53 of Clinical Trials Act Enforcement Regulations) are as follows.

- Identification of clinical-research subjects
- Items related to medical care and testing for subjects personnel in clinical studies
- Items related to participation in clinical research
- Other matters necessary to conduct clinical research

13.3.4. Source documents used in clinical studies (JCOG trials conducted under Clinical Trials Act)

The source documents for clinical studies used in this study refer to all records used for diagnosis and treatment, including medical records (including worksheets, etc.), laboratory test records, diagnostic images used for diagnosis, pathological diagnosis report, images used for response evaluation, and informed consent documents, of enrolled patients in this study. These should be available for direct access for monitoring (14.1.) and audit (14.2.) conducted by Data Center, Operations Office, Certified Review Board and regulatory persons

13.3.5. Storage of samples and information

Samples and information of enrolled patients on this study shall be stored in accordance with Article 53 of Clinical Trials Act Enforcement Regulations (MHLW ordinance No. 17, 2018). The retention time of records related to this study at participating institutions and the retention time of source documents will be 5 years from the date of completion of the study. It is recommended that the records be stored for as long as possible after the expiration date.

The retention time of data collected in JCOG Data Center will be semi-permanent in view of the possibility of long-term follow-up and secondary use for other studies. In addition, as records for the provision of samples and information, the study protocols and the model informed consent forms will be stored semi-permanently in JCOG Data Center.

13.3.6. Anonymization and control of response tables

In JCOG studies, information that can clearly identify individuals, such as patients' names, is not collected, and individuals are identified using registration numbers and medical record numbers (anonymized). Correspondence tables (not always in the form of a table) of information and registration numbers that can clearly identify individuals by themselves, such as patient names, are positioned as present at each participating institution and are appropriately managed according to the policy of participating institutions so that the identification of enrolled patients can be ensured.

13.3.7. Secondary use of data

Data from this study may be used in Japan or abroad for secondary use (e.g., meta-analysis) only if approved by the relevant committee of JCOG (e.g., Protocol Review Committee, Data and Safety Monitoring Committee). However, when providing data to external organization(e.g., a meta-analysis), data are provided so that it is not possible to identify individuals.

Secondary use of data should be disclosed on JCOG website to ensure opportunities for patients to refuse using data.

13.3.8. Safety management responsibility system

JCOG Data Center establishes a privacy protection control manager and a privacy protection manager and takes various safety control measures to minimize the risks of information leakage when using personal information, etc.

13.3.9. Response to request of disclosure of patient information

In the event that JCOG is asked to disclose privacy-related information, etc., the responder shall, in principle, be a researcher (site investigator, subinvestigator) at the institution of the relevant patient.

13.3.10. Receipt of inquiries about JCOG

General inquiries and complaints about privacy policies will be received by either mail, e-mail, or fax below.

Inquiry liaison: JCOG Data Center Privacy Protective

Postal destination :〒104-0045 Tsukiji 5-1-1, Chuo-ku, Tokyo
Clinical Research Support Office, National Cancer Center Hospital
E-mail :JCOG_privacy@ml.JCOG.jp
FAX :03-3542-3374

13.4. Adherence to the protocol

Researchers participating in this study will adhere to this protocol unless they compromise patient safety and human rights.

13.5. Application to Certified Review Board and Notification of Implementation Plans

When conducting this study, the approval of Certified Review Board and the permission by the administrator of each institution to conduct the study using this protocol and written informed consent from patients must be obtained. Prior to the commencement of the study, the implementation plan ※1 shall be submitted to the Minister of Health, Labour and Welfare, and the test data shall be published to jRCT※2. Principal Investigator is responsible for applying to Certified Review Board, submitting Implementation Plans to the MHLW, and registration in jRCT, and JCOG Operations Office supports these application procedures.

※ 1 Form No.1 of the Ministerial Ordinance stipulated in Article 39, Paragraph 1 of the Ordinance for Clinical Trials Act Enforcement Regulations

※ 2 Databases (Japan Registry of Clinical Trials) prepared by MHLW as specified in Article 24, Paragraph 1 of Clinical Trials Act Enforcement Regulations https://jrct.niph.go.jp/

13.5.1. Procedures for new application

<u>1) Procedures performed by Principal Investigator</u>

<Procedures from initial submission to study initiation>

(i) Review and approval of the study protocol by JCOG Protocol Review Committee (ver.1.0.0).

(ii) Principal Investigator will prepare the following documents for this study and submit these documents to Certified Review Board for review through JCOG Operations Office.

- ➢ New Review Request Form (Unified Form No.2 for Clinical Research)
- ➢ Implementation Plan (Ministerial Ordinance Form No.1)
- ➢ Study plan (this protocol) (including response to "disease or the like")
- ➢ Informed consent form
- ➢ Documents describing summary of drugs, etc. (e.g., package inserts of drugs used as a part of protocol treatment)
- ➢ List of Subinvestigators (Unified Form No.1 for Clinical Research)
- ➢ Conflict of Interest Management Standard (Guidance Form A), Conflict of Interest Management Plan (Guidance Form E) (see 13.X.)
- ➢ Other documents to be submitted when prepared (review documents of JCOG Protocol Review Committee, sample Case Report Forms, and a draft agreement on funding with the marketing authorisation of drugs, etc. or its special associates)

(iii) Modifications such as protocols and informed consent forms are made to review opinions submitted by Certified Review Board as needed.

※ Response to the indications by Certified Review Board: Prepare modified versions with review and approval by the Director of Data Center if modification of the protocol or informed consent forms is needed (ver.1.0.1, ver.1.0.2, ver.1.0.3…). .

(iv) After Certified Review Board approval is obtained, JCOG Operations Office should have Certified Review Board approval date and the approved version number on the cover page of the protocol and informed consent form and appear on JCOG website's Protocol Download page.

(v) Principal Investigator will use Certified Review Board review results notification and the documents submitted in ② to obtain the approval of the administrator of the institution which he/she belongs to, send these documents to the site investigators of all participating institutions described in the Implementation Plan, and request that the administrators of each participating institution permit conduct the research.

(vi) JCOG Operations Office shall apply for jRCT registration under the supervision of Principal Investigator after obtaining the permission by the administrators of all participating institutions described in the Implementation Plan and confirming that the patient registration system is open. "Study progress" in the trial registration will be registered as "being recruited."

(vii) JCOG Operations Office will inform Principal Investigator that jRCT registration has been completed after submission of the registration application. Principal Investigator will output the Implementation Plan from jRCT and submit the Implementation Plan, informed consent form, and Certified Review Board review result notification to the MHLW (Local Health and Welfare Bureau, which is responsible for the location of Certified Review Board; the same below). After submission, Principal Investigator will promptly inform Certified Review Board described in the Implementation Plan. In addition, Principal Investigator shall promptly report the submission of the Implementation Plan to the administrator of the institution to which he/she belongs, and provide this information to the site investigators of all participating institutions listed in the Implementation Plan and JCOG Operations Office.

(viii) After confirming that the notification of the Implementation Plan, etc. to the Local Ministry of Health and Welfare has been accepted and that jRCT's status has been updated from "under registration application" to "registration open," Principal Investigator will inform JCOG Operations Office that it has become "registration open." JCOG Operations Office opens JCOG Data Center's patient registration system and provides trial initiation announcements to study group. The date of jRCT publication is the start date of the study. "】

JCOG Operations Office should be contacted for procedures involving the addition of participating institutions after the initial submission.

"16.X. Medical institutions" changes (adding or replacing participating institutions) correspond to changes in the content of the protocol, then the change procedure shall be performed in accordance with the "13.6.2.Procedures for

when a change in the protocol occurs after the start of the study"

2) Procedures performed by the investigators at each participating institution

Following Certified Review Board approval, the investigator at each participating institution will obtain permission to conduct the research by the administrator of the participating institution using a set of documents received from Principal Investigator (Certified Review Board review results notification and documents submitted to Certified Review Board). The site investigator shall promptly send a copy of the letter of permission of the relevant institution to the Data Center after obtaining the permission by the administrator to conduct the research.

If the study is Specified Clinical Trial under Clinical Trials Act, the site investigator who has been informed that the Implementation Plan has been submitted to the MHLW by Principal Investigator should promptly report that the Implementation Plan has been submitted to the administrator of the affiliated institution.

3) Permission to conduct research at participating institutions

The procedures for obtaining permission for conducting research from the administrator of the affiliated institution shall be in accordance with the regulations of each institution.

When a copy of the institutional approval form is sent to JCOG Data Center, either the site investigator or the site coordinator should send the copy. Original copies of the institutional approval form will be stored by the site coordinator, and copies will be stored by JCOG Data Center.

When a affiliated institution has restrictions on providing personal information, such as medical record numbers, when sending copies of the institutional approval form to JCOG Data Center, the site coordinator shall communicate that personal information cannot be provided, and also send documents, such as the corresponding table of the ID number for registration and the medical record number, which is specified in JCOG privacy policy.

In addition, the informed consent form for patients approved by Certified Review Board may not be modified except for the contact information of the institution or prespecified selection items. A common protocol will be used for all institutions, since no changes in the protocols will be permitted for each institution. If it is necessary to change the content of the protocol and informed consent form, consult with Principal Investigator and Study Coordinator if the administrator of the institution asks for modification of the protocol and the text of the informed consent form in order to make the change as the protocol and informed consent form used by all institutions.

13.5.2. Procedures for changing the study plan after the start of the study

1) Procedures performed by Principal Investigator

If any of the changes of (1), (2), or (3) below occurs in the conduct of this study from the <Procedures from the initial application to the commencement of the study> in "13.6.1. Procedures at the time of new application", Principal Investigator shall hear Certified Review Board's opinions by applying for a change to Certified Review Board.

The procedures for applying for changes to the Certified Review Board are in accordance with "13.6.1. Procedures at the time of new submission" in the procedures from initial submission to the start of the study in ②, ③, and ④, respectively. In the absence of a change in the Implementation Plan, notification to the MHLW is not necessary. In the event that a change in the Implementation Plan occurs, notification to the MHLW is required. If Principal Investigator becomes aware of the planned change in the Implementation Plan, he or she should promptly contact JCOG Operations Office. JCOG Operations Office will assist in the application procedure for change to Certified Review Board and registration in change to jRCT.

（1） When the content of the protocol or informed consent form is changed (when it is amendment or revision in 13.7.1.).

（2） When the protocol or informed consent form is not changed, but the Implementation Plan (registered in jRCT) is changed.

（3） When changing Conflict of Interest Management Standards or Conflict of Interest Management Plans

Because notification of changes in the Implementation Plan (registered in jRCT) to the MHLW except for changes in research progress must be done in advance, Principal Investigator should inform site investigators and JCOG Operations Office of the change including the replacement of the investigator or the subinvestigator at each

participating institution. In addition, inform other investigators as soon as there are any changes that need to be made known.

Principal Investigator shall promptly report to the administrator of the institution to which he/she belongs and provide information to other research investigators when informed by Certified Review Board.

Procedures when it is necessary to notify changes to the Certified Review Board and the MHLW before changes are made.

1. Changes to the Implementation Plan

If changes to any of the above (1), (2), or (3) are made to the Implementation Plan (registered in jRCT) after hearing Certified Review Board's opinions, JCOG Operations Office should enter changes to jRCT under the supervision of Principal Investigator. In addition, Principal Investigator submits the following notification to the MHLW. After the date of coming into force of the predefined changes, the study shall be conducted in accordance with the changes.

- Notification of Change in Implementation Plan (Form No.2 of the Ministerial Ordinance)
- Implementation Plan after the change (output of the change in jRCT)
- Certified Review Board Review Results Notification

※ On institution transitioning to JCOG Collaborating Institution by replacement of participating institution: For institutions where patients were not enrolled prior to transfer to collaborating institution, Principal Investigator should remove it from the participating institutions by notifying changes in the Implementation Plan.

Procedures when it is necessary to notify a change to the Certified Review Board and the MHLW after the change***

1. Change in progress

(2) Among the changes in the Implementation Plan (registered in jRCT), the change in "3. Issues related to checking the implementation of Specified Clinical Trials (2) Specified Clinical Trials progress" shall be made without delay after the change. Under the supervision of Principal Investigator, JCOG Operations Office shall change "Research Progress" in jRCT. Subsequently, Principal Investigator will promptly apply for a change to Certified Review Board. After obtaining approval from Certified Review Board, Principal Investigator will submit the following notification to the MHLW.

- Notification of Change in Implementation Plan (Form No.2 of the Ministerial Ordinance)
- Implementation Plan after the change (the content of the change in jRCT was outputted)
- Certified Review Board Review Results Notification].

2. Changes in Implementation Plan after registration of the first patient

At the time of the initial submission, the "date of registration of the first patient" in the Implementation Plan is provided in a blank space. The Implementation Plan should be changed without delay after registration in the first patient. In this instance, JCOG Operations Office enters and registers the "First Patient Registration Date" in jRCT under the supervision of Principal Investigator. Subsequently, Principal Investigator will promptly apply for a change to Certified Review Board.

- Notification of Change in Implementation Plan (Form No.2 of the Ministerial Ordinance)
- Implementation Plan after the change (output of the change in jRCT)
- Certified Review Board Review Results Notification]

3. Minor changes specified by MHLW ordinance (application for change to Certified Review Board is not required)

If the following minor changes are made to Implementation Planning and the registration of jRCT, Principal Investigator will not need to hear the opinions of Certified Review Board and will notify Certified Review Board of the changes within 10 days of the date of the change. In addition, a notification (Form No.3) shall be submitted to the MHLW.

Scope of minor changes to the Implementation Plan of Article 42 of Clinical Trials Act Enforcement Regulations

- A change in the name of a person engaged in Specified Clinical Trials that does not involve a replacement of the person engaged in Specified Clinical Trials.
- Changes due to a change in the name or address number of the area

2) Procedures performed by the investigators at each participating institution

If any changes occur in the part of the protocol that corresponds to the institution to which he/she belongs, (4) Items related to investigators in multi-institutional studies, inform Principal Investigator and JCOG Operations Office of the changes to be made prior to the changes. Depending on the content of the change, the investigator shall confirm the confirmation of institutional requirements, prepare documents for conflicts of interest and submits to Certified Review Board such as lists of subinvestigators, and inform Principal Investigator and JCOG Operations Office.

The content of the most recent Implementation Plan for the institution to which they belong (the same as the content of jRCT registration) should be checked on jRCT website (https://jrct.niph.go.jp/).

13.5.3. Review and approval of study progress and study continuation (periodic reports)

Principal Investigator will report periodic reports on the progress of the study, the occurrence of adverse events, and conflict of interest management (see 13.X.1.⑱) to the administrators of their institutions and report them to Certified Review Board. Within two months after the expiration of each year from the date of submission of the Implementation Plan to the Minister of Health, Labour and Welfare.

When the report is made to Certified Review Board, Principal Investigator shall promptly inform the investigators of other participating institutions of the fact. The investigator who receives the information shall promptly report the details of the information to the administrator of the institution to which he/she belongs.

If the study falls under Specified Clinical Trials above Clinical Trials Act, Principal Investigator will report to the MHLW regarding the implementation status of Specified Clinical Trials. Periodic reports to Certified Review Board shall be made within 1 month of the date on which the results on the appropriateness of continuation of the relevant Specified Clinical Trials are obtained. The report shall be made by submitting the attached Form 3※ to the Minister of Health, Labour and Welfare.

※ Enforcement of Clinical Trials Act Enforcement Regulations, etc. (Notification No. 0228-1 of the Sector of Economics, Ministry of Health, Labour and Welfare, Notification No. 0228-1 of the Notification No. 1 of the Evaluation and Development Division, Health Policy Bureau, Ministry of Health, Labour and Welfare, dated February 28, 2018)

13.6. Protocol revision/amendment

13.6.1. Categorization of protocol changes and procedures for changes

For a change in the protocol, the Protocol Revision Application must be submitted to Data and Safety Monitoring Committee Office prior to submission to Certified Review Board [in the case of Specified Clinical Trials, add the following: "and report to the MHLW"] (see 13.6.2).

JCOG deals with the changes in the content of the protocol after approval by the Protocol Review Committee, divided into amendment and revision. Data and Safety Monitoring Committee Office will distinguish between amendment and revision, so all of applications are submitted as revision. If it is classified as an amendment by the Secretary-General of Data and Safety Monitoring Committee, it is reviewed by Data and Safety Monitoring Committee. If it is classified as a revision, Secretary-General of Data and Safety Monitoring Committee will issue a verification form and will not be reviewed by Data and Safety Monitoring Committee. We also distinguish the addition of supplementary explanations that do not fall into a change in protocol content as "Memorandum". Definitions and handling are as follows:

When the protocol or informed consent form is revised or amended, Principal Investigator will promptly distribute the most recent protocol or informed consent form to those involved in the study. Individuals involved in the study should always conduct the study in accordance with the most recent protocol since the dates of entry into force of the amendment/revision.

1) Amendment

Partial protocol change which meets one or more of the followings: i) Potential to increase the risk of patients enrolled in the study, ii) Having substantial effects on primary endpoint of the study, iii) having essential effects on the study's implementation structure.

The amended version of the protocol and informed consent form version numbers are shown as in 2.0.0, 3.0.0, and 4.0.0….

Approval by the Group Chair and the Data Center Director must be obtained prior to submission to Data and Safety Monitoring Committee.

When classified as "amendment" by the Secretary-General of Data and Safety Monitoring Committee, review of changes by Data and Safety Monitoring Committee is performed prior to Certified Review Board review. After the protocol amendment has been approved by Data and Safety Monitoring Committee, an application for a change in the protocol will be submitted to Certified Review Board through JCOG Operations Office (at this time the version number is ver. 2.0.0, 3.0.0, 4.0.0…). When the protocol was changed based on the review opinions of Certified Review Board, the version number is ver. 2.0.1, ver.2.0.2… If the protocol is changed by review of Certified Review Board, the change will be reported to Data and Safety Monitoring Committee, but in principle, the change will not be reexamined by Data and Safety Monitoring Committee. When a protocol change is approved by Certified Review Board, the protocol cover page should include the date of approval by Data and Safety Monitoring Committee and Certified Review Board [If Specified Clinical Trials, the following is added: "and a notification of the change in the Implementation Plan to the Regional Bureau of Health and Welfare should be made"].

Following Certified Review Board approval, permission for the contents of the amendment by the administrator of each institution shall be obtained. If permission is obtained, the site coordinator of each institution will send copies of the permission notice by the administrator of each institution to the Data Center. After the permission of the administrator of all institutions is obtained, the protocol changes will come into effect (during this time, the patient registration will not be suspended unless there is a special need). The actual date of entry into force will be announced by JCOG Operations Office. Researchers in all participating institutions conduct the study according to revisions approved by Certified Review Board since the date of entry into force.

Treatment and assessment of enrolled patients will be performed according to the pre-change version protocol until entry into force. Protocol deviations to enhance patient safety during treatment will be permitted if pre-change protocols, such as inadequate treatment modification criteria, threaten patient safety. If protocol deviations occur, they should be listed in the monitoring report.

2) Revision

Protocol change which meets all of the followings: i) does not have an increased risk for patients enrolled in the study; ii) does not have a substantial effect on primary endpoint of the study; iii) does not have an inherent effect on the system in which the study is conducted. Includes changes in protocols due to mistakes or changes in institution-specific information, changes in institution-specific information without changes in protocols (changes in Implementation Plans and jRCT registrations), and changes in conflicts of interest at individual institutions. In principle, suspension of patient registration is not performed in case of revision.

The revised version of the Protocol and Informed Consent Form version numbers are shown as in 1.1.0, 1.2.0, and 1.3.0….

Approval by the Group Chair and the Data Center Director must be obtained prior to submission to Data and Safety Monitoring Committee.

If classified as "Revision" by the Secretary-General of Data and Safety Monitoring Committee, the Secretary-General of Data and Safety Monitoring Committee issued a verification form, Data and Safety Monitoring Committee did not review the changes. The Principal Investigator submit an application for a change in the protocol to Certified Review Board through JCOG Operations Office (at this time, the version number is ver.1.1.0, 1.2.0, 1.3.0…). If the protocol was changed based on the indication by the Certified Review Board, version number shall be as ver.1.1.1, ver.1.1.2… When a protocol change is approved by Certified Review Board, the date of approval by the Certified Review Board should be entered on the protocol cover page. [If Specified Clinical Trials, the followings are added: "and a notification of the change in the Implementation Plan to the Regional Bureau of Health

and Welfare should be made"]

The date of entry into force of the protocol change will be 2 weeks after Certified Review Board approval date unless otherwise specified. [in the case of a Specified Clinical Trials, the followings are added: "The effective date shall be the date after the notification of change of the Implementation Plan to the Regional Bureau of Health and Welfare"]

The actual date of entry into force will be announced by JCOG Operations Office. Researchers in all participating institutions conduct the study according to revisions approved by Certified Review Board since the date of entry into force.

At that time, the site investigator should obtain permission by the administrator of each institution after the date of approval of Certified Review Board and before the date of entry into force. In this case, the protocol revision may be permitted with a report to the administrator of the institution. However, the procedures for obtaining permission from the administrator shall be in accordance with the regulations of each institution. For protocol revisions, confirm with the institution prior to initiation of the study to ensure that permission is obtained within the aforementioned time periods, and contact JCOG Operations Office if this is difficult. Reports to the administrator and approval form by the administrator at each institution need not be sent to the Data Center, but the original copy will be retained by the site coordinator as it will be checked during site visit audits.

Treatment and assessment of enrolled patients will be performed according to the pre-change version protocol until entry into force. Protocol deviations to enhance patient safety during treatment will be permitted if pre-change protocols, such as inadequate treatment modification criteria, threaten patient safety. If protocol deviations occur, they should be listed in the monitoring report.

<u>3) Memorandum</u>

Supplementary description of the protocol distributed from Principal Investigator/Study Coordinator to study personnel in objective, such as reduction of interpretive variation in sentences, and special precautions, rather than change of protocol content. Any form is used.

Approval by Group Chair and the Director of the Data Center is needed prior to distribution. Reporting to Data and Safety Monitoring Committee before distribution or immediately after distribution is required.

It is not necessary to include the protocol on the cover page.

13.6.2. Patient explanation and re-consent at the time of protocol amendment/revision

In the event of a change in the content of the study, the investigator or the subinvestigator shall provide appropriate explanation to the enrolled patients (regarding protocols based on revision, treatment, follow-up, etc.). In addition, if Certified Review Board comments that re-consent of enrolled patients in writing is required, informed consent should be obtained in writing.

13.7. Conflicts of Interest (COIs) involved in this study

13.7.1. COI management involved in this study

The COIs involved in this study will be managed according to the "Guidance for Conflict of Interest Management in Clinical Trials Act (Notification No.1130-17 of the PMSB dated November 30, 2018) ※ (Guidance) of the Division, Research and Development, Ministry of Health, Labour and Welfare, in accordance with the following:

※ http://www.mhlw.go.jp/stf/seisakunitsuite/bunya/0000163417.html

The format used for COI control should be the latest version of the guidance.

- Conflict of Interest Management Standards: Form A
- Reports of related companies, etc.: Form B
- Investigator Conflict of Interest Self-Report Form: Form C
- Conflict of Interest Confirmation Report: Form D
- Conflict of Interest Management Plan; Form E

<u>1) Procedures for New Application</u>

<u>Conflict of Interest Management Standards</u>

(i) All JCOG trials according to Clinical Trials Act will employ Conflict of Interest Management Standards (Form A) in accordance with guidance.

Request for Confirmation of Conflicts of Interest

(ii) At the time of initiation of the primary review of the protocol, JCOG Operations Office shall identify companies, etc. related to this study that require conflict of interest management based on information on drugs and medical devices specified in the Protocol Treatment (see 6.1.), enter the relationship with the company in Form B, and ask Principal Investigator to confirm the accuracy of the entry.

(iii) After checking the entries in Form B received from JCOG Operations Office, Principal Investigator will send a format to the site investigator and site coordinator at each participating institution and ask to confirm the entries in Form B, create Form C, and create Form E. In addition, a form shall be sent from Principal Investigator to those who correspond to the "person responsible for statistical analysis" and the "administrator other than Principal Investigator and Investigators" of the Implementation Plan, and they shall be asked to prepare Forms C and E.

Confirmation of Conflicts of Interest (participating institutions)

(iv) The site investigator or site coordinator at each participating institution will register the investigators, subinvestigators, and research associates involved in the study in JCOG Web Entry System. Investigators and subinvestigators registered in JCOG Web Entry System will be the reporters of conflicts of interest in the study.

(v) The site coordinator will request the investigator of the study to confirm the description of Form B received from Principal Investigator, create Form C, and create Form E. The investigator will then summarize conflict of interest management within the study site.

(vi) The investigator checks the forms received from Principal Investigator for any relevant items in Q2 through Q5 of Form B and notifies Principal Investigator through JCOG Operations Office within a week if any.

(vii) In the column of Form C [Persons requiring a Form C self-declaration of conflict of interest (Form C)] ※, the site investigator inputs the information of the investigator and subinvestigator registered in JCOG Web Entry System, and asks subinvestigator to prepare Form C.

※ The information entered in Form C is automatically entered in Form E. This entry should be consistent with the physician's information contained in the "List of Subinvestigators" (Clinical Trials Act Uniform Form No.1) submitted to Certified Review Board with Form E, and physicians who do not agree may not be involved in this study. Therefore, when entering the information in the ※ column for [those who require a Conflict of Interest Self-Reporting Form (Form C)], the investigator or subinvestigator list should be downloaded in JCOG Web Entry System and the physician listed in the Study Subinvestigator list should be entered without missing the person who will be the investigator and the subinvestigator in the study. If the information in the downloaded "Study Subinvestigator List" is not consistent with the physician involved in the study, the registered information should be updated in JCOG Web Entry System, and the "Study Subinvestigator List" with the most recent information reflected should be downloaded and used.

(viii) The investigator and the subinvestigator shall complete the necessary information regarding the relationship with the company, etc. described in advance in Form C, and submit Form C to the Conflict of Interest Confirmation Department of the institution to which he/she belongs. In doing so, the investigator also submits Form A.

(ix) The investigator receives the results of confirmation of conflicts of interest (Form D) of the investigator and the study subinvestigator from the institution to which he/she belongs.

(x) The investigator will confirm the content of Form A, Form B, and Form D of the investigator and all study subinvestigators to create Form E. The investigator will notify Principal Investigator through JCOG Operations Office of Form E and the Study Subinvestigator List downloaded in Form 7.

Confirmation of Conflicts of Interest (outside participating institutions)

(xi) The person responsible for statistical analysis and the person overseeing a study other than Principal Investigator or investigator shall complete, in the form received from Principal Investigator, the necessary information regarding the relationship with the company, etc. described in advance in Form C, and submit Form A and Form C to the Conflict of Interest Confirmation Department of the Affiliated Institution.

(xii) The person responsible for statistical analysis and the person overseeing studies other than Principal Investigator or investigator shall receive confirmation results (Form D) from the institution.

(xiii) The person responsible for statistical analysis and the person overseeing studies other than Principal Investigator and investigator shall confirm the content of Form A and Form D, prepare Form E, and notify Form E to Principal Investigator through JCOG Operations Office.

Description of Conflicts of Interest in the protocol and informed consent form

(xiv) Principal Investigator and JCOG Operations Office will review Form A and Form E received from participating institutions and, as appropriate, will accurately describe the conflicts of interest (study COIs) between the study and the drug marketing authorisation holder, etc. in the protocol and informed consent form. Conflicts of interest (personal COI) between the reporters of conflicts of interest in this study and the marketing authorisation of drugs, etc. (personal COI) can change over time, so the personal COI is not described in the protocol and informed consent form, and the information is updated on JCOG website as needed.

Certified Review Board review

(xv) Principal Investigator compiles and submits the Form E and Study Subinvestigator List of all institutions to Certified Review Board for review.

2) Procedures for new involvement with companies after the start of the study

(xvi) When a new involvement with companies (research COI) occurs in the study after the start of the study

a. In the event of any change in the enterprises involved in this study, etc. to be described in Q1 of Form B, repeat the procedures ② to ⑭. However, the site investigator and site coordinator roles in ④, ⑤ shall be assumed by the investigator at each institution. If a new study COI needs to be added to the protocol and informed consent form, a protocol revision (see 13.X.X.) should be submitted to Certified Review Board for review.

b. If a change occurs from Q2 to Q5 in Form B, the investigator at the participating institution affected by the change will change the appropriate description in Form B and update Form E and send it to Principal Investigator and JCOG Operations Office. A Principal Investigator who has received a Form E will revise the protocol as needed (see 13.X.X.) and submitted to Certified Review Board for review.

(xvii) In the event that a conflict of interest reporter is newly involved with a company (personal COI) after the start of the study

Conflict of interest reporters repeat procedures from ⑧ to ⑩ or from ⑪ to ⑬. However, if there is no change in Form E, these procedures will be performed at each institution but will not be sent to Principal Investigator and JCOG Operations Office. Principal Investigator will submit Post-change Form E sent from the site investigator to Certified Review Board for review. Personal COIs are also disclosed on JCOG website.

3) Procedures for periodic reporting

(xviii) Principal Investigator checks for changes in study COI and personal COI annually at the timing of periodic reporting and reports to Certified Review Board.

13.7.2. COI with companies involved in this study(study COI)

There are no conflicts of interest to disclose about relationship between the company manufacturing and marketing drugs specified as a part of protocol treatments in this study (See 6.1).

13.7.3. COIs of JCOG Committee members and JCOG Data Center/Operations Office staff

COIs of the committee members of JCOG committees, the staffs of the office of committees, and JCOG Data Center/Operations Office staff involved in the study will be managed by JCOG Conflict of Interest Committee in accordance with Clinical Trials Act control standards.

13.8. Compensation

In order to comply with Clinical Trials Act, this study must take necessary measures such as sing up for insurance and ensuring a system to provide medical care in order to compensate for the health damage caused by the study

and provide medical care.

Therefore, regarding the health damage caused by participating in this study, appropriate treatment according to the condition is provided similarly to the usual insurance medical care. In addition, this study will be covered by clinical study insurance, and the following will be compensated based on insurance conditions, and this will be explained to patients and understood.

13.9. Intellectual property

The results, data, and intellectual property rights obtained from this study are attributed to the followings: National Cancer Center, Principal Investigator, Study Coordinator, and Group Chair. Specific procedures and allocation must be determined through consultation among four parties. Whether the intellectual property related to Principal Investigator, Study Coordinator, Group Chair will belong to the individuals or the affiliated institution will be determined according to the agreements of the affiliated institution.

13.10. Disclosure of information on this study

Summary, progress, and main results of this study will be published on JCOG website (www.jcog.jp) and on jRCT (https://jrct.niph.go.jp/).

14. Monitoring and audit

14.1. Periodic monitoring

In this study, monitoring is performed in order to ensure that the study is conducted safely and in accordance with this protocol and that data are collected accurately. Periodic monitoring should be performed twice a year in principle, using the entered data on the CRFs collected in the Data Center. Specific procedures for periodic monitoring are provided separately in the Monitoring Plan.

The Data Center submits a "Monitoring Report" summarizing the results of central monitoring to Principal Investigator, Study Coordinator and investigators. Together, they are submitted to Group Chair, Data and Safety Monitoring Committee, and JCOG Chair.

The Monitoring Report is a material for periodic reports to be made every year from the date of submission of the Implementation Plan.

14.1.1. Monitoring items

① Registration status: number of registration - cumulative/by month, by arm/site
② Eligibility: ineligibles/potentially ineligible cases: by arm/site
③ Pre-treatment baseline factors: by arm
④ On/off-treatment, reason for treatment termination: arm/site
⑤ Protocol deviation: arm/site
⑥ Serious Adverse Events: arm/site
⑦ Adverse reaction/event: arm
⑧ Overall survival, progression-free survival (or relapse-free survival, etc.): all registered patients
⑨ Other issues related to study progress and safety (studies in accordance with Clinical Trials Act: status of occurrence of non-compliance and subsequent response, number of subjects for compensation, number of disease or the like reports in accordance with Article 13 of Clinical Trials Act)

14.1.2. Eigibility (Eligible/Ineligible)

For all registered patients, eligibility will be classified according to the following definitions as either: In monitoring, Data Center lists potentially ineligible cases in the "Evaluation of Eligibility" section of the monitoring report, and after review by Study Coordinator, confirms them to be either 1), 2), 9), or 99) with Group Chair approval prior to performing primary analysis.

Only 1) eligible shall be "eligible case", 2) post hoc ineligible,9) de facto ineligible and 99) violation of registration shall be "ineligible case". This is a category established from the perspective of analysis set setting.

In the study in accordance with Clinical Trials Act, "99) violation of registration" will be regarded as "major non-compliance" in Clinical Trials Act, and Principal Investigator will promptly report the situation to Certified Review Board as soon as possible. See 14.3. for management of non-compliance.

9) de facto ineligibles corresponds to "non-compliance" on Clinical Trials Act and is reported to the administrator of participating medical organizations with the submission of the monitoring report on which they were described (twice a year).

2) Post hoc ineligibles is not treated as "non-compliance" on Clinical Trials Act because it does not correspond to non-compliance with the study protocol, as discussed below.

1) Eligible

All information generated prior to registration meets all o the Patients Selection Criteria according to the methods and criteria specified in the study protocol.

2) Post hoc ineligible

The information generated after registration does not meet either Patients Selection Criteria, or the information generated prior to registration does not meet either Patients Selection Criteria by methods or criteria other than those specified in the protocol.

Examples)

(i) In the study for Stage II-III, bone scintigraphy performed immediately after registration revealed bone

metastases, and the patient was diagnosed as Stage IV. The protocol treatment was terminated.

(ii) In the study for early gastric cancer, bloody stools is seen after registration, and colonoscopy revealed advanced colorectal cancer (synchronous double cancer). Colectomy was performed after termination of the protocol treatment.

(iii) In the study for gastric cancer (adenocarcinoma), the institution's pathological diagnosis was changed to malignant lymphoma after registration.

9) De facto ineligible

Information generated prior to registration according to protocol-specified methods (performed in all cases) and criteria does not meet either Patients Selection Criteria. This includes cases where it is determined after registration that the information that occurred before registration had been incorrect.

Example: When the supervising physician reviews the CT images performed before registration as specified, there is obvious liver metastasis (if it is a mistake by the attending physician and it is considered that there is no future).

99) Violation of registration

Deliberately (falsely) enroll while knowing that Patients Selection Criteria is not met. Corresponds to a misconduct and treats it as a serious problem.

14.1.3. Protocol Deviations/Violations

Protocol deviations are defined as those in which treatment, such as drug administration, radiotherapy, or surgical resection, as well as laboratory tests and evaluation of toxicity and efficacy, etc. were not performed according to the protocols.

In monitoring, deviations that exceed a certain acceptable range limit for each study decided by the Data Center and Principal Investigator/Study Coordinator prior to or after the initiation of the study are listed in the monitoring report as "possible deviations" and are classified into one of the following categories after consideration by Study Coordinator and study groups: Except for those described in the protocol and monitoring report, the acceptable deviations agreed between the Data Center and Principal Investigator/Study Coordinator may be changed through periodic monitoring during the study, so they should be described in the supplemental material rather than in the text of the Monitoring Plan, and the Monitoring Plan should be described as "defining the acceptable ranges separately".

1) Violation

Any deviation from the protocol that is clinically inappropriate and caused by the treating physician/institution and that meets two or more following criteria shall be classified as a violation. [When conducted in accordance with Clinical Trials Act, the following shall be added. [In the study according to Clinical Trials Act, the violation shall be treated as "major non-compliance" and Principal Investigator should report the situation to Certified Review Board as soon as possible].

① Have a substantial impact on the assessment of study endpoints

② Intentional or systematic

③ Dangerous or remarkable deviation

For "violations", in principle, the content of each violation should be described in a paper when publishing.

2) Deviation

Deviations that do not fall into 1) violation or 3) acceptable deviation. If same kind of deviations are frequent, they should preferably be included in the publication of the article. They are classified as either of the following at the time of monitoring report review:

Because deviations correspond to "non-compliance" in Clinical Trials Act, they are reported (biannually) to the administrators of the institution with the submission of the monitoring report. Not to be "major non-compliance".

(i) Deviations - Undesirable and to be reduced

(ii) Deviations (unavoidable) - things that are not proactively reducing (e.g. delay by the New Year period, equipment breakdown, etc.)

(iii) Deviations (clinically relevant) - Those in which the decision of the treating physician/institution are positively affirmed (if a similar situation again arises similar deviations are considered desirable).

※ Deviations do not always mean that the treating physician at the institution is problematic. Since patient safety is a primary priority in clinical trials, deviations should rather be made by the medical judgment of the treating physician if the condition of the individual patient are considered to be dangerous when following the protocols. If the deviation is judged to be clinically relevant for the safety of the patient, it is recorded as ③ Deviation (clinically relevant). Clinically relevant deviations in a small number of patients are not required to be particularly problematic; however, protocol revision should be considered when multiple deviations occur because protocol specification is likely to be inadequate. However, deviations that are not intended to be safety (e.g., increased doses of anticancer drugs in the hope of increasing efficacy, shortened treatment periods not specified in the protocol) are not considered clinically relevant deviations.

3) Acceptable deviation

Deviations from protocols within acceptable range agreed by the entire JCOG, study groups, or Study Chair/Study Coordinator and Data Center, pre- or post-study initiation, on a trial-by-trial basis. When conducted according to Clinical Trials Act, the following are added. Not considered to be "non-compliance" in Clinical Trials Act. Deviations within the pre-specified acceptable ranges are not included in the monitoring report.

14.2. Site visit audits

In this study, site visit audits will be conducted to ensure the reliability of clinical research and the reliability of data and information collected by clinical research from the perspective of protecting human subjects in clinical research.

Site visit audits are conducted by auditors appointed by Principal Investigator by visiting a medical institution to confirm the approval documents of the medical institution, check the list of subinvestigators in the research institution, confirm the informed consent documents, and verify CRF entry data with medical records (direct access to source documents). Specific procedures for site visit audits are provided separately in the operating procedures.

The auditor shall report the audit report summarizing the audit results to Principal Investigator/Study Coordinator, site investigators. Together, the report will be submitted to Group Chair, Director of JCOG Data Center, Director of JCOG Operations Office, and JCOG Chair. Reports should also be submitted to the site investigators of the relevant groups and JCOG Executive Committee as appropriate.

14.2.1. Items to be audited

In the site visit audits, the following items are checked by direct access to source documents:

<Confirmation Items by Study>

- Approval documents (including initial approval forms, revision approval forms, and annual report approvals/reports) from administrators of medical institutions
- Management status of the protocol
- Contents of explanatory documents and informed consent forms

<Confirmation Items by Patient>

- Patient consent (presence or absence of consent form, signature, and date of consent)
- Implementation of pre-registration mandatory tests, eligibility for registration (inclusion criteria and exclusion criteria)
- Accuracy of reported data
    - Pre-treatment evaluation, course of treatment (protocol treatment)
    - Various test results (including diagnostic imaging reports and pathology reports)
    - Accuracy of test date, response evaluation, adverse event, survival or death information, and other reported data

<Other items>

- Presence or absence of study misconduct (possibility of false reporting, fabrication, or falsification)
- Improvement status of the items pointed out in the previous audit

14.2.2. Reporting of major non-compliance found in audits to Certified Review Board

Principal Investigator/Study Coordinator should report to Certified Review Board immediately when finding possible "major non-compliance (See 14.3.2.)" as a result of site visit audits.

14.3. Management of non-compliance

14.3.1. Non-compliance.

Non-compliance in Clinical Trials Act refers to the condition in which clinical research is not compliant with Clinical Trials Act Enforcement Regulations or study protocols. In the Clinical Trials Act Enforcement Regulations, etc. (February 28, 2018), non-compliance with regulations, study protocols, operation procedures, etc., and fabrication of falsification of research data, etc. are listed as examples.

If the site investigator knows that there is non-compliance, the site investigator should report to the administrator of the medical institution and inform Principal Investigator/Study Coordinator.

If site investigator find non-compliance (regardless of major non-compliance or not) prior to the implementation of central monitoring or site visit audits, the site investigator will promptly report to Principal Investigator/Study Coordinator and JCOG Data Center.

"14.1.3.2) Deviations" correspond to "non-compliance" in Clinical Trials Act. As described in 14.1.3.2), these deviations are reported by submitting monitoring reports or their excerpts or summaries issued twice a year to the administrators of the medical institution.

Changes in protocols and implementation plans associated with investigator transfer require Certified Review Board review and notification of implementation plans to the MHLW, therefore, require a certain time to complete the sequence of procedures. In addition, it is often difficult to complete the change procedure before the transfer because the transfer may not be open until just before the transfer. Therefore, even if the site investigator is absent for a certain period of time due to the transfer, the study does not fall into "non-compliance" in this study if the research management system is maintained by the subinvestigator and the medical care system of the enrolled patients who are surviving is ensured.

14.3.2. Major non-compliance

"Major non-compliance" is that affect the human rights and safety of subjects of clinical research, the study progress and the reliability of study results. Examples of "major non-compliance" in JCOG study are provided in. If major non-compliance is likely, Principal Investigator/Study Coordinator will report the situation to Certified Review Board immediately.

1) Major non-compliance with respect to eligibility

Violation of registration

- Enrolled intentionally (falsely) while knowing that eligibility criteria was not met
- Patient enrollment was performed without necessary informed consent, and protocol treatment was performed
- The source documents for the determination of eligibility cannot be identified (including the loss of the consent form).

2) Protocol violation

Violations that affect the increased risk of enrolled patients or that affect the reliability of the study results

- Significant violation in inclusion criteria or exclusion criteria
- Violation threatening patient safety in off-treatment criteria
- Serious violation of prohibited concomitant drug, prohibited concomitant treatment, etc.
- e.g. intentional or systematic non-compliance with protocol regulations

3) Other major non-compliance

- Study was conducted prior to Certified Review Board approval or prior to approval of site administrator
- The study was continued without providing information to the enrolled patient that could affect the willingness to continue to participate the study.
- Those judged to be research misconduct (fabrication or falsification of data, etc.)

- Any leakage of personal information or violation of human rights that may have a significant impact on the enrolled patient.

15. Special Instructions

15.1 Central pathology diagnosis and related matters

15.1.1 Central pathological diagnosis

In this study, the pathological tissues of enrolled patients will be collected and the central pathological diagnosis will be determined after the fact. Since the central pathological diagnosis is not performed in real time for each individual patient enrollment, the main analysis target and the decision on treatment strategy will be based solely on the pathological diagnosis at the institution. The details of the central pathological diagnosis procedure will be specified separately in the Central Pathological Diagnosis Procedure Manual.

Timing: Once a year throughout the study period.

Subjects: All registered patients

Methods: Pathological specimens (or duplicates made from the same paraffin block) used for eligibility criteria determination at the registered facilities will be collected, and after necessary staining (chromogranin A, synaptophysin) is added, pathological eligibility will be re-determined by two or more pathology judges (16.8 Pathology judges) appointed by the group representative. (16.8 Central Pathological Review Comittie).

Management of the collected specimens: The Hepatobiliary and Pancreatic Group Study Office will be responsible for the management of the collected specimens.

Staining: If additional staining is required, it will be performed by the Central Pathology Coordinator (16.7).

Notification of the central judgment to each facility:

After the results of the central pathological diagnosis are fixed, the research secretariat will notify the results of the judgment to the registered facility (facility coordinator) of each patient. At that time, the rationale for the decision should be attached in writing. If there is a difference between the institutional diagnosis and the central diagnosis, the facility principal investigator/facility coordinator reports the decision results to the facility pathologist and discusses it with the facility pathologist, and carefully decides on the final pathological diagnosis at the facility (whether to change the diagnosis or not) and what to do if the patient is under treatment (whether to change the treatment or not).

15.1.2 Providing information to the institutional pathologist

In view of the high level of difficulty in NEC pathological diagnosis and the unique nature of this study, which covers multiple target organs, the following information will be provided to institutional pathologists.

(1) Pre-registration pathological diagnosis consultation

Since it is anticipated that there will be cases of confusion in diagnosis at facilities, pre-enrollment pathology consultation will be available as needed, with the pathology judgment committee members of this study serving as consultants. Details of the consultation operation are described in the Central Pathology Procedures.

(2) Holding of pathology-related meetings

In this study, the research secretariat and the pathology secretariat first held a pathology-related meeting (attended by pathologists from participating institutions) on February 11, 2014, during which a lecture was given to form a consensus on diagnostic criteria according to the WHO 2010 classification. The slides used at the meeting were revised as necessary based on the discussion at the meeting and posted on the JCOG website for reference by pathologists at each facility.

15.1.3 Response to cases in which pathological diagnosis was performed at a facility other than the home facility

In any of the following cases, be sure to ask a pathologist at your own institution to make the diagnosis, and confirm that the diagnosis at your institution is also NEC before enrolling in this study.

(1) When borrowing only stained tissue specimens from a previous physician for registration

Register after obtaining permission from the attending physician to the previous physician to submit the borrowed tissue specimen to JCOG 1213 for central diagnosis (loan again) and to borrow the specimen for a long period of time (up to about 1 year).

(2) If you have received virtual slides of "all tissue specimens for which a pathological diagnosis has been made" from

your previous doctor

The patient will be registered after obtaining permission from the attending physician of the previous physician to submit the virtual slides provided by the previous physician for the central diagnosis of this study.

(3) If you have received a "borrowed" pre-stained tissue specimen and an "offered" unstained preparative from your previous physician

The borrowed stained tissue specimens from the previous physician can be promptly reviewed at the patient's own institution, but the provided unstained preparations require time for pathological diagnosis after immunostaining at the patient's own institution, which may cause a time lag. There have been cases in which patients were enrolled in the study based only on the results of pathological review of borrowed tissue specimens at their own institutions, and later the results of staining and pathological diagnosis of undyed preparations at their own institutions overturned the pathological diagnosis (diagnosis of a different disease was made), resulting in post-hoc ineligible cases. Therefore, we will consider the timing of registration after sufficient consultation with pathologists at our own institution to avoid the occurrence of posterior ineligible cases as much as possible. However, if there is enough time, it is preferable to enroll unstained preparations into the study after staining and pathological diagnosis is obtained at your own institution.

15.2. JCOG BioBank Japan (BBJ) Biorepository

This study will participate in the banking of blood samples (DNA/plasma) in JCOG BBJ Biorepository based on a common protocol for all JCOG studies (hereafter referred to as common banking).

Subjects:

Among patients who agreed to participate in this study, patients whose consent to shared banking was obtained.

Sample:

1) Whole blood

Blood sampling is performed before the start of the protocol treatment in this study in principle. However, blood sampling after initiation of protocol treatment is allowed. Blood samples of 7 mL×2 (total 14 mL) of venous blood are collected using a blood collection tube (for blood counting) with a EDTA Na dedicated to the shared banking of JCOG-BBJ Biorepository, and stored at 4°C at the respective institutions until they are handed over to the sample transport/processing company (See "JCOG-Biobank Japan Biorepository protocol" for details).

2) Pathological specimens

Archived pathological tissues in daily clinical practice such as surgery, biopsy and laboratory tests can also be used in future translational researches, but the type of pathology specimen, preparation method and tissue quantity required by the studies are varied and there is no consensus that it is efficient to bank pathological tissues in a certain way prospectively. In addition, there is the opinion that the sample deteriorates (DNA fragmentation) when the thin-section sample from pathological tissue is stored for a long time.

Consent on the use of archival pathological tissue after medical care should therefore be obtained at the time of consent to banking, but actual collection should be initiated on an individual basis by creating a protocol and defining the most appropriate procedure for the study content in the protocol.

16. Organization

Changes to this chapter are considered to be revision rather than amendment.

16.1. Main study fund (funding source) of this study.

Practical Research for Innovative Cancer Control from Japan Agency for Medical Research and Development
“Establishment of standard treatments for neuroendocrine carcinoma of the digestive system” JP15ck0106138, JP16ck0106138, JP17ck0106355, JP18ck0106355, JP19ck0106355, JP20ck0106618
National Cancer Center Research and Development Fund (23-A-22, 26-A-4, 29-A-3, 2020-J-3)
“Scientific research on multi-institutional trials to establish new standard treatment of solid tumors in adults”

16.2. Japan Clinical Oncology Group (JCOG)

JCOG is a multi-institutional clinical research group consisting of research teams funded by public research grants

mainly on National Cancer Center Research and Development Fund and Japan Agency for Medical Research and Development research costs that receive direct support for research by the Clinical Research Support Office of the National Cancer Center Hospital in accordance with JCOG Policy (http://www.jcog.jp/).

This study is conducted using JCOG research organisations and in accordance with the regulations set out by JCOG Executive Committee.

16.3. JCOG Chair

Yuichiro Ohe National Cancer Center Hospital

16.4. Study group and Group Chair

JCOG Hepatobiliary and Pancreatic Oncology Group

Group Chair: Junji FURUSE

Department of Medical Oncology, Kyorin University Faculty of Medicine

〒181-8611 6-20-2, Shinkawa Mitaka-shi, Tokyo

TEL:+81-422-47-5511

FAX:+81-422-44-1858

E-mail: jfuruse@ks.kyorin-u.ac.jp

Group Secretary: Hirohi Ishii

Clinical Research Center, Chiba Cancer Center

〒260-8717, 666-2 Nitona, 260-8717, Chuo-Ku, Chiba

TEL:+81-43-264-5431

FAX:+81-43-264-8680

E-mail: hirishii@chiba-cc.jp

JCOG Stomach Cancer Study Group:

Group Chair: Masanori Terashima

Shizuoka Cancer Center

〒411-8777 1007 Shimonagakubo, Nagaizumi-cho, Sunto-gun, Shizuoka Prefecture

TEL:+81-55-989-5222

FAX:+81-55-989-5783

E-mail: m.terashima@scchr.jp

Group Secretary: Takaki Yoshikawa

National Cancer Center Hospital

〒104-0045 5-1-1 Tsukiji, Chuo-ku, Tokyo

TEL:+81-3-3542-2511

FAX:+81-3-3542-3815

E-mail: tayoshik@ncc.go.jp

Group Secretary: Narikazu Boku

National Cancer Center Hospital

〒104-0045 5-1-1 Tsukiji, Chuo-ku, Tokyo

TEL:+81-3-3542-2511

FAX:+81-3-3542-3815

E-mail: nboku@ncc.go.jp

JCOG Japan Esophageal Oncology Group

Group Chair: Yuko Kitagawa

Keio University Hospital

〒160-8582 35 Shinanomachi, Shinjuku-ku, Tokyo

TEL:+81-3-3353-1211

FAX:+81-3-3355-4707

E-mail: kitagawa@a3.keio.jp

Group Secretary: Ken Kato
National Cancer Center Hospital
〒104-0045 5-1-1 Tsukiji, Chuo-ku, Tokyo
TEL:+81-3-3542-2511
FAX:+81-3-3542-3815
E-mail: kenkato@ncc.go.jp

16.5. Study Chair (Principal Investigator)

JCOG Hepatobiliary and Pancreatic Oncology Group
Takuji Okusaka
National Cancer Center Hospital
〒104-0045 5-1-1 Tsukiji, Chuo-ku, Tokyo
TEL: +81-3-3542-2511
FAX: +81-3-3542-3815
E-mail: tokusaka@ncc.go.jp

JCOG Stomach Cancer Study Group:
Narikazu Boku
National Cancer Center Hospital
〒104-0045 5-1-1 Tsukiji, Chuo-ku, Tokyo
TEL: +81-3-3542-2511
FAX: +81-3-3542-3815
E-mail:nboku@ncc.go.jp

JCOG Japan Esophageal Oncology Group
Ken Kato
National Cancer Center Hospital
〒104-0045 5-1-1 Tsukiji, Chuo-ku, Tokyo
TEL: +81-3-3542-2511
FAX: +81-3-3542-3815
E-mail: kenkato@ncc.go.jp

16.6. Study Coordinator

JCOG Hepatobiliary and Pancreatic Oncology Group
Chigusa Morizane
National Cancer Center Hospital
〒104-0045 5-1-1 Tsukiji, Chuo-ku, Tokyo
TEL: +81-3-3542-2511
FAX: +81-3-3542-3815
E-mail: cmorizan@ncc.go.jp

JCOG Stomach Cancer Study Group:
Nozomu Machida
Shizuoka Cancer Center
〒411-8777 1007 Shimonagakubo, Nagaizumi-cho, Sunto-gun, Shizuoka Prefecture
TEL:+81-55-989-5222
FAX:+81-55-989-5631
E-mail：no.machida@scchr.jp

JCOG Japan Esophageal Oncology Group
Ken Kato

National Cancer Center Hospital
〒104-0045 5-1-1 Tsukiji, Chuo-ku, Tokyo
TEL: +81-3-3542-2511
FAX: +81-3-3542-3815
E-mail: kenkato@ncc.go.jp

Yoshitaka Honma
National Cancer Center Hospital
〒104-0045 5-1-1 Tsukiji, Chuo-ku, Tokyo
TEL: +81-3-3542-2511
FAX: +81-3-3542-3815
E-mail:yohonma@ncc.go.jp

16.7. Central Pathological Review Coordinator

Nobuyoshi Hiraoka
National Cancer Center Hospital
〒104-0045 5-1-1 Tsukiji, Chuo-ku, Tokyo
TEL: +81-3-3542-2511
FAX: +81-3-3542-3815
E-mail: nhiraoka@ncc.go.jp

Hirokazu Taniguchi
National Cancer Center
〒104-0045 5-1-1 Tsukiji, Chuo-ku, Tokyo
TEL: +81-3-3542-2511
FAX: +81-3-3542-3815
E-mail: hitanigu@ncc.go.jp

16.8. Central Pathological Review Coordinator

Nobuyoshi Hiraoka (National Cancer Center Hospital)
Noriyoshi Fukushima (Jichi Medical University)
Nobuyuki Ohike (Showa University Fujigaoka Hospital)
Ryoji Kushima (Shiga University of Medical Science)
Mitsuya Iwafuchi (Scool of Health Sciences Faculty of Medicine, Niigata University)
Tetsuo Ushiku (The University of Tokyo)

16.9. Participating sites (participating institutions)

HBP	SCSG	JEOG	Participating institutions
○			Sapporo-Kosei General Hospital
○		○	Hokkaido University Hospital
	○		Keiyukai Sapporo Hospital
	○	○	Iwate Medical University
		○	Tohoku University Hospital
	○		Miyagi Cancer Center
○	○	○	Tochigi Cancer Center
○			Jichi Medical University
○	○	○	Saitama Cancer Center
○	○	○	National Cancer Center Hospital East
○		○	Chiba Cancer Center
○		○	Chiba University, Graduate School of Medicine
○	○	○	National Cancer Center Hospital
○			Kyorin University Faculty of Medicine
○			National Center for Global Health and Medicine (NCGM)
○	○	○	Cancer Institute Hospital of Japanese Foundation for Cancer Research
	○	○	Toranomon Hospital
○			Teikyo University School of Medicine
○			St.Marianna University School of Medicine
○	○	○	Kanagawa Cancer Center
○	○		Yokohama City University Medical Center
○	○		Niigata Cancer Center Hospital
○			Toyama University Hospital
○			Kanazawa University School of Medicine
	○		Ishikawa Prefectural Central Hospital
	○		Ogaki Municipal Hospital
○	○	○	Shizuoka Cancer Center
○	○	○	Aichi Cancer Center Hospital
		○	Kyoto University Hospital
	○	○	Osaka University Graduate School of Medicine
○	○		Kindai University Hospital
○	○	○	Osaka International Cancer Institute
○	○	○	National Hospital Organization Osaka National Hospital
	○	○	Osaka General Medical Center
	○	○	Osaka Medical and Pharmaceutical University
	○		Osaka Rosai Hospital
○	○	○	Kobe University Graduate School of Medicine
	○	○	Kansai Rosai Hospital
○	○		Hyogo College of Medicine
○	○	○	Hyogo Cancer Center
	○		Itami City Hospital
	○		Shimane University Faculty of Medicine
	○	○	Hiroshima University Hospital
	○		Fukuyama City Hospital
	○		Tokushima Red Cross Hospital
○	○	○	National Hospital Organization Shikoku Cancer Center
○		○	Kochi Health Sciences Center
○		○	National Kyushu Cancer Center
○		○	Kyushu University Hospital

	◯	◯	Oita University Faculty of Medicine

HBP: Hepatobiliary and Pancreatic Oncology Group
SCSG: Stomach Cancer Study Group:
JEOG: Japan Esophageal Oncology Group

16.10. JCOG Protocol Review Committee

This protocol was approved by JCOG Protocol Review Committee prior to submit to Certified Review Board.
(For membership, see website http://www.jcog.jp/basic/org/committee/protocol.html)
Contact: Protocol Review Committee Office

JCOG Operations Office/Clinical Research Support Office, National Cancer Center Hospital
〒104-0045 5-1-1, Tsukiji, Chuo-ku, Tokyo
TEL: 03-3542-2511 (ext. 2302)
FAX:03-3542-7006
E-mail: jcogoffice@ml.jcog.jp

16.11. JCOG Data and Safety Monitoring Committee

During study period, the study will be monitored by Data and Safety Monitoring Committee (e.g., adverse event reports, interim analysis reviews, monitoring report reviews, protocol revision reviews). However, the committee members from the study group conducting this study do not participate directly in the review of this study.
(For membership, see website http://www.jcog.jp/basic/org/committee/jury.html)
Contact: JCOG Data and Safety Monitoring Committee Office

JCOG Operations Office/Clinical Research Support Office, National Cancer Center Hospital
〒104-0045 5-1-1, Tsukiji, Chuo-ku, Tokyo
TEL:03-3542-2511 (ext. 2403)
FAX:03-3542-7006
E-mail: jcogoffice@ml.jcog.jp

16.12. JCOG Audit Committee

Site-visit audits by Audit Committee will be conducted during study period.
(For membership, see website http://www.jcog.jp/basic/org/committee/audit.html)
Contact: JCOG Auditing Committee Office

JCOG Operations Office/Clinical Research Support Office, National Cancer Center Hospital
〒104-0045 5-1-1, Tsukiji, Chuo-ku, Tokyo
TEL:03-3542-2511 (ext. 2403)
FAX:03-3542-7006
E-mail: jcogoffice@ml.jcog.jp

16.13. JCOG Conflict of Interest Committee

During study period, JCOG investigators involved in this study will be managed by the Conflict of Interest Committee.
(For membership, see website http://www.jcog.jp/basic/org/committee/coi.html)
Contact: JCOG Conflict of Interest Committee Office

JCOG Operations Office/Clinical Research Support Office, National Cancer Center Hospital
〒104-0045 5-1-1, Tsukiji, Chuo-ku, Tokyo
TEL:03-3542-2511 (ext. 2404)
FAX:03-3547-1002
E-mail: jcogoffice@ml.jcog.jp

16.14. Data Center/Operations Office

JCOG Data Center

Director of Data Center: Haruhiko Fukuda
Clinical Research Support Office, National Cancer Center Hospital

〒104-0045 5-1-1, Tsukiji, Chuo-ku, Tokyo
TEL:03-3542-3373
FAX:03-3542-3374
E-mail: jcogdata@ml.jcog.jp

JCOG Operations Office
Director of Operations Office: Kenichi Nakamura
Clinical Research Support Office, National Cancer Center Hospital
〒104-0045 5-1-1, Tsukiji, Chuo-ku, Tokyo
TEL:03-3547-1002
FAX:03-3547-1002
E-mail: jcogoffice@ml.jcog.jp

Official website: http://www.jcog.jp/

16.14.1. Data management administrator

Data management organization		JCOG Data Center
Data management administrator	Name	Harumi Kaba
	e-Rad number	40543442
	Affiliation	Clinical Research Support Office, National Cancer Center Hospital
	Title	Head of Multi-institutional Data Management Section

16.14.2. Monitoring administrator

Monitoring organization		JCOG Data Center
Monitoring administrator	Name	Haruhiko Fukuda
	e-Rad number	70263390
	Affiliation	Clinical Research Support Office, National Cancer Center Hospital
	Title	Chief of Data Management Division

16.14.3. Site-visit audit administrator

Auditing organization		JCOG Operations Office
Site-visit audit administrator	Name	Kenichi Nakamura
	e-Rad number	40543533
	Affiliation	Clinical Research Support Office, National Cancer Center Hospital
	Title	Director of Research Management Division

16.14.4. Statistical analysis administrator

Statistical analysis organization		JCOG Data Center
Statistical analysis Administrator	Name	Junki Mizusawa
	e-Rad number	60706646
	Affiliation	Biostatistics Division, Center for Research Administration and Support, National Cancer Center/ Biostatistics Section, Research Management Division, Clinical Research Support Office, National Cancer Center Hospital
	Title	Biostatistics Section Head

16.14.5. Research and development plan support personnel

Organizations in charge of supporting research and development plans		JCOG Operations Office
Research and development plan support personnel	Name	Tomoko Kataoka
	e-Rad number	70569863
	Affiliation	Clinical Research Support Office, National Cancer Center Hospital
	Title	Medical officer

16.14.6. Coordination management practitioner

Organizations in charge of coordination and management		JCOG Operations Office
Coordinating and managing practitioners	Name	Junko Eba
	e-Rad number	80754085
	Affiliation	Clinical Research Support Office, National Cancer Center Hospital
	Title	Medical officer

16.14.7. Personnel who oversees the study other than Principal Investigator and site investigators

Personnel who oversees the study other than Principal Investigator and site investigators	Name	Not applicable	
	e-Rad number		
	Affiliation		
	Title		
	Relevance of Secondary Sponsor	□ Applicable	□ Not applicable

16.14.8. Study group personnel

JCOG Data Center

Statistics Section　　Gakuto OGAWA

Data Management Section　　Kyoko HASEGAWA

JCOG Operations Office

Science Section　　Tomoko KATAOKA/Tadayoshi HASHIMOTO

In addition, JCOG Data Center/Operations Office commissioned some of theire research support activities (such as support for the preparation of study protocols, data management, and site-visit audits) to other corporations. The commissioned duties are supervised by the National Cancer Center through routine work, as well as by receiving work reports from the institution and providing supervisory guidance. The current consignee is as follows

Clinical Oncology Research and Education, a specified non-profit organization

〒104-0061 DJ Ginza Building 7F, 8-18-3, Ginza, Chuo-ku, Tokyo

Official website　　http://www.core.or.jp/

16.15. Developing a study protocol

Chigusa MORIZANE, National Cancer Center Hospital

Nozomu MACHIDA, Shizuoka Cancer Center

Yoshitaka HONMA/Ken KATO, National Cancer Center Hospital

Support for protocol development

JCOG Data Center

Statistics Section (in charge of study design)　　Junki MIZUSAWA

Data Management Section (CRF preparation)　　Harumi KABA

JCOG Operations Office

Protocol development　　Hiroshi KATAYAMA/Kozo KATAOKA/Aya MIURA

Person in charge of IC documents　　Aya KIMURA/Noriko TSUJI

17. Publication of the study results and completion of the study

17.1. Paper and conference presentations

Primary publication will be published in English journals.

Paper publication including review article and conference presentation of Introduction of the study, by Principal Investigator or Study Coordinator, which does not include the analytical results of the endpoint of the study, are allowed when Group Chair and JCOG Data Center Director agree to them. Publication of the distribution of baseline factors or the safety data after the end of accrual are also allowed. No publication other than primary analysis and final analysis will be performed unless previously approved by Data and Safety Monitoring Committee.

In principle, the authors of the main published papers on the results of the study (the first publication of the results of primary endpoint) shall be the first Study Coordinator (HBPOG), followed by Study Coordinator (JEOG or SCSG), Study Coordinator (JEOG or SCSG), Principal Investigator (HBPOG), Principal Investigator (JEOG or SCSG), Principal Investigator (JEOG or SCSG), the statistical staff of Data Center (one statistician in charge at the time of the analysis for publication), Centralized Pathological diagnosis Coordinator (HBP), Centralized Pathological diagnosis Coordinator (GI). In accordance with the limitations imposed by the article's posting provisions, researchers who contributed in descending order of their number of patients registrations were selected for each institution as co-authors, and the last author was Group Chair (or Study Chair). The inclusion of staffs of JCOG Operations Office as co-authors will be determined by Group Chair depending on their contributions. Authors of articles other than the primary publication (e.g., Secondary endpoints articles, secondary analysis articles) will be determined by Study Chair with Group Chair approval.

All co-authors will review the article contents prior to submission for publication and only those who agree to the article contents. If there is no consensus on the contents, principle investigator may not include the investigator in the co-authorship with the approval of Group Chair. If there is no consensus between groups and JCOG Data Center/Operations Office, ultimately follow JCOG Chair instructions.

17.2. Primary Endpoint Report and Clinical Summary Report

The procedures are specified from the preparation of the primary endpoint report and clinical summary report. If primary analysis is the final analysis, the primary endpoint report will not be prepared and the clinical summary report will be prepared.

17.2.1. Clinical Summary Report

Based on the final analysis report, Principal Investigator will prepare a "Abstract of the Clinical Summary Report" containing subjects background information (age, sex, etc.), study design and study progress, results of analyses for each endpoint, conclusions of the entire study, interpretations and discussion of the results, etc. within 6 months from the issue date of the final analysis report, submit it to the Data Center, and undergo review. In addition, the issue date of the final analysis report shall be "the date when the period for collecting data on all endpoints is completed" as specified in the Enforcement Regulations.

After obtaining approval from Group Chair and the Director of the Data Center, abstract of the clinical summary report will be submitted to Certified Review Board review as a "clinical summary report" with the final analysis report within 1 year of the issue date of the final analysis report.

Principal Investigator/Study Coordinator will disclose abstract of the clinical summary report (Notification of Completion of Form 1 of Article 24 of Regulation) to jRCT within 1 month after obtaining Certified Review Board approval (if the report is unpublished, abstract will not be released in jRCT and will be published immediately after publication).

The approved abstract of the clinical summary report will be submitted to the administrators of each participating institution through the site investigators at each institution and to JCOG Chair. Abstract of the clinical summary report is available on JCOG website (http://www.JCOG.jp/).

The timing of distribution of the final analysis report to participating institutions will be determined by Principal Investigator/Study Coordinator considering the timing of publication of the final analysis results, and the final analysis report will be distributed to the researchers at participating institutions by themselves or through the Data Center.

17.3. Completion of the study

On the date that abstract of the clinical summary report was released to iRCT, the study is completed.

In institutions where patients were not enrolled, the institution may be withdrawn from the institution list by submitting a request for change (Unified Form 3 for Clinical Research) and a Notification of Changes in Protocol (Form 2) stating that Principal Investigator withdraws the institution from the institution list in question to Certified Review Board, and after obtaining approval, notifying the MHLW (Local Health Bureau responsible for the location of Certified Review Board). When a protocol change notification is received by the Local Health Service, the investigator at the institution shall report the withdrawal to the Administrator of the institution.

18. References

omit

19. Appendix

- Informed consent form
- Body surface area table
- Toxicity Criteria (CTCAE v5.0-JCOG)
- CRF samples* (CRF draft attached for the first review submission)
- JCOG-Biobank Japan Biorepository Protocol
- JCOG-Biobank Japan Biorepository Informed Consent Form

Japan Clinical Oncology Group
Hepatobiliary and Pancreatic Oncology Group/Stomach Cancer Study Group/Japan Esophageal Oncology Group

Practical Research for Innovative Cancer Control from Japan Agency for Medical Research and Development
"Establishment of standard treatments for neuroendocrine carcinoma of the digestive system"
National Cancer Center Research and Development Fund 29-A-3

JCOG1213

Randomized phase III study of etoposide plus cisplatin combination therapy versus irinotecan plus cisplatin combination therapy in advanced neuroendocrine carcinoma of the digestive system. ver.1.2.2

TOPIC-NEC

HBPOG
Group Chair: Junji FURUSE
Department of Medical Oncology, Kyorin University Faculty of Medicine

Study Chair: Takuji OKUSAKA
Department of Hepatobiliary and Pancreatic Oncology, National Cancer Center Hospital 5-1-1 Tsukiji, Chuo-ku, Tokyo 104-0045 Japan
Tel: +81-3-3542-2511
E -mail：tokusaka@ncc.go.jp

Study Coordinator: Chigusa MORIZANE
Department of Hepatobiliary and Pancreatic Oncology, National Cancer Center Hospital 5-1-1 Tsukiji, Chuo-ku, Tokyo 104-0045 Japan
Tel: +81-3-3542-2511
E -mail：cmorizan@ncc.go.jp

SCSG
Group Chair: Masanori TERASHIMA
Division of Gastric Surgery, Shizuoka Cancer Center Hospital

Study Chair: Narikazu BOKU
Department of Gastrointestinal Oncology, National Cancer Center Hospital
5-1-1 Tsukiji, Chuo-ku, Tokyo 104-0045 Japan
Tel: +81-3-3542-2511
E -mail： nboku@ncc.go.jp

Study Coordinator: Nozomu MACHIDA
Department of Hepatobiliary and Pancreatic Oncology, Shizuoka Cancer Center
1007 Shimonagakubo, Nagaizumi-cho, Sunto-gun, Shizuoka Prefecture 411-8777 Japan
Tel: +81-55-989-5222
E -mail：no.machida@scchr.jp

JEOG
Group Chair: Yuko KITAGAWA
General and Gastroenterological Surgery, Keio University Hospital

Study Chair: Ken KATO
Department of Gastrointestinal Oncology, National Cancer Center Hospital 5-1-1 Tsukiji, Chuo-ku, Tokyo 104-0045 Japan
Tel: +81-3-3542-2511
E -mail：kenkato@ncc.go.jp

Study Coordinator: Yoshitaka HONMA/Ken KATO
Department of Gastrointestinal Oncology, National Cancer Center Hospital 5-1-1 Tsukiji, Chuo-ku, Tokyo 104-0045 Japan
Tel: +81-3-3542-2511
E-mail:yohonma@ncc.go.jp
/kenkato@ncc.go.jp

2013/3/16	Protocol concept approved by JCOG Executive Committee (PC1213/1214/1215)
2014/6/27	Protocol approved byJCOG Protocol Review Committee
2017/5/23	Revision ver. 1.1 approved by JCOG Data and Safety Monitring Committee
2018/8/16	Revision ver. 1.2 approved by JCOG Data and Safety Monitring Committee
2018/10/11	Approved by Certified Review Board of National Cancer Center Hospital

0. Summary

This study is conducted as a "specified clinical trial" based on the Clinical Trials Act (Act No. 16 of April 14, 2017). In this protocol, the Principal Investigator is the Study Chair in the Hepatobiliary and Pancreatic Oncology Group of JCOG.

Name of study: "Randomized phase III study of etoposide plus cisplatin combination therapy versus irinotecan plus cisplatin combination therapy in advanced neuroendocrine carcinoma of the digestive system. (TOPIC-NEC) ".

Public study title: "Randomized phase III study of etoposide plus cisplatin combination therapy versus irinotecan plus cisplatin combination therapy in advanced neuroendocrine carcinoma of the digestive system. (TOPIC-NEC) "

0.1. Schema

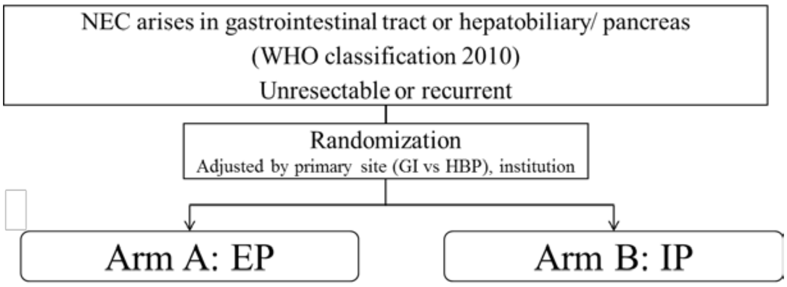

0.2. Objectives

A randomized phase III study was conducted to determine the better treatment option between etoposide/cisplatin combined therapy (EP therapy) or irinotecan/cisplatin combined therapy (IP therapy), both of which are standard treatments for non-resectable/recurrent neuroendocrine carcinoma (NEC as classified by WHO in 2010) with primary lesions in the gastrointestinal tract/hepatobiliary pancreatic organs.

Primary endpoint: Overall survival

Secondary endpoints: Response rate in case of measurable lesions, Progression-free survival (PFS), incidence rate of adverse events, dose intensity of Cisplatin, and incidence rate of serious adverse events.

0.3. Subjects

1) Any of the following is applicable based on pathological diagnosis taking findings of immunohistochemistry into consideration (see 3.1. to 3.3.).

[1] Pathologically diagnosed as neuroendocrine carcinoma (NEC*1) in the resected sample.

[2] Containing pathologically confirmed component of neuroendocrine carcinoma (NEC*1) in the biopsy sample.

1: Based on WHO 2010 classification

2) Any of the following is applicable

[1] NEC arise in esophagus, stomach, duodenum, intestine, appendix, colon, rectum, gallbladder, intrahepatic bile duct, extrahepatic bile duct, ampulla of Vater, pancreas,

[2] Liver NEC (primary liver or liver metastasis of unknown primary) *2.

*2: The tumor is only in one of the following sites after a thorough examination of the primary site by contrast CT (from the neck to pelvic) and upper/lower gastrointestinal endoscopy, FDG-PET scan, otolaryngology (head and neck) examination, urology examination (male patients only), and gynecology examination (female patients only).

a. Liver only

b. Bone, lymph nodes below the diaphragm, peritoneum, subcutaneous, muscle, spleen, and liver

3) Unresectable (see 3.6) or recurrent cancer (see 3.7). It is not essential for a pathological confirmation of the metastatic lesion or recurrent site. Cases of esophageal NEC is ineligible if corresponding to any of the following.

[1] cT4.

[2] No distant metastasis rather than supraclavicular lymph node

[3] Stenosis indicated for palliative radiotherapy

4) No previous chemotherapy or radiotherapy for NEC. Pre- or post-operative chemotherapy except irinotecan or

etoposide for NEC is allowed as long as it was completed at least 8 weeks prior to registration.

5) No previous chemotherapy using platinum agents for any malignancies.

6) No serious tumor-related complications.

Serious tumor-related complications include superior vena cava syndrome, inferior vena cava syndrome, pleural, ascites, or pericardial effusions that are large or uncontrollable (e.g., effusions that accumulate quickly after drainage or even after adhesive surgery), and brain metastases with neurological symptoms.

7) Aged 20 to 75 years old.

8) ECOG performance status of 0 or 1.

9) Sufficient oral intake

10) Measurable region is not required.

11) Adequate organ functions.

[1] WBC ≥3,000/ mm^3

[2] Neutrophils ≥ 1,500/ mm^3

[3] Hemoglobin ≥ 9.0 g/dL

[4] Platelets ≥ 10×10^4/ mm^3

[5] Total bilirubin≤ 1.5 mg/dL[※4]

[6] AST(sGOT)≦100 IU/L[※4]（for hepatic NEC and liver metastasis, ≦150 IU/L）

[7] ALT(sGPT)≦100 IU/L[※4]（for hepatic NEC and liver metastasis, ≦150 IU/L）

※4：Presence or absence of biliary drainage is not relevant

[8] Serum creatinine≦ 1.3 mg/dL

[9] Creatinine clearance[※5]≥ 60 mL/min

※5： Creatinine clearance must have been estimated using the Cockcroft-Gault formula, and must be 60 mL/min or more. If the estimation is less than 60 mL/min, but the actual measurement is 60 mL/min or more, the patient can be deemed eligible.

Cockcroft-Gault formula

Male：Ccr ={(140－age) × body weight (kg)}/{72×serum creatinine (mg/dL)}

Female：Ccr = 0.85×{(140－age) × body weight (kg)}/{72×serum creatinine(mg/dL)}

12) Written informed consent.

0.4. Treatments

Arm A: etoposide plus cisplatin (EP) arm

The following chemotherapy is continued until the patient meets discontinuation criteria, with 3-weeks of treatments counting as one cycle.

Drug	Dosage (mg/m^2)	Dosing regimen/Dosing time	Dose day
Etoposide	100	IV/60-120 min	Day 1, 2, 3
Cisplatin	80	IV/60-120 min	Day 1

Arm B: Irinotecan plus Cisplatin (IP) arm

The following chemotherapy would be continued until the patient meets discontinuation criteria, with 4-weeks of treatments counting as one cycle.

Drug	Dose (mg/m^2)	Dosing regimen/Dosing time	Dose day
Irinotecan	60	IV/90 min	Day 1, 8, 15
Cisplatin	60	IV/60-120 min	Day 1

0.5. Planned sample size and study period

The planned sample size is 170.

Accrual period: 6 years.

Follow-up period: 1 years after accrual completion.

Analysis period: 1 year.

Total study duration 8 years.

Scheduled start date of the study Aug 8, 2014

Expected completion date of the study Aug 8, 2028

0.6. Contact information

Eligibility criteria, treatment modification, and other issues requiring clinical decisions: Study Coordinator (front cover and 16.6.)

Enrollment procedure protocol, case report form (CRF) entries, etc.: JCOG Data Center, (16.14.)

Adverse event reporting: JCOG Data and Safety Monitoring Committee (16.11.),

Contents

0. SUMMARY ... 2

0.1. SCHEMA ... 2
0.2. OBJECTIVES ... 2
0.3. SUBJECTS ... 2
0.4. TREATMENTS ... 3
0.5. PLANNED SAMPLE SIZE AND STUDY PERIOD ... 3
0.6. CONTACT INFORMATION ... 4

1. OBJECTIVES ... 8

2. BACKGROUND ... 8

2.1. TARGET ... 8
2.2. STANDARD TREATMENT FOR TARGET DISEASE ... 16
2.3. RATIONALE FOR ESTABLISHMENT OF TREATMENT PLAN ... 18
2.4. STUDY DESIGN ... 21
2.5. SUMMARY OF EXPECTED ADVANTAGES AND DISADVANTAGES ASSOCIATED WITH STUDY PARTICIPATION ... 22
2.6. SIGNIFICANCE OF THIS STUDY ... 23
2.7. ASSOCIATED RESEARCH (INCLUDING SAMPLE ANALYSIS RESEARCH) ... 23
2.8. JCOG-BIOBANK JAPAN (BBJ) COLLABORATING BIOBANK ... 23

3. CRITERIA/DEFINITIONS USED IN THIS STUDY ... 25

3.1. TISSUE CLASSIFICATION (WHO 2010 CLASSIFICATION) ... 25
3.2. GRADE CLASSIFICATION （ENETS [EUROPEAN NEUROENDOCRINE TUMOR SOCIETY] / WHO2010 CLASSIFICATION） ... 25
3.3. HISTOPATHOLOGICAL DIAGNOSIS ... 25
3.4. DISEASE STAGE CLASSIFICATION CRITERIA (UICC-TNM 7TH EDITION) ... 25
3.5. RESIDUAL TUMOR (R) CLASSIFICATION (UICC-TNM 7TH EDITION) ... 33
3.6. DEFINITION OF NON-RESECTABLE NEC ... 33
3.7. DEFINITION OF RECURRENT NEC ... 34
3.8. DEFINITION OF HEPATIC NEC (HEPATIC PRIMARY LESION OR LIVER METASTASIS FROM AN UNKNOWN PRIMARY LESION) 34

4. PATIENT SELECTION CRITERIA ... 34

4.1. INCLUSION CRITERIA (FOR ENROLLMENT) ... 34
4.2. EXCLUSION CRITERIA ... 35

5. REGISTRATION AND RANDOMIZATION ... 36

5.1. PROCEDURE OF REGISTRATION ... 36
5.2. RANDOMIZATION AND ALLOCATION ADJUSTMENT FACTOR ... 37

6. TREATMENT PLAN AND TREATMENT MODIFICATION CRITERIA ... 38

6.1. PROTOCOL TREATMENT ... 38
6.2. PROTOCOL TREATMENT TERMINATION/COMPLETION CRITERIA ... 41
6.3. TREATMENT MODIFICATION CRITERIA ... 42
6.4. CONCOMITANT TREATMENT AND SUPPORTIVE CARE ... 48
6.5. POST-STUDY TREATMENT ... 55

7. ANTICIPATED ADVERSE EVENTS ... 56

7.1. ANTICIPATED ADVERSE REACTIONS ... 56

7.2. ANTICIPATED ADVERSE EVENTS DUE TO PATHOGENESIS 59
7.3. EVALUATION OF ADVERSE EVENTS/REACTIONS 60

8. EXAMINATION AND EVALUATION 62

8.1. BASELINE EXAMINATION AND EVALUATION BEFORE REGISTRATION 62
8.2. EXAMINATION AND EVALUATION DURING TREATMENT 63
8.3. EXAMINATION AND EVALUATION AFTER COMPLETION OF TREATMENT 64
8.4. INFORMATION ON POST- STUDY TREATMENT 64
8.5. STUDY CALENDAR 65

9. DATA COLLECTION 69

9.1. CASE REPORT FORM (CRF) 69

10. REPORTING OF "DISEASE OR THE LIKE"(ADVERSE EVENTS) 70

10.1. SERIOUS ADVERSE EVENTS AND SUBJECTS OF EXPEDITED REPORTING. 70
10.2. INVESTIGATOR'S REPORTING REQUIREMENTS AND PROCEDURES 71
10.3. RESPONSIBILITIES OF PRINCIPAL INVESTIGATOR/STUDY COORDINATOR 73
10.4. RESPONSIBILITIES OF THE SITE INVESTIGATORS AT THE PARTICIPATING INSTITUTIONS (INCLUDING THE RELEVANT INSTITUTION) 75
10.5. RESPONSIBILITIES OF THE DATA AND SAFETY MONITORING COMMITTEE 75

11. RESPONSE EVALUATION AND ENDPOINT DEFINITION 76

11.1. RESPONSE ASSESSMENT (ONLY FOR PATIENTS WITH MEASURABLE DISEASE) 76
11.2. DEFINITIONS OF ANALYSES SET 82
11.3. DEFINITION OF ENDPOINTS 82

12. STATISTICAL CONSIDERATION 85

12.1. PRINCIPAL ANALYSIS AND DECISION CRITERIA 85
12.2. PLANNED ACCRUAL, ACCRUAL PERIOD, AND FOLLOW-UP PERIODS 86
12.3. INTERIM ANALYSIS AND EARLY TERMINATION OF THE STUDY 86
12.4. ANALYSIS OF SECONDARY ENDPOINTS 88
12.5. FINAL ANALYSIS 88
12.6. EXPLORATORY ANALYSIS 89
12.7. PREMATURE WITHDRAWAL FROM THE TRIAL 89
12.8. PROCEDURES AFTER EARLY TERMINATION OF THE STUDY 90

13. ETHICAL CONSIDERATIONS 92

13.1. PROTECTION OF HUMAN SUBJECTS 92
13.2. INFORMED CONSENT 92
13.3. PROTECTION OF PERSONAL INFORMATION AND PATIENT IDENTIFICATION 94
13.4. ADHERENCE TO THE PROTOCOL 96
13.5. APPLICATION TO CERTIFIED REVIEW BOARD AND NOTIFICATION OF IMPLEMENTATION PLANS 96
13.6. PROTOCOL REVISION/AMENDMENT 100
13.7. CONFLICTS OF INTEREST (COIS) INVOLVED IN THIS STUDY 102
13.8. COMPENSATION 104
13.9. INTELLECTUAL PROPERTY 105
13.10. DISCLOSURE OF INFORMATION ON THIS STUDY 105

14. MONITORING AND AUDIT 106

14.1. PERIODIC MONITORING 106
14.2. SITE VISIT AUDITS 108

14.3. MANAGEMENT OF NON-COMPLIANCE 109

15. SPECIAL INSTRUCTIONS 110

15.2. JCOG BIOBANK JAPAN (BBJ) BIOREPOSITORY 111

16. ORGANIZATION 111

16.1. MAIN STUDY FUND (FUNDING SOURCE) OF THIS STUDY. 111
16.2. JAPAN CLINICAL ONCOLOGY GROUP (JCOG) 111
16.3. JCOG CHAIR 112
16.4. STUDY GROUP AND GROUP CHAIR 112
16.5. STUDY CHAIR (PRINCIPAL INVESTIGATOR) 113
16.6. STUDY COORDINATOR 113
16.7. CENTRAL PATHOLOGICAL REVIEW COORDINATOR 114
16.8. CENTRAL PATHOLOGICAL REVIEW COORDINATOR 114
16.9. PARTICIPATING SITES (PARTICIPATING INSTITUTIONS) 115
16.10. JCOG PROTOCOL REVIEW COMMITTEE 116
16.11. JCOG DATA AND SAFETY MONITORING COMMITTEE 116
16.12. JCOG AUDIT COMMITTEE 116
16.13. JCOG CONFLICT OF INTEREST COMMITTEE 116
16.14. DATA CENTER/OPERATIONS OFFICE 116
16.15. DEVELOPING A STUDY PROTOCOL 118

17. PUBLICATION OF THE STUDY RESULTS AND COMPLETION OF THE STUDY 119

17.1. PAPER AND CONFERENCE PRESENTATIONS 119
17.2. PRIMARY ENDPOINT REPORT AND CLINICAL SUMMARY REPORT 119
17.3. COMPLETION OF THE STUDY 120

18. REFERENCES 121

19. APPENDIX 121

1. Objectives

A randomized phase III study was conducted to determine the better treatment option between etoposide/cisplatin combined therapy (EP therapy) or irinotecan/cisplatin combined therapy (IP therapy), both of which are standard treatments for non-resectable/recurrent neuroendocrine carcinoma (NEC as classified by WHO in 2010) with primary lesions in the gastrointestinal tract/hepatobiliary pancreatic organs.

Primary endpoint: Overall survival

Secondary endpoints: Response rate in case of measurable lesions
Progression-free survival (PFS), incidence rate of adverse events, dose intensity of Cisplatin, and incidence rate of serious adverse events

2. Background

2.1. Target

2.1.1. Epidemiology

Neuroendocrine tumors (NETs) and neuroendocrine carcinomas (NECs) can arise in various organs in the body. Of these, NECs with primary lesions occurring in the lungs, namely small cell lung cancer and large cell lung cancer, have been collectively referred to as high-grade NECs (HGNECs). However, terms such as extrapulmonary small cell carcinoma and extrapulmonary NEC have been used to refer collectively to NECs, with primary lesions outside the lungs.

The number of newly reported cases of neuroendocrine neoplasms (NENs) annually, estimated based on the cases registered in the U.S. SEER (Surveillance, Epidemiology, and End Results) database between 1973 to 2004 is 5 in 100,000 people[1]. In Japan, the Neuroendocrine Tumor Workshop Japan (NET Work Japan) carried out a nationwide survey of pancreatic and gastrointestinal NENs, and estimated the number of new cases in 2005 at approximately 1.01 people with pancreatic primary lesion per 100,000 people, and 2.10 people with gastrointestinal primary lesion per 100,000 people[2, 3].

The report by the PRONET Study Group in France concerning their prospective observational study is a good reference for the proportion of NENs that can be classified as NECs. Out of the 778 patients diagnosed with gastrointestinal primary lesion NENs in 80 facilities from August 2010 to July 2011, 104 patients (13.4%) had NEC[4].

The frequency of incidence of NECs according to the primary lesion organ has been shown in the tables below (Table 2.1.1a and Table 2.1.1b). Despite variations between reports, gastrointestinal primary lesion NECs account for approximately 20–68% of all extrapulmonary NEC cases. The breakdown of gastrointestinal primary lesion NEC cases with primary lesion in the gastrointestinal tract (esophagus, duodenum, small and large intestines) indicated that these cases accounted for 56–84% of the total cases, while hepatobiliary pancreatic cancer cases accounted for 15–35% of all cases.

Table 2.1.1a. Frequency of extrapulmonary NECs according to the organ of primary lesion

Reported year/Reporter	N	Gastrointestinal	Gynecological organs	Urology	Head and Neck	Adrenal	Primary unknown	Other
2012/Terashima [5]	136	68%	12%	6%	NA	NA	8%	7%
2010/Brennan [6]	74	20%	35%	15%	19%	NA	9%	1%
2009/Wong [7]	1618	33%	NA	20%	11%	10%	4%	22%
2006/Haider [8]	101	20%	11%	18%	12%	9%	31%	NA
2007/Lee [9]	61	56%	20%	10%	8%	NA	6%	NA

Table 2.1.1b. Percentage breakdown of NECs as per the primary lesion organ of gastrointestinal NEC

Reported year/Reporter	N	Esophageal	Gastric	Duodenal/small intestine	Colorectal	Hepatobiliary	Pancreas	Other
2012/Machida [10]	258	33%	27%	2%	12%	12%	14%	NA
2012/Sorbye [11)]	205	6%	10%	NA	40%	NA	35%	10%
2004/Brenner [12]	544	53%	11%	0.2%	20%	11%	4%	NA

2.1.2. Clinical pathology

1) About the term of target disease

The target of this study has a complex disease concept, and since the method of classification has changed with time, various names have been assigned that could cause confusion. In this study, we use classification and nomenclature defined according to the WHO 2010 classification（WHO Classification of Tumors of the Digestive System 2010）[13].

All the tumors originating from neuroendocrine cells or differentiation into endocrine cells are called neuroendocrine neoplasms (NENs). Depending on the malignancy, NENs were classified as Grade 1 NETs (NET G1), Grade 2 NETs (NET G2), or NEC.

Furthermore, prior to the WHO 2010 classification, all disease concepts equivalent to NENs were called NETs, but in this study, we would have referred to all descriptions of the disease concept as NENs. Furthermore, according to the 2000 and 2004 WHO classifications, the term well-differentiated NEC (WDNEC) has been used for disease units equivalent to NET G2, but in this study NEC does not mean the same.

2) Classification and clinical pathology

NEN is a tumor that develops from neuroendocrine cells present in various tissues or shows a tendency of differentiation into neuroendocrine cells. Histologically these tumors express neuroendocrine markers, such as Chromogranin A, NSE (neuron-specific enolase), and synaptophysin. The WHO classifications in 2000 and 2004 combined the presence or absence of metastasis/local infiltration and cellular proliferative capacity (evaluated based on Ki-67 expression intensity and mitotic presentation), and thereby classified these tumors into well-differentiated (neuro)endocrine tumors without metastasis/local infiltration, well-differentiated (neuro)endocrine tumors with metastasis/local infiltration, and poorly differentiated (neuro)endocrine tumors with even greater cell proliferative capacity. On the other hand, European Neuroendocrine Tumor Society (ENETS) has proposed a method of classification that evaluates the malignancy as Grade 1, 2, or 3 (G1, G2, or G3), according to the cell proliferative capacity (Ki-67 index or number of mitotic presentations), which has proven to be most useful for classification of prognosis[14, 15]。

Against this backdrop, the WHO classification that targeted gastrointestinal diseases was published in 2010. The disease as a whole was considered “neuroendocrine neoplasms (NEN)”, and the disease malignancy was largely classified as NET G1 (neuroendocrine tumor Grade 1), NET G2 (Grade 2), NEC, and mixed adenoneuroendocrine carcinoma (MANEC). MANEC refers to the cancers wherein adenocarcinoma components account for more than 30% of the cancer, while those under 30% are classified as NECs. However, even though this method of classification is applicable while evaluating the entire tumor based on resected specimen, it is not possible to evaluate the proportion of each component in the entire tumor during a diagnosis based on biopsy sample.

Each of NET G1, NET G2, and NECs described in the WHO 2010 classification is equivalent to G1, G2, and G3 as per the ENETS classification. NETs are well-differentiated tumors, with relatively low atypicality and malignancy, and the clinical course is slow with a 5-year survival in the range of 62–85%[16, 17]. On the other hand, NECs are poorly differentiated tumors, where tumor cells with poor cellular constituents proliferate diffusely, and are pathologically similar to small cell lung cancers, since these show many mitotic presentations, involve necrotic lesions, and present with neuroendocrinological features during immunohistochemical staining[1]. The clinical presentations are also similar in terms of the rapid proliferation of NECs and relatively high sensitivity to anticancer drugs[18].

Similar to lung cancer, NECs also exist as small cell cancer type or large cell type (LCNEC：large cell NEC）[13]. Future challenges include studies on the frequency and differences in clinical presentation of each disease. Table 2.1.2 shows the WHO and Grade classification.

Table 2.1.2. Changes in WHO classification and grading; Shaded parts are targets of this study

WHO 2000 classification	WHO 2010 classification		ENETS Grade
Well-differentiated endocrine tumor（WDNET）<br>1.1‘Benign’ behavior<br>1.2 Uncertain behavior	Neuroendocrine tumors, NETs<br>• Well-differentiated<br>• Composed of cells similar to normal gastrointestinal	NET G1<br>（carcinoid）	G1<br>Same as 2010 WHO classification<br>Number of mitotic presentations < 2 per 10

	endocrine cells<br>• Expression of neuroendocrine markers<br>• Hormone production<br>• Mild to moderate nuclear atypia, low proliferative capacity（Grade：G1,G2）		high power fields（HPF） and/or Ki-67 index ≤2%
Well-differentiated endocrine carcinoma（WDEC）		NET G2	G2<br>Number of mitotic presentations 2–20 per 10 HPF and/or Ki-67 index 3-20%
Poorly differentiated endocrine carcinoma/small cell carcinoma（PDEC）	Neuroendocrine carcinoma：NEC（large cell or small cell type）:<br>• Poorly differentiated, highly malignant<br>• Include small cell to large cell type carcinoma<br>• Sometimes present tissue structure similar to NET<br>• Expression of neuroendocrine markers<br>• Significant nuclear atypia, multifocal necrosis, and high proliferative capacity (Grade: G3)		G3<br>Number of mitotic presentations >20 per 10 HPF and/or Ki-67 index > 20%
Mixed exocrine-endocrine carcinoma（MEEC）	Mixed adenoendocrine carcinoma（MANEC）		
Tumor-like lesions（TLL）	Hyperplastic and preneoplastic lesions		

2.1.3. Staging

Disease staging for NENs is represented by the TNM classification proposed by ENETS (hereafter, ENETS TNM[14, 15], AJCC 7th Edition TNM classification and UICC 7th Edition TNM classification published in 2009. The AJCC 7th Edition and UICC 7th Edition TNM classifications are unified, and in this study we would have used the UICC 7th Edition nomenclature).

In the UICC 7th edition, there are independent TNM classifications of NET G1 and NET G2 with primary lesions in the stomach, small intestine, appendix, and colon, but with respect to NEC, it describes "classification according to main tissue type in each organ of primary lesion (squamous cell carcinoma for esophagus, and adenocarcinoma in all other organs of primary lesion)." While there are TNM classifications for hepatocellular carcinoma and intrahepatic cholangiocarcinoma in the liver, there is no description for the classification of the tissue type for NECs with primary lesion in the liver. In this study, however, we would have used the TNM classification of intrahepatic cholangiocarcinoma, which shows similar clinical presentations. Details of each TNM classification would have been mentioned in “3.2 staging criteria”.

2.1.4. Standard treatment according to disease staging and outline of prognosis

1) Standard treatment for resectable cases

Surgical resection is performed for resectable cases. The significance of postoperative adjuvant chemotherapy is not clear because a verification study has not yet been performed. Although the NCCN Guidelines[19] describe postoperative adjuvant therapy as the chemotherapy regimen for small cell lung cancers that is typically applied to advanced cases of NEC, it has relatively high toxicity and patient burden (see Table 2.2.2, 2.3.2); hence we cannot claim that it is widely used in routine practice. For NECs with gastrointestinal primary lesion with mixed adenocarcinoma, a chemotherapy regimen is suggested typically after surgery for adenocarcinoma; however, in practice, there is no consensus on postoperative adjuvant chemotherapy.

2) Standard treatment for locally advanced cases

While the NCCN Guidelines propose chemoradiotherapy for small cell lung cancer for locally advanced cases, the ENETS Guidelines provide no clear indication[20, 21] for the same. As there is no sufficient information related to radiation dose, efficacy, and safety for NECs with a multitude of primary lesion organs (scope of irradiation), radiation therapy is not widely used for pathologies other than NEC with esophageal primary lesion that is prone to stenosis, and treatment is often provided for distant metastasis.

For locally advanced cases of NEC with esophageal primary lesion, chemoradiotherapy is actively performed. Furthermore, supraclavicular lymph node metastasis in thoracic esophageal tumor is classified as Stage IV, and chemoradiotherapy is applied if metastatic lymph nodes are included in the field of irradiation.

3) Standard treatment for distal metastasis cases

Systemic chemotherapy is indicated for distal metastasis and recurrent cases regardless of the organ, and a regimen is chosen for the small cell lung cancer. With respect to NECs with esophageal primary lesion, even if there is organ metastasis, palliative chemoradiotherapy is prioritized for patients incapable of oral intake of drugs due to esophageal constriction.

The efficacy of everolimus and sunitinib has been demonstrated for NETs with pancreatic primary lesions. Furthermore, Octreotide and Lantreotide effectively inhibit proliferation of NETs that originate in the midgut and pancreatic/gastrointestinal NETs, respectively. However, these results are from clinical trials conducted in patient populations corresponding to NETs. Since the clinical presentations of NETs and NECs differ significantly, these treatments cannot be indicated for NECs during routine practice.

4) Indication of resection for distal metastasis

There is no consensus on the resection criteria specific to the NECs. Since the progression of NETs is slow, resection is actively considered even where there is distal metastasis to the liver, but as NEC progresses fast, surgical resection cannot be indicated for cases with distal metastasis[19, 21].

5) Prognosis

In a preceding study, Yamaguchi and Machida carried out a multicenter joint observational study of poorly

differentiated NEC (WHO 2010 NEC, including patients with clinical diagnosis of poor differentiation) in a total of 23 facilities, including facilities participating in JCOG Hepatobiliary and Pancreatic Oncology Group, former Gastroenterology Group, and observer facilities.

In the multicenter joint study by Yamaguchi and Machida, 89% of cases (229/258 patients) receiving systemic chemotherapy had distal metastasis, with a median survival time (MST) of 11.5 months for all patients, 11.2 months for distal metastasis cases, and 15.9 months for locally advanced cases. MST according to organ was 13.4 months for esophageal primary lesion (N = 85), 13.3 months for gastric primary lesion (N = 70), 29.7 months for small intestine/duodenal primary lesion (N = 6), 7.6 months for colonic primary lesion (N = 31), 8.5 months for pancreatic primary lesion (N = 35), and 7.9 months for hepatobiliary primary lesions (N = 31) (Table 2.1.4). While there were 9 cases of five-year survival among all the gastrointestinal cases, there were no five-year survival cases amongst those with primary lesions in hepatobiliary and pancreatic organs[10]. In a multicenter joint observational study for NECs conducted in four Northern European countries (hereafter referred to as NORDIC NEC Study), the MST for NECs with gastrointestinal primary lesion (albeit including ~30% of NECs with primary lesion sites unknown) was 11 months[11]. According to the U.S. SEER database, the MST of NEC was 10 months[3]. Based on the above information, despite differences in the primary lesion organ, the prognosis of the target population of this study is thought to have an overall MST of 10–11 months.

Table 2.1.4. Treatment results according to primary lesion organ for non-resectable/recurrent NECs that underwent systemic chemotherapy

	Esophageal	Gastric	Duodenal/small intestine	Colonic	**Gastrointestinal overall**	Pancreatic	Hepatobiliary	**Hepatobiliary and pancreatic organs overall**
N	85	70	6	31	**192**	35	31	**66**
Response rate（%）	58%	43%	50%	29%	**47%**	17%	16%	**17%**
Median PFS（month）	5.8	4.9	7.3	3.7	**5.1**	3.2	4.1	**3.7**
MST（month）	13.4	13.3	29.7	7.6	**13**	7.9	8.5	**7.9**

2.1.5. Tumor-related complications

We, here, report the complications that require attention during patient management, according to the organ of primary lesion. It is a rare disease and frequencies remain unknown.

1) Esophageal primary

Tumor bleeding/pain/esophageal stenosis/fistula formation due to primary lesion in the esophagus; hoarseness/difficulty swallowing/aspiration pneumonia caused by recurrent laryngeal nerve palsy due to lesion in lymph node metastasis; jaundice and liver failure associated with liver metastasis; respiratory failure and hemoptysis associated with lung metastasis, airway constriction, and pain due to lymph node metastasis; hypercalcemia, tracheal stenosis, tracheal obstruction, and suture failure due to disease progression.

2) Gastric primary

Chronic bleeding from primary lesion, accompanied by anemia, stomach pain, nausea, vomiting, gastric perforation, bloating, suture failure, fistula formation, cardiac/pyloric stenosis, ascites retention due to peritoneal metastasis, ileus, hydronephrosis due to ureteral stenosis, obstructive jaundice/liver failure due to bile duct stenosis, and obstructive jaundice/liver failure due to hepatic portal lymph node metastasis.

3) Small intestine/colonic primary

Tumor hemorrhage, ileus, fistula formation in the small intestine/bladder/vagina, intestinal obstruction, intestinal stenosis, intestinal perforation, and pelvic infection

4) Hepatobiliary and pancreatic primary

Cancer pain, rupture, obstructive jaundice associated with the growth of primary tumor, weight loss, fever, pancreatitis, cholangitis, liver abscess, cholecystitis, biliary hemorrhage, duodenal hemorrhage, duodenal stenosis, anastomotic ulcer/stenosis/leakage, symptoms associated with stenosis/obstruction of portal vein (liver

dysfunction/liver failure, esophageal varices and their rupture, gastritis, ascites retention, and hepatic encephalopathy/coma associated with portal hypertension).

5) Paraneoplastic syndrome

In addition to NECs, there are reports of paraneoplastic syndrome that is often associated with small cell lung carcinoma[22-24]. We report examples of paraneoplastic syndrome seen with small cell lung carcinoma below.

Hyponatremia due to inappropriate sodium secretion, psychological symptoms (changes in personality) due to ectopic ACTH syndrome, hypertension, hypokalemia, high blood sugar, Lambert-Eaton myasthenic syndrome, paraneoplastic cerebellar degeneration associated with autoantibody production (ataxia in the limbs, dysarthria, and nystagmus), paraneoplastic encephalomyelitis/sensory neuron disease (dementia, cranial nerve symptoms, dizziness, ataxia, autonomic imbalance, transverse paralysis, and sensory disorders).

Apart from these, there are thromboembolic events, aspiration (due to gastrointestinal stenosis), anemia, tumor pain, acute renal failure, myositis, and pulmonary fibrosis.

6) Complications due to metastatic lesions

Liver failure, pain, hepatobiliary infection, hemorrhage, bile duct stenosis, bile duct obstruction, respiratory failure, tracheal hemorrhage, respiratory tract infection, atelectasis, airway constriction, ascites, bloating, ileus, ureteral stenosis, ureteral obstruction, urinary retention, urinary tract infection, hiccups, pleural effusion, pleural hemorrhage, chest pain, pathological fracture, pain, hypercalcemia, meningeal carcinomatosis, ataxia, ischemia cerebrovascular, intracranial hemorrhage, nausea, vomiting, dizziness, consciousness disorder, cognitive disturbance, dysphasia, seizure, spasticity, edema limbs, intestinal obstruction/stenosis/perforation, pancreatitis, disseminated intravascular coagulation, thrombocytopenia, anorectal infection, anal hemorrhage, hoarseness, and superior vena cava syndrome

7) Others

General pain management including narcotic analgesics is performed for cancer pain.

Gastrointestinal stenting and bypass surgery are performed for esophageal stenosis, cardiac/pyloric stenosis, and duodenal stenosis.

Percutaneous transhepatic cholangial drainage (PTCD), percutaneous or endoscopic stenting, and bile duct jejunostomy is performed for obstructive jaundice. In addition, complications associated with biliary drainage and gastrointestinal stenting are listed below.

- PTCD, including internal and external fistula tube placement:
  Cholangitis, pancreatitis, cholecystitis, liver abscess, sepsis, biliary hemorrhage, PTCD tube obstruction/deviation, peritonitis, and pneumothorax/pleurisy
- Biliary stenting:
  Cholangitis, pancreatitis, cholecystitis, liver abscess, sepsis, biliary hemorrhage, peritonitis, pneumothorax/pleurisy (for percutaneous stenting), duodenal perforation, pneumonia (for endoscopic stenting), stent obstruction/deviation, duodenal ulcer, and duodenal hemorrhage
- Bile duct jejunostomy:
  Cholangitis, pancreatitis, cholecystitis, liver abscess, and sepsis
- Gastrointestinal stenting:
  Hemorrhage, perforation, pain, stent deviation, stent obstruction, foreign-body sensation, intestinal strangulation, ulcer formation, fever, sepsis, infection, diarrhea, constipation, tenesmus or uncontrollable urination/incontinence symptoms (colon), thyroid injury (esophagus), carotid artery injury (esophagus), and mediastinal abscess (esophagus).

2.1.6. Recurrent/progression

NEC is a very rare disease, and most reports of cases of surgery coincide with the case reports. According to the review by Arai et al., out of the 55 patients with NEC with gastric primary lesion who underwent surgical resection, only three patients survived for two years or more[25]. According to the report by Fischer et al., the MST of 13 patients with NEC with pancreatic primary lesion who underwent surgical resection was 11.7 months (seven of the patients showed distal metastasis)[26]. The form of recurrence among surgery cases remains unknown. The multicenter joint

study by Yamaguchi and Machida reported PFS among cases that underwent systemic chemotherapy with respect to the median as 5.8 months for esophageal primary lesions, 4.8 months for gastric primary lesions, 7.3 months for duodenal small intestine primary lesions, 3.7 months for colonic primary lesions, 3.2 months for pancreatic primary lesions, and 4.1 months for hepatic/biliary primary lesions[10]. In the NORDIC NEC study, the median PFS was 3 months for esophageal primary lesions, 5 months for gastric primary lesions, 3 months for colonic primary lesions, 4 months for rectal primary lesions, 5 months for pancreatic primary lesions, and 4 months for cases with unknown primary lesion site [11].

2.1.7. Prognostic/predictive factors

When prognostic factors were examined by multivariate analysis in the multicenter joint study by Yamaguchi and Machida, out of the various factors such as sex, age (younger or older than 60 years), PS (0 or 1 vs. 2 or more), primary lesion organ (gastrointestinal primary lesions vs. hepatobiliary or pancreatic primary lesions), LDH levels (below vs. above the upper limit of facility standard), presence or absence of liver metastasis, presence or absence of history of radical resection and treatment regimen (IP therapy vs. EP therapy); the independent prognostic factors identified were gastrointestinal primary lesion (vs. hepatobiliary or pancreatic primary lesion, hazard ratio (HR): 0.58), and LDH levels being below the upper limit of facility standard for LDH (vs. above the upper limit of facility standard for LDH, HR: 0.65).

Although IP therapy exhibited slightly better impact on overall survival as compared to EP therapy with an HR of 0.8, the *p*-value of 0.389 meant that there was no significant difference between the two therapies[10]. The NORIDIC NEC study reported poor PS, colonic primary lesions, high platelet count, and high LDH levels to be the main factors behind poor prognosis[11]. However, these prognostic/predictive factors were not obtained with a global consensus.

2.1.8. Rationale for selection of the target population

The objective of this study is to develop a primary chemotherapy regimen for non-resectable/recurrent NECs, and out of the non-resectable or postoperatively recurrent NECs with gastrointestinal primary lesions or hepatobiliary or pancreatic primary lesions (shaded parts in Table 2.1.2 of WHO 2010 classification of NEC), we established chemotherapy-naïve patients to be the target population.

In terms of whether or not to consider MANEC a target of this study, a discussion is needed from the viewpoint of standard treatment. MANEC is considered when 30% or more of the carcinoma consists of adenocarcinoma components, and it is treated as either adenocarcinoma or NEC by discretion of the attending physician, with no real consensus on standard treatment. MANEC was however, excluded from this study, as its disease concept is different from NEC in terms of the standard treatment, which has a consensus regarding the treatment by a “regimen according to small cell lung carcinoma”. In fact, a questionnaire was provided to the three groups participating in this JCOG study (response obtained from 52 facilities). The results showed that 79% of facilities considered the WHO 2010 classification of NEC as the appropriate tissue type for this study, while only 13% responded that consideration of NEC+MANEC would be appropriate. Therefore, majority of facilities considered “WHO 2010 classification of NEC as the appropriate target of this study”.

While diagnosis of NECs is generally carried out using tissue samples and biopsy samples, as mentioned in 2.1.3, there is no consensus methodology to strictly distinguish NEC and MANEC using biopsy samples. As a result of group discussions, a consensus was reached between the three groups that cases presenting with NEC components by biopsy sample-based diagnosis can be enrolled. Although the use of this method would mean that a certain percentage of patients with MANEC, who were not intended to participate in this study, would be enrolled however, currently there are no appropriate means to avoid this. Therefore, we decided to create a consensus for the future based on information obtained from this study.

1）<u>Reason for targeting gastrointestinal/hepatobiliary or pancreatic primary lesion NEC</u>

Various guidelines recommend treatment regimens suited for small cell lung carcinoma, regardless of the organ of primary lesion, and is the rationale showing the validity of the treatment development for NEC across many organs. Furthermore, taking into consideration the frequency of the disease, it is unrealistic to develop treatments according to each organ, and in practice the Minnie-Pearl Cancer Research Network Study (mentioned later in 2.2.2), which is a relatively large-scale clinical study of NEC, development of treatment was carried out in a cross-organ

manner[27].

On the other hand, if we take hypothesize that we do not sufficiently understand how the clinical presentations of the disease vary according to the organ, the fact that this is the first randomized study in Japan, and that the maintenance of foundation for clinical studies of this disease in Japan is inadequate at present time, the hurdle is too high to plan a study across all organs including the fields of gynecology and urology. Based on the above consideration, we have decided to target all gastrointestinal organs which have a relatively large number of common points between each other in terms of clinical presentation and types of complication.

According to the multicenter observatory study by Yamaguchi and Machida, the prognosis of NEC with hepatobiliary or pancreatic primary lesion was significantly poorer than that of NEC with gastrointestinal primary lesion (MST: 7.9 vs. 13.0 months, respectively), but we determined that it is possible to accurately evaluate the efficacy of the treatment regimen by randomizing the subjects using the primary lesion site (gastrointestinal vs. hepatobiliary or pancreatic) as an allocation adjustment factor. Therefore, we considered patients with both, gastrointestinal/hepatobiliary or pancreatic primary lesions as one target population of this study, and to examine differences between organs exploratively by subgroup analysis.

2) Reason for including hepatic NEC cases (hepatic primary lesion or liver metastasis with primary lesion site unknown)

Even among NECs, cases with hepatic primary lesions are particularly rare. There are no specific reports on the frequency of incidence and differences from the other organs and reports are limited only to case reports and their reviews. Moreover, since the liver is a major organ for metastasis of primary lesions into other organs, even when tumors have been clinically identified in the liver, in many cases the primary lesion tends to be in another organ. Therefore, liver should not be deemed the primary lesion site without sufficient examination for a primary lesion. On the other hand, although very rare, there have been reports of NEC with hepatic primary lesion[28]. Even if sufficient search for primary lesion leads to no indication of the same outside of the liver, currently it is not possible to distinguish whether it is a case of “NEC with hepatic primary lesion” or “lesion in the liver is a metastatic lesion, with the primary lesion unknown”. In this study, such situations wherein “liver alone has identifiable lesions” would be henceforth, referred to as “hepatic NEC (hepatic primary lesion or liver metastasis from an unknown primary lesion site) for convenience. As the NCCN Guidelines[19] and ENETS Guidelines[20, 21] indicate the usefulness of FDG-PET for detailed examination of the primary lesion, we would also examine the primary lesion using FDG-PET. Furthermore, by referring to the diagnostic procedures for cancers with unknown primary lesion site, detailed examinations into the primary lesion would be carried out by otolaryngological (head and neck) examination and urological examination for men only, and gynecological examination for women only).

Such hepatic NEC (hepatic primary lesion or liver metastasis from an unknown primary lesion site) is anticipated even from an anatomical viewpoint, since the tumor-related complications are the same as that for other gastrointestinal primary lesion NECs, and there is no issue with treating them the same as for gastrointestinal primary lesions.

3) Reason for orienting the study for non-resectable or recurrent cases

As mentioned in 2.1.4 “Standard treatment according to disease staging and outline of prognosis”, systemic chemotherapy is indicated for cases with distal metastasis or recurrent cases. Locally advanced cases are particularly prone to pancreatic or bile duct primary lesions situated in the vicinity of vital vessels, and while the NCCN Guidelines propose chemoradiotherapy according to treatment for small cell lung carcinoma[19], the ENETS Guidelines provide no clear indication[21]. In reality, for the treatment of NEC which has a variety of primary lesion organs (scope of irradiation) there is not enough information available for the chemotherapy regimen that should be combined in terms of its optimum dose, the radiation dose, efficacy, and safety. For this reason, chemoradiotherapy cannot be considered the standard treatment with consensus, and systemic chemotherapy for distal metastasis is used widely except for pathologies involving frequent stenotic symptoms when the primary lesion is in the esophagus. For this reason, this study would enroll non-resectable or recurrent cases that include locally advanced cases.

The section “3.6 Definition of non-resectable NEC” shows the definition of non-resectable cases summarized by referring to the JCOG protocol for clinical studies of systemic chemotherapy (esophagus: JCOG0807, stomach: JCOG1013 or JCOG1002, bile duct: JCOG0805, pancreas: JCOG1106). Furthermore, while radical resection has

been considered for cases of colonic primary lesion adenocarcinoma with liver or lung metastasis, as cases of NEC with distal metastasis are not indicated for resection, we defined non-resectable cases as those in “Stage IV”, and followed this definition for duodenal primary lesions, small intestine primary lesions, and appendix primary lesions. Furthermore, with respect to NEC with esophageal primary lesion, chemoradiotherapy is well-indicated for cases with supraclavicular lymph node metastasis of thoracic esophageal tumor even in Stage IV, and hence were not included in this study.

4) Treatment of draft of new classification of NEC

In recent years, proposals have been made to further differentiate the WHO 2010 classification of NECs (G3 in ENETS) to “Grade 3 proliferative tumors showing the same morphological presentations as NET” and “Grade 3 tumors with strong morphological atypicality (previously classified as poorly differentiated NECs)”[29, 30], or by Ki-67 index of 20–50% and 50% or more[11, 19]. This is yet to be confirmed; however, in this study we would have used the WHO 2010 classification. However, we would have used samples collected for central pathological diagnosis to carry out studies related to classification which exploratively adds grade classification and morphological differentiation levels.

2.2. Standard treatment for target disease

Currently, there is no standard drug treatment for non-resectable/recurrent NECs with efficacy verified by a randomized controlled trial. However, given the similarity between pathological and clinical presentations, treatments according to small cell lung carcinoma have been attempted, and there have been reports of positive treatment outcome in small-scale clinical studies and observational studies.

2.2.1. Standard treatment for small cell lung carcinoma

Cisplatin-based multidrug combined therapy is the standard first-line treatment of extensive disease (ED) small cell lung carcinoma. While cyclophosphamide/doxorubicin/vincristine (CAV therapy) was established as a standard treatment in the 1970s in Europe and U.S., the etoposide/cisplatin combined therapy (EP therapy) was introduced in the late 1980s. A comparative study of EP therapy and CAV therapy did not show superiority of EP therapy over CAV therapy in terms of survival, but the response rate (CAV: 51% vs. EP: 61%) and MST (CAV: 8.3 months vs. EP: 8.6 months) were almost the same, and the lighter toxicity level (mucositis, interstitial pneumonia, hemotoxicity) meant that EP could be used as a standard treatment[31]. Thereafter, the JCOG Lung Cancer Group carried out the “comparison between EP therapy and Irinotecan/Cisplatin combined therapy (IP therapy) for Extensive-stage Small Cell Lung Cancer (JCOG9511)”, and reported that the IP therapy provided a significantly better overall survival, with MST being 9.4 months vs. 12.8 months ($p = 0.002$) for IP therapy[32]. However, the two additional large-scale studies conducted primarily in U.S. did not show superiority of IP therapy over EP therapy, and for this reason IP therapy has not been not used as the standard treatment overseas[33, 34]. Furthermore, while the results of the “Randomized controlled trial to verify the non-inferiority of amrubicin/cisplatin combined therapy (AP therapy) over IP therapy (JCOG0509)” were reported in the 2012 conference of the American Society of Clinical Oncology (ASCO), but AP therapy was still considered significantly inferior to IP therapy[35]. Presently, the JCOG Lung Cancer Group considers IP therapy to be the standard treatment for extensive-disease small cell lung carcinoma.

2.2.2. Standard treatment for extrapulmonary NEC

While there are scattered reports relating to use of CAV therapy, EP therapy, IP therapy, and other multidrug combined therapies for extrapulmonary NEC, these reports are primarily from observational studies. Since these reports precede the unification of concept and classification methods of this disease, the nomenclature such as NEC (anaplastic type), NET (poorly differentiated type) and extrapulmonary small cell lung carcinoma have still been used. These reports, however, are presumed to be targeting almost the same disease group as NEC. There are no reports of prospective studies using EP therapy for extrapulmonary NEC. Observational studies have reported response rates between 42–67%, and MST between 15–19 months (Table 2.2.2a). On the other hand, IP therapy has reported results from observational studies and clinical studies, with response rates between 7–83% and MST of 10.1–22.6 months (Table 2.2.2b). As mentioned above, reports related to extrapulmonary NEC are limited to use of EP therapy and IP therapy in observational studies and small-scale clinical studies. The largest number of case enrollments in a clinical study of extrapulmonary NEC was seen in the Minnie-Pearl Cancer Research Network Study, which evaluated the efficacy of triple-drug combined therapy using carboplatin/etoposide/paclitaxel. The

response rate to this triple-drug combined therapy was 53%, while the MST was 14.5 months and adequate, there was intense toxicity involved, and the results were not significantly different from EP therapy, so this regimen was not considered the standard treatment[27].

The 2014 NCCN Guidelines recommend treating extrapulmonary NEC using a regimen according to small cell lung carcinoma, and the guidelines for treatment of small cell lung carcinoma mention both EP therapy and IP therapy as recommended regimens.

Therefore, the standard treatment for extrapulmonary NEC is taken as EP therapy or IP therapy.

Table 2.2.2.a. EP therapy for NEC (observational studies)

Reported year/Reporter	Target	N	Response rate	MST (in months)
1991/ Moertel[18]	Pancreatic/gastrointestinal NEC (anaplastic type)	18	67%	19
1999/ Mitry [36]	Pancreatic/gastrointestinal NEC	41	42%	15
2001/ Marie-Louise [37]	Pancreatic NET (well: 11, poorly: 4) Gastrointestinal cartinoid: 21	33	18%	19
1994/ Lo Re G [38]	Extrapulmonary SCLC	13	69%	NE
2010/ Iwasa [39]	Hepatobiliary or pancreatic NEC	21	14%	7.3
2012/ Yamaguchi [10]	Gastrointestinal NEC	12	75%	14
2012/ Yamaguchi [10]	Hepatobiliary or pancreatic NEC	34	12%	6.9
2012/ Sorbye [11]	Gastrointestinal primary lesion NEC (including primary lesion unknown)	129	31%	12

Table 2.2.2.b. IP therapy for NEC (shaded are clinical studies, others are observational studies)

Reported year/Reporter	Target	N	Response rate	MST (in months)
2003/ Hou [40]	NEC (gastrointestinal-80%)	18	43%	NE
2005/ Chin [41]	Esophageal NEC	12	83%	14
2011/ Okita [42]	Gastric NEC	12	75%	22.6
2012/ Yamaguchi [10]	Gastrointestinal NEC	142	51%	13.4
2012/ Yamaguchi [10]	Hepatobiliary or pancreatic NEC	18	39%	10.1
2006/ Kulke [43]	Pancreatic/gastrointestinal (including NET G1/G2)	15	7%	11.4
2008/ Mani [44]	Pancreatic/gastrointestinal NEC	20	58%	NE
2008/ Jin [45]	Extrapulmonary NEC	15	67%	11.4.

Table 2.2.2.c. Phase II study of carboplatin/etoposide/paclitaxel triple-drug combined therapy for NEC

Reported year/Reporter	Target (breakdown)	N	Response rate	MST (in months)
2006/ Hainsworth [27]	NEC (Colon: 9, lung: 7, skin: 4, pancreas: 3, gall bladder: 1, thyroid:1, stomach: 1, esophagus: 1, endometrium:1, maxillary sinus: 1, prostate: 1, primary lesion unknown: 48)	78	53%	14.5

2.2.3. Standard treatment for gastrointestinal/hepatobiliary or pancreatic primary NEC

Treatments for extrapulmonary NEC have not been developed according to any specific organ, and the standard treatment for gastrointestinal/hepatobiliary or pancreatic NEC is also both, EP therapy and IP therapy. While both treatments are considered standard treatments, since this study considers them both to be study treatments, the anticipated effect and expected adverse reactions from EP therapy and IP therapy have been described in "2.3.2 Study treatment(s) of this study".

Herewith, we describe the current status of the usage of each treatment in Japan and overseas. According to the multicenter joint study by Yamaguchi and Machida, 160 out of 258 patients (62%) who received systemic chemotherapy underwent IP therapy, the most common treatment, followed by EP therapy (46 patients, 18%). Although there is big deviation where 92% of gastrointestinal primary lesion NEC (142/154 patients) were given IP therapy, and 65% of hepatobiliary or pancreatic primary lesion NEC (34/52 patients) were given EP therapy, the present situation shows that treatments are being selected by discretion or preference of the facility/physician.

Furthermore, even with respect to the dosing schedule, the method of administration has not necessarily as per the treatment of small cell lung carcinoma. There is no unified consensus, as some facilities use methods used for treatment of gastric cancer (JCOG9912 regimen), based on the reasoning that they have familiarity with IP therapy. On the other hand, in the NORDIC NEC study the most common treatment used was the EP therapy, which was administered to 129 out of 252 patients (51%) who received systemic chemotherapy, followed by a combined therapy consisting of carboplatin and etoposide therapy (67 patients, 27%). Combined therapy of platinum and etoposide was used often, which is another choice of treatment in Japan[11].

2.3. Rationale for establishment of treatment plan

2.3.1. Drugs

1) Etoposide

Etoposide exerts an antitumor effect by inhibiting topoisomerase II, which catalyzes the untangling of supercoiled DNA strands. Main toxicities include myelosuppression, nausea/vomiting, alopecia, and stomatitis.

2) Cisplatin

It is a complex ion form of the heavy metal platinum which shows anti-tumor effect by cross-linking double-stranded DNA. Currently, it is considered one of the key drugs for NEC treatment, being a central medicine for the treatment of lung cancer due to its synergistic effect with radiation therapy and various drugs, and also due to its low bone marrow toxicity when used alone. Toxicities include nausea/vomiting, nephrotoxicity, and neurotoxicity. Sufficient infusion of cisplatin before and after drug administration is necessary to prevent nephrotoxicity.

3) Irinotecan

Irinotecan is a topoisomerase I inhibitor developed in Japan that inhibits DNA synthesis. The drug is directly converted into the active metabolite (SN-38) in human liver and various tissues by carboxyesterase. As it shows a potent antitumor effect against SCLC even when used on its own, this drug is used very often in routine clinical practice to treat NEC as well. Diarrhea and myelosuppression are observed as dose-dependent toxicities. Nausea/vomiting and interstitial pneumonia have also been noted in patients.

2.3.2. Study treatment(s) of this study

1) Etoposide/cisplatin combined therapy (EP therapy)

In the multicenter joint study performed by Yamaguchi and Machida, the response rate of gastrointestinal primary lesion NEC to EP therapy was 75% (9/12 patients), with an MST of 14 months. The response rate of hepatobiliary and pancreatic primary lesion NEC to EP therapy was 12% (4/34 patients), and the MST was 6.9 months. With respect to safety, the multicenter joint study by Yamaguchi and Machida showed no treatment-related deaths associated with EP therapy (N = 46) administered as first-line treatment for gastrointestinal/hepatobiliary and pancreatic primary lesion NEC, and toxicity-related study discontinuation was observed in 6.5% of cases. Details of toxicities remain unknown as they were not investigated. The observational study of EP therapy for hepatobiliary and pancreatic primary lesion NEC (N = 21) carried out by Iwasa et al. at the National Cancer Center Hospital showed major Grade 3/4 adverse events to be neutropenia (90%), nausea (33%), and anorexia (24%). Grade 3 febrile neutropenia was observed in 8 patients (38%)[39]. Table 2.3.2 shows the toxicity profile of EP therapy from the JCOG9511 study which treated small cell lung carcinoma (Grade 3 or higher as per JCOG toxicity criteria).

Table 2.3.2. Toxicity of EP therapy and IP therapy in studies treating small cell lung carcinoma※
※Report by Iwasa et al., JCOG0509：CTCAE v3.0 Grade 3 or higher, JCOG9511：JCOG toxicity criteria Grade 3 or higher

	EP therapy		IP therapy	
	Report by Iwasa et al.[34]	JCOG9511[27]	JCOG9511[27]	JCOG0509[30]
Neutropenia	90%	92.2%	65.3%	58.5%
Leukopenia	71%	51.9%	26.7%	22.5%
Decreased hemoglobin	29%	29.9%	26.7%	23.2%
Thrombocytopenia	24%	18.2%	5.3%	2.1%
Diarrhea	0%	0%	16%	7.7%

Nausea	33%	6.5%	13.3%	6.3%
Aspartate aminotransferase increased	19%	2.6%	0%	-
Alanine aminotransferase increased	24%	3.9%	4%	-
Blood bilirubin increased	19%	0%	0%	-
Creatinine increased	0%	0%	0%	-
Peripheral motor neuropathy	0%	0%	0%	-
Febrile neutropenia	38%	0%	0%	1.4%

There are several reports concerning the specific dosage schedule/administered dose for EP therapy, and four dosing methods have been described in the NCCN Guidelines for SCLC as well. However, it is not clear as to which of the dosing methods is the best, and the dosing method for NEC varies between reports. In Japan, the dosing for EP therapy according to JCOG9511 is also often used for NEC. For this reason, this study would also follow the same method of administration. In terms of the number of cycles, a comparison of 4 courses vs. 8 courses of combined chemotherapy including cyclophosphamide for SCLC showed poor efficacy even when the treatment continued for long-term[46], and treatment of 4 cycles has also been specified in JCOG9511. There have been no such comparative studies for gastrointestinal/hepatobiliary and pancreatic primary lesion NEC, rather, there is no evidence to suggest that first-line chemotherapy should be discontinued if the chemotherapy is efficacious and toxicity is within the permitted scope. Unlike SCLC, NEC does not respond well to chemotherapy and tends to re-exacerbation to the underlying disease soon after the end of chemotherapy. For this reason, continuation of chemotherapy is very likely to be beneficial for patients with NEC as long as the treatment is effective.

While peripheral motor neuropathy, hearing impairment, and renal disorder are known accumulation toxicities of cisplatin, if sufficiently safe, it is ideal for treatments to continue as much as possible, considering the very few treatment options available for NEC. Based on the above rationale, we decided to ensure safety by establishing strict protocol treatment discontinuation criteria and chose not to limit the total dose or prescribe the number of treatment cycles.

2）Irinotecan/Cisplatin combined therapy (IP therapy)

In the multicenter joint study by Yamaguchi and Machida, the response rate of gastrointestinal primary lesion NEC to IP therapy was 51% (73/142 patients), with an MST of 13.4 months. The response rate of hepatobiliary and pancreatic primary lesion NEC to IP therapy was 39% (7/18 patients), and the MST was 10.1 months. There were no treatment-related deaths among 160 patients, and discontinuation due to toxicity in first-line chemotherapy was seen in 11.2% of cases. Details about toxicity were not collected. As a reference, Table 2.3.2 shows the toxicity profile of IP therapy from the JCOG9511 and JCOG0509 studies which treated small cell lung carcinoma.

There are several reports concerning specific dosing methods for IP therapy, and two dosing methods have been described in the NCCN Guidelines for SCLC as well. It is not known which of the dosing methods is the best, and the dosing method has also been variable in the NEC. In Japan, the dosing method of IP therapy, according to JCOG9511 is considered the standard treatment for SCLC and the same method has also been used in this study.

The total dose and number of cycles would have not been prescribed for the same reason as EP therapy, and safety has been ensured by establishing strict protocol treatment discontinuation criteria.

3）Other study treatment candidates

Presently, there are no drugs or treatment methods, including molecular-targeted drugs, which surpass the EP and IP therapy. There are also no planned or ongoing large-scale clinical trials globally, related to first-line treatment of NEC.

As the second-line treatment, a phase II study of Everolimus is underway for pancreatic primary lesion NEC resistant or unresponsive to platinum preparations. It is a multicenter study being carried out in 31 facilities in Japan, led by the National Cancer Center East Hospital, through the cancer research and development fund (UMIN000012752）.

2.3.3. Summary of risk/benefit balance of the standard treatment and study treatment

In the multicenter joint study by Yamaguchi and Machida, IP therapy had better prognosis compared to EP therapy (MST: 13.0 months vs. 7.3 months; p < 0.0001). However, as shown in Table 2.3.3, since IP therapy was more frequently chosen for gastrointestinal primary lesions (142/154 patients) and EP therapy for hepatobiliary and

pancreatic primary lesions (34/52 patients), it has not been possible to determine whether this difference is due to difference in efficacy between the regimens, or due to differences in the primary lesion organ. Primary lesion organ remained a significant prognostic factor after multivariate analysis, and while IP therapy turned out to be a slightly better regimen than EP therapy with HR = 0.8 (95% CI. 0.48–1.33), $p = 0.389$ indicated that there was no significant difference between the two (see “2.1.5 Prognostic/predictive factors”). Taking into account the number of EP therapy cases (46 patients), the fact that it was an observational study, and including other unknown bias, the data interpretation is fairly limited. Therefore, based on these results we are unable to deem either of IP or EP therapies to be more efficacious than the other.

With regard to toxicity, while myelosuppression such as neutropenia is milder in IP therapy than EP therapy, frequencies of events such as diarrhea and nausea are high. While the two treatments have different toxicity profiles, it is difficult to conclude that one has clearly worse toxicity than the other. The risk of bile duct obstruction is high in biliary and pancreatic primary lesion NECs and the use of irinotecan (which undergoes biliary excretion) in patients exhibiting biliary excretion disorder runs the risk of increased toxicity, hence care must be taken while administering irinotecan. We believe that this risk can be avoided by appropriate pre-treatment drainage and proper monitoring during treatment. Based on the above, it is difficult to assign superiority to IP therapy or EP therapy over the other in terms of risk/benefit balance, and both the regimens can be considered suitable standard treatments.

Table 2.3.3. Summary of results from multicenter study by Yamaguchi and Machida

		IP	EP	*p*-value*
Total	N	160	46	
	Response rate（%）	50	27	＜0.001
	Median PFS (in months)	5.2	4.0	0.033
	Median OS (in months)	13.0	7.3	＜0.0001
Gastrointestinal primary lesion NEC	N	142	12	
	Response rate（%）	51	75	0.14
	Median PFS (in months)	5.4	4.9	0.585
	Median OS (in months)	13.4	14.0	0.976
Hepatobiliary and pancreatic primary lesion NEC	N	18	34	
	Response rate（%）	39%	12%	0.034
	Median PFS (in months)	4.4	3.7	0.056
	Median OS (in months)	10.1	6.9	0.05

*Response rate by chi-squared test, PFS and OS by log-rank test

2.3.4. Post-treatment(s)

In the multicenter joint study by Yamaguchi and Machida, 56% (116/206 patients) of patients with NEC who received IP therapy or EP therapy were administered chemotherapy as the second-line of treatment. The most common second-line chemotherapy after IP therapy was amurcibin (22/88 patients), while irinotecan was the most common second-line treatment after EP therapy (13/28 patients), and we expected a similar pattern for the second-line treatments in this study as well. The overall performance of second-line chemotherapy was poor with a response rate of 11%, and PFS of 2.1 months. As such, the significance of the second-line treatment has not been indicated, and hence, no standard treatment has been established.

2.4. Study design

2.4.1. Rationale for establishing endpoints

For NECs, an alternative endpoint for overall survival, such as PFS has not been established as an indicator for comparing the usefulness of the treatment regimens. Therefore, comparison of overall survival was considered appropriate when examining the therapeutic effects. For the same, overall survival was examined as the primary endpoint. The secondary endpoints to evaluate the efficacy and safety were the response rate (only for patients with a measurable lesion), progression-free survival, incidence rate of adverse events, and dose intensity of cisplatin.

2.4.2. Clinical hypothesis and rationale for setting the number of enrollments

This study compares the two standard treatments, both of which are difficult to deem superior than the other in terms of efficacy and safety. We have, therefore, adopted a study design using bilateral testing. The main clinical hypothesis of this study is that either IP therapy or EP therapy is likely to provide better overall survival than the other treatment group, and should this hypothesis be validated, the superior treatment with statistical significance would be deemed the better treatment, and would therefore be positioned as the standard treatment in future. If the study fails to validate this hypothesis, it would indicate that there was no clinically significant difference in overall survival between the two groups, and if there are no large differences in terms of toxicity, we would conclude that both treatments are viable options as the standard treatment. However, if unlike our initial hypothesis, one treatment shows clearly more toxicity than the other, we would reconsider the study design before carrying out the main analysis.

Furthermore, as mentioned in “section 2.1.1 Epidemiology”, gastroenterological primary lesion NEC, which is the primary target of this study, is a very rare disease with an annual prevalence of around 3 in 100,000 people, hence, the significance level of testing has been placed at 10% bilaterally, instead of 5% bilaterally. In the multicenter joint study conducted by Yamaguchi and Machida, the MST of gastrointestinal/hepatobiliary or pancreatic primary lesion NEC was 11.5 months. To determine if one treatment is superior to the other, we expected a difference of four months in terms of MST. If we expect the more inferior treatment to have an MST of 8.0 months and the superior treatment to have an MST of 12.0 months, the number of enrollments are calculated using the aforementioned parameters and based on the discussion mentioned later (see “12.2 Expected number of enrollments/Enrollment period/Follow-up period”). This provided a research period of 7- years, consisting of 6-years of enrollment period and one year of follow-up period, and with $\alpha = 0.1$ bilaterally and detection power of 70% to detect differences between the two groups, 63 subjects were required per group. Taking into consideration some subjects who could be lost post follow-up, we aimed to enroll 70 subjects per group, and a total of 140 subjects between two groups.

If enrollment proceeds better than expected than prior to the start of the study (i.e. if the number of enrolled subjects reach 70 in less than 2.5 years from the start of enrollment), the number of enrollments is likely to be re-established by changing the detection power from 70% to 80% during the study, with the aim of obtaining more accurate results.

＜Addition to ver.1.1＞

The pace of enrollment after the start of enrollment period exceeded expectations, and the number of enrollments reached 70 subjects in October 2016, which was 2 years and 2 months after the start. As this exceeded the criteria of 70 subjects in less than 2.5 years, since the start as provided above, we obtained the approval of the Hepatobiliary and Pancreatic Oncology Group Meeting on October 29, 2016, Esophageal Cancer Group Meeting on November 19, 2016, and Gastric Cancer Group Meeting on January 7, 2017 to change the detection power to 80% according to provisions at the time of study planning. Consequently, the number of intended enrollments was changed to 170 subjects.

2.4.3. Expected patient enrollment

In the multicenter joint study by Yamaguchi and Machida, 258 cases of gastrointestinal/hepatobiliary or pancreatic primary lesion NECs were reported from 23 sites during the 11 years between 2000 and 2011, but between 2000 and 2006, information could not be obtained because of old cases. When limited to the most recent five years (2006–2011), when sufficient information was obtained from each facility, the number of enrollments was placed at162 subjects.

This is a joint study between three groups, namely the JCOG Hepatobiliary and Pancreatic Oncology Group,

JCOG Gastric Cancer Group, and the JCOG Esophageal Cancer Group. Therefore, the total number of facilities, excluding overlaps, amounted to 82 facilities. Compared to the multicenter joint study by Yamaguchi and Machida, we expect an increase in the number of patients enrolled, and simple calculations lead us to extrapolate 115 subjects to be enrolled annually. On the other hand, if we take into account the possibility of patient enrollments being biased to some high volume centers, it would be difficult to estimate the effect of increasing facility count to proceed. In addition, considering that the study is a randomized, we estimate the annual number of patients enrolled to be between 30–50 people. Taking into account ineligible cases, we provided 6-years for patient enrollment period.

2.4.4. Rationale for setting allocation adjustment factors

1) Facility

It is widely known that background, treatment, efficacy evaluation, and safety evaluation of enrolled patients vary depending on the facility, and JCOG standards have been used to make adjustments between facilities.

2) Primary lesion organ (gastrointestinal tract vs. hepatobiliary or pancreatic organ)

In the multicenter joint study by Yamaguchi and Machida, multivariate analysis using the Cox proportional hazard model was carried out with 183 subjects, which indicated primary lesion organ (gastrointestinal tract vs. hepatobiliary or pancreatic organ) to be a significant prognostic factor.

2.4.5. Centralized pathological diagnosis

In this study, a centralized pathological diagnosis would be performed for analysis of NEC, despite patients being diagnosed by a third party. The operation of the centralized pathological diagnosis is described in section 15.1, while details of the operation have been described in the Centralized Pathological Diagnosis Procedure Manual. Furthermore, an analysis of endpoints based on the results of centralized pathological diagnosis would be provided as a reference.

2.5. Summary of expected advantages and disadvantages associated with study participation

2.5.1. Expected advantages

Drugs used for both groups of this study are treatments used in routine medical practice. As mentioned in the next section, although it would be necessary to use some drugs for which insurance coverage does not apply, since insurance claims for these treatments are made in a similar manner to general medical care however, in practice they do not receive insurance assessments.

Furthermore, cases of off-label medication have been studied according to "18th Case Providing Review Information" at the Case Review Committee established by the Health Insurance Claims Review & Reimbursement Services. Based on the review information provided as on February 26, 2018;, The use of "irinotecan hydrochloride hydrate, etoposide, cisplatin, and carboplatin [injections]" is approved for treatment of "neuroendocrine carcinoma"], and the notices issued by the Ministry of Health, Labor and Welfare on February 26, 2016the results of this study have been considered valid by the ministry. For the same reason, essentially since February 26, 2018, there have been no concerns about claims of insurance assessments.

Moreover, since the medical fees of study participants during the study, which includes drug fees, are paid in principle by the patients themselves and their insurance coverage, so the patients would receive no special medical or financial benefits from participation in this study.

2.5.2. Expected risks and disadvantages

Both treatment arms A and B would receive chemotherapy regimen used in routine medical practice, so they would be unlikely to be exposed to special risks or disadvantages not observed in routine medical practice. The descriptions in "2.3.3 Risk/benefit balance of standard treatment and study treatments" outline the expected risks and disadvantages for each treatment arm.

To minimize the risk of adverse events and disadvantages, the " Patient selection criteria (Section 4)", " Criteria to change treatment (Section 6.3)" and "Concomitant/Supportive therapies (Section 6.4)" have been carefully considered for the three groups. Furthermore, the Institutional Review Board would monitor if the adverse events are within the scope of expectation, while any serious adverse events or unexpected adverse events would be carefully examined and reviewed according to the provisions related to "JCTN-Adverse Events Report Guideline"

and “JCOG Guidelines for Handling Clinical Safety Information”, and a system has been provided to take any necessary countermeasures.

Precautions related to etoposide, irinotecan, and cisplatin

As of December 2013, etoposide therapy, which is the intended to be used in this study had not received insurance approval for the treatment of gastrointestinal or hepatobiliary and pancreatic primary lesion cancers. Irinotecan has also not received insurance approval for treatment of cancers with primary lesions in organs other than the stomach, colon, and rectum. Furthermore, as cisplatin is only indicated for esophageal, gastric cancer, and for biliary tract cancers when combined with gemcitabine (25 mg/m^2), the dosage and administration used in this study have not been approved previously.

As each facility in this study may carry out the insurance claims in the same manner as routine general medical practice, the treatment may receive insurance assessment after-the-fact. However, if a facility incurs losses, the loss must be borne by the relevant facility (medical institution), as there is no system of compensation arranged by the JCOG Research Organization. If actual losses occur, the continued participation in the study would then be carefully discussed between the facility supervisor and the principal investigator/clinical trial secretariat. The facility supervisor would be expected to gain approval from the facility IRB and the head of the medical institutions.

2.6. Significance of this study

While both, EP therapy and IP therapy are standard treatments, the efficiency of either treatment being better than the other is not yet clear, and currently in routine medical practice, the choice of treatment is being made on the discretion or preference of the facility/attending physician.

If this study is able to clearly indicate the efficiency of EP therapy or IP therapy over the other, we expect that the diminished use of the relatively inferior treatment would contribute to improvement in patient prognosis. If the study indicates no clinically meaningful differences between the two treatments, the results would provide evidence that there is no significant issue with the treatments being chosen by discretion or preference of the facility/attending physician, which has been done traditionally without any data that directly compare the two treatments, although that would not indicate that the effects of the two treatments are equivalent. We also expect that the establishment of a highly reliable standard treatment in this study which would provide a foundation for therapeutic development when promising treatment regimen in the future.

As NEC is a rare disease, and since each clinician is not likely to have adequate experience, we believe that work associated with multicenter studies such as confirmation of diagnosis by centralized diagnosis/results feedback and sharing results of treatments by unified treatment regimen would lead to improvement of quality of care for NEC in Japan.

2.7. Associated research (including sample analysis research)

No such studies have been planned or carried out at the time of preparation of the protocol.

2.8. JCOG-Biobank Japan (BBJ) collaborating biobank

This study would participate in the banking of blood samples (DNA/plasma) through the JCOG-BBJ collaboration biobank based on the protocol common to all JCOG studies (hereafter termed as “common banking”).

Common banking by JCOG-BBJ collaborating biobank would collect and store the samples of patients enrolled in clinical studies conducted by JCOG regardless of presence/absence of pre-planned sample analysis research, would also provide samples for future analysis research and diagnostic information obtained through the main research.

The target of such biobanking are those patients who provided consent to participate in this study and gave consent to provide samples to the JCOG-BBJ collaborating biobank for their use in future sample analysis research (hereafter termed as consent for banking).

Samples collected during common banking include whole blood and preserved pathological tissue samples from routine clinical practice. Plasma and DNA separated/isolated from blood would be stored in the JCOG-BBJ collaborating biobank and would be provided for sample analysis research in the future. Preserved pathological tissue samples from routine medical practices such as surgery, and biopsy/clinical laboratory tests would also likely to be used for future sample analysis research. Although the type, sample preparation methods, and tissue quantity required would vary from study to study, there is no definite consensus that banking pathological tissue in a certain

method is more efficient than others. Furthermore, it has been suggested that long-term preservation of a sectioned specimen from preserved pathological tissue may lead to sample deterioration (DNA fragmentation). The discussions between the of JCOG and BBJ personnel about these problems, led to the conclusion that preserved pathological tissue taken after medical examination shall require only patients' consent for future use, and actual collection must be started after standardizing a separate protocol and specifying in it the procedures best suited for the details of the research.

The detailed procedures for sample collection, storage, and method of sample provision for future sample analysis research in common banking have been stipulated in the "JCOG-BioBank Japan Collaborating Biobank Protocols" that apply to all the JCOG studies. To participate in the common banking carried out by JCOG-BBJ collaborating biobanks, the subject matter must be reviewed and approved by the ethics committees of each participating facility.

Furthermore, to conduct sample analysis research in future using samples stored in the JCOG-BBJ collaborating biobank, it would be necessary to prepare a "Sample Analysis Research Protocol" and have it reviewed and approved by the JCOG Protocol Review Committee and the ethics committee of facilities involved in the sample analysis.

3. Criteria/definitions used in this study

Tissue classification would be performed according to WHO 2010 classification and ENETS (European Neuroendocrine Tumor Society) classification[14, 15], while disease staging would be done according to "UICC-TNM 7th Edition".

3.1. Tissue classification (WHO 2010 classification)

The shaded parts are the targets of this study

Neuroendocrine neoplasms

1) Neuroendocrine tumor: NET Grade 1 (NETG1)
2) Neuroendocrine tumor: NET Grade 2 (NETG2)
3) Neuroendocrine carcinoma: NEC (large cell or small cell type)
4) Mixed adenoendocrine carcinoma (MANEC)
5) Hyperplastic and preneoplastic lesions

3.2. Grade classification (ENETS [European Neuroendocrine Tumor Society] / WHO2010 classification)

Grade 1 (G1) Number of mitotic presentations < 2 per 10 high power fields (HPF) and/or Ki-67 index ≤2%*

Grade 2 (G2) Number of mitotic presentations 2–20 per 10 HPF and/or Ki-67 index 3-20%

Grade 3 (G3) Number of mitotic presentations > 20 per 10 HPF and/or Ki-67 index >20%

*The description of number of mitotic presentations in WHO 2010 classification is ≤2, but 2–3% are classified to G1[47].

3.3. Histopathological diagnosis

- Immunostaining (Chromogranin A and synaptophysin) is essential for a pathological diagnosis of NEC.
- Either1. or 2., or both are studied to determine proliferative activity. Number of mitotic presentations and Ki-67 index would adopt a high grade evaluation.
  1. Number of mitotic presentations (to evaluate 50HPF)
  2. Ki-67 index (500–2000 tumor cells*)

  *(Only in this study, if the number of tumor cells in the collected sample is less than 500, the number of tumor cells measured is also listed. A minimum of 100 tumor cells is required.)
- If sufficient amount of biopsy sample cannot be collected for a pathological diagnosis, a cell block prepared using material obtained from EUS-FNA, brushing or needle biopsy may be used. However, the use of a cell block prepared from ascites or pleural effusion is not permitted.
- During a histological diagnosis using a resected tumor sample, a slide with a representative section shall be used to evaluate the proportion of NEC components (differential diagnosis with mixed adenoendocrine carcinoma). For a comprehensive examination, the entire tumor must be evaluated.

For further details NEC Pathological Diagnosis Handbook would be referred (posted on JCOG website).

3.4. Disease stage classification criteria (UICC-TNM 7th Edition)

Special notice regarding disease stage classification: Although there is an independent TNM classification for NET G1 and NET G2 with primary lesions in the stomach, small intestines, and colon; since this study concerns NEC, the disease would be classified according to the classification method of major tissue type in each primary lesion organ (squamous cell carcinoma for esophagus, and adenocarcinoma for all other primary lesion organs). In this study, the classification of intrahepatic cholangiocarcinoma is used for hepatic NEC, due to their similar clinical presentation.

3.4.1. Esophagus

T –Primary tumor

TX: Primary tumor cannot be evaluated

T0: Primary tumor unidentified

Tis: Epithelial carcinoma/highly dysplastic

T1: Tumor invading mucosal lamina propria, muscularis mucosae, or submucosa

T1a: Tumor invading the mucosal lamina propria or muscularis mucosae

T1b: Tumor invading the submucosa

T2 : Tumor invading the muscularis propia

T3 : Tumor invading the adventitia

T4 : Tumor invading the surrounding tissue

T4a : Tumor invading the pleura, pericardium, and diaphragm

T4b : Tumor invading other surrounding tissues such as the aorta, centrum, trachea

N –Regional lymph nodes

NX : Regional lymph node metastasis cannot be evaluated

N0 : No regional lymph node metastasis

N1 : 1–2 foci of regional lymph node metastases

N2 : 3–6 regional foci of lymph node metastases

N3 : 7 or more foci of regional lymph node metastases

M –Distal metastasis

M0 : No distal metastasis

M1 : Distal metastasis present

Stage	T	N	M
**0**	Tis	N0	M0
**IA**	T1	N0	M0
**IB**	T2	N0	M0
**IIA**	T3	N0	M0
**IIB**	T1, T2	N1	M0
**IIIA**	T4a	N0	M0
	T3	N1	M0
	T1, T2	N2	M0
**IIIB**	T3	N2	M0
**IIIC**	T4a	N1、N2	M0
	T4b	Unrelated to N	M0
	Any T	N3	M0
**IV**	Any T	Any N	M1

3.4.2. Stomach

T –Primary tumor

TX : Primary tumor cannot be evaluated

T0 : Primary tumor unidentified

Tis : Epithelial carcinoma: Epithelial carcinoma/highly dysplastic carcinoma not invading the lamina propia mucosa

T1 : Tumor invading mucosal lamina propria, muscularis mucosae, or submucosa

T1a : Tumor invading the mucosal lamina propria or muscularis mucosae

T1b : Tumor invading the submucosa

T2 : Tumor invading the muscularis propia

T3 : Tumor invading the subserosa

T4 : Tumor perforating the serosa, or invading adjacent structures[1,2,3]

T4a : Tumor perforating the serosa

T4b : Tumor invading adjacent structures[1,2,3]

1. Adjacent organs of the stomach are spleen, transverse colon, liver, diaphragm, pancreas, abdominal wall, adrenal glands, kidneys, small intestine, and retroperitoneum.
2. If the invasion has spread from the stomach to the duodenum or esophagus, classification is made in terms of depth.
3. A tumor that advances into the gastrocolic ligament, into the hepatogastric ligament, or into the greater or lesser omentum, and is classified as T3, when there is no perforation of the visceral peritoneum.

N –Regional lymph nodes

NX : Regional lymph node metastasis cannot be evaluated

N0 : No regional lymph node metastasis

N1 : 1–2 regional foci of lymph node metastases

N2 : 3–6 regional foci of lymph node metastases

N3 : 7 or more regional foci of lymph node metastases

N3a : 7–15 regional foci of lymph node metastases

N3b : 16 or more regional foci of lymph node metastases

M –Distal metastasis

M0 : No distal metastasis

M1 : Distal metastasis present

Stage	T	N	M
**0**	Tis	N0	M0
**IA**	T1	N0	M0
**IB**	T2	N0	M0
	T1	N1	M0
**IIA**	T3	N0	M0
	T2	N1	M0
	T1	N2	M0
**IIB**	T4a	N0	M0
	T3	N1	M0
	T2	N2	M0
	T1	N3	M0
**IIIA**	T4a	N1	M0
	T3	N2	M0
	T2	N3	M0
**IIIB**	T4b	N0, N1	M0
	T4a	N2	M0
	T3	N3	M0
**IIIC**	T4a	N3	M0
	T4b	N2, N3	M0
**IV**	Any T	Any N	M1

3.4.3. Small intestines (including duodenum)

T –Primary tumor

TX : Primary tumor cannot be evaluated

T0 : Primary tumor unidentified

Tis : Epithelial carcinoma

T1 : Tumor invading mucosal lamina propria, muscularis mucosae, or submucosa

T1a : Tumor infiltrating the mucosal lamina propria or muscularis mucosae

T1b : Tumor infiltrating the submucosa

T2 : Tumor infiltrating the muscularis propia

T3 : Tumor invading the subserosa, or tumor invading within 2 cm of surrounding tissue of muscularis externa without peritoneal cover (mesenterium, retroperitoneum)*

*Surrounding tissue of muscularis externa without peritoneal cover refers to the mesenterium in the jejunum and ileum, and the retroperitoneum in the duodenum without serosa.

T4 : Tumor penetrating the visceral peritoneum, or tumor directly invading another organ or tissue (invasion of other loops of the small intestine, invasion by 2 cm or more into the mesenterium and retroperitoneum, including invasion into the abdominal wall through the serosa; invasion to the pancreas only for duodenum)

N –Regional lymph nodes

NX : Regional lymph node metastasis cannot be evaluated

N0 : No regional lymph node metastasis

N1 : 1–3 regional foci of lymph node metastases

N2 : 4 or more regional foci of lymph node metastases

M –Distal metastasis

M0 : No distal metastasis

M1 : Distal metastasis present

Stage	T	N	M
**0**	Tis	N0	M0
**I**	T1, T2	N0	M0
**IIA**	T3	N0	M0
**IIB**	T4	N0	M0
**IIIA**	Any T	N1	M0
**IIIB**	Any T	N2	M0
**IV**	Any T	Any N	M1

3.4.4. Appendix (partial revision of UICC-TNM 7th Edition)

T –Primary tumor

TX : Primary tumor cannot be evaluated

T0 : Primary tumor unidentified

Tis : Epithelial carcinoma: Tumor invading epithelium or lamina propia mucosae

T1 : Tumor invading the submucosa

T2 : Tumor invading the muscularis propia

T3 : Tumor invading the subserosa or mesoappendix

T4 : Tumor penetrating the visceral peritoneum, tumor including a peritoneal mucosal tumor in the lower right abdomen and/or tumor directly invading other organs or tissue

T4a : Tumor penetrating the visceral peritoneum, or peritoneal mucosal tumor in the lower right abdomen

T4b : Tumor directly invading other organs or tissues

N –Regional lymph nodes

NX : Regional lymph node metastasis cannot be evaluated

N0 : No regional lymph node metastasis

N1 : 1–3 regional foci of lymph node metastases

N2 : 4 or more foci of regional lymph node metastases

M –Distal metastasis

M0 : No distal metastasis

M1 : Distal metastasis present

M1a : Peritoneal metastasis beyond the lower right abdomen, including pseudomyxoma peritonei

M1b : Distal metastasis other than peritoneal metastasis

Stage	T	N	M
**0**	Tis	N0	M0
**I**	T1, T2	N0	M0
**IIA**	T3	N0	M0
**IIB**	T4a	N0	M0
**IIC**	T4b	N0	M0
**IIIA**	T1, T2	N1	M0
**IIIB**	T3, T4	N1	M0
**IIIC**	Any T	N2	M0
**IVA**	Any T	N0	M1a
**IVB**	Any T	N0	M1a
	Any T	N1, N2	M1a
**IVC**	Any T	Any N	M1b

3.4.5. Colon and rectum

T –Primary tumor

TX: Primary tumor cannot be evaluated

T0: Primary tumor unidentified

Tis: Carcinoma in situ: Tumor invading epithelium or lamina propia mucosae

T1: Tumor invading the submucosa

T2: Tumor invading the muscularis propia

T3: Tumor invading the subserosa, or tissue surrounding the colon or rectum without peritoneal covering

T4: Tumor penetrating the visceral peritoneum, and/or directly invading another organ or tissues

T4a: Tumor penetrating the visceral peritoneum

T4b: Tumor directly invading another organ or tissues

N –Regional lymph nodes

NX: Regional lymph node metastasis cannot be evaluate

N0: No regional lymph node metastasis

N1: 1–3 regional foci of lymph node metastases

N1a: 1 regional lymph node metastasis

N1b: 2–3 regional foci of lymph node metastases

N1c: Presence of tumor deposits, that is, satellite nodes in the subserosa or in the soft tissue surrounding colon or rectum without peritoneal covering, but with no regional lymph node metastasis

N2: 4 or more regional lymph node metastases

N2a: 4–6 regional foci of lymph node metastases

N2b: 7 or more regional foci of lymph node metastases

M –Distal metastasis

M0: No distal metastasis

M1: Distal metastasis present

M1a: Local metastasis to one organ (liver, lungs, ovaries, or lymph nodes other than regional lymph nodes)

M1b: 2 or more organs, or peritoneal metastasis

Stage	T	N	M
**0**	Tis	N0	M0
**I**	T1, T2	N0	M0
**II**	T3, T4	N0	M0
**IIA**	T3	N0	M0
**IIB**	T4a	N0	M0
**IIC**	T4b	N0	M0
**III**	Any T	N1, N2	M0
**IIIA**	T1, T2	N1	M0
	T1	N2a	M0
**IIIB**	T3, T4	N1	M0
	T2, T3	N2a	M0
	T1, T2	N2b	M0
**IIIC**	T4a	N2a	M0
	T3, T4a	N2b	M0
	T4b	N1, N2	M0
**IVA**	Any T	Any N	M1a
**IVB**	Any T	Any N	M1b

3.4.6. Applies to hepatic NEC (hepatic primary lesion or liver metastasis from unknown primary lesion)

T –Primary tumor

TX: Primary tumor cannot be evaluated

T0: Primary tumor unidentified

Tis： Carcinoma *in situ*

T1： Isolated tumor without vascular invasion

T2a： Isolated tumor with vascular invasion

T2b： Multifocal tumor regardless of vascular invasion

T3： Tumor penetrating visceral peritoneum or directly invading adjacent extrahepatic structures

T4： Tumor with bile duct invasion (bile duct proliferative type)

N –Regional lymph nodes

NX： Regional lymph node metastasis cannot be evaluated

N0： No regional lymph node metastasis

N1： Regional lymph node metastasis present

M –Distal metastasis

M0： No distal metastasis

M1： Distal metastasis present

Stage	T	N	M
**I**	T1	N0	M0
**II**	T2	N0	M0
**III**	T3	N0	M0
**IVA**	T4	N0	M0
	Any T	N1	M0
**IVB**	Any T	Any N	M1

3.4.7. Gall bladder

T –Primary tumor

TX： Primary tumor cannot be evaluated

T0： Primary tumor unidentified

Tis： Carcinoma *in situ*

T1： Tumor invading mucosal lamina propria or muscularis externa

T1a： Tumor invading mucosal lamina propria

T1b： Tumor invading muscularis externa

T2： Tumor invading the connective tissue around the muscularis externa, but shows no progression beyond the serosa or to the liver

T3： Tumor perforating the serosa (visceral peritoneum), tumor directly advancing to the liver and/or an adjacent organ that is not the liver (stomach, duodenum, colon, pancreas, omentum, or extrahepatic bile duct)

T4： Tumor invading the main trunk of the portal vein or the hepatic artery, or tumor advancing to two or more adjacent organs which are not the liver

N –Regional lymph nodes

NX： Regional lymph node metastasis cannot be evaluated

N0： No regional lymph node metastasis

N1： Regional lymph node metastasis present (cystic duct, common bile duct, proper hepatic artery, including lymph nodes along the portal vein)

M –Distal metastasis

M0： No distal metastasis

M1： Distal metastasis present

Stage	T	N	M
**0**	Tis	N0	M0
**I**	T1	N0	M0
**II**	T2	N0	M0
**IIIA**	T3	N0	M0
**IIIB**	T1, T2, T3	N1	M0
**IVA**	T4	Any N	M0

**IVB**	Any T	Any N	M1

3.4.8. Extrahepatic bile duct-hepatic portal region

T –Primary tumor

TX: Primary tumor cannot be evaluated

T0: Primary tumor unidentified

Tis: Carcinoma *in situ*

T1: Tumor localized to the bile duct which advances until muscularis externa or fibrous tissue

T2a: Tumor invading beyond the bile duct wall and into the surrounding adipose tissue

T2b: Tumor invading the adjacent liver parenchyma

T3: Tumor invading the branch of one side of portal vein or hepatic artery

T4: Tumor invades the main trunk of portal vein, and branches on both sides of the portal vein, proper hepatic artery or the secondary branches of the bile duct on both left and right side, or to the secondary bile duct branches on one side and portal vein or hepatic artery on the other side

N –Regional lymph nodes

NX: Regional lymph node metastasis cannot be evaluated

N0: No regional lymph node metastasis

N1: Presence of regional lymph node metastasis in the cystic duct, common bile duct, proper hepatic artery, and lymph nodes along portal vein

M –Distal metastasis

M0: No distal metastasis

M1: Distal metastasis present

Stage	T	N	M
**0**	Tis	N0	M0
**I**	T1	N0	M0
**II**	T2a, T2b	N0	M0
**IIIA**	T3	N0	M0
**IIIB**	T1,T2, T3	N1	M0
**IVA**	T4	N0, N1	M0
**IVB**	Any T	Any N	M1

3.4.9. Extrahepatic bile duct-distal

T –Primary tumor

TX: Primary tumor cannot be evaluated

T0: Primary tumor unidentified

Tis: Carcinoma *in situ*

T1: Tumor localized in the bile duct wall

T2: Tumor invading beyond the bile duct wall

T3: Tumor invading the gallbladder, liver, pancreas, duodenum, or other adjacent organs

T4: Tumor invading the celiac axis or superior mesenteric artery

N –Regional lymph nodes

NX: Regional lymph node metastasis cannot be evaluated

N0: No regional lymph node metastasis

N1: Regional lymph node metastasis present

M –Distal metastasis

M0: No distal metastasis

M1: Distal metastasis present

Stage	T	N	M
**0**	Tis	N0	M0
**IA**	T1	N0	M0
**IB**	T2	N0	M0

**IIA**	T3	N0	M0
**IIB**	T1, T2, T3	N1	M0
**III**	T4	Any N	M0
**IV**	Any T	Any N	M1

3.4.10. Ampulla of Vater

T –Primary tumor

TX: Primary tumor cannot be evaluated

T0: Primary tumor unidentified

Tis: Carcinoma *in situ*

T1: Tumor localized to the ampulla of Vater, or sphincter of Oddi

T2: Tumor invading the duodenal wall

T3: Tumor invading the pancreas

T4: Tumor invading the soft tissue around the pancreas or other adjacent organs

N –Regional lymph nodes

NX: Regional lymph node metastasis cannot be evaluated

N0: No regional lymph node metastasis

N1: Regional lymph node metastasis present

M –Distal metastasis

M0: No distal metastasis

M1: Distal metastasis present

Stage	T	N	M
**0**	Tis	N0	M0
**IA**	T1	N0	M0
**IB**	T2	N0	M0
**IIA**	T3	N0	M0
**IIB**	T1, T2, T3	N1	M0
**III**	T4	Any N	M0
**IV**	Any T	Any N	M1

3.4.11. Pancreas

T –Primary tumor

TX: Primary tumor cannot be evaluated

T0: Primary tumor unidentified

Tis: Carcinoma *in situ*

T1: Tumor localized within the pancreas, with a maximum size of < 2 cm

T2: Tumor localized within the pancreas, with a maximum size > cm

T3: Tumor advancing outside the pancreas, but with no invasion to the celiac axis or superior mesenteric artery

T4: Tumor invading the celiac axis or superior mesenteric artery

N –Regional lymph nodes

NX: Regional lymph node metastasis cannot be evaluated

N0: No regional lymph node metastasis

N1: Regional lymph node metastasis present

M –Distal metastasis

M0: No distal metastasis

M1: Distal metastasis present

Stage	T	N	M
**0**	Tis	N0	M0
**IA**	T1	N0	M0
**IB**	T2	N0	M0
**IIA**	T3	N0	M0

**IIB**	T1, T2, T3	N1	M0
**III**	T4	Any N	M0
**IV**	Any T	Any N	M1

3.5. Residual tumor (R) classification (UICC-TNM 7th Edition)

RX : Presence of residual tumor cannot be evaluated

R0 : No residual tumor

R1 : Presence of residual tumor by microscopic examination

R2 : Presence of residual tumor by macroscopic examination

3.6. Definition of non-resectable NEC

Unoperated cases for which surgery as a curative process was not indicated based on clinical findings, including image-based diagnosis, or operated cases that underwent resection (including experimental laparotomy) but ultimately ended as R2 resection are considered non-resectable.

The details by organs are shown below. One or more criterion must be fulfilled for each organ.

<Esopghagus>

- Diagnose as Stage IV (UICC 7th edition) based on clinical findings.

<Stomach>

- Diagnose as Stage IV (UICC 7th edition) based on clinical or surgical findings. However, this does not include situations where Stage IV diagnosis is provided based only on peritoneal lavage cytology (CY1).
- Bulky lymph node* metastasis is identified.

  *Bulky lymph node: Two or more lymph nodes with a major axis of 1.5 cm or more, that are present in contact with each other around the celiac artery, the common hepatic artery, the splenic artery, and the proper hepatic artery, or on the front surface of the superior mesenteric vein, where single or multiple large and small lymph nodes have formed an aggregate with the overall major axis length of 3.0 cm or more.

<Duodenum (excluding the ampulla of Vater), small intestines, appendix, and colon>

- Diagnosed as Stage IV (UICC 7th edition) based on the clinical findings.

<Extrahepatic bile duct, ampulla of Vater, gallbladder>

- Distant metastasis is identified
- Para-aortic lymph node metastasis is identified
- Bulky metastasis identified in the hepatoduodenal mesentery and lymph nodes surrounding the head of the pancreas
- Invasion in the proper hepatic artery, common hepatic artery, celiac artery, or the superior mesenteric artery identified
- Invasion in the hepatic artery branches on both the left and right side identified
- Extensive invasion or occlusion of the main trunk of portal vein, or invasion in the portal vein branches on both the left and right side identified
- Invasion in the blood vessels on the lobe on one side of the liver (portal vein or artery) or atrophy of lobe on one side of liver identified, and advancement into the bile duct on the other side is identified up to the secondary branch level
- Advancement into the bile ducts on both sides identified up to the secondary branch level

<Pancreas>

- Diagnosed as Stage III or Stage IV (UICC 7th edition) based on clinical findings.

＜Hepatic NEC (hepatic primary lesion or liver metastasis from unknown primary lesion)>

- Distal metastasis* other than intrahepatic metastasis identified.

  *Limited to organs that cannot be the primary lesion site (bone, lymph nodes below the diaphragm,

peritoneum, subcutaneous, muscle, and spleen) (see 3.8)

- Para-aortic lymph node metastasis is identified
- Bulky metastasis identified in the hepatoduodenal mesentery and lymph nodes surrounding the head of the pancreas
- Multiple masses identified in the liver.
- Invasion in one of the proper hepatic artery, common hepatic artery, celiac artery, or the superior mesenteric artery identified.
- Invasion to hepatic artery branches on both the left and right side identified
- Extensive invasion or occlusion of the main trunk of portal vein, or invasion in portal vein branches on both the left and right side identified
- Invasion in blood vessels on lobe on one side of the liver (portal vein or artery) or atrophy of lobe on one side of liver identified, and advancement into the bile duct on the other side is identified up to the secondary branch level
- Advancement into the bile ducts on both sides identified up to the secondary branch level

3.7. Definition of recurrent NEC

If surgery (R0 resection or R1 resection) was performed for gastrointestinal/hepatobiliary or pancreatic primary lesion, malignant tumor and the condition was diagnosed as NEC based on pathological samples taken from surgery, and were deemed as a clinical relapse thereafter.

If there are past histological samples from the primary lesion, no histological biopsy of the relapse lesion is necessary.

3.8. Definition of hepatic NEC (hepatic primary lesion or liver metastasis from an unknown primary lesion)

After a detailed investigation into the primary lesion by cervical-pelvic contrast CT, gastrointestinal endoscopy, FDG-PET, otolaryngology (head and neck examination), urology examination (for men only) and gynecological examination (for women only), if the tumor is in one of the following sites, the case is defined as hepatic primary lesion (or primary lesion unknown).

- Tumor is present only in the liver
- Tumor is present in an organ that cannot be a primary lesion site (bone, lymph nodes below the diaphragm, peritoneum, subcutaneous, muscle, and spleen), and in the liver.

4. Patient selection criteria

Patients that fulfill all of the following inclusion criteria and do not correspond to any of the exclusion criteria are eligible for enrollment.

4.1. Inclusion criteria (for enrollment)

1) Any of the following is applicable based on pathological diagnosis taking findings of immunohistochemistry into consideration (see 3.1. to 3.3.).

[1] Pathologically diagnosed as neuroendocrine carcinoma (NEC*1) in the resected sample.

[2] Containing pathologically confirmed component of neuroendocrine carcinoma (NEC*1) in the biopsy sample.

1: Based on WHO 2010 classification

2) Any of the following is applicable

[1] NEC arise in esophagus, stomach, duodenum, intestine, appendix, colon, rectum, gallbladder, intrahepatic bile duct, extrahepatic bile duct, ampulla of Vater, pancreas,

[2] Liver NEC (primary liver or liver metastasis of unknown primary) *2.

*2: The tumor is only in one of the following sites after a thorough examination of the primary site by contrast CT (from the neck to pelvic) and upper/lower gastrointestinal endoscopy, FDG-PET scan, otolaryngology (head and neck) examination, urology examination (male patients only), and gynecology examination (female patients only).

a. Liver only

b. Bone, lymph nodes below the diaphragm, peritoneum, subcutaneous, muscle, spleen, and liver

3) Unresectable (see 3.6) or recurrent cancer (see 3.7). It is not essential for a pathological confirmation of the metastatic lesion or recurrent site. Cases of esophageal NEC is ineligible if corresponding to any of the following.

[1] cT4.

[2] No distant metastasis rather than supraclavicular lymph node

[3] Stenosis indicated for palliative radiotherapy

4) No previous chemotherapy or radiotherapy for NEC. Pre- or post-operative chemotherapy except irinotecan or etoposide for NEC is allowed as long as it was completed at least 8 weeks prior to registration.

5) No previous chemotherapy using platinum agents for any malignancies.

6) No serious tumor-related complications.

Serious tumor-related complications include superior vena cava syndrome, inferior vena cava syndrome, pleural, ascites, or pericardial effusions that are large or uncontrollable (e.g., effusions that accumulate quickly after drainage or even after adhesive surgery), and brain metastases with neurological symptoms.

7) Aged 20 to 75 years old.

8) ECOG performance status of 0 or 1.

9) Sufficient oral intake

10) Measurable region is not required.

11) Adequate organ functions.

[1] WBC ≥3,000/ mm^3

[2] Neutrophils ≥ 1,500/ mm^3

[3] Hemoglobin ≥ 9.0 g/dL

[4] Platelets ≥ 10×10^4/ mm^3

[5] Total bilirubin≤ 1.5 mg/dL※4

[6] AST(sGOT)≦100 IU/L※4（for hepatic NEC and liver metastasis, ≦150 IU/L）

[7] ALT(sGPT)≦100 IU/L※4（for hepatic NEC and liver metastasis, ≦150 IU/L）

※4：Presence or absence of biliary drainage is not relevant

[8] Serum creatinine≦ 1.3 mg/dL

[9] Creatinine clearance※5≥ 60 mL/min

※5： Creatinine clearance must have been estimated using the Cockcroft-Gault formula, and must be 60 mL/min or more. If the estimation is less than 60 mL/min, but the actual measurement is 60 mL/min or more, the patient can be deemed eligible.

Cockcroft-Gault formula

Male：Ccr ={(140－age) × body weight (kg)}/{72×serum creatinine (mg/dL)}

Female：Ccr = 0.85×{(140－age) × body weight (kg)}/{72×serum creatinine(mg/dL)}

12) Written informed consent.

4.2. Exclusion criteria

1) Synchronous or metachronous (within 5 years) malignancies except carcinoma in situ or intramucosal tumor curatively treated with local therapy.

2) Active infection requiring systemic therapy.

3) Fever of 38 degrees Celsius or higher.

4) Pregnant or lactating women, women of childbearing potential, or women within 28 days after delivery.

5) Psychiatric disease.

6) Patients requiring systemic steroids medication.

7) Interstitial pneumonia, pulmonary fibrosis.

8) Serious co-existing illness.

9) Unstable angina pectoris within 3 weeks, or with a history of myocardial infarction within 6 months.

10) Impossible to use both iodine and gadolinium due to being allergic to contrast agent.

11) Uncontrolled diabetes mellitus or routine administration of insulin.

5. Registration and randomization

5.1. Procedure of registration

Ensure that a patient to be registered meets all eligibility criteria and does not meet any of exclusion criteria and register the patient by using JCOG Web Entry System. JCOG Web System Personal Account and password are required for web registration. If unknown, contact JCOG Data Center.

Patient registration JCOG Web Entry System
URL:https://secure.jcog.jp/dc/
(Web registration can be used for 24 hours.)

Contact information for patient registration and JCOG Web Entry System

JCOG Data Center
TEL: 03-3542-3373
Weekdays 9:00-17:00 (not available in holidays, Saturdays and Sundays, New Year's holidays)
E-mail:JCOGdata@ml.jcog.jp

Contact information on Patients Selection Criteria

Study Coordinator Contact : Chigusa Morizane
Hepato-Biliary Pancreatic Group: Futomi Mori (Main Research Secretariat)
Department of Hepatobiliary and Pancreatic Medicine, National Cancer Center
TEL:03-3542-2511
FAX:03-3542-3815
E-mail:cmorizan@ncc.go.jp

Gastric Cancer Group: Yoshinori Machida
Shizuoka Cancer Center
〒411-8777 1007 Shimonagakubo, Nagaizumi-cho, Sunto-gun, Shizuoka Prefecture
TEL:+81-55-989-5222
FAX:+81-55-989-5631
E-mail：no.machida@scchr.jp

Esophageal Cancer Group: Ken Kato/Yushi Homma
Department of Gastrointestinal Oncology, National Cancer Center
TEL: 03-3542-2511
FAX: 03-3542-3815
E-mail: kenkato@ncc.go.jp / yohonma@ncc.go.jp

5.1.1. Precautions for patient registration

① Registration after initiation of protocol treatment is unacceptable.
② Registration is performed by accessing the URL in '5.1. Procedure of registration'.
③ Eligibility checks are performed on the screen of Registration Form, so it is not necessary to send a Registration Form to Data Center by mail or fax.
④ If input data are insufficient, registration is not accepted until all are met.
⑤ The registration number is issued after the confirmation of eligibility on the registration screen, then the registration is completed.
⑥ Once registered, patients will not be retracted (retracted from the database) unless there is withdrawal of consent, including refusal to use the data for research. For duplicate registration, the information at the initial registration (registration number, allocated arm) are used in any case.
⑦ When misregistration or duplicate registration is found, contact Data Center immediately.
⑧ Body surface area and drug dose calculations are institutional responsibilities, and the body surface area

and drug dose displayed on Web Entry System at registration are only for double-checking. Those should always be calculated and checked at the institution. When the body surface area calculation formula adopted in the hospital information system of the institution differs from calculation formula adopted by JCOG (Dubois formula: Body surface area (m^2) = Body weight (kg) $^{0.425}$ x Height (cm) $^{0.725}$ 84 ÷ 10,000), there can be a difference in the dose by the hospital information system of the institution and the dose by the calculation formula adopted by JCOG, but in that case, either dosage used is decided by the site investigator.

5.2. Randomization and allocation adjustment factor

During enrollment, the treatment arm for a patient is allocated randomly by the data center.

Randomization would use a method of minimization using the 1) institutions, 2) Primary lesion organ (gastrointestinal tract [esophagus, stomach, duodenum, small intestines, appendix, colon, and rectum] vs. hepatobiliary and pancreatic organ [hepatic NEC, gallbladder, extrahepatic bile duct, ampulla of Vater, or pancreas]) as the adjustment factors, so that there are no large discrepancies between them. Researchers at participating facilities would not be informed of the detailed procedures of randomization.

6. Treatment Plan and Treatment Modification Criteria

Unless patient safety is threatened, treatment and treatment modifications is done in compliance with the specifications in this chapter.

If it is considered that the protocol specification may cause medically dangerous situation of the patient, treatment modifications should be made according to the medical judgment of the investigators/sub-investigators. Such protocol deviation is considered to be "clinically relevant deviation" if considered medically appropriate (see 14.1.4.Protocol deviation/violation). Deviations that occur with intentions other than safety, such as increasing efficacy, are not considered clinically relevant deviations.

6.1. Protocol treatment

Protocol treatment is initiated within 7 days of enrollment.

If treatment initiation occurs after 8 days from registration for any reason, the reason should be documented on the Treatment Course Form. If it is determined that treatment cannot be initiated, describe the details in the Off-treatment Form as Protocol Treatment Termination.

When laboratory parameters worsen and eligibility criteria are no longer met by the start of treatment after registration, the investigator/sub-investigator is allowed to decide whether initiate or terminate protocol treatment at their own discretion.

「6.3. The course initiation criteria is not applied at treatment changes.

Drugs used

- Etoposide ※
- Cisplatin ※
- Irinotecan ※
- Entecavir, tenofovir disoproxil fumarate, tenofovir alafenamide fumarate

The use of generic drugs is not restricted.

※ The company that manufactures or distributes these drugs, or intends to manufacture or sell these drugs, requires conflicts of interest control in the Clinical Trials Act as a company involved in this study (see 13.8.).

6.1.1. Arm A: Etoposide plus cisplatin (EP) therapy

The following chemotherapy courses will be given once a week for 3 weeks and treatment will be continued until the patient meets the discontinuation criteria.

Drug	Dosage(mg/m^2)	Dosing regimen/Dosing time	Dose day
Etoposide	100	IV/60-120 min	Day 1、2、3
Cisplatin	80	IV/60-120 min	Day 1

1) Calculation of the dosage

① Body surface area is calculated by determining drug dose to the third decimal point.

② For both etoposide and cisplatin, the calculated dose is determined by truncating the decimal point. For drug doses, ±10% is the acceptable range.

③ Dose recalculation due to body weight change after treatment initiation is not performed.

2) Administration of anticancer drugs

Etoposide and cisplatin are given in any order.

Examples of administration in the package insert are described below.

(i) Before administration: Before administration of anticancer drugs, 1,000-2,000 mL of hydration is done to achieve adequate diuresis.

(ii) Etoposide: Etoposide is mixed with infusion solutions such as isotonic sodium chloride solution of 250 mL or more, and infused intravenously in about 60-120 minutes. DEHP (2-ethylhexyl) phthalate: di-(2-ethylhexyl) phthalate), which is a plasticizer, elutes from polyvinyl chloride infusion sets and catheters. Avoid the use of polyvinyl chloride infusion sets and catheters.

(iii) Cisplatin administration: Cisplatin is mixed with stock solution or 500 mL of saline (or glucose-saline) and given intravenously in 60-120 min. During the administration, caution should be exercised in ensuring urine volume, and diuretics such as mannitol and furosemide should be administered as

necessary.

(iv) After administration: After administration of cisplatin, 1,000-2,000 mL of hydration should be done so that adequate diuresis can be achieved.

In addition, short hydration administration at the discretion of each institution is permitted. (see Table 6.1.1. for treatment cases).

Table 6.1.1. Example of short hydration

	Dose	Time
5HT3 antagonist<br>Dexamethasone<br>Physiological saline	 9.9 mg<br>50 mL	 15<br>min
Etoposide<br>Physiological saline	100 mg/m^2<br>250 mL	60<br>min
No. 1 solution<br>Potassium chloride<br>Magnesium sulfate	500 mL<br>10 mEq<br>8 mEq	 60<br>min
20% mannitol	200 mL	30<br>min
Cisplatin<br>Physiological saline	80 mg/m^2<br>250 mL	60<br>min
No. 1 solution<br>Potassium chloride	500 mL<br>10 mEq	60<br>min

(Referred from Horinouchi H, et al., Japan Society of Clinical Oncology 2012

3) Precautions for treatment

JCOG9511, an upfront trial in small-cell lung cancer, suggested an association between treatment-related deaths and first-course neutropenia. Therefore, caution should be exercised when neutropenia is strongly observed from the first course.

6.1.2. Arm B: Irinotecan plus cisplatin (IP) therapy

One 4-week course of the following chemotherapy will be continued until the discontinuation criteria are met. However, if day 15 irinotecan is skipped, 3 weeks should be used as one course, and the next course should be started with day 22.

Drug	Dosage(mg/m^2)	Dosing regimen/Dosing time	Dose day
Irinotecan	60	IV/90 min	Day 1、8、15
Cisplatin	60	IV/60-120 min	Day 1

1) Calculation of the dosage

(i) Body surface area is determined by determining the dose of drug administered until the third decimal point.

(ii) The dose is calculated for both irinotecan and cisplatin by truncating the decimal point. For drug doses, ±10% is the acceptable range.

(iii) Dose recalculation due to body weight change after treatment initiation is not performed.

2) Administration of anticancer drugs

Irinotecan and cisplatin are given in any order.

Examples of administration in the package insert are described below.

(i) Before administration: Before administration of anticancer drugs, 1,000-2,000 mL of hydration is done to achieve adequate diuresis.

(ii) Irinotecan: Irinotecan is mixed with 500 mL or more of isotonic sodium chloride solution, glucose solution, or electrolyte maintenance solution and given by intravenous drip infusion at about 90 minutes.

(iii) Cisplatin administration: Cisplatin is mixed with stock solution or 500 mL of saline (or glucose-saline) and given intravenously in 60-120 min. During the administration, caution should be exercised in ensuring urine volume, and diuretics such as mannitol and furosemide should be administered as necessary.

(iv) After administration: After administration of cisplatin, 1,000-2,000 mL of hydration should be done so that adequate diuresis can be achieved.

In addition, short hydration administration at the discretion of each institution is permitted. (See Table 6.1.1.

3) Precautions for treatment

JCOG9511, an upfront trial in small-cell lung cancer, suggested an association between treatment-related deaths and first-course neutropenia. Therefore, caution should be exercised when neutropenia is strongly observed from the first course.

6.2. Protocol Treatment Termination/Completion Criteria

6.2.1. Definition of protocol treatment completion

Protocol treatment is continued in this study unless the protocol treatment discontinuation criteria are met, so no definition of protocol treatment completion is provided.

6.2.2. Criteria for termination of protocol treatment

Protocol treatment is terminated in any of the following cases:

1) Judged as protocol treatment is ineffective
   - When a definite tumor exacerbation is confirmed by imaging or clinically
     ※ Protocol treatment should not be discontinued if the clinical judgement of PD based on the assessment of response based on imaging indicates that continuation of protocol treatment is appropriate, and protocol treatment should be continued.
2) Protocol treatment cannot be continued due to adverse events
   ① If Grade 4 non-hematological toxicity is observed (with the exception of the following adverse events) 'hypernatremia', 'hyponatremia', 'hyperkalemia', 'hypokalemia', 'hyperglycemia', 'hypoglycemia', 'alkaline phosphatase increase', 'alanine aminotransferase increase', 'aspartate aminotransferase increase', 'blood bilirubin increase','serum amylase increase', 'lipase increase', 'GGT increase'.
   (*Adverse events other than "anaemia," "myeloid cytopenia," "lymphocyte count decreased," "neutrophil count decreased," "white blood cell count decreased," "platelet count decreased," "CD4 lymphopenia" in CTCAE v4 0)
   ② If the next course cannot be initiated beyond 21 days from the expected start date of the course due to an adverse event
   ③ When the criteria for terminating protocol treatment in the treatment modification criteria (6.3.) are met.
   ④ Adverse events other than the treatment modification criteria that the investigator/sub-investigator judges to require termination of protocol treatment
3) If the patient offers termination of protocol treatment for reasons not denied to be associated with the adverse event
   - This category should be used if an association with an adverse event cannot be ruled out.
4) When the patient offers termination of protocol treatment because of reasons for denial of association with adverse events
   - Patient refusal after enrollment and before initiation of protocol treatment
   - When the association with an adverse event can first be denied, such as the relocation of the person or household member during protocol treatment.
5) Death during protocol treatment
   - Death before deciding to terminate protocol treatment for other reasons
6) Palliative surgery for pathogenic adverse events or surgery for patients with complete response to chemotherapy (see Section 6.3.9).
7) In addition, exacerbations before the start of treatment after enrollment (protocol treatment could not be initiated due to rapid exacerbation), protocol violations were found, ineligibility was determined due to modifications in pathological diagnosis after enrollment, etc., treatment was changed, and it was judged that it was difficult to continue protocol treatment due to social reasons and safety management problems, etc.

The date of discontinuation of protocol treatment is defined as the date of death in 6.2.2.5), the date of surgery in 6.2.2.6), and otherwise, the date on which the treating physician judges that protocol treatment is discontinued.

In this study, non-NEC may be diagnosed by central pathological diagnosis during protocol treatment, because central pathological diagnosis is performed. If the patient is continuing on protocol treatment when the results of the central pathology diagnosis are reported to the registry, the attending physician and the institutional pathologist will review the results and take a clinically appropriate response. Protocol treatment is discontinued if discontinuation of protocol treatment is judged to be appropriate, and the reason for discontinuation is other.

6.3. Treatment modification criteria

The following terms shall be used for the treatment modification.

Delay: Delay the start of the course or administration of treatment from the planned date.

Termination: Discontinuation of a part of or all of the treatment without restarting.

Suspending: temporary interruptions or withdrawals that may be resumed if conditions are met

Skip: Do not administer one or more drugs and proceed to the next schedule.

Categories of infection (CTCAEv4.0) used in this study are as follows

Infection: CTCAEv4.0 infections and infestations

Bronchial infection; pulmonary infection; upper respiratory tract infection; mediastinal infection; pleural infection; catheter-related infection; Biliary tract infection; Gallbladder infection; urinary tract infection

6.3.1. Arm A (EP-therapy): Dose level

1) Etoposide

Drug	Dose level	Dosing schedule	Dose day
Etoposide	Level 0	100 mg/m^2 div	Day 1、2、3
	Level -1	80 mg/m^2 div	Day 1、2、3
	Level -2	60 mg/m^2 div	Day 1、2、3

2) Cisplatin

Drug	Dose level	Dosing schedule	Dose day
Cisplatin	Level 0	80 mg/m^2 div	Day 1
	Level -1	60 mg/m^2 div	Day 1
	Level -2	40 mg/m^2 div	Day 1

6.3.2. A arm (EP therapy): Course initiation criteria

- Initiate the course after confirming that all of the following "Course Initiation Criteria" are met on the scheduled start date of the course or the day before the plan start date of the course.
- If any one is not met, the initiation of the course is delayed.
- If the course cannot be initiated within 21 days of the expected start date of the course (if the start date of the previous course was day 1 and the course could not be initiated by day 42), discontinue the protocol treatment.
- The course initiation criteria is not applied at the start of the first course.

Course initiation criteria

① Neutrophil count Grade 0-1 (≧1500 per mm^3).

② Platelet count ≧10×10^4 /mm^3

③ AST≦100 IU/L (in the presence of hepatic metastases vs. hepatic NECs) was ≦200 IU/L).

④ ALT≦100 IU/L (≦200 IU/L for liver metastases vs liver NECs).

⑤ Total bilirubin <= 2.0 mg/dL

⑥ Serum creatinine <= 1.5 mg/dL

⑦ Fever Grade 0 (axillary temperature, no antipyretic)

⑧ Constipation, fatigue, phlebitis, oral mucositis, and infection ※1 are all Grade 2 or less.

Infected ※1: bronchial infection, pulmonary infection, upper respiratory tract infection, mediastinal infection, pleural infection, catheter-related infection, biliary tract infection, gallbladder infection, urinary tract infection

⑨ Anorexia, nausea, and emesis are all Grade 0-1.

⑩ Diarrhoeal Grade 0

6.3.3. Arm A (EP therapy): Dose reduction criteria

If any of the following toxicities are identified during the course, dose reduction should be performed in accordance with the dose reduction criteria (Table 6.3.3.) from the following course (no dose reduction in the course).

However, even if two or more items are met, the dose reduction for each drug is only one step. Re-escalation after dose reduction is not performed. Protocol treatment is terminated if the dose reduction criteria are met again after the dose reduction to Level-2.

Table 6.3.3. Arm A (EP therapy): Dose reduction criteria

Item	Etoposide	Cisplatin
Neutrophil count Grade 4 (<500 per mm^3).	Reduce the level by 1	No change
Platelet count Grade 4 (<2.5×10^4/mm^3)	Reduce the level by 1	No change
1.5 <serum creatinine ≦2.0 mg/dL.	No change	Reduce the level by 1
Serum creatinine <= >2.0 mg/dL	Termination of protocol treatment	Termination of protocol treatment
Grade 3<br>Infected ※1	Reduce the level by 1	Reduce the level by 1
Grade 3<br>Assessment at onset of febrile.	Reduce the level by 1	Reduce the level by 1
Grade 2<br>Peripheral sensory neuropathy<br>Peripheral motor neuropathy,<br>Myalgia; arthralgia; tinnitus; hearing impairment	No change	Reduce the level by 1
Grade 3<br>Peripheral sensory neuropathy<br>Peripheral motor neuropathy,<br>Myalgia; arthralgia; tinnitus; hearing impairment	Termination of protocol treatment	Termination of protocol treatment
Non-hematologic toxicities of Grade 3 other than those listed above in ※2 that are causally related to EP-therapy (excluding hyponatremia, hypokalemia, hypomagnesemia, hypocalcemia, hyperglycemia, and weight loss)	Reduce the level by 1	Reduce the level by 1

※1:※1 of infection: bronchial infection, pulmonary infection, upper respiratory tract infection, mediastinal infection, pleural infection, catheter-related infection,
Biliary tract infection; Gallbladder infection; Urinary tract infection

※2:Causal relationship is judged as either of possible, probable, definite

6.3.4. Arm A (EP therapy): Within-course pause, skipping criteria

Following initiation of treatment with each course after the course initiation criteria are met, if any of the following adverse events are observed, day 2, day 3 etoposide will be suspended.

- Fever (axillary temperature) Grade 1-3
- Grade 3 of infection (bronchial infection, pulmonary infection, upper respiratory tract infection, mediastinal infection, pleural infection, catheter-related infection,
  Biliary tract infection, gallbladder infection, urinary tract infection)

Resting etoposide should be resumed after confirming that all initiation criteria are met until day 7. However, it should not be administered after day 8.

That is, if etoposide could not be administered by day 7, the remaining etoposide should be skipped.

If the above suspension and/or skip occur, the next course of etoposide should be started day 22 (after 3 weeks) counting from day 1 of the previous course if the ininiation criteria are met.

6.3.5. Arm B (IP therapy): Dose-level

1) Irinotecan

Drug	Dose level	Dosing schedule	Dose day
Irinotecan	Level 0	60 mg/m^2 div	Day 1、8、15
	Level -1	50 mg/m^2 div	Day 1、8、15
	Level -2	40 mg/m^2 div	Day 1、8、15

2) Cisplatin

Drug	Dose level	Dosing schedule	Dose day
Cisplatin	Level 0	60 mg/m^2 div	Day 1
	Level -1	50 mg/m^2 div	Day 1
	Level -2	40 mg/m^2 div	Day 1

6.3.6. Arm B (IP therapy): Course initiation criteria

- On the day of the initiation of the course or the day before the expected start of the course, start the course after confirming that all of the following "Course Initiation Criteria" are met.
- If any one is not met, the initiation of the course is delayed.
- If the course cannot be initiated within 21 days of the expected start date of the course (if the start date of the previous course was day 1 and the course could not be initiated by day 49), discontinue the protocol treatment.
- However, if day 15 irinotecan is skipped in the previous course, day 22 of the previous course is set as the scheduled start date of the next course regarded as one course per 3 weeks.
- The course initiation criteria is not applied at the start of the first course.

Course initiation criteria

① Neutrophil count Grade 0-1 (≧1500/mm^3).
② Platelet count ≧10×10^4 /mm^3
③ AST≦100 IU/L (≦200 IU/L for liver metastases versus liver NECs)).
④ ALT≦100 IU/L (≦200 IU/L for liver metastases vs liver NECs).
⑤ Total bilirubin <= 2.0 mg/dL
⑥ Serum creatinine <= 1.5 mg/dL
⑦ Fever Grade 0 (measured by axillary temperature, temperature <38°C without antipyretic use)
⑧ Constipation, fatigue, phlebitis, oral mucositis, and infection ※1 are all Grade 2 or less.
Infected ※1: bronchial infection, pulmonary infection, upper respiratory tract infection, mediastinal infection, pleural infection, catheter-related infection, biliary tract infection, gallbladder infection, urinary tract infection
⑨ Anorexia, nausea, and emesis are all Grade 0-1.
⑩ Diarrhoea Grade 0

6.3.7. Arm B (IP-therapy): Dosing criteria for day 8, day 15

After confirming that all of the following ①-③ are met, the second (day 8) or third (day 15) dose of irinotecan is administered. If day 8, day 15 dosing criteria are not met, skip day 8, day 15 dosing.

① All of the following are met with the most recent laboratory data on the scheduled day of administration or the day before the scheduled day of administration.
i) Neutrophil count Grade 0-2 (≧1000 per mm^3).
ii) Platelet count ≧10×10^4 /mm^3
iii) AST ≦ 100 IU/L (≦200 IU/L for liver metastases vs liver NECs).
iv) ALT ≦ 100 IU/L (≦200 IU/L in the presence of hepatic metastases versus hepatic NECs).
v) Total bilirubin <= 2.0 mg/dL
vi) Serum creatinine <= 2.0 mg/dL
② All of the following are met on the scheduled day of administration:
i) Fever Grade 0 (measured by axillary temperature, temperature <38°C without antipyretic use)

ii） Diarrhoeal Grade 0

③ Constipation, anorexia, nausea, emesis, fatigue, phlebitis, oral mucositis, and infection ※1 are all Grade 2 or less.

Infected ※1: bronchial infection, pulmonary infection, upper respiratory tract infection, mediastinal infection, pleural infection, catheter-related infection, biliary tract infection, gallbladder infection, urinary tract infection

6.3.8. Arm B (IP therapy): Dose reduction criteria

If any of the following toxicities are identified during the course, dose reduction should be performed in accordance with the dose reduction criteria (Table 6.3.8.) from the following course (no dose reduction in the course).

However, even if two or more items are met, the dose reduction for each drug is only one step. Re-escalation after dose reduction is not performed. Protocol treatment is terminated if the dose reduction criteria are met again after the dose reduction to Level-2.

Table 6.3.8. Arm B (IP therapy): Dose reduction criteria

Item	Irinotecan	Cisplatin
Neutrophil count Grade 4 (<500 per mm^3).	Reduce the level by 1	No change
Platelet count Grade 4 (< 2.5×10^4/mm^3)	Reduce the level by 1	No change
1.5 <serum creatinine ≦2.0 mg/dL.	No change	Reduce the level by 1
Serum creatinine <= >2.0 mg/dL	Termination of protocol treatment	Termination of protocol treatment
Grade 3<br>Infected ※1	Reduce the level by 1	Reduce the level by 1
Grade 3<br>Assessment at onset of febrile.	Reduce the level by 1	Reduce the level by 1
Grade2<br>Peripheral sensory neuropathy<br>Peripheral motor neuropathy,<br>Myalgia; arthralgia; tinnitus; hearing impairment	No change	Reduce the level by 1
Grade 3<br>Peripheral sensory neuropathy<br>Peripheral motor neuropathy,<br>Myalgia; arthralgia; tinnitus; hearing impairment	Termination of protocol treatment	Termination of protocol treatment
Non-hematologic toxicities of Grade 3 other than those listed above in ※2 that are causally related to IP-therapy (excluding hyponatremia, hypokalemia, hypomagnesemia, hypocalcemia, hyperglycemia, and weight loss)	Reduce the level by 1	Reduce the level by 1

※1:※1 of infection: bronchial infection, pulmonary infection, upper respiratory tract infection, mediastinal infection, pleural infection, catheter-related infection,

Biliary tract infection; Gallbladder infection; Urinary tract infection

※2:Causal relationship is judged as either of possible, probable, definite

6.3.9. Surgical of after end of chemotherapy

1) Surgery for Adverse Events Associated with Pathogenesis

Surgery may be performed if it is judged clinically desirable to perform surgery for newly appearing symptoms such as hemorrhage or stenosis. Protocol treatment is discontinued if surgery is performed, regardless of the content. In this case, the date of discontinuation of protocol treatment is the date of surgery.

2) Surgery for patients with complete response to chemotherapy

- When chemotherapy is highly effective, all metastases present at the time of enrollment on imaging studies disappear, and curative resection (R0 resection) is considered possible, resection including primary and metastatic disease may be performed.
- Surgical procedures are not specifically specified, but the Research Office will collect information on the details of the surgery performed individually in order to be reviewed by the research representative/research office.

- In all surgical cases, the group group conference shall confirm the validity of the judgment that surgery is indicated.
- When surgery is performed, protocol treatment is discontinued, regardless of whether curative resection was performed or not. In this case, the date of discontinuation of protocol treatment is the date of surgery.

6.3.10. Consultation on treatment modification

If there are any questions about treatment modification, contact "16.6. Study Coordinator".

Study Coordinator Contact : Chigusa Morizane

Hepato-Biliary Pancreatic Group: Futomi Mori (Main Research Secretariat)
Department of Hepatobiliary and Pancreatic Medicine, National Cancer Center
TEL:03-3542-2511
FAX:03-3542-3815
E-mail:cmorizan@ncc.go.jp

Gastric Cancer Group: Yoshinori Machida
Shizuoka Cancer Center
〒411-8777 1007 Shimonagakubo, Nagaizumi-cho, Sunto-gun, Shizuoka Prefecture
TEL:+81-55-989-5222
FAX:+81-55-989-5631
E-mail：no.machida@scchr.jp

Esophageal Cancer Group: Ken Kato/Yushi Homma
Department of Gastrointestinal Oncology, National Cancer Center
TEL:03-3542-2511
FAX:03-3542-3815
E-mail: kenkato@ncc.go.jp / yohonma@ncc.go.jp

6.4. Concomitant treatment and supportive care

6.4.1. Required concomitant treatment/supportive care

1) Testing and Supportive Care for HBsAg-Positive Cases.

In HBsAg-positive cases, steroids and chemotherapy can lead to rapid hepatitis B virus (HBV) expansion (reactivation: reactivation) and potentially fatal severe hepatitis. Therefore, the following tests and supportive care are performed based on the "Guidelines for the Treatment of Hepatitis B, Third Edition (Japanese Society of Hepatology)." It is advisable to consult a hepatologist at the time prior to initiation of a nucleic acid analogue (entecavir, tenofovir disoproxil fumarate, and tenofovir alafenamide fumarate).

①Testing prior to initiation of chemotherapy: HBV-DNA quantitation

HBV-DNA quantitative analysis should be performed at least once prior to initiation of chemotherapy.

HBV-DNA assays are performed by real-time PCRs.

HBeAg and HBe antibodies should also be measured in accordance with the Guidelines for the Treatment of Hepatitis B, Third Edition (Japanese Society of Hepatology).

②Dosing schedule for supportive care (nucleic acid analogues prophylaxis)

- **Drugs used :**
  - **Entecavir (Bristol-Myers: Baraclude Tablets 0.5 mg)**
  - **Tenofovir disoproxil fumarate (GlaxoSmithKline: Tenozet Tablets 300 mg)**
  - **Tenofovir alafenamide fumarate (Giliado: Vemuridi Tablets 25 mg)**

The following dosage regimen should be followed, starting at least 1 week before the start of chemotherapy (as soon as possible), and continuing for at least 12 months after the end of chemotherapy. However, fulminant hepatitis has been reported in HBsAg-positive patients with high viral load, even during NA prophylaxis, and it is desirable to reduce the viral load before starting immunosuppression/chemotherapy. Nucleic acid analogues may be discontinued after 12 months after completion of chemotherapy if conditions *1 and 2 for discontinuation of NAs are met. However, if the administration of a nucleic acid analogue is discontinued, consultation with a hepatologist is always obtained, and the administration is discontinued only if the hepatologist deems it appropriate.

※1 Requirements for discontinuation of nucleic acid analogues (entecavir, tenofovir disoproxil fumarate, tenofovir alafenamide fumarate): all of the following

1. The patient has been on NA for more than 2 years.
2. HBV-DNA assays are not sensitive to detect
3. Be negative for HBeAg

※2 Patient background requirements: All of the following

1. Both the treating physician and the patient have sufficiently understood that the hepatitis exacerbation is frequently observed after the nucleic acid analog withdrawal, and that there is a danger of becoming serious in the time.
2. Follow-up is possible after treatment cessation, and appropriate treatment is possible even if hepatitis recurs
3. It is judged that the liver fibrillation is slight and the hepatic reserve is good, and it is difficult to become serious even if the hepatitis is exacerbated.

(Adapted from Guidelines for the Treatment of Hepatitis B, the 3rd edition (Japanese Society of Hepatology))

Entecavir

- **Dosage regimen: Take this medicine on an empty stomach (2 hours after meals and more than 2 hours before the next meal).**
- **Dosage :**

Creatinine clearance (mL/min).	Dosage
50 or more	0.5 mg once daily
30 Beyond 50	0.5 mg once every 2 days
10 Beyond 30	0.5 mg once every 3 days
10 Less than	0.5 mg once every 7 days

- **Adverse drug reactions (incidence of all grades): nucleoside analog-naïve patients**

Diarrhea (6.0%), nausea (4.5%), constipation (3.7%), upper abdominal pain (3.0%), malaise (1.5%), nasopharyngitis (3.0%), muscle stiffness (2.2%), headache (3.0%), rash (incidence unknown), laboratory tests: elevated AST (3.7%), increased blood bilirubin (6.0%), increased blood amylase (10.4%), and increased lipase

(10.4%).Blood glucose increased (6.0%), blood lactate increased (6.7%), urine occult blood positive (4.5%), white blood cell count decreased (8.2%), and eosinophil count increased (0.7%). [Significant adverse reactions (incidence unknown)] Hepatitis worsened after completion of treatment, anaphylactoid symptoms, lactic acidosis, and severe hepatomegaly due to fatty liver

Tenofovir disoproxil fumarate

- **Dosage and administration: 300 mg is orally administered once daily.**
- **Dosage :**

Creatinine clearance (mL/min).	Dosage
50 or more	300 mg once daily
30 Beyond 50	300 mg once every 2 days
10 Beyond 30	300 mg once every 3 to 4 days
Hemodialysis	注) of 300 mg once every 7 days<br>Or 300 mg after completion of cumulative approximately 12 hours of dialysis<br>NOTE) After hemodialysis was performed. The pharmacokinetics in patients with creatinine clearance < 10 mL/min and not on hemodialysis have not been investigated.

- **Dosing Precautions:**

In the long-term administration of tenofovir disoproxil fumarate, attention should be paid to renal dysfunction, hypophosphatemia (including Fanconi syndrome), and decrease in bone mineral density. It is recommended that renal function and serum phosphorus should be measured regularly during tenofovir disoproxil fumarate administration.

- **Adverse reactions (incidence of all grades):**

Abnormal liver function tests (AST, ALT and γ-GTP increased, etc.) in 7 patients (4.9%), increased creatinine in 4 patients (2.8%), increased amylase, increased lipase and nausea in 3 patients each (2.1%), abdominal pain in 2 patients (1.4%), [major adverse reactions (incidence unknown)] renal dysfunction, renal failure, acute renal failure, proximal renal tubular dysfunction, Fanconi syndrome, severe renal dysfunction such as acute renal tubular necrosis, nephrogenic diabetes insipidus or nephritis, severe hepatomegaly due to lactic acidosis and fatty deposition (steatohepatitis), pancreatitis

Tenofovir alafenamide fumarate

- **Dosage and administration: 25 mg is orally administered once daily.**
- **Dosage :**

Creatinine clearance (mL/min).	Dosage
15 or more	25 mg once daily
Less than 15	Consider discontinuation

- **Dosing Precautions:**

In the long-term administration of tenofovir alafenamide fumarate, attention should be paid to renal dysfunction, hypophosphatemia (including Fanconi syndrome), and decrease in bone density. It is recommended that renal function and serum phosphorus should be measured periodically during tenofovir alafenamide fumarate administration.

- **Adverse reactions (incidence of all grades):**

Nausea and abdominal distension, headache, fatigue (≧1%), dyspepsia and diarrhea, flatus, upper abdominal pain, constipation, ALT increased, arthralgia, dizziness, insomnia, pruritus, rash (≧0.5% to <1%), [significant adverse reactions (incidence unknown)] renal dysfunction, renal failure, acute renal failure, proximal renal tubular dysfunction, severe renal impairment such as Fanconi syndrome, acute renal tubular necrosis, renal diabetes insipidus or nephritis, severe hepatomegaly due to lactic acidosis and fatty deposits (fatty liver)

③Monitoring: Quantitative analysis of HBV-DNA (during and after administration of nucleic acid analogues)

During nucleic acid analogue administration:

They are monitored every 4 weeks by both HBV-DNA quantitative analysis and liver function (ASTs, ALTs). However, if HBV-DNA level is less than 20 IU/mL (1.3 log IU/mL) during administration of nucleic acid analogues, it is acceptable to perform tests every 4 to 12 weeks.

After discontinuation of nucleic acid analogue administration:

Bearing in mind that reactivation may occur even after discontinuation of administration of a nucleic acid analogues, the patient should be consulted with a hepatologist, and the patient should be monitored for HBV-DNA determination and hepatic function (AST/ALT) every 4 weeks for at least 1 year after discontinuation of administration of a nucleic acid analogues. Subsequent follow-up will be decided after consulting a hepatologist.

2) Laboratory Teste and Supportive Care for HBsAg-Negative and HBc Antibody-Positive and/or HBs Antibody-Positive Cases.

HBV-DNA quantitative analysis should be performed at least once prior to initiation of chemotherapy. HBV-DNA assays are performed by real-time PCRs.

i) HBV-DNA ≧ 20 IU/mL (1. 3 log IU/mL) prior to initiation of chemotherapy

It has been clarified that HBV-DNA replicates persist at low levels in the livers and peripheral blood mononuclear cells when HBc or HBs are positive, even if they are HBs-Ag negative. It has been reported that reactivation of HBV and development of severe hepatitis are caused by the use of potent immunosuppressive agents even in such patients with previous infections.

If HBV-DNA ≧ 20 IU/mL (1.3 log IU/mL), the risk of HBV reactivation is judged to be as high as in HBsAg-positive cases, and prophylactic administration of nucleic acid analogues (entecavir or tenofovir disoproxil fumarate, tenofovir alafenamide fumarate) is administered. The following laboratory tests and supportive care are performed in accordance with the "Guideline for the Treatment of Hepatitis B, 3rd edition (Japanese Society of Hepatology)" with reference to the following for examination, dosage, and monitoring of supportive care before the start of chemotherapy.

However, these are not applicable if the HBs antibody alone is positive and the HBV vaccination history is obvious.

①Dosing schedule for supportive care (nucleic acid analogues prophylaxis)

According to the dosage and administration of nucleic acid analogues (entecavir, tenofovir disoproxil fumarate, tenofovir alafenamide fumarate) in "1) Test and supportive care for HBsAg positive cases". Same conditions for NA discontinuation.

② Monitoring: Quantitative analysis of HBV-DNA (during and after administration of nucleic acid analogues)

The intervals for monitoring during and after discontinuation of NA are in accordance with the provision of "1) Testing and supportive care for HBsAg-positive patients".

ii) HBV-DNA less than 20 IU/mL (1.3 log IU/mL) prior to initiation of chemotherapy

HBV-DNA quantitative analysis and hepatic function (AST, ALT) will be monitored, and nucleic acid analogues (entecavir, tenofovir disoproxil fumarate, tenofovir alafenamide fumarate) will be started when ≧20 IU/mL (1.3 log IU/mL) is achieved.

The Guidelines for the Treatment of Hepatitis B, the 3rd edition (Japanese Society of Hepatology) recommends monitoring with HBV-DNA quantitative analysis or high-sensitivity HBs antibodies during and after chemotherapy, depending on the risks of revitalization.

①Monitor:HBV-DNA quantitative analysis

HBV-DNA quantitative analysis should be performed every 4-12 weeks from the start of chemotherapy until at least 12 months after the end of chemotherapy.

If HBV-DNA level is more than 20 IU/mL (1.3 log IU/mL), administration of nucleic acid analogues should be started immediately in accordance with the Guidelines for the Treatment of Hepatitis B, the 3rd edition (Japanese Society of Hepatology). If HBsAg monitoring is positive for < 1 IU/mL (low positive), nucleic acid analogues should be administered after additional HBV DNA determinations of ≧ 20 IU/mL (1.3 log IU/mL).

It is advisable to consult a hepatologist at a time prior to initiation of NAs.

②Supportive care in reactivation

Nucleic acid analogues should be administered according to the supportive care described in i) When HBV-DNA prior to the initiation of chemotherapy is 20 IU/mL (1.3 log IU/mL) or more in 6.4.1.2). Once administration of nucleic acid analogues is started, nucleic acid analogues should be discontinued only if appropriate by the hepatologist.

6.4.2. Recommended/not recommended concomitant treatment/supportive care

The following concomitant treatment and supportive care are recommended. Even if it is not carried out, it is not regarded as protocol deviation,

1) Addressing Febrile Neutropenia.

① Assessment at onset of febrile neutropenia (FN).

a) If the count is less than 500 per mm^3, or is less than 1000 per mm^3 and is expected to be less than 500 per mm^3 within 48 hours, and if the axillary temperature is 37.5°C or higher (mouth temperature is 38.0°C or higher), a severity-risk assessment should be performed promptly and anti-virus treatment initiated as appropriate.

b) Severity risk assessment is performed with reference to Multinational Association for Supportive Care in Cancer(MASCC) scoring system ※1.

c) For initial evaluation, complete blood count including differential WHITE BLOOD CELL and platelet count, renal function (BUN, creatinine), electrolytes, liver function (transaminases, total bilirubin, alkaline phosphatase) tests, ≧2 sets of venous blood cultures prior to initiation of antimicrobials, one set of cultures from the catheter lumen and one set from the peripheral vein if a central venous catheter is in place, cultures of suspected infection areas, and chest x-rays if respiratory symptoms and signs are present.

d) When febrile neutropenia (FN) develops in a patient with a central venous catheter, blood cultures from the catheter and peripheral blood are performed, and catheter-related infections are considered if there is a time difference of more than 120 minutes in the positivity of both. If appropriate antimicrobial therapy does not improve after more than 72 hours, catheter should be removed. For infections caused by Staphylococcus aureus, Pseudomonas aeruginosa, Bacillus, fungi, and acid-fast bacilli, the catheter should be removed and appropriate antimicrobial therapy based on culture results should be performed.

② Antibiotic use

a) In high-risk patients, β-lactams with anti-Pseudomonas aeruginosa activity are administered intravenously as a single agent. However, other antimicrobials (aminoglycosides, fluoroquinolones, and/or vancomycin) may be added to a single agent in the initial regimen in patients with unstable or complicated conditions or when drug-resistant organisms are strongly suspected. Low-risk patients may be treated with antibiotics orally or intravenously, hospitalized, or with adequate evaluation, if appropriate, as outpatients.

b) Re-evaluation will be performed 3-4 days after initiation of antibiotics to investigate the continuation or change of antibiotics. In principle, antibiotics will be continued until the neutrophil count recovers to 500 cells per mm^3 or more.

c) Empiric antifungal therapy is recommended in high-risk patients who do not respond to 4-7 days of broad-spectrum antibiotics.

d) Fluoroquinolone prophylaxis is recommended in high-risk individuals with an expected neutrophil count of 100 cells per mm^3 or less lasting >7 days.

③ Therapeutic administration of G-CSF

For therapeutic administration of G-CSF during the incidence of FNs, refer to "6.4.5. 3) Therapeutic administration of G-CSF".

※1 Multinational Association for Supportive Care in Cancer (MASCC) scoring system.
(Adapted in part from the Practice Guideline for Febrile Neutropenia (FN) [Japanese Society of Medical Oncology]. *2)

Item	Score
Clinical manifestations (select one of the following * mark 3 sections)<br>*No symptoms<br>*Mild symptoms<br>*Moderate symptoms	 5<br>5<br>3
No decrease in blood pressure	5
No chronic obstructive pulmonary disease	4
Solid tumors, or hematopoietic tumors without a history of fungal infection	4
No dehydration symptoms	3
Patients with fever during outpatient management	3
Age < 60	2

The total score is up to 26 points. Twenty-one points or more are considered low risk and 20 points or less are considered high risk

※2 Since patients aged 20 years or older are subjects in this study, we deleted "Not applicable to patients younger than 16 years old" from the original edition of the Practice Guideline for Febrile Neutropenia (FN) [Japanese Society of Medical Oncology].

2) Nausea and vomiting

Regarding nausea and vomiting, antiemetics are positively administered according to Clinical Practice Guidelines for Antiemesis in Oncology [48], and fluid and electrolyte repletion are performed when oral intake is severely reduced.

3) Anorexia

If oral intake drops markedly, fluid and electrolyte supplements should be given as needed. Especially, in the cases with diabetes mellitus, the abnormality of blood sugar level and electrolyte is noticed.

4) Anemia, thrombocytopenia

If anaemia (haemoglobin < 8.0 g/dL) or thrombocytopenia (platelet count < $2\times10^4/mm^3$) is observed, blood should be transfused as appropriate at the discretion of the treating physician.

5) Diarrhea

Severe diarrhoea occasionally occurs with arm B (IP therapy arm) and is extremely dangerous when complicated by febrile neutropenia. Patients should be fully informed about toxicities and their management, and should be instructed to measure body temperature, especially when neutrophils counts are most decreasing, and to contact a physician or nurse immediately during fever and diarrhea. Nonsteroidal anti-inflammatory drugs may not cause fever to become overt, so unnecessary anti-inflammatory drugs are not given.

If irinotecan-induced diarrhea occurs, the following supportive measures are recommended:

High-dose loperamide hydrochloride therapy

① Loperamide hydrochloride was started after signs of diarrhea were observed.
② Initial dose of 4 mg followed by 2 mg/2 hours (4 mg/4 hours at night)
③ It is administered until watery stool does not appear for more than 12 hours.
④ Doses should not be given for more than 48 hours.

6) Precautions on the day of cisplatin administration

Aminoglycoside antibiotics, vancomycin, and nonsteroidal anti-inflammatory drugs are not administered on the day of cisplatin administration or are used with caution.

6.4.3. Acceptable concomitant treatment and supportive care

The following concomitant treatment and supportive care may be used as needed.

Concomitant use of drugs for the treatment of complications such as hypertension and diabetes mellitus and symptomatic drugs such as morphine may be performed, but this drug should be administered with caution when furosemide, piretanide and phenytoin are used. Oral antibiotics for febrile neutropenia prophylaxis may be given at the discretion of the investigator/sub-investigator. Bisphosphonate denosumab may be used in combination for bone metastases.

6.4.4. Unacceptable concomitant treatment and supportive care

None of the following treatments will be given during protocol treatment:

① Administration of anticancer drugs other than protocol treatment

② Radiation therapy

6.4.5. Granulocyte colony-stimulating factor (granulocyte-colony stimulating factor:G-CSF

※This study permits the use of G-CSF biogenerics (biosimilars).

1)※ of primary prophylaxis with G-CSF

※Primary prophylaxis: G-CSF administration before developing febrile neutropenia or prolonged neutropenia to prevent them during anticancer therapy.

Primary prophylaxis with G-CSF was not recommended at the beginning of the study in this study. However, since febrile neutropenia in group A was 21.4% in the late 2016 periodic monitoring report and 15.8% in the early 2017 periodic monitoring report (18.4% when the time of occurrence was April 2017 and SAE reports not reflected in the early 2017 periodic monitoring report were included), it was decided that the patient would meet at least the recommended grade B or higher (almost A) according to G-CSF Appropriate Use Guideline 2013 and JSMO Febrile Neutropenia (FN) Practice Guideline. Primary prophylactic administration of G-CSF was recommended in group A. However, it is not considered a protocol deviation even if it is not administered. Since it is practical to administer pegfilgrastim from the viewpoint of convenience, when the primary preventive administration of G-CSF is carried out in the actual medical field, the administration example of pegfilgrastim is shown below.

(Administration cases)

Group A: 3.6 mg of pegfilgrastim (genetical recombination) will be injected subcutaneously (once per chemotherapy course) between day 4(day 3's completion of etoposide treatment and day 7 at least 24 hours after the completion of etoposide treatment.

Since the safety of pegfilgrastim administered 14 days prior to the initiation of cancer chemotherapy and within 24 hours after completion of administration has not been established, the primary prophylactic administration of pegfilgrastim is not performed in group B where day 8 is administered the drug.

Table 6.4.5. Primary prophylactic administration of G-CSF should be performed according to the approved dosage and administration shown in the table below.

Drug	・・ Pegfilgrastim (arm A only)<br>・・ Filgrastim<br>・・ Naltograstim<br>・・ Lenograstim
Time of initiation	Twenty-four hours after completion of chemotherapy
Dosage Dosing regimen	・Pegfilgrastim (genetical recombination) at a dose of 3.6 mg subcutaneously once per chemotherapy course (group A only)<br>・Filgrastim: 50 μg per m^2 SC once daily or 100 μg per m^2 IV once daily<br>・Naltograstim: 1 μg/kg SC once daily or 2μg/kg IV once daily<br>・Lenograstim: 2 μg/kg SC once daily or 5μg/kg IV once daily
Timing of discontinuation (other than pegfilgrastim)	・If the neutrophil count reaches 5000 per mm^3 or more after the course, administration should be discontinued.<br>・If the neutrophil count recovers to ≧2000 cells per mm^3, there are no suspected infections, and the patient's safety is judged to be ensured by the responsiveness to the drug used, the drug should be discontinued or reduced.

2)Secondary prophylactic※ of G-CSF

※ Secondary prophylaxis: G-CSF prophylactic administration after once occurrence of febrile neutropenia or prolonged neutropenia to prevent febrile neutropenia or prolonged neutropenia from occurring again during anticancer therapy.

If febrile neutropenia occurs in the previous course, secondary prophylaxis with G-CSF after the subsequent course is recommended, even if dose reduction or schedule modification or antimicrobial therapy is considered to be associated with a lower risk of febrile neutropenia (group A). However, it is not considered a protocol deviation even if it is not administered. As it is practical to administer pegfilgrastim in terms of convenience when secondary prophylactic administration of G-CSF is carried out in real medical practice, the following examples of administration of pegfilgrastim are shown.

(Administration cases)

Group A: 3.6 mg of pegfilgrastim (genetical recombination) is injected subcutaneously (once per course of chemotherapy) between the time of completion of etoposide treatment of day 4(day 3 and day 7 of at least 24 hours.

However, the safety of pegfilgrastim administered 14 days prior to the start of cancer chemotherapy and 24 hours after the end of treatment has not been established, so secondary prophylactic pegfilgrastim administration is not performed in group B, where day 8 is given the drug.

Secondary prophylactic administration of G-CSF should be performed according to the approved dosage and administration shown in the table below.

Drug	•• Pegfilgrastim (arm A only)<br>•• Filgrastim<br>•• Naltograstim<br>•• Lenograstim
Time of initiation	•• Pegfilgrastim (arm A only)<br>Twenty-four hours after completion of chemotherapy<br>•• Filgrastim, nartograstim, and lenograstim<br>When neutrophil counts <1000 per mm^3 are observed
Dosage Dosing regimen	• Pegfilgrastim (genetical recombination) at a dose of 3.6 mg subcutaneously once per chemotherapy course (group A only)<br>•Filgrastim: 50 μg per m^2 SC once daily or 100 μg per m^2 IV once daily<br>•Naltograstim: 1 μg/kg SC once daily or 2μg/kg IV once daily<br>•Lenograstim: 2 μg/kg SC once daily or 5μg/kg IV once daily
Timing of discontinuation (other than pegfilgrastim)	•If the neutrophil count reaches 5000 per mm^3 or more after the course, administration should be discontinued.<br>•If the neutrophil count recovers to ≧2000 cells per mm^3, there are no suspected infections, and the patient's safety is judged to be ensured by the responsiveness to the drug used, the drug should be discontinued or reduced.

3) Therapeutic administration of G-CSF

Therapeutic administration of G-CSF should be performed according to the approved dosage and administration shown in the table below.

Time of initiation	•When the neutrophil count is less than 1000 per mm^3 and fever (in principle, > 38.0°C) is observed<br>•When neutrophil counts <500 per mm^3 are observed
Dosage Dosing regimen	•Filgrastim: 50 μg per m^2 SC once daily or 100 μg per m^2 IV once daily<br>•Naltograstim: 1 μg/kg SC once daily or 2μg/kg IV once daily<br>•Lenograstim: 2 μg/kg SC once daily or 5μg/kg IV once daily
Timing of discontinuation	•If the neutrophil count reaches 5000 per mm^3 or more after the course, administration should be discontinued.<br>•If the neutrophil count recovers to ≧2000 cells per mm^3, there are no suspected infections, and the patient's safety is judged to be ensured by the responsiveness to the drug used, the drug should be discontinued or reduced.

6.5. Post-study treatment

Treatment after discontinuation of protocol treatment and treatment after progression or recurrence after completion are not specified.

Treatment (cross over) with drugs included in treatment regimens in the unassigned groups may be used, but if the total dose of cisplatin is greater than 500 mg/m^2, careful attention should be given to accumulating toxicities such as peripheral sensory/motor neuropathy, hearing loss, and renal impairment. Patients should be carefully monitored with adequate risk explanation only if the benefits are apparently outweighed by the risks, such as those with persistent sensitivity to cisplatin and mild cumulative toxicity.

If primary analysis or interim analysis reveals the primary conclusions of the trial, the results of the study will be explained to patients enrolled in this study as needed, and the best treatment will be provided, taking into account the course of treatment of individual patients.

In addition, if the protocol treatment discontinuation criteria apply but clinically "protocol treatment continuation" is judged to be appropriate, consult the research office through the institutional research director or institutional coordinator rather than at the physician level as a general rule (except when time is not allowed). In agreement between the Research Secretariat and the Investigator/Institution Coordinator, decide whether to treat as a → after discontinuation of protocol treatment or to deviate and continue protocol treatment. The details of the consultation

with the Research Secretariat and the decision-making process should be provided in the comment column for the patient's end-of-treatment report and progress record. If continuing the protocol treatment with protocol deviation occurs frequently, the Study Coordinator should consider revising protocol treatment termination criteria using group meetings and group mailing lists, because it is considered that the protocol treatment termination criteria is clinically inappropriate in such situation.

7. Anticipated Adverse Events

7.1. Anticipated adverse reactions

Anticipated adverse reactions in this study are as follows:

7.1.1. Anticipated Adverse Drug Reactions with Drugs

Adverse drug reactions anticipated with protocol treatments and drugs used in protocol-specified tests are referred to the latest version of the drug package insert.

7.1.2. Anticipated adverse reactions in the standard treatment arm (arm A)

Table 7.1.2. Adverse events in the EP-therapy group at JCOG9511 (excerpt from the final analysis report)

Examination Items	Grade 0	Grade 1	Grade 2	Grade 3	Grade 4	Grade 3,4(%)	Grade4(%)	Total
White blood cells	2	5	30	35	5	51.9%	6.5%	77
Neutrophils	1	0	5	21	50	92.2%	64.9%	77
Hemoglobin	2	9	43	23	-	29.9%	-	77
Platelet	31	19	13	14	0	18.2%	0%	77
Total bilirubin	57	-	20	0	0	0%	0%	77
GOT	49	24	2	1	1	2.6%	1.3%	77
GPT	40	28	6	2	1	3.9%	1.3%	77
Creatinine	56	16	5	0	0	0%	0%	77
Oxygen tension	13	27	9	2	1	5.8%	1.9%	52
Nausea and vomiting	13	36	23	5	-	6.5%	-	77
Diarrhea	64	8	5	0	0	0%	0%	77
Oral cavity (stomatitis)	68	6	2	1	0	1.3%	0%	77
Infection	42	23	9	1	2	3.9%	2.6%	77
Hair loss (hair)	9	46	19	-	-	-	-	74
Fever (uninfected)	45	14	16	2	0	2.6%	0%	77
Perception (peripheral nerve)	66	10	1	0	-	0%	-	77
Rash	74	2	1	0	0	0%	0%	77

※: Use JCOG Toxicity Criteria.

7.1.3. Anticipated adverse reactions in the study treatment arm (Arm B)

Table 7.1.3. Adverse Events in the IP-Therapy Group in a JCOG9511 (Extracted from the Final Analysis Report)

Examination Items	Grade 0	Grade 1	Grade 2	Grade 3	Grade 4	Grade 3,4(%)	Grade 4(%)	Total
White blood cells	1	16	38	17	3	26.7%	4.0%	75
Neutrophils	1	8	17	30	19	65.3%	25.3%	75
Hemoglobin	7	10	38	20	-	26.7%	-	75
Platelet	56	6	9	1	3	5.3%	4.0%	75
Total bilirubin	59	-	16	0	0	0%	0%	75
GOT	40	30	5	0	0	0%	0%	75
GPT	35	30	7	3	0	4.0%	0%	75
Creatinine	56	15	4	0	0	0%	0%	75
Oxygen tension	13	20	5	1	1	5.0%	2.5	40
Nausea and vomiting	11	26	28	10	-	13.3%	-	75
Diarrhea	23	19	21	8	4	16.0%	5.3%	75
Oral cavity (stomatitis)	66	9	0	0	0	0%	0%	75

Infection	45	17	9	3	1	5.3%	1.3%	75
Hair loss (hair)	17	44	13	-	-	-	-	74
Fever (uninfected)	45	12	17	1	0	1.3%	0%	75
Perception (peripheral nerve)	71	4	0	0	-	0%	-	75
Rash	70	5	0	0	0	0%	0%	75

※: Use JCOG Toxicity Criteria.

Table 7.1.3. Adverse events in the IP-therapy group in b JCOG0509 (abstracted from the main analysis report)

Examination Items	Grade 0	Grade 1	Grade 2	Grade 3	Grade 4	Grade 3,4(%)	Grade 4(%)	Total
White blood cells	16	36	58	29	3	22.5	2.1	142
Neutrophils	6	10	43	51	32	58.5	22.5	142
Hemoglobin	20	24	65	24	9	23.2	6.3	142
Platelet	125	6	8	2	1	2.1	0.7	142
Total bilirubin	116	15	10	1	0	0.7	0	142
GOT	98	37	5	2	0	1.4	0	142
GPT	80	56	4	2	0	1.4	0	142
Creatinine	97	37	8	0	0	0	0	142
Anorexia	22	67	33	19	1	14.1	0.7	142
Nausea	30	67	36	9	0	6.3	0	142
Vomiting	89	38	10	5	0	3.5	0	142
Diarrhea	52	51	28	11	0	7.7	0	142
Mucositis (examination findings)-oral cavity	126	15	0	0	1	0.7	0.7	142
Hair loss	64	66	12	-	-	-	-	142
Onset of febrile neutropenia	127	-	-	14	1	10.6	0.7	142
Infection with Grade3-4 neutropenia -Bronchus	142	-	0	0	0	0	0	142
-Lung (pneumonia)	137	-	0	4	1	3.5	0.7	142
-Pharynx	142	-	0	0	0	0	0	142
-Upper respiratory tract-unclassifiable	141	-	1	0	0	0	0	142
-Bladder	142	-	0	0	0	0	0	142
-Kidney	142	-	0	0	0	0	0	142
-Urinary tract-subclassification impossible	142	-	0	0	0	0	0	142
Neuropathy: Sensory	127	13	2	0	0	0	0	142

Table 7.1.3.c Adverse events from a pilot trial of postoperative adjuvant chemotherapy with irinotecan plus cisplatin for high-grade neuroendocrine lung cancer (excerpt)

Examination Items	Grade 2	Grade 3	Grade 4	Grade 3,4(%)	Grade 4(%)	Total
White blood cells	17	7	0	17.5%	0%	40
Neutrophils	12	15	4	47.5%	10.0%	40
Hemoglobin	14	6	4	25.0%	10.0%	40
Platelet	2	0	0	0%	0%	40
Total bilirubin	0	0	0	0%	0%	40
GOT	0	0	0	0%	0%	40
GPT	1	0	0	0%	0%	40
Creatinine	0	0	0	0%	0%	40
Hyponatremia	0	5	0	12.5%	0%	40
Endotoxemia	3	1	0	2.5%	0%	40
Hypokalemia	0	4	0	10.0%	0%	40
Nausea	8	4	-	10.0%	-	40
Vomiting	4	2	0	5.0%	0%	40
Anorexia	2	0	-	0%	0%	40

Diarrhea	11	2	0	5.0%	0%	40
Fatigue	10	5	-	12.5%	0%	40
Infection	2	0	0	0%	0%	40

7.2. Anticipated Adverse Events Due to Pathogenesis

1) Esophageal primary

Esophageal bleeding; Esophageal pain; Esophageal stenosis; Esophageal obstruction; Esophageal perforation; Esophageal ulcer; Esophageal fistula; Hoarseness; Pharyngolaryngeal dysesthesia; Hypercalcemia of advanced disease; Tracheal stenosis; Tracheal obstruction; Esophageal anastomotic leakage; Recurrent laryngeal nerve palsy

2) Gastric primary

Gastric bleeding, upper gastrointestinal bleeding, gastric pain, gastrointestinal pain, back pain, nausea, vomiting, dyspepsia, gastroparesis, abdominal fullness, gastric stenosis, gastric obstruction, gastric perforation, duodenal bleeding, duodenal fistula, duodenal perforation, duodenal perforation, duodenal stenosis, gastrostomy, gastrointestinal fistula, gastrointestinal fistula, ileus, gastric anastomotic leak, gastrointestinal anastomotic leak

3) Small intestin/Colon and rectum

Bleeding from tumor; Ileus; Duodenal bleeding; Duodenal obstruction; Duodenal perforation; Duodenal stenosis; Small bowel obstruction; Small bowel stenosis; Small bowel ulcer; Small bowel perforation; Small bowel fistula; Vaginal fistula; Colonic fistula; Colonic stenosis; Colonic obstruction; Colonic perforation; Rectal stenosis; Rectal obstruction; Rectal fistula; Rectal perforation; Rectal fistula; Jejunal perforation; Jejunal perforation; Ileostomy; Ileovesical fistula; Intestinal fistula; Anal pain; Small bowel anastomotic leakage; Large bowel anastomotic leakage; Rectal anastomotic leakage; Pelvic infection

4) Pancreas, biliary tract, and liver

Body weight loss; Fever; Pancreatitis; Cholecystitis; Cholecystic obstruction; Gallbladder pain; Liver failure; Biliary tract infection; Hemorrhage from biliary tract; Duodenal hemorrhage; Duodenal fistula; Duodenal obstruction; Duodenal perforation; Duodenal stricture; Anastomotic ulcer; Biliary anastomosis leak; Pancreatic anastomosis leak; Anastomotic hemorrhage; Anastomotic stricture; Hemorrhage from tumor; Hemorrhage from portal vein stenosis/obstruction; Symptoms associated with portal vein stenosis/obstruction (Portal hypertension, Hepatic failure; Esophageal variceal hemorrhage; Gastritis; Ascites); Back pain; Abdominal pain

5) Anticipated Adverse Events Due to Metastasis

Liver failure, hepatic pain, fever, hepatic infection, respiratory failure, pulmonary infection, superior vena cava syndrome, abdominal distention, abdominal distention, ileus, esophageal obstruction, reverse smoking, gastric obstruction, duodenal obstruction, small bowel obstruction, colonic obstruction, rectal obstruction, rectal stenosis, anal bleeding, anorectal infection, nausea, diarrhea, constipation, ureteral obstruction, and urinary retention,urethral infection, urinary tract infection, bile duct obstruction, biliary obstruction, cholecystitis, biliary hemorrhage, gallbladder pain, pancreatitis, pain, narrowing of luminal organs near metastatic sites (tracheal/gastrointestinal tract, etc.), tracheal obstruction, bronchial stenosis, penetration with adjacent organs, perforation, hoarseness, pleural effusion, chest wall pain, pleural pain, atelectasis, hypercalcemia, disseminated intravascular coagulation, fracture, ataxia, cerebrovascular ischemia, intracranial hemorrhage,headache, dizziness, decreased level of consciousness, aphasia, seizures, spasticity

6) Paraneoplastic syndrome, etc.

(due to incompatible secretory syndrome) hyponatremia, (due to ectopic ACTH syndrome) personality changes, hypertension, hypokalemia, hyperglycemia, Lambert-Eaton myasthenic syndrome, subacute cerebellar degeneration associated with autoantibody production (including ataxia, dysarthria, and nystagmus of limbs), paraneoplastic encephalomyelitis, sensory neuropathy (including dementia, cranial nerve symptoms, dizziness, ataxia, autonomic ataxia, transverse paralysis, and sensory disturbance), thromboembolism, (due to gastrointestinal stenosis) aspiration, anaemia, tumor pain, acute renal failure, myositis, and pulmonary fibrosis

Complications associated with etiolation and gastrointestinal stent insertion are shown below.

- Percutaneous transhepatic cholangiodrainage (PTCD) procedures, including internal-external fistula tube placement:

  Pancreatitis, bile duct stricture, cholecystitis, gallbladder obstruction, gallbladder pain, liver failure, biliary

tract infection, liver infection, cholecystitis, septicemia, hemorrhage, PTCD tubing obstruction/deviation, peritoneal infection, pneumothorax, pleural pain, pleural effusion, intrapleural hemorrhage

- Biliary stenting:
  Pancreatitis, biliary stricture, cholecystitis, gallbladder obstruction, gallbladder pain, liver failure, biliary tract infection, liver infection, cholecystitis, sepsis, hemorrhage, peritoneum infection, pneumothorax, pleural pain, pleural effusion, intrapleural hemorrhage (in case of percutaneous placement), duodenal perforation, pneumonitis (in case of transendoscopic placement), stent obstruction/deviation, duodenal ulcer, and duodenal hemorrhage
- Choledochojejunostomy:
  Biliary tract infection; Pancreatitis; Cholecystitis; Liver infection; Sepsis
- Gastrointestinal stent insertion:
  Bleeding, perforation, pain, stent deviation, stent obstruction, ulceration, fever, sepsis, infection, diarrhea, constipation, urgency (colorectal), thyroid injury (esophagus), jugular arteriovenous injury (esophagus), and mediastinal abscess (esophagus).

7.3. Evaluation of Adverse Events/Reactions

The Common Terminology Criteria for Adverse Events v4.0 Japanese Translated JCOG Version (Japanese translation of NCI-Common Terminology Criteria for Adverse Events v4.0 (CTCAE v4.0)) (CTCAE v4.0-JCOG) will be used to assess adverse events/reactions. For CTCAE v4 0-JCOG in which Grade is defined by laboratory reference values at the institutional reference value, the "JCOG sharing reference range" will be used instead of the institutional reference value at each medical institution. For more information on JCOG sharing reference ranges, see JCOG website (see http://www.jcog.jp/doctor/tool/kijun.html)).

7.3.1. Grading of s adverse events

In grading of adverse events, each grading is closest to the definitions of Grade 0 4 (nearest match). Grading to a higher Grade when the definition of more than one Grade is comparable and when it is difficult to decide on either (highest grade).

Grading should also be given to Grade if specific actions are described, due to their clinical need. For example, patients may refuse oxygen inhalation or chest drainage, even when the patient's pleural effusion is increasing and oxygen inhalation or chest drainage is indicated. In such cases, grading is based on the medical judgment of what should have been done (what should be done) rather than on whether the treatment was actually given (what was actually done).

In the event of treatment-related deaths, original NCI-CTCAE states that the causative adverse event should be Grade 5, but the outcome of the serious adverse event is reported in the SAE report and reviewed in detail. Therefore, Grade 5 of the institutional physician's judgment is not likely to be changed, and whether or not the serious adverse event will result in death will be significantly affected by other factors than the event is not appropriate, so it is not appropriate to compare the frequency of the adverse event by Grade (%Grade 4 and%Grade 5, respectively) between treatment groups or between studies. Because of the poor significance of distinguishing between Grade 4 and Grade 5 in the tabulation, Grade 4 is not considered "Grade 5" in the recording form of this study. A discussion of the causal relationship between adverse events observed in treatment-related deaths and deaths should be included in the "Situation at Death" section of the treatment completion report form and follow-up form, and an urgent report should be made. For the adverse event items specified in "8.2. Testing and Assessment during Treatment" and "8.3. Testing and Endpoints after Treatment Completion" that are determined to be Grade 5 in the post-hoc review including the emergency report, Grade and the date of the first occurrence of the event should be included in the relevant record form (Treatment Course Record Form). If Grade 3 or greater is observed for any other adverse event, or if Grade 3/2/1 adverse event and treatment requires at least 24 hours of hospital stay or prolongation of hospital stay (see 10.1.1.3.)), the AE and Grade and the date of first occurrence should be included in the free form of the treatment course record.

Any Grade on the record form should be recorded in the medical record. Confirmed during site visit audit.

7.3.2. Determination of the causal relationship between adverse events and treatment

In determining the causal relationship between adverse events and treatment, patients are classified into 5 categories of "definite, probable, possible, unlikely, unrelated". Each "causality" is defined as "causality" when judged to be either "definite, probable, possible" and "no causality" when judged to be either "unlikely, unrelated" (see TABLE 7.3.2.

According to Grade of adverse events, serious adverse events requiring expedited reporting as specified in "10.1. Serious Adverse Events and Expedited Reporting" should be reported to the Research Secretariat in accordance with "10.2. Mandatory Reporting and Reporting Procedures of the Investigator".

Table 7.3.2. Criteria for a Causal Relationship Between Adverse Events and Treatment

	Determination	Approach to determination
Cause Fruit Concern Associate Ah Fistula	Definite	The AE is clearly related to the intervention<br>Adverse events are apparently caused/aggravated by protocol treatment and are unlikely to be due to exacerbation of the etiology or other factors (comorbidities, other medications/treatments, or incidents).
	Probable	The AE is likely related to the intervention<br>It is unlikely that the adverse event was caused/aggravated by progression of the underlying pathology or other factors (comorbidity, other drugs/treatments, incident) and is likely to be due to protocol treatment.
	Possible	The AE may be related to the intervention<br>It is plausible (plausible) that an adverse event is considered to have occurred/become more severe with protocol treatment, and unlikely to be due to exacerbation of the etiology or other factors (comorbidities, other medications/treatments, or incidents).
Cause Fruit Concern Associate In Pouch	Unlikely	The AE is doubtfully related to the intervention<br>It is considered plausible (plausible) that the adverse event is due to exacerbation of the pathogenic disease or other factors (comorbidity, other drugs/treatments, incident) rather than to the protocol treatment that it is caused/aggravated.
	Unrelated	The AE is clearly NOT related to the intervention<br>It is judged that the adverse event was caused/aggravated by aggravation of the pathogenic disease or other factors (comorbidity, other drugs/treatments, incident) and is unlikely to be caused by protocol treatment.

8. Examination and Evaluation

8.1. Baseline examination and evaluation before registration

8.1.1. Test conducted before registration (regardless of time before registration)

1） Histopathology (immunostaining requires chromogranin A and synaptophysin) (see Section 3.3)
2） HBs antigen; HBc antibody ※1; HBs antibody ※1, HBV-DNA※2

※1: For HBsAg positive, HBc and HBs antibodies are not required, and HBV-DNA, HBeAg, and HBe antibodies are measured.

※2: Positive results for at least one of the HBc and HBs antibodies also indicate HBV-DNA prior to initiation of therapy (see Section 6.4.1).

8.1.2. Test performed within 56 days before enrollment (liver primary (or unknown primary))

If the liver is primary (or of unknown primary), the following tests should be performed (all allowing for tests performed in other hospitals):

1）Upper gastrointestinal endoscope
2）Lower gastrointestinal endoscope
3）FDG-PET test
4）Otolaryngology (head and neck) examination
5）Urology consultation (male only)
6）Gynecologic exam (female only)

8.1.3. Tests performed within 28 days before enrollment

1) Contrast-enhanced CT※1 (slice thickness of 5 mm or less, if the patient is allergic to iodine), both contrast-enhanced MRI of the abdomen and plain computed tomography (CT) of the imaging range, which is considered to be indispensable below, are performed. All tests are not performed in other hospitals.

※The following areas are indispensable for each primary organ, and if there is another site suspected of metastasis, the radiographic extent is added accordingly.

Primary Organ	Essential radiographic area
Esophagus	Cervical, chest, or abdominal regions
Stomach, duodenum, small intestine, colon, appendix, rectum,<br>Extrahepatic bile duct, Vater ampulla, gallbladder, and pancreas	Chest, abdomen, and pelvis
Hepatic NEC<br>(liver primary or liver metastasis of unknown primary)	Neck, chest, abdomen, and pelvis

2) Endoscopic ※2

Primary Organ	Mandatory test ranges
Esophagus, stomach, duodenum, and ampulla of Vater	Upper gastrointestinal tract (no examination performed at another hospital)
Small intestine, extrahepatic bile ducts, gallbladder, and pancreas	Be not mandatory
Colon, appendix, and rectum	Lower gastrointestinal tract (no examination performed at another hospital)
Hepatic NEC<br>(liver primary or liver metastasis of unknown primary)	8.1.2. Refer to the test performed within 56 days before registration.

※2 Unnecessary if the primary lesion has been resected

3) 12-lead, resting electrocardiography

8.1.4. Tests performed within 14 days before enrollment

1) General condition: PS (ECOG), body weight
2) Physical findings
3) Peripheral blood count: white blood cell count, neutrophil count (ANC: rod + segmented karyocyte), hemoglobin, platelet count

4) Blood biochemistry: total protein, albumin, total bilirubin, AST (GOT), ALT (GPT), BUN, creatinine, LDH, ALP, sodium, potassium, calcium, magnesium, CRP, FBS (fasting blood glucose)
5) Creatinine clearance (CCr): CCr estimates by Cockcroft-Gault equation
Cockcroft-Gault formula
Male: Ccr = {(140-Age) × Body Weight (kg)}/{72 × Serum Creatinine Level (mg/dL)}
Women: Ccr = 0.85 × {(140-Age) × Body Weight (kg)}/{72 × Serum Creatinine Level (mg/dL)}
6) Tumour markers: NSEs, ProGRP, CEAs, SCCs (esophagus primary only), CA19-9 (non-esophageal primary only)
7) Chest X-P (frontal) (substitutable if contrast-enhanced and plain chest CT is obtained)

8.2. Examination and evaluation during treatment

The following safety examination and evaluation are minimal in frequency: Performing examinations more frequently at the discretion of the treating physician is not prohibited.

However, the examination for efficacy evaluation should be performed at specified frequencies, unless progression is suspected, because dense frequency may lead to bias in the efficacy evaluation.

8.2.1. Safety endpoint assessed weekly (CTCAE v4 0 Japanese translation)

The following 1)-3) tests or evaluations should be performed at least weekly. In addition, all of the following items should be checked and evaluated on the scheduled date or the day before anticancer drug administration. However, at least weekly examinations or evaluations should be performed in the same manner until day 28 using the starting date of the last course as day 1, even when the treatment is completed or terminated.

1） Peripheral blood count: white blood cell count, neutrophil count (rod + segmented count), hemoglobin, and platelet count
2） Biochemical tests: albumin, total bilirubin, AST (GOT), ALT (GPT), creatinine, sodium, potassium, calcium, magnesium, CRP
3） Subjective and objective findings (described according to CTCAE v4.0JAPANESE TRANSLATION)
- General disorders and administration site conditions: fever, fatigue
- Ear and labyrinth disorders: Tinnitus, hearing impairment
- Skin and subcutaneous tissue disorders: alopecia
- Gastrointestinal disorders: Constipation, diarrhea, nausea, vomiting, oral mucositis
- Metabolism and nutrition disorders: anorexia, dehydration
- Nervous system disorders: dysgeusia, peripheral sensory neuropathy, peripheral motor neuropathy
- Musculoskeletal and connective tissue disorders: myalgia, arthralgia
- Infections and parasites: bronchial infection, pulmonary infection, upper respiratory tract infection, mediastinal infection, pleural infection, catheter-related infection, biliary tract infection, gallbladder infection, and urinary tract infection
- Blood and lymphatic system disorders: Febrile Neutropenia
- Vascular disorders: Phlebitis
- Respiratory, thoracic and mediastinal disorders: dyspnea, hypoxia, pneumonitis

8.2.2. Safety examination and evaluation for each course

1) General condition: Body weight
2) Blood chemistry: LDH, ALP FBS (fasting glucose)

8.2.3. Safety examination and evaluation to be performed as necessary

1） When dyspnea is observed
- Chest X-P, percutaneous oxygen saturation: SpO2, arterial blood gases: PaO_2

2） If an arrhythmia is observed
- 12-lead, resting electrocardiography

3） When HBs antigen, HBs antibody, or HBc antibody is positive
- HBV-DNA (see 6.4.1.).

8.2.4. Efficacy end point

The following tests will be performed every 6 weeks during protocol treatment (±1 week allowed: 6 ± 1 week, 12 ± 1 week, and 18 ± 1 week after the start date of protocol treatment). Tumor response will be assessed according to "11.1. Response Evaluation". Evaluation of the response will be performed using the same test conditions and test methods as the baseline evaluation.

If CT is allergic to contrast material, it is evaluated with plain CT and/or contrast-enhanced MRI. Allergy to contrast media on MRI is assessed by plain CT or plain MRI. When the use of CT contrast medium and MRI contrast medium becomes difficult due to renal dysfunction, it is evaluated by simple CT or simple MRI.

1） Tumour markers: NSEs, ProGRP, CEAs (≧all cases), SCCs (esophagus primary only), CA19-9 (non-esophageal primary only)

2） Contrast-enhanced CT: In principle, the extent of imaging is as follows. () Areas in can be omitted if there is no lesion

Primary Organ	Essential radiographic area
Esophagus	Cervical, chest, or abdominal regions
Stomach, duodenum, small intestine, colon, appendix, rectum,<br>Extrahepatic bile duct, Vater ampulla, gallbladder, and pancreas	(chest), abdomen, and pelvis
Liver NEC (liver primary or liver metastasis of unknown primary)	(neck), (chest), abdomen, and pelvis

8.3. Examination and evaluation after completion of treatment

8.3.1. Efficacy evaluation after completion of treatment

After completion of the protocol treatment, examination and evaluation are made at the following timing:

The following tests should be performed at least every 6 weeks until progressions are confirmed or death.

If progressions are observed, only observation of the outcome will be continued.

1） Tumour markers: NSEs, ProGRP, CEAs (≧all cases), SCCs (esophagus primary only), CA19-9 (non-esophageal primary only)

2） Contrast-enhanced CT: In principle, the extent of imaging is as follows. Areas in parentheses can be omitted if there is no lesion

Primary Organ	Essential radiographic area
Esophagus	Cervical, chest, or abdominal regions
Stomach, duodenum, small intestine, colon, appendix, rectum,<br>Extrahepatic bile duct, Vater ampulla, gallbladder, and pancreas	(chest), abdomen, and pelvis
Liver NEC (liver primary or liver metastasis of unknown primary)	(neck), (chest), abdomen, and pelvis

8.4. Information on post- study treatment

After completion/termination of protocol treatment, the following items will be recorded on the Follow-up Form at each follow-up survey:

1） Content of post-study treatment (if post-study treatment is performed)

2） After protocol treatment termination, the initiation date of the first post-study treatment ((if post-study treatment is performed)

3） PS at the start of post-study treatment

8.5. Study calendar

If the primary site is the esophagus, stomach, duodenum, small intestine, colon, appendix, rectum, extrahepatic bile duct, ampulla of Vater, gallbladder, or pancreas

	Before registratikon Record Pre	After end of chemotherapy		From the date of protocol treatment discontinuation Within 28 days	Until progression after completion of protocol treatment	
		Course Before initiation of the course	During the course		Only at the start of post-treatment	Every 6 weeks
Physical findings						
Body weight	○[14]	○				
PS	○[14]				■	
Physical findings	○[14]	○			■	
Laboratory tests						
WBC, differential (neutrophil) Hb, platelets	○[14]	○	●	●		
Alb, T-Bil, AST, ALT, Cr, Na, K, Ca, Mg, CRP	○[14]	○	●	●		
LDH, ALP, FBS	○[14]	○				
Total protein, BUN, Ca	○[14]					
NSE, ProGRP、CEA	○[14]		△			
SCC (esophagus primary only), CA19-9 (other than primary esophageal)	○[14]		△			
HBs antigen, HBc antibody, and HBs antibody	○[前]					
Chest X-P (can be substituted if CT is taken)	○[14]					
12-lead, resting electrocardiography	○[28]					
Upper gastrointestinal endoscope (Primary: esophagus, stomach, duodenum)	○[28]					
Lower gastrointestinal endoscope (Primary: colonic, appendix, rectum)	○[28]					
Efficacy evaluation						
Contrast-Enhanced CT*	○[28]		△			△
Toxicity evaluation						
Subjective symptom check		○	●	●		
Objective symptom check		○	●	●		
Submission of CRFs						
Pre-treatment Form		□				
Treatment Form			□			
Off-treatment Form				□		
Follow-up Form					2 times/year	

○[前]: Conduct before registration, ○[28]: Perform within 28 days before registration, ○[14]: Perform within 14 days before registration

○:Conduct, ♀: Implementation at least once a week

△: Every 6 weeks (see 8.2.4.), ∞: Only once at the beginning of aftertreatment, □: Submitted.

*See 8.1.3 for the shooting range. Contrast-induced allergy or renal dysfunction is assessed by plain CT or contrast-

enhanced MRI.

※Follow-up Forms will be sent up to 1 years after completion of accrual and will be submitted after 1 years of registration in the individual patient according to the closing date of registration.

For liver NEC (liver primary or liver metastasis of unknown primary)

	Before registratikon Record Pre	After end of chemotherapy		From the date of protocol treatment discontinuation Within 28 days	Until progression after completion of protocol treatment	
		Course Before initiation of the course	During the course		Only at the start of post-treatment	Every 6 weeks
Physical findings						
Body weight	○[14]	○				
PS	○[14]				■	
Physical findings	○[14]	○			■	
Laboratory tests						
WBC, differential (neutrophil) Hb, platelets	○[14]	○	●	●		
Alb, T-Bil, AST, ALT, Cr, Na,K, Ca, Mg, CRP	○[14]	○	●	●		
LDH, ALP, FBS	○[14]	○				
Total protein, BUN, Ca	○[14]					
NSE, ProGRP、CEA、CA19-9	○[14]		△			
HBs antigen, HBc antibody, and HBs antibody	○[前]					
Chest X-P (can be substituted if CT is taken)	○[14]					
12-lead, resting electrocardiography	○[28]					
Upper gastrointestinal endoscope	○[56]					
Lower gastrointestinal endoscope	○[56]					
FDG-PET	○[56]					
Otolaryngologic examination	○[56]					
Urology consultation (male only)	○[56]					
Gynecologic exam (female only)	○[56]					
Efficacy evaluation						
Contrast-Enhanced CT*	○[28]		△			△
Toxicity evaluation						
Subjective symptom check		○	●	●		
Objective symptom check		○	●	●		
Submission of CRFs						
Pre-treatment Form		□				
Treatment Form			□			
Off-treatment Form				□		
Follow-up Form					□ 2 times/year	

○[前]: Conduct before registration, ○[56]: Perform within 56 days before registration, ○[28]: Perform within 28 days before registration,

○[14]: Implemented within 14 days prior to enrollment

○:Conduct, ♀: Implementation at least once a week

△: Every 6 weeks (see 8.2.4.), ∞: Only once at the commencement of post-treatment, □: Submitted.

*See 8.1.3 for the shooting range. Contrast-induced allergy and renal dysfunction are evaluated by plain CT or contrast-enhanced MRI.

※Follow-up Forms will be sent up to 1 years after completion of accrual and will be submitted after 1 years of

registration in the individual patient according to the closing date of registration.

9. Data collection

9.1. Case Report Form (CRF)

9.1.1. Types of CRF and submission deadlines

The case report forms (CRF) used in this study and their submission deadlines are as follows:

1) Pre-treatment report (blue)- Less than 2 weeks after enrollment
2) Treatment course records- Every 2 cycles/less than 2 weeks after end of protocol treatment
   3)-1 Treatment (yellow)
   3)-2 Test (yellow)
   3)-3 Adverse events (yellow)
3) Tumor shrinkage report (green) - Less than 2 weeks after judgment of effect
4) End of treatment report (red) - Less than 2 weeks after discontinuation/end of protocol treatment
5) Follow-up investigation (white)- By the deadline indicated in the Follow-up Investigation Form

- For "1) Pre-treatment report to 4) End of treatment report", CRFs with basic patient information (enrollment code, facility name) pre-printed on them would be sent by post from the data center. If the CRFs do not arrive within one week of enrollment, or if the CRFs have been lost/damaged, the data center should be contacted by telephone, and a request should be made for them to be re-issued.
- "5) Follow-up investigation" would be sent by post from the data center at the time of follow-up investigations that are conducted at the same time as monitoring and interim/final analyses at the data center.

9.1.2. Storage CRF

- Completed CRFs must all be archived at the facility as photocopies or in electronic form.
- Copies of CRFs should be kept archived until the final analysis report is issued for reference while filling other CRFs, or for review while retrieving information from the data center.

9.1.3. Method of sending CRF

- All CRF must be sent by post or handed over in person at the data center. They must not be sent by FAX.
- To avoid the risk of personal patient information being leaked, the patient enrollment code should be used, instead of using the patient medical chart number at the facility, when contacting the data center for request of CRF dispatch.

9.1.4. Correction of the contents of CRFs

If any data necessary for the CRF are found to be missing or there are inappropriate category classifications after the start of the study, the CRFs may be corrected with the agreement of the head of the data center and the clinical trial secretariat, in a manner not exceeding the scope of data collected as prescribed in "8. Evaluation item/Clinical laboratory tests/Evaluation schedule", and within the scope deemed not to increase medical and financial burden on the enrolled patient from the CRF correction. Modification of CRFs that do not require the main body of the protocol to be revised is not considered a protocol revision by JCOG. Reports to the head of the medical institution related to CRF correction and the request of application for revision should follow the rules of the facility.

10. Reporting of "disease or the like"(adverse events)

Site investigator should report to Study Coordinator/Principal Investigator (Study Chair) if a serious adverse event ("disease or the like" on Clinical Trials Act) occurs in accordance with the regulations of Clinical Trials Act (Law No. 16, 2017), Enforcement Regulations of Clinical Trials Act (MHLW Notification No. 17, 2018) and the regulations in this chapter based on the relevant notifications.

The most recent version of the report is available on the MHLW website 1) and on the JCOG website 2). Use the most recent version of the report.

Serious adverse events occurring after the initiation of protocol treatment (after the date of registration if death) by the date of final follow-up will be subjects.

1) http://www.mhlw.go.jp/stf/seisakunitsuite/bunya/0000163417.html
2) http://www.jcog.jp/doctor/todo/researcher/harmfulness.html
3) http://www.pmda.go.jp/safety/reports/hcp/pmd-act/0002.html

10.1. Serious Adverse Events

Serious adverse events are defined as any of the following:
(These are classified as "disease or the like" on Clinical Trials Act.)

1) Death
2) Diseases that may lead to death
3) "Disease or the like" requiring hospitalization or prolongation of hospital stay for treatment.
4) Disability
5) "Disease or the like" that may lead to disability
6) Serious "disease or the like" according to 1) to 5)
7) Congenital disease or abnormality in later generations

1) Death

(i) All deaths that occur after registration and before the start of protocol treatment
(ii) All deaths (with or without causality to protocol treatment) that occur during protocol treatment or within 30 days of the last treatment day
(iii) Death that occur after 31 days from the last treatment date that are causally related to protocol treatment (definite, probable, possible)

2) "Disease or the like" that may lead to death

(i) Grade 4 adverse events that occur during protocol treatment or within 30 days of the last treatment day (excluding events in Table 10.1)
(ii) Grade 4 adverse events that occur after 31 days from the last treatment date (excluding events in Table 10.1) that are causally related to the protocol treatment (definite, probable, possible)

3) Hospitalization or prolongation of hospital※1 stay for treatment

(i) Grade 3/2/1 adverse events that occur during or within 30 days of protocol treatment and requiring at least 24 hours of hospitalization or prolongation of hospital stay※1 to treat the adverse event (excluding the event in Table 10.1).
(ii) Grade 3/2/1 adverse events that occur 31 days after the last treatment day and requires 24-hour or longer hospitalization or prolongation of hospital stay※1 for treatment and causally related to protocol treatment (definite, probable, possible) (excluding the events in Table 10.1)

※1 "Hospitalization or prolongation of hospital stay" refers only to those for which hospitalization of at least 24 hours or prolongation of hospital stay is medically required for the treatment of an adverse event. The followings are not subjects for reporting:

- Hospitalization or prolongation of hospital stay performed for follow-up of adverse event that has disappeared or improved
- Hospitalization or prolongation of hospital stay for reducing patient burden, e.g. patients from distant areas.

- Hospitalization or prolongation of hospital stay for other medically unnecessary situation

4) Disability, 5) Disease that may lead to disability

Permanent or marked disability/dysfunction (excluding myelodysplastic syndromes, secondary cancers, etc.) or possible medical situation

6) Serious disease similar to 1) to 5)

7) Congenital disorders or abnormalities in later generations

Table 10.1. Adverse events excluded from the subjects of Expedited Reporting

SOC※(CTCAE ver4.0)	AE term
Blood and lymphocyte disorders	Anemia, bone marrow hypocellular
Gastrointestinal disorders	Constipation
General disorders and local symptoms	Fever
Infections and infestations	Viral hepatitis
Clinical laboratory test	ALP increased, CD4 lymphocytes decreased, high cholesterol, GGT increased, lipase increased, lymphocytes decreased, neutrophils decreased, platelet count decreased, serum amylase increased, WBC decreased, hyponatremia, hypokalemia, hyperglycemia, and hypoglycemia
Metabolism and nutritional disorders	Obesity, anorexia, hyperuricemia, and hypoalbuminemia
Musculoskeletal and connective tissue disorders	Fibrosis deep connective tissue and superficial soft tissue fibrosis
Renal and urinary disorders	Chronic kidney disease
Respiratory, thoracic, and mediastinal disorders	Sinus disorder and sleep apnea
Skin and subcutaneous tissue disorders	Hypohidrosis

※ SOC:System Organ Class

10.2. Investigator's reporting requirements and procedures

10.2.1. Expedited Reporting

In the event of a serious adverse event, the Subinvestigator must promptly inform the Investigator. If the Investigator cannot be contacted, the Site Coordinator or Subinvestigator must take over the responsibility of the Investigator. The Investigator must report adverse events according to the following procedures.

Attention should be paid not to include the patient's name and medical record number when sent.

Serious adverse events that occur after the initiation of protocol treatment (after the date of registration if death) by the date of final follow-up are subjects of Expedited Reporting.

1) "Disease or the like" that may lead to death or death specified in 10.1 1) and 2).

Primary reporting:

The Subinvestigator who is aware of the occurrence of adverse events will promptly notify the Investigator. The Investigator who receives the notice should fill out JCOG Adverse Event Report Form(for institution) and the "Disease or the like" Report Form to Certified Review Board specified in Clinical Trials Act Enforcement Regulations as far as possible and contact Principal Investigator/Study Coordinator via e-mail within 72 hours of knowledge of the occurrence of the adverse event.

Secondary reporting:

The Investigator should add detailed information on adverse events to JCOG Adverse Event Report Form(for institution) and the "Disease or the like" Report Form to Certified Review Board specified in Clinical Trials Act Enforcement Regulations within 7 days of knowledge of the occurrence of adverse events and send them to the Principal Investigator/Study Coordinator via e-mail. If necessary, attach copies of laboratory data, images, autopsy report, etc.

2) 10.1. 3) Disease or other medically important condition requiring hospitalization or prolongation of hospital stay for treatment. Adverse events are judged to be either of 10.1. 4)-7)

The Subinvestigator who is aware of the occurrence of adverse events will promptly notify the Investigator. The Investigator who receives notice must fill out JCOG Adverse Event Report Form (for institution) and the "Disease or the like" Report Form to Certified Review Board specified in Clinical Trials Act Enforcement Regulations within 10 days of knowledge of the occurrence of an adverse event and send them to the Principal Investigator/Study Coordinator via e-mail. If necessary, attach copies of laboratory data, images, autopsy report, etc.

3) Additional reporting

If new information is obtained after conducting the above reporting, the Investigator must add information to JCOG Adverse Event Report Form (for institution) and the "Disease or the like" Report Form to Certified Review Board specified in Clinical Trials Act Enforcement Regulations and report it as needed.

Table 10.2.1. Summary of Adverse Events which are subjects for Expedited Reporting and the deadline of reporting to Principal Investigator/Study Coordinator

Causal relationship	The patient was hospitalized due to Grade 1-3 AE. Other medically important conditions		Grade 4		Death	
	Expected	Not expected	Expected	Not expected	Expected	Not expected
Present	Primary reporting: within 10 days Additional reporting: as needed	Primary reporting: within 10 days Additional reporting: as needed	Primary reporting: within 72 hours Secondary reporting: within 7 days Additional reporting: as needed			
None	<Only on-treatment or within 30 days of last protocol treatment day>					
	Primary reporting: within 10 days Additional reporting: as needed	Primary reporting: within 10 days Additional reporting: as needed	Primary reporting: within 72 hours Secondary reporting: within 7 days Additional reporting: as needed			

* 4) Disability, 5) "Disease or the like" that may lead to disability, and 6) "Disease or the like" that are serious similar to 1) to 5) in 10.1., 7) Congenital disorders or abnormalities in later generations

※ "Unexpected" refers to those not listed in "7. Expected Adverse Events"

Table 10.2.1. Summary of Adverse Events which are subjects for Expedited Reporting and the deadline of reporting to Principal Investigator/Study Coordinator

Causal relationship	The patient was hospitalized due to Grade 1-3 AE. *Other medically important conditions		Grade 4		Death	
	Expected	Not expected	Expected	Not expected	Expected	Not expected
Present	Primary reporting: within 10 days Additional reporting: as needed	Primary reporting: within 10 days Additional reporting: as needed	Primary reporting: within 72 hours Secondary reporting: within 7 days Additional reporting: as needed			
None	<Only on-treatment or within 30 days of last protocol treatment day>					
	Primary reporting: within 10 days Additional reporting: as needed	Primary reporting: within 10 days Additional reporting: as needed	Primary reporting: within 72 hours Secondary reporting: within 7 days Additional reporting: as needed			

* 4) Disability, 5) "Disease or the like" that may lead to disability, and 6) "Disease or the like" that are serious similar to 1) to 5) in 10.1., 7) Congenital disorders or abnormalities in later generations

※ "Unexpected" refers to those not listed in "7. Expected Adverse Events"

10.2.2. Reporting to the Administrator of participating medical organizations

If an Adverse Event which is subjects for Expedited Reporting occurs and is assessed as causal after reporting to Principal Investigator and reported to Certified Review Board, the Investigator must report it to the Administrator of the relevant medical institution in accordance with the requirements of the medical institution.

10.3. Responsibilities of Principal Investigator/Study Coordinator

10.3.1. Determination of necessity of suspension of registration and emergency notification to institutions

Principal Investigator/Study Coordinator who received the report from the Site Investigator should report to Group Chair and determine the urgency, significance, and impact of the report. If needed, take measures such as suspending registration (contacting JCOG Data Center and all participating institutions) and urgently communicating information to participating institutions. Telephone calls can be made to Data Center and institutions as urgent, but they should also be promptly contacted by document (e-mail).

10.3.2. Reporting to JCOG Operations Office and Certified Review Board and MHLW

1) Reporting from the Principal Investigator/Study Coordinator to JCOG Operations Office

Principal Investigator/Study Coordinator should consult with Group Chair and report to JCOG Operations Office (Safety Contact) by e-mail within 72 hours of knowledge of the occurrence of the adverse event, if reported AE is considered to meet the adverse events specified in 10.1.1 1)~7). In doing so, to the extent feasible, Principal Investigator/Study Coordinator should send "JCOG Adverse Event Report Form (for institution)" sent from the institution, "Disease or the like Report Form" addressed to Certified Review Board as stipulated in Clinical Trials Act Enforcement Regulations, and attach "JCOG Adverse Event Report (for Study Coordinator)" with Study Coordinator/Principal Investigator's view (including judgments of causality and expectation, and judgments of continuation/discontinuation of the study)". For the expected adverse events of 10.1.1 1)~7)., include a discussion not only of the individual patient's course but also of whether the frequency of appearance is within the expected range.

2) Reporting to Certified Review Board

JCOG Operations Office (Safety Contact) reviews the appropriateness of the judgement of causality and expectation of adverse events reported in the above procedures and can ask Principal Investigator/Study Coordinator to reconsider them if there is any doubt. Adverse events considered by Principal Investigator/Study Coordinator and JCOG Operations Office to be related to the protocol treatment and to be the subject of reporting in the following tables should reported to Certified Review Board through JCOG Operations Office.

If there is a disagreement between Principal Investigator/Study Coordinator and JCOG Operations Office, report it to Data and Safety Monitoring Committee and seek final judgment from the Chair of Data and Safety Monitoring Committee. However, if the reporting may exceed deadlines of reporting, the report can be tentatively reported as "causal" to Certified Review Board.

Reporting subjects and reporting deadline

Principal Investigator/Study Coordinator must report to Certified Review Board through JCOG Operations Office within the following time periods after knowledge of the occurrence of adverse events.

Causal relation ship	The patient was hospitalized due to Grade 1-3 AE. Other medically important condition		Grade 4		Death	
	Expected	Not expected	Expected	Not expected	Expected	Not expected
Present	No need of reporting	Within 15 days	Within 15 days	Within 7 days	Within 15 days	Within 7 days
None	No need of reporting	No need of reporting	No need of reporting	No need of reporting	No need of reporting	No need of reporting

※ "Unexpected" refers to those not listed in "7. Expected Adverse Events"

Principal Investigator/Study Coordinator must report adverse events to Certified Review Board through JCOG

Operations Office within the following deadlines after knowledge of the occurrence of adverse events.

Causal relation ship	The patient was hospitalized due to Grade 1-3 AE. Other medically important condition		Grade 4		Death	
	Expected	Not expected	Expected	Not expected	Expected	Not expected
Present	Within 30 days	Within 15 days	Within 30 days	Within 15 days	Within 15 days	Within 15 days
None	No need of reporting	No need of reporting	No need of reporting	No need of reporting	No need of reporting	No need of reporting

※ "Unexpected" refers to those not listed in "7. Expected Adverse Events"

3) Reporting to MHLW

Principal Investigator/Study Coordinator of the study with unapproved or off-label health care should report "Disease or the like" Report Form specified in Clinical Trials Act Enforcement Regulations to MHLW through JCOG Operations Office, if an adverse event is considered to be unexpected and to have a causal relationship to the protocol treatment (See 10.5. Responsibilities of the Data and Safety Monitoring Committee).

Reporting subjects and reporting deadlines

Principal Investigator/Study Coordinator should report to the MHLW through JCOG Operations Office within the following deadlines after knowledge of the occurrence of adverse events.

※ Pharmaceuticals and Medical Devices Agency Safety Division I (trk-shippeitouhokoku@pmda.go.jp)

Causal relation ship	The patient was hospitalized due to Grade 1-3 AE. Other medically important condition		Grade 4		Death	
	Expected	Not expected	Expected	Not expected	Expected	Not expected
Present	No need of reporting	Within 15 days	No need of reporting	Within 7 days	No need of reporting	Within 7 days
None	No need of reporting	No need of reporting	No need of reporting	No need of reporting	No need of reporting	No need of reporting

※ "Unexpected" refers to those not listed in "7. Expected Adverse Events"

4) Additional reporting

Following receipt of secondary or additional reports from the Investigator, Principal Investigator/Study Coordinator must add additional information from the primary report and their views to JCOG Adverse Event Report Form (for the institution) and the "Disease or the like" Report Form to Certified Review Board set out in Clinical Trials Act Enforcement Regulations, and promptly contact JCOG Operations Office (Safety Contact) by e-mail. If the report was sent to Certified Review Board and the MHLW in the primary reporting,, the secondary reporting and additional reporting must be made in the same manner.

10.3.3. Notification to the Site Investigators

When reported to Certified Review Board, Principal Investigator/Study Coordinator should inform the Investigators of all participating institutions of the review results and recommendations by documents (e-mail is allowed). Principal Investigator/Study Coordinator must inform the Investigators without waiting for Certified Review Board review if there is any urgent information to be disseminated. In the event that reported to the MHLW, Principal Investigator/Study Coordinator should notify the Investigators of all participating institutions.

In addition, even if no reporting is made to Certified Review Board, Principal Investigator/Study Coordinator must inform the Investigator of the reporting institution of the decision of Principal Investigator/Study Coordinator by documents (e-mail is allowed).

10.3.4. Assessment of Adverse Events in Periodic Monitoring

During Periodic Monitoring, Principal investigator/Study Coordinator should carefully review the adverse events

in the Monitoring Reports issued by the Data Center and ensure that there are no missed reporting from the participating institutions. It should also be confirmed that all reported adverse events are listed in the Monitoring Reports. The presence or absence of a missed reporting should be indicated in the column of the results of Group review on the Periodic Monitoring Report.

10.4. Responsibilities of the Site Investigators at the participating institutions (including the relevant institution)

In accordance with the instructions of Principal Investigator/Study Coordinator, the Site Investigator at the participating institution should report to the administrator of the relevant institution if the adverse event is subjects of reporting of "disease or the like" to Certified Review Board in accordance with the regulations of the relevant institution.

10.5. Responsibilities of the Data and Safety Monitoring Committee

JCOG Operations Office (Safety Contact) should check the details of the adverse event reports received from the Principal Investigator/Study Coordinator according to the procedures described in 10.3.2. and should report them to Certified Review Board and the MHLW according to the procedures described below, with the presence or absence of causality or expectation.

In addition, Principal Investigator and Director of Data Center can hear the opinions of JCOG Data and Safety Monitoring Committee according to the reported adverse events. If a review request is issued, Data and Safety Monitoring Committee can review the appropriateness of the institutional response to adverse events and the propriety of continuation of the study in a consensus or written form.

In addition, the submitted information (JCOG Adverse Event Report (for institution), JCOG Adverse Event Report (for Study Coordinator), "Disease or the like" Report, etc.) will be stored semi-permanently in JCOG Operations Office.

Subjects, destination and deadlines for reporting after the knowledge by Investigator/Study Coordinator are as described in 10.3.2.

11. Response Evaluation and Endpoint Definition

11.1. Response assessment (only for patients with measurable disease)

Tumour response assessment will be performed according to the following steps according to the [49] of version 1. 1-Japanese translational JCOG version-Revised RECIST guideline (version 1. 1) Revised new guidelines for the assessment of treatment response in solid tumours (RECIST guidelines). RECISTv1. The 0 original article stipulates that "the use of this guideline for the purpose of determining continuation of treatment is not the subject of this guideline." Similar statements continue to be included in RECISTv1 1 as follows.

> "Many oncologists make decisions about whether to continue treatment based on both objective imaging criteria and symptom-based criteria for follow-up of patients with malignancies in their daily clinical practice, but these revised guidelines are not intended to be used to make decisions about whether or not to continue treatment in these individual patients, unless the treating oncologist determines that it is appropriate."

Therefore, the "overall effect" as determined by RECIST Guideline-based response assessment should be used to determine whether a drug or regimen shows encouraging results that merit continued developmental studies. In other words, judgment of whether or not to continue treatment in individual patients should not be based on CR/PR/SD/PD of overall efficacy, but rather on "clinical judgment" based on comprehensive consideration of symptoms, physical findings, and various laboratory data, in addition to imaging findings.

Therefore, it may be clinically appropriate to continue protocolized treatment, even when PD (Progressive Disease: progression) is judged as an overall response based on the assessment of response based on imaging. In this case, the pros and cons of continuing protocol treatment should be determined based on clinical judgment, regardless of response assessment, but the date of the event for progression-free survival, which is judged to be an overall effect of PD, should be used. This is due to three reasons: (i) it may be possible to decide whether protocol treatment should be continued for each group; (ii) RECIST is a criterion intended to standardize not only response rates but also progression-free survival; and (iii) the standard definition of US Cooperative Group is that PD is the event of progression-free survival for any reason if the overall response is PD.

On the other hand, if a physician judges "clinical progression" based on clinical and comprehensive judgment not based on diagnostic imaging, even if PD is not met by the response criteria based on diagnostic imaging, protocol treatment should be discontinued in accordance with "6.2.2. Criteria for discontinuation of protocol treatment". If "clinical exacerbation" is judged, even if "PD" is not judged by the response evaluation, the day of "clinical exacerbation" is considered as an event of progression-free survival. This is because imaging is often not performed as planned after a patient is judged to have a "clinical progression" and therefore the risk of overestimating progression-free survival is greater if "clinical progression" is not an event for progression-free survival. It is also statistically incorrect (informative censoring) to treat "clinical progression" as "censoring" progression-free survival, as it would censoring patients at increased risk of progression or death.

In RECISTv1 1, the original article described "definite progression (unequivocal progression") in the PD criteria for non-target lesions as "marked progression of non-target lesions that deserves discontinuation of treatment as an increase in total tumor burden" and therefore described "marked progression of non-target lesions" as "judgement of whether or not to continue treatment in individual patients" in some of the PD criteria for non-target lesions, which is confusing. It should be noted that this "unequivocal progression" is a criterion of judgment restricted to "PD of non-target lesions".

The relation between the events of 'PD', 'clinical progression', 'progression', and progression-free survival in JCOG is as in the lower panel.

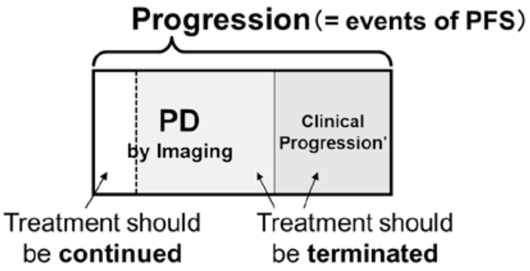

Figure 11.1. Relationship Between Exacerbations, PD on Imaging, and Clinical Exacerbations.

11.1.1. Baseline Evaluation

「8.1. According to "Pre-registration Evaluation Items" contrast enhanced-enhanced computed tomography (CT) with a range of indispensable for the primary organ is used to identify neoplastic lesions before enrollment, and each lesion is classified into "measurable lesions" and "unmeasurable lesions". If CT is allergic to contrast medium, both contrast-enhanced MRI and plain CT should be performed.

Tumor diameters are measured by CT or MRI in cross-sectional views, and sagittal and coronal measurements by three-dimensional reconstructed images are not used. Baseline assessment will be done using the latest imaging studies within 28 days prior to enrollment. If the imaging test is re-examined after enrollment and before the start of treatment, the latest imaging test with re-examination should be used.

11.1.2. Definition of measurable lesions

Lesions that fall under any of the following conditions are considered measurable lesions (measurable lesion):

1) Non-nodal disease (non-nodal disease) of 10 mm or greater in greatest dimension on CT or MRI with a slice thickness of 5 mm or less
2) CT or MRI of 5 mm or less slice thickness showing lymph node lesion of 15 mm or more in short diameter (Nodal lesions with short diameters between 10 mm and less than 15 mm are defined as non-target lesions, and those with short diameters less than 10 mm are not)

All other lesions will be non-measurable (non-measurable lesion).

Caution should be exercised because the following lesions are not measurable regardless of the examination method or the size of the lesion.

- Bone lesions (excluding osteolytic lesions with measurable soft tissue components)
- Cystic lesion
- Leptomeningeal lesions
- Ascites, pleural effusion, and pericardial effusion
- Lymphangiosis of the skin and lungs
- Palpable but not measurable abdominal mass or enlargement of abdominal organs
- Superficial skin lesions

11.1.3. Target Lesion Selection and Baseline Recording

Up to five measurable lesions, in descending order of diameter (non-lymph node lesions are long diameters and nodal lesions are short diameters), up to two lesions per organ are selected to be target lesions (target lesion) among measurable lesions at enrollment. Selection should take into account the universal inclusion of as many organs with measurable disease as possible and the reproducibility or ease of measurement (reproducible repeated measurement) of repeated measurements (avoiding lesions that are not measurable even if they are large in diameter).

For selected target lesions, in order from cranial to caudal, site (code), test method, test date, long diameter of non-lymph node target lesion, short diameter of nodal target lesion, and sum of all target lesion diameters (hereafter, sum of diameters) will be recorded in Pretreatment Report 3.

11.1.4. Baseline recording of non-target lesions

For lesions not selected as target lesions, all measurable or non-target lesions (non-target lesion) should be recorded as site of lesion (code), method of examination, and date of examination in Pretreatment Report 3. Multiple non-target lesions within the same organ may be recorded as a single lesion (e.g., multiple enlarged pelvic lymph nodes, multiple liver metastases).

11.1.5. Determining tumor response

Evaluation of target and non-target lesions will be performed every 6 weeks according to "8.2 Testing and Evaluation during Treatment" in the same manner as at enrollment. Target lesion diameter, non-target lesion disappearance or progression will be recorded in the "Tumor Reduction Effect Report".

11.1.6. Response Evaluation Criteria for Target Lesions

・CR(Complete Response): Complete response

When all non-lymph node target lesions disappear and all nodal target lesions have a short diameter of less than 10 mm. If a nodal target lesion is selected at baseline, the effect of the target lesion may be CR

even if the sum of diameters is not 0 mm.

・PR(Partial Response): Partial response

30% or more reduction in target diameter sum compared to baseline diameter sum

・PD(Progressive Disease): Progress

Compared to the minimum diameters (when the baseline is the minimum value during the passage, this is the minimum sum of diameters), the sum of the target disease increases by more than 20%, and the sum of diameters increases by more than 5 mm even in absolute value.

・SD(Stable Disease): Stability

There is no reduction corresponding to PR and no increase corresponding to PD compared to the smallest sum of diameters during the course

・Lack of study; Not all Evaluated

If the test cannot be performed for any reason or if neither CR, PR, PD, or SD can be determined

Pre-treatment sum of diameters-sum of diameters at study
Percentage of reduction of the diameter sum =---------------× x 100%
Pre-treatment diameter sum

Sum of diameters at study-minimum sum of diameters
Increasing Percentage of Diagram =---------------× x 100%
Minimum sum of diameters

※ Measured target lesion diameters are recorded whenever measurable (e.g., <5 mm). If the target lesion diameter is judged to be too small to be measured (too small to measure), the diameter should be 0 mm if the tumor lesion is judged not to be retained, and 5 mm if the tumor lesion is judged to be retained, regardless of the CT slice thickness.

※ PD is defined when the reduction ratio meets the condition of PR and the concomitant increase ratio meets the condition of PD.

※ When one lesion is separated during treatment, each diameter is added to the sum of diameters.

※ If more than one lesion fuses and the boundary cannot be distinguished during treatment, the diameter of the fused lesion is added to the sum of the diameters. The diameter of each lesion is added to the sum of diameters when the boundary of the lesion is identifiable, even if the lesion is in contact with each other.

11.1.7. Response Evaluation Criteria for Non-Target Lesions

・CR(Complete Response): Complete response

When all non-lymph node non-target lesions disappear, the short diameter of all nodal non-target lesions becomes less than 10 mm, and all tumour markers (NSEs, ProGRP※) are below the upper shared reference limits.

Because ※ProGRP is not included in the shared baseline range, the baseline range is 6.5-46.0 pg/mL.

・Non-CR/non-PD: non-CR/non-PD

Residual one or more non-target lesions (including residual nodal non-target lesions ≧10 mm in short diameter) and/or tumour markers (NSEs, ProGRP※) exceeding the shared upper reference limits.

・PD(Progressive Disease): Progress

'Apparent exacerbation' (including relapse) of pre-existing non-target lesions.

For measurable disease: A marked progression of a non-target lesion that deserves discontinuation of treatment as an increase in overall tumor burden must be observed if the effect of the target lesion is SD or PR but is judged to be "clear progression" based on the change in the non-target lesion. If the effect of the target lesion is SD or PR, then an increase in the tumor burden of the non-target lesion to a degree that far exceeds the decrease in tumor burden is considered "obvious progression" and otherwise Non-CR/non-PD.

If only unmeasurable disease is present, the increase in non-target disease, as judged to clearly exceed the tumor burden corresponding to a 20% increase in diameter and a 73% increase in tumor volume, is

considered "definite progression".

・NE(Not all Evaluated): Lack of study

If the test could not be done for any reason or if neither CR, Non-CR/non-PD nor PD could be determined.

11.1.8. Presence or absence of new lesions

If a lesion that was not present at baseline was observed after the start of treatment, it is considered "new lesion" to be present.

However, a "new lesion" requires that it is not an imaging change due to a difference in the imaging method from the baseline assessment or a change in the imaging modality, nor is it an imaging change due to a condition other than the tumor. For example, a cystic lesion arising within a lesion due to necrosis of a liver metastatic lesion is not a new lesion. New lesions will be defined as new lesions by examination of sites that were not mandatory at baseline (pre-enrollment study).

If a lesion disappears and later reappears, measurement is continued. However, the effect at the time the lesion reappears depends on the status of the other lesion. When the overall effect reappears after CR, the lesion is judged as PD at the time of reappearance. When the overall effect is PR or SD, on the other hand, once the disappeared lesion reappears, the diameter of the lesion will be added to the sum of the diameters of the remaining lesions to calculate the effect. That is, in the presence of many residual lesions, even if one lesion reappears after an apparent disappearance, it is not judged as PD by itself, and it is judged as PD when the sum of the diameters of all lesions meets the criteria for PD. This is because of the perception that the majority of lesions do not truly 'disappear' and are not only depicted by the limits of resolution of the imaging modalities used.

If there is a possibility of a new lesion but it cannot be determined, it should not be a new lesion, and imaging should be reexamined at a clinically relevant time. If a new lesion is confirmed by repeat imaging, the new lesion will appear based on the date of imaging at which the new lesion is confirmed.

11.1.9. Overall efficacy (Overall Response)

The overall response (Overall response) will be determined by combining the effects of target lesions, non-target lesions, and the presence or absence of new lesions every 6 weeks according to Table 11.1.9.a below. The overall effect in the absence of a non-target lesion at baseline will be determined by the effect of the target lesion and the presence or absence of a new lesion, and the overall effect in the absence of a target lesion at baseline will be determined according to the effect of a non-target lesion and the presence or absence of a new lesion according to Table 11.1.9.b.

Table 11.1.9.a. Overall efficacy at each time point for target lesions (with or without non-target lesions)

Target lesion	Nontarget lesions	New lesions	Overall effect
CR	CR	None	CR
CR	Non-CR/non-PD	None	PR
CR	Lack of study	None	PR
PR	Lack of Non-PD or study	None	PR
SD	Lack of Non-PD or study	None	SD
Lack of study	Non-PD	None	NE
PD (obvious progression)	Irrespective of	With or without	PD
Irrespective of	PD	With or without	PD
Irrespective of	Irrespective of	Present	PD

Table 11.1.9.b. Overall efficacy at each time point for patients with non-target lesions only

Nontarget lesions	New lesions	Overall effect
CR	None	CR
Non-CR/non-PD	None	Non-CR/non-PD
Lack of study	None	NE
PD (obvious progression)	With or without	PD
Irrespective of	Present	PD

11.1.10. Best overall effectiveness (Best Overall Response)

CR > PR > SD > PD > NE is considered good, and the best overall effect is the best overall effect throughout the entire course.

PD is defined when imaging cannot be determined due to exacerbation of obvious disease or death before the first response assessment. In addition, NE is defined if it cannot be determined by imaging due to discontinuation of toxicity before the first response assessment or patient refusal.

11.2. Definitions of analyses set

The analysis sets used in periodic central monitoring, interim analysis, and final analysis are defined as follows: The flow diagram below shows the analysis sets.

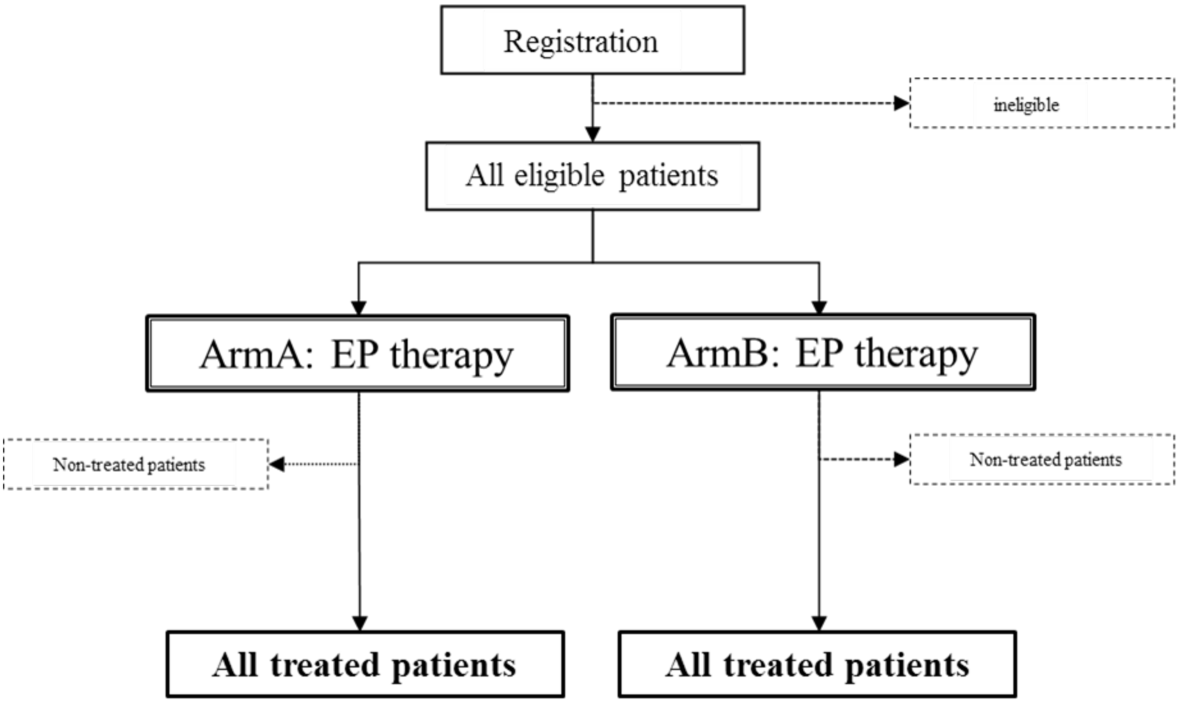

11.2.1. All registered patients

「5.1. Among the patients enrolled according to the Procedures for Enrollment, the population excluding duplicate or mis-enrollment is considered as "all enrolled cases".

11.2.2. All eligible patients

The group excluding "ineligible cases (post hoc ineligibles, de facto ineligibles, violation of registration)" determined by group review from all registered patients is regarded as all eligible patients. Ineligible cases as judged by the investigator or sub-investigator alone are included in all eligible patients. Only those judged not eligible by the central pathological diagnosis are ineligible and not included in all eligible patients.

11.2.3. All treated patients

Of all enrolled patients, all patients for whom part or all of the protocol treatment was performed will be defined as all treated patients.

The decision to treat "non-treated patients" for whom no protocol treatment has been given and whether it is excluded from the safety analysis can be determined by the data center with the consent of the Research Office. Ineligible patients will be excluded from all treated patients. However, if there are circumstances in which ineligible patients are included in the analysis, the nature of the ineligibility will be examined and determined by the Research Secretariat in consultation with JCOG Data Centre.

11.2.4. Patients eligible for central pathology diagnosis

Of all eligible patients, the population excluding ineligible patients with a central pathological diagnosis will be considered as eligible patients with a central pathological diagnosis.

11.3. Definition of endpoints

Endpoint	Events (whichever is earlier)		Censoring date
Overall survival time<br>Overall survival(OS)	All deaths	-	Date of final survival confirmation
Progression-free survival time<br>Progression-free survival(PFS)	All deaths	Progression/r ecurrence	Final date of clinically confirmed freedom from exacerbation

11.3.1. Overall survival

The duration from the date of registration to the date of death from any cause.

- Survivors are censored at the date of final survival confirmation (survival confirmation by telephone contact is also permitted, but the fact that survival confirmation was performed should be recorded in the medical record).
- Patients lost to follow-up are censored at the last date of survival confirmation before lost to follow-up.

11.3.2. Progression-free survival (PFS: Progression-free survival).

The duration from the date of registration to the date of the judgement of exacerbation or death from any cause, whichever comes first.

- The exacerbation (progression) includes both imaging-based PD (progression) and exacerbation of pathogenic disease (clinical exacerbation) that cannot be confirmed by imaging studies in Section 11.1.9. Global Effectiveness. If an exacerbation is judged based on diagnostic imaging, the exacerbation date is the test date on which the imaging was performed, and in the case of clinical exacerbation, the date of clinical judgment is the exacerbation date. PD may occur in accordance with the Response Evaluation Criteria, even if the tumor diameter is very small, but the disease is clinically judged to be "not obviously aggravated" in accordance with the Response Evaluation Criteria Criteria (in this case, clinical judgment should be prioritized for continued treatment with the protocol). In addition, even if PD is not observed according to the response evaluation criteria, the clinical judgment is prioritized for progression if it is judged to be clinically obvious exacerbation.
- In survivors who are not judged to have progression, clinically confirmed progression is censored (date of final progression-free survival confirmation) (confirmation of progression-free by imaging or specimen examination is not mandatory and clinical progression-free by outpatient examination etc. is not permitted. Telephone contact alone is not permitted. If information on progression or progression-free is obtained at a medical institution or referral center, receive and retain a medical information form describing the rationale for diagnosis. In this case, telephone contact alone is not permitted).
- Events and censoring are treated similarly, if chemotherapy is terminated for reasons such as toxicity or patient refusal, and if other therapies are added as post-study treatment. i.e., it is not censored at the time of treatment termination or at the date of initiation of post-study treatment.
- When the diagnosis of exacerbation is based on imaging, the relapse is not regarded as an event at the test date of imaging with "suspicious diagnosis of relapse", but regarded as an event at the later test date of imaging with "definite diagnosis of relapse"" If the event is judged to be clinically aggravated based on diagnostic imaging, the event is considered to be the day of the progression.
- If the definitive diagnosis of relapse or new lesion is based on biopsy pathology, the date of clinical diagnosis is defined as the date of clinical diagnosis when the diagnosis of recurrence or new lesion is made clinically, and the date of biopsy is defined as the date of event when the diagnosis of recurrence is made based on biopsy pathology diagnosis without clinical diagnosis of recurrence.
- The incidence of a second cancer (metachronous double cancer) is not censored or event, and progression-free survival is defined until other events are observed.

11.3.3. Response rate (response rate) Response proportion (Response rate)

Among all enrolled patients with measurable disease, the response rate is defined as the proportion of patients with "11.1.10. Best Global Effect" that is either CR or PR.

11.3.4. Incidence of adverse events (adverse reactions)

Using all treated patients as the denominator, the frequency of the worst Grade during the entire course by CTCAE v4 0 Japanese translation JCOG version for each of the following adverse events (toxicities) will be determined by group:

- Laboratory tests: hemoglobin decreased, white blood cell decreased, neutrophil count decreased, platelet count decreased,

  Increased blood bilirubin, aspartate aminotransferase increased (AST or GOT),
  Alanine aminotransferase increased (ALT or GPT), creatinine increased,
  Hypernatremia, hyponatremia, hyperkalemia, hypokalemia, hypercalcemia, hypocalcemia,

hypermagnesemia, hypomagnesemia

- General disorders and administration site conditions: fever, fatigue
- Ear and labyrinth disorders: Tinnitus, hearing impairment
- Skin and subcutaneous tissue disorders: alopecia
- Gastrointestinal disorders: constipation, diarrhea, nausea, vomiting, oral mucositis
- Metabolism and nutrition disorders: anorexia, dehydration
- Nervous system disorders: dysgeusia, peripheral sensory neuropathy, peripheral motor neuropathy
- Musculoskeletal and connective tissue disorders: myalgia, arthralgia
- Infections and parasites: bronchial infection, pulmonary infection, upper respiratory tract infection, catheter-related infection, biliary tract infection,
  Gallbladder infection; Mediastinal infection; Pleural infection; Urinary tract infection
- Blood and lymphatic system disorders: Febrile Neutropenia
- Musculoskeletal and connective tissue disorders: arthralgia, Vascular disorders: phlebitis
- Respiratory, thoracic and mediastinal disorders: dyspnea, hypoxia, pneumonitis

In the other adverse event (toxicitiy) than the above, the proportion of occurrence are not calculated unless a large number of specific adverse events are observed, since only Grade 3 or more non-hematological toxicity ※ is reported in the Treatment Form.

※※ Non-hematological toxicity refers to adverse events other than those listed below in CTCAE v4.0-JCJCOG.
Anemia, decreased bone marrow cells, decreased lymphocyte count, decreased neutrophil count, decreased white blood cell count, and decreased platelet count.
CD4 lymphocytopenia

11.3.5. Dose intensity of cisplatin

Dose intensity of cisplatin per patient (DOOC.) will be calculated for all treated patients to assess treatment compliance with cisplatin. The summary statistics (minimum, 25% point, median, 75% point, maximum, mean, and standard deviation) will be calculated for each group.

- ・Active dose D.I. (mg/m2/week) = total drug dosebody surface areatreatment duration (weeks)
- Body surface area: BSA is calculated by height at enrollment and body weight calculated by Data Center.
- ・ Treatment period (weeks)
  Arm A = (start date of last course-start date of 1st course + 21)/7
  Arm B = (start date of last course-start date of 1st course + 28)/ 7

11.3.6. Incidence of serious adverse events (adverse reactions)

<u>1) Grade 4 non-hematologic toxicities, early deaths, and treatment</u>

Using all treatments as denominators, the percentage of patients with one or more Grade 4 non-hematologic toxic ※ that are considered to be related (either to definite, probable, possible) to the protocol treatment among the adverse events listed in the free text of the CRFs in addition to the stereotyped items in Section 11.3.4 is used as the numerator.

※※ Non-hematological toxicity refers to adverse events other than those listed below in CTCAE v4.0-JCJCOG.
Anemia, decreased bone marrow cells, decreased lymphocyte count, decreased neutrophil count, decreased white blood cell count, and decreased platelet count.
CD4 lymphocytopenia

<u>2) Early mortality rate</u>

Proportion of all deaths during the protocol treatment or within 30 days from the last protocol treatment day among all treated patients. Causes of death irrespective of causality with protocol treatment. However, if premature death occurs in patients excluded from all treated patients, the details are provided separately.

<u>3) Proportion of treatment-related death (TRD incidence)</u>

Proportion of all deaths judged as causally related (either definite, probable, possible) to the protocol treatment among all treated patients However, if TRD occurs in patients who have been removed from all treated patients, the details are presented separately.

12. Statistical consideration

Methods for statistical analysis are as follows: In addition, the details required for conducting specific analyses are specified in the statistical analysis plan prepared separately prior to the analysis, and in documents that clarify the endpoint definition, etc. If substantial changes occur in statistical analyses as described below, follow the policy set out in "13.6. Protocol Changes." Facilities are contacted by "14.1. Periodic Monitoring" for missing or abnormal results, and data collection or exclusion is determined by review by the Research Secretariat based on the results of inquiries to the institution in accordance with the policies stipulated in "14.1.2. eligible (eligibility/ineligibility)" and "14.1.3. Protocol Deviations/Violations". 「11. Handling of missing values and abnormal data that cannot be addressed by the definition and analysis method for each endpoint, as specified in "Definition of Response Evaluation and Endpoints" and "12. Statistical Items" below, is specified in the above statistical analysis plan.

12.1. Principal Analysis and Decision Criteria

The primary analysis of this study will be the final analysis.

The purpose analysis of this trial is to test whether one of the two community standard-of-care arm A (EP-therapy) and B (IP-therapy) outperforms primary endpoint overall survival. The null hypothesis of equal overall survival in the two groups in the main analysis will be tested by stratified log-rank test stratified by non-institutional allocation adjustment factors (primary organ [gastrointestinal vs. hepatobiliary-pancreatic]) in all patient patients. However, if it is assumed that a stratified log-rank test cannot be performed appropriately, such as when the number of subjects and events in each stratum is small, the allocation adjustment factors will be addressed in the analysis plan prepared without information related to the comparison between groups before performing a confirmatory analysis with comparison between groups. Sensitivity analysis will also be performed in all eligible patients and in patients eligible for central pathology diagnosis.

Due to interest in which treatment group is superior, the test will be two-tailed. The study-wise significance level for the study is set at both sides 10%. In the main analysis, two-sided 90% confidence intervals corresponding to a two-sided 10% significance level will be calculated, and in other analyses, two-sided 95% confidence intervals will be calculated for descriptive purposes.

We conclude that EP therapy is a more useful treatment when the two-sided p-value is less than 10% and the survival curve of EP therapy exceeds that of IP therapy. Meanwhile, we conclude that IP therapy is a more useful treatment when the two-sided p-value is less than 10% and the survival curve of IP therapy exceeds that of EP therapy. If the difference was not significant at a two-sided significance level of 10%, one of the two modalities cannot be judged to be useful. In this case, we conclude that both modalities continue to be the standard of care, as there is no evidence to actively recommend either one of them.

However, if there are major differences in the toxicity profiles of EP and IP therapies, contrary to prior assumptions, during the course of the study, and if the toxic therapy is not superior to other therapies, it is judged that it will not be of significance to be used in clinical practice, the following measures should be taken. In other words, the clinical hypothesis will be changed from two-sided to one-sided in an analysis plan with no information on group comparisons before a confirmatory analysis with group comparisons will be conducted, and the superiority of the toxicity treatment over other treatment modalities will be verified at a one-sided significance level of 5% (in this case, the policy of change for the interim analysis will be described in Section 12.3.2).

Estimates such as cumulative survival curves, median survival times, and annual survival rates are performed using Kaplan-Meier method. Brookmeyer and Crowley methods are used to obtain 95% confidence intervals for median survival. Greenwood's formula is used to obtain 95% confidence intervals for annual survival rates. Hazard ratios and their confidence intervals for treatment effects between groups are obtained using stratified Cox proportional hazards models with the same factors as the test of the primary analysis as estimates of treatment effects. Cox regression adjusted by imbalance background factors in addition to adjustment factors will be performed as needed.

The main analysis results will be summarized as the "Main Analysis Report" by the Data Center one year after the completion of enrollment and submitted to the Research Secretariat, Research Representatives, Group Representatives, Group Secretariats, Efficacy and Safety Assessment Committee, and JCOG Representatives.

The principal investigator/study coordinator summarizes the content of the main analysis report, prepares a

"Clinical Study Report" summarizing the conclusions, problems, interpretations and discussion of the results, and future policies of the entire study, and submits it to the Data and Safety Monitoring Committee and JCOG chair with approval from the Group chair and the Head of JCOG Data Center.

Approval of the clinical study report by the Data and Safety Monitoring Committee shall be considered as "end of the study."

12.2. Planned accrual, accrual period, and follow-up periods

「2.4.2. Based on the background presented in Clinical Hypothesis and Rationale for Number of Enrollment, we assume a median survival of 8 and 12 months (HR=0.67) for the inferior and superior treatment groups, respectively, among the two treatment groups. When a superiority trial design is used, 63 patients per group and 126 patients in both groups (114 events required) will be included in the required analysis using Schoenfeld & Richter methodological [50] at 6 years of enrollment, 1 year of follow-up, α = 10% (two-sided), and 70% power. The number of required Inferior treatment (number of required events) when the median survival time in A is different from the assumption as shown in Table 12.2.1 below.

Table 12.2.1. Number of Analyses Required (Number of Events Required)

	Power			
Median Survival (mo)	65%	70%	75%	80%
7.0 vs. 10.5	108(100)	124(114)	142(131)	162(150)
8.0 vs. 12.0	112(100)	126(114)	144(131)	166(150)
9.0 vs. 13.5	114(100)	130(114)	148(131)	170(150)

※※ Number of required events in parentheses

Based on these findings, the following will be established in view of some cases of loss to follow-up.

Planned enrollment: 70 patients in each group and 140 patients in both groups

Enrollment period: 6 years, follow-up period: 1 year after completion of enrollment

Consider redesigning the sample size if the prognosis is obviously better than assumed, or if it reaches 70 people within 2.5 years of enrollment initiation. Clinically meaningful differences will then be reviewed and redesigned in a blinded fashion prior to the conduct of the analysis.

<Additions in ver. 1.1>

The registration pace after the enrollment start was smooth and exceeded the plan, and 70 cases were reached in October, 2016, when 2 years and 2 months passed from the enrollment start. Since it exceeded the standard of 70 patients within 2.5 years from the initiation of enrollment specified above, acceptance was obtained at the meeting of the Hepatobiliary and Pancreatic Group on October 29, 2016, the group meeting of the Esophageal Cancer Group on November 19, 2016, and the group meeting of the Gastric Cancer Group on January 7, 2017 with respect to changing the power to 80% according to the rules at the time of the study plan. Therefore, the planned enrollment number was changed to 170. In addition, the "1-year analysis period" was added to the study period, and the following changes were made.

Planned enrollment: 85 patients in each group and 170 patients in both groups

Enrollment period: 6 years; Follow-up period: 1 year after completion of enrollment; Analysis period: 1 year; Total study period: 8 years

12.3. Interim Analysis and Early Termination of the Study

12.3.1. Purpose and Timing of the Interim Analysis

Once interim analyses will be conducted to determine if the primary objective of the study has been achieved during the study period. Interim analyses will be conducted to determine if it is reasonable to continue enrollment during enrollment. If the primary objective of the study is determined to be achieved, the study will be discontinued and the study results will be published promptly at the conference and in the article.

Interim analyses will be conducted using data from the initial periodic monitoring that will be queried after the time enrollment of half of the planned enrollment was obtained. Based on the information in the periodic monitoring report, the group will submit the presence or absence of changes in clinical hypotheses and, if any, details of changes to the Efficacy and Safety Assessment Committee by the time of the interim analysis.

During the first interim, patient accrual is continued in principle. If the progress of the study progressed as planned, the expected number of events at the time of the interim analysis under the assumptions presented in 12.2 is expected to be 44 if the interim analysis is conducted at 3 years after the start of enrollment.

12.3.2. Method of interim analysis

Interim analyses will be conducted by the JCOG Data Center. To keep the study-wise alpha errors at 10%, the multiplicity of the interim and final analyses is adjusted using Lan & DeMets's alpha spending functions, and statistical significance is examined for differences in primary endpoint between arms. As α spending functions, we use O'Brien & Fleming types.[51]

For details of the interim analysis, the statistical staff in charge of the group at the Data Center will prepare the statistical analysis plan by the time of the interim analysis. Actual interim analyses will be performed by statistical staff who are not in charge of the group and an interim analysis report will be prepared.

In the interim analysis, if the survival of one treatment group exceeds that of the period, and the p-value of the stratified log-rank test falls below the level specified by the above method, it is judged statistically significant and the trial is discontinued as a rule. The ineffective discontinuation is not planned at a stage where no statistically significant differences are observed unless prior assumptions regarding toxicity are changed. However, the clinical hypothesis may be changed for the reasons described in 12.1, and the primary analysis may be performed by one-sided rather than two-sided testing, which may result in deviations from prior assumptions. In such cases, the primary hypothesis change should be specified in the analysis plan to be prepared without information on group comparisons before a confirmatory analysis with group comparisons. If the overall survival curve in the highly toxic treatment group is below the other, the consideration of ineffective discontinuation should not be judged by a test and considered comprehensively.

12.3.3. Interim analysis Reporting and review of the results

The results of the interim analysis will be submitted to the Data and Safety Monitoring Committee by the Data Center as an Interim Analysis Report and reviewed for the acceptability of continuation of the study and for publication of the results. The Data and Safety Monitoring Committee considers whether to continue the study at the meeting and recommends whether to continue the study and whether to publish the results to principal physician or group chair based on the results of the review.

Members of the Data and Safety Monitoring Committee of the relevant group are not included in the review. Unless the results of the interim analysis make recommendations for discontinuation of the study from the Efficacy and Safety Assessment Committee, the research representative, research office, participating institution researchers, group representatives, and group secretaries of the study will not be able to know the results of the interim analysis until the final follow-up is completed.

When the Interim Analysis Report has been reviewed by the Data and Safety Monitoring Committee to recommend termination or change of all or part of the study, the principle investigator and group chair review the recommendations and decide whether to discontinue or change some of the study.

If the study is discontinued or part of the study is changed, the principal investigator and group chair shall submit in written form a request to the Data and Safety Monitoring Committee for permission to discontinue the study or a request to revise the protocol. Following approval by the Data and Safety Monitoring Committee, the principal investigator may discontinue the study or change part of the study.

The Study Chair and Group Chair can disagree with the recommendations of the Data and Safety Monitoring Committee, but if they fail to coordinate their opinions with the Data and Safety Monitoring Committee, they will ultimately follow the instructions of JCOG Chair.

If the study is terminated, the subsequent follow-up period will be 1 years from last registration per study.

If the interim analysis resulted in study termination, the interim analysis will be the primary analysis of the study. The Data Center, in cooperation with the Research Representative Physicians and Research Secretariat, will conduct the analysis required to complement the incomplete data and publish the results, focusing on the results of the interim analysis, and promptly prepare the Major Analysis Report and submit it to the Group and the Efficacy and Safety Assessment Committee.

12.4. Analysis of Secondary endpoints

Secondary endpoints analyses will be conducted to provide a supplementary discussion of the primary analysis results of the study. Because the analysis of secondary endpoint is exploratory, no multiplicity adjustments are made. Comparisons between arms are made where appropriate, note that when the results of the group comparisons are not significant, they do not mean that there is no difference between the two arms.

12.4.1. Analysis of safety secondary endpoints

Among Secondary endpoints, the safety endpoints are the incidence of adverse events and the incidence of serious adverse events, which are in principle the items of periodic monitoring (14.1. Periodic monitoring).

The incidence rate of adverse events will be summarized as well as the incidence rate of Grade3 or higher. For adverse events other than laboratory data, the incidence of Grade 2 or higher is also calculated.. Non-hematological toxicity incidence, early mortality, and treatment-related mortality rates of Grade4, which are serious adverse events, are reported in periodic monitoring reports with registration numbers and details. The rates of non-hematologic toxicity, early mortality, and treatment-related mortality for Grade4 will be calculated at the time of the interim analysis and the main analysis. When interval estimation of proportions is performed, accurate confidence intervals based on binomial distributions are used. Comparisons between arms will be made using Fisher's exact test where appropriate.

12.4.2. Analysis of efficacy secondary endpoints

Among Secondary endpoints, efficacy endpoints are response rate, progression-free survival, which will only be analyzed in the interim and primary analyses.

Secondary endpoints analyses do not adjust for multiplicity.

Progression-free survival will be included in all enrolled patients, but a comparison of all eligible patients, excluding ineligible patients, as determined by group study, will also be performed as a sensitivity analysis.

The response rate will include all enrolled patients with measurable disease, but a comparison of all eligible patients excluding ineligible cases determined after group review will also be performed as a sensitivity analysis.

Fisher's exact test will be used to compare response rates between groups, and binomial distribution-based exact confidence intervals will be used for interval estimation. Estimates, including progression-free survival curves, median progression-free survival, and time-point progression-free survival, will be performed using Kaplan-Meier method, Brookmeyer and Crowley methods will be used to obtain confidence intervals for median progression-free survival, and Greenwood formulas will be used to obtain confidence intervals for progression-free survival. Log-rank test is used for comparison between arms. Hazard ratios and their 95% confidence intervals for treatment effects between arms will be calculated using Cox's proportional hazards model as an estimate of treatment effect. Cox regression adjusted by imbalance background factors in addition to adjustment factors will be performed as needed.

12.5. Final analysis

The primary analysis will be the final analysis unless this study is withdrawn from the interim analysis.

If the interim analysis is withdrawn from the study, then after the end of the 1-year follow-up period, the final analysis will be performed after the final survey confirms the data and then analyses will be performed for all endpoints.

Except for the interim analyses and the final analysis, analyses with between-arms comparisons for the primary and secondary endpoints for efficacy are not performed unless approved by the Data and Safety Monitoring Committee.

If the final analysis is performed after the main analysis, the final analysis will be summarized by the Data Center as the "Final Analysis Report" and submitted to the Research Secretariat, Research Representatives, Group Representatives, Group Secretariats, Efficacy and Safety Assessment Committee, and JCOG Representatives.

The Study Representative Physician/Research Secretariat summarizes the content of the final analysis report, prepares the "Clinical Study Report" summarizing the conclusions, issues, interpretations and discussion of the results, future policies, etc. mainly from the clinical point of view (if the "Clinical Study Report" is prepared in the previous analysis report, it will be the "Clinical Study Report (Supplementary Version)) with additional updates), and submits it to the Study Representatives and JCOG Representatives with approval from the Group Representatives and the Head of JCOG Data Center.

Approval of the clinical study report by the Data and Safety Monitoring Committee shall be considered as "end of the study."

12.6. Exploratory analysis

To investigate the interaction between treatment effect and the subpopulation, subgroup analyses will be conducted exploratory with respect to the following factors: Because these analyses are not adequately powered and do not adjust for multiplicity, the results of each subgroup analysis should be interpreted as exploratory.

- PS0/1
- Age 65 years or older/<
- Gender (male/female)
- Primary organ (gastrointestinal tract/hepatobiliary pancreas)
- Organ of origin (esophagus/stomach/small intestine/large intestine/pancreas/biliary tract/liver NEC (liver primary or liver metastasis of unknown primary)
- Organ of origin (pancreas/non-pancreas)
- Extent of extension of the primary lesion (locally advanced/distant metastasis or recurrence)
- Extent of extension of the primary lesion (locally advanced/distant metastasis/recurrence)
- Prior radical resection of the primary lesion (none/present)
- Pathological diagnosis was biopsy/resection specimen
- Grade 3 tumour with morphologically similar features of NETs but Grade 3 proliferative activity/morphologically more atypical (previously classified as poorly differentiated endocrine carcinoma) on histopathology with central pathology
- Histopathological examination by central pathological diagnosis, including Small cell carcinoma/Large cell carcinoma /
- Histopathologically diagnosed by central pathology, Ki67 50% or higher/less than 50%

12.7. Premature withdrawal from the trial

In this study, early termination of the study may occur in the following cases:

1) Early termination due to discontinuation of interim analysis
2) Early termination due to adverse events
3) Early termination due to poor enrollment
4) Early termination due to other reasons

12.7.1. Early termination by interim analysis

In this study, based on the criteria described in 12.3, early termination recommendations may be made at the interim analysis review by the Efficacy and Safety Assessment Committee. If the Data and Safety Monitoring Committee provides recommendations for early termination of the study, the principle investigator and group chair will review the recommendations and decide whether to terminate the study early.

12.7.2. Early termination due to adverse events

In JCOG9511 for small-cell lung cancer, 1/77 (1.3%) treatment-related deaths were reported with EP therapy and 3/77 (3.9%) with IP therapy; in JCOG0509, a successor study for the same subject, 1/142 (0.7%) treatment-related deaths were reported with IP therapy and 2/142 (1.4%) with amrubicin plus cisplatin therapy. Using these as reference, we believe that the treatment-related mortality rate should not exceed 3% in this study. Since it is clear that the final point estimate will be at least 3% at the time of 3 treatment-related deaths in either group, immediate entry should be suspended to consider whether or not to withdraw from the study. At this point, the subsequent treatment of the patient being treated will be reviewed. At the time of 3 or fewer treatment-related deaths in each group, each patient will be reported to the Efficacy and Safety Assessment Committee for adjudication. Enrollment will be continued until the results are obtained in principle.

12.7.3. Early termination due to poor enrollment

If the patient enrollment pace is significantly worse than at the time of planning, early termination of the study may be advised by the Data and Safety Monitoring Committee. If early termination recommendations are issued by

the Data and Safety Monitoring Committee due to poor enrollment, the principle investigator and group chair will review the recommendations and decide whether to terminate the study early.

12.7.4. Early termination due to other reasons

12.7.1.～12.7.3. If it is judged difficult to continue the study for other reasons, the research representative physician shall submit a request for early termination of the study to the Efficacy and Safety Assessment Committee. If the Data and Safety Monitoring Committee recommends early termination of the study based on the submitted data, the procedure for early termination of the study will be progressed.

12.8. Procedures after Early termination of the Study

If the Study Chair accepts the recommendations made by the Data and Safety Monitoring Committee based on Section 12.7, he/she will promptly submit a notification to the Data and Safety Monitoring Committee that early termination of the study will be performed.

The Study Chair will submit a termination notification to the Certified Review Board within 10 days of the date they decide to terminate the study early. If the study falls into a specified clinical trials under the Clinical Trials Act, the Study Chair shall submit a termination notification to the Certified Review Board within 10 days of the date on which the study was decided to be prematurely discontinued, as well as submit a specified clinical trials termination notification to the MHLW.

The Study Chair promptly informs the investigator of the decision to terminate the study early in writing, and the investigator who has received a report of early termination of the study will report in writing that the study was prematurely terminated to the institution's manager without delay.

If the study is terminated early, JCOG Data Center will promptly initiate the development of the primary analysis report or final analysis report. The subsequent follow-up period will be 1 year from the final enrollment.

13. Ethical Considerations

13.1. Protection of Human Subjects

All researchers involved in this study will conduct this trial in accordance with the "Helsinki Declaration" 1) and "Clinical Trials Act" (2017 Law No. 16) 2) "Clinical Trials Act Enforcement Regulations" (2018 Ministry of Health, Labour and Welfare Order No. 17) and related notices.

1） http://dl.med.or.jp/dl-med/wma/helsinki2013j.pdf

2） http://www.mhlw.go.jp/stf/seisakunitsuite/bunya/0000163417.html

Prior to commencing this study, the site investigator is required to obtain input from Certified Review Board※1 regarding the conduct of the study, and to obtain approval from the Administrator of the participating institution, and submit the Implementation Plan ※2 to the Ministry of Health, Labour and Welfare.

※ 1 JCOG study will be submitted to the following Certified Review Board.
National Cancer Center Hospital Certified Review Board (accreditation number CRB3180008)

※ 2 "Implementation Plan" means "Documented plan formatted according to the Form No.1 (Form No.1 of the Ministerial Ordinance) specified in Article 39 of the Clinical Trials Act Enforcement Regulations"

13.2. Informed consent

13.2.1. Explanation to the patient

Prior to patient registration, the investigator or subinvestigator will provide the patient with written informed consent form approved by Certified Review Board and explain the following details verbally.

Descriptions

1） Disease names, Stages, and expected prognosis

2） That this study is a clinical trial and is conducted by JCOG
Name of Certified Review Board and contact information for receiving complaints and inquiries to the committee

3） Design and rationale of the study

4） Protocol treatment content

5） Effects expected by protocol treatment

6） Expected adverse events, complications, and sequelae and how to deal with them
Explanation of the extent and frequency of expected adverse events, including complications, sequelae, and treatment-related deaths, and how to deal with them when they occur. In addition to these explanation, obtain the most recent version of the drug package insert and deliver it to patients (PMDA Prescription Pharmaceutical Information Search http ://www.pmda.go. jp/PmdaSearch/iyakuSearch/)

7） Post-study treatment after end of protocol treatment should also be performed appropriately

8） Cost burden and compensations
Explanation of the cost of treatment, compensation that can be received in the event of a health hazard (equivalent to measures taken in general practice, etc.)

9） Alternative treatment
Explanation of treatments that can be received if not participating in this study

10） Anticipated benefits and possible disadvantages
Explanation of anticipated benefits and possible disadvantages by participating in this study

11） Direct access to the medical records
Explanations on acceptance of site visit audits, such as "direct access to medical records etc. by healthcare professionals at other medical institutions for quality control with permission from the administrator of the participating institution."

12） Refusal of consent and withdrawal of consent
Refusal to consent prior to participation in the study is free, and withdrawal after having given consent is free, thereby not causing undue medical disadvantage.

13） Protecting human rights
Every effort should be made to ensure that personal information, such as names, is kept confidential.

14） Secondary use of data

The possibility of secondary use of data obtained from this study in Japan and overseas (ancillary studies, meta-analyses, etc.) only when approved by either committee in JCOG

15) Method of disclosure of information on the study
The study is registered and published in jRCT※. In addition, the results of clinical studies should also be published in jRCT (* Databases (Japan Registry of Clinical Trials) https://jrct.niph.go.jp/ prepared by the MHLW as stipulated in Paragraph 1 in Article 24 of Clinical Trials Act Enforcement Regulations)

16) Freedom of questions
Explanation that investigators, written contact information for consultations on study details, on the Principal Investigator and the Study Coordinator, and freely asking questions about study and treatment

17) Explanation of the use and burden of medicinal products not covered by insurance

18) Central pathological review

19) Central image review for response

20) Sample collection for ancillary studies

13.2.2. Consent

Explain the study, give sufficient time to think, confirm that the patient understood the study well, and ask for participation in the study. If the patient agrees to participate in the study, the written informed consent form in the appendix will be used to obtain the patient's own signature. The site investigator or the subinvestigator confirms that the study consent form contains the name of the physician who provided the explanation and the date of explanation, the name of the patient who gave informed consent, and the date of informed consent.

In addition, when it is not possible to read the documents due to visual impairment, etc., but the details can be understood by verbal explanation, or the documents can not be signed due to limb disorders, etc., but the documents can be read and understood, signatures may be obtained from the proxy author under the consent by the patient. However, the signature of the proxy author should be based on this study's consent, and should be described as "Signing by the proxy author" and "Relationship with the patient" so that the person can be found to be the proxy author.

Two copies of the consent form will be provided, one will be handed over to the patient, and one will be stored by the site coordinator. Original copies will be stored in the medical records or in the archives specified by the participating institution.

13.2.3. Response to inquiries, consultations, etc. after consent

In principle, the investigator or subinvestigator of the relevant patient's participating institution responds to any consultation related to the study by the patient or his/her family after registration. If it is unclear how to respond, respond in consultation with Principal Investigator, Study Coordinator, the Group Secretariat, Group Chair, JCOG Data Center/Operations Office, etc. in accordance with the content of the consultation.

13.2.4. Withdrawal of consent

After obtaining informed consent for participation in the study, consent will be withdrawn if the patient expressed withdrawal of consent.

Withdrawal of consent refers to withdrawal of consent to participate in research and is distinguished from refusal to continue protocol treatment (below (i)). If the withdrawal of consent is expressed, clarify whether (ii) or (iii) below and promptly notify JCOG Data Center. If consent is withdrawn, record it in the medical record as well as (ii) or (iii).

Data Center discontinues subsequent follow-up requests according to the protocol in case of (ii) withdrawal of consent, In the case of (iii) full withdrawal of consent, the data of the patient are removed from the database when it is confirmed that the patient has full withdrawn of consent.

The procedures for discontinuation of the patient's follow-up request and removal of patient data will be specified separately in the procedural manual, and the completion of each task will be reported to Principal Investigator and Study Coordinator.

(i) Patient refusal: Refusal to continue subsequent protocol treatment (follow-up continues).

(ii) Withdrawal of consent: Withdrawal of consent to participate in the study and termination of all subsequent treatment and follow-up in accordance with the study protocol. Research use of data prior to withdrawal of consent is permitted.

(iii) Full withdrawal of consent: Withdrawal of consent to participate in the study and unavailability of all data from the time of patient registration, including information at registration.

In addition, some medical institutions may request that a "withdrawal of consent" form be prepared as a written document. However, in the event of withdrawal of consent, written expressions of willingness are required to increase the psychological barriers to withdrawal of consent (i.e., it is difficult to withdraw consent), and it is considered unwanted from the viewpoint of protecting human subjects. Therefore, in JCOG, written expressions of willingness are not mandatory for withdrawal of consent, verbal withdrawal of consent is valid, and the "withdrawal of consent" form is not prepared. If "withdrawal of consent" form is required by the participating institution, it should be prepared by the institution.

- This should be used in studies conducted in accordance with the Ethical Guidelines for Medical and Health Research Involving Human Subjects.

The procedures for discontinuation of the patient's follow-up request and removal of patient data will be specified separately in the procedural manual, and the completion of each task will be reported to Study Chair and Study Coordinator.

(i) Patient refusal: Refusal to continue subsequent protocol treatment (follow-up continues).

(ii) Withdrawal of consent: Withdrawal of consent to participate in the study and termination of all subsequent treatment and follow-up in accordance with the study protocol. Research use of data prior to withdrawal of consent is permitted.

(iii) Full withdrawal of consent: Withdrawal of consent to participate in the study and unavailability of all data from the time of patient registration, including information at registration.

In addition, some medical institutions may request that a "withdrawal of consent" form be prepared as a written document. However, in the event of withdrawal of consent, written expressions of willingness are required to increase the psychological barriers to withdrawal of consent (i.e., it is difficult to withdraw consent), and it is considered unwanted from the viewpoint of protecting human subjects. Therefore, in JCOG, written expressions of willingness are not mandatory for withdrawal of consent, verbal withdrawal of consent is valid, and the "withdrawal of consent" form is not prepared. If "withdrawal of consent" form is required by the participating institution, it should be prepared by the institution.

13.3. Protection of personal Information and patient identification

JCOG recognizes that information on privacy, such as personal information and medical information, should be protected and handled carefully, based on the principle of respecting the personality of individuals, and has formulated JCOG Privacy Policy, and will take all possible measures to protect privacy. For more information, see JCOG website (http://www.JCOG.jp/).

13.3.1. Policies, legislation, and norms followed by JCOG

In conducting JCOG study, JCOG follows, in principle, JCOG Privacy Policy as well as the following laws and norms depending on the content of the research. If other laws, norms, and policies are applicable, they should be followed.

- Clinical Trials Act (Law No. 16, 2017).
- Act on the Protection of Personal Information (Law No. 57, 2003, Final Amendment: Law No. 65, September 9, 2015).
- Helsinki Declaration (Translation by the Japan Medical Association)
- Ethical Guidelines for Medical and Health Research Involving Human Subjects (No.1 of Notice of the Ministry of Education, Culture, Sports, Science and Technology and the Ministry of Health, Labour and Welfare, 2017).

13.3.2. Use of personal information objective, items to be used, and methods of use

1) Objective of use

In accordance with the basic philosophy "Providing the best treatment to more patients," JCOG uses personal information, etc. of patients for objective of "Identifying patients and conducting surveys not only during treatment but also for a long period after end of treatment in order to obtain the correct results of clinical studies, and appropriately managing the acquired information."

2) Items to be used

Information to identify individuals who will be used by JCOG as minimally require for identification and inquiry of patients is as follows.

Medical record number, date of birth, initials, and registration number, Pathology specimen number

In other words, information that can identify individuals other than those listed above, such as the patient's name, is not informed to Data Center by participating institutions, and if they are falsely informed, they should be destroyed without using the recording medium or stored after performing appropriate processing, such as masking, which is incapable of reading.

3) Method of use

Personal information of patients used by JCOG will be collected by entering the CRFs etc. by researchers at participating institutions and submitting them to Data Center either by JCOG Web Entry System, mailing, or handover as a rule. However, telephone calls will be used only for patient registration where prompt contact is necessary.

In addition, in order to confirm the accuracy of the collected information, inquiries regarding various types of CRFs, including personal information, between Data Center and researchers at medical institutions are limited to either JCOG Web Entry System, mailing, or handover. Only the more anonymous registration number should be used when interacting with e-mail inquiries, and medical record numbers and initials should not be used.

13.3.3. Preparation of records for provision of samples and information, etc. at participating institutions

The investigator at each site will prepare records for this study of enrolled patients. The matters prescribed by the Ordinance of the Ministry of Health, Labour and Welfare (Article 53 of Clinical Trials Act Enforcement Regulations) are as follows.

- Identification of clinical-research subjects
- Items related to medical care and testing for subjects personnel in clinical studies
- Items related to participation in clinical research
- Other matters necessary to conduct clinical research

13.3.4. Source documents used in clinical studies (JCOG trials conducted under Clinical Trials Act)

The source documents for clinical studies used in this study refer to all records used for diagnosis and treatment, including medical records (including worksheets, etc.), laboratory test records, diagnostic images used for diagnosis, pathological diagnosis report, images used for response evaluation, and informed consent documents, of enrolled patients in this study. These should be available for direct access for monitoring (14.1.) and audit (14.2.) conducted by Data Center, Operations Office, Certified Review Board and regulatory persons

13.3.5. Storage of samples and information

Samples and information of enrolled patients on this study shall be stored in accordance with Article 53 of Clinical Trials Act Enforcement Regulations (MHLW ordinance No. 17, 2018). The retention time of records related to this study at participating institutions and the retention time of source documents will be 5 years from the date of completion of the study. It is recommended that the records be stored for as long as possible after the expiration date.

The retention time of data collected in JCOG Data Center will be semi-permanent in view of the possibility of long-term follow-up and secondary use for other studies. In addition, as records for the provision of samples and information, the study protocols and the model informed consent forms will be stored semi-permanently in JCOG Data Center.

13.3.6. Anonymization and control of response tables

In JCOG studies, information that can clearly identify individuals, such as patients' names, is not collected, and individuals are identified using registration numbers and medical record numbers (anonymized). Correspondence tables (not always in the form of a table) of information and registration numbers that can clearly identify individuals by themselves, such as patient names, are positioned as present at each participating institution and are appropriately managed according to the policy of participating institutions so that the identification of enrolled patients can be ensured.

13.3.7. Secondary use of data

Data from this study may be used in Japan or abroad for secondary use (e.g., meta-analysis) only if approved by the relevant committee of JCOG (e.g., Protocol Review Committee, Data and Safety Monitoring Committee). However, when providing data to external organization(e.g., a meta-analysis), data are provided so that it is not possible to identify individuals.

Secondary use of data should be disclosed on JCOG website to ensure opportunities for patients to refuse using data.

13.3.8. Safety management responsibility system

JCOG Data Center establishes a privacy protection control manager and a privacy protection manager and takes various safety control measures to minimize the risks of information leakage when using personal information, etc.

13.3.9. Response to request of disclosure of patient information

In the event that JCOG is asked to disclose privacy-related information, etc., the responder shall, in principle, be a researcher (site investigator, subinvestigator) at the institution of the relevant patient.

13.3.10. Receipt of inquiries about JCOG

General inquiries and complaints about privacy policies will be received by either mail, e-mail, or fax below.

Inquiry liaison: JCOG Data Center Privacy Protective

Postal destination :〒104-0045 Tsukiji 5-1-1, Chuo-ku, Tokyo
Clinical Research Support Office, National Cancer Center Hospital
E-mail :JCOG_privacy@ml.JCOG.jp
FAX :03-3542-3374

13.4. Adherence to the protocol

Researchers participating in this study will adhere to this protocol unless they compromise patient safety and human rights.

13.5. Application to Certified Review Board and Notification of Implementation Plans

When conducting this study, the approval of Certified Review Board and the permission by the administrator of each institution to conduct the study using this protocol and written informed consent from patients must be obtained. Prior to the commencement of the study, the implementation plan [※1] shall be submitted to the Minister of Health, Labour and Welfare, and the test data shall be published to jRCT[※2]. Principal Investigator is responsible for applying to Certified Review Board, submitting Implementation Plans to the MHLW, and registration in jRCT, and JCOG Operations Office supports these application procedures.

※ 1 Form No.1 of the Ministerial Ordinance stipulated in Article 39, Paragraph 1 of the Ordinance for Clinical Trials Act Enforcement Regulations

※ 2 Databases (Japan Registry of Clinical Trials) prepared by MHLW as specified in Article 24, Paragraph 1 of Clinical Trials Act Enforcement Regulations https://jrct.niph.go.jp/

13.5.1. Procedures for new application

<u>1) Procedures performed by Principal Investigator</u>

<Procedures from initial submission to study initiation>

(i) Review and approval of the study protocol by JCOG Protocol Review Committee (ver.1.0.0).

(ii) Principal Investigator will prepare the following documents for this study and submit these documents to Certified Review Board for review through JCOG Operations Office.

- ➢ New Review Request Form (Unified Form No.2 for Clinical Research)
- ➢ Implementation Plan (Ministerial Ordinance Form No.1)
- ➢ Study plan (this protocol) (including response to "disease or the like")
- ➢ Informed consent form
- ➢ Documents describing summary of drugs, etc. (e.g., package inserts of drugs used as a part of protocol treatment)
- ➢ List of Subinvestigators (Unified Form No.1 for Clinical Research)
- ➢ Conflict of Interest Management Standard (Guidance Form A), Conflict of Interest Management Plan (Guidance Form E) (see 13.X.)
- ➢ Other documents to be submitted when prepared (review documents of JCOG Protocol Review Committee, sample Case Report Forms, and a draft agreement on funding with the marketing authorisation of drugs, etc. or its special associates)

(iii) Modifications such as protocols and informed consent forms are made to review opinions submitted by Certified Review Board as needed.

※ Response to the indications by Certified Review Board: Prepare modified versions with review and approval by the Director of Data Center if modification of the protocol or informed consent forms is needed (ver.1.0.1, ver.1.0.2, ver.1.0.3…). .

(iv) After Certified Review Board approval is obtained, JCOG Operations Office should have Certified Review Board approval date and the approved version number on the cover page of the protocol and informed consent form and appear on JCOG website's Protocol Download page.

(v) Principal Investigator will use Certified Review Board review results notification and the documents submitted in ② to obtain the approval of the administrator of the institution which he/she belongs to, send these documents to the site investigators of all participating institutions described in the Implementation Plan, and request that the administrators of each participating institution permit conduct the research.

(vi) JCOG Operations Office shall apply for jRCT registration under the supervision of Principal Investigator after obtaining the permission by the administrators of all participating institutions described in the Implementation Plan and confirming that the patient registration system is open. "Study progress" in the trial registration will be registered as "being recruited."

(vii) JCOG Operations Office will inform Principal Investigator that jRCT registration has been completed after submission of the registration application. Principal Investigator will output the Implementation Plan from jRCT and submit the Implementation Plan, informed consent form, and Certified Review Board review result notification to the MHLW (Local Health and Welfare Bureau, which is responsible for the location of Certified Review Board; the same below). After submission, Principal Investigator will promptly inform Certified Review Board described in the Implementation Plan. In addition, Principal Investigator shall promptly report the submission of the Implementation Plan to the administrator of the institution to which he/she belongs, and provide this information to the site investigators of all participating institutions listed in the Implementation Plan and JCOG Operations Office.

(viii) After confirming that the notification of the Implementation Plan, etc. to the Local Ministry of Health and Welfare has been accepted and that jRCT's status has been updated from "under registration application" to "registration open," Principal Investigator will inform JCOG Operations Office that it has become "registration open." JCOG Operations Office opens JCOG Data Center's patient registration system and provides trial initiation announcements to study group. The date of jRCT publication is the start date of the study. "】

JCOG Operations Office should be contacted for procedures involving the addition of participating institutions after the initial submission.

"16.X. Medical institutions" changes (adding or replacing participating institutions) correspond to changes in the content of the protocol, then the change procedure shall be performed in accordance with the "13.6.2.Procedures for

when a change in the protocol occurs after the start of the study"

2) Procedures performed by the investigators at each participating institution

Following Certified Review Board approval, the investigator at each participating institution will obtain permission to conduct the research by the administrator of the participating institution using a set of documents received from Principal Investigator (Certified Review Board review results notification and documents submitted to Certified Review Board). The site investigator shall promptly send a copy of the letter of permission of the relevant institution to the Data Center after obtaining the permission by the administrator to conduct the research.

If the study is Specified Clinical Trial under Clinical Trials Act, the site investigator who has been informed that the Implementation Plan has been submitted to the MHLW by Principal Investigator should promptly report that the Implementation Plan has been submitted to the administrator of the affiliated institution.

3) Permission to conduct research at participating institutions

The procedures for obtaining permission for conducting research from the administrator of the affiliated institution shall be in accordance with the regulations of each institution.

When a copy of the institutional approval form is sent to JCOG Data Center, either the site investigator or the site coordinator should send the copy. Original copies of the institutional approval form will be stored by the site coordinator, and copies will be stored by JCOG Data Center.

When a affiliated institution has restrictions on providing personal information, such as medical record numbers, when sending copies of the institutional approval form to JCOG Data Center, the site coordinator shall communicate that personal information cannot be provided, and also send documents, such as the corresponding table of the ID number for registration and the medical record number, which is specified in JCOG privacy policy.

In addition, the informed consent form for patients approved by Certified Review Board may not be modified except for the contact information of the institution or prespecified selection items. A common protocol will be used for all institutions, since no changes in the protocols will be permitted for each institution. If it is necessary to change the content of the protocol and informed consent form, consult with Principal Investigator and Study Coordinator if the administrator of the institution asks for modification of the protocol and the text of the informed consent form in order to make the change as the protocol and informed consent form used by all institutions.

13.5.2. Procedures for changing the study plan after the start of the study

1) Procedures performed by Principal Investigator

If any of the changes of (1), (2), or (3) below occurs in the conduct of this study from the <Procedures from the initial application to the commencement of the study> in "13.6.1. Procedures at the time of new application", Principal Investigator shall hear Certified Review Board's opinions by applying for a change to Certified Review Board.

The procedures for applying for changes to the Certified Review Board are in accordance with "13.6.1. Procedures at the time of new submission" in the procedures from initial submission to the start of the study in ②, ③, and ④, respectively. In the absence of a change in the Implementation Plan, notification to the MHLW is not necessary. In the event that a change in the Implementation Plan occurs, notification to the MHLW is required. If Principal Investigator becomes aware of the planned change in the Implementation Plan, he or she should promptly contact JCOG Operations Office. JCOG Operations Office will assist in the application procedure for change to Certified Review Board and registration in change to jRCT.

（1） When the content of the protocol or informed consent form is changed (when it is amendment or revision in 13.7.1.).

（2） When the protocol or informed consent form is not changed, but the Implementation Plan (registered in jRCT) is changed.

（3） When changing Conflict of Interest Management Standards or Conflict of Interest Management Plans

Because notification of changes in the Implementation Plan (registered in jRCT) to the MHLW except for changes in research progress must be done in advance, Principal Investigator should inform site investigators and JCOG Operations Office of the change including the replacement of the investigator or the subinvestigator at each

participating institution. In addition, inform other investigators as soon as there are any changes that need to be made known.

Principal Investigator shall promptly report to the administrator of the institution to which he/she belongs and provide information to other research investigators when informed by Certified Review Board.

Procedures when it is necessary to notify changes to the Certified Review Board and the MHLW before changes are made.

1. Changes to the Implementation Plan

If changes to any of the above (1), (2), or (3) are made to the Implementation Plan (registered in jRCT) after hearing Certified Review Board's opinions, JCOG Operations Office should enter changes to jRCT under the supervision of Principal Investigator. In addition, Principal Investigator submits the following notification to the MHLW. After the date of coming into force of the predefined changes, the study shall be conducted in accordance with the changes.

- ➢ Notification of Change in Implementation Plan (Form No.2 of the Ministerial Ordinance)
- ➢ Implementation Plan after the change (output of the change in jRCT)
- ➢ Certified Review Board Review Results Notification

※ On institution transitioning to JCOG Collaborating Institution by replacement of participating institution: For institutions where patients were not enrolled prior to transfer to collaborating institution, Principal Investigator should remove it from the participating institutions by notifying changes in the Implementation Plan.

Procedures when it is necessary to notify a change to the Certified Review Board and the MHLW after the change***

1. Change in progress

(2) Among the changes in the Implementation Plan (registered in jRCT), the change in "3. Issues related to checking the implementation of Specified Clinical Trials (2) Specified Clinical Trials progress" shall be made without delay after the change. Under the supervision of Principal Investigator, JCOG Operations Office shall change "Research Progress" in jRCT. Subsequently, Principal Investigator will promptly apply for a change to Certified Review Board. After obtaining approval from Certified Review Board, Principal Investigator will submit the following notification to the MHLW.

- ➢ Notification of Change in Implementation Plan (Form No.2 of the Ministerial Ordinance)
- ➢ Implementation Plan after the change (the content of the change in jRCT was outputted)
- ➢ Certified Review Board Review Results Notification].

2. Changes in Implementation Plan after registration of the first patient

At the time of the initial submission, the "date of registration of the first patient" in the Implementation Plan is provided in a blank space. The Implementation Plan should be changed without delay after registration in the first patient. In this instance, JCOG Operations Office enters and registers the "First Patient Registration Date" in jRCT under the supervision of Principal Investigator. Subsequently, Principal Investigator will promptly apply for a change to Certified Review Board.

- ➢ Notification of Change in Implementation Plan (Form No.2 of the Ministerial Ordinance)
- ➢ Implementation Plan after the change (output of the change in jRCT)
- ➢ Certified Review Board Review Results Notification]

3. Minor changes specified by MHLW ordinance (application for change to Certified Review Board is not required)

If the following minor changes are made to Implementation Planning and the registration of jRCT, Principal Investigator will not need to hear the opinions of Certified Review Board and will notify Certified Review Board of the changes within 10 days of the date of the change. In addition, a notification (Form No.3) shall be submitted to the MHLW.

Scope of minor changes to the Implementation Plan of Article 42 of Clinical Trials Act Enforcement Regulations

- A change in the name of a person engaged in Specified Clinical Trials that does not involve a replacement of the person engaged in Specified Clinical Trials.
- Changes due to a change in the name or address number of the area

2) Procedures performed by the investigators at each participating institution

If any changes occur in the part of the protocol that corresponds to the institution to which he/she belongs, (4) Items related to investigators in multi-institutional studies, inform Principal Investigator and JCOG Operations Office of the changes to be made prior to the changes. Depending on the content of the change, the investigator shall confirm the confirmation of institutional requirements, prepare documents for conflicts of interest and submits to Certified Review Board such as lists of subinvestigators, and inform Principal Investigator and JCOG Operations Office.

The content of the most recent Implementation Plan for the institution to which they belong (the same as the content of jRCT registration) should be checked on jRCT website (https://jrct.niph.go.jp/).

13.5.3. Review and approval of study progress and study continuation (periodic reports)

Principal Investigator will report periodic reports on the progress of the study, the occurrence of adverse events, and conflict of interest management (see 13.X.1.⑱) to the administrators of their institutions and report them to Certified Review Board. Within two months after the expiration of each year from the date of submission of the Implementation Plan to the Minister of Health, Labour and Welfare.

When the report is made to Certified Review Board, Principal Investigator shall promptly inform the investigators of other participating institutions of the fact. The investigator who receives the information shall promptly report the details of the information to the administrator of the institution to which he/she belongs.

If the study falls under Specified Clinical Trials above Clinical Trials Act, Principal Investigator will report to the MHLW regarding the implementation status of Specified Clinical Trials. Periodic reports to Certified Review Board shall be made within 1 month of the date on which the results on the appropriateness of continuation of the relevant Specified Clinical Trials are obtained. The report shall be made by submitting the attached Form 3※ to the Minister of Health, Labour and Welfare.

※ Enforcement of Clinical Trials Act Enforcement Regulations, etc. (Notification No. 0228-1 of the Sector of Economics, Ministry of Health, Labour and Welfare, Notification No. 0228-1 of the Notification No. 1 of the Evaluation and Development Division, Health Policy Bureau, Ministry of Health, Labour and Welfare, dated February 28, 2018)

13.6. Protocol revision/amendment

13.6.1. Categorization of protocol changes and procedures for changes

For a change in the protocol, the Protocol Revision Application must be submitted to Data and Safety Monitoring Committee Office prior to submission to Certified Review Board [in the case of Specified Clinical Trials, add the following: "and report to the MHLW"] (see 13.6.2).

JCOG deals with the changes in the content of the protocol after approval by the Protocol Review Committee, divided into amendment and revision. Data and Safety Monitoring Committee Office will distinguish between amendment and revision, so all of applications are submitted as revision. If it is classified as an amendment by the Secretary-General of Data and Safety Monitoring Committee, it is reviewed by Data and Safety Monitoring Committee. If it is classified as a revision, Secretary-General of Data and Safety Monitoring Committee will issue a verification form and will not be reviewed by Data and Safety Monitoring Committee. We also distinguish the addition of supplementary explanations that do not fall into a change in protocol content as "Memorandum". Definitions and handling are as follows:

When the protocol or informed consent form is revised or amended, Principal Investigator will promptly distribute the most recent protocol or informed consent form to those involved in the study. Individuals involved in the study should always conduct the study in accordance with the most recent protocol since the dates of entry into force of the amendment/revision.

1) Amendment

Partial protocol change which meets one or more of the followings: i) Potential to increase the risk of patients enrolled in the study, ii) Having substantial effects on primary endpoint of the study, iii) having essential effects on the study's implementation structure.

The amended version of the protocol and informed consent form version numbers are shown as in 2.0.0, 3.0.0, and 4.0.0….

Approval by the Group Chair and the Data Center Director must be obtained prior to submission to Data and Safety Monitoring Committee.

When classified as "amendment" by the Secretary-General of Data and Safety Monitoring Committee, review of changes by Data and Safety Monitoring Committee is performed prior to Certified Review Board review. After the protocol amendment has been approved by Data and Safety Monitoring Committee, an application for a change in the protocol will be submitted to Certified Review Board through JCOG Operations Office (at this time the version number is ver. 2.0.0, 3.0.0, 4.0.0…). When the protocol was changed based on the review opinions of Certified Review Board, the version number is ver. 2.0.1, ver.2.0.2… If the protocol is changed by review of Certified Review Board, the change will be reported to Data and Safety Monitoring Committee, but in principle, the change will not be reexamined by Data and Safety Monitoring Committee. When a protocol change is approved by Certified Review Board, the protocol cover page should include the date of approval by Data and Safety Monitoring Committee and Certified Review Board [If Specified Clinical Trials, the following is added: "and a notification of the change in the Implementation Plan to the Regional Bureau of Health and Welfare should be made"].

Following Certified Review Board approval, permission for the contents of the amendment by the administrator of each institution shall be obtained. If permission is obtained, the site coordinator of each institution will send copies of the permission notice by the administrator of each institution to the Data Center. After the permission of the administrator of all institutions is obtained, the protocol changes will come into effect (during this time, the patient registration will not be suspended unless there is a special need). The actual date of entry into force will be announced by JCOG Operations Office. Researchers in all participating institutions conduct the study according to revisions approved by Certified Review Board since the date of entry into force.

Treatment and assessment of enrolled patients will be performed according to the pre-change version protocol until entry into force. Protocol deviations to enhance patient safety during treatment will be permitted if pre-change protocols, such as inadequate treatment modification criteria, threaten patient safety. If protocol deviations occur, they should be listed in the monitoring report.

2) Revision

Protocol change which meets all of the followings: i) does not have an increased risk for patients enrolled in the study; ii) does not have a substantial effect on primary endpoint of the study; iii) does not have an inherent effect on the system in which the study is conducted. Includes changes in protocols due to mistakes or changes in institution-specific information, changes in institution-specific information without changes in protocols (changes in Implementation Plans and jRCT registrations), and changes in conflicts of interest at individual institutions. In principle, suspension of patient registration is not performed in case of revision.

The revised version of the Protocol and Informed Consent Form version numbers are shown as in 1.1.0, 1.2.0, and 1.3.0….

Approval by the Group Chair and the Data Center Director must be obtained prior to submission to Data and Safety Monitoring Committee.

If classified as "Revision" by the Secretary-General of Data and Safety Monitoring Committee, the Secretary-General of Data and Safety Monitoring Committee issued a verification form, Data and Safety Monitoring Committee did not review the changes. The Principal Investigator submit an application for a change in the protocol to Certified Review Board through JCOG Operations Office (at this time, the version number is ver.1.1.0, 1.2.0, 1.3.0…). If the protocol was changed based on the indication by the Certified Review Board, version number shall be as ver.1.1.1, ver.1.1.2… When a protocol change is approved by Certified Review Board, the date of approval by the Certified Review Board should be entered on the protocol cover page. [If Specified Clinical Trials, the followings are added: "and a notification of the change in the Implementation Plan to the Regional Bureau of Health

and Welfare should be made"]

The date of entry into force of the protocol change will be 2 weeks after Certified Review Board approval date unless otherwise specified. [in the case of a Specified Clinical Trials, the followings are added: "The effective date shall be the date after the notification of change of the Implementation Plan to the Regional Bureau of Health and Welfare"]

The actual date of entry into force will be announced by JCOG Operations Office. Researchers in all participating institutions conduct the study according to revisions approved by Certified Review Board since the date of entry into force.

At that time, the site investigator should obtain permission by the administrator of each institution after the date of approval of Certified Review Board and before the date of entry into force. In this case, the protocol revision may be permitted with a report to the administrator of the institution. However, the procedures for obtaining permission from the administrator shall be in accordance with the regulations of each institution. For protocol revisions, confirm with the institution prior to initiation of the study to ensure that permission is obtained within the aforementioned time periods, and contact JCOG Operations Office if this is difficult. Reports to the administrator and approval form by the administrator at each institution need not be sent to the Data Center, but the original copy will be retained by the site coordinator as it will be checked during site visit audits.

Treatment and assessment of enrolled patients will be performed according to the pre-change version protocol until entry into force. Protocol deviations to enhance patient safety during treatment will be permitted if pre-change protocols, such as inadequate treatment modification criteria, threaten patient safety. If protocol deviations occur, they should be listed in the monitoring report.

3) Memorandum

Supplementary description of the protocol distributed from Principal Investigator/Study Coordinator to study personnel in objective, such as reduction of interpretive variation in sentences, and special precautions, rather than change of protocol content. Any form is used.

Approval by Group Chair and the Director of the Data Center is needed prior to distribution. Reporting to Data and Safety Monitoring Committee before distribution or immediately after distribution is required.

It is not necessary to include the protocol on the cover page.

13.6.2. Patient explanation and re-consent at the time of protocol amendment/revision

In the event of a change in the content of the study, the investigator or the subinvestigator shall provide appropriate explanation to the enrolled patients (regarding protocols based on revision, treatment, follow-up, etc.). In addition, if Certified Review Board comments that re-consent of enrolled patients in writing is required, informed consent should be obtained in writing.

13.7. Conflicts of Interest (COIs) involved in this study

13.7.1. COI management involved in this study

The COIs involved in this study will be managed according to the "Guidance for Conflict of Interest Management in Clinical Trials Act (Notification No.1130-17 of the PMSB dated November 30, 2018) ※ (Guidance) of the Division, Research and Development, Ministry of Health, Labour and Welfare, in accordance with the following:

※ http://www.mhlw.go.jp/stf/seisakunitsuite/bunya/0000163417.html

The format used for COI control should be the latest version of the guidance.

- Conflict of Interest Management Standards: Form A
- Reports of related companies, etc.: Form B
- Investigator Conflict of Interest Self-Report Form: Form C
- Conflict of Interest Confirmation Report: Form D
- Conflict of Interest Management Plan; Form E

1) Procedures for New Application

Conflict of Interest Management Standards

(i) All JCOG trials according to Clinical Trials Act will employ Conflict of Interest Management Standards (Form A) in accordance with guidance.

Request for Confirmation of Conflicts of Interest

(ii) At the time of initiation of the primary review of the protocol, JCOG Operations Office shall identify companies, etc. related to this study that require conflict of interest management based on information on drugs and medical devices specified in the Protocol Treatment (see 6.1.), enter the relationship with the company in Form B, and ask Principal Investigator to confirm the accuracy of the entry.

(iii) After checking the entries in Form B received from JCOG Operations Office, Principal Investigator will send a format to the site investigator and site coordinator at each participating institution and ask to confirm the entries in Form B, create Form C, and create Form E. In addition, a form shall be sent from Principal Investigator to those who correspond to the "person responsible for statistical analysis" and the "administrator other than Principal Investigator and Investigators" of the Implementation Plan, and they shall be asked to prepare Forms C and E.

Confirmation of Conflicts of Interest (participating institutions)

(iv) The site investigator or site coordinator at each participating institution will register the investigators, subinvestigators, and research associates involved in the study in JCOG Web Entry System. Investigators and subinvestigators registered in JCOG Web Entry System will be the reporters of conflicts of interest in the study.

(v) The site coordinator will request the investigator of the study to confirm the description of Form B received from Principal Investigator, create Form C, and create Form E. The investigator will then summarize conflict of interest management within the study site.

(vi) The investigator checks the forms received from Principal Investigator for any relevant items in Q2 through Q5 of Form B and notifies Principal Investigator through JCOG Operations Office within a week if any.

(vii) In the column of Form C [Persons requiring a Form C self-declaration of conflict of interest (Form C)] ※, the site investigator inputs the information of the investigator and subinvestigator registered in JCOG Web Entry System, and asks subinvestigator to prepare Form C.

※ The information entered in Form C is automatically entered in Form E. This entry should be consistent with the physician's information contained in the "List of Subinvestigators" (Clinical Trials Act Uniform Form No.1) submitted to Certified Review Board with Form E, and physicians who do not agree may not be involved in this study. Therefore, when entering the information in the ※ column for [those who require a Conflict of Interest Self-Reporting Form (Form C)], the investigator or subinvestigator list should be downloaded in JCOG Web Entry System and the physician listed in the Study Subinvestigator list should be entered without missing the person who will be the investigator and the subinvestigator in the study. If the information in the downloaded "Study Subinvestigator List" is not consistent with the physician involved in the study, the registered information should be updated in JCOG Web Entry System, and the "Study Subinvestigator List" with the most recent information reflected should be downloaded and used.

(viii) The investigator and the subinvestigator shall complete the necessary information regarding the relationship with the company, etc. described in advance in Form C, and submit Form C to the Conflict of Interest Confirmation Department of the institution to which he/she belongs. In doing so, the investigator also submits Form A.

(ix) The investigator receives the results of confirmation of conflicts of interest (Form D) of the investigator and the study subinvestigator from the institution to which he/she belongs.

(x) The investigator will confirm the content of Form A, Form B, and Form D of the investigator and all study subinvestigators to create Form E. The investigator will notify Principal Investigator through JCOG Operations Office of Form E and the Study Subinvestigator List downloaded in Form 7.

Confirmation of Conflicts of Interest (outside participating institutions)

(xi) The person responsible for statistical analysis and the person overseeing a study other than Principal Investigator or investigator shall complete, in the form received from Principal Investigator, the necessary information regarding the relationship with the company, etc. described in advance in Form C, and submit Form A and Form C to the Conflict of Interest Confirmation Department of the Affiliated Institution.

(xii) The person responsible for statistical analysis and the person overseeing studies other than Principal Investigator or investigator shall receive confirmation results (Form D) from the institution.

(xiii) The person responsible for statistical analysis and the person overseeing studies other than Principal Investigator and investigator shall confirm the content of Form A and Form D, prepare Form E, and notify Form E to Principal Investigator through JCOG Operations Office.

Description of Conflicts of Interest in the protocol and informed consent form

(xiv) Principal Investigator and JCOG Operations Office will review Form A and Form E received from participating institutions and, as appropriate, will accurately describe the conflicts of interest (study COIs) between the study and the drug marketing authorisation holder, etc. in the protocol and informed consent form. Conflicts of interest (personal COI) between the reporters of conflicts of interest in this study and the marketing authorisation of drugs, etc. (personal COI) can change over time, so the personal COI is not described in the protocol and informed consent form, and the information is updated on JCOG website as needed.

Certified Review Board review

(xv) Principal Investigator compiles and submits the Form E and Study Subinvestigator List of all institutions to Certified Review Board for review.

2) Procedures for new involvement with companies after the start of the study

(xvi) When a new involvement with companies (research COI) occurs in the study after the start of the study

a. In the event of any change in the enterprises involved in this study, etc. to be described in Q1 of Form B, repeat the procedures ② to ⑭. However, the site investigator and site coordinator roles in ④, ⑤ shall be assumed by the investigator at each institution. If a new study COI needs to be added to the protocol and informed consent form, a protocol revision (see 13.X.X.) should be submitted to Certified Review Board for review.

b. If a change occurs from Q2 to Q5 in Form B, the investigator at the participating institution affected by the change will change the appropriate description in Form B and update Form E and send it to Principal Investigator and JCOG Operations Office. A Principal Investigator who has received a Form E will revise the protocol as needed (see 13.X.X.) and submitted to Certified Review Board for review.

(xvii) In the event that a conflict of interest reporter is newly involved with a company (personal COI) after the start of the study

Conflict of interest reporters repeat procedures from ⑧ to ⑩ or from ⑪ to ⑬. However, if there is no change in Form E, these procedures will be performed at each institution but will not be sent to Principal Investigator and JCOG Operations Office. Principal Investigator will submit Post-change Form E sent from the site investigator to Certified Review Board for review. Personal COIs are also disclosed on JCOG website.

3) Procedures for periodic reporting

(xviii) Principal Investigator checks for changes in study COI and personal COI annually at the timing of periodic reporting and reports to Certified Review Board.

13.7.2. COI with companies involved in this study(study COI)

There are no conflicts of interest to disclose about relationship between the company manufacturing and marketing drugs specified as a part of protocol treatments in this study (See 6.1).

13.7.3. COIs of JCOG Committee members and JCOG Data Center/Operations Office staff

COIs of the committee members of JCOG committees, the staffs of the office of committees, and JCOG Data Center/Operations Office staff involved in the study will be managed by JCOG Conflict of Interest Committee in accordance with Clinical Trials Act control standards.

13.8. Compensation

In order to comply with Clinical Trials Act, this study must take necessary measures such as sing up for insurance and ensuring a system to provide medical care in order to compensate for the health damage caused by the study

and provide medical care.

Therefore, regarding the health damage caused by participating in this study, appropriate treatment according to the condition is provided similarly to the usual insurance medical care. In addition, this study will be covered by clinical study insurance, and the following will be compensated based on insurance conditions, and this will be explained to patients and understood.

13.9. Intellectual property

The results, data, and intellectual property rights obtained from this study are attributed to the followings: National Cancer Center, Principal Investigator, Study Coordinator, and Group Chair. Specific procedures and allocation must be determined through consultation among four parties. Whether the intellectual property related to Principal Investigator, Study Coordinator, Group Chair will belong to the individuals or the affiliated institution will be determined according to the agreements of the affiliated institution.

13.10. Disclosure of information on this study

Summary, progress, and main results of this study will be published on JCOG website (www.jcog.jp) and on jRCT (https://jrct.niph.go.jp/).

14. Monitoring and audit

14.1. Periodic monitoring

In this study, monitoring is performed in order to ensure that the study is conducted safely and in accordance with this protocol and that data are collected accurately. Periodic monitoring should be performed twice a year in principle, using the entered data on the CRFs collected in the Data Center. Specific procedures for periodic monitoring are provided separately in the Monitoring Plan.

The Data Center submits a "Monitoring Report" summarizing the results of central monitoring to Principal Investigator, Study Coordinator and investigators. Together, they are submitted to Group Chair, Data and Safety Monitoring Committee, and JCOG Chair.

The Monitoring Report is a material for periodic reports to be made every year from the date of submission of the Implementation Plan.

14.1.1. Monitoring items

① Registration status: number of registration - cumulative/by month, by arm/site
② Eligibility: ineligibles/potentially ineligible cases: by arm/site
③ Pre-treatment baseline factors: by arm
④ On/off-treatment, reason for treatment termination: arm/site
⑤ Protocol deviation: arm/site
⑥ Serious Adverse Events: arm/site
⑦ Adverse reaction/event: arm
⑧ Overall survival, progression-free survival (or relapse-free survival, etc.): all registered patients
⑨ Other issues related to study progress and safety (studies in accordance with Clinical Trials Act: status of occurrence of non-compliance and subsequent response, number of subjects for compensation, number of disease or the like reports in accordance with Article 13 of Clinical Trials Act)

14.1.2. Eligibility (Eligible/Ineligible)

For all registered patients, eligibility will be classified according to the following definitions as either: In monitoring, Data Center lists potentially ineligible cases in the "Evaluation of Eligibility" section of the monitoring report, and after review by Study Coordinator, confirms them to be either 1), 2), 9), or 99) with Group Chair approval prior to performing primary analysis.

Only 1) eligible shall be "eligible case", 2) post hoc ineligible,9) de facto ineligible and 99) violation of registration shall be "ineligible case". This is a category established from the perspective of analysis set setting.

In the study in accordance with Clinical Trials Act, "99) violation of registration" will be regarded as "major non-compliance" in Clinical Trials Act, and Principal Investigator will promptly report the situation to Certified Review Board as soon as possible. See 14.3. for management of non-compliance.

9) de facto ineligibles corresponds to "non-compliance" on Clinical Trials Act and is reported to the administrator of participating medical organizations with the submission of the monitoring report on which they were described (twice a year).

2) Post hoc ineligibles is not treated as "non-compliance" on Clinical Trials Act because it does not correspond to non-compliance with the study protocol, as discussed below.

1) Eligible

All information generated prior to registration meets all o the Patients Selection Criteria according to the methods and criteria specified in the study protocol.

2) Post hoc ineligible

The information generated after registration does not meet either Patients Selection Criteria, or the information generated prior to registration does not meet either Patients Selection Criteria by methods or criteria other than those specified in the protocol.

Examples)

(i) In the study for Stage II-III, bone scintigraphy performed immediately after registration revealed bone

metastases, and the patient was diagnosed as Stage IV. The protocol treatment was terminated.

(ii) In the study for early gastric cancer, bloody stools is seen after registration, and colonoscopy revealed advanced colorectal cancer (synchronous double cancer). Colectomy was performed after termination of the protocol treatment.

(iii) In the study for gastric cancer (adenocarcinoma), the institution's pathological diagnosis was changed to malignant lymphoma after registration.

9) De facto ineligible

Information generated prior to registration according to protocol-specified methods (performed in all cases) and criteria does not meet either Patients Selection Criteria. This includes cases where it is determined after registration that the information that occurred before registration had been incorrect.

Example: When the supervising physician reviews the CT images performed before registration as specified, there is obvious liver metastasis (if it is a mistake by the attending physician and it is considered that there is no future).

99) Violation of registration

Deliberately (falsely) enroll while knowing that Patients Selection Criteria is not met. Corresponds to a misconduct and treats it as a serious problem.

14.1.3. Protocol Deviations/Violations

Protocol deviations are defined as those in which treatment, such as drug administration, radiotherapy, or surgical resection, as well as laboratory tests and evaluation of toxicity and efficacy, etc. were not performed according to the protocols.

In monitoring, deviations that exceed a certain acceptable range limit for each study decided by the Data Center and Principal Investigator/Study Coordinator prior to or after the initiation of the study are listed in the monitoring report as "possible deviations" and are classified into one of the following categories after consideration by Study Coordinator and study groups: Except for those described in the protocol and monitoring report, the acceptable deviations agreed between the Data Center and Principal Investigator/Study Coordinator may be changed through periodic monitoring during the study, so they should be described in the supplemental material rather than in the text of the Monitoring Plan, and the Monitoring Plan should be described as "defining the acceptable ranges separately".

1) Violation

Any deviation from the protocol that is clinically inappropriate and caused by the treating physician/institution and that meets two or more following criteria shall be classified as a violation. [When conducted in accordance with Clinical Trials Act, the following shall be added. [In the study according to Clinical Trials Act, the violation shall be treated as "major non-compliance" and Principal Investigator should report the situation to Certified Review Board as soon as possible].

① Have a substantial impact on the assessment of study endpoints

② Intentional or systematic

③ Dangerous or remarkable deviation

For "violations", in principle, the content of each violation should be described in a paper when publishing.

2) Deviation

Deviations that do not fall into 1) violation or 3) acceptable deviation. If same kind of deviations are frequent, they should preferably be included in the publication of the article. They are classified as either of the following at the time of monitoring report review:

Because deviations correspond to "non-compliance" in Clinical Trials Act, they are reported (biannually) to the administrators of the institution with the submission of the monitoring report. Not to be "major non-compliance".

(i) Deviations - Undesirable and to be reduced

(ii) Deviations (unavoidable) - things that are not proactively reducing (e.g. delay by the New Year period, equipment breakdown, etc.)

(iii) Deviations (clinically relevant) - Those in which the decision of the treating physician/institution are positively affirmed (if a similar situation again arises similar deviations are considered desirable).

※ Deviations do not always mean that the treating physician at the institution is problematic. Since patient safety is a primary priority in clinical trials, deviations should rather be made by the medical judgment of the treating physician if the condition of the individual patient are considered to be dangerous when following the protocols. If the deviation is judged to be clinically relevant for the safety of the patient, it is recorded as ③ Deviation (clinically relevant). Clinically relevant deviations in a small number of patients are not required to be particularly problematic; however, protocol revision should be considered when multiple deviations occur because protocol specification is likely to be inadequate. However, deviations that are not intended to be safety (e.g., increased doses of anticancer drugs in the hope of increasing efficacy, shortened treatment periods not specified in the protocol) are not considered clinically relevant deviations.

3) Acceptable deviation

Deviations from protocols within acceptable range agreed by the entire JCOG, study groups, or Study Chair/Study Coordinator and Data Center, pre- or post-study initiation, on a trial-by-trial basis. When conducted according to Clinical Trials Act, the following are added. Not considered to be "non-compliance" in Clinical Trials Act. Deviations within the pre-specified acceptable ranges are not included in the monitoring report.

14.2. Site visit audits

In this study, site visit audits will be conducted to ensure the reliability of clinical research and the reliability of data and information collected by clinical research from the perspective of protecting human subjects in clinical research.

Site visit audits are conducted by auditors appointed by Principal Investigator by visiting a medical institution to confirm the approval documents of the medical institution, check the list of subinvestigators in the research institution, confirm the informed consent documents, and verify CRF entry data with medical records (direct access to source documents). Specific procedures for site visit audits are provided separately in the operating procedures.

The auditor shall report the audit report summarizing the audit results to Principal Investigator/Study Coordinator, site investigators. Together, the report will be submitted to Group Chair, Director of JCOG Data Center, Director of JCOG Operations Office, and JCOG Chair. Reports should also be submitted to the site investigators of the relevant groups and JCOG Executive Committee as appropriate.

14.2.1. Items to be audited

In the site visit audits, the following items are checked by direct access to source documents:

<Confirmation Items by Study>

- Approval documents (including initial approval forms, revision approval forms, and annual report approvals/reports) from administrators of medical institutions
- Management status of the protocol
- Contents of explanatory documents and informed consent forms

<Confirmation Items by Patient>

- Patient consent (presence or absence of consent form, signature, and date of consent)
- Implementation of pre-registration mandatory tests, eligibility for registration (inclusion criteria and exclusion criteria)
- Accuracy of reported data
    - Pre-treatment evaluation, course of treatment (protocol treatment)
    - Various test results (including diagnostic imaging reports and pathology reports)
    - Accuracy of test date, response evaluation, adverse event, survival or death information, and other reported data

<Other items>

- Presence or absence of study misconduct (possibility of false reporting, fabrication, or falsification)
- Improvement status of the items pointed out in the previous audit

14.2.2. Reporting of major non-compliance found in audits to Certified Review Board

Principal Investigator/Study Coordinator should report to Certified Review Board immediately when finding possible "major non-compliance (See 14.3.2.)" as a result of site visit audits.

14.3. Management of non-compliance

14.3.1. Non-compliance.

Non-compliance in Clinical Trials Act refers to the condition in which clinical research is not compliant with Clinical Trials Act Enforcement Regulations or study protocols. In the Clinical Trials Act Enforcement Regulations, etc. (February 28, 2018), non-compliance with regulations, study protocols, operation procedures, etc., and fabrication of falsification of research data, etc. are listed as examples.

If the site investigator knows that there is non-compliance, the site investigator should report to the administrator of the medical institution and inform Principal Investigator/Study Coordinator.

If site investigator find non-compliance (regardless of major non-compliance or not) prior to the implementation of central monitoring or site visit audits, the site investigator will promptly report to Principal Investigator/Study Coordinator and JCOG Data Center.

"14.1.3.2) Deviations" correspond to "non-compliance" in Clinical Trials Act. As described in 14.1.3.2), these deviations are reported by submitting monitoring reports or their excerpts or summaries issued twice a year to the administrators of the medical institution.

Changes in protocols and implementation plans associated with investigator transfer require Certified Review Board review and notification of implementation plans to the MHLW, therefore, require a certain time to complete the sequence of procedures. In addition, it is often difficult to complete the change procedure before the transfer because the transfer may not be open until just before the transfer. Therefore, even if the site investigator is absent for a certain period of time due to the transfer, the study does not fall into "non-compliance" in this study if the research management system is maintained by the subinvestigator and the medical care system of the enrolled patients who are surviving is ensured.

14.3.2. Major non-compliance

"Major non-compliance" is that affect the human rights and safety of subjects of clinical research, the study progress and the reliability of study results. Examples of "major non-compliance" in JCOG study are provided in. If major non-compliance is likely, Principal Investigator/Study Coordinator will report the situation to Certified Review Board immediately.

1) Major non-compliance with respect to eligibility

Violation of registration

- Enrolled intentionally (falsely) while knowing that eligibility criteria was not met
- Patient enrollment was performed without necessary informed consent, and protocol treatment was performed
- The source documents for the determination of eligibility cannot be identified (including the loss of the consent form).

2) Protocol violation

Violations that affect the increased risk of enrolled patients or that affect the reliability of the study results

- Significant violation in inclusion criteria or exclusion criteria
- Violation threatening patient safety in off-treatment criteria
- Serious violation of prohibited concomitant drug, prohibited concomitant treatment, etc.
- e.g. intentional or systematic non-compliance with protocol regulations

3) Other major non-compliance

- Study was conducted prior to Certified Review Board approval or prior to approval of site administrator
- The study was continued without providing information to the enrolled patient that could affect the willingness to continue to participate the study.
- Those judged to be research misconduct (fabrication or falsification of data, etc.)

- Any leakage of personal information or violation of human rights that may have a significant impact on the enrolled patient.

15. Special Instructions

15.1 Central pathology diagnosis and related matters

15.1.1 Central pathological diagnosis

In this study, the pathological tissues of enrolled patients will be collected and the central pathological diagnosis will be determined after the fact. Since the central pathological diagnosis is not performed in real time for each individual patient enrollment, the main analysis target and the decision on treatment strategy will be based solely on the pathological diagnosis at the institution. The details of the central pathological diagnosis procedure will be specified separately in the Central Pathological Diagnosis Procedure Manual.

Timing: Once a year throughout the study period.

Subjects: All registered patients

Methods: Pathological specimens (or duplicates made from the same paraffin block) used for eligibility criteria determination at the registered facilities will be collected, and after necessary staining (chromogranin A, synaptophysin) is added, pathological eligibility will be re-determined by two or more pathology judges (16.8 Pathology judges) appointed by the group representative. (16.8 Central Pathological Review Comittie).

Management of the collected specimens: The Hepatobiliary and Pancreatic Group Study Office will be responsible for the management of the collected specimens.

Staining: If additional staining is required, it will be performed by the Central Pathology Coordinator (16.7).

Notification of the central judgment to each facility:

After the results of the central pathological diagnosis are fixed, the research secretariat will notify the results of the judgment to the registered facility (facility coordinator) of each patient. At that time, the rationale for the decision should be attached in writing. If there is a difference between the institutional diagnosis and the central diagnosis, the facility principal investigator/facility coordinator reports the decision results to the facility pathologist and discusses it with the facility pathologist, and carefully decides on the final pathological diagnosis at the facility (whether to change the diagnosis or not) and what to do if the patient is under treatment (whether to change the treatment or not).

15.1.2 Providing information to the institutional pathologist

In view of the high level of difficulty in NEC pathological diagnosis and the unique nature of this study, which covers multiple target organs, the following information will be provided to institutional pathologists.

(1) Pre-registration pathological diagnosis consultation

Since it is anticipated that there will be cases of confusion in diagnosis at facilities, pre-enrollment pathology consultation will be available as needed, with the pathology judgment committee members of this study serving as consultants. Details of the consultation operation are described in the Central Pathology Procedures.

(2) Holding of pathology-related meetings

In this study, the research secretariat and the pathology secretariat first held a pathology-related meeting (attended by pathologists from participating institutions) on February 11, 2014, during which a lecture was given to form a consensus on diagnostic criteria according to the WHO 2010 classification. The slides used at the meeting were revised as necessary based on the discussion at the meeting and posted on the JCOG website for reference by pathologists at each facility.

15.1.3 Response to cases in which pathological diagnosis was performed at a facility other than the home facility

In any of the following cases, be sure to ask a pathologist at your own institution to make the diagnosis, and confirm that the diagnosis at your institution is also NEC before enrolling in this study.

(1) When borrowing only stained tissue specimens from a previous physician for registration

Register after obtaining permission from the attending physician to the previous physician to submit the borrowed tissue specimen to JCOG 1213 for central diagnosis (loan again) and to borrow the specimen for a long period of time (up to about 1 year).

(2) If you have received virtual slides of "all tissue specimens for which a pathological diagnosis has been made" from

your previous doctor

The patient will be registered after obtaining permission from the attending physician of the previous physician to submit the virtual slides provided by the previous physician for the central diagnosis of this study.

(3) If you have received a "borrowed" pre-stained tissue specimen and an "offered" unstained preparative from your previous physician

The borrowed stained tissue specimens from the previous physician can be promptly reviewed at the patient's own institution, but the provided unstained preparations require time for pathological diagnosis after immunostaining at the patient's own institution, which may cause a time lag. There have been cases in which patients were enrolled in the study based only on the results of pathological review of borrowed tissue specimens at their own institutions, and later the results of staining and pathological diagnosis of undyed preparations at their own institutions overturned the pathological diagnosis (diagnosis of a different disease was made), resulting in post-hoc ineligible cases. Therefore, we will consider the timing of registration after sufficient consultation with pathologists at our own institution to avoid the occurrence of posterior ineligible cases as much as possible. However, if there is enough time, it is preferable to enroll unstained preparations into the study after staining and pathological diagnosis is obtained at your own institution.

15.2. JCOG BioBank Japan (BBJ) Biorepository

This study will participate in the banking of blood samples (DNA/plasma) in JCOG BBJ Biorepository based on a common protocol for all JCOG studies (hereafter referred to as common banking).

Subjects:

Among patients who agreed to participate in this study, patients whose consent to shared banking was obtained.

Sample:

1) Whole blood

Blood sampling is performed before the start of the protocol treatment in this study in principle. However, blood sampling after initiation of protocol treatment is allowed. Blood samples of 7 mL×2 (total 14 mL) of venous blood are collected using a blood collection tube (for blood counting) with a EDTA Na dedicated to the shared banking of JCOG-BBJ Biorepository, and stored at 4°C at the respective institutions until they are handed over to the sample transport/processing company (See "JCOG-Biobank Japan Biorepository protocol" for details).

2) Pathological specimens

Archived pathological tissues in daily clinical practice such as surgery, biopsy and laboratory tests can also be used in future translational researches, but the type of pathology specimen, preparation method and tissue quantity required by the studies are varied and there is no consensus that it is efficient to bank pathological tissues in a certain way prospectively. In addition, there is the opinion that the sample deteriorates (DNA fragmentation) when the thin-section sample from pathological tissue is stored for a long time.

Consent on the use of archival pathological tissue after medical care should therefore be obtained at the time of consent to banking, but actual collection should be initiated on an individual basis by creating a protocol and defining the most appropriate procedure for the study content in the protocol.

16. Organization

Changes to this chapter are considered to be revision rather than amendment.

16.1. Main study fund (funding source) of this study.

Practical Research for Innovative Cancer Control from Japan Agency for Medical Research and Development “Establishment of standard treatments for neuroendocrine carcinoma of the digestive system” JP15ck0106138, JP16ck0106138, JP17ck0106355, JP18ck0106355, JP19ck0106355, JP20ck0106618

National Cancer Center Research and Development Fund (23-A-22, 26-A-4, 29-A-3, 2020-J-3)

“Scientific research on multi-institutional trials to establish new standard treatment of solid tumors in adults”

16.2. Japan Clinical Oncology Group (JCOG)

JCOG is a multi-institutional clinical research group consisting of research teams funded by public research grants

mainly on National Cancer Center Research and Development Fund and Japan Agency for Medical Research and Development research costs that receive direct support for research by the Clinical Research Support Office of the National Cancer Center Hospital in accordance with JCOG Policy (http://www.jcog.jp/).

This study is conducted using JCOG research organisations and in accordance with the regulations set out by JCOG Executive Committee.

16.3. JCOG Chair

Yuichiro Ohe National Cancer Center Hospital

16.4. Study group and Group Chair

JCOG Hepatobiliary and Pancreatic Oncology Group
Group Chair: Junji FURUSE
Department of Medical Oncology, Kyorin University Faculty of Medicine
〒181-8611 6-20-2, Shinkawa Mitaka-shi, Tokyo
TEL:+81-422-47-5511
FAX:+81-422-44-1858
E-mail: jfuruse@ks.kyorin-u.ac.jp
Group Secretary: Hirohi Ishii
Clinical Research Center, Chiba Cancer Center

〒260-8717, 666-2 Nitona, 260-8717, Chuo-Ku, Chiba
TEL:+81-43-264-5431
FAX:+81-43-264-8680
E-mail: hirishii@chiba-cc.jp
JCOG Stomach Cancer Study Group:
Group Chair: Masanori Terashima
Shizuoka Cancer Center
〒411-8777 1007 Shimonagakubo, Nagaizumi-cho, Sunto-gun, Shizuoka Prefecture
TEL:+81-55-989-5222
FAX:+81-55-989-5783
E-mail: m.terashima@scchr.jp
Group Secretary: Takaki Yoshikawa
National Cancer Center Hospital
〒104-0045 5-1-1 Tsukiji, Chuo-ku, Tokyo
TEL:+81-3-3542-2511
FAX:+81-3-3542-3815
E-mail: tayoshik@ncc.go.jp
Group Secretary: Narikazu Boku
National Cancer Center Hospital
〒104-0045 5-1-1 Tsukiji, Chuo-ku, Tokyo
TEL:+81-3-3542-2511
FAX:+81-3-3542-3815
E-mail: nboku@ncc.go.jp

JCOG Japan Esophageal Oncology Group
Group Chair: Yuko Kitagawa
Keio University Hospital
〒160-8582 35 Shinanomachi, Shinjuku-ku, Tokyo
TEL:+81-3-3353-1211
FAX:+81-3-3355-4707
E-mail: kitagawa@a3.keio.jp

Group Secretary: Ken Kato
National Cancer Center Hospital
〒104-0045 5-1-1 Tsukiji, Chuo-ku, Tokyo
TEL:+81-3-3542-2511
FAX:+81-3-3542-3815
E-mail: kenkato@ncc.go.jp

16.5. Study Chair (Principal Investigator)

JCOG Hepatobiliary and Pancreatic Oncology Group
Takuji Okusaka
National Cancer Center Hospital
〒104-0045 5-1-1 Tsukiji, Chuo-ku, Tokyo
TEL: +81-3-3542-2511
FAX: +81-3-3542-3815
E-mail: tokusaka@ncc.go.jp

JCOG Stomach Cancer Study Group:
Narikazu Boku
National Cancer Center Hospital
〒104-0045 5-1-1 Tsukiji, Chuo-ku, Tokyo
TEL: +81-3-3542-2511
FAX: +81-3-3542-3815
E-mail:nboku@ncc.go.jp

JCOG Japan Esophageal Oncology Group
Ken Kato
National Cancer Center Hospital
〒104-0045 5-1-1 Tsukiji, Chuo-ku, Tokyo
TEL: +81-3-3542-2511
FAX: +81-3-3542-3815
E-mail: kenkato@ncc.go.jp

16.6. Study Coordinator

JCOG Hepatobiliary and Pancreatic Oncology Group
Chigusa Morizane
National Cancer Center Hospital
〒104-0045 5-1-1 Tsukiji, Chuo-ku, Tokyo
TEL: +81-3-3542-2511
FAX: +81-3-3542-3815
E-mail: cmorizan@ncc.go.jp

JCOG Stomach Cancer Study Group:
Nozomu Machida
Shizuoka Cancer Center
〒411-8777 1007 Shimonagakubo, Nagaizumi-cho, Sunto-gun, Shizuoka Prefecture
TEL:+81-55-989-5222
FAX:+81-55-989-5631
E-mail：no.machida@scchr.jp
JCOG Japan Esophageal Oncology Group
Ken Kato

National Cancer Center Hospital
〒104-0045 5-1-1 Tsukiji, Chuo-ku, Tokyo
TEL: +81-3-3542-2511
FAX: +81-3-3542-3815
E-mail: kenkato@ncc.go.jp

Yoshitaka Honma
National Cancer Center Hospital
〒104-0045 5-1-1 Tsukiji, Chuo-ku, Tokyo
TEL: +81-3-3542-2511
FAX: +81-3-3542-3815
E-mail:yohonma@ncc.go.jp

16.7. Central Pathological Review Coordinator

Nobuyoshi Hiraoka
National Cancer Center Hospital
〒104-0045 5-1-1 Tsukiji, Chuo-ku, Tokyo
TEL: +81-3-3542-2511
FAX: +81-3-3542-3815
E-mail: nhiraoka@ncc.go.jp

Hirokazu Taniguchi
National Cancer Center
〒104-0045 5-1-1 Tsukiji, Chuo-ku, Tokyo
TEL: +81-3-3542-2511
FAX: +81-3-3542-3815
E-mail: hitanigu@ncc.go.jp

16.8. Central Pathological Review Coordinator

Nobuyoshi Hiraoka (National Cancer Center Hospital)
Noriyoshi Fukushima (Jichi Medical University)
Nobuyuki Ohike (Showa University Fujigaoka Hospital)
Ryoji Kushima (Shiga University of Medical Science)
Mitsuya Iwafuchi (Scool of Health Sciences Faculty of Medicine, Niigata University)
Tetsuo Ushiku (The University of Tokyo)

16.9. Participating sites (participating institutions)

HBP	SCSG	JEOG	Participating institutions
◯			Sapporo-Kosei General Hospital
◯		◯	Hokkaido University Hospital
	◯		Keiyukai Sapporo Hospital
	◯	◯	Iwate Medical University
		◯	Tohoku University Hospital
	◯		Miyagi Cancer Center
◯	◯	◯	Tochigi Cancer Center
◯			Jichi Medical University
◯	◯	◯	Saitama Cancer Center
◯	◯	◯	National Cancer Center Hospital East
◯		◯	Chiba Cancer Center
◯		◯	Chiba University, Graduate School of Medicine
◯	◯	◯	National Cancer Center Hospital
◯			Kyorin University Faculty of Medicine
◯			National Center for Global Health and Medicine (NCGM)
◯	◯	◯	Cancer Institute Hospital of Japanese Foundation for Cancer Research
	◯	◯	Toranomon Hospital
◯			Teikyo University School of Medicine
◯			St.Marianna University School of Medicine
◯	◯	◯	Kanagawa Cancer Center
◯	◯		Yokohama City University Medical Center
◯	◯		Niigata Cancer Center Hospital
◯			Toyama University Hospital
◯			Kanazawa University School of Medicine
	◯		Ishikawa Prefectural Central Hospital
	◯		Ogaki Municipal Hospital
◯	◯	◯	Shizuoka Cancer Center
◯	◯	◯	Aichi Cancer Center Hospital
		◯	Kyoto University Hospital
	◯	◯	Osaka University Graduate School of Medicine
◯	◯		Kindai University Hospital
◯	◯	◯	Osaka International Cancer Institute
◯	◯	◯	National Hospital Organization Osaka National Hospital
	◯	◯	Osaka General Medical Center
	◯	◯	Osaka Medical and Pharmaceutical University
	◯		Osaka Rosai Hospital
◯	◯	◯	Kobe University Graduate School of Medicine
	◯	◯	Kansai Rosai Hospital
◯	◯		Hyogo College of Medicine
◯	◯	◯	Hyogo Cancer Center
	◯		Itami City Hospital
	◯		Shimane University Faculty of Medicine
	◯	◯	Hiroshima University Hospital
	◯		Fukuyama City Hospital
	◯		Tokushima Red Cross Hospital
◯	◯	◯	National Hospital Organization Shikoku Cancer Center
◯		◯	Kochi Health Sciences Center
◯		◯	National Kyushu Cancer Center
◯		◯	Kyushu University Hospital

	◯	◯	Oita University Faculty of Medicine

HBP: Hepatobiliary and Pancreatic Oncology Group
SCSG: Stomach Cancer Study Group:
JEOG: Japan Esophageal Oncology Group

16.10. JCOG Protocol Review Committee

This protocol was approved by JCOG Protocol Review Committee prior to submit to Certified Review Board.
(For membership, see website http://www.jcog.jp/basic/org/committee/protocol.html)
Contact: Protocol Review Committee Office

JCOG Operations Office/Clinical Research Support Office, National Cancer Center Hospital
〒104-0045 5-1-1, Tsukiji, Chuo-ku, Tokyo
TEL: 03-3542-2511 (ext. 2302)
FAX:03-3542-7006
E-mail: jcogoffice@ml.jcog.jp

16.11. JCOG Data and Safety Monitoring Committee

During study period, the study will be monitored by Data and Safety Monitoring Committee (e.g., adverse event reports, interim analysis reviews, monitoring report reviews, protocol revision reviews). However, the committee members from the study group conducting this study do not participate directly in the review of this study.
(For membership, see website http://www.jcog.jp/basic/org/committee/jury.html)
Contact: JCOG Data and Safety Monitoring Committee Office

JCOG Operations Office/Clinical Research Support Office, National Cancer Center Hospital
〒104-0045 5-1-1, Tsukiji, Chuo-ku, Tokyo
TEL:03-3542-2511 (ext. 2403)
FAX:03-3542-7006
E-mail: jcogoffice@ml.jcog.jp

16.12. JCOG Audit Committee

Site-visit audits by Audit Committee will be conducted during study period.
(For membership, see website http://www.jcog.jp/basic/org/committee/audit.html)
Contact: JCOG Auditing Committee Office

JCOG Operations Office/Clinical Research Support Office, National Cancer Center Hospital
〒104-0045 5-1-1, Tsukiji, Chuo-ku, Tokyo
TEL:03-3542-2511 (ext. 2403)
FAX:03-3542-7006
E-mail: jcogoffice@ml.jcog.jp

16.13. JCOG Conflict of Interest Committee

During study period, JCOG investigators involved in this study will be managed by the Conflict of Interest Committee.
(For membership, see website http://www.jcog.jp/basic/org/committee/coi.html)
Contact: JCOG Conflict of Interest Committee Office

JCOG Operations Office/Clinical Research Support Office, National Cancer Center Hospital
〒104-0045 5-1-1, Tsukiji, Chuo-ku, Tokyo
TEL:03-3542-2511 (ext. 2404)
FAX:03-3547-1002
E-mail: jcogoffice@ml.jcog.jp

16.14. Data Center/Operations Office

JCOG Data Center

Director of Data Center: Haruhiko Fukuda
Clinical Research Support Office, National Cancer Center Hospital

〒104-0045 5-1-1, Tsukiji, Chuo-ku, Tokyo
TEL:03-3542-3373
FAX:03-3542-3374
E-mail: jcogdata@ml.jcog.jp

JCOG Operations Office
Director of Operations Office: Kenichi Nakamura
Clinical Research Support Office, National Cancer Center Hospital
〒104-0045 5-1-1, Tsukiji, Chuo-ku, Tokyo
TEL:03-3547-1002
FAX:03-3547-1002
E-mail: jcogoffice@ml.jcog.jp

Official website: http://www.jcog.jp/

16.14.1. Data management administrator

Data management organization		JCOG Data Center
Data management administrator	Name	Harumi Kaba
	e-Rad number	40543442
	Affiliation	Clinical Research Support Office, National Cancer Center Hospital
	Title	Head of Multi-institutional Data Management Section

16.14.2. Monitoring administrator

Monitoring organization		JCOG Data Center
Monitoring administrator	Name	Haruhiko Fukuda
	e-Rad number	70263390
	Affiliation	Clinical Research Support Office, National Cancer Center Hospital
	Title	Chief of Data Management Division

16.14.3. Site-visit audit administrator

Auditing organization		JCOG Operations Office
Site-visit audit administrator	Name	Kenichi Nakamura
	e-Rad number	40543533
	Affiliation	Clinical Research Support Office, National Cancer Center Hospital
	Title	Director of Research Management Division

16.14.4. Statistical analysis administrator

Statistical analysis organization		JCOG Data Center
Statistical analysis Administrator	Name	Junki Mizusawa
	e-Rad number	60706646
	Affiliation	Biostatistics Division, Center for Research Administration and Support, National Cancer Center/<br>Biostatistics Section, Research Management Division, Clinical Research Support Office, National Cancer Center Hospital
	Title	Biostatistics Section Head

16.14.5. Research and development plan support personnel

Organizations in charge of supporting research and development plans		JCOG Operations Office
Research and development plan support personnel	Name	Tomoko Kataoka
	e-Rad number	70569863
	Affiliation	Clinical Research Support Office, National Cancer Center Hospital
	Title	Medical officer

16.14.6. Coordination management practitioner

Organizations in charge of coordination and management		JCOG Operations Office
Coordinating and managing practitioners	Name	Junko Eba
	e-Rad number	80754085
	Affiliation	Clinical Research Support Office, National Cancer Center Hospital
	Title	Medical officer

16.14.7. Personnel who oversees the study other than Principal Investigator and site investigators

<table>
<tr><td rowspan="5">Personnel who oversees the study other than Principal Investigator and site investigators</td><td>Name</td><td colspan="2">Not applicable</td></tr>
<tr><td>e-Rad number</td><td colspan="2"></td></tr>
<tr><td>Affiliation</td><td colspan="2"></td></tr>
<tr><td>Title</td><td colspan="2"></td></tr>
<tr><td>Relevance of Secondary Sponsor</td><td>□ Applicable</td><td>□ Not applicable</td></tr>
<tr><td></td><td></td><td colspan="2"></td></tr>
</table>

16.14.8. Study group personnel

JCOG Data Center
- Statistics Section: Gakuto OGAWA
- Data Management Section: Kyoko HASEGAWA

JCOG Operations Office
- Science Section: Tomoko KATAOKA/Tadayoshi HASHIMOTO

In addition, JCOG Data Center/Operations Office commissioned some of theire research support activities (such as support for the preparation of study protocols, data management, and site-visit audits) to other corporations. The commissioned duties are supervised by the National Cancer Center through routine work, as well as by receiving work reports from the institution and providing supervisory guidance. The current consignee is as follows

Clinical Oncology Research and Education, a specified non-profit organization
〒104-0061 DJ Ginza Building 7F, 8-18-3, Ginza, Chuo-ku, Tokyo
Official website http://www.core.or.jp/

16.15. Developing a study protocol

Chigusa MORIZANE, National Cancer Center Hospital
Nozomu MACHIDA, Shizuoka Cancer Center
Yoshitaka HONMA/Ken KATO, National Cancer Center Hospital

Support for protocol development

JCOG Data Center
- Statistics Section (in charge of study design): Junki MIZUSAWA
- Data Management Section (CRF preparation): Harumi KABA

JCOG Operations Office
- Protocol development: Hiroshi KATAYAMA/Kozo KATAOKA/Aya MIURA
- Person in charge of IC documents: Aya KIMURA/Noriko TSUJI

17. Publication of the study results and completion of the study

17.1. Paper and conference presentations

Primary publication will be published in English journals.

Paper publication including review article and conference presentation of Introduction of the study, by Principal Investigator or Study Coordinator, which does not include the analytical results of the endpoint of the study, are allowed when Group Chair and JCOG Data Center Director agree to them. Publication of the distribution of baseline factors or the safety data after the end of accrual are also allowed. No publication other than primary analysis and final analysis will be performed unless previously approved by Data and Safety Monitoring Committee.

In principle, the authors of the main published papers on the results of the study (the first publication of the results of primary endpoint) shall be the first Study Coordinator (HBPOG), followed by Study Coordinator (JEOG or SCSG), Study Coordinator (JEOG or SCSG), Principal Investigator (HBPOG), Principal Investigator (JEOG or SCSG), Principal Investigator (JEOG or SCSG), the statistical staff of Data Center (one statistician in charge at the time of the analysis for publication), Centralized Pathological diagnosis Coordinator (HBP), Centralized Pathological diagnosis Coordinator (GI). In accordance with the limitations imposed by the article's posting provisions, researchers who contributed in descending order of their number of patients registrations were selected for each institution as co-authors, and the last author was Group Chair (or Study Chair). The inclusion of staffs of JCOG Operations Office as co-authors will be determined by Group Chair depending on their contributions. Authors of articles other than the primary publication (e.g., Secondary endpoints articles, secondary analysis articles) will be determined by Study Chair with Group Chair approval.

All co-authors will review the article contents prior to submission for publication and only those who agree to the article contents. If there is no consensus on the contents, principle investigator may not include the investigator in the co-authorship with the approval of Group Chair. If there is no consensus between groups and JCOG Data Center/Operations Office, ultimately follow JCOG Chair instructions.

17.2. Primary Endpoint Report and Clinical Summary Report

The procedures are specified from the preparation of the primary endpoint report and clinical summary report. If primary analysis is the final analysis, the primary endpoint report will not be prepared and the clinical summary report will be prepared.

17.2.1. Clinical Summary Report

Based on the final analysis report, Principal Investigator will prepare a "Abstract of the Clinical Summary Report" containing subjects background information (age, sex, etc.), study design and study progress, results of analyses for each endpoint, conclusions of the entire study, interpretations and discussion of the results, etc. within 6 months from the issue date of the final analysis report, submit it to the Data Center, and undergo review. In addition, the issue date of the final analysis report shall be "the date when the period for collecting data on all endpoints is completed" as specified in the Enforcement Regulations.

After obtaining approval from Group Chair and the Director of the Data Center, abstract of the clinical summary report will be submitted to Certified Review Board review as a "clinical summary report" with the final analysis report within 1 year of the issue date of the final analysis report.

Principal Investigator/Study Coordinator will disclose abstract of the clinical summary report (Notification of Completion of Form 1 of Article 24 of Regulation) to jRCT within 1 month after obtaining Certified Review Board approval (if the report is unpublished, abstract will not be released in jRCT and will be published immediately after publication).

The approved abstract of the clinical summary report will be submitted to the administrators of each participating institution through the site investigators at each institution and to JCOG Chair. Abstract of the clinical summary report is available on JCOG website (http://www.JCOG.jp/).

The timing of distribution of the final analysis report to participating institutions will be determined by Principal Investigator/Study Coordinator considering the timing of publication of the final analysis results, and the final analysis report will be distributed to the researchers at participating institutions by themselves or through the Data Center.

17.3. Completion of the study

On the date that abstract of the clinical summary report was released to iRCT, the study is completed.

In institutions where patients were not enrolled, the institution may be withdrawn from the institution list by submitting a request for change (Unified Form 3 for Clinical Research) and a Notification of Changes in Protocol (Form 2) stating that Principal Investigator withdraws the institution from the institution list in question to Certified Review Board, and after obtaining approval, notifying the MHLW (Local Health Bureau responsible for the location of Certified Review Board). When a protocol change notification is received by the Local Health Service, the investigator at the institution shall report the withdrawal to the Administrator of the institution.

18. References

omit

19. Appendix

- Informed consent form
- Body surface area table
- Toxicity Criteria (CTCAE v5.0-JCOG)
- CRF samples* (CRF draft attached for the first review submission)
- JCOG-Biobank Japan Biorepository Protocol
- JCOG-Biobank Japan Biorepository Informed Consent Form

Japan Clinical Oncology Group
Hepatobiliary and Pancreatic Oncology Group/Stomach Cancer Study Group/Japan Esophageal Oncology Group

Practical Research for Innovative Cancer Control from Japan Agency for Medical Research and Development
"Establishment of standard treatments for neuroendocrine carcinoma of the digestive system"
National Cancer Center Research and Development Fund 29-A-3

JCOG1213

Randomized phase III study of etoposide plus cisplatin combination therapy versus irinotecan plus cisplatin combination therapy in advanced neuroendocrine carcinoma of the digestive system. ver.1.3.0

TOPIC-NEC

HBPOG
Group Chair: Junji FURUSE
Department of Medical Oncology, Kyorin University Faculty of Medicine

Study Chair: Takuji OKUSAKA
Department of Hepatobiliary and Pancreatic Oncology, National Cancer Center Hospital 5-1-1 Tsukiji, Chuo-ku, Tokyo 104-0045 Japan
Tel: +81-3-3542-2511
E -mail：tokusaka@ncc.go.jp

Study Coordinator: Chigusa MORIZANE
Department of Hepatobiliary and Pancreatic Oncology, National Cancer Center Hospital 5-1-1 Tsukiji, Chuo-ku, Tokyo 104-0045 Japan
Tel: +81-3-3542-2511
E -mail：cmorizan@ncc.go.jp

SCSG
Group Chair: Masanori TERASHIMA
Division of Gastric Surgery, Shizuoka Cancer Center Hospital

Study Chair: Narikazu BOKU
Department of Gastrointestinal Oncology, National Cancer Center Hospital
5-1-1 Tsukiji, Chuo-ku, Tokyo 104-0045 Japan
Tel: +81-3-3542-2511
E -mail： nboku@ncc.go.jp

Study Coordinator: Nozomu MACHIDA
Department of Hepatobiliary and Pancreatic Oncology, Shizuoka Cancer Center
1007 Shimonagakubo, Nagaizumi-cho, Sunto-gun, Shizuoka Prefecture 411-8777 Japan
Tel: +81-55-989-5222
E -mail：no.machida@scchr.jp

JEOG
Group Chair: Yuko KITAGAWA
General and Gastroenterological Surgery, Keio University Hospital

Study Chair: Ken KATO
Department of Gastrointestinal Oncology, National Cancer Center Hospital 5-1-1 Tsukiji, Chuo-ku, Tokyo 104-0045 Japan
Tel: +81-3-3542-2511
E -mail：kenkato@ncc.go.jp

Study Coordinator: Yoshitaka HONMA/Ken KATO
Department of Gastrointestinal Oncology, National Cancer Center Hospital 5-1-1 Tsukiji, Chuo-ku, Tokyo 104-0045 Japan
Tel: +81-3-3542-2511
E-mail:yohonma@ncc.go.jp
/kenkato@ncc.go.jp

2013/3/16	Protocol concept approved by JCOG Executive Committee (PC1213/1214/1215)
2014/6/27	Protocol approved byJCOG Protocol Review Committee
2017/5/23	Revision ver. 1.1 approved by JCOG Data and Safety Monitring Committee
2018/8/16	Revision ver. 1.2 approved by JCOG Data and Safety Monitring Committee
2018/10/11	Approved by Certified Review Board of National Cancer Center Hospital
2019/5/27	Revision ver. 1.3.0. approved by JCOG Data and Safety Monitring Committee
2019/6/27	Approved by Certified Review Board of National Cancer Center Hospital

0. Summary

This study is conducted as a "specified clinical trial" based on the Clinical Trials Act (Act No. 16 of April 14, 2017). In this protocol, the Principal Investigator is the Study Chair in the Hepatobiliary and Pancreatic Oncology Group of JCOG.

Name of study: "Randomized phase III study of etoposide plus cisplatin combination therapy versus irinotecan plus cisplatin combination therapy in advanced neuroendocrine carcinoma of the digestive system. (TOPIC-NEC) ".

Public study title: "Randomized phase III study of etoposide plus cisplatin combination therapy versus irinotecan plus cisplatin combination therapy in advanced neuroendocrine carcinoma of the digestive system. (TOPIC-NEC) "

0.1. Schema

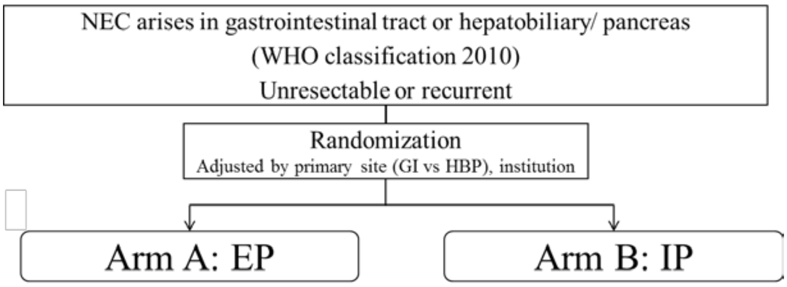

0.2. Objectives

A randomized phase III study was conducted to determine the better treatment option between etoposide/cisplatin combined therapy (EP therapy) or irinotecan/cisplatin combined therapy (IP therapy), both of which are standard treatments for non-resectable/recurrent neuroendocrine carcinoma (NEC as classified by WHO in 2010) with primary lesions in the gastrointestinal tract/hepatobiliary pancreatic organs.

Primary endpoint: Overall survival

Secondary endpoints: Response rate in case of measurable lesions, Progression-free survival (PFS), incidence rate of adverse events, dose intensity of Cisplatin, and incidence rate of serious adverse events.

0.3. Subjects

1) Any of the following is applicable based on pathological diagnosis taking findings of immunohistochemistry into consideration (see 3.1. to 3.3.).

[1] Pathologically diagnosed as neuroendocrine carcinoma (NEC*1) in the resected sample.

[2] Containing pathologically confirmed component of neuroendocrine carcinoma (NEC*1) in the biopsy sample.

1: Based on WHO 2010 classification

2) Any of the following is applicable

[1] NEC arise in esophagus, stomach, duodenum, intestine, appendix, colon, rectum, gallbladder, intrahepatic bile duct, extrahepatic bile duct, ampulla of Vater, pancreas,

[2] Liver NEC (primary liver or liver metastasis of unknown primary) *2.

*2: The tumor is only in one of the following sites after a thorough examination of the primary site by contrast CT (from the neck to pelvic) and upper/lower gastrointestinal endoscopy, FDG-PET scan, otolaryngology (head and neck) examination, urology examination (male patients only), and gynecology examination (female patients only).

a. Liver only

b. Bone, lymph nodes below the diaphragm, peritoneum, subcutaneous, muscle, spleen, and liver

3) Unresectable (see 3.6) or recurrent cancer (see 3.7). It is not essential for a pathological confirmation of the metastatic lesion or recurrent site. Cases of esophageal NEC is ineligible if corresponding to any of the following.

[1] cT4.

[2] No distant metastasis rather than supraclavicular lymph node

[3] Stenosis indicated for palliative radiotherapy

4) No previous chemotherapy or radiotherapy for NEC. Pre- or post-operative chemotherapy except irinotecan or

etoposide for NEC is allowed as long as it was completed at least 8 weeks prior to registration.

5) No previous chemotherapy using platinum agents for any malignancies.

6) No serious tumor-related complications.

Serious tumor-related complications include superior vena cava syndrome, inferior vena cava syndrome, pleural, ascites, or pericardial effusions that are large or uncontrollable (e.g., effusions that accumulate quickly after drainage or even after adhesive surgery), and brain metastases with neurological symptoms.

7) Aged 20 to 75 years old.

8) ECOG performance status of 0 or 1.

9) Sufficient oral intake

10) Measurable region is not required.

11) Adequate organ functions.

[1] WBC ≥3,000/ mm^3

[2] Neutrophils ≥ 1,500/ mm^3

[3] Hemoglobin ≥ 9.0 g/dL

[4] Platelets ≥ 10×10^4/ mm^3

[5] Total bilirubin≤ 1.5 mg/dL[※4]

[6] AST(sGOT)≦100 IU/L[※4]（for hepatic NEC and liver metastasis, ≦150 IU/L）

[7] ALT(sGPT)≦100 IU/L[※4]（for hepatic NEC and liver metastasis, ≦150 IU/L）

※4：Presence or absence of biliary drainage is not relevant

[8] Serum creatinine≦ 1.3 mg/dL

[9] Creatinine clearance[※5]≥ 60 mL/min

※5： Creatinine clearance must have been estimated using the Cockcroft-Gault formula, and must be 60 mL/min or more. If the estimation is less than 60 mL/min, but the actual measurement is 60 mL/min or more, the patient can be deemed eligible.

Cockcroft-Gault formula

Male：Ccr ={(140－age) × body weight (kg)}/{72×serum creatinine (mg/dL)}

Female：Ccr = 0.85×{(140－age) × body weight (kg)}/{72×serum creatinine(mg/dL)}

12) Written informed consent.

0.4. Treatments

Arm A: etoposide plus cisplatin (EP) arm

The following chemotherapy is continued until the patient meets discontinuation criteria, with 3-weeks of treatments counting as one cycle.

Drug	Dosage (mg/m^2)	Dosing regimen/Dosing time	Dose day
Etoposide	100	IV/60-120 min	Day 1, 2, 3
Cisplatin	80	IV/60-120 min	Day 1

Arm B: Irinotecan plus Cisplatin (IP) arm

The following chemotherapy would be continued until the patient meets discontinuation criteria, with 4-weeks of treatments counting as one cycle.

Drug	Dose (mg/m^2)	Dosing regimen/Dosing time	Dose day
Irinotecan	60	IV/90 min	Day 1, 8, 15
Cisplatin	60	IV/60-120 min	Day 1

0.5. Planned sample size and study period

The planned sample size is 170.

Accrual period: 6 years.

Follow-up period: 1 years after accrual completion.

Analysis period: 1 year.

Total study duration 8 years.

Scheduled start date of the study Aug 8, 2014

Expected completion date of the study Aug 8, 2028

0.6. Contact information

Eligibility criteria, treatment modification, and other issues requiring clinical decisions: Study Coordinator (front cover and 16.6.)

Enrollment procedure protocol, case report form (CRF) entries, etc.: JCOG Data Center, (16.14.)

Adverse event reporting: JCOG Data and Safety Monitoring Committee (16.11.),

Contents

0. SUMMARY 2

0.1. SCHEMA 2
0.2. OBJECTIVES 2
0.3. SUBJECTS 2
0.4. TREATMENTS 3
0.5. PLANNED SAMPLE SIZE AND STUDY PERIOD 3
0.6. CONTACT INFORMATION 4

1. OBJECTIVES 8

2. BACKGROUND 8

2.1. TARGET 8
2.2. STANDARD TREATMENT FOR TARGET DISEASE 16
2.3. RATIONALE FOR ESTABLISHMENT OF TREATMENT PLAN 18
2.4. STUDY DESIGN 21
2.5. SUMMARY OF EXPECTED ADVANTAGES AND DISADVANTAGES ASSOCIATED WITH STUDY PARTICIPATION 22
2.6. SIGNIFICANCE OF THIS STUDY 23
2.7. ASSOCIATED RESEARCH (INCLUDING SAMPLE ANALYSIS RESEARCH) 23
2.8. JCOG-BIOBANK JAPAN (BBJ) COLLABORATING BIOBANK 23

3. CRITERIA/DEFINITIONS USED IN THIS STUDY 25

3.1. TISSUE CLASSIFICATION (WHO 2010 CLASSIFICATION) 25
3.2. GRADE CLASSIFICATION （ENETS [EUROPEAN NEUROENDOCRINE TUMOR SOCIETY] / WHO2010 CLASSIFICATION） 25
3.3. HISTOPATHOLOGICAL DIAGNOSIS 25
3.4. DISEASE STAGE CLASSIFICATION CRITERIA (UICC-TNM 7TH EDITION) 25
3.5. RESIDUAL TUMOR (R) CLASSIFICATION (UICC-TNM 7TH EDITION) 33
3.6. DEFINITION OF NON-RESECTABLE NEC 33
3.7. DEFINITION OF RECURRENT NEC 34
3.8. DEFINITION OF HEPATIC NEC (HEPATIC PRIMARY LESION OR LIVER METASTASIS FROM AN UNKNOWN PRIMARY LESION) 34

4. PATIENT SELECTION CRITERIA 34

4.1. INCLUSION CRITERIA (FOR ENROLLMENT) 34
4.2. EXCLUSION CRITERIA 35

5. REGISTRATION AND RANDOMIZATION 36

5.1. PROCEDURE OF REGISTRATION 36
5.2. RANDOMIZATION AND ALLOCATION ADJUSTMENT FACTOR 37

6. TREATMENT PLAN AND TREATMENT MODIFICATION CRITERIA 38

6.1. PROTOCOL TREATMENT 38
6.2. PROTOCOL TREATMENT TERMINATION/COMPLETION CRITERIA 41
6.3. TREATMENT MODIFICATION CRITERIA 42
6.4. CONCOMITANT TREATMENT AND SUPPORTIVE CARE 48
6.5. POST-STUDY TREATMENT 55

7. ANTICIPATED ADVERSE EVENTS 56

7.1. ANTICIPATED ADVERSE REACTIONS 56

7.2. ANTICIPATED ADVERSE EVENTS DUE TO PATHOGENESIS 59
7.3. EVALUATION OF ADVERSE EVENTS/REACTIONS 60

8. EXAMINATION AND EVALUATION 62

8.1. BASELINE EXAMINATION AND EVALUATION BEFORE REGISTRATION 62
8.2. EXAMINATION AND EVALUATION DURING TREATMENT 63
8.3. EXAMINATION AND EVALUATION AFTER COMPLETION OF TREATMENT 64
8.4. INFORMATION ON POST- STUDY TREATMENT 64
8.5. STUDY CALENDAR 65

9. DATA COLLECTION 69

9.1. CASE REPORT FORM (CRF) 69

10. REPORTING OF "DISEASE OR THE LIKE"(ADVERSE EVENTS) 70

10.1. SERIOUS ADVERSE EVENTS AND SUBJECTS OF EXPEDITED REPORTING. 70
10.2. INVESTIGATOR'S REPORTING REQUIREMENTS AND PROCEDURES 71
10.3. RESPONSIBILITIES OF PRINCIPAL INVESTIGATOR/STUDY COORDINATOR 73
10.4. RESPONSIBILITIES OF THE SITE INVESTIGATORS AT THE PARTICIPATING INSTITUTIONS (INCLUDING THE RELEVANT INSTITUTION) 75
10.5. RESPONSIBILITIES OF THE DATA AND SAFETY MONITORING COMMITTEE 75

11. RESPONSE EVALUATION AND ENDPOINT DEFINITION 76

11.1. RESPONSE ASSESSMENT (ONLY FOR PATIENTS WITH MEASURABLE DISEASE) 76
11.2. DEFINITIONS OF ANALYSES SET 82
11.3. DEFINITION OF ENDPOINTS 82

12. STATISTICAL CONSIDERATION 85

12.1. PRINCIPAL ANALYSIS AND DECISION CRITERIA 85
12.2. PLANNED ACCRUAL, ACCRUAL PERIOD, AND FOLLOW-UP PERIODS 86
12.3. INTERIM ANALYSIS AND EARLY TERMINATION OF THE STUDY 86
12.4. ANALYSIS OF SECONDARY ENDPOINTS 88
12.5. FINAL ANALYSIS 88
12.6. EXPLORATORY ANALYSIS 89
12.7. PREMATURE WITHDRAWAL FROM THE TRIAL 89
12.8. PROCEDURES AFTER EARLY TERMINATION OF THE STUDY 90

13. ETHICAL CONSIDERATIONS 92

13.1. PROTECTION OF HUMAN SUBJECTS 92
13.2. INFORMED CONSENT 92
13.3. PROTECTION OF PERSONAL INFORMATION AND PATIENT IDENTIFICATION 94
13.4. ADHERENCE TO THE PROTOCOL 96
13.5. APPLICATION TO CERTIFIED REVIEW BOARD AND NOTIFICATION OF IMPLEMENTATION PLANS 96
13.6. PROTOCOL REVISION/AMENDMENT 100
13.7. CONFLICTS OF INTEREST (COIS) INVOLVED IN THIS STUDY 102
13.8. COMPENSATION 104
13.9. INTELLECTUAL PROPERTY 105
13.10. DISCLOSURE OF INFORMATION ON THIS STUDY 105

14. MONITORING AND AUDIT 106

14.1. PERIODIC MONITORING 106
14.2. SITE VISIT AUDITS 108

14.3. MANAGEMENT OF NON-COMPLIANCE 109

15. SPECIAL INSTRUCTIONS 110

15.2. JCOG BIOBANK JAPAN (BBJ) BIOREPOSITORY 111

16. ORGANIZATION 111

16.1. MAIN STUDY FUND (FUNDING SOURCE) OF THIS STUDY. 111
16.2. JAPAN CLINICAL ONCOLOGY GROUP (JCOG) 111
16.3. JCOG CHAIR 112
16.4. STUDY GROUP AND GROUP CHAIR 112
16.5. STUDY CHAIR (PRINCIPAL INVESTIGATOR) 113
16.6. STUDY COORDINATOR 113
16.7. CENTRAL PATHOLOGICAL REVIEW COORDINATOR 114
16.8. CENTRAL PATHOLOGICAL REVIEW COORDINATOR 114
16.9. PARTICIPATING SITES (PARTICIPATING INSTITUTIONS) 115
16.10. JCOG PROTOCOL REVIEW COMMITTEE 116
16.11. JCOG DATA AND SAFETY MONITORING COMMITTEE 116
16.12. JCOG AUDIT COMMITTEE 116
16.13. JCOG CONFLICT OF INTEREST COMMITTEE 116
16.14. DATA CENTER/OPERATIONS OFFICE 116
16.15. DEVELOPING A STUDY PROTOCOL 118

17. PUBLICATION OF THE STUDY RESULTS AND COMPLETION OF THE STUDY 119

17.1. PAPER AND CONFERENCE PRESENTATIONS 119
17.2. PRIMARY ENDPOINT REPORT AND CLINICAL SUMMARY REPORT 119
17.3. COMPLETION OF THE STUDY 120

18. REFERENCES 121

19. APPENDIX 121

1. Objectives

A randomized phase III study was conducted to determine the better treatment option between etoposide/cisplatin combined therapy (EP therapy) or irinotecan/cisplatin combined therapy (IP therapy), both of which are standard treatments for non-resectable/recurrent neuroendocrine carcinoma (NEC as classified by WHO in 2010) with primary lesions in the gastrointestinal tract/hepatobiliary pancreatic organs.

Primary endpoint: Overall survival

Secondary endpoints: Response rate in case of measurable lesions
Progression-free survival (PFS), incidence rate of adverse events, dose intensity of Cisplatin, and incidence rate of serious adverse events

2. Background

2.1. Target

2.1.1. Epidemiology

Neuroendocrine tumors (NETs) and neuroendocrine carcinomas (NECs) can arise in various organs in the body. Of these, NECs with primary lesions occurring in the lungs, namely small cell lung cancer and large cell lung cancer, have been collectively referred to as high-grade NECs (HGNECs). However, terms such as extrapulmonary small cell carcinoma and extrapulmonary NEC have been used to refer collectively to NECs, with primary lesions outside the lungs.

The number of newly reported cases of neuroendocrine neoplasms (NENs) annually, estimated based on the cases registered in the U.S. SEER (Surveillance, Epidemiology, and End Results) database between 1973 to 2004 is 5 in 100,000 people[1]. In Japan, the Neuroendocrine Tumor Workshop Japan (NET Work Japan) carried out a nationwide survey of pancreatic and gastrointestinal NENs, and estimated the number of new cases in 2005 at approximately 1.01 people with pancreatic primary lesion per 100,000 people, and 2.10 people with gastrointestinal primary lesion per 100,000 people[2, 3].

The report by the PRONET Study Group in France concerning their prospective observational study is a good reference for the proportion of NENs that can be classified as NECs. Out of the 778 patients diagnosed with gastrointestinal primary lesion NENs in 80 facilities from August 2010 to July 2011, 104 patients (13.4%) had NEC[4].

The frequency of incidence of NECs according to the primary lesion organ has been shown in the tables below (Table 2.1.1a and Table 2.1.1b). Despite variations between reports, gastrointestinal primary lesion NECs account for approximately 20–68% of all extrapulmonary NEC cases. The breakdown of gastrointestinal primary lesion NEC cases with primary lesion in the gastrointestinal tract (esophagus, duodenum, small and large intestines) indicated that these cases accounted for 56–84% of the total cases, while hepatobiliary pancreatic cancer cases accounted for 15–35% of all cases.

Table 2.1.1a. Frequency of extrapulmonary NECs according to the organ of primary lesion

Reported year/Reporter	N	Gastrointestinal	Gynecologal organs	Urology	Head and Neck	Adrenal	Primary unknown	Other
2012/Terashima [5]	136	68%	12%	6%	NA	NA	8%	7%
2010/Brennan [6]	74	20%	35%	15%	19%	NA	9%	1%
2009/Wong [7]	1618	33%	NA	20%	11%	10%	4%	22%
2006/Haider [8]	101	20%	11%	18%	12%	9%	31%	NA
2007/Lee [9]	61	56%	20%	10%	8%	NA	6%	NA

Table 2.1.1b. Percentage breakdown of NECs as per the primary lesion organ of gastrointestinal NEC

Reported year/Reporter	N	Esophageal	Gastric	Duodenal/small intestine	Colorectal	Hepatobiliary	Pancreas	Other
2012/Machida [10]	258	33%	27%	2%	12%	12%	14%	NA
2012/Sorbye [11)]	205	6%	10%	NA	40%	NA	35%	10%
2004/Brenner [12]	544	53%	11%	0.2%	20%	11%	4%	NA

2.1.2. Clinical pathology

1）About the term of target disease

The target of this study has a complex disease concept, and since the method of classification has changed with time, various names have been assigned that could cause confusion. In this study, we use classification and nomenclature defined according to the WHO 2010 classification （WHO Classification of Tumors of the Digestive System 2010）[13].

All the tumors originating from neuroendocrine cells or differentiation into endocrine cells are called neuroendocrine neoplasms (NENs). Depending on the malignancy, NENs were classified as Grade 1 NETs (NET G1), Grade 2 NETs (NET G2), or NEC.

Furthermore, prior to the WHO 2010 classification, all disease concepts equivalent to NENs were called NETs, but in this study, we would have referred to all descriptions of the disease concept as NENs. Furthermore, according to the 2000 and 2004 WHO classifications, the term well-differentiated NEC (WDNEC) has been used for disease units equivalent to NET G2, but in this study NEC does not mean the same.

2）Classification and clinical pathology

NEN is a tumor that develops from neuroendocrine cells present in various tissues or shows a tendency of differentiation into neuroendocrine cells. Histologically these tumors express neuroendocrine markers, such as Chromogranin A, NSE (neuron-specific enolase), and synaptophysin. The WHO classifications in 2000 and 2004 combined the presence or absence of metastasis/local infiltration and cellular proliferative capacity (evaluated based on Ki-67 expression intensity and mitotic presentation), and thereby classified these tumors into well-differentiated (neuro)endocrine tumors without metastasis/local infiltration, well-differentiated (neuro)endocrine tumors with metastasis/local infiltration, and poorly differentiated (neuro)endocrine tumors with even greater cell proliferative capacity. On the other hand, European Neuroendocrine Tumor Society (ENETS) has proposed a method of classification that evaluates the malignancy as Grade 1, 2, or 3 (G1, G2, or G3), according to the cell proliferative capacity (Ki-67 index or number of mitotic presentations), which has proven to be most useful for classification of prognosis[14, 15]。

Against this backdrop, the WHO classification that targeted gastrointestinal diseases was published in 2010. The disease as a whole was considered “neuroendocrine neoplasms (NEN)”, and the disease malignancy was largely classified as NET G1 (neuroendocrine tumor Grade 1), NET G2 (Grade 2), NEC, and mixed adenoneuroendocrine carcinoma (MANEC). MANEC refers to the cancers wherein adenocarcinoma components account for more than 30% of the cancer, while those under 30% are classified as NECs. However, even though this method of classification is applicable while evaluating the entire tumor based on resected specimen, it is not possible to evaluate the proportion of each component in the entire tumor during a diagnosis based on biopsy sample.

Each of NET G1, NET G2, and NECs described in the WHO 2010 classification is equivalent to G1, G2, and G3 as per the ENETS classification. NETs are well-differentiated tumors, with relatively low atypicality and malignancy, and the clinical course is slow with a 5-year survival in the range of 62–85%[16, 17]. On the other hand, NECs are poorly differentiated tumors, where tumor cells with poor cellular constituents proliferate diffusely, and are pathologically similar to small cell lung cancers, since these show many mitotic presentations, involve necrotic lesions, and present with neuroendocrinological features during immunohistochemical staining[1]. The clinical presentations are also similar in terms of the rapid proliferation of NECs and relatively high sensitivity to anticancer drugs[18].

Similar to lung cancer, NECs also exist as small cell cancer type or large cell type (LCNEC：large cell NEC）[13]. Future challenges include studies on the frequency and differences in clinical presentation of each disease. Table 2.1.2 shows the WHO and Grade classification.

Table 2.1.2. Changes in WHO classification and grading; Shaded parts are targets of this study

WHO 2000 classification	WHO 2010 classification		ENETS Grade
Well-differentiated endocrine tumor（WDNET）<br>1.1‘Benign’ behavior<br>1.2 Uncertain behavior	Neuroendocrine tumors, NETs<br>• Well-differentiated<br>• Composed of cells similar to normal gastrointestinal	NET G1<br>（carcinoid）	G1<br>Same as 2010 WHO classification<br>Number of mitotic presentations < 2 per 10

	endocrine cells<br>• Expression of neuroendocrine markers		high power fields（HPF） and/or Ki-67 index ≤2%
Well-differentiated endocrine carcinoma（WDEC）	• Hormone production<br>• Mild to moderate nuclear atypia, low proliferative capacity（Grade：G1,G2）	NET G2	G2<br>Number of mitotic presentations 2–20 per 10 HPF and/or Ki-67 index 3-20%
Poorly differentiated endocrine carcinoma/small cell carcinoma（PDEC）	Neuroendocrine carcinoma：NEC（large cell or small cell type）：<br>• Poorly differentiated, highly malignant<br>• Include small cell to large cell type carcinoma<br>• Sometimes present tissue structure similar to NET<br>• Expression of neuroendocrine markers<br>• Significant nuclear atypia, multifocal necrosis, and high proliferative capacity (Grade: G3)		G3<br>Number of mitotic presentations >20 per 10 HPF and/or Ki-67 index > 20%
Mixed exocrine-endocrine carcinoma（MEEC）	Mixed adenoendocrine carcinoma（MANEC）		
Tumor-like lesions（TLL）	Hyperplastic and preneoplastic lesions		

2.1.3. Staging

Disease staging for NENs is represented by the TNM classification proposed by ENETS (hereafter, ENETS TNM[14, 15], AJCC 7th Edition TNM classification and UICC 7th Edition TNM classification published in 2009. The AJCC 7th Edition and UICC 7th Edition TNM classifications are unified, and in this study we would have used the UICC 7th Edition nomenclature).

In the UICC 7th edition, there are independent TNM classifications of NET G1 and NET G2 with primary lesions in the stomach, small intestine, appendix, and colon, but with respect to NEC, it describes "classification according to main tissue type in each organ of primary lesion (squamous cell carcinoma for esophagus, and adenocarcinoma in all other organs of primary lesion)." While there are TNM classifications for hepatocellular carcinoma and intrahepatic cholangiocarcinoma in the liver, there is no description for the classification of the tissue type for NECs with primary lesion in the liver. In this study, however, we would have used the TNM classification of intrahepatic cholangiocarcinoma, which shows similar clinical presentations. Details of each TNM classification would have been mentioned in “3.2 staging criteria”.

2.1.4. Standard treatment according to disease staging and outline of prognosis

1) Standard treatment for resectable cases

Surgical resection is performed for resectable cases. The significance of postoperative adjuvant chemotherapy is not clear because a verification study has not yet been performed. Although the NCCN Guidelines[19] describe postoperative adjuvant therapy as the chemotherapy regimen for small cell lung cancers that is typically applied to advanced cases of NEC, it has relatively high toxicity and patient burden (see Table 2.2.2, 2.3.2); hence we cannot claim that it is widely used in routine practice. For NECs with gastrointestinal primary lesion with mixed adenocarcinoma, a chemotherapy regimen is suggested typically after surgery for adenocarcinoma; however, in practice, there is no consensus on postoperative adjuvant chemotherapy.

2) Standard treatment for locally advanced cases

While the NCCN Guidelines propose chemoradiotherapy for small cell lung cancer for locally advanced cases, the ENETS Guidelines provide no clear indication[20, 21] for the same. As there is no sufficient information related to radiation dose, efficacy, and safety for NECs with a multitude of primary lesion organs (scope of irradiation), radiation therapy is not widely used for pathologies other than NEC with esophageal primary lesion that is prone to stenosis, and treatment is often provided for distant metastasis.

For locally advanced cases of NEC with esophageal primary lesion, chemoradiotherapy is actively performed. Furthermore, supraclavicular lymph node metastasis in thoracic esophageal tumor is classified as Stage IV, and chemoradiotherapy is applied if metastatic lymph nodes are included in the field of irradiation.

3) Standard treatment for distal metastasis cases

Systemic chemotherapy is indicated for distal metastasis and recurrent cases regardless of the organ, and a regimen is chosen for the small cell lung cancer. With respect to NECs with esophageal primary lesion, even if there is organ metastasis, palliative chemoradiotherapy is prioritized for patients incapable of oral intake of drugs due to esophageal constriction.

The efficacy of everolimus and sunitinib has been demonstrated for NETs with pancreatic primary lesions. Furthermore, Octreotide and Lantreotide effectively inhibit proliferation of NETs that originate in the midgut and pancreatic/gastrointestinal NETs, respectively. However, these results are from clinical trials conducted in patient populations corresponding to NETs. Since the clinical presentations of NETs and NECs differ significantly, these treatments cannot be indicated for NECs during routine practice.

4) Indication of resection for distal metastasis

There is no consensus on the resection criteria specific to the NECs. Since the progression of NETs is slow, resection is actively considered even where there is distal metastasis to the liver, but as NEC progresses fast, surgical resection cannot be indicated for cases with distal metastasis[19, 21].

5) Prognosis

In a preceding study, Yamaguchi and Machida carried out a multicenter joint observational study of poorly

differentiated NEC (WHO 2010 NEC, including patients with clinical diagnosis of poor differentiation) in a total of 23 facilities, including facilities participating in JCOG Hepatobiliary and Pancreatic Oncology Group, former Gastroenterology Group, and observer facilities.

In the multicenter joint study by Yamaguchi and Machida, 89% of cases (229/258 patients) receiving systemic chemotherapy had distal metastasis, with a median survival time (MST) of 11.5 months for all patients, 11.2 months for distal metastasis cases, and 15.9 months for locally advanced cases. MST according to organ was 13.4 months for esophageal primary lesion (N = 85), 13.3 months for gastric primary lesion (N = 70), 29.7 months for small intestine/duodenal primary lesion (N = 6), 7.6 months for colonic primary lesion (N = 31), 8.5 months for pancreatic primary lesion (N = 35), and 7.9 months for hepatobiliary primary lesions (N = 31) (Table 2.1.4). While there were 9 cases of five-year survival among all the gastrointestinal cases, there were no five-year survival cases amongst those with primary lesions in hepatobiliary and pancreatic organs[10]. In a multicenter joint observational study for NECs conducted in four Northern European countries (hereafter referred to as NORDIC NEC Study), the MST for NECs with gastrointestinal primary lesion (albeit including ~30% of NECs with primary lesion sites unknown) was 11 months[11]. According to the U.S. SEER database, the MST of NEC was 10 months[3]. Based on the above information, despite differences in the primary lesion organ, the prognosis of the target population of this study is thought to have an overall MST of 10–11 months.

Table 2.1.4. Treatment results according to primary lesion organ for non-resectable/recurrent NECs that underwent systemic chemotherapy

	Esophageal	Gastric	Duodenal/small intestine	Colonic	**Gastrointestinal overall**	Pancreatic	Hepatobiliary	**Hepatobiliary and pancreatic organs overall**
N	85	70	6	31	**192**	35	31	**66**
Response rate（%）	58%	43%	50%	29%	**47%**	17%	16%	**17%**
Median PFS（month）	5.8	4.9	7.3	3.7	**5.1**	3.2	4.1	**3.7**
MST（month）	13.4	13.3	29.7	7.6	**13**	7.9	8.5	**7.9**

2.1.5. Tumor-related complications

We, here, report the complications that require attention during patient management, according to the organ of primary lesion. It is a rare disease and frequencies remain unknown.

1）Esophageal primary

Tumor bleeding/pain/esophageal stenosis/fistula formation due to primary lesion in the esophagus; hoarseness/difficulty swallowing/aspiration pneumonia caused by recurrent laryngeal nerve palsy due to lesion in lymph node metastasis; jaundice and liver failure associated with liver metastasis; respiratory failure and hemoptysis associated with lung metastasis, airway constriction, and pain due to lymph node metastasis; hypercalcemia, tracheal stenosis, tracheal obstruction, and suture failure due to disease progression.

2）Gastric primary

Chronic bleeding from primary lesion, accompanied by anemia, stomach pain, nausea, vomiting, gastric perforation, bloating, suture failure, fistula formation, cardiac/pyloric stenosis, ascites retention due to peritoneal metastasis, ileus, hydronephrosis due to ureteral stenosis, obstructive jaundice/liver failure due to bile duct stenosis, and obstructive jaundice/liver failure due to hepatic portal lymph node metastasis.

3）Small intestine/colonic primary

Tumor hemorrhage, ileus, fistula formation in the small intestine/bladder/vagina, intestinal obstruction, intestinal stenosis, intestinal perforation, and pelvic infection

4）Hepatobiliary and pancreatic primary

Cancer pain, rupture, obstructive jaundice associated with the growth of primary tumor, weight loss, fever, pancreatitis, cholangitis, liver abscess, cholecystitis, biliary hemorrhage, duodenal hemorrhage, duodenal stenosis, anastomotic ulcer/stenosis/leakage, symptoms associated with stenosis/obstruction of portal vein (liver

dysfunction/liver failure, esophageal varices and their rupture, gastritis, ascites retention, and hepatic encephalopathy/coma associated with portal hypertension).

5) Paraneoplastic syndrome

In addition to NECs, there are reports of paraneoplastic syndrome that is often associated with small cell lung carcinoma[22-24]. We report examples of paraneoplastic syndrome seen with small cell lung carcinoma below.

Hyponatremia due to inappropriate sodium secretion, psychological symptoms (changes in personality) due to ectopic ACTH syndrome, hypertension, hypokalemia, high blood sugar, Lambert-Eaton myasthenic syndrome, paraneoplastic cerebellar degeneration associated with autoantibody production (ataxia in the limbs, dysarthria, and nystagmus), paraneoplastic encephalomyelitis/sensory neuron disease (dementia, cranial nerve symptoms, dizziness, ataxia, autonomic imbalance, transverse paralysis, and sensory disorders).

Apart from these, there are thromboembolic events, aspiration (due to gastrointestinal stenosis), anemia, tumor pain, acute renal failure, myositis, and pulmonary fibrosis.

6) Complications due to metastatic lesions

Liver failure, pain, hepatobiliary infection, hemorrhage, bile duct stenosis, bile duct obstruction, respiratory failure, tracheal hemorrhage, respiratory tract infection, atelectasis, airway constriction, ascites, bloating, ileus, ureteral stenosis, ureteral obstruction, urinary retention, urinary tract infection, hiccups, pleural effusion, pleural hemorrhage, chest pain, pathological fracture, pain, hypercalcemia, meningeal carcinomatosis, ataxia, ischemia cerebrovascular, intracranial hemorrhage, nausea, vomiting, dizziness, consciousness disorder, cognitive disturbance, dysphasia, seizure, spasticity, edema limbs, intestinal obstruction/stenosis/perforation, pancreatitis, disseminated intravascular coagulation, thrombocytopenia, anorectal infection, anal hemorrhage, hoarseness, and superior vena cava syndrome

7) Others

General pain management including narcotic analgesics is performed for cancer pain.

Gastrointestinal stenting and bypass surgery are performed for esophageal stenosis, cardiac/pyloric stenosis, and duodenal stenosis.

Percutaneous transhepatic cholangial drainage (PTCD), percutaneous or endoscopic stenting, and bile duct jejunostomy is performed for obstructive jaundice. In addition, complications associated with biliary drainage and gastrointestinal stenting are listed below.

- PTCD, including internal and external fistula tube placement:
  Cholangitis, pancreatitis, cholecystitis, liver abscess, sepsis, biliary hemorrhage, PTCD tube obstruction/deviation, peritonitis, and pneumothorax/pleurisy
- Biliary stenting:
  Cholangitis, pancreatitis, cholecystitis, liver abscess, sepsis, biliary hemorrhage, peritonitis, pneumothorax/pleurisy (for percutaneous stenting), duodenal perforation, pneumonia (for endoscopic stenting), stent obstruction/deviation, duodenal ulcer, and duodenal hemorrhage
- Bile duct jejunostomy:
  Cholangitis, pancreatitis, cholecystitis, liver abscess, and sepsis
- Gastrointestinal stenting:
  Hemorrhage, perforation, pain, stent deviation, stent obstruction, foreign-body sensation, intestinal strangulation, ulcer formation, fever, sepsis, infection, diarrhea, constipation, tenesmus or uncontrollable urination/incontinence symptoms (colon), thyroid injury (esophagus), carotid artery injury (esophagus), and mediastinal abscess (esophagus).

2.1.6. Recurrent/progression

NEC is a very rare disease, and most reports of cases of surgery coincide with the case reports. According to the review by Arai et al., out of the 55 patients with NEC with gastric primary lesion who underwent surgical resection, only three patients survived for two years or more[25]. According to the report by Fischer et al., the MST of 13 patients with NEC with pancreatic primary lesion who underwent surgical resection was 11.7 months (seven of the patients showed distal metastasis)[26]. The form of recurrence among surgery cases remains unknown. The multicenter joint

study by Yamaguchi and Machida reported PFS among cases that underwent systemic chemotherapy with respect to the median as 5.8 months for esophageal primary lesions, 4.8 months for gastric primary lesions, 7.3 months for duodenal small intestine primary lesions, 3.7 months for colonic primary lesions, 3.2 months for pancreatic primary lesions, and 4.1 months for hepatic/biliary primary lesions[10]. In the NORDIC NEC study, the median PFS was 3 months for esophageal primary lesions, 5 months for gastric primary lesions, 3 months for colonic primary lesions, 4 months for rectal primary lesions, 5 months for pancreatic primary lesions, and 4 months for cases with unknown primary lesion site [11].

2.1.7. Prognostic/predictive factors

When prognostic factors were examined by multivariate analysis in the multicenter joint study by Yamaguchi and Machida, out of the various factors such as sex, age (younger or older than 60 years), PS (0 or 1 vs. 2 or more), primary lesion organ (gastrointestinal primary lesions vs. hepatobiliary or pancreatic primary lesions), LDH levels (below vs. above the upper limit of facility standard), presence or absence of liver metastasis, presence or absence of history of radical resection and treatment regimen (IP therapy vs. EP therapy); the independent prognostic factors identified were gastrointestinal primary lesion (vs. hepatobiliary or pancreatic primary lesion, hazard ratio (HR): 0.58), and LDH levels being below the upper limit of facility standard for LDH (vs. above the upper limit of facility standard for LDH, HR: 0.65).

Although IP therapy exhibited slightly better impact on overall survival as compared to EP therapy with an HR of 0.8, the *p*-value of 0.389 meant that there was no significant difference between the two therapies[10]. The NORIDIC NEC study reported poor PS, colonic primary lesions, high platelet count, and high LDH levels to be the main factors behind poor prognosis[11]. However, these prognostic/predictive factors were not obtained with a global consensus.

2.1.8. Rationale for selection of the target population

The objective of this study is to develop a primary chemotherapy regimen for non-resectable/recurrent NECs, and out of the non-resectable or postoperatively recurrent NECs with gastrointestinal primary lesions or hepatobiliary or pancreatic primary lesions (shaded parts in Table 2.1.2 of WHO 2010 classification of NEC), we established chemotherapy-naïve patients to be the target population.

In terms of whether or not to consider MANEC a target of this study, a discussion is needed from the viewpoint of standard treatment. MANEC is considered when 30% or more of the carcinoma consists of adenocarcinoma components, and it is treated as either adenocarcinoma or NEC by discretion of the attending physician, with no real consensus on standard treatment. MANEC was however, excluded from this study, as its disease concept is different from NEC in terms of the standard treatment, which has a consensus regarding the treatment by a "regimen according to small cell lung carcinoma". In fact, a questionnaire was provided to the three groups participating in this JCOG study (response obtained from 52 facilities). The results showed that 79% of facilities considered the WHO 2010 classification of NEC as the appropriate tissue type for this study, while only 13% responded that consideration of NEC+MANEC would be appropriate. Therefore, majority of facilities considered "WHO 2010 classification of NEC as the appropriate target of this study".

While diagnosis of NECs is generally carried out using tissue samples and biopsy samples, as mentioned in 2.1.3, there is no consensus methodology to strictly distinguish NEC and MANEC using biopsy samples. As a result of group discussions, a consensus was reached between the three groups that cases presenting with NEC components by biopsy sample-based diagnosis can be enrolled. Although the use of this method would mean that a certain percentage of patients with MANEC, who were not intended to participate in this study, would be enrolled however, currently there are no appropriate means to avoid this. Therefore, we decided to create a consensus for the future based on information obtained from this study.

1）**<u>Reason for targeting gastrointestinal/hepatobiliary or pancreatic primary lesion NEC</u>**

Various guidelines recommend treatment regimens suited for small cell lung carcinoma, regardless of the organ of primary lesion, and is the rationale showing the validity of the treatment development for NEC across many organs. Furthermore, taking into consideration the frequency of the disease, it is unrealistic to develop treatments according to each organ, and in practice the Minnie-Pearl Cancer Research Network Study (mentioned later in 2.2.2), which is a relatively large-scale clinical study of NEC, development of treatment was carried out in a cross-organ

manner[27].

On the other hand, if we take hypothesize that we do not sufficiently understand how the clinical presentations of the disease vary according to the organ, the fact that this is the first randomized study in Japan, and that the maintenance of foundation for clinical studies of this disease in Japan is inadequate at present time, the hurdle is too high to plan a study across all organs including the fields of gynecology and urology. Based on the above consideration, we have decided to target all gastrointestinal organs which have a relatively large number of common points between each other in terms of clinical presentation and types of complication.

According to the multicenter observatory study by Yamaguchi and Machida, the prognosis of NEC with hepatobiliary or pancreatic primary lesion was significantly poorer than that of NEC with gastrointestinal primary lesion (MST: 7.9 vs. 13.0 months, respectively), but we determined that it is possible to accurately evaluate the efficacy of the treatment regimen by randomizing the subjects using the primary lesion site (gastrointestinal vs. hepatobiliary or pancreatic) as an allocation adjustment factor. Therefore, we considered patients with both, gastrointestinal/hepatobiliary or pancreatic primary lesions as one target population of this study, and to examine differences between organs exploratively by subgroup analysis.

2) Reason for including hepatic NEC cases (hepatic primary lesion or liver metastasis with primary lesion site unknown)

Even among NECs, cases with hepatic primary lesions are particularly rare. There are no specific reports on the frequency of incidence and differences from the other organs and reports are limited only to case reports and their reviews. Moreover, since the liver is a major organ for metastasis of primary lesions into other organs, even when tumors have been clinically identified in the liver, in many cases the primary lesion tends to be in another organ. Therefore, liver should not be deemed the primary lesion site without sufficient examination for a primary lesion. On the other hand, although very rare, there have been reports of NEC with hepatic primary lesion[28]. Even if sufficient search for primary lesion leads to no indication of the same outside of the liver, currently it is not possible to distinguish whether it is a case of “NEC with hepatic primary lesion” or “lesion in the liver is a metastatic lesion, with the primary lesion unknown”. In this study, such situations wherein “liver alone has identifiable lesions” would be henceforth, referred to as “hepatic NEC (hepatic primary lesion or liver metastasis from an unknown primary lesion site) for convenience. As the NCCN Guidelines[19] and ENETS Guidelines[20, 21] indicate the usefulness of FDG-PET for detailed examination of the primary lesion, we would also examine the primary lesion using FDG-PET. Furthermore, by referring to the diagnostic procedures for cancers with unknown primary lesion site, detailed examinations into the primary lesion would be carried out by otolaryngological (head and neck) examination and urological examination for men only, and gynecological examination for women only).

Such hepatic NEC (hepatic primary lesion or liver metastasis from an unknown primary lesion site) is anticipated even from an anatomical viewpoint, since the tumor-related complications are the same as that for other gastrointestinal primary lesion NECs, and there is no issue with treating them the same as for gastrointestinal primary lesions.

3) Reason for orienting the study for non-resectable or recurrent cases

As mentioned in 2.1.4 “Standard treatment according to disease staging and outline of prognosis”, systemic chemotherapy is indicated for cases with distal metastasis or recurrent cases. Locally advanced cases are particularly prone to pancreatic or bile duct primary lesions situated in the vicinity of vital vessels, and while the NCCN Guidelines propose chemoradiotherapy according to treatment for small cell lung carcinoma[19], the ENETS Guidelines provide no clear indication[21]. In reality, for the treatment of NEC which has a variety of primary lesion organs (scope of irradiation) there is not enough information available for the chemotherapy regimen that should be combined in terms of its optimum dose, the radiation dose, efficacy, and safety. For this reason, chemoradiotherapy cannot be considered the standard treatment with consensus, and systemic chemotherapy for distal metastasis is used widely except for pathologies involving frequent stenotic symptoms when the primary lesion is in the esophagus. For this reason, this study would enroll non-resectable or recurrent cases that include locally advanced cases.

The section “3.6 Definition of non-resectable NEC” shows the definition of non-resectable cases summarized by referring to the JCOG protocol for clinical studies of systemic chemotherapy (esophagus: JCOG0807, stomach: JCOG1013 or JCOG1002, bile duct: JCOG0805, pancreas: JCOG1106). Furthermore, while radical resection has

been considered for cases of colonic primary lesion adenocarcinoma with liver or lung metastasis, as cases of NEC with distal metastasis are not indicated for resection, we defined non-resectable cases as those in “Stage IV”, and followed this definition for duodenal primary lesions, small intestine primary lesions, and appendix primary lesions. Furthermore, with respect to NEC with esophageal primary lesion, chemoradiotherapy is well-indicated for cases with supraclavicular lymph node metastasis of thoracic esophageal tumor even in Stage IV, and hence were not included in this study.

4) Treatment of draft of new classification of NEC

In recent years, proposals have been made to further differentiate the WHO 2010 classification of NECs (G3 in ENETS) to “Grade 3 proliferative tumors showing the same morphological presentations as NET” and “Grade 3 tumors with strong morphological atypicality (previously classified as poorly differentiated NECs)”[29, 30], or by Ki-67 index of 20–50% and 50% or more[11, 19]. This is yet to be confirmed; however, in this study we would have used the WHO 2010 classification. However, we would have used samples collected for central pathological diagnosis to carry out studies related to classification which exploratively adds grade classification and morphological differentiation levels.

2.2. Standard treatment for target disease

Currently, there is no standard drug treatment for non-resectable/recurrent NECs with efficacy verified by a randomized controlled trial. However, given the similarity between pathological and clinical presentations, treatments according to small cell lung carcinoma have been attempted, and there have been reports of positive treatment outcome in small-scale clinical studies and observational studies.

2.2.1. Standard treatment for small cell lung carcinoma

Cisplatin-based multidrug combined therapy is the standard first-line treatment of extensive disease (ED) small cell lung carcinoma. While cyclophosphamide/doxorubicin/vincristine (CAV therapy) was established as a standard treatment in the 1970s in Europe and U.S., the etoposide/cisplatin combined therapy (EP therapy) was introduced in the late 1980s. A comparative study of EP therapy and CAV therapy did not show superiority of EP therapy over CAV therapy in terms of survival, but the response rate (CAV: 51% vs. EP: 61%) and MST (CAV: 8.3 months vs. EP: 8.6 months) were almost the same, and the lighter toxicity level (mucositis, interstitial pneumonia, hemotoxicity) meant that EP could be used as a standard treatment[31]. Thereafter, the JCOG Lung Cancer Group carried out the “comparison between EP therapy and Irinotecan/Cisplatin combined therapy (IP therapy) for Extensive-stage Small Cell Lung Cancer (JCOG9511)”, and reported that the IP therapy provided a significantly better overall survival, with MST being 9.4 months vs. 12.8 months (p = 0.002) for IP therapy[32]. However, the two additional large-scale studies conducted primarily in U.S. did not show superiority of IP therapy over EP therapy, and for this reason IP therapy has not been not used as the standard treatment overseas[33, 34]. Furthermore, while the results of the “Randomized controlled trial to verify the non-inferiority of amrubicin/cisplatin combined therapy (AP therapy) over IP therapy (JCOG0509)” were reported in the 2012 conference of the American Society of Clinical Oncology (ASCO), but AP therapy was still considered significantly inferior to IP therapy[35]. Presently, the JCOG Lung Cancer Group considers IP therapy to be the standard treatment for extensive-disease small cell lung carcinoma.

2.2.2. Standard treatment for extrapulmonary NEC

While there are scattered reports relating to use of CAV therapy, EP therapy, IP therapy, and other multidrug combined therapies for extrapulmonary NEC, these reports are primarily from observational studies. Since these reports precede the unification of concept and classification methods of this disease, the nomenclature such as NEC (anaplastic type), NET (poorly differentiated type) and extrapulmonary small cell lung carcinoma have still been used. These reports, however, are presumed to be targeting almost the same disease group as NEC. There are no reports of prospective studies using EP therapy for extrapulmonary NEC. Observational studies have reported response rates between 42–67%, and MST between 15–19 months (Table 2.2.2a). On the other hand, IP therapy has reported results from observational studies and clinical studies, with response rates between 7–83% and MST of 10.1–22.6 months (Table 2.2.2b). As mentioned above, reports related to extrapulmonary NEC are limited to use of EP therapy and IP therapy in observational studies and small-scale clinical studies. The largest number of case enrollments in a clinical study of extrapulmonary NEC was seen in the Minnie-Pearl Cancer Research Network Study, which evaluated the efficacy of triple-drug combined therapy using carboplatin/etoposide/paclitaxel. The

response rate to this triple-drug combined therapy was 53%, while the MST was 14.5 months and adequate, there was intense toxicity involved, and the results were not significantly different from EP therapy, so this regimen was not considered the standard treatment[27].

The 2014 NCCN Guidelines recommend treating extrapulmonary NEC using a regimen according to small cell lung carcinoma, and the guidelines for treatment of small cell lung carcinoma mention both EP therapy and IP therapy as recommended regimens.

Therefore, the standard treatment for extrapulmonary NEC is taken as EP therapy or IP therapy.

Table 2.2.2.a. EP therapy for NEC (observational studies)

Reported year/Reporter	Target	N	Response rate	MST (in months)
1991/ Moertel[18]	Pancreatic/gastrointestinal NEC (anaplastic type)	18	67%	19
1999/ Mitry [36]	Pancreatic/gastrointestinal NEC	41	42%	15
2001/ Marie-Louise [37]	Pancreatic NET (well: 11, poorly: 4) Gastrointestinal cartinoid: 21	33	18%	19
1994/ Lo Re G [38]	Extrapulmonary SCLC	13	69%	NE
2010/ Iwasa [39]	Hepatobiliary or pancreatic NEC	21	14%	7.3
2012/ Yamaguchi [10]	Gastrointestinal NEC	12	75%	14
2012/ Yamaguchi [10]	Hepatobiliary or pancreatic NEC	34	12%	6.9
2012/ Sorbye [11]	Gastrointestinal primary lesion NEC (including primary lesion unknown)	129	31%	12

Table 2.2.2.b. IP therapy for NEC (shaded are clinical studies, others are observational studies)

Reported year/Reporter	Target	N	Response rate	MST (in months)
2003/ Hou [40]	NEC (gastrointestinal-80%)	18	43%	NE
2005/ Chin [41]	Esophageal NEC	12	83%	14
2011/ Okita [42]	Gastric NEC	12	75%	22.6
2012/ Yamaguchi [10]	Gastrointestinal NEC	142	51%	13.4
2012/ Yamaguchi [10]	Hepatobiliary or pancreatic NEC	18	39%	10.1
2006/ Kulke [43]	Pancreatic/gastrointestinal (including NET G1/G2)	15	7%	11.4
2008/ Mani [44]	Pancreatic/gastrointestinal NEC	20	58%	NE
2008/ Jin [45]	Extrapulmonary NEC	15	67%	11.4.

Table 2.2.2.c. Phase II study of carboplatin/etoposide/paclitaxel triple-drug combined therapy for NEC

Reported year/Reporter	Target (breakdown)	N	Response rate	MST (in months)
2006/ Hainsworth [27]	NEC (Colon: 9, lung: 7, skin: 4, pancreas: 3, gall bladder: 1, thyroid:1, stomach: 1, esophagus: 1, endometrium:1, maxillary sinus: 1, prostate: 1, primary lesion unknown: 48)	78	53%	14.5

2.2.3. Standard treatment for gastrointestinal/hepatobiliary or pancreatic primary NEC

Treatments for extrapulmonary NEC have not been developed according to any specific organ, and the standard treatment for gastrointestinal/hepatobiliary or pancreatic NEC is also both, EP therapy and IP therapy. While both treatments are considered standard treatments, since this study considers them both to be study treatments, the anticipated effect and expected adverse reactions from EP therapy and IP therapy have been described in "2.3.2 Study treatment(s) of this study".

Herewith, we describe the current status of the usage of each treatment in Japan and overseas. According to the multicenter joint study by Yamaguchi and Machida, 160 out of 258 patients (62%) who received systemic chemotherapy underwent IP therapy, the most common treatment, followed by EP therapy (46 patients, 18%). Although there is big deviation where 92% of gastrointestinal primary lesion NEC (142/154 patients) were given IP therapy, and 65% of hepatobiliary or pancreatic primary lesion NEC (34/52 patients) were given EP therapy, the present situation shows that treatments are being selected by discretion or preference of the facility/physician.

Furthermore, even with respect to the dosing schedule, the method of administration has not necessarily as per the treatment of small cell lung carcinoma. There is no unified consensus, as some facilities use methods used for treatment of gastric cancer (JCOG9912 regimen), based on the reasoning that they have familiarity with IP therapy. On the other hand, in the NORDIC NEC study the most common treatment used was the EP therapy, which was administered to 129 out of 252 patients (51%) who received systemic chemotherapy, followed by a combined therapy consisting of carboplatin and etoposide therapy (67 patients, 27%). Combined therapy of platinum and etoposide was used often, which is another choice of treatment in Japan[11].

2.3. Rationale for establishment of treatment plan

2.3.1. Drugs

1) Etoposide

Etoposide exerts an antitumor effect by inhibiting topoisomerase II, which catalyzes the untangling of supercoiled DNA strands. Main toxicities include myelosuppression, nausea/vomiting, alopecia, and stomatitis.

2) Cisplatin

It is a complex ion form of the heavy metal platinum which shows anti-tumor effect by cross-linking double-stranded DNA. Currently, it is considered one of the key drugs for NEC treatment, being a central medicine for the treatment of lung cancer due to its synergistic effect with radiation therapy and various drugs, and also due to its low bone marrow toxicity when used alone. Toxicities include nausea/vomiting, nephrotoxicity, and neurotoxicity. Sufficient infusion of cisplatin before and after drug administration is necessary to prevent nephrotoxicity.

3) Irinotecan

Irinotecan is a topoisomerase I inhibitor developed in Japan that inhibits DNA synthesis. The drug is directly converted into the active metabolite (SN-38) in human liver and various tissues by carboxyesterase. As it shows a potent antitumor effect against SCLC even when used on its own, this drug is used very often in routine clinical practice to treat NEC as well. Diarrhea and myelosuppression are observed as dose-dependent toxicities. Nausea/vomiting and interstitial pneumonia have also been noted in patients.

2.3.2. Study treatment(s) of this study

1) Etoposide/cisplatin combined therapy (EP therapy)

In the multicenter joint study performed by Yamaguchi and Machida, the response rate of gastrointestinal primary lesion NEC to EP therapy was 75% (9/12 patients), with an MST of 14 months. The response rate of hepatobiliary and pancreatic primary lesion NEC to EP therapy was 12% (4/34 patients), and the MST was 6.9 months. With respect to safety, the multicenter joint study by Yamaguchi and Machida showed no treatment-related deaths associated with EP therapy (N = 46) administered as first-line treatment for gastrointestinal/hepatobiliary and pancreatic primary lesion NEC, and toxicity-related study discontinuation was observed in 6.5% of cases. Details of toxicities remain unknown as they were not investigated. The observational study of EP therapy for hepatobiliary and pancreatic primary lesion NEC (N = 21) carried out by Iwasa et al. at the National Cancer Center Hospital showed major Grade 3/4 adverse events to be neutropenia (90%), nausea (33%), and anorexia (24%). Grade 3 febrile neutropenia was observed in 8 patients (38%)[39]. Table 2.3.2 shows the toxicity profile of EP therapy from the JCOG9511 study which treated small cell lung carcinoma (Grade 3 or higher as per JCOG toxicity criteria).

Table 2.3.2. Toxicity of EP therapy and IP therapy in studies treating small cell lung carcinoma※
※Report by Iwasa et al., JCOG0509：CTCAE v3.0 Grade 3 or higher, JCOG9511：JCOG toxicity criteria Grade 3 or higher

	EP therapy		IP therapy	
	Report by Iwasa et al. [34]	JCOG9511[27]	JCOG9511[27]	JCOG0509[30]
Neutropenia	90%	92.2%	65.3%	58.5%
Leukopenia	71%	51.9%	26.7%	22.5%
Decreased hemoglobin	29%	29.9%	26.7%	23.2%
Thrombocytopenia	24%	18.2%	5.3%	2.1%
Diarrhea	0%	0%	16%	7.7%

Nausea	33%	6.5%	13.3%	6.3%
Aspartate aminotransferase increased	19%	2.6%	0%	-
Alanine aminotransferase increased	24%	3.9%	4%	-
Blood bilirubin increased	19%	0%	0%	-
Creatinine increased	0%	0%	0%	-
Peripheral motor neuropathy	0%	0%	0%	-
Febrile neutropenia	38%	0%	0%	1.4%

There are several reports concerning the specific dosage schedule/administered dose for EP therapy, and four dosing methods have been described in the NCCN Guidelines for SCLC as well. However, it is not clear as to which of the dosing methods is the best, and the dosing method for NEC varies between reports. In Japan, the dosing for EP therapy according to JCOG9511 is also often used for NEC. For this reason, this study would also follow the same method of administration. In terms of the number of cycles, a comparison of 4 courses vs. 8 courses of combined chemotherapy including cyclophosphamide for SCLC showed poor efficacy even when the treatment continued for long-term[46], and treatment of 4 cycles has also been specified in JCOG9511. There have been no such comparative studies for gastrointestinal/hepatobiliary and pancreatic primary lesion NEC, rather, there is no evidence to suggest that first-line chemotherapy should be discontinued if the chemotherapy is efficacious and toxicity is within the permitted scope. Unlike SCLC, NEC does not respond well to chemotherapy and tends to re-exacerbation to the underlying disease soon after the end of chemotherapy. For this reason, continuation of chemotherapy is very likely to be beneficial for patients with NEC as long as the treatment is effective.

While peripheral motor neuropathy, hearing impairment, and renal disorder are known accumulation toxicities of cisplatin, if sufficiently safe, it is ideal for treatments to continue as much as possible, considering the very few treatment options available for NEC. Based on the above rationale, we decided to ensure safety by establishing strict protocol treatment discontinuation criteria and chose not to limit the total dose or prescribe the number of treatment cycles.

2) Irinotecan/Cisplatin combined therapy (IP therapy)

In the multicenter joint study by Yamaguchi and Machida, the response rate of gastrointestinal primary lesion NEC to IP therapy was 51% (73/142 patients), with an MST of 13.4 months. The response rate of hepatobiliary and pancreatic primary lesion NEC to IP therapy was 39% (7/18 patients), and the MST was 10.1 months. There were no treatment-related deaths among 160 patients, and discontinuation due to toxicity in first-line chemotherapy was seen in 11.2% of cases. Details about toxicity were not collected. As a reference, Table 2.3.2 shows the toxicity profile of IP therapy from the JCOG9511 and JCOG0509 studies which treated small cell lung carcinoma.

There are several reports concerning specific dosing methods for IP therapy, and two dosing methods have been described in the NCCN Guidelines for SCLC as well. It is not known which of the dosing methods is the best, and the dosing method has also been variable in the NEC. In Japan, the dosing method of IP therapy, according to JCOG9511 is considered the standard treatment for SCLC and the same method has also been used in this study.

The total dose and number of cycles would have not been prescribed for the same reason as EP therapy, and safety has been ensured by establishing strict protocol treatment discontinuation criteria.

3) Other study treatment candidates

Presently, there are no drugs or treatment methods, including molecular-targeted drugs, which surpass the EP and IP therapy. There are also no planned or ongoing large-scale clinical trials globally, related to first-line treatment of NEC.

As the second-line treatment, a phase II study of Everolimus is underway for pancreatic primary lesion NEC resistant or unresponsive to platinum preparations. It is a multicenter study being carried out in 31 facilities in Japan, led by the National Cancer Center East Hospital, through the cancer research and development fund (UMIN000012752).

2.3.3. Summary of risk/benefit balance of the standard treatment and study treatment

In the multicenter joint study by Yamaguchi and Machida, IP therapy had better prognosis compared to EP therapy (MST: 13.0 months vs. 7.3 months; $p < 0.0001$). However, as shown in Table 2.3.3, since IP therapy was more frequently chosen for gastrointestinal primary lesions (142/154 patients) and EP therapy for hepatobiliary and

pancreatic primary lesions (34/52 patients), it has not been possible to determine whether this difference is due to difference in efficacy between the regimens, or due to differences in the primary lesion organ. Primary lesion organ remained a significant prognostic factor after multivariate analysis, and while IP therapy turned out to be a slightly better regimen than EP therapy with HR = 0.8 (95% CI. 0.48–1.33), $p = 0.389$ indicated that there was no significant difference between the two (see "2.1.5 Prognostic/predictive factors"). Taking into account the number of EP therapy cases (46 patients), the fact that it was an observational study, and including other unknown bias, the data interpretation is fairly limited. Therefore, based on these results we are unable to deem either of IP or EP therapies to be more efficacious than the other.

With regard to toxicity, while myelosuppression such as neutropenia is milder in IP therapy than EP therapy, frequencies of events such as diarrhea and nausea are high. While the two treatments have different toxicity profiles, it is difficult to conclude that one has clearly worse toxicity than the other. The risk of bile duct obstruction is high in biliary and pancreatic primary lesion NECs and the use of irinotecan (which undergoes biliary excretion) in patients exhibiting biliary excretion disorder runs the risk of increased toxicity, hence care must be taken while administering irinotecan. We believe that this risk can be avoided by appropriate pre-treatment drainage and proper monitoring during treatment. Based on the above, it is difficult to assign superiority to IP therapy or EP therapy over the other in terms of risk/benefit balance, and both the regimens can be considered suitable standard treatments.

Table 2.3.3. Summary of results from multicenter study by Yamaguchi and Machida

		IP	EP	*p*-value*
Total	N	160	46	
	Response rate （%）	50	27	＜0.001
	Median PFS (in months)	5.2	4.0	0.033
	Median OS (in months)	13.0	7.3	＜0.0001
Gastrointestinal primary lesion NEC	N	142	12	
	Response rate （%）	51	75	0.14
	Median PFS (in months)	5.4	4.9	0.585
	Median OS (in months)	13.4	14.0	0.976
Hepatobiliary and pancreatic primary lesion NEC	N	18	34	
	Response rate （%）	39%	12%	0.034
	Median PFS (in months)	4.4	3.7	0.056
	Median OS (in months)	10.1	6.9	0.05

*Response rate by chi-squared test, PFS and OS by log-rank test

2.3.4. Post-treatment(s)

In the multicenter joint study by Yamaguchi and Machida, 56% (116/206 patients) of patients with NEC who received IP therapy or EP therapy were administered chemotherapy as the second-line of treatment. The most common second-line chemotherapy after IP therapy was amurcibin (22/88 patients), while irinotecan was the most common second-line treatment after EP therapy (13/28 patients), and we expected a similar pattern for the second-line treatments in this study as well. The overall performance of second-line chemotherapy was poor with a response rate of 11%, and PFS of 2.1 months. As such, the significance of the second-line treatment has not been indicated, and hence, no standard treatment has been established.

2.4. Study design

2.4.1. Rationale for establishing endpoints

For NECs, an alternative endpoint for overall survival, such as PFS has not been established as an indicator for comparing the usefulness of the treatment regimens. Therefore, comparison of overall survival was considered appropriate when examining the therapeutic effects. For the same, overall survival was examined as the primary endpoint. The secondary endpoints to evaluate the efficacy and safety were the response rate (only for patients with a measurable lesion), progression-free survival, incidence rate of adverse events, and dose intensity of cisplatin.

2.4.2. Clinical hypothesis and rationale for setting the number of enrollments

This study compares the two standard treatments, both of which are difficult to deem superior than the other in terms of efficacy and safety. We have, therefore, adopted a study design using bilateral testing. The main clinical hypothesis of this study is that either IP therapy or EP therapy is likely to provide better overall survival than the other treatment group, and should this hypothesis be validated, the superior treatment with statistical significance would be deemed the better treatment, and would therefore be positioned as the standard treatment in future. If the study fails to validate this hypothesis, it would indicate that there was no clinically significant difference in overall survival between the two groups, and if there are no large differences in terms of toxicity, we would conclude that both treatments are viable options as the standard treatment. However, if unlike our initial hypothesis, one treatment shows clearly more toxicity than the other, we would reconsider the study design before carrying out the main analysis.

Furthermore, as mentioned in “section 2.1.1 Epidemiology”, gastroenterological primary lesion NEC, which is the primary target of this study, is a very rare disease with an annual prevalence of around 3 in 100,000 people, hence, the significance level of testing has been placed at 10% bilaterally, instead of 5% bilaterally. In the multicenter joint study conducted by Yamaguchi and Machida, the MST of gastrointestinal/hepatobiliary or pancreatic primary lesion NEC was 11.5 months. To determine if one treatment is superior to the other, we expected a difference of four months in terms of MST. If we expect the more inferior treatment to have an MST of 8.0 months and the superior treatment to have an MST of 12.0 months, the number of enrollments are calculated using the aforementioned parameters and based on the discussion mentioned later (see “12.2 Expected number of enrollments/Enrollment period/Follow-up period”). This provided a research period of 7- years, consisting of 6-years of enrollment period and one year of follow-up period, and with $\alpha = 0.1$ bilaterally and detection power of 70% to detect differences between the two groups, 63 subjects were required per group. Taking into consideration some subjects who could be lost post follow-up, we aimed to enroll 70 subjects per group, and a total of 140 subjects between two groups.

If enrollment proceeds better than expected than prior to the start of the study (i.e. if the number of enrolled subjects reach 70 in less than 2.5 years from the start of enrollment), the number of enrollments is likely to be re-established by changing the detection power from 70% to 80% during the study, with the aim of obtaining more accurate results.

＜Addition to ver.1.1＞

The pace of enrollment after the start of enrollment period exceeded expectations, and the number of enrollments reached 70 subjects in October 2016, which was 2 years and 2 months after the start. As this exceeded the criteria of 70 subjects in less than 2.5 years, since the start as provided above, we obtained the approval of the Hepatobiliary and Pancreatic Oncology Group Meeting on October 29, 2016, Esophageal Cancer Group Meeting on November 19, 2016, and Gastric Cancer Group Meeting on January 7, 2017 to change the detection power to 80% according to provisions at the time of study planning. Consequently, the number of intended enrollments was changed to 170 subjects.

2.4.3. Expected patient enrollment

In the multicenter joint study by Yamaguchi and Machida, 258 cases of gastrointestinal/hepatobiliary or pancreatic primary lesion NECs were reported from 23 sites during the 11 years between 2000 and 2011, but between 2000 and 2006, information could not be obtained because of old cases. When limited to the most recent five years (2006–2011), when sufficient information was obtained from each facility, the number of enrollments was placed at162 subjects.

This is a joint study between three groups, namely the JCOG Hepatobiliary and Pancreatic Oncology Group,

JCOG Gastric Cancer Group, and the JCOG Esophageal Cancer Group. Therefore, the total number of facilities, excluding overlaps, amounted to 82 facilities. Compared to the multicenter joint study by Yamaguchi and Machida, we expect an increase in the number of patients enrolled, and simple calculations lead us to extrapolate 115 subjects to be enrolled annually. On the other hand, if we take into account the possibility of patient enrollments being biased to some high volume centers, it would be difficult to estimate the effect of increasing facility count to proceed. In addition, considering that the study is a randomized, we estimate the annual number of patients enrolled to be between 30–50 people. Taking into account ineligible cases, we provided 6-years for patient enrollment period.

2.4.4. Rationale for setting allocation adjustment factors

1) Facility

It is widely known that background, treatment, efficacy evaluation, and safety evaluation of enrolled patients vary depending on the facility, and JCOG standards have been used to make adjustments between facilities.

2) Primary lesion organ (gastrointestinal tract vs. hepatobiliary or pancreatic organ)

In the multicenter joint study by Yamaguchi and Machida, multivariate analysis using the Cox proportional hazard model was carried out with 183 subjects, which indicated primary lesion organ (gastrointestinal tract vs. hepatobiliary or pancreatic organ) to be a significant prognostic factor.

2.4.5. Centralized pathological diagnosis

In this study, a centralized pathological diagnosis would be performed for analysis of NEC, despite patients being diagnosed by a third party. The operation of the centralized pathological diagnosis is described in section 15.1, while details of the operation have been described in the Centralized Pathological Diagnosis Procedure Manual. Furthermore, an analysis of endpoints based on the results of centralized pathological diagnosis would be provided as a reference.

2.5. Summary of expected advantages and disadvantages associated with study participation

2.5.1. Expected advantages

Drugs used for both groups of this study are treatments used in routine medical practice. As mentioned in the next section, although it would be necessary to use some drugs for which insurance coverage does not apply, since insurance claims for these treatments are made in a similar manner to general medical care however, in practice they do not receive insurance assessments.

Furthermore, cases of off-label medication have been studied according to "18th Case Providing Review Information" at the Case Review Committee established by the Health Insurance Claims Review & Reimbursement Services. Based on the review information provided as on February 26, 2018;, The use of "irinotecan hydrochloride hydrate, etoposide, cisplatin, and carboplatin [injections]" is approved for treatment of "neuroendocrine carcinoma"], and the notices issued by the Ministry of Health, Labor and Welfare on February 26, 2016the results of this study have been considered valid by the ministry. For the same reason, essentially since February 26, 2018, there have been no concerns about claims of insurance assessments.

Moreover, since the medical fees of study participants during the study, which includes drug fees, are paid in principle by the patients themselves and their insurance coverage, so the patients would receive no special medical or financial benefits from participation in this study.

2.5.2. Expected risks and disadvantages

Both treatment arms A and B would receive chemotherapy regimen used in routine medical practice, so they would be unlikely to be exposed to special risks or disadvantages not observed in routine medical practice. The descriptions in "2.3.3 Risk/benefit balance of standard treatment and study treatments" outline the expected risks and disadvantages for each treatment arm.

To minimize the risk of adverse events and disadvantages, the " Patient selection criteria (Section 4)", " Criteria to change treatment (Section 6.3)" and "Concomitant/Supportive therapies (Section 6.4)" have been carefully considered for the three groups. Furthermore, the Institutional Review Board would monitor if the adverse events are within the scope of expectation, while any serious adverse events or unexpected adverse events would be carefully examined and reviewed according to the provisions related to "JCTN-Adverse Events Report Guideline"

and "JCOG Guidelines for Handling Clinical Safety Information", and a system has been provided to take any necessary countermeasures.

Precautions related to etoposide, irinotecan, and cisplatin

As of December 2013, etoposide therapy, which is the intended to be used in this study had not received insurance approval for the treatment of gastrointestinal or hepatobiliary and pancreatic primary lesion cancers. Irinotecan has also not received insurance approval for treatment of cancers with primary lesions in organs other than the stomach, colon, and rectum. Furthermore, as cisplatin is only indicated for esophageal, gastric cancer, and for biliary tract cancers when combined with gemcitabine (25 mg/m^2), the dosage and administration used in this study have not been approved previously.

As each facility in this study may carry out the insurance claims in the same manner as routine general medical practice, the treatment may receive insurance assessment after-the-fact. However, if a facility incurs losses, the loss must be borne by the relevant facility (medical institution), as there is no system of compensation arranged by the JCOG Research Organization. If actual losses occur, the continued participation in the study would then be carefully discussed between the facility supervisor and the principal investigator/clinical trial secretariat. The facility supervisor would be expected to gain approval from the facility IRB and the head of the medical institutions.

2.6. Significance of this study

While both, EP therapy and IP therapy are standard treatments, the efficiency of either treatment being better than the other is not yet clear, and currently in routine medical practice, the choice of treatment is being made on the discretion or preference of the facility/attending physician.

If this study is able to clearly indicate the efficiency of EP therapy or IP therapy over the other, we expect that the diminished use of the relatively inferior treatment would contribute to improvement in patient prognosis. If the study indicates no clinically meaningful differences between the two treatments, the results would provide evidence that there is no significant issue with the treatments being chosen by discretion or preference of the facility/attending physician, which has been done traditionally without any data that directly compare the two treatments, although that would not indicate that the effects of the two treatments are equivalent. We also expect that the establishment of a highly reliable standard treatment in this study which would provide a foundation for therapeutic development when promising treatment regimen in the future.

As NEC is a rare disease, and since each clinician is not likely to have adequate experience, we believe that work associated with multicenter studies such as confirmation of diagnosis by centralized diagnosis/results feedback and sharing results of treatments by unified treatment regimen would lead to improvement of quality of care for NEC in Japan.

2.7. Associated research (including sample analysis research)

No such studies have been planned or carried out at the time of preparation of the protocol.

2.8. JCOG-Biobank Japan (BBJ) collaborating biobank

This study would participate in the banking of blood samples (DNA/plasma) through the JCOG-BBJ collaboration biobank based on the protocol common to all JCOG studies (hereafter termed as "common banking").

Common banking by JCOG-BBJ collaborating biobank would collect and store the samples of patients enrolled in clinical studies conducted by JCOG regardless of presence/absence of pre-planned sample analysis research, would also provide samples for future analysis research and diagnostic information obtained through the main research.

The target of such biobanking are those patients who provided consent to participate in this study and gave consent to provide samples to the JCOG-BBJ collaborating biobank for their use in future sample analysis research (hereafter termed as consent for banking).

Samples collected during common banking include whole blood and preserved pathological tissue samples from routine clinical practice. Plasma and DNA separated/isolated from blood would be stored in the JCOG-BBJ collaborating biobank and would be provided for sample analysis research in the future. Preserved pathological tissue samples from routine medical practices such as surgery, and biopsy/clinical laboratory tests would also likely to be used for future sample analysis research. Although the type, sample preparation methods, and tissue quantity required would vary from study to study, there is no definite consensus that banking pathological tissue in a certain

method is more efficient than others. Furthermore, it has been suggested that long-term preservation of a sectioned specimen from preserved pathological tissue may lead to sample deterioration (DNA fragmentation). The discussions between the of JCOG and BBJ personnel about these problems, led to the conclusion that preserved pathological tissue taken after medical examination shall require only patients' consent for future use, and actual collection must be started after standardizing a separate protocol and specifying in it the procedures best suited for the details of the research.

The detailed procedures for sample collection, storage, and method of sample provision for future sample analysis research in common banking have been stipulated in the "JCOG-BioBank Japan Collaborating Biobank Protocols" that apply to all the JCOG studies. To participate in the common banking carried out by JCOG-BBJ collaborating biobanks, the subject matter must be reviewed and approved by the ethics committees of each participating facility.

Furthermore, to conduct sample analysis research in future using samples stored in the JCOG-BBJ collaborating biobank, it would be necessary to prepare a "Sample Analysis Research Protocol" and have it reviewed and approved by the JCOG Protocol Review Committee and the ethics committee of facilities involved in the sample analysis.

3. Criteria/definitions used in this study

Tissue classification would be performed according to WHO 2010 classification and ENETS（European Neuroendocrine Tumor Society） classification[14, 15], while disease staging would be done according to "UICC-TNM 7th Edition".

3.1. Tissue classification (WHO 2010 classification)

The shaded parts are the targets of this study

Neuroendocrine neoplasms

1) Neuroendocrine tumor：NET Grade 1（NETG1）
2) Neuroendocrine tumor：NET Grade 2（NETG2）
3) Neuroendocrine carcinoma：NEC（large cell or small cell type）
4) Mixed adenoendocrine carcinoma（MANEC）
5) Hyperplastic and preneoplastic lesions

3.2. Grade classification （ENETS [European Neuroendocrine Tumor Society] / WHO2010 classification）

Grade 1（G1）	Number of mitotic presentations < 2 per 10 high power fields（HPF）and/or Ki-67 index ≤2%*
Grade 2（G2）	Number of mitotic presentations 2–20 per 10 HPF and/or Ki-67 index 3-20%
Grade 3（G3）	Number of mitotic presentations > 20 per 10 HPF and/or Ki-67 index >20%

*The description of number of mitotic presentations in WHO 2010 classification is ≤2, but 2–3% are classified to G1[47].

3.3. Histopathological diagnosis

- Immunostaining (Chromogranin A and synaptophysin) is essential for a pathological diagnosis of NEC.
- Either1. or 2., or both are studied to determine proliferative activity. Number of mitotic presentations and Ki-67 index would adopt a high grade evaluation.
  1. Number of mitotic presentations (to evaluate 50HPF)
  2. Ki-67 index（500–2000 tumor cells*）

  *（Only in this study, if the number of tumor cells in the collected sample is less than 500, the number of tumor cells measured is also listed. A minimum of 100 tumor cells is required.）
- If sufficient amount of biopsy sample cannot be collected for a pathological diagnosis, a cell block prepared using material obtained from EUS-FNA, brushing or needle biopsy may be used. However, the use of a cell block prepared from ascites or pleural effusion is not permitted.
- During a histological diagnosis using a resected tumor sample, a slide with a representative section shall be used to evaluate the proportion of NEC components (differential diagnosis with mixed adenoendocrine carcinoma). For a comprehensive examination, the entire tumor must be evaluated.

For further details NEC Pathological Diagnosis Handbook would be referred (posted on JCOG website).

3.4. Disease stage classification criteria (UICC-TNM 7th Edition)

Special notice regarding disease stage classification: Although there is an independent TNM classification for NET G1 and NET G2 with primary lesions in the stomach, small intestines, and colon; since this study concerns NEC, the disease would be classified according to the classification method of major tissue type in each primary lesion organ (squamous cell carcinoma for esophagus, and adenocarcinoma for all other primary lesion organs). In this study, the classification of intrahepatic cholangiocarcinoma is used for hepatic NEC, due to their similar clinical presentation.

3.4.1. Esophagus

T –Primary tumor

TX： Primary tumor cannot be evaluated
T0： Primary tumor unidentified
Tis： Epithelial carcinoma/highly dysplastic
T1： Tumor invading mucosal lamina propria, muscularis mucosae, or submucosa
  T1a：Tumor invading the mucosal lamina propria or muscularis mucosae
  T1b： Tumor invading the submucosa

T2 : Tumor invading the muscularis propia

T3 : Tumor invading the adventitia

T4 : Tumor invading the surrounding tissue

T4a : Tumor invading the pleura, pericardium, and diaphragm

T4b : Tumor invading other surrounding tissues such as the aorta, centrum, trachea

N –Regional lymph nodes

NX : Regional lymph node metastasis cannot be evaluated

N0 : No regional lymph node metastasis

N1 : 1–2 foci of regional lymph node metastases

N2 : 3–6 regional foci of lymph node metastases

N3 : 7 or more foci of regional lymph node metastases

M –Distal metastasis

M0 : No distal metastasis

M1 : Distal metastasis present

Stage	T	N	M
**0**	Tis	N0	M0
**IA**	T1	N0	M0
**IB**	T2	N0	M0
**IIA**	T3	N0	M0
**IIB**	T1, T2	N1	M0
**IIIA**	T4a	N0	M0
	T3	N1	M0
	T1, T2	N2	M0
**IIIB**	T3	N2	M0
**IIIC**	T4a	N1、N2	M0
	T4b	Unrelated to N	M0
	Any T	N3	M0
**IV**	Any T	Any N	M1

3.4.2. Stomach

T –Primary tumor

TX : Primary tumor cannot be evaluated

T0 : Primary tumor unidentified

Tis : Epithelial carcinoma: Epithelial carcinoma/highly dysplastic carcinoma not invading the lamina propia mucosa

T1 : Tumor invading mucosal lamina propria, muscularis mucosae, or submucosa

T1a : Tumor invading the mucosal lamina propria or muscularis mucosae

T1b : Tumor invading the submucosa

T2 : Tumor invading the muscularis propia

T3 : Tumor invading the subserosa

T4 : Tumor perforating the serosa, or invading adjacent structures[1,2,3]

T4a : Tumor perforating the serosa

T4b : Tumor invading adjacent structures[1,2,3]

1. Adjacent organs of the stomach are spleen, transverse colon, liver, diaphragm, pancreas, abdominal wall, adrenal glands, kidneys, small intestine, and retroperitoneum.
2. If the invasion has spread from the stomach to the duodenum or esophagus, classification is made in terms of depth.
3. A tumor that advances into the gastrocolic ligament, into the hepatogastric ligament, or into the greater or lesser omentum, and is classified as T3, when there is no perforation of the visceral peritoneum.

N –Regional lymph nodes

NX: Regional lymph node metastasis cannot be evaluated
N0: No regional lymph node metastasis
N1: 1–2 regional foci of lymph node metastases
N2: 3–6 regional foci of lymph node metastases
N3: 7 or more regional foci of lymph node metastases
N3a: 7–15 regional foci of lymph node metastases
N3b: 16 or more regional foci of lymph node metastases

M –Distal metastasis
M0: No distal metastasis
M1: Distal metastasis present

Stage	T	N	M
**0**	Tis	N0	M0
**IA**	T1	N0	M0
**IB**	T2	N0	M0
	T1	N1	M0
**IIA**	T3	N0	M0
	T2	N1	M0
	T1	N2	M0
**IIB**	T4a	N0	M0
	T3	N1	M0
	T2	N2	M0
	T1	N3	M0
**IIIA**	T4a	N1	M0
	T3	N2	M0
	T2	N3	M0
**IIIB**	T4b	N0, N1	M0
	T4a	N2	M0
	T3	N3	M0
**IIIC**	T4a	N3	M0
	T4b	N2, N3	M0
**IV**	Any T	Any N	M1

3.4.3. Small intestines (including duodenum)

T –Primary tumor
TX: Primary tumor cannot be evaluated
T0: Primary tumor unidentified
Tis: Epithelial carcinoma
T1: Tumor invading mucosal lamina propria, muscularis mucosae, or submucosa
T1a: Tumor infiltrating the mucosal lamina propria or muscularis mucosae
T1b: Tumor infiltrating the submucosa
T2: Tumor infiltrating the muscularis propia
T3: Tumor invading the subserosa, or tumor invading within 2 cm of surrounding tissue of muscularis externa without peritoneal cover (mesenterium, retroperitoneum)*
*Surrounding tissue of muscularis externa without peritoneal cover refers to the mesenterium in the jejunum and ileum, and the retroperitoneum in the duodenum without serosa.
T4: Tumor penetrating the visceral peritoneum, or tumor directly invading another organ or tissue (invasion of other loops of the small intestine, invasion by 2 cm or more into the mesenterium and retroperitoneum, including invasion into the abdominal wall through the serosa; invasion to the pancreas only for duodenum)

N –Regional lymph nodes
NX: Regional lymph node metastasis cannot be evaluated
N0: No regional lymph node metastasis

N1 : 1–3 regional foci of lymph node metastases

N2 : 4 or more regional foci of lymph node metastases

M –Distal metastasis

M0 : No distal metastasis

M1 : Distal metastasis present

Stage	T	N	M
**0**	Tis	N0	M0
**I**	T1, T2	N0	M0
**IIA**	T3	N0	M0
**IIB**	T4	N0	M0
**IIIA**	Any T	N1	M0
**IIIB**	Any T	N2	M0
**IV**	Any T	Any N	M1

3.4.4. Appendix (partial revision of UICC-TNM 7th Edition)

T –Primary tumor

TX : Primary tumor cannot be evaluated

T0 : Primary tumor unidentified

Tis : Epithelial carcinoma: Tumor invading epithelium or lamina propia mucosae

T1 : Tumor invading the submucosa

T2 : Tumor invading the muscularis propia

T3 : Tumor invading the subserosa or mesoappendix

T4 : Tumor penetrating the visceral peritoneum, tumor including a peritoneal mucosal tumor in the lower right abdomen and/or tumor directly invading other organs or tissue

T4a : Tumor penetrating the visceral peritoneum, or peritoneal mucosal tumor in the lower right abdomen

T4b : Tumor directly invading other organs or tissues

N –Regional lymph nodes

NX : Regional lymph node metastasis cannot be evaluated

N0 : No regional lymph node metastasis

N1 : 1–3 regional foci of lymph node metastases

N2 : 4 or more foci of regional lymph node metastases

M –Distal metastasis

M0 : No distal metastasis

M1 : Distal metastasis present

M1a : Peritoneal metastasis beyond the lower right abdomen, including pseudomyxoma peritonei

M1b : Distal metastasis other than peritoneal metastasis

Stage	T	N	M
**0**	Tis	N0	M0
**I**	T1, T2	N0	M0
**IIA**	T3	N0	M0
**IIB**	T4a	N0	M0
**IIC**	T4b	N0	M0
**IIIA**	T1, T2	N1	M0
**IIIB**	T3, T4	N1	M0
**IIIC**	Any T	N2	M0
**IVA**	Any T	N0	M1a
**IVB**	Any T	N0	M1a
	Any T	N1, N2	M1a
**IVC**	Any T	Any N	M1b

3.4.5. Colon and rectum

T –Primary tumor

TX: Primary tumor cannot be evaluated

T0: Primary tumor unidentified

Tis: Carcinoma in situ: Tumor invading epithelium or lamina propia mucosae

T1: Tumor invading the submucosa

T2: Tumor invading the muscularis propia

T3: Tumor invading the subserosa, or tissue surrounding the colon or rectum without peritoneal covering

T4: Tumor penetrating the visceral peritoneum, and/or directly invading another organ or tissues

T4a: Tumor penetrating the visceral peritoneum

T4b: Tumor directly invading another organ or tissues

N –Regional lymph nodes

NX: Regional lymph node metastasis cannot be evaluate

N0: No regional lymph node metastasis

N1: 1–3 regional foci of lymph node metastases

N1a: 1 regional lymph node metastasis

N1b: 2–3 regional foci of lymph node metastases

N1c: Presence of tumor deposits, that is, satellite nodes in the subserosa or in the soft tissue surrounding colon or rectum without peritoneal covering, but with no regional lymph node metastasis

N2: 4 or more regional lymph node metastases

N2a: 4–6 regional foci of lymph node metastases

N2b: 7 or more regional foci of lymph node metastases

M –Distal metastasis

M0: No distal metastasis

M1: Distal metastasis present

M1a: Local metastasis to one organ (liver, lungs, ovaries, or lymph nodes other than regional lymph nodes)

M1b: 2 or more organs, or peritoneal metastasis

Stage	T	N	M
**0**	Tis	N0	M0
**I**	T1, T2	N0	M0
**II**	T3, T4	N0	M0
**IIA**	T3	N0	M0
**IIB**	T4a	N0	M0
**IIC**	T4b	N0	M0
**III**	Any T	N1, N2	M0
**IIIA**	T1, T2	N1	M0
	T1	N2a	M0
**IIIB**	T3, T4	N1	M0
	T2, T3	N2a	M0
	T1, T2	N2b	M0
**IIIC**	T4a	N2a	M0
	T3, T4a	N2b	M0
	T4b	N1, N2	M0
**IVA**	Any T	Any N	M1a
**IVB**	Any T	Any N	M1b

3.4.6. Applies to hepatic NEC (hepatic primary lesion or liver metastasis from unknown primary lesion)

T –Primary tumor

TX: Primary tumor cannot be evaluated

T0: Primary tumor unidentified

Tis : Carcinoma *in situ*

T1 : Isolated tumor without vascular invasion

T2a : Isolated tumor with vascular invasion

T2b : Multifocal tumor regardless of vascular invasion

T3 : Tumor penetrating visceral peritoneum or directly invading adjacent extrahepatic structures

T4 : Tumor with bile duct invasion (bile duct proliferative type)

N –Regional lymph nodes

NX : Regional lymph node metastasis cannot be evaluated

N0 : No regional lymph node metastasis

N1 : Regional lymph node metastasis present

M –Distal metastasis

M0 : No distal metastasis

M1 : Distal metastasis present

Stage	T	N	M
**I**	T1	N0	M0
**II**	T2	N0	M0
**III**	T3	N0	M0
**IVA**	T4	N0	M0
	Any T	N1	M0
**IVB**	Any T	Any N	M1

3.4.7. Gall bladder

T –Primary tumor

TX : Primary tumor cannot be evaluated

T0 : Primary tumor unidentified

Tis : Carcinoma *in situ*

T1 : Tumor invading mucosal lamina propria or muscularis externa

T1a : Tumor invading mucosal lamina propria

T1b : Tumor invading muscularis externa

T2 : Tumor invading the connective tissue around the muscularis externa, but shows no progression beyond the serosa or to the liver

T3 : Tumor perforating the serosa (visceral peritoneum), tumor directly advancing to the liver and/or an adjacent organ that is not the liver (stomach, duodenum, colon, pancreas, omentum, or extrahepatic bile duct)

T4 : Tumor invading the main trunk of the portal vein or the hepatic artery, or tumor advancing to two or more adjacent organs which are not the liver

N –Regional lymph nodes

NX : Regional lymph node metastasis cannot be evaluated

N0 : No regional lymph node metastasis

N1 : Regional lymph node metastasis present (cystic duct, common bile duct, proper hepatic artery, including lymph nodes along the portal vein)

M –Distal metastasis

M0 : No distal metastasis

M1 : Distal metastasis present

Stage	T	N	M
**0**	Tis	N0	M0
**I**	T1	N0	M0
**II**	T2	N0	M0
**IIIA**	T3	N0	M0
**IIIB**	T1, T2, T3	N1	M0
**IVA**	T4	Any N	M0

**IVB**	Any T	Any N	M1

3.4.8. Extrahepatic bile duct-hepatic portal region

T –Primary tumor

TX: Primary tumor cannot be evaluated

T0: Primary tumor unidentified

Tis: Carcinoma *in situ*

T1: Tumor localized to the bile duct which advances until muscularis externa or fibrous tissue

T2a: Tumor invading beyond the bile duct wall and into the surrounding adipose tissue

T2b: Tumor invading the adjacent liver parenchyma

T3: Tumor invading the branch of one side of portal vein or hepatic artery

T4: Tumor invades the main trunk of portal vein, and branches on both sides of the portal vein, proper hepatic artery or the secondary branches of the bile duct on both left and right side, or to the secondary bile duct branches on one side and portal vein or hepatic artery on the other side

N –Regional lymph nodes

NX: Regional lymph node metastasis cannot be evaluated

N0: No regional lymph node metastasis

N1: Presence of regional lymph node metastasis in the cystic duct, common bile duct, proper hepatic artery, and lymph nodes along portal vein

M –Distal metastasis

M0: No distal metastasis

M1: Distal metastasis present

Stage	T	N	M
**0**	Tis	N0	M0
**I**	T1	N0	M0
**II**	T2a, T2b	N0	M0
**IIIA**	T3	N0	M0
**IIIB**	T1,T2, T3	N1	M0
**IVA**	T4	N0, N1	M0
**IVB**	Any T	Any N	M1

3.4.9. Extrahepatic bile duct-distal

T –Primary tumor

TX: Primary tumor cannot be evaluated

T0: Primary tumor unidentified

Tis: Carcinoma *in situ*

T1: Tumor localized in the bile duct wall

T2: Tumor invading beyond the bile duct wall

T3: Tumor invading the gallbladder, liver, pancreas, duodenum, or other adjacent organs

T4: Tumor invading the celiac axis or superior mesenteric artery

N –Regional lymph nodes

NX: Regional lymph node metastasis cannot be evaluated

N0: No regional lymph node metastasis

N1: Regional lymph node metastasis present

M –Distal metastasis

M0: No distal metastasis

M1: Distal metastasis present

Stage	T	N	M
**0**	Tis	N0	M0
**IA**	T1	N0	M0
**IB**	T2	N0	M0

**IIA**	T3	N0	M0
**IIB**	T1, T2, T3	N1	M0
**III**	T4	Any N	M0
**IV**	Any T	Any N	M1

3.4.10. Ampulla of Vater

T –Primary tumor

TX: Primary tumor cannot be evaluated

T0: Primary tumor unidentified

Tis: Carcinoma *in situ*

T1: Tumor localized to the ampulla of Vater, or sphincter of Oddi

T2: Tumor invading the duodenal wall

T3: Tumor invading the pancreas

T4: Tumor invading the soft tissue around the pancreas or other adjacent organs

N –Regional lymph nodes

NX: Regional lymph node metastasis cannot be evaluated

N0: No regional lymph node metastasis

N1: Regional lymph node metastasis present

M –Distal metastasis

M0: No distal metastasis

M1: Distal metastasis present

Stage	T	N	M
**0**	Tis	N0	M0
**IA**	T1	N0	M0
**IB**	T2	N0	M0
**IIA**	T3	N0	M0
**IIB**	T1, T2, T3	N1	M0
**III**	T4	Any N	M0
**IV**	Any T	Any N	M1

3.4.11. Pancreas

T –Primary tumor

TX: Primary tumor cannot be evaluated

T0: Primary tumor unidentified

Tis: Carcinoma *in situ*

T1: Tumor localized within the pancreas, with a maximum size of < 2 cm

T2: Tumor localized within the pancreas, with a maximum size > cm

T3: Tumor advancing outside the pancreas, but with no invasion to the celiac axis or superior mesenteric artery

T4: Tumor invading the celiac axis or superior mesenteric artery

N –Regional lymph nodes

NX: Regional lymph node metastasis cannot be evaluated

N0: No regional lymph node metastasis

N1: Regional lymph node metastasis present

M –Distal metastasis

M0: No distal metastasis

M1: Distal metastasis present

Stage	T	N	M
**0**	Tis	N0	M0
**IA**	T1	N0	M0
**IB**	T2	N0	M0
**IIA**	T3	N0	M0

**IIB**	T1, T2, T3	N1	M0
**III**	T4	Any N	M0
**IV**	Any T	Any N	M1

3.5. Residual tumor (R) classification (UICC-TNM 7th Edition)

RX : Presence of residual tumor cannot be evaluated

R0 : No residual tumor

R1 : Presence of residual tumor by microscopic examination

R2 : Presence of residual tumor by macroscopic examination

3.6. Definition of non-resectable NEC

Unoperated cases for which surgery as a curative process was not indicated based on clinical findings, including image-based diagnosis, or operated cases that underwent resection (including experimental laparotomy) but ultimately ended as R2 resection are considered non-resectable.

The details by organs are shown below. One or more criterion must be fulfilled for each organ.

<Esopghagus>

- Diagnose as Stage IV (UICC 7th edition) based on clinical findings.

<Stomach>

- Diagnose as Stage IV (UICC 7th edition) based on clinical or surgical findings. However, this does not include situations where Stage IV diagnosis is provided based only on peritoneal lavage cytology (CY1).
- Bulky lymph node* metastasis is identified.

  *Bulky lymph node: Two or more lymph nodes with a major axis of 1.5 cm or more, that are present in contact with each other around the celiac artery, the common hepatic artery, the splenic artery, and the proper hepatic artery, or on the front surface of the superior mesenteric vein, where single or multiple large and small lymph nodes have formed an aggregate with the overall major axis length of 3.0 cm or more.

<Duodenum (excluding the ampulla of Vater), small intestines, appendix, and colon>

- Diagnosed as Stage IV (UICC 7th edition) based on the clinical findings.

<Extrahepatic bile duct, ampulla of Vater, gallbladder>

- Distant metastasis is identified
- Para-aortic lymph node metastasis is identified
- Bulky metastasis identified in the hepatoduodenal mesentery and lymph nodes surrounding the head of the pancreas
- Invasion in the proper hepatic artery, common hepatic artery, celiac artery, or the superior mesenteric artery identified
- Invasion in the hepatic artery branches on both the left and right side identified
- Extensive invasion or occlusion of the main trunk of portal vein, or invasion in the portal vein branches on both the left and right side identified
- Invasion in the blood vessels on the lobe on one side of the liver (portal vein or artery) or atrophy of lobe on one side of liver identified, and advancement into the bile duct on the other side is identified up to the secondary branch level
- Advancement into the bile ducts on both sides identified up to the secondary branch level

<Pancreas>

- Diagnosed as Stage III or Stage IV (UICC 7th edition) based on clinical findings.

＜Hepatic NEC (hepatic primary lesion or liver metastasis from unknown primary lesion)>

- Distal metastasis* other than intrahepatic metastasis identified.

  *Limited to organs that cannot be the primary lesion site (bone, lymph nodes below the diaphragm,

peritoneum, subcutaneous, muscle, and spleen) (see 3.8)

- Para-aortic lymph node metastasis is identified
- Bulky metastasis identified in the hepatoduodenal mesentery and lymph nodes surrounding the head of the pancreas
- Multiple masses identified in the liver.
- Invasion in one of the proper hepatic artery, common hepatic artery, celiac artery, or the superior mesenteric artery identified.
- Invasion to hepatic artery branches on both the left and right side identified
- Extensive invasion or occlusion of the main trunk of portal vein, or invasion in portal vein branches on both the left and right side identified
- Invasion in blood vessels on lobe on one side of the liver (portal vein or artery) or atrophy of lobe on one side of liver identified, and advancement into the bile duct on the other side is identified up to the secondary branch level
- Advancement into the bile ducts on both sides identified up to the secondary branch level

3.7. Definition of recurrent NEC

If surgery (R0 resection or R1 resection) was performed for gastrointestinal/hepatobiliary or pancreatic primary lesion, malignant tumor and the condition was diagnosed as NEC based on pathological samples taken from surgery, and were deemed as a clinical relapse thereafter.

If there are past histological samples from the primary lesion, no histological biopsy of the relapse lesion is necessary.

3.8. Definition of hepatic NEC (hepatic primary lesion or liver metastasis from an unknown primary lesion)

After a detailed investigation into the primary lesion by cervical-pelvic contrast CT, gastrointestinal endoscopy, FDG-PET, otolaryngology (head and neck examination), urology examination (for men only) and gynecological examination (for women only), if the tumor is in one of the following sites, the case is defined as hepatic primary lesion (or primary lesion unknown).

- Tumor is present only in the liver
- Tumor is present in an organ that cannot be a primary lesion site (bone, lymph nodes below the diaphragm, peritoneum, subcutaneous, muscle, and spleen), and in the liver.

4. Patient selection criteria

Patients that fulfill all of the following inclusion criteria and do not correspond to any of the exclusion criteria are eligible for enrollment.

4.1. Inclusion criteria (for enrollment)

1) Any of the following is applicable based on pathological diagnosis taking findings of immunohistochemistry into consideration (see 3.1. to 3.3.).

[1] Pathologically diagnosed as neuroendocrine carcinoma (NEC*1) in the resected sample.

[2] Containing pathologically confirmed component of neuroendocrine carcinoma (NEC*1) in the biopsy sample.

1: Based on WHO 2010 classification

2) Any of the following is applicable

[1] NEC arise in esophagus, stomach, duodenum, intestine, appendix, colon, rectum, gallbladder, intrahepatic bile duct, extrahepatic bile duct, ampulla of Vater, pancreas,

[2] Liver NEC (primary liver or liver metastasis of unknown primary) *2.

*2: The tumor is only in one of the following sites after a thorough examination of the primary site by contrast CT (from the neck to pelvic) and upper/lower gastrointestinal endoscopy, FDG-PET scan, otolaryngology (head and neck) examination, urology examination (male patients only), and gynecology examination (female patients only).

a. Liver only

b. Bone, lymph nodes below the diaphragm, peritoneum, subcutaneous, muscle, spleen, and liver

3) Unresectable (see 3.6) or recurrent cancer (see 3.7). It is not essential for a pathological confirmation of the metastatic lesion or recurrent site. Cases of esophageal NEC is ineligible if corresponding to any of the following.

[1] cT4.
[2] No distant metastasis rather than supraclavicular lymph node
[3] Stenosis indicated for palliative radiotherapy

4) No previous chemotherapy or radiotherapy for NEC. Pre- or post-operative chemotherapy except irinotecan or etoposide for NEC is allowed as long as it was completed at least 8 weeks prior to registration.
5) No previous chemotherapy using platinum agents for any malignancies.
6) No serious tumor-related complications.
Serious tumor-related complications include superior vena cava syndrome, inferior vena cava syndrome, pleural, ascites, or pericardial effusions that are large or uncontrollable (e.g., effusions that accumulate quickly after drainage or even after adhesive surgery), and brain metastases with neurological symptoms.
7) Aged 20 to 75 years old.
8) ECOG performance status of 0 or 1.
9) Sufficient oral intake
10) Measurable region is not required.
11) Adequate organ functions.

[1] WBC ≥3,000/ mm^3
[2] Neutrophils ≥ 1,500/ mm^3
[3] Hemoglobin ≥ 9.0 g/dL
[4] Platelets ≥ $10×10^4$/ mm^3
[5] Total bilirubin≤ 1.5 mg/dL※4
[6] AST(sGOT)≦100 IU/L※4（for hepatic NEC and liver metastasis, ≦150 IU/L）
[7] ALT(sGPT)≦100 IU/L※4（for hepatic NEC and liver metastasis, ≦150 IU/L）
※4：Presence or absence of biliary drainage is not relevant
[8] Serum creatinine≦ 1.3 mg/dL
[9] Creatinine clearance※5≥ 60 mL/min
※5： Creatinine clearance must have been estimated using the Cockcroft-Gault formula, and must be 60 mL/min or more. If the estimation is less than 60 mL/min, but the actual measurement is 60 mL/min or more, the patient can be deemed eligible.
Cockcroft-Gault formula
Male：Ccr ={(140－age) × body weight (kg)}/{72×serum creatinine (mg/dL)}
Female：Ccr = 0.85×{(140－age) × body weight (kg)}/{72×serum creatinine(mg/dL)}

12) Written informed consent.

4.2. Exclusion criteria

1) Synchronous or metachronous (within 5 years) malignancies except carcinoma in situ or intramucosal tumor curatively treated with local therapy.
2) Active infection requiring systemic therapy.
3) Fever of 38 degrees Celsius or higher.
4) Pregnant or lactating women, women of childbearing potential, or women within 28 days after delivery.
5) Psychiatric disease.
6) Patients requiring systemic steroids medication.
7) Interstitial pneumonia, pulmonary fibrosis.
8) Serious co-existing illness.
9) Unstable angina pectoris within 3 weeks, or with a history of myocardial infarction within 6 months.
10) Impossible to use both iodine and gadolinium due to being allergic to contrast agent.
11) Uncontrolled diabetes mellitus or routine administration of insulin.

5. Registration and randomization

5.1. Procedure of registration

Ensure that a patient to be registered meets all eligibility criteria and does not meet any of exclusion criteria and register the patient by using JCOG Web Entry System. JCOG Web System Personal Account and password are required for web registration. If unknown, contact JCOG Data Center.

Patient registration JCOG Web Entry System
URL:https://secure.jcog.jp/dc/
(Web registration can be used for 24 hours.)

Contact information for patient registration and JCOG Web Entry System
JCOG Data Center
TEL: 03-3542-3373
Weekdays 9:00-17:00 (not available in holidays, Saturdays and Sundays, New Year's holidays)
E-mail:JCOGdata@ml.jcog.jp

Contact information on Patients Selection Criteria
Study Coordinator Contact : Chigusa Morizane
Hepato-Biliary Pancreatic Group: Futomi Mori (Main Research Secretariat)
Department of Hepatobiliary and Pancreatic Medicine, National Cancer Center
TEL:03-3542-2511
FAX:03-3542-3815
E-mail:cmorizan@ncc.go.jp

Gastric Cancer Group: Yoshinori Machida
Shizuoka Cancer Center
〒411-8777 1007 Shimonagakubo, Nagaizumi-cho, Sunto-gun, Shizuoka Prefecture
TEL:+81-55-989-5222
FAX:+81-55-989-5631
E-mail：no.machida@scchr.jp

Esophageal Cancer Group: Ken Kato/Yushi Homma
Department of Gastrointestinal Oncology, National Cancer Center
TEL: 03-3542-2511
FAX: 03-3542-3815
E-mail: kenkato@ncc.go.jp / yohonma@ncc.go.jp

5.1.1. Precautions for patient registration

① Registration after initiation of protocol treatment is unacceptable.
② Registration is performed by accessing the URL in '5.1. Procedure of registration'.
③ Eligibility checks are performed on the screen of Registration Form, so it is not necessary to send a Registration Form to Data Center by mail or fax.
④ If input data are insufficient, registration is not accepted until all are met.
⑤ The registration number is issued after the confirmation of eligibility on the registration screen, then the registration is completed.
⑥ Once registered, patients will not be retracted (retracted from the database) unless there is withdrawal of consent, including refusal to use the data for research. For duplicate registration, the information at the initial registration (registration number, allocated arm) are used in any case.
⑦ When misregistration or duplicate registration is found, contact Data Center immediately.
⑧ Body surface area and drug dose calculations are institutional responsibilities, and the body surface area

and drug dose displayed on Web Entry System at registration are only for double-checking. Those should always be calculated and checked at the institution. When the body surface area calculation formula adopted in the hospital information system of the institution differs from calculation formula adopted by JCOG (Dubois formula: Body surface area (m^2) = Body weight (kg) $^{0.425}$ x Height (cm) $^{0.725}$ 84 ÷ 10,000), there can be a difference in the dose by the hospital information system of the institution and the dose by the calculation formula adopted by JCOG, but in that case, either dosage used is decided by the site investigator.

5.2. Randomization and allocation adjustment factor

During enrollment, the treatment arm for a patient is allocated randomly by the data center.

Randomization would use a method of minimization using the 1) institutions, 2) Primary lesion organ (gastrointestinal tract [esophagus, stomach, duodenum, small intestines, appendix, colon, and rectum] vs. hepatobiliary and pancreatic organ [hepatic NEC, gallbladder, extrahepatic bile duct, ampulla of Vater, or pancreas]) as the adjustment factors, so that there are no large discrepancies between them. Researchers at participating facilities would not be informed of the detailed procedures of randomization.

6. Treatment Plan and Treatment Modification Criteria

Unless patient safety is threatened, treatment and treatment modifications is done in compliance with the specifications in this chapter.

If it is considered that the protocol specification may cause medically dangerous situation of the patient, treatment modifications should be made according to the medical judgment of the investigators/sub-investigators. Such protocol deviation is considered to be "clinically relevant deviation" if considered medically appropriate (see 14.1.4.Protocol deviation/violation). Deviations that occur with intentions other than safety, such as increasing efficacy, are not considered clinically relevant deviations.

6.1. Protocol treatment

Protocol treatment is initiated within 7 days of enrollment.

If treatment initiation occurs after 8 days from registration for any reason, the reason should be documented on the Treatment Course Form. If it is determined that treatment cannot be initiated, describe the details in the Off-treatment Form as Protocol Treatment Termination.

When laboratory parameters worsen and eligibility criteria are no longer met by the start of treatment after registration, the investigator/sub-investigator is allowed to decide whether initiate or terminate protocol treatment at their own discretion.

「6.3. The course initiation criteria is not applied at treatment changes.

Drugs used

- Etoposide ※
- Cisplatin ※
- Irinotecan ※
- Entecavir, tenofovir disoproxil fumarate, tenofovir alafenamide fumarate

The use of generic drugs is not restricted.

※ The company that manufactures or distributes these drugs, or intends to manufacture or sell these drugs, requires conflicts of interest control in the Clinical Trials Act as a company involved in this study (see 13.8.).

6.1.1. Arm A: Etoposide plus cisplatin (EP) therapy

The following chemotherapy courses will be given once a week for 3 weeks and treatment will be continued until the patient meets the discontinuation criteria.

Drug	Dosage(mg/m^2)	Dosing regimen/Dosing time	Dose day
Etoposide	100	IV/60-120 min	Day 1、2、3
Cisplatin	80	IV/60-120 min	Day 1

1) Calculation of the dosage

① Body surface area is calculated by determining drug dose to the third decimal point.

② For both etoposide and cisplatin, the calculated dose is determined by truncating the decimal point. For drug doses, ±10% is the acceptable range.

③ Dose recalculation due to body weight change after treatment initiation is not performed.

2) Administration of anticancer drugs

Etoposide and cisplatin are given in any order.

Examples of administration in the package insert are described below.

(i) Before administration: Before administration of anticancer drugs, 1,000-2,000 mL of hydration is done to achieve adequate diuresis.

(ii) Etoposide: Etoposide is mixed with infusion solutions such as isotonic sodium chloride solution of 250 mL or more, and infused intravenously in about 60-120 minutes. DEHP (2-ethylhexyl) phthalate: di-(2-ethylhexyl) phthalate), which is a plasticizer, elutes from polyvinyl chloride infusion sets and catheters. Avoid the use of polyvinyl chloride infusion sets and catheters.

(iii) Cisplatin administration: Cisplatin is mixed with stock solution or 500 mL of saline (or glucose-saline) and given intravenously in 60-120 min. During the administration, caution should be exercised in ensuring urine volume, and diuretics such as mannitol and furosemide should be administered as

necessary.

(iv) After administration: After administration of cisplatin, 1,000-2,000 mL of hydration should be done so that adequate diuresis can be achieved.

In addition, short hydration administration at the discretion of each institution is permitted. (see Table 6.1.1. for treatment cases).

Table 6.1.1. Example of short hydration

	Dose	Time
5HT3 antagonist<br>Dexamethasone<br>Physiological saline	 9.9 mg<br>50 mL	 15<br>min
Etoposide<br>Physiological saline	100 mg/m^2<br>250 mL	60<br>min
No. 1 solution<br>Potassium chloride<br>Magnesium sulfate	500 mL<br>10 mEq<br>8 mEq	 60<br>min
20% mannitol	200 mL	30<br>min
Cisplatin<br>Physiological saline	80 mg/m^2<br>250 mL	60<br>min
No. 1 solution<br>Potassium chloride	500 mL<br>10 mEq	60<br>min

(Referred from Horinouchi H, et al., Japan Society of Clinical Oncology 2012

3) Precautions for treatment

JCOG9511, an upfront trial in small-cell lung cancer, suggested an association between treatment-related deaths and first-course neutropenia. Therefore, caution should be exercised when neutropenia is strongly observed from the first course.

6.1.2. Arm B: Irinotecan plus cisplatin (IP) therapy

One 4-week course of the following chemotherapy will be continued until the discontinuation criteria are met. However, if day 15 irinotecan is skipped, 3 weeks should be used as one course, and the next course should be started with day 22.

Drug	Dosage(mg/m^2)	Dosing regimen/Dosing time	Dose day
Irinotecan	60	IV/90 min	Day 1、8、15
Cisplatin	60	IV/60-120 min	Day 1

1) Calculation of the dosage

(i) Body surface area is determined by determining the dose of drug administered until the third decimal point.

(ii) The dose is calculated for both irinotecan and cisplatin by truncating the decimal point. For drug doses, ±10% is the acceptable range.

(iii) Dose recalculation due to body weight change after treatment initiation is not performed.

2) Administration of anticancer drugs

Irinotecan and cisplatin are given in any order.

Examples of administration in the package insert are described below.

(i) Before administration: Before administration of anticancer drugs, 1,000-2,000 mL of hydration is done to achieve adequate diuresis.

(ii) Irinotecan: Irinotecan is mixed with 500 mL or more of isotonic sodium chloride solution, glucose solution, or electrolyte maintenance solution and given by intravenous drip infusion at about 90 minutes.

(iii) Cisplatin administration: Cisplatin is mixed with stock solution or 500 mL of saline (or glucose-saline) and given intravenously in 60-120 min. During the administration, caution should be exercised in ensuring urine volume, and diuretics such as mannitol and furosemide should be administered as necessary.

(iv) After administration: After administration of cisplatin, 1,000-2,000 mL of hydration should be done so that adequate diuresis can be achieved.

In addition, short hydration administration at the discretion of each institution is permitted. (See Table 6.1.1.

3) Precautions for treatment

JCOG9511, an upfront trial in small-cell lung cancer, suggested an association between treatment-related deaths and first-course neutropenia. Therefore, caution should be exercised when neutropenia is strongly observed from the first course.

6.2. Protocol Treatment Termination/Completion Criteria

6.2.1. Definition of protocol treatment completion

Protocol treatment is continued in this study unless the protocol treatment discontinuation criteria are met, so no definition of protocol treatment completion is provided.

6.2.2. Criteria for termination of protocol treatment

Protocol treatment is terminated in any of the following cases:

1) Judged as protocol treatment is ineffective
   - When a definite tumor exacerbation is confirmed by imaging or clinically
     ※ Protocol treatment should not be discontinued if the clinical judgement of PD based on the assessment of response based on imaging indicates that continuation of protocol treatment is appropriate, and protocol treatment should be continued.
2) Protocol treatment cannot be continued due to adverse events
   ① If Grade 4 non-hematological toxicity is observed (with the exception of the following adverse events) 'hypernatremia', 'hyponatremia', 'hyperkalemia', 'hypokalemia', 'hyperglycemia', 'hypoglycemia', 'alkaline phosphatase increase', 'alanine aminotransferase increase', 'aspartate aminotransferase increase', 'blood bilirubin increase','serum amylase increase', 'lipase increase', 'GGT increase'.
   (*Adverse events other than "anaemia," "myeloid cytopenia," "lymphocyte count decreased," "neutrophil count decreased," "white blood cell count decreased," "platelet count decreased," "CD4 lymphopenia" in CTCAE v4 0)
   ② If the next course cannot be initiated beyond 21 days from the expected start date of the course due to an adverse event
   ③ When the criteria for terminating protocol treatment in the treatment modification criteria (6.3.) are met.
   ④ Adverse events other than the treatment modification criteria that the investigator/sub-investigator judges to require termination of protocol treatment
3) If the patient offers termination of protocol treatment for reasons not denied to be associated with the adverse event
   - This category should be used if an association with an adverse event cannot be ruled out.
4) When the patient offers termination of protocol treatment because of reasons for denial of association with adverse events
   - Patient refusal after enrollment and before initiation of protocol treatment
   - When the association with an adverse event can first be denied, such as the relocation of the person or household member during protocol treatment.
5) Death during protocol treatment
   - Death before deciding to terminate protocol treatment for other reasons
6) Palliative surgery for pathogenic adverse events or surgery for patients with complete response to chemotherapy (see Section 6.3.9).
7) In addition, exacerbations before the start of treatment after enrollment (protocol treatment could not be initiated due to rapid exacerbation), protocol violations were found, ineligibility was determined due to modifications in pathological diagnosis after enrollment, etc., treatment was changed, and it was judged that it was difficult to continue protocol treatment due to social reasons and safety management problems, etc.

The date of discontinuation of protocol treatment is defined as the date of death in 6.2.2.5), the date of surgery in 6.2.2.6), and otherwise, the date on which the treating physician judges that protocol treatment is discontinued.

In this study, non-NEC may be diagnosed by central pathological diagnosis during protocol treatment, because central pathological diagnosis is performed. If the patient is continuing on protocol treatment when the results of the central pathology diagnosis are reported to the registry, the attending physician and the institutional pathologist will review the results and take a clinically appropriate response. Protocol treatment is discontinued if discontinuation of protocol treatment is judged to be appropriate, and the reason for discontinuation is other.

6.3. Treatment modification criteria

The following terms shall be used for the treatment modification.

Delay: Delay the start of the course or administration of treatment from the planned date.

Termination: Discontinuation of a part of or all of the treatment without restarting.

Suspending: temporary interruptions or withdrawals that may be resumed if conditions are met

Skip: Do not administer one or more drugs and proceed to the next schedule.

Categories of infection (CTCAEv4.0) used in this study are as follows

Infection: CTCAEv4.0 infections and infestations

Bronchial infection; pulmonary infection; upper respiratory tract infection; mediastinal infection; pleural infection; catheter-related infection; Biliary tract infection; Gallbladder infection; urinary tract infection

6.3.1. Arm A (EP-therapy): Dose level

1) Etoposide

Drug	Dose level	Dosing schedule	Dose day
Etoposide	Level 0	100 mg/m^2 div	Day 1、2、3
	Level -1	80 mg/m^2 div	Day 1、2、3
	Level -2	60 mg/m^2 div	Day 1、2、3

2) Cisplatin

Drug	Dose level	Dosing schedule	Dose day
Cisplatin	Level 0	80 mg/m^2 div	Day 1
	Level -1	60 mg/m^2 div	Day 1
	Level -2	40 mg/m^2 div	Day 1

6.3.2. A arm (EP therapy): Course initiation criteria

- Initiate the course after confirming that all of the following "Course Initiation Criteria" are met on the scheduled start date of the course or the day before the plan start date of the course.
- If any one is not met, the initiation of the course is delayed.
- If the course cannot be initiated within 21 days of the expected start date of the course (if the start date of the previous course was day 1 and the course could not be initiated by day 42), discontinue the protocol treatment.
- The course initiation criteria is not applied at the start of the first course.

Course initiation criteria

① Neutrophil count Grade 0-1 (≧1500 per mm^3).

② Platelet count ≧10×10^4 /mm^3

③ AST≦100 IU/L (in the presence of hepatic metastases vs. hepatic NECs) was ≦200 IU/L).

④ ALT≦100 IU/L (≦200 IU/L for liver metastases vs liver NECs).

⑤ Total bilirubin <= 2.0 mg/dL

⑥ Serum creatinine <= 1.5 mg/dL

⑦ Fever Grade 0 (axillary temperature, no antipyretic)

⑧ Constipation, fatigue, phlebitis, oral mucositis, and infection ※1 are all Grade 2 or less.

Infected ※1: bronchial infection, pulmonary infection, upper respiratory tract infection, mediastinal infection, pleural infection, catheter-related infection, biliary tract infection, gallbladder infection, urinary tract infection

⑨ Anorexia, nausea, and emesis are all Grade 0-1.

⑩ Diarrhoeal Grade 0

6.3.3. Arm A (EP therapy): Dose reduction criteria

If any of the following toxicities are identified during the course, dose reduction should be performed in accordance with the dose reduction criteria (Table 6.3.3.) from the following course (no dose reduction in the course).

However, even if two or more items are met, the dose reduction for each drug is only one step. Re-escalation after dose reduction is not performed. Protocol treatment is terminated if the dose reduction criteria are met again after the dose reduction to Level-2.

Table 6.3.3. Arm A (EP therapy): Dose reduction criteria

Item	Etoposide	Cisplatin
Neutrophil count Grade 4 (<500 per mm^3).	Reduce the level by 1	No change
Platelet count Grade 4 (<$2.5\times10^4/mm^3$)	Reduce the level by 1	No change
1.5 <serum creatinine ≦2.0 mg/dL.	No change	Reduce the level by 1
Serum creatinine <= >2.0 mg/dL	Termination of protocol treatment	Termination of protocol treatment
Grade 3<br>Infected ※1	Reduce the level by 1	Reduce the level by 1
Grade 3<br>Assessment at onset of febrile.	Reduce the level by 1	Reduce the level by 1
Grade 2<br>Peripheral sensory neuropathy<br>Peripheral motor neuropathy,<br>Myalgia; arthralgia; tinnitus; hearing impairment	No change	Reduce the level by 1
Grade 3<br>Peripheral sensory neuropathy<br>Peripheral motor neuropathy,<br>Myalgia; arthralgia; tinnitus; hearing impairment	Termination of protocol treatment	Termination of protocol treatment
Non-hematologic toxicities of Grade 3 other than those listed above in ※2 that are causally related to EP-therapy (excluding hyponatremia, hypokalemia, hypomagnesemia, hypocalcemia, hyperglycemia, and weight loss)	Reduce the level by 1	Reduce the level by 1

※1:※1 of infection: bronchial infection, pulmonary infection, upper respiratory tract infection, mediastinal infection, pleural infection, catheter-related infection,
Biliary tract infection; Gallbladder infection; Urinary tract infection

※2:Causal relationship is judged as either of possible, probable, definite

6.3.4. Arm A (EP therapy): Within-course pause, skipping criteria

Following initiation of treatment with each course after the course initiation criteria are met, if any of the following adverse events are observed, day 2, day 3 etoposide will be suspended.

- Fever (axillary temperature) Grade 1-3
- Grade 3 of infection (bronchial infection, pulmonary infection, upper respiratory tract infection, mediastinal infection, pleural infection, catheter-related infection,
  Biliary tract infection, gallbladder infection, urinary tract infection)

Resting etoposide should be resumed after confirming that all initiation criteria are met until day 7. However, it should not be administered after day 8.

That is, if etoposide could not be administered by day 7, the remaining etoposide should be skipped.

If the above suspension and/or skip occur, the next course of etoposide should be started day 22 (after 3 weeks) counting from day 1 of the previous course if the ininiation criteria are met.

6.3.5. Arm B (IP therapy): Dose-level

1) Irinotecan

Drug	Dose level	Dosing schedule	Dose day
Irinotecan	Level 0	60 mg/m^2 div	Day 1、8、15
	Level -1	50 mg/m^2 div	Day 1、8、15
	Level -2	40 mg/m^2 div	Day 1、8、15

2) Cisplatin

Drug	Dose level	Dosing schedule	Dose day
Cisplatin	Level 0	60 mg/m^2 div	Day 1
	Level -1	50 mg/m^2 div	Day 1
	Level -2	40 mg/m^2 div	Day 1

6.3.6. Arm B (IP therapy): Course initiation criteria

- On the day of the initiation of the course or the day before the expected start of the course, start the course after confirming that all of the following "Course Initiation Criteria" are met.
- If any one is not met, the initiation of the course is delayed.
- If the course cannot be initiated within 21 days of the expected start date of the course (if the start date of the previous course was day 1 and the course could not be initiated by day 49), discontinue the protocol treatment.
- However, if day 15 irinotecan is skipped in the previous course, day 22 of the previous course is set as the scheduled start date of the next course regarded as one course per 3 weeks.
- The course initiation criteria is not applied at the start of the first course.

Course initiation criteria

① Neutrophil count Grade 0-1 (≧1500/mm^3).
② Platelet count ≧10×10^4 /mm^3
③ AST≦100 IU/L (≦200 IU/L for liver metastases versus liver NECs)).
④ ALT≦100 IU/L (≦200 IU/L for liver metastases vs liver NECs).
⑤ Total bilirubin <= 2.0 mg/dL
⑥ Serum creatinine <= 1.5 mg/dL
⑦ Fever Grade 0 (measured by axillary temperature, temperature <38°C without antipyretic use)
⑧ Constipation, fatigue, phlebitis, oral mucositis, and infection ※1 are all Grade 2 or less.
Infected ※1: bronchial infection, pulmonary infection, upper respiratory tract infection, mediastinal infection, pleural infection, catheter-related infection, biliary tract infection, gallbladder infection, urinary tract infection
⑨ Anorexia, nausea, and emesis are all Grade 0-1.
⑩ Diarrhoea Grade 0

6.3.7. Arm B (IP-therapy): Dosing criteria for day 8, day 15

After confirming that all of the following ①-③ are met, the second (day 8) or third (day 15) dose of irinotecan is administered. If day 8, day 15 dosing criteria are not met, skip day 8, day 15 dosing.

① All of the following are met with the most recent laboratory data on the scheduled day of administration or the day before the scheduled day of administration.
i) Neutrophil count Grade 0-2 (≧1000 per mm^3).
ii) Platelet count ≧10×10^4 /mm^3
iii) AST ≦ 100 IU/L (≦200 IU/L for liver metastases vs liver NECs).
iv) ALT ≦ 100 IU/L (≦200 IU/L in the presence of hepatic metastases versus hepatic NECs).
v) Total bilirubin <= 2.0 mg/dL
vi) Serum creatinine <= 2.0 mg/dL
② All of the following are met on the scheduled day of administration:
i) Fever Grade 0 (measured by axillary temperature, temperature <38°C without antipyretic use)

ii） Diarrhoeal Grade 0

③ Constipation, anorexia, nausea, emesis, fatigue, phlebitis, oral mucositis, and infection ※1 are all Grade 2 or less.

Infected ※1: bronchial infection, pulmonary infection, upper respiratory tract infection, mediastinal infection, pleural infection, catheter-related infection, biliary tract infection, gallbladder infection, urinary tract infection

6.3.8. Arm B (IP therapy): Dose reduction criteria

If any of the following toxicities are identified during the course, dose reduction should be performed in accordance with the dose reduction criteria (Table 6.3.8.) from the following course (no dose reduction in the course).

However, even if two or more items are met, the dose reduction for each drug is only one step. Re-escalation after dose reduction is not performed. Protocol treatment is terminated if the dose reduction criteria are met again after the dose reduction to Level-2.

Table 6.3.8. Arm B (IP therapy): Dose reduction criteria

Item	Irinotecan	Cisplatin
Neutrophil count Grade 4 (<500 per mm^3).	Reduce the level by 1	No change
Platelet count Grade 4 (< 2.5×10^4/mm^3)	Reduce the level by 1	No change
1.5 <serum creatinine ≦2.0 mg/dL.	No change	Reduce the level by 1
Serum creatinine <= >2.0 mg/dL	Termination of protocol treatment	Termination of protocol treatment
Grade 3<br>Infected ※1	Reduce the level by 1	Reduce the level by 1
Grade 3<br>Assessment at onset of febrile.	Reduce the level by 1	Reduce the level by 1
Grade2<br>Peripheral sensory neuropathy<br>Peripheral motor neuropathy,<br>Myalgia; arthralgia; tinnitus; hearing impairment	No change	Reduce the level by 1
Grade 3<br>Peripheral sensory neuropathy<br>Peripheral motor neuropathy,<br>Myalgia; arthralgia; tinnitus; hearing impairment	Termination of protocol treatment	Termination of protocol treatment
Non-hematologic toxicities of Grade 3 other than those listed above in ※2 that are causally related to IP-therapy (excluding hyponatremia, hypokalemia, hypomagnesemia, hypocalcemia, hyperglycemia, and weight loss)	Reduce the level by 1	Reduce the level by 1

※1:※1 of infection: bronchial infection, pulmonary infection, upper respiratory tract infection, mediastinal infection, pleural infection, catheter-related infection,

Biliary tract infection; Gallbladder infection; Urinary tract infection

※2:Causal relationship is judged as either of possible, probable, definite

6.3.9. Surgical of after end of chemotherapy

1) Surgery for Adverse Events Associated with Pathogenesis

Surgery may be performed if it is judged clinically desirable to perform surgery for newly appearing symptoms such as hemorrhage or stenosis. Protocol treatment is discontinued if surgery is performed, regardless of the content. In this case, the date of discontinuation of protocol treatment is the date of surgery.

2) Surgery for patients with complete response to chemotherapy

- When chemotherapy is highly effective, all metastases present at the time of enrollment on imaging studies disappear, and curative resection (R0 resection) is considered possible, resection including primary and metastatic disease may be performed.
- Surgical procedures are not specifically specified, but the Research Office will collect information on the details of the surgery performed individually in order to be reviewed by the research representative/research office.

- In all surgical cases, the group group conference shall confirm the validity of the judgment that surgery is indicated.
- When surgery is performed, protocol treatment is discontinued, regardless of whether curative resection was performed or not. In this case, the date of discontinuation of protocol treatment is the date of surgery.

6.3.10. Consultation on treatment modification

If there are any questions about treatment modification, contact "16.6. Study Coordinator".

Study Coordinator Contact : Chigusa Morizane

Hepato-Biliary Pancreatic Group: Futomi Mori (Main Research Secretariat)
Department of Hepatobiliary and Pancreatic Medicine, National Cancer Center
TEL:03-3542-2511
FAX:03-3542-3815
E-mail:cmorizan@ncc.go.jp

Gastric Cancer Group: Yoshinori Machida
Shizuoka Cancer Center
〒411-8777 1007 Shimonagakubo, Nagaizumi-cho, Sunto-gun, Shizuoka Prefecture
TEL:+81-55-989-5222
FAX:+81-55-989-5631
E-mail：no.machida@scchr.jp

Esophageal Cancer Group: Ken Kato/Yushi Homma
Department of Gastrointestinal Oncology, National Cancer Center
TEL:03-3542-2511
FAX:03-3542-3815
E-mail: kenkato@ncc.go.jp / yohonma@ncc.go.jp

6.4. Concomitant treatment and supportive care

6.4.1. Required concomitant treatment/supportive care

1) Testing and Supportive Care for HBsAg-Positive Cases.

In HBsAg-positive cases, steroids and chemotherapy can lead to rapid hepatitis B virus (HBV) expansion (reactivation: reactivation) and potentially fatal severe hepatitis. Therefore, the following tests and supportive care are performed based on the "Guidelines for the Treatment of Hepatitis B, Third Edition (Japanese Society of Hepatology)." It is advisable to consult a hepatologist at the time prior to initiation of a nucleic acid analogue (entecavir, tenofovir disoproxil fumarate, and tenofovir alafenamide fumarate).

①Testing prior to initiation of chemotherapy: HBV-DNA quantitation

HBV-DNA quantitative analysis should be performed at least once prior to initiation of chemotherapy.

HBV-DNA assays are performed by real-time PCRs.

HBeAg and HBe antibodies should also be measured in accordance with the Guidelines for the Treatment of Hepatitis B, Third Edition (Japanese Society of Hepatology).

②Dosing schedule for supportive care (nucleic acid analogues prophylaxis)

- **Drugs used :**
  - **Entecavir (Bristol-Myers: Baraclude Tablets 0.5 mg)**
  - **Tenofovir disoproxil fumarate (GlaxoSmithKline: Tenozet Tablets 300 mg)**
  - **Tenofovir alafenamide fumarate (Giliado: Vemuridi Tablets 25 mg)**

The following dosage regimen should be followed, starting at least 1 week before the start of chemotherapy (as soon as possible), and continuing for at least 12 months after the end of chemotherapy. However, fulminant hepatitis has been reported in HBsAg-positive patients with high viral load, even during NA prophylaxis, and it is desirable to reduce the viral load before starting immunosuppression/chemotherapy. Nucleic acid analogues may be discontinued after 12 months after completion of chemotherapy if conditions *1 and 2 for discontinuation of NAs are met. However, if the administration of a nucleic acid analogue is discontinued, consultation with a hepatologist is always obtained, and the administration is discontinued only if the hepatologist deems it appropriate.

※1 Requirements for discontinuation of nucleic acid analogues (entecavir, tenofovir disoproxil fumarate, tenofovir alafenamide fumarate): all of the following

1. The patient has been on NA for more than 2 years.
2. HBV-DNA assays are not sensitive to detect
3. Be negative for HBeAg

※2 Patient background requirements: All of the following

1. Both the treating physician and the patient have sufficiently understood that the hepatitis exacerbation is frequently observed after the nucleic acid analog withdrawal, and that there is a danger of becoming serious in the time.
2. Follow-up is possible after treatment cessation, and appropriate treatment is possible even if hepatitis recurs
3. It is judged that the liver fibrillation is slight and the hepatic reserve is good, and it is difficult to become serious even if the hepatitis is exacerbated.

(Adapted from Guidelines for the Treatment of Hepatitis B, the 3rd edition (Japanese Society of Hepatology))

Entecavir

- **Dosage regimen: Take this medicine on an empty stomach (2 hours after meals and more than 2 hours before the next meal).**
- **Dosage :**

Creatinine clearance (mL/min).	Dosage
50 or more	0.5 mg once daily
30 Beyond 50	0.5 mg once every 2 days
10 Beyond 30	0.5 mg once every 3 days
10 Less than	0.5 mg once every 7 days

- **Adverse drug reactions (incidence of all grades): nucleoside analog-naïve patients**

Diarrhea (6.0%), nausea (4.5%), constipation (3.7%), upper abdominal pain (3.0%), malaise (1.5%), nasopharyngitis (3.0%), muscle stiffness (2.2%), headache (3.0%), rash (incidence unknown), laboratory tests: elevated AST (3.7%), increased blood bilirubin (6.0%), increased blood amylase (10.4%), and increased lipase

(10.4%).Blood glucose increased (6.0%), blood lactate increased (6.7%), urine occult blood positive (4.5%), white blood cell count decreased (8.2%), and eosinophil count increased (0.7%). [Significant adverse reactions (incidence unknown)] Hepatitis worsened after completion of treatment, anaphylactoid symptoms, lactic acidosis, and severe hepatomegaly due to fatty liver

Tenofovir disoproxil fumarate

- **Dosage and administration: 300 mg is orally administered once daily.**
- **Dosage :**

Creatinine clearance (mL/min).	Dosage
50 or more	300 mg once daily
30 Beyond 50	300 mg once every 2 days
10 Beyond 30	300 mg once every 3 to 4 days
Hemodialysis	注) of 300 mg once every 7 days<br>Or 300 mg after completion of cumulative approximately 12 hours of dialysis<br>NOTE) After hemodialysis was performed. The pharmacokinetics in patients with creatinine clearance < 10 mL/min and not on hemodialysis have not been investigated.

- **Dosing Precautions:**

In the long-term administration of tenofovir disoproxil fumarate, attention should be paid to renal dysfunction, hypophosphatemia (including Fanconi syndrome), and decrease in bone mineral density. It is recommended that renal function and serum phosphorus should be measured regularly during tenofovir disoproxil fumarate administration.

- **Adverse reactions (incidence of all grades):**

Abnormal liver function tests (AST, ALT and γ-GTP increased, etc.) in 7 patients (4.9%), increased creatinine in 4 patients (2.8%), increased amylase, increased lipase and nausea in 3 patients each (2.1%), abdominal pain in 2 patients (1.4%), [major adverse reactions (incidence unknown)] renal dysfunction, renal failure, acute renal failure, proximal renal tubular dysfunction, Fanconi syndrome, severe renal dysfunction such as acute renal tubular necrosis, nephrogenic diabetes insipidus or nephritis, severe hepatomegaly due to lactic acidosis and fatty deposition (steatohepatitis), pancreatitis

Tenofovir alafenamide fumarate

- **Dosage and administration: 25 mg is orally administered once daily.**
- **Dosage :**

Creatinine clearance (mL/min).	Dosage
15 or more	25 mg once daily
Less than 15	Consider discontinuation

- **Dosing Precautions:**

In the long-term administration of tenofovir alafenamide fumarate, attention should be paid to renal dysfunction, hypophosphatemia (including Fanconi syndrome), and decrease in bone density. It is recommended that renal function and serum phosphorus should be measured periodically during tenofovir alafenamide fumarate administration.

- **Adverse reactions (incidence of all grades):**

Nausea and abdominal distension, headache, fatigue (≧1%), dyspepsia and diarrhea, flatus, upper abdominal pain, constipation, ALT increased, arthralgia, dizziness, insomnia, pruritus, rash (≧0.5% to <1%), [significant adverse reactions (incidence unknown)] renal dysfunction, renal failure, acute renal failure, proximal renal tubular dysfunction, severe renal impairment such as Fanconi syndrome, acute renal tubular necrosis, renal diabetes insipidus or nephritis, severe hepatomegaly due to lactic acidosis and fatty deposits (fatty liver)

③Monitoring: Quantitative analysis of HBV-DNA (during and after administration of nucleic acid analogues)

During nucleic acid analogue administration:

They are monitored every 4 weeks by both HBV-DNA quantitative analysis and liver function (ASTs, ALTs). However, if HBV-DNA level is less than 20 IU/mL (1.3 log IU/mL) during administration of nucleic acid analogues, it is acceptable to perform tests every 4 to 12 weeks.

After discontinuation of nucleic acid analogue administration:

Bearing in mind that reactivation may occur even after discontinuation of administration of a nucleic acid analogues, the patient should be consulted with a hepatologist, and the patient should be monitored for HBV-DNA determination and hepatic function (AST/ALT) every 4 weeks for at least 1 year after discontinuation of administration of a nucleic acid analogues. Subsequent follow-up will be decided after consulting a hepatologist.

2) Laboratory Teste and Supportive Care for HBsAg-Negative and HBc Antibody-Positive and/or HBs Antibody-Positive Cases.

HBV-DNA quantitative analysis should be performed at least once prior to initiation of chemotherapy. HBV-DNA assays are performed by real-time PCRs.

i) HBV-DNA ≧ 20 IU/mL (1. 3 log IU/mL) prior to initiation of chemotherapy

It has been clarified that HBV-DNA replicates persist at low levels in the livers and peripheral blood mononuclear cells when HBc or HBs are positive, even if they are HBs-Ag negative. It has been reported that reactivation of HBV and development of severe hepatitis are caused by the use of potent immunosuppressive agents even in such patients with previous infections.

If HBV-DNA ≧ 20 IU/mL (1.3 log IU/mL), the risk of HBV reactivation is judged to be as high as in HBsAg-positive cases, and prophylactic administration of nucleic acid analogues (entecavir or tenofovir disoproxil fumarate, tenofovir alafenamide fumarate) is administered. The following laboratory tests and supportive care are performed in accordance with the "Guideline for the Treatment of Hepatitis B, 3rd edition (Japanese Society of Hepatology)" with reference to the following for examination, dosage, and monitoring of supportive care before the start of chemotherapy.

However, these are not applicable if the HBs antibody alone is positive and the HBV vaccination history is obvious.

①Dosing schedule for supportive care (nucleic acid analogues prophylaxis)

According to the dosage and administration of nucleic acid analogues (entecavir, tenofovir disoproxil fumarate, tenofovir alafenamide fumarate) in "1) Test and supportive care for HBsAg positive cases". Same conditions for NA discontinuation.

② Monitoring: Quantitative analysis of HBV-DNA (during and after administration of nucleic acid analogues)

The intervals for monitoring during and after discontinuation of NA are in accordance with the provision of "1) Testing and supportive care for HBsAg-positive patients".

ii) HBV-DNA less than 20 IU/mL (1.3 log IU/mL) prior to initiation of chemotherapy

HBV-DNA quantitative analysis and hepatic function (AST, ALT) will be monitored, and nucleic acid analogues (entecavir, tenofovir disoproxil fumarate, tenofovir alafenamide fumarate) will be started when ≧20 IU/mL (1.3 log IU/mL) is achieved.

The Guidelines for the Treatment of Hepatitis B, the 3rd edition (Japanese Society of Hepatology) recommends monitoring with HBV-DNA quantitative analysis or high-sensitivity HBs antibodies during and after chemotherapy, depending on the risks of revitalization.

①Monitor:HBV-DNA quantitative analysis

HBV-DNA quantitative analysis should be performed every 4-12 weeks from the start of chemotherapy until at least 12 months after the end of chemotherapy.

If HBV-DNA level is more than 20 IU/mL (1.3 log IU/mL), administration of nucleic acid analogues should be started immediately in accordance with the Guidelines for the Treatment of Hepatitis B, the 3rd edition (Japanese Society of Hepatology). If HBsAg monitoring is positive for < 1 IU/mL (low positive), nucleic acid analogues should be administered after additional HBV DNA determinations of ≧ 20 IU/mL (1.3 log IU/mL).

It is advisable to consult a hepatologist at a time prior to initiation of NAs.

②Supportive care in reactivation

Nucleic acid analogues should be administered according to the supportive care described in i) When HBV-DNA prior to the initiation of chemotherapy is 20 IU/mL (1.3 log IU/mL) or more in 6.4.1.2). Once administration of nucleic acid analogues is started, nucleic acid analogues should be discontinued only if appropriate by the hepatologist.

6.4.2. Recommended/not recommended concomitant treatment/supportive care

The following concomitant treatment and supportive care are recommended. Even if it is not carried out, it is not regarded as protocol deviation,

1) Addressing Febrile Neutropenia.

① Assessment at onset of febrile neutropenia (FN).

a) If the count is less than 500 per mm^3, or is less than 1000 per mm^3 and is expected to be less than 500 per mm^3 within 48 hours, and if the axillary temperature is 37.5°C or higher (mouth temperature is 38.0°C or higher), a severity-risk assessment should be performed promptly and anti-virus treatment initiated as appropriate.

b) Severity risk assessment is performed with reference to Multinational Association for Supportive Care in Cancer(MASCC) scoring system ※1.

c) For initial evaluation, complete blood count including differential WHITE BLOOD CELL and platelet count, renal function (BUN, creatinine), electrolytes, liver function (transaminases, total bilirubin, alkaline phosphatase) tests, ≧2 sets of venous blood cultures prior to initiation of antimicrobials, one set of cultures from the catheter lumen and one set from the peripheral vein if a central venous catheter is in place, cultures of suspected infection areas, and chest x-rays if respiratory symptoms and signs are present.

d) When febrile neutropenia (FN) develops in a patient with a central venous catheter, blood cultures from the catheter and peripheral blood are performed, and catheter-related infections are considered if there is a time difference of more than 120 minutes in the positivity of both. If appropriate antimicrobial therapy does not improve after more than 72 hours, catheter should be removed. For infections caused by Staphylococcus aureus, Pseudomonas aeruginosa, Bacillus, fungi, and acid-fast bacilli, the catheter should be removed and appropriate antimicrobial therapy based on culture results should be performed.

② Antibiotic use

a) In high-risk patients, β-lactams with anti-Pseudomonas aeruginosa activity are administered intravenously as a single agent. However, other antimicrobials (aminoglycosides, fluoroquinolones, and/or vancomycin) may be added to a single agent in the initial regimen in patients with unstable or complicated conditions or when drug-resistant organisms are strongly suspected. Low-risk patients may be treated with antibiotics orally or intravenously, hospitalized, or with adequate evaluation, if appropriate, as outpatients.

b) Re-evaluation will be performed 3-4 days after initiation of antibiotics to investigate the continuation or change of antibiotics. In principle, antibiotics will be continued until the neutrophil count recovers to 500 cells per mm^3 or more.

c) Empiric antifungal therapy is recommended in high-risk patients who do not respond to 4-7 days of broad-spectrum antibiotics.

d) Fluoroquinolone prophylaxis is recommended in high-risk individuals with an expected neutrophil count of 100 cells per mm^3 or less lasting >7 days.

③ Therapeutic administration of G-CSF

For therapeutic administration of G-CSF during the incidence of FNs, refer to "6.4.5. 3) Therapeutic administration of G-CSF".

※1 Multinational Association for Supportive Care in Cancer (MASCC) scoring system.
(Adapted in part from the Practice Guideline for Febrile Neutropenia (FN) [Japanese Society of Medical Oncology]. *2)

Item	Score
Clinical manifestations (select one of the following * mark 3 sections)	
*No symptoms	5
*Mild symptoms	5
*Moderate symptoms	3
No decrease in blood pressure	5
No chronic obstructive pulmonary disease	4
Solid tumors, or hematopoietic tumors without a history of fungal infection	4
No dehydration symptoms	3
Patients with fever during outpatient management	3
Age < 60	2

The total score is up to 26 points. Twenty-one points or more are considered low risk and 20 points or less are considered high risk

※2 Since patients aged 20 years or older are subjects in this study, we deleted "Not applicable to patients younger than 16 years old" from the original edition of the Practice Guideline for Febrile Neutropenia (FN) [Japanese Society of Medical Oncology].

2) Nausea and vomiting

Regarding nausea and vomiting, antiemetics are positively administered according to Clinical Practice Guidelines for Antiemesis in Oncology [48], and fluid and electrolyte repletion are performed when oral intake is severely reduced.

3) Anorexia

If oral intake drops markedly, fluid and electrolyte supplements should be given as needed. Especially, in the cases with diabetes mellitus, the abnormality of blood sugar level and electrolyte is noticed.

4) Anemia, thrombocytopenia

If anaemia (haemoglobin < 8.0 g/dL) or thrombocytopenia (platelet count $< 2\times10^4/mm^3$) is observed, blood should be transfused as appropriate at the discretion of the treating physician.

5) Diarrhea

Severe diarrhoea occasionally occurs with arm B (IP therapy arm) and is extremely dangerous when complicated by febrile neutropenia. Patients should be fully informed about toxicities and their management, and should be instructed to measure body temperature, especially when neutrophils counts are most decreasing, and to contact a physician or nurse immediately during fever and diarrhea. Nonsteroidal anti-inflammatory drugs may not cause fever to become overt, so unnecessary anti-inflammatory drugs are not given.

If irinotecan-induced diarrhea occurs, the following supportive measures are recommended:

High-dose loperamide hydrochloride therapy

① Loperamide hydrochloride was started after signs of diarrhea were observed.
② Initial dose of 4 mg followed by 2 mg/2 hours (4 mg/4 hours at night)
③ It is administered until watery stool does not appear for more than 12 hours.
④ Doses should not be given for more than 48 hours.

6) Precautions on the day of cisplatin administration

Aminoglycoside antibiotics, vancomycin, and nonsteroidal anti-inflammatory drugs are not administered on the day of cisplatin administration or are used with caution.

6.4.3. Acceptable concomitant treatment and supportive care

The following concomitant treatment and supportive care may be used as needed.

Concomitant use of drugs for the treatment of complications such as hypertension and diabetes mellitus and symptomatic drugs such as morphine may be performed, but this drug should be administered with caution when furosemide, piretanide and phenytoin are used. Oral antibiotics for febrile neutropenia prophylaxis may be given at the discretion of the investigator/sub-investigator. Bisphosphonate denosumab may be used in combination for bone metastases.

6.4.4. Unacceptable concomitant treatment and supportive care

None of the following treatments will be given during protocol treatment:

① Administration of anticancer drugs other than protocol treatment

② Radiation therapy

6.4.5. Granulocyte colony-stimulating factor (granulocyte-colony stimulating factor:G-CSF

※This study permits the use of G-CSF biogenerics (biosimilars).

1)※ of primary prophylaxis with G-CSF

※Primary prophylaxis: G-CSF administration before developing febrile neutropenia or prolonged neutropenia to prevent them during anticancer therapy.

Primary prophylaxis with G-CSF was not recommended at the beginning of the study in this study. However, since febrile neutropenia in group A was 21.4% in the late 2016 periodic monitoring report and 15.8% in the early 2017 periodic monitoring report (18.4% when the time of occurrence was April 2017 and SAE reports not reflected in the early 2017 periodic monitoring report were included), it was decided that the patient would meet at least the recommended grade B or higher (almost A) according to G-CSF Appropriate Use Guideline 2013 and JSMO Febrile Neutropenia (FN) Practice Guideline. Primary prophylactic administration of G-CSF was recommended in group A. However, it is not considered a protocol deviation even if it is not administered. Since it is practical to administer pegfilgrastim from the viewpoint of convenience, when the primary preventive administration of G-CSF is carried out in the actual medical field, the administration example of pegfilgrastim is shown below.

(Administration cases)

Group A: 3.6 mg of pegfilgrastim (genetical recombination) will be injected subcutaneously (once per chemotherapy course) between day 4(day 3's completion of etoposide treatment and day 7 at least 24 hours after the completion of etoposide treatment.

Since the safety of pegfilgrastim administered 14 days prior to the initiation of cancer chemotherapy and within 24 hours after completion of administration has not been established, the primary prophylactic administration of pegfilgrastim is not performed in group B where day 8 is administered the drug.

Table 6.4.5. Primary prophylactic administration of G-CSF should be performed according to the approved dosage and administration shown in the table below.

Drug	・・ Pegfilgrastim (arm A only)<br>・・ Filgrastim<br>・・ Naltograstim<br>・・ Lenograstim
Time of initiation	Twenty-four hours after completion of chemotherapy
Dosage Dosing regimen	・Pegfilgrastim (genetical recombination) at a dose of 3.6 mg subcutaneously once per chemotherapy course (group A only)<br>・Filgrastim: 50 µg per m^2 SC once daily or 100 µg per m^2 IV once daily<br>・Naltograstim: 1 µg/kg SC once daily or 2µg/kg IV once daily<br>・Lenograstim: 2 µg/kg SC once daily or 5µg/kg IV once daily
Timing of discontinuation (other than pegfilgrastim)	・If the neutrophil count reaches 5000 per mm^3 or more after the course, administration should be discontinued.<br>・If the neutrophil count recovers to ≧2000 cells per mm^3, there are no suspected infections, and the patient's safety is judged to be ensured by the responsiveness to the drug used, the drug should be discontinued or reduced.

2)Secondary prophylactic※ of G-CSF

※ Secondary prophylaxis: G-CSF prophylactic administration after once occurrence of febrile neutropenia or prolonged neutropenia to prevent febrile neutropenia or prolonged neutropenia from occurring again during anticancer therapy.

If febrile neutropenia occurs in the previous course, secondary prophylaxis with G-CSF after the subsequent course is recommended, even if dose reduction or schedule modification or antimicrobial therapy is considered to be associated with a lower risk of febrile neutropenia (group A). However, it is not considered a protocol deviation even if it is not administered. As it is practical to administer pegfilgrastim in terms of convenience when secondary prophylactic administration of G-CSF is carried out in real medical practice, the following examples of administration of pegfilgrastim are shown.

(Administration cases)

Group A: 3.6 mg of pegfilgrastim (genetical recombination) is injected subcutaneously (once per course of chemotherapy) between the time of completion of etoposide treatment of day 4(day 3 and day 7 of at least 24 hours.

However, the safety of pegfilgrastim administered 14 days prior to the start of cancer chemotherapy and 24 hours after the end of treatment has not been established, so secondary prophylactic pegfilgrastim administration is not performed in group B, where day 8 is given the drug.

Secondary prophylactic administration of G-CSF should be performed according to the approved dosage and administration shown in the table below.

Drug	・・ Pegfilgrastim (arm A only)<br>・・ Filgrastim<br>・・ Naltograstim<br>・・ Lenograstim
Time of initiation	・・ Pegfilgrastim (arm A only)<br>Twenty-four hours after completion of chemotherapy<br>・・ Filgrastim, nartograstim, and lenograstim<br>When neutrophil counts <1000 per mm^3 are observed
Dosage Dosing regimen	・Pegfilgrastim (genetical recombination) at a dose of 3.6 mg subcutaneously once per chemotherapy course (group A only)<br>・Filgrastim: 50 µg per m^2 SC once daily or 100 µg per m^2 IV once daily<br>・Naltograstim: 1 µg/kg SC once daily or 2µg/kg IV once daily<br>・Lenograstim: 2 µg/kg SC once daily or 5µg/kg IV once daily
Timing of discontinuation (other than pegfilgrastim)	・If the neutrophil count reaches 5000 per mm^3 or more after the course, administration should be discontinued.<br>・If the neutrophil count recovers to ≧2000 cells per mm^3, there are no suspected infections, and the patient's safety is judged to be ensured by the responsiveness to the drug used, the drug should be discontinued or reduced.

3) Therapeutic administration of G-CSF

Therapeutic administration of G-CSF should be performed according to the approved dosage and administration shown in the table below.

Time of initiation	・When the neutrophil count is less than 1000 per mm^3 and fever (in principle, > 38.0°C) is observed<br>・When neutrophil counts <500 per mm^3 are observed
Dosage Dosing regimen	・Filgrastim: 50 µg per m^2 SC once daily or 100 µg per m^2 IV once daily<br>・Naltograstim: 1 µg/kg SC once daily or 2µg/kg IV once daily<br>・Lenograstim: 2 µg/kg SC once daily or 5µg/kg IV once daily
Timing of discontinuation	・If the neutrophil count reaches 5000 per mm^3 or more after the course, administration should be discontinued.<br>・If the neutrophil count recovers to ≧2000 cells per mm^3, there are no suspected infections, and the patient's safety is judged to be ensured by the responsiveness to the drug used, the drug should be discontinued or reduced.

6.5. Post-study treatment

Treatment after discontinuation of protocol treatment and treatment after progression or recurrence after completion are not specified.

Treatment (cross over) with drugs included in treatment regimens in the unassigned groups may be used, but if the total dose of cisplatin is greater than 500 mg/m^2, careful attention should be given to accumulating toxicities such as peripheral sensory/motor neuropathy, hearing loss, and renal impairment. Patients should be carefully monitored with adequate risk explanation only if the benefits are apparently outweighed by the risks, such as those with persistent sensitivity to cisplatin and mild cumulative toxicity.

If primary analysis or interim analysis reveals the primary conclusions of the trial, the results of the study will be explained to patients enrolled in this study as needed, and the best treatment will be provided, taking into account the course of treatment of individual patients.

In addition, if the protocol treatment discontinuation criteria apply but clinically "protocol treatment continuation" is judged to be appropriate, consult the research office through the institutional research director or institutional coordinator rather than at the physician level as a general rule (except when time is not allowed). In agreement between the Research Secretariat and the Investigator/Institution Coordinator, decide whether to treat as a → after discontinuation of protocol treatment or to deviate and continue protocol treatment. The details of the consultation

with the Research Secretariat and the decision-making process should be provided in the comment column for the patient's end-of-treatment report and progress record. If continuing the protocol treatment with protocol deviation occurs frequently, the Study Coordinator should consider revising protocol treatment termination criteria using group meetings and group mailing lists, because it is considered that the protocol treatment termination criteria is clinically inappropriate in such situation.

7. Anticipated Adverse Events

7.1. Anticipated adverse reactions

Anticipated adverse reactions in this study are as follows:

7.1.1. Anticipated Adverse Drug Reactions with Drugs

Adverse drug reactions anticipated with protocol treatments and drugs used in protocol-specified tests are referred to the latest version of the drug package insert.

7.1.2. Anticipated adverse reactions in the standard treatment arm (arm A)

Table 7.1.2. Adverse events in the EP-therapy group at JCOG9511 (excerpt from the final analysis report)

Examination Items	Grade 0	Grade 1	Grade 2	Grade 3	Grade 4	Grade 3,4(%)	Grade4(%)	Total
White blood cells	2	5	30	35	5	51.9%	6.5%	77
Neutrophils	1	0	5	21	50	92.2%	64.9%	77
Hemoglobin	2	9	43	23	-	29.9%	-	77
Platelet	31	19	13	14	0	18.2%	0%	77
Total bilirubin	57	-	20	0	0	0%	0%	77
GOT	49	24	2	1	1	2.6%	1.3%	77
GPT	40	28	6	2	1	3.9%	1.3%	77
Creatinine	56	16	5	0	0	0%	0%	77
Oxygen tension	13	27	9	2	1	5.8%	1.9%	52
Nausea and vomiting	13	36	23	5	-	6.5%	-	77
Diarrhea	64	8	5	0	0	0%	0%	77
Oral cavity (stomatitis)	68	6	2	1	0	1.3%	0%	77
Infection	42	23	9	1	2	3.9%	2.6%	77
Hair loss (hair)	9	46	19	-	-	-	-	74
Fever (uninfected)	45	14	16	2	0	2.6%	0%	77
Perception (peripheral nerve)	66	10	1	0	-	0%	-	77
Rash	74	2	1	0	0	0%	0%	77

※: Use JCOG Toxicity Criteria.

7.1.3. Anticipated adverse reactions in the study treatment arm (Arm B)

Table 7.1.3. Adverse Events in the IP-Therapy Group in a JCOG9511 (Extracted from the Final Analysis Report)

Examination Items	Grade 0	Grade 1	Grade 2	Grade 3	Grade 4	Grade 3,4(%)	Grade 4(%)	Total
White blood cells	1	16	38	17	3	26.7%	4.0%	75
Neutrophils	1	8	17	30	19	65.3%	25.3%	75
Hemoglobin	7	10	38	20	-	26.7%	-	75
Platelet	56	6	9	1	3	5.3%	4.0%	75
Total bilirubin	59	-	16	0	0	0%	0%	75
GOT	40	30	5	0	0	0%	0%	75
GPT	35	30	7	3	0	4.0%	0%	75
Creatinine	56	15	4	0	0	0%	0%	75
Oxygen tension	13	20	5	1	1	5.0%	2.5	40
Nausea and vomiting	11	26	28	10	-	13.3%	-	75
Diarrhea	23	19	21	8	4	16.0%	5.3%	75
Oral cavity (stomatitis)	66	9	0	0	0	0%	0%	75

Infection	45	17	9	3	1	5.3%	1.3%	75
Hair loss (hair)	17	44	13	-	-	-	-	74
Fever (uninfected)	45	12	17	1	0	1.3%	0%	75
Perception (peripheral nerve)	71	4	0	0	-	0%	-	75
Rash	70	5	0	0	0	0%	0%	75

※: Use JCOG Toxicity Criteria.

Table 7.1.3. Adverse events in the IP-therapy group in b JCOG0509 (abstracted from the main analysis report)

Examination Items	Grade 0	Grade 1	Grade 2	Grade 3	Grade 4	Grade 3,4(%)	Grade 4(%)	Total
White blood cells	16	36	58	29	3	22.5	2.1	142
Neutrophils	6	10	43	51	32	58.5	22.5	142
Hemoglobin	20	24	65	24	9	23.2	6.3	142
Platelet	125	6	8	2	1	2.1	0.7	142
Total bilirubin	116	15	10	1	0	0.7	0	142
GOT	98	37	5	2	0	1.4	0	142
GPT	80	56	4	2	0	1.4	0	142
Creatinine	97	37	8	0	0	0	0	142
Anorexia	22	67	33	19	1	14.1	0.7	142
Nausea	30	67	36	9	0	6.3	0	142
Vomiting	89	38	10	5	0	3.5	0	142
Diarrhea	52	51	28	11	0	7.7	0	142
Mucositis (examination findings)-oral cavity	126	15	0	0	1	0.7	0.7	142
Hair loss	64	66	12	-	-	-	-	142
Onset of febrile neutropenia	127	-	-	14	1	10.6	0.7	142
Infection with Grade3-4 neutropenia -Bronchus	142	-	0	0	0	0	0	142
-Lung (pneumonia)	137	-	0	4	1	3.5	0.7	142
-Pharynx	142	-	0	0	0	0	0	142
-Upper respiratory tract-unclassifiable	141	-	1	0	0	0	0	142
-Bladder	142	-	0	0	0	0	0	142
-Kidney	142	-	0	0	0	0	0	142
-Urinary tract-subclassification impossible	142	-	0	0	0	0	0	142
Neuropathy: Sensory	127	13	2	0	0	0	0	142

Table 7.1.3.c Adverse events from a pilot trial of postoperative adjuvant chemotherapy with irinotecan plus cisplatin for high-grade neuroendocrine lung cancer (excerpt)

Examination Items	Grade 2	Grade 3	Grade 4	Grade 3,4(%)	Grade 4(%)	Total
White blood cells	17	7	0	17.5%	0%	40
Neutrophils	12	15	4	47.5%	10.0%	40
Hemoglobin	14	6	4	25.0%	10.0%	40
Platelet	2	0	0	0%	0%	40
Total bilirubin	0	0	0	0%	0%	40
GOT	0	0	0	0%	0%	40
GPT	1	0	0	0%	0%	40
Creatinine	0	0	0	0%	0%	40
Hyponatremia	0	5	0	12.5%	0%	40
Endotoxemia	3	1	0	2.5%	0%	40
Hypokalemia	0	4	0	10.0%	0%	40
Nausea	8	4	-	10.0%	-	40
Vomiting	4	2	0	5.0%	0%	40
Anorexia	2	0	-	0%	0%	40

Diarrhea	11	2	0	5.0%	0%	40
Fatigue	10	5	-	12.5%	0%	40
Infection	2	0	0	0%	0%	40

7.2. Anticipated Adverse Events Due to Pathogenesis

1) Esophageal primary

Esophageal bleeding; Esophageal pain; Esophageal stenosis; Esophageal obstruction; Esophageal perforation; Esophageal ulcer; Esophageal fistula; Hoarseness; Pharyngolaryngeal dysesthesia; Hypercalcemia of advanced disease; Tracheal stenosis; Tracheal obstruction; Esophageal anastomotic leakage; Recurrent laryngeal nerve palsy

2) Gastric primary

Gastric bleeding, upper gastrointestinal bleeding, gastric pain, gastrointestinal pain, back pain, nausea, vomiting, dyspepsia, gastroparesis, abdominal fullness, gastric stenosis, gastric obstruction, gastric perforation, duodenal bleeding, duodenal fistula, duodenal perforation, duodenal perforation, duodenal stenosis, gastrostomy, gastrointestinal fistula, gastrointestinal fistula, ileus, gastric anastomotic leak, gastrointestinal anastomotic leak

3) Small intestin/Colon and rectum

Bleeding from tumor; Ileus; Duodenal bleeding; Duodenal obstruction; Duodenal perforation; Duodenal stenosis; Small bowel obstruction; Small bowel stenosis; Small bowel ulcer; Small bowel perforation; Small bowel fistula; Vaginal fistula; Colonic fistula; Colonic stenosis; Colonic obstruction; Colonic perforation; Rectal stenosis; Rectal obstruction; Rectal fistula; Rectal perforation; Rectal fistula; Jejunal perforation; Jejunal perforation; Ileostomy; Ileovesical fistula; Intestinal fistula; Anal pain; Small bowel anastomotic leakage; Large bowel anastomotic leakage; Rectal anastomotic leakage; Pelvic infection

4) Pancreas, biliary tract, and liver

Body weight loss; Fever; Pancreatitis; Cholecystitis; Cholecystic obstruction; Gallbladder pain; Liver failure; Biliary tract infection; Hemorrhage from biliary tract; Duodenal hemorrhage; Duodenal fistula; Duodenal obstruction; Duodenal perforation; Duodenal stricture; Anastomotic ulcer; Biliary anastomosis leak; Pancreatic anastomosis leak; Anastomotic hemorrhage; Anastomotic stricture; Hemorrhage from tumor; Hemorrhage from portal vein stenosis/obstruction; Symptoms associated with portal vein stenosis/obstruction (Portal hypertension, Hepatic failure; Esophageal variceal hemorrhage; Gastritis; Ascites); Back pain; Abdominal pain

5) Anticipated Adverse Events Due to Metastasis

Liver failure, hepatic pain, fever, hepatic infection, respiratory failure, pulmonary infection, superior vena cava syndrome, abdominal distention, abdominal distention, ileus, esophageal obstruction, reverse smoking, gastric obstruction, duodenal obstruction, small bowel obstruction, colonic obstruction, rectal obstruction, rectal stenosis, anal bleeding, anorectal infection, nausea, diarrhea, constipation, ureteral obstruction, and urinary retention,urethral infection, urinary tract infection, bile duct obstruction, biliary obstruction, cholecystitis, biliary hemorrhage, gallbladder pain, pancreatitis, pain, narrowing of luminal organs near metastatic sites (tracheal/gastrointestinal tract, etc.), tracheal obstruction, bronchial stenosis, penetration with adjacent organs, perforation, hoarseness, pleural effusion, chest wall pain, pleural pain, atelectasis, hypercalcemia, disseminated intravascular coagulation, fracture, ataxia, cerebrovascular ischemia, intracranial hemorrhage,headache, dizziness, decreased level of consciousness, aphasia, seizures, spasticity

6) Paraneoplastic syndrome, etc.

(due to incompatible secretory syndrome) hyponatremia, (due to ectopic ACTH syndrome) personality changes, hypertension, hypokalemia, hyperglycemia, Lambert-Eaton myasthenic syndrome, subacute cerebellar degeneration associated with autoantibody production (including ataxia, dysarthria, and nystagmus of limbs), paraneoplastic encephalomyelitis, sensory neuropathy (including dementia, cranial nerve symptoms, dizziness, ataxia, autonomic ataxia, transverse paralysis, and sensory disturbance), thromboembolism, (due to gastrointestinal stenosis) aspiration, anaemia, tumor pain, acute renal failure, myositis, and pulmonary fibrosis

Complications associated with etiolation and gastrointestinal stent insertion are shown below.

- Percutaneous transhepatic cholangiodrainage (PTCD) procedures, including internal-external fistula tube placement:

  Pancreatitis, bile duct stricture, cholecystitis, gallbladder obstruction, gallbladder pain, liver failure, biliary

tract infection, liver infection, cholecystitis, septicemia, hemorrhage, PTCD tubing obstruction/deviation, peritoneal infection, pneumothorax, pleural pain, pleural effusion, intrapleural hemorrhage

- Biliary stenting:
  Pancreatitis, biliary stricture, cholecystitis, gallbladder obstruction, gallbladder pain, liver failure, biliary tract infection, liver infection, cholecystitis, sepsis, hemorrhage, peritoneum infection, pneumothorax, pleural pain, pleural effusion, intrapleural hemorrhage (in case of percutaneous placement), duodenal perforation, pneumonitis (in case of transendoscopic placement), stent obstruction/deviation, duodenal ulcer, and duodenal hemorrhage
- Choledochojejunostomy:
  Biliary tract infection; Pancreatitis; Cholecystitis; Liver infection; Sepsis
- Gastrointestinal stent insertion:
  Bleeding, perforation, pain, stent deviation, stent obstruction, ulceration, fever, sepsis, infection, diarrhea, constipation, urgency (colorectal), thyroid injury (esophagus), jugular arteriovenous injury (esophagus), and mediastinal abscess (esophagus).

7.3. Evaluation of Adverse Events/Reactions

The Common Terminology Criteria for Adverse Events v4.0 Japanese Translated JCOG Version (Japanese translation of NCI-Common Terminology Criteria for Adverse Events v4.0 (CTCAE v4.0)) (CTCAE v4.0-JCOG) will be used to assess adverse events/reactions. For CTCAE v4 0-JCOG in which Grade is defined by laboratory reference values at the institutional reference value, the "JCOG sharing reference range" will be used instead of the institutional reference value at each medical institution. For more information on JCOG sharing reference ranges, see JCOG website (see http://www.jcog.jp/doctor/tool/kijun.html)).

7.3.1. Grading of s adverse events

In grading of adverse events, each grading is closest to the definitions of Grade 0 4 (nearest match). Grading to a higher Grade when the definition of more than one Grade is comparable and when it is difficult to decide on either (highest grade).

Grading should also be given to Grade if specific actions are described, due to their clinical need. For example, patients may refuse oxygen inhalation or chest drainage, even when the patient's pleural effusion is increasing and oxygen inhalation or chest drainage is indicated. In such cases, grading is based on the medical judgment of what should have been done (what should be done) rather than on whether the treatment was actually given (what was actually done).

In the event of treatment-related deaths, original NCI-CTCAE states that the causative adverse event should be Grade 5, but the outcome of the serious adverse event is reported in the SAE report and reviewed in detail. Therefore, Grade 5 of the institutional physician's judgment is not likely to be changed, and whether or not the serious adverse event will result in death will be significantly affected by other factors than the event is not appropriate, so it is not appropriate to compare the frequency of the adverse event by Grade (%Grade 4 and%Grade 5, respectively) between treatment groups or between studies. Because of the poor significance of distinguishing between Grade 4 and Grade 5 in the tabulation, Grade 4 is not considered "Grade 5" in the recording form of this study. A discussion of the causal relationship between adverse events observed in treatment-related deaths and deaths should be included in the "Situation at Death" section of the treatment completion report form and follow-up form, and an urgent report should be made. For the adverse event items specified in "8.2. Testing and Assessment during Treatment" and "8.3. Testing and Endpoints after Treatment Completion" that are determined to be Grade 5 in the post-hoc review including the emergency report, Grade and the date of the first occurrence of the event should be included in the relevant record form (Treatment Course Record Form). If Grade 3 or greater is observed for any other adverse event, or if Grade 3/2/1 adverse event and treatment requires at least 24 hours of hospital stay or prolongation of hospital stay (see 10.1.1.3.)), the AE and Grade and the date of first occurrence should be included in the free form of the treatment course record.

Any Grade on the record form should be recorded in the medical record. Confirmed during site visit audit.

7.3.2. Determination of the causal relationship between adverse events and treatment

In determining the causal relationship between adverse events and treatment, patients are classified into 5 categories of "definite, probable, possible, unlikely, unrelated". Each "causality" is defined as "causality" when judged to be either "definite, probable, possible" and "no causality" when judged to be either "unlikely, unrelated" (see TABLE 7.3.2.

According to Grade of adverse events, serious adverse events requiring expedited reporting as specified in "10.1. Serious Adverse Events and Expedited Reporting" should be reported to the Research Secretariat in accordance with "10.2. Mandatory Reporting and Reporting Procedures of the Investigator".

Table 7.3.2. Criteria for a Causal Relationship Between Adverse Events and Treatment

	Determination	Approach to determination
Cause Fruit Concern Associate Ah Fistula	Definite	The AE is clearly related to the intervention<br>Adverse events are apparently caused/aggravated by protocol treatment and are unlikely to be due to exacerbation of the etiology or other factors (comorbidities, other medications/treatments, or incidents).
	Probable	The AE is likely related to the intervention<br>It is unlikely that the adverse event was caused/aggravated by progression of the underlying pathology or other factors (comorbidity, other drugs/treatments, incident) and is likely to be due to protocol treatment.
	Possible	The AE may be related to the intervention<br>It is plausible (plausible) that an adverse event is considered to have occurred/become more severe with protocol treatment, and unlikely to be due to exacerbation of the etiology or other factors (comorbidities, other medications/treatments, or incidents).
Cause Fruit Concern Associate In Pouch	Unlikely	The AE is doubtfully related to the intervention<br>It is considered plausible (plausible) that the adverse event is due to exacerbation of the pathogenic disease or other factors (comorbidity, other drugs/treatments, incident) rather than to the protocol treatment that it is caused/aggravated.
	Unrelated	The AE is clearly NOT related to the intervention<br>It is judged that the adverse event was caused/aggravated by aggravation of the pathogenic disease or other factors (comorbidity, other drugs/treatments, incident) and is unlikely to be caused by protocol treatment.

8. Examination and Evaluation

8.1. Baseline examination and evaluation before registration

8.1.1. Test conducted before registration (regardless of time before registration)

1) Histopathology (immunostaining requires chromogranin A and synaptophysin) (see Section 3.3)
2) HBs antigen; HBc antibody ※1; HBs antibody ※1, HBV-DNA※2

※1: For HBsAg positive, HBc and HBs antibodies are not required, and HBV-DNA, HBeAg, and HBe antibodies are measured.

※2: Positive results for at least one of the HBc and HBs antibodies also indicate HBV-DNA prior to initiation of therapy (see Section 6.4.1).

8.1.2. Test performed within 56 days before enrollment (liver primary (or unknown primary))

If the liver is primary (or of unknown primary), the following tests should be performed (all allowing for tests performed in other hospitals):

1) Upper gastrointestinal endoscope
2) Lower gastrointestinal endoscope
3) FDG-PET test
4) Otolaryngology (head and neck) examination
5) Urology consultation (male only)
6) Gynecologic exam (female only)

8.1.3. Tests performed within 28 days before enrollment

1) Contrast-enhanced CT※1 (slice thickness of 5 mm or less, if the patient is allergic to iodine), both contrast-enhanced MRI of the abdomen and plain computed tomography (CT) of the imaging range, which is considered to be indispensable below, are performed. All tests are not performed in other hospitals.

※The following areas are indispensable for each primary organ, and if there is another site suspected of metastasis, the radiographic extent is added accordingly.

Primary Organ	Essential radiographic area
Esophagus	Cervical, chest, or abdominal regions
Stomach, duodenum, small intestine, colon, appendix, rectum,<br>Extrahepatic bile duct, Vater ampulla, gallbladder, and pancreas	Chest, abdomen, and pelvis
Hepatic NEC<br>(liver primary or liver metastasis of unknown primary)	Neck, chest, abdomen, and pelvis

2) Endoscopic ※2

Primary Organ	Mandatory test ranges
Esophagus, stomach, duodenum, and ampulla of Vater	Upper gastrointestinal tract (no examination performed at another hospital)
Small intestine, extrahepatic bile ducts, gallbladder, and pancreas	Be not mandatory
Colon, appendix, and rectum	Lower gastrointestinal tract (no examination performed at another hospital)
Hepatic NEC<br>(liver primary or liver metastasis of unknown primary)	8.1.2. Refer to the test performed within 56 days before registration.

※2 Unnecessary if the primary lesion has been resected

3) 12-lead, resting electrocardiography

8.1.4. Tests performed within 14 days before enrollment

1) General condition: PS (ECOG), body weight
2) Physical findings
3) Peripheral blood count: white blood cell count, neutrophil count (ANC: rod + segmented karyocyte), hemoglobin, platelet count

4) Blood biochemistry: total protein, albumin, total bilirubin, AST (GOT), ALT (GPT), BUN, creatinine, LDH, ALP, sodium, potassium, calcium, magnesium, CRP, FBS (fasting blood glucose)
5) Creatinine clearance (CCr): CCr estimates by Cockcroft-Gault equation
   Cockcroft-Gault formula
   Male: Ccr = {(140-Age) × Body Weight (kg)}/{72 × Serum Creatinine Level (mg/dL)}
   Women: Ccr = 0.85 × {(140-Age) × Body Weight (kg)}/{72 × Serum Creatinine Level (mg/dL)}
6) Tumour markers: NSEs, ProGRP, CEAs, SCCs (esophagus primary only), CA19-9 (non-esophageal primary only)
7) Chest X-P (frontal) (substitutable if contrast-enhanced and plain chest CT is obtained)

8.2. Examination and evaluation during treatment

The following safety examination and evaluation are minimal in frequency: Performing examinations more frequently at the discretion of the treating physician is not prohibited.

However, the examination for efficacy evaluation should be performed at specified frequencies, unless progression is suspected, because dense frequency may lead to bias in the efficacy evaluation.

8.2.1. Safety endpoint assessed weekly (CTCAE v4 0 Japanese translation)

The following 1)-3) tests or evaluations should be performed at least weekly. In addition, all of the following items should be checked and evaluated on the scheduled date or the day before anticancer drug administration. However, at least weekly examinations or evaluations should be performed in the same manner until day 28 using the starting date of the last course as day 1, even when the treatment is completed or terminated.

1) Peripheral blood count: white blood cell count, neutrophil count (rod + segmented count), hemoglobin, and platelet count
2) Biochemical tests: albumin, total bilirubin, AST (GOT), ALT (GPT), creatinine, sodium, potassium, calcium, magnesium, CRP
3) Subjective and objective findings (described according to CTCAE v4.0JAPANESE TRANSLATION)
   - General disorders and administration site conditions: fever, fatigue
   - Ear and labyrinth disorders: Tinnitus, hearing impairment
   - Skin and subcutaneous tissue disorders: alopecia
   - Gastrointestinal disorders: Constipation, diarrhea, nausea, vomiting, oral mucositis
   - Metabolism and nutrition disorders: anorexia, dehydration
   - Nervous system disorders: dysgeusia, peripheral sensory neuropathy, peripheral motor neuropathy
   - Musculoskeletal and connective tissue disorders: myalgia, arthralgia
   - Infections and parasites: bronchial infection, pulmonary infection, upper respiratory tract infection, mediastinal infection, pleural infection, catheter-related infection, biliary tract infection, gallbladder infection, and urinary tract infection
   - Blood and lymphatic system disorders: Febrile Neutropenia
   - Vascular disorders: Phlebitis
   - Respiratory, thoracic and mediastinal disorders: dyspnea, hypoxia, pneumonitis

8.2.2. Safety examination and evaluation for each course

1) General condition: Body weight
2) Blood chemistry: LDH, ALP FBS (fasting glucose)

8.2.3. Safety examination and evaluation to be performed as necessary

1) When dyspnea is observed
   - Chest X-P, percutaneous oxygen saturation: SpO2, arterial blood gases: PaO_2
2) If an arrhythmia is observed
   - 12-lead, resting electrocardiography
3) When HBs antigen, HBs antibody, or HBc antibody is positive
   - HBV-DNA (see 6.4.1.).

8.2.4. Efficacy end point

The following tests will be performed every 6 weeks during protocol treatment (±1 week allowed: 6 ± 1 week, 12 ± 1 week, and 18 ± 1 week after the start date of protocol treatment). Tumor response will be assessed according to "11.1. Response Evaluation". Evaluation of the response will be performed using the same test conditions and test methods as the baseline evaluation.

If CT is allergic to contrast material, it is evaluated with plain CT and/or contrast-enhanced MRI. Allergy to contrast media on MRI is assessed by plain CT or plain MRI. When the use of CT contrast medium and MRI contrast medium becomes difficult due to renal dysfunction, it is evaluated by simple CT or simple MRI.

1) Tumour markers: NSEs, ProGRP, CEAs (≧all cases), SCCs (esophagus primary only), CA19-9 (non-esophageal primary only)
2) Contrast-enhanced CT: In principle, the extent of imaging is as follows. () Areas in can be omitted if there is no lesion

Primary Organ	Essential radiographic area
Esophagus	Cervical, chest, or abdominal regions
Stomach, duodenum, small intestine, colon, appendix, rectum,<br>Extrahepatic bile duct, Vater ampulla, gallbladder, and pancreas	(chest), abdomen, and pelvis
Liver NEC (liver primary or liver metastasis of unknown primary)	(neck), (chest), abdomen, and pelvis

8.3. Examination and evaluation after completion of treatment

8.3.1. Efficacy evaluation after completion of treatment

After completion of the protocol treatment, examination and evaluation are made at the following timing:
The following tests should be performed at least every 6 weeks until progressions are confirmed or death.
If progressions are observed, only observation of the outcome will be continued.

1) Tumour markers: NSEs, ProGRP, CEAs (≧all cases), SCCs (esophagus primary only), CA19-9 (non-esophageal primary only)
2) Contrast-enhanced CT: In principle, the extent of imaging is as follows. Areas in parentheses can be omitted if there is no lesion

Primary Organ	Essential radiographic area
Esophagus	Cervical, chest, or abdominal regions
Stomach, duodenum, small intestine, colon, appendix, rectum,<br>Extrahepatic bile duct, Vater ampulla, gallbladder, and pancreas	(chest), abdomen, and pelvis
Liver NEC (liver primary or liver metastasis of unknown primary)	(neck), (chest), abdomen, and pelvis

8.4. Information on post- study treatment

After completion/termination of protocol treatment, the following items will be recorded on the Follow-up Form at each follow-up survey:

1) Content of post-study treatment (if post-study treatment is performed)
2) After protocol treatment termination, the initiation date of the first post-study treatment ((if post-study treatment is performed)
3) PS at the start of post-study treatment

8.5. Study calendar

If the primary site is the esophagus, stomach, duodenum, small intestine, colon, appendix, rectum, extrahepatic bile duct, ampulla of Vater, gallbladder, or pancreas

	Before registratikon Record Pre	After end of chemotherapy		From the date of protocol treatment discontinuation Within 28 days	Until progression after completion of protocol treatment	
		Course Before initiation of the course	During the course		Only at the start of post-treatment	Every 6 weeks
Physical findings						
Body weight	○^{14}	○				
PS	○^{14}				■	
Physical findings	○^{14}	○			■	
Laboratory tests						
WBC, differential (neutrophil) Hb, platelets	○^{14}	○	●	●		
Alb, T-Bil, AST, ALT, Cr, Na, K, Ca, Mg, CRP	○^{14}	○	●	●		
LDH, ALP, FBS	○^{14}	○				
Total protein, BUN, Ca	○^{14}					
NSE, ProGRP、CEA	○^{14}		△			
SCC (esophagus primary only), CA19-9 (other than primary esophageal)	○^{14}		△			
HBs antigen, HBc antibody, and HBs antibody	○^{前}					
Chest X-P (can be substituted if CT is taken)	○^{14}					
12-lead, resting electrocardiography	○^{28}					
Upper gastrointestinal endoscope (Primary: esophagus, stomach, duodenum)	○^{28}					
Lower gastrointestinal endoscope (Primary: colonic, appendix, rectum)	○^{28}					
Efficacy evaluation						
Contrast-Enhanced CT*	○^{28}		△			△
Toxicity evaluation						
Subjective symptom check		○	●	●		
Objective symptom check		○	●	●		
Submission of CRFs						
Pre-treatment Form		□				
Treatment Form			□			
Off-treatment Form				□		
Follow-up Form					2 times/year	

○^{前}: Conduct before registration, ○^{28}: Perform within 28 days before registration, ○^{14}: Perform within 14 days before registration

○:Conduct, ♀: Implementation at least once a week

△: Every 6 weeks (see 8.2.4.), ∞: Only once at the beginning of aftertreatment, □: Submitted.

*See 8.1.3 for the shooting range. Contrast-induced allergy or renal dysfunction is assessed by plain CT or contrast-

enhanced MRI.

※Follow-up Forms will be sent up to 1 years after completion of accrual and will be submitted after 1 years of registration in the individual patient according to the closing date of registration.

For liver NEC (liver primary or liver metastasis of unknown primary)

	Before registratikon Record Pre	After end of chemotherapy		From the date of protocol treatment discontinuation Within 28 days	Until progression after completion of protocol treatment	
		Course Before initiation of the course	During the course		Only at the start of post-treatment	Every 6 weeks
Physical findings						
Body weight	○[14]	○				
PS	○[14]				■	
Physical findings	○[14]	○			■	
Laboratory tests						
WBC, differential (neutrophil) Hb, platelets	○[14]	○	●	●		
Alb, T-Bil, AST, ALT, Cr, Na,K, Ca, Mg, CRP	○[14]	○	●	●		
LDH, ALP, FBS	○[14]	○				
Total protein, BUN, Ca	○[14]					
NSE, ProGRP、CEA、CA19-9	○[14]		△			
HBs antigen, HBc antibody, and HBs antibody	○[前]					
Chest X-P (can be substituted if CT is taken)	○[14]					
12-lead, resting electrocardiography	○[28]					
Upper gastrointestinal endoscope	○[56]					
Lower gastrointestinal endoscope	○[56]					
FDG-PET	○[56]					
Otolaryngologic examination	○[56]					
Urology consultation (male only)	○[56]					
Gynecologic exam (female only)	○[56]					
Efficacy evaluation						
Contrast-Enhanced CT*	○[28]		△			△
Toxicity evaluation						
Subjective symptom check		○	●	●		
Objective symptom check		○	●	●		
Submission of CRFs						
Pre-treatment Form		□				
Treatment Form			□			
Off-treatment Form				□		
Follow-up Form					□ 2 times/year	

○[前]: Conduct before registration, ○[56]: Perform within 56 days before registration, ○[28]: Perform within 28 days before registration,

○[14]: Implemented within 14 days prior to enrollment

○:Conduct, ♀: Implementation at least once a week

△: Every 6 weeks (see 8.2.4.), ∞: Only once at the commencement of post-treatment, □: Submitted.

*See 8.1.3 for the shooting range. Contrast-induced allergy and renal dysfunction are evaluated by plain CT or contrast-enhanced MRI.

※Follow-up Forms will be sent up to 1 years after completion of accrual and will be submitted after 1 years of

registration in the individual patient according to the closing date of registration.

9. Data collection

9.1. Case Report Form (CRF）

9.1.1. Types of CRF and submission deadlines

The case report forms (CRF) used in this study and their submission deadlines are as follows:

1) Pre-treatment report (blue)- Less than 2 weeks after enrollment
2) Treatment course records- Every 2 cycles/less than 2 weeks after end of protocol treatment
   3）-1 Treatment (yellow)
   3）-2 Test (yellow)
   3）-3 Adverse events (yellow)
3) Tumor shrinkage report (green) - Less than 2 weeks after judgment of effect
4) End of treatment report (red) - Less than 2 weeks after discontinuation/end of protocol treatment
5) Follow-up investigation (white)- By the deadline indicated in the Follow-up Investigation Form

- For “1) Pre-treatment report to 4) End of treatment report”, CRFs with basic patient information (enrollment code, facility name) pre-printed on them would be sent by post from the data center. If the CRFs do not arrive within one week of enrollment, or if the CRFs have been lost/damaged, the data center should be contacted by telephone, and a request should be made for them to be re-issued.
- “5) Follow-up investigation” would be sent by post from the data center at the time of follow-up investigations that are conducted at the same time as monitoring and interim/final analyses at the data center.

9.1.2. Storage CRF

- Completed CRFs must all be archived at the facility as photocopies or in electronic form.
- Copies of CRFs should be kept archived until the final analysis report is issued for reference while filling other CRFs, or for review while retrieving information from the data center.

9.1.3. Method of sending CRF

- All CRF must be sent by post or handed over in person at the data center. They must not be sent by FAX.
- To avoid the risk of personal patient information being leaked, the patient enrollment code should be used, instead of using the patient medical chart number at the facility, when contacting the data center for request of CRF dispatch.

9.1.4. Correction of the contents of CRFs

If any data necessary for the CRF are found to be missing or there are inappropriate category classifications after the start of the study, the CRFs may be corrected with the agreement of the head of the data center and the clinical trial secretariat, in a manner not exceeding the scope of data collected as prescribed in “8. Evaluation item/Clinical laboratory tests/Evaluation schedule”, and within the scope deemed not to increase medical and financial burden on the enrolled patient from the CRF correction. Modification of CRFs that do not require the main body of the protocol to be revised is not considered a protocol revision by JCOG. Reports to the head of the medical institution related to CRF correction and the request of application for revision should follow the rules of the facility.

10. Reporting of "disease or the like"(adverse events)

Site investigator should report to Study Coordinator/Principal Investigator (Study Chair) if a serious adverse event ("disease or the like" on Clinical Trials Act) occurs in accordance with the regulations of Clinical Trials Act (Law No. 16, 2017), Enforcement Regulations of Clinical Trials Act (MHLW Notification No. 17, 2018) and the regulations in this chapter based on the relevant notifications.

The most recent version of the report is available on the MHLW website [1)] and on the JCOG website [2)]. Use the most recent version of the report.

Serious adverse events occurring after the initiation of protocol treatment (after the date of registration if death) by the date of final follow-up will be subjects.

1）http://www.mhlw.go.jp/stf/seisakunitsuite/bunya/0000163417.html
2）http://www.jcog.jp/doctor/todo/researcher/harmfulness.html
3）http://www.pmda.go.jp/safety/reports/hcp/pmd-act/0002.html

10.1. Serious Adverse Events

Serious adverse events are defined as any of the following:
(These are classified as "disease or the like" on Clinical Trials Act.)

1） Death
2） Diseases that may lead to death
3） "Disease or the like" requiring hospitalization or prolongation of hospital stay for treatment.
4） Disability
5） "Disease or the like" that may lead to disability
6） Serious "disease or the like" according to 1) to 5)
7） Congenital disease or abnormality in later generations

1) Death

(i) All deaths that occur after registration and before the start of protocol treatment
(ii) All deaths (with or without causality to protocol treatment) that occur during protocol treatment or within 30 days of the last treatment day
(iii) Death that occur after 31 days from the last treatment date that are causally related to protocol treatment (definite, probable, possible)

2) "Disease or the like" that may lead to death

(i) Grade 4 adverse events that occur during protocol treatment or within 30 days of the last treatment day (excluding events in Table 10.1)
(ii) Grade 4 adverse events that occur after 31 days from the last treatment date (excluding events in Table 10.1) that are causally related to the protocol treatment (definite, probable, possible)

3) Hospitalization or prolongation of hospital[※1] stay for treatment

(i) Grade 3/2/1 adverse events that occur during or within 30 days of protocol treatment and requiring at least 24 hours of hospitalization or prolongation of hospital stay[※1] to treat the adverse event (excluding the event in Table 10.1).
(ii) Grade 3/2/1 adverse events that occur 31 days after the last treatment day and requires 24-hour or longer hospitalization or prolongation of hospital stay[※1] for treatment and causally related to protocol treatment (definite, probable, possible) (excluding the events in Table 10.1)

※ 1 "Hospitalization or prolongation of hospital stay" refers only to those for which hospitalization of at least 24 hours or prolongation of hospital stay is medically required for the treatment of an adverse event. The followings are not subjects for reporting:

- Hospitalization or prolongation of hospital stay performed for follow-up of adverse event that has disappeared or improved
- Hospitalization or prolongation of hospital stay for reducing patient burden, e.g. patients from distant areas.

- Hospitalization or prolongation of hospital stay for other medically unnecessary situation

4) Disability, 5) Disease that may lead to disability

Permanent or marked disability/dysfunction (excluding myelodysplastic syndromes, secondary cancers, etc.) or possible medical situation

6) Serious disease similar to 1) to 5)

7) Congenital disorders or abnormalities in later generations

Table 10.1. Adverse events excluded from the subjects of Expedited Reporting

SOC※(CTCAE ver4.0)	AE term
Blood and lymphocyte disorders	Anemia, bone marrow hypocellular
Gastrointestinal disorders	Constipation
General disorders and local symptoms	Fever
Infections and infestations	Viral hepatitis
Clinical laboratory test	ALP increased, CD4 lymphocytes decreased, high cholesterol, GGT increased, lipase increased, lymphocytes decreased, neutrophils decreased, platelet count decreased, serum amylase increased, WBC decreased, hyponatremia, hypokalemia, hyperglycemia, and hypoglycemia
Metabolism and nutritional disorders	Obesity, anorexia, hyperuricemia, and hypoalbuminemia
Musculoskeletal and connective tissue disorders	Fibrosis deep connective tissue and superficial soft tissue fibrosis
Renal and urinary disorders	Chronic kidney disease
Respiratory, thoracic, and mediastinal disorders	Sinus disorder and sleep apnea
Skin and subcutaneous tissue disorders	Hypohidrosis

※ SOC:System Organ Class

10.2. Investigator's reporting requirements and procedures

10.2.1. Expedited Reporting

In the event of a serious adverse event, the Subinvestigator must promptly inform the Investigator. If the Investigator cannot be contacted, the Site Coordinator or Subinvestigator must take over the responsibility of the Investigator. The Investigator must report adverse events according to the following procedures.

Attention should be paid not to include the patient's name and medical record number when sent.

Serious adverse events that occur after the initiation of protocol treatment (after the date of registration if death) by the date of final follow-up are subjects of Expedited Reporting.

1) "Disease or the like" that may lead to death or death specified in 10.1 1) and 2).

Primary reporting:

The Subinvestigator who is aware of the occurrence of adverse events will promptly notify the Investigator. The Investigator who receives the notice should fill out JCOG Adverse Event Report Form(for institution) and the "Disease or the like" Report Form to Certified Review Board specified in Clinical Trials Act Enforcement Regulations as far as possible and contact Principal Investigator/Study Coordinator via e-mail within 72 hours of knowledge of the occurrence of the adverse event.

Secondary reporting:

The Investigator should add detailed information on adverse events to JCOG Adverse Event Report Form(for institution) and the "Disease or the like" Report Form to Certified Review Board specified in Clinical Trials Act Enforcement Regulations within 7 days of knowledge of the occurrence of adverse events and send them to the Principal Investigator/Study Coordinator via e-mail. If necessary, attach copies of laboratory data, images, autopsy report, etc.

2) 10.1. 3) Disease or other medically important condition requiring hospitalization or prolongation of hospital stay for treatment. Adverse events are judged to be either of 10.1. 4)-7)

The Subinvestigator who is aware of the occurrence of adverse events will promptly notify the Investigator. The Investigator who receives notice must fill out JCOG Adverse Event Report Form (for institution) and the "Disease or the like" Report Form to Certified Review Board specified in Clinical Trials Act Enforcement Regulations within 10 days of knowledge of the occurrence of an adverse event and send them to the Principal Investigator/Study Coordinator via e-mail. If necessary, attach copies of laboratory data, images, autopsy report, etc.

3) Additional reporting

If new information is obtained after conducting the above reporting, the Investigator must add information to JCOG Adverse Event Report Form (for institution) and the "Disease or the like" Report Form to Certified Review Board specified in Clinical Trials Act Enforcement Regulations and report it as needed.

Table 10.2.1. Summary of Adverse Events which are subjects for Expedited Reporting and the deadline of reporting to Principal Investigator/Study Coordinator

Causal relationship	The patient was hospitalized due to Grade 1-3 AE.<br>Other medically important conditions		Grade 4		Death	
	Expected	Not expected	Expected	Not expected	Expected	Not expected
Present	Primary reporting: within 10 days<br>Additional reporting: as needed	Primary reporting: within 10 days<br>Additional reporting: as needed	Primary reporting: within 72 hours<br>Secondary reporting: within 7 days<br>Additional reporting: as needed			
None	<Only on-treatment or within 30 days of last protocol treatment day>					
	Primary reporting: within 10 days<br>Additional reporting: as needed	Primary reporting: within 10 days<br>Additional reporting: as needed	Primary reporting: within 72 hours<br>Secondary reporting: within 7 days<br>Additional reporting: as needed			

* 4) Disability, 5) "Disease or the like" that may lead to disability, and 6) "Disease or the like" that are serious similar to 1) to 5) in 10.1., 7) Congenital disorders or abnormalities in later generations

※ "Unexpected" refers to those not listed in "7. Expected Adverse Events"

Table 10.2.1. Summary of Adverse Events which are subjects for Expedited Reporting and the deadline of reporting to Principal Investigator/Study Coordinator

Causal relationship	The patient was hospitalized due to Grade 1-3 AE.<br>*Other medically important conditions		Grade 4		Death	
	Expected	Not expected	Expected	Not expected	Expected	Not expected
Present	Primary reporting: within 10 days<br>Additional reporting: as needed	Primary reporting: within 10 days<br>Additional reporting: as needed	Primary reporting: within 72 hours<br>Secondary reporting: within 7 days<br>Additional reporting: as needed			
None	<Only on-treatment or within 30 days of last protocol treatment day>					
	Primary reporting: within 10 days<br>Additional reporting: as needed	Primary reporting: within 10 days<br>Additional reporting: as needed	Primary reporting: within 72 hours<br>Secondary reporting: within 7 days<br>Additional reporting: as needed			

* 4) Disability, 5) "Disease or the like" that may lead to disability, and 6) "Disease or the like" that are serious similar to 1) to 5) in 10.1., 7) Congenital disorders or abnormalities in later generations

※ "Unexpected" refers to those not listed in "7. Expected Adverse Events"

10.2.2. Reporting to the Administrator of participating medical organizations

If an Adverse Event which is subjects for Expedited Reporting occurs and is assessed as causal after reporting to Principal Investigator and reported to Certified Review Board, the Investigator must report it to the Administrator of the relevant medical institution in accordance with the requirements of the medical institution.

10.3. Responsibilities of Principal Investigator/Study Coordinator

10.3.1. Determination of necessity of suspension of registration and emergency notification to institutions

Principal Investigator/Study Coordinator who received the report from the Site Investigator should report to Group Chair and determine the urgency, significance, and impact of the report. If needed, take measures such as suspending registration (contacting JCOG Data Center and all participating institutions) and urgently communicating information to participating institutions. Telephone calls can be made to Data Center and institutions as urgent, but they should also be promptly contacted by document (e-mail).

10.3.2. Reporting to JCOG Operations Office and Certified Review Board and MHLW

1) Reporting from the Principal Investigator/Study Coordinator to JCOG Operations Office

Principal Investigator/Study Coordinator should consult with Group Chair and report to JCOG Operations Office (Safety Contact) by e-mail within 72 hours of knowledge of the occurrence of the adverse event, if reported AE is considered to meet the adverse events specified in 10.1.1 1)~7). In doing so, to the extent feasible, Principal Investigator/Study Coordinator should send "JCOG Adverse Event Report Form (for institution)" sent from the institution, "Disease or the like Report Form" addressed to Certified Review Board as stipulated in Clinical Trials Act Enforcement Regulations, and attach "JCOG Adverse Event Report (for Study Coordinator)" with Study Coordinator/Principal Investigator's view (including judgments of causality and expectation, and judgments of continuation/discontinuation of the study)". For the expected adverse events of 10.1.1 1)~7)., include a discussion not only of the individual patient's course but also of whether the frequency of appearance is within the expected range.

2) Reporting to Certified Review Board

JCOG Operations Office (Safety Contact) reviews the appropriateness of the judgement of causality and expectation of adverse events reported in the above procedures and can ask Principal Investigator/Study Coordinator to reconsider them if there is any doubt. Adverse events considered by Principal Investigator/Study Coordinator and JCOG Operations Office to be related to the protocol treatment and to be the subject of reporting in the following tables should reported to Certified Review Board through JCOG Operations Office.

If there is a disagreement between Principal Investigator/Study Coordinator and JCOG Operations Office, report it to Data and Safety Monitoring Committee and seek final judgment from the Chair of Data and Safety Monitoring Committee. However, if the reporting may exceed deadlines of reporting, the report can be tentatively reported as "causal" to Certified Review Board.

Reporting subjects and reporting deadline

Principal Investigator/Study Coordinator must report to Certified Review Board through JCOG Operations Office within the following time periods after knowledge of the occurrence of adverse events.

Causal relation ship	The patient was hospitalized due to Grade 1-3 AE. Other medically important condition		Grade 4		Death	
	Expected	Not expected	Expected	Not expected	Expected	Not expected
Present	No need of reporting	Within 15 days	Within 15 days	Within 7 days	Within 15 days	Within 7 days
None	No need of reporting	No need of reporting	No need of reporting	No need of reporting	No need of reporting	No need of reporting

※ "Unexpected" refers to those not listed in "7. Expected Adverse Events"

Principal Investigator/Study Coordinator must report adverse events to Certified Review Board through JCOG

Operations Office within the following deadlines after knowledge of the occurrence of adverse events.

Causal relation ship	The patient was hospitalized due to Grade 1-3 AE. Other medically important condition		Grade 4		Death	
	Expected	Not expected	Expected	Not expected	Expected	Not expected
Present	Within 30 days	Within 15 days	Within 30 days	Within 15 days	Within 15 days	Within 15 days
None	No need of reporting	No need of reporting	No need of reporting	No need of reporting	No need of reporting	No need of reporting

※ "Unexpected" refers to those not listed in "7. Expected Adverse Events"

3) Reporting to MHLW

Principal Investigator/Study Coordinator of the study with unapproved or off-label health care should report "Disease or the like" Report Form specified in Clinical Trials Act Enforcement Regulations to MHLW through JCOG Operations Office, if an adverse event is considered to be unexpected and to have a causal relationship to the protocol treatment (See 10.5. Responsibilities of the Data and Safety Monitoring Committee).

Reporting subjects and reporting deadlines

Principal Investigator/Study Coordinator should report to the MHLW through JCOG Operations Office within the following deadlines after knowledge of the occurrence of adverse events.

※ Pharmaceuticals and Medical Devices Agency Safety Division I (trk-shippeitouhokoku@pmda.go.jp)

Causal relation ship	The patient was hospitalized due to Grade 1-3 AE. Other medically important condition		Grade 4		Death	
	Expected	Not expected	Expected	Not expected	Expected	Not expected
Present	No need of reporting	Within 15 days	No need of reporting	Within 7 days	No need of reporting	Within 7 days
None	No need of reporting	No need of reporting	No need of reporting	No need of reporting	No need of reporting	No need of reporting

※ "Unexpected" refers to those not listed in "7. Expected Adverse Events"

4) Additional reporting

Following receipt of secondary or additional reports from the Investigator, Principal Investigator/Study Coordinator must add additional information from the primary report and their views to JCOG Adverse Event Report Form (for the institution) and the "Disease or the like" Report Form to Certified Review Board set out in Clinical Trials Act Enforcement Regulations, and promptly contact JCOG Operations Office (Safety Contact) by e-mail. If the report was sent to Certified Review Board and the MHLW in the primary reporting,, the secondary reporting and additional reporting must be made in the same manner.

10.3.3. Notification to the Site Investigators

When reported to Certified Review Board, Principal Investigator/Study Coordinator should inform the Investigators of all participating institutions of the review results and recommendations by documents (e-mail is allowed). Principal Investigator/Study Coordinator must inform the Investigators without waiting for Certified Review Board review if there is any urgent information to be disseminated. In the event that reported to the MHLW, Principal Investigator/Study Coordinator should notify the Investigators of all participating institutions.

In addition, even if no reporting is made to Certified Review Board, Principal Investigator/Study Coordinator must inform the Investigator of the reporting institution of the decision of Principal Investigator/Study Coordinator by documents (e-mail is allowed).

10.3.4. Assessment of Adverse Events in Periodic Monitoring

During Periodic Monitoring, Principal investigator/Study Coordinator should carefully review the adverse events

in the Monitoring Reports issued by the Data Center and ensure that there are no missed reporting from the participating institutions. It should also be confirmed that all reported adverse events are listed in the Monitoring Reports. The presence or absence of a missed reporting should be indicated in the column of the results of Group review on the Periodic Monitoring Report.

10.4. Responsibilities of the Site Investigators at the participating institutions (including the relevant institution)

In accordance with the instructions of Principal Investigator/Study Coordinator, the Site Investigator at the participating institution should report to the administrator of the relevant institution if the adverse event is subjects of reporting of "disease or the like" to Certified Review Board in accordance with the regulations of the relevant institution.

10.5. Responsibilities of the Data and Safety Monitoring Committee

JCOG Operations Office (Safety Contact) should check the details of the adverse event reports received from the Principal Investigator/Study Coordinator according to the procedures described in 10.3.2. and should report them to Certified Review Board and the MHLW according to the procedures described below, with the presence or absence of causality or expectation.

In addition, Principal Investigator and Director of Data Center can hear the opinions of JCOG Data and Safety Monitoring Committee according to the reported adverse events. If a review request is issued, Data and Safety Monitoring Committee can review the appropriateness of the institutional response to adverse events and the propriety of continuation of the study in a consensus or written form.

In addition, the submitted information (JCOG Adverse Event Report (for institution), JCOG Adverse Event Report (for Study Coordinator), "Disease or the like" Report, etc.) will be stored semi-permanently in JCOG Operations Office.

Subjects, destination and deadlines for reporting after the knowledge by Investigator/Study Coordinator are as described in 10.3.2.

11. Response Evaluation and Endpoint Definition

11.1. Response assessment (only for patients with measurable disease)

Tumour response assessment will be performed according to the following steps according to the [49] of version 1. 1-Japanese translational JCOG version-Revised RECIST guideline (version 1. 1) Revised new guidelines for the assessment of treatment response in solid tumours (RECIST guidelines). RECISTv1. The 0 original article stipulates that "the use of this guideline for the purpose of determining continuation of treatment is not the subject of this guideline." Similar statements continue to be included in RECISTv1 1 as follows.

> "Many oncologists make decisions about whether to continue treatment based on both objective imaging criteria and symptom-based criteria for follow-up of patients with malignancies in their daily clinical practice, but these revised guidelines are not intended to be used to make decisions about whether or not to continue treatment in these individual patients, unless the treating oncologist determines that it is appropriate."

Therefore, the "overall effect" as determined by RECIST Guideline-based response assessment should be used to determine whether a drug or regimen shows encouraging results that merit continued developmental studies. In other words, judgment of whether or not to continue treatment in individual patients should not be based on CR/PR/SD/PD of overall efficacy, but rather on "clinical judgment" based on comprehensive consideration of symptoms, physical findings, and various laboratory data, in addition to imaging findings.

Therefore, it may be clinically appropriate to continue protocolized treatment, even when PD (Progressive Disease: progression) is judged as an overall response based on the assessment of response based on imaging. In this case, the pros and cons of continuing protocol treatment should be determined based on clinical judgment, regardless of response assessment, but the date of the event for progression-free survival, which is judged to be an overall effect of PD, should be used. This is due to three reasons: (i) it may be possible to decide whether protocol treatment should be continued for each group; (ii) RECIST is a criterion intended to standardize not only response rates but also progression-free survival; and (iii) the standard definition of US Cooperative Group is that PD is the event of progression-free survival for any reason if the overall response is PD.

On the other hand, if a physician judges "clinical progression" based on clinical and comprehensive judgment not based on diagnostic imaging, even if PD is not met by the response criteria based on diagnostic imaging, protocol treatment should be discontinued in accordance with "6.2.2. Criteria for discontinuation of protocol treatment". If "clinical exacerbation" is judged, even if "PD" is not judged by the response evaluation, the day of "clinical exacerbation" is considered as an event of progression-free survival. This is because imaging is often not performed as planned after a patient is judged to have a "clinical progression" and therefore the risk of overestimating progression-free survival is greater if "clinical progression" is not an event for progression-free survival. It is also statistically incorrect (informative censoring) to treat "clinical progression" as "censoring" progression-free survival, as it would censoring patients at increased risk of progression or death.

In RECISTv1 1, the original article described "definite progression (unequivocal progression") in the PD criteria for non-target lesions as "marked progression of non-target lesions that deserves discontinuation of treatment as an increase in total tumor burden" and therefore described "marked progression of non-target lesions" as "judgement of whether or not to continue treatment in individual patients" in some of the PD criteria for non-target lesions, which is confusing. It should be noted that this "unequivocal progression" is a criterion of judgment restricted to "PD of non-target lesions".

The relation between the events of 'PD', 'clinical progression', 'progression', and progression-free survival in JCOG is as in the lower panel.

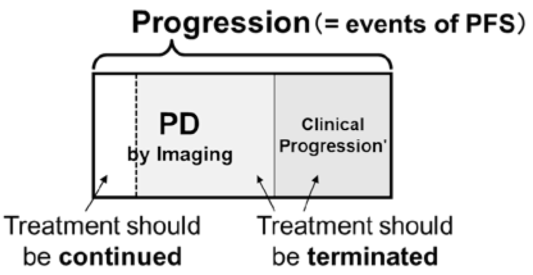

Figure 11.1. Relationship Between Exacerbations, PD on Imaging, and Clinical Exacerbations.

11.1.1. Baseline Evaluation

「8.1. According to "Pre-registration Evaluation Items" contrast enhanced-enhanced computed tomography (CT) with a range of indispensable for the primary organ is used to identify neoplastic lesions before enrollment, and each lesion is classified into "measurable lesions" and "unmeasurable lesions". If CT is allergic to contrast medium, both contrast-enhanced MRI and plain CT should be performed.

Tumor diameters are measured by CT or MRI in cross-sectional views, and sagittal and coronal measurements by three-dimensional reconstructed images are not used. Baseline assessment will be done using the latest imaging studies within 28 days prior to enrollment. If the imaging test is re-examined after enrollment and before the start of treatment, the latest imaging test with re-examination should be used.

11.1.2. Definition of measurable lesions

Lesions that fall under any of the following conditions are considered measurable lesions (measurable lesion):

1) Non-nodal disease (non-nodal disease) of 10 mm or greater in greatest dimension on CT or MRI with a slice thickness of 5 mm or less
2) CT or MRI of 5 mm or less slice thickness showing lymph node lesion of 15 mm or more in short diameter (Nodal lesions with short diameters between 10 mm and less than 15 mm are defined as non-target lesions, and those with short diameters less than 10 mm are not)

All other lesions will be non-measurable (non-measurable lesion).

Caution should be exercised because the following lesions are not measurable regardless of the examination method or the size of the lesion.

- Bone lesions (excluding osteolytic lesions with measurable soft tissue components)
- Cystic lesion
- Leptomeningeal lesions
- Ascites, pleural effusion, and pericardial effusion
- Lymphangiosis of the skin and lungs
- Palpable but not measurable abdominal mass or enlargement of abdominal organs
- Superficial skin lesions

11.1.3. Target Lesion Selection and Baseline Recording

Up to five measurable lesions, in descending order of diameter (non-lymph node lesions are long diameters and nodal lesions are short diameters), up to two lesions per organ are selected to be target lesions (target lesion) among measurable lesions at enrollment. Selection should take into account the universal inclusion of as many organs with measurable disease as possible and the reproducibility or ease of measurement (reproducible repeated measurement) of repeated measurements (avoiding lesions that are not measurable even if they are large in diameter).

For selected target lesions, in order from cranial to caudal, site (code), test method, test date, long diameter of non-lymph node target lesion, short diameter of nodal target lesion, and sum of all target lesion diameters (hereafter, sum of diameters) will be recorded in Pretreatment Report 3.

11.1.4. Baseline recording of non-target lesions

For lesions not selected as target lesions, all measurable or non-target lesions (non-target lesion) should be recorded as site of lesion (code), method of examination, and date of examination in Pretreatment Report 3. Multiple non-target lesions within the same organ may be recorded as a single lesion (e.g., multiple enlarged pelvic lymph nodes, multiple liver metastases).

11.1.5. Determining tumor response

Evaluation of target and non-target lesions will be performed every 6 weeks according to "8.2 Testing and Evaluation during Treatment" in the same manner as at enrollment. Target lesion diameter, non-target lesion disappearance or progression will be recorded in the "Tumor Reduction Effect Report".

11.1.6. Response Evaluation Criteria for Target Lesions

・CR(Complete Response): Complete response

When all non-lymph node target lesions disappear and all nodal target lesions have a short diameter of less than 10 mm. If a nodal target lesion is selected at baseline, the effect of the target lesion may be CR

even if the sum of diameters is not 0 mm.

・PR(Partial Response): Partial response

30% or more reduction in target diameter sum compared to baseline diameter sum

・PD(Progressive Disease): Progress

Compared to the minimum diameters (when the baseline is the minimum value during the passage, this is the minimum sum of diameters), the sum of the target disease increases by more than 20%, and the sum of diameters increases by more than 5 mm even in absolute value.

・SD(Stable Disease): Stability

There is no reduction corresponding to PR and no increase corresponding to PD compared to the smallest sum of diameters during the course

・Lack of study; Not all Evaluated

If the test cannot be performed for any reason or if neither CR, PR, PD, or SD can be determined

$$\text{Percentage of reduction of the diameter sum} = \frac{\text{Pre-treatment sum of diameters-sum of diameters at study}}{\text{Pre-treatment diameter sum}} \times 100\%$$

$$\text{Increasing Percentage of Diagram} = \frac{\text{Sum of diameters at study-minimum sum of diameters}}{\text{Minimum sum of diameters}} \times 100\%$$

※ Measured target lesion diameters are recorded whenever measurable (e.g., <5 mm). If the target lesion diameter is judged to be too small to be measured (too small to measure), the diameter should be 0 mm if the tumor lesion is judged not to be retained, and 5 mm if the tumor lesion is judged to be retained, regardless of the CT slice thickness.

※ PD is defined when the reduction ratio meets the condition of PR and the concomitant increase ratio meets the condition of PD.

※ When one lesion is separated during treatment, each diameter is added to the sum of diameters.

※ If more than one lesion fuses and the boundary cannot be distinguished during treatment, the diameter of the fused lesion is added to the sum of the diameters. The diameter of each lesion is added to the sum of diameters when the boundary of the lesion is identifiable, even if the lesion is in contact with each other.

11.1.7. Response Evaluation Criteria for Non-Target Lesions

・CR(Complete Response): Complete response

When all non-lymph node non-target lesions disappear, the short diameter of all nodal non-target lesions becomes less than 10 mm, and all tumour markers (NSEs, ProGRP※) are below the upper shared reference limits.

Because ※ProGRP is not included in the shared baseline range, the baseline range is 6.5-46.0 pg/mL.

・Non-CR/non-PD: non-CR/non-PD

Residual one or more non-target lesions (including residual nodal non-target lesions ≧10 mm in short diameter) and/or tumour markers (NSEs, ProGRP※) exceeding the shared upper reference limits.

・PD(Progressive Disease): Progress

'Apparent exacerbation' (including relapse) of pre-existing non-target lesions.

For measurable disease: A marked progression of a non-target lesion that deserves discontinuation of treatment as an increase in overall tumor burden must be observed if the effect of the target lesion is SD or PR but is judged to be "clear progression" based on the change in the non-target lesion. If the effect of the target lesion is SD or PR, then an increase in the tumor burden of the non-target lesion to a degree that far exceeds the decrease in tumor burden is considered "obvious progression" and otherwise Non-CR/non-PD.

If only unmeasurable disease is present, the increase in non-target disease, as judged to clearly exceed the tumor burden corresponding to a 20% increase in diameter and a 73% increase in tumor volume, is

considered "definite progression".

・NE(Not all Evaluated): Lack of study

If the test could not be done for any reason or if neither CR, Non-CR/non-PD nor PD could be determined.

11.1.8. Presence or absence of new lesions

If a lesion that was not present at baseline was observed after the start of treatment, it is considered "new lesion" to be present.

However, a "new lesion" requires that it is not an imaging change due to a difference in the imaging method from the baseline assessment or a change in the imaging modality, nor is it an imaging change due to a condition other than the tumor. For example, a cystic lesion arising within a lesion due to necrosis of a liver metastatic lesion is not a new lesion. New lesions will be defined as new lesions by examination of sites that were not mandatory at baseline (pre-enrollment study).

If a lesion disappears and later reappears, measurement is continued. However, the effect at the time the lesion reappears depends on the status of the other lesion. When the overall effect reappears after CR, the lesion is judged as PD at the time of reappearance. When the overall effect is PR or SD, on the other hand, once the disappeared lesion reappears, the diameter of the lesion will be added to the sum of the diameters of the remaining lesions to calculate the effect. That is, in the presence of many residual lesions, even if one lesion reappears after an apparent disappearance, it is not judged as PD by itself, and it is judged as PD when the sum of the diameters of all lesions meets the criteria for PD. This is because of the perception that the majority of lesions do not truly 'disappear' and are not only depicted by the limits of resolution of the imaging modalities used.

If there is a possibility of a new lesion but it cannot be determined, it should not be a new lesion, and imaging should be reexamined at a clinically relevant time. If a new lesion is confirmed by repeat imaging, the new lesion will appear based on the date of imaging at which the new lesion is confirmed.

11.1.9. Overall efficacy (Overall Response)

The overall response (Overall response) will be determined by combining the effects of target lesions, non-target lesions, and the presence or absence of new lesions every 6 weeks according to Table 11.1.9.a below. The overall effect in the absence of a non-target lesion at baseline will be determined by the effect of the target lesion and the presence or absence of a new lesion, and the overall effect in the absence of a target lesion at baseline will be determined according to the effect of a non-target lesion and the presence or absence of a new lesion according to Table 11.1.9.b.

Table 11.1.9.a. Overall efficacy at each time point for target lesions (with or without non-target lesions)

Target lesion	Nontarget lesions	New lesions	Overall effect
CR	CR	None	CR
CR	Non-CR/non-PD	None	PR
CR	Lack of study	None	PR
PR	Lack of Non-PD or study	None	PR
SD	Lack of Non-PD or study	None	SD
Lack of study	Non-PD	None	NE
PD (obvious progression)	Irrespective of	With or without	PD
Irrespective of	PD	With or without	PD
Irrespective of	Irrespective of	Present	PD

Table 11.1.9.b. Overall efficacy at each time point for patients with non-target lesions only

Nontarget lesions	New lesions	Overall effect
CR	None	CR
Non-CR/non-PD	None	Non-CR/non-PD
Lack of study	None	NE
PD (obvious progression)	With or without	PD
Irrespective of	Present	PD

11.1.10. Best overall effectiveness (Best Overall Response)

CR > PR > SD > PD > NE is considered good, and the best overall effect is the best overall effect throughout the entire course.

PD is defined when imaging cannot be determined due to exacerbation of obvious disease or death before the first response assessment. In addition, NE is defined if it cannot be determined by imaging due to discontinuation of toxicity before the first response assessment or patient refusal.

11.2. Definitions of analyses set

The analysis sets used in periodic central monitoring, interim analysis, and final analysis are defined as follows: The flow diagram below shows the analysis sets.

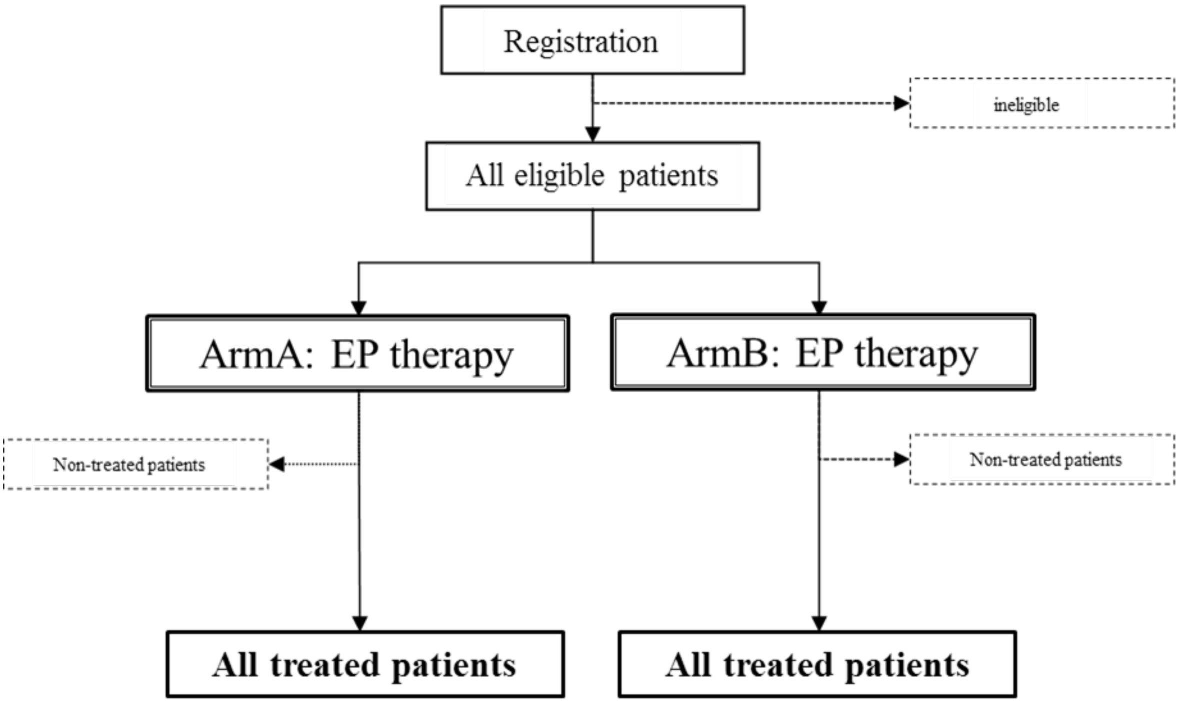

11.2.1. All registered patients

「5.1. Among the patients enrolled according to the Procedures for Enrollment, the population excluding duplicate or mis-enrollment is considered as "all enrolled cases".

11.2.2. All eligible patients

The group excluding "ineligible cases (post hoc ineligibles, de facto ineligibles, violation of registration)" determined by group review from all registered patients is regarded as all eligible patients. Ineligible cases as judged by the investigator or sub-investigator alone are included in all eligible patients. Only those judged not eligible by the central pathological diagnosis are ineligible and not included in all eligible patients.

11.2.3. All treated patients

Of all enrolled patients, all patients for whom part or all of the protocol treatment was performed will be defined as all treated patients.

The decision to treat "non-treated patients" for whom no protocol treatment has been given and whether it is excluded from the safety analysis can be determined by the data center with the consent of the Research Office. Ineligible patients will be excluded from all treated patients. However, if there are circumstances in which ineligible patients are included in the analysis, the nature of the ineligibility will be examined and determined by the Research Secretariat in consultation with JCOG Data Centre.

11.2.4. Patients eligible for central pathology diagnosis

Of all eligible patients, the population excluding ineligible patients with a central pathological diagnosis will be considered as eligible patients with a central pathological diagnosis.

11.3. Definition of endpoints

Endpoint	Events (whichever is earlier)		Censoring date
Overall survival time<br>Overall survival(OS)	All deaths	-	Date of final survival confirmation
Progression-free survival time<br>Progression-free survival(PFS)	All deaths	Progression/recurrence	Final date of clinically confirmed freedom from exacerbation

11.3.1. Overall survival

The duration from the date of registration to the date of death from any cause.

- Survivors are censored at the date of final survival confirmation (survival confirmation by telephone contact is also permitted, but the fact that survival confirmation was performed should be recorded in the medical record).
- Patients lost to follow-up are censored at the last date of survival confirmation before lost to follow-up.

11.3.2. Progression-free survival (PFS: Progression-free survival).

The duration from the date of registration to the date of the judgement of exacerbation or death from any cause, whichever comes first.

- The exacerbation (progression) includes both imaging-based PD (progression) and exacerbation of pathogenic disease (clinical exacerbation) that cannot be confirmed by imaging studies in Section 11.1.9. Global Effectiveness. If an exacerbation is judged based on diagnostic imaging, the exacerbation date is the test date on which the imaging was performed, and in the case of clinical exacerbation, the date of clinical judgment is the exacerbation date. PD may occur in accordance with the Response Evaluation Criteria, even if the tumor diameter is very small, but the disease is clinically judged to be "not obviously aggravated" in accordance with the Response Evaluation Criteria Criteria (in this case, clinical judgment should be prioritized for continued treatment with the protocol). In addition, even if PD is not observed according to the response evaluation criteria, the clinical judgment is prioritized for progression if it is judged to be clinically obvious exacerbation.
- In survivors who are not judged to have progression, clinically confirmed progression is censored (date of final progression-free survival confirmation) (confirmation of progression-free by imaging or specimen examination is not mandatory and clinical progression-free by outpatient examination etc. is not permitted. Telephone contact alone is not permitted. If information on progression or progression-free is obtained at a medical institution or referral center, receive and retain a medical information form describing the rationale for diagnosis. In this case, telephone contact alone is not permitted).
- Events and censoring are treated similarly, if chemotherapy is terminated for reasons such as toxicity or patient refusal, and if other therapies are added as post-study treatment. i.e., it is not censored at the time of treatment termination or at the date of initiation of post-study treatment.
- When the diagnosis of exacerbation is based on imaging, the relapse is not regarded as an event at the test date of imaging with "suspicious diagnosis of relapse", but regarded as an event at the later test date of imaging with "definite diagnosis of relapse"" If the event is judged to be clinically aggravated based on diagnostic imaging, the event is considered to be the day of the progression.
- If the definitive diagnosis of relapse or new lesion is based on biopsy pathology, the date of clinical diagnosis is defined as the date of clinical diagnosis when the diagnosis of recurrence or new lesion is made clinically, and the date of biopsy is defined as the date of event when the diagnosis of recurrence is made based on biopsy pathology diagnosis without clinical diagnosis of recurrence.
- The incidence of a second cancer (metachronous double cancer) is not censored or event, and progression-free survival is defined until other events are observed.

11.3.3. Response rate (response rate) Response proportion (Response rate)

Among all enrolled patients with measurable disease, the response rate is defined as the proportion of patients with "11.1.10. Best Global Effect" that is either CR or PR.

11.3.4. Incidence of adverse events (adverse reactions)

Using all treated patients as the denominator, the frequency of the worst Grade during the entire course by CTCAE v4 0 Japanese translation JCOG version for each of the following adverse events (toxicities) will be determined by group:

- Laboratory tests: hemoglobin decreased, white blood cell decreased, neutrophil count decreased, platelet count decreased,

  Increased blood bilirubin, aspartate aminotransferase increased (AST or GOT),
  Alanine aminotransferase increased (ALT or GPT), creatinine increased,
  Hypernatremia, hyponatremia, hyperkalemia, hypokalemia, hypercalcemia, hypocalcemia,

hypermagnesemia, hypomagnesemia

- General disorders and administration site conditions: fever, fatigue
- Ear and labyrinth disorders: Tinnitus, hearing impairment
- Skin and subcutaneous tissue disorders: alopecia
- Gastrointestinal disorders: constipation, diarrhea, nausea, vomiting, oral mucositis
- Metabolism and nutrition disorders: anorexia, dehydration
- Nervous system disorders: dysgeusia, peripheral sensory neuropathy, peripheral motor neuropathy
- Musculoskeletal and connective tissue disorders: myalgia, arthralgia
- Infections and parasites: bronchial infection, pulmonary infection, upper respiratory tract infection, catheter-related infection, biliary tract infection,

  Gallbladder infection; Mediastinal infection; Pleural infection; Urinary tract infection
- Blood and lymphatic system disorders: Febrile Neutropenia
- Musculoskeletal and connective tissue disorders: arthralgia, Vascular disorders: phlebitis
- Respiratory, thoracic and mediastinal disorders: dyspnea, hypoxia, pneumonitis

In the other adverse event (toxicitiy) than the above, the proportion of occurrence are not calculated unless a large number of specific adverse events are observed, since only Grade 3 or more non-hematological toxicity ※ is reported in the Treatment Form.

※※ Non-hematological toxicity refers to adverse events other than those listed below in CTCAE v4.0-JCJCOG.
Anemia, decreased bone marrow cells, decreased lymphocyte count, decreased neutrophil count, decreased white blood cell count, and decreased platelet count.
CD4 lymphocytopenia

11.3.5. Dose intensity of cisplatin

Dose intensity of cisplatin per patient (DOOC.) will be calculated for all treated patients to assess treatment compliance with cisplatin. The summary statistics (minimum, 25% point, median, 75% point, maximum, mean, and standard deviation) will be calculated for each group.

- ・Active dose D.I. (mg/m2/week) = total drug dosebody surface areatreatment duration (weeks)
- Body surface area: BSA is calculated by height at enrollment and body weight calculated by Data Center.
- ・ Treatment period (weeks)

  Arm A = (start date of last course-start date of 1st course + 21)/7

  Arm B = (start date of last course-start date of 1st course + 28)/ 7

11.3.6. Incidence of serious adverse events (adverse reactions)

1) Grade 4 non-hematologic toxicities, early deaths, and treatment

Using all treatments as denominators, the percentage of patients with one or more Grade 4 non-hematologic toxic ※ that are considered to be related (either to definite, probable, possible) to the protocol treatment among the adverse events listed in the free text of the CRFs in addition to the stereotyped items in Section 11.3.4 is used as the numerator.

※※ Non-hematological toxicity refers to adverse events other than those listed below in CTCAE v4.0-JCJCOG.
Anemia, decreased bone marrow cells, decreased lymphocyte count, decreased neutrophil count, decreased white blood cell count, and decreased platelet count.
CD4 lymphocytopenia

2) Early mortality rate

Proportion of all deaths during the protocol treatment or within 30 days from the last protocol treatment day among all treated patients. Causes of death irrespective of causality with protocol treatment. However, if premature death occurs in patients excluded from all treated patients, the details are provided separately.

3) Proportion of treatment-related death (TRD incidence)

Proportion of all deaths judged as causally related (either definite, probable, possible) to the protocol treatment among all treated patients However, if TRD occurs in patients who have been removed from all treated patients, the details are presented separately.

12. Statistical consideration

Methods for statistical analysis are as follows: In addition, the details required for conducting specific analyses are specified in the statistical analysis plan prepared separately prior to the analysis, and in documents that clarify the endpoint definition, etc. If substantial changes occur in statistical analyses as described below, follow the policy set out in "13.6. Protocol Changes." Facilities are contacted by "14.1. Periodic Monitoring" for missing or abnormal results, and data collection or exclusion is determined by review by the Research Secretariat based on the results of inquiries to the institution in accordance with the policies stipulated in "14.1.2. eligible (eligibility/ineligibility)" and "14.1.3. Protocol Deviations/Violations". 「11. Handling of missing values and abnormal data that cannot be addressed by the definition and analysis method for each endpoint, as specified in "Definition of Response Evaluation and Endpoints" and "12. Statistical Items" below, is specified in the above statistical analysis plan.

12.1. Principal Analysis and Decision Criteria

The primary analysis of this study will be the final analysis.

The purpose analysis of this trial is to test whether one of the two community standard-of-care arm A (EP-therapy) and B (IP-therapy) outperforms primary endpoint overall survival. The null hypothesis of equal overall survival in the two groups in the main analysis will be tested by stratified log-rank test stratified by non-institutional allocation adjustment factors (primary organ [gastrointestinal vs. hepatobiliary-pancreatic]) in all patient patients. However, if it is assumed that a stratified log-rank test cannot be performed appropriately, such as when the number of subjects and events in each stratum is small, the allocation adjustment factors will be addressed in the analysis plan prepared without information related to the comparison between groups before performing a confirmatory analysis with comparison between groups. Sensitivity analysis will also be performed in all eligible patients and in patients eligible for central pathology diagnosis.

Due to interest in which treatment group is superior, the test will be two-tailed. The study-wise significance level for the study is set at both sides 10%. In the main analysis, two-sided 90% confidence intervals corresponding to a two-sided 10% significance level will be calculated, and in other analyses, two-sided 95% confidence intervals will be calculated for descriptive purposes.

We conclude that EP therapy is a more useful treatment when the two-sided p-value is less than 10% and the survival curve of EP therapy exceeds that of IP therapy. Meanwhile, we conclude that IP therapy is a more useful treatment when the two-sided p-value is less than 10% and the survival curve of IP therapy exceeds that of EP therapy. If the difference was not significant at a two-sided significance level of 10%, one of the two modalities cannot be judged to be useful. In this case, we conclude that both modalities continue to be the standard of care, as there is no evidence to actively recommend either one of them.

However, if there are major differences in the toxicity profiles of EP and IP therapies, contrary to prior assumptions, during the course of the study, and if the toxic therapy is not superior to other therapies, it is judged that it will not be of significance to be used in clinical practice, the following measures should be taken. In other words, the clinical hypothesis will be changed from two-sided to one-sided in an analysis plan with no information on group comparisons before a confirmatory analysis with group comparisons will be conducted, and the superiority of the toxicity treatment over other treatment modalities will be verified at a one-sided significance level of 5% (in this case, the policy of change for the interim analysis will be described in Section 12.3.2).

Estimates such as cumulative survival curves, median survival times, and annual survival rates are performed using Kaplan-Meier method. Brookmeyer and Crowley methods are used to obtain 95% confidence intervals for median survival. Greenwood's formula is used to obtain 95% confidence intervals for annual survival rates. Hazard ratios and their confidence intervals for treatment effects between groups are obtained using stratified Cox proportional hazards models with the same factors as the test of the primary analysis as estimates of treatment effects. Cox regression adjusted by imbalance background factors in addition to adjustment factors will be performed as needed.

The main analysis results will be summarized as the "Main Analysis Report" by the Data Center one year after the completion of enrollment and submitted to the Research Secretariat, Research Representatives, Group Representatives, Group Secretariats, Efficacy and Safety Assessment Committee, and JCOG Representatives.

The principal investigator/study coordinator summarizes the content of the main analysis report, prepares a

"Clinical Study Report" summarizing the conclusions, problems, interpretations and discussion of the results, and future policies of the entire study, and submits it to the Data and Safety Monitoring Committee and JCOG chair with approval from the Group chair and the Head of JCOG Data Center.

Approval of the clinical study report by the Data and Safety Monitoring Committee shall be considered as "end of the study."

12.2. Planned accrual, accrual period, and follow-up periods

「2.4.2. Based on the background presented in Clinical Hypothesis and Rationale for Number of Enrollment, we assume a median survival of 8 and 12 months (HR=0.67) for the inferior and superior treatment groups, respectively, among the two treatment groups. When a superiority trial design is used, 63 patients per group and 126 patients in both groups (114 events required) will be included in the required analysis using Schoenfeld & Richter methodological [50] at 6 years of enrollment, 1 year of follow-up, α = 10% (two-sided), and 70% power. The number of required Inferior treatment (number of required events) when the median survival time in A is different from the assumption as shown in Table 12.2.1 below.

Table 12.2.1. Number of Analyses Required (Number of Events Required)

	Power			
Median Survival (mo)	65%	70%	75%	80%
7.0 vs. 10.5	108(100)	124(114)	142(131)	162(150)
8.0 vs. 12.0	112(100)	126(114)	144(131)	166(150)
9.0 vs. 13.5	114(100)	130(114)	148(131)	170(150)

※※ Number of required events in parentheses

Based on these findings, the following will be established in view of some cases of loss to follow-up.

Planned enrollment: 70 patients in each group and 140 patients in both groups

Enrollment period: 6 years, follow-up period: 1 year after completion of enrollment

Consider redesigning the sample size if the prognosis is obviously better than assumed, or if it reaches 70 people within 2.5 years of enrollment initiation. Clinically meaningful differences will then be reviewed and redesigned in a blinded fashion prior to the conduct of the analysis.

<Additions in ver. 1.1>

The registration pace after the enrollment start was smooth and exceeded the plan, and 70 cases were reached in October, 2016, when 2 years and 2 months passed from the enrollment start. Since it exceeded the standard of 70 patients within 2.5 years from the initiation of enrollment specified above, acceptance was obtained at the meeting of the Hepatobiliary and Pancreatic Group on October 29, 2016, the group meeting of the Esophageal Cancer Group on November 19, 2016, and the group meeting of the Gastric Cancer Group on January 7, 2017 with respect to changing the power to 80% according to the rules at the time of the study plan. Therefore, the planned enrollment number was changed to 170. In addition, the "1-year analysis period" was added to the study period, and the following changes were made.

Planned enrollment: 85 patients in each group and 170 patients in both groups

Enrollment period: 6 years; Follow-up period: 1 year after completion of enrollment; Analysis period: 1 year; Total study period: 8 years

12.3. Interim Analysis and Early Termination of the Study

12.3.1. Purpose and Timing of the Interim Analysis

Once interim analyses will be conducted to determine if the primary objective of the study has been achieved during the study period. Interim analyses will be conducted to determine if it is reasonable to continue enrollment during enrollment. If the primary objective of the study is determined to be achieved, the study will be discontinued and the study results will be published promptly at the conference and in the article.

Interim analyses will be conducted using data from the initial periodic monitoring that will be queried after the time enrollment of half of the planned enrollment was obtained. Based on the information in the periodic monitoring report, the group will submit the presence or absence of changes in clinical hypotheses and, if any, details of changes to the Efficacy and Safety Assessment Committee by the time of the interim analysis.

During the first interim, patient accrual is continued in principle. If the progress of the study progressed as planned, the expected number of events at the time of the interim analysis under the assumptions presented in 12.2 is expected to be 44 if the interim analysis is conducted at 3 years after the start of enrollment.

12.3.2. Method of interim analysis

Interim analyses will be conducted by the JCOG Data Center. To keep the study-wise alpha errors at 10%, the multiplicity of the interim and final analyses is adjusted using Lan & DeMets's alpha spending functions, and statistical significance is examined for differences in primary endpoint between arms. As α spending functions, we use O'Brien & Fleming types.[51]

For details of the interim analysis, the statistical staff in charge of the group at the Data Center will prepare the statistical analysis plan by the time of the interim analysis. Actual interim analyses will be performed by statistical staff who are not in charge of the group and an interim analysis report will be prepared.

In the interim analysis, if the survival of one treatment group exceeds that of the period, and the p-value of the stratified log-rank test falls below the level specified by the above method, it is judged statistically significant and the trial is discontinued as a rule. The ineffective discontinuation is not planned at a stage where no statistically significant differences are observed unless prior assumptions regarding toxicity are changed. However, the clinical hypothesis may be changed for the reasons described in 12.1, and the primary analysis may be performed by one-sided rather than two-sided testing, which may result in deviations from prior assumptions. In such cases, the primary hypothesis change should be specified in the analysis plan to be prepared without information on group comparisons before a confirmatory analysis with group comparisons. If the overall survival curve in the highly toxic treatment group is below the other, the consideration of ineffective discontinuation should not be judged by a test and considered comprehensively.

12.3.3. Interim analysis Reporting and review of the results

The results of the interim analysis will be submitted to the Data and Safety Monitoring Committee by the Data Center as an Interim Analysis Report and reviewed for the acceptability of continuation of the study and for publication of the results. The Data and Safety Monitoring Committee considers whether to continue the study at the meeting and recommends whether to continue the study and whether to publish the results to principal physician or group chair based on the results of the review.

Members of the Data and Safety Monitoring Committee of the relevant group are not included in the review. Unless the results of the interim analysis make recommendations for discontinuation of the study from the Efficacy and Safety Assessment Committee, the research representative, research office, participating institution researchers, group representatives, and group secretaries of the study will not be able to know the results of the interim analysis until the final follow-up is completed.

When the Interim Analysis Report has been reviewed by the Data and Safety Monitoring Committee to recommend termination or change of all or part of the study, the principle investigator and group chair review the recommendations and decide whether to discontinue or change some of the study.

If the study is discontinued or part of the study is changed, the principal investigator and group chair shall submit in written form a request to the Data and Safety Monitoring Committee for permission to discontinue the study or a request to revise the protocol. Following approval by the Data and Safety Monitoring Committee, the principal investigator may discontinue the study or change part of the study.

The Study Chair and Group Chair can disagree with the recommendations of the Data and Safety Monitoring Committee, but if they fail to coordinate their opinions with the Data and Safety Monitoring Committee, they will ultimately follow the instructions of JCOG Chair.

If the study is terminated, the subsequent follow-up period will be 1 years from last registration per study.

If the interim analysis resulted in study termination, the interim analysis will be the primary analysis of the study. The Data Center, in cooperation with the Research Representative Physicians and Research Secretariat, will conduct the analysis required to complement the incomplete data and publish the results, focusing on the results of the interim analysis, and promptly prepare the Major Analysis Report and submit it to the Group and the Efficacy and Safety Assessment Committee.

12.4. Analysis of Secondary endpoints

Secondary endpoints analyses will be conducted to provide a supplementary discussion of the primary analysis results of the study. Because the analysis of secondary endpoint is exploratory, no multiplicity adjustments are made. Comparisons between arms are made where appropriate, note that when the results of the group comparisons are not significant, they do not mean that there is no difference between the two arms.

12.4.1. Analysis of safety secondary endpoints

Among Secondary endpoints, the safety endpoints are the incidence of adverse events and the incidence of serious adverse events, which are in principle the items of periodic monitoring (14.1. Periodic monitoring).

The incidence rate of adverse events will be summarized as well as the incidence rate of Grade3 or higher. For adverse events other than laboratory data, the incidence of Grade 2 or higher is also calculated.. Non-hematological toxicity incidence, early mortality, and treatment-related mortality rates of Grade4, which are serious adverse events, are reported in periodic monitoring reports with registration numbers and details. The rates of non-hematologic toxicity, early mortality, and treatment-related mortality for Grade4 will be calculated at the time of the interim analysis and the main analysis. When interval estimation of proportions is performed, accurate confidence intervals based on binomial distributions are used. Comparisons between arms will be made using Fisher's exact test where appropriate.

12.4.2. Analysis of efficacy secondary endpoints

Among Secondary endpoints, efficacy endpoints are response rate, progression-free survival, which will only be analyzed in the interim and primary analyses.

Secondary endpoints analyses do not adjust for multiplicity.

Progression-free survival will be included in all enrolled patients, but a comparison of all eligible patients, excluding ineligible patients, as determined by group study, will also be performed as a sensitivity analysis.

The response rate will include all enrolled patients with measurable disease, but a comparison of all eligible patients excluding ineligible cases determined after group review will also be performed as a sensitivity analysis.

Fisher's exact test will be used to compare response rates between groups, and binomial distribution-based exact confidence intervals will be used for interval estimation. Estimates, including progression-free survival curves, median progression-free survival, and time-point progression-free survival, will be performed using Kaplan-Meier method, Brookmeyer and Crowley methods will be used to obtain confidence intervals for median progression-free survival, and Greenwood formulas will be used to obtain confidence intervals for progression-free survival. Log-rank test is used for comparison between arms. Hazard ratios and their 95% confidence intervals for treatment effects between arms will be calculated using Cox's proportional hazards model as an estimate of treatment effect. Cox regression adjusted by imbalance background factors in addition to adjustment factors will be performed as needed.

12.5. Final analysis

The primary analysis will be the final analysis unless this study is withdrawn from the interim analysis.

If the interim analysis is withdrawn from the study, then after the end of the 1-year follow-up period, the final analysis will be performed after the final survey confirms the data and then analyses will be performed for all endpoints.

Except for the interim analyses and the final analysis, analyses with between-arms comparisons for the primary and secondary endpoints for efficacy are not performed unless approved by the Data and Safety Monitoring Committee.

If the final analysis is performed after the main analysis, the final analysis will be summarized by the Data Center as the "Final Analysis Report" and submitted to the Research Secretariat, Research Representatives, Group Representatives, Group Secretariats, Efficacy and Safety Assessment Committee, and JCOG Representatives.

The Study Representative Physician/Research Secretariat summarizes the content of the final analysis report, prepares the "Clinical Study Report" summarizing the conclusions, issues, interpretations and discussion of the results, future policies, etc. mainly from the clinical point of view (if the "Clinical Study Report" is prepared in the previous analysis report, it will be the "Clinical Study Report (Supplementary Version)) with additional updates), and submits it to the Study Representatives and JCOG Representatives with approval from the Group Representatives and the Head of JCOG Data Center.

Approval of the clinical study report by the Data and Safety Monitoring Committee shall be considered as "end of the study."

12.6. Exploratory analysis

To investigate the interaction between treatment effect and the subpopulation, subgroup analyses will be conducted exploratory with respect to the following factors: Because these analyses are not adequately powered and do not adjust for multiplicity, the results of each subgroup analysis should be interpreted as exploratory.

- PS0/1
- Age 65 years or older/<
- Gender (male/female)
- Primary organ (gastrointestinal tract/hepatobiliary pancreas)
- Organ of origin (esophagus/stomach/small intestine/large intestine/pancreas/biliary tract/liver NEC (liver primary or liver metastasis of unknown primary)
- Organ of origin (pancreas/non-pancreas)
- Extent of extension of the primary lesion (locally advanced/distant metastasis or recurrence)
- Extent of extension of the primary lesion (locally advanced/distant metastasis/recurrence)
- Prior radical resection of the primary lesion (none/present)
- Pathological diagnosis was biopsy/resection specimen
- Grade 3 tumour with morphologically similar features of NETs but Grade 3 proliferative activity/morphologically more atypical (previously classified as poorly differentiated endocrine carcinoma) on histopathology with central pathology
- Histopathological examination by central pathological diagnosis, including Small cell carcinoma/Large cell carcinoma /
- Histopathologically diagnosed by central pathology, Ki67 50% or higher/less than 50%

12.7. Premature withdrawal from the trial

In this study, early termination of the study may occur in the following cases:

1) Early termination due to discontinuation of interim analysis
2) Early termination due to adverse events
3) Early termination due to poor enrollment
4) Early termination due to other reasons

12.7.1. Early termination by interim analysis

In this study, based on the criteria described in 12.3, early termination recommendations may be made at the interim analysis review by the Efficacy and Safety Assessment Committee. If the Data and Safety Monitoring Committee provides recommendations for early termination of the study, the principle investigator and group chair will review the recommendations and decide whether to terminate the study early.

12.7.2. Early termination due to adverse events

In JCOG9511 for small-cell lung cancer, 1/77 (1.3%) treatment-related deaths were reported with EP therapy and 3/77 (3.9%) with IP therapy; in JCOG0509, a successor study for the same subject, 1/142 (0.7%) treatment-related deaths were reported with IP therapy and 2/142 (1.4%) with amrubicin plus cisplatin therapy. Using these as reference, we believe that the treatment-related mortality rate should not exceed 3% in this study. Since it is clear that the final point estimate will be at least 3% at the time of 3 treatment-related deaths in either group, immediate entry should be suspended to consider whether or not to withdraw from the study. At this point, the subsequent treatment of the patient being treated will be reviewed. At the time of 3 or fewer treatment-related deaths in each group, each patient will be reported to the Efficacy and Safety Assessment Committee for adjudication. Enrollment will be continued until the results are obtained in principle.

12.7.3. Early termination due to poor enrollment

If the patient enrollment pace is significantly worse than at the time of planning, early termination of the study may be advised by the Data and Safety Monitoring Committee. If early termination recommendations are issued by

the Data and Safety Monitoring Committee due to poor enrollment, the principle investigator and group chair will review the recommendations and decide whether to terminate the study early.

12.7.4. Early termination due to other reasons

12.7.1.～12.7.3. If it is judged difficult to continue the study for other reasons, the research representative physician shall submit a request for early termination of the study to the Efficacy and Safety Assessment Committee. If the Data and Safety Monitoring Committee recommends early termination of the study based on the submitted data, the procedure for early termination of the study will be progressed.

12.8. Procedures after Early termination of the Study

If the Study Chair accepts the recommendations made by the Data and Safety Monitoring Committee based on Section 12.7, he/she will promptly submit a notification to the Data and Safety Monitoring Committee that early termination of the study will be performed.

The Study Chair will submit a termination notification to the Certified Review Board within 10 days of the date they decide to terminate the study early. If the study falls into a specified clinical trials under the Clinical Trials Act, the Study Chair shall submit a termination notification to the Certified Review Board within 10 days of the date on which the study was decided to be prematurely discontinued, as well as submit a specified clinical trials termination notification to the MHLW.

The Study Chair promptly informs the investigator of the decision to terminate the study early in writing, and the investigator who has received a report of early termination of the study will report in writing that the study was prematurely terminated to the institution's manager without delay.

If the study is terminated early, JCOG Data Center will promptly initiate the development of the primary analysis report or final analysis report. The subsequent follow-up period will be 1 year from the final enrollment.

13. Ethical Considerations

13.1. Protection of Human Subjects

All researchers involved in this study will conduct this trial in accordance with the "Helsinki Declaration" [1] and "Clinical Trials Act" (2017 Law No. 16) [2] "Clinical Trials Act Enforcement Regulations" (2018 Ministry of Health, Labour and Welfare Order No. 17) and related notices.

1） http://dl.med.or.jp/dl-med/wma/helsinki2013j.pdf

2） http://www.mhlw.go.jp/stf/seisakunitsuite/bunya/0000163417.html

Prior to commencing this study, the site investigator is required to obtain input from Certified Review Board[※1] regarding the conduct of the study, and to obtain approval from the Administrator of the participating institution, and submit the Implementation Plan [※2] to the Ministry of Health, Labour and Welfare.

※ 1 JCOG study will be submitted to the following Certified Review Board.
National Cancer Center Hospital Certified Review Board (accreditation number CRB3180008)

※ 2 "Implementation Plan" means "Documented plan formatted according to the Form No.1 (Form No.1 of the Ministerial Ordinance) specified in Article 39 of the Clinical Trials Act Enforcement Regulations"

13.2. Informed consent

13.2.1. Explanation to the patient

Prior to patient registration, the investigator or subinvestigator will provide the patient with written informed consent form approved by Certified Review Board and explain the following details verbally.

Descriptions

1） Disease names, Stages, and expected prognosis

2） That this study is a clinical trial and is conducted by JCOG
Name of Certified Review Board and contact information for receiving complaints and inquiries to the committee

3） Design and rationale of the study

4） Protocol treatment content

5） Effects expected by protocol treatment

6） Expected adverse events, complications, and sequelae and how to deal with them
Explanation of the extent and frequency of expected adverse events, including complications, sequelae, and treatment-related deaths, and how to deal with them when they occur. In addition to these explanation, obtain the most recent version of the drug package insert and deliver it to patients (PMDA Prescription Pharmaceutical Information Search http ://www.pmda.go. jp/PmdaSearch/iyakuSearch/)

7） Post-study treatment after end of protocol treatment should also be performed appropriately

8） Cost burden and compensations
Explanation of the cost of treatment, compensation that can be received in the event of a health hazard (equivalent to measures taken in general practice, etc.)

9） Alternative treatment
Explanation of treatments that can be received if not participating in this study

10） Anticipated benefits and possible disadvantages
Explanation of anticipated benefits and possible disadvantages by participating in this study

11） Direct access to the medical records
Explanations on acceptance of site visit audits, such as "direct access to medical records etc. by healthcare professionals at other medical institutions for quality control with permission from the administrator of the participating institution."

12） Refusal of consent and withdrawal of consent
Refusal to consent prior to participation in the study is free, and withdrawal after having given consent is free, thereby not causing undue medical disadvantage.

13） Protecting human rights
Every effort should be made to ensure that personal information, such as names, is kept confidential.

14） Secondary use of data

The possibility of secondary use of data obtained from this study in Japan and overseas (ancillary studies, meta-analyses, etc.) only when approved by either committee in JCOG

15） Method of disclosure of information on the study

The study is registered and published in jRCT※. In addition, the results of clinical studies should also be published in jRCT (∗ Databases (Japan Registry of Clinical Trials) https://jrct.niph.go.jp/ prepared by the MHLW as stipulated in Paragraph 1 in Article 24 of Clinical Trials Act Enforcement Regulations)

16） Freedom of questions

Explanation that investigators, written contact information for consultations on study details, on the Principal Investigator and the Study Coordinator, and freely asking questions about study and treatment

17） Explanation of the use and burden of medicinal products not covered by insurance

18） Central pathological review

19） Central image review for response

20） Sample collection for ancillary studies

13.2.2. Consent

Explain the study, give sufficient time to think, confirm that the patient understood the study well, and ask for participation in the study. If the patient agrees to participate in the study, the written informed consent form in the appendix will be used to obtain the patient's own signature. The site investigator or the subinvestigator confirms that the study consent form contains the name of the physician who provided the explanation and the date of explanation, the name of the patient who gave informed consent, and the date of informed consent.

In addition, when it is not possible to read the documents due to visual impairment, etc., but the details can be understood by verbal explanation, or the documents can not be signed due to limb disorders, etc., but the documents can be read and understood, signatures may be obtained from the proxy author under the consent by the patient. However, the signature of the proxy author should be based on this study's consent, and should be described as "Signing by the proxy author" and "Relationship with the patient" so that the person can be found to be the proxy author.

Two copies of the consent form will be provided, one will be handed over to the patient, and one will be stored by the site coordinator. Original copies will be stored in the medical records or in the archives specified by the participating institution.

13.2.3. Response to inquiries, consultations, etc. after consent

In principle, the investigator or subinvestigator of the relevant patient's participating institution responds to any consultation related to the study by the patient or his/her family after registration. If it is unclear how to respond, respond in consultation with Principal Investigator, Study Coordinator, the Group Secretariat, Group Chair, JCOG Data Center/Operations Office, etc. in accordance with the content of the consultation.

13.2.4. Withdrawal of consent

After obtaining informed consent for participation in the study, consent will be withdrawn if the patient expressed withdrawal of consent.

Withdrawal of consent refers to withdrawal of consent to participate in research and is distinguished from refusal to continue protocol treatment (below (i)). If the withdrawal of consent is expressed, clarify whether (ii) or (iii) below and promptly notify JCOG Data Center. If consent is withdrawn, record it in the medical record as well as (ii) or (iii).

Data Center discontinues subsequent follow-up requests according to the protocol in case of (ii) withdrawal of consent, In the case of (iii) full withdrawal of consent, the data of the patient are removed from the database when it is confirmed that the patient has full withdrawn of consent.

The procedures for discontinuation of the patient's follow-up request and removal of patient data will be specified separately in the procedural manual, and the completion of each task will be reported to Principal Investigator and Study Coordinator.

(i) Patient refusal: Refusal to continue subsequent protocol treatment (follow-up continues).

(ii) Withdrawal of consent: Withdrawal of consent to participate in the study and termination of all subsequent treatment and follow-up in accordance with the study protocol. Research use of data prior to withdrawal of consent is permitted.

(iii) Full withdrawal of consent: Withdrawal of consent to participate in the study and unavailability of all data from the time of patient registration, including information at registration.

In addition, some medical institutions may request that a "withdrawal of consent" form be prepared as a written document. However, in the event of withdrawal of consent, written expressions of willingness are required to increase the psychological barriers to withdrawal of consent (i.e., it is difficult to withdraw consent), and it is considered unwanted from the viewpoint of protecting human subjects. Therefore, in JCOG, written expressions of willingness are not mandatory for withdrawal of consent, verbal withdrawal of consent is valid, and the "withdrawal of consent" form is not prepared. If "withdrawal of consent" form is required by the participating institution, it should be prepared by the institution.

- This should be used in studies conducted in accordance with the Ethical Guidelines for Medical and Health Research Involving Human Subjects.

The procedures for discontinuation of the patient's follow-up request and removal of patient data will be specified separately in the procedural manual, and the completion of each task will be reported to Study Chair and Study Coordinator.

(i) Patient refusal: Refusal to continue subsequent protocol treatment (follow-up continues).

(ii) Withdrawal of consent: Withdrawal of consent to participate in the study and termination of all subsequent treatment and follow-up in accordance with the study protocol. Research use of data prior to withdrawal of consent is permitted.

(iii) Full withdrawal of consent: Withdrawal of consent to participate in the study and unavailability of all data from the time of patient registration, including information at registration.

In addition, some medical institutions may request that a "withdrawal of consent" form be prepared as a written document. However, in the event of withdrawal of consent, written expressions of willingness are required to increase the psychological barriers to withdrawal of consent (i.e., it is difficult to withdraw consent), and it is considered unwanted from the viewpoint of protecting human subjects. Therefore, in JCOG, written expressions of willingness are not mandatory for withdrawal of consent, verbal withdrawal of consent is valid, and the "withdrawal of consent" form is not prepared. If "withdrawal of consent" form is required by the participating institution, it should be prepared by the institution.

13.3. Protection of personal Information and patient identification

JCOG recognizes that information on privacy, such as personal information and medical information, should be protected and handled carefully, based on the principle of respecting the personality of individuals, and has formulated JCOG Privacy Policy, and will take all possible measures to protect privacy. For more information, see JCOG website (http://www.JCOG.jp/).

13.3.1. Policies, legislation, and norms followed by JCOG

In conducting JCOG study, JCOG follows, in principle, JCOG Privacy Policy as well as the following laws and norms depending on the content of the research. If other laws, norms, and policies are applicable, they should be followed.

- Clinical Trials Act (Law No. 16, 2017).
- Act on the Protection of Personal Information (Law No. 57, 2003, Final Amendment: Law No. 65, September 9, 2015).
- Helsinki Declaration (Translation by the Japan Medical Association)
- Ethical Guidelines for Medical and Health Research Involving Human Subjects (No.1 of Notice of the Ministry of Education, Culture, Sports, Science and Technology and the Ministry of Health, Labour and Welfare, 2017).

13.3.2. Use of personal information objective, items to be used, and methods of use

1) Objective of use

In accordance with the basic philosophy "Providing the best treatment to more patients," JCOG uses personal information, etc. of patients for objective of "Identifying patients and conducting surveys not only during treatment but also for a long period after end of treatment in order to obtain the correct results of clinical studies, and appropriately managing the acquired information."

2) Items to be used

Information to identify individuals who will be used by JCOG as minimally require for identification and inquiry of patients is as follows.

Medical record number, date of birth, initials, and registration number, Pathology specimen number

In other words, information that can identify individuals other than those listed above, such as the patient's name, is not informed to Data Center by participating institutions, and if they are falsely informed, they should be destroyed without using the recording medium or stored after performing appropriate processing, such as masking, which is incapable of reading.

3) Method of use

Personal information of patients used by JCOG will be collected by entering the CRFs etc. by researchers at participating institutions and submitting them to Data Center either by JCOG Web Entry System, mailing, or handover as a rule. However, telephone calls will be used only for patient registration where prompt contact is necessary.

In addition, in order to confirm the accuracy of the collected information, inquiries regarding various types of CRFs, including personal information, between Data Center and researchers at medical institutions are limited to either JCOG Web Entry System, mailing, or handover. Only the more anonymous registration number should be used when interacting with e-mail inquiries, and medical record numbers and initials should not be used.

13.3.3. Preparation of records for provision of samples and information, etc. at participating institutions

The investigator at each site will prepare records for this study of enrolled patients. The matters prescribed by the Ordinance of the Ministry of Health, Labour and Welfare (Article 53 of Clinical Trials Act Enforcement Regulations) are as follows.

- Identification of clinical-research subjects
- Items related to medical care and testing for subjects personnel in clinical studies
- Items related to participation in clinical research
- Other matters necessary to conduct clinical research

13.3.4. Source documents used in clinical studies (JCOG trials conducted under Clinical Trials Act)

The source documents for clinical studies used in this study refer to all records used for diagnosis and treatment, including medical records (including worksheets, etc.), laboratory test records, diagnostic images used for diagnosis, pathological diagnosis report, images used for response evaluation, and informed consent documents, of enrolled patients in this study. These should be available for direct access for monitoring (14.1.) and audit (14.2.) conducted by Data Center, Operations Office, Certified Review Board and regulatory persons

13.3.5. Storage of samples and information

Samples and information of enrolled patients on this study shall be stored in accordance with Article 53 of Clinical Trials Act Enforcement Regulations (MHLW ordinance No. 17, 2018). The retention time of records related to this study at participating institutions and the retention time of source documents will be 5 years from the date of completion of the study. It is recommended that the records be stored for as long as possible after the expiration date.

The retention time of data collected in JCOG Data Center will be semi-permanent in view of the possibility of long-term follow-up and secondary use for other studies. In addition, as records for the provision of samples and information, the study protocols and the model informed consent forms will be stored semi-permanently in JCOG Data Center.

13.3.6. Anonymization and control of response tables

In JCOG studies, information that can clearly identify individuals, such as patients' names, is not collected, and individuals are identified using registration numbers and medical record numbers (anonymized). Correspondence tables (not always in the form of a table) of information and registration numbers that can clearly identify individuals by themselves, such as patient names, are positioned as present at each participating institution and are appropriately managed according to the policy of participating institutions so that the identification of enrolled patients can be ensured.

13.3.7. Secondary use of data

Data from this study may be used in Japan or abroad for secondary use (e.g., meta-analysis) only if approved by the relevant committee of JCOG (e.g., Protocol Review Committee, Data and Safety Monitoring Committee). However, when providing data to external organization(e.g., a meta-analysis), data are provided so that it is not possible to identify individuals.

Secondary use of data should be disclosed on JCOG website to ensure opportunities for patients to refuse using data.

13.3.8. Safety management responsibility system

JCOG Data Center establishes a privacy protection control manager and a privacy protection manager and takes various safety control measures to minimize the risks of information leakage when using personal information, etc.

13.3.9. Response to request of disclosure of patient information

In the event that JCOG is asked to disclose privacy-related information, etc., the responder shall, in principle, be a researcher (site investigator, subinvestigator) at the institution of the relevant patient.

13.3.10. Receipt of inquiries about JCOG

General inquiries and complaints about privacy policies will be received by either mail, e-mail, or fax below.

Inquiry liaison: JCOG Data Center Privacy Protective

Postal destination :〒104-0045 Tsukiji 5-1-1, Chuo-ku, Tokyo
Clinical Research Support Office, National Cancer Center Hospital
E-mail :JCOG_privacy@ml.JCOG.jp
FAX :03-3542-3374

13.4. Adherence to the protocol

Researchers participating in this study will adhere to this protocol unless they compromise patient safety and human rights.

13.5. Application to Certified Review Board and Notification of Implementation Plans

When conducting this study, the approval of Certified Review Board and the permission by the administrator of each institution to conduct the study using this protocol and written informed consent from patients must be obtained. Prior to the commencement of the study, the implementation plan ※1 shall be submitted to the Minister of Health, Labour and Welfare, and the test data shall be published to jRCT※2. Principal Investigator is responsible for applying to Certified Review Board, submitting Implementation Plans to the MHLW, and registration in jRCT, and JCOG Operations Office supports these application procedures.

※ 1 Form No.1 of the Ministerial Ordinance stipulated in Article 39, Paragraph 1 of the Ordinance for Clinical Trials Act Enforcement Regulations

※ 2 Databases (Japan Registry of Clinical Trials) prepared by MHLW as specified in Article 24, Paragraph 1 of Clinical Trials Act Enforcement Regulations https://jrct.niph.go.jp/

13.5.1. Procedures for new application

<u>1) Procedures performed by Principal Investigator</u>

<Procedures from initial submission to study initiation>

(i) Review and approval of the study protocol by JCOG Protocol Review Committee (ver.1.0.0).

(ii) Principal Investigator will prepare the following documents for this study and submit these documents to Certified Review Board for review through JCOG Operations Office.

- ➢ New Review Request Form (Unified Form No.2 for Clinical Research)
- ➢ Implementation Plan (Ministerial Ordinance Form No.1)
- ➢ Study plan (this protocol) (including response to "disease or the like")
- ➢ Informed consent form
- ➢ Documents describing summary of drugs, etc. (e.g., package inserts of drugs used as a part of protocol treatment)
- ➢ List of Subinvestigators (Unified Form No.1 for Clinical Research)
- ➢ Conflict of Interest Management Standard (Guidance Form A), Conflict of Interest Management Plan (Guidance Form E) (see 13.X.)
- ➢ Other documents to be submitted when prepared (review documents of JCOG Protocol Review Committee, sample Case Report Forms, and a draft agreement on funding with the marketing authorisation of drugs, etc. or its special associates)

(iii) Modifications such as protocols and informed consent forms are made to review opinions submitted by Certified Review Board as needed.

※ Response to the indications by Certified Review Board: Prepare modified versions with review and approval by the Director of Data Center if modification of the protocol or informed consent forms is needed (ver.1.0.1, ver.1.0.2, ver.1.0.3…). .

(iv) After Certified Review Board approval is obtained, JCOG Operations Office should have Certified Review Board approval date and the approved version number on the cover page of the protocol and informed consent form and appear on JCOG website's Protocol Download page.

(v) Principal Investigator will use Certified Review Board review results notification and the documents submitted in ② to obtain the approval of the administrator of the institution which he/she belongs to, send these documents to the site investigators of all participating institutions described in the Implementation Plan, and request that the administrators of each participating institution permit conduct the research.

(vi) JCOG Operations Office shall apply for jRCT registration under the supervision of Principal Investigator after obtaining the permission by the administrators of all participating institutions described in the Implementation Plan and confirming that the patient registration system is open. "Study progress" in the trial registration will be registered as "being recruited."

(vii) JCOG Operations Office will inform Principal Investigator that jRCT registration has been completed after submission of the registration application. Principal Investigator will output the Implementation Plan from jRCT and submit the Implementation Plan, informed consent form, and Certified Review Board review result notification to the MHLW (Local Health and Welfare Bureau, which is responsible for the location of Certified Review Board; the same below). After submission, Principal Investigator will promptly inform Certified Review Board described in the Implementation Plan. In addition, Principal Investigator shall promptly report the submission of the Implementation Plan to the administrator of the institution to which he/she belongs, and provide this information to the site investigators of all participating institutions listed in the Implementation Plan and JCOG Operations Office.

(viii) After confirming that the notification of the Implementation Plan, etc. to the Local Ministry of Health and Welfare has been accepted and that jRCT's status has been updated from "under registration application" to "registration open," Principal Investigator will inform JCOG Operations Office that it has become "registration open." JCOG Operations Office opens JCOG Data Center's patient registration system and provides trial initiation announcements to study group. The date of jRCT publication is the start date of the study. "】

JCOG Operations Office should be contacted for procedures involving the addition of participating institutions after the initial submission.

"16.X. Medical institutions" changes (adding or replacing participating institutions) correspond to changes in the content of the protocol, then the change procedure shall be performed in accordance with the "13.6.2.Procedures for

when a change in the protocol occurs after the start of the study"

2) Procedures performed by the investigators at each participating institution

Following Certified Review Board approval, the investigator at each participating institution will obtain permission to conduct the research by the administrator of the participating institution using a set of documents received from Principal Investigator (Certified Review Board review results notification and documents submitted to Certified Review Board). The site investigator shall promptly send a copy of the letter of permission of the relevant institution to the Data Center after obtaining the permission by the administrator to conduct the research.

If the study is Specified Clinical Trial under Clinical Trials Act, the site investigator who has been informed that the Implementation Plan has been submitted to the MHLW by Principal Investigator should promptly report that the Implementation Plan has been submitted to the administrator of the affiliated institution.

3) Permission to conduct research at participating institutions

The procedures for obtaining permission for conducting research from the administrator of the affiliated institution shall be in accordance with the regulations of each institution.

When a copy of the institutional approval form is sent to JCOG Data Center, either the site investigator or the site coordinator should send the copy. Original copies of the institutional approval form will be stored by the site coordinator, and copies will be stored by JCOG Data Center.

When a affiliated institution has restrictions on providing personal information, such as medical record numbers, when sending copies of the institutional approval form to JCOG Data Center, the site coordinator shall communicate that personal information cannot be provided, and also send documents, such as the corresponding table of the ID number for registration and the medical record number, which is specified in JCOG privacy policy.

In addition, the informed consent form for patients approved by Certified Review Board may not be modified except for the contact information of the institution or prespecified selection items. A common protocol will be used for all institutions, since no changes in the protocols will be permitted for each institution. If it is necessary to change the content of the protocol and informed consent form, consult with Principal Investigator and Study Coordinator if the administrator of the institution asks for modification of the protocol and the text of the informed consent form in order to make the change as the protocol and informed consent form used by all institutions.

13.5.2. Procedures for changing the study plan after the start of the study

1) Procedures performed by Principal Investigator

If any of the changes of (1), (2), or (3) below occurs in the conduct of this study from the <Procedures from the initial application to the commencement of the study> in "13.6.1. Procedures at the time of new application", Principal Investigator shall hear Certified Review Board's opinions by applying for a change to Certified Review Board.

The procedures for applying for changes to the Certified Review Board are in accordance with "13.6.1. Procedures at the time of new submission" in the procedures from initial submission to the start of the study in ②, ③, and ④, respectively. In the absence of a change in the Implementation Plan, notification to the MHLW is not necessary. In the event that a change in the Implementation Plan occurs, notification to the MHLW is required. If Principal Investigator becomes aware of the planned change in the Implementation Plan, he or she should promptly contact JCOG Operations Office. JCOG Operations Office will assist in the application procedure for change to Certified Review Board and registration in change to jRCT.

（1） When the content of the protocol or informed consent form is changed (when it is amendment or revision in 13.7.1.).

（2） When the protocol or informed consent form is not changed, but the Implementation Plan (registered in jRCT) is changed.

（3） When changing Conflict of Interest Management Standards or Conflict of Interest Management Plans

Because notification of changes in the Implementation Plan (registered in jRCT) to the MHLW except for changes in research progress must be done in advance, Principal Investigator should inform site investigators and JCOG Operations Office of the change including the replacement of the investigator or the subinvestigator at each

participating institution. In addition, inform other investigators as soon as there are any changes that need to be made known.

Principal Investigator shall promptly report to the administrator of the institution to which he/she belongs and provide information to other research investigators when informed by Certified Review Board.

Procedures when it is necessary to notify changes to the Certified Review Board and the MHLW before changes are made.

1. Changes to the Implementation Plan

If changes to any of the above (1), (2), or (3) are made to the Implementation Plan (registered in jRCT) after hearing Certified Review Board's opinions, JCOG Operations Office should enter changes to jRCT under the supervision of Principal Investigator. In addition, Principal Investigator submits the following notification to the MHLW. After the date of coming into force of the predefined changes, the study shall be conducted in accordance with the changes.

- Notification of Change in Implementation Plan (Form No.2 of the Ministerial Ordinance)
- Implementation Plan after the change (output of the change in jRCT)
- Certified Review Board Review Results Notification

※ On institution transitioning to JCOG Collaborating Institution by replacement of participating institution: For institutions where patients were not enrolled prior to transfer to collaborating institution, Principal Investigator should remove it from the participating institutions by notifying changes in the Implementation Plan.

Procedures when it is necessary to notify a change to the Certified Review Board and the MHLW after the change***

1. Change in progress

(2) Among the changes in the Implementation Plan (registered in jRCT), the change in "3. Issues related to checking the implementation of Specified Clinical Trials (2) Specified Clinical Trials progress" shall be made without delay after the change. Under the supervision of Principal Investigator, JCOG Operations Office shall change "Research Progress" in jRCT. Subsequently, Principal Investigator will promptly apply for a change to Certified Review Board. After obtaining approval from Certified Review Board, Principal Investigator will submit the following notification to the MHLW.

- Notification of Change in Implementation Plan (Form No.2 of the Ministerial Ordinance)
- Implementation Plan after the change (the content of the change in jRCT was outputted)
- Certified Review Board Review Results Notification].

2. Changes in Implementation Plan after registration of the first patient

At the time of the initial submission, the "date of registration of the first patient" in the Implementation Plan is provided in a blank space. The Implementation Plan should be changed without delay after registration in the first patient. In this instance, JCOG Operations Office enters and registers the "First Patient Registration Date" in jRCT under the supervision of Principal Investigator. Subsequently, Principal Investigator will promptly apply for a change to Certified Review Board.

- Notification of Change in Implementation Plan (Form No.2 of the Ministerial Ordinance)
- Implementation Plan after the change (output of the change in jRCT)
- Certified Review Board Review Results Notification]

3. Minor changes specified by MHLW ordinance (application for change to Certified Review Board is not required)

If the following minor changes are made to Implementation Planning and the registration of jRCT, Principal Investigator will not need to hear the opinions of Certified Review Board and will notify Certified Review Board of the changes within 10 days of the date of the change. In addition, a notification (Form No.3) shall be submitted to the MHLW.

Scope of minor changes to the Implementation Plan of Article 42 of Clinical Trials Act Enforcement Regulations

- A change in the name of a person engaged in Specified Clinical Trials that does not involve a replacement of the person engaged in Specified Clinical Trials.
- Changes due to a change in the name or address number of the area

2) Procedures performed by the investigators at each participating institution

If any changes occur in the part of the protocol that corresponds to the institution to which he/she belongs, (4) Items related to investigators in multi-institutional studies, inform Principal Investigator and JCOG Operations Office of the changes to be made prior to the changes. Depending on the content of the change, the investigator shall confirm the confirmation of institutional requirements, prepare documents for conflicts of interest and submits to Certified Review Board such as lists of subinvestigators, and inform Principal Investigator and JCOG Operations Office.

The content of the most recent Implementation Plan for the institution to which they belong (the same as the content of jRCT registration) should be checked on jRCT website (https://jrct.niph.go.jp/).

13.5.3. Review and approval of study progress and study continuation (periodic reports)

Principal Investigator will report periodic reports on the progress of the study, the occurrence of adverse events, and conflict of interest management (see 13.X.1.⑱) to the administrators of their institutions and report them to Certified Review Board. Within two months after the expiration of each year from the date of submission of the Implementation Plan to the Minister of Health, Labour and Welfare.

When the report is made to Certified Review Board, Principal Investigator shall promptly inform the investigators of other participating institutions of the fact. The investigator who receives the information shall promptly report the details of the information to the administrator of the institution to which he/she belongs.

If the study falls under Specified Clinical Trials above Clinical Trials Act, Principal Investigator will report to the MHLW regarding the implementation status of Specified Clinical Trials. Periodic reports to Certified Review Board shall be made within 1 month of the date on which the results on the appropriateness of continuation of the relevant Specified Clinical Trials are obtained. The report shall be made by submitting the attached Form 3[※] to the Minister of Health, Labour and Welfare.

※ Enforcement of Clinical Trials Act Enforcement Regulations, etc. (Notification No. 0228-1 of the Sector of Economics, Ministry of Health, Labour and Welfare, Notification No. 0228-1 of the Notification No. 1 of the Evaluation and Development Division, Health Policy Bureau, Ministry of Health, Labour and Welfare, dated February 28, 2018)

13.6. Protocol revision/amendment

13.6.1. Categorization of protocol changes and procedures for changes

For a change in the protocol, the Protocol Revision Application must be submitted to Data and Safety Monitoring Committee Office prior to submission to Certified Review Board [in the case of Specified Clinical Trials, add the following: "and report to the MHLW"] (see 13.6.2).

JCOG deals with the changes in the content of the protocol after approval by the Protocol Review Committee, divided into amendment and revision. Data and Safety Monitoring Committee Office will distinguish between amendment and revision, so all of applications are submitted as revision. If it is classified as an amendment by the Secretary-General of Data and Safety Monitoring Committee, it is reviewed by Data and Safety Monitoring Committee. If it is classified as a revision, Secretary-General of Data and Safety Monitoring Committee will issue a verification form and will not be reviewed by Data and Safety Monitoring Committee. We also distinguish the addition of supplementary explanations that do not fall into a change in protocol content as "Memorandum". Definitions and handling are as follows:

When the protocol or informed consent form is revised or amended, Principal Investigator will promptly distribute the most recent protocol or informed consent form to those involved in the study. Individuals involved in the study should always conduct the study in accordance with the most recent protocol since the dates of entry into force of the amendment/revision.

1) Amendment

Partial protocol change which meets one or more of the followings: i) Potential to increase the risk of patients enrolled in the study, ii) Having substantial effects on primary endpoint of the study, iii) having essential effects on the study's implementation structure.

The amended version of the protocol and informed consent form version numbers are shown as in 2.0.0, 3.0.0, and 4.0.0….

Approval by the Group Chair and the Data Center Director must be obtained prior to submission to Data and Safety Monitoring Committee.

When classified as "amendment" by the Secretary-General of Data and Safety Monitoring Committee, review of changes by Data and Safety Monitoring Committee is performed prior to Certified Review Board review. After the protocol amendment has been approved by Data and Safety Monitoring Committee, an application for a change in the protocol will be submitted to Certified Review Board through JCOG Operations Office (at this time the version number is ver. 2.0.0, 3.0.0, 4.0.0…). When the protocol was changed based on the review opinions of Certified Review Board, the version number is ver. 2.0.1, ver.2.0.2… If the protocol is changed by review of Certified Review Board, the change will be reported to Data and Safety Monitoring Committee, but in principle, the change will not be reexamined by Data and Safety Monitoring Committee. When a protocol change is approved by Certified Review Board, the protocol cover page should include the date of approval by Data and Safety Monitoring Committee and Certified Review Board [If Specified Clinical Trials, the following is added: "and a notification of the change in the Implementation Plan to the Regional Bureau of Health and Welfare should be made"].

Following Certified Review Board approval, permission for the contents of the amendment by the administrator of each institution shall be obtained. If permission is obtained, the site coordinator of each institution will send copies of the permission notice by the administrator of each institution to the Data Center. After the permission of the administrator of all institutions is obtained, the protocol changes will come into effect (during this time, the patient registration will not be suspended unless there is a special need). The actual date of entry into force will be announced by JCOG Operations Office. Researchers in all participating institutions conduct the study according to revisions approved by Certified Review Board since the date of entry into force.

Treatment and assessment of enrolled patients will be performed according to the pre-change version protocol until entry into force. Protocol deviations to enhance patient safety during treatment will be permitted if pre-change protocols, such as inadequate treatment modification criteria, threaten patient safety. If protocol deviations occur, they should be listed in the monitoring report.

2) Revision

Protocol change which meets all of the followings: i) does not have an increased risk for patients enrolled in the study; ii) does not have a substantial effect on primary endpoint of the study; iii) does not have an inherent effect on the system in which the study is conducted. Includes changes in protocols due to mistakes or changes in institution-specific information, changes in institution-specific information without changes in protocols (changes in Implementation Plans and jRCT registrations), and changes in conflicts of interest at individual institutions. In principle, suspension of patient registration is not performed in case of revision.

The revised version of the Protocol and Informed Consent Form version numbers are shown as in 1.1.0, 1.2.0, and 1.3.0….

Approval by the Group Chair and the Data Center Director must be obtained prior to submission to Data and Safety Monitoring Committee.

If classified as "Revision" by the Secretary-General of Data and Safety Monitoring Committee, the Secretary-General of Data and Safety Monitoring Committee issued a verification form, Data and Safety Monitoring Committee did not review the changes. The Principal Investigator submit an application for a change in the protocol to Certified Review Board through JCOG Operations Office (at this time, the version number is ver.1.1.0, 1.2.0, 1.3.0…). If the protocol was changed based on the indication by the Certified Review Board, version number shall be as ver.1.1.1, ver.1.1.2… When a protocol change is approved by Certified Review Board, the date of approval by the Certified Review Board should be entered on the protocol cover page. [If Specified Clinical Trials, the followings are added: "and a notification of the change in the Implementation Plan to the Regional Bureau of Health

and Welfare should be made"]

The date of entry into force of the protocol change will be 2 weeks after Certified Review Board approval date unless otherwise specified. [in the case of a Specified Clinical Trials, the followings are added: "The effective date shall be the date after the notification of change of the Implementation Plan to the Regional Bureau of Health and Welfare"]

The actual date of entry into force will be announced by JCOG Operations Office. Researchers in all participating institutions conduct the study according to revisions approved by Certified Review Board since the date of entry into force.

At that time, the site investigator should obtain permission by the administrator of each institution after the date of approval of Certified Review Board and before the date of entry into force. In this case, the protocol revision may be permitted with a report to the administrator of the institution. However, the procedures for obtaining permission from the administrator shall be in accordance with the regulations of each institution. For protocol revisions, confirm with the institution prior to initiation of the study to ensure that permission is obtained within the aforementioned time periods, and contact JCOG Operations Office if this is difficult. Reports to the administrator and approval form by the administrator at each institution need not be sent to the Data Center, but the original copy will be retained by the site coordinator as it will be checked during site visit audits.

Treatment and assessment of enrolled patients will be performed according to the pre-change version protocol until entry into force. Protocol deviations to enhance patient safety during treatment will be permitted if pre-change protocols, such as inadequate treatment modification criteria, threaten patient safety. If protocol deviations occur, they should be listed in the monitoring report.

3) Memorandum

Supplementary description of the protocol distributed from Principal Investigator/Study Coordinator to study personnel in objective, such as reduction of interpretive variation in sentences, and special precautions, rather than change of protocol content. Any form is used.

Approval by Group Chair and the Director of the Data Center is needed prior to distribution. Reporting to Data and Safety Monitoring Committee before distribution or immediately after distribution is required.

It is not necessary to include the protocol on the cover page.

13.6.2. Patient explanation and re-consent at the time of protocol amendment/revision

In the event of a change in the content of the study, the investigator or the subinvestigator shall provide appropriate explanation to the enrolled patients (regarding protocols based on revision, treatment, follow-up, etc.). In addition, if Certified Review Board comments that re-consent of enrolled patients in writing is required, informed consent should be obtained in writing.

13.7. Conflicts of Interest (COIs) involved in this study

13.7.1. COI management involved in this study

The COIs involved in this study will be managed according to the "Guidance for Conflict of Interest Management in Clinical Trials Act (Notification No.1130-17 of the PMSB dated November 30, 2018) ※ (Guidance) of the Division, Research and Development, Ministry of Health, Labour and Welfare, in accordance with the following:

※ http://www.mhlw.go.jp/stf/seisakunitsuite/bunya/0000163417.html

The format used for COI control should be the latest version of the guidance.

- Conflict of Interest Management Standards: Form A
- Reports of related companies, etc.: Form B
- Investigator Conflict of Interest Self-Report Form: Form C
- Conflict of Interest Confirmation Report: Form D
- Conflict of Interest Management Plan; Form E

1) Procedures for New Application

Conflict of Interest Management Standards

(i) All JCOG trials according to Clinical Trials Act will employ Conflict of Interest Management Standards (Form A) in accordance with guidance.

Request for Confirmation of Conflicts of Interest

(ii) At the time of initiation of the primary review of the protocol, JCOG Operations Office shall identify companies, etc. related to this study that require conflict of interest management based on information on drugs and medical devices specified in the Protocol Treatment (see 6.1.), enter the relationship with the company in Form B, and ask Principal Investigator to confirm the accuracy of the entry.

(iii) After checking the entries in Form B received from JCOG Operations Office, Principal Investigator will send a format to the site investigator and site coordinator at each participating institution and ask to confirm the entries in Form B, create Form C, and create Form E. In addition, a form shall be sent from Principal Investigator to those who correspond to the "person responsible for statistical analysis" and the "administrator other than Principal Investigator and Investigators" of the Implementation Plan, and they shall be asked to prepare Forms C and E.

Confirmation of Conflicts of Interest (participating institutions)

(iv) The site investigator or site coordinator at each participating institution will register the investigators, subinvestigators, and research associates involved in the study in JCOG Web Entry System. Investigators and subinvestigators registered in JCOG Web Entry System will be the reporters of conflicts of interest in the study.

(v) The site coordinator will request the investigator of the study to confirm the description of Form B received from Principal Investigator, create Form C, and create Form E. The investigator will then summarize conflict of interest management within the study site.

(vi) The investigator checks the forms received from Principal Investigator for any relevant items in Q2 through Q5 of Form B and notifies Principal Investigator through JCOG Operations Office within a week if any.

(vii) In the column of Form C [Persons requiring a Form C self-declaration of conflict of interest (Form C)] ※, the site investigator inputs the information of the investigator and subinvestigator registered in JCOG Web Entry System, and asks subinvestigator to prepare Form C.

※ The information entered in Form C is automatically entered in Form E. This entry should be consistent with the physician's information contained in the "List of Subinvestigators" (Clinical Trials Act Uniform Form No.1) submitted to Certified Review Board with Form E, and physicians who do not agree may not be involved in this study. Therefore, when entering the information in the ※ column for [those who require a Conflict of Interest Self-Reporting Form (Form C)], the investigator or subinvestigator list should be downloaded in JCOG Web Entry System and the physician listed in the Study Subinvestigator list should be entered without missing the person who will be the investigator and the subinvestigator in the study. If the information in the downloaded "Study Subinvestigator List" is not consistent with the physician involved in the study, the registered information should be updated in JCOG Web Entry System, and the "Study Subinvestigator List" with the most recent information reflected should be downloaded and used.

(viii) The investigator and the subinvestigator shall complete the necessary information regarding the relationship with the company, etc. described in advance in Form C, and submit Form C to the Conflict of Interest Confirmation Department of the institution to which he/she belongs. In doing so, the investigator also submits Form A.

(ix) The investigator receives the results of confirmation of conflicts of interest (Form D) of the investigator and the study subinvestigator from the institution to which he/she belongs.

(x) The investigator will confirm the content of Form A, Form B, and Form D of the investigator and all study subinvestigators to create Form E. The investigator will notify Principal Investigator through JCOG Operations Office of Form E and the Study Subinvestigator List downloaded in Form 7.

Confirmation of Conflicts of Interest (outside participating institutions)

(xi) The person responsible for statistical analysis and the person overseeing a study other than Principal Investigator or investigator shall complete, in the form received from Principal Investigator, the necessary information regarding the relationship with the company, etc. described in advance in Form C, and submit Form A and Form C to the Conflict of Interest Confirmation Department of the Affiliated Institution.

(xii) The person responsible for statistical analysis and the person overseeing studies other than Principal Investigator or investigator shall receive confirmation results (Form D) from the institution.

(xiii) The person responsible for statistical analysis and the person overseeing studies other than Principal Investigator and investigator shall confirm the content of Form A and Form D, prepare Form E, and notify Form E to Principal Investigator through JCOG Operations Office.

Description of Conflicts of Interest in the protocol and informed consent form

(xiv) Principal Investigator and JCOG Operations Office will review Form A and Form E received from participating institutions and, as appropriate, will accurately describe the conflicts of interest (study COIs) between the study and the drug marketing authorisation holder, etc. in the protocol and informed consent form. Conflicts of interest (personal COI) between the reporters of conflicts of interest in this study and the marketing authorisation of drugs, etc. (personal COI) can change over time, so the personal COI is not described in the protocol and informed consent form, and the information is updated on JCOG website as needed.

Certified Review Board review

(xv) Principal Investigator compiles and submits the Form E and Study Subinvestigator List of all institutions to Certified Review Board for review.

2) Procedures for new involvement with companies after the start of the study

(xvi) When a new involvement with companies (research COI) occurs in the study after the start of the study

a. In the event of any change in the enterprises involved in this study, etc. to be described in Q1 of Form B, repeat the procedures ② to ⑭. However, the site investigator and site coordinator roles in ④, ⑤ shall be assumed by the investigator at each institution. If a new study COI needs to be added to the protocol and informed consent form, a protocol revision (see 13.X.X.) should be submitted to Certified Review Board for review.

b. If a change occurs from Q2 to Q5 in Form B, the investigator at the participating institution affected by the change will change the appropriate description in Form B and update Form E and send it to Principal Investigator and JCOG Operations Office. A Principal Investigator who has received a Form E will revise the protocol as needed (see 13.X.X.) and submitted to Certified Review Board for review.

(xvii) In the event that a conflict of interest reporter is newly involved with a company (personal COI) after the start of the study

Conflict of interest reporters repeat procedures from ⑧ to ⑩ or from ⑪ to ⑬. However, if there is no change in Form E, these procedures will be performed at each institution but will not be sent to Principal Investigator and JCOG Operations Office. Principal Investigator will submit Post-change Form E sent from the site investigator to Certified Review Board for review. Personal COIs are also disclosed on JCOG website.

3) Procedures for periodic reporting

(xviii) Principal Investigator checks for changes in study COI and personal COI annually at the timing of periodic reporting and reports to Certified Review Board.

13.7.2. COI with companies involved in this study(study COI)

There are no conflicts of interest to disclose about relationship between the company manufacturing and marketing drugs specified as a part of protocol treatments in this study (See 6.1).

13.7.3. COIs of JCOG Committee members and JCOG Data Center/Operations Office staff

COIs of the committee members of JCOG committees, the staffs of the office of committees, and JCOG Data Center/Operations Office staff involved in the study will be managed by JCOG Conflict of Interest Committee in accordance with Clinical Trials Act control standards.

13.8. Compensation

In order to comply with Clinical Trials Act, this study must take necessary measures such as sing up for insurance and ensuring a system to provide medical care in order to compensate for the health damage caused by the study

and provide medical care.

Therefore, regarding the health damage caused by participating in this study, appropriate treatment according to the condition is provided similarly to the usual insurance medical care. In addition, this study will be covered by clinical study insurance, and the following will be compensated based on insurance conditions, and this will be explained to patients and understood.

13.9. Intellectual property

The results, data, and intellectual property rights obtained from this study are attributed to the followings: National Cancer Center, Principal Investigator, Study Coordinator, and Group Chair. Specific procedures and allocation must be determined through consultation among four parties. Whether the intellectual property related to Principal Investigator, Study Coordinator, Group Chair will belong to the individuals or the affiliated institution will be determined according to the agreements of the affiliated institution.

13.10. Disclosure of information on this study

Summary, progress, and main results of this study will be published on JCOG website (www.jcog.jp) and on jRCT (https://jrct.niph.go.jp/).

14. Monitoring and audit

14.1. Periodic monitoring

In this study, monitoring is performed in order to ensure that the study is conducted safely and in accordance with this protocol and that data are collected accurately. Periodic monitoring should be performed twice a year in principle, using the entered data on the CRFs collected in the Data Center. Specific procedures for periodic monitoring are provided separately in the Monitoring Plan.

The Data Center submits a "Monitoring Report" summarizing the results of central monitoring to Principal Investigator, Study Coordinator and investigators. Together, they are submitted to Group Chair, Data and Safety Monitoring Committee, and JCOG Chair.

The Monitoring Report is a material for periodic reports to be made every year from the date of submission of the Implementation Plan.

14.1.1. Monitoring items

① Registration status: number of registration - cumulative/by month, by arm/site
② Eligibility: ineligibles/potentially ineligible cases: by arm/site
③ Pre-treatment baseline factors: by arm
④ On/off-treatment, reason for treatment termination: arm/site
⑤ Protocol deviation: arm/site
⑥ Serious Adverse Events: arm/site
⑦ Adverse reaction/event: arm
⑧ Overall survival, progression-free survival (or relapse-free survival, etc.): all registered patients
⑨ Other issues related to study progress and safety (studies in accordance with Clinical Trials Act: status of occurrence of non-compliance and subsequent response, number of subjects for compensation, number of disease or the like reports in accordance with Article 13 of Clinical Trials Act)

14.1.2. Eligibility (Eligible/Ineligible)

For all registered patients, eligibility will be classified according to the following definitions as either: In monitoring, Data Center lists potentially ineligible cases in the "Evaluation of Eligibility" section of the monitoring report, and after review by Study Coordinator, confirms them to be either 1), 2), 9), or 99) with Group Chair approval prior to performing primary analysis.

Only 1) eligible shall be "eligible case", 2) post hoc ineligible,9) de facto ineligible and 99) violation of registration shall be "ineligible case". This is a category established from the perspective of analysis set setting.

In the study in accordance with Clinical Trials Act, "99) violation of registration" will be regarded as "major non-compliance" in Clinical Trials Act, and Principal Investigator will promptly report the situation to Certified Review Board as soon as possible. See 14.3. for management of non-compliance.

9) de facto ineligibles corresponds to "non-compliance" on Clinical Trials Act and is reported to the administrator of participating medical organizations with the submission of the monitoring report on which they were described (twice a year).

2) Post hoc ineligibles is not treated as "non-compliance" on Clinical Trials Act because it does not correspond to non-compliance with the study protocol, as discussed below.

1) Eligible

All information generated prior to registration meets all o the Patients Selection Criteria according to the methods and criteria specified in the study protocol.

2) Post hoc ineligible

The information generated after registration does not meet either Patients Selection Criteria, or the information generated prior to registration does not meet either Patients Selection Criteria by methods or criteria other than those specified in the protocol.

Examples)

(i) In the study for Stage II-III, bone scintigraphy performed immediately after registration revealed bone

metastases, and the patient was diagnosed as Stage IV. The protocol treatment was terminated.

(ii) In the study for early gastric cancer, bloody stools is seen after registration, and colonoscopy revealed advanced colorectal cancer (synchronous double cancer). Colectomy was performed after termination of the protocol treatment.

(iii) In the study for gastric cancer (adenocarcinoma), the institution's pathological diagnosis was changed to malignant lymphoma after registration.

9) De facto ineligible

Information generated prior to registration according to protocol-specified methods (performed in all cases) and criteria does not meet either Patients Selection Criteria. This includes cases where it is determined after registration that the information that occurred before registration had been incorrect.

Example: When the supervising physician reviews the CT images performed before registration as specified, there is obvious liver metastasis (if it is a mistake by the attending physician and it is considered that there is no future).

99) Violation of registration

Deliberately (falsely) enroll while knowing that Patients Selection Criteria is not met. Corresponds to a misconduct and treats it as a serious problem.

14.1.3. Protocol Deviations/Violations

Protocol deviations are defined as those in which treatment, such as drug administration, radiotherapy, or surgical resection, as well as laboratory tests and evaluation of toxicity and efficacy, etc. were not performed according to the protocols.

In monitoring, deviations that exceed a certain acceptable range limit for each study decided by the Data Center and Principal Investigator/Study Coordinator prior to or after the initiation of the study are listed in the monitoring report as "possible deviations" and are classified into one of the following categories after consideration by Study Coordinator and study groups: Except for those described in the protocol and monitoring report, the acceptable deviations agreed between the Data Center and Principal Investigator/Study Coordinator may be changed through periodic monitoring during the study, so they should be described in the supplemental material rather than in the text of the Monitoring Plan, and the Monitoring Plan should be described as "defining the acceptable ranges separately".

1) Violation

Any deviation from the protocol that is clinically inappropriate and caused by the treating physician/institution and that meets two or more following criteria shall be classified as a violation. [When conducted in accordance with Clinical Trials Act, the following shall be added. [In the study according to Clinical Trials Act, the violation shall be treated as "major non-compliance" and Principal Investigator should report the situation to Certified Review Board as soon as possible].

① Have a substantial impact on the assessment of study endpoints

② Intentional or systematic

③ Dangerous or remarkable deviation

For "violations", in principle, the content of each violation should be described in a paper when publishing.

2) Deviation

Deviations that do not fall into 1) violation or 3) acceptable deviation. If same kind of deviations are frequent, they should preferably be included in the publication of the article. They are classified as either of the following at the time of monitoring report review:

Because deviations correspond to "non-compliance" in Clinical Trials Act, they are reported (biannually) to the administrators of the institution with the submission of the monitoring report. Not to be "major non-compliance".

(i) Deviations - Undesirable and to be reduced

(ii) Deviations (unavoidable) - things that are not proactively reducing (e.g. delay by the New Year period, equipment breakdown, etc.)

(iii) Deviations (clinically relevant) - Those in which the decision of the treating physician/institution are positively affirmed (if a similar situation again arises similar deviations are considered desirable).

※ Deviations do not always mean that the treating physician at the institution is problematic. Since patient safety is a primary priority in clinical trials, deviations should rather be made by the medical judgment of the treating physician if the condition of the individual patient are considered to be dangerous when following the protocols. If the deviation is judged to be clinically relevant for the safety of the patient, it is recorded as ③ Deviation (clinically relevant). Clinically relevant deviations in a small number of patients are not required to be particularly problematic; however, protocol revision should be considered when multiple deviations occur because protocol specification is likely to be inadequate. However, deviations that are not intended to be safety (e.g., increased doses of anticancer drugs in the hope of increasing efficacy, shortened treatment periods not specified in the protocol) are not considered clinically relevant deviations.

3) Acceptable deviation

Deviations from protocols within acceptable range agreed by the entire JCOG, study groups, or Study Chair/Study Coordinator and Data Center, pre- or post-study initiation, on a trial-by-trial basis. When conducted according to Clinical Trials Act, the following are added. Not considered to be "non-compliance" in Clinical Trials Act. Deviations within the pre-specified acceptable ranges are not included in the monitoring report.

14.2. Site visit audits

In this study, site visit audits will be conducted to ensure the reliability of clinical research and the reliability of data and information collected by clinical research from the perspective of protecting human subjects in clinical research.

Site visit audits are conducted by auditors appointed by Principal Investigator by visiting a medical institution to confirm the approval documents of the medical institution, check the list of subinvestigators in the research institution, confirm the informed consent documents, and verify CRF entry data with medical records (direct access to source documents). Specific procedures for site visit audits are provided separately in the operating procedures.

The auditor shall report the audit report summarizing the audit results to Principal Investigator/Study Coordinator, site investigators. Together, the report will be submitted to Group Chair, Director of JCOG Data Center, Director of JCOG Operations Office, and JCOG Chair. Reports should also be submitted to the site investigators of the relevant groups and JCOG Executive Committee as appropriate.

14.2.1. Items to be audited

In the site visit audits, the following items are checked by direct access to source documents:

<Confirmation Items by Study>

- Approval documents (including initial approval forms, revision approval forms, and annual report approvals/reports) from administrators of medical institutions
- Management status of the protocol
- Contents of explanatory documents and informed consent forms

<Confirmation Items by Patient>

- Patient consent (presence or absence of consent form, signature, and date of consent)
- Implementation of pre-registration mandatory tests, eligibility for registration (inclusion criteria and exclusion criteria)
- Accuracy of reported data
  - Pre-treatment evaluation, course of treatment (protocol treatment)
  - Various test results (including diagnostic imaging reports and pathology reports)
  - Accuracy of test date, response evaluation, adverse event, survival or death information, and other reported data

<Other items>

- Presence or absence of study misconduct (possibility of false reporting, fabrication, or falsification)
- Improvement status of the items pointed out in the previous audit

14.2.2. Reporting of major non-compliance found in audits to Certified Review Board

Principal Investigator/Study Coordinator should report to Certified Review Board immediately when finding possible "major non-compliance (See 14.3.2.)" as a result of site visit audits.

14.3. Management of non-compliance

14.3.1. Non-compliance.

Non-compliance in Clinical Trials Act refers to the condition in which clinical research is not compliant with Clinical Trials Act Enforcement Regulations or study protocols. In the Clinical Trials Act Enforcement Regulations, etc. (February 28, 2018), non-compliance with regulations, study protocols, operation procedures, etc., and fabrication of falsification of research data, etc. are listed as examples.

If the site investigator knows that there is non-compliance, the site investigator should report to the administrator of the medical institution and inform Principal Investigator/Study Coordinator.

If site investigator find non-compliance (regardless of major non-compliance or not) prior to the implementation of central monitoring or site visit audits, the site investigator will promptly report to Principal Investigator/Study Coordinator and JCOG Data Center.

"14.1.3.2) Deviations" correspond to "non-compliance" in Clinical Trials Act. As described in 14.1.3.2), these deviations are reported by submitting monitoring reports or their excerpts or summaries issued twice a year to the administrators of the medical institution.

Changes in protocols and implementation plans associated with investigator transfer require Certified Review Board review and notification of implementation plans to the MHLW, therefore, require a certain time to complete the sequence of procedures. In addition, it is often difficult to complete the change procedure before the transfer because the transfer may not be open until just before the transfer. Therefore, even if the site investigator is absent for a certain period of time due to the transfer, the study does not fall into "non-compliance" in this study if the research management system is maintained by the subinvestigator and the medical care system of the enrolled patients who are surviving is ensured.

14.3.2. Major non-compliance

"Major non-compliance" is that affect the human rights and safety of subjects of clinical research, the study progress and the reliability of study results. Examples of "major non-compliance" in JCOG study are provided in. If major non-compliance is likely, Principal Investigator/Study Coordinator will report the situation to Certified Review Board immediately.

1) Major non-compliance with respect to eligibility

Violation of registration

- Enrolled intentionally (falsely) while knowing that eligibility criteria was not met
- Patient enrollment was performed without necessary informed consent, and protocol treatment was performed
- The source documents for the determination of eligibility cannot be identified (including the loss of the consent form).

2) Protocol violation

Violations that affect the increased risk of enrolled patients or that affect the reliability of the study results

- Significant violation in inclusion criteria or exclusion criteria
- Violation threatening patient safety in off-treatment criteria
- Serious violation of prohibited concomitant drug, prohibited concomitant treatment, etc.
- e.g. intentional or systematic non-compliance with protocol regulations

3) Other major non-compliance

- Study was conducted prior to Certified Review Board approval or prior to approval of site administrator
- The study was continued without providing information to the enrolled patient that could affect the willingness to continue to participate the study.
- Those judged to be research misconduct (fabrication or falsification of data, etc.)

- Any leakage of personal information or violation of human rights that may have a significant impact on the enrolled patient.

15. Special Instructions
15.1 Central pathology diagnosis and related matters
15.1.1 Central pathological diagnosis

In this study, the pathological tissues of enrolled patients will be collected and the central pathological diagnosis will be determined after the fact. Since the central pathological diagnosis is not performed in real time for each individual patient enrollment, the main analysis target and the decision on treatment strategy will be based solely on the pathological diagnosis at the institution. The details of the central pathological diagnosis procedure will be specified separately in the Central Pathological Diagnosis Procedure Manual.

Timing: Once a year throughout the study period.

Subjects: All registered patients

Methods: Pathological specimens (or duplicates made from the same paraffin block) used for eligibility criteria determination at the registered facilities will be collected, and after necessary staining (chromogranin A, synaptophysin) is added, pathological eligibility will be re-determined by two or more pathology judges (16.8 Pathology judges) appointed by the group representative. (16.8 Central Pathological Review Comittie).

Management of the collected specimens: The Hepatobiliary and Pancreatic Group Study Office will be responsible for the management of the collected specimens.

Staining: If additional staining is required, it will be performed by the Central Pathology Coordinator (16.7).

Notification of the central judgment to each facility:

After the results of the central pathological diagnosis are fixed, the research secretariat will notify the results of the judgment to the registered facility (facility coordinator) of each patient. At that time, the rationale for the decision should be attached in writing. If there is a difference between the institutional diagnosis and the central diagnosis, the facility principal investigator/facility coordinator reports the decision results to the facility pathologist and discusses it with the facility pathologist, and carefully decides on the final pathological diagnosis at the facility (whether to change the diagnosis or not) and what to do if the patient is under treatment (whether to change the treatment or not).

15.1.2 Providing information to the institutional pathologist

In view of the high level of difficulty in NEC pathological diagnosis and the unique nature of this study, which covers multiple target organs, the following information will be provided to institutional pathologists.

(1) Pre-registration pathological diagnosis consultation

Since it is anticipated that there will be cases of confusion in diagnosis at facilities, pre-enrollment pathology consultation will be available as needed, with the pathology judgment committee members of this study serving as consultants. Details of the consultation operation are described in the Central Pathology Procedures.

(2) Holding of pathology-related meetings

In this study, the research secretariat and the pathology secretariat first held a pathology-related meeting (attended by pathologists from participating institutions) on February 11, 2014, during which a lecture was given to form a consensus on diagnostic criteria according to the WHO 2010 classification. The slides used at the meeting were revised as necessary based on the discussion at the meeting and posted on the JCOG website for reference by pathologists at each facility.

15.1.3 Response to cases in which pathological diagnosis was performed at a facility other than the home facility

In any of the following cases, be sure to ask a pathologist at your own institution to make the diagnosis, and confirm that the diagnosis at your institution is also NEC before enrolling in this study.

(1) When borrowing only stained tissue specimens from a previous physician for registration

Register after obtaining permission from the attending physician to the previous physician to submit the borrowed tissue specimen to JCOG 1213 for central diagnosis (loan again) and to borrow the specimen for a long period of time (up to about 1 year).

(2) If you have received virtual slides of "all tissue specimens for which a pathological diagnosis has been made" from

your previous doctor

The patient will be registered after obtaining permission from the attending physician of the previous physician to submit the virtual slides provided by the previous physician for the central diagnosis of this study.

(3) If you have received a "borrowed" pre-stained tissue specimen and an "offered" unstained preparative from your previous physician

The borrowed stained tissue specimens from the previous physician can be promptly reviewed at the patient's own institution, but the provided unstained preparations require time for pathological diagnosis after immunostaining at the patient's own institution, which may cause a time lag. There have been cases in which patients were enrolled in the study based only on the results of pathological review of borrowed tissue specimens at their own institutions, and later the results of staining and pathological diagnosis of undyed preparations at their own institutions overturned the pathological diagnosis (diagnosis of a different disease was made), resulting in post-hoc ineligible cases. Therefore, we will consider the timing of registration after sufficient consultation with pathologists at our own institution to avoid the occurrence of posterior ineligible cases as much as possible. However, if there is enough time, it is preferable to enroll unstained preparations into the study after staining and pathological diagnosis is obtained at your own institution.

15.2. JCOG BioBank Japan (BBJ) Biorepository

This study will participate in the banking of blood samples (DNA/plasma) in JCOG BBJ Biorepository based on a common protocol for all JCOG studies (hereafter referred to as common banking).

Subjects:

Among patients who agreed to participate in this study, patients whose consent to shared banking was obtained.

Sample:

1) Whole blood

Blood sampling is performed before the start of the protocol treatment in this study in principle. However, blood sampling after initiation of protocol treatment is allowed. Blood samples of 7 mL×2 (total 14 mL) of venous blood are collected using a blood collection tube (for blood counting) with a EDTA Na dedicated to the shared banking of JCOG-BBJ Biorepository, and stored at 4℃ at the respective institutions until they are handed over to the sample transport/processing company (See "JCOG-Biobank Japan Biorepository protocol" for details).

2) Pathological specimens

Archived pathological tissues in daily clinical practice such as surgery, biopsy and laboratory tests can also be used in future translational researches, but the type of pathology specimen, preparation method and tissue quantity required by the studies are varied and there is no consensus that it is efficient to bank pathological tissues in a certain way prospectively. In addition, there is the opinion that the sample deteriorates (DNA fragmentation) when the thin-section sample from pathological tissue is stored for a long time.

Consent on the use of archival pathological tissue after medical care should therefore be obtained at the time of consent to banking, but actual collection should be initiated on an individual basis by creating a protocol and defining the most appropriate procedure for the study content in the protocol.

16. Organization

Changes to this chapter are considered to be revision rather than amendment.

16.1. Main study fund (funding source) of this study.

Practical Research for Innovative Cancer Control from Japan Agency for Medical Research and Development
“Establishment of standard treatments for neuroendocrine carcinoma of the digestive system” JP15ck0106138, JP16ck0106138, JP17ck0106355, JP18ck0106355, JP19ck0106355, JP20ck0106618
National Cancer Center Research and Development Fund (23-A-22, 26-A-4, 29-A-3, 2020-J-3)
“Scientific research on multi-institutional trials to establish new standard treatment of solid tumors in adults”

16.2. Japan Clinical Oncology Group (JCOG)

JCOG is a multi-institutional clinical research group consisting of research teams funded by public research grants

mainly on National Cancer Center Research and Development Fund and Japan Agency for Medical Research and Development research costs that receive direct support for research by the Clinical Research Support Office of the National Cancer Center Hospital in accordance with JCOG Policy (http://www.jcog.jp/).

This study is conducted using JCOG research organisations and in accordance with the regulations set out by JCOG Executive Committee.

16.3. JCOG Chair

Yuichiro Ohe National Cancer Center Hospital

16.4. Study group and Group Chair

JCOG Hepatobiliary and Pancreatic Oncology Group
Group Chair: Junji FURUSE
Department of Medical Oncology, Kyorin University Faculty of Medicine
〒181-8611 6-20-2, Shinkawa Mitaka-shi, Tokyo
TEL:+81-422-47-5511
FAX:+81-422-44-1858
E-mail: jfuruse@ks.kyorin-u.ac.jp
Group Secretary: Hirohi Ishii
Clinical Research Center, Chiba Cancer Center

〒260-8717, 666-2 Nitona, 260-8717, Chuo-Ku, Chiba
TEL:+81-43-264-5431
FAX:+81-43-264-8680
E-mail: hirishii@chiba-cc.jp
JCOG Stomach Cancer Study Group:
Group Chair: Masanori Terashima
Shizuoka Cancer Center
〒411-8777 1007 Shimonagakubo, Nagaizumi-cho, Sunto-gun, Shizuoka Prefecture
TEL:+81-55-989-5222
FAX:+81-55-989-5783
E-mail: m.terashima@scchr.jp
Group Secretary: Takaki Yoshikawa
National Cancer Center Hospital
〒104-0045 5-1-1 Tsukiji, Chuo-ku, Tokyo
TEL:+81-3-3542-2511
FAX:+81-3-3542-3815
E-mail: tayoshik@ncc.go.jp
Group Secretary: Narikazu Boku
National Cancer Center Hospital
〒104-0045 5-1-1 Tsukiji, Chuo-ku, Tokyo
TEL:+81-3-3542-2511
FAX:+81-3-3542-3815
E-mail: nboku@ncc.go.jp

JCOG Japan Esophageal Oncology Group
Group Chair: Yuko Kitagawa
Keio University Hospital
〒160-8582 35 Shinanomachi, Shinjuku-ku, Tokyo
TEL:+81-3-3353-1211
FAX:+81-3-3355-4707
E-mail: kitagawa@a3.keio.jp

Group Secretary: Ken Kato
National Cancer Center Hospital
〒104-0045 5-1-1 Tsukiji, Chuo-ku, Tokyo
TEL:+81-3-3542-2511
FAX:+81-3-3542-3815
E-mail: kenkato@ncc.go.jp

16.5. Study Chair (Principal Investigator)

JCOG Hepatobiliary and Pancreatic Oncology Group
Takuji Okusaka
National Cancer Center Hospital
〒104-0045 5-1-1 Tsukiji, Chuo-ku, Tokyo
TEL: +81-3-3542-2511
FAX: +81-3-3542-3815
E-mail: tokusaka@ncc.go.jp

JCOG Stomach Cancer Study Group:
Narikazu Boku
National Cancer Center Hospital
〒104-0045 5-1-1 Tsukiji, Chuo-ku, Tokyo
TEL: +81-3-3542-2511
FAX: +81-3-3542-3815
E-mail:nboku@ncc.go.jp

JCOG Japan Esophageal Oncology Group
Ken Kato
National Cancer Center Hospital
〒104-0045 5-1-1 Tsukiji, Chuo-ku, Tokyo
TEL: +81-3-3542-2511
FAX: +81-3-3542-3815
E-mail: kenkato@ncc.go.jp

16.6. Study Coordinator

JCOG Hepatobiliary and Pancreatic Oncology Group
Chigusa Morizane
National Cancer Center Hospital
〒104-0045 5-1-1 Tsukiji, Chuo-ku, Tokyo
TEL: +81-3-3542-2511
FAX: +81-3-3542-3815
E-mail: cmorizan@ncc.go.jp

JCOG Stomach Cancer Study Group:
Nozomu Machida
Shizuoka Cancer Center
〒411-8777 1007 Shimonagakubo, Nagaizumi-cho, Sunto-gun, Shizuoka Prefecture
TEL:+81-55-989-5222
FAX:+81-55-989-5631
E-mail：no.machida@scchr.jp
JCOG Japan Esophageal Oncology Group
Ken Kato

National Cancer Center Hospital
〒104-0045 5-1-1 Tsukiji, Chuo-ku, Tokyo
TEL: +81-3-3542-2511
FAX: +81-3-3542-3815
E-mail: kenkato@ncc.go.jp

Yoshitaka Honma
National Cancer Center Hospital
〒104-0045 5-1-1 Tsukiji, Chuo-ku, Tokyo
TEL: +81-3-3542-2511
FAX: +81-3-3542-3815
E-mail:yohonma@ncc.go.jp

16.7. Central Pathological Review Coordinator

Nobuyoshi Hiraoka
National Cancer Center Hospital
〒104-0045 5-1-1 Tsukiji, Chuo-ku, Tokyo
TEL: +81-3-3542-2511
FAX: +81-3-3542-3815
E-mail: nhiraoka@ncc.go.jp

Hirokazu Taniguchi
National Cancer Center
〒104-0045 5-1-1 Tsukiji, Chuo-ku, Tokyo
TEL: +81-3-3542-2511
FAX: +81-3-3542-3815
E-mail: hitanigu@ncc.go.jp

16.8. Central Pathological Review Coordinator

Nobuyoshi Hiraoka (National Cancer Center Hospital)
Noriyoshi Fukushima (Jichi Medical University)
Nobuyuki Ohike (Showa University Fujigaoka Hospital)
Ryoji Kushima (Shiga University of Medical Science)
Mitsuya Iwafuchi (Scool of Health Sciences Faculty of Medicine, Niigata University)
Tetsuo Ushiku (The University of Tokyo)

16.9. Participating sites (participating institutions)

HBP	SCSG	JEOG	Participating institutions
○			Sapporo-Kosei General Hospital
○		○	Hokkaido University Hospital
	○		Keiyukai Sapporo Hospital
	○	○	Iwate Medical University
		○	Tohoku University Hospital
	○		Miyagi Cancer Center
○	○	○	Tochigi Cancer Center
○			Jichi Medical University
○	○	○	Saitama Cancer Center
○	○	○	National Cancer Center Hospital East
○		○	Chiba Cancer Center
○		○	Chiba University, Graduate School of Medicine
○	○	○	National Cancer Center Hospital
○			Kyorin University Faculty of Medicine
○			National Center for Global Health and Medicine (NCGM)
○	○	○	Cancer Institute Hospital of Japanese Foundation for Cancer Research
	○	○	Toranomon Hospital
○			Teikyo University School of Medicine
○			St.Marianna University School of Medicine
○	○	○	Kanagawa Cancer Center
○	○		Yokohama City University Medical Center
○	○		Niigata Cancer Center Hospital
○			Toyama University Hospital
○			Kanazawa University School of Medicine
	○		Ishikawa Prefectural Central Hospital
	○		Ogaki Municipal Hospital
○	○	○	Shizuoka Cancer Center
○	○	○	Aichi Cancer Center Hospital
		○	Kyoto University Hospital
	○	○	Osaka University Graduate School of Medicine
○	○		Kindai University Hospital
○	○	○	Osaka International Cancer Institute
○	○	○	National Hospital Organization Osaka National Hospital
	○	○	Osaka General Medical Center
	○	○	Osaka Medical and Pharmaceutical University
	○		Osaka Rosai Hospital
○	○	○	Kobe University Graduate School of Medicine
	○	○	Kansai Rosai Hospital
○	○		Hyogo College of Medicine
○	○	○	Hyogo Cancer Center
	○		Itami City Hospital
	○		Shimane University Faculty of Medicine
	○	○	Hiroshima University Hospital
	○		Fukuyama City Hospital
	○		Tokushima Red Cross Hospital
○	○	○	National Hospital Organization Shikoku Cancer Center
○		○	Kochi Health Sciences Center
○		○	National Kyushu Cancer Center
○		○	Kyushu University Hospital

	○	○	Oita University Faculty of Medicine

HBP: Hepatobiliary and Pancreatic Oncology Group
SCSG: Stomach Cancer Study Group:
JEOG: Japan Esophageal Oncology Group

16.10. JCOG Protocol Review Committee

This protocol was approved by JCOG Protocol Review Committee prior to submit to Certified Review Board.
(For membership, see website http://www.jcog.jp/basic/org/committee/protocol.html)
Contact: Protocol Review Committee Office

JCOG Operations Office/Clinical Research Support Office, National Cancer Center Hospital
〒104-0045 5-1-1, Tsukiji, Chuo-ku, Tokyo
TEL: 03-3542-2511 (ext. 2302)
FAX:03-3542-7006
E-mail: jcogoffice@ml.jcog.jp

16.11. JCOG Data and Safety Monitoring Committee

During study period, the study will be monitored by Data and Safety Monitoring Committee (e.g., adverse event reports, interim analysis reviews, monitoring report reviews, protocol revision reviews). However, the committee members from the study group conducting this study do not participate directly in the review of this study.
(For membership, see website http://www.jcog.jp/basic/org/committee/jury.html)
Contact: JCOG Data and Safety Monitoring Committee Office

JCOG Operations Office/Clinical Research Support Office, National Cancer Center Hospital
〒104-0045 5-1-1, Tsukiji, Chuo-ku, Tokyo
TEL:03-3542-2511 (ext. 2403)
FAX:03-3542-7006
E-mail: jcogoffice@ml.jcog.jp

16.12. JCOG Audit Committee

Site-visit audits by Audit Committee will be conducted during study period.
(For membership, see website http://www.jcog.jp/basic/org/committee/audit.html)
Contact: JCOG Auditing Committee Office

JCOG Operations Office/Clinical Research Support Office, National Cancer Center Hospital
〒104-0045 5-1-1, Tsukiji, Chuo-ku, Tokyo
TEL:03-3542-2511 (ext. 2403)
FAX:03-3542-7006
E-mail: jcogoffice@ml.jcog.jp

16.13. JCOG Conflict of Interest Committee

During study period, JCOG investigators involved in this study will be managed by the Conflict of Interest Committee.
(For membership, see website http://www.jcog.jp/basic/org/committee/coi.html)
Contact: JCOG Conflict of Interest Committee Office

JCOG Operations Office/Clinical Research Support Office, National Cancer Center Hospital
〒104-0045 5-1-1, Tsukiji, Chuo-ku, Tokyo
TEL:03-3542-2511 (ext. 2404)
FAX:03-3547-1002
E-mail: jcogoffice@ml.jcog.jp

16.14. Data Center/Operations Office

JCOG Data Center

Director of Data Center: Haruhiko Fukuda
Clinical Research Support Office, National Cancer Center Hospital

〒104-0045 5-1-1, Tsukiji, Chuo-ku, Tokyo
TEL:03-3542-3373
FAX:03-3542-3374
E-mail: jcogdata@ml.jcog.jp

JCOG Operations Office
Director of Operations Office: Kenichi Nakamura
Clinical Research Support Office, National Cancer Center Hospital
〒104-0045 5-1-1, Tsukiji, Chuo-ku, Tokyo
TEL:03-3547-1002
FAX:03-3547-1002
E-mail: jcogoffice@ml.jcog.jp

Official website: http://www.jcog.jp/

16.14.1. Data management administrator

Data management organization		JCOG Data Center
Data management administrator	Name	Harumi Kaba
	e-Rad number	40543442
	Affiliation	Clinical Research Support Office, National Cancer Center Hospital
	Title	Head of Multi-institutional Data Management Section

16.14.2. Monitoring administrator

Monitoring organization		JCOG Data Center
Monitoring administrator	Name	Haruhiko Fukuda
	e-Rad number	70263390
	Affiliation	Clinical Research Support Office, National Cancer Center Hospital
	Title	Chief of Data Management Division

16.14.3. Site-visit audit administrator

Auditing organization		JCOG Operations Office
Site-visit audit administrator	Name	Kenichi Nakamura
	e-Rad number	40543533
	Affiliation	Clinical Research Support Office, National Cancer Center Hospital
	Title	Director of Research Management Division

16.14.4. Statistical analysis administrator

Statistical analysis organization		JCOG Data Center
Statistical analysis Administrator	Name	Junki Mizusawa
	e-Rad number	60706646
	Affiliation	Biostatistics Division, Center for Research Administration and Support, National Cancer Center/<br>Biostatistics Section, Research Management Division, Clinical Research Support Office, National Cancer Center Hospital
	Title	Biostatistics Section Head

16.14.5. Research and development plan support personnel

Organizations in charge of supporting research and development plans		JCOG Operations Office
Research and development plan support personnel	Name	Tomoko Kataoka
	e-Rad number	70569863
	Affiliation	Clinical Research Support Office, National Cancer Center Hospital
	Title	Medical officer

16.14.6. Coordination management practitioner

Organizations in charge of coordination and management		JCOG Operations Office
Coordinating and managing practitioners	Name	Junko Eba
	e-Rad number	80754085
	Affiliation	Clinical Research Support Office, National Cancer Center Hospital
	Title	Medical officer

16.14.7. Personnel who oversees the study other than Principal Investigator and site investigators

Personnel who oversees the study other than Principal Investigator and site investigators	Name	Not applicable	
	e-Rad number		
	Affiliation		
	Title		
	Relevance of Secondary Sponsor	□ Applicable	□ Not applicable

16.14.8. Study group personnel

JCOG Data Center

Statistics Section Gakuto OGAWA

Data Management Section Kyoko HASEGAWA

JCOG Operations Office

Science Section Tomoko KATAOKA/Tadayoshi HASHIMOTO

In addition, JCOG Data Center/Operations Office commissioned some of theire research support activities (such as support for the preparation of study protocols, data management, and site-visit audits) to other corporations. The commissioned duties are supervised by the National Cancer Center through routine work, as well as by receiving work reports from the institution and providing supervisory guidance. The current consignee is as follows

Clinical Oncology Research and Education, a specified non-profit organization

〒104-0061 DJ Ginza Building 7F, 8-18-3, Ginza, Chuo-ku, Tokyo

Official website http://www.core.or.jp/

16.15. Developing a study protocol

Chigusa MORIZANE, National Cancer Center Hospital

Nozomu MACHIDA, Shizuoka Cancer Center

Yoshitaka HONMA/Ken KATO, National Cancer Center Hospital

Support for protocol development

JCOG Data Center

Statistics Section (in charge of study design) Junki MIZUSAWA

Data Management Section (CRF preparation) Harumi KABA

JCOG Operations Office

Protocol development Hiroshi KATAYAMA/Kozo KATAOKA/Aya MIURA

Person in charge of IC documents Aya KIMURA/Noriko TSUJI

17. Publication of the study results and completion of the study

17.1. Paper and conference presentations

Primary publication will be published in English journals.

Paper publication including review article and conference presentation of Introduction of the study, by Principal Investigator or Study Coordinator, which does not include the analytical results of the endpoint of the study, are allowed when Group Chair and JCOG Data Center Director agree to them. Publication of the distribution of baseline factors or the safety data after the end of accrual are also allowed. No publication other than primary analysis and final analysis will be performed unless previously approved by Data and Safety Monitoring Committee.

In principle, the authors of the main published papers on the results of the study (the first publication of the results of primary endpoint) shall be the first Study Coordinator (HBPOG), followed by Study Coordinator (JEOG or SCSG), Study Coordinator (JEOG or SCSG), Principal Investigator (HBPOG), Principal Investigator (JEOG or SCSG), Principal Investigator (JEOG or SCSG), the statistical staff of Data Center (one statistician in charge at the time of the analysis for publication), Centralized Pathological diagnosis Coordinator (HBP), Centralized Pathological diagnosis Coordinator (GI). In accordance with the limitations imposed by the article's posting provisions, researchers who contributed in descending order of their number of patients registrations were selected for each institution as co-authors, and the last author was Group Chair (or Study Chair). The inclusion of staffs of JCOG Operations Office as co-authors will be determined by Group Chair depending on their contributions. Authors of articles other than the primary publication (e.g., Secondary endpoints articles, secondary analysis articles) will be determined by Study Chair with Group Chair approval.

All co-authors will review the article contents prior to submission for publication and only those who agree to the article contents. If there is no consensus on the contents, principle investigator may not include the investigator in the co-authorship with the approval of Group Chair. If there is no consensus between groups and JCOG Data Center/Operations Office, ultimately follow JCOG Chair instructions.

17.2. Primary Endpoint Report and Clinical Summary Report

The procedures are specified from the preparation of the primary endpoint report and clinical summary report. If primary analysis is the final analysis, the primary endpoint report will not be prepared and the clinical summary report will be prepared.

17.2.1. Clinical Summary Report

Based on the final analysis report, Principal Investigator will prepare a "Abstract of the Clinical Summary Report" containing subjects background information (age, sex, etc.), study design and study progress, results of analyses for each endpoint, conclusions of the entire study, interpretations and discussion of the results, etc. within 6 months from the issue date of the final analysis report, submit it to the Data Center, and undergo review. In addition, the issue date of the final analysis report shall be "the date when the period for collecting data on all endpoints is completed" as specified in the Enforcement Regulations.

After obtaining approval from Group Chair and the Director of the Data Center, abstract of the clinical summary report will be submitted to Certified Review Board review as a "clinical summary report" with the final analysis report within 1 year of the issue date of the final analysis report.

Principal Investigator/Study Coordinator will disclose abstract of the clinical summary report (Notification of Completion of Form 1 of Article 24 of Regulation) to jRCT within 1 month after obtaining Certified Review Board approval (if the report is unpublished, abstract will not be released in jRCT and will be published immediately after publication).

The approved abstract of the clinical summary report will be submitted to the administrators of each participating institution through the site investigators at each institution and to JCOG Chair. Abstract of the clinical summary report is available on JCOG website (http://www.JCOG.jp/).

The timing of distribution of the final analysis report to participating institutions will be determined by Principal Investigator/Study Coordinator considering the timing of publication of the final analysis results, and the final analysis report will be distributed to the researchers at participating institutions by themselves or through the Data Center.

17.3. Completion of the study

On the date that abstract of the clinical summary report was released to iRCT, the study is completed.

In institutions where patients were not enrolled, the institution may be withdrawn from the institution list by submitting a request for change (Unified Form 3 for Clinical Research) and a Notification of Changes in Protocol (Form 2) stating that Principal Investigator withdraws the institution from the institution list in question to Certified Review Board, and after obtaining approval, notifying the MHLW (Local Health Bureau responsible for the location of Certified Review Board). When a protocol change notification is received by the Local Health Service, the investigator at the institution shall report the withdrawal to the Administrator of the institution.

18. References

omit

19. Appendix

- Informed consent form
- Body surface area table
- Toxicity Criteria (CTCAE v5.0-JCOG)
- CRF samples* (CRF draft attached for the first review submission)
- JCOG-Biobank Japan Biorepository Protocol
- JCOG-Biobank Japan Biorepository Informed Consent Form

Japan Clinical Oncology Group
Hepatobiliary and Pancreatic Oncology Group/Stomach Cancer Study Group/Japan Esophageal Oncology Group

Practical Research for Innovative Cancer Control from Japan Agency for Medical Research and Development
"Establishment of standard treatments for neuroendocrine carcinoma of the digestive system"
National Cancer Center Research and Development Fund 29-A-3

JCOG1213

Randomized phase III study of etoposide plus cisplatin combination therapy versus irinotecan plus cisplatin combination therapy in advanced neuroendocrine carcinoma of the digestive system. ver.1.4.1

TOPIC-NEC

HBPOG
Group Chair: Junji FURUSE
Department of Medical Oncology, Kyorin University Faculty of Medicine

Study Chair: Takuji OKUSAKA
Department of Hepatobiliary and Pancreatic Oncology, National Cancer Center Hospital 5-1-1 Tsukiji, Chuo-ku, Tokyo 104-0045 Japan
Tel: +81-3-3542-2511
E -mail : tokusaka@ncc.go.jp

Study Coordinator: Chigusa MORIZANE
Department of Hepatobiliary and Pancreatic Oncology, National Cancer Center Hospital 5-1-1 Tsukiji, Chuo-ku, Tokyo 104-0045 Japan
Tel: +81-3-3542-2511
E -mail : cmorizan@ncc.go.jp

SCSG
Group Chair: Masanori TERASHIMA
Division of Gastric Surgery, Shizuoka Cancer Center Hospital

Study Chair: Narikazu BOKU
Department of Gastrointestinal Oncology, National Cancer Center Hospital
5-1-1 Tsukiji, Chuo-ku, Tokyo 104-0045 Japan
Tel: +81-3-3542-2511
E -mail : nboku@ncc.go.jp

Study Coordinator: Nozomu MACHIDA
Department of Hepatobiliary and Pancreatic Oncology, Shizuoka Cancer Center
1007 Shimonagakubo, Nagaizumi-cho, Sunto-gun, Shizuoka Prefecture 411-8777 Japan
Tel: +81-55-989-5222
E -mail : no.machida@scchr.jp

JEOG
Group Chair: Yuko KITAGAWA
General and Gastroenterological Surgery, Keio University Hospital

Study Chair: Ken KATO
Department of Gastrointestinal Oncology, National Cancer Center Hospital 5-1-1 Tsukiji, Chuo-ku, Tokyo 104-0045 Japan
Tel: +81-3-3542-2511
E -mail : kenkato@ncc.go.jp

Study Coordinator: Yoshitaka HONMA/Ken KATO
Department of Gastrointestinal Oncology, National Cancer Center Hospital 5-1-1 Tsukiji, Chuo-ku, Tokyo 104-0045 Japan
Tel: +81-3-3542-2511
E-mail:yohonma@ncc.go.jp
/kenkato@ncc.go.jp

2013/3/16	Protocol concept approved by JCOG Executive Committee (PC1213/1214/1215)
2014/6/27	Protocol approved byJCOG Protocol Review Committee
2017/5/23	Revision ver. 1.1 approved by JCOG Data and Safety Monitring Committee
2018/8/16	Revision ver. 1.2 approved by JCOG Data and Safety Monitring Committee
2018/10/11	Approved by Certified Review Board of National Cancer Center Hospital
2019/5/27	Revision ver. 1.3.0. approved by JCOG Data and Safety Monitring Committee
2019/6/27	Approved by Certified Review Board of National Cancer Center Hospital
2020/1/24	Revision ver. 1.4.0. approved by JCOG Data and Safety Monitring Committee
2020/2/27	Approved by Certified Review Board of National Cancer Center Hospital

0. Summary

This study is conducted as a "specified clinical trial" based on the Clinical Trials Act (Act No. 16 of April 14, 2017). In this protocol, the Principal Investigator is the Study Chair in the Hepatobiliary and Pancreatic Oncology Group of JCOG.

Name of study: "Randomized phase III study of etoposide plus cisplatin combination therapy versus irinotecan plus cisplatin combination therapy in advanced neuroendocrine carcinoma of the digestive system. (TOPIC-NEC) ".

Public study title: "Randomized phase III study of etoposide plus cisplatin combination therapy versus irinotecan plus cisplatin combination therapy in advanced neuroendocrine carcinoma of the digestive system. (TOPIC-NEC) "

0.1. Schema

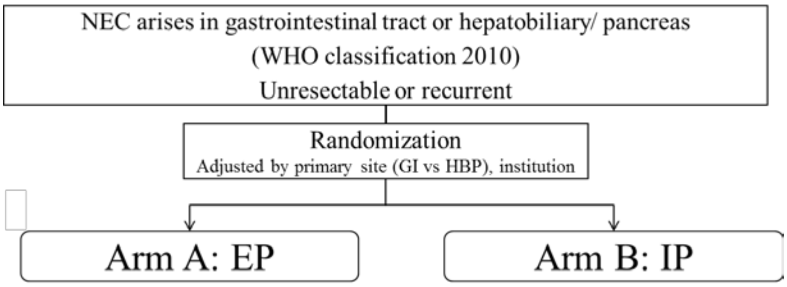

0.2. Objectives

A randomized phase III study was conducted to determine the better treatment option between etoposide/cisplatin combined therapy (EP therapy) or irinotecan/cisplatin combined therapy (IP therapy), both of which are standard treatments for non-resectable/recurrent neuroendocrine carcinoma (NEC as classified by WHO in 2010) with primary lesions in the gastrointestinal tract/hepatobiliary pancreatic organs.

Primary endpoint: Overall survival

Secondary endpoints: Response rate in case of measurable lesions, Progression-free survival (PFS), incidence rate of adverse events, dose intensity of Cisplatin, and incidence rate of serious adverse events.

0.3. Subjects

1) Any of the following is applicable based on pathological diagnosis taking findings of immunohistochemistry into consideration (see 3.1. to 3.3.).

[1] Pathologically diagnosed as neuroendocrine carcinoma (NEC*1) in the resected sample.

[2] Containing pathologically confirmed component of neuroendocrine carcinoma (NEC*1) in the biopsy sample.

1: Based on WHO 2010 classification

2) Any of the following is applicable

[1] NEC arise in esophagus, stomach, duodenum, intestine, appendix, colon, rectum, gallbladder, intrahepatic bile duct, extrahepatic bile duct, ampulla of Vater, pancreas,

[2] Liver NEC (primary liver or liver metastasis of unknown primary) *2.

*2: The tumor is only in one of the following sites after a thorough examination of the primary site by contrast CT (from the neck to pelvic) and upper/lower gastrointestinal endoscopy, FDG-PET scan, otolaryngology (head and neck) examination, urology examination (male patients only), and gynecology examination (female patients only).

a. Liver only

b. Bone, lymph nodes below the diaphragm, peritoneum, subcutaneous, muscle, spleen, and liver

3) Unresectable (see 3.6) or recurrent cancer (see 3.7). It is not essential for a pathological confirmation of the metastatic lesion or recurrent site. Cases of esophageal NEC is ineligible if corresponding to any of the following.

[1] cT4.

[2] No distant metastasis rather than supraclavicular lymph node

[3] Stenosis indicated for palliative radiotherapy

4) No previous chemotherapy or radiotherapy for NEC. Pre- or post-operative chemotherapy except irinotecan or

etoposide for NEC is allowed as long as it was completed at least 8 weeks prior to registration.

5) No previous chemotherapy using platinum agents for any malignancies.

6) No serious tumor-related complications.

Serious tumor-related complications include superior vena cava syndrome, inferior vena cava syndrome, pleural, ascites, or pericardial effusions that are large or uncontrollable (e.g., effusions that accumulate quickly after drainage or even after adhesive surgery), and brain metastases with neurological symptoms.

7) Aged 20 to 75 years old.

8) ECOG performance status of 0 or 1.

9) Sufficient oral intake

10) Measurable region is not required.

11) Adequate organ functions.

[1] WBC ≥3,000/ mm^3

[2] Neutrophils ≥ 1,500/ mm^3

[3] Hemoglobin ≥ 9.0 g/dL

[4] Platelets ≥ 10×10^4/ mm^3

[5] Total bilirubin≤ 1.5 mg/dL※4

[6] AST(sGOT)≦100 IU/L※4（for hepatic NEC and liver metastasis, ≦150 IU/L）

[7] ALT(sGPT)≦100 IU/L※4（for hepatic NEC and liver metastasis, ≦150 IU/L）

※4：Presence or absence of biliary drainage is not relevant

[8] Serum creatinine≦ 1.3 mg/dL

[9] Creatinine clearance※5≥ 60 mL/min

※5： Creatinine clearance must have been estimated using the Cockcroft-Gault formula, and must be 60 mL/min or more. If the estimation is less than 60 mL/min, but the actual measurement is 60 mL/min or more, the patient can be deemed eligible.

Cockcroft-Gault formula

Male：Ccr ={(140－age) × body weight (kg)}/{72×serum creatinine (mg/dL)}

Female：Ccr = 0.85×{(140－age) × body weight (kg)}/{72×serum creatinine(mg/dL)}

12) Written informed consent.

0.4. Treatments

Arm A: etoposide plus cisplatin (EP) arm

The following chemotherapy is continued until the patient meets discontinuation criteria, with 3-weeks of treatments counting as one cycle.

Drug	Dosage (mg/m^2)	Dosing regimen/Dosing time	Dose day
Etoposide	100	IV/60-120 min	Day 1, 2, 3
Cisplatin	80	IV/60-120 min	Day 1

Arm B: Irinotecan plus Cisplatin (IP) arm

The following chemotherapy would be continued until the patient meets discontinuation criteria, with 4-weeks of treatments counting as one cycle.

Drug	Dose (mg/m^2)	Dosing regimen/Dosing time	Dose day
Irinotecan	60	IV/90 min	Day 1, 8, 15
Cisplatin	60	IV/60-120 min	Day 1

0.5. Planned sample size and study period

The planned sample size is 170.

Accrual period: 6 years.

Follow-up period: 1 years after accrual completion.

Analysis period: 1 year.

Total study duration 8 years.

Scheduled start date of the study Aug 8, 2014

Expected completion date of the study Aug 8, 2028

0.6. Contact information

Eligibility criteria, treatment modification, and other issues requiring clinical decisions: Study Coordinator (front cover and 16.6.)

Enrollment procedure protocol, case report form (CRF) entries, etc.: JCOG Data Center, (16.14.)

Adverse event reporting: JCOG Data and Safety Monitoring Committee (16.11.),

Contents

0. SUMMARY 2

0.1. SCHEMA 2
0.2. OBJECTIVES 2
0.3. SUBJECTS 2
0.4. TREATMENTS 3
0.5. PLANNED SAMPLE SIZE AND STUDY PERIOD 3
0.6. CONTACT INFORMATION 4

1. OBJECTIVES 8

2. BACKGROUND 8

2.1. TARGET 8
2.2. STANDARD TREATMENT FOR TARGET DISEASE 16
2.3. RATIONALE FOR ESTABLISHMENT OF TREATMENT PLAN 18
2.4. STUDY DESIGN 21
2.5. SUMMARY OF EXPECTED ADVANTAGES AND DISADVANTAGES ASSOCIATED WITH STUDY PARTICIPATION 22
2.6. SIGNIFICANCE OF THIS STUDY 23
2.7. ASSOCIATED RESEARCH (INCLUDING SAMPLE ANALYSIS RESEARCH) 23
2.8. JCOG-BIOBANK JAPAN (BBJ) COLLABORATING BIOBANK 23

3. CRITERIA/DEFINITIONS USED IN THIS STUDY 25

3.1. TISSUE CLASSIFICATION (WHO 2010 CLASSIFICATION) 25
3.2. GRADE CLASSIFICATION（ENETS [EUROPEAN NEUROENDOCRINE TUMOR SOCIETY] / WHO2010 CLASSIFICATION）........ 25
3.3. HISTOPATHOLOGICAL DIAGNOSIS 25
3.4. DISEASE STAGE CLASSIFICATION CRITERIA (UICC-TNM 7TH EDITION) 25
3.5. RESIDUAL TUMOR (R) CLASSIFICATION (UICC-TNM 7TH EDITION) 33
3.6. DEFINITION OF NON-RESECTABLE NEC 33
3.7. DEFINITION OF RECURRENT NEC 34
3.8. DEFINITION OF HEPATIC NEC (HEPATIC PRIMARY LESION OR LIVER METASTASIS FROM AN UNKNOWN PRIMARY LESION) 34

4. PATIENT SELECTION CRITERIA 34

4.1. INCLUSION CRITERIA (FOR ENROLLMENT) 34
4.2. EXCLUSION CRITERIA 34

5. REGISTRATION AND RANDOMIZATION 36

5.1. PROCEDURE OF REGISTRATION 36
5.2. RANDOMIZATION AND ALLOCATION ADJUSTMENT FACTOR 37

6. TREATMENT PLAN AND TREATMENT MODIFICATION CRITERIA 38

6.1. PROTOCOL TREATMENT 38
6.2. PROTOCOL TREATMENT TERMINATION/COMPLETION CRITERIA 41
6.3. TREATMENT MODIFICATION CRITERIA 42
6.4. CONCOMITANT TREATMENT AND SUPPORTIVE CARE 48
6.5. POST-STUDY TREATMENT 55

7. ANTICIPATED ADVERSE EVENTS 56

7.1. ANTICIPATED ADVERSE REACTIONS 56

7.2. ANTICIPATED ADVERSE EVENTS DUE TO PATHOGENESIS 59
7.3. EVALUATION OF ADVERSE EVENTS/REACTIONS 60

8. EXAMINATION AND EVALUATION 62

8.1. BASELINE EXAMINATION AND EVALUATION BEFORE REGISTRATION 62
8.2. EXAMINATION AND EVALUATION DURING TREATMENT 63
8.3. EXAMINATION AND EVALUATION AFTER COMPLETION OF TREATMENT 64
8.4. INFORMATION ON POST- STUDY TREATMENT 64
8.5. STUDY CALENDAR 65

9. DATA COLLECTION 69

9.1. CASE REPORT FORM (CRF) 69

10. REPORTING OF "DISEASE OR THE LIKE"(ADVERSE EVENTS) 70

10.1. SERIOUS ADVERSE EVENTS AND SUBJECTS OF EXPEDITED REPORTING. 70
10.2. INVESTIGATOR'S REPORTING REQUIREMENTS AND PROCEDURES 71
10.3. RESPONSIBILITIES OF PRINCIPAL INVESTIGATOR/STUDY COORDINATOR 73
10.4. RESPONSIBILITIES OF THE SITE INVESTIGATORS AT THE PARTICIPATING INSTITUTIONS (INCLUDING THE RELEVANT INSTITUTION) 75
10.5. RESPONSIBILITIES OF THE DATA AND SAFETY MONITORING COMMITTEE 75

11. RESPONSE EVALUATION AND ENDPOINT DEFINITION 76

11.1. RESPONSE ASSESSMENT (ONLY FOR PATIENTS WITH MEASURABLE DISEASE) 76
11.2. DEFINITIONS OF ANALYSES SET 82
11.3. DEFINITION OF ENDPOINTS 82

12. STATISTICAL CONSIDERATION 85

12.1. PRINCIPAL ANALYSIS AND DECISION CRITERIA 85
12.2. PLANNED ACCRUAL, ACCRUAL PERIOD, AND FOLLOW-UP PERIODS 86
12.3. INTERIM ANALYSIS AND EARLY TERMINATION OF THE STUDY 86
12.4. ANALYSIS OF SECONDARY ENDPOINTS 88
12.5. FINAL ANALYSIS 88
12.6. EXPLORATORY ANALYSIS 89
12.7. PREMATURE WITHDRAWAL FROM THE TRIAL 89
12.8. PROCEDURES AFTER EARLY TERMINATION OF THE STUDY 90

13. ETHICAL CONSIDERATIONS 92

13.1. PROTECTION OF HUMAN SUBJECTS 92
13.2. INFORMED CONSENT 92
13.3. PROTECTION OF PERSONAL INFORMATION AND PATIENT IDENTIFICATION 94
13.4. ADHERENCE TO THE PROTOCOL 96
13.5. APPLICATION TO CERTIFIED REVIEW BOARD AND NOTIFICATION OF IMPLEMENTATION PLANS 96
13.6. PROTOCOL REVISION/AMENDMENT 100
13.7. CONFLICTS OF INTEREST (COIS) INVOLVED IN THIS STUDY 102
13.8. COMPENSATION 104
13.9. INTELLECTUAL PROPERTY 105
13.10. DISCLOSURE OF INFORMATION ON THIS STUDY 105

14. MONITORING AND AUDIT 106

14.1. PERIODIC MONITORING 106
14.2. SITE VISIT AUDITS 108

14.3. MANAGEMENT OF NON-COMPLIANCE 109

15. SPECIAL INSTRUCTIONS 110

15.2. JCOG BIOBANK JAPAN (BBJ) BIOREPOSITORY 111

16. ORGANIZATION 111

16.1. MAIN STUDY FUND (FUNDING SOURCE) OF THIS STUDY. 111
16.2. JAPAN CLINICAL ONCOLOGY GROUP (JCOG) 111
16.3. JCOG CHAIR 112
16.4. STUDY GROUP AND GROUP CHAIR 112
16.5. STUDY CHAIR (PRINCIPAL INVESTIGATOR) 113
16.6. STUDY COORDINATOR 113
16.7. CENTRAL PATHOLOGICAL REVIEW COORDINATOR 114
16.8. CENTRAL PATHOLOGICAL REVIEW COORDINATOR 114
16.9. PARTICIPATING SITES (PARTICIPATING INSTITUTIONS) 115
16.10. JCOG PROTOCOL REVIEW COMMITTEE 116
16.11. JCOG DATA AND SAFETY MONITORING COMMITTEE 116
16.12. JCOG AUDIT COMMITTEE 116
16.13. JCOG CONFLICT OF INTEREST COMMITTEE 116
16.14. DATA CENTER/OPERATIONS OFFICE 116
16.15. DEVELOPING A STUDY PROTOCOL 118

17. PUBLICATION OF THE STUDY RESULTS AND COMPLETION OF THE STUDY 119

17.1. PAPER AND CONFERENCE PRESENTATIONS 119
17.2. PRIMARY ENDPOINT REPORT AND CLINICAL SUMMARY REPORT 119
17.3. COMPLETION OF THE STUDY 120

18. REFERENCES 121

19. APPENDIX 121

1. Objectives

A randomized phase III study was conducted to determine the better treatment option between etoposide/cisplatin combined therapy (EP therapy) or irinotecan/cisplatin combined therapy (IP therapy), both of which are standard treatments for non-resectable/recurrent neuroendocrine carcinoma (NEC as classified by WHO in 2010) with primary lesions in the gastrointestinal tract/hepatobiliary pancreatic organs.

Primary endpoint：Overall survival

Secondary endpoints：Response rate in case of measurable lesions
Progression-free survival (PFS), incidence rate of adverse events, dose intensity of Cisplatin, and incidence rate of serious adverse events

2. Background

2.1. Target

2.1.1. Epidemiology

Neuroendocrine tumors (NETs) and neuroendocrine carcinomas (NECs) can arise in various organs in the body. Of these, NECs with primary lesions occurring in the lungs, namely small cell lung cancer and large cell lung cancer, have been collectively referred to as high-grade NECs (HGNECs). However, terms such as extrapulmonary small cell carcinoma and extrapulmonary NEC have been used to refer collectively to NECs, with primary lesions outside the lungs.

The number of newly reported cases of neuroendocrine neoplasms (NENs) annually, estimated based on the cases registered in the U.S. SEER（Surveillance, Epidemiology, and End Results）database between 1973 to 2004 is 5 in 100,000 people[1]. In Japan, the Neuroendocrine Tumor Workshop Japan （NET Work Japan）carried out a nationwide survey of pancreatic and gastrointestinal NENs, and estimated the number of new cases in 2005 at approximately 1.01 people with pancreatic primary lesion per 100,000 people, and 2.10 people with gastrointestinal primary lesion per 100,000 people[2, 3].

The report by the PRONET Study Group in France concerning their prospective observational study is a good reference for the proportion of NENs that can be classified as NECs. Out of the 778 patients diagnosed with gastrointestinal primary lesion NENs in 80 facilities from August 2010 to July 2011, 104 patients (13.4%) had NEC[4].

The frequency of incidence of NECs according to the primary lesion organ has been shown in the tables below (Table 2.1.1a and Table 2.1.1b). Despite variations between reports, gastrointestinal primary lesion NECs account for approximately 20–68% of all extrapulmonary NEC cases. The breakdown of gastrointestinal primary lesion NEC cases with primary lesion in the gastrointestinal tract (esophagus, duodenum, small and large intestines) indicated that these cases accounted for 56–84% of the total cases, while hepatobiliary pancreatic cancer cases accounted for 15–35% of all cases.

Table 2.1.1a. Frequency of extrapulmonary NECs according to the organ of primary lesion

Reported year/Reporter	N	Gastrointestinal	Gynecologal organs	Urology	Head and Neck	Adrenal	Primary unknown	Other
2012/Terashima [5]	136	68%	12%	6%	NA	NA	8%	7%
2010/Brennan [6]	74	20%	35%	15%	19%	NA	9%	1%
2009/Wong [7]	1618	33%	NA	20%	11%	10%	4%	22%
2006/Haider [8]	101	20%	11%	18%	12%	9%	31%	NA
2007/Lee [9]	61	56%	20%	10%	8%	NA	6%	NA

Table 2.1.1b. Percentage breakdown of NECs as per the primary lesion organ of gastrointestinal NEC

Reported year/Reporter	N	Esophageal	Gastric	Duodenal/small intestine	Colorectal	Hepatobiliary	Pancreas	Other
2012/Machida [10]	258	33%	27%	2%	12%	12%	14%	NA
2012/Sorbye [11)]	205	6%	10%	NA	40%	NA	35%	10%
2004/Brenner [12]	544	53%	11%	0.2%	20%	11%	4%	NA

2.1.2. Clinical pathology

1）About the term of target disease

The target of this study has a complex disease concept, and since the method of classification has changed with time, various names have been assigned that could cause confusion. In this study, we use classification and nomenclature defined according to the WHO 2010 classification （WHO Classification of Tumors of the Digestive System 2010）[13].

All the tumors originating from neuroendocrine cells or differentiation into endocrine cells are called neuroendocrine neoplasms (NENs). Depending on the malignancy, NENs were classified as Grade 1 NETs (NET G1), Grade 2 NETs (NET G2), or NEC.

Furthermore, prior to the WHO 2010 classification, all disease concepts equivalent to NENs were called NETs, but in this study, we would have referred to all descriptions of the disease concept as NENs. Furthermore, according to the 2000 and 2004 WHO classifications, the term well-differentiated NEC (WDNEC) has been used for disease units equivalent to NET G2, but in this study NEC does not mean the same.

2）Classification and clinical pathology

NEN is a tumor that develops from neuroendocrine cells present in various tissues or shows a tendency of differentiation into neuroendocrine cells. Histologically these tumors express neuroendocrine markers, such as Chromogranin A, NSE (neuron-specific enolase), and synaptophysin. The WHO classifications in 2000 and 2004 combined the presence or absence of metastasis/local infiltration and cellular proliferative capacity (evaluated based on Ki-67 expression intensity and mitotic presentation), and thereby classified these tumors into well-differentiated (neuro)endocrine tumors without metastasis/local infiltration, well-differentiated (neuro)endocrine tumors with metastasis/local infiltration, and poorly differentiated (neuro)endocrine tumors with even greater cell proliferative capacity. On the other hand, European Neuroendocrine Tumor Society (ENETS) has proposed a method of classification that evaluates the malignancy as Grade 1, 2, or 3 (G1, G2, or G3), according to the cell proliferative capacity (Ki-67 index or number of mitotic presentations), which has proven to be most useful for classification of prognosis[14, 15]。

Against this backdrop, the WHO classification that targeted gastrointestinal diseases was published in 2010. The disease as a whole was considered “neuroendocrine neoplasms (NEN)”, and the disease malignancy was largely classified as NET G1 (neuroendocrine tumor Grade 1), NET G2 (Grade 2), NEC, and mixed adenoneuroendocrine carcinoma (MANEC). MANEC refers to the cancers wherein adenocarcinoma components account for more than 30% of the cancer, while those under 30% are classified as NECs. However, even though this method of classification is applicable while evaluating the entire tumor based on resected specimen, it is not possible to evaluate the proportion of each component in the entire tumor during a diagnosis based on biopsy sample.

Each of NET G1, NET G2, and NECs described in the WHO 2010 classification is equivalent to G1, G2, and G3 as per the ENETS classification. NETs are well-differentiated tumors, with relatively low atypicality and malignancy, and the clinical course is slow with a 5-year survival in the range of 62–85%[16, 17]. On the other hand, NECs are poorly differentiated tumors, where tumor cells with poor cellular constituents proliferate diffusely, and are pathologically similar to small cell lung cancers, since these show many mitotic presentations, involve necrotic lesions, and present with neuroendocrinological features during immunohistochemical staining[1]. The clinical presentations are also similar in terms of the rapid proliferation of NECs and relatively high sensitivity to anticancer drugs[18].

Similar to lung cancer, NECs also exist as small cell cancer type or large cell type (LCNEC：large cell NEC）[13]. Future challenges include studies on the frequency and differences in clinical presentation of each disease. Table 2.1.2 shows the WHO and Grade classification.

Table 2.1.2. Changes in WHO classification and grading; Shaded parts are targets of this study

WHO 2000 classification	WHO 2010 classification		ENETS Grade
Well-differentiated endocrine tumor（WDNET）<br>1.1‘Benign’ behavior<br>1.2 Uncertain behavior	Neuroendocrine tumors, NETs<br>• Well-differentiated<br>• Composed of cells similar to normal gastrointestinal	NET G1（carcinoid）	G1<br>Same as 2010 WHO classification<br>Number of mitotic presentations < 2 per 10

	endocrine cells<br>• Expression of neuroendocrine markers<br>• Hormone production<br>• Mild to moderate nuclear atypia, low proliferative capacity (Grade : G1,G2)		high power fields (HPF) and/or Ki-67 index ≤2%
Well-differentiated endocrine carcinoma (WDEC)		NET G2	G2<br>Number of mitotic presentations 2–20 per 10 HPF and/or Ki-67 index 3-20%
Poorly differentiated endocrine carcinoma/small cell carcinoma (PDEC)	Neuroendocrine carcinoma : NEC (large cell or small cell type) :<br>• Poorly differentiated, highly malignant<br>• Include small cell to large cell type carcinoma<br>• Sometimes present tissue structure similar to NET<br>• Expression of neuroendocrine markers<br>• Significant nuclear atypia, multifocal necrosis, and high proliferative capacity (Grade: G3)		G3<br>Number of mitotic presentations >20 per 10 HPF and/or Ki-67 index > 20%
Mixed exocrine-endocrine carcinoma (MEEC)	Mixed adenoendocrine carcinoma (MANEC)		
Tumor-like lesions (TLL)	Hyperplastic and preneoplastic lesions		

2.1.3. Staging

Disease staging for NENs is represented by the TNM classification proposed by ENETS (hereafter, ENETS TNM[14, 15], AJCC 7th Edition TNM classification and UICC 7th Edition TNM classification published in 2009. The AJCC 7th Edition and UICC 7th Edition TNM classifications are unified, and in this study we would have used the UICC 7th Edition nomenclature).

In the UICC 7th edition, there are independent TNM classifications of NET G1 and NET G2 with primary lesions in the stomach, small intestine, appendix, and colon, but with respect to NEC, it describes "classification according to main tissue type in each organ of primary lesion (squamous cell carcinoma for esophagus, and adenocarcinoma in all other organs of primary lesion)." While there are TNM classifications for hepatocellular carcinoma and intrahepatic cholangiocarcinoma in the liver, there is no description for the classification of the tissue type for NECs with primary lesion in the liver. In this study, however, we would have used the TNM classification of intrahepatic cholangiocarcinoma, which shows similar clinical presentations. Details of each TNM classification would have been mentioned in “3.2 staging criteria”.

2.1.4. Standard treatment according to disease staging and outline of prognosis

1) Standard treatment for resectable cases

Surgical resection is performed for resectable cases. The significance of postoperative adjuvant chemotherapy is not clear because a verification study has not yet been performed. Although the NCCN Guidelines[19] describe postoperative adjuvant therapy as the chemotherapy regimen for small cell lung cancers that is typically applied to advanced cases of NEC, it has relatively high toxicity and patient burden (see Table 2.2.2, 2.3.2); hence we cannot claim that it is widely used in routine practice. For NECs with gastrointestinal primary lesion with mixed adenocarcinoma, a chemotherapy regimen is suggested typically after surgery for adenocarcinoma; however, in practice, there is no consensus on postoperative adjuvant chemotherapy.

2) Standard treatment for locally advanced cases

While the NCCN Guidelines propose chemoradiotherapy for small cell lung cancer for locally advanced cases, the ENETS Guidelines provide no clear indication[20, 21] for the same. As there is no sufficient information related to radiation dose, efficacy, and safety for NECs with a multitude of primary lesion organs (scope of irradiation), radiation therapy is not widely used for pathologies other than NEC with esophageal primary lesion that is prone to stenosis, and treatment is often provided for distant metastasis.

For locally advanced cases of NEC with esophageal primary lesion, chemoradiotherapy is actively performed. Furthermore, supraclavicular lymph node metastasis in thoracic esophageal tumor is classified as Stage IV, and chemoradiotherapy is applied if metastatic lymph nodes are included in the field of irradiation.

3) Standard treatment for distal metastasis cases

Systemic chemotherapy is indicated for distal metastasis and recurrent cases regardless of the organ, and a regimen is chosen for the small cell lung cancer. With respect to NECs with esophageal primary lesion, even if there is organ metastasis, palliative chemoradiotherapy is prioritized for patients incapable of oral intake of drugs due to esophageal constriction.

The efficacy of everolimus and sunitinib has been demonstrated for NETs with pancreatic primary lesions. Furthermore, Octreotide and Lantreotide effectively inhibit proliferation of NETs that originate in the midgut and pancreatic/gastrointestinal NETs, respectively. However, these results are from clinical trials conducted in patient populations corresponding to NETs. Since the clinical presentations of NETs and NECs differ significantly, these treatments cannot be indicated for NECs during routine practice.

4) Indication of resection for distal metastasis

There is no consensus on the resection criteria specific to the NECs. Since the progression of NETs is slow, resection is actively considered even where there is distal metastasis to the liver, but as NEC progresses fast, surgical resection cannot be indicated for cases with distal metastasis[19, 21].

5) Prognosis

In a preceding study, Yamaguchi and Machida carried out a multicenter joint observational study of poorly

differentiated NEC (WHO 2010 NEC, including patients with clinical diagnosis of poor differentiation) in a total of 23 facilities, including facilities participating in JCOG Hepatobiliary and Pancreatic Oncology Group, former Gastroenterology Group, and observer facilities.

In the multicenter joint study by Yamaguchi and Machida, 89% of cases (229/258 patients) receiving systemic chemotherapy had distal metastasis, with a median survival time (MST) of 11.5 months for all patients, 11.2 months for distal metastasis cases, and 15.9 months for locally advanced cases. MST according to organ was 13.4 months for esophageal primary lesion (N = 85), 13.3 months for gastric primary lesion (N = 70), 29.7 months for small intestine/duodenal primary lesion (N = 6), 7.6 months for colonic primary lesion (N = 31), 8.5 months for pancreatic primary lesion (N = 35), and 7.9 months for hepatobiliary primary lesions (N = 31) (Table 2.1.4). While there were 9 cases of five-year survival among all the gastrointestinal cases, there were no five-year survival cases amongst those with primary lesions in hepatobiliary and pancreatic organs[10]. In a multicenter joint observational study for NECs conducted in four Northern European countries (hereafter referred to as NORDIC NEC Study), the MST for NECs with gastrointestinal primary lesion (albeit including ~30% of NECs with primary lesion sites unknown) was 11 months[11]. According to the U.S. SEER database, the MST of NEC was 10 months[3]. Based on the above information, despite differences in the primary lesion organ, the prognosis of the target population of this study is thought to have an overall MST of 10–11 months.

Table 2.1.4. Treatment results according to primary lesion organ for non-resectable/recurrent NECs that underwent systemic chemotherapy

	Esophageal	Gastric	Duodenal/small intestine	Colonic	**Gastrointestinal overall**	Pancreatic	Hepatobiliary	**Hepatobiliary and pancreatic organs overall**
N	85	70	6	31	**192**	35	31	**66**
Response rate（%）	58%	43%	50%	29%	**47%**	17%	16%	**17%**
Median PFS（month）	5.8	4.9	7.3	3.7	**5.1**	3.2	4.1	**3.7**
MST（month）	13.4	13.3	29.7	7.6	**13**	7.9	8.5	**7.9**

2.1.5. Tumor-related complications

We, here, report the complications that require attention during patient management, according to the organ of primary lesion. It is a rare disease and frequencies remain unknown.

1）Esophageal primary

Tumor bleeding/pain/esophageal stenosis/fistula formation due to primary lesion in the esophagus; hoarseness/difficulty swallowing/aspiration pneumonia caused by recurrent laryngeal nerve palsy due to lesion in lymph node metastasis; jaundice and liver failure associated with liver metastasis; respiratory failure and hemoptysis associated with lung metastasis, airway constriction, and pain due to lymph node metastasis; hypercalcemia, tracheal stenosis, tracheal obstruction, and suture failure due to disease progression.

2）Gastric primary

Chronic bleeding from primary lesion, accompanied by anemia, stomach pain, nausea, vomiting, gastric perforation, bloating, suture failure, fistula formation, cardiac/pyloric stenosis, ascites retention due to peritoneal metastasis, ileus, hydronephrosis due to ureteral stenosis, obstructive jaundice/liver failure due to bile duct stenosis, and obstructive jaundice/liver failure due to hepatic portal lymph node metastasis.

3）Small intestine/colonic primary

Tumor hemorrhage, ileus, fistula formation in the small intestine/bladder/vagina, intestinal obstruction, intestinal stenosis, intestinal perforation, and pelvic infection

4）Hepatobiliary and pancreatic primary

Cancer pain, rupture, obstructive jaundice associated with the growth of primary tumor, weight loss, fever, pancreatitis, cholangitis, liver abscess, cholecystitis, biliary hemorrhage, duodenal hemorrhage, duodenal stenosis, anastomotic ulcer/stenosis/leakage, symptoms associated with stenosis/obstruction of portal vein (liver

dysfunction/liver failure, esophageal varices and their rupture, gastritis, ascites retention, and hepatic encephalopathy/coma associated with portal hypertension).

5) Paraneoplastic syndrome

In addition to NECs, there are reports of paraneoplastic syndrome that is often associated with small cell lung carcinoma[22-24]. We report examples of paraneoplastic syndrome seen with small cell lung carcinoma below.

Hyponatremia due to inappropriate sodium secretion, psychological symptoms (changes in personality) due to ectopic ACTH syndrome, hypertension, hypokalemia, high blood sugar, Lambert-Eaton myasthenic syndrome, paraneoplastic cerebellar degeneration associated with autoantibody production (ataxia in the limbs, dysarthria, and nystagmus), paraneoplastic encephalomyelitis/sensory neuron disease (dementia, cranial nerve symptoms, dizziness, ataxia, autonomic imbalance, transverse paralysis, and sensory disorders).

Apart from these, there are thromboembolic events, aspiration (due to gastrointestinal stenosis), anemia, tumor pain, acute renal failure, myositis, and pulmonary fibrosis.

6) Complications due to metastatic lesions

Liver failure, pain, hepatobiliary infection, hemorrhage, bile duct stenosis, bile duct obstruction, respiratory failure, tracheal hemorrhage, respiratory tract infection, atelectasis, airway constriction, ascites, bloating, ileus, ureteral stenosis, ureteral obstruction, urinary retention, urinary tract infection, hiccups, pleural effusion, pleural hemorrhage, chest pain, pathological fracture, pain, hypercalcemia, meningeal carcinomatosis, ataxia, ischemia cerebrovascular, intracranial hemorrhage, nausea, vomiting, dizziness, consciousness disorder, cognitive disturbance, dysphasia, seizure, spasticity, edema limbs, intestinal obstruction/stenosis/perforation, pancreatitis, disseminated intravascular coagulation, thrombocytopenia, anorectal infection, anal hemorrhage, hoarseness, and superior vena cava syndrome

7) Others

General pain management including narcotic analgesics is performed for cancer pain.

Gastrointestinal stenting and bypass surgery are performed for esophageal stenosis, cardiac/pyloric stenosis, and duodenal stenosis.

Percutaneous transhepatic cholangial drainage (PTCD), percutaneous or endoscopic stenting, and bile duct jejunostomy is performed for obstructive jaundice. In addition, complications associated with biliary drainage and gastrointestinal stenting are listed below.

- PTCD, including internal and external fistula tube placement:
  Cholangitis, pancreatitis, cholecystitis, liver abscess, sepsis, biliary hemorrhage, PTCD tube obstruction/deviation, peritonitis, and pneumothorax/pleurisy
- Biliary stenting:
  Cholangitis, pancreatitis, cholecystitis, liver abscess, sepsis, biliary hemorrhage, peritonitis, pneumothorax/pleurisy (for percutaneous stenting), duodenal perforation, pneumonia (for endoscopic stenting), stent obstruction/deviation, duodenal ulcer, and duodenal hemorrhage
- Bile duct jejunostomy:
  Cholangitis, pancreatitis, cholecystitis, liver abscess, and sepsis
- Gastrointestinal stenting:
  Hemorrhage, perforation, pain, stent deviation, stent obstruction, foreign-body sensation, intestinal strangulation, ulcer formation, fever, sepsis, infection, diarrhea, constipation, tenesmus or uncontrollable urination/incontinence symptoms (colon), thyroid injury (esophagus), carotid artery injury (esophagus), and mediastinal abscess (esophagus).

2.1.6. Recurrent/progression

NEC is a very rare disease, and most reports of cases of surgery coincide with the case reports. According to the review by Arai et al., out of the 55 patients with NEC with gastric primary lesion who underwent surgical resection, only three patients survived for two years or more[25]. According to the report by Fischer et al., the MST of 13 patients with NEC with pancreatic primary lesion who underwent surgical resection was 11.7 months (seven of the patients showed distal metastasis)[26]. The form of recurrence among surgery cases remains unknown. The multicenter joint

study by Yamaguchi and Machida reported PFS among cases that underwent systemic chemotherapy with respect to the median as 5.8 months for esophageal primary lesions, 4.8 months for gastric primary lesions, 7.3 months for duodenal small intestine primary lesions, 3.7 months for colonic primary lesions, 3.2 months for pancreatic primary lesions, and 4.1 months for hepatic/biliary primary lesions[10]. In the NORDIC NEC study, the median PFS was 3 months for esophageal primary lesions, 5 months for gastric primary lesions, 3 months for colonic primary lesions, 4 months for rectal primary lesions, 5 months for pancreatic primary lesions, and 4 months for cases with unknown primary lesion site [11].

2.1.7. Prognostic/predictive factors

When prognostic factors were examined by multivariate analysis in the multicenter joint study by Yamaguchi and Machida, out of the various factors such as sex, age (younger or older than 60 years), PS (0 or 1 vs. 2 or more), primary lesion organ (gastrointestinal primary lesions vs. hepatobiliary or pancreatic primary lesions), LDH levels (below vs. above the upper limit of facility standard), presence or absence of liver metastasis, presence or absence of history of radical resection and treatment regimen (IP therapy vs. EP therapy); the independent prognostic factors identified were gastrointestinal primary lesion (vs. hepatobiliary or pancreatic primary lesion, hazard ratio (HR): 0.58), and LDH levels being below the upper limit of facility standard for LDH (vs. above the upper limit of facility standard for LDH, HR: 0.65).

Although IP therapy exhibited slightly better impact on overall survival as compared to EP therapy with an HR of 0.8, the *p*-value of 0.389 meant that there was no significant difference between the two therapies[10]. The NORIDIC NEC study reported poor PS, colonic primary lesions, high platelet count, and high LDH levels to be the main factors behind poor prognosis[11]. However, these prognostic/predictive factors were not obtained with a global consensus.

2.1.8. Rationale for selection of the target population

The objective of this study is to develop a primary chemotherapy regimen for non-resectable/recurrent NECs, and out of the non-resectable or postoperatively recurrent NECs with gastrointestinal primary lesions or hepatobiliary or pancreatic primary lesions (shaded parts in Table 2.1.2 of WHO 2010 classification of NEC), we established chemotherapy-naïve patients to be the target population.

In terms of whether or not to consider MANEC a target of this study, a discussion is needed from the viewpoint of standard treatment. MANEC is considered when 30% or more of the carcinoma consists of adenocarcinoma components, and it is treated as either adenocarcinoma or NEC by discretion of the attending physician, with no real consensus on standard treatment. MANEC was however, excluded from this study, as its disease concept is different from NEC in terms of the standard treatment, which has a consensus regarding the treatment by a "regimen according to small cell lung carcinoma". In fact, a questionnaire was provided to the three groups participating in this JCOG study (response obtained from 52 facilities). The results showed that 79% of facilities considered the WHO 2010 classification of NEC as the appropriate tissue type for this study, while only 13% responded that consideration of NEC+MANEC would be appropriate. Therefore, majority of facilities considered "WHO 2010 classification of NEC as the appropriate target of this study".

While diagnosis of NECs is generally carried out using tissue samples and biopsy samples, as mentioned in 2.1.3, there is no consensus methodology to strictly distinguish NEC and MANEC using biopsy samples. As a result of group discussions, a consensus was reached between the three groups that cases presenting with NEC components by biopsy sample-based diagnosis can be enrolled. Although the use of this method would mean that a certain percentage of patients with MANEC, who were not intended to participate in this study, would be enrolled however, currently there are no appropriate means to avoid this. Therefore, we decided to create a consensus for the future based on information obtained from this study.

1) **<u>Reason for targeting gastrointestinal/hepatobiliary or pancreatic primary lesion NEC</u>**

Various guidelines recommend treatment regimens suited for small cell lung carcinoma, regardless of the organ of primary lesion, and is the rationale showing the validity of the treatment development for NEC across many organs. Furthermore, taking into consideration the frequency of the disease, it is unrealistic to develop treatments according to each organ, and in practice the Minnie-Pearl Cancer Research Network Study (mentioned later in 2.2.2), which is a relatively large-scale clinical study of NEC, development of treatment was carried out in a cross-organ

manner[27].

On the other hand, if we take hypothesize that we do not sufficiently understand how the clinical presentations of the disease vary according to the organ, the fact that this is the first randomized study in Japan, and that the maintenance of foundation for clinical studies of this disease in Japan is inadequate at present time, the hurdle is too high to plan a study across all organs including the fields of gynecology and urology. Based on the above consideration, we have decided to target all gastrointestinal organs which have a relatively large number of common points between each other in terms of clinical presentation and types of complication.

According to the multicenter observatory study by Yamaguchi and Machida, the prognosis of NEC with hepatobiliary or pancreatic primary lesion was significantly poorer than that of NEC with gastrointestinal primary lesion (MST: 7.9 vs. 13.0 months, respectively), but we determined that it is possible to accurately evaluate the efficacy of the treatment regimen by randomizing the subjects using the primary lesion site (gastrointestinal vs. hepatobiliary or pancreatic) as an allocation adjustment factor. Therefore, we considered patients with both, gastrointestinal/hepatobiliary or pancreatic primary lesions as one target population of this study, and to examine differences between organs exploratively by subgroup analysis.

2）Reason for including hepatic NEC cases (hepatic primary lesion or liver metastasis with primary lesion site unknown)

Even among NECs, cases with hepatic primary lesions are particularly rare. There are no specific reports on the frequency of incidence and differences from the other organs and reports are limited only to case reports and their reviews. Moreover, since the liver is a major organ for metastasis of primary lesions into other organs, even when tumors have been clinically identified in the liver, in many cases the primary lesion tends to be in another organ. Therefore, liver should not be deemed the primary lesion site without sufficient examination for a primary lesion. On the other hand, although very rare, there have been reports of NEC with hepatic primary lesion[28]. Even if sufficient search for primary lesion leads to no indication of the same outside of the liver, currently it is not possible to distinguish whether it is a case of “NEC with hepatic primary lesion” or “lesion in the liver is a metastatic lesion, with the primary lesion unknown”. In this study, such situations wherein “liver alone has identifiable lesions” would be henceforth, referred to as “hepatic NEC (hepatic primary lesion or liver metastasis from an unknown primary lesion site) for convenience. As the NCCN Guidelines[19] and ENETS Guidelines[20, 21] indicate the usefulness of FDG-PET for detailed examination of the primary lesion, we would also examine the primary lesion using FDG-PET. Furthermore, by referring to the diagnostic procedures for cancers with unknown primary lesion site, detailed examinations into the primary lesion would be carried out by otolaryngological (head and neck) examination and urological examination for men only, and gynecological examination for women only).

Such hepatic NEC (hepatic primary lesion or liver metastasis from an unknown primary lesion site) is anticipated even from an anatomical viewpoint, since the tumor-related complications are the same as that for other gastrointestinal primary lesion NECs, and there is no issue with treating them the same as for gastrointestinal primary lesions.

3）Reason for orienting the study for non-resectable or recurrent cases

As mentioned in 2.1.4 “Standard treatment according to disease staging and outline of prognosis”, systemic chemotherapy is indicated for cases with distal metastasis or recurrent cases. Locally advanced cases are particularly prone to pancreatic or bile duct primary lesions situated in the vicinity of vital vessels, and while the NCCN Guidelines propose chemoradiotherapy according to treatment for small cell lung carcinoma[19], the ENETS Guidelines provide no clear indication[21]. In reality, for the treatment of NEC which has a variety of primary lesion organs (scope of irradiation) there is not enough information available for the chemotherapy regimen that should be combined in terms of its optimum dose, the radiation dose, efficacy, and safety. For this reason, chemoradiotherapy cannot be considered the standard treatment with consensus, and systemic chemotherapy for distal metastasis is used widely except for pathologies involving frequent stenotic symptoms when the primary lesion is in the esophagus. For this reason, this study would enroll non-resectable or recurrent cases that include locally advanced cases.

The section “3.6 Definition of non-resectable NEC” shows the definition of non-resectable cases summarized by referring to the JCOG protocol for clinical studies of systemic chemotherapy (esophagus: JCOG0807, stomach: JCOG1013 or JCOG1002, bile duct: JCOG0805, pancreas: JCOG1106). Furthermore, while radical resection has

been considered for cases of colonic primary lesion adenocarcinoma with liver or lung metastasis, as cases of NEC with distal metastasis are not indicated for resection, we defined non-resectable cases as those in "Stage IV", and followed this definition for duodenal primary lesions, small intestine primary lesions, and appendix primary lesions. Furthermore, with respect to NEC with esophageal primary lesion, chemoradiotherapy is well-indicated for cases with supraclavicular lymph node metastasis of thoracic esophageal tumor even in Stage IV, and hence were not included in this study.

4) Treatment of draft of new classification of NEC

In recent years, proposals have been made to further differentiate the WHO 2010 classification of NECs (G3 in ENETS) to "Grade 3 proliferative tumors showing the same morphological presentations as NET" and "Grade 3 tumors with strong morphological atypicality (previously classified as poorly differentiated NECs)"[29, 30], or by Ki-67 index of 20–50% and 50% or more[11, 19]. This is yet to be confirmed; however, in this study we would have used the WHO 2010 classification. However, we would have used samples collected for central pathological diagnosis to carry out studies related to classification which exploratively adds grade classification and morphological differentiation levels.

2.2. Standard treatment for target disease

Currently, there is no standard drug treatment for non-resectable/recurrent NECs with efficacy verified by a randomized controlled trial. However, given the similarity between pathological and clinical presentations, treatments according to small cell lung carcinoma have been attempted, and there have been reports of positive treatment outcome in small-scale clinical studies and observational studies.

2.2.1. Standard treatment for small cell lung carcinoma

Cisplatin-based multidrug combined therapy is the standard first-line treatment of extensive disease (ED) small cell lung carcinoma. While cyclophosphamide/doxorubicin/vincristine (CAV therapy) was established as a standard treatment in the 1970s in Europe and U.S., the etoposide/cisplatin combined therapy (EP therapy) was introduced in the late 1980s. A comparative study of EP therapy and CAV therapy did not show superiority of EP therapy over CAV therapy in terms of survival, but the response rate (CAV: 51% vs. EP: 61%) and MST (CAV: 8.3 months vs. EP: 8.6 months) were almost the same, and the lighter toxicity level (mucositis, interstitial pneumonia, hemotoxicity) meant that EP could be used as a standard treatment[31]. Thereafter, the JCOG Lung Cancer Group carried out the "comparison between EP therapy and Irinotecan/Cisplatin combined therapy (IP therapy) for Extensive-stage Small Cell Lung Cancer (JCOG9511)", and reported that the IP therapy provided a significantly better overall survival, with MST being 9.4 months vs. 12.8 months (p = 0.002) for IP therapy[32]. However, the two additional large-scale studies conducted primarily in U.S. did not show superiority of IP therapy over EP therapy, and for this reason IP therapy has not been not used as the standard treatment overseas[33, 34]. Furthermore, while the results of the "Randomized controlled trial to verify the non-inferiority of amrubicin/cisplatin combined therapy (AP therapy) over IP therapy (JCOG0509)" were reported in the 2012 conference of the American Society of Clinical Oncology (ASCO), but AP therapy was still considered significantly inferior to IP therapy[35]. Presently, the JCOG Lung Cancer Group considers IP therapy to be the standard treatment for extensive-disease small cell lung carcinoma.

2.2.2. Standard treatment for extrapulmonary NEC

While there are scattered reports relating to use of CAV therapy, EP therapy, IP therapy, and other multidrug combined therapies for extrapulmonary NEC, these reports are primarily from observational studies. Since these reports precede the unification of concept and classification methods of this disease, the nomenclature such as NEC (anaplastic type), NET (poorly differentiated type) and extrapulmonary small cell lung carcinoma have still been used. These reports, however, are presumed to be targeting almost the same disease group as NEC. There are no reports of prospective studies using EP therapy for extrapulmonary NEC. Observational studies have reported response rates between 42–67%, and MST between 15–19 months (Table 2.2.2a). On the other hand, IP therapy has reported results from observational studies and clinical studies, with response rates between 7–83% and MST of 10.1–22.6 months (Table 2.2.2b). As mentioned above, reports related to extrapulmonary NEC are limited to use of EP therapy and IP therapy in observational studies and small-scale clinical studies. The largest number of case enrollments in a clinical study of extrapulmonary NEC was seen in the Minnie-Pearl Cancer Research Network Study, which evaluated the efficacy of triple-drug combined therapy using carboplatin/etoposide/paclitaxel. The

response rate to this triple-drug combined therapy was 53%, while the MST was 14.5 months and adequate, there was intense toxicity involved, and the results were not significantly different from EP therapy, so this regimen was not considered the standard treatment[27].

The 2014 NCCN Guidelines recommend treating extrapulmonary NEC using a regimen according to small cell lung carcinoma, and the guidelines for treatment of small cell lung carcinoma mention both EP therapy and IP therapy as recommended regimens.

Therefore, the standard treatment for extrapulmonary NEC is taken as EP therapy or IP therapy.

Table 2.2.2.a. EP therapy for NEC (observational studies)

Reported year/Reporter	Target	N	Response rate	MST (in months)
1991/ Moertel[18]	Pancreatic/gastrointestinal NEC (anaplastic type)	18	67%	19
1999/ Mitry [36]	Pancreatic/gastrointestinal NEC	41	42%	15
2001/ Marie-Louise [37]	Pancreatic NET (well: 11, poorly: 4) Gastrointestinal cartinoid: 21	33	18%	19
1994/ Lo Re G [38]	Extrapulmonary SCLC	13	69%	NE
2010/ Iwasa [39]	Hepatobiliary or pancreatic NEC	21	14%	7.3
2012/ Yamaguchi [10]	Gastrointestinal NEC	12	75%	14
2012/ Yamaguchi [10]	Hepatobiliary or pancreatic NEC	34	12%	6.9
2012/ Sorbye [11]	Gastrointestinal primary lesion NEC (including primary lesion unknown)	129	31%	12

Table 2.2.2.b. IP therapy for NEC (shaded are clinical studies, others are observational studies)

Reported year/Reporter	Target	N	Response rate	MST (in months)
2003/ Hou [40]	NEC (gastrointestinal-80%)	18	43%	NE
2005/ Chin [41]	Esophageal NEC	12	83%	14
2011/ Okita [42]	Gastric NEC	12	75%	22.6
2012/ Yamaguchi [10]	Gastrointestinal NEC	142	51%	13.4
2012/ Yamaguchi [10]	Hepatobiliary or pancreatic NEC	18	39%	10.1
2006/ Kulke [43]	Pancreatic/gastrointestinal (including NET G1/G2)	15	7%	11.4
2008/ Mani [44]	Pancreatic/gastrointestinal NEC	20	58%	NE
2008/ Jin [45]	Extrapulmonary NEC	15	67%	11.4.

Table 2.2.2.c. Phase II study of carboplatin/etoposide/paclitaxel triple-drug combined therapy for NEC

Reported year/Reporter	Target (breakdown)	N	Response rate	MST (in months)
2006/ Hainsworth [27]	NEC (Colon: 9, lung: 7, skin: 4, pancreas: 3, gall bladder: 1, thyroid:1, stomach: 1, esophagus: 1, endometrium:1, maxillary sinus: 1, prostate: 1, primary lesion unknown: 48)	78	53%	14.5

2.2.3. Standard treatment for gastrointestinal/hepatobiliary or pancreatic primary NEC

Treatments for extrapulmonary NEC have not been developed according to any specific organ, and the standard treatment for gastrointestinal/hepatobiliary or pancreatic NEC is also both, EP therapy and IP therapy. While both treatments are considered standard treatments, since this study considers them both to be study treatments, the anticipated effect and expected adverse reactions from EP therapy and IP therapy have been described in “2.3.2 Study treatment(s) of this study”.

Herewith, we describe the current status of the usage of each treatment in Japan and overseas. According to the multicenter joint study by Yamaguchi and Machida, 160 out of 258 patients (62%) who received systemic chemotherapy underwent IP therapy, the most common treatment, followed by EP therapy (46 patients, 18%). Although there is big deviation where 92% of gastrointestinal primary lesion NEC (142/154 patients) were given IP therapy, and 65% of hepatobiliary or pancreatic primary lesion NEC (34/52 patients) were given EP therapy, the present situation shows that treatments are being selected by discretion or preference of the facility/physician.

Furthermore, even with respect to the dosing schedule, the method of administration has not necessarily as per the treatment of small cell lung carcinoma. There is no unified consensus, as some facilities use methods used for treatment of gastric cancer (JCOG9912 regimen), based on the reasoning that they have familiarity with IP therapy. On the other hand, in the NORDIC NEC study the most common treatment used was the EP therapy, which was administered to 129 out of 252 patients (51%) who received systemic chemotherapy, followed by a combined therapy consisting of carboplatin and etoposide therapy (67 patients, 27%). Combined therapy of platinum and etoposide was used often, which is another choice of treatment in Japan[11].

2.3. Rationale for establishment of treatment plan

2.3.1. Drugs

1) Etoposide

Etoposide exerts an antitumor effect by inhibiting topoisomerase II, which catalyzes the untangling of supercoiled DNA strands. Main toxicities include myelosuppression, nausea/vomiting, alopecia, and stomatitis.

2) Cisplatin

It is a complex ion form of the heavy metal platinum which shows anti-tumor effect by cross-linking double-stranded DNA. Currently, it is considered one of the key drugs for NEC treatment, being a central medicine for the treatment of lung cancer due to its synergistic effect with radiation therapy and various drugs, and also due to its low bone marrow toxicity when used alone. Toxicities include nausea/vomiting, nephrotoxicity, and neurotoxicity. Sufficient infusion of cisplatin before and after drug administration is necessary to prevent nephrotoxicity.

3) Irinotecan

Irinotecan is a topoisomerase I inhibitor developed in Japan that inhibits DNA synthesis. The drug is directly converted into the active metabolite (SN-38) in human liver and various tissues by carboxyesterase. As it shows a potent antitumor effect against SCLC even when used on its own, this drug is used very often in routine clinical practice to treat NEC as well. Diarrhea and myelosuppression are observed as dose-dependent toxicities. Nausea/vomiting and interstitial pneumonia have also been noted in patients.

2.3.2. Study treatment(s) of this study

1) Etoposide/cisplatin combined therapy (EP therapy)

In the multicenter joint study performed by Yamaguchi and Machida, the response rate of gastrointestinal primary lesion NEC to EP therapy was 75% (9/12 patients), with an MST of 14 months. The response rate of hepatobiliary and pancreatic primary lesion NEC to EP therapy was 12% (4/34 patients), and the MST was 6.9 months. With respect to safety, the multicenter joint study by Yamaguchi and Machida showed no treatment-related deaths associated with EP therapy (N = 46) administered as first-line treatment for gastrointestinal/hepatobiliary and pancreatic primary lesion NEC, and toxicity-related study discontinuation was observed in 6.5% of cases. Details of toxicities remain unknown as they were not investigated. The observational study of EP therapy for hepatobiliary and pancreatic primary lesion NEC (N = 21) carried out by Iwasa et al. at the National Cancer Center Hospital showed major Grade 3/4 adverse events to be neutropenia (90%), nausea (33%), and anorexia (24%). Grade 3 febrile neutropenia was observed in 8 patients (38%)[39]. Table 2.3.2 shows the toxicity profile of EP therapy from the JCOG9511 study which treated small cell lung carcinoma (Grade 3 or higher as per JCOG toxicity criteria).

Table 2.3.2. Toxicity of EP therapy and IP therapy in studies treating small cell lung carcinoma※
※Report by Iwasa et al., JCOG0509：CTCAE v3.0 Grade 3 or higher, JCOG9511：JCOG toxicity criteria Grade 3 or higher

	EP therapy		IP therapy	
	Report by Iwasa et al.[34]	JCOG9511[27]	JCOG9511[27]	JCOG0509[30]
Neutropenia	90%	92.2%	65.3%	58.5%
Leukopenia	71%	51.9%	26.7%	22.5%
Decreased hemoglobin	29%	29.9%	26.7%	23.2%
Thrombocytopenia	24%	18.2%	5.3%	2.1%
Diarrhea	0%	0%	16%	7.7%

Nausea	33%	6.5%	13.3%	6.3%
Aspartate aminotransferase increased	19%	2.6%	0%	-
Alanine aminotransferase increased	24%	3.9%	4%	-
Blood bilirubin increased	19%	0%	0%	-
Creatinine increased	0%	0%	0%	-
Peripheral motor neuropathy	0%	0%	0%	-
Febrile neutropenia	38%	0%	0%	1.4%

There are several reports concerning the specific dosage schedule/administered dose for EP therapy, and four dosing methods have been described in the NCCN Guidelines for SCLC as well. However, it is not clear as to which of the dosing methods is the best, and the dosing method for NEC varies between reports. In Japan, the dosing for EP therapy according to JCOG9511 is also often used for NEC. For this reason, this study would also follow the same method of administration. In terms of the number of cycles, a comparison of 4 courses vs. 8 courses of combined chemotherapy including cyclophosphamide for SCLC showed poor efficacy even when the treatment continued for long-term[46], and treatment of 4 cycles has also been specified in JCOG9511. There have been no such comparative studies for gastrointestinal/hepatobiliary and pancreatic primary lesion NEC, rather, there is no evidence to suggest that first-line chemotherapy should be discontinued if the chemotherapy is efficacious and toxicity is within the permitted scope. Unlike SCLC, NEC does not respond well to chemotherapy and tends to re-exacerbation to the underlying disease soon after the end of chemotherapy. For this reason, continuation of chemotherapy is very likely to be beneficial for patients with NEC as long as the treatment is effective.

While peripheral motor neuropathy, hearing impairment, and renal disorder are known accumulation toxicities of cisplatin, if sufficiently safe, it is ideal for treatments to continue as much as possible, considering the very few treatment options available for NEC. Based on the above rationale, we decided to ensure safety by establishing strict protocol treatment discontinuation criteria and chose not to limit the total dose or prescribe the number of treatment cycles.

2）Irinotecan/Cisplatin combined therapy (IP therapy)

In the multicenter joint study by Yamaguchi and Machida, the response rate of gastrointestinal primary lesion NEC to IP therapy was 51% (73/142 patients), with an MST of 13.4 months. The response rate of hepatobiliary and pancreatic primary lesion NEC to IP therapy was 39% (7/18 patients), and the MST was 10.1 months. There were no treatment-related deaths among 160 patients, and discontinuation due to toxicity in first-line chemotherapy was seen in 11.2% of cases. Details about toxicity were not collected. As a reference, Table 2.3.2 shows the toxicity profile of IP therapy from the JCOG9511 and JCOG0509 studies which treated small cell lung carcinoma.

There are several reports concerning specific dosing methods for IP therapy, and two dosing methods have been described in the NCCN Guidelines for SCLC as well. It is not known which of the dosing methods is the best, and the dosing method has also been variable in the NEC. In Japan, the dosing method of IP therapy, according to JCOG9511 is considered the standard treatment for SCLC and the same method has also been used in this study.

The total dose and number of cycles would have not been prescribed for the same reason as EP therapy, and safety has been ensured by establishing strict protocol treatment discontinuation criteria.

3）Other study treatment candidates

Presently, there are no drugs or treatment methods, including molecular-targeted drugs, which surpass the EP and IP therapy. There are also no planned or ongoing large-scale clinical trials globally, related to first-line treatment of NEC.

As the second-line treatment, a phase II study of Everolimus is underway for pancreatic primary lesion NEC resistant or unresponsive to platinum preparations. It is a multicenter study being carried out in 31 facilities in Japan, led by the National Cancer Center East Hospital, through the cancer research and development fund (UMIN000012752）.

2.3.3. Summary of risk/benefit balance of the standard treatment and study treatment

In the multicenter joint study by Yamaguchi and Machida, IP therapy had better prognosis compared to EP therapy (MST: 13.0 months vs. 7.3 months; $p < 0.0001$). However, as shown in Table 2.3.3, since IP therapy was more frequently chosen for gastrointestinal primary lesions (142/154 patients) and EP therapy for hepatobiliary and

pancreatic primary lesions (34/52 patients), it has not been possible to determine whether this difference is due to difference in efficacy between the regimens, or due to differences in the primary lesion organ. Primary lesion organ remained a significant prognostic factor after multivariate analysis, and while IP therapy turned out to be a slightly better regimen than EP therapy with HR = 0.8 (95% CI. 0.48–1.33), $p = 0.389$ indicated that there was no significant difference between the two (see "2.1.5 Prognostic/predictive factors"). Taking into account the number of EP therapy cases (46 patients), the fact that it was an observational study, and including other unknown bias, the data interpretation is fairly limited. Therefore, based on these results we are unable to deem either of IP or EP therapies to be more efficacious than the other.

With regard to toxicity, while myelosuppression such as neutropenia is milder in IP therapy than EP therapy, frequencies of events such as diarrhea and nausea are high. While the two treatments have different toxicity profiles, it is difficult to conclude that one has clearly worse toxicity than the other. The risk of bile duct obstruction is high in biliary and pancreatic primary lesion NECs and the use of irinotecan (which undergoes biliary excretion) in patients exhibiting biliary excretion disorder runs the risk of increased toxicity, hence care must be taken while administering irinotecan. We believe that this risk can be avoided by appropriate pre-treatment drainage and proper monitoring during treatment. Based on the above, it is difficult to assign superiority to IP therapy or EP therapy over the other in terms of risk/benefit balance, and both the regimens can be considered suitable standard treatments.

Table 2.3.3. Summary of results from multicenter study by Yamaguchi and Machida

		IP	EP	*p*-value*
Total	N	160	46	
	Response rate（%）	50	27	＜0.001
	Median PFS (in months)	5.2	4.0	0.033
	Median OS (in months)	13.0	7.3	＜0.0001
Gastrointestinal primary lesion NEC	N	142	12	
	Response rate（%）	51	75	0.14
	Median PFS (in months)	5.4	4.9	0.585
	Median OS (in months)	13.4	14.0	0.976
Hepatobiliary and pancreatic primary lesion NEC	N	18	34	
	Response rate（%）	39%	12%	0.034
	Median PFS (in months)	4.4	3.7	0.056
	Median OS (in months)	10.1	6.9	0.05

*Response rate by chi-squared test, PFS and OS by log-rank test

2.3.4. Post-treatment(s)

In the multicenter joint study by Yamaguchi and Machida, 56% (116/206 patients) of patients with NEC who received IP therapy or EP therapy were administered chemotherapy as the second-line of treatment. The most common second-line chemotherapy after IP therapy was amurcibin (22/88 patients), while irinotecan was the most common second-line treatment after EP therapy (13/28 patients), and we expected a similar pattern for the second-line treatments in this study as well. The overall performance of second-line chemotherapy was poor with a response rate of 11%, and PFS of 2.1 months. As such, the significance of the second-line treatment has not been indicated, and hence, no standard treatment has been established.

2.4. Study design

2.4.1. Rationale for establishing endpoints

For NECs, an alternative endpoint for overall survival, such as PFS has not been established as an indicator for comparing the usefulness of the treatment regimens. Therefore, comparison of overall survival was considered appropriate when examining the therapeutic effects. For the same, overall survival was examined as the primary endpoint. The secondary endpoints to evaluate the efficacy and safety were the response rate (only for patients with a measurable lesion), progression-free survival, incidence rate of adverse events, and dose intensity of cisplatin.

2.4.2. Clinical hypothesis and rationale for setting the number of enrollments

This study compares the two standard treatments, both of which are difficult to deem superior than the other in terms of efficacy and safety. We have, therefore, adopted a study design using bilateral testing. The main clinical hypothesis of this study is that either IP therapy or EP therapy is likely to provide better overall survival than the other treatment group, and should this hypothesis be validated, the superior treatment with statistical significance would be deemed the better treatment, and would therefore be positioned as the standard treatment in future. If the study fails to validate this hypothesis, it would indicate that there was no clinically significant difference in overall survival between the two groups, and if there are no large differences in terms of toxicity, we would conclude that both treatments are viable options as the standard treatment. However, if unlike our initial hypothesis, one treatment shows clearly more toxicity than the other, we would reconsider the study design before carrying out the main analysis.

Furthermore, as mentioned in "section 2.1.1 Epidemiology", gastroenterological primary lesion NEC, which is the primary target of this study, is a very rare disease with an annual prevalence of around 3 in 100,000 people, hence, the significance level of testing has been placed at 10% bilaterally, instead of 5% bilaterally. In the multicenter joint study conducted by Yamaguchi and Machida, the MST of gastrointestinal/hepatobiliary or pancreatic primary lesion NEC was 11.5 months. To determine if one treatment is superior to the other, we expected a difference of four months in terms of MST. If we expect the more inferior treatment to have an MST of 8.0 months and the superior treatment to have an MST of 12.0 months, the number of enrollments are calculated using the aforementioned parameters and based on the discussion mentioned later (see "12.2 Expected number of enrollments/Enrollment period/Follow-up period"). This provided a research period of 7- years, consisting of 6-years of enrollment period and one year of follow-up period, and with $\alpha = 0.1$ bilaterally and detection power of 70% to detect differences between the two groups, 63 subjects were required per group. Taking into consideration some subjects who could be lost post follow-up, we aimed to enroll 70 subjects per group, and a total of 140 subjects between two groups.

If enrollment proceeds better than expected than prior to the start of the study (i.e. if the number of enrolled subjects reach 70 in less than 2.5 years from the start of enrollment), the number of enrollments is likely to be re-established by changing the detection power from 70% to 80% during the study, with the aim of obtaining more accurate results.

＜Addition to ver.1.1＞

The pace of enrollment after the start of enrollment period exceeded expectations, and the number of enrollments reached 70 subjects in October 2016, which was 2 years and 2 months after the start. As this exceeded the criteria of 70 subjects in less than 2.5 years, since the start as provided above, we obtained the approval of the Hepatobiliary and Pancreatic Oncology Group Meeting on October 29, 2016, Esophageal Cancer Group Meeting on November 19, 2016, and Gastric Cancer Group Meeting on January 7, 2017 to change the detection power to 80% according to provisions at the time of study planning. Consequently, the number of intended enrollments was changed to 170 subjects.

2.4.3. Expected patient enrollment

In the multicenter joint study by Yamaguchi and Machida, 258 cases of gastrointestinal/hepatobiliary or pancreatic primary lesion NECs were reported from 23 sites during the 11 years between 2000 and 2011, but between 2000 and 2006, information could not be obtained because of old cases. When limited to the most recent five years (2006–2011), when sufficient information was obtained from each facility, the number of enrollments was placed at162 subjects.

This is a joint study between three groups, namely the JCOG Hepatobiliary and Pancreatic Oncology Group,

JCOG Gastric Cancer Group, and the JCOG Esophageal Cancer Group. Therefore, the total number of facilities, excluding overlaps, amounted to 82 facilities. Compared to the multicenter joint study by Yamaguchi and Machida, we expect an increase in the number of patients enrolled, and simple calculations lead us to extrapolate 115 subjects to be enrolled annually. On the other hand, if we take into account the possibility of patient enrollments being biased to some high volume centers, it would be difficult to estimate the effect of increasing facility count to proceed. In addition, considering that the study is a randomized, we estimate the annual number of patients enrolled to be between 30–50 people. Taking into account ineligible cases, we provided 6-years for patient enrollment period.

2.4.4. Rationale for setting allocation adjustment factors

1) Facility

It is widely known that background, treatment, efficacy evaluation, and safety evaluation of enrolled patients vary depending on the facility, and JCOG standards have been used to make adjustments between facilities.

2) Primary lesion organ (gastrointestinal tract vs. hepatobiliary or pancreatic organ)

In the multicenter joint study by Yamaguchi and Machida, multivariate analysis using the Cox proportional hazard model was carried out with 183 subjects, which indicated primary lesion organ (gastrointestinal tract vs. hepatobiliary or pancreatic organ) to be a significant prognostic factor.

2.4.5. Centralized pathological diagnosis

In this study, a centralized pathological diagnosis would be performed for analysis of NEC, despite patients being diagnosed by a third party. The operation of the centralized pathological diagnosis is described in section 15.1, while details of the operation have been described in the Centralized Pathological Diagnosis Procedure Manual. Furthermore, an analysis of endpoints based on the results of centralized pathological diagnosis would be provided as a reference.

2.5. Summary of expected advantages and disadvantages associated with study participation

2.5.1. Expected advantages

Drugs used for both groups of this study are treatments used in routine medical practice. As mentioned in the next section, although it would be necessary to use some drugs for which insurance coverage does not apply, since insurance claims for these treatments are made in a similar manner to general medical care however, in practice they do not receive insurance assessments.

Furthermore, cases of off-label medication have been studied according to “18th Case Providing Review Information” at the Case Review Committee established by the Health Insurance Claims Review & Reimbursement Services. Based on the review information provided as on February 26, 2018;, The use of “irinotecan hydrochloride hydrate, etoposide, cisplatin, and carboplatin [injections]” is approved for treatment of “neuroendocrine carcinoma”], and the notices issued by the Ministry of Health, Labor and Welfare on February 26, 2016the results of this study have been considered valid by the ministry. For the same reason, essentially since February 26, 2018, there have been no concerns about claims of insurance assessments.

Moreover, since the medical fees of study participants during the study, which includes drug fees, are paid in principle by the patients themselves and their insurance coverage, so the patients would receive no special medical or financial benefits from participation in this study.

2.5.2. Expected risks and disadvantages

Both treatment arms A and B would receive chemotherapy regimen used in routine medical practice, so they would be unlikely to be exposed to special risks or disadvantages not observed in routine medical practice. The descriptions in “2.3.3 Risk/benefit balance of standard treatment and study treatments” outline the expected risks and disadvantages for each treatment arm.

To minimize the risk of adverse events and disadvantages, the “ Patient selection criteria (Section 4)”, “ Criteria to change treatment (Section 6.3)” and “Concomitant/Supportive therapies (Section 6.4)” have been carefully considered for the three groups. Furthermore, the Institutional Review Board would monitor if the adverse events are within the scope of expectation, while any serious adverse events or unexpected adverse events would be carefully examined and reviewed according to the provisions related to “JCTN-Adverse Events Report Guideline”

and "JCOG Guidelines for Handling Clinical Safety Information", and a system has been provided to take any necessary countermeasures.

Precautions related to etoposide, irinotecan, and cisplatin

As of December 2013, etoposide therapy, which is the intended to be used in this study had not received insurance approval for the treatment of gastrointestinal or hepatobiliary and pancreatic primary lesion cancers. Irinotecan has also not received insurance approval for treatment of cancers with primary lesions in organs other than the stomach, colon, and rectum. Furthermore, as cisplatin is only indicated for esophageal, gastric cancer, and for biliary tract cancers when combined with gemcitabine (25 mg/m^2), the dosage and administration used in this study have not been approved previously.

As each facility in this study may carry out the insurance claims in the same manner as routine general medical practice, the treatment may receive insurance assessment after-the-fact. However, if a facility incurs losses, the loss must be borne by the relevant facility (medical institution), as there is no system of compensation arranged by the JCOG Research Organization. If actual losses occur, the continued participation in the study would then be carefully discussed between the facility supervisor and the principal investigator/clinical trial secretariat. The facility supervisor would be expected to gain approval from the facility IRB and the head of the medical institutions.

2.6. Significance of this study

While both, EP therapy and IP therapy are standard treatments, the efficiency of either treatment being better than the other is not yet clear, and currently in routine medical practice, the choice of treatment is being made on the discretion or preference of the facility/attending physician.

If this study is able to clearly indicate the efficiency of EP therapy or IP therapy over the other, we expect that the diminished use of the relatively inferior treatment would contribute to improvement in patient prognosis. If the study indicates no clinically meaningful differences between the two treatments, the results would provide evidence that there is no significant issue with the treatments being chosen by discretion or preference of the facility/attending physician, which has been done traditionally without any data that directly compare the two treatments, although that would not indicate that the effects of the two treatments are equivalent. We also expect that the establishment of a highly reliable standard treatment in this study which would provide a foundation for therapeutic development when promising treatment regimen in the future.

As NEC is a rare disease, and since each clinician is not likely to have adequate experience, we believe that work associated with multicenter studies such as confirmation of diagnosis by centralized diagnosis/results feedback and sharing results of treatments by unified treatment regimen would lead to improvement of quality of care for NEC in Japan.

2.7. Associated research (including sample analysis research)

No such studies have been planned or carried out at the time of preparation of the protocol.

2.8. JCOG-Biobank Japan (BBJ) collaborating biobank

This study would participate in the banking of blood samples (DNA/plasma) through the JCOG-BBJ collaboration biobank based on the protocol common to all JCOG studies (hereafter termed as "common banking").

Common banking by JCOG-BBJ collaborating biobank would collect and store the samples of patients enrolled in clinical studies conducted by JCOG regardless of presence/absence of pre-planned sample analysis research, would also provide samples for future analysis research and diagnostic information obtained through the main research.

The target of such biobanking are those patients who provided consent to participate in this study and gave consent to provide samples to the JCOG-BBJ collaborating biobank for their use in future sample analysis research (hereafter termed as consent for banking).

Samples collected during common banking include whole blood and preserved pathological tissue samples from routine clinical practice. Plasma and DNA separated/isolated from blood would be stored in the JCOG-BBJ collaborating biobank and would be provided for sample analysis research in the future. Preserved pathological tissue samples from routine medical practices such as surgery, and biopsy/clinical laboratory tests would also likely to be used for future sample analysis research. Although the type, sample preparation methods, and tissue quantity required would vary from study to study, there is no definite consensus that banking pathological tissue in a certain

method is more efficient than others. Furthermore, it has been suggested that long-term preservation of a sectioned specimen from preserved pathological tissue may lead to sample deterioration (DNA fragmentation). The discussions between the of JCOG and BBJ personnel about these problems, led to the conclusion that preserved pathological tissue taken after medical examination shall require only patients' consent for future use, and actual collection must be started after standardizing a separate protocol and specifying in it the procedures best suited for the details of the research.

The detailed procedures for sample collection, storage, and method of sample provision for future sample analysis research in common banking have been stipulated in the "JCOG-BioBank Japan Collaborating Biobank Protocols" that apply to all the JCOG studies. To participate in the common banking carried out by JCOG-BBJ collaborating biobanks, the subject matter must be reviewed and approved by the ethics committees of each participating facility.

Furthermore, to conduct sample analysis research in future using samples stored in the JCOG-BBJ collaborating biobank, it would be necessary to prepare a "Sample Analysis Research Protocol" and have it reviewed and approved by the JCOG Protocol Review Committee and the ethics committee of facilities involved in the sample analysis.

3. Criteria/definitions used in this study

Tissue classification would be performed according to WHO 2010 classification and ENETS（European Neuroendocrine Tumor Society） classification[14, 15], while disease staging would be done according to "UICC-TNM 7th Edition".

3.1. Tissue classification (WHO 2010 classification)

The shaded parts are the targets of this study

Neuroendocrine neoplasms

1) Neuroendocrine tumor：NET Grade 1（NETG1）
2) Neuroendocrine tumor：NET Grade 2（NETG2）
3) Neuroendocrine carcinoma：NEC（large cell or small cell type）
4) Mixed adenoendocrine carcinoma（MANEC）
5) Hyperplastic and preneoplastic lesions

3.2. Grade classification （ENETS [European Neuroendocrine Tumor Society] / WHO2010 classification）

Grade 1（G1） Number of mitotic presentations < 2 per 10 high power fields（HPF）and/or Ki-67 index ≤2%*
Grade 2（G2） Number of mitotic presentations 2–20 per 10 HPF and/or Ki-67 index 3-20%
Grade 3（G3） Number of mitotic presentations > 20 per 10 HPF and/or Ki-67 index >20%

*The description of number of mitotic presentations in WHO 2010 classification is ≤2, but 2–3% are classified to G1[47].

3.3. Histopathological diagnosis

- Immunostaining (Chromogranin A and synaptophysin) is essential for a pathological diagnosis of NEC.
- Either1. or 2., or both are studied to determine proliferative activity. Number of mitotic presentations and Ki-67 index would adopt a high grade evaluation.
  1. Number of mitotic presentations (to evaluate 50HPF)
  2. Ki-67 index（500–2000 tumor cells*）

  *（Only in this study, if the number of tumor cells in the collected sample is less than 500, the number of tumor cells measured is also listed. A minimum of 100 tumor cells is required.）
- If sufficient amount of biopsy sample cannot be collected for a pathological diagnosis, a cell block prepared using material obtained from EUS-FNA, brushing or needle biopsy may be used. However, the use of a cell block prepared from ascites or pleural effusion is not permitted.
- During a histological diagnosis using a resected tumor sample, a slide with a representative section shall be used to evaluate the proportion of NEC components (differential diagnosis with mixed adenoendocrine carcinoma). For a comprehensive examination, the entire tumor must be evaluated.

For further details NEC Pathological Diagnosis Handbook would be referred (posted on JCOG website).

3.4. Disease stage classification criteria (UICC-TNM 7th Edition)

Special notice regarding disease stage classification: Although there is an independent TNM classification for NET G1 and NET G2 with primary lesions in the stomach, small intestines, and colon; since this study concerns NEC, the disease would be classified according to the classification method of major tissue type in each primary lesion organ (squamous cell carcinoma for esophagus, and adenocarcinoma for all other primary lesion organs). In this study, the classification of intrahepatic cholangiocarcinoma is used for hepatic NEC, due to their similar clinical presentation.

3.4.1. Esophagus

T –Primary tumor

TX： Primary tumor cannot be evaluated
T0： Primary tumor unidentified
Tis： Epithelial carcinoma/highly dysplastic
T1： Tumor invading mucosal lamina propria, muscularis mucosae, or submucosa
  T1a：Tumor invading the mucosal lamina propria or muscularis mucosae
  T1b： Tumor invading the submucosa

T2 : Tumor invading the muscularis propia

T3 : Tumor invading the adventitia

T4 : Tumor invading the surrounding tissue

T4a : Tumor invading the pleura, pericardium, and diaphragm

T4b : Tumor invading other surrounding tissues such as the aorta, centrum, trachea

N –Regional lymph nodes

NX : Regional lymph node metastasis cannot be evaluated

N0 : No regional lymph node metastasis

N1 : 1–2 foci of regional lymph node metastases

N2 : 3–6 regional foci of lymph node metastases

N3 : 7 or more foci of regional lymph node metastases

M –Distal metastasis

M0 : No distal metastasis

M1 : Distal metastasis present

Stage	T	N	M
**0**	Tis	N0	M0
**IA**	T1	N0	M0
**IB**	T2	N0	M0
**IIA**	T3	N0	M0
**IIB**	T1, T2	N1	M0
**IIIA**	T4a	N0	M0
	T3	N1	M0
	T1, T2	N2	M0
**IIIB**	T3	N2	M0
**IIIC**	T4a	N1、N2	M0
	T4b	Unrelated to N	M0
	Any T	N3	M0
**IV**	Any T	Any N	M1

3.4.2. Stomach

T –Primary tumor

TX : Primary tumor cannot be evaluated

T0 : Primary tumor unidentified

Tis : Epithelial carcinoma: Epithelial carcinoma/highly dysplastic carcinoma not invading the lamina propia mucosa

T1 : Tumor invading mucosal lamina propria, muscularis mucosae, or submucosa

T1a : Tumor invading the mucosal lamina propria or muscularis mucosae

T1b : Tumor invading the submucosa

T2 : Tumor invading the muscularis propia

T3 : Tumor invading the subserosa

T4 : Tumor perforating the serosa, or invading adjacent structures[1,2,3]

T4a : Tumor perforating the serosa

T4b : Tumor invading adjacent structures[1,2,3]

1. Adjacent organs of the stomach are spleen, transverse colon, liver, diaphragm, pancreas, abdominal wall, adrenal glands, kidneys, small intestine, and retroperitoneum.
2. If the invasion has spread from the stomach to the duodenum or esophagus, classification is made in terms of depth.
3. A tumor that advances into the gastrocolic ligament, into the hepatogastric ligament, or into the greater or lesser omentum, and is classified as T3, when there is no perforation of the visceral peritoneum.

N –Regional lymph nodes

NX: Regional lymph node metastasis cannot be evaluated

N0: No regional lymph node metastasis

N1: 1–2 regional foci of lymph node metastases

N2: 3–6 regional foci of lymph node metastases

N3: 7 or more regional foci of lymph node metastases

N3a: 7–15 regional foci of lymph node metastases

N3b: 16 or more regional foci of lymph node metastases

M –Distal metastasis

M0: No distal metastasis

M1: Distal metastasis present

Stage	T	N	M
**0**	Tis	N0	M0
**IA**	T1	N0	M0
**IB**	T2	N0	M0
	T1	N1	M0
**IIA**	T3	N0	M0
	T2	N1	M0
	T1	N2	M0
**IIB**	T4a	N0	M0
	T3	N1	M0
	T2	N2	M0
	T1	N3	M0
**IIIA**	T4a	N1	M0
	T3	N2	M0
	T2	N3	M0
**IIIB**	T4b	N0, N1	M0
	T4a	N2	M0
	T3	N3	M0
**IIIC**	T4a	N3	M0
	T4b	N2, N3	M0
**IV**	Any T	Any N	M1

3.4.3. Small intestines (including duodenum)

T –Primary tumor

TX: Primary tumor cannot be evaluated

T0: Primary tumor unidentified

Tis: Epithelial carcinoma

T1: Tumor invading mucosal lamina propria, muscularis mucosae, or submucosa

T1a: Tumor infiltrating the mucosal lamina propria or muscularis mucosae

T1b: Tumor infiltrating the submucosa

T2: Tumor infiltrating the muscularis propia

T3: Tumor invading the subserosa, or tumor invading within 2 cm of surrounding tissue of muscularis externa without peritoneal cover (mesenterium, retroperitoneum)*

*Surrounding tissue of muscularis externa without peritoneal cover refers to the mesenterium in the jejunum and ileum, and the retroperitoneum in the duodenum without serosa.

T4: Tumor penetrating the visceral peritoneum, or tumor directly invading another organ or tissue (invasion of other loops of the small intestine, invasion by 2 cm or more into the mesenterium and retroperitoneum, including invasion into the abdominal wall through the serosa; invasion to the pancreas only for duodenum)

N –Regional lymph nodes

NX: Regional lymph node metastasis cannot be evaluated

N0: No regional lymph node metastasis

N1 : 1–3 regional foci of lymph node metastases

N2 : 4 or more regional foci of lymph node metastases

M –Distal metastasis

M0 : No distal metastasis

M1 : Distal metastasis present

Stage	T	N	M
**0**	Tis	N0	M0
**I**	T1, T2	N0	M0
**IIA**	T3	N0	M0
**IIB**	T4	N0	M0
**IIIA**	Any T	N1	M0
**IIIB**	Any T	N2	M0
**IV**	Any T	Any N	M1

3.4.4. Appendix (partial revision of UICC-TNM 7th Edition)

T –Primary tumor

TX : Primary tumor cannot be evaluated

T0 : Primary tumor unidentified

Tis : Epithelial carcinoma: Tumor invading epithelium or lamina propia mucosae

T1 : Tumor invading the submucosa

T2 : Tumor invading the muscularis propia

T3 : Tumor invading the subserosa or mesoappendix

T4 : Tumor penetrating the visceral peritoneum, tumor including a peritoneal mucosal tumor in the lower right abdomen and/or tumor directly invading other organs or tissue

T4a : Tumor penetrating the visceral peritoneum, or peritoneal mucosal tumor in the lower right abdomen

T4b : Tumor directly invading other organs or tissues

N –Regional lymph nodes

NX : Regional lymph node metastasis cannot be evaluated

N0 : No regional lymph node metastasis

N1 : 1–3 regional foci of lymph node metastases

N2 : 4 or more foci of regional lymph node metastases

M –Distal metastasis

M0 : No distal metastasis

M1 : Distal metastasis present

M1a : Peritoneal metastasis beyond the lower right abdomen, including pseudomyxoma peritonei

M1b : Distal metastasis other than peritoneal metastasis

Stage	T	N	M
**0**	Tis	N0	M0
**I**	T1, T2	N0	M0
**IIA**	T3	N0	M0
**IIB**	T4a	N0	M0
**IIC**	T4b	N0	M0
**IIIA**	T1, T2	N1	M0
**IIIB**	T3, T4	N1	M0
**IIIC**	Any T	N2	M0
**IVA**	Any T	N0	M1a
**IVB**	Any T	N0	M1a
	Any T	N1, N2	M1a
**IVC**	Any T	Any N	M1b

3.4.5. Colon and rectum

T –Primary tumor

TX : Primary tumor cannot be evaluated

T0 : Primary tumor unidentified

Tis : Carcinoma in situ: Tumor invading epithelium or lamina propia mucosae

T1 : Tumor invading the submucosa

T2 : Tumor invading the muscularis propia

T3 : Tumor invading the subserosa, or tissue surrounding the colon or rectum without peritoneal covering

T4 : Tumor penetrating the visceral peritoneum, and/or directly invading another organ or tissues

T4a : Tumor penetrating the visceral peritoneum

T4b : Tumor directly invading another organ or tissues

N –Regional lymph nodes

NX : Regional lymph node metastasis cannot be evaluate

N0 : No regional lymph node metastasis

N1 : 1–3 regional foci of lymph node metastases

N1a : 1 regional lymph node metastasis

N1b : 2–3 regional foci of lymph node metastases

N1c : Presence of tumor deposits, that is, satellite nodes in the subserosa or in the soft tissue surrounding colon or rectum without peritoneal covering, but with no regional lymph node metastasis

N2 : 4 or more regional lymph node metastases

N2a : 4–6 regional foci of lymph node metastases

N2b : 7 or more regional foci of lymph node metastases

M –Distal metastasis

M0 : No distal metastasis

M1 : Distal metastasis present

M1a : Local metastasis to one organ (liver, lungs, ovaries, or lymph nodes other than regional lymph nodes)

M1b : 2 or more organs, or peritoneal metastasis

Stage	T	N	M
**0**	Tis	N0	M0
**I**	T1, T2	N0	M0
**II**	T3, T4	N0	M0
**IIA**	T3	N0	M0
**IIB**	T4a	N0	M0
**IIC**	T4b	N0	M0
**III**	Any T	N1, N2	M0
**IIIA**	T1, T2	N1	M0
	T1	N2a	M0
**IIIB**	T3, T4	N1	M0
	T2, T3	N2a	M0
	T1, T2	N2b	M0
**IIIC**	T4a	N2a	M0
	T3, T4a	N2b	M0
	T4b	N1, N2	M0
**IVA**	Any T	Any N	M1a
**IVB**	Any T	Any N	M1b

3.4.6. Applies to hepatic NEC (hepatic primary lesion or liver metastasis from unknown primary lesion)

T –Primary tumor

TX : Primary tumor cannot be evaluated

T0 : Primary tumor unidentified

Tis : Carcinoma *in situ*

T1 : Isolated tumor without vascular invasion

T2a : Isolated tumor with vascular invasion

T2b : Multifocal tumor regardless of vascular invasion

T3 : Tumor penetrating visceral peritoneum or directly invading adjacent extrahepatic structures

T4 : Tumor with bile duct invasion (bile duct proliferative type)

N –Regional lymph nodes

NX : Regional lymph node metastasis cannot be evaluated

N0 : No regional lymph node metastasis

N1 : Regional lymph node metastasis present

M –Distal metastasis

M0 : No distal metastasis

M1 : Distal metastasis present

Stage	T	N	M
**I**	T1	N0	M0
**II**	T2	N0	M0
**III**	T3	N0	M0
**IVA**	T4	N0	M0
	Any T	N1	M0
**IVB**	Any T	Any N	M1

3.4.7. Gall bladder

T –Primary tumor

TX : Primary tumor cannot be evaluated

T0 : Primary tumor unidentified

Tis : Carcinoma *in situ*

T1 : Tumor invading mucosal lamina propria or muscularis externa

T1a : Tumor invading mucosal lamina propria

T1b : Tumor invading muscularis externa

T2 : Tumor invading the connective tissue around the muscularis externa, but shows no progression beyond the serosa or to the liver

T3 : Tumor perforating the serosa (visceral peritoneum), tumor directly advancing to the liver and/or an adjacent organ that is not the liver (stomach, duodenum, colon, pancreas, omentum, or extrahepatic bile duct)

T4 : Tumor invading the main trunk of the portal vein or the hepatic artery, or tumor advancing to two or more adjacent organs which are not the liver

N –Regional lymph nodes

NX : Regional lymph node metastasis cannot be evaluated

N0 : No regional lymph node metastasis

N1 : Regional lymph node metastasis present (cystic duct, common bile duct, proper hepatic artery, including lymph nodes along the portal vein)

M –Distal metastasis

M0 : No distal metastasis

M1 : Distal metastasis present

Stage	T	N	M
**0**	Tis	N0	M0
**I**	T1	N0	M0
**II**	T2	N0	M0
**IIIA**	T3	N0	M0
**IIIB**	T1, T2, T3	N1	M0
**IVA**	T4	Any N	M0

**IVB**	Any T	Any N	M1

3.4.8. Extrahepatic bile duct-hepatic portal region

T –Primary tumor

TX : Primary tumor cannot be evaluated

T0 : Primary tumor unidentified

Tis : Carcinoma *in situ*

T1 : Tumor localized to the bile duct which advances until muscularis externa or fibrous tissue

T2a : Tumor invading beyond the bile duct wall and into the surrounding adipose tissue

T2b : Tumor invading the adjacent liver parenchyma

T3 : Tumor invading the branch of one side of portal vein or hepatic artery

T4 : Tumor invades the main trunk of portal vein, and branches on both sides of the portal vein, proper hepatic artery or the secondary branches of the bile duct on both left and right side, or to the secondary bile duct branches on one side and portal vein or hepatic artery on the other side

N –Regional lymph nodes

NX : Regional lymph node metastasis cannot be evaluated

N0 : No regional lymph node metastasis

N1 : Presence of regional lymph node metastasis in the cystic duct, common bile duct, proper hepatic artery, and lymph nodes along portal vein

M –Distal metastasis

M0 : No distal metastasis

M1 : Distal metastasis present

Stage	T	N	M
**0**	Tis	N0	M0
**I**	T1	N0	M0
**II**	T2a, T2b	N0	M0
**IIIA**	T3	N0	M0
**IIIB**	T1,T2, T3	N1	M0
**IVA**	T4	N0, N1	M0
**IVB**	Any T	Any N	M1

3.4.9. Extrahepatic bile duct-distal

T –Primary tumor

TX : Primary tumor cannot be evaluated

T0 : Primary tumor unidentified

Tis : Carcinoma *in situ*

T1 : Tumor localized in the bile duct wall

T2 : Tumor invading beyond the bile duct wall

T3 : Tumor invading the gallbladder, liver, pancreas, duodenum, or other adjacent organs

T4 : Tumor invading the celiac axis or superior mesenteric artery

N –Regional lymph nodes

NX : Regional lymph node metastasis cannot be evaluated

N0 : No regional lymph node metastasis

N1 : Regional lymph node metastasis present

M –Distal metastasis

M0 : No distal metastasis

M1 : Distal metastasis present

Stage	T	N	M
**0**	Tis	N0	M0
**IA**	T1	N0	M0
**IB**	T2	N0	M0

**IIA**	T3	N0	M0
**IIB**	T1, T2, T3	N1	M0
**III**	T4	Any N	M0
**IV**	Any T	Any N	M1

3.4.10. Ampulla of Vater

T –Primary tumor

TX: Primary tumor cannot be evaluated

T0: Primary tumor unidentified

Tis: Carcinoma *in situ*

T1: Tumor localized to the ampulla of Vater, or sphincter of Oddi

T2: Tumor invading the duodenal wall

T3: Tumor invading the pancreas

T4: Tumor invading the soft tissue around the pancreas or other adjacent organs

N –Regional lymph nodes

NX: Regional lymph node metastasis cannot be evaluated

N0: No regional lymph node metastasis

N1: Regional lymph node metastasis present

M –Distal metastasis

M0: No distal metastasis

M1: Distal metastasis present

Stage	T	N	M
**0**	Tis	N0	M0
**IA**	T1	N0	M0
**IB**	T2	N0	M0
**IIA**	T3	N0	M0
**IIB**	T1, T2, T3	N1	M0
**III**	T4	Any N	M0
**IV**	Any T	Any N	M1

3.4.11. Pancreas

T –Primary tumor

TX: Primary tumor cannot be evaluated

T0: Primary tumor unidentified

Tis: Carcinoma *in situ*

T1: Tumor localized within the pancreas, with a maximum size of < 2 cm

T2: Tumor localized within the pancreas, with a maximum size > cm

T3: Tumor advancing outside the pancreas, but with no invasion to the celiac axis or superior mesenteric artery

T4: Tumor invading the celiac axis or superior mesenteric artery

N –Regional lymph nodes

NX: Regional lymph node metastasis cannot be evaluated

N0: No regional lymph node metastasis

N1: Regional lymph node metastasis present

M –Distal metastasis

M0: No distal metastasis

M1: Distal metastasis present

Stage	T	N	M
**0**	Tis	N0	M0
**IA**	T1	N0	M0
**IB**	T2	N0	M0
**IIA**	T3	N0	M0

**IIB**	T1, T2, T3	N1	M0
**III**	T4	Any N	M0
**IV**	Any T	Any N	M1

3.5. Residual tumor (R) classification (UICC-TNM 7th Edition)

RX : Presence of residual tumor cannot be evaluated

R0 : No residual tumor

R1 : Presence of residual tumor by microscopic examination

R2 : Presence of residual tumor by macroscopic examination

3.6. Definition of non-resectable NEC

Unoperated cases for which surgery as a curative process was not indicated based on clinical findings, including image-based diagnosis, or operated cases that underwent resection (including experimental laparotomy) but ultimately ended as R2 resection are considered non-resectable.

The details by organs are shown below. One or more criterion must be fulfilled for each organ.

<Esopghagus>

- Diagnose as Stage IV (UICC 7th edition) based on clinical findings.

<Stomach>

- Diagnose as Stage IV (UICC 7th edition) based on clinical or surgical findings. However, this does not include situations where Stage IV diagnosis is provided based only on peritoneal lavage cytology (CY1).
- Bulky lymph node* metastasis is identified.

  *Bulky lymph node: Two or more lymph nodes with a major axis of 1.5 cm or more, that are present in contact with each other around the celiac artery, the common hepatic artery, the splenic artery, and the proper hepatic artery, or on the front surface of the superior mesenteric vein, where single or multiple large and small lymph nodes have formed an aggregate with the overall major axis length of 3.0 cm or more.

<Duodenum (excluding the ampulla of Vater), small intestines, appendix, and colon>

- Diagnosed as Stage IV (UICC 7th edition) based on the clinical findings.

<Extrahepatic bile duct, ampulla of Vater, gallbladder>

- Distant metastasis is identified
- Para-aortic lymph node metastasis is identified
- Bulky metastasis identified in the hepatoduodenal mesentery and lymph nodes surrounding the head of the pancreas
- Invasion in the proper hepatic artery, common hepatic artery, celiac artery, or the superior mesenteric artery identified
- Invasion in the hepatic artery branches on both the left and right side identified
- Extensive invasion or occlusion of the main trunk of portal vein, or invasion in the portal vein branches on both the left and right side identified
- Invasion in the blood vessels on the lobe on one side of the liver (portal vein or artery) or atrophy of lobe on one side of liver identified, and advancement into the bile duct on the other side is identified up to the secondary branch level
- Advancement into the bile ducts on both sides identified up to the secondary branch level

<Pancreas>

- Diagnosed as Stage III or Stage IV (UICC 7th edition) based on clinical findings.

＜Hepatic NEC (hepatic primary lesion or liver metastasis from unknown primary lesion)>

- Distal metastasis* other than intrahepatic metastasis identified.

  *Limited to organs that cannot be the primary lesion site (bone, lymph nodes below the diaphragm,

peritoneum, subcutaneous, muscle, and spleen) (see 3.8)

- Para-aortic lymph node metastasis is identified
- Bulky metastasis identified in the hepatoduodenal mesentery and lymph nodes surrounding the head of the pancreas
- Multiple masses identified in the liver.
- Invasion in one of the proper hepatic artery, common hepatic artery, celiac artery, or the superior mesenteric artery identified.
- Invasion to hepatic artery branches on both the left and right side identified
- Extensive invasion or occlusion of the main trunk of portal vein, or invasion in portal vein branches on both the left and right side identified
- Invasion in blood vessels on lobe on one side of the liver (portal vein or artery) or atrophy of lobe on one side of liver identified, and advancement into the bile duct on the other side is identified up to the secondary branch level
- Advancement into the bile ducts on both sides identified up to the secondary branch level

3.7. Definition of recurrent NEC

If surgery (R0 resection or R1 resection) was performed for gastrointestinal/hepatobiliary or pancreatic primary lesion, malignant tumor and the condition was diagnosed as NEC based on pathological samples taken from surgery, and were deemed as a clinical relapse thereafter.

If there are past histological samples from the primary lesion, no histological biopsy of the relapse lesion is necessary.

3.8. Definition of hepatic NEC (hepatic primary lesion or liver metastasis from an unknown primary lesion)

After a detailed investigation into the primary lesion by cervical-pelvic contrast CT, gastrointestinal endoscopy, FDG-PET, otolaryngology (head and neck examination), urology examination (for men only) and gynecological examination (for women only), if the tumor is in one of the following sites, the case is defined as hepatic primary lesion (or primary lesion unknown).

- Tumor is present only in the liver
- Tumor is present in an organ that cannot be a primary lesion site (bone, lymph nodes below the diaphragm, peritoneum, subcutaneous, muscle, and spleen), and in the liver.

4. Patient selection criteria

Patients that fulfill all of the following inclusion criteria and do not correspond to any of the exclusion criteria are eligible for enrollment.

4.1. Inclusion criteria (for enrollment)

1) Any of the following is applicable based on pathological diagnosis taking findings of immunohistochemistry into consideration (see 3.1. to 3.3.).

[1] Pathologically diagnosed as neuroendocrine carcinoma (NEC*1) in the resected sample.

[2] Containing pathologically confirmed component of neuroendocrine carcinoma (NEC*1) in the biopsy sample.

1: Based on WHO 2010 classification

2) Any of the following is applicable

[1] NEC arise in esophagus, stomach, duodenum, intestine, appendix, colon, rectum, gallbladder, intrahepatic bile duct, extrahepatic bile duct, ampulla of Vater, pancreas,

[2] Liver NEC (primary liver or liver metastasis of unknown primary) *2.

*2: The tumor is only in one of the following sites after a thorough examination of the primary site by contrast CT (from the neck to pelvic) and upper/lower gastrointestinal endoscopy, FDG-PET scan, otolaryngology (head and neck) examination, urology examination (male patients only), and gynecology examination (female patients only).

a. Liver only

b. Bone, lymph nodes below the diaphragm, peritoneum, subcutaneous, muscle, spleen, and liver

3) Unresectable (see 3.6) or recurrent cancer (see 3.7). It is not essential for a pathological confirmation of the metastatic lesion or recurrent site. Cases of esophageal NEC is ineligible if corresponding to any of the following.

[1] cT4.

[2] No distant metastasis rather than supraclavicular lymph node

[3] Stenosis indicated for palliative radiotherapy

4) No previous chemotherapy or radiotherapy for NEC. Pre- or post-operative chemotherapy except irinotecan or etoposide for NEC is allowed as long as it was completed at least 8 weeks prior to registration.

5) No previous chemotherapy using platinum agents for any malignancies.

6) No serious tumor-related complications.

Serious tumor-related complications include superior vena cava syndrome, inferior vena cava syndrome, pleural, ascites, or pericardial effusions that are large or uncontrollable (e.g., effusions that accumulate quickly after drainage or even after adhesive surgery), and brain metastases with neurological symptoms.

7) Aged 20 to 75 years old.

8) ECOG performance status of 0 or 1.

9) Sufficient oral intake

10) Measurable region is not required.

11) Adequate organ functions.

[1] WBC ≥3,000/ mm^3

[2] Neutrophils ≥ 1,500/ mm^3

[3] Hemoglobin ≥ 9.0 g/dL

[4] Platelets ≥ 10×10^4/ mm^3

[5] Total bilirubin≤ 1.5 mg/dL[※4]

[6] AST(sGOT)≦100 IU/L[※4]（for hepatic NEC and liver metastasis, ≦150 IU/L）

[7] ALT(sGPT)≦100 IU/L[※4]（for hepatic NEC and liver metastasis, ≦150 IU/L）

※4：Presence or absence of biliary drainage is not relevant

[8] Serum creatinine≦ 1.3 mg/dL

[9] Creatinine clearance[※5]≥ 60 mL/min

※5： Creatinine clearance must have been estimated using the Cockcroft-Gault formula, and must be 60 mL/min or more. If the estimation is less than 60 mL/min, but the actual measurement is 60 mL/min or more, the patient can be deemed eligible.

Cockcroft-Gault formula

Male：Ccr ={(140－age) × body weight (kg)}/{72×serum creatinine (mg/dL)}

Female：Ccr = 0.85×{(140－age) × body weight (kg)}/{72×serum creatinine(mg/dL)}

12) Written informed consent.

4.2. Exclusion criteria

1) Synchronous or metachronous (within 5 years) malignancies except carcinoma in situ or intramucosal tumor curatively treated with local therapy.

2) Active infection requiring systemic therapy.

3) Fever of 38 degrees Celsius or higher.

4) Pregnant or lactating women, women of childbearing potential, or women within 28 days after delivery.

5) Psychiatric disease.

6) Patients requiring systemic steroids medication.

7) Interstitial pneumonia, pulmonary fibrosis.

8) Serious co-existing illness.

9) Unstable angina pectoris within 3 weeks, or with a history of myocardial infarction within 6 months.

10) Impossible to use both iodine and gadolinium due to being allergic to contrast agent.

11) Uncontrolled diabetes mellitus or routine administration of insulin.

5. Registration and randomization

5.1. Procedure of registration

Ensure that a patient to be registered meets all eligibility criteria and does not meet any of exclusion criteria and register the patient by using JCOG Web Entry System. JCOG Web System Personal Account and password are required for web registration. If unknown, contact JCOG Data Center.

Patient registration JCOG Web Entry System
URL:https://secure.jcog.jp/dc/
(Web registration can be used for 24 hours.)

Contact information for patient registration and JCOG Web Entry System
JCOG Data Center
TEL: 03-3542-3373
Weekdays 9:00-17:00 (not available in holidays, Saturdays and Sundays, New Year's holidays)
E-mail:JCOGdata@ml.jcog.jp

Contact information on Patients Selection Criteria
Study Coordinator Contact : Chigusa Morizane
Hepato-Biliary Pancreatic Group: Futomi Mori (Main Research Secretariat)
Department of Hepatobiliary and Pancreatic Medicine, National Cancer Center
TEL:03-3542-2511
FAX:03-3542-3815
E-mail:cmorizan@ncc.go.jp

Gastric Cancer Group: Yoshinori Machida
Shizuoka Cancer Center
〒411-8777 1007 Shimonagakubo, Nagaizumi-cho, Sunto-gun, Shizuoka Prefecture
TEL:+81-55-989-5222
FAX:+81-55-989-5631
E-mail：no.machida@scchr.jp

Esophageal Cancer Group: Ken Kato/Yushi Homma
Department of Gastrointestinal Oncology, National Cancer Center
TEL: 03-3542-2511
FAX: 03-3542-3815
E-mail: kenkato@ncc.go.jp / yohonma@ncc.go.jp

5.1.1. Precautions for patient registration

① Registration after initiation of protocol treatment is unacceptable.
② Registration is performed by accessing the URL in '5.1. Procedure of registration'.
③ Eligibility checks are performed on the screen of Registration Form, so it is not necessary to send a Registration Form to Data Center by mail or fax.
④ If input data are insufficient, registration is not accepted until all are met.
⑤ The registration number is issued after the confirmation of eligibility on the registration screen, then the registration is completed.
⑥ Once registered, patients will not be retracted (retracted from the database) unless there is withdrawal of consent, including refusal to use the data for research. For duplicate registration, the information at the initial registration (registration number, allocated arm) are used in any case.
⑦ When misregistration or duplicate registration is found, contact Data Center immediately.
⑧ Body surface area and drug dose calculations are institutional responsibilities, and the body surface area

and drug dose displayed on Web Entry System at registration are only for double-checking. Those should always be calculated and checked at the institution. When the body surface area calculation formula adopted in the hospital information system of the institution differs from calculation formula adopted by JCOG (Dubois formula: Body surface area (m^2) = Body weight (kg) $^{0.425}$ x Height (cm) $^{0.725}$ 84 ÷ 10,000), there can be a difference in the dose by the hospital information system of the institution and the dose by the calculation formula adopted by JCOG, but in that case, either dosage used is decided by the site investigator.

5.2. Randomization and allocation adjustment factor

During enrollment, the treatment arm for a patient is allocated randomly by the data center.

Randomization would use a method of minimization using the 1) institutions, 2) Primary lesion organ (gastrointestinal tract [esophagus, stomach, duodenum, small intestines, appendix, colon, and rectum] vs. hepatobiliary and pancreatic organ [hepatic NEC, gallbladder, extrahepatic bile duct, ampulla of Vater, or pancreas]) as the adjustment factors, so that there are no large discrepancies between them. Researchers at participating facilities would not be informed of the detailed procedures of randomization.

6. Treatment Plan and Treatment Modification Criteria

Unless patient safety is threatened, treatment and treatment modifications is done in compliance with the specifications in this chapter.

If it is considered that the protocol specification may cause medically dangerous situation of the patient, treatment modifications should be made according to the medical judgment of the investigators/sub-investigators. Such protocol deviation is considered to be "clinically relevant deviation" if considered medically appropriate (see 14.1.4.Protocol deviation/violation). Deviations that occur with intentions other than safety, such as increasing efficacy, are not considered clinically relevant deviations.

6.1. Protocol treatment

Protocol treatment is initiated within 7 days of enrollment.

If treatment initiation occurs after 8 days from registration for any reason, the reason should be documented on the Treatment Course Form. If it is determined that treatment cannot be initiated, describe the details in the Off-treatment Form as Protocol Treatment Termination.

When laboratory parameters worsen and eligibility criteria are no longer met by the start of treatment after registration, the investigator/sub-investigator is allowed to decide whether initiate or terminate protocol treatment at their own discretion.

「6.3. The course initiation criteria is not applied at treatment changes.

Drugs used

- Etoposide ※
- Cisplatin ※
- Irinotecan ※
- Entecavir, tenofovir disoproxil fumarate, tenofovir alafenamide fumarate

The use of generic drugs is not restricted.

※ The company that manufactures or distributes these drugs, or intends to manufacture or sell these drugs, requires conflicts of interest control in the Clinical Trials Act as a company involved in this study (see 13.8.).

6.1.1. Arm A: Etoposide plus cisplatin (EP) therapy

The following chemotherapy courses will be given once a week for 3 weeks and treatment will be continued until the patient meets the discontinuation criteria.

Drug	Dosage(mg/m^2)	Dosing regimen/Dosing time	Dose day
Etoposide	100	IV/60-120 min	Day 1、2、3
Cisplatin	80	IV/60-120 min	Day 1

1) Calculation of the dosage

① Body surface area is calculated by determining drug dose to the third decimal point.

② For both etoposide and cisplatin, the calculated dose is determined by truncating the decimal point. For drug doses, ±10% is the acceptable range.

③ Dose recalculation due to body weight change after treatment initiation is not performed.

2) Administration of anticancer drugs

Etoposide and cisplatin are given in any order.

Examples of administration in the package insert are described below.

(i) Before administration: Before administration of anticancer drugs, 1,000-2,000 mL of hydration is done to achieve adequate diuresis.

(ii) Etoposide: Etoposide is mixed with infusion solutions such as isotonic sodium chloride solution of 250 mL or more, and infused intravenously in about 60-120 minutes. DEHP (2-ethylhexyl) phthalate: di-(2-ethylhexyl) phthalate), which is a plasticizer, elutes from polyvinyl chloride infusion sets and catheters. Avoid the use of polyvinyl chloride infusion sets and catheters.

(iii) Cisplatin administration: Cisplatin is mixed with stock solution or 500 mL of saline (or glucose-saline) and given intravenously in 60-120 min. During the administration, caution should be exercised in ensuring urine volume, and diuretics such as mannitol and furosemide should be administered as

necessary.

(iv) After administration: After administration of cisplatin, 1,000-2,000 mL of hydration should be done so that adequate diuresis can be achieved.

In addition, short hydration administration at the discretion of each institution is permitted. (see Table 6.1.1. for treatment cases).

Table 6.1.1. Example of short hydration

	Dose	Time
5HT3 antagonist<br>Dexamethasone<br>Physiological saline	9.9 mg<br>50 mL	15 min
Etoposide<br>Physiological saline	100 mg/m^2<br>250 mL	60 min
No. 1 solution<br>Potassium chloride<br>Magnesium sulfate	500 mL<br>10 mEq<br>8 mEq	60 min
20% mannitol	200 mL	30 min
Cisplatin<br>Physiological saline	80 mg/m^2<br>250 mL	60 min
No. 1 solution<br>Potassium chloride	500 mL<br>10 mEq	60 min

(Referred from Horinouchi H, et al., Japan Society of Clinical Oncology 2012

3) Precautions for treatment

JCOG9511, an upfront trial in small-cell lung cancer, suggested an association between treatment-related deaths and first-course neutropenia. Therefore, caution should be exercised when neutropenia is strongly observed from the first course.

6.1.2. Arm B: Irinotecan plus cisplatin (IP) therapy

One 4-week course of the following chemotherapy will be continued until the discontinuation criteria are met. However, if day 15 irinotecan is skipped, 3 weeks should be used as one course, and the next course should be started with day 22.

Drug	Dosage(mg/m^2)	Dosing regimen/Dosing time	Dose day
Irinotecan	60	IV/90 min	Day 1、8、15
Cisplatin	60	IV/60-120 min	Day 1

1) Calculation of the dosage

(i) Body surface area is determined by determining the dose of drug administered until the third decimal point.

(ii) The dose is calculated for both irinotecan and cisplatin by truncating the decimal point. For drug doses, ±10% is the acceptable range.

(iii) Dose recalculation due to body weight change after treatment initiation is not performed.

2) Administration of anticancer drugs

Irinotecan and cisplatin are given in any order.

Examples of administration in the package insert are described below.

(i) Before administration: Before administration of anticancer drugs, 1,000-2,000 mL of hydration is done to achieve adequate diuresis.

(ii) Irinotecan: Irinotecan is mixed with 500 mL or more of isotonic sodium chloride solution, glucose solution, or electrolyte maintenance solution and given by intravenous drip infusion at about 90 minutes.

(iii) Cisplatin administration: Cisplatin is mixed with stock solution or 500 mL of saline (or glucose-saline) and given intravenously in 60-120 min. During the administration, caution should be exercised in ensuring urine volume, and diuretics such as mannitol and furosemide should be administered as necessary.

(iv) After administration: After administration of cisplatin, 1,000-2,000 mL of hydration should be done so that adequate diuresis can be achieved.

In addition, short hydration administration at the discretion of each institution is permitted. (See Table 6.1.1.

3) Precautions for treatment

JCOG9511, an upfront trial in small-cell lung cancer, suggested an association between treatment-related deaths and first-course neutropenia. Therefore, caution should be exercised when neutropenia is strongly observed from the first course.

6.2. Protocol Treatment Termination/Completion Criteria

6.2.1. Definition of protocol treatment completion

Protocol treatment is continued in this study unless the protocol treatment discontinuation criteria are met, so no definition of protocol treatment completion is provided.

6.2.2. Criteria for termination of protocol treatment

Protocol treatment is terminated in any of the following cases:

1) Judged as protocol treatment is ineffective
   - When a definite tumor exacerbation is confirmed by imaging or clinically
     ※ Protocol treatment should not be discontinued if the clinical judgement of PD based on the assessment of response based on imaging indicates that continuation of protocol treatment is appropriate, and protocol treatment should be continued.
2) Protocol treatment cannot be continued due to adverse events
   ① If Grade 4 non-hematological toxicity is observed (with the exception of the following adverse events) 'hypernatremia', 'hyponatremia', 'hyperkalemia', 'hypokalemia', 'hyperglycemia', 'hypoglycemia', 'alkaline phosphatase increase', 'alanine aminotransferase increase', 'aspartate aminotransferase increase', 'blood bilirubin increase','serum amylase increase', 'lipase increase', 'GGT increase'.
   (*Adverse events other than "anaemia," "myeloid cytopenia," "lymphocyte count decreased," "neutrophil count decreased," "white blood cell count decreased," "platelet count decreased," "CD4 lymphopenia" in CTCAE v4 0)
   ② If the next course cannot be initiated beyond 21 days from the expected start date of the course due to an adverse event
   ③ When the criteria for terminating protocol treatment in the treatment modification criteria (6.3.) are met.
   ④ Adverse events other than the treatment modification criteria that the investigator/sub-investigator judges to require termination of protocol treatment
3) If the patient offers termination of protocol treatment for reasons not denied to be associated with the adverse event
   - This category should be used if an association with an adverse event cannot be ruled out.
4) When the patient offers termination of protocol treatment because of reasons for denial of association with adverse events
   - Patient refusal after enrollment and before initiation of protocol treatment
   - When the association with an adverse event can first be denied, such as the relocation of the person or household member during protocol treatment.
5) Death during protocol treatment
   - Death before deciding to terminate protocol treatment for other reasons
6) Palliative surgery for pathogenic adverse events or surgery for patients with complete response to chemotherapy (see Section 6.3.9).
7) In addition, exacerbations before the start of treatment after enrollment (protocol treatment could not be initiated due to rapid exacerbation), protocol violations were found, ineligibility was determined due to modifications in pathological diagnosis after enrollment, etc., treatment was changed, and it was judged that it was difficult to continue protocol treatment due to social reasons and safety management problems, etc.

The date of discontinuation of protocol treatment is defined as the date of death in 6.2.2.5), the date of surgery in 6.2.2.6), and otherwise, the date on which the treating physician judges that protocol treatment is discontinued.

In this study, non-NEC may be diagnosed by central pathological diagnosis during protocol treatment, because central pathological diagnosis is performed. If the patient is continuing on protocol treatment when the results of the central pathology diagnosis are reported to the registry, the attending physician and the institutional pathologist will review the results and take a clinically appropriate response. Protocol treatment is discontinued if discontinuation of protocol treatment is judged to be appropriate, and the reason for discontinuation is other.

6.3. Treatment modification criteria

The following terms shall be used for the treatment modification.

Delay: Delay the start of the course or administration of treatment from the planned date.

Termination: Discontinuation of a part of or all of the treatment without restarting.

Suspending: temporary interruptions or withdrawals that may be resumed if conditions are met

Skip: Do not administer one or more drugs and proceed to the next schedule.

Categories of infection (CTCAEv4.0) used in this study are as follows

Infection: CTCAEv4.0 infections and infestations

Bronchial infection; pulmonary infection; upper respiratory tract infection; mediastinal infection; pleural infection; catheter-related infection; Biliary tract infection; Gallbladder infection; urinary tract infection

6.3.1. Arm A (EP-therapy): Dose level

1) Etoposide

Drug	Dose level	Dosing schedule	Dose day
Etoposide	Level 0	100 mg/m^2 div	Day 1、2、3
	Level -1	80 mg/m^2 div	Day 1、2、3
	Level -2	60 mg/m^2 div	Day 1、2、3

2) Cisplatin

Drug	Dose level	Dosing schedule	Dose day
Cisplatin	Level 0	80 mg/m^2 div	Day 1
	Level -1	60 mg/m^2 div	Day 1
	Level -2	40 mg/m^2 div	Day 1

6.3.2. A arm (EP therapy): Course initiation criteria

- Initiate the course after confirming that all of the following "Course Initiation Criteria" are met on the scheduled start date of the course or the day before the plan start date of the course.
- If any one is not met, the initiation of the course is delayed.
- If the course cannot be initiated within 21 days of the expected start date of the course (if the start date of the previous course was day 1 and the course could not be initiated by day 42), discontinue the protocol treatment.
- The course initiation criteria is not applied at the start of the first course.

Course initiation criteria

① Neutrophil count Grade 0-1 (≧1500 per mm^3).

② Platelet count ≧10×10^4 /mm^3

③ AST≦100 IU/L (in the presence of hepatic metastases vs. hepatic NECs) was ≦200 IU/L).

④ ALT≦100 IU/L (≦200 IU/L for liver metastases vs liver NECs).

⑤ Total bilirubin <= 2.0 mg/dL

⑥ Serum creatinine <= 1.5 mg/dL

⑦ Fever Grade 0 (axillary temperature, no antipyretic)

⑧ Constipation, fatigue, phlebitis, oral mucositis, and infection ※1 are all Grade 2 or less.
Infected ※1: bronchial infection, pulmonary infection, upper respiratory tract infection, mediastinal infection, pleural infection, catheter-related infection, biliary tract infection, gallbladder infection, urinary tract infection

⑨ Anorexia, nausea, and emesis are all Grade 0-1.

⑩ Diarrhoeal Grade 0

6.3.3. Arm A (EP therapy): Dose reduction criteria

If any of the following toxicities are identified during the course, dose reduction should be performed in accordance with the dose reduction criteria (Table 6.3.3.) from the following course (no dose reduction in the course).

However, even if two or more items are met, the dose reduction for each drug is only one step. Re-escalation after dose reduction is not performed. Protocol treatment is terminated if the dose reduction criteria are met again after the dose reduction to Level-2.

Table 6.3.3. Arm A (EP therapy): Dose reduction criteria

Item	Etoposide	Cisplatin
Neutrophil count Grade 4 (<500 per mm^3).	Reduce the level by 1	No change
Platelet count Grade 4 (<$2.5\times10^4/mm^3$)	Reduce the level by 1	No change
1.5 <serum creatinine ≦2.0 mg/dL.	No change	Reduce the level by 1
Serum creatinine <= >2.0 mg/dL	Termination of protocol treatment	Termination of protocol treatment
Grade 3<br>Infected ※1	Reduce the level by 1	Reduce the level by 1
Grade 3<br>Assessment at onset of febrile.	Reduce the level by 1	Reduce the level by 1
Grade 2<br>Peripheral sensory neuropathy<br>Peripheral motor neuropathy,<br>Myalgia; arthralgia; tinnitus; hearing impairment	No change	Reduce the level by 1
Grade 3<br>Peripheral sensory neuropathy<br>Peripheral motor neuropathy,<br>Myalgia; arthralgia; tinnitus; hearing impairment	Termination of protocol treatment	Termination of protocol treatment
Non-hematologic toxicities of Grade 3 other than those listed above in ※2 that are causally related to EP-therapy (excluding hyponatremia, hypokalemia, hypomagnesemia, hypocalcemia, hyperglycemia, and weight loss)	Reduce the level by 1	Reduce the level by 1

※1:※1 of infection: bronchial infection, pulmonary infection, upper respiratory tract infection, mediastinal infection, pleural infection, catheter-related infection,
Biliary tract infection; Gallbladder infection; Urinary tract infection

※2:Causal relationship is judged as either of possible, probable, definite

6.3.4. Arm A (EP therapy): Within-course pause, skipping criteria

Following initiation of treatment with each course after the course initiation criteria are met, if any of the following adverse events are observed, day 2, day 3 etoposide will be suspended.

- Fever (axillary temperature) Grade 1-3
- Grade 3 of infection (bronchial infection, pulmonary infection, upper respiratory tract infection, mediastinal infection, pleural infection, catheter-related infection,
  Biliary tract infection, gallbladder infection, urinary tract infection)

Resting etoposide should be resumed after confirming that all initiation criteria are met until day 7. However, it should not be administered after day 8.

That is, if etoposide could not be administered by day 7, the remaining etoposide should be skipped.

If the above suspension and/or skip occur, the next course of etoposide should be started day 22 (after 3 weeks) counting from day 1 of the previous course if the ininiation criteria are met.

6.3.5. Arm B (IP therapy): Dose-level

1) Irinotecan

Drug	Dose level	Dosing schedule	Dose day
Irinotecan	Level 0	60 mg/m^2 div	Day 1、8、15
	Level -1	50 mg/m^2 div	Day 1、8、15
	Level -2	40 mg/m^2 div	Day 1、8、15

2) Cisplatin

Drug	Dose level	Dosing schedule	Dose day
Cisplatin	Level 0	60 mg/m^2 div	Day 1
	Level -1	50 mg/m^2 div	Day 1
	Level -2	40 mg/m^2 div	Day 1

6.3.6. Arm B (IP therapy): Course initiation criteria

- On the day of the initiation of the course or the day before the expected start of the course, start the course after confirming that all of the following "Course Initiation Criteria" are met.
- If any one is not met, the initiation of the course is delayed.
- If the course cannot be initiated within 21 days of the expected start date of the course (if the start date of the previous course was day 1 and the course could not be initiated by day 49), discontinue the protocol treatment.
- However, if day 15 irinotecan is skipped in the previous course, day 22 of the previous course is set as the scheduled start date of the next course regarded as one course per 3 weeks.
- The course initiation criteria is not applied at the start of the first course.

Course initiation criteria

① Neutrophil count Grade 0-1 (≧1500/mm^3).
② Platelet count ≧10×10^4 /mm^3
③ AST≦100 IU/L (≦200 IU/L for liver metastases versus liver NECs)).
④ ALT≦100 IU/L (≦200 IU/L for liver metastases vs liver NECs).
⑤ Total bilirubin <= 2.0 mg/dL
⑥ Serum creatinine <= 1.5 mg/dL
⑦ Fever Grade 0 (measured by axillary temperature, temperature <38°C without antipyretic use)
⑧ Constipation, fatigue, phlebitis, oral mucositis, and infection ※1 are all Grade 2 or less.
Infected ※1: bronchial infection, pulmonary infection, upper respiratory tract infection, mediastinal infection, pleural infection, catheter-related infection, biliary tract infection, gallbladder infection, urinary tract infection
⑨ Anorexia, nausea, and emesis are all Grade 0-1.
⑩ Diarrhoea Grade 0

6.3.7. Arm B (IP-therapy): Dosing criteria for day 8, day 15

After confirming that all of the following ①-③ are met, the second (day 8) or third (day 15) dose of irinotecan is administered. If day 8, day 15 dosing criteria are not met, skip day 8, day 15 dosing.

① All of the following are met with the most recent laboratory data on the scheduled day of administration or the day before the scheduled day of administration.
i) Neutrophil count Grade 0-2 (≧1000 per mm^3).
ii) Platelet count ≧10×10^4 /mm^3
iii) AST ≦ 100 IU/L (≦200 IU/L for liver metastases vs liver NECs).
iv) ALT ≦ 100 IU/L (≦200 IU/L in the presence of hepatic metastases versus hepatic NECs).
v) Total bilirubin <= 2.0 mg/dL
vi) Serum creatinine <= 2.0 mg/dL

② All of the following are met on the scheduled day of administration:
i) Fever Grade 0 (measured by axillary temperature, temperature <38°C without antipyretic use)

ii） Diarrhoeal Grade 0

③ Constipation, anorexia, nausea, emesis, fatigue, phlebitis, oral mucositis, and infection ※1 are all Grade 2 or less.

Infected ※1: bronchial infection, pulmonary infection, upper respiratory tract infection, mediastinal infection, pleural infection, catheter-related infection, biliary tract infection, gallbladder infection, urinary tract infection

6.3.8. Arm B (IP therapy): Dose reduction criteria

If any of the following toxicities are identified during the course, dose reduction should be performed in accordance with the dose reduction criteria (Table 6.3.8.) from the following course (no dose reduction in the course).

However, even if two or more items are met, the dose reduction for each drug is only one step. Re-escalation after dose reduction is not performed. Protocol treatment is terminated if the dose reduction criteria are met again after the dose reduction to Level-2.

Table 6.3.8. Arm B (IP therapy): Dose reduction criteria

Item	Irinotecan	Cisplatin
Neutrophil count Grade 4 (<500 per mm^3).	Reduce the level by 1	No change
Platelet count Grade 4 (< $2.5\times10^4/mm^3$)	Reduce the level by 1	No change
1.5 <serum creatinine ≦2.0 mg/dL.	No change	Reduce the level by 1
Serum creatinine <= >2.0 mg/dL	Termination of protocol treatment	Termination of protocol treatment
Grade 3<br>Infected ※1	Reduce the level by 1	Reduce the level by 1
Grade 3<br>Assessment at onset of febrile.	Reduce the level by 1	Reduce the level by 1
Grade2<br>Peripheral sensory neuropathy<br>Peripheral motor neuropathy,<br>Myalgia; arthralgia; tinnitus; hearing impairment	No change	Reduce the level by 1
Grade 3<br>Peripheral sensory neuropathy<br>Peripheral motor neuropathy,<br>Myalgia; arthralgia; tinnitus; hearing impairment	Termination of protocol treatment	Termination of protocol treatment
Non-hematologic toxicities of Grade 3 other than those listed above in ※2 that are causally related to IP-therapy (excluding hyponatremia, hypokalemia, hypomagnesemia, hypocalcemia, hyperglycemia, and weight loss)	Reduce the level by 1	Reduce the level by 1

※1:※1 of infection: bronchial infection, pulmonary infection, upper respiratory tract infection, mediastinal infection, pleural infection, catheter-related infection,

Biliary tract infection; Gallbladder infection; Urinary tract infection

※2:Causal relationship is judged as either of possible, probable, definite

6.3.9. Surgical of after end of chemotherapy

1) Surgery for Adverse Events Associated with Pathogenesis

Surgery may be performed if it is judged clinically desirable to perform surgery for newly appearing symptoms such as hemorrhage or stenosis. Protocol treatment is discontinued if surgery is performed, regardless of the content. In this case, the date of discontinuation of protocol treatment is the date of surgery.

2) Surgery for patients with complete response to chemotherapy

- When chemotherapy is highly effective, all metastases present at the time of enrollment on imaging studies disappear, and curative resection (R0 resection) is considered possible, resection including primary and metastatic disease may be performed.
- Surgical procedures are not specifically specified, but the Research Office will collect information on the details of the surgery performed individually in order to be reviewed by the research representative/research office.

- In all surgical cases, the group group conference shall confirm the validity of the judgment that surgery is indicated.
- When surgery is performed, protocol treatment is discontinued, regardless of whether curative resection was performed or not. In this case, the date of discontinuation of protocol treatment is the date of surgery.

6.3.10. Consultation on treatment modification

If there are any questions about treatment modification, contact "16.6. Study Coordinator".

Study Coordinator Contact : Chigusa Morizane

Hepato-Biliary Pancreatic Group: Futomi Mori (Main Research Secretariat)
Department of Hepatobiliary and Pancreatic Medicine, National Cancer Center
TEL:03-3542-2511
FAX:03-3542-3815
E-mail:cmorizan@ncc.go.jp

Gastric Cancer Group: Yoshinori Machida
Shizuoka Cancer Center
〒411-8777 1007 Shimonagakubo, Nagaizumi-cho, Sunto-gun, Shizuoka Prefecture
TEL:+81-55-989-5222
FAX:+81-55-989-5631
E-mail：no.machida@scchr.jp

Esophageal Cancer Group: Ken Kato/Yushi Homma
Department of Gastrointestinal Oncology, National Cancer Center
TEL:03-3542-2511
FAX:03-3542-3815
E-mail: kenkato@ncc.go.jp / yohonma@ncc.go.jp

6.4. Concomitant treatment and supportive care

6.4.1. Required concomitant treatment/supportive care

1) Testing and Supportive Care for HBsAg-Positive Cases.

In HBsAg-positive cases, steroids and chemotherapy can lead to rapid hepatitis B virus (HBV) expansion (reactivation: reactivation) and potentially fatal severe hepatitis. Therefore, the following tests and supportive care are performed based on the "Guidelines for the Treatment of Hepatitis B, Third Edition (Japanese Society of Hepatology)." It is advisable to consult a hepatologist at the time prior to initiation of a nucleic acid analogue (entecavir, tenofovir disoproxil fumarate, and tenofovir alafenamide fumarate).

①Testing prior to initiation of chemotherapy: HBV-DNA quantitation

HBV-DNA quantitative analysis should be performed at least once prior to initiation of chemotherapy.

HBV-DNA assays are performed by real-time PCRs.

HBeAg and HBe antibodies should also be measured in accordance with the Guidelines for the Treatment of Hepatitis B, Third Edition (Japanese Society of Hepatology).

②Dosing schedule for supportive care (nucleic acid analogues prophylaxis)

- **Drugs used :**
  - ➢ **Entecavir (Bristol-Myers: Baraclude Tablets 0.5 mg)**
  - ➢ **Tenofovir disoproxil fumarate (GlaxoSmithKline: Tenozet Tablets 300 mg)**
  - ➢ **Tenofovir alafenamide fumarate (Giliado: Vemuridi Tablets 25 mg)**

The following dosage regimen should be followed, starting at least 1 week before the start of chemotherapy (as soon as possible), and continuing for at least 12 months after the end of chemotherapy. However, fulminant hepatitis has been reported in HBsAg-positive patients with high viral load, even during NA prophylaxis, and it is desirable to reduce the viral load before starting immunosuppression/chemotherapy. Nucleic acid analogues may be discontinued after 12 months after completion of chemotherapy if conditions *1 and 2 for discontinuation of NAs are met. However, if the administration of a nucleic acid analogue is discontinued, consultation with a hepatologist is always obtained, and the administration is discontinued only if the hepatologist deems it appropriate.

※1 Requirements for discontinuation of nucleic acid analogues (entecavir, tenofovir disoproxil fumarate, tenofovir alafenamide fumarate): all of the following

1. The patient has been on NA for more than 2 years.
2. HBV-DNA assays are not sensitive to detect
3. Be negative for HBeAg

※2 Patient background requirements: All of the following

1. Both the treating physician and the patient have sufficiently understood that the hepatitis exacerbation is frequently observed after the nucleic acid analog withdrawal, and that there is a danger of becoming serious in the time.
2. Follow-up is possible after treatment cessation, and appropriate treatment is possible even if hepatitis recurs
3. It is judged that the liver fibrillation is slight and the hepatic reserve is good, and it is difficult to become serious even if the hepatitis is exacerbated.

(Adapted from Guidelines for the Treatment of Hepatitis B, the 3rd edition (Japanese Society of Hepatology))

Entecavir

- **Dosage regimen: Take this medicine on an empty stomach (2 hours after meals and more than 2 hours before the next meal).**
- **Dosage :**

Creatinine clearance (mL/min).	Dosage
50 or more	0.5 mg once daily
30 Beyond 50	0.5 mg once every 2 days
10 Beyond 30	0.5 mg once every 3 days
10 Less than	0.5 mg once every 7 days

- **Adverse drug reactions (incidence of all grades): nucleoside analog-naïve patients**

Diarrhea (6.0%), nausea (4.5%), constipation (3.7%), upper abdominal pain (3.0%), malaise (1.5%), nasopharyngitis (3.0%), muscle stiffness (2.2%), headache (3.0%), rash (incidence unknown), laboratory tests: elevated AST (3.7%), increased blood bilirubin (6.0%), increased blood amylase (10.4%), and increased lipase

(10.4%).Blood glucose increased (6.0%), blood lactate increased (6.7%), urine occult blood positive (4.5%), white blood cell count decreased (8.2%), and eosinophil count increased (0.7%). [Significant adverse reactions (incidence unknown)] Hepatitis worsened after completion of treatment, anaphylactoid symptoms, lactic acidosis, and severe hepatomegaly due to fatty liver

Tenofovir disoproxil fumarate

- **Dosage and administration: 300 mg is orally administered once daily.**
- **Dosage :**

Creatinine clearance (mL/min).	Dosage
50 or more	300 mg once daily
30 Beyond 50	300 mg once every 2 days
10 Beyond 30	300 mg once every 3 to 4 days
Hemodialysis	注) of 300 mg once every 7 days<br>Or 300 mg after completion of cumulative approximately 12 hours of dialysis<br>NOTE) After hemodialysis was performed. The pharmacokinetics in patients with creatinine clearance < 10 mL/min and not on hemodialysis have not been investigated.

- **Dosing Precautions:**

In the long-term administration of tenofovir disoproxil fumarate, attention should be paid to renal dysfunction, hypophosphatemia (including Fanconi syndrome), and decrease in bone mineral density. It is recommended that renal function and serum phosphorus should be measured regularly during tenofovir disoproxil fumarate administration.

- **Adverse reactions (incidence of all grades):**

Abnormal liver function tests (AST, ALT and γ-GTP increased, etc.) in 7 patients (4.9%), increased creatinine in 4 patients (2.8%), increased amylase, increased lipase and nausea in 3 patients each (2.1%), abdominal pain in 2 patients (1.4%), [major adverse reactions (incidence unknown)] renal dysfunction, renal failure, acute renal failure, proximal renal tubular dysfunction, Fanconi syndrome, severe renal dysfunction such as acute renal tubular necrosis, nephrogenic diabetes insipidus or nephritis, severe hepatomegaly due to lactic acidosis and fatty deposition (steatohepatitis), pancreatitis

Tenofovir alafenamide fumarate

- **Dosage and administration: 25 mg is orally administered once daily.**
- **Dosage :**

Creatinine clearance (mL/min).	Dosage
15 or more	25 mg once daily
Less than 15	Consider discontinuation

- **Dosing Precautions:**

In the long-term administration of tenofovir alafenamide fumarate, attention should be paid to renal dysfunction, hypophosphatemia (including Fanconi syndrome), and decrease in bone density. It is recommended that renal function and serum phosphorus should be measured periodically during tenofovir alafenamide fumarate administration.

- **Adverse reactions (incidence of all grades):**

Nausea and abdominal distension, headache, fatigue (≧1%), dyspepsia and diarrhea, flatus, upper abdominal pain, constipation, ALT increased, arthralgia, dizziness, insomnia, pruritus, rash (≧0.5% to <1%), [significant adverse reactions (incidence unknown)] renal dysfunction, renal failure, acute renal failure, proximal renal tubular dysfunction, severe renal impairment such as Fanconi syndrome, acute renal tubular necrosis, renal diabetes insipidus or nephritis, severe hepatomegaly due to lactic acidosis and fatty deposits (fatty liver)

③Monitoring: Quantitative analysis of HBV-DNA (during and after administration of nucleic acid analogues)

During nucleic acid analogue administration:

They are monitored every 4 weeks by both HBV-DNA quantitative analysis and liver function (ASTs, ALTs). However, if HBV-DNA level is less than 20 IU/mL (1.3 log IU/mL) during administration of nucleic acid analogues, it is acceptable to perform tests every 4 to 12 weeks.

After discontinuation of nucleic acid analogue administration:

Bearing in mind that reactivation may occur even after discontinuation of administration of a nucleic acid analogues, the patient should be consulted with a hepatologist, and the patient should be monitored for HBV-DNA determination and hepatic function (AST/ALT) every 4 weeks for at least 1 year after discontinuation of administration of a nucleic acid analogues. Subsequent follow-up will be decided after consulting a hepatologist.

2) Laboratory Teste and Supportive Care for HBsAg-Negative and HBc Antibody-Positive and/or HBs Antibody-Positive Cases.

HBV-DNA quantitative analysis should be performed at least once prior to initiation of chemotherapy. HBV-DNA assays are performed by real-time PCRs.

i) HBV-DNA ≧ 20 IU/mL (1. 3 log IU/mL) prior to initiation of chemotherapy

It has been clarified that HBV-DNA replicates persist at low levels in the livers and peripheral blood mononuclear cells when HBc or HBs are positive, even if they are HBs-Ag negative. It has been reported that reactivation of HBV and development of severe hepatitis are caused by the use of potent immunosuppressive agents even in such patients with previous infections.

If HBV-DNA ≧ 20 IU/mL (1.3 log IU/mL), the risk of HBV reactivation is judged to be as high as in HBsAg-positive cases, and prophylactic administration of nucleic acid analogues (entecavir or tenofovir disoproxil fumarate, tenofovir alafenamide fumarate) is administered. The following laboratory tests and supportive care are performed in accordance with the "Guideline for the Treatment of Hepatitis B, 3rd edition (Japanese Society of Hepatology)" with reference to the following for examination, dosage, and monitoring of supportive care before the start of chemotherapy.

However, these are not applicable if the HBs antibody alone is positive and the HBV vaccination history is obvious.

①Dosing schedule for supportive care (nucleic acid analogues prophylaxis)

According to the dosage and administration of nucleic acid analogues (entecavir, tenofovir disoproxil fumarate, tenofovir alafenamide fumarate) in "1) Test and supportive care for HBsAg positive cases". Same conditions for NA discontinuation.

② Monitoring: Quantitative analysis of HBV-DNA (during and after administration of nucleic acid analogues)

The intervals for monitoring during and after discontinuation of NA are in accordance with the provision of "1) Testing and supportive care for HBsAg-positive patients".

ii) HBV-DNA less than 20 IU/mL (1.3 log IU/mL) prior to initiation of chemotherapy

HBV-DNA quantitative analysis and hepatic function (AST, ALT) will be monitored, and nucleic acid analogues (entecavir, tenofovir disoproxil fumarate, tenofovir alafenamide fumarate) will be started when ≧20 IU/mL (1.3 log IU/mL) is achieved.

The Guidelines for the Treatment of Hepatitis B, the 3rd edition (Japanese Society of Hepatology) recommends monitoring with HBV-DNA quantitative analysis or high-sensitivity HBs antibodies during and after chemotherapy, depending on the risks of revitalization.

①Monitor:HBV-DNA quantitative analysis

HBV-DNA quantitative analysis should be performed every 4-12 weeks from the start of chemotherapy until at least 12 months after the end of chemotherapy.

If HBV-DNA level is more than 20 IU/mL (1.3 log IU/mL), administration of nucleic acid analogues should be started immediately in accordance with the Guidelines for the Treatment of Hepatitis B, the 3rd edition (Japanese Society of Hepatology). If HBsAg monitoring is positive for < 1 IU/mL (low positive), nucleic acid analogues should be administered after additional HBV DNA determinations of ≧ 20 IU/mL (1.3 log IU/mL).

It is advisable to consult a hepatologist at a time prior to initiation of NAs.

②Supportive care in reactivation

Nucleic acid analogues should be administered according to the supportive care described in i) When HBV-DNA prior to the initiation of chemotherapy is 20 IU/mL (1.3 log IU/mL) or more in 6.4.1.2). Once administration of nucleic acid analogues is started, nucleic acid analogues should be discontinued only if appropriate by the hepatologist.

6.4.2. Recommended/not recommended concomitant treatment/supportive care

The following concomitant treatment and supportive care are recommended. Even if it is not carried out, it is not regarded as protocol deviation,

1) Addressing Febrile Neutropenia.

① Assessment at onset of febrile neutropenia (FN).

a) If the count is less than 500 per mm^3, or is less than 1000 per mm^3 and is expected to be less than 500 per mm^3 within 48 hours, and if the axillary temperature is 37.5°C or higher (mouth temperature is 38.0°C or higher), a severity-risk assessment should be performed promptly and anti-virus treatment initiated as appropriate.

b) Severity risk assessment is performed with reference to Multinational Association for Supportive Care in Cancer(MASCC) scoring system [※1].

c) For initial evaluation, complete blood count including differential WHITE BLOOD CELL and platelet count, renal function (BUN, creatinine), electrolytes, liver function (transaminases, total bilirubin, alkaline phosphatase) tests, ≧2 sets of venous blood cultures prior to initiation of antimicrobials, one set of cultures from the catheter lumen and one set from the peripheral vein if a central venous catheter is in place, cultures of suspected infection areas, and chest x-rays if respiratory symptoms and signs are present.

d) When febrile neutropenia (FN) develops in a patient with a central venous catheter, blood cultures from the catheter and peripheral blood are performed, and catheter-related infections are considered if there is a time difference of more than 120 minutes in the positivity of both. If appropriate antimicrobial therapy does not improve after more than 72 hours, catheter should be removed. For infections caused by Staphylococcus aureus, Pseudomonas aeruginosa, Bacillus, fungi, and acid-fast bacilli, the catheter should be removed and appropriate antimicrobial therapy based on culture results should be performed.

② Antibiotic use

a) In high-risk patients, β-lactams with anti-Pseudomonas aeruginosa activity are administered intravenously as a single agent. However, other antimicrobials (aminoglycosides, fluoroquinolones, and/or vancomycin) may be added to a single agent in the initial regimen in patients with unstable or complicated conditions or when drug-resistant organisms are strongly suspected. Low-risk patients may be treated with antibiotics orally or intravenously, hospitalized, or with adequate evaluation, if appropriate, as outpatients.

b) Re-evaluation will be performed 3-4 days after initiation of antibiotics to investigate the continuation or change of antibiotics. In principle, antibiotics will be continued until the neutrophil count recovers to 500 cells per mm^3 or more.

c) Empiric antifungal therapy is recommended in high-risk patients who do not respond to 4-7 days of broad-spectrum antibiotics.

d) Fluoroquinolone prophylaxis is recommended in high-risk individuals with an expected neutrophil count of 100 cells per mm^3 or less lasting >7 days.

③ Therapeutic administration of G-CSF

For therapeutic administration of G-CSF during the incidence of FNs, refer to "6.4.5. 3) Therapeutic administration of G-CSF".

※1 Multinational Association for Supportive Care in Cancer (MASCC) scoring system.
(Adapted in part from the Practice Guideline for Febrile Neutropenia (FN) [Japanese Society of Medical Oncology]. *2)

Item	Score
Clinical manifestations (select one of the following * mark 3 sections)<br>*No symptoms<br>*Mild symptoms<br>*Moderate symptoms	 5<br>5<br>3
No decrease in blood pressure	5
No chronic obstructive pulmonary disease	4
Solid tumors, or hematopoietic tumors without a history of fungal infection	4
No dehydration symptoms	3
Patients with fever during outpatient management	3
Age < 60	2

The total score is up to 26 points. Twenty-one points or more are considered low risk and 20 points or less are considered high risk

※2 Since patients aged 20 years or older are subjects in this study, we deleted "Not applicable to patients younger than 16 years old" from the original edition of the Practice Guideline for Febrile Neutropenia (FN) [Japanese Society of Medical Oncology].

2) Nausea and vomiting

Regarding nausea and vomiting, antiemetics are positively administered according to Clinical Practice Guidelines for Antiemesis in Oncology [48], and fluid and electrolyte repletion are performed when oral intake is severely reduced.

3) Anorexia

If oral intake drops markedly, fluid and electrolyte supplements should be given as needed. Especially, in the cases with diabetes mellitus, the abnormality of blood sugar level and electrolyte is noticed.

4) Anemia, thrombocytopenia

If anaemia (haemoglobin < 8.0 g/dL) or thrombocytopenia (platelet count < $2 \times 10^4/mm^3$) is observed, blood should be transfused as appropriate at the discretion of the treating physician.

5) Diarrhea

Severe diarrhoea occasionally occurs with arm B (IP therapy arm) and is extremely dangerous when complicated by febrile neutropenia. Patients should be fully informed about toxicities and their management, and should be instructed to measure body temperature, especially when neutrophils counts are most decreasing, and to contact a physician or nurse immediately during fever and diarrhea. Nonsteroidal anti-inflammatory drugs may not cause fever to become overt, so unnecessary anti-inflammatory drugs are not given.

If irinotecan-induced diarrhea occurs, the following supportive measures are recommended:

High-dose loperamide hydrochloride therapy

① Loperamide hydrochloride was started after signs of diarrhea were observed.
② Initial dose of 4 mg followed by 2 mg/2 hours (4 mg/4 hours at night)
③ It is administered until watery stool does not appear for more than 12 hours.
④ Doses should not be given for more than 48 hours.

6) Precautions on the day of cisplatin administration

Aminoglycoside antibiotics, vancomycin, and nonsteroidal anti-inflammatory drugs are not administered on the day of cisplatin administration or are used with caution.

6.4.3. Acceptable concomitant treatment and supportive care

The following concomitant treatment and supportive care may be used as needed.

Concomitant use of drugs for the treatment of complications such as hypertension and diabetes mellitus and symptomatic drugs such as morphine may be performed, but this drug should be administered with caution when furosemide, piretanide and phenytoin are used. Oral antibiotics for febrile neutropenia prophylaxis may be given at the discretion of the investigator/sub-investigator. Bisphosphonate denosumab may be used in combination for bone metastases.

6.4.4. Unacceptable concomitant treatment and supportive care

None of the following treatments will be given during protocol treatment:

① Administration of anticancer drugs other than protocol treatment

② Radiation therapy

6.4.5. Granulocyte colony-stimulating factor (granulocyte-colony stimulating factor:G-CSF

※This study permits the use of G-CSF biogenerics (biosimilars).

1)※ of primary prophylaxis with G-CSF

※Primary prophylaxis: G-CSF administration before developing febrile neutropenia or prolonged neutropenia to prevent them during anticancer therapy.

Primary prophylaxis with G-CSF was not recommended at the beginning of the study in this study. However, since febrile neutropenia in group A was 21.4% in the late 2016 periodic monitoring report and 15.8% in the early 2017 periodic monitoring report (18.4% when the time of occurrence was April 2017 and SAE reports not reflected in the early 2017 periodic monitoring report were included), it was decided that the patient would meet at least the recommended grade B or higher (almost A) according to G-CSF Appropriate Use Guideline 2013 and JSMO Febrile Neutropenia (FN) Practice Guideline. Primary prophylactic administration of G-CSF was recommended in group A. However, it is not considered a protocol deviation even if it is not administered. Since it is practical to administer pegfilgrastim from the viewpoint of convenience, when the primary preventive administration of G-CSF is carried out in the actual medical field, the administration example of pegfilgrastim is shown below.

(Administration cases)

Group A: 3.6 mg of pegfilgrastim (genetical recombination) will be injected subcutaneously (once per chemotherapy course) between day 4(day 3's completion of etoposide treatment and day 7 at least 24 hours after the completion of etoposide treatment.

Since the safety of pegfilgrastim administered 14 days prior to the initiation of cancer chemotherapy and within 24 hours after completion of administration has not been established, the primary prophylactic administration of pegfilgrastim is not performed in group B where day 8 is administered the drug.

Table 6.4.5. Primary prophylactic administration of G-CSF should be performed according to the approved dosage and administration shown in the table below.

Drug	・・ Pegfilgrastim (arm A only)<br>・・ Filgrastim<br>・・ Naltograstim<br>・・ Lenograstim
Time of initiation	Twenty-four hours after completion of chemotherapy
Dosage Dosing regimen	・Pegfilgrastim (genetical recombination) at a dose of 3.6 mg subcutaneously once per chemotherapy course (group A only)<br>・Filgrastim: 50 μg per m^2 SC once daily or 100 μg per m^2 IV once daily<br>・Naltograstim: 1 μg/kg SC once daily or 2μg/kg IV once daily<br>・Lenograstim: 2 μg/kg SC once daily or 5μg/kg IV once daily
Timing of discontinuation (other than pegfilgrastim)	・If the neutrophil count reaches 5000 per mm^3 or more after the course, administration should be discontinued.<br>・If the neutrophil count recovers to ≧2000 cells per mm^3, there are no suspected infections, and the patient's safety is judged to be ensured by the responsiveness to the drug used, the drug should be discontinued or reduced.

2)Secondary prophylactic※ of G-CSF

※ Secondary prophylaxis: G-CSF prophylactic administration after once occurrence of febrile neutropenia or prolonged neutropenia to prevent febrile neutropenia or prolonged neutropenia from occurring again during anticancer therapy.

If febrile neutropenia occurs in the previous course, secondary prophylaxis with G-CSF after the subsequent course is recommended, even if dose reduction or schedule modification or antimicrobial therapy is considered to be associated with a lower risk of febrile neutropenia (group A). However, it is not considered a protocol deviation even if it is not administered. As it is practical to administer pegfilgrastim in terms of convenience when secondary prophylactic administration of G-CSF is carried out in real medical practice, the following examples of administration of pegfilgrastim are shown.

(Administration cases)

Group A: 3.6 mg of pegfilgrastim (genetical recombination) is injected subcutaneously (once per course of chemotherapy) between the time of completion of etoposide treatment of day 4(day 3 and day 7 of at least 24 hours.

However, the safety of pegfilgrastim administered 14 days prior to the start of cancer chemotherapy and 24 hours after the end of treatment has not been established, so secondary prophylactic pegfilgrastim administration is not performed in group B, where day 8 is given the drug.

Secondary prophylactic administration of G-CSF should be performed according to the approved dosage and administration shown in the table below.

Drug	・・ Pegfilgrastim (arm A only)<br>・・ Filgrastim<br>・・ Naltograstim<br>・・ Lenograstim
Time of initiation	・・ Pegfilgrastim (arm A only)<br>Twenty-four hours after completion of chemotherapy<br>・・ Filgrastim, nartograstim, and lenograstim<br>When neutrophil counts <1000 per mm^3 are observed
Dosage Dosing regimen	・Pegfilgrastim (genetical recombination) at a dose of 3.6 mg subcutaneously once per chemotherapy course (group A only)<br>・Filgrastim: 50 μg per m^2 SC once daily or 100 μg per m^2 IV once daily<br>・Naltograstim: 1 μg/kg SC once daily or 2μg/kg IV once daily<br>・Lenograstim: 2 μg/kg SC once daily or 5μg/kg IV once daily
Timing of discontinuation (other than pegfilgrastim)	・If the neutrophil count reaches 5000 per mm^3 or more after the course, administration should be discontinued.<br>・If the neutrophil count recovers to ≧2000 cells per mm^3, there are no suspected infections, and the patient's safety is judged to be ensured by the responsiveness to the drug used, the drug should be discontinued or reduced.

3) Therapeutic administration of G-CSF

Therapeutic administration of G-CSF should be performed according to the approved dosage and administration shown in the table below.

Time of initiation	・When the neutrophil count is less than 1000 per mm^3 and fever (in principle, > 38.0°C) is observed<br>・When neutrophil counts <500 per mm^3 are observed
Dosage Dosing regimen	・Filgrastim: 50 μg per m^2 SC once daily or 100 μg per m^2 IV once daily<br>・Naltograstim: 1 μg/kg SC once daily or 2μg/kg IV once daily<br>・Lenograstim: 2 μg/kg SC once daily or 5μg/kg IV once daily
Timing of discontinuation	・If the neutrophil count reaches 5000 per mm^3 or more after the course, administration should be discontinued.<br>・If the neutrophil count recovers to ≧2000 cells per mm^3, there are no suspected infections, and the patient's safety is judged to be ensured by the responsiveness to the drug used, the drug should be discontinued or reduced.

6.5. Post-study treatment

Treatment after discontinuation of protocol treatment and treatment after progression or recurrence after completion are not specified.

Treatment (cross over) with drugs included in treatment regimens in the unassigned groups may be used, but if the total dose of cisplatin is greater than 500 mg/m^2, careful attention should be given to accumulating toxicities such as peripheral sensory/motor neuropathy, hearing loss, and renal impairment. Patients should be carefully monitored with adequate risk explanation only if the benefits are apparently outweighed by the risks, such as those with persistent sensitivity to cisplatin and mild cumulative toxicity.

If primary analysis or interim analysis reveals the primary conclusions of the trial, the results of the study will be explained to patients enrolled in this study as needed, and the best treatment will be provided, taking into account the course of treatment of individual patients.

In addition, if the protocol treatment discontinuation criteria apply but clinically "protocol treatment continuation" is judged to be appropriate, consult the research office through the institutional research director or institutional coordinator rather than at the physician level as a general rule (except when time is not allowed). In agreement between the Research Secretariat and the Investigator/Institution Coordinator, decide whether to treat as a → after discontinuation of protocol treatment or to deviate and continue protocol treatment. The details of the consultation

with the Research Secretariat and the decision-making process should be provided in the comment column for the patient's end-of-treatment report and progress record. If continuing the protocol treatment with protocol deviation occurs frequently, the Study Coordinator should consider revising protocol treatment termination criteria using group meetings and group mailing lists, because it is considered that the protocol treatment termination criteria is clinically inappropriate in such situation.

7. Anticipated Adverse Events

7.1. Anticipated adverse reactions

Anticipated adverse reactions in this study are as follows:

7.1.1. Anticipated Adverse Drug Reactions with Drugs

Adverse drug reactions anticipated with protocol treatments and drugs used in protocol-specified tests are referred to the latest version of the drug package insert.

7.1.2. Anticipated adverse reactions in the standard treatment arm (arm A)

Table 7.1.2. Adverse events in the EP-therapy group at JCOG9511 (excerpt from the final analysis report)

Examination Items	Grade 0	Grade 1	Grade 2	Grade 3	Grade 4	Grade 3,4(%)	Grade4(%)	Total
White blood cells	2	5	30	35	5	51.9%	6.5%	77
Neutrophils	1	0	5	21	50	92.2%	64.9%	77
Hemoglobin	2	9	43	23	-	29.9%	-	77
Platelet	31	19	13	14	0	18.2%	0%	77
Total bilirubin	57	-	20	0	0	0%	0%	77
GOT	49	24	2	1	1	2.6%	1.3%	77
GPT	40	28	6	2	1	3.9%	1.3%	77
Creatinine	56	16	5	0	0	0%	0%	77
Oxygen tension	13	27	9	2	1	5.8%	1.9%	52
Nausea and vomiting	13	36	23	5	-	6.5%	-	77
Diarrhea	64	8	5	0	0	0%	0%	77
Oral cavity (stomatitis)	68	6	2	1	0	1.3%	0%	77
Infection	42	23	9	1	2	3.9%	2.6%	77
Hair loss (hair)	9	46	19	-	-	-	-	74
Fever (uninfected)	45	14	16	2	0	2.6%	0%	77
Perception (peripheral nerve)	66	10	1	0	-	0%	-	77
Rash	74	2	1	0	0	0%	0%	77

※: Use JCOG Toxicity Criteria.

7.1.3. Anticipated adverse reactions in the study treatment arm (Arm B)

Table 7.1.3. Adverse Events in the IP-Therapy Group in a JCOG9511 (Extracted from the Final Analysis Report)

Examination Items	Grade 0	Grade 1	Grade 2	Grade 3	Grade 4	Grade 3,4(%)	Grade 4(%)	Total
White blood cells	1	16	38	17	3	26.7%	4.0%	75
Neutrophils	1	8	17	30	19	65.3%	25.3%	75
Hemoglobin	7	10	38	20	-	26.7%	-	75
Platelet	56	6	9	1	3	5.3%	4.0%	75
Total bilirubin	59	-	16	0	0	0%	0%	75
GOT	40	30	5	0	0	0%	0%	75
GPT	35	30	7	3	0	4.0%	0%	75
Creatinine	56	15	4	0	0	0%	0%	75
Oxygen tension	13	20	5	1	1	5.0%	2.5	40
Nausea and vomiting	11	26	28	10	-	13.3%	-	75
Diarrhea	23	19	21	8	4	16.0%	5.3%	75
Oral cavity (stomatitis)	66	9	0	0	0	0%	0%	75

Infection	45	17	9	3	1	5.3%	1.3%	75
Hair loss (hair)	17	44	13	-	-	-	-	74
Fever (uninfected)	45	12	17	1	0	1.3%	0%	75
Perception (peripheral nerve)	71	4	0	0	-	0%	-	75
Rash	70	5	0	0	0	0%	0%	75

※: Use JCOG Toxicity Criteria.

Table 7.1.3. Adverse events in the IP-therapy group in b JCOG0509 (abstracted from the main analysis report)

Examination Items	Grade 0	Grade 1	Grade 2	Grade 3	Grade 4	Grade 3,4(%)	Grade 4(%)	Total
White blood cells	16	36	58	29	3	22.5	2.1	142
Neutrophils	6	10	43	51	32	58.5	22.5	142
Hemoglobin	20	24	65	24	9	23.2	6.3	142
Platelet	125	6	8	2	1	2.1	0.7	142
Total bilirubin	116	15	10	1	0	0.7	0	142
GOT	98	37	5	2	0	1.4	0	142
GPT	80	56	4	2	0	1.4	0	142
Creatinine	97	37	8	0	0	0	0	142
Anorexia	22	67	33	19	1	14.1	0.7	142
Nausea	30	67	36	9	0	6.3	0	142
Vomiting	89	38	10	5	0	3.5	0	142
Diarrhea	52	51	28	11	0	7.7	0	142
Mucositis (examination findings)-oral cavity	126	15	0	0	1	0.7	0.7	142
Hair loss	64	66	12	-	-	-	-	142
Onset of febrile neutropenia	127	-	-	14	1	10.6	0.7	142
Infection with Grade3-4 neutropenia -Bronchus	142	-	0	0	0	0	0	142
-Lung (pneumonia)	137	-	0	4	1	3.5	0.7	142
-Pharynx	142	-	0	0	0	0	0	142
-Upper respiratory tract-unclassifiable	141	-	1	0	0	0	0	142
-Bladder	142	-	0	0	0	0	0	142
-Kidney	142	-	0	0	0	0	0	142
-Urinary tract-subclassification impossible	142	-	0	0	0	0	0	142
Neuropathy: Sensory	127	13	2	0	0	0	0	142

Table 7.1.3.c Adverse events from a pilot trial of postoperative adjuvant chemotherapy with irinotecan plus cisplatin for high-grade neuroendocrine lung cancer (excerpt)

Examination Items	Grade 2	Grade 3	Grade 4	Grade 3,4(%)	Grade 4(%)	Total
White blood cells	17	7	0	17.5%	0%	40
Neutrophils	12	15	4	47.5%	10.0%	40
Hemoglobin	14	6	4	25.0%	10.0%	40
Platelet	2	0	0	0%	0%	40
Total bilirubin	0	0	0	0%	0%	40
GOT	0	0	0	0%	0%	40
GPT	1	0	0	0%	0%	40
Creatinine	0	0	0	0%	0%	40
Hyponatremia	0	5	0	12.5%	0%	40
Endotoxemia	3	1	0	2.5%	0%	40
Hypokalemia	0	4	0	10.0%	0%	40
Nausea	8	4	-	10.0%	-	40
Vomiting	4	2	0	5.0%	0%	40
Anorexia	2	0	-	0%	0%	40

Diarrhea	11	2	0	5.0%	0%	40
Fatigue	10	5	-	12.5%	0%	40
Infection	2	0	0	0%	0%	40

7.2. Anticipated Adverse Events Due to Pathogenesis

1） Esophageal primary

Esophageal bleeding; Esophageal pain; Esophageal stenosis; Esophageal obstruction; Esophageal perforation; Esophageal ulcer; Esophageal fistula; Hoarseness; Pharyngolaryngeal dysesthesia; Hypercalcemia of advanced disease; Tracheal stenosis; Tracheal obstruction; Esophageal anastomotic leakage; Recurrent laryngeal nerve palsy

2） Gastric primary

Gastric bleeding, upper gastrointestinal bleeding, gastric pain, gastrointestinal pain, back pain, nausea, vomiting, dyspepsia, gastroparesis, abdominal fullness, gastric stenosis, gastric obstruction, gastric perforation, duodenal bleeding, duodenal fistula, duodenal perforation, duodenal perforation, duodenal stenosis, gastrostomy, gastrointestinal fistula, gastrointestinal fistula, ileus, gastric anastomotic leak, gastrointestinal anastomotic leak

3） Small intestin/Colon and rectum

Bleeding from tumor; Ileus; Duodenal bleeding; Duodenal obstruction; Duodenal perforation; Duodenal stenosis; Small bowel obstruction; Small bowel stenosis; Small bowel ulcer; Small bowel perforation; Small bowel fistula; Vaginal fistula; Colonic fistula; Colonic stenosis; Colonic obstruction; Colonic perforation; Rectal stenosis; Rectal obstruction; Rectal fistula; Rectal perforation; Rectal fistula; Jejunal perforation; Jejunal perforation; Ileostomy; Ileovesical fistula; Intestinal fistula; Anal pain; Small bowel anastomotic leakage; Large bowel anastomotic leakage; Rectal anastomotic leakage; Pelvic infection

4） Pancreas, biliary tract, and liver

Body weight loss; Fever; Pancreatitis; Cholecystitis; Cholecystic obstruction; Gallbladder pain; Liver failure; Biliary tract infection; Hemorrhage from biliary tract; Duodenal hemorrhage; Duodenal fistula; Duodenal obstruction; Duodenal perforation; Duodenal stricture; Anastomotic ulcer; Biliary anastomosis leak; Pancreatic anastomosis leak; Anastomotic hemorrhage; Anastomotic stricture; Hemorrhage from tumor; Hemorrhage from portal vein stenosis/obstruction; Symptoms associated with portal vein stenosis/obstruction (Portal hypertension, Hepatic failure; Esophageal variceal hemorrhage; Gastritis; Ascites); Back pain; Abdominal pain

5） Anticipated Adverse Events Due to Metastasis

Liver failure, hepatic pain, fever, hepatic infection, respiratory failure, pulmonary infection, superior vena cava syndrome, abdominal distention, abdominal distention, ileus, esophageal obstruction, reverse smoking, gastric obstruction, duodenal obstruction, small bowel obstruction, colonic obstruction, rectal obstruction, rectal stenosis, anal bleeding, anorectal infection, nausea, diarrhea, constipation, ureteral obstruction, and urinary retention,urethral infection, urinary tract infection, bile duct obstruction, biliary obstruction, cholecystitis, biliary hemorrhage, gallbladder pain, pancreatitis, pain, narrowing of luminal organs near metastatic sites (tracheal/gastrointestinal tract, etc.), tracheal obstruction, bronchial stenosis, penetration with adjacent organs, perforation, hoarseness, pleural effusion, chest wall pain, pleural pain, atelectasis, hypercalcemia, disseminated intravascular coagulation, fracture, ataxia, cerebrovascular ischemia, intracranial hemorrhage,headache, dizziness, decreased level of consciousness, aphasia, seizures, spasticity

6） Paraneoplastic syndrome, etc.

(due to incompatible secretory syndrome) hyponatremia, (due to ectopic ACTH syndrome) personality changes, hypertension, hypokalemia, hyperglycemia, Lambert-Eaton myasthenic syndrome, subacute cerebellar degeneration associated with autoantibody production (including ataxia, dysarthria, and nystagmus of limbs), paraneoplastic encephalomyelitis, sensory neuropathy (including dementia, cranial nerve symptoms, dizziness, ataxia, autonomic ataxia, transverse paralysis, and sensory disturbance), thromboembolism, (due to gastrointestinal stenosis) aspiration, anaemia, tumor pain, acute renal failure, myositis, and pulmonary fibrosis

Complications associated with etiolation and gastrointestinal stent insertion are shown below.

- Percutaneous transhepatic cholangiodrainage (PTCD) procedures, including internal-external fistula tube placement:

  Pancreatitis, bile duct stricture, cholecystitis, gallbladder obstruction, gallbladder pain, liver failure, biliary

tract infection, liver infection, cholecystitis, septicemia, hemorrhage, PTCD tubing obstruction/deviation, peritoneal infection, pneumothorax, pleural pain, pleural effusion, intrapleural hemorrhage

- Biliary stenting:
  Pancreatitis, biliary stricture, cholecystitis, gallbladder obstruction, gallbladder pain, liver failure, biliary tract infection, liver infection, cholecystitis, sepsis, hemorrhage, peritoneum infection, pneumothorax, pleural pain, pleural effusion, intrapleural hemorrhage (in case of percutaneous placement), duodenal perforation, pneumonitis (in case of transendoscopic placement), stent obstruction/deviation, duodenal ulcer, and duodenal hemorrhage
- Choledochojejunostomy:
  Biliary tract infection; Pancreatitis; Cholecystitis; Liver infection; Sepsis
- Gastrointestinal stent insertion:
  Bleeding, perforation, pain, stent deviation, stent obstruction, ulceration, fever, sepsis, infection, diarrhea, constipation, urgency (colorectal), thyroid injury (esophagus), jugular arteriovenous injury (esophagus), and mediastinal abscess (esophagus).

7.3. Evaluation of Adverse Events/Reactions

The Common Terminology Criteria for Adverse Events v4.0 Japanese Translated JCOG Version (Japanese translation of NCI-Common Terminology Criteria for Adverse Events v4.0 (CTCAE v4.0)) (CTCAE v4.0-JCOG) will be used to assess adverse events/reactions. For CTCAE v4 0-JCOG in which Grade is defined by laboratory reference values at the institutional reference value, the "JCOG sharing reference range" will be used instead of the institutional reference value at each medical institution. For more information on JCOG sharing reference ranges, see JCOG website (see http://www.jcog.jp/doctor/tool/kijun.html)).

7.3.1. Grading of s adverse events

In grading of adverse events, each grading is closest to the definitions of Grade 0 4 (nearest match). Grading to a higher Grade when the definition of more than one Grade is comparable and when it is difficult to decide on either (highest grade).

Grading should also be given to Grade if specific actions are described, due to their clinical need. For example, patients may refuse oxygen inhalation or chest drainage, even when the patient's pleural effusion is increasing and oxygen inhalation or chest drainage is indicated. In such cases, grading is based on the medical judgment of what should have been done (what should be done) rather than on whether the treatment was actually given (what was actually done).

In the event of treatment-related deaths, original NCI-CTCAE states that the causative adverse event should be Grade 5, but the outcome of the serious adverse event is reported in the SAE report and reviewed in detail. Therefore, Grade 5 of the institutional physician's judgment is not likely to be changed, and whether or not the serious adverse event will result in death will be significantly affected by other factors than the event is not appropriate, so it is not appropriate to compare the frequency of the adverse event by Grade (%Grade 4 and%Grade 5, respectively) between treatment groups or between studies. Because of the poor significance of distinguishing between Grade 4 and Grade 5 in the tabulation, Grade 4 is not considered "Grade 5" in the recording form of this study. A discussion of the causal relationship between adverse events observed in treatment-related deaths and deaths should be included in the "Situation at Death" section of the treatment completion report form and follow-up form, and an urgent report should be made. For the adverse event items specified in "8.2. Testing and Assessment during Treatment" and "8.3. Testing and Endpoints after Treatment Completion" that are determined to be Grade 5 in the post-hoc review including the emergency report, Grade and the date of the first occurrence of the event should be included in the relevant record form (Treatment Course Record Form). If Grade 3 or greater is observed for any other adverse event, or if Grade 3/2/1 adverse event and treatment requires at least 24 hours of hospital stay or prolongation of hospital stay (see 10.1.1.3.)), the AE and Grade and the date of first occurrence should be included in the free form of the treatment course record.

Any Grade on the record form should be recorded in the medical record. Confirmed during site visit audit.

7.3.2. Determination of the causal relationship between adverse events and treatment

In determining the causal relationship between adverse events and treatment, patients are classified into 5 categories of "definite, probable, possible, unlikely, unrelated". Each "causality" is defined as "causality" when judged to be either "definite, probable, possible" and "no causality" when judged to be either "unlikely, unrelated" (see TABLE 7.3.2.

According to Grade of adverse events, serious adverse events requiring expedited reporting as specified in "10.1. Serious Adverse Events and Expedited Reporting" should be reported to the Research Secretariat in accordance with "10.2. Mandatory Reporting and Reporting Procedures of the Investigator".

Table 7.3.2. Criteria for a Causal Relationship Between Adverse Events and Treatment

	Determination	Approach to determination
Cause Fruit Concern Associate Ah Fistula	Definite	The AE is clearly related to the intervention<br>Adverse events are apparently caused/aggravated by protocol treatment and are unlikely to be due to exacerbation of the etiology or other factors (comorbidities, other medications/treatments, or incidents).
	Probable	The AE is likely related to the intervention<br>It is unlikely that the adverse event was caused/aggravated by progression of the underlying pathology or other factors (comorbidity, other drugs/treatments, incident) and is likely to be due to protocol treatment.
	Possible	The AE may be related to the intervention<br>It is plausible (plausible) that an adverse event is considered to have occurred/become more severe with protocol treatment, and unlikely to be due to exacerbation of the etiology or other factors (comorbidities, other medications/treatments, or incidents).
Cause Fruit Concern Associate In Pouch	Unlikely	The AE is doubtfully related to the intervention<br>It is considered plausible (plausible) that the adverse event is due to exacerbation of the pathogenic disease or other factors (comorbidity, other drugs/treatments, incident) rather than to the protocol treatment that it is caused/aggravated.
	Unrelated	The AE is clearly NOT related to the intervention<br>It is judged that the adverse event was caused/aggravated by aggravation of the pathogenic disease or other factors (comorbidity, other drugs/treatments, incident) and is unlikely to be caused by protocol treatment.

8. Examination and Evaluation

8.1. Baseline examination and evaluation before registration

8.1.1. Test conducted before registration (regardless of time before registration)

1） Histopathology (immunostaining requires chromogranin A and synaptophysin) (see Section 3.3)
2） HBs antigen; HBc antibody ※1; HBs antibody ※1, HBV-DNA※2

※1: For HBsAg positive, HBc and HBs antibodies are not required, and HBV-DNA, HBeAg, and HBe antibodies are measured.

※2: Positive results for at least one of the HBc and HBs antibodies also indicate HBV-DNA prior to initiation of therapy (see Section 6.4.1).

8.1.2. Test performed within 56 days before enrollment (liver primary (or unknown primary))

If the liver is primary (or of unknown primary), the following tests should be performed (all allowing for tests performed in other hospitals):

1） Upper gastrointestinal endoscope
2） Lower gastrointestinal endoscope
3） FDG-PET test
4） Otolaryngology (head and neck) examination
5） Urology consultation (male only)
6） Gynecologic exam (female only)

8.1.3. Tests performed within 28 days before enrollment

1) Contrast-enhanced CT※1 (slice thickness of 5 mm or less, if the patient is allergic to iodine), both contrast-enhanced MRI of the abdomen and plain computed tomography (CT) of the imaging range, which is considered to be indispensable below, are performed. All tests are not performed in other hospitals.

※The following areas are indispensable for each primary organ, and if there is another site suspected of metastasis, the radiographic extent is added accordingly.

Primary Organ	Essential radiographic area
Esophagus	Cervical, chest, or abdominal regions
Stomach, duodenum, small intestine, colon, appendix, rectum,<br>Extrahepatic bile duct, Vater ampulla, gallbladder, and pancreas	Chest, abdomen, and pelvis
Hepatic NEC<br>(liver primary or liver metastasis of unknown primary)	Neck, chest, abdomen, and pelvis

2) Endoscopic ※2

Primary Organ	Mandatory test ranges
Esophagus, stomach, duodenum, and ampulla of Vater	Upper gastrointestinal tract (no examination performed at another hospital)
Small intestine, extrahepatic bile ducts, gallbladder, and pancreas	Be not mandatory
Colon, appendix, and rectum	Lower gastrointestinal tract (no examination performed at another hospital)
Hepatic NEC<br>(liver primary or liver metastasis of unknown primary)	8.1.2. Refer to the test performed within 56 days before registration.

※2 Unnecessary if the primary lesion has been resected

3) 12-lead, resting electrocardiography

8.1.4. Tests performed within 14 days before enrollment

1) General condition: PS (ECOG), body weight
2) Physical findings
3) Peripheral blood count: white blood cell count, neutrophil count (ANC: rod + segmented karyocyte), hemoglobin, platelet count

4) Blood biochemistry: total protein, albumin, total bilirubin, AST (GOT), ALT (GPT), BUN, creatinine, LDH, ALP, sodium, potassium, calcium, magnesium, CRP, FBS (fasting blood glucose)
5) Creatinine clearance (CCr): CCr estimates by Cockcroft-Gault equation
   Cockcroft-Gault formula
   Male: Ccr = {(140-Age) × Body Weight (kg)}/{72 × Serum Creatinine Level (mg/dL)}
   Women: Ccr = 0.85 × {(140-Age) × Body Weight (kg)}/{72 × Serum Creatinine Level (mg/dL)}
6) Tumour markers: NSEs, ProGRP, CEAs, SCCs (esophagus primary only), CA19-9 (non-esophageal primary only)
7) Chest X-P (frontal) (substitutable if contrast-enhanced and plain chest CT is obtained)

8.2. Examination and evaluation during treatment

The following safety examination and evaluation are minimal in frequency: Performing examinations more frequently at the discretion of the treating physician is not prohibited.

However, the examination for efficacy evaluation should be performed at specified frequencies, unless progression is suspected, because dense frequency may lead to bias in the efficacy evaluation.

8.2.1. Safety endpoint assessed weekly (CTCAE v4 0 Japanese translation)

The following 1)-3) tests or evaluations should be performed at least weekly. In addition, all of the following items should be checked and evaluated on the scheduled date or the day before anticancer drug administration. However, at least weekly examinations or evaluations should be performed in the same manner until day 28 using the starting date of the last course as day 1, even when the treatment is completed or terminated.

1） Peripheral blood count: white blood cell count, neutrophil count (rod + segmented count), hemoglobin, and platelet count
2） Biochemical tests: albumin, total bilirubin, AST (GOT), ALT (GPT), creatinine, sodium, potassium, calcium, magnesium, CRP
3） Subjective and objective findings (described according to CTCAE v4.0JAPANESE TRANSLATION)
   - General disorders and administration site conditions: fever, fatigue
   - Ear and labyrinth disorders: Tinnitus, hearing impairment
   - Skin and subcutaneous tissue disorders: alopecia
   - Gastrointestinal disorders: Constipation, diarrhea, nausea, vomiting, oral mucositis
   - Metabolism and nutrition disorders: anorexia, dehydration
   - Nervous system disorders: dysgeusia, peripheral sensory neuropathy, peripheral motor neuropathy
   - Musculoskeletal and connective tissue disorders: myalgia, arthralgia
   - Infections and parasites: bronchial infection, pulmonary infection, upper respiratory tract infection, mediastinal infection, pleural infection, catheter-related infection, biliary tract infection, gallbladder infection, and urinary tract infection
   - Blood and lymphatic system disorders: Febrile Neutropenia
   - Vascular disorders: Phlebitis
   - Respiratory, thoracic and mediastinal disorders: dyspnea, hypoxia, pneumonitis

8.2.2. Safety examination and evaluation for each course

1) General condition: Body weight
2) Blood chemistry: LDH, ALP FBS (fasting glucose)

8.2.3. Safety examination and evaluation to be performed as necessary

1） When dyspnea is observed
   - Chest X-P, percutaneous oxygen saturation: SpO2, arterial blood gases: PaO_2
2） If an arrhythmia is observed
   - 12-lead, resting electrocardiography
3） When HBs antigen, HBs antibody, or HBc antibody is positive
   - HBV-DNA (see 6.4.1.).

8.2.4. Efficacy end point

The following tests will be performed every 6 weeks during protocol treatment (±1 week allowed: 6 ± 1 week, 12 ± 1 week, and 18 ± 1 week after the start date of protocol treatment). Tumor response will be assessed according to "11.1. Response Evaluation". Evaluation of the response will be performed using the same test conditions and test methods as the baseline evaluation.

If CT is allergic to contrast material, it is evaluated with plain CT and/or contrast-enhanced MRI. Allergy to contrast media on MRI is assessed by plain CT or plain MRI. When the use of CT contrast medium and MRI contrast medium becomes difficult due to renal dysfunction, it is evaluated by simple CT or simple MRI.

1） Tumour markers: NSEs, ProGRP, CEAs (≧all cases), SCCs (esophagus primary only), CA19-9 (non-esophageal primary only)

2） Contrast-enhanced CT: In principle, the extent of imaging is as follows. () Areas in can be omitted if there is no lesion

Primary Organ	Essential radiographic area
Esophagus	Cervical, chest, or abdominal regions
Stomach, duodenum, small intestine, colon, appendix, rectum,<br>Extrahepatic bile duct, Vater ampulla, gallbladder, and pancreas	(chest), abdomen, and pelvis
Liver NEC (liver primary or liver metastasis of unknown primary)	(neck), (chest), abdomen, and pelvis

8.3. Examination and evaluation after completion of treatment

8.3.1. Efficacy evaluation after completion of treatment

After completion of the protocol treatment, examination and evaluation are made at the following timing:

The following tests should be performed at least every 6 weeks until progressions are confirmed or death.

If progressions are observed, only observation of the outcome will be continued.

1） Tumour markers: NSEs, ProGRP, CEAs (≧all cases), SCCs (esophagus primary only), CA19-9 (non-esophageal primary only)

2） Contrast-enhanced CT: In principle, the extent of imaging is as follows. Areas in parentheses can be omitted if there is no lesion

Primary Organ	Essential radiographic area
Esophagus	Cervical, chest, or abdominal regions
Stomach, duodenum, small intestine, colon, appendix, rectum,<br>Extrahepatic bile duct, Vater ampulla, gallbladder, and pancreas	(chest), abdomen, and pelvis
Liver NEC (liver primary or liver metastasis of unknown primary)	(neck), (chest), abdomen, and pelvis

8.4. Information on post- study treatment

After completion/termination of protocol treatment, the following items will be recorded on the Follow-up Form at each follow-up survey:

1） Content of post-study treatment (if post-study treatment is performed)

2） After protocol treatment termination, the initiation date of the first post-study treatment ((if post-study treatment is performed)

3） PS at the start of post-study treatment

8.5. Study calendar

If the primary site is the esophagus, stomach, duodenum, small intestine, colon, appendix, rectum, extrahepatic bile duct, ampulla of Vater, gallbladder, or pancreas

	Before registratikon Record Pre	After end of chemotherapy		From the date of protocol treatment discontinuation Within 28 days	Until progression after completion of protocol treatment	
		Course Before initiation of the course	During the course		Only at the start of post-treatment	Every 6 weeks
Physical findings						
Body weight	○^{14}	○				
PS	○^{14}				■	
Physical findings	○^{14}	○			■	
Laboratory tests						
WBC, differential (neutrophil) Hb, platelets	○^{14}	○	●	●		
Alb, T-Bil, AST, ALT, Cr, Na, K, Ca, Mg, CRP	○^{14}	○	●	●		
LDH, ALP, FBS	○^{14}	○				
Total protein, BUN, Ca	○^{14}					
NSE, ProGRP、CEA	○^{14}		△			
SCC (esophagus primary only), CA19-9 (other than primary esophageal)	○^{14}		△			
HBs antigen, HBc antibody, and HBs antibody	○^{前}					
Chest X-P (can be substituted if CT is taken)	○^{14}					
12-lead, resting electrocardiography	○^{28}					
Upper gastrointestinal endoscope (Primary: esophagus, stomach, duodenum)	○^{28}					
Lower gastrointestinal endoscope (Primary: colonic, appendix, rectum)	○^{28}					
Efficacy evaluation						
Contrast-Enhanced CT*	○^{28}		△			△
Toxicity evaluation						
Subjective symptom check		○	●	●		
Objective symptom check		○	●	●		
Submission of CRFs						
Pre-treatment Form		□				
Treatment Form			□			
Off-treatment Form				□		
Follow-up Form					2 times/year	

○^{前}: Conduct before registration, ○^{28}: Perform within 28 days before registration, ○^{14}: Perform within 14 days before registration

○:Conduct, ♀: Implementation at least once a week

△: Every 6 weeks (see 8.2.4.), ∞: Only once at the beginning of aftertreatment, □: Submitted.

*See 8.1.3 for the shooting range. Contrast-induced allergy or renal dysfunction is assessed by plain CT or contrast-

enhanced MRI.

※Follow-up Forms will be sent up to 1 years after completion of accrual and will be submitted after 1 years of registration in the individual patient according to the closing date of registration.

For liver NEC (liver primary or liver metastasis of unknown primary)

	Before registratikon Record Pre	After end of chemotherapy		From the date of protocol treatment discontinuation Within 28 days	Until progression after completion of protocol treatment	
		Course Before initiation of the course	During the course		Only at the start of post-treatment	Every 6 weeks
Physical findings						
Body weight	○[14]	○				
PS	○[14]				■	
Physical findings	○[14]	○			■	
Laboratory tests						
WBC, differential (neutrophil) Hb, platelets	○[14]	○	●	●		
Alb, T-Bil, AST, ALT, Cr, Na,K, Ca, Mg, CRP	○[14]	○	●	●		
LDH, ALP, FBS	○[14]	○				
Total protein, BUN, Ca	○[14]					
NSE, ProGRP、CEA、CA19-9	○[14]		△			
HBs antigen, HBc antibody, and HBs antibody	○[前]					
Chest X-P (can be substituted if CT is taken)	○[14]					
12-lead, resting electrocardiography	○[28]					
Upper gastrointestinal endoscope	○[56]					
Lower gastrointestinal endoscope	○[56]					
FDG-PET	○[56]					
Otolaryngologic examination	○[56]					
Urology consultation (male only)	○[56]					
Gynecologic exam (female only)	○[56]					
Efficacy evaluation						
Contrast-Enhanced CT*	○[28]		△			△
Toxicity evaluation						
Subjective symptom check		○	●	●		
Objective symptom check		○	●	●		
Submission of CRFs						
Pre-treatment Form		□				
Treatment Form			□			
Off-treatment Form				□		
Follow-up Form					□ 2 times/year	

○[前]: Conduct before registration, ○[56]: Perform within 56 days before registration, ○[28]: Perform within 28 days before registration,

○[14]: Implemented within 14 days prior to enrollment

○:Conduct, ♀: Implementation at least once a week

△: Every 6 weeks (see 8.2.4.), ∞: Only once at the commencement of post-treatment, □: Submitted.

*See 8.1.3 for the shooting range. Contrast-induced allergy and renal dysfunction are evaluated by plain CT or contrast-enhanced MRI.

※Follow-up Forms will be sent up to 1 years after completion of accrual and will be submitted after 1 years of

registration in the individual patient according to the closing date of registration.

9. Data collection

9.1. Case Report Form (CRF)

9.1.1. Types of CRF and submission deadlines

The case report forms (CRF) used in this study and their submission deadlines are as follows:

1) Pre-treatment report (blue)- Less than 2 weeks after enrollment
2) Treatment course records- Every 2 cycles/less than 2 weeks after end of protocol treatment
   3）-1 Treatment (yellow)
   3）-2 Test (yellow)
   3）-3 Adverse events (yellow)
3) Tumor shrinkage report (green) - Less than 2 weeks after judgment of effect
4) End of treatment report (red) - Less than 2 weeks after discontinuation/end of protocol treatment
5) Follow-up investigation (white)- By the deadline indicated in the Follow-up Investigation Form

- For "1) Pre-treatment report to 4) End of treatment report", CRFs with basic patient information (enrollment code, facility name) pre-printed on them would be sent by post from the data center. If the CRFs do not arrive within one week of enrollment, or if the CRFs have been lost/damaged, the data center should be contacted by telephone, and a request should be made for them to be re-issued.
- "5) Follow-up investigation" would be sent by post from the data center at the time of follow-up investigations that are conducted at the same time as monitoring and interim/final analyses at the data center.

9.1.2. Storage CRF

- Completed CRFs must all be archived at the facility as photocopies or in electronic form.
- Copies of CRFs should be kept archived until the final analysis report is issued for reference while filling other CRFs, or for review while retrieving information from the data center.

9.1.3. Method of sending CRF

- All CRF must be sent by post or handed over in person at the data center. They must not be sent by FAX.
- To avoid the risk of personal patient information being leaked, the patient enrollment code should be used, instead of using the patient medical chart number at the facility, when contacting the data center for request of CRF dispatch.

9.1.4. Correction of the contents of CRFs

If any data necessary for the CRF are found to be missing or there are inappropriate category classifications after the start of the study, the CRFs may be corrected with the agreement of the head of the data center and the clinical trial secretariat, in a manner not exceeding the scope of data collected as prescribed in "8. Evaluation item/Clinical laboratory tests/Evaluation schedule", and within the scope deemed not to increase medical and financial burden on the enrolled patient from the CRF correction. Modification of CRFs that do not require the main body of the protocol to be revised is not considered a protocol revision by JCOG. Reports to the head of the medical institution related to CRF correction and the request of application for revision should follow the rules of the facility.

10. Reporting of "disease or the like"(adverse events)

Site investigator should report to Study Coordinator/Principal Investigator (Study Chair) if a serious adverse event ("disease or the like" on Clinical Trials Act) occurs in accordance with the regulations of Clinical Trials Act (Law No. 16, 2017), Enforcement Regulations of Clinical Trials Act (MHLW Notification No. 17, 2018) and the regulations in this chapter based on the relevant notifications.

The most recent version of the report is available on the MHLW website [1)] and on the JCOG website [2)]. Use the most recent version of the report.

Serious adverse events occurring after the initiation of protocol treatment (after the date of registration if death) by the date of final follow-up will be subjects.

1）http://www.mhlw.go.jp/stf/seisakunitsuite/bunya/0000163417.html
2）http://www.jcog.jp/doctor/todo/researcher/harmfulness.html
3）http://www.pmda.go.jp/safety/reports/hcp/pmd-act/0002.html

10.1. Serious Adverse Events and subjects of Expedited Reporting.

Serious adverse events are defined as any of the following:
(These are classified as "disease or the like" on Clinical Trials Act.)

1） Death
2） Diseases that may lead to death
3） "Disease or the like" requiring hospitalization or prolongation of hospital stay for treatment.
4） Disability
5） "Disease or the like" that may lead to disability
6） Serious "disease or the like" according to 1) to 5)
7） Congenital disease or abnormality in later generations

Of these, those that fall under any of the following categories shall be regarded as serious adverse events for which Expedited Reporting is mandatory.

1) Death

(i) All deaths that occur after registration and before the start of protocol treatment
(ii) All deaths (with or without causality to protocol treatment) that occur during protocol treatment or within 30 days of the last treatment day
(iii) Death that occur after 31 days from the last treatment date that are causally related to protocol treatment (definite, probable, possible)

2) "Disease or the like" that may lead to death

(i) Grade 4 adverse events that occur during protocol treatment or within 30 days of the last treatment day (excluding events in Table 10.1)
(ii) Grade 4 adverse events that occur after 31 days from the last treatment date (excluding events in Table 10.1) that are causally related to the protocol treatment (definite, probable, possible)

3) "Disease or the like" requiring hospitalization or prolongation of hospital stay※1 for treatment

(i) Grade 3/2/1 adverse events that occur during or within 30 days of protocol treatment and requiring at least 24 hours of hospitalization or prolongation of hospital stay※1 to treat the adverse event (excluding the event in Table 10.1).
(ii) Grade 3/2/1 adverse events that occur 31 days after the last treatment day and requires 24-hour or longer hospitalization or prolongation of hospital stay※1 for treatment and causally related to protocol treatment (definite, probable, possible) (excluding the events in Table 10.1)

※ 1 "Hospitalization or prolongation of hospital stay" refers only to those for which hospitalization of at least 24 hours or prolongation of hospital stay is medically required for the treatment of an adverse event. The followings are not subjects for reporting:

- Hospitalization or prolongation of hospital stay performed for follow-up of adverse event that has disappeared or improved

- Hospitalization or prolongation of hospital stay for reducing patient burden, e.g. patients from distant areas.
- Hospitalization or prolongation of hospital stay for other medically unnecessary situation

※ 2 "Unexpected" refers to those not listed in "7. Expected Adverse Events"

4) Disability, 5) "Disease or the like" that may lead to disability

Permanent or marked disability/dysfunction (excluding myelodysplastic syndromes, secondary cancers, etc.) or possible medical situation

6) Serious "disease or the like" similar to 1) to 5)

7) Congenital disorders or abnormalities in later generations

Table 10.1. Adverse events excluded from the subjects of Expedited Reporting

SOC※(CTCAE ver4.0)	AE term
Blood and lymphocyte disorders	Anemia, bone marrow hypocellular
Gastrointestinal disorders	Constipation
General disorders and local symptoms	Fever
Infections and infestations	Viral hepatitis
Clinical laboratory test	ALP increased, CD4 lymphocytes decreased, high cholesterol, GGT increased, lipase increased, lymphocytes decreased, neutrophils decreased, platelet count decreased, serum amylase increased, WBC decreased, hyponatremia, hypokalemia, hyperglycemia, and hypoglycemia
Metabolism and nutritional disorders	Obesity, anorexia, hyperuricemia, and hypoalbuminemia
Musculoskeletal and connective tissue disorders	Fibrosis deep connective tissue and superficial soft tissue fibrosis
Renal and urinary disorders	Chronic kidney disease
Respiratory, thoracic, and mediastinal disorders	Sinus disorder and sleep apnea
Skin and subcutaneous tissue disorders	Hypohidrosis

※ SOC:System Organ Class

10.2. Investigator's reporting requirements and procedures

10.2.1. Expedited Reporting

In the event of a serious adverse event for which Expedited Reporting is mandatory, the Subinvestigator must promptly inform the Investigator. If the Investigator cannot be contacted, the Site Coordinator or Subinvestigator must take over the responsibility of the Investigator. The Investigator must report adverse events according to the following procedures.

Attention should be paid not to include the patient's name and medical record number when sent.

Serious adverse events that occur after the initiation of protocol treatment (after the date of registration if death) by the date of final follow-up are subjects of Expedited Reporting.

1) "Disease or the like" that may lead to death or death specified in 10.1 1) and 2).

Primary reporting:

The Subinvestigator who is aware of the occurrence of adverse events will promptly notify the Investigator. The Investigator who receives the notice should fill out JCOG Adverse Event Report Form(for institution) and the "Disease or the like" Report Form to Certified Review Board specified in Clinical Trials Act Enforcement Regulations as far as possible and contact Principal Investigator/Study Coordinator via e-mail within 72 hours of knowledge of the occurrence of the adverse event.

Secondary reporting:

The Investigator should add detailed information on adverse events to JCOG Adverse Event Report Form(for

institution) and the "Disease or the like" Report Form to Certified Review Board specified in Clinical Trials Act Enforcement Regulations within 7 days of knowledge of the occurrence of adverse events and send them to the Principal Investigator/Study Coordinator via e-mail. If necessary, attach copies of laboratory data, images, autopsy report, etc.

2) 10.1. 3)"Disease or the like" or other medically important condition requiring hospitalization or prolongation of hospital stay for treatment. Adverse events are judged to be either of 10.1. 4)-7)

The Subinvestigator who is aware of the occurrence of adverse events will promptly notify the Investigator. The Investigator who receives notice must fill out JCOG Adverse Event Report Form (for institution) and the "Disease or the like" Report Form to Certified Review Board specified in Clinical Trials Act Enforcement Regulations within 10 days of knowledge of the occurrence of an adverse event and send them to the Principal Investigator/Study Coordinator via e-mail. If necessary, attach copies of laboratory data, images, autopsy report, etc.

3) Additional reporting

If new information is obtained after conducting the above reporting, the Investigator must add information to JCOG Adverse Event Report Form (for institution) and the "Disease or the like" Report Form to Certified Review Board specified in Clinical Trials Act Enforcement Regulations and report it as needed.

Table 10.2.1. Summary of Adverse Events which are subjects for Expedited Reporting and the deadline of reporting to Principal Investigator/Study Coordinator

Causal relationship	The patient was hospitalized due to Grade 1-3 AE. Other medically important conditions		Grade 4		Death	
	Expected	Not expected	Expected	Not expected	Expected	Not expected
Present	No need of reporting	Primary reporting: within 10 days Additional reporting: as needed	Primary reporting: within 72 hours Secondary reporting: within 7 days Additional reporting: as needed			
None	<Only on-treatment or within 30 days of last protocol treatment day>					
	No need of reporting	Primary reporting: within 10 days Additional reporting: as needed	Primary reporting: within 72 hours Secondary reporting: within 7 days Additional reporting: as needed			

* 4) Disability, 5) "Disease or the like" that may lead to disability, and 6) "Disease or the like" that are serious similar to 1) to 5) in 10.1., 7) Congenital disorders or abnormalities in later generations

※ "Unexpected" refers to those not listed in "7. Expected Adverse Events"

Table 10.2.1. Summary of Adverse Events which are subjects for Expedited Reporting and the deadline of reporting to Principal Investigator/Study Coordinator

Causal relationship	The patient was hospitalized due to Grade 1-3 AE. *Other medically important conditions		Grade 4		Death	
	Expected	Not expected	Expected	Not expected	Expected	Not expected
Present	Primary reporting: within 10 days Additional reporting: as needed	Primary reporting: within 10 days Additional reporting: as needed	Primary reporting: within 72 hours Secondary reporting: within 7 days Additional reporting: as needed			
None	<Only on-treatment or within 30 days of last protocol treatment day>					
	Primary reporting: within 10 days Additional reporting: as needed	Primary reporting: within 10 days Additional reporting: as needed	Primary reporting: within 72 hours Secondary reporting: within 7 days Additional reporting: as needed			

* 4) Disability, 5) "Disease or the like" that may lead to disability, and 6) "Disease or the like" that are serious similar to 1) to 5) in 10.1., 7) Congenital disorders or abnormalities in later generations

※ "Unexpected" refers to those not listed in "7. Expected Adverse Events"

10.2.2. Reporting to the Administrator of participating medical organizations

If an Adverse Event which is subjects for Expedited Reporting occurs and is assessed as causal after reporting to Principal Investigator and reported to Certified Review Board, the Investigator must report it to the Administrator of the relevant medical institution in accordance with the requirements of the medical institution.

10.3. Responsibilities of Principal Investigator/Study Coordinator

10.3.1. Determination of necessity of suspension of registration and emergency notification to institutions

Principal Investigator/Study Coordinator who received the report from the Site Investigator should report to Group Chair and determine the urgency, significance, and impact of the report. If needed, take measures such as suspending registration (contacting JCOG Data Center and all participating institutions) and urgently communicating information to participating institutions. Telephone calls can be made to Data Center and institutions as urgent, but they should also be promptly contacted by document (e-mail).

10.3.2. Reporting to JCOG Operations Office and Certified Review Board and MHLW

1) Reporting from the Principal Investigator/Study Coordinator to JCOG Operations Office

Principal Investigator/Study Coordinator should consult with Group Chair and report to JCOG Operations Office (Safety Contact) by e-mail within 72 hours of knowledge of the occurrence of the adverse event, if reported AE is considered to meet the adverse events specified in 10.2.1. 1) and 2) In doing so, to the extent feasible, Principal Investigator/Study Coordinator should send "JCOG Adverse Event Report Form (for institution)" sent from the institution, "Disease or the like Report Form" addressed to Certified Review Board as stipulated in Clinical Trials Act Enforcement Regulations, and attach "JCOG Adverse Event Report (for Study Coordinator)" with Study Coordinator/Principal Investigator's view (including judgments of causality and expectation, and judgments of continuation/discontinuation of the study)". For the expected adverse events of 1) or 2) in 10.2.1., include a discussion not only of the individual patient's course but also of whether the frequency of appearance is within the expected range.

2) Reporting to Certified Review Board

JCOG Operations Office (Safety Contact) reviews the appropriateness of the judgement of causality and expectation of adverse events reported in the above procedures and can ask Principal Investigator/Study Coordinator to reconsider them if there is any doubt. Adverse events considered by Principal Investigator/Study Coordinator and JCOG Operations Office to be related to the protocol treatment and to be the subject of reporting in the following tables should reported to Certified Review Board through JCOG Operations Office.

If there is a disagreement between Principal Investigator/Study Coordinator and JCOG Operations Office, report it to Data and Safety Monitoring Committee and seek final judgment from the Chair of Data and Safety Monitoring Committee. However, if the reporting may exceed deadlines of reporting, the report can be tentatively reported as "causal" to Certified Review Board.

Reporting subjects and reporting deadline

Principal Investigator/Study Coordinator must report to Certified Review Board through JCOG Operations Office within the following time periods after knowledge of the occurrence of adverse events.

Causal relation ship	The patient was hospitalized due to Grade 1-3 AE. Other medically important condition		Grade 4		Death	
	Expected	Not expected	Expected	Not expected	Expected	Not expected
Present	No need of reporting	Within 15 days	Within 15 days	Within 7 days	Within 15 days	Within 7 days
None	No need of	No need of	No need of	No need of	No need of	No need of

	reporting	reporting	reporting	reporting	reporting	reporting

※ "Unexpected" refers to those not listed in "7. Expected Adverse Events"

Principal Investigator/Study Coordinator must report adverse events to Certified Review Board through JCOG Operations Office within the following deadlines after knowledge of the occurrence of adverse events.

Causal relation ship	The patient was hospitalized due to Grade 1-3 AE. Other medically important condition		Grade 4		Death	
	Expected	Not expected	Expected	Not expected	Expected	Not expected
Present	Within 30 days	Within 15 days	Within 30 days	Within 15 days	Within 15 days	Within 15 days
None	No need of reporting	No need of reporting	No need of reporting	No need of reporting	No need of reporting	No need of reporting

※ "Unexpected" refers to those not listed in "7. Expected Adverse Events"

3) Reporting to MHLW

Principal Investigator/Study Coordinator of the study with unapproved or off-label health care should report "Disease or the like" Report Form specified in Clinical Trials Act Enforcement Regulations to MHLW through JCOG Operations Office, if an adverse event is considered to be unexpected and to have a causal relationship to the protocol treatment (See 10.5. Responsibilities of the Data and Safety Monitoring Committee).

Reporting subjects and reporting deadlines

Principal Investigator/Study Coordinator should report to the MHLW through JCOG Operations Office within the following deadlines after knowledge of the occurrence of adverse events.

※ Pharmaceuticals and Medical Devices Agency Safety Division I (trk-shippeitouhokoku@pmda.go.jp)

Causal relation ship	The patient was hospitalized due to Grade 1-3 AE. Other medically important condition		Grade 4		Death	
	Expected	Not expected	Expected	Not expected	Expected	Not expected
Present	No need of reporting	Within 15 days	No need of reporting	Within 7 days	No need of reporting	Within 7 days
None	No need of reporting	No need of reporting	No need of reporting	No need of reporting	No need of reporting	No need of reporting

※ "Unexpected" refers to those not listed in "7. Expected Adverse Events"

4) Additional reporting

Following receipt of secondary or additional reports from the Investigator, Principal Investigator/Study Coordinator must add additional information from the primary report and their views to JCOG Adverse Event Report Form (for the institution) and the "Disease or the like" Report Form to Certified Review Board set out in Clinical Trials Act Enforcement Regulations, and promptly contact JCOG Operations Office (Safety Contact) by e-mail. If the report was sent to Certified Review Board and the MHLW in the primary reporting,, the secondary reporting and additional reporting must be made in the same manner.

10.3.3. Notification to the Site Investigators

When reported to Certified Review Board, Principal Investigator/Study Coordinator should inform the Investigators of all participating institutions of the review results and recommendations by documents (e-mail is allowed). Principal Investigator/Study Coordinator must inform the Investigators without waiting for Certified Review Board review if there is any urgent information to be disseminated. In the event that reported to the MHLW, Principal Investigator/Study Coordinator should notify the Investigators of all participating institutions.

In addition, even if no reporting is made to Certified Review Board, Principal Investigator/Study Coordinator must inform the Investigator of the reporting institution of the decision of Principal Investigator/Study Coordinator by documents (e-mail is allowed).

10.3.4. Assessment of Adverse Events in Periodic Monitoring

During Periodic Monitoring, Principal investigator/Study Coordinator should carefully review the adverse events in the Monitoring Reports issued by the Data Center and ensure that there are no missed reporting from the participating institutions. It should also be confirmed that all reported adverse events are listed in the Monitoring Reports. The presence or absence of a missed reporting should be indicated in the column of the results of Group review on the Periodic Monitoring Report.

10.4. Responsibilities of the Site Investigators at the participating institutions (including the relevant institution)

In accordance with the instructions of Principal Investigator/Study Coordinator, the Site Investigator at the participating institution should report to the administrator of the relevant institution if the adverse event is subjects of reporting of "disease or the like" to Certified Review Board in accordance with the regulations of the relevant institution.

10.5. Responsibilities of the Data and Safety Monitoring Committee

JCOG Operations Office (Safety Contact) should check the details of the adverse event reports received from the Principal Investigator/Study Coordinator according to the procedures described in 10.3.2. and should report them to Certified Review Board and the MHLW according to the procedures described below, with the presence or absence of causality or expectation.

In addition, Principal Investigator and JCOG Operations Office can hear the opinions of JCOG Data and Safety Monitoring Committee according to the reported adverse events. If a review request is issued, Data and Safety Monitoring Committee can review the appropriateness of the institutional response to adverse events and the propriety of continuation of the study in a consensus or written form.

In addition, the submitted information (JCOG Adverse Event Report (for institution), JCOG Adverse Event Report (for Study Coordinator), "Disease or the like" Report, etc.) will be stored semi-permanently in JCOG Operations Office.

Subjects, destination and deadlines for reporting after the knowledge by Investigator/Study Coordinator are as described in 10.3.2.

11. Response Evaluation and Endpoint Definition

11.1. Response assessment (only for patients with measurable disease)

Tumour response assessment will be performed according to the following steps according to the [49] of version 1. 1-Japanese translational JCOG version-Revised RECIST guideline (version 1. 1) Revised new guidelines for the assessment of treatment response in solid tumours (RECIST guidelines). RECISTv1. The 0 original article stipulates that "the use of this guideline for the purpose of determining continuation of treatment is not the subject of this guideline." Similar statements continue to be included in RECISTv1 1 as follows.

> "Many oncologists make decisions about whether to continue treatment based on both objective imaging criteria and symptom-based criteria for follow-up of patients with malignancies in their daily clinical practice, but these revised guidelines are not intended to be used to make decisions about whether or not to continue treatment in these individual patients, unless the treating oncologist determines that it is appropriate."

Therefore, the "overall effect" as determined by RECIST Guideline-based response assessment should be used to determine whether a drug or regimen shows encouraging results that merit continued developmental studies. In other words, judgment of whether or not to continue treatment in individual patients should not be based on CR/PR/SD/PD of overall efficacy, but rather on "clinical judgment" based on comprehensive consideration of symptoms, physical findings, and various laboratory data, in addition to imaging findings.

Therefore, it may be clinically appropriate to continue protocolized treatment, even when PD (Progressive Disease: progression) is judged as an overall response based on the assessment of response based on imaging. In this case, the pros and cons of continuing protocol treatment should be determined based on clinical judgment, regardless of response assessment, but the date of the event for progression-free survival, which is judged to be an overall effect of PD, should be used. This is due to three reasons: (i) it may be possible to decide whether protocol treatment should be continued for each group; (ii) RECIST is a criterion intended to standardize not only response rates but also progression-free survival; and (iii) the standard definition of US Cooperative Group is that PD is the event of progression-free survival for any reason if the overall response is PD.

On the other hand, if a physician judges "clinical progression" based on clinical and comprehensive judgment not based on diagnostic imaging, even if PD is not met by the response criteria based on diagnostic imaging, protocol treatment should be discontinued in accordance with "6.2.2. Criteria for discontinuation of protocol treatment". If "clinical exacerbation" is judged, even if "PD" is not judged by the response evaluation, the day of "clinical exacerbation" is considered as an event of progression-free survival. This is because imaging is often not performed as planned after a patient is judged to have a "clinical progression" and therefore the risk of overestimating progression-free survival is greater if "clinical progression" is not an event for progression-free survival. It is also statistically incorrect (informative censoring) to treat "clinical progression" as "censoring" progression-free survival, as it would censoring patients at increased risk of progression or death.

In RECISTv1 1, the original article described "definite progression (unequivocal progression") in the PD criteria for non-target lesions as "marked progression of non-target lesions that deserves discontinuation of treatment as an increase in total tumor burden" and therefore described "marked progression of non-target lesions" as "judgement of whether or not to continue treatment in individual patients" in some of the PD criteria for non-target lesions, which is confusing. It should be noted that this "unequivocal progression" is a criterion of judgment restricted to "PD of non-target lesions".

The relation between the events of 'PD', 'clinical progression', 'progression', and progression-free survival in JCOG is as in the lower panel.

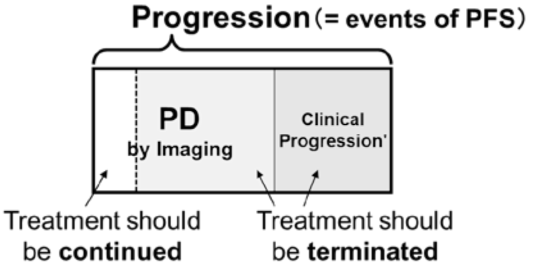

Figure 11.1. Relationship Between Exacerbations, PD on Imaging, and Clinical Exacerbations.

11.1.1. Baseline Evaluation

「8.1. According to "Pre-registration Evaluation Items" contrast enhanced-enhanced computed tomography (CT) with a range of indispensable for the primary organ is used to identify neoplastic lesions before enrollment, and each lesion is classified into "measurable lesions" and "unmeasurable lesions". If CT is allergic to contrast medium, both contrast-enhanced MRI and plain CT should be performed.

Tumor diameters are measured by CT or MRI in cross-sectional views, and sagittal and coronal measurements by three-dimensional reconstructed images are not used. Baseline assessment will be done using the latest imaging studies within 28 days prior to enrollment. If the imaging test is re-examined after enrollment and before the start of treatment, the latest imaging test with re-examination should be used.

In this study, the tumour markers NSEs and ProGRP are used to evaluate the response of non-target lesions.

11.1.2. Definition of measurable lesions

Lesions that fall under any of the following conditions are considered measurable lesions (measurable lesion):

1) Non-nodal disease (non-nodal disease) of 10 mm or greater in greatest dimension on CT or MRI with a slice thickness of 5 mm or less
2) CT or MRI of 5 mm or less slice thickness showing lymph node lesion of 15 mm or more in short diameter (Nodal lesions with short diameters between 10 mm and less than 15 mm are defined as non-target lesions, and those with short diameters less than 10 mm are not)

All other lesions will be non-measurable (non-measurable lesion).

Caution should be exercised because the following lesions are not measurable regardless of the examination method or the size of the lesion.

- Bone lesions (excluding osteolytic lesions with measurable soft tissue components)
- Cystic lesion
- Leptomeningeal lesions
- Ascites, pleural effusion, and pericardial effusion
- Lymphangiosis of the skin and lungs
- Palpable but not measurable abdominal mass or enlargement of abdominal organs
- Superficial skin lesions

11.1.3. Target Lesion Selection and Baseline Recording

Up to five measurable lesions, in descending order of diameter (non-lymph node lesions are long diameters and nodal lesions are short diameters), up to two lesions per organ are selected to be target lesions (target lesion) among measurable lesions at enrollment. Selection should take into account the universal inclusion of as many organs with measurable disease as possible and the reproducibility or ease of measurement (reproducible repeated measurement) of repeated measurements (avoiding lesions that are not measurable even if they are large in diameter).

For selected target lesions, in order from cranial to caudal, site (code), test method, test date, long diameter of non-lymph node target lesion, short diameter of nodal target lesion, and sum of all target lesion diameters (hereafter, sum of diameters) will be recorded in Pretreatment Report 3.

11.1.4. Baseline recording of non-target lesions

For lesions not selected as target lesions, all measurable or non-target lesions (non-target lesion) should be recorded as site of lesion (code), method of examination, and date of examination in Pretreatment Report 3. Multiple non-target lesions within the same organ may be recorded as a single lesion (e.g., multiple enlarged pelvic lymph nodes, multiple liver metastases).

11.1.5. Determining tumor response

Evaluation of target and non-target lesions will be performed every 6 weeks according to "8.2 Testing and Evaluation during Treatment" in the same manner as at enrollment. Target lesion diameter, non-target lesion disappearance or progression will be recorded in the "Tumor Reduction Effect Report".

11.1.6. Response Evaluation Criteria for Target Lesions

・CR(Complete Response): Complete response

When all non-lymph node target lesions disappear and all nodal target lesions have a short diameter of

less than 10 mm. If a nodal target lesion is selected at baseline, the effect of the target lesion may be CR even if the sum of diameters is not 0 mm.

・PR(Partial Response): Partial response

30% or more reduction in target diameter sum compared to baseline diameter sum

・PD(Progressive Disease): Progress

Compared to the minimum diameters (when the baseline is the minimum value during the passage, this is the minimum sum of diameters), the sum of the target disease increases by more than 20%, and the sum of diameters increases by more than 5 mm even in absolute value.

・SD(Stable Disease): Stability

There is no reduction corresponding to PR and no increase corresponding to PD compared to the smallest sum of diameters during the course

・Lack of study; Not all Evaluated

If the test cannot be performed for any reason or if neither CR, PR, PD, or SD can be determined

Pre-treatment sum of diameters-sum of diameters at study
Percentage of reduction of the diameter sum =---------------× x 100%
Pre-treatment diameter sum

Sum of diameters at study-minimum sum of diameters
Increasing Percentage of Diagram =---------------× x 100%
Minimum sum of diameters

※ Measured target lesion diameters are recorded whenever measurable (e.g., <5 mm). If the target lesion diameter is judged to be too small to be measured (too small to measure), the diameter should be 0 mm if the tumor lesion is judged not to be retained, and 5 mm if the tumor lesion is judged to be retained, regardless of the CT slice thickness.

※ PD is defined when the reduction ratio meets the condition of PR and the concomitant increase ratio meets the condition of PD.

※ When one lesion is separated during treatment, each diameter is added to the sum of diameters.

※ If more than one lesion fuses and the boundary cannot be distinguished during treatment, the diameter of the fused lesion is added to the sum of the diameters. The diameter of each lesion is added to the sum of diameters when the boundary of the lesion is identifiable, even if the lesion is in contact with each other.

11.1.7. Response Evaluation Criteria for Non-Target Lesions

・CR(Complete Response): Complete response

When all non-lymph node non-target lesions disappear, the short diameter of all nodal non-target lesions becomes less than 10 mm, and all tumour markers (NSEs, ProGRP※) are below the upper shared reference limits. When there are no (on the image) non-target lesions at baseline assessment and all tumor markers are below the shared reference range upper limit.

Because ※ProGRP is not included in the shared baseline range, the baseline range is 6.5-46.0 pg/mL.

・Non-CR/non-PD: non-CR/non-PD

Residual one or more non-target lesions (including residual nodal non-target lesions ≧10 mm in short diameter) and/or tumour markers (NSEs, ProGRP※) exceeding the shared upper reference limits. No non-target lesions (on images) at baseline assessment and any of the tumor markers exceeding the shared reference range upper limit.

・PD(Progressive Disease): Progress

'Apparent exacerbation' (including relapse) of pre-existing non-target lesions.

For measurable disease: A marked progression of a non-target lesion that deserves discontinuation of treatment as an increase in overall tumor burden must be observed if the effect of the target lesion is SD or PR but is judged to be "clear progression" based on the change in the non-target lesion. If the effect of the target lesion is SD or PR, then an increase in the tumor burden of the non-target lesion to a degree

that far exceeds the decrease in tumor burden is considered "obvious progression" and otherwise Non-CR/non-PD.

If only unmeasurable disease is present, the increase in non-target disease, as judged to clearly exceed the tumor burden corresponding to a 20% increase in diameter and a 73% increase in tumor volume, is considered "definite progression". Elevated tumor markers alone are not considered PD.

・NE(Not all Evaluated): Lack of study

If the test could not be done for any reason or if neither CR, Non-CR/non-PD nor PD could be determined.

11.1.8. Presence or absence of new lesions

If a lesion that was not present at baseline was observed after the start of treatment, it is considered "new lesion" to be present.

However, a "new lesion" requires that it is not an imaging change due to a difference in the imaging method from the baseline assessment or a change in the imaging modality, nor is it an imaging change due to a condition other than the tumor. For example, a cystic lesion arising within a lesion due to necrosis of a liver metastatic lesion is not a new lesion. New lesions will be defined as new lesions by examination of sites that were not mandatory at baseline (pre-enrollment study).

If a lesion disappears and later reappears, measurement is continued. However, the effect at the time the lesion reappears depends on the status of the other lesion. When the overall effect reappears after CR, the lesion is judged as PD at the time of reappearance. When the overall effect is PR or SD, on the other hand, once the disappeared lesion reappears, the diameter of the lesion will be added to the sum of the diameters of the remaining lesions to calculate the effect. That is, in the presence of many residual lesions, even if one lesion reappears after an apparent disappearance, it is not judged as PD by itself, and it is judged as PD when the sum of the diameters of all lesions meets the criteria for PD. This is because of the perception that the majority of lesions do not truly 'disappear' and are not only depicted by the limits of resolution of the imaging modalities used.

If there is a possibility of a new lesion but it cannot be determined, it should not be a new lesion, and imaging should be reexamined at a clinically relevant time. If a new lesion is confirmed by repeat imaging, the new lesion will appear based on the date of imaging at which the new lesion is confirmed.

11.1.9. Overall efficacy (Overall Response)

The overall response (Overall response) will be determined by combining the effects of target lesions, non-target lesions, and the presence or absence of new lesions every 6 weeks according to Table 11.1.9.a below. The overall effect in the absence of a non-target lesion at baseline will be determined by the effect of the target lesion and the presence or absence of a new lesion, and the overall effect in the absence of a target lesion at baseline will be determined according to the effect of a non-target lesion and the presence or absence of a new lesion according to Table 11.1.9.b.

Table 11.1.9.a. Overall efficacy at each time point for target lesions (with or without non-target lesions)

Target lesion	Nontarget lesions	New lesions	Overall effect
CR	CR	None	CR
CR	Non-CR/non-PD	None	PR
CR	Lack of study	None	PR
PR	Lack of Non-PD or study	None	PR
SD	Lack of Non-PD or study	None	SD
Lack of study	Non-PD	None	NE
PD (obvious progression)	Irrespective of	With or without	PD
Irrespective of	PD	With or without	PD
Irrespective of	Irrespective of	Present	PD

Table 11.1.9.b. Overall efficacy at each time point for patients with non-target lesions only

Nontarget lesions	New lesions	Overall effect
CR	None	CR

Non-CR/non-PD	None	Non-CR/non-PD
Lack of study	None	NE
PD (obvious progression)	With or without	PD
Irrespective of	Present	PD

11.1.10. Best overall effectiveness (Best Overall Response)

CR > PR > SD > PD > NE is considered good, and the best overall effect is the best overall effect throughout the entire course.

PD is defined when imaging cannot be determined due to exacerbation of obvious disease or death before the first response assessment. In addition, NE is defined if it cannot be determined by imaging due to discontinuation of toxicity before the first response assessment or patient refusal.

11.2. Definitions of analyses set

The analysis sets used in periodic central monitoring, interim analysis, and final analysis are defined as follows: The flow diagram below shows the analysis sets.

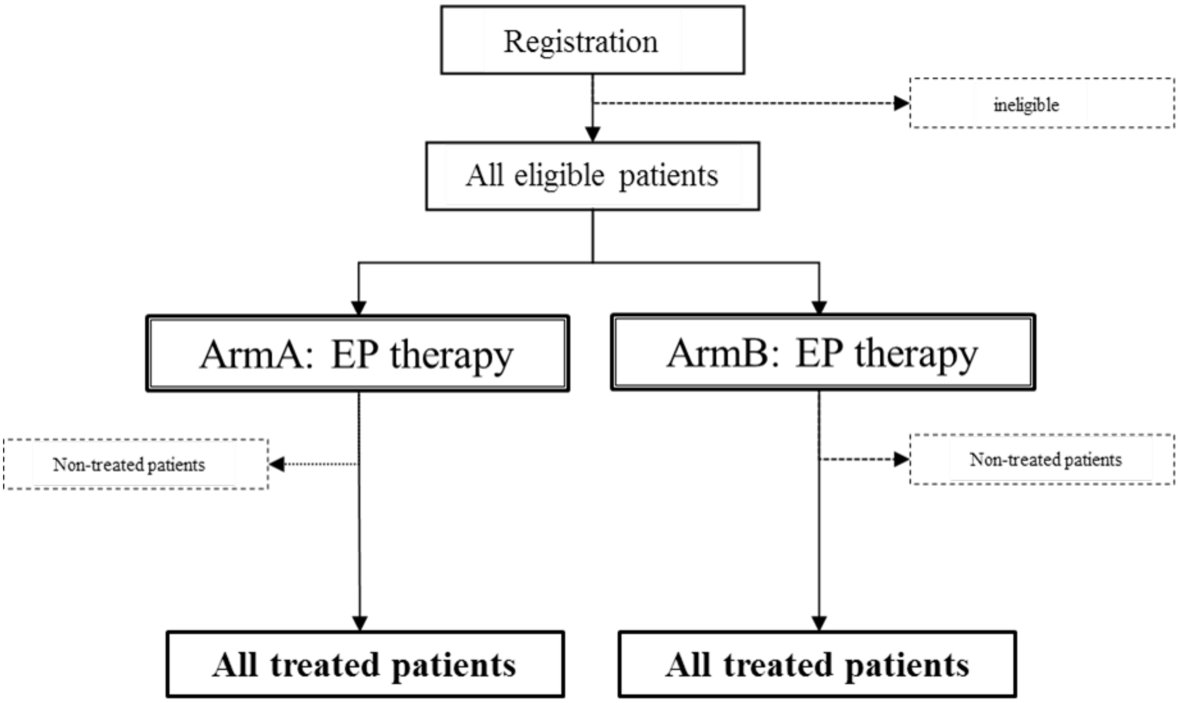

11.2.1. All registered patients

「5.1. Among the patients enrolled according to the Procedures for Enrollment, the population excluding duplicate or mis-enrollment is considered as "all enrolled cases".

11.2.2. All eligible patients

The group excluding "ineligible cases (post hoc ineligibles, de facto ineligibles, violation of registration)" determined by group review from all registered patients is regarded as all eligible patients. Ineligible cases as judged by the investigator or sub-investigator alone are included in all eligible patients. Only those judged not eligible by the central pathological diagnosis are ineligible and not included in all eligible patients.

11.2.3. All treated patients

Of all enrolled patients, all patients for whom part or all of the protocol treatment was performed will be defined as all treated patients.

The decision to treat "non-treated patients" for whom no protocol treatment has been given and whether it is excluded from the safety analysis can be determined by the data center with the consent of the Research Office. Ineligible patients will be excluded from all treated patients. However, if there are circumstances in which ineligible patients are included in the analysis, the nature of the ineligibility will be examined and determined by the Research Secretariat in consultation with JCOG Data Centre.

11.2.4. Patients eligible for central pathology diagnosis

Of all eligible patients, the population excluding ineligible patients with a central pathological diagnosis will be considered as eligible patients with a central pathological diagnosis.

11.3. Definition of endpoints

Endpoint	Events (whichever is earlier)		Censoring date
Overall survival time<br>Overall survival(OS)	All deaths	-	Date of final survival confirmation
Progression-free survival time<br>Progression-free survival(PFS)	All deaths	Progression/recurrence	Final date of clinically confirmed freedom from exacerbation

11.3.1. Overall survival

The duration from the date of registration to the date of death from any cause.

- Survivors are censored at the date of final survival confirmation (survival confirmation by telephone contact is also permitted, but the fact that survival confirmation was performed should be recorded in the medical record).
- Patients lost to follow-up are censored at the last date of survival confirmation before lost to follow-up.

11.3.2. Progression-free survival (PFS: Progression-free survival).

The duration from the date of registration to the date of the judgement of exacerbation or death from any cause, whichever comes first.

- The exacerbation (progression) includes both imaging-based PD (progression) and exacerbation of pathogenic disease (clinical exacerbation) that cannot be confirmed by imaging studies in Section 11.1.9. Global Effectiveness. If an exacerbation is judged based on diagnostic imaging, the exacerbation date is the test date on which the imaging was performed, and in the case of clinical exacerbation, the date of clinical judgment is the exacerbation date. PD may occur in accordance with the Response Evaluation Criteria, even if the tumor diameter is very small, but the disease is clinically judged to be "not obviously aggravated" in accordance with the Response Evaluation Criteria Criteria (in this case, clinical judgment should be prioritized for continued treatment with the protocol). In addition, even if PD is not observed according to the response evaluation criteria, the clinical judgment is prioritized for progression if it is judged to be clinically obvious exacerbation.
- In survivors who are not judged to have progression, clinically confirmed progression is censored (date of final progression-free survival confirmation) (confirmation of progression-free by imaging or specimen examination is not mandatory and clinical progression-free by outpatient examination etc. is not permitted. Telephone contact alone is not permitted. If information on progression or progression-free is obtained at a medical institution or referral center, receive and retain a medical information form describing the rationale for diagnosis. In this case, telephone contact alone is not permitted).
- Events and censoring are treated similarly, if chemotherapy is terminated for reasons such as toxicity or patient refusal, and if other therapies are added as post-study treatment. i.e., it is not censored at the time of treatment termination or at the date of initiation of post-study treatment.
- When the diagnosis of exacerbation is based on imaging, the relapse is not regarded as an event at the test date of imaging with "suspicious diagnosis of relapse", but regarded as an event at the later test date of imaging with "definite diagnosis of relapse"" If the event is judged to be clinically aggravated based on diagnostic imaging, the event is considered to be the day of the progression.
- If the definitive diagnosis of relapse or new lesion is based on biopsy pathology, the date of clinical diagnosis is defined as the date of clinical diagnosis when the diagnosis of recurrence or new lesion is made clinically, and the date of biopsy is defined as the date of event when the diagnosis of recurrence is made based on biopsy pathology diagnosis without clinical diagnosis of recurrence.
- The incidence of a second cancer (metachronous double cancer) is not censored or event, and progression-free survival is defined until other events are observed.

11.3.3. Response rate (response rate) Response proportion (Response rate)

Among all eligible patients with measurable disease, the response rate is defined as the proportion of patients with "11.1.10. Best Global Effect" that is either CR or PR.

11.3.4. Incidence of adverse events (adverse reactions)

Using all treated patients as the denominator, the frequency of the worst Grade during the entire course by CTCAE v4 0 Japanese translation JCOG version for each of the following adverse events (toxicities) will be determined by group:

- Laboratory tests: hemoglobin decreased, white blood cell decreased, neutrophil count decreased, platelet count decreased,

  Increased blood bilirubin, aspartate aminotransferase increased (AST or GOT),
  Alanine aminotransferase increased (ALT or GPT), creatinine increased,
  Hypernatremia, hyponatremia, hyperkalemia, hypokalemia, hypercalcemia, hypocalcemia,

hypermagnesemia, hypomagnesemia

- General disorders and administration site conditions: fever, fatigue
- Ear and labyrinth disorders: Tinnitus, hearing impairment
- Skin and subcutaneous tissue disorders: alopecia
- Gastrointestinal disorders: constipation, diarrhea, nausea, vomiting, oral mucositis
- Metabolism and nutrition disorders: anorexia, dehydration
- Nervous system disorders: dysgeusia, peripheral sensory neuropathy, peripheral motor neuropathy
- Musculoskeletal and connective tissue disorders: myalgia, arthralgia
- Infections and parasites: bronchial infection, pulmonary infection, upper respiratory tract infection, catheter-related infection, biliary tract infection,
  Gallbladder infection; Mediastinal infection; Pleural infection; Urinary tract infection
- Blood and lymphatic system disorders: Febrile Neutropenia
- Musculoskeletal and connective tissue disorders: arthralgia, Vascular disorders: phlebitis
- Respiratory, thoracic and mediastinal disorders: dyspnea, hypoxia, pneumonitis

In the other adverse event (toxicitiy) than the above, the proportion of occurrence are not calculated unless a large number of specific adverse events are observed, since only Grade 3 or more non-hematological toxicity ※ is reported in the Treatment Form.

※※ Non-hematological toxicity refers to adverse events other than those listed below in CTCAE v4.0-JCJCOG.
Anemia, decreased bone marrow cells, decreased lymphocyte count, decreased neutrophil count, decreased white blood cell count, and decreased platelet count.
CD4 lymphocytopenia

11.3.5. Dose intensity of cisplatin

Dose intensity of cisplatin per patient (DOOC.) will be calculated for all treated patients to assess treatment compliance with cisplatin. The summary statistics (minimum, 25% point, median, 75% point, maximum, mean, and standard deviation) will be calculated for each group.

- ・Active dose D.I. (mg/m2/week) = total drug dosebody surface areatreatment duration (weeks)
- Body surface area: BSA is calculated by height at enrollment and body weight calculated by Data Center.
- ・ Treatment period (weeks)
  Arm A = (start date of last course-start date of 1st course + 21)/7
  Arm B = (start date of last course-start date of 1st course + 28)/ 7

11.3.6. Incidence of serious adverse events (adverse reactions)

1) Grade 4 non-hematologic toxicities, early deaths, and treatment

Using all treatments as denominators, the percentage of patients with one or more Grade 4 non-hematologic toxic ※ that are considered to be related (either to definite, probable, possible) to the protocol treatment among the adverse events listed in the free text of the CRFs in addition to the stereotyped items in Section 11.3.4 is used as the numerator.

※※ Non-hematological toxicity refers to adverse events other than those listed below in CTCAE v4.0-JCJCOG.
Anemia, decreased bone marrow cells, decreased lymphocyte count, decreased neutrophil count, decreased white blood cell count, and decreased platelet count.
CD4 lymphocytopenia

2) Early mortality rate

Proportion of all deaths during the protocol treatment or within 30 days from the last protocol treatment day among all treated patients. Causes of death irrespective of causality with protocol treatment. However, if premature death occurs in patients excluded from all treated patients, the details are provided separately.

3) Proportion of treatment-related death (TRD incidence)

Proportion of all deaths judged as causally related (either definite, probable, possible) to the protocol treatment among all treated patients However, if TRD occurs in patients who have been removed from all treated patients, the details are presented separately.

12. Statistical consideration

Methods for statistical analysis are as follows: In addition, the details required for conducting specific analyses are specified in the statistical analysis plan prepared separately prior to the analysis, and in documents that clarify the endpoint definition, etc. If substantial changes occur in statistical analyses as described below, follow the policy set out in "13.6. Protocol Changes." Facilities are contacted by "14.1. Periodic Monitoring" for missing or abnormal results, and data collection or exclusion is determined by review by the Research Secretariat based on the results of inquiries to the institution in accordance with the policies stipulated in "14.1.2. eligible (eligibility/ineligibility)" and "14.1.3. Protocol Deviations/Violations". 「11. Handling of missing values and abnormal data that cannot be addressed by the definition and analysis method for each endpoint, as specified in "Definition of Response Evaluation and Endpoints" and "12. Statistical Items" below, is specified in the above statistical analysis plan.

12.1. Principal Analysis and Decision Criteria

The primary analysis of this study will be the final analysis.

The purpose analysis of this trial is to test whether one of the two community standard-of-care arm A (EP-therapy) and B (IP-therapy) outperforms primary endpoint overall survival. The null hypothesis of equal overall survival in the two groups in the main analysis will be tested by stratified log-rank test stratified by non-institutional allocation adjustment factors (primary organ [gastrointestinal vs. hepatobiliary-pancreatic]) in all patient patients. However, if it is assumed that a stratified log-rank test cannot be performed appropriately, such as when the number of subjects and events in each stratum is small, the allocation adjustment factors will be addressed in the analysis plan prepared without information related to the comparison between groups before performing a confirmatory analysis with comparison between groups. Sensitivity analysis will also be performed in all eligible patients and in patients eligible for central pathology diagnosis.

Due to interest in which treatment group is superior, the test will be two-tailed. The study-wise significance level for the study is set at both sides 10%. In the main analysis, two-sided 90% confidence intervals corresponding to a two-sided 10% significance level will be calculated, and in other analyses, two-sided 95% confidence intervals will be calculated for descriptive purposes.

We conclude that EP therapy is a more useful treatment when the two-sided p-value is less than 10% and the survival curve of EP therapy exceeds that of IP therapy. Meanwhile, we conclude that IP therapy is a more useful treatment when the two-sided p-value is less than 10% and the survival curve of IP therapy exceeds that of EP therapy. If the difference was not significant at a two-sided significance level of 10%, one of the two modalities cannot be judged to be useful. In this case, we conclude that both modalities continue to be the standard of care, as there is no evidence to actively recommend either one of them.

However, if there are major differences in the toxicity profiles of EP and IP therapies, contrary to prior assumptions, during the course of the study, and if the toxic therapy is not superior to other therapies, it is judged that it will not be of significance to be used in clinical practice, the following measures should be taken. In other words, the clinical hypothesis will be changed from two-sided to one-sided in an analysis plan with no information on group comparisons before a confirmatory analysis with group comparisons will be conducted, and the superiority of the toxicity treatment over other treatment modalities will be verified at a one-sided significance level of 5% (in this case, the policy of change for the interim analysis will be described in Section 12.3.2).

Estimates such as cumulative survival curves, median survival times, and annual survival rates are performed using Kaplan-Meier method. Brookmeyer and Crowley methods are used to obtain 95% confidence intervals for median survival. Greenwood's formula is used to obtain 95% confidence intervals for annual survival rates. Hazard ratios and their confidence intervals for treatment effects between groups are obtained using stratified Cox proportional hazards models with the same factors as the test of the primary analysis as estimates of treatment effects. Cox regression adjusted by imbalance background factors in addition to adjustment factors will be performed as needed.

The main analysis results will be summarized as the "Main Analysis Report" by the Data Center one year after the completion of enrollment and submitted to the Research Secretariat, Research Representatives, Group Representatives, Group Secretariats, Efficacy and Safety Assessment Committee, and JCOG Representatives.

The principal investigator/study coordinator summarizes the content of the main analysis report, prepares a

"Clinical Study Report" summarizing the conclusions, problems, interpretations and discussion of the results, and future policies of the entire study, and submits it to the Data and Safety Monitoring Committee and JCOG chair with approval from the Group chair and the Head of JCOG Data Center.

Approval of the clinical study report by the Data and Safety Monitoring Committee shall be considered as "end of the study."

12.2. Planned accrual, accrual period, and follow-up periods

「2.4.2. Based on the background presented in Clinical Hypothesis and Rationale for Number of Enrollment, we assume a median survival of 8 and 12 months (HR=0.67) for the inferior and superior treatment groups, respectively, among the two treatment groups. When a superiority trial design is used, 63 patients per group and 126 patients in both groups (114 events required) will be included in the required analysis using Schoenfeld & Richter methodological [50] at 6 years of enrollment, 1 year of follow-up, $\alpha = 10\%$ (two-sided), and 70% power. The number of required Inferior treatment (number of required events) when the median survival time in A is different from the assumption as shown in Table 12.2.1 below.

Table 12.2.1. Number of Analyses Required (Number of Events Required)

	Power			
Median Survival (mo)	65%	70%	75%	80%
7.0 vs. 10.5	108(100)	124(114)	142(131)	162(150)
8.0 vs. 12.0	112(100)	126(114)	144(131)	166(150)
9.0 vs. 13.5	114(100)	130(114)	148(131)	170(150)

※※ Number of required events in parentheses

Based on these findings, the following will be established in view of some cases of loss to follow-up.

Planned enrollment: 70 patients in each group and 140 patients in both groups

Enrollment period: 6 years, follow-up period: 1 year after completion of enrollment

Consider redesigning the sample size if the prognosis is obviously better than assumed, or if it reaches 70 people within 2.5 years of enrollment initiation. Clinically meaningful differences will then be reviewed and redesigned in a blinded fashion prior to the conduct of the analysis.

<Additions in ver. 1.1>

The registration pace after the enrollment start was smooth and exceeded the plan, and 70 cases were reached in October, 2016, when 2 years and 2 months passed from the enrollment start. Since it exceeded the standard of 70 patients within 2.5 years from the initiation of enrollment specified above, acceptance was obtained at the meeting of the Hepatobiliary and Pancreatic Group on October 29, 2016, the group meeting of the Esophageal Cancer Group on November 19, 2016, and the group meeting of the Gastric Cancer Group on January 7, 2017 with respect to changing the power to 80% according to the rules at the time of the study plan. Therefore, the planned enrollment number was changed to 170. In addition, the "1-year analysis period" was added to the study period, and the following changes were made.

Planned enrollment: 85 patients in each group and 170 patients in both groups

Enrollment period: 6 years; Follow-up period: 1 year after completion of enrollment; Analysis period: 1 year; Total study period: 8 years

12.3. Interim Analysis and Early Termination of the Study

12.3.1. Purpose and Timing of the Interim Analysis

Once interim analyses will be conducted to determine if the primary objective of the study has been achieved during the study period. Interim analyses will be conducted to determine if it is reasonable to continue enrollment during enrollment. If the primary objective of the study is determined to be achieved, the study will be discontinued and the study results will be published promptly at the conference and in the article.

Interim analyses will be conducted using data from the initial periodic monitoring that will be queried after the time enrollment of half of the planned enrollment was obtained. Based on the information in the periodic monitoring report, the group will submit the presence or absence of changes in clinical hypotheses and, if any, details of changes to the Efficacy and Safety Assessment Committee by the time of the interim analysis.

During the first interim, patient accrual is continued in principle. If the progress of the study progressed as planned, the expected number of events at the time of the interim analysis under the assumptions presented in 12.2 is expected to be 44 if the interim analysis is conducted at 3 years after the start of enrollment.

12.3.2. Method of interim analysis

Interim analyses will be conducted by the JCOG Data Center. To keep the study-wise alpha errors at 10%, the multiplicity of the interim and final analyses is adjusted using Lan & DeMets's alpha spending functions, and statistical significance is examined for differences in primary endpoint between arms. As α spending functions, we use O'Brien & Fleming types.[51]

For details of the interim analysis, the statistical staff in charge of the group at the Data Center will prepare the statistical analysis plan by the time of the interim analysis. Actual interim analyses will be performed by statistical staff who are not in charge of the group and an interim analysis report will be prepared.

In the interim analysis, if the survival of one treatment group exceeds that of the period, and the p-value of the stratified log-rank test falls below the level specified by the above method, it is judged statistically significant and the trial is discontinued as a rule. The ineffective discontinuation is not planned at a stage where no statistically significant differences are observed unless prior assumptions regarding toxicity are changed. However, the clinical hypothesis may be changed for the reasons described in 12.1, and the primary analysis may be performed by one-sided rather than two-sided testing, which may result in deviations from prior assumptions. In such cases, the primary hypothesis change should be specified in the analysis plan to be prepared without information on group comparisons before a confirmatory analysis with group comparisons. If the overall survival curve in the highly toxic treatment group is below the other, the consideration of ineffective discontinuation should not be judged by a test and considered comprehensively.

12.3.3. Interim analysis Reporting and review of the results

The results of the interim analysis will be submitted to the Data and Safety Monitoring Committee by the Data Center as an Interim Analysis Report and reviewed for the acceptability of continuation of the study and for publication of the results. The Data and Safety Monitoring Committee considers whether to continue the study at the meeting and recommends whether to continue the study and whether to publish the results to principal physician or group chair based on the results of the review.

Members of the Data and Safety Monitoring Committee of the relevant group are not included in the review. Unless the results of the interim analysis make recommendations for discontinuation of the study from the Efficacy and Safety Assessment Committee, the research representative, research office, participating institution researchers, group representatives, and group secretaries of the study will not be able to know the results of the interim analysis until the final follow-up is completed.

When the Interim Analysis Report has been reviewed by the Data and Safety Monitoring Committee to recommend termination or change of all or part of the study, the principle investigator and group chair review the recommendations and decide whether to discontinue or change some of the study.

If the study is discontinued or part of the study is changed, the principal investigator and group chair shall submit in written form a request to the Data and Safety Monitoring Committee for permission to discontinue the study or a request to revise the protocol. Following approval by the Data and Safety Monitoring Committee, the principal investigator may discontinue the study or change part of the study.

The Study Chair and Group Chair can disagree with the recommendations of the Data and Safety Monitoring Committee, but if they fail to coordinate their opinions with the Data and Safety Monitoring Committee, they will ultimately follow the instructions of JCOG Chair.

If the study is terminated, the subsequent follow-up period will be 1 years from last registration per study.

If the interim analysis resulted in study termination, the interim analysis will be the primary analysis of the study. The Data Center, in cooperation with the Research Representative Physicians and Research Secretariat, will conduct the analysis required to complement the incomplete data and publish the results, focusing on the results of the interim analysis, and promptly prepare the Major Analysis Report and submit it to the Group and the Efficacy and Safety Assessment Committee.

12.4. Analysis of Secondary endpoints

Secondary endpoints analyses will be conducted to provide a supplementary discussion of the primary analysis results of the study. Because the analysis of secondary endpoint is exploratory, no multiplicity adjustments are made. Comparisons between arms are made where appropriate, note that when the results of the group comparisons are not significant, they do not mean that there is no difference between the two arms.

12.4.1. Analysis of safety secondary endpoints

Among Secondary endpoints, the safety endpoints are the incidence of adverse events and the incidence of serious adverse events, which are in principle the items of periodic monitoring (14.1. Periodic monitoring).

The incidence rate of adverse events will be summarized as well as the incidence rate of Grade3 or higher. For adverse events other than laboratory data, the incidence of Grade 2 or higher is also calculated.. Non-hematological toxicity incidence, early mortality, and treatment-related mortality rates of Grade4, which are serious adverse events, are reported in periodic monitoring reports with registration numbers and details. The rates of non-hematologic toxicity, early mortality, and treatment-related mortality for Grade4 will be calculated at the time of the interim analysis and the main analysis. When interval estimation of proportions is performed, accurate confidence intervals based on binomial distributions are used. Comparisons between arms will be made using Fisher's exact test where appropriate.

12.4.2. Analysis of efficacy secondary endpoints

Among Secondary endpoints, efficacy endpoints are response rate, progression-free survival, which will only be analyzed in the interim and primary analyses.

Secondary endpoints analyses do not adjust for multiplicity.

Progression-free survival will be included in all enrolled patients, but a comparison of all eligible patients, excluding ineligible patients, as determined by group study, will also be performed as a sensitivity analysis.

The response rate will include all eligible patients with measurable disease, but a comparison of all enrolled patients will also be performed as a sensitivity analysis.

Fisher's exact test will be used to compare response rates between groups, and binomial distribution-based exact confidence intervals will be used for interval estimation. Estimates, including progression-free survival curves, median progression-free survival, and time-point progression-free survival, will be performed using Kaplan-Meier method, Brookmeyer and Crowley methods will be used to obtain confidence intervals for median progression-free survival, and Greenwood formulas will be used to obtain confidence intervals for progression-free survival. Log-rank test is used for comparison between arms. Hazard ratios and their 95% confidence intervals for treatment effects between arms will be calculated using Cox's proportional hazards model as an estimate of treatment effect. Cox regression adjusted by imbalance background factors in addition to adjustment factors will be performed as needed.

12.5. Final analysis

The primary analysis will be the final analysis unless this study is withdrawn from the interim analysis.

If the interim analysis is withdrawn from the study, then after the end of the 1-year follow-up period, the final analysis will be performed after the final survey confirms the data and then analyses will be performed for all endpoints.

Except for the interim analyses and the final analysis, analyses with between-arms comparisons for the primary and secondary endpoints for efficacy are not performed unless approved by the Data and Safety Monitoring Committee.

If the final analysis is performed after the main analysis, the final analysis will be summarized by the Data Center as the "Final Analysis Report" and submitted to the Research Secretariat, Research Representatives, Group Representatives, Group Secretariats, Efficacy and Safety Assessment Committee, and JCOG Representatives.

The Study Representative Physician/Research Secretariat summarizes the content of the final analysis report, prepares the "Clinical Study Report" summarizing the conclusions, issues, interpretations and discussion of the results, future policies, etc. mainly from the clinical point of view (if the "Clinical Study Report" is prepared in the previous analysis report, it will be the "Clinical Study Report (Supplementary Version)) with additional updates), and submits it to the Study Representatives and JCOG Representatives with approval from the Group Representatives and the Head of JCOG Data Center.

Approval of the clinical study report by the Data and Safety Monitoring Committee shall be considered as "end of the study."

12.6. Exploratory analysis

To investigate the interaction between treatment effect and the subpopulation, subgroup analyses will be conducted exploratory with respect to the following factors: Because these analyses are not adequately powered and do not adjust for multiplicity, the results of each subgroup analysis should be interpreted as exploratory.

- PS0/1
- Age 65 years or older/<
- Gender (male/female)
- Primary organ (gastrointestinal tract/hepatobiliary pancreas)
- Organ of origin (esophagus/stomach/small intestine/large intestine/pancreas/biliary tract/liver NEC (liver primary or liver metastasis of unknown primary)
- Organ of origin (pancreas/non-pancreas)
- Extent of extension of the primary lesion (locally advanced/distant metastasis or recurrence)
- Extent of extension of the primary lesion (locally advanced/distant metastasis/recurrence)
- Prior radical resection of the primary lesion (none/present)
- Pathological diagnosis was biopsy/resection specimen
- Grade 3 tumour with morphologically similar features of NETs but Grade 3 proliferative activity/morphologically more atypical (previously classified as poorly differentiated endocrine carcinoma) on histopathology with central pathology
- Histopathological examination by central pathological diagnosis, including Small cell carcinoma/Large cell carcinoma /
- Histopathologically diagnosed by central pathology, Ki67 50% or higher/less than 50%

12.7. Premature withdrawal from the trial

In this study, early termination of the study may occur in the following cases:

1) Early termination due to discontinuation of interim analysis
2) Early termination due to adverse events
3) Early termination due to poor enrollment
4) Early termination due to other reasons

12.7.1. Early termination by interim analysis

In this study, based on the criteria described in 12.3, early termination recommendations may be made at the interim analysis review by the Efficacy and Safety Assessment Committee. If the Data and Safety Monitoring Committee provides recommendations for early termination of the study, the principle investigator and group chair will review the recommendations and decide whether to terminate the study early.

12.7.2. Early termination due to adverse events

In JCOG9511 for small-cell lung cancer, 1/77 (1.3%) treatment-related deaths were reported with EP therapy and 3/77 (3.9%) with IP therapy; in JCOG0509, a successor study for the same subject, 1/142 (0.7%) treatment-related deaths were reported with IP therapy and 2/142 (1.4%) with amrubicin plus cisplatin therapy. Using these as reference, we believe that the treatment-related mortality rate should not exceed 3% in this study. Since it is clear that the final point estimate will be at least 3% at the time of 3 treatment-related deaths in either group, immediate entry should be suspended to consider whether or not to withdraw from the study. At this point, the subsequent treatment of the patient being treated will be reviewed. At the time of 3 or fewer treatment-related deaths in each group, each patient will be reported to the Efficacy and Safety Assessment Committee for adjudication. Enrollment will be continued until the results are obtained in principle.

12.7.3. Early termination due to poor enrollment

If the patient enrollment pace is significantly worse than at the time of planning, early termination of the study may be advised by the Data and Safety Monitoring Committee. If early termination recommendations are issued by

the Data and Safety Monitoring Committee due to poor enrollment, the principle investigator and group chair will review the recommendations and decide whether to terminate the study early.

12.7.4. Early termination due to other reasons

12.7.1.～12.7.3. If it is judged difficult to continue the study for other reasons, the research representative physician shall submit a request for early termination of the study to the Efficacy and Safety Assessment Committee. If the Data and Safety Monitoring Committee recommends early termination of the study based on the submitted data, the procedure for early termination of the study will be progressed.

12.8. Procedures after Early termination of the Study

If the Study Chair accepts the recommendations made by the Data and Safety Monitoring Committee based on Section 12.7, he/she will promptly submit a notification to the Data and Safety Monitoring Committee that early termination of the study will be performed.

The Study Chair will submit a termination notification to the Certified Review Board within 10 days of the date they decide to terminate the study early. If the study falls into a specified clinical trials under the Clinical Trials Act, the Study Chair shall submit a termination notification to the Certified Review Board within 10 days of the date on which the study was decided to be prematurely discontinued, as well as submit a specified clinical trials termination notification to the MHLW.

The Study Chair promptly informs the investigator of the decision to terminate the study early in writing, and the investigator who has received a report of early termination of the study will report in writing that the study was prematurely terminated to the institution's manager without delay.

If the study is terminated early, JCOG Data Center will promptly initiate the development of the primary analysis report or final analysis report. The subsequent follow-up period will be 1 year from the final enrollment.

13. Ethical Considerations

13.1. Protection of Human Subjects

All researchers involved in this study will conduct this trial in accordance with the "Helsinki Declaration" 1) and "Clinical Trials Act" (2017 Law No. 16) 2) "Clinical Trials Act Enforcement Regulations" (2018 Ministry of Health, Labour and Welfare Order No. 17) and related notices.

1） http://dl.med.or.jp/dl-med/wma/helsinki2013j.pdf

2） http://www.mhlw.go.jp/stf/seisakunitsuite/bunya/0000163417.html

Prior to commencing this study, the site investigator is required to obtain input from Certified Review Board※1 regarding the conduct of the study, and to obtain approval from the Administrator of the participating institution, and submit the Implementation Plan ※2 to the Ministry of Health, Labour and Welfare.

※ 1 JCOG study will be submitted to the following Certified Review Board.
National Cancer Center Hospital Certified Review Board (accreditation number CRB3180008)

※ 2 "Implementation Plan" means "Documented plan formatted according to the Form No.1 (Form No.1 of the Ministerial Ordinance) specified in Article 39 of the Clinical Trials Act Enforcement Regulations"

13.2. Informed consent

13.2.1. Explanation to the patient

Prior to patient registration, the investigator or subinvestigator will provide the patient with written informed consent form approved by Certified Review Board and explain the following details verbally.

Descriptions

1） Disease names, Stages, and expected prognosis

2） That this study is a clinical trial and is conducted by JCOG
Name of Certified Review Board and contact information for receiving complaints and inquiries to the committee

3） Design and rationale of the study

4） Protocol treatment content

5） Effects expected by protocol treatment

6） Expected adverse events, complications, and sequelae and how to deal with them
Explanation of the extent and frequency of expected adverse events, including complications, sequelae, and treatment-related deaths, and how to deal with them when they occur. In addition to these explanation, obtain the most recent version of the drug package insert and deliver it to patients (PMDA Prescription Pharmaceutical Information Search http ://www.pmda.go. jp/PmdaSearch/iyakuSearch/)

7） Post-study treatment after end of protocol treatment should also be performed appropriately

8） Cost burden and compensations
Explanation of the cost of treatment, compensation that can be received in the event of a health hazard (equivalent to measures taken in general practice, etc.)

9） Alternative treatment
Explanation of treatments that can be received if not participating in this study

10） Anticipated benefits and possible disadvantages
Explanation of anticipated benefits and possible disadvantages by participating in this study

11） Direct access to the medical records
Explanations on acceptance of site visit audits, such as "direct access to medical records etc. by healthcare professionals at other medical institutions for quality control with permission from the administrator of the participating institution."

12） Refusal of consent and withdrawal of consent
Refusal to consent prior to participation in the study is free, and withdrawal after having given consent is free, thereby not causing undue medical disadvantage.

13） Protecting human rights
Every effort should be made to ensure that personal information, such as names, is kept confidential.

14） Secondary use of data

The possibility of secondary use of data obtained from this study in Japan and overseas (ancillary studies, meta-analyses, etc.) only when approved by either committee in JCOG

15) Method of disclosure of information on the study
The study is registered and published in jRCT※. In addition, the results of clinical studies should also be published in jRCT (＊ Databases (Japan Registry of Clinical Trials) https://jrct.niph.go.jp/ prepared by the MHLW as stipulated in Paragraph 1 in Article 24 of Clinical Trials Act Enforcement Regulations)

16) Freedom of questions
Explanation that investigators, written contact information for consultations on study details, on the Principal Investigator and the Study Coordinator, and freely asking questions about study and treatment

17) Explanation of the use and burden of medicinal products not covered by insurance

18) Central pathological review

19) Central image review for response

20) Sample collection for ancillary studies

13.2.2. Consent

Explain the study, give sufficient time to think, confirm that the patient understood the study well, and ask for participation in the study. If the patient agrees to participate in the study, the written informed consent form in the appendix will be used to obtain the patient's own signature. The site investigator or the subinvestigator confirms that the study consent form contains the name of the physician who provided the explanation and the date of explanation, the name of the patient who gave informed consent, and the date of informed consent.

In addition, when it is not possible to read the documents due to visual impairment, etc., but the details can be understood by verbal explanation, or the documents can not be signed due to limb disorders, etc., but the documents can be read and understood, signatures may be obtained from the proxy author under the consent by the patient. However, the signature of the proxy author should be based on this study's consent, and should be described as "Signing by the proxy author" and "Relationship with the patient" so that the person can be found to be the proxy author.

Two copies of the consent form will be provided, one will be handed over to the patient, and one will be stored by the site coordinator. Original copies will be stored in the medical records or in the archives specified by the participating institution.

13.2.3. Response to inquiries, consultations, etc. after consent

In principle, the investigator or subinvestigator of the relevant patient's participating institution responds to any consultation related to the study by the patient or his/her family after registration. If it is unclear how to respond, respond in consultation with Principal Investigator, Study Coordinator, the Group Secretariat, Group Chair, JCOG Data Center/Operations Office, etc. in accordance with the content of the consultation.

13.2.4. Withdrawal of consent

After obtaining informed consent for participation in the study, consent will be withdrawn if the patient expressed withdrawal of consent.

Withdrawal of consent refers to withdrawal of consent to participate in research and is distinguished from refusal to continue protocol treatment (below (i)). If the withdrawal of consent is expressed, clarify whether (ii) or (iii) below and promptly notify JCOG Data Center. If consent is withdrawn, record it in the medical record as well as (ii) or (iii).

Data Center discontinues subsequent follow-up requests according to the protocol in case of (ii) withdrawal of consent, In the case of (iii) full withdrawal of consent, the data of the patient are removed from the database when it is confirmed that the patient has full withdrawn of consent.

The procedures for discontinuation of the patient's follow-up request and removal of patient data will be specified separately in the procedural manual, and the completion of each task will be reported to Principal Investigator and Study Coordinator.

(i) Patient refusal: Refusal to continue subsequent protocol treatment (follow-up continues).

(ii) Withdrawal of consent: Withdrawal of consent to participate in the study and termination of all subsequent treatment and follow-up in accordance with the study protocol. Research use of data prior to withdrawal of consent is permitted.

(iii) Full withdrawal of consent: Withdrawal of consent to participate in the study and unavailability of all data from the time of patient registration, including information at registration.

In addition, some medical institutions may request that a "withdrawal of consent" form be prepared as a written document. However, in the event of withdrawal of consent, written expressions of willingness are required to increase the psychological barriers to withdrawal of consent (i.e., it is difficult to withdraw consent), and it is considered unwanted from the viewpoint of protecting human subjects. Therefore, in JCOG, written expressions of willingness are not mandatory for withdrawal of consent, verbal withdrawal of consent is valid, and the "withdrawal of consent" form is not prepared. If "withdrawal of consent" form is required by the participating institution, it should be prepared by the institution.

- This should be used in studies conducted in accordance with the Ethical Guidelines for Medical and Health Research Involving Human Subjects.

The procedures for discontinuation of the patient's follow-up request and removal of patient data will be specified separately in the procedural manual, and the completion of each task will be reported to Study Chair and Study Coordinator.

(i) Patient refusal: Refusal to continue subsequent protocol treatment (follow-up continues).

(ii) Withdrawal of consent: Withdrawal of consent to participate in the study and termination of all subsequent treatment and follow-up in accordance with the study protocol. Research use of data prior to withdrawal of consent is permitted.

(iii) Full withdrawal of consent: Withdrawal of consent to participate in the study and unavailability of all data from the time of patient registration, including information at registration.

In addition, some medical institutions may request that a "withdrawal of consent" form be prepared as a written document. However, in the event of withdrawal of consent, written expressions of willingness are required to increase the psychological barriers to withdrawal of consent (i.e., it is difficult to withdraw consent), and it is considered unwanted from the viewpoint of protecting human subjects. Therefore, in JCOG, written expressions of willingness are not mandatory for withdrawal of consent, verbal withdrawal of consent is valid, and the "withdrawal of consent" form is not prepared. If "withdrawal of consent" form is required by the participating institution, it should be prepared by the institution.

13.3. Protection of personal Information and patient identification

JCOG recognizes that information on privacy, such as personal information and medical information, should be protected and handled carefully, based on the principle of respecting the personality of individuals, and has formulated JCOG Privacy Policy, and will take all possible measures to protect privacy. For more information, see JCOG website (http://www.JCOG.jp/).

13.3.1. Policies, legislation, and norms followed by JCOG

In conducting JCOG study, JCOG follows, in principle, JCOG Privacy Policy as well as the following laws and norms depending on the content of the research. If other laws, norms, and policies are applicable, they should be followed.

- Clinical Trials Act (Law No. 16, 2017).
- Act on the Protection of Personal Information (Law No. 57, 2003, Final Amendment: Law No. 65, September 9, 2015).
- Helsinki Declaration (Translation by the Japan Medical Association)
- Ethical Guidelines for Medical and Health Research Involving Human Subjects (No.1 of Notice of the Ministry of Education, Culture, Sports, Science and Technology and the Ministry of Health, Labour and Welfare, 2017).

13.3.2. Use of personal information objective, items to be used, and methods of use

1) Objective of use

In accordance with the basic philosophy "Providing the best treatment to more patients," JCOG uses personal information, etc. of patients for objective of "Identifying patients and conducting surveys not only during treatment but also for a long period after end of treatment in order to obtain the correct results of clinical studies, and appropriately managing the acquired information."

2) Items to be used

Information to identify individuals who will be used by JCOG as minimally require for identification and inquiry of patients is as follows.

Medical record number, date of birth, initials, and registration number, Pathology specimen number

In other words, information that can identify individuals other than those listed above, such as the patient's name, is not informed to Data Center by participating institutions, and if they are falsely informed, they should be destroyed without using the recording medium or stored after performing appropriate processing, such as masking, which is incapable of reading.

3) Method of use

Personal information of patients used by JCOG will be collected by entering the CRFs etc. by researchers at participating institutions and submitting them to Data Center either by JCOG Web Entry System, mailing, or handover as a rule. However, telephone calls will be used only for patient registration where prompt contact is necessary.

In addition, in order to confirm the accuracy of the collected information, inquiries regarding various types of CRFs, including personal information, between Data Center and researchers at medical institutions are limited to either JCOG Web Entry System, mailing, or handover. Only the more anonymous registration number should be used when interacting with e-mail inquiries, and medical record numbers and initials should not be used.

13.3.3. Preparation of records for provision of samples and information, etc. at participating institutions

The investigator at each site will prepare records for this study of enrolled patients. The matters prescribed by the Ordinance of the Ministry of Health, Labour and Welfare (Article 53 of Clinical Trials Act Enforcement Regulations) are as follows.

- Identification of clinical-research subjects
- Items related to medical care and testing for subjects personnel in clinical studies
- Items related to participation in clinical research
- Other matters necessary to conduct clinical research

13.3.4. Source documents used in clinical studies (JCOG trials conducted under Clinical Trials Act)

The source documents for clinical studies used in this study refer to all records used for diagnosis and treatment, including medical records (including worksheets, etc.), laboratory test records, diagnostic images used for diagnosis, pathological diagnosis report, images used for response evaluation, and informed consent documents, of enrolled patients in this study. These should be available for direct access for monitoring (14.1.) and audit (14.2.) conducted by Data Center, Operations Office, Certified Review Board and regulatory persons

13.3.5. Storage of samples and information

Samples and information of enrolled patients on this study shall be stored in accordance with Article 53 of Clinical Trials Act Enforcement Regulations (MHLW ordinance No. 17, 2018). The retention time of records related to this study at participating institutions and the retention time of source documents will be 5 years from the date of completion of the study. It is recommended that the records be stored for as long as possible after the expiration date.

The retention time of data collected in JCOG Data Center will be semi-permanent in view of the possibility of long-term follow-up and secondary use for other studies. In addition, as records for the provision of samples and information, the study protocols and the model informed consent forms will be stored semi-permanently in JCOG Data Center.

13.3.6. Anonymization and control of response tables

In JCOG studies, information that can clearly identify individuals, such as patients' names, is not collected, and individuals are identified using registration numbers and medical record numbers (anonymized). Correspondence tables (not always in the form of a table) of information and registration numbers that can clearly identify individuals by themselves, such as patient names, are positioned as present at each participating institution and are appropriately managed according to the policy of participating institutions so that the identification of enrolled patients can be ensured.

13.3.7. Secondary use of data

Data from this study may be used in Japan or abroad for secondary use (e.g., meta-analysis) only if approved by the relevant committee of JCOG (e.g., Protocol Review Committee, Data and Safety Monitoring Committee). However, when providing data to external organization(e.g., a meta-analysis), data are provided so that it is not possible to identify individuals.

Secondary use of data should be disclosed on JCOG website to ensure opportunities for patients to refuse using data.

13.3.8. Safety management responsibility system

JCOG Data Center establishes a privacy protection control manager and a privacy protection manager and takes various safety control measures to minimize the risks of information leakage when using personal information, etc.

13.3.9. Response to request of disclosure of patient information

In the event that JCOG is asked to disclose privacy-related information, etc., the responder shall, in principle, be a researcher (site investigator, subinvestigator) at the institution of the relevant patient.

13.3.10. Receipt of inquiries about JCOG

General inquiries and complaints about privacy policies will be received by either mail, e-mail, or fax below.

Inquiry liaison: JCOG Data Center Privacy Protective

Postal destination :〒104-0045 Tsukiji 5-1-1, Chuo-ku, Tokyo
Clinical Research Support Office, National Cancer Center Hospital
E-mail :JCOG_privacy@ml.JCOG.jp
FAX :03-3542-3374

13.4. Adherence to the protocol

Researchers participating in this study will adhere to this protocol unless they compromise patient safety and human rights.

13.5. Application to Certified Review Board and Notification of Implementation Plans

When conducting this study, the approval of Certified Review Board and the permission by the administrator of each institution to conduct the study using this protocol and written informed consent from patients must be obtained. Prior to the commencement of the study, the implementation plan ※1 shall be submitted to the Minister of Health, Labour and Welfare, and the test data shall be published to jRCT※2. Principal Investigator is responsible for applying to Certified Review Board, submitting Implementation Plans to the MHLW, and registration in jRCT, and JCOG Operations Office supports these application procedures.

※ 1 Form No.1 of the Ministerial Ordinance stipulated in Article 39, Paragraph 1 of the Ordinance for Clinical Trials Act Enforcement Regulations

※ 2 Databases (Japan Registry of Clinical Trials) prepared by MHLW as specified in Article 24, Paragraph 1 of Clinical Trials Act Enforcement Regulations https://jrct.niph.go.jp/

13.5.1. Procedures for new application

1) Procedures performed by Principal Investigator

<Procedures from initial submission to study initiation>

(i) Review and approval of the study protocol by JCOG Protocol Review Committee (ver.1.0.0).

(ii) Principal Investigator will prepare the following documents for this study and submit these documents to Certified Review Board for review through JCOG Operations Office.

- ➢ New Review Request Form (Unified Form No.2 for Clinical Research)
- ➢ Implementation Plan (Ministerial Ordinance Form No.1)
- ➢ Study plan (this protocol) (including response to "disease or the like")
- ➢ Informed consent form
- ➢ Documents describing summary of drugs, etc. (e.g., package inserts of drugs used as a part of protocol treatment)
- ➢ List of Subinvestigators (Unified Form No.1 for Clinical Research)
- ➢ Conflict of Interest Management Standard (Guidance Form A), Conflict of Interest Management Plan (Guidance Form E) (see 13.X.)
- ➢ Other documents to be submitted when prepared (review documents of JCOG Protocol Review Committee, sample Case Report Forms, and a draft agreement on funding with the marketing authorisation of drugs, etc. or its special associates)

(iii) Modifications such as protocols and informed consent forms are made to review opinions submitted by Certified Review Board as needed.

※ Response to the indications by Certified Review Board: Prepare modified versions with review and approval by the Director of Data Center if modification of the protocol or informed consent forms is needed (ver.1.0.1, ver.1.0.2, ver.1.0.3…). .

(iv) After Certified Review Board approval is obtained, JCOG Operations Office should have Certified Review Board approval date and the approved version number on the cover page of the protocol and informed consent form and appear on JCOG website's Protocol Download page.

(v) Principal Investigator will use Certified Review Board review results notification and the documents submitted in ② to obtain the approval of the administrator of the institution which he/she belongs to, send these documents to the site investigators of all participating institutions described in the Implementation Plan, and request that the administrators of each participating institution permit conduct the research.

(vi) JCOG Operations Office shall apply for jRCT registration under the supervision of Principal Investigator after obtaining the permission by the administrators of all participating institutions described in the Implementation Plan and confirming that the patient registration system is open. "Study progress" in the trial registration will be registered as "being recruited."

(vii) JCOG Operations Office will inform Principal Investigator that jRCT registration has been completed after submission of the registration application. Principal Investigator will output the Implementation Plan from jRCT and submit the Implementation Plan, informed consent form, and Certified Review Board review result notification to the MHLW (Local Health and Welfare Bureau, which is responsible for the location of Certified Review Board; the same below). After submission, Principal Investigator will promptly inform Certified Review Board described in the Implementation Plan. In addition, Principal Investigator shall promptly report the submission of the Implementation Plan to the administrator of the institution to which he/she belongs, and provide this information to the site investigators of all participating institutions listed in the Implementation Plan and JCOG Operations Office.

(viii) After confirming that the notification of the Implementation Plan, etc. to the Local Ministry of Health and Welfare has been accepted and that jRCT's status has been updated from "under registration application" to "registration open," Principal Investigator will inform JCOG Operations Office that it has become "registration open." JCOG Operations Office opens JCOG Data Center's patient registration system and provides trial initiation announcements to study group. The date of jRCT publication is the start date of the study. "】

JCOG Operations Office should be contacted for procedures involving the addition of participating institutions after the initial submission.

"16.X. Medical institutions" changes (adding or replacing participating institutions) correspond to changes in the content of the protocol, then the change procedure shall be performed in accordance with the "13.6.2.Procedures for

when a change in the protocol occurs after the start of the study"

2) Procedures performed by the investigators at each participating institution

Following Certified Review Board approval, the investigator at each participating institution will obtain permission to conduct the research by the administrator of the participating institution using a set of documents received from Principal Investigator (Certified Review Board review results notification and documents submitted to Certified Review Board). The site investigator shall promptly send a copy of the letter of permission of the relevant institution to the Data Center after obtaining the permission by the administrator to conduct the research.

If the study is Specified Clinical Trial under Clinical Trials Act, the site investigator who has been informed that the Implementation Plan has been submitted to the MHLW by Principal Investigator should promptly report that the Implementation Plan has been submitted to the administrator of the affiliated institution.

3) Permission to conduct research at participating institutions

The procedures for obtaining permission for conducting research from the administrator of the affiliated institution shall be in accordance with the regulations of each institution.

When a copy of the institutional approval form is sent to JCOG Data Center, either the site investigator or the site coordinator should send the copy. Original copies of the institutional approval form will be stored by the site coordinator, and copies will be stored by JCOG Data Center.

When a affiliated institution has restrictions on providing personal information, such as medical record numbers, when sending copies of the institutional approval form to JCOG Data Center, the site coordinator shall communicate that personal information cannot be provided, and also send documents, such as the corresponding table of the ID number for registration and the medical record number, which is specified in JCOG privacy policy.

In addition, the informed consent form for patients approved by Certified Review Board may not be modified except for the contact information of the institution or prespecified selection items. A common protocol will be used for all institutions, since no changes in the protocols will be permitted for each institution. If it is necessary to change the content of the protocol and informed consent form, consult with Principal Investigator and Study Coordinator if the administrator of the institution asks for modification of the protocol and the text of the informed consent form in order to make the change as the protocol and informed consent form used by all institutions.

13.5.2. Procedures for changing the study plan after the start of the study

1) Procedures performed by Principal Investigator

If any of the changes of (1), (2), or (3) below occurs in the conduct of this study from the <Procedures from the initial application to the commencement of the study> in "13.6.1. Procedures at the time of new application", Principal Investigator shall hear Certified Review Board's opinions by applying for a change to Certified Review Board.

The procedures for applying for changes to the Certified Review Board are in accordance with "13.6.1. Procedures at the time of new submission" in the procedures from initial submission to the start of the study in ②, ③, and ④, respectively. In the absence of a change in the Implementation Plan, notification to the MHLW is not necessary. In the event that a change in the Implementation Plan occurs, notification to the MHLW is required. If Principal Investigator becomes aware of the planned change in the Implementation Plan, he or she should promptly contact JCOG Operations Office. JCOG Operations Office will assist in the application procedure for change to Certified Review Board and registration in change to jRCT.

(1) When the content of the protocol or informed consent form is changed (when it is amendment or revision in 13.7.1.).

(2) When the protocol or informed consent form is not changed, but the Implementation Plan (registered in jRCT) is changed.

(3) When changing Conflict of Interest Management Standards or Conflict of Interest Management Plans

Because notification of changes in the Implementation Plan (registered in jRCT) to the MHLW except for changes in research progress must be done in advance, Principal Investigator should inform site investigators and JCOG Operations Office of the change including the replacement of the investigator or the subinvestigator at each

participating institution. In addition, inform other investigators as soon as there are any changes that need to be made known.

Principal Investigator shall promptly report to the administrator of the institution to which he/she belongs and provide information to other research investigators when informed by Certified Review Board.

Procedures when it is necessary to notify changes to the Certified Review Board and the MHLW before changes are made.

1. Changes to the Implementation Plan

If changes to any of the above (1), (2), or (3) are made to the Implementation Plan (registered in jRCT) after hearing Certified Review Board's opinions, JCOG Operations Office should enter changes to jRCT under the supervision of Principal Investigator. In addition, Principal Investigator submits the following notification to the MHLW. After the date of coming into force of the predefined changes, the study shall be conducted in accordance with the changes.

- Notification of Change in Implementation Plan (Form No.2 of the Ministerial Ordinance)
- Implementation Plan after the change (output of the change in jRCT)
- Certified Review Board Review Results Notification

※ On institution transitioning to JCOG Collaborating Institution by replacement of participating institution: For institutions where patients were not enrolled prior to transfer to collaborating institution, Principal Investigator should remove it from the participating institutions by notifying changes in the Implementation Plan.

Procedures when it is necessary to notify a change to the Certified Review Board and the MHLW after the change***

1. Change in progress

(2) Among the changes in the Implementation Plan (registered in jRCT), the change in "3. Issues related to checking the implementation of Specified Clinical Trials (2) Specified Clinical Trials progress" shall be made without delay after the change. Under the supervision of Principal Investigator, JCOG Operations Office shall change "Research Progress" in jRCT. Subsequently, Principal Investigator will promptly apply for a change to Certified Review Board. After obtaining approval from Certified Review Board, Principal Investigator will submit the following notification to the MHLW.

- Notification of Change in Implementation Plan (Form No.2 of the Ministerial Ordinance)
- Implementation Plan after the change (the content of the change in jRCT was outputted)
- Certified Review Board Review Results Notification].

2. Changes in Implementation Plan after registration of the first patient

At the time of the initial submission, the "date of registration of the first patient" in the Implementation Plan is provided in a blank space. The Implementation Plan should be changed without delay after registration in the first patient. In this instance, JCOG Operations Office enters and registers the "First Patient Registration Date" in jRCT under the supervision of Principal Investigator. Subsequently, Principal Investigator will promptly apply for a change to Certified Review Board.

- Notification of Change in Implementation Plan (Form No.2 of the Ministerial Ordinance)
- Implementation Plan after the change (output of the change in jRCT)
- Certified Review Board Review Results Notification]

3. Minor changes specified by MHLW ordinance (application for change to Certified Review Board is not required)

If the following minor changes are made to Implementation Planning and the registration of jRCT, Principal Investigator will not need to hear the opinions of Certified Review Board and will notify Certified Review Board of the changes within 10 days of the date of the change. In addition, a notification (Form No.3) shall be submitted to the MHLW.

Scope of minor changes to the Implementation Plan of Article 42 of Clinical Trials Act Enforcement Regulations

- A change in the name of a person engaged in Specified Clinical Trials that does not involve a replacement of the person engaged in Specified Clinical Trials.
- Changes due to a change in the name or address number of the area

2) Procedures performed by the investigators at each participating institution

If any changes occur in the part of the protocol that corresponds to the institution to which he/she belongs, (4) Items related to investigators in multi-institutional studies, inform Principal Investigator and JCOG Operations Office of the changes to be made prior to the changes. Depending on the content of the change, the investigator shall confirm the confirmation of institutional requirements, prepare documents for conflicts of interest and submits to Certified Review Board such as lists of subinvestigators, and inform Principal Investigator and JCOG Operations Office.

The content of the most recent Implementation Plan for the institution to which they belong (the same as the content of jRCT registration) should be checked on jRCT website (https://jrct.niph.go.jp/).

13.5.3. Review and approval of study progress and study continuation (periodic reports)

Principal Investigator will report periodic reports on the progress of the study, the occurrence of adverse events, and conflict of interest management (see 13.X.1.⑱) to the administrators of their institutions and report them to Certified Review Board. Within two months after the expiration of each year from the date of submission of the Implementation Plan to the Minister of Health, Labour and Welfare.

When the report is made to Certified Review Board, Principal Investigator shall promptly inform the investigators of other participating institutions of the fact. The investigator who receives the information shall promptly report the details of the information to the administrator of the institution to which he/she belongs.

If the study falls under Specified Clinical Trials above Clinical Trials Act, Principal Investigator will report to the MHLW regarding the implementation status of Specified Clinical Trials. Periodic reports to Certified Review Board shall be made within 1 month of the date on which the results on the appropriateness of continuation of the relevant Specified Clinical Trials are obtained. The report shall be made by submitting the attached Form 3※ to the Minister of Health, Labour and Welfare.

※ Enforcement of Clinical Trials Act Enforcement Regulations, etc. (Notification No. 0228-1 of the Sector of Economics, Ministry of Health, Labour and Welfare, Notification No. 0228-1 of the Notification No. 1 of the Evaluation and Development Division, Health Policy Bureau, Ministry of Health, Labour and Welfare, dated February 28, 2018)

13.6. Protocol revision/amendment

13.6.1. Categorization of protocol changes and procedures for changes

For a change in the protocol, the Protocol Revision Application must be submitted to Data and Safety Monitoring Committee Office prior to submission to Certified Review Board [in the case of Specified Clinical Trials, add the following: "and report to the MHLW"] (see 13.6.2).

JCOG deals with the changes in the content of the protocol after approval by the Protocol Review Committee, divided into amendment and revision. Data and Safety Monitoring Committee Office will distinguish between amendment and revision, so all of applications are submitted as revision. If it is classified as an amendment by the Secretary-General of Data and Safety Monitoring Committee, it is reviewed by Data and Safety Monitoring Committee. If it is classified as a revision, Secretary-General of Data and Safety Monitoring Committee will issue a verification form and will not be reviewed by Data and Safety Monitoring Committee. We also distinguish the addition of supplementary explanations that do not fall into a change in protocol content as "Memorandum". Definitions and handling are as follows:

When the protocol or informed consent form is revised or amended, Principal Investigator will promptly distribute the most recent protocol or informed consent form to those involved in the study. Individuals involved in the study should always conduct the study in accordance with the most recent protocol since the dates of entry into force of the amendment/revision.

1) Amendment

Partial protocol change which meets one or more of the followings: i) Potential to increase the risk of patients enrolled in the study, ii) Having substantial effects on primary endpoint of the study, iii) having essential effects on the study's implementation structure.

The amended version of the protocol and informed consent form version numbers are shown as in 2.0.0, 3.0.0, and 4.0.0….

Approval by the Group Chair and the Data Center Director must be obtained prior to submission to Data and Safety Monitoring Committee.

When classified as "amendment" by the Secretary-General of Data and Safety Monitoring Committee, review of changes by Data and Safety Monitoring Committee is performed prior to Certified Review Board review. After the protocol amendment has been approved by Data and Safety Monitoring Committee, an application for a change in the protocol will be submitted to Certified Review Board through JCOG Operations Office (at this time the version number is ver. 2.0.0, 3.0.0, 4.0.0…). When the protocol was changed based on the review opinions of Certified Review Board, the version number is ver. 2.0.1, ver.2.0.2… If the protocol is changed by review of Certified Review Board, the change will be reported to Data and Safety Monitoring Committee, but in principle, the change will not be reexamined by Data and Safety Monitoring Committee. When a protocol change is approved by Certified Review Board, the protocol cover page should include the date of approval by Data and Safety Monitoring Committee and Certified Review Board [If Specified Clinical Trials, the following is added: "and a notification of the change in the Implementation Plan to the Regional Bureau of Health and Welfare should be made"].

Following Certified Review Board approval, permission for the contents of the amendment by the administrator of each institution shall be obtained. If permission is obtained, the site coordinator of each institution will send copies of the permission notice by the administrator of each institution to the Data Center. After the permission of the administrator of all institutions is obtained, the protocol changes will come into effect (during this time, the patient registration will not be suspended unless there is a special need). The actual date of entry into force will be announced by JCOG Operations Office. Researchers in all participating institutions conduct the study according to revisions approved by Certified Review Board since the date of entry into force.

Treatment and assessment of enrolled patients will be performed according to the pre-change version protocol until entry into force. Protocol deviations to enhance patient safety during treatment will be permitted if pre-change protocols, such as inadequate treatment modification criteria, threaten patient safety. If protocol deviations occur, they should be listed in the monitoring report.

2) Revision

Protocol change which meets all of the followings: i) does not have an increased risk for patients enrolled in the study; ii) does not have a substantial effect on primary endpoint of the study; iii) does not have an inherent effect on the system in which the study is conducted. Includes changes in protocols due to mistakes or changes in institution-specific information, changes in institution-specific information without changes in protocols (changes in Implementation Plans and jRCT registrations), and changes in conflicts of interest at individual institutions. In principle, suspension of patient registration is not performed in case of revision.

The revised version of the Protocol and Informed Consent Form version numbers are shown as in 1.1.0, 1.2.0, and 1.3.0….

Approval by the Group Chair and the Data Center Director must be obtained prior to submission to Data and Safety Monitoring Committee.

If classified as "Revision" by the Secretary-General of Data and Safety Monitoring Committee, the Secretary-General of Data and Safety Monitoring Committee issued a verification form, Data and Safety Monitoring Committee did not review the changes. The Principal Investigator submit an application for a change in the protocol to Certified Review Board through JCOG Operations Office (at this time, the version number is ver.1.1.0, 1.2.0, 1.3.0…). If the protocol was changed based on the indication by the Certified Review Board, version number shall be as ver.1.1.1, ver.1.1.2… When a protocol change is approved by Certified Review Board, the date of approval by the Certified Review Board should be entered on the protocol cover page. [If Specified Clinical Trials, the followings are added: "and a notification of the change in the Implementation Plan to the Regional Bureau of Health

and Welfare should be made"]

The date of entry into force of the protocol change will be 2 weeks after Certified Review Board approval date unless otherwise specified. [in the case of a Specified Clinical Trials, the followings are added: "The effective date shall be the date after the notification of change of the Implementation Plan to the Regional Bureau of Health and Welfare"]

The actual date of entry into force will be announced by JCOG Operations Office. Researchers in all participating institutions conduct the study according to revisions approved by Certified Review Board since the date of entry into force.

At that time, the site investigator should obtain permission by the administrator of each institution after the date of approval of Certified Review Board and before the date of entry into force. In this case, the protocol revision may be permitted with a report to the administrator of the institution. However, the procedures for obtaining permission from the administrator shall be in accordance with the regulations of each institution. For protocol revisions, confirm with the institution prior to initiation of the study to ensure that permission is obtained within the aforementioned time periods, and contact JCOG Operations Office if this is difficult. Reports to the administrator and approval form by the administrator at each institution need not be sent to the Data Center, but the original copy will be retained by the site coordinator as it will be checked during site visit audits.

Treatment and assessment of enrolled patients will be performed according to the pre-change version protocol until entry into force. Protocol deviations to enhance patient safety during treatment will be permitted if pre-change protocols, such as inadequate treatment modification criteria, threaten patient safety. If protocol deviations occur, they should be listed in the monitoring report.

3) Memorandum

Supplementary description of the protocol distributed from Principal Investigator/Study Coordinator to study personnel in objective, such as reduction of interpretive variation in sentences, and special precautions, rather than change of protocol content. Any form is used.

Approval by Group Chair and the Director of the Data Center is needed prior to distribution. Reporting to Data and Safety Monitoring Committee before distribution or immediately after distribution is required.

It is not necessary to include the protocol on the cover page.

13.6.2. Patient explanation and re-consent at the time of protocol amendment/revision

In the event of a change in the content of the study, the investigator or the subinvestigator shall provide appropriate explanation to the enrolled patients (regarding protocols based on revision, treatment, follow-up, etc.). In addition, if Certified Review Board comments that re-consent of enrolled patients in writing is required, informed consent should be obtained in writing.

13.7. Conflicts of Interest (COIs) involved in this study

13.7.1. COI management involved in this study

The COIs involved in this study will be managed according to the "Guidance for Conflict of Interest Management in Clinical Trials Act (Notification No.1130-17 of the PMSB dated November 30, 2018) ※ (Guidance) of the Division, Research and Development, Ministry of Health, Labour and Welfare, in accordance with the following:

※ http://www.mhlw.go.jp/stf/seisakunitsuite/bunya/0000163417.html

The format used for COI control should be the latest version of the guidance.

- Conflict of Interest Management Standards: Form A
- Reports of related companies, etc.: Form B
- Investigator Conflict of Interest Self-Report Form: Form C
- Conflict of Interest Confirmation Report: Form D
- Conflict of Interest Management Plan; Form E

1) Procedures for New Application

Conflict of Interest Management Standards

(i) All JCOG trials according to Clinical Trials Act will employ Conflict of Interest Management Standards (Form A) in accordance with guidance.

Request for Confirmation of Conflicts of Interest

(ii) At the time of initiation of the primary review of the protocol, JCOG Operations Office shall identify companies, etc. related to this study that require conflict of interest management based on information on drugs and medical devices specified in the Protocol Treatment (see 6.1.), enter the relationship with the company in Form B, and ask Principal Investigator to confirm the accuracy of the entry.

(iii) After checking the entries in Form B received from JCOG Operations Office, Principal Investigator will send a format to the site investigator and site coordinator at each participating institution and ask to confirm the entries in Form B, create Form C, and create Form E. In addition, a form shall be sent from Principal Investigator to those who correspond to the "person responsible for statistical analysis" and the "administrator other than Principal Investigator and Investigators" of the Implementation Plan, and they shall be asked to prepare Forms C and E.

Confirmation of Conflicts of Interest (participating institutions)

(iv) The site investigator or site coordinator at each participating institution will register the investigators, subinvestigators, and research associates involved in the study in JCOG Web Entry System. Investigators and subinvestigators registered in JCOG Web Entry System will be the reporters of conflicts of interest in the study.

(v) The site coordinator will request the investigator of the study to confirm the description of Form B received from Principal Investigator, create Form C, and create Form E. The investigator will then summarize conflict of interest management within the study site.

(vi) The investigator checks the forms received from Principal Investigator for any relevant items in Q2 through Q5 of Form B and notifies Principal Investigator through JCOG Operations Office within a week if any.

(vii) In the column of Form C [Persons requiring a Form C self-declaration of conflict of interest (Form C)] ※, the site investigator inputs the information of the investigator and subinvestigator registered in JCOG Web Entry System, and asks subinvestigator to prepare Form C.

※ The information entered in Form C is automatically entered in Form E. This entry should be consistent with the physician's information contained in the "List of Subinvestigators" (Clinical Trials Act Uniform Form No.1) submitted to Certified Review Board with Form E, and physicians who do not agree may not be involved in this study. Therefore, when entering the information in the ※ column for [those who require a Conflict of Interest Self-Reporting Form (Form C)], the investigator or subinvestigator list should be downloaded in JCOG Web Entry System and the physician listed in the Study Subinvestigator list should be entered without missing the person who will be the investigator and the subinvestigator in the study. If the information in the downloaded "Study Subinvestigator List" is not consistent with the physician involved in the study, the registered information should be updated in JCOG Web Entry System, and the "Study Subinvestigator List" with the most recent information reflected should be downloaded and used.

(viii) The investigator and the subinvestigator shall complete the necessary information regarding the relationship with the company, etc. described in advance in Form C, and submit Form C to the Conflict of Interest Confirmation Department of the institution to which he/she belongs. In doing so, the investigator also submits Form A.

(ix) The investigator receives the results of confirmation of conflicts of interest (Form D) of the investigator and the study subinvestigator from the institution to which he/she belongs.

(x) The investigator will confirm the content of Form A, Form B, and Form D of the investigator and all study subinvestigators to create Form E. The investigator will notify Principal Investigator through JCOG Operations Office of Form E and the Study Subinvestigator List downloaded in Form 7.

Confirmation of Conflicts of Interest (outside participating institutions)

(xi) The person responsible for statistical analysis and the person overseeing a study other than Principal Investigator or investigator shall complete, in the form received from Principal Investigator, the necessary information regarding the relationship with the company, etc. described in advance in Form C, and submit Form A and Form C to the Conflict of Interest Confirmation Department of the Affiliated Institution.

(xii) The person responsible for statistical analysis and the person overseeing studies other than Principal Investigator or investigator shall receive confirmation results (Form D) from the institution.

(xiii) The person responsible for statistical analysis and the person overseeing studies other than Principal Investigator and investigator shall confirm the content of Form A and Form D, prepare Form E, and notify Form E to Principal Investigator through JCOG Operations Office.

Description of Conflicts of Interest in the protocol and informed consent form

(xiv) Principal Investigator and JCOG Operations Office will review Form A and Form E received from participating institutions and, as appropriate, will accurately describe the conflicts of interest (study COIs) between the study and the drug marketing authorisation holder, etc. in the protocol and informed consent form. Conflicts of interest (personal COI) between the reporters of conflicts of interest in this study and the marketing authorisation of drugs, etc. (personal COI) can change over time, so the personal COI is not described in the protocol and informed consent form, and the information is updated on JCOG website as needed.

Certified Review Board review

(xv) Principal Investigator compiles and submits the Form E and Study Subinvestigator List of all institutions to Certified Review Board for review.

2) Procedures for new involvement with companies after the start of the study

(xvi) When a new involvement with companies (research COI) occurs in the study after the start of the study

a. In the event of any change in the enterprises involved in this study, etc. to be described in Q1 of Form B, repeat the procedures ② to ⑭. However, the site investigator and site coordinator roles in ④, ⑤ shall be assumed by the investigator at each institution. If a new study COI needs to be added to the protocol and informed consent form, a protocol revision (see 13.X.X.) should be submitted to Certified Review Board for review.

b. If a change occurs from Q2 to Q5 in Form B, the investigator at the participating institution affected by the change will change the appropriate description in Form B and update Form E and send it to Principal Investigator and JCOG Operations Office. A Principal Investigator who has received a Form E will revise the protocol as needed (see 13.X.X.) and submitted to Certified Review Board for review.

(xvii) In the event that a conflict of interest reporter is newly involved with a company (personal COI) after the start of the study

Conflict of interest reporters repeat procedures from ⑧ to ⑩ or from ⑪ to ⑬. However, if there is no change in Form E, these procedures will be performed at each institution but will not be sent to Principal Investigator and JCOG Operations Office. Principal Investigator will submit Post-change Form E sent from the site investigator to Certified Review Board for review. Personal COIs are also disclosed on JCOG website.

3) Procedures for periodic reporting

(xviii) Principal Investigator checks for changes in study COI and personal COI annually at the timing of periodic reporting and reports to Certified Review Board.

13.7.2. COI with companies involved in this study(study COI)

There are no conflicts of interest to disclose about relationship between the company manufacturing and marketing drugs specified as a part of protocol treatments in this study (See 6.1).

13.7.3. COIs of JCOG Committee members and JCOG Data Center/Operations Office staff

COIs of the committee members of JCOG committees, the staffs of the office of committees, and JCOG Data Center/Operations Office staff involved in the study will be managed by JCOG Conflict of Interest Committee in accordance with Clinical Trials Act control standards.

13.8. Compensation

In order to comply with Clinical Trials Act, this study must take necessary measures such as sing up for insurance and ensuring a system to provide medical care in order to compensate for the health damage caused by the study

and provide medical care.

Therefore, regarding the health damage caused by participating in this study, appropriate treatment according to the condition is provided similarly to the usual insurance medical care. In addition, this study will be covered by clinical study insurance, and the following will be compensated based on insurance conditions, and this will be explained to patients and understood.

13.9. Intellectual property

The results, data, and intellectual property rights obtained from this study are attributed to the followings: National Cancer Center, Principal Investigator, Study Coordinator, and Group Chair. Specific procedures and allocation must be determined through consultation among four parties. Whether the intellectual property related to Principal Investigator, Study Coordinator, Group Chair will belong to the individuals or the affiliated institution will be determined according to the agreements of the affiliated institution.

13.10. Disclosure of information on this study

Summary, progress, and main results of this study will be published on JCOG website (www.jcog.jp) and on jRCT (https://jrct.niph.go.jp/).

14. Monitoring and audit

14.1. Periodic monitoring

In this study, monitoring is performed in order to ensure that the study is conducted safely and in accordance with this protocol and that data are collected accurately. Periodic monitoring should be performed twice a year in principle, using the entered data on the CRFs collected in the Data Center. Specific procedures for periodic monitoring are provided separately in the Monitoring Plan.

The Data Center submits a "Monitoring Report" summarizing the results of central monitoring to Principal Investigator, Study Coordinator and investigators. Together, they are submitted to Group Chair, Data and Safety Monitoring Committee, and JCOG Chair.

The Monitoring Report is a material for periodic reports to be made every year from the date of submission of the Implementation Plan.

14.1.1. Monitoring items

① Registration status: number of registration - cumulative/by month, by arm/site
② Eligibility: ineligibles/potentially ineligible cases: by arm/site
③ Pre-treatment baseline factors: by arm
④ On/off-treatment, reason for treatment termination: arm/site
⑤ Protocol deviation: arm/site
⑥ Serious Adverse Events: arm/site
⑦ Adverse reaction/event: arm
⑧ Overall survival, progression-free survival (or relapse-free survival, etc.): all registered patients
⑨ Other issues related to study progress and safety (studies in accordance with Clinical Trials Act: status of occurrence of non-compliance and subsequent response, number of subjects for compensation, number of disease or the like reports in accordance with Article 13 of Clinical Trials Act)

14.1.2. Eligibility (Eligible/Ineligible)

For all registered patients, eligibility will be classified according to the following definitions as either: In monitoring, Data Center lists potentially ineligible cases in the "Evaluation of Eligibility" section of the monitoring report, and after review by Study Coordinator, confirms them to be either 1), 2), 9), or 99) with Group Chair approval prior to performing primary analysis.

Only 1) eligible shall be "eligible case", 2) post hoc ineligible,9) de facto ineligible and 99) violation of registration shall be "ineligible case". This is a category established from the perspective of analysis set setting.

In the study in accordance with Clinical Trials Act, "99) violation of registration" will be regarded as "major non-compliance" in Clinical Trials Act, and Principal Investigator will promptly report the situation to Certified Review Board as soon as possible. See 14.3. for management of non-compliance.

9) de facto ineligibles corresponds to "non-compliance" on Clinical Trials Act and is reported to the administrator of participating medical organizations with the submission of the monitoring report on which they were described (twice a year).

2) Post hoc ineligibles is not treated as "non-compliance" on Clinical Trials Act because it does not correspond to non-compliance with the study protocol, as discussed below.

<u>1) Eligible</u>

All information generated prior to registration meets all o the Patients Selection Criteria according to the methods and criteria specified in the study protocol.

<u>2) Post hoc ineligible</u>

The information generated after registration does not meet either Patients Selection Criteria, or the information generated prior to registration does not meet either Patients Selection Criteria by methods or criteria other than those specified in the protocol.

Examples)

(i) In the study for Stage II-III, bone scintigraphy performed immediately after registration revealed bone

metastases, and the patient was diagnosed as Stage IV. The protocol treatment was terminated.

(ii) In the study for early gastric cancer, bloody stools is seen after registration, and colonoscopy revealed advanced colorectal cancer (synchronous double cancer). Colectomy was performed after termination of the protocol treatment.

(iii) In the study for gastric cancer (adenocarcinoma), the institution's pathological diagnosis was changed to malignant lymphoma after registration.

9) De facto ineligible

Information generated prior to registration according to protocol-specified methods (performed in all cases) and criteria does not meet either Patients Selection Criteria. This includes cases where it is determined after registration that the information that occurred before registration had been incorrect.

Example: When the supervising physician reviews the CT images performed before registration as specified, there is obvious liver metastasis (if it is a mistake by the attending physician and it is considered that there is no future).

99) Violation of registration

Deliberately (falsely) enroll while knowing that Patients Selection Criteria is not met. Corresponds to a misconduct and treats it as a serious problem.

14.1.3. Protocol Deviations/Violations

Protocol deviations are defined as those in which treatment, such as drug administration, radiotherapy, or surgical resection, as well as laboratory tests and evaluation of toxicity and efficacy, etc. were not performed according to the protocols.

In monitoring, deviations that exceed a certain acceptable range limit for each study decided by the Data Center and Principal Investigator/Study Coordinator prior to or after the initiation of the study are listed in the monitoring report as "possible deviations" and are classified into one of the following categories after consideration by Study Coordinator and study groups: Except for those described in the protocol and monitoring report, the acceptable deviations agreed between the Data Center and Principal Investigator/Study Coordinator may be changed through periodic monitoring during the study, so they should be described in the supplemental material rather than in the text of the Monitoring Plan, and the Monitoring Plan should be described as "defining the acceptable ranges separately".

1) Violation

Any deviation from the protocol that is clinically inappropriate and caused by the treating physician/institution and that meets two or more following criteria shall be classified as a violation. [When conducted in accordance with Clinical Trials Act, the following shall be added. [In the study according to Clinical Trials Act, the violation shall be treated as "major non-compliance" and Principal Investigator should report the situation to Certified Review Board as soon as possible].

① Have a substantial impact on the assessment of study endpoints

② Intentional or systematic

③ Dangerous or remarkable deviation

For "violations", in principle, the content of each violation should be described in a paper when publishing.

2) Deviation

Deviations that do not fall into 1) violation or 3) acceptable deviation. If same kind of deviations are frequent, they should preferably be included in the publication of the article. They are classified as either of the following at the time of monitoring report review:

Because deviations correspond to "non-compliance" in Clinical Trials Act, they are reported (biannually) to the administrators of the institution with the submission of the monitoring report. Not to be "major non-compliance".

(i) Deviations - Undesirable and to be reduced

(ii) Deviations (unavoidable) - things that are not proactively reducing (e.g. delay by the New Year period, equipment breakdown, etc.)

(iii) Deviations (clinically relevant) - Those in which the decision of the treating physician/institution are positively affirmed (if a similar situation again arises similar deviations are considered desirable).

※ Deviations do not always mean that the treating physician at the institution is problematic. Since patient safety is a primary priority in clinical trials, deviations should rather be made by the medical judgment of the treating physician if the condition of the individual patient are considered to be dangerous when following the protocols. If the deviation is judged to be clinically relevant for the safety of the patient, it is recorded as ③ Deviation (clinically relevant). Clinically relevant deviations in a small number of patients are not required to be particularly problematic; however, protocol revision should be considered when multiple deviations occur because protocol specification is likely to be inadequate. However, deviations that are not intended to be safety (e.g., increased doses of anticancer drugs in the hope of increasing efficacy, shortened treatment periods not specified in the protocol) are not considered clinically relevant deviations.

3) Acceptable deviation

Deviations from protocols within acceptable range agreed by the entire JCOG, study groups, or Study Chair/Study Coordinator and Data Center, pre- or post-study initiation, on a trial-by-trial basis. When conducted according to Clinical Trials Act, the following are added. Not considered to be "non-compliance" in Clinical Trials Act. Deviations within the pre-specified acceptable ranges are not included in the monitoring report.

14.2. Site visit audits

In this study, site visit audits will be conducted to ensure the reliability of clinical research and the reliability of data and information collected by clinical research from the perspective of protecting human subjects in clinical research.

Site visit audits are conducted by auditors appointed by Principal Investigator by visiting a medical institution to confirm the approval documents of the medical institution, check the list of subinvestigators in the research institution, confirm the informed consent documents, and verify CRF entry data with medical records (direct access to source documents). Specific procedures for site visit audits are provided separately in the operating procedures.

The auditor shall report the audit report summarizing the audit results to Principal Investigator/Study Coordinator, site investigators. Together, the report will be submitted to Group Chair, Director of JCOG Data Center, Director of JCOG Operations Office, and JCOG Chair. Reports should also be submitted to the site investigators of the relevant groups and JCOG Executive Committee as appropriate.

14.2.1. Items to be audited

In the site visit audits, the following items are checked by direct access to source documents:

<Confirmation Items by Study>

- Approval documents (including initial approval forms, revision approval forms, and annual report approvals/reports) from administrators of medical institutions
- Management status of the protocol
- Contents of explanatory documents and informed consent forms

<Confirmation Items by Patient>

- Patient consent (presence or absence of consent form, signature, and date of consent)
- Implementation of pre-registration mandatory tests, eligibility for registration (inclusion criteria and exclusion criteria)
- Accuracy of reported data
  - Pre-treatment evaluation, course of treatment (protocol treatment)
  - Various test results (including diagnostic imaging reports and pathology reports)
  - Accuracy of test date, response evaluation, adverse event, survival or death information, and other reported data

<Other items>

- Presence or absence of study misconduct (possibility of false reporting, fabrication, or falsification)
- Improvement status of the items pointed out in the previous audit

14.2.2. Reporting of major non-compliance found in audits to Certified Review Board

Principal Investigator/Study Coordinator should report to Certified Review Board immediately when finding possible "major non-compliance (See 14.3.2.)" as a result of site visit audits.

14.3. Management of non-compliance

14.3.1. Non-compliance.

Non-compliance in Clinical Trials Act refers to the condition in which clinical research is not compliant with Clinical Trials Act Enforcement Regulations or study protocols. In the Clinical Trials Act Enforcement Regulations, etc. (February 28, 2018), non-compliance with regulations, study protocols, operation procedures, etc., and fabrication of falsification of research data, etc. are listed as examples.

If the site investigator knows that there is non-compliance, the site investigator should report to the administrator of the medical institution and inform Principal Investigator/Study Coordinator.

If site investigator find non-compliance (regardless of major non-compliance or not) prior to the implementation of central monitoring or site visit audits, the site investigator will promptly report to Principal Investigator/Study Coordinator and JCOG Data Center.

"14.1.3.2) Deviations" correspond to "non-compliance" in Clinical Trials Act. As described in 14.1.3.2), these deviations are reported by submitting monitoring reports or their excerpts or summaries issued twice a year to the administrators of the medical institution.

Changes in protocols and implementation plans associated with investigator transfer require Certified Review Board review and notification of implementation plans to the MHLW, therefore, require a certain time to complete the sequence of procedures. In addition, it is often difficult to complete the change procedure before the transfer because the transfer may not be open until just before the transfer. Therefore, even if the site investigator is absent for a certain period of time due to the transfer, the study does not fall into "non-compliance" in this study if the research management system is maintained by the subinvestigator and the medical care system of the enrolled patients who are surviving is ensured.

14.3.2. Major non-compliance

"Major non-compliance" is that affect the human rights and safety of subjects of clinical research, the study progress and the reliability of study results. Examples of "major non-compliance" in JCOG study are provided in. If major non-compliance is likely, Principal Investigator/Study Coordinator will report the situation to Certified Review Board immediately.

1) Major non-compliance with respect to eligibility

Violation of registration

- Enrolled intentionally (falsely) while knowing that eligibility criteria was not met
- Patient enrollment was performed without necessary informed consent, and protocol treatment was performed
- The source documents for the determination of eligibility cannot be identified (including the loss of the consent form).

2) Protocol violation

Violations that affect the increased risk of enrolled patients or that affect the reliability of the study results

- Significant violation in inclusion criteria or exclusion criteria
- Violation threatening patient safety in off-treatment criteria
- Serious violation of prohibited concomitant drug, prohibited concomitant treatment, etc.
- e.g. intentional or systematic non-compliance with protocol regulations

3) Other major non-compliance

- Study was conducted prior to Certified Review Board approval or prior to approval of site administrator
- The study was continued without providing information to the enrolled patient that could affect the willingness to continue to participate the study.
- Those judged to be research misconduct (fabrication or falsification of data, etc.)

- Any leakage of personal information or violation of human rights that may have a significant impact on the enrolled patient.

15. Special Instructions

15.1 Central pathology diagnosis and related matters

15.1.1 Central pathological diagnosis

In this study, the pathological tissues of enrolled patients will be collected and the central pathological diagnosis will be determined after the fact. Since the central pathological diagnosis is not performed in real time for each individual patient enrollment, the main analysis target and the decision on treatment strategy will be based solely on the pathological diagnosis at the institution. The details of the central pathological diagnosis procedure will be specified separately in the Central Pathological Diagnosis Procedure Manual.

Timing: Once a year throughout the study period.

Subjects: All registered patients

Methods: Pathological specimens (or duplicates made from the same paraffin block) used for eligibility criteria determination at the registered facilities will be collected, and after necessary staining (chromogranin A, synaptophysin) is added, pathological eligibility will be re-determined by two or more pathology judges (16.8 Pathology judges) appointed by the group representative. (16.8 Central Pathological Review Comittie).

Management of the collected specimens: The Hepatobiliary and Pancreatic Group Study Office will be responsible for the management of the collected specimens.

Staining: If additional staining is required, it will be performed by the Central Pathology Coordinator (16.7).

Notification of the central judgment to each facility:

After the results of the central pathological diagnosis are fixed, the research secretariat will notify the results of the judgment to the registered facility (facility coordinator) of each patient. At that time, the rationale for the decision should be attached in writing. If there is a difference between the institutional diagnosis and the central diagnosis, the facility principal investigator/facility coordinator reports the decision results to the facility pathologist and discusses it with the facility pathologist, and carefully decides on the final pathological diagnosis at the facility (whether to change the diagnosis or not) and what to do if the patient is under treatment (whether to change the treatment or not).

15.1.2 Providing information to the institutional pathologist

In view of the high level of difficulty in NEC pathological diagnosis and the unique nature of this study, which covers multiple target organs, the following information will be provided to institutional pathologists.

(1) Pre-registration pathological diagnosis consultation

Since it is anticipated that there will be cases of confusion in diagnosis at facilities, pre-enrollment pathology consultation will be available as needed, with the pathology judgment committee members of this study serving as consultants. Details of the consultation operation are described in the Central Pathology Procedures.

(2) Holding of pathology-related meetings

In this study, the research secretariat and the pathology secretariat first held a pathology-related meeting (attended by pathologists from participating institutions) on February 11, 2014, during which a lecture was given to form a consensus on diagnostic criteria according to the WHO 2010 classification. The slides used at the meeting were revised as necessary based on the discussion at the meeting and posted on the JCOG website for reference by pathologists at each facility.

15.1.3 Response to cases in which pathological diagnosis was performed at a facility other than the home facility

In any of the following cases, be sure to ask a pathologist at your own institution to make the diagnosis, and confirm that the diagnosis at your institution is also NEC before enrolling in this study.

(1) When borrowing only stained tissue specimens from a previous physician for registration

Register after obtaining permission from the attending physician to the previous physician to submit the borrowed tissue specimen to JCOG 1213 for central diagnosis (loan again) and to borrow the specimen for a long period of time (up to about 1 year).

(2) If you have received virtual slides of "all tissue specimens for which a pathological diagnosis has been made" from

your previous doctor

The patient will be registered after obtaining permission from the attending physician of the previous physician to submit the virtual slides provided by the previous physician for the central diagnosis of this study.

(3) If you have received a "borrowed" pre-stained tissue specimen and an "offered" unstained preparative from your previous physician

The borrowed stained tissue specimens from the previous physician can be promptly reviewed at the patient's own institution, but the provided unstained preparations require time for pathological diagnosis after immunostaining at the patient's own institution, which may cause a time lag. There have been cases in which patients were enrolled in the study based only on the results of pathological review of borrowed tissue specimens at their own institutions, and later the results of staining and pathological diagnosis of undyed preparations at their own institutions overturned the pathological diagnosis (diagnosis of a different disease was made), resulting in post-hoc ineligible cases. Therefore, we will consider the timing of registration after sufficient consultation with pathologists at our own institution to avoid the occurrence of posterior ineligible cases as much as possible. However, if there is enough time, it is preferable to enroll unstained preparations into the study after staining and pathological diagnosis is obtained at your own institution.

15.2. JCOG BioBank Japan (BBJ) Biorepository

This study will participate in the banking of blood samples (DNA/plasma) in JCOG BBJ Biorepository based on a common protocol for all JCOG studies (hereafter referred to as common banking).

Subjects:

Among patients who agreed to participate in this study, patients whose consent to shared banking was obtained.

Sample:

1) Whole blood

Blood sampling is performed before the start of the protocol treatment in this study in principle. However, blood sampling after initiation of protocol treatment is allowed. Blood samples of 7 mL×2 (total 14 mL) of venous blood are collected using a blood collection tube (for blood counting) with a EDTA Na dedicated to the shared banking of JCOG-BBJ Biorepository, and stored at 4°C at the respective institutions until they are handed over to the sample transport/processing company (See "JCOG-Biobank Japan Biorepository protocol" for details).

2) Pathological specimens

Archived pathological tissues in daily clinical practice such as surgery, biopsy and laboratory tests can also be used in future translational researches, but the type of pathology specimen, preparation method and tissue quantity required by the studies are varied and there is no consensus that it is efficient to bank pathological tissues in a certain way prospectively. In addition, there is the opinion that the sample deteriorates (DNA fragmentation) when the thin-section sample from pathological tissue is stored for a long time.

Consent on the use of archival pathological tissue after medical care should therefore be obtained at the time of consent to banking, but actual collection should be initiated on an individual basis by creating a protocol and defining the most appropriate procedure for the study content in the protocol.

16. Organization

Changes to this chapter are considered to be revision rather than amendment.

16.1. Main study fund (funding source) of this study.

Practical Research for Innovative Cancer Control from Japan Agency for Medical Research and Development "Establishment of standard treatments for neuroendocrine carcinoma of the digestive system" JP15ck0106138, JP16ck0106138, JP17ck0106355, JP18ck0106355, JP19ck0106355, JP20ck0106618

National Cancer Center Research and Development Fund (23-A-22, 26-A-4, 29-A-3, 2020-J-3)

"Scientific research on multi-institutional trials to establish new standard treatment of solid tumors in adults"

16.2. Japan Clinical Oncology Group (JCOG)

JCOG is a multi-institutional clinical research group consisting of research teams funded by public research grants

mainly on National Cancer Center Research and Development Fund and Japan Agency for Medical Research and Development research costs that receive direct support for research by the Clinical Research Support Office of the National Cancer Center Hospital in accordance with JCOG Policy (http://www.jcog.jp/).

This study is conducted using JCOG research organisations and in accordance with the regulations set out by JCOG Executive Committee.

16.3. JCOG Chair

Yuichiro Ohe National Cancer Center Hospital

16.4. Study group and Group Chair

JCOG Hepatobiliary and Pancreatic Oncology Group
Group Chair: Junji FURUSE
Department of Medical Oncology, Kyorin University Faculty of Medicine
〒181-8611 6-20-2, Shinkawa Mitaka-shi, Tokyo
TEL:+81-422-47-5511
FAX:+81-422-44-1858
E-mail: jfuruse@ks.kyorin-u.ac.jp
Group Secretary: Hirohi Ishii
Clinical Research Center, Chiba Cancer Center

〒260-8717, 666-2 Nitona, 260-8717, Chuo-Ku, Chiba
TEL:+81-43-264-5431
FAX:+81-43-264-8680
E-mail: hirishii@chiba-cc.jp
JCOG Stomach Cancer Study Group:
Group Chair: Masanori Terashima
Shizuoka Cancer Center
〒411-8777 1007 Shimonagakubo, Nagaizumi-cho, Sunto-gun, Shizuoka Prefecture
TEL:+81-55-989-5222
FAX:+81-55-989-5783
E-mail: m.terashima@scchr.jp
Group Secretary: Takaki Yoshikawa
National Cancer Center Hospital
〒104-0045 5-1-1 Tsukiji, Chuo-ku, Tokyo
TEL:+81-3-3542-2511
FAX:+81-3-3542-3815
E-mail: tayoshik@ncc.go.jp
Group Secretary: Narikazu Boku
National Cancer Center Hospital
〒104-0045 5-1-1 Tsukiji, Chuo-ku, Tokyo
TEL:+81-3-3542-2511
FAX:+81-3-3542-3815
E-mail: nboku@ncc.go.jp

JCOG Japan Esophageal Oncology Group
Group Chair: Yuko Kitagawa
Keio University Hospital
〒160-8582 35 Shinanomachi, Shinjuku-ku, Tokyo
TEL:+81-3-3353-1211
FAX:+81-3-3355-4707
E-mail: kitagawa@a3.keio.jp

Group Secretary: Ken Kato
National Cancer Center Hospital
〒104-0045 5-1-1 Tsukiji, Chuo-ku, Tokyo
TEL:+81-3-3542-2511
FAX:+81-3-3542-3815
E-mail: kenkato@ncc.go.jp

16.5. Study Chair (Principal Investigator)

JCOG Hepatobiliary and Pancreatic Oncology Group
Takuji Okusaka
National Cancer Center Hospital
〒104-0045 5-1-1 Tsukiji, Chuo-ku, Tokyo
TEL: +81-3-3542-2511
FAX: +81-3-3542-3815
E-mail: tokusaka@ncc.go.jp

JCOG Stomach Cancer Study Group:
Narikazu Boku
National Cancer Center Hospital
〒104-0045 5-1-1 Tsukiji, Chuo-ku, Tokyo
TEL: +81-3-3542-2511
FAX: +81-3-3542-3815
E-mail:nboku@ncc.go.jp

JCOG Japan Esophageal Oncology Group
Ken Kato
National Cancer Center Hospital
〒104-0045 5-1-1 Tsukiji, Chuo-ku, Tokyo
TEL: +81-3-3542-2511
FAX: +81-3-3542-3815
E-mail: kenkato@ncc.go.jp

16.6. Study Coordinator

JCOG Hepatobiliary and Pancreatic Oncology Group
Chigusa Morizane
National Cancer Center Hospital
〒104-0045 5-1-1 Tsukiji, Chuo-ku, Tokyo
TEL: +81-3-3542-2511
FAX: +81-3-3542-3815
E-mail: cmorizan@ncc.go.jp

JCOG Stomach Cancer Study Group:
Nozomu Machida
Shizuoka Cancer Center
〒411-8777 1007 Shimonagakubo, Nagaizumi-cho, Sunto-gun, Shizuoka Prefecture
TEL:+81-55-989-5222
FAX:+81-55-989-5631
E-mail：no.machida@scchr.jp
JCOG Japan Esophageal Oncology Group
Ken Kato

National Cancer Center Hospital
〒104-0045 5-1-1 Tsukiji, Chuo-ku, Tokyo
TEL: +81-3-3542-2511
FAX: +81-3-3542-3815
E-mail: kenkato@ncc.go.jp

Yoshitaka Honma
National Cancer Center Hospital
〒104-0045 5-1-1 Tsukiji, Chuo-ku, Tokyo
TEL: +81-3-3542-2511
FAX: +81-3-3542-3815
E-mail:yohonma@ncc.go.jp

16.7. Central Pathological Review Coordinator

Nobuyoshi Hiraoka
National Cancer Center Hospital
〒104-0045 5-1-1 Tsukiji, Chuo-ku, Tokyo
TEL: +81-3-3542-2511
FAX: +81-3-3542-3815
E-mail: nhiraoka@ncc.go.jp

Shigeki Sekine
National Cancer Center
〒104-0045 5-1-1 Tsukiji, Chuo-ku, Tokyo
TEL: +81-3-3542-2511
FAX: +81-3-3542-3815
E-mail: ssekine@ncc.go.jp

16.8. Central Pathological Review Coordinator

Nobuyoshi Hiraoka (National Cancer Center)
Noriyoshi Fukushima (Jichi Medical University)
Nobuyuki Ohike (Showa University Fujigaoka Hospital)
Ryoji Kushima (Shiga University of Medical Science)
Mitsuya Iwafuchi (Scool of Health Sciences Faculty of Medicine, Niigata University)
Tetsuo Ushiku (The University of Tokyo)

16.9. Participating sites (participating institutions)

HBP	SCSG	JEOG	Participating institutions
○			Sapporo-Kosei General Hospital
○		○	Hokkaido University Hospital
	○		Keiyukai Sapporo Hospital
	○	○	Iwate Medical University
		○	Tohoku University Hospital
	○		Miyagi Cancer Center
○	○	○	Tochigi Cancer Center
○			Jichi Medical University
○	○	○	Saitama Cancer Center
○	○	○	National Cancer Center Hospital East
○		○	Chiba Cancer Center
○		○	Chiba University, Graduate School of Medicine
○	○	○	National Cancer Center Hospital
○			Kyorin University Faculty of Medicine
○			National Center for Global Health and Medicine (NCGM)
○	○	○	Cancer Institute Hospital of Japanese Foundation for Cancer Research
	○	○	Toranomon Hospital
○			Teikyo University School of Medicine
○			St.Marianna University School of Medicine
○	○	○	Kanagawa Cancer Center
○	○		Yokohama City University Medical Center
○	○		Niigata Cancer Center Hospital
○			Toyama University Hospital
○			Kanazawa University School of Medicine
	○		Ishikawa Prefectural Central Hospital
	○		Ogaki Municipal Hospital
○	○	○	Shizuoka Cancer Center
○	○	○	Aichi Cancer Center Hospital
		○	Kyoto University Hospital
	○	○	Osaka University Graduate School of Medicine
○	○		Kindai University Hospital
○	○	○	Osaka International Cancer Institute
○	○	○	National Hospital Organization Osaka National Hospital
	○	○	Osaka General Medical Center
	○	○	Osaka Medical and Pharmaceutical University
	○		Osaka Rosai Hospital
○	○	○	Kobe University Graduate School of Medicine
	○	○	Kansai Rosai Hospital
○	○		Hyogo College of Medicine
○	○	○	Hyogo Cancer Center
	○		Itami City Hospital
	○		Shimane University Faculty of Medicine
	○	○	Hiroshima University Hospital
	○		Fukuyama City Hospital
	○		Tokushima Red Cross Hospital
○	○	○	National Hospital Organization Shikoku Cancer Center
○		○	Kochi Health Sciences Center
○		○	National Kyushu Cancer Center
○		○	Kyushu University Hospital

	◯	◯	Oita University Faculty of Medicine

HBP: Hepatobiliary and Pancreatic Oncology Group
SCSG: Stomach Cancer Study Group:
JEOG: Japan Esophageal Oncology Group

16.10. JCOG Protocol Review Committee

This protocol was approved by JCOG Protocol Review Committee prior to submit to Certified Review Board.
(For membership, see website http://www.jcog.jp/basic/org/committee/protocol.html)
Contact: Protocol Review Committee Office

JCOG Operations Office/Clinical Research Support Office, National Cancer Center Hospital
〒104-0045 5-1-1, Tsukiji, Chuo-ku, Tokyo
TEL: 03-3542-2511 (ext. 2302)
FAX:03-3542-7006
E-mail: jcogoffice@ml.jcog.jp

16.11. JCOG Data and Safety Monitoring Committee

During study period, the study will be monitored by Data and Safety Monitoring Committee (e.g., adverse event reports, interim analysis reviews, monitoring report reviews, protocol revision reviews). However, the committee members from the study group conducting this study do not participate directly in the review of this study.
(For membership, see website http://www.jcog.jp/basic/org/committee/jury.html)
Contact: JCOG Data and Safety Monitoring Committee Office

JCOG Operations Office/Clinical Research Support Office, National Cancer Center Hospital
〒104-0045 5-1-1, Tsukiji, Chuo-ku, Tokyo
TEL:03-3542-2511 (ext. 2403)
FAX:03-3542-7006
E-mail: jcogoffice@ml.jcog.jp

16.12. JCOG Audit Committee

Site-visit audits by Audit Committee will be conducted during study period.
(For membership, see website http://www.jcog.jp/basic/org/committee/audit.html)
Contact: JCOG Auditing Committee Office

JCOG Operations Office/Clinical Research Support Office, National Cancer Center Hospital
〒104-0045 5-1-1, Tsukiji, Chuo-ku, Tokyo
TEL:03-3542-2511 (ext. 2403)
FAX:03-3542-7006
E-mail: jcogoffice@ml.jcog.jp

16.13. JCOG Conflict of Interest Committee

During study period, JCOG investigators involved in this study will be managed by the Conflict of Interest Committee.
(For membership, see website http://www.jcog.jp/basic/org/committee/coi.html)
Contact: JCOG Conflict of Interest Committee Office

JCOG Operations Office/Clinical Research Support Office, National Cancer Center Hospital
〒104-0045 5-1-1, Tsukiji, Chuo-ku, Tokyo
TEL:03-3542-2511 (ext. 2404)
FAX:03-3547-1002
E-mail: jcogoffice@ml.jcog.jp

16.14. Data Center/Operations Office

JCOG Data Center

Director of Data Center: Haruhiko Fukuda
Clinical Research Support Office, National Cancer Center Hospital

〒104-0045 5-1-1, Tsukiji, Chuo-ku, Tokyo
TEL:03-3542-3373
FAX:03-3542-3374
E-mail: jcogdata@ml.jcog.jp

JCOG Operations Office
Director of Operations Office: Kenichi Nakamura
Clinical Research Support Office, National Cancer Center Hospital
〒104-0045 5-1-1, Tsukiji, Chuo-ku, Tokyo
TEL:03-3547-1002
FAX:03-3547-1002
E-mail: jcogoffice@ml.jcog.jp

Official website: http://www.jcog.jp/

16.14.1. Data management administrator

Data management organization		JCOG Data Center
Data management administrator	Name	Harumi Kaba
	e-Rad number	40543442
	Affiliation	Clinical Research Support Office, National Cancer Center Hospital
	Title	Head of Multi-institutional Data Management Section

16.14.2. Monitoring administrator

Monitoring organization		JCOG Data Center
Monitoring administrator	Name	Haruhiko Fukuda
	e-Rad number	70263390
	Affiliation	Clinical Research Support Office, National Cancer Center Hospital
	Title	Chief of Data Management Division

16.14.3. Site-visit audit administrator

Auditing organization		JCOG Operations Office
Site-visit audit administrator	Name	Kenichi Nakamura
	e-Rad number	40543533
	Affiliation	Clinical Research Support Office, National Cancer Center Hospital
	Title	Director of Research Management Division

16.14.4. Statistical analysis administrator

Statistical analysis organization		JCOG Data Center
Statistical analysis Administrator	Name	Junki Mizusawa
	e-Rad number	60706646
	Affiliation	Biostatistics Division, Center for Research Administration and Support, National Cancer Center/ Biostatistics Section, Research Management Division, Clinical Research Support Office, National Cancer Center Hospital
	Title	Biostatistics Section Head

16.14.5. Research and development plan support personnel

Organizations in charge of supporting research and development plans		JCOG Operations Office
Research and development plan support personnel	Name	Tomoko Kataoka
	e-Rad number	70569863
	Affiliation	Clinical Research Support Office, National Cancer Center Hospital
	Title	Medical officer

16.14.6. Coordination management practitioner

Organizations in charge of coordination and management		JCOG Operations Office
Coordinating and managing practitioners	Name	Junko Eba
	e-Rad number	80754085
	Affiliation	Clinical Research Support Office, National Cancer Center Hospital
	Title	Medical officer

16.14.7. Personnel who oversees the study other than Principal Investigator and site investigators

Personnel who oversees the study other than Principal Investigator and site investigators	Name	Not applicable	
	e-Rad number		
	Affiliation		
	Title		
	Relevance of Secondary Sponsor	□ Applicable	□ Not applicable

16.14.8. Study group personnel

JCOG Data Center

Statistics Section Gakuto OGAWA

Data Management Section Kyoko HASEGAWA

JCOG Operations Office

Science Section Tomoko KATAOKA/Tadayoshi HASHIMOTO

In addition, JCOG Data Center/Operations Office commissioned some of theire research support activities (such as support for the preparation of study protocols, data management, and site-visit audits) to other corporations. The commissioned duties are supervised by the National Cancer Center through routine work, as well as by receiving work reports from the institution and providing supervisory guidance. The current consignee is as follows

Clinical Oncology Research and Education, a specified non-profit organization

〒104-0061 DJ Ginza Building 7F, 8-18-3, Ginza, Chuo-ku, Tokyo

Official website http://www.core.or.jp/

16.15. Developing a study protocol

Chigusa MORIZANE, National Cancer Center Hospital

Nozomu MACHIDA, Shizuoka Cancer Center

Yoshitaka HONMA/Ken KATO, National Cancer Center Hospital

Support for protocol development

JCOG Data Center

Statistics Section (in charge of study design) Junki MIZUSAWA

Data Management Section (CRF preparation) Harumi KABA

JCOG Operations Office

Protocol development Hiroshi KATAYAMA/Kozo KATAOKA/Aya MIURA

Person in charge of IC documents Aya KIMURA/Noriko TSUJI

17. Publication of the study results and completion of the study

17.1. Paper and conference presentations

Primary publication will be published in English journals.

Paper publication including review article and conference presentation of Introduction of the study, by Principal Investigator or Study Coordinator, which does not include the analytical results of the endpoint of the study, are allowed when Group Chair and JCOG Data Center Director agree to them. Publication of the distribution of baseline factors or the safety data after the end of accrual are also allowed. No publication other than primary analysis and final analysis will be performed unless previously approved by Data and Safety Monitoring Committee.

In principle, the authors of the main published papers on the results of the study (the first publication of the results of primary endpoint) shall be the first Study Coordinator (HBPOG), followed by Study Coordinator (JEOG or SCSG), Study Coordinator (JEOG or SCSG), Principal Investigator (HBPOG), Principal Investigator (JEOG or SCSG), Principal Investigator (JEOG or SCSG), the statistical staff of Data Center (one statistician in charge at the time of the analysis for publication), Centralized Pathological diagnosis Coordinator (HBP), Centralized Pathological diagnosis Coordinator (GI). In accordance with the limitations imposed by the article's posting provisions, researchers who contributed in descending order of their number of patients registrations were selected for each institution as co-authors, and the last author was Group Chair (or Study Chair). The inclusion of staffs of JCOG Operations Office as co-authors will be determined by Group Chair depending on their contributions. Authors of articles other than the primary publication (e.g., Secondary endpoints articles, secondary analysis articles) will be determined by Study Chair with Group Chair approval.

All co-authors will review the article contents prior to submission for publication and only those who agree to the article contents. If there is no consensus on the contents, principle investigator may not include the investigator in the co-authorship with the approval of Group Chair. If there is no consensus between groups and JCOG Data Center/Operations Office, ultimately follow JCOG Chair instructions.

17.2. Primary Endpoint Report and Clinical Summary Report

The procedures are specified from the preparation of the primary endpoint report and clinical summary report. If primary analysis is the final analysis, the primary endpoint report will not be prepared and the clinical summary report will be prepared.

17.2.1. Clinical Summary Report

Based on the final analysis report, Principal Investigator will prepare a "Abstract of the Clinical Summary Report" containing subjects background information (age, sex, etc.), study design and study progress, results of analyses for each endpoint, conclusions of the entire study, interpretations and discussion of the results, etc. within 6 months from the issue date of the final analysis report, submit it to the Data Center, and undergo review. In addition, the issue date of the final analysis report shall be "the date when the period for collecting data on all endpoints is completed" as specified in the Enforcement Regulations.

After obtaining approval from Group Chair and the Director of the Data Center, abstract of the clinical summary report will be submitted to Certified Review Board review as a "clinical summary report" with the final analysis report within 1 year of the issue date of the final analysis report.

Principal Investigator/Study Coordinator will disclose abstract of the clinical summary report (Notification of Completion of Form 1 of Article 24 of Regulation) to jRCT within 1 month after obtaining Certified Review Board approval (if the report is unpublished, abstract will not be released in jRCT and will be published immediately after publication).

The approved abstract of the clinical summary report will be submitted to the administrators of each participating institution through the site investigators at each institution and to JCOG Chair. Abstract of the clinical summary report is available on JCOG website (http://www.JCOG.jp/).

The timing of distribution of the final analysis report to participating institutions will be determined by Principal Investigator/Study Coordinator considering the timing of publication of the final analysis results, and the final analysis report will be distributed to the researchers at participating institutions by themselves or through the Data Center.

17.3. Completion of the study

On the date that abstract of the clinical summary report was released to iRCT, the study is completed.

In institutions where patients were not enrolled, the institution may be withdrawn from the institution list by submitting a request for change (Unified Form 3 for Clinical Research) and a Notification of Changes in Protocol (Form 2) stating that Principal Investigator withdraws the institution from the institution list in question to Certified Review Board, and after obtaining approval, notifying the MHLW (Local Health Bureau responsible for the location of Certified Review Board). When a protocol change notification is received by the Local Health Service, the investigator at the institution shall report the withdrawal to the Administrator of the institution.

18. References

omit

19. Appendix

- Informed consent form
- Body surface area table
- Toxicity Criteria (CTCAE v5.0-JCOG)
- CRF samples* (CRF draft attached for the first review submission)
- JCOG-Biobank Japan Biorepository Protocol
- JCOG-Biobank Japan Biorepository Informed Consent Form

Japan Clinical Oncology Group

Hepatobiliary and Pancreatic Oncology Group/Stomach Cancer Study Group/Japan Esophageal Oncology Group

Practical Research for Innovative Cancer Control from Japan Agency for Medical Research and Development
“Establishment of standard treatments for neuroendocrine carcinoma of the digestive system”
National Cancer Center Research and Development Fund 2020-J-3
“Scientific research on multi-institutional trials to establish new standard treatment of solid tumors in adults”

JCOG1213

Randomized phase III study of etoposide plus cisplatin combination therapy versus irinotecan plus cisplatin combination therapy in advanced neuroendocrine carcinoma of the digestive system. ver.1.5.0
TOPIC-NEC

HBPOG
Group Chair: Junji FURUSE
Department of Medical Oncology, Kyorin University Faculty of Medicine

Study Chair: Takuji OKUSAKA
Department of Hepatobiliary and Pancreatic Oncology, National Cancer Center Hospital 5-1-1 Tsukiji, Chuo-ku, Tokyo 104-0045 Japan
Tel: +81-3-3542-2511
E -mail：tokusaka@ncc.go.jp

Study Coordinator: Chigusa MORIZANE
Department of Hepatobiliary and Pancreatic Oncology, National Cancer Center Hospital 5-1-1 Tsukiji, Chuo-ku, Tokyo 104-0045 Japan
Tel: +81-3-3542-2511
E -mail：cmorizan@ncc.go.jp

SCSG
Group Chair: Masanori TERASHIMA
Division of Gastric Surgery, Shizuoka Cancer Center Hospital

Study Chair: Narikazu BOKU
Department of Gastrointestinal Oncology, National Cancer Center Hospital
5-1-1 Tsukiji, Chuo-ku, Tokyo 104-0045 Japan
Tel: +81-3-3542-2511
E -mail： nboku@ncc.go.jp

Study Coordinator: Nozomu MACHIDA
Department of Hepatobiliary and Pancreatic Oncology, Shizuoka Cancer Center
1007 Shimonagakubo, Nagaizumi-cho, Sunto-gun, Shizuoka Prefecture 411-8777 Japan
Tel: +81-55-989-5222
E -mail：no.machida@scchr.jp

JEOG
Group Chair: Yuko KITAGAWA
General and Gastroenterological Surgery, Keio University Hospital

Study Chair: Ken KATO
Department of Gastrointestinal Oncology, National Cancer Center Hospital 5-1-1 Tsukiji, Chuo-ku, Tokyo 104-0045 Japan
Tel: +81-3-3542-2511
E -mail：kenkato@ncc.go.jp

Study Coordinator: Yoshitaka HONMA/Ken KATO
Department of Gastrointestinal Oncology, National Cancer Center Hospital 5-1-1 Tsukiji, Chuo-ku, Tokyo 104-0045 Japan
Tel: +81-3-3542-2511
E-mail:yohonma@ncc.go.jp /kenkato@ncc.go.jp

2013/3/16	Protocol concept approved by JCOG Executive Committee (PC1213/1214/1215)
2014/6/27	Protocol approved byJCOG Protocol Review Committee
2017/5/23	Revision ver. 1.1 approved by JCOG Data and Safety Monitring Committee
2018/8/16	Revision ver. 1.2 approved by JCOG Data and Safety Monitring Committee
2018/10/11	Approved by Certified Review Board of National Cancer Center Hospital
2019/5/27	Revision ver. 1.3.0. approved by JCOG Data and Safety Monitring Committee
2019/6/27	Approved by Certified Review Board of National Cancer Center Hospital
2020/1/24	Revision ver. 1.4.0. approved by JCOG Data and Safety Monitring Committee
2020/2/27	Approved by Certified Review Board of National Cancer Center Hospital
2020/7/17	Revision ver. 1.5.0. approved by JCOG Data and Safety Monitring Committee
2020/9/24	Approved by Certified Review Board of National Cancer Center Hospital

0. Summary

This study is conducted as a "specified clinical trial" based on the Clinical Trials Act (Act No. 16 of April 14, 2017). In this protocol, the Principal Investigator is the Study Chair in the Hepatobiliary and Pancreatic Oncology Group of JCOG.

Name of study: "Randomized phase III study of etoposide plus cisplatin combination therapy versus irinotecan plus cisplatin combination therapy in advanced neuroendocrine carcinoma of the digestive system. (TOPIC-NEC) ".

Public study title: "Randomized phase III study of etoposide plus cisplatin combination therapy versus irinotecan plus cisplatin combination therapy in advanced neuroendocrine carcinoma of the digestive system. (TOPIC-NEC) "

0.1. Schema

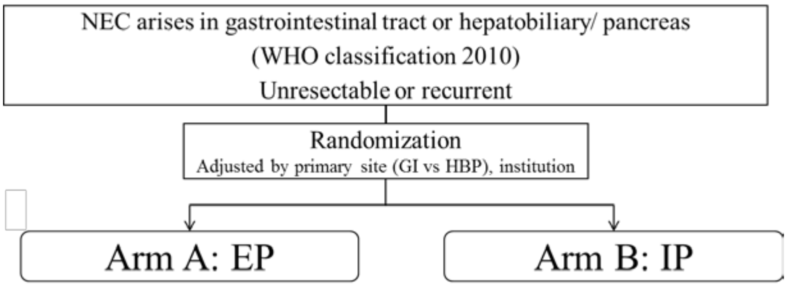

0.2. Objectives

A randomized phase III study was conducted to determine the better treatment option between etoposide/cisplatin combined therapy (EP therapy) or irinotecan/cisplatin combined therapy (IP therapy), both of which are standard treatments for non-resectable/recurrent neuroendocrine carcinoma (NEC as classified by WHO in 2010) with primary lesions in the gastrointestinal tract/hepatobiliary pancreatic organs.

Primary endpoint: Overall survival

Secondary endpoints: Response rate in case of measurable lesions, Progression-free survival (PFS), incidence rate of adverse events, dose intensity of Cisplatin, and incidence rate of serious adverse events.

0.3. Subjects

1) Any of the following is applicable based on pathological diagnosis taking findings of immunohistochemistry into consideration (see 3.1. to 3.3.).

[1] Pathologically diagnosed as neuroendocrine carcinoma (NEC*1) in the resected sample.

[2] Containing pathologically confirmed component of neuroendocrine carcinoma (NEC*1) in the biopsy sample.

1: Based on WHO 2010 classification

2) Any of the following is applicable

[1] NEC arise in esophagus, stomach, duodenum, intestine, appendix, colon, rectum, gallbladder, intrahepatic bile duct, extrahepatic bile duct, ampulla of Vater, pancreas,

[2] Liver NEC (primary liver or liver metastasis of unknown primary) *2.

*2: The tumor is only in one of the following sites after a thorough examination of the primary site by contrast CT (from the neck to pelvic) and upper/lower gastrointestinal endoscopy, FDG-PET scan, otolaryngology (head and neck) examination, urology examination (male patients only), and gynecology examination (female patients only).

a. Liver only

b. Bone, lymph nodes below the diaphragm, peritoneum, subcutaneous, muscle, spleen, and liver

3) Unresectable (see 3.6) or recurrent cancer (see 3.7). It is not essential for a pathological confirmation of the metastatic lesion or recurrent site. Cases of esophageal NEC is ineligible if corresponding to any of the following.

[1] cT4.

[2] No distant metastasis rather than supraclavicular lymph node

[3] Stenosis indicated for palliative radiotherapy

4) No previous chemotherapy or radiotherapy for NEC. Pre- or post-operative chemotherapy except irinotecan or

etoposide for NEC is allowed as long as it was completed at least 8 weeks prior to registration.

5) No previous chemotherapy using platinum agents for any malignancies.

6) No serious tumor-related complications.

Serious tumor-related complications include superior vena cava syndrome, inferior vena cava syndrome, pleural, ascites, or pericardial effusions that are large or uncontrollable (e.g., effusions that accumulate quickly after drainage or even after adhesive surgery), and brain metastases with neurological symptoms.

7) Aged 20 to 75 years old.

8) ECOG performance status of 0 or 1.

9) Sufficient oral intake

10) Measurable region is not required.

11) Adequate organ functions.

[1] WBC ≥3,000/ mm^3

[2] Neutrophils ≥ 1,500/ mm^3

[3] Hemoglobin ≥ 9.0 g/dL

[4] Platelets ≥ 10×10^4/ mm^3

[5] Total bilirubin≤ 1.5 mg/dL※4

[6] AST(sGOT)≦100 IU/L※4（for hepatic NEC and liver metastasis, ≦150 IU/L）

[7] ALT(sGPT)≦100 IU/L※4（for hepatic NEC and liver metastasis, ≦150 IU/L）

※4：Presence or absence of biliary drainage is not relevant

[8] Serum creatinine≦ 1.3 mg/dL

[9] Creatinine clearance※5≥ 60 mL/min

※5： Creatinine clearance must have been estimated using the Cockcroft-Gault formula, and must be 60 mL/min or more. If the estimation is less than 60 mL/min, but the actual measurement is 60 mL/min or more, the patient can be deemed eligible.

Cockcroft-Gault formula

Male：Ccr ={(140－age) × body weight (kg)}/{72×serum creatinine (mg/dL)}

Female：Ccr = 0.85×{(140－age) × body weight (kg)}/{72×serum creatinine(mg/dL)}

12) Written informed consent.

0.4. Treatments

Arm A: etoposide plus cisplatin (EP) arm

The following chemotherapy is continued until the patient meets discontinuation criteria, with 3-weeks of treatments counting as one cycle.

Drug	Dosage (mg/m^2)	Dosing regimen/Dosing time	Dose day
Etoposide	100	IV/60-120 min	Day 1, 2, 3
Cisplatin	80	IV/60-120 min	Day 1

Arm B: Irinotecan plus Cisplatin (IP) arm

The following chemotherapy would be continued until the patient meets discontinuation criteria, with 4-weeks of treatments counting as one cycle.

Drug	Dose (mg/m^2)	Dosing regimen/Dosing time	Dose day
Irinotecan	60	IV/90 min	Day 1, 8, 15
Cisplatin	60	IV/60-120 min	Day 1

0.5. Planned sample size and study period

The planned sample size is 170.

Accrual period: 6 years.

Follow-up period: 2 years after accrual completion.

Analysis period: 1 year.

Total study duration 9 years.

Primary analysis: 9 years after accrual completion.

Scheduled start date of the study	Aug 8, 2014
Expected completion date of the study	Aug 8, 2023

0.6. Contact information

Eligibility criteria, treatment modification, and other issues requiring clinical decisions: Study Coordinator (front cover and 16.6.)

Enrollment procedure protocol, case report form (CRF) entries, etc.: JCOG Data Center, (16.14.)

Adverse event reporting: JCOG Data and Safety Monitoring Committee (16.11.),

Contents

0. SUMMARY 2

0.1. SCHEMA 2
0.2. OBJECTIVES 2
0.3. SUBJECTS 2
0.4. TREATMENTS 3
0.5. PLANNED SAMPLE SIZE AND STUDY PERIOD 3
0.6. CONTACT INFORMATION 4

1. OBJECTIVES 8

2. BACKGROUND 8

2.1. TARGET 8
2.2. STANDARD TREATMENT FOR TARGET DISEASE 16
2.3. RATIONALE FOR ESTABLISHMENT OF TREATMENT PLAN 18
2.4. STUDY DESIGN 21
2.5. SUMMARY OF EXPECTED ADVANTAGES AND DISADVANTAGES ASSOCIATED WITH STUDY PARTICIPATION 22
2.6. SIGNIFICANCE OF THIS STUDY 23
2.7. ASSOCIATED RESEARCH (INCLUDING SAMPLE ANALYSIS RESEARCH) 23
2.8. JCOG-BIOBANK JAPAN (BBJ) COLLABORATING BIOBANK 23

3. CRITERIA/DEFINITIONS USED IN THIS STUDY 25

3.1. TISSUE CLASSIFICATION (WHO 2010 CLASSIFICATION) 25
3.2. GRADE CLASSIFICATION （ENETS [EUROPEAN NEUROENDOCRINE TUMOR SOCIETY] / WHO2010 CLASSIFICATION） 25
3.3. HISTOPATHOLOGICAL DIAGNOSIS 25
3.4. DISEASE STAGE CLASSIFICATION CRITERIA (UICC-TNM 7TH EDITION) 25
3.5. RESIDUAL TUMOR (R) CLASSIFICATION (UICC-TNM 7TH EDITION) 33
3.6. DEFINITION OF NON-RESECTABLE NEC 33
3.7. DEFINITION OF RECURRENT NEC 34
3.8. DEFINITION OF HEPATIC NEC (HEPATIC PRIMARY LESION OR LIVER METASTASIS FROM AN UNKNOWN PRIMARY LESION) 34

4. PATIENT SELECTION CRITERIA 34

4.1. INCLUSION CRITERIA (FOR ENROLLMENT) 34
4.2. EXCLUSION CRITERIA 35

5. REGISTRATION AND RANDOMIZATION 36

5.1. PROCEDURE OF REGISTRATION 36
5.2. RANDOMIZATION AND ALLOCATION ADJUSTMENT FACTOR 37

6. TREATMENT PLAN AND TREATMENT MODIFICATION CRITERIA 38

6.1. PROTOCOL TREATMENT 38
6.2. PROTOCOL TREATMENT TERMINATION/COMPLETION CRITERIA 41
6.3. TREATMENT MODIFICATION CRITERIA 42
6.4. CONCOMITANT TREATMENT AND SUPPORTIVE CARE 48
6.5. POST-STUDY TREATMENT 55

7. ANTICIPATED ADVERSE EVENTS 56

7.1. ANTICIPATED ADVERSE REACTIONS 56

7.2. ANTICIPATED ADVERSE EVENTS DUE TO PATHOGENESIS 59
7.3. EVALUATION OF ADVERSE EVENTS/REACTIONS 60

8. EXAMINATION AND EVALUATION 62

8.1. BASELINE EXAMINATION AND EVALUATION BEFORE REGISTRATION 62
8.2. EXAMINATION AND EVALUATION DURING TREATMENT 63
8.3. EXAMINATION AND EVALUATION AFTER COMPLETION OF TREATMENT 64
8.4. INFORMATION ON POST- STUDY TREATMENT 64
8.5. STUDY CALENDAR 65

9. DATA COLLECTION 69

9.1. CASE REPORT FORM (CRF) 69

10. REPORTING OF "DISEASE OR THE LIKE"(ADVERSE EVENTS) 70

10.1. SERIOUS ADVERSE EVENTS AND SUBJECTS OF EXPEDITED REPORTING. 70
10.2. INVESTIGATOR'S REPORTING REQUIREMENTS AND PROCEDURES 71
10.3. RESPONSIBILITIES OF PRINCIPAL INVESTIGATOR/STUDY COORDINATOR 73
10.4. RESPONSIBILITIES OF THE SITE INVESTIGATORS AT THE PARTICIPATING INSTITUTIONS (INCLUDING THE RELEVANT INSTITUTION) 75
10.5. RESPONSIBILITIES OF THE DATA AND SAFETY MONITORING COMMITTEE 75

11. RESPONSE EVALUATION AND ENDPOINT DEFINITION 76

11.1. RESPONSE ASSESSMENT (ONLY FOR PATIENTS WITH MEASURABLE DISEASE) 76
11.2. DEFINITIONS OF ANALYSES SET 82
11.3. DEFINITION OF ENDPOINTS 82

12. STATISTICAL CONSIDERATION 85

12.1. PRINCIPAL ANALYSIS AND DECISION CRITERIA 85
12.2. PLANNED ACCRUAL, ACCRUAL PERIOD, AND FOLLOW-UP PERIODS 86
12.3. INTERIM ANALYSIS AND EARLY TERMINATION OF THE STUDY 87
12.4. ANALYSIS OF SECONDARY ENDPOINTS 88
12.5. FINAL ANALYSIS 88
12.6. EXPLORATORY ANALYSIS 89
12.7. PREMATURE WITHDRAWAL FROM THE TRIAL 89
12.8. PROCEDURES AFTER EARLY TERMINATION OF THE STUDY 90

13. ETHICAL CONSIDERATIONS 92

13.1. PROTECTION OF HUMAN SUBJECTS 92
13.2. INFORMED CONSENT 92
13.3. PROTECTION OF PERSONAL INFORMATION AND PATIENT IDENTIFICATION 94
13.4. ADHERENCE TO THE PROTOCOL 96
13.5. APPLICATION TO CERTIFIED REVIEW BOARD AND NOTIFICATION OF IMPLEMENTATION PLANS 96
13.6. PROTOCOL REVISION/AMENDMENT 100
13.7. CONFLICTS OF INTEREST (COIS) INVOLVED IN THIS STUDY 102
13.8. COMPENSATION 104
13.9. INTELLECTUAL PROPERTY 105
13.10. DISCLOSURE OF INFORMATION ON THIS STUDY 105

14. MONITORING AND AUDIT 106

14.1. PERIODIC MONITORING 106
14.2. SITE VISIT AUDITS 108

14.3. MANAGEMENT OF NON-COMPLIANCE 109

15. SPECIAL INSTRUCTIONS 110

15.2. JCOG BIOBANK JAPAN (BBJ) BIOREPOSITORY 111

16. ORGANIZATION 111

16.1. MAIN STUDY FUND (FUNDING SOURCE) OF THIS STUDY. 111
16.2. JAPAN CLINICAL ONCOLOGY GROUP (JCOG) 111
16.3. JCOG CHAIR 112
16.4. STUDY GROUP AND GROUP CHAIR 112
16.5. STUDY CHAIR (PRINCIPAL INVESTIGATOR) 113
16.6. STUDY COORDINATOR 113
16.7. CENTRAL PATHOLOGICAL REVIEW COORDINATOR 114
16.8. CENTRAL PATHOLOGICAL REVIEW COORDINATOR 114
16.9. PARTICIPATING SITES (PARTICIPATING INSTITUTIONS) 115
16.10. JCOG PROTOCOL REVIEW COMMITTEE 116
16.11. JCOG DATA AND SAFETY MONITORING COMMITTEE 116
16.12. JCOG AUDIT COMMITTEE 116
16.13. JCOG CONFLICT OF INTEREST COMMITTEE 116
16.14. DATA CENTER/OPERATIONS OFFICE 116
16.15. DEVELOPING A STUDY PROTOCOL 118

17. PUBLICATION OF THE STUDY RESULTS AND COMPLETION OF THE STUDY 119

17.1. PAPER AND CONFERENCE PRESENTATIONS 119
17.2. PRIMARY ENDPOINT REPORT AND CLINICAL SUMMARY REPORT 119
17.3. COMPLETION OF THE STUDY 120

18. REFERENCES 121

19. APPENDIX 121

1. Objectives

A randomized phase III study was conducted to determine the better treatment option between etoposide/cisplatin combined therapy (EP therapy) or irinotecan/cisplatin combined therapy (IP therapy), both of which are standard treatments for non-resectable/recurrent neuroendocrine carcinoma (NEC as classified by WHO in 2010) with primary lesions in the gastrointestinal tract/hepatobiliary pancreatic organs.

Primary endpoint: Overall survival

Secondary endpoints: Response rate in case of measurable lesions
Progression-free survival (PFS), incidence rate of adverse events, dose intensity of Cisplatin, and incidence rate of serious adverse events

2. Background

2.1. Target

2.1.1. Epidemiology

Neuroendocrine tumors (NETs) and neuroendocrine carcinomas (NECs) can arise in various organs in the body. Of these, NECs with primary lesions occurring in the lungs, namely small cell lung cancer and large cell lung cancer, have been collectively referred to as high-grade NECs (HGNECs). However, terms such as extrapulmonary small cell carcinoma and extrapulmonary NEC have been used to refer collectively to NECs, with primary lesions outside the lungs.

The number of newly reported cases of neuroendocrine neoplasms (NENs) annually, estimated based on the cases registered in the U.S. SEER (Surveillance, Epidemiology, and End Results) database between 1973 to 2004 is 5 in 100,000 people[1]. In Japan, the Neuroendocrine Tumor Workshop Japan (NET Work Japan) carried out a nationwide survey of pancreatic and gastrointestinal NENs, and estimated the number of new cases in 2005 at approximately 1.01 people with pancreatic primary lesion per 100,000 people, and 2.10 people with gastrointestinal primary lesion per 100,000 people[2, 3].

The report by the PRONET Study Group in France concerning their prospective observational study is a good reference for the proportion of NENs that can be classified as NECs. Out of the 778 patients diagnosed with gastrointestinal primary lesion NENs in 80 facilities from August 2010 to July 2011, 104 patients (13.4%) had NEC[4].

The frequency of incidence of NECs according to the primary lesion organ has been shown in the tables below (Table 2.1.1a and Table 2.1.1b). Despite variations between reports, gastrointestinal primary lesion NECs account for approximately 20–68% of all extrapulmonary NEC cases. The breakdown of gastrointestinal primary lesion NEC cases with primary lesion in the gastrointestinal tract (esophagus, duodenum, small and large intestines) indicated that these cases accounted for 56–84% of the total cases, while hepatobiliary pancreatic cancer cases accounted for 15–35% of all cases.

Table 2.1.1a. Frequency of extrapulmonary NECs according to the organ of primary lesion

Reported year/Reporter	N	Gastrointestinal	Gynecologal organs	Urology	Head and Neck	Adrenal	Primary unknown	Other
2012/Terashima [5]	136	68%	12%	6%	NA	NA	8%	7%
2010/Brennan [6]	74	20%	35%	15%	19%	NA	9%	1%
2009/Wong [7]	1618	33%	NA	20%	11%	10%	4%	22%
2006/Haider [8]	101	20%	11%	18%	12%	9%	31%	NA
2007/Lee [9]	61	56%	20%	10%	8%	NA	6%	NA

Table 2.1.1b. Percentage breakdown of NECs as per the primary lesion organ of gastrointestinal NEC

Reported year/Reporter	N	Esophageal	Gastric	Duodenal/small intestine	Colorectal	Hepatobiliary	Pancreas	Other
2012/Machida [10]	258	33%	27%	2%	12%	12%	14%	NA
2012/Sorbye [11)]	205	6%	10%	NA	40%	NA	35%	10%
2004/Brenner [12]	544	53%	11%	0.2%	20%	11%	4%	NA

2.1.2. Clinical pathology

1) About the term of target disease

The target of this study has a complex disease concept, and since the method of classification has changed with time, various names have been assigned that could cause confusion. In this study, we use classification and nomenclature defined according to the WHO 2010 classification（WHO Classification of Tumors of the Digestive System 2010）[13].

All the tumors originating from neuroendocrine cells or differentiation into endocrine cells are called neuroendocrine neoplasms (NENs). Depending on the malignancy, NENs were classified as Grade 1 NETs (NET G1), Grade 2 NETs (NET G2), or NEC.

Furthermore, prior to the WHO 2010 classification, all disease concepts equivalent to NENs were called NETs, but in this study, we would have referred to all descriptions of the disease concept as NENs. Furthermore, according to the 2000 and 2004 WHO classifications, the term well-differentiated NEC (WDNEC) has been used for disease units equivalent to NET G2, but in this study NEC does not mean the same.

2) Classification and clinical pathology

NEN is a tumor that develops from neuroendocrine cells present in various tissues or shows a tendency of differentiation into neuroendocrine cells. Histologically these tumors express neuroendocrine markers, such as Chromogranin A, NSE (neuron-specific enolase), and synaptophysin. The WHO classifications in 2000 and 2004 combined the presence or absence of metastasis/local infiltration and cellular proliferative capacity (evaluated based on Ki-67 expression intensity and mitotic presentation), and thereby classified these tumors into well-differentiated (neuro)endocrine tumors without metastasis/local infiltration, well-differentiated (neuro)endocrine tumors with metastasis/local infiltration, and poorly differentiated (neuro)endocrine tumors with even greater cell proliferative capacity. On the other hand, European Neuroendocrine Tumor Society (ENETS) has proposed a method of classification that evaluates the malignancy as Grade 1, 2, or 3 (G1, G2, or G3), according to the cell proliferative capacity (Ki-67 index or number of mitotic presentations), which has proven to be most useful for classification of prognosis[14, 15]。

Against this backdrop, the WHO classification that targeted gastrointestinal diseases was published in 2010. The disease as a whole was considered “neuroendocrine neoplasms (NEN)”, and the disease malignancy was largely classified as NET G1 (neuroendocrine tumor Grade 1), NET G2 (Grade 2), NEC, and mixed adenoneuroendocrine carcinoma (MANEC). MANEC refers to the cancers wherein adenocarcinoma components account for more than 30% of the cancer, while those under 30% are classified as NECs. However, even though this method of classification is applicable while evaluating the entire tumor based on resected specimen, it is not possible to evaluate the proportion of each component in the entire tumor during a diagnosis based on biopsy sample.

Each of NET G1, NET G2, and NECs described in the WHO 2010 classification is equivalent to G1, G2, and G3 as per the ENETS classification. NETs are well-differentiated tumors, with relatively low atypicality and malignancy, and the clinical course is slow with a 5-year survival in the range of 62–85%[16, 17]. On the other hand, NECs are poorly differentiated tumors, where tumor cells with poor cellular constituents proliferate diffusely, and are pathologically similar to small cell lung cancers, since these show many mitotic presentations, involve necrotic lesions, and present with neuroendocrinological features during immunohistochemical staining[1]. The clinical presentations are also similar in terms of the rapid proliferation of NECs and relatively high sensitivity to anticancer drugs[18].

Similar to lung cancer, NECs also exist as small cell cancer type or large cell type (LCNEC：large cell NEC）[13]. Future challenges include studies on the frequency and differences in clinical presentation of each disease. Table 2.1.2 shows the WHO and Grade classification.

Table 2.1.2. Changes in WHO classification and grading; Shaded parts are targets of this study

WHO 2000 classification	WHO 2010 classification		ENETS Grade
Well-differentiated endocrine tumor（WDNET）<br>1.1‘Benign’ behavior<br>1.2 Uncertain behavior	Neuroendocrine tumors, NETs<br>• Well-differentiated<br>• Composed of cells similar to normal gastrointestinal	NET G1（carcinoid）	G1<br>Same as 2010 WHO classification<br>Number of mitotic presentations < 2 per 10

	endocrine cells		high power fields（HPF） and/or Ki-67 index ≤2%
Well-differentiated endocrine carcinoma（WDEC）	• Expression of neuroendocrine markers<br>• Hormone production<br>• Mild to moderate nuclear atypia, low proliferative capacity（Grade：G1,G2）	NET G2	G2<br>Number of mitotic presentations 2–20 per 10 HPF and/or Ki-67 index 3-20%
Poorly differentiated endocrine carcinoma/small cell carcinoma（PDEC）	Neuroendocrine carcinoma：NEC（large cell or small cell type）:<br>• Poorly differentiated, highly malignant<br>• Include small cell to large cell type carcinoma<br>• Sometimes present tissue structure similar to NET<br>• Expression of neuroendocrine markers<br>• Significant nuclear atypia, multifocal necrosis, and high proliferative capacity (Grade: G3)		G3<br>Number of mitotic presentations >20 per 10 HPF and/or Ki-67 index > 20%
Mixed exocrine-endocrine carcinoma（MEEC）	Mixed adenoendocrine carcinoma（MANEC）		
Tumor-like lesions（TLL）	Hyperplastic and preneoplastic lesions		

2.1.3. Staging

Disease staging for NENs is represented by the TNM classification proposed by ENETS (hereafter, ENETS TNM[14, 15], AJCC 7th Edition TNM classification and UICC 7th Edition TNM classification published in 2009. The AJCC 7th Edition and UICC 7th Edition TNM classifications are unified, and in this study we would have used the UICC 7th Edition nomenclature).

In the UICC 7th edition, there are independent TNM classifications of NET G1 and NET G2 with primary lesions in the stomach, small intestine, appendix, and colon, but with respect to NEC, it describes "classification according to main tissue type in each organ of primary lesion (squamous cell carcinoma for esophagus, and adenocarcinoma in all other organs of primary lesion)." While there are TNM classifications for hepatocellular carcinoma and intrahepatic cholangiocarcinoma in the liver, there is no description for the classification of the tissue type for NECs with primary lesion in the liver. In this study, however, we would have used the TNM classification of intrahepatic cholangiocarcinoma, which shows similar clinical presentations. Details of each TNM classification would have been mentioned in “3.2 staging criteria”.

2.1.4. Standard treatment according to disease staging and outline of prognosis

1) Standard treatment for resectable cases

Surgical resection is performed for resectable cases. The significance of postoperative adjuvant chemotherapy is not clear because a verification study has not yet been performed. Although the NCCN Guidelines[19] describe postoperative adjuvant therapy as the chemotherapy regimen for small cell lung cancers that is typically applied to advanced cases of NEC, it has relatively high toxicity and patient burden (see Table 2.2.2, 2.3.2); hence we cannot claim that it is widely used in routine practice. For NECs with gastrointestinal primary lesion with mixed adenocarcinoma, a chemotherapy regimen is suggested typically after surgery for adenocarcinoma; however, in practice, there is no consensus on postoperative adjuvant chemotherapy.

2) Standard treatment for locally advanced cases

While the NCCN Guidelines propose chemoradiotherapy for small cell lung cancer for locally advanced cases, the ENETS Guidelines provide no clear indication[20, 21] for the same. As there is no sufficient information related to radiation dose, efficacy, and safety for NECs with a multitude of primary lesion organs (scope of irradiation), radiation therapy is not widely used for pathologies other than NEC with esophageal primary lesion that is prone to stenosis, and treatment is often provided for distant metastasis.

For locally advanced cases of NEC with esophageal primary lesion, chemoradiotherapy is actively performed. Furthermore, supraclavicular lymph node metastasis in thoracic esophageal tumor is classified as Stage IV, and chemoradiotherapy is applied if metastatic lymph nodes are included in the field of irradiation.

3) Standard treatment for distal metastasis cases

Systemic chemotherapy is indicated for distal metastasis and recurrent cases regardless of the organ, and a regimen is chosen for the small cell lung cancer. With respect to NECs with esophageal primary lesion, even if there is organ metastasis, palliative chemoradiotherapy is prioritized for patients incapable of oral intake of drugs due to esophageal constriction.

The efficacy of everolimus and sunitinib has been demonstrated for NETs with pancreatic primary lesions. Furthermore, Octreotide and Lantreotide effectively inhibit proliferation of NETs that originate in the midgut and pancreatic/gastrointestinal NETs, respectively. However, these results are from clinical trials conducted in patient populations corresponding to NETs. Since the clinical presentations of NETs and NECs differ significantly, these treatments cannot be indicated for NECs during routine practice.

4) Indication of resection for distal metastasis

There is no consensus on the resection criteria specific to the NECs. Since the progression of NETs is slow, resection is actively considered even where there is distal metastasis to the liver, but as NEC progresses fast, surgical resection cannot be indicated for cases with distal metastasis[19, 21].

5) Prognosis

In a preceding study, Yamaguchi and Machida carried out a multicenter joint observational study of poorly

differentiated NEC (WHO 2010 NEC, including patients with clinical diagnosis of poor differentiation) in a total of 23 facilities, including facilities participating in JCOG Hepatobiliary and Pancreatic Oncology Group, former Gastroenterology Group, and observer facilities.

In the multicenter joint study by Yamaguchi and Machida, 89% of cases (229/258 patients) receiving systemic chemotherapy had distal metastasis, with a median survival time (MST) of 11.5 months for all patients, 11.2 months for distal metastasis cases, and 15.9 months for locally advanced cases. MST according to organ was 13.4 months for esophageal primary lesion (N = 85), 13.3 months for gastric primary lesion (N = 70), 29.7 months for small intestine/duodenal primary lesion (N = 6), 7.6 months for colonic primary lesion (N = 31), 8.5 months for pancreatic primary lesion (N = 35), and 7.9 months for hepatobiliary primary lesions (N = 31) (Table 2.1.4). While there were 9 cases of five-year survival among all the gastrointestinal cases, there were no five-year survival cases amongst those with primary lesions in hepatobiliary and pancreatic organs[10]. In a multicenter joint observational study for NECs conducted in four Northern European countries (hereafter referred to as NORDIC NEC Study), the MST for NECs with gastrointestinal primary lesion (albeit including ~30% of NECs with primary lesion sites unknown) was 11 months[11]. According to the U.S. SEER database, the MST of NEC was 10 months[3]. Based on the above information, despite differences in the primary lesion organ, the prognosis of the target population of this study is thought to have an overall MST of 10–11 months.

Table 2.1.4. Treatment results according to primary lesion organ for non-resectable/recurrent NECs that underwent systemic chemotherapy

	Esophageal	Gastric	Duodenal/small intestine	Colonic	**Gastrointestinal overall**	Pancreatic	Hepatobiliary	**Hepatobiliary and pancreatic organs overall**
N	85	70	6	31	**192**	35	31	**66**
Response rate（%）	58%	43%	50%	29%	**47%**	17%	16%	**17%**
Median PFS（month）	5.8	4.9	7.3	3.7	**5.1**	3.2	4.1	**3.7**
MST（month）	13.4	13.3	29.7	7.6	**13**	7.9	8.5	**7.9**

2.1.5. Tumor-related complications

We, here, report the complications that require attention during patient management, according to the organ of primary lesion. It is a rare disease and frequencies remain unknown.

1) Esophageal primary

Tumor bleeding/pain/esophageal stenosis/fistula formation due to primary lesion in the esophagus; hoarseness/difficulty swallowing/aspiration pneumonia caused by recurrent laryngeal nerve palsy due to lesion in lymph node metastasis; jaundice and liver failure associated with liver metastasis; respiratory failure and hemoptysis associated with lung metastasis, airway constriction, and pain due to lymph node metastasis; hypercalcemia, tracheal stenosis, tracheal obstruction, and suture failure due to disease progression.

2) Gastric primary

Chronic bleeding from primary lesion, accompanied by anemia, stomach pain, nausea, vomiting, gastric perforation, bloating, suture failure, fistula formation, cardiac/pyloric stenosis, ascites retention due to peritoneal metastasis, ileus, hydronephrosis due to ureteral stenosis, obstructive jaundice/liver failure due to bile duct stenosis, and obstructive jaundice/liver failure due to hepatic portal lymph node metastasis.

3) Small intestine/colonic primary

Tumor hemorrhage, ileus, fistula formation in the small intestine/bladder/vagina, intestinal obstruction, intestinal stenosis, intestinal perforation, and pelvic infection

4) Hepatobiliary and pancreatic primary

Cancer pain, rupture, obstructive jaundice associated with the growth of primary tumor, weight loss, fever, pancreatitis, cholangitis, liver abscess, cholecystitis, biliary hemorrhage, duodenal hemorrhage, duodenal stenosis, anastomotic ulcer/stenosis/leakage, symptoms associated with stenosis/obstruction of portal vein (liver

dysfunction/liver failure, esophageal varices and their rupture, gastritis, ascites retention, and hepatic encephalopathy/coma associated with portal hypertension).

5) Paraneoplastic syndrome

In addition to NECs, there are reports of paraneoplastic syndrome that is often associated with small cell lung carcinoma[22-24]. We report examples of paraneoplastic syndrome seen with small cell lung carcinoma below.

Hyponatremia due to inappropriate sodium secretion, psychological symptoms (changes in personality) due to ectopic ACTH syndrome, hypertension, hypokalemia, high blood sugar, Lambert-Eaton myasthenic syndrome, paraneoplastic cerebellar degeneration associated with autoantibody production (ataxia in the limbs, dysarthria, and nystagmus), paraneoplastic encephalomyelitis/sensory neuron disease (dementia, cranial nerve symptoms, dizziness, ataxia, autonomic imbalance, transverse paralysis, and sensory disorders).

Apart from these, there are thromboembolic events, aspiration (due to gastrointestinal stenosis), anemia, tumor pain, acute renal failure, myositis, and pulmonary fibrosis.

6) Complications due to metastatic lesions

Liver failure, pain, hepatobiliary infection, hemorrhage, bile duct stenosis, bile duct obstruction, respiratory failure, tracheal hemorrhage, respiratory tract infection, atelectasis, airway constriction, ascites, bloating, ileus, ureteral stenosis, ureteral obstruction, urinary retention, urinary tract infection, hiccups, pleural effusion, pleural hemorrhage, chest pain, pathological fracture, pain, hypercalcemia, meningeal carcinomatosis, ataxia, ischemia cerebrovascular, intracranial hemorrhage, nausea, vomiting, dizziness, consciousness disorder, cognitive disturbance, dysphasia, seizure, spasticity, edema limbs, intestinal obstruction/stenosis/perforation, pancreatitis, disseminated intravascular coagulation, thrombocytopenia, anorectal infection, anal hemorrhage, hoarseness, and superior vena cava syndrome

7) Others

General pain management including narcotic analgesics is performed for cancer pain.

Gastrointestinal stenting and bypass surgery are performed for esophageal stenosis, cardiac/pyloric stenosis, and duodenal stenosis.

Percutaneous transhepatic cholangial drainage (PTCD), percutaneous or endoscopic stenting, and bile duct jejunostomy is performed for obstructive jaundice. In addition, complications associated with biliary drainage and gastrointestinal stenting are listed below.

- PTCD, including internal and external fistula tube placement:
  Cholangitis, pancreatitis, cholecystitis, liver abscess, sepsis, biliary hemorrhage, PTCD tube obstruction/deviation, peritonitis, and pneumothorax/pleurisy
- Biliary stenting:
  Cholangitis, pancreatitis, cholecystitis, liver abscess, sepsis, biliary hemorrhage, peritonitis, pneumothorax/pleurisy (for percutaneous stenting), duodenal perforation, pneumonia (for endoscopic stenting), stent obstruction/deviation, duodenal ulcer, and duodenal hemorrhage
- Bile duct jejunostomy:
  Cholangitis, pancreatitis, cholecystitis, liver abscess, and sepsis
- Gastrointestinal stenting:
  Hemorrhage, perforation, pain, stent deviation, stent obstruction, foreign-body sensation, intestinal strangulation, ulcer formation, fever, sepsis, infection, diarrhea, constipation, tenesmus or uncontrollable urination/incontinence symptoms (colon), thyroid injury (esophagus), carotid artery injury (esophagus), and mediastinal abscess (esophagus).

2.1.6. Recurrent/progression

NEC is a very rare disease, and most reports of cases of surgery coincide with the case reports. According to the review by Arai et al., out of the 55 patients with NEC with gastric primary lesion who underwent surgical resection, only three patients survived for two years or more[25]. According to the report by Fischer et al., the MST of 13 patients with NEC with pancreatic primary lesion who underwent surgical resection was 11.7 months (seven of the patients showed distal metastasis)[26]. The form of recurrence among surgery cases remains unknown. The multicenter joint

study by Yamaguchi and Machida reported PFS among cases that underwent systemic chemotherapy with respect to the median as 5.8 months for esophageal primary lesions, 4.8 months for gastric primary lesions, 7.3 months for duodenal small intestine primary lesions, 3.7 months for colonic primary lesions, 3.2 months for pancreatic primary lesions, and 4.1 months for hepatic/biliary primary lesions[10]. In the NORDIC NEC study, the median PFS was 3 months for esophageal primary lesions, 5 months for gastric primary lesions, 3 months for colonic primary lesions, 4 months for rectal primary lesions, 5 months for pancreatic primary lesions, and 4 months for cases with unknown primary lesion site [11].

2.1.7. Prognostic/predictive factors

When prognostic factors were examined by multivariate analysis in the multicenter joint study by Yamaguchi and Machida, out of the various factors such as sex, age (younger or older than 60 years), PS (0 or 1 vs. 2 or more), primary lesion organ (gastrointestinal primary lesions vs. hepatobiliary or pancreatic primary lesions), LDH levels (below vs. above the upper limit of facility standard), presence or absence of liver metastasis, presence or absence of history of radical resection and treatment regimen (IP therapy vs. EP therapy); the independent prognostic factors identified were gastrointestinal primary lesion (vs. hepatobiliary or pancreatic primary lesion, hazard ratio (HR): 0.58), and LDH levels being below the upper limit of facility standard for LDH (vs. above the upper limit of facility standard for LDH, HR: 0.65).

Although IP therapy exhibited slightly better impact on overall survival as compared to EP therapy with an HR of 0.8, the *p*-value of 0.389 meant that there was no significant difference between the two therapies[10]. The NORIDIC NEC study reported poor PS, colonic primary lesions, high platelet count, and high LDH levels to be the main factors behind poor prognosis[11]. However, these prognostic/predictive factors were not obtained with a global consensus.

2.1.8. Rationale for selection of the target population

The objective of this study is to develop a primary chemotherapy regimen for non-resectable/recurrent NECs, and out of the non-resectable or postoperatively recurrent NECs with gastrointestinal primary lesions or hepatobiliary or pancreatic primary lesions (shaded parts in Table 2.1.2 of WHO 2010 classification of NEC), we established chemotherapy-naïve patients to be the target population.

In terms of whether or not to consider MANEC a target of this study, a discussion is needed from the viewpoint of standard treatment. MANEC is considered when 30% or more of the carcinoma consists of adenocarcinoma components, and it is treated as either adenocarcinoma or NEC by discretion of the attending physician, with no real consensus on standard treatment. MANEC was however, excluded from this study, as its disease concept is different from NEC in terms of the standard treatment, which has a consensus regarding the treatment by a “regimen according to small cell lung carcinoma”. In fact, a questionnaire was provided to the three groups participating in this JCOG study (response obtained from 52 facilities). The results showed that 79% of facilities considered the WHO 2010 classification of NEC as the appropriate tissue type for this study, while only 13% responded that consideration of NEC+MANEC would be appropriate. Therefore, majority of facilities considered “WHO 2010 classification of NEC as the appropriate target of this study”.

While diagnosis of NECs is generally carried out using tissue samples and biopsy samples, as mentioned in 2.1.3, there is no consensus methodology to strictly distinguish NEC and MANEC using biopsy samples. As a result of group discussions, a consensus was reached between the three groups that cases presenting with NEC components by biopsy sample-based diagnosis can be enrolled. Although the use of this method would mean that a certain percentage of patients with MANEC, who were not intended to participate in this study, would be enrolled however, currently there are no appropriate means to avoid this. Therefore, we decided to create a consensus for the future based on information obtained from this study.

1）Reason for targeting gastrointestinal/hepatobiliary or pancreatic primary lesion NEC

Various guidelines recommend treatment regimens suited for small cell lung carcinoma, regardless of the organ of primary lesion, and is the rationale showing the validity of the treatment development for NEC across many organs. Furthermore, taking into consideration the frequency of the disease, it is unrealistic to develop treatments according to each organ, and in practice the Minnie-Pearl Cancer Research Network Study (mentioned later in 2.2.2), which is a relatively large-scale clinical study of NEC, development of treatment was carried out in a cross-organ

manner[27].

On the other hand, if we take hypothesize that we do not sufficiently understand how the clinical presentations of the disease vary according to the organ, the fact that this is the first randomized study in Japan, and that the maintenance of foundation for clinical studies of this disease in Japan is inadequate at present time, the hurdle is too high to plan a study across all organs including the fields of gynecology and urology. Based on the above consideration, we have decided to target all gastrointestinal organs which have a relatively large number of common points between each other in terms of clinical presentation and types of complication.

According to the multicenter observatory study by Yamaguchi and Machida, the prognosis of NEC with hepatobiliary or pancreatic primary lesion was significantly poorer than that of NEC with gastrointestinal primary lesion (MST: 7.9 vs. 13.0 months, respectively), but we determined that it is possible to accurately evaluate the efficacy of the treatment regimen by randomizing the subjects using the primary lesion site (gastrointestinal vs. hepatobiliary or pancreatic) as an allocation adjustment factor. Therefore, we considered patients with both, gastrointestinal/hepatobiliary or pancreatic primary lesions as one target population of this study, and to examine differences between organs exploratively by subgroup analysis.

2) Reason for including hepatic NEC cases (hepatic primary lesion or liver metastasis with primary lesion site unknown)

Even among NECs, cases with hepatic primary lesions are particularly rare. There are no specific reports on the frequency of incidence and differences from the other organs and reports are limited only to case reports and their reviews. Moreover, since the liver is a major organ for metastasis of primary lesions into other organs, even when tumors have been clinically identified in the liver, in many cases the primary lesion tends to be in another organ. Therefore, liver should not be deemed the primary lesion site without sufficient examination for a primary lesion. On the other hand, although very rare, there have been reports of NEC with hepatic primary lesion[28]. Even if sufficient search for primary lesion leads to no indication of the same outside of the liver, currently it is not possible to distinguish whether it is a case of “NEC with hepatic primary lesion” or “lesion in the liver is a metastatic lesion, with the primary lesion unknown”. In this study, such situations wherein “liver alone has identifiable lesions” would be henceforth, referred to as “hepatic NEC (hepatic primary lesion or liver metastasis from an unknown primary lesion site) for convenience. As the NCCN Guidelines[19] and ENETS Guidelines[20, 21] indicate the usefulness of FDG-PET for detailed examination of the primary lesion, we would also examine the primary lesion using FDG-PET. Furthermore, by referring to the diagnostic procedures for cancers with unknown primary lesion site, detailed examinations into the primary lesion would be carried out by otolaryngological (head and neck) examination and urological examination for men only, and gynecological examination for women only).

Such hepatic NEC (hepatic primary lesion or liver metastasis from an unknown primary lesion site) is anticipated even from an anatomical viewpoint, since the tumor-related complications are the same as that for other gastrointestinal primary lesion NECs, and there is no issue with treating them the same as for gastrointestinal primary lesions.

3) Reason for orienting the study for non-resectable or recurrent cases

As mentioned in 2.1.4 “Standard treatment according to disease staging and outline of prognosis”, systemic chemotherapy is indicated for cases with distal metastasis or recurrent cases. Locally advanced cases are particularly prone to pancreatic or bile duct primary lesions situated in the vicinity of vital vessels, and while the NCCN Guidelines propose chemoradiotherapy according to treatment for small cell lung carcinoma[19], the ENETS Guidelines provide no clear indication[21]. In reality, for the treatment of NEC which has a variety of primary lesion organs (scope of irradiation) there is not enough information available for the chemotherapy regimen that should be combined in terms of its optimum dose, the radiation dose, efficacy, and safety. For this reason, chemoradiotherapy cannot be considered the standard treatment with consensus, and systemic chemotherapy for distal metastasis is used widely except for pathologies involving frequent stenotic symptoms when the primary lesion is in the esophagus. For this reason, this study would enroll non-resectable or recurrent cases that include locally advanced cases.

The section “3.6 Definition of non-resectable NEC” shows the definition of non-resectable cases summarized by referring to the JCOG protocol for clinical studies of systemic chemotherapy (esophagus: JCOG0807, stomach: JCOG1013 or JCOG1002, bile duct: JCOG0805, pancreas: JCOG1106). Furthermore, while radical resection has

been considered for cases of colonic primary lesion adenocarcinoma with liver or lung metastasis, as cases of NEC with distal metastasis are not indicated for resection, we defined non-resectable cases as those in "Stage IV", and followed this definition for duodenal primary lesions, small intestine primary lesions, and appendix primary lesions. Furthermore, with respect to NEC with esophageal primary lesion, chemoradiotherapy is well-indicated for cases with supraclavicular lymph node metastasis of thoracic esophageal tumor even in Stage IV, and hence were not included in this study.

4) Treatment of draft of new classification of NEC

In recent years, proposals have been made to further differentiate the WHO 2010 classification of NECs (G3 in ENETS) to "Grade 3 proliferative tumors showing the same morphological presentations as NET" and "Grade 3 tumors with strong morphological atypicality (previously classified as poorly differentiated NECs)"[29, 30], or by Ki-67 index of 20–50% and 50% or more[11, 19]. This is yet to be confirmed; however, in this study we would have used the WHO 2010 classification. However, we would have used samples collected for central pathological diagnosis to carry out studies related to classification which exploratively adds grade classification and morphological differentiation levels.

2.2. Standard treatment for target disease

Currently, there is no standard drug treatment for non-resectable/recurrent NECs with efficacy verified by a randomized controlled trial. However, given the similarity between pathological and clinical presentations, treatments according to small cell lung carcinoma have been attempted, and there have been reports of positive treatment outcome in small-scale clinical studies and observational studies.

2.2.1. Standard treatment for small cell lung carcinoma

Cisplatin-based multidrug combined therapy is the standard first-line treatment of extensive disease (ED) small cell lung carcinoma. While cyclophosphamide/doxorubicin/vincristine (CAV therapy) was established as a standard treatment in the 1970s in Europe and U.S., the etoposide/cisplatin combined therapy (EP therapy) was introduced in the late 1980s. A comparative study of EP therapy and CAV therapy did not show superiority of EP therapy over CAV therapy in terms of survival, but the response rate (CAV: 51% vs. EP: 61%) and MST (CAV: 8.3 months vs. EP: 8.6 months) were almost the same, and the lighter toxicity level (mucositis, interstitial pneumonia, hemotoxicity) meant that EP could be used as a standard treatment[31]. Thereafter, the JCOG Lung Cancer Group carried out the "comparison between EP therapy and Irinotecan/Cisplatin combined therapy (IP therapy) for Extensive-stage Small Cell Lung Cancer (JCOG9511)", and reported that the IP therapy provided a significantly better overall survival, with MST being 9.4 months vs. 12.8 months (p = 0.002) for IP therapy[32]. However, the two additional large-scale studies conducted primarily in U.S. did not show superiority of IP therapy over EP therapy, and for this reason IP therapy has not been not used as the standard treatment overseas[33, 34]. Furthermore, while the results of the "Randomized controlled trial to verify the non-inferiority of amrubicin/cisplatin combined therapy (AP therapy) over IP therapy (JCOG0509)" were reported in the 2012 conference of the American Society of Clinical Oncology (ASCO), but AP therapy was still considered significantly inferior to IP therapy[35]. Presently, the JCOG Lung Cancer Group considers IP therapy to be the standard treatment for extensive-disease small cell lung carcinoma.

2.2.2. Standard treatment for extrapulmonary NEC

While there are scattered reports relating to use of CAV therapy, EP therapy, IP therapy, and other multidrug combined therapies for extrapulmonary NEC, these reports are primarily from observational studies. Since these reports precede the unification of concept and classification methods of this disease, the nomenclature such as NEC (anaplastic type), NET (poorly differentiated type) and extrapulmonary small cell lung carcinoma have still been used. These reports, however, are presumed to be targeting almost the same disease group as NEC. There are no reports of prospective studies using EP therapy for extrapulmonary NEC. Observational studies have reported response rates between 42–67%, and MST between 15–19 months (Table 2.2.2a). On the other hand, IP therapy has reported results from observational studies and clinical studies, with response rates between 7–83% and MST of 10.1–22.6 months (Table 2.2.2b). As mentioned above, reports related to extrapulmonary NEC are limited to use of EP therapy and IP therapy in observational studies and small-scale clinical studies. The largest number of case enrollments in a clinical study of extrapulmonary NEC was seen in the Minnie-Pearl Cancer Research Network Study, which evaluated the efficacy of triple-drug combined therapy using carboplatin/etoposide/paclitaxel. The

response rate to this triple-drug combined therapy was 53%, while the MST was 14.5 months and adequate, there was intense toxicity involved, and the results were not significantly different from EP therapy, so this regimen was not considered the standard treatment[27].

The 2014 NCCN Guidelines recommend treating extrapulmonary NEC using a regimen according to small cell lung carcinoma, and the guidelines for treatment of small cell lung carcinoma mention both EP therapy and IP therapy as recommended regimens.

Therefore, the standard treatment for extrapulmonary NEC is taken as EP therapy or IP therapy.

Table 2.2.2.a. EP therapy for NEC (observational studies)

Reported year/Reporter	Target	N	Response rate	MST (in months)
1991/ Moertel[18]	Pancreatic/gastrointestinal NEC （anaplastic type）	18	67%	19
1999/ Mitry [36]	Pancreatic/gastrointestinal NEC	41	42%	15
2001/ Marie-Louise [37]	Pancreatic NET（well: 11, poorly: 4） Gastrointestinal cartinoid: 21	33	18%	19
1994/ Lo Re G [38]	Extrapulmonary SCLC	13	69%	NE
2010/ Iwasa [39]	Hepatobiliary or pancreatic NEC	21	14%	7.3
2012/ Yamaguchi [10]	Gastrointestinal NEC	12	75%	14
2012/ Yamaguchi [10]	Hepatobiliary or pancreatic NEC	34	12%	6.9
2012/ Sorbye [11]	Gastrointestinal primary lesion NEC (including primary lesion unknown)	129	31%	12

Table 2.2.2.b. IP therapy for NEC (shaded are clinical studies, others are observational studies)

Reported year/Reporter	Target	N	Response rate	MST (in months)
2003/ Hou [40]	NEC（gastrointestinal-80%）	18	43%	NE
2005/ Chin [41]	Esophageal NEC	12	83%	14
2011/ Okita [42]	Gastric NEC	12	75%	22.6
2012/ Yamaguchi [10]	Gastrointestinal NEC	142	51%	13.4
2012/ Yamaguchi [10]	Hepatobiliary or pancreatic NEC	18	39%	10.1
2006/ Kulke [43]	Pancreatic/gastrointestinal (including NET G1/G2)	15	7%	11.4
2008/ Mani [44]	Pancreatic/gastrointestinal NEC	20	58%	NE
2008/ Jin [45]	Extrapulmonary NEC	15	67%	11.4.

Table 2.2.2.c. Phase II study of carboplatin/etoposide/paclitaxel triple-drug combined therapy for NEC

Reported year/Reporter	Target (breakdown)	N	Response rate	MST (in months)
2006/ Hainsworth [27]	NEC （Colon：9, lung: 7, skin: 4, pancreas：3, gall bladder：1, thyroid:1, stomach: 1, esophagus: 1, endometrium:1, maxillary sinus: 1, prostate: 1, primary lesion unknown: 48）	78	53%	14.5

2.2.3. Standard treatment for gastrointestinal/hepatobiliary or pancreatic primary NEC

Treatments for extrapulmonary NEC have not been developed according to any specific organ, and the standard treatment for gastrointestinal/hepatobiliary or pancreatic NEC is also both, EP therapy and IP therapy. While both treatments are considered standard treatments, since this study considers them both to be study treatments, the anticipated effect and expected adverse reactions from EP therapy and IP therapy have been described in “2.3.2 Study treatment(s) of this study”.

Herewith, we describe the current status of the usage of each treatment in Japan and overseas. According to the multicenter joint study by Yamaguchi and Machida, 160 out of 258 patients (62%) who received systemic chemotherapy underwent IP therapy, the most common treatment, followed by EP therapy (46 patients, 18%). Although there is big deviation where 92% of gastrointestinal primary lesion NEC (142/154 patients) were given IP therapy, and 65% of hepatobiliary or pancreatic primary lesion NEC (34/52 patients) were given EP therapy, the present situation shows that treatments are being selected by discretion or preference of the facility/physician.

Furthermore, even with respect to the dosing schedule, the method of administration has not necessarily as per the treatment of small cell lung carcinoma. There is no unified consensus, as some facilities use methods used for treatment of gastric cancer (JCOG9912 regimen), based on the reasoning that they have familiarity with IP therapy. On the other hand, in the NORDIC NEC study the most common treatment used was the EP therapy, which was administered to 129 out of 252 patients (51%) who received systemic chemotherapy, followed by a combined therapy consisting of carboplatin and etoposide therapy (67 patients, 27%). Combined therapy of platinum and etoposide was used often, which is another choice of treatment in Japan[11].

2.3. Rationale for establishment of treatment plan

2.3.1. Drugs

1) Etoposide

Etoposide exerts an antitumor effect by inhibiting topoisomerase II, which catalyzes the untangling of supercoiled DNA strands. Main toxicities include myelosuppression, nausea/vomiting, alopecia, and stomatitis.

2) Cisplatin

It is a complex ion form of the heavy metal platinum which shows anti-tumor effect by cross-linking double-stranded DNA. Currently, it is considered one of the key drugs for NEC treatment, being a central medicine for the treatment of lung cancer due to its synergistic effect with radiation therapy and various drugs, and also due to its low bone marrow toxicity when used alone. Toxicities include nausea/vomiting, nephrotoxicity, and neurotoxicity. Sufficient infusion of cisplatin before and after drug administration is necessary to prevent nephrotoxicity.

3) Irinotecan

Irinotecan is a topoisomerase I inhibitor developed in Japan that inhibits DNA synthesis. The drug is directly converted into the active metabolite (SN-38) in human liver and various tissues by carboxyesterase. As it shows a potent antitumor effect against SCLC even when used on its own, this drug is used very often in routine clinical practice to treat NEC as well. Diarrhea and myelosuppression are observed as dose-dependent toxicities. Nausea/vomiting and interstitial pneumonia have also been noted in patients.

2.3.2. Study treatment(s) of this study

1) Etoposide/cisplatin combined therapy (EP therapy)

In the multicenter joint study performed by Yamaguchi and Machida, the response rate of gastrointestinal primary lesion NEC to EP therapy was 75% (9/12 patients), with an MST of 14 months. The response rate of hepatobiliary and pancreatic primary lesion NEC to EP therapy was 12% (4/34 patients), and the MST was 6.9 months. With respect to safety, the multicenter joint study by Yamaguchi and Machida showed no treatment-related deaths associated with EP therapy (N = 46) administered as first-line treatment for gastrointestinal/hepatobiliary and pancreatic primary lesion NEC, and toxicity-related study discontinuation was observed in 6.5% of cases. Details of toxicities remain unknown as they were not investigated. The observational study of EP therapy for hepatobiliary and pancreatic primary lesion NEC (N = 21) carried out by Iwasa et al. at the National Cancer Center Hospital showed major Grade 3/4 adverse events to be neutropenia (90%), nausea (33%), and anorexia (24%). Grade 3 febrile neutropenia was observed in 8 patients (38%)[39]. Table 2.3.2 shows the toxicity profile of EP therapy from the JCOG9511 study which treated small cell lung carcinoma (Grade 3 or higher as per JCOG toxicity criteria).

Table 2.3.2. Toxicity of EP therapy and IP therapy in studies treating small cell lung carcinoma※
※Report by Iwasa et al., JCOG0509：CTCAE v3.0 Grade 3 or higher, JCOG9511：JCOG toxicity criteria Grade 3 or higher

	EP therapy		IP therapy	
	Report by Iwasa et al.[34]	JCOG9511[27]	JCOG9511[27]	JCOG0509[30]
Neutropenia	90%	92.2%	65.3%	58.5%
Leukopenia	71%	51.9%	26.7%	22.5%
Decreased hemoglobin	29%	29.9%	26.7%	23.2%
Thrombocytopenia	24%	18.2%	5.3%	2.1%
Diarrhea	0%	0%	16%	7.7%

Nausea	33%	6.5%	13.3%	6.3%
Aspartate aminotransferase increased	19%	2.6%	0%	-
Alanine aminotransferase increased	24%	3.9%	4%	-
Blood bilirubin increased	19%	0%	0%	-
Creatinine increased	0%	0%	0%	-
Peripheral motor neuropathy	0%	0%	0%	-
Febrile neutropenia	38%	0%	0%	1.4%

There are several reports concerning the specific dosage schedule/administered dose for EP therapy, and four dosing methods have been described in the NCCN Guidelines for SCLC as well. However, it is not clear as to which of the dosing methods is the best, and the dosing method for NEC varies between reports. In Japan, the dosing for EP therapy according to JCOG9511 is also often used for NEC. For this reason, this study would also follow the same method of administration. In terms of the number of cycles, a comparison of 4 courses vs. 8 courses of combined chemotherapy including cyclophosphamide for SCLC showed poor efficacy even when the treatment continued for long-term[46], and treatment of 4 cycles has also been specified in JCOG9511. There have been no such comparative studies for gastrointestinal/hepatobiliary and pancreatic primary lesion NEC, rather, there is no evidence to suggest that first-line chemotherapy should be discontinued if the chemotherapy is efficacious and toxicity is within the permitted scope. Unlike SCLC, NEC does not respond well to chemotherapy and tends to re-exacerbation to the underlying disease soon after the end of chemotherapy. For this reason, continuation of chemotherapy is very likely to be beneficial for patients with NEC as long as the treatment is effective.

While peripheral motor neuropathy, hearing impairment, and renal disorder are known accumulation toxicities of cisplatin, if sufficiently safe, it is ideal for treatments to continue as much as possible, considering the very few treatment options available for NEC. Based on the above rationale, we decided to ensure safety by establishing strict protocol treatment discontinuation criteria and chose not to limit the total dose or prescribe the number of treatment cycles.

2）Irinotecan/Cisplatin combined therapy (IP therapy)

In the multicenter joint study by Yamaguchi and Machida, the response rate of gastrointestinal primary lesion NEC to IP therapy was 51% (73/142 patients), with an MST of 13.4 months. The response rate of hepatobiliary and pancreatic primary lesion NEC to IP therapy was 39% (7/18 patients), and the MST was 10.1 months. There were no treatment-related deaths among 160 patients, and discontinuation due to toxicity in first-line chemotherapy was seen in 11.2% of cases. Details about toxicity were not collected. As a reference, Table 2.3.2 shows the toxicity profile of IP therapy from the JCOG9511 and JCOG0509 studies which treated small cell lung carcinoma.

There are several reports concerning specific dosing methods for IP therapy, and two dosing methods have been described in the NCCN Guidelines for SCLC as well. It is not known which of the dosing methods is the best, and the dosing method has also been variable in the NEC. In Japan, the dosing method of IP therapy, according to JCOG9511 is considered the standard treatment for SCLC and the same method has also been used in this study.

The total dose and number of cycles would have not been prescribed for the same reason as EP therapy, and safety has been ensured by establishing strict protocol treatment discontinuation criteria.

3）Other study treatment candidates

Presently, there are no drugs or treatment methods, including molecular-targeted drugs, which surpass the EP and IP therapy. There are also no planned or ongoing large-scale clinical trials globally, related to first-line treatment of NEC.

As the second-line treatment, a phase II study of Everolimus is underway for pancreatic primary lesion NEC resistant or unresponsive to platinum preparations. It is a multicenter study being carried out in 31 facilities in Japan, led by the National Cancer Center East Hospital, through the cancer research and development fund (UMIN000012752）.

2.3.3. Summary of risk/benefit balance of the standard treatment and study treatment

In the multicenter joint study by Yamaguchi and Machida, IP therapy had better prognosis compared to EP therapy (MST: 13.0 months vs. 7.3 months; $p < 0.0001$). However, as shown in Table 2.3.3, since IP therapy was more frequently chosen for gastrointestinal primary lesions (142/154 patients) and EP therapy for hepatobiliary and

pancreatic primary lesions (34/52 patients), it has not been possible to determine whether this difference is due to difference in efficacy between the regimens, or due to differences in the primary lesion organ. Primary lesion organ remained a significant prognostic factor after multivariate analysis, and while IP therapy turned out to be a slightly better regimen than EP therapy with HR = 0.8 (95% CI. 0.48–1.33), $p = 0.389$ indicated that there was no significant difference between the two (see “2.1.5 Prognostic/predictive factors”). Taking into account the number of EP therapy cases (46 patients), the fact that it was an observational study, and including other unknown bias, the data interpretation is fairly limited. Therefore, based on these results we are unable to deem either of IP or EP therapies to be more efficacious than the other.

With regard to toxicity, while myelosuppression such as neutropenia is milder in IP therapy than EP therapy, frequencies of events such as diarrhea and nausea are high. While the two treatments have different toxicity profiles, it is difficult to conclude that one has clearly worse toxicity than the other. The risk of bile duct obstruction is high in biliary and pancreatic primary lesion NECs and the use of irinotecan (which undergoes biliary excretion) in patients exhibiting biliary excretion disorder runs the risk of increased toxicity, hence care must be taken while administering irinotecan. We believe that this risk can be avoided by appropriate pre-treatment drainage and proper monitoring during treatment. Based on the above, it is difficult to assign superiority to IP therapy or EP therapy over the other in terms of risk/benefit balance, and both the regimens can be considered suitable standard treatments.

Table 2.3.3. Summary of results from multicenter study by Yamaguchi and Machida

		IP	EP	*p*-value*
Total	N	160	46	
	Response rate（%）	50	27	＜0.001
	Median PFS (in months)	5.2	4.0	0.033
	Median OS (in months)	13.0	7.3	＜0.0001
Gastrointestinal primary lesion NEC	N	142	12	
	Response rate（%）	51	75	0.14
	Median PFS (in months)	5.4	4.9	0.585
	Median OS (in months)	13.4	14.0	0.976
Hepatobiliary and pancreatic primary lesion NEC	N	18	34	
	Response rate（%）	39%	12%	0.034
	Median PFS (in months)	4.4	3.7	0.056
	Median OS (in months)	10.1	6.9	0.05

*Response rate by chi-squared test, PFS and OS by log-rank test

2.3.4. Post-treatment(s)

In the multicenter joint study by Yamaguchi and Machida, 56% (116/206 patients) of patients with NEC who received IP therapy or EP therapy were administered chemotherapy as the second-line of treatment. The most common second-line chemotherapy after IP therapy was amurcibin (22/88 patients), while irinotecan was the most common second-line treatment after EP therapy (13/28 patients), and we expected a similar pattern for the second-line treatments in this study as well. The overall performance of second-line chemotherapy was poor with a response rate of 11%, and PFS of 2.1 months. As such, the significance of the second-line treatment has not been indicated, and hence, no standard treatment has been established.

2.4. Study design

2.4.1. Rationale for establishing endpoints

For NECs, an alternative endpoint for overall survival, such as PFS has not been established as an indicator for comparing the usefulness of the treatment regimens. Therefore, comparison of overall survival was considered appropriate when examining the therapeutic effects. For the same, overall survival was examined as the primary endpoint. The secondary endpoints to evaluate the efficacy and safety were the response rate (only for patients with a measurable lesion), progression-free survival, incidence rate of adverse events, and dose intensity of cisplatin.

2.4.2. Clinical hypothesis and rationale for setting the number of enrollments

This study compares the two standard treatments, both of which are difficult to deem superior than the other in terms of efficacy and safety. We have, therefore, adopted a study design using bilateral testing. The main clinical hypothesis of this study is that either IP therapy or EP therapy is likely to provide better overall survival than the other treatment group, and should this hypothesis be validated, the superior treatment with statistical significance would be deemed the better treatment, and would therefore be positioned as the standard treatment in future. If the study fails to validate this hypothesis, it would indicate that there was no clinically significant difference in overall survival between the two groups, and if there are no large differences in terms of toxicity, we would conclude that both treatments are viable options as the standard treatment. However, if unlike our initial hypothesis, one treatment shows clearly more toxicity than the other, we would reconsider the study design before carrying out the main analysis.

Furthermore, as mentioned in “section 2.1.1 Epidemiology”, gastroenterological primary lesion NEC, which is the primary target of this study, is a very rare disease with an annual prevalence of around 3 in 100,000 people, hence, the significance level of testing has been placed at 10% bilaterally, instead of 5% bilaterally. In the multicenter joint study conducted by Yamaguchi and Machida, the MST of gastrointestinal/hepatobiliary or pancreatic primary lesion NEC was 11.5 months. To determine if one treatment is superior to the other, we expected a difference of four months in terms of MST. If we expect the more inferior treatment to have an MST of 8.0 months and the superior treatment to have an MST of 12.0 months, the number of enrollments are calculated using the aforementioned parameters and based on the discussion mentioned later (see “12.2 Expected number of enrollments/Enrollment period/Follow-up period”). This provided a research period of 7- years, consisting of 6-years of enrollment period and one year of follow-up period, and with $\alpha = 0.1$ bilaterally and detection power of 70% to detect differences between the two groups, 63 subjects were required per group. Taking into consideration some subjects who could be lost post follow-up, we aimed to enroll 70 subjects per group, and a total of 140 subjects between two groups.

If enrollment proceeds better than expected than prior to the start of the study (i.e. if the number of enrolled subjects reach 70 in less than 2.5 years from the start of enrollment), the number of enrollments is likely to be re-established by changing the detection power from 70% to 80% during the study, with the aim of obtaining more accurate results.

＜Addition to ver.1.1＞

The pace of enrollment after the start of enrollment period exceeded expectations, and the number of enrollments reached 70 subjects in October 2016, which was 2 years and 2 months after the start. As this exceeded the criteria of 70 subjects in less than 2.5 years, since the start as provided above, we obtained the approval of the Hepatobiliary and Pancreatic Oncology Group Meeting on October 29, 2016, Esophageal Cancer Group Meeting on November 19, 2016, and Gastric Cancer Group Meeting on January 7, 2017 to change the detection power to 80% according to provisions at the time of study planning. Consequently, the number of intended enrollments was changed to 170 subjects.

2.4.3. Expected patient enrollment

In the multicenter joint study by Yamaguchi and Machida, 258 cases of gastrointestinal/hepatobiliary or pancreatic primary lesion NECs were reported from 23 sites during the 11 years between 2000 and 2011, but between 2000 and 2006, information could not be obtained because of old cases. When limited to the most recent five years (2006–2011), when sufficient information was obtained from each facility, the number of enrollments was placed at162 subjects.

This is a joint study between three groups, namely the JCOG Hepatobiliary and Pancreatic Oncology Group,

JCOG Gastric Cancer Group, and the JCOG Esophageal Cancer Group. Therefore, the total number of facilities, excluding overlaps, amounted to 82 facilities. Compared to the multicenter joint study by Yamaguchi and Machida, we expect an increase in the number of patients enrolled, and simple calculations lead us to extrapolate 115 subjects to be enrolled annually. On the other hand, if we take into account the possibility of patient enrollments being biased to some high volume centers, it would be difficult to estimate the effect of increasing facility count to proceed. In addition, considering that the study is a randomized, we estimate the annual number of patients enrolled to be between 30–50 people. Taking into account ineligible cases, we provided 6-years for patient enrollment period.

2.4.4. Rationale for setting allocation adjustment factors

1) Facility

It is widely known that background, treatment, efficacy evaluation, and safety evaluation of enrolled patients vary depending on the facility, and JCOG standards have been used to make adjustments between facilities.

2) Primary lesion organ (gastrointestinal tract vs. hepatobiliary or pancreatic organ)

In the multicenter joint study by Yamaguchi and Machida, multivariate analysis using the Cox proportional hazard model was carried out with 183 subjects, which indicated primary lesion organ (gastrointestinal tract vs. hepatobiliary or pancreatic organ) to be a significant prognostic factor.

2.4.5. Centralized pathological diagnosis

In this study, a centralized pathological diagnosis would be performed for analysis of NEC, despite patients being diagnosed by a third party. The operation of the centralized pathological diagnosis is described in section 15.1, while details of the operation have been described in the Centralized Pathological Diagnosis Procedure Manual. Furthermore, an analysis of endpoints based on the results of centralized pathological diagnosis would be provided as a reference.

2.5. Summary of expected advantages and disadvantages associated with study participation

2.5.1. Expected advantages

Drugs used for both groups of this study are treatments used in routine medical practice. As mentioned in the next section, although it would be necessary to use some drugs for which insurance coverage does not apply, since insurance claims for these treatments are made in a similar manner to general medical care however, in practice they do not receive insurance assessments.

Furthermore, cases of off-label medication have been studied according to “18th Case Providing Review Information” at the Case Review Committee established by the Health Insurance Claims Review & Reimbursement Services. Based on the review information provided as on February 26, 2018;, The use of “irinotecan hydrochloride hydrate, etoposide, cisplatin, and carboplatin [injections]” is approved for treatment of “neuroendocrine carcinoma”], and the notices issued by the Ministry of Health, Labor and Welfare on February 26, 2016the results of this study have been considered valid by the ministry. For the same reason, essentially since February 26, 2018, there have been no concerns about claims of insurance assessments.

Moreover, since the medical fees of study participants during the study, which includes drug fees, are paid in principle by the patients themselves and their insurance coverage, so the patients would receive no special medical or financial benefits from participation in this study.

2.5.2. Expected risks and disadvantages

Both treatment arms A and B would receive chemotherapy regimen used in routine medical practice, so they would be unlikely to be exposed to special risks or disadvantages not observed in routine medical practice. The descriptions in “2.3.3 Risk/benefit balance of standard treatment and study treatments” outline the expected risks and disadvantages for each treatment arm.

To minimize the risk of adverse events and disadvantages, the “ Patient selection criteria (Section 4)”, “ Criteria to change treatment (Section 6.3)” and “Concomitant/Supportive therapies (Section 6.4)” have been carefully considered for the three groups. Furthermore, the Institutional Review Board would monitor if the adverse events are within the scope of expectation, while any serious adverse events or unexpected adverse events would be carefully examined and reviewed according to the provisions related to “JCTN-Adverse Events Report Guideline”

and "JCOG Guidelines for Handling Clinical Safety Information", and a system has been provided to take any necessary countermeasures.

Precautions related to etoposide, irinotecan, and cisplatin

As of December 2013, etoposide therapy, which is the intended to be used in this study had not received insurance approval for the treatment of gastrointestinal or hepatobiliary and pancreatic primary lesion cancers. Irinotecan has also not received insurance approval for treatment of cancers with primary lesions in organs other than the stomach, colon, and rectum. Furthermore, as cisplatin is only indicated for esophageal, gastric cancer, and for biliary tract cancers when combined with gemcitabine (25 mg/m^2), the dosage and administration used in this study have not been approved previously.

As each facility in this study may carry out the insurance claims in the same manner as routine general medical practice, the treatment may receive insurance assessment after-the-fact. However, if a facility incurs losses, the loss must be borne by the relevant facility (medical institution), as there is no system of compensation arranged by the JCOG Research Organization. If actual losses occur, the continued participation in the study would then be carefully discussed between the facility supervisor and the principal investigator/clinical trial secretariat. The facility supervisor would be expected to gain approval from the facility IRB and the head of the medical institutions.

2.6. Significance of this study

While both, EP therapy and IP therapy are standard treatments, the efficiency of either treatment being better than the other is not yet clear, and currently in routine medical practice, the choice of treatment is being made on the discretion or preference of the facility/attending physician.

If this study is able to clearly indicate the efficiency of EP therapy or IP therapy over the other, we expect that the diminished use of the relatively inferior treatment would contribute to improvement in patient prognosis. If the study indicates no clinically meaningful differences between the two treatments, the results would provide evidence that there is no significant issue with the treatments being chosen by discretion or preference of the facility/attending physician, which has been done traditionally without any data that directly compare the two treatments, although that would not indicate that the effects of the two treatments are equivalent. We also expect that the establishment of a highly reliable standard treatment in this study which would provide a foundation for therapeutic development when promising treatment regimen in the future.

As NEC is a rare disease, and since each clinician is not likely to have adequate experience, we believe that work associated with multicenter studies such as confirmation of diagnosis by centralized diagnosis/results feedback and sharing results of treatments by unified treatment regimen would lead to improvement of quality of care for NEC in Japan.

2.7. Associated research (including sample analysis research)

No such studies have been planned or carried out at the time of preparation of the protocol.

2.8. JCOG-Biobank Japan (BBJ) collaborating biobank

This study would participate in the banking of blood samples (DNA/plasma) through the JCOG-BBJ collaboration biobank based on the protocol common to all JCOG studies (hereafter termed as "common banking").

Common banking by JCOG-BBJ collaborating biobank would collect and store the samples of patients enrolled in clinical studies conducted by JCOG regardless of presence/absence of pre-planned sample analysis research, would also provide samples for future analysis research and diagnostic information obtained through the main research.

The target of such biobanking are those patients who provided consent to participate in this study and gave consent to provide samples to the JCOG-BBJ collaborating biobank for their use in future sample analysis research (hereafter termed as consent for banking).

Samples collected during common banking include whole blood and preserved pathological tissue samples from routine clinical practice. Plasma and DNA separated/isolated from blood would be stored in the JCOG-BBJ collaborating biobank and would be provided for sample analysis research in the future. Preserved pathological tissue samples from routine medical practices such as surgery, and biopsy/clinical laboratory tests would also likely to be used for future sample analysis research. Although the type, sample preparation methods, and tissue quantity required would vary from study to study, there is no definite consensus that banking pathological tissue in a certain

method is more efficient than others. Furthermore, it has been suggested that long-term preservation of a sectioned specimen from preserved pathological tissue may lead to sample deterioration (DNA fragmentation). The discussions between the of JCOG and BBJ personnel about these problems, led to the conclusion that preserved pathological tissue taken after medical examination shall require only patients' consent for future use, and actual collection must be started after standardizing a separate protocol and specifying in it the procedures best suited for the details of the research.

The detailed procedures for sample collection, storage, and method of sample provision for future sample analysis research in common banking have been stipulated in the "JCOG-BioBank Japan Collaborating Biobank Protocols" that apply to all the JCOG studies. To participate in the common banking carried out by JCOG-BBJ collaborating biobanks, the subject matter must be reviewed and approved by the ethics committees of each participating facility.

Furthermore, to conduct sample analysis research in future using samples stored in the JCOG-BBJ collaborating biobank, it would be necessary to prepare a "Sample Analysis Research Protocol" and have it reviewed and approved by the JCOG Protocol Review Committee and the ethics committee of facilities involved in the sample analysis.

3. Criteria/definitions used in this study

Tissue classification would be performed according to WHO 2010 classification and ENETS（European Neuroendocrine Tumor Society） classification[14, 15], while disease staging would be done according to “UICC-TNM 7th Edition”.

3.1. Tissue classification (WHO 2010 classification)

The shaded parts are the targets of this study

Neuroendocrine neoplasms

1) Neuroendocrine tumor：NET Grade 1（NETG1）
2) Neuroendocrine tumor：NET Grade 2（NETG2）
3) Neuroendocrine carcinoma：NEC（large cell or small cell type）
4) Mixed adenoendocrine carcinoma（MANEC）
5) Hyperplastic and preneoplastic lesions

3.2. Grade classification （ENETS [European Neuroendocrine Tumor Society] / WHO2010 classification）

Grade 1（G1）	Number of mitotic presentations < 2 per 10 high power fields（HPF）and/or Ki-67 index ≤2%＊
Grade 2（G2）	Number of mitotic presentations 2–20 per 10 HPF and/or Ki-67 index 3-20%
Grade 3（G3）	Number of mitotic presentations > 20 per 10 HPF and/or Ki-67 index >20%

＊The description of number of mitotic presentations in WHO 2010 classification is ≤2, but 2–3% are classified to G1[47].

3.3. Histopathological diagnosis

- Immunostaining (Chromogranin A and synaptophysin) is essential for a pathological diagnosis of NEC.
- Either1. or 2., or both are studied to determine proliferative activity. Number of mitotic presentations and Ki-67 index would adopt a high grade evaluation.
  1. Number of mitotic presentations (to evaluate 50HPF)
  2. Ki-67 index（500–2000 tumor cells*）

  *（Only in this study, if the number of tumor cells in the collected sample is less than 500, the number of tumor cells measured is also listed. A minimum of 100 tumor cells is required.）
- If sufficient amount of biopsy sample cannot be collected for a pathological diagnosis, a cell block prepared using material obtained from EUS-FNA, brushing or needle biopsy may be used. However, the use of a cell block prepared from ascites or pleural effusion is not permitted.
- During a histological diagnosis using a resected tumor sample, a slide with a representative section shall be used to evaluate the proportion of NEC components (differential diagnosis with mixed adenoendocrine carcinoma). For a comprehensive examination, the entire tumor must be evaluated.

For further details NEC Pathological Diagnosis Handbook would be referred (posted on JCOG website).

3.4. Disease stage classification criteria (UICC-TNM 7th Edition)

Special notice regarding disease stage classification: Although there is an independent TNM classification for NET G1 and NET G2 with primary lesions in the stomach, small intestines, and colon; since this study concerns NEC, the disease would be classified according to the classification method of major tissue type in each primary lesion organ (squamous cell carcinoma for esophagus, and adenocarcinoma for all other primary lesion organs). In this study, the classification of intrahepatic cholangiocarcinoma is used for hepatic NEC, due to their similar clinical presentation.

3.4.1. Esophagus

T –Primary tumor

TX： Primary tumor cannot be evaluated
T0： Primary tumor unidentified
Tis： Epithelial carcinoma/highly dysplastic
T1： Tumor invading mucosal lamina propria, muscularis mucosae, or submucosa
  T1a：Tumor invading the mucosal lamina propria or muscularis mucosae
  T1b： Tumor invading the submucosa

T2 : Tumor invading the muscularis propia
T3 : Tumor invading the adventitia
T4 : Tumor invading the surrounding tissue
T4a : Tumor invading the pleura, pericardium, and diaphragm
T4b : Tumor invading other surrounding tissues such as the aorta, centrum, trachea

N –Regional lymph nodes
NX : Regional lymph node metastasis cannot be evaluated
N0 : No regional lymph node metastasis
N1 : 1–2 foci of regional lymph node metastases
N2 : 3–6 regional foci of lymph node metastases
N3 : 7 or more foci of regional lymph node metastases

M –Distal metastasis
M0 : No distal metastasis
M1 : Distal metastasis present

Stage	T	N	M
**0**	Tis	N0	M0
**IA**	T1	N0	M0
**IB**	T2	N0	M0
**IIA**	T3	N0	M0
**IIB**	T1, T2	N1	M0
**IIIA**	T4a	N0	M0
	T3	N1	M0
	T1, T2	N2	M0
**IIIB**	T3	N2	M0
**IIIC**	T4a	N1、N2	M0
	T4b	Unrelated to N	M0
	Any T	N3	M0
**IV**	Any T	Any N	M1

3.4.2. Stomach

T –Primary tumor
TX : Primary tumor cannot be evaluated
T0 : Primary tumor unidentified
Tis : Epithelial carcinoma: Epithelial carcinoma/highly dysplastic carcinoma not invading the lamina propia mucosa
T1 : Tumor invading mucosal lamina propria, muscularis mucosae, or submucosa
T1a : Tumor invading the mucosal lamina propria or muscularis mucosae
T1b : Tumor invading the submucosa
T2 : Tumor invading the muscularis propia
T3 : Tumor invading the subserosa
T4 : Tumor perforating the serosa, or invading adjacent structures[1,2,3]
T4a : Tumor perforating the serosa
T4b : Tumor invading adjacent structures[1,2,3]

1. Adjacent organs of the stomach are spleen, transverse colon, liver, diaphragm, pancreas, abdominal wall, adrenal glands, kidneys, small intestine, and retroperitoneum.
2. If the invasion has spread from the stomach to the duodenum or esophagus, classification is made in terms of depth.
3. A tumor that advances into the gastrocolic ligament, into the hepatogastric ligament, or into the greater or lesser omentum, and is classified as T3, when there is no perforation of the visceral peritoneum.

N –Regional lymph nodes

NX : Regional lymph node metastasis cannot be evaluated
N0 : No regional lymph node metastasis
N1 : 1–2 regional foci of lymph node metastases
N2 : 3–6 regional foci of lymph node metastases
N3 : 7 or more regional foci of lymph node metastases
N3a : 7–15 regional foci of lymph node metastases
N3b : 16 or more regional foci of lymph node metastases

M –Distal metastasis
M0 : No distal metastasis
M1 : Distal metastasis present

Stage	T	N	M
**0**	Tis	N0	M0
**IA**	T1	N0	M0
**IB**	T2	N0	M0
	T1	N1	M0
**IIA**	T3	N0	M0
	T2	N1	M0
	T1	N2	M0
**IIB**	T4a	N0	M0
	T3	N1	M0
	T2	N2	M0
	T1	N3	M0
**IIIA**	T4a	N1	M0
	T3	N2	M0
	T2	N3	M0
**IIIB**	T4b	N0, N1	M0
	T4a	N2	M0
	T3	N3	M0
**IIIC**	T4a	N3	M0
	T4b	N2, N3	M0
**IV**	Any T	Any N	M1

3.4.3. Small intestines (including duodenum)

T –Primary tumor
TX : Primary tumor cannot be evaluated
T0 : Primary tumor unidentified
Tis : Epithelial carcinoma
T1 : Tumor invading mucosal lamina propria, muscularis mucosae, or submucosa
T1a : Tumor infiltrating the mucosal lamina propria or muscularis mucosae
T1b : Tumor infiltrating the submucosa
T2 : Tumor infiltrating the muscularis propia
T3 : Tumor invading the subserosa, or tumor invading within 2 cm of surrounding tissue of muscularis externa without peritoneal cover (mesenterium, retroperitoneum)*
*Surrounding tissue of muscularis externa without peritoneal cover refers to the mesenterium in the jejunum and ileum, and the retroperitoneum in the duodenum without serosa.
T4 : Tumor penetrating the visceral peritoneum, or tumor directly invading another organ or tissue (invasion of other loops of the small intestine, invasion by 2 cm or more into the mesenterium and retroperitoneum, including invasion into the abdominal wall through the serosa; invasion to the pancreas only for duodenum)

N –Regional lymph nodes
NX : Regional lymph node metastasis cannot be evaluated
N0 : No regional lymph node metastasis

N1 : 1–3 regional foci of lymph node metastases

N2 : 4 or more regional foci of lymph node metastases

M –Distal metastasis

M0 : No distal metastasis

M1 : Distal metastasis present

Stage	T	N	M
**0**	Tis	N0	M0
**I**	T1, T2	N0	M0
**IIA**	T3	N0	M0
**IIB**	T4	N0	M0
**IIIA**	Any T	N1	M0
**IIIB**	Any T	N2	M0
**IV**	Any T	Any N	M1

3.4.4. Appendix (partial revision of UICC-TNM 7th Edition)

T –Primary tumor

TX : Primary tumor cannot be evaluated

T0 : Primary tumor unidentified

Tis : Epithelial carcinoma: Tumor invading epithelium or lamina propia mucosae

T1 : Tumor invading the submucosa

T2 : Tumor invading the muscularis propia

T3 : Tumor invading the subserosa or mesoappendix

T4 : Tumor penetrating the visceral peritoneum, tumor including a peritoneal mucosal tumor in the lower right abdomen and/or tumor directly invading other organs or tissue

T4a : Tumor penetrating the visceral peritoneum, or peritoneal mucosal tumor in the lower right abdomen

T4b : Tumor directly invading other organs or tissues

N –Regional lymph nodes

NX : Regional lymph node metastasis cannot be evaluated

N0 : No regional lymph node metastasis

N1 : 1–3 regional foci of lymph node metastases

N2 : 4 or more foci of regional lymph node metastases

M –Distal metastasis

M0 : No distal metastasis

M1 : Distal metastasis present

M1a : Peritoneal metastasis beyond the lower right abdomen, including pseudomyxoma peritonei

M1b : Distal metastasis other than peritoneal metastasis

Stage	T	N	M
**0**	Tis	N0	M0
**I**	T1, T2	N0	M0
**IIA**	T3	N0	M0
**IIB**	T4a	N0	M0
**IIC**	T4b	N0	M0
**IIIA**	T1, T2	N1	M0
**IIIB**	T3, T4	N1	M0
**IIIC**	Any T	N2	M0
**IVA**	Any T	N0	M1a
**IVB**	Any T	N0	M1a
	Any T	N1, N2	M1a
**IVC**	Any T	Any N	M1b

3.4.5. Colon and rectum

T –Primary tumor

TX: Primary tumor cannot be evaluated

T0: Primary tumor unidentified

Tis: Carcinoma in situ: Tumor invading epithelium or lamina propia mucosae

T1: Tumor invading the submucosa

T2: Tumor invading the muscularis propia

T3: Tumor invading the subserosa, or tissue surrounding the colon or rectum without peritoneal covering

T4: Tumor penetrating the visceral peritoneum, and/or directly invading another organ or tissues

T4a: Tumor penetrating the visceral peritoneum

T4b: Tumor directly invading another organ or tissues

N –Regional lymph nodes

NX: Regional lymph node metastasis cannot be evaluate

N0: No regional lymph node metastasis

N1: 1–3 regional foci of lymph node metastases

N1a: 1 regional lymph node metastasis

N1b: 2–3 regional foci of lymph node metastases

N1c: Presence of tumor deposits, that is, satellite nodes in the subserosa or in the soft tissue surrounding colon or rectum without peritoneal covering, but with no regional lymph node metastasis

N2: 4 or more regional lymph node metastases

N2a: 4–6 regional foci of lymph node metastases

N2b: 7 or more regional foci of lymph node metastases

M –Distal metastasis

M0: No distal metastasis

M1: Distal metastasis present

M1a: Local metastasis to one organ (liver, lungs, ovaries, or lymph nodes other than regional lymph nodes)

M1b: 2 or more organs, or peritoneal metastasis

Stage	T	N	M
**0**	Tis	N0	M0
**I**	T1, T2	N0	M0
**II**	T3, T4	N0	M0
**IIA**	T3	N0	M0
**IIB**	T4a	N0	M0
**IIC**	T4b	N0	M0
**III**	Any T	N1, N2	M0
**IIIA**	T1, T2	N1	M0
	T1	N2a	M0
**IIIB**	T3, T4	N1	M0
	T2, T3	N2a	M0
	T1, T2	N2b	M0
**IIIC**	T4a	N2a	M0
	T3, T4a	N2b	M0
	T4b	N1, N2	M0
**IVA**	Any T	Any N	M1a
**IVB**	Any T	Any N	M1b

3.4.6. Applies to hepatic NEC (hepatic primary lesion or liver metastasis from unknown primary lesion)

T –Primary tumor

TX: Primary tumor cannot be evaluated

T0: Primary tumor unidentified

Tis: Carcinoma *in situ*

T1: Isolated tumor without vascular invasion

T2a: Isolated tumor with vascular invasion

T2b: Multifocal tumor regardless of vascular invasion

T3: Tumor penetrating visceral peritoneum or directly invading adjacent extrahepatic structures

T4: Tumor with bile duct invasion (bile duct proliferative type)

N –Regional lymph nodes

NX: Regional lymph node metastasis cannot be evaluated

N0: No regional lymph node metastasis

N1: Regional lymph node metastasis present

M –Distal metastasis

M0: No distal metastasis

M1: Distal metastasis present

Stage	T	N	M
**I**	T1	N0	M0
**II**	T2	N0	M0
**III**	T3	N0	M0
**IVA**	T4	N0	M0
	Any T	N1	M0
**IVB**	Any T	Any N	M1

3.4.7. Gall bladder

T –Primary tumor

TX: Primary tumor cannot be evaluated

T0: Primary tumor unidentified

Tis: Carcinoma *in situ*

T1: Tumor invading mucosal lamina propria or muscularis externa

T1a: Tumor invading mucosal lamina propria

T1b: Tumor invading muscularis externa

T2: Tumor invading the connective tissue around the muscularis externa, but shows no progression beyond the serosa or to the liver

T3: Tumor perforating the serosa (visceral peritoneum), tumor directly advancing to the liver and/or an adjacent organ that is not the liver (stomach, duodenum, colon, pancreas, omentum, or extrahepatic bile duct)

T4: Tumor invading the main trunk of the portal vein or the hepatic artery, or tumor advancing to two or more adjacent organs which are not the liver

N –Regional lymph nodes

NX: Regional lymph node metastasis cannot be evaluated

N0: No regional lymph node metastasis

N1: Regional lymph node metastasis present (cystic duct, common bile duct, proper hepatic artery, including lymph nodes along the portal vein)

M –Distal metastasis

M0: No distal metastasis

M1: Distal metastasis present

Stage	T	N	M
**0**	Tis	N0	M0
**I**	T1	N0	M0
**II**	T2	N0	M0
**IIIA**	T3	N0	M0
**IIIB**	T1, T2, T3	N1	M0
**IVA**	T4	Any N	M0

**IVB**	Any T	Any N	M1

3.4.8. Extrahepatic bile duct-hepatic portal region

T –Primary tumor

TX: Primary tumor cannot be evaluated

T0: Primary tumor unidentified

Tis: Carcinoma *in situ*

T1: Tumor localized to the bile duct which advances until muscularis externa or fibrous tissue

T2a: Tumor invading beyond the bile duct wall and into the surrounding adipose tissue

T2b: Tumor invading the adjacent liver parenchyma

T3: Tumor invading the branch of one side of portal vein or hepatic artery

T4: Tumor invades the main trunk of portal vein, and branches on both sides of the portal vein, proper hepatic artery or the secondary branches of the bile duct on both left and right side, or to the secondary bile duct branches on one side and portal vein or hepatic artery on the other side

N –Regional lymph nodes

NX: Regional lymph node metastasis cannot be evaluated

N0: No regional lymph node metastasis

N1: Presence of regional lymph node metastasis in the cystic duct, common bile duct, proper hepatic artery, and lymph nodes along portal vein

M –Distal metastasis

M0: No distal metastasis

M1: Distal metastasis present

Stage	T	N	M
**0**	Tis	N0	M0
**I**	T1	N0	M0
**II**	T2a, T2b	N0	M0
**IIIA**	T3	N0	M0
**IIIB**	T1,T2, T3	N1	M0
**IVA**	T4	N0, N1	M0
**IVB**	Any T	Any N	M1

3.4.9. Extrahepatic bile duct-distal

T –Primary tumor

TX: Primary tumor cannot be evaluated

T0: Primary tumor unidentified

Tis: Carcinoma *in situ*

T1: Tumor localized in the bile duct wall

T2: Tumor invading beyond the bile duct wall

T3: Tumor invading the gallbladder, liver, pancreas, duodenum, or other adjacent organs

T4: Tumor invading the celiac axis or superior mesenteric artery

N –Regional lymph nodes

NX: Regional lymph node metastasis cannot be evaluated

N0: No regional lymph node metastasis

N1: Regional lymph node metastasis present

M –Distal metastasis

M0: No distal metastasis

M1: Distal metastasis present

Stage	T	N	M
**0**	Tis	N0	M0
**IA**	T1	N0	M0
**IB**	T2	N0	M0

**IIA**	T3	N0	M0
**IIB**	T1, T2, T3	N1	M0
**III**	T4	Any N	M0
**IV**	Any T	Any N	M1

3.4.10. Ampulla of Vater

T –Primary tumor

TX : Primary tumor cannot be evaluated

T0 : Primary tumor unidentified

Tis : Carcinoma *in situ*

T1 : Tumor localized to the ampulla of Vater, or sphincter of Oddi

T2 : Tumor invading the duodenal wall

T3 : Tumor invading the pancreas

T4 : Tumor invading the soft tissue around the pancreas or other adjacent organs

N –Regional lymph nodes

NX : Regional lymph node metastasis cannot be evaluated

N0 : No regional lymph node metastasis

N1 : Regional lymph node metastasis present

M –Distal metastasis

M0 : No distal metastasis

M1 : Distal metastasis present

Stage	T	N	M
**0**	Tis	N0	M0
**IA**	T1	N0	M0
**IB**	T2	N0	M0
**IIA**	T3	N0	M0
**IIB**	T1, T2, T3	N1	M0
**III**	T4	Any N	M0
**IV**	Any T	Any N	M1

3.4.11. Pancreas

T –Primary tumor

TX : Primary tumor cannot be evaluated

T0 : Primary tumor unidentified

Tis : Carcinoma *in situ*

T1 : Tumor localized within the pancreas, with a maximum size of < 2 cm

T2 : Tumor localized within the pancreas, with a maximum size > cm

T3 : Tumor advancing outside the pancreas, but with no invasion to the celiac axis or superior mesenteric artery

T4 : Tumor invading the celiac axis or superior mesenteric artery

N –Regional lymph nodes

NX : Regional lymph node metastasis cannot be evaluated

N0 : No regional lymph node metastasis

N1 : Regional lymph node metastasis present

M –Distal metastasis

M0 : No distal metastasis

M1 : Distal metastasis present

Stage	T	N	M
**0**	Tis	N0	M0
**IA**	T1	N0	M0
**IB**	T2	N0	M0
**IIA**	T3	N0	M0

**IIB**	T1, T2, T3	N1	M0
**III**	T4	Any N	M0
**IV**	Any T	Any N	M1

3.5. Residual tumor (R) classification (UICC-TNM 7th Edition)

RX: Presence of residual tumor cannot be evaluated

R0: No residual tumor

R1: Presence of residual tumor by microscopic examination

R2: Presence of residual tumor by macroscopic examination

3.6. Definition of non-resectable NEC

Unoperated cases for which surgery as a curative process was not indicated based on clinical findings, including image-based diagnosis, or operated cases that underwent resection (including experimental laparotomy) but ultimately ended as R2 resection are considered non-resectable.

The details by organs are shown below. One or more criterion must be fulfilled for each organ.

<Esopghagus>

- Diagnose as Stage IV (UICC 7th edition) based on clinical findings.

<Stomach>

- Diagnose as Stage IV (UICC 7th edition) based on clinical or surgical findings. However, this does not include situations where Stage IV diagnosis is provided based only on peritoneal lavage cytology (CY1).
- Bulky lymph node* metastasis is identified.

  *Bulky lymph node: Two or more lymph nodes with a major axis of 1.5 cm or more, that are present in contact with each other around the celiac artery, the common hepatic artery, the splenic artery, and the proper hepatic artery, or on the front surface of the superior mesenteric vein, where single or multiple large and small lymph nodes have formed an aggregate with the overall major axis length of 3.0 cm or more.

<Duodenum (excluding the ampulla of Vater), small intestines, appendix, and colon>

- Diagnosed as Stage IV (UICC 7th edition) based on the clinical findings.

<Extrahepatic bile duct, ampulla of Vater, gallbladder>

- Distant metastasis is identified
- Para-aortic lymph node metastasis is identified
- Bulky metastasis identified in the hepatoduodenal mesentery and lymph nodes surrounding the head of the pancreas
- Invasion in the proper hepatic artery, common hepatic artery, celiac artery, or the superior mesenteric artery identified
- Invasion in the hepatic artery branches on both the left and right side identified
- Extensive invasion or occlusion of the main trunk of portal vein, or invasion in the portal vein branches on both the left and right side identified
- Invasion in the blood vessels on the lobe on one side of the liver (portal vein or artery) or atrophy of lobe on one side of liver identified, and advancement into the bile duct on the other side is identified up to the secondary branch level
- Advancement into the bile ducts on both sides identified up to the secondary branch level

<Pancreas>

- Diagnosed as Stage III or Stage IV (UICC 7th edition) based on clinical findings.

＜Hepatic NEC (hepatic primary lesion or liver metastasis from unknown primary lesion)>

- Distal metastasis* other than intrahepatic metastasis identified.

  *Limited to organs that cannot be the primary lesion site (bone, lymph nodes below the diaphragm,

peritoneum, subcutaneous, muscle, and spleen) (see 3.8)

- Para-aortic lymph node metastasis is identified
- Bulky metastasis identified in the hepatoduodenal mesentery and lymph nodes surrounding the head of the pancreas
- Multiple masses identified in the liver.
- Invasion in one of the proper hepatic artery, common hepatic artery, celiac artery, or the superior mesenteric artery identified.
- Invasion to hepatic artery branches on both the left and right side identified
- Extensive invasion or occlusion of the main trunk of portal vein, or invasion in portal vein branches on both the left and right side identified
- Invasion in blood vessels on lobe on one side of the liver (portal vein or artery) or atrophy of lobe on one side of liver identified, and advancement into the bile duct on the other side is identified up to the secondary branch level
- Advancement into the bile ducts on both sides identified up to the secondary branch level

3.7. Definition of recurrent NEC

If surgery (R0 resection or R1 resection) was performed for gastrointestinal/hepatobiliary or pancreatic primary lesion, malignant tumor and the condition was diagnosed as NEC based on pathological samples taken from surgery, and were deemed as a clinical relapse thereafter.

If there are past histological samples from the primary lesion, no histological biopsy of the relapse lesion is necessary.

3.8. Definition of hepatic NEC (hepatic primary lesion or liver metastasis from an unknown primary lesion)

After a detailed investigation into the primary lesion by cervical-pelvic contrast CT, gastrointestinal endoscopy, FDG-PET, otolaryngology (head and neck examination), urology examination (for men only) and gynecological examination (for women only), if the tumor is in one of the following sites, the case is defined as hepatic primary lesion (or primary lesion unknown).

- Tumor is present only in the liver
- Tumor is present in an organ that cannot be a primary lesion site (bone, lymph nodes below the diaphragm, peritoneum, subcutaneous, muscle, and spleen), and in the liver.

4. Patient selection criteria

Patients that fulfill all of the following inclusion criteria and do not correspond to any of the exclusion criteria are eligible for enrollment.

4.1. Inclusion criteria (for enrollment)

1) Any of the following is applicable based on pathological diagnosis taking findings of immunohistochemistry into consideration (see 3.1. to 3.3.).

[1] Pathologically diagnosed as neuroendocrine carcinoma (NEC*1) in the resected sample.

[2] Containing pathologically confirmed component of neuroendocrine carcinoma (NEC*1) in the biopsy sample.

1: Based on WHO 2010 classification

2) Any of the following is applicable

[1] NEC arise in esophagus, stomach, duodenum, intestine, appendix, colon, rectum, gallbladder, intrahepatic bile duct, extrahepatic bile duct, ampulla of Vater, pancreas,

[2] Liver NEC (primary liver or liver metastasis of unknown primary) *2.

*2: The tumor is only in one of the following sites after a thorough examination of the primary site by contrast CT (from the neck to pelvic) and upper/lower gastrointestinal endoscopy, FDG-PET scan, otolaryngology (head and neck) examination, urology examination (male patients only), and gynecology examination (female patients only).

a. Liver only

b. Bone, lymph nodes below the diaphragm, peritoneum, subcutaneous, muscle, spleen, and liver

3) Unresectable (see 3.6) or recurrent cancer (see 3.7). It is not essential for a pathological confirmation of the metastatic lesion or recurrent site. Cases of esophageal NEC is ineligible if corresponding to any of the following.

[1] cT4.

[2] No distant metastasis rather than supraclavicular lymph node

[3] Stenosis indicated for palliative radiotherapy

4) No previous chemotherapy or radiotherapy for NEC. Pre- or post-operative chemotherapy except irinotecan or etoposide for NEC is allowed as long as it was completed at least 8 weeks prior to registration.

5) No previous chemotherapy using platinum agents for any malignancies.

6) No serious tumor-related complications.

Serious tumor-related complications include superior vena cava syndrome, inferior vena cava syndrome, pleural, ascites, or pericardial effusions that are large or uncontrollable (e.g., effusions that accumulate quickly after drainage or even after adhesive surgery), and brain metastases with neurological symptoms.

7) Aged 20 to 75 years old.

8) ECOG performance status of 0 or 1.

9) Sufficient oral intake

10) Measurable region is not required.

11) Adequate organ functions.

[1] WBC ≥3,000/ mm^3

[2] Neutrophils ≥ 1,500/ mm^3

[3] Hemoglobin ≥ 9.0 g/dL

[4] Platelets ≥ $10×10^4$/ mm^3

[5] Total bilirubin≤ 1.5 mg/dL※4

[6] AST(sGOT)≦100 IU/L※4（for hepatic NEC and liver metastasis, ≦150 IU/L）

[7] ALT(sGPT)≦100 IU/L※4（for hepatic NEC and liver metastasis, ≦150 IU/L）

※4：Presence or absence of biliary drainage is not relevant

[8] Serum creatinine≦ 1.3 mg/dL

[9] Creatinine clearance※5≥ 60 mL/min

※5： Creatinine clearance must have been estimated using the Cockcroft-Gault formula, and must be 60 mL/min or more. If the estimation is less than 60 mL/min, but the actual measurement is 60 mL/min or more, the patient can be deemed eligible.

Cockcroft-Gault formula

Male：Ccr ={(140－age) × body weight (kg)}/{72×serum creatinine (mg/dL)}

Female：Ccr = 0.85×{(140－age) × body weight (kg)}/{72×serum creatinine(mg/dL)}

12) Written informed consent.

4.2. Exclusion criteria

1) Synchronous or metachronous (within 5 years) malignancies except carcinoma in situ or intramucosal tumor curatively treated with local therapy.

2) Active infection requiring systemic therapy.

3) Fever of 38 degrees Celsius or higher.

4) Pregnant or lactating women, women of childbearing potential, or women within 28 days after delivery.

5) Psychiatric disease.

6) Patients requiring systemic steroids medication.

7) Interstitial pneumonia, pulmonary fibrosis.

8) Serious co-existing illness.

9) Unstable angina pectoris within 3 weeks, or with a history of myocardial infarction within 6 months.

10) Impossible to use both iodine and gadolinium due to being allergic to contrast agent.

11) Uncontrolled diabetes mellitus or routine administration of insulin.

5. Registration and randomization

5.1. Procedure of registration

Ensure that a patient to be registered meets all eligibility criteria and does not meet any of exclusion criteria and register the patient by using JCOG Web Entry System. JCOG Web System Personal Account and password are required for web registration. If unknown, contact JCOG Data Center.

Patient registration JCOG Web Entry System
URL:https://secure.jcog.jp/dc/
(Web registration can be used for 24 hours.)

Contact information for patient registration and JCOG Web Entry System

JCOG Data Center
TEL: 03-3542-3373
Weekdays 9:00-17:00 (not available in holidays, Saturdays and Sundays, New Year's holidays)
E-mail:JCOGdata@ml.jcog.jp

Contact information on Patients Selection Criteria

Study Coordinator Contact : Chigusa Morizane
Hepato-Biliary Pancreatic Group: Futomi Mori (Main Research Secretariat)
Department of Hepatobiliary and Pancreatic Medicine, National Cancer Center
TEL:03-3542-2511
FAX:03-3542-3815
E-mail:cmorizan@ncc.go.jp

Gastric Cancer Group: Yoshinori Machida
Department of Gastroenterology, Kanagawa Cancer Center (Gastrointestinal Tract)
TEL:045-520-2222
FAX:045-520-2202
E-mail:n-machida@kcch.jp

Esophageal Cancer Group: Ken Kato/Yushi Homma
Department of Gastrointestinal Oncology, National Cancer Center
TEL: 03-3542-2511
FAX: 03-3542-3815
E-mail: kenkato@ncc.go.jp / yohonma@ncc.go.jp

5.1.1. Precautions for patient registration

① Registration after initiation of protocol treatment is unacceptable.
② Registration is performed by accessing the URL in '5.1. Procedure of registration'.
③ Eligibility checks are performed on the screen of Registration Form, so it is not necessary to send a Registration Form to Data Center by mail or fax.
④ If input data are insufficient, registration is not accepted until all are met.
⑤ The registration number is issued after the confirmation of eligibility on the registration screen, then the registration is completed.
⑥ Once registered, patients will not be retracted (retracted from the database) unless there is withdrawal of consent, including refusal to use the data for research. For duplicate registration, the information at the initial registration (registration number, allocated arm) are used in any case.
⑦ When misregistration or duplicate registration is found, contact Data Center immediately.
⑧ Body surface area and drug dose calculations are institutional responsibilities, and the body surface area and drug dose displayed on Web Entry System at registration are only for double-checking. Those should

always be calculated and checked at the institution. When the body surface area calculation formula adopted in the hospital information system of the institution differs from calculation formula adopted by JCOG (Dubois formula: Body surface area (m^2) = Body weight (kg) $^{0.425}$ x Height (cm) $^{0.725}$ 84 ÷ 10,000), there can be a difference in the dose by the hospital information system of the institution and the dose by the calculation formula adopted by JCOG, but in that case, either dosage used is decided by the site investigator.

5.2. Randomization and allocation adjustment factor

During enrollment, the treatment arm for a patient is allocated randomly by the data center.

Randomization would use a method of minimization using the 1) institutions, 2) Primary lesion organ (gastrointestinal tract [esophagus, stomach, duodenum, small intestines, appendix, colon, and rectum] vs. hepatobiliary and pancreatic organ [hepatic NEC, gallbladder, extrahepatic bile duct, ampulla of Vater, or pancreas]) as the adjustment factors, so that there are no large discrepancies between them. Researchers at participating facilities would not be informed of the detailed procedures of randomization.

6. Treatment Plan and Treatment Modification Criteria

Unless patient safety is threatened, treatment and treatment modifications is done in compliance with the specifications in this chapter.

If it is considered that the protocol specification may cause medically dangerous situation of the patient, treatment modifications should be made according to the medical judgment of the investigators/sub-investigators. Such protocol deviation is considered to be "clinically relevant deviation" if considered medically appropriate (see 14.1.4.Protocol deviation/violation). Deviations that occur with intentions other than safety, such as increasing efficacy, are not considered clinically relevant deviations.

6.1. Protocol treatment

Protocol treatment is initiated within 7 days of enrollment.

If treatment initiation occurs after 8 days from registration for any reason, the reason should be documented on the Treatment Course Form. If it is determined that treatment cannot be initiated, describe the details in the Off-treatment Form as Protocol Treatment Termination.

When laboratory parameters worsen and eligibility criteria are no longer met by the start of treatment after registration, the investigator/sub-investigator is allowed to decide whether initiate or terminate protocol treatment at their own discretion.

「6.3. The course initiation criteria is not applied at treatment changes.

Drugs used

- Etoposide ※
- Cisplatin ※
- Irinotecan ※
- Entecavir, tenofovir disoproxil fumarate, tenofovir alafenamide fumarate

The use of generic drugs is not restricted.

※ The company that manufactures or distributes these drugs, or intends to manufacture or sell these drugs, requires conflicts of interest control in the Clinical Trials Act as a company involved in this study (see 13.8.).

6.1.1. Arm A: Etoposide plus cisplatin (EP) therapy

The following chemotherapy courses will be given once a week for 3 weeks and treatment will be continued until the patient meets the discontinuation criteria.

Drug	Dosage(mg/m^2)	Dosing regimen/Dosing time	Dose day
Etoposide	100	IV/60-120 min	Day 1、2、3
Cisplatin	80	IV/60-120 min	Day 1

1) Calculation of the dosage

① Body surface area is calculated by determining drug dose to the third decimal point.

② For both etoposide and cisplatin, the calculated dose is determined by truncating the decimal point. For drug doses, ±10% is the acceptable range.

③ Dose recalculation due to body weight change after treatment initiation is not performed.

2) Administration of anticancer drugs

Etoposide and cisplatin are given in any order.

Examples of administration in the package insert are described below.

(i) Before administration: Before administration of anticancer drugs, 1,000-2,000 mL of hydration is done to achieve adequate diuresis.

(ii) Etoposide: Etoposide is mixed with infusion solutions such as isotonic sodium chloride solution of 250 mL or more, and infused intravenously in about 60-120 minutes. DEHP (2-ethylhexyl) phthalate: di-(2-ethylhexyl) phthalate), which is a plasticizer, elutes from polyvinyl chloride infusion sets and catheters. Avoid the use of polyvinyl chloride infusion sets and catheters.

(iii) Cisplatin administration: Cisplatin is mixed with stock solution or 500 mL of saline (or glucose-saline) and given intravenously in 60-120 min. During the administration, caution should be exercised in ensuring urine volume, and diuretics such as mannitol and furosemide should be administered as

necessary.

(iv) After administration: After administration of cisplatin, 1,000-2,000 mL of hydration should be done so that adequate diuresis can be achieved.

In addition, short hydration administration at the discretion of each institution is permitted. (see Table 6.1.1. for treatment cases).

Table 6.1.1. Example of short hydration

	Dose	Time
5HT3 antagonist<br>Dexamethasone<br>Physiological saline	9.9 mg<br>50 mL	15 min
Etoposide<br>Physiological saline	100 mg/m^2<br>250 mL	60 min
No. 1 solution<br>Potassium chloride<br>Magnesium sulfate	500 mL<br>10 mEq<br>8 mEq	60 min
20% mannitol	200 mL	30 min
Cisplatin<br>Physiological saline	80 mg/m^2<br>250 mL	60 min
No. 1 solution<br>Potassium chloride	500 mL<br>10 mEq	60 min

(Referred from Horinouchi H, et al., Japan Society of Clinical Oncology 2012

3) Precautions for treatment

JCOG9511, an upfront trial in small-cell lung cancer, suggested an association between treatment-related deaths and first-course neutropenia. Therefore, caution should be exercised when neutropenia is strongly observed from the first course.

6.1.2. Arm B: Irinotecan plus cisplatin (IP) therapy

One 4-week course of the following chemotherapy will be continued until the discontinuation criteria are met. However, if day 15 irinotecan is skipped, 3 weeks should be used as one course, and the next course should be started with day 22.

Drug	Dosage(mg/m^2)	Dosing regimen/Dosing time	Dose day
Irinotecan	60	IV/90 min	Day 1、8、15
Cisplatin	60	IV/60-120 min	Day 1

1) Calculation of the dosage

(i) Body surface area is determined by determining the dose of drug administered until the third decimal point.

(ii) The dose is calculated for both irinotecan and cisplatin by truncating the decimal point. For drug doses, ±10% is the acceptable range.

(iii) Dose recalculation due to body weight change after treatment initiation is not performed.

2) Administration of anticancer drugs

Irinotecan and cisplatin are given in any order.

Examples of administration in the package insert are described below.

(i) Before administration: Before administration of anticancer drugs, 1,000-2,000 mL of hydration is done to achieve adequate diuresis.

(ii) Irinotecan: Irinotecan is mixed with 500 mL or more of isotonic sodium chloride solution, glucose solution, or electrolyte maintenance solution and given by intravenous drip infusion at about 90 minutes.

(iii) Cisplatin administration: Cisplatin is mixed with stock solution or 500 mL of saline (or glucose-saline) and given intravenously in 60-120 min. During the administration, caution should be exercised in ensuring urine volume, and diuretics such as mannitol and furosemide should be administered as necessary.

(iv) After administration: After administration of cisplatin, 1,000-2,000 mL of hydration should be done so that adequate diuresis can be achieved.

In addition, short hydration administration at the discretion of each institution is permitted. (See Table 6.1.1.

3) Precautions for treatment

JCOG9511, an upfront trial in small-cell lung cancer, suggested an association between treatment-related deaths and first-course neutropenia. Therefore, caution should be exercised when neutropenia is strongly observed from the first course.

6.2. Protocol Treatment Termination/Completion Criteria

6.2.1. Definition of protocol treatment completion

Protocol treatment is continued in this study unless the protocol treatment discontinuation criteria are met, so no definition of protocol treatment completion is provided.

6.2.2. Criteria for termination of protocol treatment

Protocol treatment is terminated in any of the following cases:

1) Judged as protocol treatment is ineffective
   - When a definite tumor exacerbation is confirmed by imaging or clinically
     ※ Protocol treatment should not be discontinued if the clinical judgement of PD based on the assessment of response based on imaging indicates that continuation of protocol treatment is appropriate, and protocol treatment should be continued.
2) Protocol treatment cannot be continued due to adverse events
   ① If Grade 4 non-hematological toxicity is observed (with the exception of the following adverse events) 'hypernatremia', 'hyponatremia', 'hyperkalemia', 'hypokalemia', 'hyperglycemia', 'hypoglycemia', 'alkaline phosphatase increase', 'alanine aminotransferase increase', 'aspartate aminotransferase increase', 'blood bilirubin increase','serum amylase increase', 'lipase increase', 'GGT increase'.
   (*Adverse events other than "anaemia," "myeloid cytopenia," "lymphocyte count decreased," "neutrophil count decreased," "white blood cell count decreased," "platelet count decreased," "CD4 lymphopenia" in CTCAE v4 0)
   ② If the next course cannot be initiated beyond 21 days from the expected start date of the course due to an adverse event
   ③ When the criteria for terminating protocol treatment in the treatment modification criteria (6.3.) are met.
   ④ Adverse events other than the treatment modification criteria that the investigator/sub-investigator judges to require termination of protocol treatment
3) If the patient offers termination of protocol treatment for reasons not denied to be associated with the adverse event
   - This category should be used if an association with an adverse event cannot be ruled out.
4) When the patient offers termination of protocol treatment because of reasons for denial of association with adverse events
   - Patient refusal after enrollment and before initiation of protocol treatment
   - When the association with an adverse event can first be denied, such as the relocation of the person or household member during protocol treatment.
5) Death during protocol treatment
   - Death before deciding to terminate protocol treatment for other reasons
6) Palliative surgery for pathogenic adverse events or surgery for patients with complete response to chemotherapy (see Section 6.3.9).
7) In addition, exacerbations before the start of treatment after enrollment (protocol treatment could not be initiated due to rapid exacerbation), protocol violations were found, ineligibility was determined due to modifications in pathological diagnosis after enrollment, etc., treatment was changed, and it was judged that it was difficult to continue protocol treatment due to social reasons and safety management problems, etc.

The date of discontinuation of protocol treatment is defined as the date of death in 6.2.2.5), the date of surgery in 6.2.2.6), and otherwise, the date on which the treating physician judges that protocol treatment is discontinued.

In this study, non-NEC may be diagnosed by central pathological diagnosis during protocol treatment, because central pathological diagnosis is performed. If the patient is continuing on protocol treatment when the results of the central pathology diagnosis are reported to the registry, the attending physician and the institutional pathologist will review the results and take a clinically appropriate response. Protocol treatment is discontinued if discontinuation of protocol treatment is judged to be appropriate, and the reason for discontinuation is other.

6.3. Treatment modification criteria

The following terms shall be used for the treatment modification.

Delay: Delay the start of the course or administration of treatment from the planned date.

Termination: Discontinuation of a part of or all of the treatment without restarting.

Suspending: temporary interruptions or withdrawals that may be resumed if conditions are met

Skip: Do not administer one or more drugs and proceed to the next schedule.

Categories of infection (CTCAEv4.0) used in this study are as follows

Infection: CTCAEv4.0 infections and infestations

Bronchial infection; pulmonary infection; upper respiratory tract infection; mediastinal infection; pleural infection; catheter-related infection; Biliary tract infection; Gallbladder infection; urinary tract infection

6.3.1. Arm A (EP-therapy): Dose level

1) Etoposide

Drug	Dose level	Dosing schedule	Dose day
Etoposide	Level 0	100 mg/m² div	Day 1、2、3
	Level -1	80 mg/m² div	Day 1、2、3
	Level -2	60 mg/m² div	Day 1、2、3

2) Cisplatin

Drug	Dose level	Dosing schedule	Dose day
Cisplatin	Level 0	80 mg/m² div	Day 1
	Level -1	60 mg/m² div	Day 1
	Level -2	40 mg/m² div	Day 1

6.3.2. A arm (EP therapy): Course initiation criteria

- Initiate the course after confirming that all of the following "Course Initiation Criteria" are met on the scheduled start date of the course or the day before the plan start date of the course.
- If any one is not met, the initiation of the course is delayed.
- If the course cannot be initiated within 21 days of the expected start date of the course (if the start date of the previous course was day 1 and the course could not be initiated by day 42), discontinue the protocol treatment.
- The course initiation criteria is not applied at the start of the first course.

Course initiation criteria

① Neutrophil count Grade 0-1 (≧1500 per mm³).

② Platelet count ≧10×10⁴ /mm³

③ AST≦100 IU/L (in the presence of hepatic metastases vs. hepatic NECs) was ≦200 IU/L).

④ ALT≦100 IU/L (≦200 IU/L for liver metastases vs liver NECs).

⑤ Total bilirubin <= 2.0 mg/dL

⑥ Serum creatinine <= 1.5 mg/dL

⑦ Fever Grade 0 (axillary temperature, no antipyretic)

⑧ Constipation, fatigue, phlebitis, oral mucositis, and infection ※1 are all Grade 2 or less.

Infected ※1: bronchial infection, pulmonary infection, upper respiratory tract infection, mediastinal infection, pleural infection, catheter-related infection, biliary tract infection, gallbladder infection, urinary tract infection

⑨ Anorexia, nausea, and emesis are all Grade 0-1.

⑩ Diarrhoeal Grade 0

6.3.3. Arm A (EP therapy): Dose reduction criteria

If any of the following toxicities are identified during the course, dose reduction should be performed in accordance with the dose reduction criteria (Table 6.3.3.) from the following course (no dose reduction in the course).

However, even if two or more items are met, the dose reduction for each drug is only one step. Re-escalation after dose reduction is not performed. Protocol treatment is terminated if the dose reduction criteria are met again after the dose reduction to Level-2.

Table 6.3.3. Arm A (EP therapy): Dose reduction criteria

**Item**	**Etoposide**	**Cisplatin**
Neutrophil count Grade 4 (<500 per mm^3).	Reduce the level by 1	No change
Platelet count Grade 4 (<$2.5\times10^4/mm^3$)	Reduce the level by 1	No change
1.5 <serum creatinine ≦2.0 mg/dL.	No change	Reduce the level by 1
Serum creatinine <= >2.0 mg/dL	Termination of protocol treatment	Termination of protocol treatment
Grade 3<br>Infected ※1	Reduce the level by 1	Reduce the level by 1
Grade 3<br>Assessment at onset of febrile.	Reduce the level by 1	Reduce the level by 1
Grade 2<br>Peripheral sensory neuropathy<br>Peripheral motor neuropathy,<br>Myalgia; arthralgia; tinnitus; hearing impairment	No change	Reduce the level by 1
Grade 3<br>Peripheral sensory neuropathy<br>Peripheral motor neuropathy,<br>Myalgia; arthralgia; tinnitus; hearing impairment	Termination of protocol treatment	Termination of protocol treatment
Non-hematologic toxicities of Grade 3 other than those listed above in ※2 that are causally related to EP-therapy (excluding hyponatremia, hypokalemia, hypomagnesemia, hypocalcemia, hyperglycemia, and weight loss)	Reduce the level by 1	Reduce the level by 1

※1:※1 of infection: bronchial infection, pulmonary infection, upper respiratory tract infection, mediastinal infection, pleural infection, catheter-related infection,
Biliary tract infection; Gallbladder infection; Urinary tract infection

※2:Causal relationship is judged as either of possible, probable, definite

6.3.4. Arm A (EP therapy): Within-course pause, skipping criteria

Following initiation of treatment with each course after the course initiation criteria are met, if any of the following adverse events are observed, day 2, day 3 etoposide will be suspended.

- Fever (axillary temperature) Grade 1-3
- Grade 3 of infection (bronchial infection, pulmonary infection, upper respiratory tract infection, mediastinal infection, pleural infection, catheter-related infection,
  Biliary tract infection, gallbladder infection, urinary tract infection)

Resting etoposide should be resumed after confirming that all initiation criteria are met until day 7. However, it should not be administered after day 8.

That is, if etoposide could not be administered by day 7, the remaining etoposide should be skipped.

If the above suspension and/or skip occur, the next course of etoposide should be started day 22 (after 3 weeks) counting from day 1 of the previous course if the ininiation criteria are met.

6.3.5. Arm B (IP therapy): Dose-level

1) Irinotecan

Drug	Dose level	Dosing schedule	Dose day
Irinotecan	Level 0	60 mg/m^2 div	Day 1、8、15
	Level -1	50 mg/m^2 div	Day 1、8、15
	Level -2	40 mg/m^2 div	Day 1、8、15

2) Cisplatin

Drug	Dose level	Dosing schedule	Dose day
Cisplatin	Level 0	60 mg/m^2 div	Day 1
	Level -1	50 mg/m^2 div	Day 1
	Level -2	40 mg/m^2 div	Day 1

6.3.6. Arm B (IP therapy): Course initiation criteria

- On the day of the initiation of the course or the day before the expected start of the course, start the course after confirming that all of the following "Course Initiation Criteria" are met.
- If any one is not met, the initiation of the course is delayed.
- If the course cannot be initiated within 21 days of the expected start date of the course (if the start date of the previous course was day 1 and the course could not be initiated by day 49), discontinue the protocol treatment.
- However, if day 15 irinotecan is skipped in the previous course, day 22 of the previous course is set as the scheduled start date of the next course regarded as one course per 3 weeks.
- The course initiation criteria is not applied at the start of the first course.

Course initiation criteria

① Neutrophil count Grade 0-1 (≧1500/mm^3).
② Platelet count ≧10×10^4 /mm^3
③ AST≦100 IU/L (≦200 IU/L for liver metastases versus liver NECs)).
④ ALT≦100 IU/L (≦200 IU/L for liver metastases vs liver NECs).
⑤ Total bilirubin <= 2.0 mg/dL
⑥ Serum creatinine <= 1.5 mg/dL
⑦ Fever Grade 0 (measured by axillary temperature, temperature <38°C without antipyretic use)
⑧ Constipation, fatigue, phlebitis, oral mucositis, and infection ※1 are all Grade 2 or less.
Infected ※1: bronchial infection, pulmonary infection, upper respiratory tract infection, mediastinal infection, pleural infection, catheter-related infection, biliary tract infection, gallbladder infection, urinary tract infection
⑨ Anorexia, nausea, and emesis are all Grade 0-1.
⑩ Diarrhoea Grade 0

6.3.7. Arm B (IP-therapy): Dosing criteria for day 8, day 15

After confirming that all of the following ①-③ are met, the second (day 8) or third (day 15) dose of irinotecan is administered. If day 8, day 15 dosing criteria are not met, skip day 8, day 15 dosing.

① All of the following are met with the most recent laboratory data on the scheduled day of administration or the day before the scheduled day of administration.
i) Neutrophil count Grade 0-2 (≧1000 per mm^3).
ii) Platelet count ≧10×10^4 /mm^3
iii) AST ≦ 100 IU/L (≦200 IU/L for liver metastases vs liver NECs).
iv) ALT ≦ 100 IU/L (≦200 IU/L in the presence of hepatic metastases versus hepatic NECs).
v) Total bilirubin <= 2.0 mg/dL
vi) Serum creatinine <= 2.0 mg/dL

② All of the following are met on the scheduled day of administration:
i) Fever Grade 0 (measured by axillary temperature, temperature <38°C without antipyretic use)

ii） Diarrhoeal Grade 0

③ Constipation, anorexia, nausea, emesis, fatigue, phlebitis, oral mucositis, and infection ※1 are all Grade 2 or less.

Infected ※1: bronchial infection, pulmonary infection, upper respiratory tract infection, mediastinal infection, pleural infection, catheter-related infection, biliary tract infection, gallbladder infection, urinary tract infection

6.3.8. Arm B (IP therapy): Dose reduction criteria

If any of the following toxicities are identified during the course, dose reduction should be performed in accordance with the dose reduction criteria (Table 6.3.8.) from the following course (no dose reduction in the course).

However, even if two or more items are met, the dose reduction for each drug is only one step. Re-escalation after dose reduction is not performed. Protocol treatment is terminated if the dose reduction criteria are met again after the dose reduction to Level-2.

Table 6.3.8. Arm B (IP therapy): Dose reduction criteria

Item	Irinotecan	Cisplatin
Neutrophil count Grade 4 (<500 per mm^3).	Reduce the level by 1	No change
Platelet count Grade 4 (< $2.5\times10^4/mm^3$)	Reduce the level by 1	No change
1.5 <serum creatinine ≦2.0 mg/dL.	No change	Reduce the level by 1
Serum creatinine <= >2.0 mg/dL	Termination of protocol treatment	Termination of protocol treatment
Grade 3<br>Infected ※1	Reduce the level by 1	Reduce the level by 1
Grade 3<br>Assessment at onset of febrile.	Reduce the level by 1	Reduce the level by 1
Grade2<br>Peripheral sensory neuropathy<br>Peripheral motor neuropathy,<br>Myalgia; arthralgia; tinnitus; hearing impairment	No change	Reduce the level by 1
Grade 3<br>Peripheral sensory neuropathy<br>Peripheral motor neuropathy,<br>Myalgia; arthralgia; tinnitus; hearing impairment	Termination of protocol treatment	Termination of protocol treatment
Non-hematologic toxicities of Grade 3 other than those listed above in ※2 that are causally related to IP-therapy (excluding hyponatremia, hypokalemia, hypomagnesemia, hypocalcemia, hyperglycemia, and weight loss)	Reduce the level by 1	Reduce the level by 1

※1:※1 of infection: bronchial infection, pulmonary infection, upper respiratory tract infection, mediastinal infection, pleural infection, catheter-related infection,

Biliary tract infection; Gallbladder infection; Urinary tract infection

※2:Causal relationship is judged as either of possible, probable, definite

6.3.9. Surgical of after end of chemotherapy

1) Surgery for Adverse Events Associated with Pathogenesis

Surgery may be performed if it is judged clinically desirable to perform surgery for newly appearing symptoms such as hemorrhage or stenosis. Protocol treatment is discontinued if surgery is performed, regardless of the content. In this case, the date of discontinuation of protocol treatment is the date of surgery.

2) Surgery for patients with complete response to chemotherapy

- When chemotherapy is highly effective, all metastases present at the time of enrollment on imaging studies disappear, and curative resection (R0 resection) is considered possible, resection including primary and metastatic disease may be performed.
- Surgical procedures are not specifically specified, but the Research Office will collect information on the details of the surgery performed individually in order to be reviewed by the research representative/research office.

- In all surgical cases, the group group conference shall confirm the validity of the judgment that surgery is indicated.
- When surgery is performed, protocol treatment is discontinued, regardless of whether curative resection was performed or not. In this case, the date of discontinuation of protocol treatment is the date of surgery.

6.3.10. Consultation on treatment modification

If there are any questions about treatment modification, contact "16.6. Study Coordinator".

Study Coordinator Contact : Chigusa Morizane

Hepato-Biliary Pancreatic Group: Futomi Mori (Main Research Secretariat)
Department of Hepatobiliary and Pancreatic Medicine, National Cancer Center
TEL:03-3542-2511
FAX:03-3542-3815
E-mail:cmorizan@ncc.go.jp

Gastric Cancer Group: Yoshinori Machida
Department of Gastroenterology, Kanagawa Cancer Center (Gastrointestinal Tract)
TEL:045-520-2222
FAX:045-520-2202
E-mail:n-machida@kcch.jp

Esophageal Cancer Group: Ken Kato/Yushi Homma
Department of Gastrointestinal Oncology, National Cancer Center
TEL:03-3542-2511
FAX:03-3542-3815
E-mail: kenkato@ncc.go.jp / yohonma@ncc.go.jp

6.4. Concomitant treatment and supportive care

6.4.1. Required concomitant treatment/supportive care

1) Testing and Supportive Care for HBsAg-Positive Cases.

In HBsAg-positive cases, steroids and chemotherapy can lead to rapid hepatitis B virus (HBV) expansion (reactivation: reactivation) and potentially fatal severe hepatitis. Therefore, the following tests and supportive care are performed based on the "Guidelines for the Treatment of Hepatitis B, Third Edition (Japanese Society of Hepatology)." It is advisable to consult a hepatologist at the time prior to initiation of a nucleic acid analogue (entecavir, tenofovir disoproxil fumarate, and tenofovir alafenamide fumarate).

①Testing prior to initiation of chemotherapy: HBV-DNA quantitation

HBV-DNA quantitative analysis should be performed at least once prior to initiation of chemotherapy.

HBV-DNA assays are performed by real-time PCRs.

HBeAg and HBe antibodies should also be measured in accordance with the Guidelines for the Treatment of Hepatitis B, Third Edition (Japanese Society of Hepatology).

②Dosing schedule for supportive care (nucleic acid analogues prophylaxis)

- **Drugs used :**
  - **Entecavir (Bristol-Myers: Baraclude Tablets 0.5 mg)**
  - **Tenofovir disoproxil fumarate (GlaxoSmithKline: Tenozet Tablets 300 mg)**
  - **Tenofovir alafenamide fumarate (Giliado: Vemuridi Tablets 25 mg)**

The following dosage regimen should be followed, starting at least 1 week before the start of chemotherapy (as soon as possible), and continuing for at least 12 months after the end of chemotherapy. However, fulminant hepatitis has been reported in HBsAg-positive patients with high viral load, even during NA prophylaxis, and it is desirable to reduce the viral load before starting immunosuppression/chemotherapy. Nucleic acid analogues may be discontinued after 12 months after completion of chemotherapy if conditions *1 and 2 for discontinuation of NAs are met. However, if the administration of a nucleic acid analogue is discontinued, consultation with a hepatologist is always obtained, and the administration is discontinued only if the hepatologist deems it appropriate.

※1 Requirements for discontinuation of nucleic acid analogues (entecavir, tenofovir disoproxil fumarate, tenofovir alafenamide fumarate): all of the following

1. The patient has been on NA for more than 2 years.
2. HBV-DNA assays are not sensitive to detect
3. Be negative for HBeAg

※2 Patient background requirements: All of the following

1. Both the treating physician and the patient have sufficiently understood that the hepatitis exacerbation is frequently observed after the nucleic acid analog withdrawal, and that there is a danger of becoming serious in the time.
2. Follow-up is possible after treatment cessation, and appropriate treatment is possible even if hepatitis recurs
3. It is judged that the liver fibrillation is slight and the hepatic reserve is good, and it is difficult to become serious even if the hepatitis is exacerbated.

(Adapted from Guidelines for the Treatment of Hepatitis B, the 3rd edition (Japanese Society of Hepatology))

Entecavir

- **Dosage regimen: Take this medicine on an empty stomach (2 hours after meals and more than 2 hours before the next meal).**
- **Dosage :**

Creatinine clearance (mL/min).	Dosage
50 or more	0.5 mg once daily
30 Beyond 50	0.5 mg once every 2 days
10 Beyond 30	0.5 mg once every 3 days
10 Less than	0.5 mg once every 7 days

- **Adverse drug reactions (incidence of all grades): nucleoside analog-naïve patients**

Diarrhea (6.0%), nausea (4.5%), constipation (3.7%), upper abdominal pain (3.0%), malaise (1.5%), nasopharyngitis (3.0%), muscle stiffness (2.2%), headache (3.0%), rash (incidence unknown), laboratory tests: elevated AST (3.7%), increased blood bilirubin (6.0%), increased blood amylase (10.4%), and increased lipase

(10.4%).Blood glucose increased (6.0%), blood lactate increased (6.7%), urine occult blood positive (4.5%), white blood cell count decreased (8.2%), and eosinophil count increased (0.7%). [Significant adverse reactions (incidence unknown)] Hepatitis worsened after completion of treatment, anaphylactoid symptoms, lactic acidosis, and severe hepatomegaly due to fatty liver

Tenofovir disoproxil fumarate

- **Dosage and administration: 300 mg is orally administered once daily.**
- **Dosage :**

Creatinine clearance (mL/min).	Dosage
50 or more	300 mg once daily
30 Beyond 50	300 mg once every 2 days
10 Beyond 30	300 mg once every 3 to 4 days
Hemodialysis	[注)] of 300 mg once every 7 days<br>Or 300 mg after completion of cumulative approximately 12 hours of dialysis<br>NOTE) After hemodialysis was performed. The pharmacokinetics in patients with creatinine clearance < 10 mL/min and not on hemodialysis have not been investigated.

- **Dosing Precautions:**

In the long-term administration of tenofovir disoproxil fumarate, attention should be paid to renal dysfunction, hypophosphatemia (including Fanconi syndrome), and decrease in bone mineral density. It is recommended that renal function and serum phosphorus should be measured regularly during tenofovir disoproxil fumarate administration.

- **Adverse reactions (incidence of all grades):**

Abnormal liver function tests (AST, ALT and γ-GTP increased, etc.) in 7 patients (4.9%), increased creatinine in 4 patients (2.8%), increased amylase, increased lipase and nausea in 3 patients each (2.1%), abdominal pain in 2 patients (1.4%), [major adverse reactions (incidence unknown)] renal dysfunction, renal failure, acute renal failure, proximal renal tubular dysfunction, Fanconi syndrome, severe renal dysfunction such as acute renal tubular necrosis, nephrogenic diabetes insipidus or nephritis, severe hepatomegaly due to lactic acidosis and fatty deposition (steatohepatitis), pancreatitis

Tenofovir alafenamide fumarate

- **Dosage and administration: 25 mg is orally administered once daily.**
- **Dosage :**

Creatinine clearance (mL/min).	Dosage
15 or more	25 mg once daily
Less than 15	Consider discontinuation

- **Dosing Precautions:**

In the long-term administration of tenofovir alafenamide fumarate, attention should be paid to renal dysfunction, hypophosphatemia (including Fanconi syndrome), and decrease in bone density. It is recommended that renal function and serum phosphorus should be measured periodically during tenofovir alafenamide fumarate administration.

- **Adverse reactions (incidence of all grades):**

Nausea and abdominal distension, headache, fatigue (≧1%), dyspepsia and diarrhea, flatus, upper abdominal pain, constipation, ALT increased, arthralgia, dizziness, insomnia, pruritus, rash (≧0.5% to <1%), [significant adverse reactions (incidence unknown)] renal dysfunction, renal failure, acute renal failure, proximal renal tubular dysfunction, severe renal impairment such as Fanconi syndrome, acute renal tubular necrosis, renal diabetes insipidus or nephritis, severe hepatomegaly due to lactic acidosis and fatty deposits (fatty liver)

③Monitoring: Quantitative analysis of HBV-DNA (during and after administration of nucleic acid analogues)

During nucleic acid analogue administration:

They are monitored every 4 weeks by both HBV-DNA quantitative analysis and liver function (ASTs, ALTs). However, if HBV-DNA level is less than 20 IU/mL (1.3 log IU/mL) during administration of nucleic acid analogues, it is acceptable to perform tests every 4 to 12 weeks.

After discontinuation of nucleic acid analogue administration:

Bearing in mind that reactivation may occur even after discontinuation of administration of a nucleic acid analogues, the patient should be consulted with a hepatologist, and the patient should be monitored for HBV-DNA determination and hepatic function (AST/ALT) every 4 weeks for at least 1 year after discontinuation of administration of a nucleic acid analogues. Subsequent follow-up will be decided after consulting a hepatologist.

2) Laboratory Teste and Supportive Care for HBsAg-Negative and HBc Antibody-Positive and/or HBs Antibody-Positive Cases.

HBV-DNA quantitative analysis should be performed at least once prior to initiation of chemotherapy. HBV-DNA assays are performed by real-time PCRs.

i) HBV-DNA ≧ 20 IU/mL (1. 3 log IU/mL) prior to initiation of chemotherapy

It has been clarified that HBV-DNA replicates persist at low levels in the livers and peripheral blood mononuclear cells when HBc or HBs are positive, even if they are HBs-Ag negative. It has been reported that reactivation of HBV and development of severe hepatitis are caused by the use of potent immunosuppressive agents even in such patients with previous infections.

If HBV-DNA ≧ 20 IU/mL (1.3 log IU/mL), the risk of HBV reactivation is judged to be as high as in HBsAg-positive cases, and prophylactic administration of nucleic acid analogues (entecavir or tenofovir disoproxil fumarate, tenofovir alafenamide fumarate) is administered. The following laboratory tests and supportive care are performed in accordance with the "Guideline for the Treatment of Hepatitis B, 3rd edition (Japanese Society of Hepatology)" with reference to the following for examination, dosage, and monitoring of supportive care before the start of chemotherapy.

However, these are not applicable if the HBs antibody alone is positive and the HBV vaccination history is obvious.

①Dosing schedule for supportive care (nucleic acid analogues prophylaxis)

According to the dosage and administration of nucleic acid analogues (entecavir, tenofovir disoproxil fumarate, tenofovir alafenamide fumarate) in "1) Test and supportive care for HBsAg positive cases". Same conditions for NA discontinuation.

② Monitoring: Quantitative analysis of HBV-DNA (during and after administration of nucleic acid analogues)

The intervals for monitoring during and after discontinuation of NA are in accordance with the provision of "1) Testing and supportive care for HBsAg-positive patients".

ii) HBV-DNA less than 20 IU/mL (1.3 log IU/mL) prior to initiation of chemotherapy

HBV-DNA quantitative analysis and hepatic function (AST, ALT) will be monitored, and nucleic acid analogues (entecavir, tenofovir disoproxil fumarate, tenofovir alafenamide fumarate) will be started when ≧20 IU/mL (1.3 log IU/mL) is achieved.

The Guidelines for the Treatment of Hepatitis B, the 3rd edition (Japanese Society of Hepatology) recommends monitoring with HBV-DNA quantitative analysis or high-sensitivity HBs antibodies during and after chemotherapy, depending on the risks of revitalization.

①Monitor:HBV-DNA quantitative analysis

HBV-DNA quantitative analysis should be performed every 4-12 weeks from the start of chemotherapy until at least 12 months after the end of chemotherapy.

If HBV-DNA level is more than 20 IU/mL (1.3 log IU/mL), administration of nucleic acid analogues should be started immediately in accordance with the Guidelines for the Treatment of Hepatitis B, the 3rd edition (Japanese Society of Hepatology). If HBsAg monitoring is positive for < 1 IU/mL (low positive), nucleic acid analogues should be administered after additional HBV DNA determinations of ≧ 20 IU/mL (1.3 log IU/mL).

It is advisable to consult a hepatologist at a time prior to initiation of NAs.

②Supportive care in reactivation

Nucleic acid analogues should be administered according to the supportive care described in i) When HBV-DNA prior to the initiation of chemotherapy is 20 IU/mL (1.3 log IU/mL) or more in 6.4.1.2). Once administration of nucleic acid analogues is started, nucleic acid analogues should be discontinued only if appropriate by the hepatologist.

6.4.2. Recommended/not recommended concomitant treatment/supportive care

The following concomitant treatment and supportive care are recommended. Even if it is not carried out, it is not regarded as protocol deviation,

1) Addressing Febrile Neutropenia.

① Assessment at onset of febrile neutropenia (FN).

a) If the count is less than 500 per mm^3, or is less than 1000 per mm^3 and is expected to be less than 500 per mm^3 within 48 hours, and if the axillary temperature is 37.5°C or higher (mouth temperature is 38.0°C or higher), a severity-risk assessment should be performed promptly and anti-virus treatment initiated as appropriate.

b) Severity risk assessment is performed with reference to Multinational Association for Supportive Care in Cancer(MASCC) scoring system [※1].

c) For initial evaluation, complete blood count including differential WHITE BLOOD CELL and platelet count, renal function (BUN, creatinine), electrolytes, liver function (transaminases, total bilirubin, alkaline phosphatase) tests, ≧2 sets of venous blood cultures prior to initiation of antimicrobials, one set of cultures from the catheter lumen and one set from the peripheral vein if a central venous catheter is in place, cultures of suspected infection areas, and chest x-rays if respiratory symptoms and signs are present.

d) When febrile neutropenia (FN) develops in a patient with a central venous catheter, blood cultures from the catheter and peripheral blood are performed, and catheter-related infections are considered if there is a time difference of more than 120 minutes in the positivity of both. If appropriate antimicrobial therapy does not improve after more than 72 hours, catheter should be removed. For infections caused by Staphylococcus aureus, Pseudomonas aeruginosa, Bacillus, fungi, and acid-fast bacilli, the catheter should be removed and appropriate antimicrobial therapy based on culture results should be performed.

② Antibiotic use

a) In high-risk patients, β-lactams with anti-Pseudomonas aeruginosa activity are administered intravenously as a single agent. However, other antimicrobials (aminoglycosides, fluoroquinolones, and/or vancomycin) may be added to a single agent in the initial regimen in patients with unstable or complicated conditions or when drug-resistant organisms are strongly suspected. Low-risk patients may be treated with antibiotics orally or intravenously, hospitalized, or with adequate evaluation, if appropriate, as outpatients.

b) Re-evaluation will be performed 3-4 days after initiation of antibiotics to investigate the continuation or change of antibiotics. In principle, antibiotics will be continued until the neutrophil count recovers to 500 cells per mm^3 or more.

c) Empiric antifungal therapy is recommended in high-risk patients who do not respond to 4-7 days of broad-spectrum antibiotics.

d) Fluoroquinolone prophylaxis is recommended in high-risk individuals with an expected neutrophil count of 100 cells per mm^3 or less lasting >7 days.

③ Therapeutic administration of G-CSF

For therapeutic administration of G-CSF during the incidence of FNs, refer to "6.4.5. 3) Therapeutic administration of G-CSF".

※1 Multinational Association for Supportive Care in Cancer (MASCC) scoring system.
(Adapted in part from the Practice Guideline for Febrile Neutropenia (FN) [Japanese Society of Medical Oncology]. *2)

Item	Score
Clinical manifestations (select one of the following * mark 3 sections)<br>*No symptoms<br>*Mild symptoms<br>*Moderate symptoms	 5<br>5<br>3
No decrease in blood pressure	5
No chronic obstructive pulmonary disease	4
Solid tumors, or hematopoietic tumors without a history of fungal infection	4
No dehydration symptoms	3
Patients with fever during outpatient management	3
Age < 60	2

The total score is up to 26 points. Twenty-one points or more are considered low risk and 20 points or less are considered high risk

※2 Since patients aged 20 years or older are subjects in this study, we deleted "Not applicable to patients younger than 16 years old" from the original edition of the Practice Guideline for Febrile Neutropenia (FN) [Japanese Society of Medical Oncology].

2) Nausea and vomiting

Regarding nausea and vomiting, antiemetics are positively administered according to Clinical Practice Guidelines for Antiemesis in Oncology [48], and fluid and electrolyte repletion are performed when oral intake is severely reduced.

3) Anorexia

If oral intake drops markedly, fluid and electrolyte supplements should be given as needed. Especially, in the cases with diabetes mellitus, the abnormality of blood sugar level and electrolyte is noticed.

4) Anemia, thrombocytopenia

If anaemia (haemoglobin < 8.0 g/dL) or thrombocytopenia (platelet count < 2×10^4/mm^3) is observed, blood should be transfused as appropriate at the discretion of the treating physician.

5) Diarrhea

Severe diarrhoea occasionally occurs with arm B (IP therapy arm) and is extremely dangerous when complicated by febrile neutropenia. Patients should be fully informed about toxicities and their management, and should be instructed to measure body temperature, especially when neutrophils counts are most decreasing, and to contact a physician or nurse immediately during fever and diarrhea. Nonsteroidal anti-inflammatory drugs may not cause fever to become overt, so unnecessary anti-inflammatory drugs are not given.

If irinotecan-induced diarrhea occurs, the following supportive measures are recommended:

High-dose loperamide hydrochloride therapy

① Loperamide hydrochloride was started after signs of diarrhea were observed.
② Initial dose of 4 mg followed by 2 mg/2 hours (4 mg/4 hours at night)
③ It is administered until watery stool does not appear for more than 12 hours.
④ Doses should not be given for more than 48 hours.

6) Precautions on the day of cisplatin administration

Aminoglycoside antibiotics, vancomycin, and nonsteroidal anti-inflammatory drugs are not administered on the day of cisplatin administration or are used with caution.

6.4.3. Acceptable concomitant treatment and supportive care

The following concomitant treatment and supportive care may be used as needed.

Concomitant use of drugs for the treatment of complications such as hypertension and diabetes mellitus and symptomatic drugs such as morphine may be performed, but this drug should be administered with caution when furosemide, piretanide and phenytoin are used. Oral antibiotics for febrile neutropenia prophylaxis may be given at the discretion of the investigator/sub-investigator. Bisphosphonate denosumab may be used in combination for bone metastases.

6.4.4. Unacceptable concomitant treatment and supportive care

None of the following treatments will be given during protocol treatment:

① Administration of anticancer drugs other than protocol treatment

② Radiation therapy

6.4.5. Granulocyte colony-stimulating factor (granulocyte-colony stimulating factor:G-CSF

※This study permits the use of G-CSF biogenerics (biosimilars).

1)※ of primary prophylaxis with G-CSF

※Primary prophylaxis: G-CSF administration before developing febrile neutropenia or prolonged neutropenia to prevent them during anticancer therapy.

Primary prophylaxis with G-CSF was not recommended at the beginning of the study in this study. However, since febrile neutropenia in group A was 21.4% in the late 2016 periodic monitoring report and 15.8% in the early 2017 periodic monitoring report (18.4% when the time of occurrence was April 2017 and SAE reports not reflected in the early 2017 periodic monitoring report were included), it was decided that the patient would meet at least the recommended grade B or higher (almost A) according to G-CSF Appropriate Use Guideline 2013 and JSMO Febrile Neutropenia (FN) Practice Guideline. Primary prophylactic administration of G-CSF was recommended in group A. However, it is not considered a protocol deviation even if it is not administered. Since it is practical to administer pegfilgrastim from the viewpoint of convenience, when the primary preventive administration of G-CSF is carried out in the actual medical field, the administration example of pegfilgrastim is shown below.

(Administration cases)

Group A: 3.6 mg of pegfilgrastim (genetical recombination) will be injected subcutaneously (once per chemotherapy course) between day 4(day 3's completion of etoposide treatment and day 7 at least 24 hours after the completion of etoposide treatment.

Since the safety of pegfilgrastim administered 14 days prior to the initiation of cancer chemotherapy and within 24 hours after completion of administration has not been established, the primary prophylactic administration of pegfilgrastim is not performed in group B where day 8 is administered the drug.

Table 6.4.5. Primary prophylactic administration of G-CSF should be performed according to the approved dosage and administration shown in the table below.

Drug	・・ Pegfilgrastim (arm A only)<br>・・ Filgrastim<br>・・ Naltograstim<br>・・ Lenograstim
Time of initiation	Twenty-four hours after completion of chemotherapy
Dosage Dosing regimen	・Pegfilgrastim (genetical recombination) at a dose of 3.6 mg subcutaneously once per chemotherapy course (group A only)<br>・Filgrastim: 50 μg per m^2 SC once daily or 100 μg per m^2 IV once daily<br>・Naltograstim: 1 μg/kg SC once daily or 2μg/kg IV once daily<br>・Lenograstim: 2 μg/kg SC once daily or 5μg/kg IV once daily
Timing of discontinuation (other than pegfilgrastim)	・If the neutrophil count reaches 5000 per mm^3 or more after the course, administration should be discontinued.<br>・If the neutrophil count recovers to ≧2000 cells per mm^3, there are no suspected infections, and the patient's safety is judged to be ensured by the responsiveness to the drug used, the drug should be discontinued or reduced.

2)Secondary prophylactic※ of G-CSF

※ Secondary prophylaxis: G-CSF prophylactic administration after once occurrence of febrile neutropenia or prolonged neutropenia to prevent febrile neutropenia or prolonged neutropenia from occurring again during anticancer therapy.

If febrile neutropenia occurs in the previous course, secondary prophylaxis with G-CSF after the subsequent course is recommended, even if dose reduction or schedule modification or antimicrobial therapy is considered to be associated with a lower risk of febrile neutropenia (group A). However, it is not considered a protocol deviation even if it is not administered. As it is practical to administer pegfilgrastim in terms of convenience when secondary prophylactic administration of G-CSF is carried out in real medical practice, the following examples of administration of pegfilgrastim are shown.

(Administration cases)

Group A: 3.6 mg of pegfilgrastim (genetical recombination) is injected subcutaneously (once per course of chemotherapy) between the time of completion of etoposide treatment of day 4(day 3 and day 7 of at least 24 hours.

However, the safety of pegfilgrastim administered 14 days prior to the start of cancer chemotherapy and 24 hours after the end of treatment has not been established, so secondary prophylactic pegfilgrastim administration is not performed in group B, where day 8 is given the drug.

Secondary prophylactic administration of G-CSF should be performed according to the approved dosage and administration shown in the table below.

Drug	・・ Pegfilgrastim (arm A only)<br>・・ Filgrastim<br>・・ Naltograstim<br>・・ Lenograstim
Time of initiation	・・ Pegfilgrastim (arm A only)<br>Twenty-four hours after completion of chemotherapy<br>・・ Filgrastim, nartograstim, and lenograstim<br>When neutrophil counts <1000 per mm^3 are observed
Dosage Dosing regimen	・Pegfilgrastim (genetical recombination) at a dose of 3.6 mg subcutaneously once per chemotherapy course (group A only)<br>・Filgrastim: 50 μg per m^2 SC once daily or 100 μg per m^2 IV once daily<br>・Naltograstim: 1 μg/kg SC once daily or 2μg/kg IV once daily<br>・Lenograstim: 2 μg/kg SC once daily or 5μg/kg IV once daily
Timing of discontinuation (other than pegfilgrastim)	・If the neutrophil count reaches 5000 per mm^3 or more after the course, administration should be discontinued.<br>・If the neutrophil count recovers to ≧2000 cells per mm^3, there are no suspected infections, and the patient's safety is judged to be ensured by the responsiveness to the drug used, the drug should be discontinued or reduced.

3) Therapeutic administration of G-CSF

Therapeutic administration of G-CSF should be performed according to the approved dosage and administration shown in the table below.

Time of initiation	・When the neutrophil count is less than 1000 per mm^3 and fever (in principle, > 38.0°C) is observed<br>・When neutrophil counts <500 per mm^3 are observed
Dosage Dosing regimen	・Filgrastim: 50 μg per m^2 SC once daily or 100 μg per m^2 IV once daily<br>・Naltograstim: 1 μg/kg SC once daily or 2μg/kg IV once daily<br>・Lenograstim: 2 μg/kg SC once daily or 5μg/kg IV once daily
Timing of discontinuation	・If the neutrophil count reaches 5000 per mm^3 or more after the course, administration should be discontinued.<br>・If the neutrophil count recovers to ≧2000 cells per mm^3, there are no suspected infections, and the patient's safety is judged to be ensured by the responsiveness to the drug used, the drug should be discontinued or reduced.

6.5. Post-study treatment

Treatment after discontinuation of protocol treatment and treatment after progression or recurrence after completion are not specified.

Treatment (cross over) with drugs included in treatment regimens in the unassigned groups may be used, but if the total dose of cisplatin is greater than 500 mg/m^2, careful attention should be given to accumulating toxicities such as peripheral sensory/motor neuropathy, hearing loss, and renal impairment. Patients should be carefully monitored with adequate risk explanation only if the benefits are apparently outweighed by the risks, such as those with persistent sensitivity to cisplatin and mild cumulative toxicity.

If primary analysis or interim analysis reveals the primary conclusions of the trial, the results of the study will be explained to patients enrolled in this study as needed, and the best treatment will be provided, taking into account the course of treatment of individual patients.

In addition, if the protocol treatment discontinuation criteria apply but clinically "protocol treatment continuation" is judged to be appropriate, consult the research office through the institutional research director or institutional coordinator rather than at the physician level as a general rule (except when time is not allowed). In agreement between the Research Secretariat and the Investigator/Institution Coordinator, decide whether to treat as a → after discontinuation of protocol treatment or to deviate and continue protocol treatment. The details of the consultation

with the Research Secretariat and the decision-making process should be provided in the comment column for the patient's end-of-treatment report and progress record. If continuing the protocol treatment with protocol deviation occurs frequently, the Study Coordinator should consider revising protocol treatment termination criteria using group meetings and group mailing lists, because it is considered that the protocol treatment termination criteria is clinically inappropriate in such situation.

7. Anticipated Adverse Events

7.1. Anticipated adverse reactions

Anticipated adverse reactions in this study are as follows:

7.1.1. Anticipated Adverse Drug Reactions with Drugs

Adverse drug reactions anticipated with protocol treatments and drugs used in protocol-specified tests are referred to the latest version of the drug package insert.

7.1.2. Anticipated adverse reactions in the standard treatment arm (arm A)

Table 7.1.2. Adverse events in the EP-therapy group at JCOG9511 (excerpt from the final analysis report)

Examination Items	Grade 0	Grade 1	Grade 2	Grade 3	Grade 4	Grade 3,4(%)	Grade4(%)	Total
White blood cells	2	5	30	35	5	51.9%	6.5%	77
Neutrophils	1	0	5	21	50	92.2%	64.9%	77
Hemoglobin	2	9	43	23	-	29.9%	-	77
Platelet	31	19	13	14	0	18.2%	0%	77
Total bilirubin	57	-	20	0	0	0%	0%	77
GOT	49	24	2	1	1	2.6%	1.3%	77
GPT	40	28	6	2	1	3.9%	1.3%	77
Creatinine	56	16	5	0	0	0%	0%	77
Oxygen tension	13	27	9	2	1	5.8%	1.9%	52
Nausea and vomiting	13	36	23	5	-	6.5%	-	77
Diarrhea	64	8	5	0	0	0%	0%	77
Oral cavity (stomatitis)	68	6	2	1	0	1.3%	0%	77
Infection	42	23	9	1	2	3.9%	2.6%	77
Hair loss (hair)	9	46	19	-	-	-	-	74
Fever (uninfected)	45	14	16	2	0	2.6%	0%	77
Perception (peripheral nerve)	66	10	1	0	-	0%	-	77
Rash	74	2	1	0	0	0%	0%	77

※: Use JCOG Toxicity Criteria.

7.1.3. Anticipated adverse reactions in the study treatment arm (Arm B)

Table 7.1.3. Adverse Events in the IP-Therapy Group in a JCOG9511 (Extracted from the Final Analysis Report)

Examination Items	Grade 0	Grade 1	Grade 2	Grade 3	Grade 4	Grade 3,4(%)	Grade 4(%)	Total
White blood cells	1	16	38	17	3	26.7%	4.0%	75
Neutrophils	1	8	17	30	19	65.3%	25.3%	75
Hemoglobin	7	10	38	20	-	26.7%	-	75
Platelet	56	6	9	1	3	5.3%	4.0%	75
Total bilirubin	59	-	16	0	0	0%	0%	75
GOT	40	30	5	0	0	0%	0%	75
GPT	35	30	7	3	0	4.0%	0%	75
Creatinine	56	15	4	0	0	0%	0%	75
Oxygen tension	13	20	5	1	1	5.0%	2.5	40
Nausea and vomiting	11	26	28	10	-	13.3%	-	75
Diarrhea	23	19	21	8	4	16.0%	5.3%	75
Oral cavity (stomatitis)	66	9	0	0	0	0%	0%	75

Infection	45	17	9	3	1	5.3%	1.3%	75
Hair loss (hair)	17	44	13	-	-	-	-	74
Fever (uninfected)	45	12	17	1	0	1.3%	0%	75
Perception (peripheral nerve)	71	4	0	0	-	0%	-	75
Rash	70	5	0	0	0	0%	0%	75

※: Use JCOG Toxicity Criteria.

Table 7.1.3. Adverse events in the IP-therapy group in b JCOG0509 (abstracted from the main analysis report)

Examination Items	Grade 0	Grade 1	Grade 2	Grade 3	Grade 4	Grade 3,4(%)	Grade 4(%)	Total
White blood cells	16	36	58	29	3	22.5	2.1	142
Neutrophils	6	10	43	51	32	58.5	22.5	142
Hemoglobin	20	24	65	24	9	23.2	6.3	142
Platelet	125	6	8	2	1	2.1	0.7	142
Total bilirubin	116	15	10	1	0	0.7	0	142
GOT	98	37	5	2	0	1.4	0	142
GPT	80	56	4	2	0	1.4	0	142
Creatinine	97	37	8	0	0	0	0	142
Anorexia	22	67	33	19	1	14.1	0.7	142
Nausea	30	67	36	9	0	6.3	0	142
Vomiting	89	38	10	5	0	3.5	0	142
Diarrhea	52	51	28	11	0	7.7	0	142
Mucositis (examination findings)-oral cavity	126	15	0	0	1	0.7	0.7	142
Hair loss	64	66	12	-	-	-	-	142
Onset of febrile neutropenia	127	-	-	14	1	10.6	0.7	142
Infection with Grade3-4 neutropenia -Bronchus	142	-	0	0	0	0	0	142
-Lung (pneumonia)	137	-	0	4	1	3.5	0.7	142
-Pharynx	142	-	0	0	0	0	0	142
-Upper respiratory tract-unclassifiable	141	-	1	0	0	0	0	142
-Bladder	142	-	0	0	0	0	0	142
-Kidney	142	-	0	0	0	0	0	142
-Urinary tract-subclassification impossible	142	-	0	0	0	0	0	142
Neuropathy: Sensory	127	13	2	0	0	0	0	142

Table 7.1.3.c Adverse events from a pilot trial of postoperative adjuvant chemotherapy with irinotecan plus cisplatin for high-grade neuroendocrine lung cancer (excerpt)

Examination Items	Grade 2	Grade 3	Grade 4	Grade 3,4(%)	Grade 4(%)	Total
White blood cells	17	7	0	17.5%	0%	40
Neutrophils	12	15	4	47.5%	10.0%	40
Hemoglobin	14	6	4	25.0%	10.0%	40
Platelet	2	0	0	0%	0%	40
Total bilirubin	0	0	0	0%	0%	40
GOT	0	0	0	0%	0%	40
GPT	1	0	0	0%	0%	40
Creatinine	0	0	0	0%	0%	40
Hyponatremia	0	5	0	12.5%	0%	40
Endotoxemia	3	1	0	2.5%	0%	40
Hypokalemia	0	4	0	10.0%	0%	40
Nausea	8	4	-	10.0%	-	40
Vomiting	4	2	0	5.0%	0%	40
Anorexia	2	0	-	0%	0%	40

Diarrhea	11	2	0	5.0%	0%	40
Fatigue	10	5	-	12.5%	0%	40
Infection	2	0	0	0%	0%	40

7.2. Anticipated Adverse Events Due to Pathogenesis

1) Esophageal primary
Esophageal bleeding; Esophageal pain; Esophageal stenosis; Esophageal obstruction; Esophageal perforation; Esophageal ulcer; Esophageal fistula; Hoarseness; Pharyngolaryngeal dysesthesia; Hypercalcemia of advanced disease; Tracheal stenosis; Tracheal obstruction; Esophageal anastomotic leakage; Recurrent laryngeal nerve palsy

2) Gastric primary
Gastric bleeding, upper gastrointestinal bleeding, gastric pain, gastrointestinal pain, back pain, nausea, vomiting, dyspepsia, gastroparesis, abdominal fullness, gastric stenosis, gastric obstruction, gastric perforation, duodenal bleeding, duodenal fistula, duodenal perforation, duodenal perforation, duodenal stenosis, gastrostomy, gastrointestinal fistula, gastrointestinal fistula, ileus, gastric anastomotic leak, gastrointestinal anastomotic leak

3) Small intestin/Colon and rectum
Bleeding from tumor; Ileus; Duodenal bleeding; Duodenal obstruction; Duodenal perforation; Duodenal stenosis; Small bowel obstruction; Small bowel stenosis; Small bowel ulcer; Small bowel perforation; Small bowel fistula; Vaginal fistula; Colonic fistula; Colonic stenosis; Colonic obstruction; Colonic perforation; Rectal stenosis; Rectal obstruction; Rectal fistula; Rectal perforation; Rectal fistula; Jejunal perforation; Jejunal perforation; Ileostomy; Ileovesical fistula; Intestinal fistula; Anal pain; Small bowel anastomotic leakage; Large bowel anastomotic leakage; Rectal anastomotic leakage; Pelvic infection

4) Pancreas, biliary tract, and liver
Body weight loss; Fever; Pancreatitis; Cholecystitis; Cholecystic obstruction; Gallbladder pain; Liver failure; Biliary tract infection; Hemorrhage from biliary tract; Duodenal hemorrhage; Duodenal fistula; Duodenal obstruction; Duodenal perforation; Duodenal stricture; Anastomotic ulcer; Biliary anastomosis leak; Pancreatic anastomosis leak; Anastomotic hemorrhage; Anastomotic stricture; Hemorrhage from tumor; Hemorrhage from portal vein stenosis/obstruction; Symptoms associated with portal vein stenosis/obstruction (Portal hypertension, Hepatic failure; Esophageal variceal hemorrhage; Gastritis; Ascites); Back pain; Abdominal pain

5) Anticipated Adverse Events Due to Metastasis
Liver failure, hepatic pain, fever, hepatic infection, respiratory failure, pulmonary infection, superior vena cava syndrome, abdominal distention, abdominal distention, ileus, esophageal obstruction, reverse smoking, gastric obstruction, duodenal obstruction, small bowel obstruction, colonic obstruction, rectal obstruction, rectal stenosis, anal bleeding, anorectal infection, nausea, diarrhea, constipation, ureteral obstruction, and urinary retention,urethral infection, urinary tract infection, bile duct obstruction, biliary obstruction, cholecystitis, biliary hemorrhage, gallbladder pain, pancreatitis, pain, narrowing of luminal organs near metastatic sites (tracheal/gastrointestinal tract, etc.), tracheal obstruction, bronchial stenosis, penetration with adjacent organs, perforation, hoarseness, pleural effusion, chest wall pain, pleural pain, atelectasis, hypercalcemia, disseminated intravascular coagulation, fracture, ataxia, cerebrovascular ischemia, intracranial hemorrhage,headache, dizziness, decreased level of consciousness, aphasia, seizures, spasticity

6) Paraneoplastic syndrome, etc.
(due to incompatible secretory syndrome) hyponatremia, (due to ectopic ACTH syndrome) personality changes, hypertension, hypokalemia, hyperglycemia, Lambert-Eaton myasthenic syndrome, subacute cerebellar degeneration associated with autoantibody production (including ataxia, dysarthria, and nystagmus of limbs), paraneoplastic encephalomyelitis, sensory neuropathy (including dementia, cranial nerve symptoms, dizziness, ataxia, autonomic ataxia, transverse paralysis, and sensory disturbance), thromboembolism, (due to gastrointestinal stenosis) aspiration, anaemia, tumor pain, acute renal failure, myositis, and pulmonary fibrosis

Complications associated with etiolation and gastrointestinal stent insertion are shown below.

- Percutaneous transhepatic cholangiodrainage (PTCD) procedures, including internal-external fistula tube placement:
  Pancreatitis, bile duct stricture, cholecystitis, gallbladder obstruction, gallbladder pain, liver failure, biliary

tract infection, liver infection, cholecystitis, septicemia, hemorrhage, PTCD tubing obstruction/deviation, peritoneal infection, pneumothorax, pleural pain, pleural effusion, intrapleural hemorrhage

- Biliary stenting:
  Pancreatitis, biliary stricture, cholecystitis, gallbladder obstruction, gallbladder pain, liver failure, biliary tract infection, liver infection, cholecystitis, sepsis, hemorrhage, peritoneum infection, pneumothorax, pleural pain, pleural effusion, intrapleural hemorrhage (in case of percutaneous placement), duodenal perforation, pneumonitis (in case of transendoscopic placement), stent obstruction/deviation, duodenal ulcer, and duodenal hemorrhage
- Choledochojejunostomy:
  Biliary tract infection; Pancreatitis; Cholecystitis; Liver infection; Sepsis
- Gastrointestinal stent insertion:
  Bleeding, perforation, pain, stent deviation, stent obstruction, ulceration, fever, sepsis, infection, diarrhea, constipation, urgency (colorectal), thyroid injury (esophagus), jugular arteriovenous injury (esophagus), and mediastinal abscess (esophagus).

7.3. Evaluation of Adverse Events/Reactions

The Common Terminology Criteria for Adverse Events v4.0 Japanese Translated JCOG Version (Japanese translation of NCI-Common Terminology Criteria for Adverse Events v4.0 (CTCAE v4.0)) (CTCAE v4.0-JCOG) will be used to assess adverse events/reactions. For CTCAE v4 0-JCOG in which Grade is defined by laboratory reference values at the institutional reference value, the "JCOG sharing reference range" will be used instead of the institutional reference value at each medical institution. For more information on JCOG sharing reference ranges, see JCOG website (see http://www.jcog.jp/doctor/tool/kijun.html)).

7.3.1. Grading of s adverse events

In grading of adverse events, each grading is closest to the definitions of Grade 0 4 (nearest match). Grading to a higher Grade when the definition of more than one Grade is comparable and when it is difficult to decide on either (highest grade).

Grading should also be given to Grade if specific actions are described, due to their clinical need. For example, patients may refuse oxygen inhalation or chest drainage, even when the patient's pleural effusion is increasing and oxygen inhalation or chest drainage is indicated. In such cases, grading is based on the medical judgment of what should have been done (what should be done) rather than on whether the treatment was actually given (what was actually done).

In the event of treatment-related deaths, original NCI-CTCAE states that the causative adverse event should be Grade 5, but the outcome of the serious adverse event is reported in the SAE report and reviewed in detail. Therefore, Grade 5 of the institutional physician's judgment is not likely to be changed, and whether or not the serious adverse event will result in death will be significantly affected by other factors than the event is not appropriate, so it is not appropriate to compare the frequency of the adverse event by Grade (%Grade 4 and%Grade 5, respectively) between treatment groups or between studies. Because of the poor significance of distinguishing between Grade 4 and Grade 5 in the tabulation, Grade 4 is not considered "Grade 5" in the recording form of this study. A discussion of the causal relationship between adverse events observed in treatment-related deaths and deaths should be included in the "Situation at Death" section of the treatment completion report form and follow-up form, and an urgent report should be made. For the adverse event items specified in "8.2. Testing and Assessment during Treatment" and "8.3. Testing and Endpoints after Treatment Completion" that are determined to be Grade 5 in the post-hoc review including the emergency report, Grade and the date of the first occurrence of the event should be included in the relevant record form (Treatment Course Record Form). If Grade 3 or greater is observed for any other adverse event, or if Grade 3/2/1 adverse event and treatment requires at least 24 hours of hospital stay or prolongation of hospital stay (see 10.1.1.3.)), the AE and Grade and the date of first occurrence should be included in the free form of the treatment course record.

Any Grade on the record form should be recorded in the medical record. Confirmed during site visit audit.

7.3.2. Determination of the causal relationship between adverse events and treatment

In determining the causal relationship between adverse events and treatment, patients are classified into 5 categories of "definite, probable, possible, unlikely, unrelated". Each "causality" is defined as "causality" when judged to be either "definite, probable, possible" and "no causality" when judged to be either "unlikely, unrelated" (see TABLE 7.3.2.

According to Grade of adverse events, serious adverse events requiring expedited reporting as specified in "10.1. Serious Adverse Events and Expedited Reporting" should be reported to the Research Secretariat in accordance with "10.2. Mandatory Reporting and Reporting Procedures of the Investigator".

Table 7.3.2. Criteria for a Causal Relationship Between Adverse Events and Treatment

	Determination	Approach to determination
Cause<br>Fruit<br>Concern<br>Associate<br>Ah<br>Fistula	Definite	The AE is clearly related to the intervention<br>Adverse events are apparently caused/aggravated by protocol treatment and are unlikely to be due to exacerbation of the etiology or other factors (comorbidities, other medications/treatments, or incidents).
	Probable	The AE is likely related to the intervention<br>It is unlikely that the adverse event was caused/aggravated by progression of the underlying pathology or other factors (comorbidity, other drugs/treatments, incident) and is likely to be due to protocol treatment.
	Possible	The AE may be related to the intervention<br>It is plausible (plausible) that an adverse event is considered to have occurred/become more severe with protocol treatment, and unlikely to be due to exacerbation of the etiology or other factors (comorbidities, other medications/treatments, or incidents).
Cause<br>Fruit<br>Concern<br>Associate<br>In<br>Pouch	Unlikely	The AE is doubtfully related to the intervention<br>It is considered plausible (plausible) that the adverse event is due to exacerbation of the pathogenic disease or other factors (comorbidity, other drugs/treatments, incident) rather than to the protocol treatment that it is caused/aggravated.
	Unrelated	The AE is clearly NOT related to the intervention<br>It is judged that the adverse event was caused/aggravated by aggravation of the pathogenic disease or other factors (comorbidity, other drugs/treatments, incident) and is unlikely to be caused by protocol treatment.

8. Examination and Evaluation

8.1. Baseline examination and evaluation before registration

8.1.1. Test conducted before registration (regardless of time before registration)

1） Histopathology (immunostaining requires chromogranin A and synaptophysin) (see Section 3.3)

2） HBs antigen; HBc antibody ※1; HBs antibody ※1, HBV-DNA※2

※1: For HBsAg positive, HBc and HBs antibodies are not required, and HBV-DNA, HBeAg, and HBe antibodies are measured.

※2: Positive results for at least one of the HBc and HBs antibodies also indicate HBV-DNA prior to initiation of therapy (see Section 6.4.1).

8.1.2. Test performed within 56 days before enrollment (liver primary (or unknown primary))

If the liver is primary (or of unknown primary), the following tests should be performed (all allowing for tests performed in other hospitals):

1）Upper gastrointestinal endoscope

2）Lower gastrointestinal endoscope

3）FDG-PET test

4）Otolaryngology (head and neck) examination

5）Urology consultation (male only)

6）Gynecologic exam (female only)

8.1.3. Tests performed within 28 days before enrollment

1) Contrast-enhanced CT※1 (slice thickness of 5 mm or less, if the patient is allergic to iodine), both contrast-enhanced MRI of the abdomen and plain computed tomography (CT) of the imaging range, which is considered to be indispensable below, are performed. All tests are not performed in other hospitals.

※The following areas are indispensable for each primary organ, and if there is another site suspected of metastasis, the radiographic extent is added accordingly.

Primary Organ	Essential radiographic area
Esophagus	Cervical, chest, or abdominal regions
Stomach, duodenum, small intestine, colon, appendix, rectum,<br>Extrahepatic bile duct, Vater ampulla, gallbladder, and pancreas	Chest, abdomen, and pelvis
Hepatic NEC<br>(liver primary or liver metastasis of unknown primary)	Neck, chest, abdomen, and pelvis

2) Endoscopic ※2

Primary Organ	Mandatory test ranges
Esophagus, stomach, duodenum, and ampulla of Vater	Upper gastrointestinal tract (no examination performed at another hospital)
Small intestine, extrahepatic bile ducts, gallbladder, and pancreas	Be not mandatory
Colon, appendix, and rectum	Lower gastrointestinal tract (no examination performed at another hospital)
Hepatic NEC<br>(liver primary or liver metastasis of unknown primary)	8.1.2. Refer to the test performed within 56 days before registration.

※2 Unnecessary if the primary lesion has been resected

3) 12-lead, resting electrocardiography

8.1.4. Tests performed within 14 days before enrollment

1) General condition: PS (ECOG), body weight

2) Physical findings

3) Peripheral blood count: white blood cell count, neutrophil count (ANC: rod + segmented karyocyte), hemoglobin, platelet count

4) Blood biochemistry: total protein, albumin, total bilirubin, AST (GOT), ALT (GPT), BUN, creatinine, LDH, ALP, sodium, potassium, calcium, magnesium, CRP, FBS (fasting blood glucose)
5) Creatinine clearance (CCr): CCr estimates by Cockcroft-Gault equation
   Cockcroft-Gault formula
   Male: Ccr = {(140-Age) × Body Weight (kg)}/{72 × Serum Creatinine Level (mg/dL)}
   Women: Ccr = 0.85 × {(140-Age) × Body Weight (kg)}/{72 × Serum Creatinine Level (mg/dL)}
6) Tumour markers: NSEs, ProGRP, CEAs, SCCs (esophagus primary only), CA19-9 (non-esophageal primary only)
7) Chest X-P (frontal) (substitutable if contrast-enhanced and plain chest CT is obtained)

8.2. Examination and evaluation during treatment

The following safety examination and evaluation are minimal in frequency: Performing examinations more frequently at the discretion of the treating physician is not prohibited.

However, the examination for efficacy evaluation should be performed at specified frequencies, unless progression is suspected, because dense frequency may lead to bias in the efficacy evaluation.

8.2.1. Safety endpoint assessed weekly (CTCAE v4 0 Japanese translation)

The following 1)-3) tests or evaluations should be performed at least weekly. In addition, all of the following items should be checked and evaluated on the scheduled date or the day before anticancer drug administration. However, at least weekly examinations or evaluations should be performed in the same manner until day 28 using the starting date of the last course as day 1, even when the treatment is completed or terminated.

1） Peripheral blood count: white blood cell count, neutrophil count (rod + segmented count), hemoglobin, and platelet count
2） Biochemical tests: albumin, total bilirubin, AST (GOT), ALT (GPT), creatinine, sodium, potassium, calcium, magnesium, CRP
3） Subjective and objective findings (described according to CTCAE v4.0JAPANESE TRANSLATION)
   - General disorders and administration site conditions: fever, fatigue
   - Ear and labyrinth disorders: Tinnitus, hearing impairment
   - Skin and subcutaneous tissue disorders: alopecia
   - Gastrointestinal disorders: Constipation, diarrhea, nausea, vomiting, oral mucositis
   - Metabolism and nutrition disorders: anorexia, dehydration
   - Nervous system disorders: dysgeusia, peripheral sensory neuropathy, peripheral motor neuropathy
   - Musculoskeletal and connective tissue disorders: myalgia, arthralgia
   - Infections and parasites: bronchial infection, pulmonary infection, upper respiratory tract infection, mediastinal infection, pleural infection, catheter-related infection, biliary tract infection, gallbladder infection, and urinary tract infection
   - Blood and lymphatic system disorders: Febrile Neutropenia
   - Vascular disorders: Phlebitis
   - Respiratory, thoracic and mediastinal disorders: dyspnea, hypoxia, pneumonitis

8.2.2. Safety examination and evaluation for each course

1) General condition: Body weight
2) Blood chemistry: LDH, ALP FBS (fasting glucose)

8.2.3. Safety examination and evaluation to be performed as necessary

1） When dyspnea is observed
   - Chest X-P, percutaneous oxygen saturation: SpO2, arterial blood gases: PaO_2
2） If an arrhythmia is observed
   - 12-lead, resting electrocardiography
3） When HBs antigen, HBs antibody, or HBc antibody is positive
   - HBV-DNA (see 6.4.1.).

8.2.4. Efficacy end point

The following tests will be performed every 6 weeks during protocol treatment (±1 week allowed: 6 ± 1 week, 12 ± 1 week, and 18 ± 1 week after the start date of protocol treatment). Tumor response will be assessed according to "11.1. Response Evaluation". Evaluation of the response will be performed using the same test conditions and test methods as the baseline evaluation.

If CT is allergic to contrast material, it is evaluated with plain CT and/or contrast-enhanced MRI. Allergy to contrast media on MRI is assessed by plain CT or plain MRI. When the use of CT contrast medium and MRI contrast medium becomes difficult due to renal dysfunction, it is evaluated by simple CT or simple MRI.

1） Tumour markers: NSEs, ProGRP, CEAs (≧all cases), SCCs (esophagus primary only), CA19-9 (non-esophageal primary only)

2） Contrast-enhanced CT: In principle, the extent of imaging is as follows. () Areas in can be omitted if there is no lesion

Primary Organ	Essential radiographic area
Esophagus	Cervical, chest, or abdominal regions
Stomach, duodenum, small intestine, colon, appendix, rectum,<br>Extrahepatic bile duct, Vater ampulla, gallbladder, and pancreas	(chest), abdomen, and pelvis
Liver NEC (liver primary or liver metastasis of unknown primary)	(neck), (chest), abdomen, and pelvis

8.3. Examination and evaluation after completion of treatment

8.3.1. Efficacy evaluation after completion of treatment

After completion of the protocol treatment, examination and evaluation are made at the following timing:

The following tests should be performed at least every 6 weeks until progressions are confirmed or death.

If progressions are observed, only observation of the outcome will be continued.

1） Tumour markers: NSEs, ProGRP, CEAs (≧all cases), SCCs (esophagus primary only), CA19-9 (non-esophageal primary only)

2） Contrast-enhanced CT: In principle, the extent of imaging is as follows. Areas in parentheses can be omitted if there is no lesion

Primary Organ	Essential radiographic area
Esophagus	Cervical, chest, or abdominal regions
Stomach, duodenum, small intestine, colon, appendix, rectum,<br>Extrahepatic bile duct, Vater ampulla, gallbladder, and pancreas	(chest), abdomen, and pelvis
Liver NEC (liver primary or liver metastasis of unknown primary)	(neck), (chest), abdomen, and pelvis

8.4. Information on post- study treatment

After completion/termination of protocol treatment, the following items will be recorded on the Follow-up Form at each follow-up survey:

1） Content of post-study treatment (if post-study treatment is performed)

2） After protocol treatment termination, the initiation date of the first post-study treatment ((if post-study treatment is performed)

3） PS at the start of post-study treatment

8.5. Study calendar

If the primary site is the esophagus, stomach, duodenum, small intestine, colon, appendix, rectum, extrahepatic bile duct, ampulla of Vater, gallbladder, or pancreas

	Before registratikon Record Pre	After end of chemotherapy		From the date of protocol treatment discontinuation Within 28 days	Until progression after completion of protocol treatment	
		Course Before initiation of the course	During the course		Only at the start of post-treatment	Every 6 weeks
Physical findings						
Body weight	○[14]	○				
PS	○[14]				■	
Physical findings	○[14]	○			■	
Laboratory tests						
WBC, differential (neutrophil) Hb, platelets	○[14]	○	●	●		
Alb, T-Bil, AST, ALT, Cr, Na, K, Ca, Mg, CRP	○[14]	○	●	●		
LDH, ALP, FBS	○[14]	○				
Total protein, BUN, Ca	○[14]					
NSE, ProGRP、CEA	○[14]		△			
SCC (esophagus primary only), CA19-9 (other than primary esophageal)	○[14]		△			
HBs antigen, HBc antibody, and HBs antibody	○[前]					
Chest X-P (can be substituted if CT is taken)	○[14]					
12-lead, resting electrocardiography	○[28]					
Upper gastrointestinal endoscope (Primary: esophagus, stomach, duodenum)	○[28]					
Lower gastrointestinal endoscope (Primary: colonic, appendix, rectum)	○[28]					
Efficacy evaluation						
Contrast-Enhanced CT*	○[28]		△			△
Toxicity evaluation						
Subjective symptom check		○	●	●		
Objective symptom check		○	●	●		
Submission of CRFs						
Pre-treatment Form		□				
Treatment Form			□			
Off-treatment Form				□		
Follow-up Form					2 times/year	

○[前]: Conduct before registration, ○[28]: Perform within 28 days before registration, ○[14]: Perform within 14 days before registration

○:Conduct, ♀: Implementation at least once a week

△: Every 6 weeks (see 8.2.4.), ∞: Only once at the beginning of aftertreatment, □: Submitted.

*See 8.1.3 for the shooting range. Contrast-induced allergy or renal dysfunction is assessed by plain CT or contrast-

enhanced MRI.

※Follow-up Forms will be sent up to 1 years after completion of accrual and will be submitted after 1 years of registration in the individual patient according to the closing date of registration.

For liver NEC (liver primary or liver metastasis of unknown primary)

	Before registratikon Record Pre	After end of chemotherapy		From the date of protocol treatment discontinuation Within 28 days	Until progression after completion of protocol treatment	
		Course Before initiation of the course	During the course		Only at the start of post-treatment	Every 6 weeks
Physical findings						
Body weight	○^{14}	○				
PS	○^{14}				■	
Physical findings	○^{14}	○			■	
Laboratory tests						
WBC, differential (neutrophil) Hb, platelets	○^{14}	○	●	●		
Alb, T-Bil, AST, ALT, Cr, Na,K, Ca, Mg, CRP	○^{14}	○	●	●		
LDH, ALP, FBS	○^{14}	○				
Total protein, BUN, Ca	○^{14}					
NSE, ProGRP、CEA、CA19-9	○^{14}		△			
HBs antigen, HBc antibody, and HBs antibody	○^{前}					
Chest X-P (can be substituted if CT is taken)	○^{14}					
12-lead, resting electrocardiography	○^{28}					
Upper gastrointestinal endoscope	○^{56}					
Lower gastrointestinal endoscope	○^{56}					
FDG-PET	○^{56}					
Otolaryngologic examination	○^{56}					
Urology consultation (male only)	○^{56}					
Gynecologic exam (female only)	○^{56}					
Efficacy evaluation						
Contrast-Enhanced CT*	○^{28}		△			△
Toxicity evaluation						
Subjective symptom check		○	●	●		
Objective symptom check		○	●	●		
Submission of CRFs						
Pre-treatment Form		□				
Treatment Form			□			
Off-treatment Form				□		
Follow-up Form					□ 2 times/year	

○^{前}: Conduct before registration, ○^{56}: Perform within 56 days before registration, ○^{28}: Perform within 28 days before registration,

○^{14}: Implemented within 14 days prior to enrollment

○:Conduct, ♀: Implementation at least once a week

△: Every 6 weeks (see 8.2.4.), ∞: Only once at the commencement of post-treatment, □: Submitted.

*See 8.1.3 for the shooting range. Contrast-induced allergy and renal dysfunction are evaluated by plain CT or contrast-enhanced MRI.

※Follow-up Forms will be sent up to 1 years after completion of accrual and will be submitted after 1 years of

registration in the individual patient according to the closing date of registration.

9. Data collection

9.1. Case Report Form (CRF）

9.1.1. Types of CRF and submission deadlines

The case report forms (CRF) used in this study and their submission deadlines are as follows:

1) Pre-treatment report (blue)- Less than 2 weeks after enrollment
2) Treatment course records- Every 2 cycles/less than 2 weeks after end of protocol treatment
   3）-1 Treatment (yellow)
   3）-2 Test (yellow)
   3）-3 Adverse events (yellow)
3) Tumor shrinkage report (green) - Less than 2 weeks after judgment of effect
4) End of treatment report (red) - Less than 2 weeks after discontinuation/end of protocol treatment
5) Follow-up investigation (white)- By the deadline indicated in the Follow-up Investigation Form

- For "1) Pre-treatment report to 4) End of treatment report", CRFs with basic patient information (enrollment code, facility name) pre-printed on them would be sent by post from the data center. If the CRFs do not arrive within one week of enrollment, or if the CRFs have been lost/damaged, the data center should be contacted by telephone, and a request should be made for them to be re-issued.
- "5) Follow-up investigation" would be sent by post from the data center at the time of follow-up investigations that are conducted at the same time as monitoring and interim/final analyses at the data center.

9.1.2. Storage CRF

- Completed CRFs must all be archived at the facility as photocopies or in electronic form.
- Copies of CRFs should be kept archived until the final analysis report is issued for reference while filling other CRFs, or for review while retrieving information from the data center.

9.1.3. Method of sending CRF

- All CRF must be sent by post or handed over in person at the data center. They must not be sent by FAX.
- To avoid the risk of personal patient information being leaked, the patient enrollment code should be used, instead of using the patient medical chart number at the facility, when contacting the data center for request of CRF dispatch.

9.1.4. Correction of the contents of CRFs

If any data necessary for the CRF are found to be missing or there are inappropriate category classifications after the start of the study, the CRFs may be corrected with the agreement of the head of the data center and the clinical trial secretariat, in a manner not exceeding the scope of data collected as prescribed in "8. Evaluation item/Clinical laboratory tests/Evaluation schedule", and within the scope deemed not to increase medical and financial burden on the enrolled patient from the CRF correction. Modification of CRFs that do not require the main body of the protocol to be revised is not considered a protocol revision by JCOG. Reports to the head of the medical institution related to CRF correction and the request of application for revision should follow the rules of the facility.

10. Reporting of "disease or the like"(adverse events)

Site investigator should report to Study Coordinator/Principal Investigator (Study Chair) if a serious adverse event ("disease or the like" on Clinical Trials Act) occurs in accordance with the regulations of Clinical Trials Act (Law No. 16, 2017), Enforcement Regulations of Clinical Trials Act (MHLW Notification No. 17, 2018) and the regulations in this chapter based on the relevant notifications.

The most recent version of the report is available on the MHLW website [1)] and on the JCOG website [2)]. Use the most recent version of the report.

Serious adverse events occurring after the initiation of protocol treatment (after the date of registration if death) by the date of final follow-up will be subjects.

1）http://www.mhlw.go.jp/stf/seisakunitsuite/bunya/0000163417.html
2）http://www.jcog.jp/doctor/todo/researcher/harmfulness.html
3）http://www.pmda.go.jp/safety/reports/hcp/pmd-act/0002.html

10.1. Serious Adverse Events and subjects of Expedited Reporting.

Serious adverse events are defined as any of the following:
(These are classified as "disease or the like" on Clinical Trials Act.)

1） Death
2） Diseases that may lead to death
3） "Disease or the like" requiring hospitalization or prolongation of hospital stay for treatment.
4） Disability
5） "Disease or the like" that may lead to disability
6） Serious "disease or the like" according to 1) to 5)
7） Congenital disease or abnormality in later generations

Of these, those that fall under any of the following categories shall be regarded as serious adverse events for which Expedited Reporting is mandatory.

1) Death

(i) All deaths that occur after registration and before the start of protocol treatment
(ii) All deaths (with or without causality to protocol treatment) that occur during protocol treatment or within 30 days of the last treatment day
(iii) Death that occur after 31 days from the last treatment date that are causally related to protocol treatment (definite, probable, possible)

2) "Disease or the like" that may lead to death

(i) Grade 4 adverse events that occur during protocol treatment or within 30 days of the last treatment day (excluding events in Table 10.1)
(ii) Grade 4 adverse events that occur after 31 days from the last treatment date (excluding events in Table 10.1) that are causally related to the protocol treatment (definite, probable, possible)

3) "Disease or the like" requiring hospitalization or prolongation of hospital stay※1 for treatment

(i) Grade 3/2/1 adverse events that occur during or within 30 days of protocol treatment and requiring at least 24 hours of hospitalization or prolongation of hospital stay※1 to treat the adverse event (excluding the event in Table 10.1).
(ii) Grade 3/2/1 adverse events that occur 31 days after the last treatment day and requires 24-hour or longer hospitalization or prolongation of hospital stay※1 for treatment and causally related to protocol treatment (definite, probable, possible) (excluding the events in Table 10.1)

※ 1 "Hospitalization or prolongation of hospital stay" refers only to those for which hospitalization of at least 24 hours or prolongation of hospital stay is medically required for the treatment of an adverse event. The followings are not subjects for reporting:

- Hospitalization or prolongation of hospital stay performed for follow-up of adverse event that has disappeared or improved

- Hospitalization or prolongation of hospital stay for reducing patient burden, e.g. patients from distant areas.
- Hospitalization or prolongation of hospital stay for other medically unnecessary situation

※ 2 "Unexpected" refers to those not listed in "7. Expected Adverse Events"

4) Disability, 5) "Disease or the like" that may lead to disability

Permanent or marked disability/dysfunction (excluding myelodysplastic syndromes, secondary cancers, etc.) or possible medical situation

6) Serious "disease or the like" similar to 1) to 5)

7) Congenital disorders or abnormalities in later generations

Table 10.1. Adverse events excluded from the subjects of Expedited Reporting

SOC※(CTCAE ver4.0)	AE term
Blood and lymphocyte disorders	Anemia, bone marrow hypocellular
Gastrointestinal disorders	Constipation
General disorders and local symptoms	Fever
Infections and infestations	Viral hepatitis
Clinical laboratory test	ALP increased, CD4 lymphocytes decreased, high cholesterol, GGT increased, lipase increased, lymphocytes decreased, neutrophils decreased, platelet count decreased, serum amylase increased, WBC decreased, hyponatremia, hypokalemia, hyperglycemia, and hypoglycemia
Metabolism and nutritional disorders	Obesity, anorexia, hyperuricemia, and hypoalbuminemia
Musculoskeletal and connective tissue disorders	Fibrosis deep connective tissue and superficial soft tissue fibrosis
Renal and urinary disorders	Chronic kidney disease
Respiratory, thoracic, and mediastinal disorders	Sinus disorder and sleep apnea
Skin and subcutaneous tissue disorders	Hypohidrosis

※ SOC:System Organ Class

10.2. Investigator's reporting requirements and procedures

10.2.1. Expedited Reporting

In the event of a serious adverse event for which Expedited Reporting is mandatory, the Subinvestigator must promptly inform the Investigator. If the Investigator cannot be contacted, the Site Coordinator or Subinvestigator must take over the responsibility of the Investigator. The Investigator must report adverse events according to the following procedures.

Attention should be paid not to include the patient's name and medical record number when sent.

Serious adverse events that occur after the initiation of protocol treatment (after the date of registration if death) by the date of final follow-up are subjects of Expedited Reporting.

1) "Disease or the like" that may lead to death or death specified in 10.1 1) and 2).

Primary reporting:

The Subinvestigator who is aware of the occurrence of adverse events will promptly notify the Investigator. The Investigator who receives the notice should fill out JCOG Adverse Event Report Form(for institution) and the "Disease or the like" Report Form to Certified Review Board specified in Clinical Trials Act Enforcement Regulations as far as possible and contact Principal Investigator/Study Coordinator via e-mail within 72 hours of knowledge of the occurrence of the adverse event.

Secondary reporting:

The Investigator should add detailed information on adverse events to JCOG Adverse Event Report Form(for

institution) and the "Disease or the like" Report Form to Certified Review Board specified in Clinical Trials Act Enforcement Regulations within 7 days of knowledge of the occurrence of adverse events and send them to the Principal Investigator/Study Coordinator via e-mail. If necessary, attach copies of laboratory data, images, autopsy report, etc.

2) 10.1. 3)"Disease or the like" or other medically important condition requiring hospitalization or prolongation of hospital stay for treatment. Adverse events are judged to be either of 10.1. 4)-7)

The Subinvestigator who is aware of the occurrence of adverse events will promptly notify the Investigator. The Investigator who receives notice must fill out JCOG Adverse Event Report Form (for institution) and the "Disease or the like" Report Form to Certified Review Board specified in Clinical Trials Act Enforcement Regulations within 10 days of knowledge of the occurrence of an adverse event and send them to the Principal Investigator/Study Coordinator via e-mail. If necessary, attach copies of laboratory data, images, autopsy report, etc.

3) Additional reporting

If new information is obtained after conducting the above reporting, the Investigator must add information to JCOG Adverse Event Report Form (for institution) and the "Disease or the like" Report Form to Certified Review Board specified in Clinical Trials Act Enforcement Regulations and report it as needed.

Table 10.2.1. Summary of Adverse Events which are subjects for Expedited Reporting and the deadline of reporting to Principal Investigator/Study Coordinator

Causal relationship	The patient was hospitalized due to Grade 1-3 AE. Other medically important conditions		Grade 4		Death	
	Expected	Not expected	Expected	Not expected	Expected	Not expected
Present	No need of reporting	Primary reporting: within 10 days Additional reporting: as needed	Primary reporting: within 72 hours Secondary reporting: within 7 days Additional reporting: as needed			
None	<Only on-treatment or within 30 days of last protocol treatment day>					
	No need of reporting	Primary reporting: within 10 days Additional reporting: as needed	Primary reporting: within 72 hours Secondary reporting: within 7 days Additional reporting: as needed			

* 4) Disability, 5) "Disease or the like" that may lead to disability, and 6) "Disease or the like" that are serious similar to 1) to 5) in 10.1., 7) Congenital disorders or abnormalities in later generations

※ "Unexpected" refers to those not listed in "7. Expected Adverse Events"

Table 10.2.1. Summary of Adverse Events which are subjects for Expedited Reporting and the deadline of reporting to Principal Investigator/Study Coordinator

Causal relationship	The patient was hospitalized due to Grade 1-3 AE. *Other medically important conditions		Grade 4		Death	
	Expected	Not expected	Expected	Not expected	Expected	Not expected
Present	Primary reporting: within 10 days Additional reporting: as needed	Primary reporting: within 10 days Additional reporting: as needed	Primary reporting: within 72 hours Secondary reporting: within 7 days Additional reporting: as needed			
None	<Only on-treatment or within 30 days of last protocol treatment day>					
	Primary reporting: within 10 days Additional reporting: as needed	Primary reporting: within 10 days Additional reporting: as needed	Primary reporting: within 72 hours Secondary reporting: within 7 days Additional reporting: as needed			

* 4) Disability, 5) "Disease or the like" that may lead to disability, and 6) "Disease or the like" that are serious similar to 1) to 5) in 10.1., 7) Congenital disorders or abnormalities in later generations

※ "Unexpected" refers to those not listed in "7. Expected Adverse Events"

10.2.2. Reporting to the Administrator of participating medical organizations

If an Adverse Event which is subjects for Expedited Reporting occurs and is assessed as causal after reporting to Principal Investigator and reported to Certified Review Board, the Investigator must report it to the Administrator of the relevant medical institution in accordance with the requirements of the medical institution.

10.3. Responsibilities of Principal Investigator/Study Coordinator

10.3.1. Determination of necessity of suspension of registration and emergency notification to institutions

Principal Investigator/Study Coordinator who received the report from the Site Investigator should report to Group Chair and determine the urgency, significance, and impact of the report. If needed, take measures such as suspending registration (contacting JCOG Data Center and all participating institutions) and urgently communicating information to participating institutions. Telephone calls can be made to Data Center and institutions as urgent, but they should also be promptly contacted by document (e-mail).

10.3.2. Reporting to JCOG Operations Office and Certified Review Board and MHLW

1) Reporting from the Principal Investigator/Study Coordinator to JCOG Operations Office

Principal Investigator/Study Coordinator should consult with Group Chair and report to JCOG Operations Office (Safety Contact) by e-mail within 72 hours of knowledge of the occurrence of the adverse event, if reported AE is considered to meet the adverse events specified in 10.2.1. 1) and 2) In doing so, to the extent feasible, Principal Investigator/Study Coordinator should send "JCOG Adverse Event Report Form (for institution)" sent from the institution, "Disease or the like Report Form" addressed to Certified Review Board as stipulated in Clinical Trials Act Enforcement Regulations, and attach "JCOG Adverse Event Report (for Study Coordinator)" with Study Coordinator/Principal Investigator's view (including judgments of causality and expectation, and judgments of continuation/discontinuation of the study)". For the expected adverse events of 1) or 2) in 10.2.1., include a discussion not only of the individual patient's course but also of whether the frequency of appearance is within the expected range.

2) Reporting to Certified Review Board

JCOG Operations Office (Safety Contact) reviews the appropriateness of the judgement of causality and expectation of adverse events reported in the above procedures and can ask Principal Investigator/Study Coordinator to reconsider them if there is any doubt. Adverse events considered by Principal Investigator/Study Coordinator and JCOG Operations Office to be related to the protocol treatment and to be the subject of reporting in the following tables should reported to Certified Review Board through JCOG Operations Office.

If there is a disagreement between Principal Investigator/Study Coordinator and JCOG Operations Office, report it to Data and Safety Monitoring Committee and seek final judgment from the Chair of Data and Safety Monitoring Committee. However, if the reporting may exceed deadlines of reporting, the report can be tentatively reported as "causal" to Certified Review Board.

Reporting subjects and reporting deadline

Principal Investigator/Study Coordinator must report to Certified Review Board through JCOG Operations Office within the following time periods after knowledge of the occurrence of adverse events.

Causal relation ship	The patient was hospitalized due to Grade 1-3 AE. Other medically important condition		Grade 4		Death	
	Expected	Not expected	Expected	Not expected	Expected	Not expected
Present	No need of reporting	Within 15 days	Within 15 days	Within 7 days	Within 15 days	Within 7 days
None	No need of	No need of	No need of	No need of	No need of	No need of

	reporting	reporting	reporting	reporting	reporting	reporting

※ "Unexpected" refers to those not listed in "7. Expected Adverse Events"

Principal Investigator/Study Coordinator must report adverse events to Certified Review Board through JCOG Operations Office within the following deadlines after knowledge of the occurrence of adverse events.

Causal relation ship	The patient was hospitalized due to Grade 1-3 AE. Other medically important condition		Grade 4		Death	
	Expected	Not expected	Expected	Not expected	Expected	Not expected
Present	Within 30 days	Within 15 days	Within 30 days	Within 15 days	Within 15 days	Within 15 days
None	No need of reporting	No need of reporting	No need of reporting	No need of reporting	No need of reporting	No need of reporting

※ "Unexpected" refers to those not listed in "7. Expected Adverse Events"

3) Reporting to MHLW

Principal Investigator/Study Coordinator of the study with unapproved or off-label health care should report "Disease or the like" Report Form specified in Clinical Trials Act Enforcement Regulations to MHLW through JCOG Operations Office, if an adverse event is considered to be unexpected and to have a causal relationship to the protocol treatment (See 10.5. Responsibilities of the Data and Safety Monitoring Committee).

Reporting subjects and reporting deadlines

Principal Investigator/Study Coordinator should report to the MHLW through JCOG Operations Office within the following deadlines after knowledge of the occurrence of adverse events.

※ Pharmaceuticals and Medical Devices Agency Safety Division I (trk-shippeitouhokoku@pmda.go.jp)

Causal relation ship	The patient was hospitalized due to Grade 1-3 AE. Other medically important condition		Grade 4		Death	
	Expected	Not expected	Expected	Not expected	Expected	Not expected
Present	No need of reporting	Within 15 days	No need of reporting	Within 7 days	No need of reporting	Within 7 days
None	No need of reporting	No need of reporting	No need of reporting	No need of reporting	No need of reporting	No need of reporting

※ "Unexpected" refers to those not listed in "7. Expected Adverse Events"

4) Additional reporting

Following receipt of secondary or additional reports from the Investigator, Principal Investigator/Study Coordinator must add additional information from the primary report and their views to JCOG Adverse Event Report Form (for the institution) and the "Disease or the like" Report Form to Certified Review Board set out in Clinical Trials Act Enforcement Regulations, and promptly contact JCOG Operations Office (Safety Contact) by e-mail. If the report was sent to Certified Review Board and the MHLW in the primary reporting,, the secondary reporting and additional reporting must be made in the same manner.

10.3.3. Notification to the Site Investigators

When reported to Certified Review Board, Principal Investigator/Study Coordinator should inform the Investigators of all participating institutions of the review results and recommendations by documents (e-mail is allowed). Principal Investigator/Study Coordinator must inform the Investigators without waiting for Certified Review Board review if there is any urgent information to be disseminated. In the event that reported to the MHLW, Principal Investigator/Study Coordinator should notify the Investigators of all participating institutions.

In addition, even if no reporting is made to Certified Review Board, Principal Investigator/Study Coordinator must inform the Investigator of the reporting institution of the decision of Principal Investigator/Study Coordinator by documents (e-mail is allowed).

10.3.4. Assessment of Adverse Events in Periodic Monitoring

During Periodic Monitoring, Principal investigator/Study Coordinator should carefully review the adverse events in the Monitoring Reports issued by the Data Center and ensure that there are no missed reporting from the participating institutions. It should also be confirmed that all reported adverse events are listed in the Monitoring Reports. The presence or absence of a missed reporting should be indicated in the column of the results of Group review on the Periodic Monitoring Report.

10.4. Responsibilities of the Site Investigators at the participating institutions (including the relevant institution)

In accordance with the instructions of Principal Investigator/Study Coordinator, the Site Investigator at the participating institution should report to the administrator of the relevant institution if the adverse event is subjects of reporting of "disease or the like" to Certified Review Board in accordance with the regulations of the relevant institution.

10.5. Responsibilities of the Data and Safety Monitoring Committee

JCOG Operations Office (Safety Contact) should check the details of the adverse event reports received from the Principal Investigator/Study Coordinator according to the procedures described in 10.3.2. and should report them to Certified Review Board and the MHLW according to the procedures described below, with the presence or absence of causality or expectation.

In addition, Principal Investigator and JCOG Operations Office can hear the opinions of JCOG Data and Safety Monitoring Committee according to the reported adverse events. If a review request is issued, Data and Safety Monitoring Committee can review the appropriateness of the institutional response to adverse events and the propriety of continuation of the study in a consensus or written form.

In addition, the submitted information (JCOG Adverse Event Report (for institution), JCOG Adverse Event Report (for Study Coordinator), "Disease or the like" Report, etc.) will be stored semi-permanently in JCOG Operations Office.

Subjects, destination and deadlines for reporting after the knowledge by Investigator/Study Coordinator are as described in 10.3.2.

11. Response Evaluation and Endpoint Definition

11.1. Response assessment (only for patients with measurable disease)

Tumour response assessment will be performed according to the following steps according to the [49] of version 1. 1-Japanese translational JCOG version-Revised RECIST guideline (version 1. 1) Revised new guidelines for the assessment of treatment response in solid tumours (RECIST guidelines). RECISTv1. The 0 original article stipulates that "the use of this guideline for the purpose of determining continuation of treatment is not the subject of this guideline." Similar statements continue to be included in RECISTv1 1 as follows.

> "Many oncologists make decisions about whether to continue treatment based on both objective imaging criteria and symptom-based criteria for follow-up of patients with malignancies in their daily clinical practice, but these revised guidelines are not intended to be used to make decisions about whether or not to continue treatment in these individual patients, unless the treating oncologist determines that it is appropriate."

Therefore, the "overall effect" as determined by RECIST Guideline-based response assessment should be used to determine whether a drug or regimen shows encouraging results that merit continued developmental studies. In other words, judgment of whether or not to continue treatment in individual patients should not be based on CR/PR/SD/PD of overall efficacy, but rather on "clinical judgment" based on comprehensive consideration of symptoms, physical findings, and various laboratory data, in addition to imaging findings.

Therefore, it may be clinically appropriate to continue protocolized treatment, even when PD (Progressive Disease: progression) is judged as an overall response based on the assessment of response based on imaging. In this case, the pros and cons of continuing protocol treatment should be determined based on clinical judgment, regardless of response assessment, but the date of the event for progression-free survival, which is judged to be an overall effect of PD, should be used. This is due to three reasons: (i) it may be possible to decide whether protocol treatment should be continued for each group; (ii) RECIST is a criterion intended to standardize not only response rates but also progression-free survival; and (iii) the standard definition of US Cooperative Group is that PD is the event of progression-free survival for any reason if the overall response is PD.

On the other hand, if a physician judges "clinical progression" based on clinical and comprehensive judgment not based on diagnostic imaging, even if PD is not met by the response criteria based on diagnostic imaging, protocol treatment should be discontinued in accordance with "6.2.2. Criteria for discontinuation of protocol treatment". If "clinical exacerbation" is judged, even if "PD" is not judged by the response evaluation, the day of "clinical exacerbation" is considered as an event of progression-free survival. This is because imaging is often not performed as planned after a patient is judged to have a "clinical progression" and therefore the risk of overestimating progression-free survival is greater if "clinical progression" is not an event for progression-free survival. It is also statistically incorrect (informative censoring) to treat "clinical progression" as "censoring" progression-free survival, as it would censoring patients at increased risk of progression or death.

In RECISTv1 1, the original article described "definite progression (unequivocal progression") in the PD criteria for non-target lesions as "marked progression of non-target lesions that deserves discontinuation of treatment as an increase in total tumor burden" and therefore described "marked progression of non-target lesions" as "judgement of whether or not to continue treatment in individual patients" in some of the PD criteria for non-target lesions, which is confusing. It should be noted that this "unequivocal progression" is a criterion of judgment restricted to "PD of non-target lesions".

The relation between the events of 'PD', 'clinical progression', 'progression', and progression-free survival in JCOG is as in the lower panel.

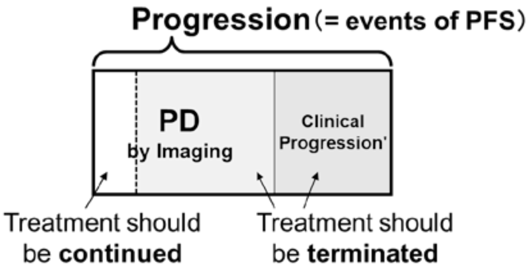

Figure 11.1. Relationship Between Exacerbations, PD on Imaging, and Clinical Exacerbations.

11.1.1. Baseline Evaluation

「8.1. According to "Pre-registration Evaluation Items" contrast enhanced-enhanced computed tomography (CT) with a range of indispensable for the primary organ is used to identify neoplastic lesions before enrollment, and each lesion is classified into "measurable lesions" and "unmeasurable lesions". If CT is allergic to contrast medium, both contrast-enhanced MRI and plain CT should be performed.

Tumor diameters are measured by CT or MRI in cross-sectional views, and sagittal and coronal measurements by three-dimensional reconstructed images are not used. Baseline assessment will be done using the latest imaging studies within 28 days prior to enrollment. If the imaging test is re-examined after enrollment and before the start of treatment, the latest imaging test with re-examination should be used.

In this study, the tumour markers NSEs and ProGRP are used to evaluate the response of non-target lesions.

11.1.2. Definition of measurable lesions

Lesions that fall under any of the following conditions are considered measurable lesions (measurable lesion):

1) Non-nodal disease (non-nodal disease) of 10 mm or greater in greatest dimension on CT or MRI with a slice thickness of 5 mm or less
2) CT or MRI of 5 mm or less slice thickness showing lymph node lesion of 15 mm or more in short diameter (Nodal lesions with short diameters between 10 mm and less than 15 mm are defined as non-target lesions, and those with short diameters less than 10 mm are not)

All other lesions will be non-measurable (non-measurable lesion).

Caution should be exercised because the following lesions are not measurable regardless of the examination method or the size of the lesion.

- Bone lesions (excluding osteolytic lesions with measurable soft tissue components)
- Cystic lesion
- Leptomeningeal lesions
- Ascites, pleural effusion, and pericardial effusion
- Lymphangiosis of the skin and lungs
- Palpable but not measurable abdominal mass or enlargement of abdominal organs
- Superficial skin lesions

11.1.3. Target Lesion Selection and Baseline Recording

Up to five measurable lesions, in descending order of diameter (non-lymph node lesions are long diameters and nodal lesions are short diameters), up to two lesions per organ are selected to be target lesions (target lesion) among measurable lesions at enrollment. Selection should take into account the universal inclusion of as many organs with measurable disease as possible and the reproducibility or ease of measurement (reproducible repeated measurement) of repeated measurements (avoiding lesions that are not measurable even if they are large in diameter).

For selected target lesions, in order from cranial to caudal, site (code), test method, test date, long diameter of non-lymph node target lesion, short diameter of nodal target lesion, and sum of all target lesion diameters (hereafter, sum of diameters) will be recorded in Pretreatment Report 3.

11.1.4. Baseline recording of non-target lesions

For lesions not selected as target lesions, all measurable or non-target lesions (non-target lesion) should be recorded as site of lesion (code), method of examination, and date of examination in Pretreatment Report 3. Multiple non-target lesions within the same organ may be recorded as a single lesion (e.g., multiple enlarged pelvic lymph nodes, multiple liver metastases).

11.1.5. Determining tumor response

Evaluation of target and non-target lesions will be performed every 6 weeks according to "8.2 Testing and Evaluation during Treatment" in the same manner as at enrollment. Target lesion diameter, non-target lesion disappearance or progression will be recorded in the "Tumor Reduction Effect Report".

11.1.6. Response Evaluation Criteria for Target Lesions

・CR(Complete Response): Complete response

When all non-lymph node target lesions disappear and all nodal target lesions have a short diameter of

less than 10 mm. If a nodal target lesion is selected at baseline, the effect of the target lesion may be CR even if the sum of diameters is not 0 mm.

・PR(Partial Response): Partial response

30% or more reduction in target diameter sum compared to baseline diameter sum

・PD(Progressive Disease): Progress

Compared to the minimum diameters (when the baseline is the minimum value during the passage, this is the minimum sum of diameters), the sum of the target disease increases by more than 20%, and the sum of diameters increases by more than 5 mm even in absolute value.

・SD(Stable Disease): Stability

There is no reduction corresponding to PR and no increase corresponding to PD compared to the smallest sum of diameters during the course

・Lack of study; Not all Evaluated

If the test cannot be performed for any reason or if neither CR, PR, PD, or SD can be determined

Pre-treatment sum of diameters-sum of diameters at study
Percentage of reduction of the diameter sum =---------------× x 100%
Pre-treatment diameter sum

Sum of diameters at study-minimum sum of diameters
Increasing Percentage of Diagram =---------------× x 100%
Minimum sum of diameters

※ Measured target lesion diameters are recorded whenever measurable (e.g., <5 mm). If the target lesion diameter is judged to be too small to be measured (too small to measure), the diameter should be 0 mm if the tumor lesion is judged not to be retained, and 5 mm if the tumor lesion is judged to be retained, regardless of the CT slice thickness.

※ PD is defined when the reduction ratio meets the condition of PR and the concomitant increase ratio meets the condition of PD.

※ When one lesion is separated during treatment, each diameter is added to the sum of diameters.

※ If more than one lesion fuses and the boundary cannot be distinguished during treatment, the diameter of the fused lesion is added to the sum of the diameters. The diameter of each lesion is added to the sum of diameters when the boundary of the lesion is identifiable, even if the lesion is in contact with each other.

11.1.7. Response Evaluation Criteria for Non-Target Lesions

・CR(Complete Response): Complete response

When all non-lymph node non-target lesions disappear, the short diameter of all nodal non-target lesions becomes less than 10 mm, and all tumour markers (NSEs, ProGRP※) are below the upper shared reference limits. When there are no (on the image) non-target lesions at baseline assessment and all tumor markers are below the shared reference range upper limit.

Because ※ProGRP is not included in the shared baseline range, the baseline range is 6.5-46.0 pg/mL.

・Non-CR/non-PD: non-CR/non-PD

Residual one or more non-target lesions (including residual nodal non-target lesions ≧10 mm in short diameter) and/or tumour markers (NSEs, ProGRP※) exceeding the shared upper reference limits. No non-target lesions (on images) at baseline assessment and any of the tumor markers exceeding the shared reference range upper limit.

・PD(Progressive Disease): Progress

'Apparent exacerbation' (including relapse) of pre-existing non-target lesions.

For measurable disease: A marked progression of a non-target lesion that deserves discontinuation of treatment as an increase in overall tumor burden must be observed if the effect of the target lesion is SD or PR but is judged to be "clear progression" based on the change in the non-target lesion. If the effect of the target lesion is SD or PR, then an increase in the tumor burden of the non-target lesion to a degree

that far exceeds the decrease in tumor burden is considered "obvious progression" and otherwise Non-CR/non-PD.

If only unmeasurable disease is present, the increase in non-target disease, as judged to clearly exceed the tumor burden corresponding to a 20% increase in diameter and a 73% increase in tumor volume, is considered "definite progression". Elevated tumor markers alone are not considered PD.

・NE(Not all Evaluated): Lack of study

If the test could not be done for any reason or if neither CR, Non-CR/non-PD nor PD could be determined.

11.1.8. Presence or absence of new lesions

If a lesion that was not present at baseline was observed after the start of treatment, it is considered "new lesion" to be present.

However, a "new lesion" requires that it is not an imaging change due to a difference in the imaging method from the baseline assessment or a change in the imaging modality, nor is it an imaging change due to a condition other than the tumor. For example, a cystic lesion arising within a lesion due to necrosis of a liver metastatic lesion is not a new lesion. New lesions will be defined as new lesions by examination of sites that were not mandatory at baseline (pre-enrollment study).

If a lesion disappears and later reappears, measurement is continued. However, the effect at the time the lesion reappears depends on the status of the other lesion. When the overall effect reappears after CR, the lesion is judged as PD at the time of reappearance. When the overall effect is PR or SD, on the other hand, once the disappeared lesion reappears, the diameter of the lesion will be added to the sum of the diameters of the remaining lesions to calculate the effect. That is, in the presence of many residual lesions, even if one lesion reappears after an apparent disappearance, it is not judged as PD by itself, and it is judged as PD when the sum of the diameters of all lesions meets the criteria for PD. This is because of the perception that the majority of lesions do not truly 'disappear' and are not only depicted by the limits of resolution of the imaging modalities used.

If there is a possibility of a new lesion but it cannot be determined, it should not be a new lesion, and imaging should be reexamined at a clinically relevant time. If a new lesion is confirmed by repeat imaging, the new lesion will appear based on the date of imaging at which the new lesion is confirmed.

11.1.9. Overall efficacy (Overall Response)

The overall response (Overall response) will be determined by combining the effects of target lesions, non-target lesions, and the presence or absence of new lesions every 6 weeks according to Table 11.1.9.a below. The overall effect in the absence of a non-target lesion at baseline will be determined by the effect of the target lesion and the presence or absence of a new lesion, and the overall effect in the absence of a target lesion at baseline will be determined according to the effect of a non-target lesion and the presence or absence of a new lesion according to Table 11.1.9.b.

Table 11.1.9.a. Overall efficacy at each time point for target lesions (with or without non-target lesions)

Target lesion	Nontarget lesions	New lesions	Overall effect
CR	CR	None	CR
CR	Non-CR/non-PD	None	PR
CR	Lack of study	None	PR
PR	Lack of Non-PD or study	None	PR
SD	Lack of Non-PD or study	None	SD
Lack of study	Non-PD	None	NE
PD (obvious progression)	Irrespective of	With or without	PD
Irrespective of	PD	With or without	PD
Irrespective of	Irrespective of	Present	PD

Table 11.1.9.b. Overall efficacy at each time point for patients with non-target lesions only

Nontarget lesions	New lesions	Overall effect
CR	None	CR

Non-CR/non-PD	None	Non-CR/non-PD
Lack of study	None	NE
PD (obvious progression)	With or without	PD
Irrespective of	Present	PD

11.1.10. Best overall effectiveness (Best Overall Response)

CR > PR > SD > PD > NE is considered good, and the best overall effect is the best overall effect throughout the entire course.

PD is defined when imaging cannot be determined due to exacerbation of obvious disease or death before the first response assessment. In addition, NE is defined if it cannot be determined by imaging due to discontinuation of toxicity before the first response assessment or patient refusal.

11.2. Definitions of analyses set

The analysis sets used in periodic central monitoring, interim analysis, and final analysis are defined as follows: The flow diagram below shows the analysis sets.

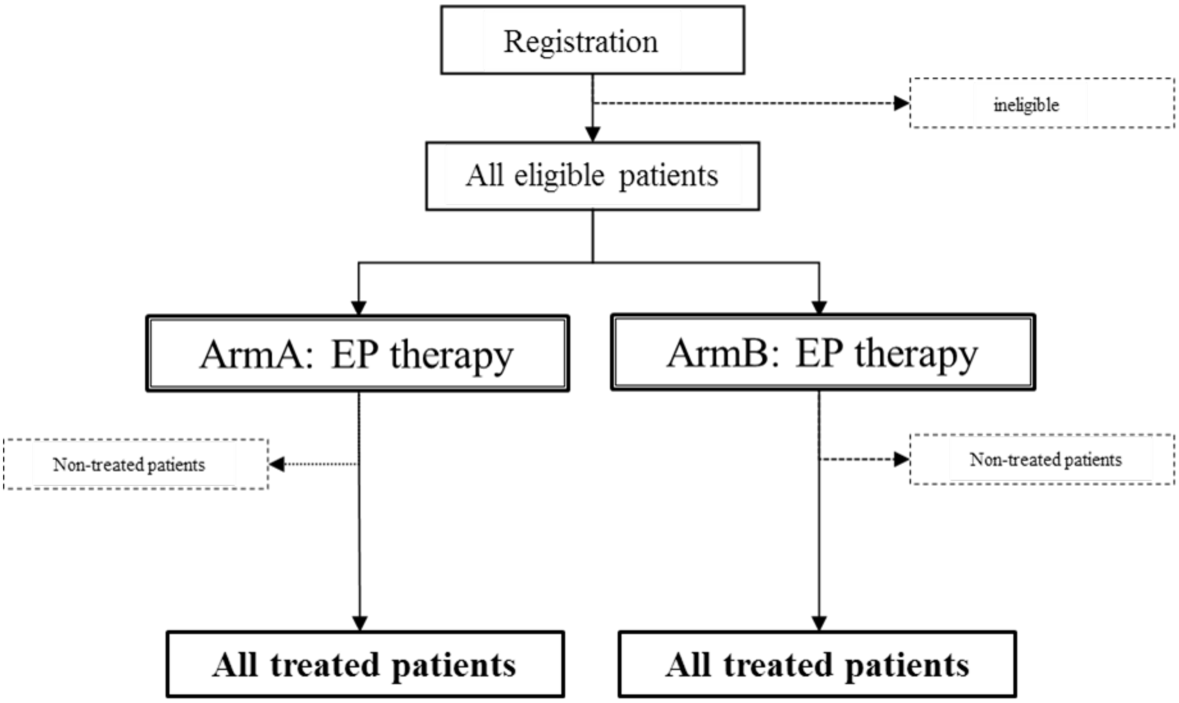

11.2.1. All registered patients

「5.1. Among the patients enrolled according to the Procedures for Enrollment, the population excluding duplicate or mis-enrollment is considered as "all enrolled cases".

11.2.2. All eligible patients

The group excluding "ineligible cases (post hoc ineligibles, de facto ineligibles, violation of registration)" determined by group review from all registered patients is regarded as all eligible patients. Ineligible cases as judged by the investigator or sub-investigator alone are included in all eligible patients. Only those judged not eligible by the central pathological diagnosis are ineligible and not included in all eligible patients.

11.2.3. All treated patients

Of all enrolled patients, all patients for whom part or all of the protocol treatment was performed will be defined as all treated patients.

The decision to treat "non-treated patients" for whom no protocol treatment has been given and whether it is excluded from the safety analysis can be determined by the data center with the consent of the Research Office. Ineligible patients will be excluded from all treated patients. However, if there are circumstances in which ineligible patients are included in the analysis, the nature of the ineligibility will be examined and determined by the Research Secretariat in consultation with JCOG Data Centre.

11.2.4. Patients eligible for central pathology diagnosis

Of all eligible patients, the population excluding ineligible patients with a central pathological diagnosis will be considered as eligible patients with a central pathological diagnosis.

11.3. Definition of endpoints

Endpoint	Events (whichever is earlier)		Censoring date
Overall survival time<br>Overall survival(OS)	All deaths	-	Date of final survival confirmation
Progression-free survival time<br>Progression-free survival(PFS)	All deaths	Progression/r ecurrence	Final date of clinically confirmed freedom from exacerbation

11.3.1. Overall survival

The duration from the date of registration to the date of death from any cause.

- Survivors are censored at the date of final survival confirmation (survival confirmation by telephone contact is also permitted, but the fact that survival confirmation was performed should be recorded in the medical record).
- Patients lost to follow-up are censored at the last date of survival confirmation before lost to follow-up.

11.3.2. Progression-free survival (PFS: Progression-free survival).

The duration from the date of registration to the date of the judgement of exacerbation or death from any cause, whichever comes first.

- The exacerbation (progression) includes both imaging-based PD (progression) and exacerbation of pathogenic disease (clinical exacerbation) that cannot be confirmed by imaging studies in Section 11.1.9. Global Effectiveness. If an exacerbation is judged based on diagnostic imaging, the exacerbation date is the test date on which the imaging was performed, and in the case of clinical exacerbation, the date of clinical judgment is the exacerbation date. PD may occur in accordance with the Response Evaluation Criteria, even if the tumor diameter is very small, but the disease is clinically judged to be "not obviously aggravated" in accordance with the Response Evaluation Criteria Criteria (in this case, clinical judgment should be prioritized for continued treatment with the protocol). In addition, even if PD is not observed according to the response evaluation criteria, the clinical judgment is prioritized for progression if it is judged to be clinically obvious exacerbation.
- In survivors who are not judged to have progression, clinically confirmed progression is censored (date of final progression-free survival confirmation) (confirmation of progression-free by imaging or specimen examination is not mandatory and clinical progression-free by outpatient examination etc. is not permitted. Telephone contact alone is not permitted. If information on progression or progression-free is obtained at a medical institution or referral center, receive and retain a medical information form describing the rationale for diagnosis. In this case, telephone contact alone is not permitted).
- Events and censoring are treated similarly, if chemotherapy is terminated for reasons such as toxicity or patient refusal, and if other therapies are added as post-study treatment. i.e., it is not censored at the time of treatment termination or at the date of initiation of post-study treatment.
- When the diagnosis of exacerbation is based on imaging, the relapse is not regarded as an event at the test date of imaging with "suspicious diagnosis of relapse", but regarded as an event at the later test date of imaging with "definite diagnosis of relapse"" If the event is judged to be clinically aggravated based on diagnostic imaging, the event is considered to be the day of the progression.
- If the definitive diagnosis of relapse or new lesion is based on biopsy pathology, the date of clinical diagnosis is defined as the date of clinical diagnosis when the diagnosis of recurrence or new lesion is made clinically, and the date of biopsy is defined as the date of event when the diagnosis of recurrence is made based on biopsy pathology diagnosis without clinical diagnosis of recurrence.
- The incidence of a second cancer (metachronous double cancer) is not censored or event, and progression-free survival is defined until other events are observed.

11.3.3. Response rate (response rate) Response proportion (Response rate)

Among all eligible patients with measurable disease, the response rate is defined as the proportion of patients with "11.1.10. Best Global Effect" that is either CR or PR.

11.3.4. Incidence of adverse events (adverse reactions)

Using all treated patients as the denominator, the frequency of the worst Grade during the entire course by CTCAE v4 0 Japanese translation JCOG version for each of the following adverse events (toxicities) will be determined by group:

- Laboratory tests: hemoglobin decreased, white blood cell decreased, neutrophil count decreased, platelet count decreased,

  Increased blood bilirubin, aspartate aminotransferase increased (AST or GOT),
  Alanine aminotransferase increased (ALT or GPT), creatinine increased,
  Hypernatremia, hyponatremia, hyperkalemia, hypokalemia, hypercalcemia, hypocalcemia,

hypermagnesemia, hypomagnesemia

- General disorders and administration site conditions: fever, fatigue
- Ear and labyrinth disorders: Tinnitus, hearing impairment
- Skin and subcutaneous tissue disorders: alopecia
- Gastrointestinal disorders: constipation, diarrhea, nausea, vomiting, oral mucositis
- Metabolism and nutrition disorders: anorexia, dehydration
- Nervous system disorders: dysgeusia, peripheral sensory neuropathy, peripheral motor neuropathy
- Musculoskeletal and connective tissue disorders: myalgia, arthralgia
- Infections and parasites: bronchial infection, pulmonary infection, upper respiratory tract infection, catheter-related infection, biliary tract infection,

  Gallbladder infection; Mediastinal infection; Pleural infection; Urinary tract infection
- Blood and lymphatic system disorders: Febrile Neutropenia
- Musculoskeletal and connective tissue disorders: arthralgia, Vascular disorders: phlebitis
- Respiratory, thoracic and mediastinal disorders: dyspnea, hypoxia, pneumonitis

In the other adverse event (toxicitiy) than the above, the proportion of occurrence are not calculated unless a large number of specific adverse events are observed, since only Grade 3 or more non-hematological toxicity ※ is reported in the Treatment Form.

※※ Non-hematological toxicity refers to adverse events other than those listed below in CTCAE v4.0-JCJCOG.
Anemia, decreased bone marrow cells, decreased lymphocyte count, decreased neutrophil count, decreased white blood cell count, and decreased platelet count.
CD4 lymphocytopenia

11.3.5. Dose intensity of cisplatin

Dose intensity of cisplatin per patient (DOOC.) will be calculated for all treated patients to assess treatment compliance with cisplatin. The summary statistics (minimum, 25% point, median, 75% point, maximum, mean, and standard deviation) will be calculated for each group.

- ・Active dose D.I. (mg/m2/week) = total drug dosebody surface areatreatment duration (weeks)
- Body surface area: BSA is calculated by height at enrollment and body weight calculated by Data Center.
- ・ Treatment period (weeks)

  Arm A = (start date of last course-start date of 1st course + 21)/7

  Arm B = (start date of last course-start date of 1st course + 28)/ 7

11.3.6. Incidence of serious adverse events (adverse reactions)

<u>1) Grade 4 non-hematologic toxicities, early deaths, and treatment</u>

Using all treatments as denominators, the percentage of patients with one or more Grade 4 non-hematologic toxic ※ that are considered to be related (either to definite, probable, possible) to the protocol treatment among the adverse events listed in the free text of the CRFs in addition to the stereotyped items in Section 11.3.4 is used as the numerator.

※※ Non-hematological toxicity refers to adverse events other than those listed below in CTCAE v4.0-JCJCOG.
Anemia, decreased bone marrow cells, decreased lymphocyte count, decreased neutrophil count, decreased white blood cell count, and decreased platelet count.
CD4 lymphocytopenia

<u>2) Early mortality rate</u>

Proportion of all deaths during the protocol treatment or within 30 days from the last protocol treatment day among all treated patients. Causes of death irrespective of causality with protocol treatment. However, if premature death occurs in patients excluded from all treated patients, the details are provided separately.

<u>3) Proportion of treatment-related death (TRD incidence)</u>

Proportion of all deaths judged as causally related (either definite, probable, possible) to the protocol treatment among all treated patients However, if TRD occurs in patients who have been removed from all treated patients, the details are presented separately.

12. Statistical consideration

Methods for statistical analysis are as follows: In addition, the details required for conducting specific analyses are specified in the statistical analysis plan prepared separately prior to the analysis, and in documents that clarify the endpoint definition, etc. If substantial changes occur in statistical analyses as described below, follow the policy set out in "13.6. Protocol Changes." Facilities are contacted by "14.1. Periodic Monitoring" for missing or abnormal results, and data collection or exclusion is determined by review by the Research Secretariat based on the results of inquiries to the institution in accordance with the policies stipulated in "14.1.2. eligible (eligibility/ineligibility)" and "14.1.3. Protocol Deviations/Violations". 「11. Handling of missing values and abnormal data that cannot be addressed by the definition and analysis method for each endpoint, as specified in "Definition of Response Evaluation and Endpoints" and "12. Statistical Items" below, is specified in the above statistical analysis plan.

12.1. Principal Analysis and Decision Criteria

The primary analysis of this study will be the final analysis.

The purpose analysis of this trial is to test whether one of the two community standard-of-care arm A (EP-therapy) and B (IP-therapy) outperforms primary endpoint overall survival. The null hypothesis of equal overall survival in the two groups in the main analysis will be tested by stratified log-rank test stratified by non-institutional allocation adjustment factors (primary organ [gastrointestinal vs. hepatobiliary-pancreatic]) in all patient patients. However, if it is assumed that a stratified log-rank test cannot be performed appropriately, such as when the number of subjects and events in each stratum is small, the allocation adjustment factors will be addressed in the analysis plan prepared without information related to the comparison between groups before performing a confirmatory analysis with comparison between groups. Sensitivity analysis will also be performed in all eligible patients and in patients eligible for central pathology diagnosis.

Due to interest in which treatment group is superior, the test will be two-tailed. The study-wise significance level for the study is set at both sides 10%. In the main analysis, two-sided 90% confidence intervals corresponding to a two-sided 10% significance level will be calculated, and in other analyses, two-sided 95% confidence intervals will be calculated for descriptive purposes.

We conclude that EP therapy is a more useful treatment when the two-sided p-value is less than 10% and the survival curve of EP therapy exceeds that of IP therapy. Meanwhile, we conclude that IP therapy is a more useful treatment when the two-sided p-value is less than 10% and the survival curve of IP therapy exceeds that of EP therapy. If the difference was not significant at a two-sided significance level of 10%, one of the two modalities cannot be judged to be useful. In this case, we conclude that both modalities continue to be the standard of care, as there is no evidence to actively recommend either one of them.

However, if there are major differences in the toxicity profiles of EP and IP therapies, contrary to prior assumptions, during the course of the study, and if the toxic therapy is not superior to other therapies, it is judged that it will not be of significance to be used in clinical practice, the following measures should be taken. In other words, the clinical hypothesis will be changed from two-sided to one-sided in an analysis plan with no information on group comparisons before a confirmatory analysis with group comparisons will be conducted, and the superiority of the toxicity treatment over other treatment modalities will be verified at a one-sided significance level of 5% (in this case, the policy of change for the interim analysis will be described in Section 12.3.2).

Estimates such as cumulative survival curves, median survival times, and annual survival rates are performed using Kaplan-Meier method. Brookmeyer and Crowley methods are used to obtain 95% confidence intervals for median survival. Greenwood's formula is used to obtain 95% confidence intervals for annual survival rates. Hazard ratios and their confidence intervals for treatment effects between groups are obtained using stratified Cox proportional hazards models with the same factors as the test of the primary analysis as estimates of treatment effects. Cox regression adjusted by imbalance background factors in addition to adjustment factors will be performed as needed.

The main analysis results will be summarized as the "Main Analysis Report" by the Data Center one year after the completion of enrollment and submitted to the Research Secretariat, Research Representatives, Group Representatives, Group Secretariats, Efficacy and Safety Assessment Committee, and JCOG Representatives.

The principal investigator/study coordinator summarizes the content of the main analysis report, prepares a

"Clinical Study Report" summarizing the conclusions, problems, interpretations and discussion of the results, and future policies of the entire study, and submits it to the Data and Safety Monitoring Committee and JCOG chair with approval from the Group chair and the Head of JCOG Data Center.

Approval of the clinical study report by the Data and Safety Monitoring Committee shall be considered as "end of the study."

12.2. Planned accrual, accrual period, and follow-up periods

「2.4.2. Based on the background presented in Clinical Hypothesis and Rationale for Number of Enrollment, we assume a median survival of 8 and 12 months (HR=0.67) for the inferior and superior treatment groups, respectively, among the two treatment groups. When a superiority trial design is used, 63 patients per group and 126 patients in both groups (114 events required) will be included in the required analysis using Schoenfeld & Richter methodological [50] at 6 years of enrollment, 1 year of follow-up, α = 10% (two-sided), and 70% power. The number of required Inferior treatment (number of required events) when the median survival time in A is different from the assumption as shown in Table 12.2.1 below.

Table 12.2.1. Number of Analyses Required (Number of Events Required)

	Power			
Median Survival (mo)	65%	70%	75%	80%
7.0 vs. 10.5	108(100)	124(114)	142(131)	162(150)
8.0 vs. 12.0	112(100)	126(114)	144(131)	166(150)
9.0 vs. 13.5	114(100)	130(114)	148(131)	170(150)

※※ Number of required events in parentheses

Based on these findings, the following will be established in view of some cases of loss to follow-up.

Planned enrollment: 70 patients in each group and 140 patients in both groups

Enrollment period: 6 years, follow-up period: 1 year after completion of enrollment

Consider redesigning the sample size if the prognosis is obviously better than assumed, or if it reaches 70 people within 2.5 years of enrollment initiation. Clinically meaningful differences will then be reviewed and redesigned in a blinded fashion prior to the conduct of the analysis.

<Additions in ver. 1.1>

The registration pace after the enrollment start was smooth and exceeded the plan, and 70 cases were reached in October, 2016, when 2 years and 2 months passed from the enrollment start. Since it exceeded the standard of 70 patients within 2.5 years from the initiation of enrollment specified above, acceptance was obtained at the meeting of the Hepatobiliary and Pancreatic Group on October 29, 2016, the group meeting of the Esophageal Cancer Group on November 19, 2016, and the group meeting of the Gastric Cancer Group on January 7, 2017 with respect to changing the power to 80% according to the rules at the time of the study plan. Therefore, the planned enrollment number was changed to 170. In addition, the "1-year analysis period" was added to the study period, and the following changes were made.

Planned enrollment: 85 patients in each group and 170 patients in both groups

Enrollment period: 6 years; Follow-up period: 1 year after completion of enrollment; Analysis period: 1 year;

Total study period: 8 years

<Additional information in ver. 1.5.0>

In the monitoring in the latter half of fiscal 2019, it was found that the number of events was less than expected. Follow-up will be extended by 1 year to 2 years after completion of enrollment because the required number of events may not be reached at 1 year after completion of enrollment for which final follow-up is planned. However, if the required number of events has been reached at 1 year after completion of enrollment, the final analysis will be performed at 1 year after completion of enrollment. Conversely, if the required number of events is not reached, follow-up will continue until the required number of events is reached to ensure power, and the final analysis will be performed without waiting for the end of the remaining follow-up period when the required number of events is found to be reached within the follow-up period.

Planned enrollment: 85 patients in each group and 170 patients in both groups

Enrollment period: 6 years; follow-up period: 2 years after completion of enrollment; analysis period: 1 year; total study period: 9 years

12.3. Interim Analysis and Early Termination of the Study

12.3.1. Purpose and Timing of the Interim Analysis

Once interim analyses will be conducted to determine if the primary objective of the study has been achieved during the study period. Interim analyses will be conducted to determine if it is reasonable to continue enrollment during enrollment. If the primary objective of the study is determined to be achieved, the study will be discontinued and the study results will be published promptly at the conference and in the article.

Interim analyses will be conducted using data from the initial periodic monitoring that will be queried after the time enrollment of half of the planned enrollment was obtained. Based on the information in the periodic monitoring report, the group will submit the presence or absence of changes in clinical hypotheses and, if any, details of changes to the Efficacy and Safety Assessment Committee by the time of the interim analysis.

During the first interim, patient accrual is continued in principle. If the progress of the study progressed as planned, the expected number of events at the time of the interim analysis under the assumptions presented in 12.2 is expected to be 44 if the interim analysis is conducted at 3 years after the start of enrollment.

12.3.2. Method of interim analysis

Interim analyses will be conducted by the JCOG Data Center. To keep the study-wise alpha errors at 10%, the multiplicity of the interim and final analyses is adjusted using Lan & DeMets's alpha spending functions, and statistical significance is examined for differences in primary endpoint between arms. As α spending functions, we use O'Brien & Fleming types.[51]

For details of the interim analysis, the statistical staff in charge of the group at the Data Center will prepare the statistical analysis plan by the time of the interim analysis. Actual interim analyses will be performed by statistical staff who are not in charge of the group and an interim analysis report will be prepared.

In the interim analysis, if the survival of one treatment group exceeds that of the period, and the p-value of the stratified log-rank test falls below the level specified by the above method, it is judged statistically significant and the trial is discontinued as a rule. The ineffective discontinuation is not planned at a stage where no statistically significant differences are observed unless prior assumptions regarding toxicity are changed. However, the clinical hypothesis may be changed for the reasons described in 12.1, and the primary analysis may be performed by one-sided rather than two-sided testing, which may result in deviations from prior assumptions. In such cases, the primary hypothesis change should be specified in the analysis plan to be prepared without information on group comparisons before a confirmatory analysis with group comparisons. If the overall survival curve in the highly toxic treatment group is below the other, the consideration of ineffective discontinuation should not be judged by a test and considered comprehensively.

12.3.3. Interim analysis Reporting and review of the results

The results of the interim analysis will be submitted to the Data and Safety Monitoring Committee by the Data Center as an Interim Analysis Report and reviewed for the acceptability of continuation of the study and for publication of the results. The Data and Safety Monitoring Committee considers whether to continue the study at the meeting and recommends whether to continue the study and whether to publish the results to principal physician or group chair based on the results of the review.

Members of the Data and Safety Monitoring Committee of the relevant group are not included in the review. Unless the results of the interim analysis make recommendations for discontinuation of the study from the Efficacy and Safety Assessment Committee, the research representative, research office, participating institution researchers, group representatives, and group secretaries of the study will not be able to know the results of the interim analysis until the final follow-up is completed.

When the Interim Analysis Report has been reviewed by the Data and Safety Monitoring Committee to recommend termination or change of all or part of the study, the principle investigator and group chair review the recommendations and decide whether to discontinue or change some of the study.

If the study is discontinued or part of the study is changed, the principal investigator and group chair shall submit

in written form a request to the Data and Safety Monitoring Committee for permission to discontinue the study or a request to revise the protocol. Following approval by the Data and Safety Monitoring Committee, the principal investigator may discontinue the study or change part of the study.

The Study Chair and Group Chair can disagree with the recommendations of the Data and Safety Monitoring Committee, but if they fail to coordinate their opinions with the Data and Safety Monitoring Committee, they will ultimately follow the instructions of JCOG Chair.

If the study is terminated, the subsequent follow-up period will be 1 years from last registration per study.

If the interim analysis resulted in study termination, the interim analysis will be the primary analysis of the study. The Data Center, in cooperation with the Research Representative Physicians and Research Secretariat, will conduct the analysis required to complement the incomplete data and publish the results, focusing on the results of the interim analysis, and promptly prepare the Major Analysis Report and submit it to the Group and the Efficacy and Safety Assessment Committee.

12.4. Analysis of Secondary endpoints

Secondary endpoints analyses will be conducted to provide a supplementary discussion of the primary analysis results of the study. Because the analysis of secondary endpoint is exploratory, no multiplicity adjustments are made. Comparisons between arms are made where appropriate, note that when the results of the group comparisons are not significant, they do not mean that there is no difference between the two arms.

12.4.1. Analysis of safety secondary endpoints

Among Secondary endpoints, the safety endpoints are the incidence of adverse events and the incidence of serious adverse events, which are in principle the items of periodic monitoring (14.1. Periodic monitoring).

The incidence rate of adverse events will be summarized as well as the incidence rate of Grade3 or higher. For adverse events other than laboratory data, the incidence of Grade 2 or higher is also calculated.. Non-hematological toxicity incidence, early mortality, and treatment-related mortality rates of Grade4, which are serious adverse events, are reported in periodic monitoring reports with registration numbers and details. The rates of non-hematologic toxicity, early mortality, and treatment-related mortality for Grade4 will be calculated at the time of the interim analysis and the main analysis. When interval estimation of proportions is performed, accurate confidence intervals based on binomial distributions are used. Comparisons between arms will be made using Fisher's exact test where appropriate.

12.4.2. Analysis of efficacy secondary endpoints

Among Secondary endpoints, efficacy endpoints are response rate, progression-free survival, which will only be analyzed in the interim and primary analyses.

Secondary endpoints analyses do not adjust for multiplicity.

Progression-free survival will be included in all enrolled patients, but a comparison of all eligible patients, excluding ineligible patients, as determined by group study, will also be performed as a sensitivity analysis.

The response rate will include all eligible patients with measurable disease, but a comparison of all enrolled patients will also be performed as a sensitivity analysis.

Fisher's exact test will be used to compare response rates between groups, and binomial distribution-based exact confidence intervals will be used for interval estimation. Estimates, including progression-free survival curves, median progression-free survival, and time-point progression-free survival, will be performed using Kaplan-Meier method, Brookmeyer and Crowley methods will be used to obtain confidence intervals for median progression-free survival, and Greenwood formulas will be used to obtain confidence intervals for progression-free survival. Log-rank test is used for comparison between arms. Hazard ratios and their 95% confidence intervals for treatment effects between arms will be calculated using Cox's proportional hazards model as an estimate of treatment effect. Cox regression adjusted by imbalance background factors in addition to adjustment factors will be performed as needed.

12.5. Final analysis

The primary analysis will be the final analysis unless this study is withdrawn from the interim analysis.

If the interim analysis is withdrawn from the study, then after the end of the 1-year follow-up period, the final analysis will be performed after the final survey confirms the data and then analyses will be performed for all

endpoints.

Except for the interim analyses and the final analysis, analyses with between-arms comparisons for the primary and secondary endpoints for efficacy are not performed unless approved by the Data and Safety Monitoring Committee.

If the final analysis is performed after the main analysis, the final analysis will be summarized by the Data Center as the "Final Analysis Report" and submitted to the Research Secretariat, Research Representatives, Group Representatives, Group Secretariats, Efficacy and Safety Assessment Committee, and JCOG Representatives.

The Study Representative Physician/Research Secretariat summarizes the content of the final analysis report, prepares the "Clinical Study Report" summarizing the conclusions, issues, interpretations and discussion of the results, future policies, etc. mainly from the clinical point of view (if the "Clinical Study Report" is prepared in the previous analysis report, it will be the "Clinical Study Report (Supplementary Version)) with additional updates), and submits it to the Study Representatives and JCOG Representatives with approval from the Group Representatives and the Head of JCOG Data Center.

Approval of the clinical study report by the Data and Safety Monitoring Committee shall be considered as "end of the study."

<Additional information in ver. 1.5.0>

If the required number of events has been reached at 1 year after completion of enrollment, the final analysis will be performed at 1 year after completion of enrollment. Conversely, if the required number of events is not reached, follow-up will continue until the required number of events is reached to ensure power, and the final analysis will be performed without waiting for the end of the remaining follow-up period when the required number of events is found to be reached within the follow-up period.

12.6. Exploratory analysis

To investigate the interaction between treatment effect and the subpopulation, subgroup analyses will be conducted exploratory with respect to the following factors: Because these analyses are not adequately powered and do not adjust for multiplicity, the results of each subgroup analysis should be interpreted as exploratory.

- PS0/1
- Age 65 years or older/<
- Gender (male/female)
- Primary organ (gastrointestinal tract/hepatobiliary pancreas)
- Organ of origin (esophagus/stomach/small intestine/large intestine/pancreas/biliary tract/liver NEC (liver primary or liver metastasis of unknown primary)
- Organ of origin (pancreas/non-pancreas)
- Extent of extension of the primary lesion (locally advanced/distant metastasis or recurrence)
- Extent of extension of the primary lesion (locally advanced/distant metastasis/recurrence)
- Prior radical resection of the primary lesion (none/present)
- Pathological diagnosis was biopsy/resection specimen
- Grade 3 tumour with morphologically similar features of NETs but Grade 3 proliferative activity/morphologically more atypical (previously classified as poorly differentiated endocrine carcinoma) on histopathology with central pathology
- Histopathological examination by central pathological diagnosis, including Small cell carcinoma/Large cell carcinoma /
- Histopathologically diagnosed by central pathology, Ki67 50% or higher/less than 50%

12.7. Premature withdrawal from the trial

In this study, early termination of the study may occur in the following cases:

1) Early termination due to discontinuation of interim analysis
2) Early termination due to adverse events
3) Early termination due to poor enrollment
4) Early termination due to other reasons

12.7.1. Early termination by interim analysis

In this study, based on the criteria described in 12.3, early termination recommendations may be made at the interim analysis review by the Efficacy and Safety Assessment Committee. If the Data and Safety Monitoring Committee provides recommendations for early termination of the study, the principle investigator and group chair will review the recommendations and decide whether to terminate the study early.

12.7.2. Early termination due to adverse events

In JCOG9511 for small-cell lung cancer, 1/77 (1.3%) treatment-related deaths were reported with EP therapy and 3/77 (3.9%) with IP therapy; in JCOG0509, a successor study for the same subject, 1/142 (0.7%) treatment-related deaths were reported with IP therapy and 2/142 (1.4%) with amrubicin plus cisplatin therapy. Using these as reference, we believe that the treatment-related mortality rate should not exceed 3% in this study. Since it is clear that the final point estimate will be at least 3% at the time of 3 treatment-related deaths in either group, immediate entry should be suspended to consider whether or not to withdraw from the study. At this point, the subsequent treatment of the patient being treated will be reviewed. At the time of 3 or fewer treatment-related deaths in each group, each patient will be reported to the Efficacy and Safety Assessment Committee for adjudication. Enrollment will be continued until the results are obtained in principle.

12.7.3. Early termination due to poor enrollment

If the patient enrollment pace is significantly worse than at the time of planning, early termination of the study may be advised by the Data and Safety Monitoring Committee. If early termination recommendations are issued by the Data and Safety Monitoring Committee due to poor enrollment, the principle investigator and group chair will review the recommendations and decide whether to terminate the study early.

12.7.4. Early termination due to other reasons

12.7.1.～12.7.3. If it is judged difficult to continue the study for other reasons, the research representative physician shall submit a request for early termination of the study to the Efficacy and Safety Assessment Committee. If the Data and Safety Monitoring Committee recommends early termination of the study based on the submitted data, the procedure for early termination of the study will be progressed.

12.8. Procedures after Early termination of the Study

If the Study Chair accepts the recommendations made by the Data and Safety Monitoring Committee based on Section 12.7, he/she will promptly submit a notification to the Data and Safety Monitoring Committee that early termination of the study will be performed.

The Study Chair will submit a termination notification to the Certified Review Board within 10 days of the date they decide to terminate the study early. If the study falls into a specified clinical trials under the Clinical Trials Act, the Study Chair shall submit a termination notification to the Certified Review Board within 10 days of the date on which the study was decided to be prematurely discontinued, as well as submit a specified clinical trials termination notification to the MHLW.

The Study Chair promptly informs the investigator of the decision to terminate the study early in writing, and the investigator who has received a report of early termination of the study will report in writing that the study was prematurely terminated to the institution's manager without delay.

If the study is terminated early, JCOG Data Center will promptly initiate the development of the primary analysis report or final analysis report. The subsequent follow-up period will be 1 year from the final enrollment.

13. Ethical Considerations

13.1. Protection of Human Subjects

All researchers involved in this study will conduct this trial in accordance with the "Helsinki Declaration" [1] and "Clinical Trials Act" (2017 Law No. 16) [2] "Clinical Trials Act Enforcement Regulations" (2018 Ministry of Health, Labour and Welfare Order No. 17) and related notices.

1） http://dl.med.or.jp/dl-med/wma/helsinki2013j.pdf
2） http://www.mhlw.go.jp/stf/seisakunitsuite/bunya/0000163417.html

Prior to commencing this study, the site investigator is required to obtain input from Certified Review Board[※1] regarding the conduct of the study, and to obtain approval from the Administrator of the participating institution, and submit the Implementation Plan [※2] to the Ministry of Health, Labour and Welfare.

※ 1 JCOG study will be submitted to the following Certified Review Board.
National Cancer Center Hospital Certified Review Board (accreditation number CRB3180008)

※ 2 "Implementation Plan" means "Documented plan formatted according to the Form No.1 (Form No.1 of the Ministerial Ordinance) specified in Article 39 of the Clinical Trials Act Enforcement Regulations"

13.2. Informed consent

13.2.1. Explanation to the patient

Prior to patient registration, the investigator or subinvestigator will provide the patient with written informed consent form approved by Certified Review Board and explain the following details verbally.

Descriptions

1） Disease names, Stages, and expected prognosis
2） That this study is a clinical trial and is conducted by JCOG
Name of Certified Review Board and contact information for receiving complaints and inquiries to the committee
3） Design and rationale of the study
4） Protocol treatment content
5） Effects expected by protocol treatment
6） Expected adverse events, complications, and sequelae and how to deal with them
Explanation of the extent and frequency of expected adverse events, including complications, sequelae, and treatment-related deaths, and how to deal with them when they occur. In addition to these explanation, obtain the most recent version of the drug package insert and deliver it to patients (PMDA Prescription Pharmaceutical Information Search http ://www.pmda.go. jp/PmdaSearch/iyakuSearch/)
7） Post-study treatment after end of protocol treatment should also be performed appropriately
8） Cost burden and compensations
Explanation of the cost of treatment, compensation that can be received in the event of a health hazard (equivalent to measures taken in general practice, etc.)
9） Alternative treatment
Explanation of treatments that can be received if not participating in this study
10） Anticipated benefits and possible disadvantages
Explanation of anticipated benefits and possible disadvantages by participating in this study
11） Direct access to the medical records
Explanations on acceptance of site visit audits, such as "direct access to medical records etc. by healthcare professionals at other medical institutions for quality control with permission from the administrator of the participating institution."
12） Refusal of consent and withdrawal of consent
Refusal to consent prior to participation in the study is free, and withdrawal after having given consent is free, thereby not causing undue medical disadvantage.
13） Protecting human rights
Every effort should be made to ensure that personal information, such as names, is kept confidential.
14） Secondary use of data

The possibility of secondary use of data obtained from this study in Japan and overseas (ancillary studies, meta-analyses, etc.) only when approved by either committee in JCOG

15) Method of disclosure of information on the study
The study is registered and published in jRCT※. In addition, the results of clinical studies should also be published in jRCT (＊ Databases (Japan Registry of Clinical Trials) https://jrct.niph.go.jp/ prepared by the MHLW as stipulated in Paragraph 1 in Article 24 of Clinical Trials Act Enforcement Regulations)

16) Freedom of questions
Explanation that investigators, written contact information for consultations on study details, on the Principal Investigator and the Study Coordinator, and freely asking questions about study and treatment

17) Explanation of the use and burden of medicinal products not covered by insurance

18) Central pathological review

19) Central image review for response

20) Sample collection for ancillary studies

13.2.2. Consent

Explain the study, give sufficient time to think, confirm that the patient understood the study well, and ask for participation in the study. If the patient agrees to participate in the study, the written informed consent form in the appendix will be used to obtain the patient's own signature. The site investigator or the subinvestigator confirms that the study consent form contains the name of the physician who provided the explanation and the date of explanation, the name of the patient who gave informed consent, and the date of informed consent.

In addition, when it is not possible to read the documents due to visual impairment, etc., but the details can be understood by verbal explanation, or the documents can not be signed due to limb disorders, etc., but the documents can be read and understood, signatures may be obtained from the proxy author under the consent by the patient. However, the signature of the proxy author should be based on this study's consent, and should be described as "Signing by the proxy author" and "Relationship with the patient" so that the person can be found to be the proxy author.

Two copies of the consent form will be provided, one will be handed over to the patient, and one will be stored by the site coordinator. Original copies will be stored in the medical records or in the archives specified by the participating institution.

13.2.3. Response to inquiries, consultations, etc. after consent

In principle, the investigator or subinvestigator of the relevant patient's participating institution responds to any consultation related to the study by the patient or his/her family after registration. If it is unclear how to respond, respond in consultation with Principal Investigator, Study Coordinator, the Group Secretariat, Group Chair, JCOG Data Center/Operations Office, etc. in accordance with the content of the consultation.

13.2.4. Withdrawal of consent

After obtaining informed consent for participation in the study, consent will be withdrawn if the patient expressed withdrawal of consent.

Withdrawal of consent refers to withdrawal of consent to participate in research and is distinguished from refusal to continue protocol treatment (below (i)). If the withdrawal of consent is expressed, clarify whether (ii) or (iii) below and promptly notify JCOG Data Center. If consent is withdrawn, record it in the medical record as well as (ii) or (iii).

Data Center discontinues subsequent follow-up requests according to the protocol in case of (ii) withdrawal of consent, In the case of (iii) full withdrawal of consent, the data of the patient are removed from the database when it is confirmed that the patient has full withdrawn of consent.

The procedures for discontinuation of the patient's follow-up request and removal of patient data will be specified separately in the procedural manual, and the completion of each task will be reported to Principal Investigator and Study Coordinator.

(i) Patient refusal: Refusal to continue subsequent protocol treatment (follow-up continues).

(ii) Withdrawal of consent: Withdrawal of consent to participate in the study and termination of all subsequent treatment and follow-up in accordance with the study protocol. Research use of data prior to withdrawal of consent is permitted.

(iii) Full withdrawal of consent: Withdrawal of consent to participate in the study and unavailability of all data from the time of patient registration, including information at registration.

In addition, some medical institutions may request that a "withdrawal of consent" form be prepared as a written document. However, in the event of withdrawal of consent, written expressions of willingness are required to increase the psychological barriers to withdrawal of consent (i.e., it is difficult to withdraw consent), and it is considered unwanted from the viewpoint of protecting human subjects. Therefore, in JCOG, written expressions of willingness are not mandatory for withdrawal of consent, verbal withdrawal of consent is valid, and the "withdrawal of consent" form is not prepared. If "withdrawal of consent" form is required by the participating institution, it should be prepared by the institution.

- This should be used in studies conducted in accordance with the Ethical Guidelines for Medical and Health Research Involving Human Subjects.

The procedures for discontinuation of the patient's follow-up request and removal of patient data will be specified separately in the procedural manual, and the completion of each task will be reported to Study Chair and Study Coordinator.

(i) Patient refusal: Refusal to continue subsequent protocol treatment (follow-up continues).

(ii) Withdrawal of consent: Withdrawal of consent to participate in the study and termination of all subsequent treatment and follow-up in accordance with the study protocol. Research use of data prior to withdrawal of consent is permitted.

(iii) Full withdrawal of consent: Withdrawal of consent to participate in the study and unavailability of all data from the time of patient registration, including information at registration.

In addition, some medical institutions may request that a "withdrawal of consent" form be prepared as a written document. However, in the event of withdrawal of consent, written expressions of willingness are required to increase the psychological barriers to withdrawal of consent (i.e., it is difficult to withdraw consent), and it is considered unwanted from the viewpoint of protecting human subjects. Therefore, in JCOG, written expressions of willingness are not mandatory for withdrawal of consent, verbal withdrawal of consent is valid, and the "withdrawal of consent" form is not prepared. If "withdrawal of consent" form is required by the participating institution, it should be prepared by the institution.

13.3. Protection of personal Information and patient identification

JCOG recognizes that information on privacy, such as personal information and medical information, should be protected and handled carefully, based on the principle of respecting the personality of individuals, and has formulated JCOG Privacy Policy, and will take all possible measures to protect privacy. For more information, see JCOG website (http://www.JCOG.jp/).

13.3.1. Policies, legislation, and norms followed by JCOG

In conducting JCOG study, JCOG follows, in principle, JCOG Privacy Policy as well as the following laws and norms depending on the content of the research. If other laws, norms, and policies are applicable, they should be followed.

- Clinical Trials Act (Law No. 16, 2017).
- Act on the Protection of Personal Information (Law No. 57, 2003, Final Amendment: Law No. 65, September 9, 2015).
- Helsinki Declaration (Translation by the Japan Medical Association)
- Ethical Guidelines for Medical and Health Research Involving Human Subjects (No.1 of Notice of the Ministry of Education, Culture, Sports, Science and Technology and the Ministry of Health, Labour and Welfare, 2017).

13.3.2. Use of personal information objective, items to be used, and methods of use

1) Objective of use

In accordance with the basic philosophy "Providing the best treatment to more patients," JCOG uses personal information, etc. of patients for objective of "Identifying patients and conducting surveys not only during treatment but also for a long period after end of treatment in order to obtain the correct results of clinical studies, and appropriately managing the acquired information."

2) Items to be used

Information to identify individuals who will be used by JCOG as minimally require for identification and inquiry of patients is as follows.

Medical record number, date of birth, initials, and registration number, Pathology specimen number

In other words, information that can identify individuals other than those listed above, such as the patient's name, is not informed to Data Center by participating institutions, and if they are falsely informed, they should be destroyed without using the recording medium or stored after performing appropriate processing, such as masking, which is incapable of reading.

3) Method of use

Personal information of patients used by JCOG will be collected by entering the CRFs etc. by researchers at participating institutions and submitting them to Data Center either by JCOG Web Entry System, mailing, or handover as a rule. However, telephone calls will be used only for patient registration where prompt contact is necessary.

In addition, in order to confirm the accuracy of the collected information, inquiries regarding various types of CRFs, including personal information, between Data Center and researchers at medical institutions are limited to either JCOG Web Entry System, mailing, or handover. Only the more anonymous registration number should be used when interacting with e-mail inquiries, and medical record numbers and initials should not be used.

13.3.3. Preparation of records for provision of samples and information, etc. at participating institutions

The investigator at each site will prepare records for this study of enrolled patients. The matters prescribed by the Ordinance of the Ministry of Health, Labour and Welfare (Article 53 of Clinical Trials Act Enforcement Regulations) are as follows.

- Identification of clinical-research subjects
- Items related to medical care and testing for subjects personnel in clinical studies
- Items related to participation in clinical research
- Other matters necessary to conduct clinical research

13.3.4. Source documents used in clinical studies (JCOG trials conducted under Clinical Trials Act)

The source documents for clinical studies used in this study refer to all records used for diagnosis and treatment, including medical records (including worksheets, etc.), laboratory test records, diagnostic images used for diagnosis, pathological diagnosis report, images used for response evaluation, and informed consent documents, of enrolled patients in this study. These should be available for direct access for monitoring (14.1.) and audit (14.2.) conducted by Data Center, Operations Office, Certified Review Board and regulatory persons

13.3.5. Storage of samples and information

Samples and information of enrolled patients on this study shall be stored in accordance with Article 53 of Clinical Trials Act Enforcement Regulations (MHLW ordinance No. 17, 2018). The retention time of records related to this study at participating institutions and the retention time of source documents will be 5 years from the date of completion of the study. It is recommended that the records be stored for as long as possible after the expiration date.

The retention time of data collected in JCOG Data Center will be semi-permanent in view of the possibility of long-term follow-up and secondary use for other studies. In addition, as records for the provision of samples and information, the study protocols and the model informed consent forms will be stored semi-permanently in JCOG Data Center.

13.3.6. Anonymization and control of response tables

In JCOG studies, information that can clearly identify individuals, such as patients' names, is not collected, and individuals are identified using registration numbers and medical record numbers (anonymized). Correspondence tables (not always in the form of a table) of information and registration numbers that can clearly identify individuals by themselves, such as patient names, are positioned as present at each participating institution and are appropriately managed according to the policy of participating institutions so that the identification of enrolled patients can be ensured.

13.3.7. Secondary use of data

Data from this study may be used in Japan or abroad for secondary use (e.g., meta-analysis) only if approved by the relevant committee of JCOG (e.g., Protocol Review Committee, Data and Safety Monitoring Committee). However, when providing data to external organization(e.g., a meta-analysis), data are provided so that it is not possible to identify individuals.

Secondary use of data should be disclosed on JCOG website to ensure opportunities for patients to refuse using data.

13.3.8. Safety management responsibility system

JCOG Data Center establishes a privacy protection control manager and a privacy protection manager and takes various safety control measures to minimize the risks of information leakage when using personal information, etc.

13.3.9. Response to request of disclosure of patient information

In the event that JCOG is asked to disclose privacy-related information, etc., the responder shall, in principle, be a researcher (site investigator, subinvestigator) at the institution of the relevant patient.

13.3.10. Receipt of inquiries about JCOG

General inquiries and complaints about privacy policies will be received by either mail, e-mail, or fax below.

Inquiry liaison: JCOG Data Center Privacy Protective

Postal destination :〒104-0045 Tsukiji 5-1-1, Chuo-ku, Tokyo
Clinical Research Support Office, National Cancer Center Hospital
E-mail :JCOG_privacy@ml.JCOG.jp
FAX :03-3542-3374

13.4. Adherence to the protocol

Researchers participating in this study will adhere to this protocol unless they compromise patient safety and human rights.

13.5. Application to Certified Review Board and Notification of Implementation Plans

When conducting this study, the approval of Certified Review Board and the permission by the administrator of each institution to conduct the study using this protocol and written informed consent from patients must be obtained. Prior to the commencement of the study, the implementation plan ※1 shall be submitted to the Minister of Health, Labour and Welfare, and the test data shall be published to jRCT※2. Principal Investigator is responsible for applying to Certified Review Board, submitting Implementation Plans to the MHLW, and registration in jRCT, and JCOG Operations Office supports these application procedures.

※ 1 Form No.1 of the Ministerial Ordinance stipulated in Article 39, Paragraph 1 of the Ordinance for Clinical Trials Act Enforcement Regulations

※ 2 Databases (Japan Registry of Clinical Trials) prepared by MHLW as specified in Article 24, Paragraph 1 of Clinical Trials Act Enforcement Regulations https://jrct.niph.go.jp/

13.5.1. Procedures for new application

<u>1) Procedures performed by Principal Investigator</u>

<Procedures from initial submission to study initiation>

(i) Review and approval of the study protocol by JCOG Protocol Review Committee (ver.1.0.0).

(ii) Principal Investigator will prepare the following documents for this study and submit these documents to Certified Review Board for review through JCOG Operations Office.

- ➢ New Review Request Form (Unified Form No.2 for Clinical Research)
- ➢ Implementation Plan (Ministerial Ordinance Form No.1)
- ➢ Study plan (this protocol) (including response to "disease or the like")
- ➢ Informed consent form
- ➢ Documents describing summary of drugs, etc. (e.g., package inserts of drugs used as a part of protocol treatment)
- ➢ List of Subinvestigators (Unified Form No.1 for Clinical Research)
- ➢ Conflict of Interest Management Standard (Guidance Form A), Conflict of Interest Management Plan (Guidance Form E) (see 13.X.)
- ➢ Other documents to be submitted when prepared (review documents of JCOG Protocol Review Committee, sample Case Report Forms, and a draft agreement on funding with the marketing authorisation of drugs, etc. or its special associates)

(iii) Modifications such as protocols and informed consent forms are made to review opinions submitted by Certified Review Board as needed.

※ Response to the indications by Certified Review Board: Prepare modified versions with review and approval by the Director of Data Center if modification of the protocol or informed consent forms is needed (ver.1.0.1, ver.1.0.2, ver.1.0.3…). .

(iv) After Certified Review Board approval is obtained, JCOG Operations Office should have Certified Review Board approval date and the approved version number on the cover page of the protocol and informed consent form and appear on JCOG website's Protocol Download page.

(v) Principal Investigator will use Certified Review Board review results notification and the documents submitted in ② to obtain the approval of the administrator of the institution which he/she belongs to, send these documents to the site investigators of all participating institutions described in the Implementation Plan, and request that the administrators of each participating institution permit conduct the research.

(vi) JCOG Operations Office shall apply for jRCT registration under the supervision of Principal Investigator after obtaining the permission by the administrators of all participating institutions described in the Implementation Plan and confirming that the patient registration system is open. "Study progress" in the trial registration will be registered as "being recruited."

(vii) JCOG Operations Office will inform Principal Investigator that jRCT registration has been completed after submission of the registration application. Principal Investigator will output the Implementation Plan from jRCT and submit the Implementation Plan, informed consent form, and Certified Review Board review result notification to the MHLW (Local Health and Welfare Bureau, which is responsible for the location of Certified Review Board; the same below). After submission, Principal Investigator will promptly inform Certified Review Board described in the Implementation Plan. In addition, Principal Investigator shall promptly report the submission of the Implementation Plan to the administrator of the institution to which he/she belongs, and provide this information to the site investigators of all participating institutions listed in the Implementation Plan and JCOG Operations Office.

(viii) After confirming that the notification of the Implementation Plan, etc. to the Local Ministry of Health and Welfare has been accepted and that jRCT's status has been updated from "under registration application" to "registration open," Principal Investigator will inform JCOG Operations Office that it has become "registration open." JCOG Operations Office opens JCOG Data Center's patient registration system and provides trial initiation announcements to study group. The date of jRCT publication is the start date of the study. "】

JCOG Operations Office should be contacted for procedures involving the addition of participating institutions after the initial submission.

"16.X. Medical institutions" changes (adding or replacing participating institutions) correspond to changes in the content of the protocol, then the change procedure shall be performed in accordance with the "13.6.2.Procedures for

when a change in the protocol occurs after the start of the study"

2) Procedures performed by the investigators at each participating institution

Following Certified Review Board approval, the investigator at each participating institution will obtain permission to conduct the research by the administrator of the participating institution using a set of documents received from Principal Investigator (Certified Review Board review results notification and documents submitted to Certified Review Board). The site investigator shall promptly send a copy of the letter of permission of the relevant institution to the Data Center after obtaining the permission by the administrator to conduct the research.

If the study is Specified Clinical Trial under Clinical Trials Act, the site investigator who has been informed that the Implementation Plan has been submitted to the MHLW by Principal Investigator should promptly report that the Implementation Plan has been submitted to the administrator of the affiliated institution.

3) Permission to conduct research at participating institutions

The procedures for obtaining permission for conducting research from the administrator of the affiliated institution shall be in accordance with the regulations of each institution.

When a copy of the institutional approval form is sent to JCOG Data Center, either the site investigator or the site coordinator should send the copy. Original copies of the institutional approval form will be stored by the site coordinator, and copies will be stored by JCOG Data Center.

When a affiliated institution has restrictions on providing personal information, such as medical record numbers, when sending copies of the institutional approval form to JCOG Data Center, the site coordinator shall communicate that personal information cannot be provided, and also send documents, such as the corresponding table of the ID number for registration and the medical record number, which is specified in JCOG privacy policy.

In addition, the informed consent form for patients approved by Certified Review Board may not be modified except for the contact information of the institution or prespecified selection items. A common protocol will be used for all institutions, since no changes in the protocols will be permitted for each institution. If it is necessary to change the content of the protocol and informed consent form, consult with Principal Investigator and Study Coordinator if the administrator of the institution asks for modification of the protocol and the text of the informed consent form in order to make the change as the protocol and informed consent form used by all institutions.

13.5.2. Procedures for changing the study plan after the start of the study

1) Procedures performed by Principal Investigator

If any of the changes of (1), (2), or (3) below occurs in the conduct of this study from the <Procedures from the initial application to the commencement of the study> in "13.6.1. Procedures at the time of new application", Principal Investigator shall hear Certified Review Board's opinions by applying for a change to Certified Review Board.

The procedures for applying for changes to the Certified Review Board are in accordance with "13.6.1. Procedures at the time of new submission" in the procedures from initial submission to the start of the study in ②, ③, and ④, respectively. In the absence of a change in the Implementation Plan, notification to the MHLW is not necessary. In the event that a change in the Implementation Plan occurs, notification to the MHLW is required. If Principal Investigator becomes aware of the planned change in the Implementation Plan, he or she should promptly contact JCOG Operations Office. JCOG Operations Office will assist in the application procedure for change to Certified Review Board and registration in change to jRCT.

（1） When the content of the protocol or informed consent form is changed (when it is amendment or revision in 13.7.1.).

（2） When the protocol or informed consent form is not changed, but the Implementation Plan (registered in jRCT) is changed.

（3） When changing Conflict of Interest Management Standards or Conflict of Interest Management Plans

Because notification of changes in the Implementation Plan (registered in jRCT) to the MHLW except for changes in research progress must be done in advance, Principal Investigator should inform site investigators and JCOG Operations Office of the change including the replacement of the investigator or the subinvestigator at each

participating institution. In addition, inform other investigators as soon as there are any changes that need to be made known.

Principal Investigator shall promptly report to the administrator of the institution to which he/she belongs and provide information to other research investigators when informed by Certified Review Board.

Procedures when it is necessary to notify changes to the Certified Review Board and the MHLW before changes are made.

1. Changes to the Implementation Plan

If changes to any of the above (1), (2), or (3) are made to the Implementation Plan (registered in jRCT) after hearing Certified Review Board's opinions, JCOG Operations Office should enter changes to jRCT under the supervision of Principal Investigator. In addition, Principal Investigator submits the following notification to the MHLW. After the date of coming into force of the predefined changes, the study shall be conducted in accordance with the changes.

- Notification of Change in Implementation Plan (Form No.2 of the Ministerial Ordinance)
- Implementation Plan after the change (output of the change in jRCT)
- Certified Review Board Review Results Notification

※ On institution transitioning to JCOG Collaborating Institution by replacement of participating institution: For institutions where patients were not enrolled prior to transfer to collaborating institution, Principal Investigator should remove it from the participating institutions by notifying changes in the Implementation Plan.

Procedures when it is necessary to notify a change to the Certified Review Board and the MHLW after the change***

1. Change in progress

(2) Among the changes in the Implementation Plan (registered in jRCT), the change in "3. Issues related to checking the implementation of Specified Clinical Trials (2) Specified Clinical Trials progress" shall be made without delay after the change. Under the supervision of Principal Investigator, JCOG Operations Office shall change "Research Progress" in jRCT. Subsequently, Principal Investigator will promptly apply for a change to Certified Review Board. After obtaining approval from Certified Review Board, Principal Investigator will submit the following notification to the MHLW.

- Notification of Change in Implementation Plan (Form No.2 of the Ministerial Ordinance)
- Implementation Plan after the change (the content of the change in jRCT was outputted)
- Certified Review Board Review Results Notification].

2. Changes in Implementation Plan after registration of the first patient

At the time of the initial submission, the "date of registration of the first patient" in the Implementation Plan is provided in a blank space. The Implementation Plan should be changed without delay after registration in the first patient. In this instance, JCOG Operations Office enters and registers the "First Patient Registration Date" in jRCT under the supervision of Principal Investigator. Subsequently, Principal Investigator will promptly apply for a change to Certified Review Board.

- Notification of Change in Implementation Plan (Form No.2 of the Ministerial Ordinance)
- Implementation Plan after the change (output of the change in jRCT)
- Certified Review Board Review Results Notification]

3. Minor changes specified by MHLW ordinance (application for change to Certified Review Board is not required)

If the following minor changes are made to Implementation Planning and the registration of jRCT, Principal Investigator will not need to hear the opinions of Certified Review Board and will notify Certified Review Board of the changes within 10 days of the date of the change. In addition, a notification (Form No.3) shall be submitted to the MHLW.

Scope of minor changes to the Implementation Plan of Article 42 of Clinical Trials Act Enforcement Regulations

- A change in the name of a person engaged in Specified Clinical Trials that does not involve a replacement of the person engaged in Specified Clinical Trials.
- Changes due to a change in the name or address number of the area

2) Procedures performed by the investigators at each participating institution

If any changes occur in the part of the protocol that corresponds to the institution to which he/she belongs, (4) Items related to investigators in multi-institutional studies, inform Principal Investigator and JCOG Operations Office of the changes to be made prior to the changes. Depending on the content of the change, the investigator shall confirm the confirmation of institutional requirements, prepare documents for conflicts of interest and submits to Certified Review Board such as lists of subinvestigators, and inform Principal Investigator and JCOG Operations Office.

The content of the most recent Implementation Plan for the institution to which they belong (the same as the content of jRCT registration) should be checked on jRCT website (https://jrct.niph.go.jp/).

13.5.3. Review and approval of study progress and study continuation (periodic reports)

Principal Investigator will report periodic reports on the progress of the study, the occurrence of adverse events, and conflict of interest management (see 13.X.1.⑱) to the administrators of their institutions and report them to Certified Review Board. Within two months after the expiration of each year from the date of submission of the Implementation Plan to the Minister of Health, Labour and Welfare.

When the report is made to Certified Review Board, Principal Investigator shall promptly inform the investigators of other participating institutions of the fact. The investigator who receives the information shall promptly report the details of the information to the administrator of the institution to which he/she belongs.

If the study falls under Specified Clinical Trials above Clinical Trials Act, Principal Investigator will report to the MHLW regarding the implementation status of Specified Clinical Trials. Periodic reports to Certified Review Board shall be made within 1 month of the date on which the results on the appropriateness of continuation of the relevant Specified Clinical Trials are obtained. The report shall be made by submitting the attached Form 3[※] to the Minister of Health, Labour and Welfare.

※ Enforcement of Clinical Trials Act Enforcement Regulations, etc. (Notification No. 0228-1 of the Sector of Economics, Ministry of Health, Labour and Welfare, Notification No. 0228-1 of the Notification No. 1 of the Evaluation and Development Division, Health Policy Bureau, Ministry of Health, Labour and Welfare, dated February 28, 2018)

13.6. Protocol revision/amendment

13.6.1. Categorization of protocol changes and procedures for changes

For a change in the protocol, the Protocol Revision Application must be submitted to Data and Safety Monitoring Committee Office prior to submission to Certified Review Board [in the case of Specified Clinical Trials, add the following: "and report to the MHLW"] (see 13.6.2).

JCOG deals with the changes in the content of the protocol after approval by the Protocol Review Committee, divided into amendment and revision. Data and Safety Monitoring Committee Office will distinguish between amendment and revision, so all of applications are submitted as revision. If it is classified as an amendment by the Secretary-General of Data and Safety Monitoring Committee, it is reviewed by Data and Safety Monitoring Committee. If it is classified as a revision, Secretary-General of Data and Safety Monitoring Committee will issue a verification form and will not be reviewed by Data and Safety Monitoring Committee. We also distinguish the addition of supplementary explanations that do not fall into a change in protocol content as "Memorandum". Definitions and handling are as follows:

When the protocol or informed consent form is revised or amended, Principal Investigator will promptly distribute the most recent protocol or informed consent form to those involved in the study. Individuals involved in the study should always conduct the study in accordance with the most recent protocol since the dates of entry into force of the amendment/revision.

1) Amendment

Partial protocol change which meets one or more of the followings: i) Potential to increase the risk of patients enrolled in the study, ii) Having substantial effects on primary endpoint of the study, iii) having essential effects on the study's implementation structure.

The amended version of the protocol and informed consent form version numbers are shown as in 2.0.0, 3.0.0, and 4.0.0….

Approval by the Group Chair and the Data Center Director must be obtained prior to submission to Data and Safety Monitoring Committee.

When classified as "amendment" by the Secretary-General of Data and Safety Monitoring Committee, review of changes by Data and Safety Monitoring Committee is performed prior to Certified Review Board review. After the protocol amendment has been approved by Data and Safety Monitoring Committee, an application for a change in the protocol will be submitted to Certified Review Board through JCOG Operations Office (at this time the version number is ver. 2.0.0, 3.0.0, 4.0.0…). When the protocol was changed based on the review opinions of Certified Review Board, the version number is ver. 2.0.1, ver.2.0.2… If the protocol is changed by review of Certified Review Board, the change will be reported to Data and Safety Monitoring Committee, but in principle, the change will not be reexamined by Data and Safety Monitoring Committee. When a protocol change is approved by Certified Review Board, the protocol cover page should include the date of approval by Data and Safety Monitoring Committee and Certified Review Board [If Specified Clinical Trials, the following is added: "and a notification of the change in the Implementation Plan to the Regional Bureau of Health and Welfare should be made"].

Following Certified Review Board approval, permission for the contents of the amendment by the administrator of each institution shall be obtained. If permission is obtained, the site coordinator of each institution will send copies of the permission notice by the administrator of each institution to the Data Center. After the permission of the administrator of all institutions is obtained, the protocol changes will come into effect (during this time, the patient registration will not be suspended unless there is a special need). The actual date of entry into force will be announced by JCOG Operations Office. Researchers in all participating institutions conduct the study according to revisions approved by Certified Review Board since the date of entry into force.

Treatment and assessment of enrolled patients will be performed according to the pre-change version protocol until entry into force. Protocol deviations to enhance patient safety during treatment will be permitted if pre-change protocols, such as inadequate treatment modification criteria, threaten patient safety. If protocol deviations occur, they should be listed in the monitoring report.

2) Revision

Protocol change which meets all of the followings: i) does not have an increased risk for patients enrolled in the study; ii) does not have a substantial effect on primary endpoint of the study; iii) does not have an inherent effect on the system in which the study is conducted. Includes changes in protocols due to mistakes or changes in institution-specific information, changes in institution-specific information without changes in protocols (changes in Implementation Plans and jRCT registrations), and changes in conflicts of interest at individual institutions. In principle, suspension of patient registration is not performed in case of revision.

The revised version of the Protocol and Informed Consent Form version numbers are shown as in 1.1.0, 1.2.0, and 1.3.0….

Approval by the Group Chair and the Data Center Director must be obtained prior to submission to Data and Safety Monitoring Committee.

If classified as "Revision" by the Secretary-General of Data and Safety Monitoring Committee, the Secretary-General of Data and Safety Monitoring Committee issued a verification form, Data and Safety Monitoring Committee did not review the changes. The Principal Investigator submit an application for a change in the protocol to Certified Review Board through JCOG Operations Office (at this time, the version number is ver.1.1.0, 1.2.0, 1.3.0…). If the protocol was changed based on the indication by the Certified Review Board, version number shall be as ver.1.1.1, ver.1.1.2… When a protocol change is approved by Certified Review Board, the date of approval by the Certified Review Board should be entered on the protocol cover page. [If Specified Clinical Trials, the followings are added: "and a notification of the change in the Implementation Plan to the Regional Bureau of Health

and Welfare should be made"]

The date of entry into force of the protocol change will be 2 weeks after Certified Review Board approval date unless otherwise specified. [in the case of a Specified Clinical Trials, the followings are added: "The effective date shall be the date after the notification of change of the Implementation Plan to the Regional Bureau of Health and Welfare"]

The actual date of entry into force will be announced by JCOG Operations Office. Researchers in all participating institutions conduct the study according to revisions approved by Certified Review Board since the date of entry into force.

At that time, the site investigator should obtain permission by the administrator of each institution after the date of approval of Certified Review Board and before the date of entry into force. In this case, the protocol revision may be permitted with a report to the administrator of the institution. However, the procedures for obtaining permission from the administrator shall be in accordance with the regulations of each institution. For protocol revisions, confirm with the institution prior to initiation of the study to ensure that permission is obtained within the aforementioned time periods, and contact JCOG Operations Office if this is difficult. Reports to the administrator and approval form by the administrator at each institution need not be sent to the Data Center, but the original copy will be retained by the site coordinator as it will be checked during site visit audits.

Treatment and assessment of enrolled patients will be performed according to the pre-change version protocol until entry into force. Protocol deviations to enhance patient safety during treatment will be permitted if pre-change protocols, such as inadequate treatment modification criteria, threaten patient safety. If protocol deviations occur, they should be listed in the monitoring report.

3) Memorandum

Supplementary description of the protocol distributed from Principal Investigator/Study Coordinator to study personnel in objective, such as reduction of interpretive variation in sentences, and special precautions, rather than change of protocol content. Any form is used.

Approval by Group Chair and the Director of the Data Center is needed prior to distribution. Reporting to Data and Safety Monitoring Committee before distribution or immediately after distribution is required.

It is not necessary to include the protocol on the cover page.

13.6.2. Patient explanation and re-consent at the time of protocol amendment/revision

In the event of a change in the content of the study, the investigator or the subinvestigator shall provide appropriate explanation to the enrolled patients (regarding protocols based on revision, treatment, follow-up, etc.). In addition, if Certified Review Board comments that re-consent of enrolled patients in writing is required, informed consent should be obtained in writing.

13.7. Conflicts of Interest (COIs) involved in this study

13.7.1. COI management involved in this study

The COIs involved in this study will be managed according to the "Guidance for Conflict of Interest Management in Clinical Trials Act (Notification No.1130-17 of the PMSB dated November 30, 2018) ※ (Guidance) of the Division, Research and Development, Ministry of Health, Labour and Welfare, in accordance with the following:

※ http://www.mhlw.go.jp/stf/seisakunitsuite/bunya/0000163417.html

The format used for COI control should be the latest version of the guidance.

- Conflict of Interest Management Standards: Form A
- Reports of related companies, etc.: Form B
- Investigator Conflict of Interest Self-Report Form: Form C
- Conflict of Interest Confirmation Report: Form D
- Conflict of Interest Management Plan; Form E

1) Procedures for New Application

Conflict of Interest Management Standards

(i) All JCOG trials according to Clinical Trials Act will employ Conflict of Interest Management Standards (Form A) in accordance with guidance.

Request for Confirmation of Conflicts of Interest

(ii) At the time of initiation of the primary review of the protocol, JCOG Operations Office shall identify companies, etc. related to this study that require conflict of interest management based on information on drugs and medical devices specified in the Protocol Treatment (see 6.1.), enter the relationship with the company in Form B, and ask Principal Investigator to confirm the accuracy of the entry.

(iii) After checking the entries in Form B received from JCOG Operations Office, Principal Investigator will send a format to the site investigator and site coordinator at each participating institution and ask to confirm the entries in Form B, create Form C, and create Form E. In addition, a form shall be sent from Principal Investigator to those who correspond to the "person responsible for statistical analysis" and the "administrator other than Principal Investigator and Investigators" of the Implementation Plan, and they shall be asked to prepare Forms C and E.

Confirmation of Conflicts of Interest (participating institutions)

(iv) The site investigator or site coordinator at each participating institution will register the investigators, subinvestigators, and research associates involved in the study in JCOG Web Entry System. Investigators and subinvestigators registered in JCOG Web Entry System will be the reporters of conflicts of interest in the study.

(v) The site coordinator will request the investigator of the study to confirm the description of Form B received from Principal Investigator, create Form C, and create Form E. The investigator will then summarize conflict of interest management within the study site.

(vi) The investigator checks the forms received from Principal Investigator for any relevant items in Q2 through Q5 of Form B and notifies Principal Investigator through JCOG Operations Office within a week if any.

(vii) In the column of Form C [Persons requiring a Form C self-declaration of conflict of interest (Form C)] ※, the site investigator inputs the information of the investigator and subinvestigator registered in JCOG Web Entry System, and asks subinvestigator to prepare Form C.

※ The information entered in Form C is automatically entered in Form E. This entry should be consistent with the physician's information contained in the "List of Subinvestigators" (Clinical Trials Act Uniform Form No.1) submitted to Certified Review Board with Form E, and physicians who do not agree may not be involved in this study. Therefore, when entering the information in the ※ column for [those who require a Conflict of Interest Self-Reporting Form (Form C)], the investigator or subinvestigator list should be downloaded in JCOG Web Entry System and the physician listed in the Study Subinvestigator list should be entered without missing the person who will be the investigator and the subinvestigator in the study. If the information in the downloaded "Study Subinvestigator List" is not consistent with the physician involved in the study, the registered information should be updated in JCOG Web Entry System, and the "Study Subinvestigator List" with the most recent information reflected should be downloaded and used.

(viii) The investigator and the subinvestigator shall complete the necessary information regarding the relationship with the company, etc. described in advance in Form C, and submit Form C to the Conflict of Interest Confirmation Department of the institution to which he/she belongs. In doing so, the investigator also submits Form A.

(ix) The investigator receives the results of confirmation of conflicts of interest (Form D) of the investigator and the study subinvestigator from the institution to which he/she belongs.

(x) The investigator will confirm the content of Form A, Form B, and Form D of the investigator and all study subinvestigators to create Form E. The investigator will notify Principal Investigator through JCOG Operations Office of Form E and the Study Subinvestigator List downloaded in Form 7.

Confirmation of Conflicts of Interest (outside participating institutions)

(xi) The person responsible for statistical analysis and the person overseeing a study other than Principal Investigator or investigator shall complete, in the form received from Principal Investigator, the necessary information regarding the relationship with the company, etc. described in advance in Form C, and submit Form A and Form C to the Conflict of Interest Confirmation Department of the Affiliated Institution.

(xii) The person responsible for statistical analysis and the person overseeing studies other than Principal Investigator or investigator shall receive confirmation results (Form D) from the institution.

(xiii) The person responsible for statistical analysis and the person overseeing studies other than Principal Investigator and investigator shall confirm the content of Form A and Form D, prepare Form E, and notify Form E to Principal Investigator through JCOG Operations Office.

Description of Conflicts of Interest in the protocol and informed consent form

(xiv) Principal Investigator and JCOG Operations Office will review Form A and Form E received from participating institutions and, as appropriate, will accurately describe the conflicts of interest (study COIs) between the study and the drug marketing authorisation holder, etc. in the protocol and informed consent form. Conflicts of interest (personal COI) between the reporters of conflicts of interest in this study and the marketing authorisation of drugs, etc. (personal COI) can change over time, so the personal COI is not described in the protocol and informed consent form, and the information is updated on JCOG website as needed.

Certified Review Board review

(xv) Principal Investigator compiles and submits the Form E and Study Subinvestigator List of all institutions to Certified Review Board for review.

2) Procedures for new involvement with companies after the start of the study

(xvi) When a new involvement with companies (research COI) occurs in the study after the start of the study

a. In the event of any change in the enterprises involved in this study, etc. to be described in Q1 of Form B, repeat the procedures ② to ⑭. However, the site investigator and site coordinator roles in ④, ⑤ shall be assumed by the investigator at each institution. If a new study COI needs to be added to the protocol and informed consent form, a protocol revision (see 13.X.X.) should be submitted to Certified Review Board for review.

b. If a change occurs from Q2 to Q5 in Form B, the investigator at the participating institution affected by the change will change the appropriate description in Form B and update Form E and send it to Principal Investigator and JCOG Operations Office. A Principal Investigator who has received a Form E will revise the protocol as needed (see 13.X.X.) and submitted to Certified Review Board for review.

(xvii) In the event that a conflict of interest reporter is newly involved with a company (personal COI) after the start of the study

Conflict of interest reporters repeat procedures from ⑧ to ⑩ or from ⑪ to ⑬. However, if there is no change in Form E, these procedures will be performed at each institution but will not be sent to Principal Investigator and JCOG Operations Office. Principal Investigator will submit Post-change Form E sent from the site investigator to Certified Review Board for review. Personal COIs are also disclosed on JCOG website.

3) Procedures for periodic reporting

(xviii) Principal Investigator checks for changes in study COI and personal COI annually at the timing of periodic reporting and reports to Certified Review Board.

13.7.2. COI with companies involved in this study(study COI)

There are no conflicts of interest to disclose about relationship between the company manufacturing and marketing drugs specified as a part of protocol treatments in this study (See 6.1).

13.7.3. COIs of JCOG Committee members and JCOG Data Center/Operations Office staff

COIs of the committee members of JCOG committees, the staffs of the office of committees, and JCOG Data Center/Operations Office staff involved in the study will be managed by JCOG Conflict of Interest Committee in accordance with Clinical Trials Act control standards.

13.8. Compensation

In order to comply with Clinical Trials Act, this study must take necessary measures such as sing up for insurance and ensuring a system to provide medical care in order to compensate for the health damage caused by the study

and provide medical care.

Therefore, regarding the health damage caused by participating in this study, appropriate treatment according to the condition is provided similarly to the usual insurance medical care. In addition, this study will be covered by clinical study insurance, and the following will be compensated based on insurance conditions, and this will be explained to patients and understood.

13.9. Intellectual property

The results, data, and intellectual property rights obtained from this study are attributed to the followings: National Cancer Center, Principal Investigator, Study Coordinator, and Group Chair. Specific procedures and allocation must be determined through consultation among four parties. Whether the intellectual property related to Principal Investigator, Study Coordinator, Group Chair will belong to the individuals or the affiliated institution will be determined according to the agreements of the affiliated institution.

13.10. Disclosure of information on this study

Summary, progress, and main results of this study will be published on JCOG website (www.jcog.jp) and on jRCT (https://jrct.niph.go.jp/).

14. Monitoring and audit

14.1. Periodic monitoring

In this study, monitoring is performed in order to ensure that the study is conducted safely and in accordance with this protocol and that data are collected accurately. Periodic monitoring should be performed twice a year in principle, using the entered data on the CRFs collected in the Data Center. Specific procedures for periodic monitoring are provided separately in the Monitoring Plan.

The Data Center submits a "Monitoring Report" summarizing the results of central monitoring to Principal Investigator, Study Coordinator and investigators. Together, they are submitted to Group Chair, Data and Safety Monitoring Committee, and JCOG Chair.

The Monitoring Report is a material for periodic reports to be made every year from the date of submission of the Implementation Plan.

14.1.1. Monitoring items

① Registration status: number of registration - cumulative/by month, by arm/site
② Eligibility: ineligibles/potentially ineligible cases: by arm/site
③ Pre-treatment baseline factors: by arm
④ On/off-treatment, reason for treatment termination: arm/site
⑤ Protocol deviation: arm/site
⑥ Serious Adverse Events: arm/site
⑦ Adverse reaction/event: arm
⑧ Overall survival, progression-free survival (or relapse-free survival, etc.): all registered patients
⑨ Other issues related to study progress and safety (studies in accordance with Clinical Trials Act: status of occurrence of non-compliance and subsequent response, number of subjects for compensation, number of disease or the like reports in accordance with Article 13 of Clinical Trials Act)

14.1.2. Eligibility (Eligible/Ineligible)

For all registered patients, eligibility will be classified according to the following definitions as either: In monitoring, Data Center lists potentially ineligible cases in the "Evaluation of Eligibility" section of the monitoring report, and after review by Study Coordinator, confirms them to be either 1), 2), 9), or 99) with Group Chair approval prior to performing primary analysis.

Only 1) eligible shall be "eligible case", 2) post hoc ineligible,9) de facto ineligible and 99) violation of registration shall be "ineligible case". This is a category established from the perspective of analysis set setting.

In the study in accordance with Clinical Trials Act, "99) violation of registration" will be regarded as "major non-compliance" in Clinical Trials Act, and Principal Investigator will promptly report the situation to Certified Review Board as soon as possible. See 14.3. for management of non-compliance.

9) de facto ineligibles corresponds to "non-compliance" on Clinical Trials Act and is reported to the administrator of participating medical organizations with the submission of the monitoring report on which they were described (twice a year).

2) Post hoc ineligibles is not treated as "non-compliance" on Clinical Trials Act because it does not correspond to non-compliance with the study protocol, as discussed below.

1) Eligible

All information generated prior to registration meets all o the Patients Selection Criteria according to the methods and criteria specified in the study protocol.

2) Post hoc ineligible

The information generated after registration does not meet either Patients Selection Criteria, or the information generated prior to registration does not meet either Patients Selection Criteria by methods or criteria other than those specified in the protocol.

Examples)

(i) In the study for Stage II-III, bone scintigraphy performed immediately after registration revealed bone

metastases, and the patient was diagnosed as Stage IV. The protocol treatment was terminated.

(ii) In the study for early gastric cancer, bloody stools is seen after registration, and colonoscopy revealed advanced colorectal cancer (synchronous double cancer). Colectomy was performed after termination of the protocol treatment.

(iii) In the study for gastric cancer (adenocarcinoma), the institution's pathological diagnosis was changed to malignant lymphoma after registration.

9) De facto ineligible

Information generated prior to registration according to protocol-specified methods (performed in all cases) and criteria does not meet either Patients Selection Criteria. This includes cases where it is determined after registration that the information that occurred before registration had been incorrect.

Example: When the supervising physician reviews the CT images performed before registration as specified, there is obvious liver metastasis (if it is a mistake by the attending physician and it is considered that there is no future).

99) Violation of registration

Deliberately (falsely) enroll while knowing that Patients Selection Criteria is not met. Corresponds to a misconduct and treats it as a serious problem.

14.1.3. Protocol Deviations/Violations

Protocol deviations are defined as those in which treatment, such as drug administration, radiotherapy, or surgical resection, as well as laboratory tests and evaluation of toxicity and efficacy, etc. were not performed according to the protocols.

In monitoring, deviations that exceed a certain acceptable range limit for each study decided by the Data Center and Principal Investigator/Study Coordinator prior to or after the initiation of the study are listed in the monitoring report as "possible deviations" and are classified into one of the following categories after consideration by Study Coordinator and study groups: Except for those described in the protocol and monitoring report, the acceptable deviations agreed between the Data Center and Principal Investigator/Study Coordinator may be changed through periodic monitoring during the study, so they should be described in the supplemental material rather than in the text of the Monitoring Plan, and the Monitoring Plan should be described as "defining the acceptable ranges separately".

1) Violation

Any deviation from the protocol that is clinically inappropriate and caused by the treating physician/institution and that meets two or more following criteria shall be classified as a violation. [When conducted in accordance with Clinical Trials Act, the following shall be added. [In the study according to Clinical Trials Act, the violation shall be treated as "major non-compliance" and Principal Investigator should report the situation to Certified Review Board as soon as possible].

① Have a substantial impact on the assessment of study endpoints

② Intentional or systematic

③ Dangerous or remarkable deviation

For "violations", in principle, the content of each violation should be described in a paper when publishing.

2) Deviation

Deviations that do not fall into 1) violation or 3) acceptable deviation. If same kind of deviations are frequent, they should preferably be included in the publication of the article. They are classified as either of the following at the time of monitoring report review:

Because deviations correspond to "non-compliance" in Clinical Trials Act, they are reported (biannually) to the administrators of the institution with the submission of the monitoring report. Not to be "major non-compliance".

(i) Deviations - Undesirable and to be reduced

(ii) Deviations (unavoidable) - things that are not proactively reducing (e.g. delay by the New Year period, equipment breakdown, etc.)

(iii) Deviations (clinically relevant) - Those in which the decision of the treating physician/institution are positively affirmed (if a similar situation again arises similar deviations are considered desirable).

※ Deviations do not always mean that the treating physician at the institution is problematic. Since patient safety is a primary priority in clinical trials, deviations should rather be made by the medical judgment of the treating physician if the condition of the individual patient are considered to be dangerous when following the protocols. If the deviation is judged to be clinically relevant for the safety of the patient, it is recorded as ③ Deviation (clinically relevant). Clinically relevant deviations in a small number of patients are not required to be particularly problematic; however, protocol revision should be considered when multiple deviations occur because protocol specification is likely to be inadequate. However, deviations that are not intended to be safety (e.g., increased doses of anticancer drugs in the hope of increasing efficacy, shortened treatment periods not specified in the protocol) are not considered clinically relevant deviations.

3) Acceptable deviation

Deviations from protocols within acceptable range agreed by the entire JCOG, study groups, or Study Chair/Study Coordinator and Data Center, pre- or post-study initiation, on a trial-by-trial basis. When conducted according to Clinical Trials Act, the following are added. Not considered to be "non-compliance" in Clinical Trials Act. Deviations within the pre-specified acceptable ranges are not included in the monitoring report.

14.2. Site visit audits

In this study, site visit audits will be conducted to ensure the reliability of clinical research and the reliability of data and information collected by clinical research from the perspective of protecting human subjects in clinical research.

Site visit audits are conducted by auditors appointed by Principal Investigator by visiting a medical institution to confirm the approval documents of the medical institution, check the list of subinvestigators in the research institution, confirm the informed consent documents, and verify CRF entry data with medical records (direct access to source documents). Specific procedures for site visit audits are provided separately in the operating procedures.

The auditor shall report the audit report summarizing the audit results to Principal Investigator/Study Coordinator, site investigators. Together, the report will be submitted to Group Chair, Director of JCOG Data Center, Director of JCOG Operations Office, and JCOG Chair. Reports should also be submitted to the site investigators of the relevant groups and JCOG Executive Committee as appropriate.

14.2.1. Items to be audited

In the site visit audits, the following items are checked by direct access to source documents:

<Confirmation Items by Study>

- Approval documents (including initial approval forms, revision approval forms, and annual report approvals/reports) from administrators of medical institutions
- Management status of the protocol
- Contents of explanatory documents and informed consent forms

<Confirmation Items by Patient>

- Patient consent (presence or absence of consent form, signature, and date of consent)
- Implementation of pre-registration mandatory tests, eligibility for registration (inclusion criteria and exclusion criteria)
- Accuracy of reported data
  - Pre-treatment evaluation, course of treatment (protocol treatment)
  - Various test results (including diagnostic imaging reports and pathology reports)
  - Accuracy of test date, response evaluation, adverse event, survival or death information, and other reported data

<Other items>

- Presence or absence of study misconduct (possibility of false reporting, fabrication, or falsification)
- Improvement status of the items pointed out in the previous audit

14.2.2. Reporting of major non-compliance found in audits to Certified Review Board

Principal Investigator/Study Coordinator should report to Certified Review Board immediately when finding possible "major non-compliance (See 14.3.2.)" as a result of site visit audits.

14.3. Management of non-compliance

14.3.1. Non-compliance.

Non-compliance in Clinical Trials Act refers to the condition in which clinical research is not compliant with Clinical Trials Act Enforcement Regulations or study protocols. In the Clinical Trials Act Enforcement Regulations, etc. (February 28, 2018), non-compliance with regulations, study protocols, operation procedures, etc., and fabrication of falsification of research data, etc. are listed as examples.

If the site investigator knows that there is non-compliance, the site investigator should report to the administrator of the medical institution and inform Principal Investigator/Study Coordinator.

If site investigator find non-compliance (regardless of major non-compliance or not) prior to the implementation of central monitoring or site visit audits, the site investigator will promptly report to Principal Investigator/Study Coordinator and JCOG Data Center.

"14.1.3.2) Deviations" correspond to "non-compliance" in Clinical Trials Act. As described in 14.1.3.2), these deviations are reported by submitting monitoring reports or their excerpts or summaries issued twice a year to the administrators of the medical institution.

Changes in protocols and implementation plans associated with investigator transfer require Certified Review Board review and notification of implementation plans to the MHLW, therefore, require a certain time to complete the sequence of procedures. In addition, it is often difficult to complete the change procedure before the transfer because the transfer may not be open until just before the transfer. Therefore, even if the site investigator is absent for a certain period of time due to the transfer, the study does not fall into "non-compliance" in this study if the research management system is maintained by the subinvestigator and the medical care system of the enrolled patients who are surviving is ensured.

14.3.2. Major non-compliance

"Major non-compliance" is that affect the human rights and safety of subjects of clinical research, the study progress and the reliability of study results. Examples of "major non-compliance" in JCOG study are provided in. If major non-compliance is likely, Principal Investigator/Study Coordinator will report the situation to Certified Review Board immediately.

1) Major non-compliance with respect to eligibility

Violation of registration

- Enrolled intentionally (falsely) while knowing that eligibility criteria was not met
- Patient enrollment was performed without necessary informed consent, and protocol treatment was performed
- The source documents for the determination of eligibility cannot be identified (including the loss of the consent form).

2) Protocol violation

Violations that affect the increased risk of enrolled patients or that affect the reliability of the study results

- Significant violation in inclusion criteria or exclusion criteria
- Violation threatening patient safety in off-treatment criteria
- Serious violation of prohibited concomitant drug, prohibited concomitant treatment, etc.
- e.g. intentional or systematic non-compliance with protocol regulations

3) Other major non-compliance

- Study was conducted prior to Certified Review Board approval or prior to approval of site administrator
- The study was continued without providing information to the enrolled patient that could affect the willingness to continue to participate the study.
- Those judged to be research misconduct (fabrication or falsification of data, etc.)

- Any leakage of personal information or violation of human rights that may have a significant impact on the enrolled patient.

15. Special Instructions

15.1 Central pathology diagnosis and related matters

15.1.1 Central pathological diagnosis

In this study, the pathological tissues of enrolled patients will be collected and the central pathological diagnosis will be determined after the fact. Since the central pathological diagnosis is not performed in real time for each individual patient enrollment, the main analysis target and the decision on treatment strategy will be based solely on the pathological diagnosis at the institution. The details of the central pathological diagnosis procedure will be specified separately in the Central Pathological Diagnosis Procedure Manual.

Timing: Once a year throughout the study period.

Subjects: All registered patients

Methods: Pathological specimens (or duplicates made from the same paraffin block) used for eligibility criteria determination at the registered facilities will be collected, and after necessary staining (chromogranin A, synaptophysin) is added, pathological eligibility will be re-determined by two or more pathology judges (16.8 Pathology judges) appointed by the group representative. (16.8 Central Pathological Review Comittie).

Management of the collected specimens: The Hepatobiliary and Pancreatic Group Study Office will be responsible for the management of the collected specimens.

Staining: If additional staining is required, it will be performed by the Central Pathology Coordinator (16.7).

Notification of the central judgment to each facility:

After the results of the central pathological diagnosis are fixed, the research secretariat will notify the results of the judgment to the registered facility (facility coordinator) of each patient. At that time, the rationale for the decision should be attached in writing. If there is a difference between the institutional diagnosis and the central diagnosis, the facility principal investigator/facility coordinator reports the decision results to the facility pathologist and discusses it with the facility pathologist, and carefully decides on the final pathological diagnosis at the facility (whether to change the diagnosis or not) and what to do if the patient is under treatment (whether to change the treatment or not).

15.1.2 Providing information to the institutional pathologist

In view of the high level of difficulty in NEC pathological diagnosis and the unique nature of this study, which covers multiple target organs, the following information will be provided to institutional pathologists.

(1) Pre-registration pathological diagnosis consultation

Since it is anticipated that there will be cases of confusion in diagnosis at facilities, pre-enrollment pathology consultation will be available as needed, with the pathology judgment committee members of this study serving as consultants. Details of the consultation operation are described in the Central Pathology Procedures.

(2) Holding of pathology-related meetings

In this study, the research secretariat and the pathology secretariat first held a pathology-related meeting (attended by pathologists from participating institutions) on February 11, 2014, during which a lecture was given to form a consensus on diagnostic criteria according to the WHO 2010 classification. The slides used at the meeting were revised as necessary based on the discussion at the meeting and posted on the JCOG website for reference by pathologists at each facility.

15.1.3 Response to cases in which pathological diagnosis was performed at a facility other than the home facility

In any of the following cases, be sure to ask a pathologist at your own institution to make the diagnosis, and confirm that the diagnosis at your institution is also NEC before enrolling in this study.

(1) When borrowing only stained tissue specimens from a previous physician for registration

Register after obtaining permission from the attending physician to the previous physician to submit the borrowed tissue specimen to JCOG 1213 for central diagnosis (loan again) and to borrow the specimen for a long period of time (up to about 1 year).

(2) If you have received virtual slides of "all tissue specimens for which a pathological diagnosis has been made" from

your previous doctor

The patient will be registered after obtaining permission from the attending physician of the previous physician to submit the virtual slides provided by the previous physician for the central diagnosis of this study.

(3) If you have received a "borrowed" pre-stained tissue specimen and an "offered" unstained preparative from your previous physician

The borrowed stained tissue specimens from the previous physician can be promptly reviewed at the patient's own institution, but the provided unstained preparations require time for pathological diagnosis after immunostaining at the patient's own institution, which may cause a time lag. There have been cases in which patients were enrolled in the study based only on the results of pathological review of borrowed tissue specimens at their own institutions, and later the results of staining and pathological diagnosis of undyed preparations at their own institutions overturned the pathological diagnosis (diagnosis of a different disease was made), resulting in post-hoc ineligible cases. Therefore, we will consider the timing of registration after sufficient consultation with pathologists at our own institution to avoid the occurrence of posterior ineligible cases as much as possible. However, if there is enough time, it is preferable to enroll unstained preparations into the study after staining and pathological diagnosis is obtained at your own institution.

15.2. JCOG BioBank Japan (BBJ) Biorepository

This study will participate in the banking of blood samples (DNA/plasma) in JCOG BBJ Biorepository based on a common protocol for all JCOG studies (hereafter referred to as common banking).

Subjects:

Among patients who agreed to participate in this study, patients whose consent to shared banking was obtained.

Sample:

1) Whole blood

Blood sampling is performed before the start of the protocol treatment in this study in principle. However, blood sampling after initiation of protocol treatment is allowed. Blood samples of 7 mL×2 (total 14 mL) of venous blood are collected using a blood collection tube (for blood counting) with a EDTA Na dedicated to the shared banking of JCOG-BBJ Biorepository, and stored at 4°C at the respective institutions until they are handed over to the sample transport/processing company (See "JCOG-Biobank Japan Biorepository protocol" for details).

2) Pathological specimens

Archived pathological tissues in daily clinical practice such as surgery, biopsy and laboratory tests can also be used in future translational researches, but the type of pathology specimen, preparation method and tissue quantity required by the studies are varied and there is no consensus that it is efficient to bank pathological tissues in a certain way prospectively. In addition, there is the opinion that the sample deteriorates (DNA fragmentation) when the thin-section sample from pathological tissue is stored for a long time.

Consent on the use of archival pathological tissue after medical care should therefore be obtained at the time of consent to banking, but actual collection should be initiated on an individual basis by creating a protocol and defining the most appropriate procedure for the study content in the protocol.

16. Organization

Changes to this chapter are considered to be revision rather than amendment.

16.1. Main study fund (funding source) of this study.

Practical Research for Innovative Cancer Control from Japan Agency for Medical Research and Development
“Establishment of standard treatments for neuroendocrine carcinoma of the digestive system” JP15ck0106138, JP16ck0106138, JP17ck0106355, JP18ck0106355, JP19ck0106355, JP20ck0106618
National Cancer Center Research and Development Fund (23-A-22, 26-A-4, 29-A-3, 2020-J-3)
“Scientific research on multi-institutional trials to establish new standard treatment of solid tumors in adults”

16.2. Japan Clinical Oncology Group (JCOG)

JCOG is a multi-institutional clinical research group consisting of research teams funded by public research grants

mainly on National Cancer Center Research and Development Fund and Japan Agency for Medical Research and Development research costs that receive direct support for research by the Clinical Research Support Office of the National Cancer Center Hospital in accordance with JCOG Policy (http://www.jcog.jp/).

This study is conducted using JCOG research organisations and in accordance with the regulations set out by JCOG Executive Committee.

16.3. JCOG Chair

Yuichiro Ohe National Cancer Center Hospital

16.4. Study group and Group Chair

JCOG Hepatobiliary and Pancreatic Oncology Group

Group Chair: Junji FURUSE

Department of Medical Oncology, Kyorin University Faculty of Medicine

〒181-8611 6-20-2, Shinkawa Mitaka-shi, Tokyo

TEL:+81-422-47-5511

FAX:+81-422-44-1858

E-mail: jfuruse@ks.kyorin-u.ac.jp

Group Secretary: Hirohi Ishii

Clinical Research Center, Chiba Cancer Center

〒260-8717, 666-2 Nitona, 260-8717, Chuo-Ku, Chiba

TEL:+81-43-264-5431

FAX:+81-43-264-8680

E-mail: hirishii@chiba-cc.jp

JCOG Stomach Cancer Study Group:

Group Chair: Masanori Terashima

Shizuoka Cancer Center

〒411-8777 1007 Shimonagakubo, Nagaizumi-cho, Sunto-gun, Shizuoka Prefecture

TEL:+81-55-989-5222

FAX:+81-55-989-5783

E-mail: m.terashima@scchr.jp

Group Secretary: Takaki Yoshikawa

National Cancer Center Hospital

〒104-0045 5-1-1 Tsukiji, Chuo-ku, Tokyo

TEL:+81-3-3542-2511

FAX:+81-3-3542-3815

E-mail: tayoshik@ncc.go.jp

Group Secretary: Narikazu Boku

National Cancer Center Hospital

〒104-0045 5-1-1 Tsukiji, Chuo-ku, Tokyo

TEL:+81-3-3542-2511

FAX:+81-3-3542-3815

E-mail: nboku@ncc.go.jp

JCOG Japan Esophageal Oncology Group

Group Chair: Yuko Kitagawa

Keio University Hospital

〒160-8582 35 Shinanomachi, Shinjuku-ku, Tokyo

TEL:+81-3-3353-1211

FAX:+81-3-3355-4707

E-mail: kitagawa@a3.keio.jp

Group Secretary: Ken Kato
National Cancer Center Hospital
〒104-0045 5-1-1 Tsukiji, Chuo-ku, Tokyo
TEL:+81-3-3542-2511
FAX:+81-3-3542-3815
E-mail: kenkato@ncc.go.jp

16.5. Study Chair (Principal Investigator)

JCOG Hepatobiliary and Pancreatic Oncology Group
Takuji Okusaka
National Cancer Center Hospital
〒104-0045 5-1-1 Tsukiji, Chuo-ku, Tokyo
TEL: +81-3-3542-2511
FAX: +81-3-3542-3815
E-mail: tokusaka@ncc.go.jp

JCOG Stomach Cancer Study Group:
Narikazu Boku
National Cancer Center Hospital
〒104-0045 5-1-1 Tsukiji, Chuo-ku, Tokyo
TEL: +81-3-3542-2511
FAX: +81-3-3542-3815
E-mail:nboku@ncc.go.jp

JCOG Japan Esophageal Oncology Group
Ken Kato
National Cancer Center Hospital
〒104-0045 5-1-1 Tsukiji, Chuo-ku, Tokyo
TEL: +81-3-3542-2511
FAX: +81-3-3542-3815
E-mail: kenkato@ncc.go.jp

16.6. Study Coordinator

JCOG Hepatobiliary and Pancreatic Oncology Group
Chigusa Morizane
National Cancer Center Hospital
〒104-0045 5-1-1 Tsukiji, Chuo-ku, Tokyo
TEL: +81-3-3542-2511
FAX: +81-3-3542-3815
E-mail: cmorizan@ncc.go.jp

JCOG Stomach Cancer Study Group:
Nozomu Machida
Kanagawa Cancer Center
〒241-8515, 2-3-2 Nakao, Asahi-Ku, Yokohama
TEL:+81-45-520-2222
FAX:+81-45-520-2202
E-mail:n-machida@kcch.jp

JCOG Japan Esophageal Oncology Group

Ken Kato
National Cancer Center Hospital
〒104-0045 5-1-1 Tsukiji, Chuo-ku, Tokyo
TEL: +81-3-3542-2511
FAX: +81-3-3542-3815
E-mail: kenkato@ncc.go.jp
Yoshitaka Honma
National Cancer Center Hospital
〒104-0045 5-1-1 Tsukiji, Chuo-ku, Tokyo
TEL: +81-3-3542-2511
FAX: +81-3-3542-3815
E-mail:yohonma@ncc.go.jp

16.7. Central Pathological Review Coordinator

Nobuyoshi Hiraoka
National Cancer Center Hospital
〒104-0045 5-1-1 Tsukiji, Chuo-ku, Tokyo
TEL: +81-3-3542-2511
FAX: +81-3-3542-3815
E-mail: nhiraoka@ncc.go.jp
Shigeki Sekine
National Cancer Center
〒104-0045 5-1-1 Tsukiji, Chuo-ku, Tokyo
TEL: +81-3-3542-2511
FAX: +81-3-3542-3815
E-mail: ssekine@ncc.go.jp

16.8. Central Pathological Review Coordinator

Nobuyoshi Hiraoka (National Cancer Center)
Noriyoshi Fukushima (Jichi Medical University)
Nobuyuki Ohike (Showa University Fujigaoka Hospital)
Ryoji Kushima (Shiga University of Medical Science)
Mitsuya Iwafuchi (Scool of Health Sciences Faculty of Medicine, Niigata University)
Tetsuo Ushiku (The University of Tokyo)

16.9. Participating sites (participating institutions)

HBP	SCSG	JEOG	Participating institutions
○			Sapporo-Kosei General Hospital
○		○	Hokkaido University Hospital
	○		Keiyukai Sapporo Hospital
	○	○	Iwate Medical University
		○	Tohoku University Hospital
	○		Miyagi Cancer Center
○	○	○	Tochigi Cancer Center
○			Jichi Medical University
○	○	○	Saitama Cancer Center
○	○	○	National Cancer Center Hospital East
○		○	Chiba Cancer Center
○		○	Chiba University, Graduate School of Medicine
○	○	○	National Cancer Center Hospital
○			Kyorin University Faculty of Medicine
○			National Center for Global Health and Medicine (NCGM)
○	○	○	Cancer Institute Hospital of Japanese Foundation for Cancer Research
	○	○	Toranomon Hospital
○			Teikyo University School of Medicine
○			St.Marianna University School of Medicine
○	○	○	Kanagawa Cancer Center
○	○		Yokohama City University Medical Center
○	○		Niigata Cancer Center Hospital
○			Toyama University Hospital
○			Kanazawa University School of Medicine
	○		Ishikawa Prefectural Central Hospital
	○		Ogaki Municipal Hospital
○	○	○	Shizuoka Cancer Center
○	○	○	Aichi Cancer Center Hospital
		○	Kyoto University Hospital
	○	○	Osaka University Graduate School of Medicine
○	○		Kindai University Hospital
○	○	○	Osaka International Cancer Institute
○	○	○	National Hospital Organization Osaka National Hospital
	○	○	Osaka General Medical Center
	○	○	Osaka Medical and Pharmaceutical University
	○		Osaka Rosai Hospital
○	○	○	Kobe University Graduate School of Medicine
	○	○	Kansai Rosai Hospital
○	○		Hyogo College of Medicine
○	○	○	Hyogo Cancer Center
	○		Itami City Hospital
	○		Shimane University Faculty of Medicine
	○	○	Hiroshima University Hospital
	○		Fukuyama City Hospital
	○		Tokushima Red Cross Hospital
○	○	○	National Hospital Organization Shikoku Cancer Center
○		○	Kochi Health Sciences Center
○		○	National Kyushu Cancer Center
○		○	Kyushu University Hospital

	○	○	Oita University Faculty of Medicine

HBP: Hepatobiliary and Pancreatic Oncology Group
SCSG: Stomach Cancer Study Group:
JEOG: Japan Esophageal Oncology Group

16.10. JCOG Protocol Review Committee

This protocol was approved by JCOG Protocol Review Committee prior to submit to Certified Review Board.
(For membership, see website http://www.jcog.jp/basic/org/committee/protocol.html)
Contact: Protocol Review Committee Office

JCOG Operations Office/Clinical Research Support Office, National Cancer Center Hospital
〒104-0045 5-1-1, Tsukiji, Chuo-ku, Tokyo
TEL: 03-3542-2511 (ext. 2302)
FAX:03-3542-7006
E-mail: jcogoffice@ml.jcog.jp

16.11. JCOG Data and Safety Monitoring Committee

During study period, the study will be monitored by Data and Safety Monitoring Committee (e.g., adverse event reports, interim analysis reviews, monitoring report reviews, protocol revision reviews). However, the committee members from the study group conducting this study do not participate directly in the review of this study.
(For membership, see website http://www.jcog.jp/basic/org/committee/jury.html)
Contact: JCOG Data and Safety Monitoring Committee Office

JCOG Operations Office/Clinical Research Support Office, National Cancer Center Hospital
〒104-0045 5-1-1, Tsukiji, Chuo-ku, Tokyo
TEL:03-3542-2511 (ext. 2403)
FAX:03-3542-7006
E-mail: jcogoffice@ml.jcog.jp

16.12. JCOG Audit Committee

Site-visit audits by Audit Committee will be conducted during study period.
(For membership, see website http://www.jcog.jp/basic/org/committee/audit.html)
Contact: JCOG Auditing Committee Office

JCOG Operations Office/Clinical Research Support Office, National Cancer Center Hospital
〒104-0045 5-1-1, Tsukiji, Chuo-ku, Tokyo
TEL:03-3542-2511 (ext. 2403)
FAX:03-3542-7006
E-mail: jcogoffice@ml.jcog.jp

16.13. JCOG Conflict of Interest Committee

During study period, JCOG investigators involved in this study will be managed by the Conflict of Interest Committee.
(For membership, see website http://www.jcog.jp/basic/org/committee/coi.html)
Contact: JCOG Conflict of Interest Committee Office

JCOG Operations Office/Clinical Research Support Office, National Cancer Center Hospital
〒104-0045 5-1-1, Tsukiji, Chuo-ku, Tokyo
TEL:03-3542-2511 (ext. 2404)
FAX:03-3547-1002
E-mail: jcogoffice@ml.jcog.jp

16.14. Data Center/Operations Office

JCOG Data Center

Director of Data Center: Haruhiko Fukuda
Clinical Research Support Office, National Cancer Center Hospital

〒104-0045 5-1-1, Tsukiji, Chuo-ku, Tokyo
TEL:03-3542-3373
FAX:03-3542-3374
E-mail: jcogdata@ml.jcog.jp

JCOG Operations Office
Director of Operations Office: Kenichi Nakamura
Clinical Research Support Office, National Cancer Center Hospital
〒104-0045 5-1-1, Tsukiji, Chuo-ku, Tokyo
TEL:03-3547-1002
FAX:03-3547-1002
E-mail: jcogoffice@ml.jcog.jp

Official website: http://www.jcog.jp/

16.14.1. Data management administrator

Data management organization		JCOG Data Center
Data management administrator	Name	Harumi Kaba
	e-Rad number	40543442
	Affiliation	Clinical Research Support Office, National Cancer Center Hospital
	Title	Head of Multi-institutional Data Management Section

16.14.2. Monitoring administrator

Monitoring organization		JCOG Data Center
Monitoring administrator	Name	Haruhiko Fukuda
	e-Rad number	70263390
	Affiliation	Clinical Research Support Office, National Cancer Center Hospital
	Title	Chief of Data Management Division

16.14.3. Site-visit audit administrator

Auditing organization		JCOG Operations Office
Site-visit audit administrator	Name	Kenichi Nakamura
	e-Rad number	40543533
	Affiliation	Clinical Research Support Office, National Cancer Center Hospital
	Title	Director of Research Management Division

16.14.4. Statistical analysis administrator

Statistical analysis organization		JCOG Data Center
Statistical analysis Administrato r	Name	Junki Mizusawa
	e-Rad number	60706646
	Affiliation	Biostatistics Division, Center for Research Administration and Support, National Cancer Center/<br>Biostatistics Section, Research Management Division, Clinical Research Support Office, National Cancer Center Hospital
	Title	Biostatistics Section Head

16.14.5. Research and development plan support personnel

Organizations in charge of supporting research and development plans		JCOG Operations Office
Research and development plan support personnel	Name	Tomoko Kataoka
	e-Rad number	70569863
	Affiliation	Clinical Research Support Office, National Cancer Center Hospital
	Title	Medical officer

16.14.6. Coordination management practitioner

Organizations in charge of coordination and management		JCOG Operations Office
Coordinating and managing practitioners	Name	Junko Eba
	e-Rad number	80754085
	Affiliation	Clinical Research Support Office, National Cancer Center Hospital
	Title	Medical officer

16.14.7. Personnel who oversees the study other than Principal Investigator and site investigators

Personnel who oversees the study other than Principal Investigator and site investigators	Name	Not applicable	
	e-Rad number		
	Affiliation		
	Title		
	Relevance of Secondary Sponsor	□ Applicable	□ Not applicable

16.14.8. Study group personnel

JCOG Data Center

Statistics Section Gakuto OGAWA

Data Management Section Kyoko HASEGAWA

JCOG Operations Office

Science Section Tomoko KATAOKA/Tadayoshi HASHIMOTO

In addition, JCOG Data Center/Operations Office commissioned some of theire research support activities (such as support for the preparation of study protocols, data management, and site-visit audits) to other corporations. The commissioned duties are supervised by the National Cancer Center through routine work, as well as by receiving work reports from the institution and providing supervisory guidance. The current consignee is as follows

Clinical Oncology Research and Education, a specified non-profit organization

〒104-0061 DJ Ginza Building 7F, 8-18-3, Ginza, Chuo-ku, Tokyo

Official website http://www.core.or.jp/

16.15. Developing a study protocol

Chigusa MORIZANE, National Cancer Center Hospital

Nozomu MACHIDA, Shizuoka Cancer Center

Yoshitaka HONMA/Ken KATO, National Cancer Center Hospital

Support for protocol development

JCOG Data Center

Statistics Section (in charge of study design) Junki MIZUSAWA

Data Management Section (CRF preparation) Harumi KABA

JCOG Operations Office

Protocol development Hiroshi KATAYAMA/Kozo KATAOKA/Aya MIURA

Person in charge of IC documents Aya KIMURA/Noriko TSUJI

17. Publication of the study results and completion of the study

17.1. Paper and conference presentations

Primary publication will be published in English journals.

Paper publication including review article and conference presentation of Introduction of the study, by Principal Investigator or Study Coordinator, which does not include the analytical results of the endpoint of the study, are allowed when Group Chair and JCOG Data Center Director agree to them. Publication of the distribution of baseline factors or the safety data after the end of accrual are also allowed. No publication other than primary analysis and final analysis will be performed unless previously approved by Data and Safety Monitoring Committee.

In principle, the authors of the main published papers on the results of the study (the first publication of the results of primary endpoint) shall be the first Study Coordinator (HBPOG), followed by Study Coordinator (JEOG or SCSG), Study Coordinator (JEOG or SCSG), Principal Investigator (HBPOG), Principal Investigator (JEOG or SCSG), Principal Investigator (JEOG or SCSG), the statistical staff of Data Center (one statistician in charge at the time of the analysis for publication), Centralized Pathological diagnosis Coordinator (HBP), Centralized Pathological diagnosis Coordinator (GI). In accordance with the limitations imposed by the article's posting provisions, researchers who contributed in descending order of their number of patients registrations were selected for each institution as co-authors, and the last author was Group Chair (or Study Chair). The inclusion of staffs of JCOG Operations Office as co-authors will be determined by Group Chair depending on their contributions. Authors of articles other than the primary publication (e.g., Secondary endpoints articles, secondary analysis articles) will be determined by Study Chair with Group Chair approval.

All co-authors will review the article contents prior to submission for publication and only those who agree to the article contents. If there is no consensus on the contents, principle investigator may not include the investigator in the co-authorship with the approval of Group Chair. If there is no consensus between groups and JCOG Data Center/Operations Office, ultimately follow JCOG Chair instructions.

17.2. Primary Endpoint Report and Clinical Summary Report

The procedures are specified from the preparation of the primary endpoint report and clinical summary report. If primary analysis is the final analysis, the primary endpoint report will not be prepared and the clinical summary report will be prepared.

17.2.1. Clinical Summary Report

Based on the final analysis report, Principal Investigator will prepare a "Abstract of the Clinical Summary Report" containing subjects background information (age, sex, etc.), study design and study progress, results of analyses for each endpoint, conclusions of the entire study, interpretations and discussion of the results, etc. within 6 months from the issue date of the final analysis report, submit it to the Data Center, and undergo review. In addition, the issue date of the final analysis report shall be "the date when the period for collecting data on all endpoints is completed" as specified in the Enforcement Regulations.

After obtaining approval from Group Chair and the Director of the Data Center, abstract of the clinical summary report will be submitted to Certified Review Board review as a "clinical summary report" with the final analysis report within 1 year of the issue date of the final analysis report.

Principal Investigator/Study Coordinator will disclose abstract of the clinical summary report (Notification of Completion of Form 1 of Article 24 of Regulation) to jRCT within 1 month after obtaining Certified Review Board approval (if the report is unpublished, abstract will not be released in jRCT and will be published immediately after publication).

The approved abstract of the clinical summary report will be submitted to the administrators of each participating institution through the site investigators at each institution and to JCOG Chair. Abstract of the clinical summary report is available on JCOG website (http://www.JCOG.jp/).

The timing of distribution of the final analysis report to participating institutions will be determined by Principal Investigator/Study Coordinator considering the timing of publication of the final analysis results, and the final analysis report will be distributed to the researchers at participating institutions by themselves or through the Data Center.

17.3. Completion of the study

On the date that abstract of the clinical summary report was released to iRCT, the study is completed.

In institutions where patients were not enrolled, the institution may be withdrawn from the institution list by submitting a request for change (Unified Form 3 for Clinical Research) and a Notification of Changes in Protocol (Form 2) stating that Principal Investigator withdraws the institution from the institution list in question to Certified Review Board, and after obtaining approval, notifying the MHLW (Local Health Bureau responsible for the location of Certified Review Board). When a protocol change notification is received by the Local Health Service, the investigator at the institution shall report the withdrawal to the Administrator of the institution.

18. References

omit

19. Appendix

- Informed consent form
- Body surface area table
- Toxicity Criteria (CTCAE v5.0-JCOG)
- CRF samples* (CRF draft attached for the first review submission)
- JCOG-Biobank Japan Biorepository Protocol
- JCOG-Biobank Japan Biorepository Informed Consent Form

Japan Clinical Oncology Group

Hepatobiliary and Pancreatic Oncology Group/Stomach Cancer Study Group/Japan Esophageal Oncology Group

Practical Research for Innovative Cancer Control from Japan Agency for Medical Research and Development
“Establishment of standard treatments for neuroendocrine carcinoma of the digestive system”
National Cancer Center Research and Development Fund 2020-J-3
“Scientific research on multi-institutional trials to establish new standard treatment of solid tumors in adults”

JCOG1213

Randomized phase III study of etoposide plus cisplatin combination therapy versus irinotecan plus cisplatin combination therapy in advanced neuroendocrine carcinoma of the digestive system. ver.1.6.0

TOPIC-NEC

HBPOG

Group Chair: Junji FURUSE
Department of Medical Oncology, Kyorin University Faculty of Medicine

Study Chair: Takuji OKUSAKA
Department of Hepatobiliary and Pancreatic Oncology, National Cancer Center Hospital 5-1-1 Tsukiji, Chuo-ku, Tokyo 104-0045 Japan
Tel: +81-3-3542-2511
E -mail : tokusaka@ncc.go.jp

Study Coordinator: Chigusa MORIZANE
Department of Hepatobiliary and Pancreatic Oncology, National Cancer Center Hospital 5-1-1 Tsukiji, Chuo-ku, Tokyo 104-0045 Japan
Tel: +81-3-3542-2511
E -mail : cmorizan@ncc.go.jp

SCSG

Group Chair: Masanori TERASHIMA
Division of Gastric Surgery, Shizuoka Cancer Center Hospital

Study Chair: Narikazu BOKU
Department of Gastrointestinal Oncology, National Cancer Center Hospital
5-1-1 Tsukiji, Chuo-ku, Tokyo 104-0045 Japan
Tel: +81-3-3542-2511
E -mail : nboku@ncc.go.jp

Study Coordinator: Nozomu MACHIDA
Department of Gastroenterology, Kanagawa Cancer Center (Gastrointestinal Tract)
TEL:+45-520-2222
FAX:+45-520-2202
E-mail:n-machida@kcch.jp

JEOG

Group Chair: Yuko KITAGAWA
General and Gastroenterological Surgery, Keio University Hospital

Study Chair: Ken KATO
Department of Head and Neck, Esphageal Medical Oncology, National Cancer Center Hospital 5-1-1 Tsukiji, Chuo-ku, Tokyo 104-0045 Japan
Tel: +81-3-3542-2511
E -mail : kenkato@ncc.go.jp

Study Coordinator: Yoshitaka HONMA/Ken KATO
Department of Head and Neck, Esphageal Medical Oncology, National Cancer Center Hospital 5-1-1 Tsukiji, Chuo-ku, Tokyo 104-0045 Japan
Tel: +81-3-3542-2511
E-mail:yohonma@ncc.go.jp
/kenkato@ncc.go.jp

2013/3/16	Protocol concept approved by JCOG Executive Committee (PC1213/1214/1215)
2014/6/27	Protocol approved byJCOG Protocol Review Committee
2017/5/23	Revision ver. 1.1 approved by JCOG Data and Safety Monitring Committee
2018/8/16	Revision ver. 1.2 approved by JCOG Data and Safety Monitring Committee
2018/10/11	Approved by Certified Review Board of National Cancer Center Hospital
2019/5/27	Revision ver. 1.3.0. approved by JCOG Data and Safety Monitring Committee
2019/6/27	Approved by Certified Review Board of National Cancer Center Hospital
2020/1/24	Revision ver. 1.4.0. approved by JCOG Data and Safety Monitring Committee
2020/2/27	Approved by Certified Review Board of National Cancer Center Hospital
2020/7/17	Revision ver. 1.5.0. approved by JCOG Data and Safety Monitring Committee
2020/9/24	Approved by Certified Review Board of National Cancer Center Hospital
2021/1/21	Revision ver. 1.6.0. approved by JCOG Data and Safety Monitring Committee
2021/2/25	Approved by Certified Review Board of National Cancer Center Hospital

0. Summary

This study is conducted as a "specified clinical trial" based on the Clinical Trials Act (Act No. 16 of April 14, 2017). In this protocol, the Principal Investigator is the Study Chair in the Hepatobiliary and Pancreatic Oncology Group of JCOG.

Name of study: "Randomized phase III study of etoposide plus cisplatin combination therapy versus irinotecan plus cisplatin combination therapy in advanced neuroendocrine carcinoma of the digestive system. (TOPIC-NEC) ".

Public study title: "Randomized phase III study of etoposide plus cisplatin combination therapy versus irinotecan plus cisplatin combination therapy in advanced neuroendocrine carcinoma of the digestive system. (TOPIC-NEC) "

0.1. Schema

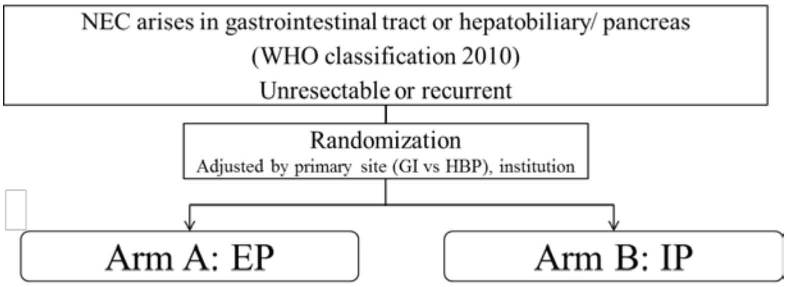

0.2. Objectives

A randomized phase III study was conducted to determine the better treatment option between etoposide/cisplatin combined therapy (EP therapy) or irinotecan/cisplatin combined therapy (IP therapy), both of which are standard treatments for non-resectable/recurrent neuroendocrine carcinoma (NEC as classified by WHO in 2010) with primary lesions in the gastrointestinal tract/hepatobiliary pancreatic organs.

Primary endpoint: Overall survival

Secondary endpoints: Response rate in case of measurable lesions, Progression-free survival (PFS), incidence rate of adverse events, dose intensity of Cisplatin, and incidence rate of serious adverse events.

0.3. Subjects

1) Any of the following is applicable based on pathological diagnosis taking findings of immunohistochemistry into consideration (see 3.1. to 3.3.).

[1] Pathologically diagnosed as neuroendocrine carcinoma (NEC*1) in the resected sample.

[2] Containing pathologically confirmed component of neuroendocrine carcinoma (NEC*1) in the biopsy sample.

1: Based on WHO 2010 classification

2) Any of the following is applicable

[1] NEC arise in esophagus, stomach, duodenum, intestine, appendix, colon, rectum, gallbladder, intrahepatic bile duct, extrahepatic bile duct, ampulla of Vater, pancreas,

[2] Liver NEC (primary liver or liver metastasis of unknown primary) *2.

*2: The tumor is only in one of the following sites after a thorough examination of the primary site by contrast CT (from the neck to pelvic) and upper/lower gastrointestinal endoscopy, FDG-PET scan, otolaryngology (head and neck) examination, urology examination (male patients only), and gynecology examination (female patients only).

a. Liver only

b. Bone, lymph nodes below the diaphragm, peritoneum, subcutaneous, muscle, spleen, and liver

3) Unresectable (see 3.6) or recurrent cancer (see 3.7). It is not essential for a pathological confirmation of the metastatic lesion or recurrent site. Cases of esophageal NEC is ineligible if corresponding to any of the following.

[1] cT4.

[2] No distant metastasis rather than supraclavicular lymph node

[3] Stenosis indicated for palliative radiotherapy

4) No previous chemotherapy or radiotherapy for NEC. Pre- or post-operative chemotherapy except irinotecan or

etoposide for NEC is allowed as long as it was completed at least 8 weeks prior to registration.

5) No previous chemotherapy using platinum agents for any malignancies.

6) No serious tumor-related complications.

Serious tumor-related complications include superior vena cava syndrome, inferior vena cava syndrome, pleural, ascites, or pericardial effusions that are large or uncontrollable (e.g., effusions that accumulate quickly after drainage or even after adhesive surgery), and brain metastases with neurological symptoms.

7) Aged 20 to 75 years old.

8) ECOG performance status of 0 or 1.

9) Sufficient oral intake

10) Measurable region is not required.

11) Adequate organ functions.

[1] WBC ≥3,000/ mm^3

[2] Neutrophils ≥ 1,500/ mm^3

[3] Hemoglobin ≥ 9.0 g/dL

[4] Platelets ≥ 10×10^4/ mm^3

[5] Total bilirubin≤ 1.5 mg/dL[※4]

[6] AST(sGOT)≦100 IU/L[※4]（for hepatic NEC and liver metastasis, ≦150 IU/L）

[7] ALT(sGPT)≦100 IU/L[※4]（for hepatic NEC and liver metastasis, ≦150 IU/L）

※4：Presence or absence of biliary drainage is not relevant

[8] Serum creatinine≦ 1.3 mg/dL

[9] Creatinine clearance[※5]≥ 60 mL/min

※5： Creatinine clearance must have been estimated using the Cockcroft-Gault formula, and must be 60 mL/min or more. If the estimation is less than 60 mL/min, but the actual measurement is 60 mL/min or more, the patient can be deemed eligible.

Cockcroft-Gault formula

Male：Ccr ={(140－age) × body weight (kg)}/{72×serum creatinine (mg/dL)}

Female：Ccr = 0.85×{(140－age) × body weight (kg)}/{72×serum creatinine(mg/dL)}

12) Written informed consent.

0.4. Treatments

Arm A: etoposide plus cisplatin (EP) arm

The following chemotherapy is continued until the patient meets discontinuation criteria, with 3-weeks of treatments counting as one cycle.

Drug	Dosage (mg/m^2)	Dosing regimen/Dosing time	Dose day
Etoposide	100	IV/60-120 min	Day 1, 2, 3
Cisplatin	80	IV/60-120 min	Day 1

Arm B: Irinotecan plus Cisplatin (IP) arm

The following chemotherapy would be continued until the patient meets discontinuation criteria, with 4-weeks of treatments counting as one cycle.

Drug	Dose (mg/m^2)	Dosing regimen/Dosing time	Dose day
Irinotecan	60	IV/90 min	Day 1, 8, 15
Cisplatin	60	IV/60-120 min	Day 1

0.5. Planned sample size and study period

The planned sample size is 170.

Accrual period: 6 years.

Follow-up period: 2 years after accrual completion.

Analysis period: 1 year.

Total study duration 9 years.

Primary analysis: 9 years after accrual completion.

Scheduled start date of the study	Aug 8, 2014
Expected completion date of the study	Aug 8, 2023

0.6. Contact information

Eligibility criteria, treatment modification, and other issues requiring clinical decisions: Study Coordinator (front cover and 16.6.)

Enrollment procedure protocol, case report form (CRF) entries, etc.: JCOG Data Center, (16.14.)

Adverse event reporting: JCOG Data and Safety Monitoring Committee (16.11.),

Contents

0. SUMMARY ... 2

0.1. SCHEMA ... 2
0.2. OBJECTIVES ... 2
0.3. SUBJECTS ... 2
0.4. TREATMENTS ... 3
0.5. PLANNED SAMPLE SIZE AND STUDY PERIOD ... 3
0.6. CONTACT INFORMATION ... 4

1. OBJECTIVES ... 8

2. BACKGROUND ... 8

2.1. TARGET ... 8
2.2. STANDARD TREATMENT FOR TARGET DISEASE ... 16
2.3. RATIONALE FOR ESTABLISHMENT OF TREATMENT PLAN ... 18
2.4. STUDY DESIGN ... 21
2.5. SUMMARY OF EXPECTED ADVANTAGES AND DISADVANTAGES ASSOCIATED WITH STUDY PARTICIPATION ... 22
2.6. SIGNIFICANCE OF THIS STUDY ... 23
2.7. ASSOCIATED RESEARCH (INCLUDING SAMPLE ANALYSIS RESEARCH) ... 23
2.8. JCOG-BIOBANK JAPAN (BBJ) COLLABORATING BIOBANK ... 23

3. CRITERIA/DEFINITIONS USED IN THIS STUDY ... 25

3.1. TISSUE CLASSIFICATION (WHO 2010 CLASSIFICATION) ... 25
3.2. GRADE CLASSIFICATION （ENETS [EUROPEAN NEUROENDOCRINE TUMOR SOCIETY] / WHO2010 CLASSIFICATION） ... 25
3.3. HISTOPATHOLOGICAL DIAGNOSIS ... 25
3.4. DISEASE STAGE CLASSIFICATION CRITERIA (UICC-TNM 7TH EDITION) ... 25
3.5. RESIDUAL TUMOR (R) CLASSIFICATION (UICC-TNM 7TH EDITION) ... 33
3.6. DEFINITION OF NON-RESECTABLE NEC ... 33
3.7. DEFINITION OF RECURRENT NEC ... 34
3.8. DEFINITION OF HEPATIC NEC (HEPATIC PRIMARY LESION OR LIVER METASTASIS FROM AN UNKNOWN PRIMARY LESION) 34

4. PATIENT SELECTION CRITERIA ... 34

4.1. INCLUSION CRITERIA (FOR ENROLLMENT) ... 34
4.2. EXCLUSION CRITERIA ... 35

5. REGISTRATION AND RANDOMIZATION ... 36

5.1. PROCEDURE OF REGISTRATION ... 36
5.2. RANDOMIZATION AND ALLOCATION ADJUSTMENT FACTOR ... 37

6. TREATMENT PLAN AND TREATMENT MODIFICATION CRITERIA ... 38

6.1. PROTOCOL TREATMENT ... 38
6.2. PROTOCOL TREATMENT TERMINATION/COMPLETION CRITERIA ... 41
6.3. TREATMENT MODIFICATION CRITERIA ... 42
6.4. CONCOMITANT TREATMENT AND SUPPORTIVE CARE ... 48
6.5. POST-STUDY TREATMENT ... 55

7. ANTICIPATED ADVERSE EVENTS ... 56

7.1. ANTICIPATED ADVERSE REACTIONS ... 56

7.2. Anticipated Adverse Events Due to Pathogenesis 59
7.3. Evaluation of Adverse Events/Reactions 60

8. EXAMINATION AND EVALUATION 62

8.1. Baseline examination and evaluation before registration 62
8.2. Examination and evaluation during treatment 63
8.3. Examination and evaluation after completion of treatment 64
8.4. Information on post- study treatment 64
8.5. Study calendar 65

9. DATA COLLECTION 69

9.1. Case Report Form (CRF) 69

10. REPORTING OF "DISEASE OR THE LIKE"(ADVERSE EVENTS) 70

10.1. Serious Adverse Events and subjects of Expedited Reporting. 70
10.2. Investigator's reporting requirements and procedures 71
10.3. Responsibilities of Principal Investigator/Study Coordinator 73
10.4. Responsibilities of the Site Investigators at the participating institutions (including the relevant institution) 75
10.5. Responsibilities of the Data and Safety Monitoring Committee 75

11. RESPONSE EVALUATION AND ENDPOINT DEFINITION 76

11.1. Response assessment (only for patients with measurable disease) 76
11.2. Definitions of analyses set 82
11.3. Definition of endpoints 82

12. STATISTICAL CONSIDERATION 85

12.1. Principal Analysis and Decision Criteria 85
12.2. Planned accrual, accrual period, and follow-up periods 86
12.3. Interim Analysis and Early Termination of the Study 87
12.4. Analysis of Secondary endpoints 88
12.5. Final analysis 88
12.6. Exploratory analysis 89
12.7. Premature withdrawal from the trial 89
12.8. Procedures after Early termination of the Study 90

13. ETHICAL CONSIDERATIONS 92

13.1. Protection of Human Subjects 92
13.2. Informed consent 92
13.3. Protection of personal Information and patient identification 94
13.4. Adherence to the protocol 96
13.5. Application to Certified Review Board and Notification of Implementation Plans 96
13.6. Protocol revision/amendment 100
13.7. Conflicts of Interest (COIs) involved in this study 102
13.8. Compensation 104
13.9. Intellectual property 105
13.10. Disclosure of information on this study 105

14. MONITORING AND AUDIT 106

14.1. Periodic monitoring 106
14.2. Site visit audits 108

14.3. MANAGEMENT OF NON-COMPLIANCE 109

15. SPECIAL INSTRUCTIONS 110

15.2. JCOG BIOBANK JAPAN (BBJ) BIOREPOSITORY 111

16. ORGANIZATION 111

16.1. MAIN STUDY FUND (FUNDING SOURCE) OF THIS STUDY. 111
16.2. JAPAN CLINICAL ONCOLOGY GROUP (JCOG) 111
16.3. JCOG CHAIR 112
16.4. STUDY GROUP AND GROUP CHAIR 112
16.5. STUDY CHAIR (PRINCIPAL INVESTIGATOR) 113
16.6. STUDY COORDINATOR 113
16.7. CENTRAL PATHOLOGICAL REVIEW COORDINATOR 114
16.8. CENTRAL PATHOLOGICAL REVIEW COORDINATOR 114
16.9. PARTICIPATING SITES (PARTICIPATING INSTITUTIONS) 115
16.10. JCOG PROTOCOL REVIEW COMMITTEE 116
16.11. JCOG DATA AND SAFETY MONITORING COMMITTEE 116
16.12. JCOG AUDIT COMMITTEE 116
16.13. JCOG CONFLICT OF INTEREST COMMITTEE 116
16.14. DATA CENTER/OPERATIONS OFFICE 116
16.15. DEVELOPING A STUDY PROTOCOL 118

17. PUBLICATION OF THE STUDY RESULTS AND COMPLETION OF THE STUDY 119

17.1. PAPER AND CONFERENCE PRESENTATIONS 119
17.2. PRIMARY ENDPOINT REPORT AND CLINICAL SUMMARY REPORT 119
17.3. COMPLETION OF THE STUDY 120

18. REFERENCES 121

19. APPENDIX 121

1. Objectives

A randomized phase III study was conducted to determine the better treatment option between etoposide/cisplatin combined therapy (EP therapy) or irinotecan/cisplatin combined therapy (IP therapy), both of which are standard treatments for non-resectable/recurrent neuroendocrine carcinoma (NEC as classified by WHO in 2010) with primary lesions in the gastrointestinal tract/hepatobiliary pancreatic organs.

Primary endpoint： Overall survival

Secondary endpoints： Response rate in case of measurable lesions
Progression-free survival (PFS), incidence rate of adverse events, dose intensity of Cisplatin, and incidence rate of serious adverse events

2. Background

2.1. Target

2.1.1. Epidemiology

Neuroendocrine tumors (NETs) and neuroendocrine carcinomas (NECs) can arise in various organs in the body. Of these, NECs with primary lesions occurring in the lungs, namely small cell lung cancer and large cell lung cancer, have been collectively referred to as high-grade NECs (HGNECs). However, terms such as extrapulmonary small cell carcinoma and extrapulmonary NEC have been used to refer collectively to NECs, with primary lesions outside the lungs.

The number of newly reported cases of neuroendocrine neoplasms (NENs) annually, estimated based on the cases registered in the U.S. SEER（Surveillance, Epidemiology, and End Results）database between 1973 to 2004 is 5 in 100,000 people[1]. In Japan, the Neuroendocrine Tumor Workshop Japan （NET Work Japan）carried out a nationwide survey of pancreatic and gastrointestinal NENs, and estimated the number of new cases in 2005 at approximately 1.01 people with pancreatic primary lesion per 100,000 people, and 2.10 people with gastrointestinal primary lesion per 100,000 people[2, 3].

The report by the PRONET Study Group in France concerning their prospective observational study is a good reference for the proportion of NENs that can be classified as NECs. Out of the 778 patients diagnosed with gastrointestinal primary lesion NENs in 80 facilities from August 2010 to July 2011, 104 patients (13.4%) had NEC[4].

The frequency of incidence of NECs according to the primary lesion organ has been shown in the tables below (Table 2.1.1a and Table 2.1.1b). Despite variations between reports, gastrointestinal primary lesion NECs account for approximately 20–68% of all extrapulmonary NEC cases. The breakdown of gastrointestinal primary lesion NEC cases with primary lesion in the gastrointestinal tract (esophagus, duodenum, small and large intestines) indicated that these cases accounted for 56–84% of the total cases, while hepatobiliary pancreatic cancer cases accounted for 15–35% of all cases.

Table 2.1.1a. Frequency of extrapulmonary NECs according to the organ of primary lesion

Reported year/Reporter	N	Gastrointestinal	Gynecological organs	Urology	Head and Neck	Adrenal	Primary unknown	Other
2012/Terashima [5]	136	68%	12%	6%	NA	NA	8%	7%
2010/Brennan [6]	74	20%	35%	15%	19%	NA	9%	1%
2009/Wong [7]	1618	33%	NA	20%	11%	10%	4%	22%
2006/Haider [8]	101	20%	11%	18%	12%	9%	31%	NA
2007/Lee [9]	61	56%	20%	10%	8%	NA	6%	NA

Table 2.1.1b. Percentage breakdown of NECs as per the primary lesion organ of gastrointestinal NEC

Reported year/Reporter	N	Esophageal	Gastric	Duodenal/small intestine	Colorectal	Hepatobiliary	Pancreas	Other
2012/Machida [10]	258	33%	27%	2%	12%	12%	14%	NA
2012/Sorbye [11)]	205	6%	10%	NA	40%	NA	35%	10%
2004/Brenner [12]	544	53%	11%	0.2%	20%	11%	4%	NA

2.1.2. Clinical pathology

1) About the term of target disease

The target of this study has a complex disease concept, and since the method of classification has changed with time, various names have been assigned that could cause confusion. In this study, we use classification and nomenclature defined according to the WHO 2010 classification（WHO Classification of Tumors of the Digestive System 2010）[13].

All the tumors originating from neuroendocrine cells or differentiation into endocrine cells are called neuroendocrine neoplasms (NENs). Depending on the malignancy, NENs were classified as Grade 1 NETs (NET G1), Grade 2 NETs (NET G2), or NEC.

Furthermore, prior to the WHO 2010 classification, all disease concepts equivalent to NENs were called NETs, but in this study, we would have referred to all descriptions of the disease concept as NENs. Furthermore, according to the 2000 and 2004 WHO classifications, the term well-differentiated NEC (WDNEC) has been used for disease units equivalent to NET G2, but in this study NEC does not mean the same.

2) Classification and clinical pathology

NEN is a tumor that develops from neuroendocrine cells present in various tissues or shows a tendency of differentiation into neuroendocrine cells. Histologically these tumors express neuroendocrine markers, such as Chromogranin A, NSE (neuron-specific enolase), and synaptophysin. The WHO classifications in 2000 and 2004 combined the presence or absence of metastasis/local infiltration and cellular proliferative capacity (evaluated based on Ki-67 expression intensity and mitotic presentation), and thereby classified these tumors into well-differentiated (neuro)endocrine tumors without metastasis/local infiltration, well-differentiated (neuro)endocrine tumors with metastasis/local infiltration, and poorly differentiated (neuro)endocrine tumors with even greater cell proliferative capacity. On the other hand, European Neuroendocrine Tumor Society (ENETS) has proposed a method of classification that evaluates the malignancy as Grade 1, 2, or 3 (G1, G2, or G3), according to the cell proliferative capacity (Ki-67 index or number of mitotic presentations), which has proven to be most useful for classification of prognosis[14, 15]。

Against this backdrop, the WHO classification that targeted gastrointestinal diseases was published in 2010. The disease as a whole was considered “neuroendocrine neoplasms (NEN)”, and the disease malignancy was largely classified as NET G1 (neuroendocrine tumor Grade 1), NET G2 (Grade 2), NEC, and mixed adenoneuroendocrine carcinoma (MANEC). MANEC refers to the cancers wherein adenocarcinoma components account for more than 30% of the cancer, while those under 30% are classified as NECs. However, even though this method of classification is applicable while evaluating the entire tumor based on resected specimen, it is not possible to evaluate the proportion of each component in the entire tumor during a diagnosis based on biopsy sample.

Each of NET G1, NET G2, and NECs described in the WHO 2010 classification is equivalent to G1, G2, and G3 as per the ENETS classification. NETs are well-differentiated tumors, with relatively low atypicality and malignancy, and the clinical course is slow with a 5-year survival in the range of 62–85%[16, 17]. On the other hand, NECs are poorly differentiated tumors, where tumor cells with poor cellular constituents proliferate diffusely, and are pathologically similar to small cell lung cancers, since these show many mitotic presentations, involve necrotic lesions, and present with neuroendocrinological features during immunohistochemical staining[1]. The clinical presentations are also similar in terms of the rapid proliferation of NECs and relatively high sensitivity to anticancer drugs[18].

Similar to lung cancer, NECs also exist as small cell cancer type or large cell type (LCNEC：large cell NEC）[13]. Future challenges include studies on the frequency and differences in clinical presentation of each disease. Table 2.1.2 shows the WHO and Grade classification.

Table 2.1.2. Changes in WHO classification and grading; Shaded parts are targets of this study

WHO 2000 classification	WHO 2010 classification		ENETS Grade
Well-differentiated endocrine tumor（WDNET）<br>1.1‘Benign’ behavior<br>1.2 Uncertain behavior	Neuroendocrine tumors, NETs<br>• Well-differentiated<br>• Composed of cells similar to normal gastrointestinal	NET G1（carcinoid）	G1<br>Same as 2010 WHO classification<br>Number of mitotic presentations < 2 per 10

	endocrine cells<br>• Expression of neuroendocrine markers<br>• Hormone production<br>• Mild to moderate nuclear atypia, low proliferative capacity (Grade：G1,G2)		high power fields (HPF) and/or Ki-67 index ≤2%
Well-differentiated endocrine carcinoma (WDEC)		NET G2	G2<br>Number of mitotic presentations 2–20 per 10 HPF and/or Ki-67 index 3-20%
Poorly differentiated endocrine carcinoma/small cell carcinoma (PDEC)	Neuroendocrine carcinoma：NEC (large cell or small cell type)：<br>• Poorly differentiated, highly malignant<br>• Include small cell to large cell type carcinoma<br>• Sometimes present tissue structure similar to NET<br>• Expression of neuroendocrine markers<br>• Significant nuclear atypia, multifocal necrosis, and high proliferative capacity (Grade: G3)		G3<br>Number of mitotic presentations >20 per 10 HPF and/or Ki-67 index > 20%
Mixed exocrine-endocrine carcinoma (MEEC)	Mixed adenoendocrine carcinoma (MANEC)		
Tumor-like lesions (TLL)	Hyperplastic and preneoplastic lesions		

2.1.3. Staging

Disease staging for NENs is represented by the TNM classification proposed by ENETS (hereafter, ENETS TNM[14, 15], AJCC 7th Edition TNM classification and UICC 7th Edition TNM classification published in 2009. The AJCC 7th Edition and UICC 7th Edition TNM classifications are unified, and in this study we would have used the UICC 7th Edition nomenclature).

In the UICC 7th edition, there are independent TNM classifications of NET G1 and NET G2 with primary lesions in the stomach, small intestine, appendix, and colon, but with respect to NEC, it describes "classification according to main tissue type in each organ of primary lesion (squamous cell carcinoma for esophagus, and adenocarcinoma in all other organs of primary lesion)." While there are TNM classifications for hepatocellular carcinoma and intrahepatic cholangiocarcinoma in the liver, there is no description for the classification of the tissue type for NECs with primary lesion in the liver. In this study, however, we would have used the TNM classification of intrahepatic cholangiocarcinoma, which shows similar clinical presentations. Details of each TNM classification would have been mentioned in “3.2 staging criteria”.

2.1.4. Standard treatment according to disease staging and outline of prognosis

1) Standard treatment for resectable cases

Surgical resection is performed for resectable cases. The significance of postoperative adjuvant chemotherapy is not clear because a verification study has not yet been performed. Although the NCCN Guidelines[19] describe postoperative adjuvant therapy as the chemotherapy regimen for small cell lung cancers that is typically applied to advanced cases of NEC, it has relatively high toxicity and patient burden (see Table 2.2.2, 2.3.2); hence we cannot claim that it is widely used in routine practice. For NECs with gastrointestinal primary lesion with mixed adenocarcinoma, a chemotherapy regimen is suggested typically after surgery for adenocarcinoma; however, in practice, there is no consensus on postoperative adjuvant chemotherapy.

2) Standard treatment for locally advanced cases

While the NCCN Guidelines propose chemoradiotherapy for small cell lung cancer for locally advanced cases, the ENETS Guidelines provide no clear indication[20, 21] for the same. As there is no sufficient information related to radiation dose, efficacy, and safety for NECs with a multitude of primary lesion organs (scope of irradiation), radiation therapy is not widely used for pathologies other than NEC with esophageal primary lesion that is prone to stenosis, and treatment is often provided for distant metastasis.

For locally advanced cases of NEC with esophageal primary lesion, chemoradiotherapy is actively performed. Furthermore, supraclavicular lymph node metastasis in thoracic esophageal tumor is classified as Stage IV, and chemoradiotherapy is applied if metastatic lymph nodes are included in the field of irradiation.

3) Standard treatment for distal metastasis cases

Systemic chemotherapy is indicated for distal metastasis and recurrent cases regardless of the organ, and a regimen is chosen for the small cell lung cancer. With respect to NECs with esophageal primary lesion, even if there is organ metastasis, palliative chemoradiotherapy is prioritized for patients incapable of oral intake of drugs due to esophageal constriction.

The efficacy of everolimus and sunitinib has been demonstrated for NETs with pancreatic primary lesions. Furthermore, Octreotide and Lantreotide effectively inhibit proliferation of NETs that originate in the midgut and pancreatic/gastrointestinal NETs, respectively. However, these results are from clinical trials conducted in patient populations corresponding to NETs. Since the clinical presentations of NETs and NECs differ significantly, these treatments cannot be indicated for NECs during routine practice.

4) Indication of resection for distal metastasis

There is no consensus on the resection criteria specific to the NECs. Since the progression of NETs is slow, resection is actively considered even where there is distal metastasis to the liver, but as NEC progresses fast, surgical resection cannot be indicated for cases with distal metastasis[19, 21].

5) Prognosis

In a preceding study, Yamaguchi and Machida carried out a multicenter joint observational study of poorly

differentiated NEC (WHO 2010 NEC, including patients with clinical diagnosis of poor differentiation) in a total of 23 facilities, including facilities participating in JCOG Hepatobiliary and Pancreatic Oncology Group, former Gastroenterology Group, and observer facilities.

In the multicenter joint study by Yamaguchi and Machida, 89% of cases (229/258 patients) receiving systemic chemotherapy had distal metastasis, with a median survival time (MST) of 11.5 months for all patients, 11.2 months for distal metastasis cases, and 15.9 months for locally advanced cases. MST according to organ was 13.4 months for esophageal primary lesion (N = 85), 13.3 months for gastric primary lesion (N = 70), 29.7 months for small intestine/duodenal primary lesion (N = 6), 7.6 months for colonic primary lesion (N = 31), 8.5 months for pancreatic primary lesion (N = 35), and 7.9 months for hepatobiliary primary lesions (N = 31) (Table 2.1.4). While there were 9 cases of five-year survival among all the gastrointestinal cases, there were no five-year survival cases amongst those with primary lesions in hepatobiliary and pancreatic organs[10]. In a multicenter joint observational study for NECs conducted in four Northern European countries (hereafter referred to as NORDIC NEC Study), the MST for NECs with gastrointestinal primary lesion (albeit including ~30% of NECs with primary lesion sites unknown) was 11 months[11]. According to the U.S. SEER database, the MST of NEC was 10 months[3]. Based on the above information, despite differences in the primary lesion organ, the prognosis of the target population of this study is thought to have an overall MST of 10–11 months.

Table 2.1.4. Treatment results according to primary lesion organ for non-resectable/recurrent NECs that underwent systemic chemotherapy

	Esophageal	Gastric	Duodenal/small intestine	Colonic	**Gastrointestinal overall**	Pancreatic	Hepatobiliary	**Hepatobiliary and pancreatic organs overall**
N	85	70	6	31	**192**	35	31	**66**
Response rate（%）	58%	43%	50%	29%	**47%**	17%	16%	**17%**
Median PFS（month）	5.8	4.9	7.3	3.7	**5.1**	3.2	4.1	**3.7**
MST（month）	13.4	13.3	29.7	7.6	**13**	7.9	8.5	**7.9**

2.1.5. Tumor-related complications

We, here, report the complications that require attention during patient management, according to the organ of primary lesion. It is a rare disease and frequencies remain unknown.

1）Esophageal primary

Tumor bleeding/pain/esophageal stenosis/fistula formation due to primary lesion in the esophagus; hoarseness/difficulty swallowing/aspiration pneumonia caused by recurrent laryngeal nerve palsy due to lesion in lymph node metastasis; jaundice and liver failure associated with liver metastasis; respiratory failure and hemoptysis associated with lung metastasis, airway constriction, and pain due to lymph node metastasis; hypercalcemia, tracheal stenosis, tracheal obstruction, and suture failure due to disease progression.

2）Gastric primary

Chronic bleeding from primary lesion, accompanied by anemia, stomach pain, nausea, vomiting, gastric perforation, bloating, suture failure, fistula formation, cardiac/pyloric stenosis, ascites retention due to peritoneal metastasis, ileus, hydronephrosis due to ureteral stenosis, obstructive jaundice/liver failure due to bile duct stenosis, and obstructive jaundice/liver failure due to hepatic portal lymph node metastasis.

3）Small intestine/colonic primary

Tumor hemorrhage, ileus, fistula formation in the small intestine/bladder/vagina, intestinal obstruction, intestinal stenosis, intestinal perforation, and pelvic infection

4）Hepatobiliary and pancreatic primary

Cancer pain, rupture, obstructive jaundice associated with the growth of primary tumor, weight loss, fever, pancreatitis, cholangitis, liver abscess, cholecystitis, biliary hemorrhage, duodenal hemorrhage, duodenal stenosis, anastomotic ulcer/stenosis/leakage, symptoms associated with stenosis/obstruction of portal vein (liver

dysfunction/liver failure, esophageal varices and their rupture, gastritis, ascites retention, and hepatic encephalopathy/coma associated with portal hypertension).

5) Paraneoplastic syndrome

In addition to NECs, there are reports of paraneoplastic syndrome that is often associated with small cell lung carcinoma[22-24]. We report examples of paraneoplastic syndrome seen with small cell lung carcinoma below.

Hyponatremia due to inappropriate sodium secretion, psychological symptoms (changes in personality) due to ectopic ACTH syndrome, hypertension, hypokalemia, high blood sugar, Lambert-Eaton myasthenic syndrome, paraneoplastic cerebellar degeneration associated with autoantibody production (ataxia in the limbs, dysarthria, and nystagmus), paraneoplastic encephalomyelitis/sensory neuron disease (dementia, cranial nerve symptoms, dizziness, ataxia, autonomic imbalance, transverse paralysis, and sensory disorders).

Apart from these, there are thromboembolic events, aspiration (due to gastrointestinal stenosis), anemia, tumor pain, acute renal failure, myositis, and pulmonary fibrosis.

6) Complications due to metastatic lesions

Liver failure, pain, hepatobiliary infection, hemorrhage, bile duct stenosis, bile duct obstruction, respiratory failure, tracheal hemorrhage, respiratory tract infection, atelectasis, airway constriction, ascites, bloating, ileus, ureteral stenosis, ureteral obstruction, urinary retention, urinary tract infection, hiccups, pleural effusion, pleural hemorrhage, chest pain, pathological fracture, pain, hypercalcemia, meningeal carcinomatosis, ataxia, ischemia cerebrovascular, intracranial hemorrhage, nausea, vomiting, dizziness, consciousness disorder, cognitive disturbance, dysphasia, seizure, spasticity, edema limbs, intestinal obstruction/stenosis/perforation, pancreatitis, disseminated intravascular coagulation, thrombocytopenia, anorectal infection, anal hemorrhage, hoarseness, and superior vena cava syndrome

7) Others

General pain management including narcotic analgesics is performed for cancer pain.

Gastrointestinal stenting and bypass surgery are performed for esophageal stenosis, cardiac/pyloric stenosis, and duodenal stenosis.

Percutaneous transhepatic cholangial drainage (PTCD), percutaneous or endoscopic stenting, and bile duct jejunostomy is performed for obstructive jaundice. In addition, complications associated with biliary drainage and gastrointestinal stenting are listed below.

- PTCD, including internal and external fistula tube placement:
  Cholangitis, pancreatitis, cholecystitis, liver abscess, sepsis, biliary hemorrhage, PTCD tube obstruction/deviation, peritonitis, and pneumothorax/pleurisy
- Biliary stenting：
  Cholangitis, pancreatitis, cholecystitis, liver abscess, sepsis, biliary hemorrhage, peritonitis, pneumothorax/pleurisy (for percutaneous stenting), duodenal perforation, pneumonia (for endoscopic stenting), stent obstruction/deviation, duodenal ulcer, and duodenal hemorrhage
- Bile duct jejunostomy：
  Cholangitis, pancreatitis, cholecystitis, liver abscess, and sepsis
- Gastrointestinal stenting：
  Hemorrhage, perforation, pain, stent deviation, stent obstruction, foreign-body sensation, intestinal strangulation, ulcer formation, fever, sepsis, infection, diarrhea, constipation, tenesmus or uncontrollable urination/incontinence symptoms (colon), thyroid injury (esophagus), carotid artery injury (esophagus), and mediastinal abscess (esophagus).

2.1.6. Recurrent/progression

NEC is a very rare disease, and most reports of cases of surgery coincide with the case reports. According to the review by Arai et al., out of the 55 patients with NEC with gastric primary lesion who underwent surgical resection, only three patients survived for two years or more[25]. According to the report by Fischer et al., the MST of 13 patients with NEC with pancreatic primary lesion who underwent surgical resection was 11.7 months (seven of the patients showed distal metastasis)[26]. The form of recurrence among surgery cases remains unknown. The multicenter joint

study by Yamaguchi and Machida reported PFS among cases that underwent systemic chemotherapy with respect to the median as 5.8 months for esophageal primary lesions, 4.8 months for gastric primary lesions, 7.3 months for duodenal small intestine primary lesions, 3.7 months for colonic primary lesions, 3.2 months for pancreatic primary lesions, and 4.1 months for hepatic/biliary primary lesions[10]. In the NORDIC NEC study, the median PFS was 3 months for esophageal primary lesions, 5 months for gastric primary lesions, 3 months for colonic primary lesions, 4 months for rectal primary lesions, 5 months for pancreatic primary lesions, and 4 months for cases with unknown primary lesion site [11].

2.1.7. Prognostic/predictive factors

When prognostic factors were examined by multivariate analysis in the multicenter joint study by Yamaguchi and Machida, out of the various factors such as sex, age (younger or older than 60 years), PS (0 or 1 vs. 2 or more), primary lesion organ (gastrointestinal primary lesions vs. hepatobiliary or pancreatic primary lesions), LDH levels (below vs. above the upper limit of facility standard), presence or absence of liver metastasis, presence or absence of history of radical resection and treatment regimen (IP therapy vs. EP therapy); the independent prognostic factors identified were gastrointestinal primary lesion (vs. hepatobiliary or pancreatic primary lesion, hazard ratio (HR): 0.58), and LDH levels being below the upper limit of facility standard for LDH (vs. above the upper limit of facility standard for LDH, HR: 0.65).

Although IP therapy exhibited slightly better impact on overall survival as compared to EP therapy with an HR of 0.8, the *p*-value of 0.389 meant that there was no significant difference between the two therapies[10]. The NORIDIC NEC study reported poor PS, colonic primary lesions, high platelet count, and high LDH levels to be the main factors behind poor prognosis[11]. However, these prognostic/predictive factors were not obtained with a global consensus.

2.1.8. Rationale for selection of the target population

The objective of this study is to develop a primary chemotherapy regimen for non-resectable/recurrent NECs, and out of the non-resectable or postoperatively recurrent NECs with gastrointestinal primary lesions or hepatobiliary or pancreatic primary lesions (shaded parts in Table 2.1.2 of WHO 2010 classification of NEC), we established chemotherapy-naïve patients to be the target population.

In terms of whether or not to consider MANEC a target of this study, a discussion is needed from the viewpoint of standard treatment. MANEC is considered when 30% or more of the carcinoma consists of adenocarcinoma components, and it is treated as either adenocarcinoma or NEC by discretion of the attending physician, with no real consensus on standard treatment. MANEC was however, excluded from this study, as its disease concept is different from NEC in terms of the standard treatment, which has a consensus regarding the treatment by a “regimen according to small cell lung carcinoma”. In fact, a questionnaire was provided to the three groups participating in this JCOG study (response obtained from 52 facilities). The results showed that 79% of facilities considered the WHO 2010 classification of NEC as the appropriate tissue type for this study, while only 13% responded that consideration of NEC+MANEC would be appropriate. Therefore, majority of facilities considered “WHO 2010 classification of NEC as the appropriate target of this study”.

While diagnosis of NECs is generally carried out using tissue samples and biopsy samples, as mentioned in 2.1.3, there is no consensus methodology to strictly distinguish NEC and MANEC using biopsy samples. As a result of group discussions, a consensus was reached between the three groups that cases presenting with NEC components by biopsy sample-based diagnosis can be enrolled. Although the use of this method would mean that a certain percentage of patients with MANEC, who were not intended to participate in this study, would be enrolled however, currently there are no appropriate means to avoid this. Therefore, we decided to create a consensus for the future based on information obtained from this study.

1) **<u>Reason for targeting gastrointestinal/hepatobiliary or pancreatic primary lesion NEC</u>**

Various guidelines recommend treatment regimens suited for small cell lung carcinoma, regardless of the organ of primary lesion, and is the rationale showing the validity of the treatment development for NEC across many organs. Furthermore, taking into consideration the frequency of the disease, it is unrealistic to develop treatments according to each organ, and in practice the Minnie-Pearl Cancer Research Network Study (mentioned later in 2.2.2), which is a relatively large-scale clinical study of NEC, development of treatment was carried out in a cross-organ

manner[27].

On the other hand, if we take hypothesize that we do not sufficiently understand how the clinical presentations of the disease vary according to the organ, the fact that this is the first randomized study in Japan, and that the maintenance of foundation for clinical studies of this disease in Japan is inadequate at present time, the hurdle is too high to plan a study across all organs including the fields of gynecology and urology. Based on the above consideration, we have decided to target all gastrointestinal organs which have a relatively large number of common points between each other in terms of clinical presentation and types of complication.

According to the multicenter observatory study by Yamaguchi and Machida, the prognosis of NEC with hepatobiliary or pancreatic primary lesion was significantly poorer than that of NEC with gastrointestinal primary lesion (MST: 7.9 vs. 13.0 months, respectively), but we determined that it is possible to accurately evaluate the efficacy of the treatment regimen by randomizing the subjects using the primary lesion site (gastrointestinal vs. hepatobiliary or pancreatic) as an allocation adjustment factor. Therefore, we considered patients with both, gastrointestinal/hepatobiliary or pancreatic primary lesions as one target population of this study, and to examine differences between organs exploratively by subgroup analysis.

2) Reason for including hepatic NEC cases (hepatic primary lesion or liver metastasis with primary lesion site unknown)

Even among NECs, cases with hepatic primary lesions are particularly rare. There are no specific reports on the frequency of incidence and differences from the other organs and reports are limited only to case reports and their reviews. Moreover, since the liver is a major organ for metastasis of primary lesions into other organs, even when tumors have been clinically identified in the liver, in many cases the primary lesion tends to be in another organ. Therefore, liver should not be deemed the primary lesion site without sufficient examination for a primary lesion. On the other hand, although very rare, there have been reports of NEC with hepatic primary lesion[28]. Even if sufficient search for primary lesion leads to no indication of the same outside of the liver, currently it is not possible to distinguish whether it is a case of “NEC with hepatic primary lesion” or “lesion in the liver is a metastatic lesion, with the primary lesion unknown”. In this study, such situations wherein “liver alone has identifiable lesions” would be henceforth, referred to as “hepatic NEC (hepatic primary lesion or liver metastasis from an unknown primary lesion site) for convenience. As the NCCN Guidelines[19] and ENETS Guidelines[20, 21] indicate the usefulness of FDG-PET for detailed examination of the primary lesion, we would also examine the primary lesion using FDG-PET. Furthermore, by referring to the diagnostic procedures for cancers with unknown primary lesion site, detailed examinations into the primary lesion would be carried out by otolaryngological (head and neck) examination and urological examination for men only, and gynecological examination for women only).

Such hepatic NEC (hepatic primary lesion or liver metastasis from an unknown primary lesion site) is anticipated even from an anatomical viewpoint, since the tumor-related complications are the same as that for other gastrointestinal primary lesion NECs, and there is no issue with treating them the same as for gastrointestinal primary lesions.

3) Reason for orienting the study for non-resectable or recurrent cases

As mentioned in 2.1.4 “Standard treatment according to disease staging and outline of prognosis”, systemic chemotherapy is indicated for cases with distal metastasis or recurrent cases. Locally advanced cases are particularly prone to pancreatic or bile duct primary lesions situated in the vicinity of vital vessels, and while the NCCN Guidelines propose chemoradiotherapy according to treatment for small cell lung carcinoma[19], the ENETS Guidelines provide no clear indication[21]. In reality, for the treatment of NEC which has a variety of primary lesion organs (scope of irradiation) there is not enough information available for the chemotherapy regimen that should be combined in terms of its optimum dose, the radiation dose, efficacy, and safety. For this reason, chemoradiotherapy cannot be considered the standard treatment with consensus, and systemic chemotherapy for distal metastasis is used widely except for pathologies involving frequent stenotic symptoms when the primary lesion is in the esophagus. For this reason, this study would enroll non-resectable or recurrent cases that include locally advanced cases.

The section “3.6 Definition of non-resectable NEC” shows the definition of non-resectable cases summarized by referring to the JCOG protocol for clinical studies of systemic chemotherapy (esophagus: JCOG0807, stomach: JCOG1013 or JCOG1002, bile duct: JCOG0805, pancreas: JCOG1106). Furthermore, while radical resection has

been considered for cases of colonic primary lesion adenocarcinoma with liver or lung metastasis, as cases of NEC with distal metastasis are not indicated for resection, we defined non-resectable cases as those in "Stage IV", and followed this definition for duodenal primary lesions, small intestine primary lesions, and appendix primary lesions. Furthermore, with respect to NEC with esophageal primary lesion, chemoradiotherapy is well-indicated for cases with supraclavicular lymph node metastasis of thoracic esophageal tumor even in Stage IV, and hence were not included in this study.

4) Treatment of draft of new classification of NEC

In recent years, proposals have been made to further differentiate the WHO 2010 classification of NECs (G3 in ENETS) to "Grade 3 proliferative tumors showing the same morphological presentations as NET" and "Grade 3 tumors with strong morphological atypicality (previously classified as poorly differentiated NECs)"[29, 30], or by Ki-67 index of 20–50% and 50% or more[11, 19]. This is yet to be confirmed; however, in this study we would have used the WHO 2010 classification. However, we would have used samples collected for central pathological diagnosis to carry out studies related to classification which exploratively adds grade classification and morphological differentiation levels.

2.2. Standard treatment for target disease

Currently, there is no standard drug treatment for non-resectable/recurrent NECs with efficacy verified by a randomized controlled trial. However, given the similarity between pathological and clinical presentations, treatments according to small cell lung carcinoma have been attempted, and there have been reports of positive treatment outcome in small-scale clinical studies and observational studies.

2.2.1. Standard treatment for small cell lung carcinoma

Cisplatin-based multidrug combined therapy is the standard first-line treatment of extensive disease (ED) small cell lung carcinoma. While cyclophosphamide/doxorubicin/vincristine (CAV therapy) was established as a standard treatment in the 1970s in Europe and U.S., the etoposide/cisplatin combined therapy (EP therapy) was introduced in the late 1980s. A comparative study of EP therapy and CAV therapy did not show superiority of EP therapy over CAV therapy in terms of survival, but the response rate (CAV: 51% vs. EP: 61%) and MST (CAV: 8.3 months vs. EP: 8.6 months) were almost the same, and the lighter toxicity level (mucositis, interstitial pneumonia, hemotoxicity) meant that EP could be used as a standard treatment[31]. Thereafter, the JCOG Lung Cancer Group carried out the "comparison between EP therapy and Irinotecan/Cisplatin combined therapy (IP therapy) for Extensive-stage Small Cell Lung Cancer (JCOG9511)", and reported that the IP therapy provided a significantly better overall survival, with MST being 9.4 months vs. 12.8 months (p = 0.002) for IP therapy[32]. However, the two additional large-scale studies conducted primarily in U.S. did not show superiority of IP therapy over EP therapy, and for this reason IP therapy has not been not used as the standard treatment overseas[33, 34]. Furthermore, while the results of the "Randomized controlled trial to verify the non-inferiority of amrubicin/cisplatin combined therapy (AP therapy) over IP therapy (JCOG0509)" were reported in the 2012 conference of the American Society of Clinical Oncology (ASCO), but AP therapy was still considered significantly inferior to IP therapy[35]. Presently, the JCOG Lung Cancer Group considers IP therapy to be the standard treatment for extensive-disease small cell lung carcinoma.

2.2.2. Standard treatment for extrapulmonary NEC

While there are scattered reports relating to use of CAV therapy, EP therapy, IP therapy, and other multidrug combined therapies for extrapulmonary NEC, these reports are primarily from observational studies. Since these reports precede the unification of concept and classification methods of this disease, the nomenclature such as NEC (anaplastic type), NET (poorly differentiated type) and extrapulmonary small cell lung carcinoma have still been used. These reports, however, are presumed to be targeting almost the same disease group as NEC. There are no reports of prospective studies using EP therapy for extrapulmonary NEC. Observational studies have reported response rates between 42–67%, and MST between 15–19 months (Table 2.2.2a). On the other hand, IP therapy has reported results from observational studies and clinical studies, with response rates between 7–83% and MST of 10.1–22.6 months (Table 2.2.2b). As mentioned above, reports related to extrapulmonary NEC are limited to use of EP therapy and IP therapy in observational studies and small-scale clinical studies. The largest number of case enrollments in a clinical study of extrapulmonary NEC was seen in the Minnie-Pearl Cancer Research Network Study, which evaluated the efficacy of triple-drug combined therapy using carboplatin/etoposide/paclitaxel. The

response rate to this triple-drug combined therapy was 53%, while the MST was 14.5 months and adequate, there was intense toxicity involved, and the results were not significantly different from EP therapy, so this regimen was not considered the standard treatment[27].

The 2014 NCCN Guidelines recommend treating extrapulmonary NEC using a regimen according to small cell lung carcinoma, and the guidelines for treatment of small cell lung carcinoma mention both EP therapy and IP therapy as recommended regimens.

Therefore, the standard treatment for extrapulmonary NEC is taken as EP therapy or IP therapy.

Table 2.2.2.a. EP therapy for NEC (observational studies)

Reported year/Reporter	Target	N	Response rate	MST (in months)
1991/ Moertel[18]	Pancreatic/gastrointestinal NEC （anaplastic type）	18	67%	19
1999/ Mitry [36]	Pancreatic/gastrointestinal NEC	41	42%	15
2001/ Marie-Louise [37]	Pancreatic NET（well: 11, poorly: 4） Gastrointestinal cartinoid: 21	33	18%	19
1994/ Lo Re G [38]	Extrapulmonary SCLC	13	69%	NE
2010/ Iwasa [39]	Hepatobiliary or pancreatic NEC	21	14%	7.3
2012/ Yamaguchi [10]	Gastrointestinal NEC	12	75%	14
2012/ Yamaguchi [10]	Hepatobiliary or pancreatic NEC	34	12%	6.9
2012/ Sorbye [11]	Gastrointestinal primary lesion NEC (including primary lesion unknown)	129	31%	12

Table 2.2.2.b. IP therapy for NEC (shaded are clinical studies, others are observational studies)

Reported year/Reporter	Target	N	Response rate	MST (in months)
2003/ Hou [40]	NEC（gastrointestinal-80%）	18	43%	NE
2005/ Chin [41]	Esophageal NEC	12	83%	14
2011/ Okita [42]	Gastric NEC	12	75%	22.6
2012/ Yamaguchi [10]	Gastrointestinal NEC	142	51%	13.4
2012/ Yamaguchi [10]	Hepatobiliary or pancreatic NEC	18	39%	10.1
2006/ Kulke [43]	Pancreatic/gastrointestinal (including NET G1/G2)	15	7%	11.4
2008/ Mani [44]	Pancreatic/gastrointestinal NEC	20	58%	NE
2008/ Jin [45]	Extrapulmonary NEC	15	67%	11.4.

Table 2.2.2.c. Phase II study of carboplatin/etoposide/paclitaxel triple-drug combined therapy for NEC

Reported year/Reporter	Target (breakdown)	N	Response rate	MST (in months)
2006/ Hainsworth [27]	NEC （Colon：9, lung: 7, skin: 4, pancreas：3, gall bladder：1, thyroid:1, stomach: 1, esophagus: 1, endometrium:1, maxillary sinus: 1, prostate: 1, primary lesion unknown: 48）	78	53%	14.5

2.2.3. Standard treatment for gastrointestinal/hepatobiliary or pancreatic primary NEC

Treatments for extrapulmonary NEC have not been developed according to any specific organ, and the standard treatment for gastrointestinal/hepatobiliary or pancreatic NEC is also both, EP therapy and IP therapy. While both treatments are considered standard treatments, since this study considers them both to be study treatments, the anticipated effect and expected adverse reactions from EP therapy and IP therapy have been described in “2.3.2 Study treatment(s) of this study”.

Herewith, we describe the current status of the usage of each treatment in Japan and overseas. According to the multicenter joint study by Yamaguchi and Machida, 160 out of 258 patients (62%) who received systemic chemotherapy underwent IP therapy, the most common treatment, followed by EP therapy (46 patients, 18%). Although there is big deviation where 92% of gastrointestinal primary lesion NEC (142/154 patients) were given IP therapy, and 65% of hepatobiliary or pancreatic primary lesion NEC (34/52 patients) were given EP therapy, the present situation shows that treatments are being selected by discretion or preference of the facility/physician.

Furthermore, even with respect to the dosing schedule, the method of administration has not necessarily as per the treatment of small cell lung carcinoma. There is no unified consensus, as some facilities use methods used for treatment of gastric cancer (JCOG9912 regimen), based on the reasoning that they have familiarity with IP therapy. On the other hand, in the NORDIC NEC study the most common treatment used was the EP therapy, which was administered to 129 out of 252 patients (51%) who received systemic chemotherapy, followed by a combined therapy consisting of carboplatin and etoposide therapy (67 patients, 27%). Combined therapy of platinum and etoposide was used often, which is another choice of treatment in Japan[11].

2.3. Rationale for establishment of treatment plan

2.3.1. Drugs

1) Etoposide

Etoposide exerts an antitumor effect by inhibiting topoisomerase II, which catalyzes the untangling of supercoiled DNA strands. Main toxicities include myelosuppression, nausea/vomiting, alopecia, and stomatitis.

2) Cisplatin

It is a complex ion form of the heavy metal platinum which shows anti-tumor effect by cross-linking double-stranded DNA. Currently, it is considered one of the key drugs for NEC treatment, being a central medicine for the treatment of lung cancer due to its synergistic effect with radiation therapy and various drugs, and also due to its low bone marrow toxicity when used alone. Toxicities include nausea/vomiting, nephrotoxicity, and neurotoxicity. Sufficient infusion of cisplatin before and after drug administration is necessary to prevent nephrotoxicity.

3) Irinotecan

Irinotecan is a topoisomerase I inhibitor developed in Japan that inhibits DNA synthesis. The drug is directly converted into the active metabolite (SN-38) in human liver and various tissues by carboxyesterase. As it shows a potent antitumor effect against SCLC even when used on its own, this drug is used very often in routine clinical practice to treat NEC as well. Diarrhea and myelosuppression are observed as dose-dependent toxicities. Nausea/vomiting and interstitial pneumonia have also been noted in patients.

2.3.2. Study treatment(s) of this study

1) Etoposide/cisplatin combined therapy (EP therapy)

In the multicenter joint study performed by Yamaguchi and Machida, the response rate of gastrointestinal primary lesion NEC to EP therapy was 75% (9/12 patients), with an MST of 14 months. The response rate of hepatobiliary and pancreatic primary lesion NEC to EP therapy was 12% (4/34 patients), and the MST was 6.9 months. With respect to safety, the multicenter joint study by Yamaguchi and Machida showed no treatment-related deaths associated with EP therapy (N = 46) administered as first-line treatment for gastrointestinal/hepatobiliary and pancreatic primary lesion NEC, and toxicity-related study discontinuation was observed in 6.5% of cases. Details of toxicities remain unknown as they were not investigated. The observational study of EP therapy for hepatobiliary and pancreatic primary lesion NEC (N = 21) carried out by Iwasa et al. at the National Cancer Center Hospital showed major Grade 3/4 adverse events to be neutropenia (90%), nausea (33%), and anorexia (24%). Grade 3 febrile neutropenia was observed in 8 patients (38%)[39]. Table 2.3.2 shows the toxicity profile of EP therapy from the JCOG9511 study which treated small cell lung carcinoma (Grade 3 or higher as per JCOG toxicity criteria).

Table 2.3.2. Toxicity of EP therapy and IP therapy in studies treating small cell lung carcinoma※
※Report by Iwasa et al., JCOG0509：CTCAE v3.0 Grade 3 or higher, JCOG9511：JCOG toxicity criteria Grade 3 or higher

	EP therapy		IP therapy	
	Report by Iwasa et al.[34]	JCOG9511[27]	JCOG9511[27]	JCOG0509[30]
Neutropenia	90%	92.2%	65.3%	58.5%
Leukopenia	71%	51.9%	26.7%	22.5%
Decreased hemoglobin	29%	29.9%	26.7%	23.2%
Thrombocytopenia	24%	18.2%	5.3%	2.1%
Diarrhea	0%	0%	16%	7.7%

Nausea	33%	6.5%	13.3%	6.3%
Aspartate aminotransferase increased	19%	2.6%	0%	-
Alanine aminotransferase increased	24%	3.9%	4%	-
Blood bilirubin increased	19%	0%	0%	-
Creatinine increased	0%	0%	0%	-
Peripheral motor neuropathy	0%	0%	0%	-
Febrile neutropenia	38%	0%	0%	1.4%

There are several reports concerning the specific dosage schedule/administered dose for EP therapy, and four dosing methods have been described in the NCCN Guidelines for SCLC as well. However, it is not clear as to which of the dosing methods is the best, and the dosing method for NEC varies between reports. In Japan, the dosing for EP therapy according to JCOG9511 is also often used for NEC. For this reason, this study would also follow the same method of administration. In terms of the number of cycles, a comparison of 4 courses vs. 8 courses of combined chemotherapy including cyclophosphamide for SCLC showed poor efficacy even when the treatment continued for long-term[46], and treatment of 4 cycles has also been specified in JCOG9511. There have been no such comparative studies for gastrointestinal/hepatobiliary and pancreatic primary lesion NEC, rather, there is no evidence to suggest that first-line chemotherapy should be discontinued if the chemotherapy is efficacious and toxicity is within the permitted scope. Unlike SCLC, NEC does not respond well to chemotherapy and tends to re-exacerbation to the underlying disease soon after the end of chemotherapy. For this reason, continuation of chemotherapy is very likely to be beneficial for patients with NEC as long as the treatment is effective.

While peripheral motor neuropathy, hearing impairment, and renal disorder are known accumulation toxicities of cisplatin, if sufficiently safe, it is ideal for treatments to continue as much as possible, considering the very few treatment options available for NEC. Based on the above rationale, we decided to ensure safety by establishing strict protocol treatment discontinuation criteria and chose not to limit the total dose or prescribe the number of treatment cycles.

2) Irinotecan/Cisplatin combined therapy (IP therapy)

In the multicenter joint study by Yamaguchi and Machida, the response rate of gastrointestinal primary lesion NEC to IP therapy was 51% (73/142 patients), with an MST of 13.4 months. The response rate of hepatobiliary and pancreatic primary lesion NEC to IP therapy was 39% (7/18 patients), and the MST was 10.1 months. There were no treatment-related deaths among 160 patients, and discontinuation due to toxicity in first-line chemotherapy was seen in 11.2% of cases. Details about toxicity were not collected. As a reference, Table 2.3.2 shows the toxicity profile of IP therapy from the JCOG9511 and JCOG0509 studies which treated small cell lung carcinoma.

There are several reports concerning specific dosing methods for IP therapy, and two dosing methods have been described in the NCCN Guidelines for SCLC as well. It is not known which of the dosing methods is the best, and the dosing method has also been variable in the NEC. In Japan, the dosing method of IP therapy, according to JCOG9511 is considered the standard treatment for SCLC and the same method has also been used in this study.

The total dose and number of cycles would have not been prescribed for the same reason as EP therapy, and safety has been ensured by establishing strict protocol treatment discontinuation criteria.

3) Other study treatment candidates

Presently, there are no drugs or treatment methods, including molecular-targeted drugs, which surpass the EP and IP therapy. There are also no planned or ongoing large-scale clinical trials globally, related to first-line treatment of NEC.

As the second-line treatment, a phase II study of Everolimus is underway for pancreatic primary lesion NEC resistant or unresponsive to platinum preparations. It is a multicenter study being carried out in 31 facilities in Japan, led by the National Cancer Center East Hospital, through the cancer research and development fund (UMIN000012752).

2.3.3. Summary of risk/benefit balance of the standard treatment and study treatment

In the multicenter joint study by Yamaguchi and Machida, IP therapy had better prognosis compared to EP therapy (MST: 13.0 months vs. 7.3 months; $p < 0.0001$). However, as shown in Table 2.3.3, since IP therapy was more frequently chosen for gastrointestinal primary lesions (142/154 patients) and EP therapy for hepatobiliary and

pancreatic primary lesions (34/52 patients), it has not been possible to determine whether this difference is due to difference in efficacy between the regimens, or due to differences in the primary lesion organ. Primary lesion organ remained a significant prognostic factor after multivariate analysis, and while IP therapy turned out to be a slightly better regimen than EP therapy with HR = 0.8 (95% CI. 0.48–1.33), $p = 0.389$ indicated that there was no significant difference between the two (see "2.1.5 Prognostic/predictive factors"). Taking into account the number of EP therapy cases (46 patients), the fact that it was an observational study, and including other unknown bias, the data interpretation is fairly limited. Therefore, based on these results we are unable to deem either of IP or EP therapies to be more efficacious than the other.

With regard to toxicity, while myelosuppression such as neutropenia is milder in IP therapy than EP therapy, frequencies of events such as diarrhea and nausea are high. While the two treatments have different toxicity profiles, it is difficult to conclude that one has clearly worse toxicity than the other. The risk of bile duct obstruction is high in biliary and pancreatic primary lesion NECs and the use of irinotecan (which undergoes biliary excretion) in patients exhibiting biliary excretion disorder runs the risk of increased toxicity, hence care must be taken while administering irinotecan. We believe that this risk can be avoided by appropriate pre-treatment drainage and proper monitoring during treatment. Based on the above, it is difficult to assign superiority to IP therapy or EP therapy over the other in terms of risk/benefit balance, and both the regimens can be considered suitable standard treatments.

Table 2.3.3. Summary of results from multicenter study by Yamaguchi and Machida

		IP	EP	*p*-value*
Total	N	160	46	
	Response rate（%）	50	27	＜0.001
	Median PFS (in months)	5.2	4.0	0.033
	Median OS (in months)	13.0	7.3	＜0.0001
Gastrointestinal primary lesion NEC	N	142	12	
	Response rate（%）	51	75	0.14
	Median PFS (in months)	5.4	4.9	0.585
	Median OS (in months)	13.4	14.0	0.976
Hepatobiliary and pancreatic primary lesion NEC	N	18	34	
	Response rate（%）	39%	12%	0.034
	Median PFS (in months)	4.4	3.7	0.056
	Median OS (in months)	10.1	6.9	0.05

*Response rate by chi-squared test, PFS and OS by log-rank test

2.3.4. Post-treatment(s)

In the multicenter joint study by Yamaguchi and Machida, 56% (116/206 patients) of patients with NEC who received IP therapy or EP therapy were administered chemotherapy as the second-line of treatment. The most common second-line chemotherapy after IP therapy was amurcibin (22/88 patients), while irinotecan was the most common second-line treatment after EP therapy (13/28 patients), and we expected a similar pattern for the second-line treatments in this study as well. The overall performance of second-line chemotherapy was poor with a response rate of 11%, and PFS of 2.1 months. As such, the significance of the second-line treatment has not been indicated, and hence, no standard treatment has been established.

2.4. Study design

2.4.1. Rationale for establishing endpoints

For NECs, an alternative endpoint for overall survival, such as PFS has not been established as an indicator for comparing the usefulness of the treatment regimens. Therefore, comparison of overall survival was considered appropriate when examining the therapeutic effects. For the same, overall survival was examined as the primary endpoint. The secondary endpoints to evaluate the efficacy and safety were the response rate (only for patients with a measurable lesion), progression-free survival, incidence rate of adverse events, and dose intensity of cisplatin.

2.4.2. Clinical hypothesis and rationale for setting the number of enrollments

This study compares the two standard treatments, both of which are difficult to deem superior than the other in terms of efficacy and safety. We have, therefore, adopted a study design using bilateral testing. The main clinical hypothesis of this study is that either IP therapy or EP therapy is likely to provide better overall survival than the other treatment group, and should this hypothesis be validated, the superior treatment with statistical significance would be deemed the better treatment, and would therefore be positioned as the standard treatment in future. If the study fails to validate this hypothesis, it would indicate that there was no clinically significant difference in overall survival between the two groups, and if there are no large differences in terms of toxicity, we would conclude that both treatments are viable options as the standard treatment. However, if unlike our initial hypothesis, one treatment shows clearly more toxicity than the other, we would reconsider the study design before carrying out the main analysis.

Furthermore, as mentioned in “section 2.1.1 Epidemiology”, gastroenterological primary lesion NEC, which is the primary target of this study, is a very rare disease with an annual prevalence of around 3 in 100,000 people, hence, the significance level of testing has been placed at 10% bilaterally, instead of 5% bilaterally. In the multicenter joint study conducted by Yamaguchi and Machida, the MST of gastrointestinal/hepatobiliary or pancreatic primary lesion NEC was 11.5 months. To determine if one treatment is superior to the other, we expected a difference of four months in terms of MST. If we expect the more inferior treatment to have an MST of 8.0 months and the superior treatment to have an MST of 12.0 months, the number of enrollments are calculated using the aforementioned parameters and based on the discussion mentioned later (see “12.2 Expected number of enrollments/Enrollment period/Follow-up period”). This provided a research period of 7- years, consisting of 6-years of enrollment period and one year of follow-up period, and with $\alpha = 0.1$ bilaterally and detection power of 70% to detect differences between the two groups, 63 subjects were required per group. Taking into consideration some subjects who could be lost post follow-up, we aimed to enroll 70 subjects per group, and a total of 140 subjects between two groups.

If enrollment proceeds better than expected than prior to the start of the study (i.e. if the number of enrolled subjects reach 70 in less than 2.5 years from the start of enrollment), the number of enrollments is likely to be re-established by changing the detection power from 70% to 80% during the study, with the aim of obtaining more accurate results.

＜Addition to ver.1.1＞

The pace of enrollment after the start of enrollment period exceeded expectations, and the number of enrollments reached 70 subjects in October 2016, which was 2 years and 2 months after the start. As this exceeded the criteria of 70 subjects in less than 2.5 years, since the start as provided above, we obtained the approval of the Hepatobiliary and Pancreatic Oncology Group Meeting on October 29, 2016, Esophageal Cancer Group Meeting on November 19, 2016, and Gastric Cancer Group Meeting on January 7, 2017 to change the detection power to 80% according to provisions at the time of study planning. Consequently, the number of intended enrollments was changed to 170 subjects.

2.4.3. Expected patient enrollment

In the multicenter joint study by Yamaguchi and Machida, 258 cases of gastrointestinal/hepatobiliary or pancreatic primary lesion NECs were reported from 23 sites during the 11 years between 2000 and 2011, but between 2000 and 2006, information could not be obtained because of old cases. When limited to the most recent five years (2006–2011), when sufficient information was obtained from each facility, the number of enrollments was placed at162 subjects.

This is a joint study between three groups, namely the JCOG Hepatobiliary and Pancreatic Oncology Group,

JCOG Gastric Cancer Group, and the JCOG Esophageal Cancer Group. Therefore, the total number of facilities, excluding overlaps, amounted to 82 facilities. Compared to the multicenter joint study by Yamaguchi and Machida, we expect an increase in the number of patients enrolled, and simple calculations lead us to extrapolate 115 subjects to be enrolled annually. On the other hand, if we take into account the possibility of patient enrollments being biased to some high volume centers, it would be difficult to estimate the effect of increasing facility count to proceed. In addition, considering that the study is a randomized, we estimate the annual number of patients enrolled to be between 30–50 people. Taking into account ineligible cases, we provided 6-years for patient enrollment period.

2.4.4. Rationale for setting allocation adjustment factors

1) Facility

It is widely known that background, treatment, efficacy evaluation, and safety evaluation of enrolled patients vary depending on the facility, and JCOG standards have been used to make adjustments between facilities.

2) Primary lesion organ (gastrointestinal tract vs. hepatobiliary or pancreatic organ)

In the multicenter joint study by Yamaguchi and Machida, multivariate analysis using the Cox proportional hazard model was carried out with 183 subjects, which indicated primary lesion organ (gastrointestinal tract vs. hepatobiliary or pancreatic organ) to be a significant prognostic factor.

2.4.5. Centralized pathological diagnosis

In this study, a centralized pathological diagnosis would be performed for analysis of NEC, despite patients being diagnosed by a third party. The operation of the centralized pathological diagnosis is described in section 15.1, while details of the operation have been described in the Centralized Pathological Diagnosis Procedure Manual. Furthermore, an analysis of endpoints based on the results of centralized pathological diagnosis would be provided as a reference.

2.5. Summary of expected advantages and disadvantages associated with study participation

2.5.1. Expected advantages

Drugs used for both groups of this study are treatments used in routine medical practice. As mentioned in the next section, although it would be necessary to use some drugs for which insurance coverage does not apply, since insurance claims for these treatments are made in a similar manner to general medical care however, in practice they do not receive insurance assessments.

Furthermore, cases of off-label medication have been studied according to “18th Case Providing Review Information” at the Case Review Committee established by the Health Insurance Claims Review & Reimbursement Services. Based on the review information provided as on February 26, 2018;, The use of “irinotecan hydrochloride hydrate, etoposide, cisplatin, and carboplatin [injections]” is approved for treatment of “neuroendocrine carcinoma”], and the notices issued by the Ministry of Health, Labor and Welfare on February 26, 2016the results of this study have been considered valid by the ministry. For the same reason, essentially since February 26, 2018, there have been no concerns about claims of insurance assessments.

Moreover, since the medical fees of study participants during the study, which includes drug fees, are paid in principle by the patients themselves and their insurance coverage, so the patients would receive no special medical or financial benefits from participation in this study.

2.5.2. Expected risks and disadvantages

Both treatment arms A and B would receive chemotherapy regimen used in routine medical practice, so they would be unlikely to be exposed to special risks or disadvantages not observed in routine medical practice. The descriptions in “2.3.3 Risk/benefit balance of standard treatment and study treatments” outline the expected risks and disadvantages for each treatment arm.

To minimize the risk of adverse events and disadvantages, the “ Patient selection criteria (Section 4)”, “ Criteria to change treatment (Section 6.3)” and “Concomitant/Supportive therapies (Section 6.4)” have been carefully considered for the three groups. Furthermore, the Institutional Review Board would monitor if the adverse events are within the scope of expectation, while any serious adverse events or unexpected adverse events would be carefully examined and reviewed according to the provisions related to “JCTN-Adverse Events Report Guideline”

and “JCOG Guidelines for Handling Clinical Safety Information”, and a system has been provided to take any necessary countermeasures.

Precautions related to etoposide, irinotecan, and cisplatin

As of December 2013, etoposide therapy, which is the intended to be used in this study had not received insurance approval for the treatment of gastrointestinal or hepatobiliary and pancreatic primary lesion cancers. Irinotecan has also not received insurance approval for treatment of cancers with primary lesions in organs other than the stomach, colon, and rectum. Furthermore, as cisplatin is only indicated for esophageal, gastric cancer, and for biliary tract cancers when combined with gemcitabine (25 mg/m^2), the dosage and administration used in this study have not been approved previously.

As each facility in this study may carry out the insurance claims in the same manner as routine general medical practice, the treatment may receive insurance assessment after-the-fact. However, if a facility incurs losses, the loss must be borne by the relevant facility (medical institution), as there is no system of compensation arranged by the JCOG Research Organization. If actual losses occur, the continued participation in the study would then be carefully discussed between the facility supervisor and the principal investigator/clinical trial secretariat. The facility supervisor would be expected to gain approval from the facility IRB and the head of the medical institutions.

2.6. Significance of this study

While both, EP therapy and IP therapy are standard treatments, the efficiency of either treatment being better than the other is not yet clear, and currently in routine medical practice, the choice of treatment is being made on the discretion or preference of the facility/attending physician.

If this study is able to clearly indicate the efficiency of EP therapy or IP therapy over the other, we expect that the diminished use of the relatively inferior treatment would contribute to improvement in patient prognosis. If the study indicates no clinically meaningful differences between the two treatments, the results would provide evidence that there is no significant issue with the treatments being chosen by discretion or preference of the facility/attending physician, which has been done traditionally without any data that directly compare the two treatments, although that would not indicate that the effects of the two treatments are equivalent. We also expect that the establishment of a highly reliable standard treatment in this study which would provide a foundation for therapeutic development when promising treatment regimen in the future.

As NEC is a rare disease, and since each clinician is not likely to have adequate experience, we believe that work associated with multicenter studies such as confirmation of diagnosis by centralized diagnosis/results feedback and sharing results of treatments by unified treatment regimen would lead to improvement of quality of care for NEC in Japan.

2.7. Associated research (including sample analysis research)

No such studies have been planned or carried out at the time of preparation of the protocol.

2.8. JCOG-Biobank Japan (BBJ) collaborating biobank

This study would participate in the banking of blood samples (DNA/plasma) through the JCOG-BBJ collaboration biobank based on the protocol common to all JCOG studies (hereafter termed as “common banking”).

Common banking by JCOG-BBJ collaborating biobank would collect and store the samples of patients enrolled in clinical studies conducted by JCOG regardless of presence/absence of pre-planned sample analysis research, would also provide samples for future analysis research and diagnostic information obtained through the main research.

The target of such biobanking are those patients who provided consent to participate in this study and gave consent to provide samples to the JCOG-BBJ collaborating biobank for their use in future sample analysis research (hereafter termed as consent for banking).

Samples collected during common banking include whole blood and preserved pathological tissue samples from routine clinical practice. Plasma and DNA separated/isolated from blood would be stored in the JCOG-BBJ collaborating biobank and would be provided for sample analysis research in the future. Preserved pathological tissue samples from routine medical practices such as surgery, and biopsy/clinical laboratory tests would also likely to be used for future sample analysis research. Although the type, sample preparation methods, and tissue quantity required would vary from study to study, there is no definite consensus that banking pathological tissue in a certain

method is more efficient than others. Furthermore, it has been suggested that long-term preservation of a sectioned specimen from preserved pathological tissue may lead to sample deterioration (DNA fragmentation). The discussions between the of JCOG and BBJ personnel about these problems, led to the conclusion that preserved pathological tissue taken after medical examination shall require only patients' consent for future use, and actual collection must be started after standardizing a separate protocol and specifying in it the procedures best suited for the details of the research.

The detailed procedures for sample collection, storage, and method of sample provision for future sample analysis research in common banking have been stipulated in the "JCOG-BioBank Japan Collaborating Biobank Protocols" that apply to all the JCOG studies. To participate in the common banking carried out by JCOG-BBJ collaborating biobanks, the subject matter must be reviewed and approved by the ethics committees of each participating facility.

Furthermore, to conduct sample analysis research in future using samples stored in the JCOG-BBJ collaborating biobank, it would be necessary to prepare a "Sample Analysis Research Protocol" and have it reviewed and approved by the JCOG Protocol Review Committee and the ethics committee of facilities involved in the sample analysis.

3. Criteria/definitions used in this study

Tissue classification would be performed according to WHO 2010 classification and ENETS（European Neuroendocrine Tumor Society） classification[14, 15], while disease staging would be done according to “UICC-TNM 7th Edition”.

3.1. Tissue classification (WHO 2010 classification)

The shaded parts are the targets of this study

Neuroendocrine neoplasms

1) Neuroendocrine tumor：NET Grade 1（NETG1）
2) Neuroendocrine tumor：NET Grade 2（NETG2）
3) Neuroendocrine carcinoma：NEC（large cell or small cell type）
4) Mixed adenoendocrine carcinoma（MANEC）
5) Hyperplastic and preneoplastic lesions

3.2. Grade classification （ENETS [European Neuroendocrine Tumor Society] / WHO2010 classification）

Grade 1（G1）	Number of mitotic presentations < 2 per 10 high power fields（HPF）and/or Ki-67 index ≤2%＊
Grade 2（G2）	Number of mitotic presentations 2–20 per 10 HPF and/or Ki-67 index 3-20%
Grade 3（G3）	Number of mitotic presentations > 20 per 10 HPF and/or Ki-67 index >20%

＊The description of number of mitotic presentations in WHO 2010 classification is ≤2, but 2–3% are classified to G1[47].

3.3. Histopathological diagnosis

- Immunostaining (Chromogranin A and synaptophysin) is essential for a pathological diagnosis of NEC.
- Either1. or 2., or both are studied to determine proliferative activity. Number of mitotic presentations and Ki-67 index would adopt a high grade evaluation.
  1. Number of mitotic presentations (to evaluate 50HPF)
  2. Ki-67 index（500–2000 tumor cells*）

  *（Only in this study, if the number of tumor cells in the collected sample is less than 500, the number of tumor cells measured is also listed. A minimum of 100 tumor cells is required.）
- If sufficient amount of biopsy sample cannot be collected for a pathological diagnosis, a cell block prepared using material obtained from EUS-FNA, brushing or needle biopsy may be used. However, the use of a cell block prepared from ascites or pleural effusion is not permitted.
- During a histological diagnosis using a resected tumor sample, a slide with a representative section shall be used to evaluate the proportion of NEC components (differential diagnosis with mixed adenoendocrine carcinoma). For a comprehensive examination, the entire tumor must be evaluated.

For further details NEC Pathological Diagnosis Handbook would be referred (posted on JCOG website).

3.4. Disease stage classification criteria (UICC-TNM 7th Edition)

Special notice regarding disease stage classification: Although there is an independent TNM classification for NET G1 and NET G2 with primary lesions in the stomach, small intestines, and colon; since this study concerns NEC, the disease would be classified according to the classification method of major tissue type in each primary lesion organ (squamous cell carcinoma for esophagus, and adenocarcinoma for all other primary lesion organs). In this study, the classification of intrahepatic cholangiocarcinoma is used for hepatic NEC, due to their similar clinical presentation.

3.4.1. Esophagus

T –Primary tumor

TX： Primary tumor cannot be evaluated
T0： Primary tumor unidentified
Tis： Epithelial carcinoma/highly dysplastic
T1： Tumor invading mucosal lamina propria, muscularis mucosae, or submucosa
  T1a：Tumor invading the mucosal lamina propria or muscularis mucosae
  T1b： Tumor invading the submucosa

T2 : Tumor invading the muscularis propia

T3 : Tumor invading the adventitia

T4 : Tumor invading the surrounding tissue

T4a : Tumor invading the pleura, pericardium, and diaphragm

T4b : Tumor invading other surrounding tissues such as the aorta, centrum, trachea

N –Regional lymph nodes

NX : Regional lymph node metastasis cannot be evaluated

N0 : No regional lymph node metastasis

N1 : 1–2 foci of regional lymph node metastases

N2 : 3–6 regional foci of lymph node metastases

N3 : 7 or more foci of regional lymph node metastases

M –Distal metastasis

M0 : No distal metastasis

M1 : Distal metastasis present

Stage	T	N	M
**0**	Tis	N0	M0
**IA**	T1	N0	M0
**IB**	T2	N0	M0
**IIA**	T3	N0	M0
**IIB**	T1, T2	N1	M0
**IIIA**	T4a	N0	M0
	T3	N1	M0
	T1, T2	N2	M0
**IIIB**	T3	N2	M0
**IIIC**	T4a	N1、N2	M0
	T4b	Unrelated to N	M0
	Any T	N3	M0
**IV**	Any T	Any N	M1

3.4.2. Stomach

T –Primary tumor

TX : Primary tumor cannot be evaluated

T0 : Primary tumor unidentified

Tis : Epithelial carcinoma: Epithelial carcinoma/highly dysplastic carcinoma not invading the lamina propia mucosa

T1 : Tumor invading mucosal lamina propria, muscularis mucosae, or submucosa

T1a : Tumor invading the mucosal lamina propria or muscularis mucosae

T1b : Tumor invading the submucosa

T2 : Tumor invading the muscularis propia

T3 : Tumor invading the subserosa

T4 : Tumor perforating the serosa, or invading adjacent structures[1,2,3]

T4a : Tumor perforating the serosa

T4b : Tumor invading adjacent structures[1,2,3]

1. Adjacent organs of the stomach are spleen, transverse colon, liver, diaphragm, pancreas, abdominal wall, adrenal glands, kidneys, small intestine, and retroperitoneum.
2. If the invasion has spread from the stomach to the duodenum or esophagus, classification is made in terms of depth.
3. A tumor that advances into the gastrocolic ligament, into the hepatogastric ligament, or into the greater or lesser omentum, and is classified as T3, when there is no perforation of the visceral peritoneum.

N –Regional lymph nodes

NX : Regional lymph node metastasis cannot be evaluated
N0 : No regional lymph node metastasis
N1 : 1–2 regional foci of lymph node metastases
N2 : 3–6 regional foci of lymph node metastases
N3 : 7 or more regional foci of lymph node metastases
N3a : 7–15 regional foci of lymph node metastases
N3b : 16 or more regional foci of lymph node metastases

M –Distal metastasis
M0 : No distal metastasis
M1 : Distal metastasis present

Stage	T	N	M
**0**	Tis	N0	M0
**IA**	T1	N0	M0
**IB**	T2	N0	M0
	T1	N1	M0
**IIA**	T3	N0	M0
	T2	N1	M0
	T1	N2	M0
**IIB**	T4a	N0	M0
	T3	N1	M0
	T2	N2	M0
	T1	N3	M0
**IIIA**	T4a	N1	M0
	T3	N2	M0
	T2	N3	M0
**IIIB**	T4b	N0, N1	M0
	T4a	N2	M0
	T3	N3	M0
**IIIC**	T4a	N3	M0
	T4b	N2, N3	M0
**IV**	Any T	Any N	M1

3.4.3. Small intestines (including duodenum)

T –Primary tumor
TX : Primary tumor cannot be evaluated
T0 : Primary tumor unidentified
Tis : Epithelial carcinoma
T1 : Tumor invading mucosal lamina propria, muscularis mucosae, or submucosa
T1a : Tumor infiltrating the mucosal lamina propria or muscularis mucosae
T1b : Tumor infiltrating the submucosa
T2 : Tumor infiltrating the muscularis propia
T3 : Tumor invading the subserosa, or tumor invading within 2 cm of surrounding tissue of muscularis externa without peritoneal cover (mesenterium, retroperitoneum)*

*Surrounding tissue of muscularis externa without peritoneal cover refers to the mesenterium in the jejunum and ileum, and the retroperitoneum in the duodenum without serosa.

T4 : Tumor penetrating the visceral peritoneum, or tumor directly invading another organ or tissue (invasion of other loops of the small intestine, invasion by 2 cm or more into the mesenterium and retroperitoneum, including invasion into the abdominal wall through the serosa; invasion to the pancreas only for duodenum)

N –Regional lymph nodes
NX : Regional lymph node metastasis cannot be evaluated
N0 : No regional lymph node metastasis

N1 : 1–3 regional foci of lymph node metastases

N2 : 4 or more regional foci of lymph node metastases

M –Distal metastasis

M0 : No distal metastasis

M1 : Distal metastasis present

Stage	T	N	M
**0**	Tis	N0	M0
**I**	T1, T2	N0	M0
**IIA**	T3	N0	M0
**IIB**	T4	N0	M0
**IIIA**	Any T	N1	M0
**IIIB**	Any T	N2	M0
**IV**	Any T	Any N	M1

3.4.4. Appendix (partial revision of UICC-TNM 7th Edition)

T –Primary tumor

TX : Primary tumor cannot be evaluated

T0 : Primary tumor unidentified

Tis : Epithelial carcinoma: Tumor invading epithelium or lamina propia mucosae

T1 : Tumor invading the submucosa

T2 : Tumor invading the muscularis propia

T3 : Tumor invading the subserosa or mesoappendix

T4 : Tumor penetrating the visceral peritoneum, tumor including a peritoneal mucosal tumor in the lower right abdomen and/or tumor directly invading other organs or tissue

T4a : Tumor penetrating the visceral peritoneum, or peritoneal mucosal tumor in the lower right abdomen

T4b : Tumor directly invading other organs or tissues

N –Regional lymph nodes

NX : Regional lymph node metastasis cannot be evaluated

N0 : No regional lymph node metastasis

N1 : 1–3 regional foci of lymph node metastases

N2 : 4 or more foci of regional lymph node metastases

M –Distal metastasis

M0 : No distal metastasis

M1 : Distal metastasis present

M1a : Peritoneal metastasis beyond the lower right abdomen, including pseudomyxoma peritonei

M1b : Distal metastasis other than peritoneal metastasis

Stage	T	N	M
**0**	Tis	N0	M0
**I**	T1, T2	N0	M0
**IIA**	T3	N0	M0
**IIB**	T4a	N0	M0
**IIC**	T4b	N0	M0
**IIIA**	T1, T2	N1	M0
**IIIB**	T3, T4	N1	M0
**IIIC**	Any T	N2	M0
**IVA**	Any T	N0	M1a
**IVB**	Any T	N0	M1a
	Any T	N1, N2	M1a
**IVC**	Any T	Any N	M1b

3.4.5. Colon and rectum

T –Primary tumor

TX: Primary tumor cannot be evaluated

T0: Primary tumor unidentified

Tis: Carcinoma in situ: Tumor invading epithelium or lamina propia mucosae

T1: Tumor invading the submucosa

T2: Tumor invading the muscularis propia

T3: Tumor invading the subserosa, or tissue surrounding the colon or rectum without peritoneal covering

T4: Tumor penetrating the visceral peritoneum, and/or directly invading another organ or tissues

T4a: Tumor penetrating the visceral peritoneum

T4b: Tumor directly invading another organ or tissues

N –Regional lymph nodes

NX: Regional lymph node metastasis cannot be evaluate

N0: No regional lymph node metastasis

N1: 1–3 regional foci of lymph node metastases

N1a: 1 regional lymph node metastasis

N1b: 2–3 regional foci of lymph node metastases

N1c: Presence of tumor deposits, that is, satellite nodes in the subserosa or in the soft tissue surrounding colon or rectum without peritoneal covering, but with no regional lymph node metastasis

N2: 4 or more regional lymph node metastases

N2a: 4–6 regional foci of lymph node metastases

N2b: 7 or more regional foci of lymph node metastases

M –Distal metastasis

M0: No distal metastasis

M1: Distal metastasis present

M1a: Local metastasis to one organ (liver, lungs, ovaries, or lymph nodes other than regional lymph nodes)

M1b: 2 or more organs, or peritoneal metastasis

Stage	T	N	M
**0**	Tis	N0	M0
**I**	T1, T2	N0	M0
**II**	T3, T4	N0	M0
**IIA**	T3	N0	M0
**IIB**	T4a	N0	M0
**IIC**	T4b	N0	M0
**III**	Any T	N1, N2	M0
**IIIA**	T1, T2	N1	M0
	T1	N2a	M0
**IIIB**	T3, T4	N1	M0
	T2, T3	N2a	M0
	T1, T2	N2b	M0
**IIIC**	T4a	N2a	M0
	T3, T4a	N2b	M0
	T4b	N1, N2	M0
**IVA**	Any T	Any N	M1a
**IVB**	Any T	Any N	M1b

3.4.6. Applies to hepatic NEC (hepatic primary lesion or liver metastasis from unknown primary lesion)

T –Primary tumor

TX: Primary tumor cannot be evaluated

T0: Primary tumor unidentified

Tis : Carcinoma *in situ*

T1 : Isolated tumor without vascular invasion

T2a : Isolated tumor with vascular invasion

T2b : Multifocal tumor regardless of vascular invasion

T3 : Tumor penetrating visceral peritoneum or directly invading adjacent extrahepatic structures

T4 : Tumor with bile duct invasion (bile duct proliferative type)

N –Regional lymph nodes

NX : Regional lymph node metastasis cannot be evaluated

N0 : No regional lymph node metastasis

N1 : Regional lymph node metastasis present

M –Distal metastasis

M0 : No distal metastasis

M1 : Distal metastasis present

Stage	T	N	M
**I**	T1	N0	M0
**II**	T2	N0	M0
**III**	T3	N0	M0
**IVA**	T4	N0	M0
	Any T	N1	M0
**IVB**	Any T	Any N	M1

3.4.7. Gall bladder

T –Primary tumor

TX : Primary tumor cannot be evaluated

T0 : Primary tumor unidentified

Tis : Carcinoma *in situ*

T1 : Tumor invading mucosal lamina propria or muscularis externa

T1a : Tumor invading mucosal lamina propria

T1b : Tumor invading muscularis externa

T2 : Tumor invading the connective tissue around the muscularis externa, but shows no progression beyond the serosa or to the liver

T3 : Tumor perforating the serosa (visceral peritoneum), tumor directly advancing to the liver and/or an adjacent organ that is not the liver (stomach, duodenum, colon, pancreas, omentum, or extrahepatic bile duct)

T4 : Tumor invading the main trunk of the portal vein or the hepatic artery, or tumor advancing to two or more adjacent organs which are not the liver

N –Regional lymph nodes

NX : Regional lymph node metastasis cannot be evaluated

N0 : No regional lymph node metastasis

N1 : Regional lymph node metastasis present (cystic duct, common bile duct, proper hepatic artery, including lymph nodes along the portal vein)

M –Distal metastasis

M0 : No distal metastasis

M1 : Distal metastasis present

Stage	T	N	M
**0**	Tis	N0	M0
**I**	T1	N0	M0
**II**	T2	N0	M0
**IIIA**	T3	N0	M0
**IIIB**	T1, T2, T3	N1	M0
**IVA**	T4	Any N	M0

**IVB**	Any T	Any N	M1

3.4.8. Extrahepatic bile duct-hepatic portal region

T –Primary tumor

TX: Primary tumor cannot be evaluated

T0: Primary tumor unidentified

Tis: Carcinoma *in situ*

T1: Tumor localized to the bile duct which advances until muscularis externa or fibrous tissue

T2a: Tumor invading beyond the bile duct wall and into the surrounding adipose tissue

T2b: Tumor invading the adjacent liver parenchyma

T3: Tumor invading the branch of one side of portal vein or hepatic artery

T4: Tumor invades the main trunk of portal vein, and branches on both sides of the portal vein, proper hepatic artery or the secondary branches of the bile duct on both left and right side, or to the secondary bile duct branches on one side and portal vein or hepatic artery on the other side

N –Regional lymph nodes

NX: Regional lymph node metastasis cannot be evaluated

N0: No regional lymph node metastasis

N1: Presence of regional lymph node metastasis in the cystic duct, common bile duct, proper hepatic artery, and lymph nodes along portal vein

M –Distal metastasis

M0: No distal metastasis

M1: Distal metastasis present

Stage	T	N	M
**0**	Tis	N0	M0
**I**	T1	N0	M0
**II**	T2a, T2b	N0	M0
**IIIA**	T3	N0	M0
**IIIB**	T1,T2, T3	N1	M0
**IVA**	T4	N0, N1	M0
**IVB**	Any T	Any N	M1

3.4.9. Extrahepatic bile duct-distal

T –Primary tumor

TX: Primary tumor cannot be evaluated

T0: Primary tumor unidentified

Tis: Carcinoma *in situ*

T1: Tumor localized in the bile duct wall

T2: Tumor invading beyond the bile duct wall

T3: Tumor invading the gallbladder, liver, pancreas, duodenum, or other adjacent organs

T4: Tumor invading the celiac axis or superior mesenteric artery

N –Regional lymph nodes

NX: Regional lymph node metastasis cannot be evaluated

N0: No regional lymph node metastasis

N1: Regional lymph node metastasis present

M –Distal metastasis

M0: No distal metastasis

M1: Distal metastasis present

Stage	T	N	M
**0**	Tis	N0	M0
**IA**	T1	N0	M0
**IB**	T2	N0	M0

**IIA**	T3	N0	M0
**IIB**	T1, T2, T3	N1	M0
**III**	T4	Any N	M0
**IV**	Any T	Any N	M1

3.4.10. Ampulla of Vater

T –Primary tumor

TX: Primary tumor cannot be evaluated

T0: Primary tumor unidentified

Tis: Carcinoma *in situ*

T1: Tumor localized to the ampulla of Vater, or sphincter of Oddi

T2: Tumor invading the duodenal wall

T3: Tumor invading the pancreas

T4: Tumor invading the soft tissue around the pancreas or other adjacent organs

N –Regional lymph nodes

NX: Regional lymph node metastasis cannot be evaluated

N0: No regional lymph node metastasis

N1: Regional lymph node metastasis present

M –Distal metastasis

M0: No distal metastasis

M1: Distal metastasis present

Stage	T	N	M
**0**	Tis	N0	M0
**IA**	T1	N0	M0
**IB**	T2	N0	M0
**IIA**	T3	N0	M0
**IIB**	T1, T2, T3	N1	M0
**III**	T4	Any N	M0
**IV**	Any T	Any N	M1

3.4.11. Pancreas

T –Primary tumor

TX: Primary tumor cannot be evaluated

T0: Primary tumor unidentified

Tis: Carcinoma *in situ*

T1: Tumor localized within the pancreas, with a maximum size of < 2 cm

T2: Tumor localized within the pancreas, with a maximum size > cm

T3: Tumor advancing outside the pancreas, but with no invasion to the celiac axis or superior mesenteric artery

T4: Tumor invading the celiac axis or superior mesenteric artery

N –Regional lymph nodes

NX: Regional lymph node metastasis cannot be evaluated

N0: No regional lymph node metastasis

N1: Regional lymph node metastasis present

M –Distal metastasis

M0: No distal metastasis

M1: Distal metastasis present

Stage	T	N	M
**0**	Tis	N0	M0
**IA**	T1	N0	M0
**IB**	T2	N0	M0
**IIA**	T3	N0	M0

**IIB**	T1, T2, T3	N1	M0
**III**	T4	Any N	M0
**IV**	Any T	Any N	M1

3.5. Residual tumor (R) classification (UICC-TNM 7th Edition)

RX: Presence of residual tumor cannot be evaluated

R0: No residual tumor

R1: Presence of residual tumor by microscopic examination

R2: Presence of residual tumor by macroscopic examination

3.6. Definition of non-resectable NEC

Unoperated cases for which surgery as a curative process was not indicated based on clinical findings, including image-based diagnosis, or operated cases that underwent resection (including experimental laparotomy) but ultimately ended as R2 resection are considered non-resectable.

The details by organs are shown below. One or more criterion must be fulfilled for each organ.

<Esopghagus>

- Diagnose as Stage IV (UICC 7th edition) based on clinical findings.

<Stomach>

- Diagnose as Stage IV (UICC 7th edition) based on clinical or surgical findings. However, this does not include situations where Stage IV diagnosis is provided based only on peritoneal lavage cytology (CY1).
- Bulky lymph node* metastasis is identified.

  *Bulky lymph node: Two or more lymph nodes with a major axis of 1.5 cm or more, that are present in contact with each other around the celiac artery, the common hepatic artery, the splenic artery, and the proper hepatic artery, or on the front surface of the superior mesenteric vein, where single or multiple large and small lymph nodes have formed an aggregate with the overall major axis length of 3.0 cm or more.

<Duodenum (excluding the ampulla of Vater), small intestines, appendix, and colon>

- Diagnosed as Stage IV (UICC 7th edition) based on the clinical findings.

<Extrahepatic bile duct, ampulla of Vater, gallbladder>

- Distant metastasis is identified
- Para-aortic lymph node metastasis is identified
- Bulky metastasis identified in the hepatoduodenal mesentery and lymph nodes surrounding the head of the pancreas
- Invasion in the proper hepatic artery, common hepatic artery, celiac artery, or the superior mesenteric artery identified
- Invasion in the hepatic artery branches on both the left and right side identified
- Extensive invasion or occlusion of the main trunk of portal vein, or invasion in the portal vein branches on both the left and right side identified
- Invasion in the blood vessels on the lobe on one side of the liver (portal vein or artery) or atrophy of lobe on one side of liver identified, and advancement into the bile duct on the other side is identified up to the secondary branch level
- Advancement into the bile ducts on both sides identified up to the secondary branch level

<Pancreas>

- Diagnosed as Stage III or Stage IV (UICC 7th edition) based on clinical findings.

＜Hepatic NEC (hepatic primary lesion or liver metastasis from unknown primary lesion)>

- Distal metastasis* other than intrahepatic metastasis identified.

  *Limited to organs that cannot be the primary lesion site (bone, lymph nodes below the diaphragm,

peritoneum, subcutaneous, muscle, and spleen) (see 3.8)

- Para-aortic lymph node metastasis is identified
- Bulky metastasis identified in the hepatoduodenal mesentery and lymph nodes surrounding the head of the pancreas
- Multiple masses identified in the liver.
- Invasion in one of the proper hepatic artery, common hepatic artery, celiac artery, or the superior mesenteric artery identified.
- Invasion to hepatic artery branches on both the left and right side identified
- Extensive invasion or occlusion of the main trunk of portal vein, or invasion in portal vein branches on both the left and right side identified
- Invasion in blood vessels on lobe on one side of the liver (portal vein or artery) or atrophy of lobe on one side of liver identified, and advancement into the bile duct on the other side is identified up to the secondary branch level
- Advancement into the bile ducts on both sides identified up to the secondary branch level

3.7. Definition of recurrent NEC

If surgery (R0 resection or R1 resection) was performed for gastrointestinal/hepatobiliary or pancreatic primary lesion, malignant tumor and the condition was diagnosed as NEC based on pathological samples taken from surgery, and were deemed as a clinical relapse thereafter.

If there are past histological samples from the primary lesion, no histological biopsy of the relapse lesion is necessary.

3.8. Definition of hepatic NEC (hepatic primary lesion or liver metastasis from an unknown primary lesion)

After a detailed investigation into the primary lesion by cervical-pelvic contrast CT, gastrointestinal endoscopy, FDG-PET, otolaryngology (head and neck examination), urology examination (for men only) and gynecological examination (for women only), if the tumor is in one of the following sites, the case is defined as hepatic primary lesion (or primary lesion unknown).

- Tumor is present only in the liver
- Tumor is present in an organ that cannot be a primary lesion site (bone, lymph nodes below the diaphragm, peritoneum, subcutaneous, muscle, and spleen), and in the liver.

4. Patient selection criteria

Patients that fulfill all of the following inclusion criteria and do not correspond to any of the exclusion criteria are eligible for enrollment.

4.1. Inclusion criteria (for enrollment)

1) Any of the following is applicable based on pathological diagnosis taking findings of immunohistochemistry into consideration (see 3.1. to 3.3.).

[1] Pathologically diagnosed as neuroendocrine carcinoma (NEC*1) in the resected sample.

[2] Containing pathologically confirmed component of neuroendocrine carcinoma (NEC*1) in the biopsy sample.

1: Based on WHO 2010 classification

2) Any of the following is applicable

[1] NEC arise in esophagus, stomach, duodenum, intestine, appendix, colon, rectum, gallbladder, intrahepatic bile duct, extrahepatic bile duct, ampulla of Vater, pancreas,

[2] Liver NEC (primary liver or liver metastasis of unknown primary) *2.

*2: The tumor is only in one of the following sites after a thorough examination of the primary site by contrast CT (from the neck to pelvic) and upper/lower gastrointestinal endoscopy, FDG-PET scan, otolaryngology (head and neck) examination, urology examination (male patients only), and gynecology examination (female patients only).

a. Liver only

b. Bone, lymph nodes below the diaphragm, peritoneum, subcutaneous, muscle, spleen, and liver

3) Unresectable (see 3.6) or recurrent cancer (see 3.7). It is not essential for a pathological confirmation of the metastatic lesion or recurrent site. Cases of esophageal NEC is ineligible if corresponding to any of the following.

[1] cT4.

[2] No distant metastasis rather than supraclavicular lymph node

[3] Stenosis indicated for palliative radiotherapy

4) No previous chemotherapy or radiotherapy for NEC. Pre- or post-operative chemotherapy except irinotecan or etoposide for NEC is allowed as long as it was completed at least 8 weeks prior to registration.

5) No previous chemotherapy using platinum agents for any malignancies.

6) No serious tumor-related complications.

Serious tumor-related complications include superior vena cava syndrome, inferior vena cava syndrome, pleural, ascites, or pericardial effusions that are large or uncontrollable (e.g., effusions that accumulate quickly after drainage or even after adhesive surgery), and brain metastases with neurological symptoms.

7) Aged 20 to 75 years old.

8) ECOG performance status of 0 or 1.

9) Sufficient oral intake

10) Measurable region is not required.

11) Adequate organ functions.

[1] WBC ≥3,000/ mm^3

[2] Neutrophils ≥ 1,500/ mm^3

[3] Hemoglobin ≥ 9.0 g/dL

[4] Platelets ≥ 10×10^4/ mm^3

[5] Total bilirubin≤ 1.5 mg/dL[※4]

[6] AST(sGOT)≦100 IU/L[※4]（for hepatic NEC and liver metastasis, ≦150 IU/L）

[7] ALT(sGPT)≦100 IU/L[※4]（for hepatic NEC and liver metastasis, ≦150 IU/L）

※4：Presence or absence of biliary drainage is not relevant

[8] Serum creatinine≦ 1.3 mg/dL

[9] Creatinine clearance[※5]≥ 60 mL/min

※5： Creatinine clearance must have been estimated using the Cockcroft-Gault formula, and must be 60 mL/min or more. If the estimation is less than 60 mL/min, but the actual measurement is 60 mL/min or more, the patient can be deemed eligible.

Cockcroft-Gault formula

Male：Ccr ={(140－age) × body weight (kg)}/{72×serum creatinine (mg/dL)}

Female：Ccr = 0.85×{(140－age) × body weight (kg)}/{72×serum creatinine(mg/dL)}

12) Written informed consent.

4.2. Exclusion criteria

1) Synchronous or metachronous (within 5 years) malignancies except carcinoma in situ or intramucosal tumor curatively treated with local therapy.

2) Active infection requiring systemic therapy.

3) Fever of 38 degrees Celsius or higher.

4) Pregnant or lactating women, women of childbearing potential, or women within 28 days after delivery.

5) Psychiatric disease.

6) Patients requiring systemic steroids medication.

7) Interstitial pneumonia, pulmonary fibrosis.

8) Serious co-existing illness.

9) Unstable angina pectoris within 3 weeks, or with a history of myocardial infarction within 6 months.

10) Impossible to use both iodine and gadolinium due to being allergic to contrast agent.

11) Uncontrolled diabetes mellitus or routine administration of insulin.

5. Registration and randomization

5.1. Procedure of registration

Ensure that a patient to be registered meets all eligibility criteria and does not meet any of exclusion criteria and register the patient by using JCOG Web Entry System. JCOG Web System Personal Account and password are required for web registration. If unknown, contact JCOG Data Center.

Patient registration JCOG Web Entry System
URL:https://secure.jcog.jp/dc/
(Web registration can be used for 24 hours.)

Contact information for patient registration and JCOG Web Entry System

JCOG Data Center
TEL: 03-3542-3373
Weekdays 9:00-17:00 (not available in holidays, Saturdays and Sundays, New Year's holidays)
E-mail:JCOGdata@ml.jcog.jp

Contact information on Patients Selection Criteria

Study Coordinator Contact : Chigusa Morizane
Hepato-Biliary Pancreatic Group: Futomi Mori (Main Research Secretariat)
Department of Hepatobiliary and Pancreatic Medicine, National Cancer Center
TEL:03-3542-2511
FAX:03-3542-3815
E-mail:cmorizan@ncc.go.jp

Gastric Cancer Group: Yoshinori Machida
Department of Gastroenterology, Kanagawa Cancer Center (Gastrointestinal Tract)
TEL:045-520-2222
FAX:045-520-2202
E-mail:n-machida@kcch.jp

Esophageal Cancer Group: Ken Kato/Yushi Homma
Department of Head and Neck, Esphageal Medical Oncology, National Cancer Center
TEL: 03-3542-2511
FAX: 03-3542-3815
E-mail: kenkato@ncc.go.jp / yohonma@ncc.go.jp

5.1.1. Precautions for patient registration

① Registration after initiation of protocol treatment is unacceptable.
② Registration is performed by accessing the URL in '5.1. Procedure of registration'.
③ Eligibility checks are performed on the screen of Registration Form, so it is not necessary to send a Registration Form to Data Center by mail or fax.
④ If input data are insufficient, registration is not accepted until all are met.
⑤ The registration number is issued after the confirmation of eligibility on the registration screen, then the registration is completed.
⑥ Once registered, patients will not be retracted (retracted from the database) unless there is withdrawal of consent, including refusal to use the data for research. For duplicate registration, the information at the initial registration (registration number, allocated arm) are used in any case.
⑦ When misregistration or duplicate registration is found, contact Data Center immediately.
⑧ Body surface area and drug dose calculations are institutional responsibilities, and the body surface area and drug dose displayed on Web Entry System at registration are only for double-checking. Those should

always be calculated and checked at the institution. When the body surface area calculation formula adopted in the hospital information system of the institution differs from calculation formula adopted by JCOG (Dubois formula: Body surface area (m^2) = Body weight (kg) $^{0.425}$ x Height (cm) $^{0.725}$ 84 ÷ 10,000), there can be a difference in the dose by the hospital information system of the institution and the dose by the calculation formula adopted by JCOG, but in that case, either dosage used is decided by the site investigator.

5.2. Randomization and allocation adjustment factor

During enrollment, the treatment arm for a patient is allocated randomly by the data center.

Randomization would use a method of minimization using the 1) institutions, 2) Primary lesion organ (gastrointestinal tract [esophagus, stomach, duodenum, small intestines, appendix, colon, and rectum] vs. hepatobiliary and pancreatic organ [hepatic NEC, gallbladder, extrahepatic bile duct, ampulla of Vater, or pancreas]) as the adjustment factors, so that there are no large discrepancies between them. Researchers at participating facilities would not be informed of the detailed procedures of randomization.

6. Treatment Plan and Treatment Modification Criteria

Unless patient safety is threatened, treatment and treatment modifications is done in compliance with the specifications in this chapter.

If it is considered that the protocol specification may cause medically dangerous situation of the patient, treatment modifications should be made according to the medical judgment of the investigators/sub-investigators. Such protocol deviation is considered to be "clinically relevant deviation" if considered medically appropriate (see 14.1.4.Protocol deviation/violation). Deviations that occur with intentions other than safety, such as increasing efficacy, are not considered clinically relevant deviations.

6.1. Protocol treatment

Protocol treatment is initiated within 7 days of enrollment.

If treatment initiation occurs after 8 days from registration for any reason, the reason should be documented on the Treatment Course Form. If it is determined that treatment cannot be initiated, describe the details in the Off-treatment Form as Protocol Treatment Termination.

When laboratory parameters worsen and eligibility criteria are no longer met by the start of treatment after registration, the investigator/sub-investigator is allowed to decide whether initiate or terminate protocol treatment at their own discretion.

「6.3. The course initiation criteria is not applied at treatment changes.

Drugs used

- Etoposide ※
- Cisplatin ※
- Irinotecan ※
- Entecavir, tenofovir disoproxil fumarate, tenofovir alafenamide fumarate

The use of generic drugs is not restricted.

※ The company that manufactures or distributes these drugs, or intends to manufacture or sell these drugs, requires conflicts of interest control in the Clinical Trials Act as a company involved in this study (see 13.8.).

6.1.1. Arm A: Etoposide plus cisplatin (EP) therapy

The following chemotherapy courses will be given once a week for 3 weeks and treatment will be continued until the patient meets the discontinuation criteria.

Drug	Dosage(mg/m^2)	Dosing regimen/Dosing time	Dose day
Etoposide	100	IV/60-120 min	Day 1、2、3
Cisplatin	80	IV/60-120 min	Day 1

1) Calculation of the dosage

① Body surface area is calculated by determining drug dose to the third decimal point.

② For both etoposide and cisplatin, the calculated dose is determined by truncating the decimal point. For drug doses, ±10% is the acceptable range.

③ Dose recalculation due to body weight change after treatment initiation is not performed.

2) Administration of anticancer drugs

Etoposide and cisplatin are given in any order.

Examples of administration in the package insert are described below.

(i) Before administration: Before administration of anticancer drugs, 1,000-2,000 mL of hydration is done to achieve adequate diuresis.

(ii) Etoposide: Etoposide is mixed with infusion solutions such as isotonic sodium chloride solution of 250 mL or more, and infused intravenously in about 60-120 minutes. DEHP (2-ethylhexyl) phthalate: di-(2-ethylhexyl) phthalate), which is a plasticizer, elutes from polyvinyl chloride infusion sets and catheters. Avoid the use of polyvinyl chloride infusion sets and catheters.

(iii) Cisplatin administration: Cisplatin is mixed with stock solution or 500 mL of saline (or glucose-saline) and given intravenously in 60-120 min. During the administration, caution should be exercised in ensuring urine volume, and diuretics such as mannitol and furosemide should be administered as

necessary.

(iv) After administration: After administration of cisplatin, 1,000-2,000 mL of hydration should be done so that adequate diuresis can be achieved.

In addition, short hydration administration at the discretion of each institution is permitted. (see Table 6.1.1. for treatment cases).

Table 6.1.1. Example of short hydration

	Dose	Time
5HT3 antagonist<br>Dexamethasone<br>Physiological saline	9.9 mg<br>50 mL	15 min
Etoposide<br>Physiological saline	100 mg/m^2<br>250 mL	60 min
No. 1 solution<br>Potassium chloride<br>Magnesium sulfate	500 mL<br>10 mEq<br>8 mEq	60 min
20% mannitol	200 mL	30 min
Cisplatin<br>Physiological saline	80 mg/m^2<br>250 mL	60 min
No. 1 solution<br>Potassium chloride	500 mL<br>10 mEq	60 min

(Referred from Horinouchi H, et al., Japan Society of Clinical Oncology 2012

3) Precautions for treatment

JCOG9511, an upfront trial in small-cell lung cancer, suggested an association between treatment-related deaths and first-course neutropenia. Therefore, caution should be exercised when neutropenia is strongly observed from the first course.

6.1.2. Arm B: Irinotecan plus cisplatin (IP) therapy

One 4-week course of the following chemotherapy will be continued until the discontinuation criteria are met. However, if day 15 irinotecan is skipped, 3 weeks should be used as one course, and the next course should be started with day 22.

Drug	Dosage(mg/m^2)	Dosing regimen/Dosing time	Dose day
Irinotecan	60	IV/90 min	Day 1、8、15
Cisplatin	60	IV/60-120 min	Day 1

1) Calculation of the dosage

(i) Body surface area is determined by determining the dose of drug administered until the third decimal point.

(ii) The dose is calculated for both irinotecan and cisplatin by truncating the decimal point. For drug doses, ±10% is the acceptable range.

(iii) Dose recalculation due to body weight change after treatment initiation is not performed.

2) Administration of anticancer drugs

Irinotecan and cisplatin are given in any order.

Examples of administration in the package insert are described below.

(i) Before administration: Before administration of anticancer drugs, 1,000-2,000 mL of hydration is done to achieve adequate diuresis.

(ii) Irinotecan: Irinotecan is mixed with 500 mL or more of isotonic sodium chloride solution, glucose solution, or electrolyte maintenance solution and given by intravenous drip infusion at about 90 minutes.

(iii) Cisplatin administration: Cisplatin is mixed with stock solution or 500 mL of saline (or glucose-saline) and given intravenously in 60-120 min. During the administration, caution should be exercised in ensuring urine volume, and diuretics such as mannitol and furosemide should be administered as necessary.

(iv) After administration: After administration of cisplatin, 1,000-2,000 mL of hydration should be done so that adequate diuresis can be achieved.

In addition, short hydration administration at the discretion of each institution is permitted. (See Table 6.1.1.

3) Precautions for treatment

JCOG9511, an upfront trial in small-cell lung cancer, suggested an association between treatment-related deaths and first-course neutropenia. Therefore, caution should be exercised when neutropenia is strongly observed from the first course.

6.2. Protocol Treatment Termination/Completion Criteria

6.2.1. Definition of protocol treatment completion

Protocol treatment is continued in this study unless the protocol treatment discontinuation criteria are met, so no definition of protocol treatment completion is provided.

6.2.2. Criteria for termination of protocol treatment

Protocol treatment is terminated in any of the following cases:

1) Judged as protocol treatment is ineffective
   - When a definite tumor exacerbation is confirmed by imaging or clinically
     ※ Protocol treatment should not be discontinued if the clinical judgement of PD based on the assessment of response based on imaging indicates that continuation of protocol treatment is appropriate, and protocol treatment should be continued.
2) Protocol treatment cannot be continued due to adverse events
   ① If Grade 4 non-hematological toxicity is observed (with the exception of the following adverse events) 'hypernatremia', 'hyponatremia', 'hyperkalemia', 'hypokalemia', 'hyperglycemia', 'hypoglycemia', 'alkaline phosphatase increase', 'alanine aminotransferase increase', 'aspartate aminotransferase increase', 'blood bilirubin increase','serum amylase increase', 'lipase increase', 'GGT increase'.
   (*Adverse events other than "anaemia," "myeloid cytopenia," "lymphocyte count decreased," "neutrophil count decreased," "white blood cell count decreased," "platelet count decreased," "CD4 lymphopenia" in CTCAE v4 0)
   ② If the next course cannot be initiated beyond 21 days from the expected start date of the course due to an adverse event
   ③ When the criteria for terminating protocol treatment in the treatment modification criteria (6.3.) are met.
   ④ Adverse events other than the treatment modification criteria that the investigator/sub-investigator judges to require termination of protocol treatment
3) If the patient offers termination of protocol treatment for reasons not denied to be associated with the adverse event
   - This category should be used if an association with an adverse event cannot be ruled out.
4) When the patient offers termination of protocol treatment because of reasons for denial of association with adverse events
   - Patient refusal after enrollment and before initiation of protocol treatment
   - When the association with an adverse event can first be denied, such as the relocation of the person or household member during protocol treatment.
5) Death during protocol treatment
   - Death before deciding to terminate protocol treatment for other reasons
6) Palliative surgery for pathogenic adverse events or surgery for patients with complete response to chemotherapy (see Section 6.3.9).
7) In addition, exacerbations before the start of treatment after enrollment (protocol treatment could not be initiated due to rapid exacerbation), protocol violations were found, ineligibility was determined due to modifications in pathological diagnosis after enrollment, etc., treatment was changed, and it was judged that it was difficult to continue protocol treatment due to social reasons and safety management problems, etc.

The date of discontinuation of protocol treatment is defined as the date of death in 6.2.2.5), the date of surgery in 6.2.2.6), and otherwise, the date on which the treating physician judges that protocol treatment is discontinued.

In this study, non-NEC may be diagnosed by central pathological diagnosis during protocol treatment, because central pathological diagnosis is performed. If the patient is continuing on protocol treatment when the results of the central pathology diagnosis are reported to the registry, the attending physician and the institutional pathologist will review the results and take a clinically appropriate response. Protocol treatment is discontinued if discontinuation of protocol treatment is judged to be appropriate, and the reason for discontinuation is other.

6.3. Treatment modification criteria

The following terms shall be used for the treatment modification.

Delay: Delay the start of the course or administration of treatment from the planned date.

Termination: Discontinuation of a part of or all of the treatment without restarting.

Suspending: temporary interruptions or withdrawals that may be resumed if conditions are met

Skip: Do not administer one or more drugs and proceed to the next schedule.

Categories of infection (CTCAEv4.0) used in this study are as follows

Infection: CTCAEv4.0 infections and infestations

Bronchial infection; pulmonary infection; upper respiratory tract infection; mediastinal infection; pleural infection; catheter-related infection; Biliary tract infection; Gallbladder infection; urinary tract infection

6.3.1. Arm A (EP-therapy): Dose level

1) Etoposide

Drug	Dose level	Dosing schedule	Dose day
Etoposide	Level 0	100 mg/m^2 div	Day 1、2、3
	Level -1	80 mg/m^2 div	Day 1、2、3
	Level -2	60 mg/m^2 div	Day 1、2、3

2) Cisplatin

Drug	Dose level	Dosing schedule	Dose day
Cisplatin	Level 0	80 mg/m^2 div	Day 1
	Level -1	60 mg/m^2 div	Day 1
	Level -2	40 mg/m^2 div	Day 1

6.3.2. A arm (EP therapy): Course initiation criteria

- Initiate the course after confirming that all of the following "Course Initiation Criteria" are met on the scheduled start date of the course or the day before the plan start date of the course.
- If any one is not met, the initiation of the course is delayed.
- If the course cannot be initiated within 21 days of the expected start date of the course (if the start date of the previous course was day 1 and the course could not be initiated by day 42), discontinue the protocol treatment.
- The course initiation criteria is not applied at the start of the first course.

Course initiation criteria

① Neutrophil count Grade 0-1 (≧1500 per mm^3).

② Platelet count ≧10×10^4 /mm^3

③ AST≦100 IU/L (in the presence of hepatic metastases vs. hepatic NECs) was ≦200 IU/L).

④ ALT≦100 IU/L (≦200 IU/L for liver metastases vs liver NECs).

⑤ Total bilirubin <= 2.0 mg/dL

⑥ Serum creatinine <= 1.5 mg/dL

⑦ Fever Grade 0 (axillary temperature, no antipyretic)

⑧ Constipation, fatigue, phlebitis, oral mucositis, and infection ※1 are all Grade 2 or less.

Infected ※1: bronchial infection, pulmonary infection, upper respiratory tract infection, mediastinal infection, pleural infection, catheter-related infection, biliary tract infection, gallbladder infection, urinary tract infection

⑨ Anorexia, nausea, and emesis are all Grade 0-1.

⑩ Diarrhoeal Grade 0

6.3.3. Arm A (EP therapy): Dose reduction criteria

If any of the following toxicities are identified during the course, dose reduction should be performed in accordance with the dose reduction criteria (Table 6.3.3.) from the following course (no dose reduction in the course).

However, even if two or more items are met, the dose reduction for each drug is only one step. Re-escalation after dose reduction is not performed. Protocol treatment is terminated if the dose reduction criteria are met again after the dose reduction to Level-2.

Table 6.3.3. Arm A (EP therapy): Dose reduction criteria

Item	Etoposide	Cisplatin
Neutrophil count Grade 4 (<500 per mm^3).	Reduce the level by 1	No change
Platelet count Grade 4 (<2.5×10^4/mm^3)	Reduce the level by 1	No change
1.5 <serum creatinine ≦2.0 mg/dL.	No change	Reduce the level by 1
Serum creatinine <= >2.0 mg/dL	Termination of protocol treatment	Termination of protocol treatment
Grade 3<br>Infected ※1	Reduce the level by 1	Reduce the level by 1
Grade 3<br>Assessment at onset of febrile.	Reduce the level by 1	Reduce the level by 1
Grade 2<br>Peripheral sensory neuropathy<br>Peripheral motor neuropathy,<br>Myalgia; arthralgia; tinnitus; hearing impairment	No change	Reduce the level by 1
Grade 3<br>Peripheral sensory neuropathy<br>Peripheral motor neuropathy,<br>Myalgia; arthralgia; tinnitus; hearing impairment	Termination of protocol treatment	Termination of protocol treatment
Non-hematologic toxicities of Grade 3 other than those listed above in ※2 that are causally related to EP-therapy (excluding hyponatremia, hypokalemia, hypomagnesemia, hypocalcemia, hyperglycemia, and weight loss)	Reduce the level by 1	Reduce the level by 1

※1:※1 of infection: bronchial infection, pulmonary infection, upper respiratory tract infection, mediastinal infection, pleural infection, catheter-related infection,
Biliary tract infection; Gallbladder infection; Urinary tract infection

※2:Causal relationship is judged as either of possible, probable, definite

6.3.4. Arm A (EP therapy): Within-course pause, skipping criteria

Following initiation of treatment with each course after the course initiation criteria are met, if any of the following adverse events are observed, day 2, day 3 etoposide will be suspended.

- Fever (axillary temperature) Grade 1-3
- Grade 3 of infection (bronchial infection, pulmonary infection, upper respiratory tract infection, mediastinal infection, pleural infection, catheter-related infection,
  Biliary tract infection, gallbladder infection, urinary tract infection)

Resting etoposide should be resumed after confirming that all initiation criteria are met until day 7. However, it should not be administered after day 8.

That is, if etoposide could not be administered by day 7, the remaining etoposide should be skipped.

If the above suspension and/or skip occur, the next course of etoposide should be started day 22 (after 3 weeks) counting from day 1 of the previous course if the ininiation criteria are met.

6.3.5. Arm B (IP therapy): Dose-level

1) Irinotecan

Drug	Dose level	Dosing schedule	Dose day
Irinotecan	Level 0	60 mg/m^2 div	Day 1、8、15
	Level -1	50 mg/m^2 div	Day 1、8、15
	Level -2	40 mg/m^2 div	Day 1、8、15

2) Cisplatin

Drug	Dose level	Dosing schedule	Dose day
Cisplatin	Level 0	60 mg/m^2 div	Day 1
	Level -1	50 mg/m^2 div	Day 1
	Level -2	40 mg/m^2 div	Day 1

6.3.6. Arm B (IP therapy): Course initiation criteria

- On the day of the initiation of the course or the day before the expected start of the course, start the course after confirming that all of the following "Course Initiation Criteria" are met.
- If any one is not met, the initiation of the course is delayed.
- If the course cannot be initiated within 21 days of the expected start date of the course (if the start date of the previous course was day 1 and the course could not be initiated by day 49), discontinue the protocol treatment.
- However, if day 15 irinotecan is skipped in the previous course, day 22 of the previous course is set as the scheduled start date of the next course regarded as one course per 3 weeks.
- The course initiation criteria is not applied at the start of the first course.

Course initiation criteria

① Neutrophil count Grade 0-1 (≧1500/mm^3).
② Platelet count ≧10×10^4 /mm^3
③ AST≦100 IU/L (≦200 IU/L for liver metastases versus liver NECs)).
④ ALT≦100 IU/L (≦200 IU/L for liver metastases vs liver NECs).
⑤ Total bilirubin <= 2.0 mg/dL
⑥ Serum creatinine <= 1.5 mg/dL
⑦ Fever Grade 0 (measured by axillary temperature, temperature <38°C without antipyretic use)
⑧ Constipation, fatigue, phlebitis, oral mucositis, and infection ※1 are all Grade 2 or less.
Infected ※1: bronchial infection, pulmonary infection, upper respiratory tract infection, mediastinal infection, pleural infection, catheter-related infection, biliary tract infection, gallbladder infection, urinary tract infection
⑨ Anorexia, nausea, and emesis are all Grade 0-1.
⑩ Diarrhoea Grade 0

6.3.7. Arm B (IP-therapy): Dosing criteria for day 8, day 15

After confirming that all of the following ①-③ are met, the second (day 8) or third (day 15) dose of irinotecan is administered. If day 8, day 15 dosing criteria are not met, skip day 8, day 15 dosing.

① All of the following are met with the most recent laboratory data on the scheduled day of administration or the day before the scheduled day of administration.
 i) Neutrophil count Grade 0-2 (≧1000 per mm^3).
 ii) Platelet count ≧10×10^4 /mm^3
 iii) AST ≦ 100 IU/L (≦200 IU/L for liver metastases vs liver NECs).
 iv) ALT ≦ 100 IU/L (≦200 IU/L in the presence of hepatic metastases versus hepatic NECs).
 v) Total bilirubin <= 2.0 mg/dL
 vi) Serum creatinine <= 2.0 mg/dL
② All of the following are met on the scheduled day of administration:
 i) Fever Grade 0 (measured by axillary temperature, temperature <38°C without antipyretic use)

ii） Diarrhoeal Grade 0

③ Constipation, anorexia, nausea, emesis, fatigue, phlebitis, oral mucositis, and infection ※1 are all Grade 2 or less.

Infected ※1: bronchial infection, pulmonary infection, upper respiratory tract infection, mediastinal infection, pleural infection, catheter-related infection, biliary tract infection, gallbladder infection, urinary tract infection

6.3.8. Arm B (IP therapy): Dose reduction criteria

If any of the following toxicities are identified during the course, dose reduction should be performed in accordance with the dose reduction criteria (Table 6.3.8.) from the following course (no dose reduction in the course).

However, even if two or more items are met, the dose reduction for each drug is only one step. Re-escalation after dose reduction is not performed. Protocol treatment is terminated if the dose reduction criteria are met again after the dose reduction to Level-2.

Table 6.3.8. Arm B (IP therapy): Dose reduction criteria

**Item**	**Irinotecan**	**Cisplatin**
Neutrophil count Grade 4 (<500 per mm^3).	Reduce the level by 1	No change
Platelet count Grade 4 (< 2.5×10^4/mm^3)	Reduce the level by 1	No change
1.5 <serum creatinine ≦2.0 mg/dL.	No change	Reduce the level by 1
Serum creatinine <= >2.0 mg/dL	Termination of protocol treatment	Termination of protocol treatment
Grade 3<br>Infected ※1	Reduce the level by 1	Reduce the level by 1
Grade 3<br>Assessment at onset of febrile.	Reduce the level by 1	Reduce the level by 1
Grade2<br>Peripheral sensory neuropathy<br>Peripheral motor neuropathy,<br>Myalgia; arthralgia; tinnitus; hearing impairment	No change	Reduce the level by 1
Grade 3<br>Peripheral sensory neuropathy<br>Peripheral motor neuropathy,<br>Myalgia; arthralgia; tinnitus; hearing impairment	Termination of protocol treatment	Termination of protocol treatment
Non-hematologic toxicities of Grade 3 other than those listed above in ※2 that are causally related to IP-therapy (excluding hyponatremia, hypokalemia, hypomagnesemia, hypocalcemia, hyperglycemia, and weight loss)	Reduce the level by 1	Reduce the level by 1

※1:※1 of infection: bronchial infection, pulmonary infection, upper respiratory tract infection, mediastinal infection, pleural infection, catheter-related infection,

Biliary tract infection; Gallbladder infection; Urinary tract infection

※2:Causal relationship is judged as either of possible, probable, definite

6.3.9. Surgical of after end of chemotherapy

1) Surgery for Adverse Events Associated with Pathogenesis

Surgery may be performed if it is judged clinically desirable to perform surgery for newly appearing symptoms such as hemorrhage or stenosis. Protocol treatment is discontinued if surgery is performed, regardless of the content. In this case, the date of discontinuation of protocol treatment is the date of surgery.

2) Surgery for patients with complete response to chemotherapy

- When chemotherapy is highly effective, all metastases present at the time of enrollment on imaging studies disappear, and curative resection (R0 resection) is considered possible, resection including primary and metastatic disease may be performed.
- Surgical procedures are not specifically specified, but the Research Office will collect information on the details of the surgery performed individually in order to be reviewed by the research representative/research office.

- In all surgical cases, the group group conference shall confirm the validity of the judgment that surgery is indicated.
- When surgery is performed, protocol treatment is discontinued, regardless of whether curative resection was performed or not. In this case, the date of discontinuation of protocol treatment is the date of surgery.

6.3.10. Consultation on treatment modification

If there are any questions about treatment modification, contact "16.6. Study Coordinator".

Study Coordinator Contact : Chigusa Morizane

Hepato-Biliary Pancreatic Group: Futomi Mori (Main Research Secretariat)
Department of Hepatobiliary and Pancreatic Medicine, National Cancer Center
TEL:03-3542-2511
FAX:03-3542-3815
E-mail:cmorizan@ncc.go.jp

Gastric Cancer Group: Yoshinori Machida
Department of Gastroenterology, Kanagawa Cancer Center (Gastrointestinal Tract)
TEL:045-520-2222
FAX:045-520-2202
E-mail:n-machida@kcch.jp

Esophageal Cancer Group: Ken Kato/Yushi Homma
Department of Head and Neck, Esphageal Medical Oncology, National Cancer Center
TEL:03-3542-2511
FAX:03-3542-3815
E-mail: kenkato@ncc.go.jp / yohonma@ncc.go.jp

6.4. Concomitant treatment and supportive care

6.4.1. Required concomitant treatment/supportive care

1) Testing and Supportive Care for HBsAg-Positive Cases.

In HBsAg-positive cases, steroids and chemotherapy can lead to rapid hepatitis B virus (HBV) expansion (reactivation: reactivation) and potentially fatal severe hepatitis. Therefore, the following tests and supportive care are performed based on the "Guidelines for the Treatment of Hepatitis B, Third Edition (Japanese Society of Hepatology)." It is advisable to consult a hepatologist at the time prior to initiation of a nucleic acid analogue (entecavir, tenofovir disoproxil fumarate, and tenofovir alafenamide fumarate).

①Testing prior to initiation of chemotherapy: HBV-DNA quantitation

HBV-DNA quantitative analysis should be performed at least once prior to initiation of chemotherapy.

HBV-DNA assays are performed by real-time PCRs.

HBeAg and HBe antibodies should also be measured in accordance with the Guidelines for the Treatment of Hepatitis B, Third Edition (Japanese Society of Hepatology).

②Dosing schedule for supportive care (nucleic acid analogues prophylaxis)

- **Drugs used :**
  - **Entecavir (Bristol-Myers: Baraclude Tablets 0.5 mg)**
  - **Tenofovir disoproxil fumarate (GlaxoSmithKline: Tenozet Tablets 300 mg)**
  - **Tenofovir alafenamide fumarate (Giliado: Vemuridi Tablets 25 mg)**

The following dosage regimen should be followed, starting at least 1 week before the start of chemotherapy (as soon as possible), and continuing for at least 12 months after the end of chemotherapy. However, fulminant hepatitis has been reported in HBsAg-positive patients with high viral load, even during NA prophylaxis, and it is desirable to reduce the viral load before starting immunosuppression/chemotherapy. Nucleic acid analogues may be discontinued after 12 months after completion of chemotherapy if conditions *1 and 2 for discontinuation of NAs are met. However, if the administration of a nucleic acid analogue is discontinued, consultation with a hepatologist is always obtained, and the administration is discontinued only if the hepatologist deems it appropriate.

※1 Requirements for discontinuation of nucleic acid analogues (entecavir, tenofovir disoproxil fumarate, tenofovir alafenamide fumarate): all of the following

1. The patient has been on NA for more than 2 years.
2. HBV-DNA assays are not sensitive to detect
3. Be negative for HBeAg

※2 Patient background requirements: All of the following

1. Both the treating physician and the patient have sufficiently understood that the hepatitis exacerbation is frequently observed after the nucleic acid analog withdrawal, and that there is a danger of becoming serious in the time.
2. Follow-up is possible after treatment cessation, and appropriate treatment is possible even if hepatitis recurs
3. It is judged that the liver fibrillation is slight and the hepatic reserve is good, and it is difficult to become serious even if the hepatitis is exacerbated.

(Adapted from Guidelines for the Treatment of Hepatitis B, the 3rd edition (Japanese Society of Hepatology))

Entecavir

- **Dosage regimen: Take this medicine on an empty stomach (2 hours after meals and more than 2 hours before the next meal).**
- **Dosage :**

Creatinine clearance (mL/min).	Dosage
50 or more	0.5 mg once daily
30 Beyond 50	0.5 mg once every 2 days
10 Beyond 30	0.5 mg once every 3 days
10 Less than	0.5 mg once every 7 days

- **Adverse drug reactions (incidence of all grades): nucleoside analog-naïve patients**

Diarrhea (6.0%), nausea (4.5%), constipation (3.7%), upper abdominal pain (3.0%), malaise (1.5%), nasopharyngitis (3.0%), muscle stiffness (2.2%), headache (3.0%), rash (incidence unknown), laboratory tests: elevated AST (3.7%), increased blood bilirubin (6.0%), increased blood amylase (10.4%), and increased lipase

(10.4%).Blood glucose increased (6.0%), blood lactate increased (6.7%), urine occult blood positive (4.5%), white blood cell count decreased (8.2%), and eosinophil count increased (0.7%). [Significant adverse reactions (incidence unknown)] Hepatitis worsened after completion of treatment, anaphylactoid symptoms, lactic acidosis, and severe hepatomegaly due to fatty liver

Tenofovir disoproxil fumarate

- **Dosage and administration: 300 mg is orally administered once daily.**
- **Dosage :**

Creatinine clearance (mL/min).	Dosage
50 or more	300 mg once daily
30 Beyond 50	300 mg once every 2 days
10 Beyond 30	300 mg once every 3 to 4 days
Hemodialysis	[注)] of 300 mg once every 7 days<br>Or 300 mg after completion of cumulative approximately 12 hours of dialysis<br>NOTE) After hemodialysis was performed. The pharmacokinetics in patients with creatinine clearance < 10 mL/min and not on hemodialysis have not been investigated.

- **Dosing Precautions:**

In the long-term administration of tenofovir disoproxil fumarate, attention should be paid to renal dysfunction, hypophosphatemia (including Fanconi syndrome), and decrease in bone mineral density. It is recommended that renal function and serum phosphorus should be measured regularly during tenofovir disoproxil fumarate administration.

- **Adverse reactions (incidence of all grades):**

Abnormal liver function tests (AST, ALT and γ-GTP increased, etc.) in 7 patients (4.9%), increased creatinine in 4 patients (2.8%), increased amylase, increased lipase and nausea in 3 patients each (2.1%), abdominal pain in 2 patients (1.4%), [major adverse reactions (incidence unknown)] renal dysfunction, renal failure, acute renal failure, proximal renal tubular dysfunction, Fanconi syndrome, severe renal dysfunction such as acute renal tubular necrosis, nephrogenic diabetes insipidus or nephritis, severe hepatomegaly due to lactic acidosis and fatty deposition (steatohepatitis), pancreatitis

Tenofovir alafenamide fumarate

- **Dosage and administration: 25 mg is orally administered once daily.**
- **Dosage :**

Creatinine clearance (mL/min).	Dosage
15 or more	25 mg once daily
Less than 15	Consider discontinuation

- **Dosing Precautions:**

In the long-term administration of tenofovir alafenamide fumarate, attention should be paid to renal dysfunction, hypophosphatemia (including Fanconi syndrome), and decrease in bone density. It is recommended that renal function and serum phosphorus should be measured periodically during tenofovir alafenamide fumarate administration.

- **Adverse reactions (incidence of all grades):**

Nausea and abdominal distension, headache, fatigue (≧1%), dyspepsia and diarrhea, flatus, upper abdominal pain, constipation, ALT increased, arthralgia, dizziness, insomnia, pruritus, rash (≧0.5% to <1%), [significant adverse reactions (incidence unknown)] renal dysfunction, renal failure, acute renal failure, proximal renal tubular dysfunction, severe renal impairment such as Fanconi syndrome, acute renal tubular necrosis, renal diabetes insipidus or nephritis, severe hepatomegaly due to lactic acidosis and fatty deposits (fatty liver)

③Monitoring: Quantitative analysis of HBV-DNA (during and after administration of nucleic acid analogues)

During nucleic acid analogue administration:

They are monitored every 4 weeks by both HBV-DNA quantitative analysis and liver function (ASTs, ALTs). However, if HBV-DNA level is less than 20 IU/mL (1.3 log IU/mL) during administration of nucleic acid analogues, it is acceptable to perform tests every 4 to 12 weeks.

After discontinuation of nucleic acid analogue administration:

Bearing in mind that reactivation may occur even after discontinuation of administration of a nucleic acid analogues, the patient should be consulted with a hepatologist, and the patient should be monitored for HBV-DNA determination and hepatic function (AST/ALT) every 4 weeks for at least 1 year after discontinuation of administration of a nucleic acid analogues. Subsequent follow-up will be decided after consulting a hepatologist.

2) Laboratory Teste and Supportive Care for HBsAg-Negative and HBc Antibody-Positive and/or HBs Antibody-Positive Cases.

HBV-DNA quantitative analysis should be performed at least once prior to initiation of chemotherapy. HBV-DNA assays are performed by real-time PCRs.

i) HBV-DNA ≧ 20 IU/mL (1. 3 log IU/mL) prior to initiation of chemotherapy

It has been clarified that HBV-DNA replicates persist at low levels in the livers and peripheral blood mononuclear cells when HBc or HBs are positive, even if they are HBs-Ag negative. It has been reported that reactivation of HBV and development of severe hepatitis are caused by the use of potent immunosuppressive agents even in such patients with previous infections.

If HBV-DNA ≧ 20 IU/mL (1.3 log IU/mL), the risk of HBV reactivation is judged to be as high as in HBsAg-positive cases, and prophylactic administration of nucleic acid analogues (entecavir or tenofovir disoproxil fumarate, tenofovir alafenamide fumarate) is administered. The following laboratory tests and supportive care are performed in accordance with the "Guideline for the Treatment of Hepatitis B, 3rd edition (Japanese Society of Hepatology)" with reference to the following for examination, dosage, and monitoring of supportive care before the start of chemotherapy.

However, these are not applicable if the HBs antibody alone is positive and the HBV vaccination history is obvious.

①Dosing schedule for supportive care (nucleic acid analogues prophylaxis)

According to the dosage and administration of nucleic acid analogues (entecavir, tenofovir disoproxil fumarate, tenofovir alafenamide fumarate) in "1) Test and supportive care for HBsAg positive cases". Same conditions for NA discontinuation.

② Monitoring: Quantitative analysis of HBV-DNA (during and after administration of nucleic acid analogues)

The intervals for monitoring during and after discontinuation of NA are in accordance with the provision of "1) Testing and supportive care for HBsAg-positive patients".

ii) HBV-DNA less than 20 IU/mL (1.3 log IU/mL) prior to initiation of chemotherapy

HBV-DNA quantitative analysis and hepatic function (AST, ALT) will be monitored, and nucleic acid analogues (entecavir, tenofovir disoproxil fumarate, tenofovir alafenamide fumarate) will be started when ≧20 IU/mL (1.3 log IU/mL) is achieved.

The Guidelines for the Treatment of Hepatitis B, the 3rd edition (Japanese Society of Hepatology) recommends monitoring with HBV-DNA quantitative analysis or high-sensitivity HBs antibodies during and after chemotherapy, depending on the risks of revitalization.

①Monitor:HBV-DNA quantitative analysis

HBV-DNA quantitative analysis should be performed every 4-12 weeks from the start of chemotherapy until at least 12 months after the end of chemotherapy.

If HBV-DNA level is more than 20 IU/mL (1.3 log IU/mL), administration of nucleic acid analogues should be started immediately in accordance with the Guidelines for the Treatment of Hepatitis B, the 3rd edition (Japanese Society of Hepatology). If HBsAg monitoring is positive for < 1 IU/mL (low positive), nucleic acid analogues should be administered after additional HBV DNA determinations of ≧ 20 IU/mL (1.3 log IU/mL).

It is advisable to consult a hepatologist at a time prior to initiation of NAs.

②Supportive care in reactivation

Nucleic acid analogues should be administered according to the supportive care described in i) When HBV-DNA prior to the initiation of chemotherapy is 20 IU/mL (1.3 log IU/mL) or more in 6.4.1.2). Once administration of nucleic acid analogues is started, nucleic acid analogues should be discontinued only if appropriate by the hepatologist.

6.4.2. Recommended/not recommended concomitant treatment/supportive care

The following concomitant treatment and supportive care are recommended. Even if it is not carried out, it is not regarded as protocol deviation,

1) Addressing Febrile Neutropenia.

① Assessment at onset of febrile neutropenia (FN).

a) If the count is less than 500 per mm^3, or is less than 1000 per mm^3 and is expected to be less than 500 per mm^3 within 48 hours, and if the axillary temperature is 37.5°C or higher (mouth temperature is 38.0°C or higher), a severity-risk assessment should be performed promptly and anti-virus treatment initiated as appropriate.

b) Severity risk assessment is performed with reference to Multinational Association for Supportive Care in Cancer(MASCC) scoring system ※1.

c) For initial evaluation, complete blood count including differential WHITE BLOOD CELL and platelet count, renal function (BUN, creatinine), electrolytes, liver function (transaminases, total bilirubin, alkaline phosphatase) tests, ≧2 sets of venous blood cultures prior to initiation of antimicrobials, one set of cultures from the catheter lumen and one set from the peripheral vein if a central venous catheter is in place, cultures of suspected infection areas, and chest x-rays if respiratory symptoms and signs are present.

d) When febrile neutropenia (FN) develops in a patient with a central venous catheter, blood cultures from the catheter and peripheral blood are performed, and catheter-related infections are considered if there is a time difference of more than 120 minutes in the positivity of both. If appropriate antimicrobial therapy does not improve after more than 72 hours, catheter should be removed. For infections caused by Staphylococcus aureus, Pseudomonas aeruginosa, Bacillus, fungi, and acid-fast bacilli, the catheter should be removed and appropriate antimicrobial therapy based on culture results should be performed.

② Antibiotic use

a) In high-risk patients, β-lactams with anti-Pseudomonas aeruginosa activity are administered intravenously as a single agent. However, other antimicrobials (aminoglycosides, fluoroquinolones, and/or vancomycin) may be added to a single agent in the initial regimen in patients with unstable or complicated conditions or when drug-resistant organisms are strongly suspected. Low-risk patients may be treated with antibiotics orally or intravenously, hospitalized, or with adequate evaluation, if appropriate, as outpatients.

b) Re-evaluation will be performed 3-4 days after initiation of antibiotics to investigate the continuation or change of antibiotics. In principle, antibiotics will be continued until the neutrophil count recovers to 500 cells per mm^3 or more.

c) Empiric antifungal therapy is recommended in high-risk patients who do not respond to 4-7 days of broad-spectrum antibiotics.

d) Fluoroquinolone prophylaxis is recommended in high-risk individuals with an expected neutrophil count of 100 cells per mm^3 or less lasting >7 days.

③ Therapeutic administration of G-CSF

For therapeutic administration of G-CSF during the incidence of FNs, refer to "6.4.5. 3) Therapeutic administration of G-CSF".

※1 Multinational Association for Supportive Care in Cancer (MASCC) scoring system.
(Adapted in part from the Practice Guideline for Febrile Neutropenia (FN) [Japanese Society of Medical Oncology]. *2)

Item	Score
Clinical manifestations (select one of the following * mark 3 sections)<br>*No symptoms<br>*Mild symptoms<br>*Moderate symptoms	 5<br>5<br>3
No decrease in blood pressure	5
No chronic obstructive pulmonary disease	4
Solid tumors, or hematopoietic tumors without a history of fungal infection	4
No dehydration symptoms	3
Patients with fever during outpatient management	3
Age < 60	2

The total score is up to 26 points. Twenty-one points or more are considered low risk and 20 points or less are considered high risk

※2 Since patients aged 20 years or older are subjects in this study, we deleted "Not applicable to patients younger than 16 years old" from the original edition of the Practice Guideline for Febrile Neutropenia (FN) [Japanese Society of Medical Oncology].

2) Nausea and vomiting

Regarding nausea and vomiting, antiemetics are positively administered according to Clinical Practice Guidelines for Antiemesis in Oncology [48], and fluid and electrolyte repletion are performed when oral intake is severely reduced.

3) Anorexia

If oral intake drops markedly, fluid and electrolyte supplements should be given as needed. Especially, in the cases with diabetes mellitus, the abnormality of blood sugar level and electrolyte is noticed.

4) Anemia, thrombocytopenia

If anaemia (haemoglobin < 8.0 g/dL) or thrombocytopenia (platelet count < $2\times10^4/mm^3$) is observed, blood should be transfused as appropriate at the discretion of the treating physician.

5) Diarrhea

Severe diarrhoea occasionally occurs with arm B (IP therapy arm) and is extremely dangerous when complicated by febrile neutropenia. Patients should be fully informed about toxicities and their management, and should be instructed to measure body temperature, especially when neutrophils counts are most decreasing, and to contact a physician or nurse immediately during fever and diarrhea. Nonsteroidal anti-inflammatory drugs may not cause fever to become overt, so unnecessary anti-inflammatory drugs are not given.

If irinotecan-induced diarrhea occurs, the following supportive measures are recommended:

High-dose loperamide hydrochloride therapy

① Loperamide hydrochloride was started after signs of diarrhea were observed.

② Initial dose of 4 mg followed by 2 mg/2 hours (4 mg/4 hours at night)

③ It is administered until watery stool does not appear for more than 12 hours.

④ Doses should not be given for more than 48 hours.

6) Precautions on the day of cisplatin administration

Aminoglycoside antibiotics, vancomycin, and nonsteroidal anti-inflammatory drugs are not administered on the day of cisplatin administration or are used with caution.

6.4.3. Acceptable concomitant treatment and supportive care

The following concomitant treatment and supportive care may be used as needed.

Concomitant use of drugs for the treatment of complications such as hypertension and diabetes mellitus and symptomatic drugs such as morphine may be performed, but this drug should be administered with caution when furosemide, piretanide and phenytoin are used. Oral antibiotics for febrile neutropenia prophylaxis may be given at the discretion of the investigator/sub-investigator. Bisphosphonate denosumab may be used in combination for bone metastases.

6.4.4. Unacceptable concomitant treatment and supportive care

None of the following treatments will be given during protocol treatment:

① Administration of anticancer drugs other than protocol treatment

② Radiation therapy

6.4.5. Granulocyte colony-stimulating factor (granulocyte-colony stimulating factor:G-CSF

※This study permits the use of G-CSF biogenerics (biosimilars).

1)※ of primary prophylaxis with G-CSF

※Primary prophylaxis: G-CSF administration before developing febrile neutropenia or prolonged neutropenia to prevent them during anticancer therapy.

Primary prophylaxis with G-CSF was not recommended at the beginning of the study in this study. However, since febrile neutropenia in group A was 21.4% in the late 2016 periodic monitoring report and 15.8% in the early 2017 periodic monitoring report (18.4% when the time of occurrence was April 2017 and SAE reports not reflected in the early 2017 periodic monitoring report were included), it was decided that the patient would meet at least the recommended grade B or higher (almost A) according to G-CSF Appropriate Use Guideline 2013 and JSMO Febrile Neutropenia (FN) Practice Guideline. Primary prophylactic administration of G-CSF was recommended in group A. However, it is not considered a protocol deviation even if it is not administered. Since it is practical to administer pegfilgrastim from the viewpoint of convenience, when the primary preventive administration of G-CSF is carried out in the actual medical field, the administration example of pegfilgrastim is shown below.

(Administration cases)

Group A: 3.6 mg of pegfilgrastim (genetical recombination) will be injected subcutaneously (once per chemotherapy course) between day 4(day 3's completion of etoposide treatment and day 7 at least 24 hours after the completion of etoposide treatment.

Since the safety of pegfilgrastim administered 14 days prior to the initiation of cancer chemotherapy and within 24 hours after completion of administration has not been established, the primary prophylactic administration of pegfilgrastim is not performed in group B where day 8 is administered the drug.

Table 6.4.5. Primary prophylactic administration of G-CSF should be performed according to the approved dosage and administration shown in the table below.

Drug	・・ Pegfilgrastim (arm A only)<br>・・ Filgrastim<br>・・ Naltograstim<br>・・ Lenograstim
Time of initiation	Twenty-four hours after completion of chemotherapy
Dosage Dosing regimen	・Pegfilgrastim (genetical recombination) at a dose of 3.6 mg subcutaneously once per chemotherapy course (group A only)<br>・Filgrastim: 50 µg per m^2 SC once daily or 100 µg per m^2 IV once daily<br>・Naltograstim: 1 µg/kg SC once daily or 2µg/kg IV once daily<br>・Lenograstim: 2 µg/kg SC once daily or 5µg/kg IV once daily
Timing of discontinuation (other than pegfilgrastim)	・If the neutrophil count reaches 5000 per mm^3 or more after the course, administration should be discontinued.<br>・If the neutrophil count recovers to ≧2000 cells per mm^3, there are no suspected infections, and the patient's safety is judged to be ensured by the responsiveness to the drug used, the drug should be discontinued or reduced.

2)Secondary prophylactic※ of G-CSF

※ Secondary prophylaxis: G-CSF prophylactic administration after once occurrence of febrile neutropenia or prolonged neutropenia to prevent febrile neutropenia or prolonged neutropenia from occurring again during anticancer therapy.

If febrile neutropenia occurs in the previous course, secondary prophylaxis with G-CSF after the subsequent course is recommended, even if dose reduction or schedule modification or antimicrobial therapy is considered to be associated with a lower risk of febrile neutropenia (group A). However, it is not considered a protocol deviation even if it is not administered. As it is practical to administer pegfilgrastim in terms of convenience when secondary prophylactic administration of G-CSF is carried out in real medical practice, the following examples of administration of pegfilgrastim are shown.

(Administration cases)

Group A: 3.6 mg of pegfilgrastim (genetical recombination) is injected subcutaneously (once per course of chemotherapy) between the time of completion of etoposide treatment of day 4(day 3 and day 7 of at least 24 hours.

However, the safety of pegfilgrastim administered 14 days prior to the start of cancer chemotherapy and 24 hours after the end of treatment has not been established, so secondary prophylactic pegfilgrastim administration is not performed in group B, where day 8 is given the drug.

Secondary prophylactic administration of G-CSF should be performed according to the approved dosage and administration shown in the table below.

Drug	・・ Pegfilgrastim (arm A only)<br>・・ Filgrastim<br>・・ Naltograstim<br>・・ Lenograstim
Time of initiation	・・ Pegfilgrastim (arm A only)<br>Twenty-four hours after completion of chemotherapy<br>・・ Filgrastim, nartograstim, and lenograstim<br>When neutrophil counts <1000 per mm^3 are observed
Dosage Dosing regimen	・Pegfilgrastim (genetical recombination) at a dose of 3.6 mg subcutaneously once per chemotherapy course (group A only)<br>・Filgrastim: 50 µg per m^2 SC once daily or 100 µg per m^2 IV once daily<br>・Naltograstim: 1 µg/kg SC once daily or 2µg/kg IV once daily<br>・Lenograstim: 2 µg/kg SC once daily or 5µg/kg IV once daily
Timing of discontinuation (other than pegfilgrastim)	・If the neutrophil count reaches 5000 per mm^3 or more after the course, administration should be discontinued.<br>・If the neutrophil count recovers to ≧2000 cells per mm^3, there are no suspected infections, and the patient's safety is judged to be ensured by the responsiveness to the drug used, the drug should be discontinued or reduced.

3) Therapeutic administration of G-CSF

Therapeutic administration of G-CSF should be performed according to the approved dosage and administration shown in the table below.

Time of initiation	・When the neutrophil count is less than 1000 per mm^3 and fever (in principle, > 38.0°C) is observed<br>・When neutrophil counts <500 per mm^3 are observed
Dosage Dosing regimen	・Filgrastim: 50 µg per m^2 SC once daily or 100 µg per m^2 IV once daily<br>・Naltograstim: 1 µg/kg SC once daily or 2µg/kg IV once daily<br>・Lenograstim: 2 µg/kg SC once daily or 5µg/kg IV once daily
Timing of discontinuation	・If the neutrophil count reaches 5000 per mm^3 or more after the course, administration should be discontinued.<br>・If the neutrophil count recovers to ≧2000 cells per mm^3, there are no suspected infections, and the patient's safety is judged to be ensured by the responsiveness to the drug used, the drug should be discontinued or reduced.

6.5. Post-study treatment

Treatment after discontinuation of protocol treatment and treatment after progression or recurrence after completion are not specified.

Treatment (cross over) with drugs included in treatment regimens in the unassigned groups may be used, but if the total dose of cisplatin is greater than 500 mg/m^2, careful attention should be given to accumulating toxicities such as peripheral sensory/motor neuropathy, hearing loss, and renal impairment. Patients should be carefully monitored with adequate risk explanation only if the benefits are apparently outweighed by the risks, such as those with persistent sensitivity to cisplatin and mild cumulative toxicity.

If primary analysis or interim analysis reveals the primary conclusions of the trial, the results of the study will be explained to patients enrolled in this study as needed, and the best treatment will be provided, taking into account the course of treatment of individual patients.

In addition, if the protocol treatment discontinuation criteria apply but clinically "protocol treatment continuation" is judged to be appropriate, consult the research office through the institutional research director or institutional coordinator rather than at the physician level as a general rule (except when time is not allowed). In agreement between the Research Secretariat and the Investigator/Institution Coordinator, decide whether to treat as a → after discontinuation of protocol treatment or to deviate and continue protocol treatment. The details of the consultation

with the Research Secretariat and the decision-making process should be provided in the comment column for the patient's end-of-treatment report and progress record. If continuing the protocol treatment with protocol deviation occurs frequently, the Study Coordinator should consider revising protocol treatment termination criteria using group meetings and group mailing lists, because it is considered that the protocol treatment termination criteria is clinically inappropriate in such situation.

7. Anticipated Adverse Events

7.1. Anticipated adverse reactions

Anticipated adverse reactions in this study are as follows:

7.1.1. Anticipated Adverse Drug Reactions with Drugs

Adverse drug reactions anticipated with protocol treatments and drugs used in protocol-specified tests are referred to the latest version of the drug package insert.

7.1.2. Anticipated adverse reactions in the standard treatment arm (arm A)

Table 7.1.2. Adverse events in the EP-therapy group at JCOG9511 (excerpt from the final analysis report)

Examination Items	Grade 0	Grade 1	Grade 2	Grade 3	Grade 4	Grade 3,4(%)	Grade4(%)	Total
White blood cells	2	5	30	35	5	51.9%	6.5%	77
Neutrophils	1	0	5	21	50	92.2%	64.9%	77
Hemoglobin	2	9	43	23	-	29.9%	-	77
Platelet	31	19	13	14	0	18.2%	0%	77
Total bilirubin	57	-	20	0	0	0%	0%	77
GOT	49	24	2	1	1	2.6%	1.3%	77
GPT	40	28	6	2	1	3.9%	1.3%	77
Creatinine	56	16	5	0	0	0%	0%	77
Oxygen tension	13	27	9	2	1	5.8%	1.9%	52
Nausea and vomiting	13	36	23	5	-	6.5%	-	77
Diarrhea	64	8	5	0	0	0%	0%	77
Oral cavity (stomatitis)	68	6	2	1	0	1.3%	0%	77
Infection	42	23	9	1	2	3.9%	2.6%	77
Hair loss (hair)	9	46	19	-	-	-	-	74
Fever (uninfected)	45	14	16	2	0	2.6%	0%	77
Perception (peripheral nerve)	66	10	1	0	-	0%	-	77
Rash	74	2	1	0	0	0%	0%	77

※: Use JCOG Toxicity Criteria.

7.1.3. Anticipated adverse reactions in the study treatment arm (Arm B)

Table 7.1.3. Adverse Events in the IP-Therapy Group in a JCOG9511 (Extracted from the Final Analysis Report)

Examination Items	Grade 0	Grade 1	Grade 2	Grade 3	Grade 4	Grade 3,4(%)	Grade 4(%)	Total
White blood cells	1	16	38	17	3	26.7%	4.0%	75
Neutrophils	1	8	17	30	19	65.3%	25.3%	75
Hemoglobin	7	10	38	20	-	26.7%	-	75
Platelet	56	6	9	1	3	5.3%	4.0%	75
Total bilirubin	59	-	16	0	0	0%	0%	75
GOT	40	30	5	0	0	0%	0%	75
GPT	35	30	7	3	0	4.0%	0%	75
Creatinine	56	15	4	0	0	0%	0%	75
Oxygen tension	13	20	5	1	1	5.0%	2.5	40
Nausea and vomiting	11	26	28	10	-	13.3%	-	75
Diarrhea	23	19	21	8	4	16.0%	5.3%	75
Oral cavity (stomatitis)	66	9	0	0	0	0%	0%	75

Infection	45	17	9	3	1	5.3%	1.3%	75
Hair loss (hair)	17	44	13	-	-	-	-	74
Fever (uninfected)	45	12	17	1	0	1.3%	0%	75
Perception (peripheral nerve)	71	4	0	0	-	0%	-	75
Rash	70	5	0	0	0	0%	0%	75

※: Use JCOG Toxicity Criteria.

Table 7.1.3. Adverse events in the IP-therapy group in b JCOG0509 (abstracted from the main analysis report)

Examination Items	Grade 0	Grade 1	Grade 2	Grade 3	Grade 4	Grade 3,4(%)	Grade 4(%)	Total
White blood cells	16	36	58	29	3	22.5	2.1	142
Neutrophils	6	10	43	51	32	58.5	22.5	142
Hemoglobin	20	24	65	24	9	23.2	6.3	142
Platelet	125	6	8	2	1	2.1	0.7	142
Total bilirubin	116	15	10	1	0	0.7	0	142
GOT	98	37	5	2	0	1.4	0	142
GPT	80	56	4	2	0	1.4	0	142
Creatinine	97	37	8	0	0	0	0	142
Anorexia	22	67	33	19	1	14.1	0.7	142
Nausea	30	67	36	9	0	6.3	0	142
Vomiting	89	38	10	5	0	3.5	0	142
Diarrhea	52	51	28	11	0	7.7	0	142
Mucositis (examination findings)-oral cavity	126	15	0	0	1	0.7	0.7	142
Hair loss	64	66	12	-	-	-	-	142
Onset of febrile neutropenia	127	-	-	14	1	10.6	0.7	142
Infection with Grade3-4 neutropenia -Bronchus	142	-	0	0	0	0	0	142
-Lung (pneumonia)	137	-	0	4	1	3.5	0.7	142
-Pharynx	142	-	0	0	0	0	0	142
-Upper respiratory tract-unclassifiable	141	-	1	0	0	0	0	142
-Bladder	142	-	0	0	0	0	0	142
-Kidney	142	-	0	0	0	0	0	142
-Urinary tract-subclassification impossible	142	-	0	0	0	0	0	142
Neuropathy: Sensory	127	13	2	0	0	0	0	142

Table 7.1.3.c Adverse events from a pilot trial of postoperative adjuvant chemotherapy with irinotecan plus cisplatin for high-grade neuroendocrine lung cancer (excerpt)

Examination Items	Grade 2	Grade 3	Grade 4	Grade 3,4(%)	Grade 4(%)	Total
White blood cells	17	7	0	17.5%	0%	40
Neutrophils	12	15	4	47.5%	10.0%	40
Hemoglobin	14	6	4	25.0%	10.0%	40
Platelet	2	0	0	0%	0%	40
Total bilirubin	0	0	0	0%	0%	40
GOT	0	0	0	0%	0%	40
GPT	1	0	0	0%	0%	40
Creatinine	0	0	0	0%	0%	40
Hyponatremia	0	5	0	12.5%	0%	40
Endotoxemia	3	1	0	2.5%	0%	40
Hypokalemia	0	4	0	10.0%	0%	40
Nausea	8	4	-	10.0%	-	40
Vomiting	4	2	0	5.0%	0%	40
Anorexia	2	0	-	0%	0%	40

Diarrhea	11	2	0	5.0%	0%	40
Fatigue	10	5	-	12.5%	0%	40
Infection	2	0	0	0%	0%	40

7.2. Anticipated Adverse Events Due to Pathogenesis

1） Esophageal primary

Esophageal bleeding; Esophageal pain; Esophageal stenosis; Esophageal obstruction; Esophageal perforation; Esophageal ulcer; Esophageal fistula; Hoarseness; Pharyngolaryngeal dysesthesia; Hypercalcemia of advanced disease; Tracheal stenosis; Tracheal obstruction; Esophageal anastomotic leakage; Recurrent laryngeal nerve palsy

2） Gastric primary

Gastric bleeding, upper gastrointestinal bleeding, gastric pain, gastrointestinal pain, back pain, nausea, vomiting, dyspepsia, gastroparesis, abdominal fullness, gastric stenosis, gastric obstruction, gastric perforation, duodenal bleeding, duodenal fistula, duodenal perforation, duodenal perforation, duodenal stenosis, gastrostomy, gastrointestinal fistula, gastrointestinal fistula, ileus, gastric anastomotic leak, gastrointestinal anastomotic leak

3） Small intestin/Colon and rectum

Bleeding from tumor; Ileus; Duodenal bleeding; Duodenal obstruction; Duodenal perforation; Duodenal stenosis; Small bowel obstruction; Small bowel stenosis; Small bowel ulcer; Small bowel perforation; Small bowel fistula; Vaginal fistula; Colonic fistula; Colonic stenosis; Colonic obstruction; Colonic perforation; Rectal stenosis; Rectal obstruction; Rectal fistula; Rectal perforation; Rectal fistula; Jejunal perforation; Jejunal perforation; Ileostomy; Ileovesical fistula; Intestinal fistula; Anal pain; Small bowel anastomotic leakage; Large bowel anastomotic leakage; Rectal anastomotic leakage; Pelvic infection

4） Pancreas, biliary tract, and liver

Body weight loss; Fever; Pancreatitis; Cholecystitis; Cholecystic obstruction; Gallbladder pain; Liver failure; Biliary tract infection; Hemorrhage from biliary tract; Duodenal hemorrhage; Duodenal fistula; Duodenal obstruction; Duodenal perforation; Duodenal stricture; Anastomotic ulcer; Biliary anastomosis leak; Pancreatic anastomosis leak; Anastomotic hemorrhage; Anastomotic stricture; Hemorrhage from tumor; Hemorrhage from portal vein stenosis/obstruction; Symptoms associated with portal vein stenosis/obstruction (Portal hypertension, Hepatic failure; Esophageal variceal hemorrhage; Gastritis; Ascites); Back pain; Abdominal pain

5） Anticipated Adverse Events Due to Metastasis

Liver failure, hepatic pain, fever, hepatic infection, respiratory failure, pulmonary infection, superior vena cava syndrome, abdominal distention, abdominal distention, ileus, esophageal obstruction, reverse smoking, gastric obstruction, duodenal obstruction, small bowel obstruction, colonic obstruction, rectal obstruction, rectal stenosis, anal bleeding, anorectal infection, nausea, diarrhea, constipation, ureteral obstruction, and urinary retention,urethral infection, urinary tract infection, bile duct obstruction, biliary obstruction, cholecystitis, biliary hemorrhage, gallbladder pain, pancreatitis, pain, narrowing of luminal organs near metastatic sites (tracheal/gastrointestinal tract, etc.), tracheal obstruction, bronchial stenosis, penetration with adjacent organs, perforation, hoarseness, pleural effusion, chest wall pain, pleural pain, atelectasis, hypercalcemia, disseminated intravascular coagulation, fracture, ataxia, cerebrovascular ischemia, intracranial hemorrhage,headache, dizziness, decreased level of consciousness, aphasia, seizures, spasticity

6） Paraneoplastic syndrome, etc.

(due to incompatible secretory syndrome) hyponatremia, (due to ectopic ACTH syndrome) personality changes, hypertension, hypokalemia, hyperglycemia, Lambert-Eaton myasthenic syndrome, subacute cerebellar degeneration associated with autoantibody production (including ataxia, dysarthria, and nystagmus of limbs), paraneoplastic encephalomyelitis, sensory neuropathy (including dementia, cranial nerve symptoms, dizziness, ataxia, autonomic ataxia, transverse paralysis, and sensory disturbance), thromboembolism, (due to gastrointestinal stenosis) aspiration, anaemia, tumor pain, acute renal failure, myositis, and pulmonary fibrosis

Complications associated with etiolation and gastrointestinal stent insertion are shown below.

- Percutaneous transhepatic cholangiodrainage (PTCD) procedures, including internal-external fistula tube placement:

  Pancreatitis, bile duct stricture, cholecystitis, gallbladder obstruction, gallbladder pain, liver failure, biliary

tract infection, liver infection, cholecystitis, septicemia, hemorrhage, PTCD tubing obstruction/deviation, peritoneal infection, pneumothorax, pleural pain, pleural effusion, intrapleural hemorrhage

- Biliary stenting:
  Pancreatitis, biliary stricture, cholecystitis, gallbladder obstruction, gallbladder pain, liver failure, biliary tract infection, liver infection, cholecystitis, sepsis, hemorrhage, peritoneum infection, pneumothorax, pleural pain, pleural effusion, intrapleural hemorrhage (in case of percutaneous placement), duodenal perforation, pneumonitis (in case of transendoscopic placement), stent obstruction/deviation, duodenal ulcer, and duodenal hemorrhage
- Choledochojejunostomy:
  Biliary tract infection; Pancreatitis; Cholecystitis; Liver infection; Sepsis
- Gastrointestinal stent insertion:
  Bleeding, perforation, pain, stent deviation, stent obstruction, ulceration, fever, sepsis, infection, diarrhea, constipation, urgency (colorectal), thyroid injury (esophagus), jugular arteriovenous injury (esophagus), and mediastinal abscess (esophagus).

7.3. Evaluation of Adverse Events/Reactions

The Common Terminology Criteria for Adverse Events v4.0 Japanese Translated JCOG Version (Japanese translation of NCI-Common Terminology Criteria for Adverse Events v4.0 (CTCAE v4.0)) (CTCAE v4.0-JCOG) will be used to assess adverse events/reactions. For CTCAE v4 0-JCOG in which Grade is defined by laboratory reference values at the institutional reference value, the "JCOG sharing reference range" will be used instead of the institutional reference value at each medical institution. For more information on JCOG sharing reference ranges, see JCOG website (see http://www.jcog.jp/doctor/tool/kijun.html)).

7.3.1. Grading of s adverse events

In grading of adverse events, each grading is closest to the definitions of Grade 0 4 (nearest match). Grading to a higher Grade when the definition of more than one Grade is comparable and when it is difficult to decide on either (highest grade).

Grading should also be given to Grade if specific actions are described, due to their clinical need. For example, patients may refuse oxygen inhalation or chest drainage, even when the patient's pleural effusion is increasing and oxygen inhalation or chest drainage is indicated. In such cases, grading is based on the medical judgment of what should have been done (what should be done) rather than on whether the treatment was actually given (what was actually done).

In the event of treatment-related deaths, original NCI-CTCAE states that the causative adverse event should be Grade 5, but the outcome of the serious adverse event is reported in the SAE report and reviewed in detail. Therefore, Grade 5 of the institutional physician's judgment is not likely to be changed, and whether or not the serious adverse event will result in death will be significantly affected by other factors than the event is not appropriate, so it is not appropriate to compare the frequency of the adverse event by Grade (%Grade 4 and%Grade 5, respectively) between treatment groups or between studies. Because of the poor significance of distinguishing between Grade 4 and Grade 5 in the tabulation, Grade 4 is not considered "Grade 5" in the recording form of this study. A discussion of the causal relationship between adverse events observed in treatment-related deaths and deaths should be included in the "Situation at Death" section of the treatment completion report form and follow-up form, and an urgent report should be made. For the adverse event items specified in "8.2. Testing and Assessment during Treatment" and "8.3. Testing and Endpoints after Treatment Completion" that are determined to be Grade 5 in the post-hoc review including the emergency report, Grade and the date of the first occurrence of the event should be included in the relevant record form (Treatment Course Record Form). If Grade 3 or greater is observed for any other adverse event, or if Grade 3/2/1 adverse event and treatment requires at least 24 hours of hospital stay or prolongation of hospital stay (see 10.1.1.3.)), the AE and Grade and the date of first occurrence should be included in the free form of the treatment course record.

Any Grade on the record form should be recorded in the medical record. Confirmed during site visit audit.

7.3.2. Determination of the causal relationship between adverse events and treatment

In determining the causal relationship between adverse events and treatment, patients are classified into 5 categories of "definite, probable, possible, unlikely, unrelated". Each "causality" is defined as "causality" when judged to be either "definite, probable, possible" and "no causality" when judged to be either "unlikely, unrelated" (see TABLE 7.3.2.

According to Grade of adverse events, serious adverse events requiring expedited reporting as specified in "10.1. Serious Adverse Events and Expedited Reporting" should be reported to the Research Secretariat in accordance with "10.2. Mandatory Reporting and Reporting Procedures of the Investigator".

Table 7.3.2. Criteria for a Causal Relationship Between Adverse Events and Treatment

	Determination	Approach to determination
Cause Fruit Concern Associate Ah Fistula	Definite	The AE is clearly related to the intervention<br>Adverse events are apparently caused/aggravated by protocol treatment and are unlikely to be due to exacerbation of the etiology or other factors (comorbidities, other medications/treatments, or incidents).
	Probable	The AE is likely related to the intervention<br>It is unlikely that the adverse event was caused/aggravated by progression of the underlying pathology or other factors (comorbidity, other drugs/treatments, incident) and is likely to be due to protocol treatment.
	Possible	The AE may be related to the intervention<br>It is plausible (plausible) that an adverse event is considered to have occurred/become more severe with protocol treatment, and unlikely to be due to exacerbation of the etiology or other factors (comorbidities, other medications/treatments, or incidents).
Cause Fruit Concern Associate In Pouch	Unlikely	The AE is doubtfully related to the intervention<br>It is considered plausible (plausible) that the adverse event is due to exacerbation of the pathogenic disease or other factors (comorbidity, other drugs/treatments, incident) rather than to the protocol treatment that it is caused/aggravated.
	Unrelated	The AE is clearly NOT related to the intervention<br>It is judged that the adverse event was caused/aggravated by aggravation of the pathogenic disease or other factors (comorbidity, other drugs/treatments, incident) and is unlikely to be caused by protocol treatment.

8. Examination and Evaluation

8.1. Baseline examination and evaluation before registration

8.1.1. Test conducted before registration (regardless of time before registration)

1) Histopathology (immunostaining requires chromogranin A and synaptophysin) (see Section 3.3)
2) HBs antigen; HBc antibody ※1; HBs antibody ※1, HBV-DNA※2

※1: For HBsAg positive, HBc and HBs antibodies are not required, and HBV-DNA, HBeAg, and HBe antibodies are measured.

※2: Positive results for at least one of the HBc and HBs antibodies also indicate HBV-DNA prior to initiation of therapy (see Section 6.4.1).

8.1.2. Test performed within 56 days before enrollment (liver primary (or unknown primary))

If the liver is primary (or of unknown primary), the following tests should be performed (all allowing for tests performed in other hospitals):

1) Upper gastrointestinal endoscope
2) Lower gastrointestinal endoscope
3) FDG-PET test
4) Otolaryngology (head and neck) examination
5) Urology consultation (male only)
6) Gynecologic exam (female only)

8.1.3. Tests performed within 28 days before enrollment

1) Contrast-enhanced CT※1 (slice thickness of 5 mm or less, if the patient is allergic to iodine), both contrast-enhanced MRI of the abdomen and plain computed tomography (CT) of the imaging range, which is considered to be indispensable below, are performed. All tests are not performed in other hospitals.

※The following areas are indispensable for each primary organ, and if there is another site suspected of metastasis, the radiographic extent is added accordingly.

Primary Organ	Essential radiographic area
Esophagus	Cervical, chest, or abdominal regions
Stomach, duodenum, small intestine, colon, appendix, rectum,<br>Extrahepatic bile duct, Vater ampulla, gallbladder, and pancreas	Chest, abdomen, and pelvis
Hepatic NEC<br>(liver primary or liver metastasis of unknown primary)	Neck, chest, abdomen, and pelvis

2) Endoscopic ※2

Primary Organ	Mandatory test ranges
Esophagus, stomach, duodenum, and ampulla of Vater	Upper gastrointestinal tract (no examination performed at another hospital)
Small intestine, extrahepatic bile ducts, gallbladder, and pancreas	Be not mandatory
Colon, appendix, and rectum	Lower gastrointestinal tract (no examination performed at another hospital)
Hepatic NEC<br>(liver primary or liver metastasis of unknown primary)	8.1.2. Refer to the test performed within 56 days before registration.

※2 Unnecessary if the primary lesion has been resected

3) 12-lead, resting electrocardiography

8.1.4. Tests performed within 14 days before enrollment

1) General condition: PS (ECOG), body weight
2) Physical findings
3) Peripheral blood count: white blood cell count, neutrophil count (ANC: rod + segmented karyocyte), hemoglobin, platelet count

4) Blood biochemistry: total protein, albumin, total bilirubin, AST (GOT), ALT (GPT), BUN, creatinine, LDH, ALP, sodium, potassium, calcium, magnesium, CRP, FBS (fasting blood glucose)
5) Creatinine clearance (CCr): CCr estimates by Cockcroft-Gault equation
   Cockcroft-Gault formula
   Male: Ccr = {(140-Age) × Body Weight (kg)}/{72 × Serum Creatinine Level (mg/dL)}
   Women: Ccr = 0.85 × {(140-Age) × Body Weight (kg)}/{72 × Serum Creatinine Level (mg/dL)}
6) Tumour markers: NSEs, ProGRP, CEAs, SCCs (esophagus primary only), CA19-9 (non-esophageal primary only)
7) Chest X-P (frontal) (substitutable if contrast-enhanced and plain chest CT is obtained)

8.2. Examination and evaluation during treatment

The following safety examination and evaluation are minimal in frequency: Performing examinations more frequently at the discretion of the treating physician is not prohibited.

However, the examination for efficacy evaluation should be performed at specified frequencies, unless progression is suspected, because dense frequency may lead to bias in the efficacy evaluation.

8.2.1. Safety endpoint assessed weekly (CTCAE v4 0 Japanese translation)

The following 1)-3) tests or evaluations should be performed at least weekly. In addition, all of the following items should be checked and evaluated on the scheduled date or the day before anticancer drug administration. However, at least weekly examinations or evaluations should be performed in the same manner until day 28 using the starting date of the last course as day 1, even when the treatment is completed or terminated.

1) Peripheral blood count: white blood cell count, neutrophil count (rod + segmented count), hemoglobin, and platelet count
2) Biochemical tests: albumin, total bilirubin, AST (GOT), ALT (GPT), creatinine, sodium, potassium, calcium, magnesium, CRP
3) Subjective and objective findings (described according to CTCAE v4.0JAPANESE TRANSLATION)
   - General disorders and administration site conditions: fever, fatigue
   - Ear and labyrinth disorders: Tinnitus, hearing impairment
   - Skin and subcutaneous tissue disorders: alopecia
   - Gastrointestinal disorders: Constipation, diarrhea, nausea, vomiting, oral mucositis
   - Metabolism and nutrition disorders: anorexia, dehydration
   - Nervous system disorders: dysgeusia, peripheral sensory neuropathy, peripheral motor neuropathy
   - Musculoskeletal and connective tissue disorders: myalgia, arthralgia
   - Infections and parasites: bronchial infection, pulmonary infection, upper respiratory tract infection, mediastinal infection, pleural infection, catheter-related infection, biliary tract infection, gallbladder infection, and urinary tract infection
   - Blood and lymphatic system disorders: Febrile Neutropenia
   - Vascular disorders: Phlebitis
   - Respiratory, thoracic and mediastinal disorders: dyspnea, hypoxia, pneumonitis

8.2.2. Safety examination and evaluation for each course

1) General condition: Body weight
2) Blood chemistry: LDH, ALP FBS (fasting glucose)

8.2.3. Safety examination and evaluation to be performed as necessary

1) When dyspnea is observed
   - Chest X-P, percutaneous oxygen saturation: SpO2, arterial blood gases: PaO_2
2) If an arrhythmia is observed
   - 12-lead, resting electrocardiography
3) When HBs antigen, HBs antibody, or HBc antibody is positive
   - HBV-DNA (see 6.4.1.).

8.2.4. Efficacy end point

The following tests will be performed every 6 weeks during protocol treatment (±1 week allowed: 6 ± 1 week, 12 ± 1 week, and 18 ± 1 week after the start date of protocol treatment). Tumor response will be assessed according to "11.1. Response Evaluation". Evaluation of the response will be performed using the same test conditions and test methods as the baseline evaluation.

If CT is allergic to contrast material, it is evaluated with plain CT and/or contrast-enhanced MRI. Allergy to contrast media on MRI is assessed by plain CT or plain MRI. When the use of CT contrast medium and MRI contrast medium becomes difficult due to renal dysfunction, it is evaluated by simple CT or simple MRI.

1) Tumour markers: NSEs, ProGRP, CEAs (≧all cases), SCCs (esophagus primary only), CA19-9 (non-esophageal primary only)
2) Contrast-enhanced CT: In principle, the extent of imaging is as follows. () Areas in can be omitted if there is no lesion

Primary Organ	Essential radiographic area
Esophagus	Cervical, chest, or abdominal regions
Stomach, duodenum, small intestine, colon, appendix, rectum,<br>Extrahepatic bile duct, Vater ampulla, gallbladder, and pancreas	(chest), abdomen, and pelvis
Liver NEC (liver primary or liver metastasis of unknown primary)	(neck), (chest), abdomen, and pelvis

8.3. Examination and evaluation after completion of treatment

8.3.1. Efficacy evaluation after completion of treatment

After completion of the protocol treatment, examination and evaluation are made at the following timing:
The following tests should be performed at least every 6 weeks until progressions are confirmed or death.
If progressions are observed, only observation of the outcome will be continued.

1) Tumour markers: NSEs, ProGRP, CEAs (≧all cases), SCCs (esophagus primary only), CA19-9 (non-esophageal primary only)
2) Contrast-enhanced CT: In principle, the extent of imaging is as follows. Areas in parentheses can be omitted if there is no lesion

Primary Organ	Essential radiographic area
Esophagus	Cervical, chest, or abdominal regions
Stomach, duodenum, small intestine, colon, appendix, rectum,<br>Extrahepatic bile duct, Vater ampulla, gallbladder, and pancreas	(chest), abdomen, and pelvis
Liver NEC (liver primary or liver metastasis of unknown primary)	(neck), (chest), abdomen, and pelvis

8.4. Information on post- study treatment

After completion/termination of protocol treatment, the following items will be recorded on the Follow-up Form at each follow-up survey:

1) Content of post-study treatment (if post-study treatment is performed)
2) After protocol treatment termination, the initiation date of the first post-study treatment ((if post-study treatment is performed)
3) PS at the start of post-study treatment

8.5. Study calendar

If the primary site is the esophagus, stomach, duodenum, small intestine, colon, appendix, rectum, extrahepatic bile duct, ampulla of Vater, gallbladder, or pancreas

	Before registratikon Record Pre	After end of chemotherapy		From the date of protocol treatment discontinuation Within 28 days	Until progression after completion of protocol treatment	
		Course Before initiation of the course	During the course		Only at the start of post-treatment	Every 6 weeks
Physical findings						
Body weight	○[14]	○				
PS	○[14]				■	
Physical findings	○[14]	○			■	
Laboratory tests						
WBC, differential (neutrophil) Hb, platelets	○[14]	○	●	●		
Alb, T-Bil, AST, ALT, Cr, Na, K, Ca, Mg, CRP	○[14]	○	●	●		
LDH, ALP, FBS	○[14]	○				
Total protein, BUN, Ca	○[14]					
NSE, ProGRP、CEA	○[14]		△			
SCC (esophagus primary only), CA19-9 (other than primary esophageal)	○[14]		△			
HBs antigen, HBc antibody, and HBs antibody	○[前]					
Chest X-P (can be substituted if CT is taken)	○[14]					
12-lead, resting electrocardiography	○[28]					
Upper gastrointestinal endoscope (Primary: esophagus, stomach, duodenum)	○[28]					
Lower gastrointestinal endoscope (Primary: colonic, appendix, rectum)	○[28]					
Efficacy evaluation						
Contrast-Enhanced CT*	○[28]		△			△
Toxicity evaluation						
Subjective symptom check		○	●	●		
Objective symptom check		○	●	●		
Submission of CRFs						
Pre-treatment Form		□				
Treatment Form			□			
Off-treatment Form				□		
Follow-up Form					2 times/year	

○[前]: Conduct before registration, ○[28]: Perform within 28 days before registration, ○[14]: Perform within 14 days before registration

○:Conduct, ♀: Implementation at least once a week

△: Every 6 weeks (see 8.2.4.), ∞: Only once at the beginning of aftertreatment, □: Submitted.

*See 8.1.3 for the shooting range. Contrast-induced allergy or renal dysfunction is assessed by plain CT or contrast-

enhanced MRI.

※Follow-up Forms will be sent up to 1 years after completion of accrual and will be submitted after 1 years of registration in the individual patient according to the closing date of registration.

For liver NEC (liver primary or liver metastasis of unknown primary)

	Before registratikon Record Pre	After end of chemotherapy		From the date of protocol treatment discontinuation Within 28 days	Until progression after completion of protocol treatment	
		Course Before initiation of the course	During the course		Only at the start of post-treatment	Every 6 weeks
Physical findings						
Body weight	○^{14}	○				
PS	○^{14}				■	
Physical findings	○^{14}	○			■	
Laboratory tests						
WBC, differential (neutrophil) Hb, platelets	○^{14}	○	●	●		
Alb, T-Bil, AST, ALT, Cr, Na,K, Ca, Mg, CRP	○^{14}	○	●	●		
LDH, ALP, FBS	○^{14}	○				
Total protein, BUN, Ca	○^{14}					
NSE, ProGRP、CEA、CA19-9	○^{14}		△			
HBs antigen, HBc antibody, and HBs antibody	○^{前}					
Chest X-P (can be substituted if CT is taken)	○^{14}					
12-lead, resting electrocardiography	○^{28}					
Upper gastrointestinal endoscope	○^{56}					
Lower gastrointestinal endoscope	○^{56}					
FDG-PET	○^{56}					
Otolaryngologic examination	○^{56}					
Urology consultation (male only)	○^{56}					
Gynecologic exam (female only)	○^{56}					
Efficacy evaluation						
Contrast-Enhanced CT*	○^{28}		△			△
Toxicity evaluation						
Subjective symptom check		○	●	●		
Objective symptom check		○	●	●		
Submission of CRFs						
Pre-treatment Form		□				
Treatment Form			□			
Off-treatment Form				□		
Follow-up Form					□ 2 times/year	

○^{前}: Conduct before registration, ○^{56}: Perform within 56 days before registration, ○^{28}: Perform within 28 days before registration,

○^{14}: Implemented within 14 days prior to enrollment

○:Conduct, ♀: Implementation at least once a week

△: Every 6 weeks (see 8.2.4.), ∞: Only once at the commencement of post-treatment, □: Submitted.

*See 8.1.3 for the shooting range. Contrast-induced allergy and renal dysfunction are evaluated by plain CT or contrast-enhanced MRI.

※Follow-up Forms will be sent up to 1 years after completion of accrual and will be submitted after 1 years of

registration in the individual patient according to the closing date of registration.

9. Data collection

9.1. Case Report Form (CRF）

9.1.1. Types of CRF and submission deadlines

The case report forms (CRF) used in this study and their submission deadlines are as follows:

1) Pre-treatment report (blue)- Less than 2 weeks after enrollment
2) Treatment course records- Every 2 cycles/less than 2 weeks after end of protocol treatment
   3）-1 Treatment (yellow)
   3）-2 Test (yellow)
   3）-3 Adverse events (yellow)
3) Tumor shrinkage report (green) - Less than 2 weeks after judgment of effect
4) End of treatment report (red) - Less than 2 weeks after discontinuation/end of protocol treatment
5) Follow-up investigation (white)- By the deadline indicated in the Follow-up Investigation Form

- For "1) Pre-treatment report to 4) End of treatment report", CRFs with basic patient information (enrollment code, facility name) pre-printed on them would be sent by post from the data center. If the CRFs do not arrive within one week of enrollment, or if the CRFs have been lost/damaged, the data center should be contacted by telephone, and a request should be made for them to be re-issued.
- "5) Follow-up investigation" would be sent by post from the data center at the time of follow-up investigations that are conducted at the same time as monitoring and interim/final analyses at the data center.

9.1.2. Storage CRF

- Completed CRFs must all be archived at the facility as photocopies or in electronic form.
- Copies of CRFs should be kept archived until the final analysis report is issued for reference while filling other CRFs, or for review while retrieving information from the data center.

9.1.3. Method of sending CRF

- All CRF must be sent by post or handed over in person at the data center. They must not be sent by FAX.
- To avoid the risk of personal patient information being leaked, the patient enrollment code should be used, instead of using the patient medical chart number at the facility, when contacting the data center for request of CRF dispatch.

9.1.4. Correction of the contents of CRFs

If any data necessary for the CRF are found to be missing or there are inappropriate category classifications after the start of the study, the CRFs may be corrected with the agreement of the head of the data center and the clinical trial secretariat, in a manner not exceeding the scope of data collected as prescribed in "8. Evaluation item/Clinical laboratory tests/Evaluation schedule", and within the scope deemed not to increase medical and financial burden on the enrolled patient from the CRF correction. Modification of CRFs that do not require the main body of the protocol to be revised is not considered a protocol revision by JCOG. Reports to the head of the medical institution related to CRF correction and the request of application for revision should follow the rules of the facility.

10. Reporting of "disease or the like"(adverse events)

Site investigator should report to Study Coordinator/Principal Investigator (Study Chair) if a serious adverse event ("disease or the like" on Clinical Trials Act) occurs in accordance with the regulations of Clinical Trials Act (Law No. 16, 2017), Enforcement Regulations of Clinical Trials Act (MHLW Notification No. 17, 2018) and the regulations in this chapter based on the relevant notifications.

The most recent version of the report is available on the MHLW website [1)] and on the JCOG website [2)]. Use the most recent version of the report.

Serious adverse events occurring after the initiation of protocol treatment (after the date of registration if death) by the date of final follow-up will be subjects.

1）http://www.mhlw.go.jp/stf/seisakunitsuite/bunya/0000163417.html
2）http://www.jcog.jp/doctor/todo/researcher/harmfulness.html
3）http://www.pmda.go.jp/safety/reports/hcp/pmd-act/0002.html

10.1. Serious Adverse Events and subjects of Expedited Reporting.

Serious adverse events are defined as any of the following:
(These are classified as "disease or the like" on Clinical Trials Act.)

1） Death
2） Diseases that may lead to death
3） "Disease or the like" requiring hospitalization or prolongation of hospital stay for treatment.
4） Disability
5） "Disease or the like" that may lead to disability
6） Serious "disease or the like" according to 1) to 5)
7） Congenital disease or abnormality in later generations

Of these, those that fall under any of the following categories shall be regarded as serious adverse events for which Expedited Reporting is mandatory.

1) Death

(i) All deaths that occur after registration and before the start of protocol treatment
(ii) All deaths (with or without causality to protocol treatment) that occur during protocol treatment or within 30 days of the last treatment day
(iii) Death that occur after 31 days from the last treatment date that are causally related to protocol treatment (definite, probable, possible)

2) "Disease or the like" that may lead to death

(i) Grade 4 adverse events that occur during protocol treatment or within 30 days of the last treatment day (excluding events in Table 10.1)
(ii) Grade 4 adverse events that occur after 31 days from the last treatment date (excluding events in Table 10.1) that are causally related to the protocol treatment (definite, probable, possible)

3) "Disease or the like" requiring hospitalization or prolongation of hospital stay[※1] for treatment

(i) Grade 3/2/1 adverse events that occur during or within 30 days of protocol treatment and requiring at least 24 hours of hospitalization or prolongation of hospital stay[※1] to treat the adverse event (excluding the event in Table 10.1).
(ii) Grade 3/2/1 adverse events that occur 31 days after the last treatment day and requires 24-hour or longer hospitalization or prolongation of hospital stay[※1] for treatment and causally related to protocol treatment (definite, probable, possible) (excluding the events in Table 10.1)

※ 1 "Hospitalization or prolongation of hospital stay" refers only to those for which hospitalization of at least 24 hours or prolongation of hospital stay is medically required for the treatment of an adverse event. The followings are not subjects for reporting:

- Hospitalization or prolongation of hospital stay performed for follow-up of adverse event that has disappeared or improved

- Hospitalization or prolongation of hospital stay for reducing patient burden, e.g. patients from distant areas.
- Hospitalization or prolongation of hospital stay for other medically unnecessary situation

※ 2 "Unexpected" refers to those not listed in "7. Expected Adverse Events"

4) Disability, 5) "Disease or the like" that may lead to disability

Permanent or marked disability/dysfunction (excluding myelodysplastic syndromes, secondary cancers, etc.) or possible medical situation

6) Serious "disease or the like" similar to 1) to 5)

7) Congenital disorders or abnormalities in later generations

Table 10.1. Adverse events excluded from the subjects of Expedited Reporting

SOC※(CTCAE ver4.0)	AE term
Blood and lymphocyte disorders	Anemia, bone marrow hypocellular
Gastrointestinal disorders	Constipation
General disorders and local symptoms	Fever
Infections and infestations	Viral hepatitis
Clinical laboratory test	ALP increased, CD4 lymphocytes decreased, high cholesterol, GGT increased, lipase increased, lymphocytes decreased, neutrophils decreased, platelet count decreased, serum amylase increased, WBC decreased, hyponatremia, hypokalemia, hyperglycemia, and hypoglycemia
Metabolism and nutritional disorders	Obesity, anorexia, hyperuricemia, and hypoalbuminemia
Musculoskeletal and connective tissue disorders	Fibrosis deep connective tissue and superficial soft tissue fibrosis
Renal and urinary disorders	Chronic kidney disease
Respiratory, thoracic, and mediastinal disorders	Sinus disorder and sleep apnea
Skin and subcutaneous tissue disorders	Hypohidrosis

※ SOC:System Organ Class

10.2. Investigator's reporting requirements and procedures

10.2.1. Expedited Reporting

In the event of a serious adverse event for which Expedited Reporting is mandatory, the Subinvestigator must promptly inform the Investigator. If the Investigator cannot be contacted, the Site Coordinator or Subinvestigator must take over the responsibility of the Investigator. The Investigator must report adverse events according to the following procedures.

Attention should be paid not to include the patient's name and medical record number when sent.

Serious adverse events that occur after the initiation of protocol treatment (after the date of registration if death) by the date of final follow-up are subjects of Expedited Reporting.

1) "Disease or the like" that may lead to death or death specified in 10.1 1) and 2).

Primary reporting:

The Subinvestigator who is aware of the occurrence of adverse events will promptly notify the Investigator. The Investigator who receives the notice should fill out JCOG Adverse Event Report Form(for institution) and the "Disease or the like" Report Form to Certified Review Board specified in Clinical Trials Act Enforcement Regulations as far as possible and contact Principal Investigator/Study Coordinator via e-mail within 72 hours of knowledge of the occurrence of the adverse event.

Secondary reporting:

The Investigator should add detailed information on adverse events to JCOG Adverse Event Report Form(for

institution) and the "Disease or the like" Report Form to Certified Review Board specified in Clinical Trials Act Enforcement Regulations within 7 days of knowledge of the occurrence of adverse events and send them to the Principal Investigator/Study Coordinator via e-mail. If necessary, attach copies of laboratory data, images, autopsy report, etc.

2) 10.1. 3)"Disease or the like" or other medically important condition requiring hospitalization or prolongation of hospital stay for treatment. Adverse events are judged to be either of 10.1. 4)-7)

The Subinvestigator who is aware of the occurrence of adverse events will promptly notify the Investigator. The Investigator who receives notice must fill out JCOG Adverse Event Report Form (for institution) and the "Disease or the like" Report Form to Certified Review Board specified in Clinical Trials Act Enforcement Regulations within 10 days of knowledge of the occurrence of an adverse event and send them to the Principal Investigator/Study Coordinator via e-mail. If necessary, attach copies of laboratory data, images, autopsy report, etc.

3) Additional reporting

If new information is obtained after conducting the above reporting, the Investigator must add information to JCOG Adverse Event Report Form (for institution) and the "Disease or the like" Report Form to Certified Review Board specified in Clinical Trials Act Enforcement Regulations and report it as needed.

Table 10.2.1. Summary of Adverse Events which are subjects for Expedited Reporting and the deadline of reporting to Principal Investigator/Study Coordinator

Causal relationship	The patient was hospitalized due to Grade 1-3 AE. Other medically important conditions		Grade 4		Death	
	Expected	Not expected	Expected	Not expected	Expected	Not expected
Present	No need of reporting	Primary reporting: within 10 days Additional reporting: as needed	Primary reporting: within 72 hours Secondary reporting: within 7 days Additional reporting: as needed			
None	<Only on-treatment or within 30 days of last protocol treatment day>					
	No need of reporting	Primary reporting: within 10 days Additional reporting: as needed	Primary reporting: within 72 hours Secondary reporting: within 7 days Additional reporting: as needed			

* 4) Disability, 5) "Disease or the like" that may lead to disability, and 6) "Disease or the like" that are serious similar to 1) to 5) in 10.1., 7) Congenital disorders or abnormalities in later generations

※ "Unexpected" refers to those not listed in "7. Expected Adverse Events"

Table 10.2.1. Summary of Adverse Events which are subjects for Expedited Reporting and the deadline of reporting to Principal Investigator/Study Coordinator

Causal relationship	The patient was hospitalized due to Grade 1-3 AE. *Other medically important conditions		Grade 4		Death	
	Expected	Not expected	Expected	Not expected	Expected	Not expected
Present	Primary reporting: within 10 days Additional reporting: as needed	Primary reporting: within 10 days Additional reporting: as needed	Primary reporting: within 72 hours Secondary reporting: within 7 days Additional reporting: as needed			
None	<Only on-treatment or within 30 days of last protocol treatment day>					
	Primary reporting: within 10 days Additional reporting: as needed	Primary reporting: within 10 days Additional reporting: as needed	Primary reporting: within 72 hours Secondary reporting: within 7 days Additional reporting: as needed			

* 4) Disability, 5) "Disease or the like" that may lead to disability, and 6) "Disease or the like" that are serious similar to 1) to 5) in 10.1., 7) Congenital disorders or abnormalities in later generations

※ "Unexpected" refers to those not listed in "7. Expected Adverse Events"

10.2.2. Reporting to the Administrator of participating medical organizations

If an Adverse Event which is subjects for Expedited Reporting occurs and is assessed as causal after reporting to Principal Investigator and reported to Certified Review Board, the Investigator must report it to the Administrator of the relevant medical institution in accordance with the requirements of the medical institution.

10.3. Responsibilities of Principal Investigator/Study Coordinator

10.3.1. Determination of necessity of suspension of registration and emergency notification to institutions

Principal Investigator/Study Coordinator who received the report from the Site Investigator should report to Group Chair and determine the urgency, significance, and impact of the report. If needed, take measures such as suspending registration (contacting JCOG Data Center and all participating institutions) and urgently communicating information to participating institutions. Telephone calls can be made to Data Center and institutions as urgent, but they should also be promptly contacted by document (e-mail).

10.3.2. Reporting to JCOG Operations Office and Certified Review Board and MHLW

1) Reporting from the Principal Investigator/Study Coordinator to JCOG Operations Office

Principal Investigator/Study Coordinator should consult with Group Chair and report to JCOG Operations Office (Safety Contact) by e-mail within 72 hours of knowledge of the occurrence of the adverse event, if reported AE is considered to meet the adverse events specified in 10.2.1. 1) and 2) In doing so, to the extent feasible, Principal Investigator/Study Coordinator should send "JCOG Adverse Event Report Form (for institution)" sent from the institution, "Disease or the like Report Form" addressed to Certified Review Board as stipulated in Clinical Trials Act Enforcement Regulations, and attach "JCOG Adverse Event Report (for Study Coordinator)" with Study Coordinator/Principal Investigator's view (including judgments of causality and expectation, and judgments of continuation/discontinuation of the study)". For the expected adverse events of 1) or 2) in 10.2.1., include a discussion not only of the individual patient's course but also of whether the frequency of appearance is within the expected range.

2) Reporting to Certified Review Board

JCOG Operations Office (Safety Contact) reviews the appropriateness of the judgement of causality and expectation of adverse events reported in the above procedures and can ask Principal Investigator/Study Coordinator to reconsider them if there is any doubt. Adverse events considered by Principal Investigator/Study Coordinator and JCOG Operations Office to be related to the protocol treatment and to be the subject of reporting in the following tables should reported to Certified Review Board through JCOG Operations Office.

If there is a disagreement between Principal Investigator/Study Coordinator and JCOG Operations Office, report it to Data and Safety Monitoring Committee and seek final judgment from the Chair of Data and Safety Monitoring Committee. However, if the reporting may exceed deadlines of reporting, the report can be tentatively reported as "causal" to Certified Review Board.

Reporting subjects and reporting deadline

Principal Investigator/Study Coordinator must report to Certified Review Board through JCOG Operations Office within the following time periods after knowledge of the occurrence of adverse events.

Causal relation ship	The patient was hospitalized due to Grade 1-3 AE. Other medically important condition		Grade 4		Death	
	Expected	Not expected	Expected	Not expected	Expected	Not expected
Present	No need of reporting	Within 15 days	Within 15 days	Within 7 days	Within 15 days	Within 7 days
None	No need of	No need of	No need of	No need of	No need of	No need of

	reporting	reporting	reporting	reporting	reporting	reporting

※ "Unexpected" refers to those not listed in "7. Expected Adverse Events"

Principal Investigator/Study Coordinator must report adverse events to Certified Review Board through JCOG Operations Office within the following deadlines after knowledge of the occurrence of adverse events.

Causal relation ship	The patient was hospitalized due to Grade 1-3 AE. Other medically important condition		Grade 4		Death	
	Expected	Not expected	Expected	Not expected	Expected	Not expected
Present	Within 30 days	Within 15 days	Within 30 days	Within 15 days	Within 15 days	Within 15 days
None	No need of reporting	No need of reporting	No need of reporting	No need of reporting	No need of reporting	No need of reporting

※ "Unexpected" refers to those not listed in "7. Expected Adverse Events"

3) Reporting to MHLW

Principal Investigator/Study Coordinator of the study with unapproved or off-label health care should report "Disease or the like" Report Form specified in Clinical Trials Act Enforcement Regulations to MHLW through JCOG Operations Office, if an adverse event is considered to be unexpected and to have a causal relationship to the protocol treatment (See 10.5. Responsibilities of the Data and Safety Monitoring Committee).

Reporting subjects and reporting deadlines

Principal Investigator/Study Coordinator should report to the MHLW through JCOG Operations Office within the following deadlines after knowledge of the occurrence of adverse events.

※ Pharmaceuticals and Medical Devices Agency Safety Division I (trk-shippeitouhokoku@pmda.go.jp)

Causal relation ship	The patient was hospitalized due to Grade 1-3 AE. Other medically important condition		Grade 4		Death	
	Expected	Not expected	Expected	Not expected	Expected	Not expected
Present	No need of reporting	Within 15 days	No need of reporting	Within 7 days	No need of reporting	Within 7 days
None	No need of reporting	No need of reporting	No need of reporting	No need of reporting	No need of reporting	No need of reporting

※ "Unexpected" refers to those not listed in "7. Expected Adverse Events"

4) Additional reporting

Following receipt of secondary or additional reports from the Investigator, Principal Investigator/Study Coordinator must add additional information from the primary report and their views to JCOG Adverse Event Report Form (for the institution) and the "Disease or the like" Report Form to Certified Review Board set out in Clinical Trials Act Enforcement Regulations, and promptly contact JCOG Operations Office (Safety Contact) by e-mail. If the report was sent to Certified Review Board and the MHLW in the primary reporting,, the secondary reporting and additional reporting must be made in the same manner.

10.3.3. Notification to the Site Investigators

When reported to Certified Review Board, Principal Investigator/Study Coordinator should inform the Investigators of all participating institutions of the review results and recommendations by documents (e-mail is allowed). Principal Investigator/Study Coordinator must inform the Investigators without waiting for Certified Review Board review if there is any urgent information to be disseminated. In the event that reported to the MHLW, Principal Investigator/Study Coordinator should notify the Investigators of all participating institutions.

In addition, even if no reporting is made to Certified Review Board, Principal Investigator/Study Coordinator must inform the Investigator of the reporting institution of the decision of Principal Investigator/Study Coordinator by documents (e-mail is allowed).

10.3.4. Assessment of Adverse Events in Periodic Monitoring

During Periodic Monitoring, Principal investigator/Study Coordinator should carefully review the adverse events in the Monitoring Reports issued by the Data Center and ensure that there are no missed reporting from the participating institutions. It should also be confirmed that all reported adverse events are listed in the Monitoring Reports. The presence or absence of a missed reporting should be indicated in the column of the results of Group review on the Periodic Monitoring Report.

10.4. Responsibilities of the Site Investigators at the participating institutions (including the relevant institution)

In accordance with the instructions of Principal Investigator/Study Coordinator, the Site Investigator at the participating institution should report to the administrator of the relevant institution if the adverse event is subjects of reporting of "disease or the like" to Certified Review Board in accordance with the regulations of the relevant institution.

10.5. Responsibilities of the Data and Safety Monitoring Committee

JCOG Operations Office (Safety Contact) should check the details of the adverse event reports received from the Principal Investigator/Study Coordinator according to the procedures described in 10.3.2. and should report them to Certified Review Board and the MHLW according to the procedures described below, with the presence or absence of causality or expectation.

In addition, Principal Investigator and JCOG Operations Office can hear the opinions of JCOG Data and Safety Monitoring Committee according to the reported adverse events. If a review request is issued, Data and Safety Monitoring Committee can review the appropriateness of the institutional response to adverse events and the propriety of continuation of the study in a consensus or written form.

In addition, the submitted information (JCOG Adverse Event Report (for institution), JCOG Adverse Event Report (for Study Coordinator), "Disease or the like" Report, etc.) will be stored semi-permanently in JCOG Operations Office.

Subjects, destination and deadlines for reporting after the knowledge by Investigator/Study Coordinator are as described in 10.3.2.

11. Response Evaluation and Endpoint Definition

11.1. Response assessment (only for patients with measurable disease)

Tumour response assessment will be performed according to the following steps according to the [49] of version 1. 1-Japanese translational JCOG version-Revised RECIST guideline (version 1. 1) Revised new guidelines for the assessment of treatment response in solid tumours (RECIST guidelines). RECISTv1. The 0 original article stipulates that "the use of this guideline for the purpose of determining continuation of treatment is not the subject of this guideline." Similar statements continue to be included in RECISTv1 1 as follows.

> "Many oncologists make decisions about whether to continue treatment based on both objective imaging criteria and symptom-based criteria for follow-up of patients with malignancies in their daily clinical practice, but these revised guidelines are not intended to be used to make decisions about whether or not to continue treatment in these individual patients, unless the treating oncologist determines that it is appropriate."

Therefore, the "overall effect" as determined by RECIST Guideline-based response assessment should be used to determine whether a drug or regimen shows encouraging results that merit continued developmental studies. In other words, judgment of whether or not to continue treatment in individual patients should not be based on CR/PR/SD/PD of overall efficacy, but rather on "clinical judgment" based on comprehensive consideration of symptoms, physical findings, and various laboratory data, in addition to imaging findings.

Therefore, it may be clinically appropriate to continue protocolized treatment, even when PD (Progressive Disease: progression) is judged as an overall response based on the assessment of response based on imaging. In this case, the pros and cons of continuing protocol treatment should be determined based on clinical judgment, regardless of response assessment, but the date of the event for progression-free survival, which is judged to be an overall effect of PD, should be used. This is due to three reasons: (i) it may be possible to decide whether protocol treatment should be continued for each group; (ii) RECIST is a criterion intended to standardize not only response rates but also progression-free survival; and (iii) the standard definition of US Cooperative Group is that PD is the event of progression-free survival for any reason if the overall response is PD.

On the other hand, if a physician judges "clinical progression" based on clinical and comprehensive judgment not based on diagnostic imaging, even if PD is not met by the response criteria based on diagnostic imaging, protocol treatment should be discontinued in accordance with "6.2.2. Criteria for discontinuation of protocol treatment". If "clinical exacerbation" is judged, even if "PD" is not judged by the response evaluation, the day of "clinical exacerbation" is considered as an event of progression-free survival. This is because imaging is often not performed as planned after a patient is judged to have a "clinical progression" and therefore the risk of overestimating progression-free survival is greater if "clinical progression" is not an event for progression-free survival. It is also statistically incorrect (informative censoring) to treat "clinical progression" as "censoring" progression-free survival, as it would censoring patients at increased risk of progression or death.

In RECISTv1 1, the original article described "definite progression (unequivocal progression") in the PD criteria for non-target lesions as "marked progression of non-target lesions that deserves discontinuation of treatment as an increase in total tumor burden" and therefore described "marked progression of non-target lesions" as "judgement of whether or not to continue treatment in individual patients" in some of the PD criteria for non-target lesions, which is confusing. It should be noted that this "unequivocal progression" is a criterion of judgment restricted to "PD of non-target lesions".

The relation between the events of 'PD', 'clinical progression', 'progression', and progression-free survival in JCOG is as in the lower panel.

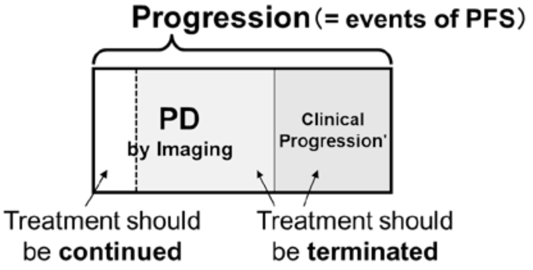

Figure 11.1. Relationship Between Exacerbations, PD on Imaging, and Clinical Exacerbations.

11.1.1. Baseline Evaluation

「8.1. According to "Pre-registration Evaluation Items" contrast enhanced-enhanced computed tomography (CT) with a range of indispensable for the primary organ is used to identify neoplastic lesions before enrollment, and each lesion is classified into "measurable lesions" and "unmeasurable lesions". If CT is allergic to contrast medium, both contrast-enhanced MRI and plain CT should be performed.

Tumor diameters are measured by CT or MRI in cross-sectional views, and sagittal and coronal measurements by three-dimensional reconstructed images are not used. Baseline assessment will be done using the latest imaging studies within 28 days prior to enrollment. If the imaging test is re-examined after enrollment and before the start of treatment, the latest imaging test with re-examination should be used.

In this study, the tumour markers NSEs and ProGRP are used to evaluate the response of non-target lesions.

11.1.2. Definition of measurable lesions

Lesions that fall under any of the following conditions are considered measurable lesions (measurable lesion):

1) Non-nodal disease (non-nodal disease) of 10 mm or greater in greatest dimension on CT or MRI with a slice thickness of 5 mm or less
2) CT or MRI of 5 mm or less slice thickness showing lymph node lesion of 15 mm or more in short diameter (Nodal lesions with short diameters between 10 mm and less than 15 mm are defined as non-target lesions, and those with short diameters less than 10 mm are not)

All other lesions will be non-measurable (non-measurable lesion).

Caution should be exercised because the following lesions are not measurable regardless of the examination method or the size of the lesion.

- Bone lesions (excluding osteolytic lesions with measurable soft tissue components)
- Cystic lesion
- Leptomeningeal lesions
- Ascites, pleural effusion, and pericardial effusion
- Lymphangiosis of the skin and lungs
- Palpable but not measurable abdominal mass or enlargement of abdominal organs
- Superficial skin lesions

11.1.3. Target Lesion Selection and Baseline Recording

Up to five measurable lesions, in descending order of diameter (non-lymph node lesions are long diameters and nodal lesions are short diameters), up to two lesions per organ are selected to be target lesions (target lesion) among measurable lesions at enrollment. Selection should take into account the universal inclusion of as many organs with measurable disease as possible and the reproducibility or ease of measurement (reproducible repeated measurement) of repeated measurements (avoiding lesions that are not measurable even if they are large in diameter).

For selected target lesions, in order from cranial to caudal, site (code), test method, test date, long diameter of non-lymph node target lesion, short diameter of nodal target lesion, and sum of all target lesion diameters (hereafter, sum of diameters) will be recorded in Pretreatment Report 3.

11.1.4. Baseline recording of non-target lesions

For lesions not selected as target lesions, all measurable or non-target lesions (non-target lesion) should be recorded as site of lesion (code), method of examination, and date of examination in Pretreatment Report 3. Multiple non-target lesions within the same organ may be recorded as a single lesion (e.g., multiple enlarged pelvic lymph nodes, multiple liver metastases).

11.1.5. Determining tumor response

Evaluation of target and non-target lesions will be performed every 6 weeks according to "8.2 Testing and Evaluation during Treatment" in the same manner as at enrollment. Target lesion diameter, non-target lesion disappearance or progression will be recorded in the "Tumor Reduction Effect Report".

11.1.6. Response Evaluation Criteria for Target Lesions

・CR(Complete Response): Complete response

When all non-lymph node target lesions disappear and all nodal target lesions have a short diameter of

less than 10 mm. If a nodal target lesion is selected at baseline, the effect of the target lesion may be CR even if the sum of diameters is not 0 mm.

・PR(Partial Response): Partial response

30% or more reduction in target diameter sum compared to baseline diameter sum

・PD(Progressive Disease): Progress

Compared to the minimum diameters (when the baseline is the minimum value during the passage, this is the minimum sum of diameters), the sum of the target disease increases by more than 20%, and the sum of diameters increases by more than 5 mm even in absolute value.

・SD(Stable Disease): Stability

There is no reduction corresponding to PR and no increase corresponding to PD compared to the smallest sum of diameters during the course

・Lack of study; Not all Evaluated

If the test cannot be performed for any reason or if neither CR, PR, PD, or SD can be determined

Pre-treatment sum of diameters-sum of diameters at study
Percentage of reduction of the diameter sum =---------------× x 100%
Pre-treatment diameter sum

Sum of diameters at study-minimum sum of diameters
Increasing Percentage of Diagram =---------------× x 100%
Minimum sum of diameters

※ Measured target lesion diameters are recorded whenever measurable (e.g., <5 mm). If the target lesion diameter is judged to be too small to be measured (too small to measure), the diameter should be 0 mm if the tumor lesion is judged not to be retained, and 5 mm if the tumor lesion is judged to be retained, regardless of the CT slice thickness.

※ PD is defined when the reduction ratio meets the condition of PR and the concomitant increase ratio meets the condition of PD.

※ When one lesion is separated during treatment, each diameter is added to the sum of diameters.

※ If more than one lesion fuses and the boundary cannot be distinguished during treatment, the diameter of the fused lesion is added to the sum of the diameters. The diameter of each lesion is added to the sum of diameters when the boundary of the lesion is identifiable, even if the lesion is in contact with each other.

11.1.7. Response Evaluation Criteria for Non-Target Lesions

・CR(Complete Response): Complete response

When all non-lymph node non-target lesions disappear, the short diameter of all nodal non-target lesions becomes less than 10 mm, and all tumour markers (NSEs, ProGRP※) are below the upper shared reference limits. When there are no (on the image) non-target lesions at baseline assessment and all tumor markers are below the shared reference range upper limit.

Because ※ProGRP is not included in the shared baseline range, the baseline range is 6.5-46.0 pg/mL.

・Non-CR/non-PD: non-CR/non-PD

Residual one or more non-target lesions (including residual nodal non-target lesions ≧10 mm in short diameter) and/or tumour markers (NSEs, ProGRP※) exceeding the shared upper reference limits. No non-target lesions (on images) at baseline assessment and any of the tumor markers exceeding the shared reference range upper limit.

・PD(Progressive Disease): Progress

'Apparent exacerbation' (including relapse) of pre-existing non-target lesions.

For measurable disease: A marked progression of a non-target lesion that deserves discontinuation of treatment as an increase in overall tumor burden must be observed if the effect of the target lesion is SD or PR but is judged to be "clear progression" based on the change in the non-target lesion. If the effect of the target lesion is SD or PR, then an increase in the tumor burden of the non-target lesion to a degree

that far exceeds the decrease in tumor burden is considered "obvious progression" and otherwise Non-CR/non-PD.

If only unmeasurable disease is present, the increase in non-target disease, as judged to clearly exceed the tumor burden corresponding to a 20% increase in diameter and a 73% increase in tumor volume, is considered "definite progression". Elevated tumor markers alone are not considered PD.

・NE(Not all Evaluated): Lack of study

If the test could not be done for any reason or if neither CR, Non-CR/non-PD nor PD could be determined.

11.1.8. Presence or absence of new lesions

If a lesion that was not present at baseline was observed after the start of treatment, it is considered "new lesion" to be present.

However, a "new lesion" requires that it is not an imaging change due to a difference in the imaging method from the baseline assessment or a change in the imaging modality, nor is it an imaging change due to a condition other than the tumor. For example, a cystic lesion arising within a lesion due to necrosis of a liver metastatic lesion is not a new lesion. New lesions will be defined as new lesions by examination of sites that were not mandatory at baseline (pre-enrollment study).

If a lesion disappears and later reappears, measurement is continued. However, the effect at the time the lesion reappears depends on the status of the other lesion. When the overall effect reappears after CR, the lesion is judged as PD at the time of reappearance. When the overall effect is PR or SD, on the other hand, once the disappeared lesion reappears, the diameter of the lesion will be added to the sum of the diameters of the remaining lesions to calculate the effect. That is, in the presence of many residual lesions, even if one lesion reappears after an apparent disappearance, it is not judged as PD by itself, and it is judged as PD when the sum of the diameters of all lesions meets the criteria for PD. This is because of the perception that the majority of lesions do not truly 'disappear' and are not only depicted by the limits of resolution of the imaging modalities used.

If there is a possibility of a new lesion but it cannot be determined, it should not be a new lesion, and imaging should be reexamined at a clinically relevant time. If a new lesion is confirmed by repeat imaging, the new lesion will appear based on the date of imaging at which the new lesion is confirmed.

11.1.9. Overall efficacy (Overall Response)

The overall response (Overall response) will be determined by combining the effects of target lesions, non-target lesions, and the presence or absence of new lesions every 6 weeks according to Table 11.1.9.a below. The overall effect in the absence of a non-target lesion at baseline will be determined by the effect of the target lesion and the presence or absence of a new lesion, and the overall effect in the absence of a target lesion at baseline will be determined according to the effect of a non-target lesion and the presence or absence of a new lesion according to Table 11.1.9.b.

Table 11.1.9.a. Overall efficacy at each time point for target lesions (with or without non-target lesions)

Target lesion	Nontarget lesions	New lesions	Overall effect
CR	CR	None	CR
CR	Non-CR/non-PD	None	PR
CR	Lack of study	None	PR
PR	Lack of Non-PD or study	None	PR
SD	Lack of Non-PD or study	None	SD
Lack of study	Non-PD	None	NE
PD (obvious progression)	Irrespective of	With or without	PD
Irrespective of	PD	With or without	PD
Irrespective of	Irrespective of	Present	PD

Table 11.1.9.b. Overall efficacy at each time point for patients with non-target lesions only

Nontarget lesions	New lesions	Overall effect
CR	None	CR

Non-CR/non-PD	None	Non-CR/non-PD
Lack of study	None	NE
PD (obvious progression)	With or without	PD
Irrespective of	Present	PD

11.1.10. Best overall effectiveness (Best Overall Response)

CR > PR > SD > PD > NE is considered good, and the best overall effect is the best overall effect throughout the entire course.

PD is defined when imaging cannot be determined due to exacerbation of obvious disease or death before the first response assessment. In addition, NE is defined if it cannot be determined by imaging due to discontinuation of toxicity before the first response assessment or patient refusal.

11.2. Definitions of analyses set

The analysis sets used in periodic central monitoring, interim analysis, and final analysis are defined as follows: The flow diagram below shows the analysis sets.

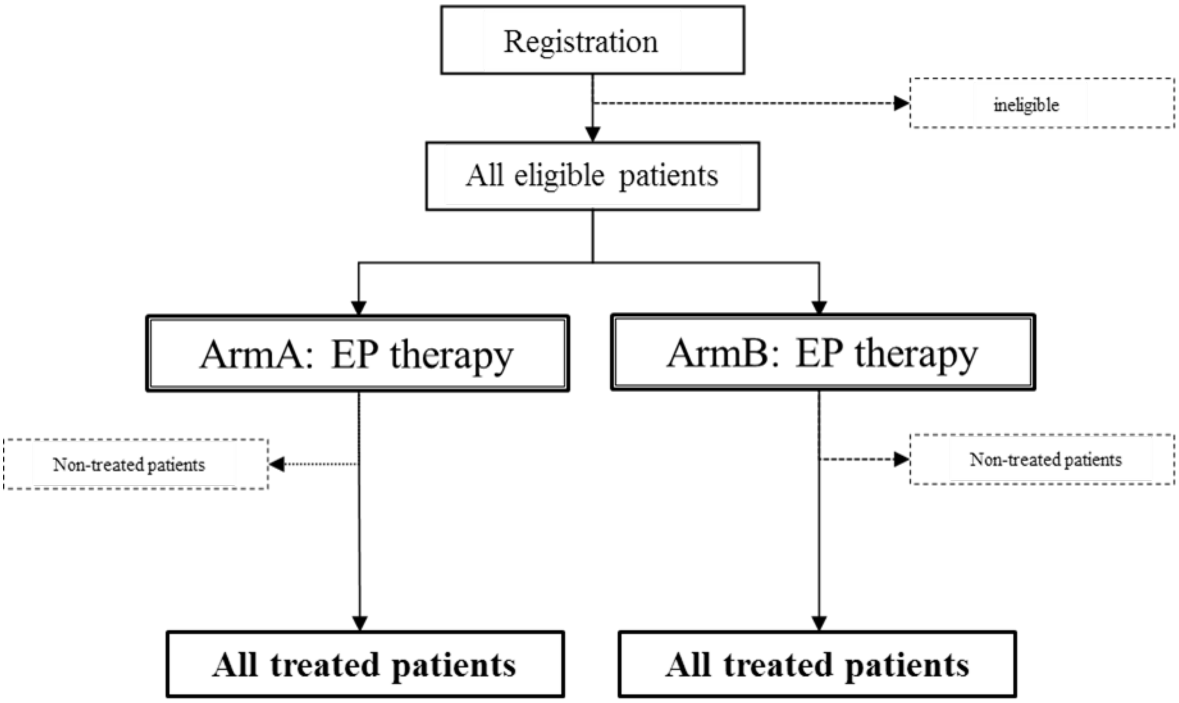

11.2.1. All registered patients

「5.1. Among the patients enrolled according to the Procedures for Enrollment, the population excluding duplicate or mis-enrollment is considered as "all enrolled cases".

11.2.2. All eligible patients

The group excluding "ineligible cases (post hoc ineligibles, de facto ineligibles, violation of registration)" determined by group review from all registered patients is regarded as all eligible patients. Ineligible cases as judged by the investigator or sub-investigator alone are included in all eligible patients. Only those judged not eligible by the central pathological diagnosis are ineligible and not included in all eligible patients.

11.2.3. All treated patients

Of all enrolled patients, all patients for whom part or all of the protocol treatment was performed will be defined as all treated patients.

The decision to treat "non-treated patients" for whom no protocol treatment has been given and whether it is excluded from the safety analysis can be determined by the data center with the consent of the Research Office. Ineligible patients will be excluded from all treated patients. However, if there are circumstances in which ineligible patients are included in the analysis, the nature of the ineligibility will be examined and determined by the Research Secretariat in consultation with JCOG Data Centre.

11.2.4. Patients eligible for central pathology diagnosis

Of all eligible patients, the population excluding ineligible patients with a central pathological diagnosis will be considered as eligible patients with a central pathological diagnosis.

11.3. Definition of endpoints

Endpoint	Events (whichever is earlier)		Censoring date
Overall survival time<br>Overall survival(OS)	All deaths	-	Date of final survival confirmation
Progression-free survival time<br>Progression-free survival(PFS)	All deaths	Progression/recurrence	Final date of clinically confirmed freedom from exacerbation

11.3.1. Overall survival

The duration from the date of registration to the date of death from any cause.

- Survivors are censored at the date of final survival confirmation (survival confirmation by telephone contact is also permitted, but the fact that survival confirmation was performed should be recorded in the medical record).
- Patients lost to follow-up are censored at the last date of survival confirmation before lost to follow-up.

11.3.2. Progression-free survival (PFS: Progression-free survival).

The duration from the date of registration to the date of the judgement of exacerbation or death from any cause, whichever comes first.

- The exacerbation (progression) includes both imaging-based PD (progression) and exacerbation of pathogenic disease (clinical exacerbation) that cannot be confirmed by imaging studies in Section 11.1.9. Global Effectiveness. If an exacerbation is judged based on diagnostic imaging, the exacerbation date is the test date on which the imaging was performed, and in the case of clinical exacerbation, the date of clinical judgment is the exacerbation date. PD may occur in accordance with the Response Evaluation Criteria, even if the tumor diameter is very small, but the disease is clinically judged to be "not obviously aggravated" in accordance with the Response Evaluation Criteria Criteria (in this case, clinical judgment should be prioritized for continued treatment with the protocol). In addition, even if PD is not observed according to the response evaluation criteria, the clinical judgment is prioritized for progression if it is judged to be clinically obvious exacerbation.
- In survivors who are not judged to have progression, clinically confirmed progression is censored (date of final progression-free survival confirmation) (confirmation of progression-free by imaging or specimen examination is not mandatory and clinical progression-free by outpatient examination etc. is not permitted. Telephone contact alone is not permitted. If information on progression or progression-free is obtained at a medical institution or referral center, receive and retain a medical information form describing the rationale for diagnosis. In this case, telephone contact alone is not permitted).
- Events and censoring are treated similarly, if chemotherapy is terminated for reasons such as toxicity or patient refusal, and if other therapies are added as post-study treatment. i.e., it is not censored at the time of treatment termination or at the date of initiation of post-study treatment.
- When the diagnosis of exacerbation is based on imaging, the relapse is not regarded as an event at the test date of imaging with "suspicious diagnosis of relapse", but regarded as an event at the later test date of imaging with "definite diagnosis of relapse"" If the event is judged to be clinically aggravated based on diagnostic imaging, the event is considered to be the day of the progression.
- If the definitive diagnosis of relapse or new lesion is based on biopsy pathology, the date of clinical diagnosis is defined as the date of clinical diagnosis when the diagnosis of recurrence or new lesion is made clinically, and the date of biopsy is defined as the date of event when the diagnosis of recurrence is made based on biopsy pathology diagnosis without clinical diagnosis of recurrence.
- The incidence of a second cancer (metachronous double cancer) is not censored or event, and progression-free survival is defined until other events are observed.

11.3.3. Response rate (response rate) Response proportion (Response rate)

Among all eligible patients with measurable disease, the response rate is defined as the proportion of patients with "11.1.10. Best Global Effect" that is either CR or PR.

11.3.4. Incidence of adverse events (adverse reactions)

Using all treated patients as the denominator, the frequency of the worst Grade during the entire course by CTCAE v4 0 Japanese translation JCOG version for each of the following adverse events (toxicities) will be determined by group:

- Laboratory tests: hemoglobin decreased, white blood cell decreased, neutrophil count decreased, platelet count decreased,

  Increased blood bilirubin, aspartate aminotransferase increased (AST or GOT),
  Alanine aminotransferase increased (ALT or GPT), creatinine increased,
  Hypernatremia, hyponatremia, hyperkalemia, hypokalemia, hypercalcemia, hypocalcemia,

hypermagnesemia, hypomagnesemia

- General disorders and administration site conditions: fever, fatigue
- Ear and labyrinth disorders: Tinnitus, hearing impairment
- Skin and subcutaneous tissue disorders: alopecia
- Gastrointestinal disorders: constipation, diarrhea, nausea, vomiting, oral mucositis
- Metabolism and nutrition disorders: anorexia, dehydration
- Nervous system disorders: dysgeusia, peripheral sensory neuropathy, peripheral motor neuropathy
- Musculoskeletal and connective tissue disorders: myalgia, arthralgia
- Infections and parasites: bronchial infection, pulmonary infection, upper respiratory tract infection, catheter-related infection, biliary tract infection,
  Gallbladder infection; Mediastinal infection; Pleural infection; Urinary tract infection
- Blood and lymphatic system disorders: Febrile Neutropenia
- Musculoskeletal and connective tissue disorders: arthralgia, Vascular disorders: phlebitis
- Respiratory, thoracic and mediastinal disorders: dyspnea, hypoxia, pneumonitis

In the other adverse event (toxicitiy) than the above, the proportion of occurrence are not calculated unless a large number of specific adverse events are observed, since only Grade 3 or more non-hematological toxicity ※ is reported in the Treatment Form.

※※ Non-hematological toxicity refers to adverse events other than those listed below in CTCAE v4.0-JCJCOG.
Anemia, decreased bone marrow cells, decreased lymphocyte count, decreased neutrophil count, decreased white blood cell count, and decreased platelet count.
CD4 lymphocytopenia

11.3.5. Dose intensity of cisplatin

Dose intensity of cisplatin per patient (DOOC.) will be calculated for all treated patients to assess treatment compliance with cisplatin. The summary statistics (minimum, 25% point, median, 75% point, maximum, mean, and standard deviation) will be calculated for each group.

- ・Active dose D.I. (mg/m2/week) = total drug dosebody surface areatreatment duration (weeks)
- Body surface area: BSA is calculated by height at enrollment and body weight calculated by Data Center.
- ・ Treatment period (weeks)
  Arm A = (start date of last course-start date of 1st course + 21)/7
  Arm B = (start date of last course-start date of 1st course + 28)/ 7

11.3.6. Incidence of serious adverse events (adverse reactions)

1) Grade 4 non-hematologic toxicities, early deaths, and treatment

Using all treatments as denominators, the percentage of patients with one or more Grade 4 non-hematologic toxic ※ that are considered to be related (either to definite, probable, possible) to the protocol treatment among the adverse events listed in the free text of the CRFs in addition to the stereotyped items in Section 11.3.4 is used as the numerator.

※※ Non-hematological toxicity refers to adverse events other than those listed below in CTCAE v4.0-JCJCOG.
Anemia, decreased bone marrow cells, decreased lymphocyte count, decreased neutrophil count, decreased white blood cell count, and decreased platelet count.
CD4 lymphocytopenia

2) Early mortality rate

Proportion of all deaths during the protocol treatment or within 30 days from the last protocol treatment day among all treated patients. Causes of death irrespective of causality with protocol treatment. However, if premature death occurs in patients excluded from all treated patients, the details are provided separately.

3) Proportion of treatment-related death (TRD incidence)

Proportion of all deaths judged as causally related (either definite, probable, possible) to the protocol treatment among all treated patients However, if TRD occurs in patients who have been removed from all treated patients, the details are presented separately.

12. Statistical consideration

Methods for statistical analysis are as follows: In addition, the details required for conducting specific analyses are specified in the statistical analysis plan prepared separately prior to the analysis, and in documents that clarify the endpoint definition, etc. If substantial changes occur in statistical analyses as described below, follow the policy set out in "13.6. Protocol Changes." Facilities are contacted by "14.1. Periodic Monitoring" for missing or abnormal results, and data collection or exclusion is determined by review by the Research Secretariat based on the results of inquiries to the institution in accordance with the policies stipulated in "14.1.2. eligible (eligibility/ineligibility)" and "14.1.3. Protocol Deviations/Violations". 「11. Handling of missing values and abnormal data that cannot be addressed by the definition and analysis method for each endpoint, as specified in "Definition of Response Evaluation and Endpoints" and "12. Statistical Items" below, is specified in the above statistical analysis plan.

12.1. Principal Analysis and Decision Criteria

The primary analysis of this study will be the final analysis.

The purpose analysis of this trial is to test whether one of the two community standard-of-care arm A (EP-therapy) and B (IP-therapy) outperforms primary endpoint overall survival. The null hypothesis of equal overall survival in the two groups in the main analysis will be tested by stratified log-rank test stratified by non-institutional allocation adjustment factors (primary organ [gastrointestinal vs. hepatobiliary-pancreatic]) in all patient patients. However, if it is assumed that a stratified log-rank test cannot be performed appropriately, such as when the number of subjects and events in each stratum is small, the allocation adjustment factors will be addressed in the analysis plan prepared without information related to the comparison between groups before performing a confirmatory analysis with comparison between groups. Sensitivity analysis will also be performed in all eligible patients and in patients eligible for central pathology diagnosis.

Due to interest in which treatment group is superior, the test will be two-tailed. The study-wise significance level for the study is set at both sides 10%. In the main analysis, two-sided 90% confidence intervals corresponding to a two-sided 10% significance level will be calculated, and in other analyses, two-sided 95% confidence intervals will be calculated for descriptive purposes.

We conclude that EP therapy is a more useful treatment when the two-sided p-value is less than 10% and the survival curve of EP therapy exceeds that of IP therapy. Meanwhile, we conclude that IP therapy is a more useful treatment when the two-sided p-value is less than 10% and the survival curve of IP therapy exceeds that of EP therapy. If the difference was not significant at a two-sided significance level of 10%, one of the two modalities cannot be judged to be useful. In this case, we conclude that both modalities continue to be the standard of care, as there is no evidence to actively recommend either one of them.

However, if there are major differences in the toxicity profiles of EP and IP therapies, contrary to prior assumptions, during the course of the study, and if the toxic therapy is not superior to other therapies, it is judged that it will not be of significance to be used in clinical practice, the following measures should be taken. In other words, the clinical hypothesis will be changed from two-sided to one-sided in an analysis plan with no information on group comparisons before a confirmatory analysis with group comparisons will be conducted, and the superiority of the toxicity treatment over other treatment modalities will be verified at a one-sided significance level of 5% (in this case, the policy of change for the interim analysis will be described in Section 12.3.2).

Estimates such as cumulative survival curves, median survival times, and annual survival rates are performed using Kaplan-Meier method. Brookmeyer and Crowley methods are used to obtain 95% confidence intervals for median survival. Greenwood's formula is used to obtain 95% confidence intervals for annual survival rates. Hazard ratios and their confidence intervals for treatment effects between groups are obtained using stratified Cox proportional hazards models with the same factors as the test of the primary analysis as estimates of treatment effects. Cox regression adjusted by imbalance background factors in addition to adjustment factors will be performed as needed.

The main analysis results will be summarized as the "Main Analysis Report" by the Data Center one year after the completion of enrollment and submitted to the Research Secretariat, Research Representatives, Group Representatives, Group Secretariats, Efficacy and Safety Assessment Committee, and JCOG Representatives.

The principal investigator/study coordinator summarizes the content of the main analysis report, prepares a

"Clinical Study Report" summarizing the conclusions, problems, interpretations and discussion of the results, and future policies of the entire study, and submits it to the Data and Safety Monitoring Committee and JCOG chair with approval from the Group chair and the Head of JCOG Data Center.

Approval of the clinical study report by the Data and Safety Monitoring Committee shall be considered as "end of the study."

12.2. Planned accrual, accrual period, and follow-up periods

「2.4.2. Based on the background presented in Clinical Hypothesis and Rationale for Number of Enrollment, we assume a median survival of 8 and 12 months (HR=0.67) for the inferior and superior treatment groups, respectively, among the two treatment groups. When a superiority trial design is used, 63 patients per group and 126 patients in both groups (114 events required) will be included in the required analysis using Schoenfeld & Richter methodological [50] at 6 years of enrollment, 1 year of follow-up, α = 10% (two-sided), and 70% power. The number of required Inferior treatment (number of required events) when the median survival time in A is different from the assumption as shown in Table 12.2.1 below.

Table 12.2.1. Number of Analyses Required (Number of Events Required)

	Power			
Median Survival (mo)	65%	70%	75%	80%
7.0 vs. 10.5	108(100)	124(114)	142(131)	162(150)
8.0 vs. 12.0	112(100)	126(114)	144(131)	166(150)
9.0 vs. 13.5	114(100)	130(114)	148(131)	170(150)

※※ Number of required events in parentheses

Based on these findings, the following will be established in view of some cases of loss to follow-up.

Planned enrollment: 70 patients in each group and 140 patients in both groups

Enrollment period: 6 years, follow-up period: 1 year after completion of enrollment

Consider redesigning the sample size if the prognosis is obviously better than assumed, or if it reaches 70 people within 2.5 years of enrollment initiation. Clinically meaningful differences will then be reviewed and redesigned in a blinded fashion prior to the conduct of the analysis.

<Additions in ver. 1.1>

The registration pace after the enrollment start was smooth and exceeded the plan, and 70 cases were reached in October, 2016, when 2 years and 2 months passed from the enrollment start. Since it exceeded the standard of 70 patients within 2.5 years from the initiation of enrollment specified above, acceptance was obtained at the meeting of the Hepatobiliary and Pancreatic Group on October 29, 2016, the group meeting of the Esophageal Cancer Group on November 19, 2016, and the group meeting of the Gastric Cancer Group on January 7, 2017 with respect to changing the power to 80% according to the rules at the time of the study plan. Therefore, the planned enrollment number was changed to 170. In addition, the "1-year analysis period" was added to the study period, and the following changes were made.

Planned enrollment: 85 patients in each group and 170 patients in both groups

Enrollment period: 6 years; Follow-up period: 1 year after completion of enrollment; Analysis period: 1 year; Total study period: 8 years

<Additional information in ver. 1.5.0>

In the monitoring in the latter half of fiscal 2019, it was found that the number of events was less than expected. Follow-up will be extended by 1 year to 2 years after completion of enrollment because the required number of events may not be reached at 1 year after completion of enrollment for which final follow-up is planned. However, if the required number of events has been reached at 1 year after completion of enrollment, the final analysis will be performed at 1 year after completion of enrollment. Conversely, if the required number of events is not reached, follow-up will continue until the required number of events is reached to ensure power, and the final analysis will be performed without waiting for the end of the remaining follow-up period when the required number of events is found to be reached within the follow-up period.

Planned enrollment: 85 patients in each group and 170 patients in both groups

Enrollment period: 6 years; follow-up period: 2 years after completion of enrollment; analysis period: 1 year; total study period: 9 years

12.3. Interim Analysis and Early Termination of the Study

12.3.1. Purpose and Timing of the Interim Analysis

Once interim analyses will be conducted to determine if the primary objective of the study has been achieved during the study period. Interim analyses will be conducted to determine if it is reasonable to continue enrollment during enrollment. If the primary objective of the study is determined to be achieved, the study will be discontinued and the study results will be published promptly at the conference and in the article.

Interim analyses will be conducted using data from the initial periodic monitoring that will be queried after the time enrollment of half of the planned enrollment was obtained. Based on the information in the periodic monitoring report, the group will submit the presence or absence of changes in clinical hypotheses and, if any, details of changes to the Efficacy and Safety Assessment Committee by the time of the interim analysis.

During the first interim, patient accrual is continued in principle. If the progress of the study progressed as planned, the expected number of events at the time of the interim analysis under the assumptions presented in 12.2 is expected to be 44 if the interim analysis is conducted at 3 years after the start of enrollment.

12.3.2. Method of interim analysis

Interim analyses will be conducted by the JCOG Data Center. To keep the study-wise alpha errors at 10%, the multiplicity of the interim and final analyses is adjusted using Lan & DeMets's alpha spending functions, and statistical significance is examined for differences in primary endpoint between arms. As α spending functions, we use O'Brien & Fleming types.[51]

For details of the interim analysis, the statistical staff in charge of the group at the Data Center will prepare the statistical analysis plan by the time of the interim analysis. Actual interim analyses will be performed by statistical staff who are not in charge of the group and an interim analysis report will be prepared.

In the interim analysis, if the survival of one treatment group exceeds that of the period, and the p-value of the stratified log-rank test falls below the level specified by the above method, it is judged statistically significant and the trial is discontinued as a rule. The ineffective discontinuation is not planned at a stage where no statistically significant differences are observed unless prior assumptions regarding toxicity are changed. However, the clinical hypothesis may be changed for the reasons described in 12.1, and the primary analysis may be performed by one-sided rather than two-sided testing, which may result in deviations from prior assumptions. In such cases, the primary hypothesis change should be specified in the analysis plan to be prepared without information on group comparisons before a confirmatory analysis with group comparisons. If the overall survival curve in the highly toxic treatment group is below the other, the consideration of ineffective discontinuation should not be judged by a test and considered comprehensively.

12.3.3. Interim analysis Reporting and review of the results

The results of the interim analysis will be submitted to the Data and Safety Monitoring Committee by the Data Center as an Interim Analysis Report and reviewed for the acceptability of continuation of the study and for publication of the results. The Data and Safety Monitoring Committee considers whether to continue the study at the meeting and recommends whether to continue the study and whether to publish the results to principal physician or group chair based on the results of the review.

Members of the Data and Safety Monitoring Committee of the relevant group are not included in the review. Unless the results of the interim analysis make recommendations for discontinuation of the study from the Efficacy and Safety Assessment Committee, the research representative, research office, participating institution researchers, group representatives, and group secretaries of the study will not be able to know the results of the interim analysis until the final follow-up is completed.

When the Interim Analysis Report has been reviewed by the Data and Safety Monitoring Committee to recommend termination or change of all or part of the study, the principle investigator and group chair review the recommendations and decide whether to discontinue or change some of the study.

If the study is discontinued or part of the study is changed, the principal investigator and group chair shall submit

in written form a request to the Data and Safety Monitoring Committee for permission to discontinue the study or a request to revise the protocol. Following approval by the Data and Safety Monitoring Committee, the principal investigator may discontinue the study or change part of the study.

The Study Chair and Group Chair can disagree with the recommendations of the Data and Safety Monitoring Committee, but if they fail to coordinate their opinions with the Data and Safety Monitoring Committee, they will ultimately follow the instructions of JCOG Chair.

If the study is terminated, the subsequent follow-up period will be 1 years from last registration per study.

If the interim analysis resulted in study termination, the interim analysis will be the primary analysis of the study. The Data Center, in cooperation with the Research Representative Physicians and Research Secretariat, will conduct the analysis required to complement the incomplete data and publish the results, focusing on the results of the interim analysis, and promptly prepare the Major Analysis Report and submit it to the Group and the Efficacy and Safety Assessment Committee.

12.4. Analysis of Secondary endpoints

Secondary endpoints analyses will be conducted to provide a supplementary discussion of the primary analysis results of the study. Because the analysis of secondary endpoint is exploratory, no multiplicity adjustments are made. Comparisons between arms are made where appropriate, note that when the results of the group comparisons are not significant, they do not mean that there is no difference between the two arms.

12.4.1. Analysis of safety secondary endpoints

Among Secondary endpoints, the safety endpoints are the incidence of adverse events and the incidence of serious adverse events, which are in principle the items of periodic monitoring (14.1. Periodic monitoring).

The incidence rate of adverse events will be summarized as well as the incidence rate of Grade3 or higher. For adverse events other than laboratory data, the incidence of Grade 2 or higher is also calculated.. Non-hematological toxicity incidence, early mortality, and treatment-related mortality rates of Grade4, which are serious adverse events, are reported in periodic monitoring reports with registration numbers and details. The rates of non-hematologic toxicity, early mortality, and treatment-related mortality for Grade4 will be calculated at the time of the interim analysis and the main analysis. When interval estimation of proportions is performed, accurate confidence intervals based on binomial distributions are used. Comparisons between arms will be made using Fisher's exact test where appropriate.

12.4.2. Analysis of efficacy secondary endpoints

Among Secondary endpoints, efficacy endpoints are response rate, progression-free survival, which will only be analyzed in the interim and primary analyses.

Secondary endpoints analyses do not adjust for multiplicity.

Progression-free survival will be included in all enrolled patients, but a comparison of all eligible patients, excluding ineligible patients, as determined by group study, will also be performed as a sensitivity analysis.

The response rate will include all eligible patients with measurable disease, but a comparison of all enrolled patients will also be performed as a sensitivity analysis.

Fisher's exact test will be used to compare response rates between groups, and binomial distribution-based exact confidence intervals will be used for interval estimation. Estimates, including progression-free survival curves, median progression-free survival, and time-point progression-free survival, will be performed using Kaplan-Meier method, Brookmeyer and Crowley methods will be used to obtain confidence intervals for median progression-free survival, and Greenwood formulas will be used to obtain confidence intervals for progression-free survival. Log-rank test is used for comparison between arms. Hazard ratios and their 95% confidence intervals for treatment effects between arms will be calculated using Cox's proportional hazards model as an estimate of treatment effect. Cox regression adjusted by imbalance background factors in addition to adjustment factors will be performed as needed.

12.5. Final analysis

The primary analysis will be the final analysis unless this study is withdrawn from the interim analysis.

If the interim analysis is withdrawn from the study, then after the end of the 1-year follow-up period, the final analysis will be performed after the final survey confirms the data and then analyses will be performed for all

endpoints.

Except for the interim analyses and the final analysis, analyses with between-arms comparisons for the primary and secondary endpoints for efficacy are not performed unless approved by the Data and Safety Monitoring Committee.

If the final analysis is performed after the main analysis, the final analysis will be summarized by the Data Center as the "Final Analysis Report" and submitted to the Research Secretariat, Research Representatives, Group Representatives, Group Secretariats, Efficacy and Safety Assessment Committee, and JCOG Representatives.

The Study Representative Physician/Research Secretariat summarizes the content of the final analysis report, prepares the "Clinical Study Report" summarizing the conclusions, issues, interpretations and discussion of the results, future policies, etc. mainly from the clinical point of view (if the "Clinical Study Report" is prepared in the previous analysis report, it will be the "Clinical Study Report (Supplementary Version)) with additional updates), and submits it to the Study Representatives and JCOG Representatives with approval from the Group Representatives and the Head of JCOG Data Center.

Approval of the clinical study report by the Data and Safety Monitoring Committee shall be considered as "end of the study."

<Additional information in ver. 1.5.0>

If the required number of events has been reached at 1 year after completion of enrollment, the final analysis will be performed at 1 year after completion of enrollment. Conversely, if the required number of events is not reached, follow-up will continue until the required number of events is reached to ensure power, and the final analysis will be performed without waiting for the end of the remaining follow-up period when the required number of events is found to be reached within the follow-up period.

12.6. Exploratory analysis

To investigate the interaction between treatment effect and the subpopulation, subgroup analyses will be conducted exploratory with respect to the following factors: Because these analyses are not adequately powered and do not adjust for multiplicity, the results of each subgroup analysis should be interpreted as exploratory.

- PS0/1
- Age 65 years or older/<
- Gender (male/female)
- Primary organ (gastrointestinal tract/hepatobiliary pancreas)
- Organ of origin (esophagus/stomach/small intestine/large intestine/pancreas/biliary tract/liver NEC (liver primary or liver metastasis of unknown primary)
- Organ of origin (pancreas/non-pancreas)
- Extent of extension of the primary lesion (locally advanced/distant metastasis or recurrence)
- Extent of extension of the primary lesion (locally advanced/distant metastasis/recurrence)
- Prior radical resection of the primary lesion (none/present)
- Pathological diagnosis was biopsy/resection specimen
- Grade 3 tumour with morphologically similar features of NETs but Grade 3 proliferative activity/morphologically more atypical (previously classified as poorly differentiated endocrine carcinoma) on histopathology with central pathology
- Histopathological examination by central pathological diagnosis, including Small cell carcinoma/Large cell carcinoma /
- Histopathologically diagnosed by central pathology, Ki67 50% or higher/less than 50%

12.7. Premature withdrawal from the trial

In this study, early termination of the study may occur in the following cases:

1) Early termination due to discontinuation of interim analysis
2) Early termination due to adverse events
3) Early termination due to poor enrollment
4) Early termination due to other reasons

12.7.1. Early termination by interim analysis

In this study, based on the criteria described in 12.3, early termination recommendations may be made at the interim analysis review by the Efficacy and Safety Assessment Committee. If the Data and Safety Monitoring Committee provides recommendations for early termination of the study, the principle investigator and group chair will review the recommendations and decide whether to terminate the study early.

12.7.2. Early termination due to adverse events

In JCOG9511 for small-cell lung cancer, 1/77 (1.3%) treatment-related deaths were reported with EP therapy and 3/77 (3.9%) with IP therapy; in JCOG0509, a successor study for the same subject, 1/142 (0.7%) treatment-related deaths were reported with IP therapy and 2/142 (1.4%) with amrubicin plus cisplatin therapy. Using these as reference, we believe that the treatment-related mortality rate should not exceed 3% in this study. Since it is clear that the final point estimate will be at least 3% at the time of 3 treatment-related deaths in either group, immediate entry should be suspended to consider whether or not to withdraw from the study. At this point, the subsequent treatment of the patient being treated will be reviewed. At the time of 3 or fewer treatment-related deaths in each group, each patient will be reported to the Efficacy and Safety Assessment Committee for adjudication. Enrollment will be continued until the results are obtained in principle.

12.7.3. Early termination due to poor enrollment

If the patient enrollment pace is significantly worse than at the time of planning, early termination of the study may be advised by the Data and Safety Monitoring Committee. If early termination recommendations are issued by the Data and Safety Monitoring Committee due to poor enrollment, the principle investigator and group chair will review the recommendations and decide whether to terminate the study early.

12.7.4. Early termination due to other reasons

12.7.1.～12.7.3. If it is judged difficult to continue the study for other reasons, the research representative physician shall submit a request for early termination of the study to the Efficacy and Safety Assessment Committee. If the Data and Safety Monitoring Committee recommends early termination of the study based on the submitted data, the procedure for early termination of the study will be progressed.

12.8. Procedures after Early termination of the Study

If the Study Chair accepts the recommendations made by the Data and Safety Monitoring Committee based on Section 12.7, he/she will promptly submit a notification to the Data and Safety Monitoring Committee that early termination of the study will be performed.

The Study Chair will submit a termination notification to the Certified Review Board within 10 days of the date they decide to terminate the study early. If the study falls into a specified clinical trials under the Clinical Trials Act, the Study Chair shall submit a termination notification to the Certified Review Board within 10 days of the date on which the study was decided to be prematurely discontinued, as well as submit a specified clinical trials termination notification to the MHLW.

The Study Chair promptly informs the investigator of the decision to terminate the study early in writing, and the investigator who has received a report of early termination of the study will report in writing that the study was prematurely terminated to the institution's manager without delay.

If the study is terminated early, JCOG Data Center will promptly initiate the development of the primary analysis report or final analysis report. The subsequent follow-up period will be 1 year from the final enrollment.

13. Ethical Considerations

13.1. Protection of Human Subjects

All researchers involved in this study will conduct this trial in accordance with the "Helsinki Declaration" [1] and "Clinical Trials Act" (2017 Law No. 16) [2] "Clinical Trials Act Enforcement Regulations" (2018 Ministry of Health, Labour and Welfare Order No. 17) and related notices.

1） http://dl.med.or.jp/dl-med/wma/helsinki2013j.pdf

2） http://www.mhlw.go.jp/stf/seisakunitsuite/bunya/0000163417.html

Prior to commencing this study, the site investigator is required to obtain input from Certified Review Board[※1] regarding the conduct of the study, and to obtain approval from the Administrator of the participating institution, and submit the Implementation Plan [※2] to the Ministry of Health, Labour and Welfare.

※ 1 JCOG study will be submitted to the following Certified Review Board.
National Cancer Center Hospital Certified Review Board (accreditation number CRB3180008)

※ 2 "Implementation Plan" means "Documented plan formatted according to the Form No.1 (Form No.1 of the Ministerial Ordinance) specified in Article 39 of the Clinical Trials Act Enforcement Regulations"

13.2. Informed consent

13.2.1. Explanation to the patient

Prior to patient registration, the investigator or subinvestigator will provide the patient with written informed consent form approved by Certified Review Board and explain the following details verbally.

Descriptions

1） Disease names, Stages, and expected prognosis

2） That this study is a clinical trial and is conducted by JCOG
Name of Certified Review Board and contact information for receiving complaints and inquiries to the committee

3） Design and rationale of the study

4） Protocol treatment content

5） Effects expected by protocol treatment

6） Expected adverse events, complications, and sequelae and how to deal with them
Explanation of the extent and frequency of expected adverse events, including complications, sequelae, and treatment-related deaths, and how to deal with them when they occur. In addition to these explanation, obtain the most recent version of the drug package insert and deliver it to patients (PMDA Prescription Pharmaceutical Information Search http ://www.pmda.go. jp/PmdaSearch/iyakuSearch/)

7） Post-study treatment after end of protocol treatment should also be performed appropriately

8） Cost burden and compensations
Explanation of the cost of treatment, compensation that can be received in the event of a health hazard (equivalent to measures taken in general practice, etc.)

9） Alternative treatment
Explanation of treatments that can be received if not participating in this study

10） Anticipated benefits and possible disadvantages
Explanation of anticipated benefits and possible disadvantages by participating in this study

11） Direct access to the medical records
Explanations on acceptance of site visit audits, such as "direct access to medical records etc. by healthcare professionals at other medical institutions for quality control with permission from the administrator of the participating institution."

12） Refusal of consent and withdrawal of consent
Refusal to consent prior to participation in the study is free, and withdrawal after having given consent is free, thereby not causing undue medical disadvantage.

13） Protecting human rights
Every effort should be made to ensure that personal information, such as names, is kept confidential.

14） Secondary use of data

The possibility of secondary use of data obtained from this study in Japan and overseas (ancillary studies, meta-analyses, etc.) only when approved by either committee in JCOG

15） Method of disclosure of information on the study
The study is registered and published in jRCT※. In addition, the results of clinical studies should also be published in jRCT (* Databases (Japan Registry of Clinical Trials) https://jrct.niph.go.jp/ prepared by the MHLW as stipulated in Paragraph 1 in Article 24 of Clinical Trials Act Enforcement Regulations)

16） Freedom of questions
Explanation that investigators, written contact information for consultations on study details, on the Principal Investigator and the Study Coordinator, and freely asking questions about study and treatment

17） Explanation of the use and burden of medicinal products not covered by insurance

18） Central pathological review

19） Central image review for response

20） Sample collection for ancillary studies

13.2.2. Consent

Explain the study, give sufficient time to think, confirm that the patient understood the study well, and ask for participation in the study. If the patient agrees to participate in the study, the written informed consent form in the appendix will be used to obtain the patient's own signature. The site investigator or the subinvestigator confirms that the study consent form contains the name of the physician who provided the explanation and the date of explanation, the name of the patient who gave informed consent, and the date of informed consent.

In addition, when it is not possible to read the documents due to visual impairment, etc., but the details can be understood by verbal explanation, or the documents can not be signed due to limb disorders, etc., but the documents can be read and understood, signatures may be obtained from the proxy author under the consent by the patient. However, the signature of the proxy author should be based on this study's consent, and should be described as "Signing by the proxy author" and "Relationship with the patient" so that the person can be found to be the proxy author.

Two copies of the consent form will be provided, one will be handed over to the patient, and one will be stored by the site coordinator. Original copies will be stored in the medical records or in the archives specified by the participating institution.

13.2.3. Response to inquiries, consultations, etc. after consent

In principle, the investigator or subinvestigator of the relevant patient's participating institution responds to any consultation related to the study by the patient or his/her family after registration. If it is unclear how to respond, respond in consultation with Principal Investigator, Study Coordinator, the Group Secretariat, Group Chair, JCOG Data Center/Operations Office, etc. in accordance with the content of the consultation.

13.2.4. Withdrawal of consent

After obtaining informed consent for participation in the study, consent will be withdrawn if the patient expressed withdrawal of consent.

Withdrawal of consent refers to withdrawal of consent to participate in research and is distinguished from refusal to continue protocol treatment (below (i)). If the withdrawal of consent is expressed, clarify whether (ii) or (iii) below and promptly notify JCOG Data Center. If consent is withdrawn, record it in the medical record as well as (ii) or (iii).

Data Center discontinues subsequent follow-up requests according to the protocol in case of (ii) withdrawal of consent, In the case of (iii) full withdrawal of consent, the data of the patient are removed from the database when it is confirmed that the patient has full withdrawn of consent.

The procedures for discontinuation of the patient's follow-up request and removal of patient data will be specified separately in the procedural manual, and the completion of each task will be reported to Principal Investigator and Study Coordinator.

(i) Patient refusal: Refusal to continue subsequent protocol treatment (follow-up continues).

(ii) Withdrawal of consent: Withdrawal of consent to participate in the study and termination of all subsequent treatment and follow-up in accordance with the study protocol. Research use of data prior to withdrawal of consent is permitted.

(iii) Full withdrawal of consent: Withdrawal of consent to participate in the study and unavailability of all data from the time of patient registration, including information at registration.

In addition, some medical institutions may request that a "withdrawal of consent" form be prepared as a written document. However, in the event of withdrawal of consent, written expressions of willingness are required to increase the psychological barriers to withdrawal of consent (i.e., it is difficult to withdraw consent), and it is considered unwanted from the viewpoint of protecting human subjects. Therefore, in JCOG, written expressions of willingness are not mandatory for withdrawal of consent, verbal withdrawal of consent is valid, and the "withdrawal of consent" form is not prepared. If "withdrawal of consent" form is required by the participating institution, it should be prepared by the institution.

- This should be used in studies conducted in accordance with the Ethical Guidelines for Medical and Health Research Involving Human Subjects.

The procedures for discontinuation of the patient's follow-up request and removal of patient data will be specified separately in the procedural manual, and the completion of each task will be reported to Study Chair and Study Coordinator.

(i) Patient refusal: Refusal to continue subsequent protocol treatment (follow-up continues).

(ii) Withdrawal of consent: Withdrawal of consent to participate in the study and termination of all subsequent treatment and follow-up in accordance with the study protocol. Research use of data prior to withdrawal of consent is permitted.

(iii) Full withdrawal of consent: Withdrawal of consent to participate in the study and unavailability of all data from the time of patient registration, including information at registration.

In addition, some medical institutions may request that a "withdrawal of consent" form be prepared as a written document. However, in the event of withdrawal of consent, written expressions of willingness are required to increase the psychological barriers to withdrawal of consent (i.e., it is difficult to withdraw consent), and it is considered unwanted from the viewpoint of protecting human subjects. Therefore, in JCOG, written expressions of willingness are not mandatory for withdrawal of consent, verbal withdrawal of consent is valid, and the "withdrawal of consent" form is not prepared. If "withdrawal of consent" form is required by the participating institution, it should be prepared by the institution.

13.3. Protection of personal Information and patient identification

JCOG recognizes that information on privacy, such as personal information and medical information, should be protected and handled carefully, based on the principle of respecting the personality of individuals, and has formulated JCOG Privacy Policy, and will take all possible measures to protect privacy. For more information, see JCOG website (http://www.JCOG.jp/).

13.3.1. Policies, legislation, and norms followed by JCOG

In conducting JCOG study, JCOG follows, in principle, JCOG Privacy Policy as well as the following laws and norms depending on the content of the research. If other laws, norms, and policies are applicable, they should be followed.

- Clinical Trials Act (Law No. 16, 2017).
- Act on the Protection of Personal Information (Law No. 57, 2003, Final Amendment: Law No. 65, September 9, 2015).
- Helsinki Declaration (Translation by the Japan Medical Association)
- Ethical Guidelines for Medical and Health Research Involving Human Subjects (No.1 of Notice of the Ministry of Education, Culture, Sports, Science and Technology and the Ministry of Health, Labour and Welfare, 2017).

13.3.2. Use of personal information objective, items to be used, and methods of use

1) Objective of use

In accordance with the basic philosophy "Providing the best treatment to more patients," JCOG uses personal information, etc. of patients for objective of "Identifying patients and conducting surveys not only during treatment but also for a long period after end of treatment in order to obtain the correct results of clinical studies, and appropriately managing the acquired information."

2) Items to be used

Information to identify individuals who will be used by JCOG as minimally require for identification and inquiry of patients is as follows.

Medical record number, date of birth, initials, and registration number, Pathology specimen number

In other words, information that can identify individuals other than those listed above, such as the patient's name, is not informed to Data Center by participating institutions, and if they are falsely informed, they should be destroyed without using the recording medium or stored after performing appropriate processing, such as masking, which is incapable of reading.

3) Method of use

Personal information of patients used by JCOG will be collected by entering the CRFs etc. by researchers at participating institutions and submitting them to Data Center either by JCOG Web Entry System, mailing, or handover as a rule. However, telephone calls will be used only for patient registration where prompt contact is necessary.

In addition, in order to confirm the accuracy of the collected information, inquiries regarding various types of CRFs, including personal information, between Data Center and researchers at medical institutions are limited to either JCOG Web Entry System, mailing, or handover. Only the more anonymous registration number should be used when interacting with e-mail inquiries, and medical record numbers and initials should not be used.

13.3.3. Preparation of records for provision of samples and information, etc. at participating institutions

The investigator at each site will prepare records for this study of enrolled patients. The matters prescribed by the Ordinance of the Ministry of Health, Labour and Welfare (Article 53 of Clinical Trials Act Enforcement Regulations) are as follows.

- Identification of clinical-research subjects
- Items related to medical care and testing for subjects personnel in clinical studies
- Items related to participation in clinical research
- Other matters necessary to conduct clinical research

13.3.4. Source documents used in clinical studies (JCOG trials conducted under Clinical Trials Act)

The source documents for clinical studies used in this study refer to all records used for diagnosis and treatment, including medical records (including worksheets, etc.), laboratory test records, diagnostic images used for diagnosis, pathological diagnosis report, images used for response evaluation, and informed consent documents, of enrolled patients in this study. These should be available for direct access for monitoring (14.1.) and audit (14.2.) conducted by Data Center, Operations Office, Certified Review Board and regulatory persons

13.3.5. Storage of samples and information

Samples and information of enrolled patients on this study shall be stored in accordance with Article 53 of Clinical Trials Act Enforcement Regulations (MHLW ordinance No. 17, 2018). The retention time of records related to this study at participating institutions and the retention time of source documents will be 5 years from the date of completion of the study. It is recommended that the records be stored for as long as possible after the expiration date.

The retention time of data collected in JCOG Data Center will be semi-permanent in view of the possibility of long-term follow-up and secondary use for other studies. In addition, as records for the provision of samples and information, the study protocols and the model informed consent forms will be stored semi-permanently in JCOG Data Center.

13.3.6. Anonymization and control of response tables

In JCOG studies, information that can clearly identify individuals, such as patients' names, is not collected, and individuals are identified using registration numbers and medical record numbers (anonymized). Correspondence tables (not always in the form of a table) of information and registration numbers that can clearly identify individuals by themselves, such as patient names, are positioned as present at each participating institution and are appropriately managed according to the policy of participating institutions so that the identification of enrolled patients can be ensured.

13.3.7. Secondary use of data

Data from this study may be used in Japan or abroad for secondary use (e.g., meta-analysis) only if approved by the relevant committee of JCOG (e.g., Protocol Review Committee, Data and Safety Monitoring Committee). However, when providing data to external organization(e.g., a meta-analysis), data are provided so that it is not possible to identify individuals.

Secondary use of data should be disclosed on JCOG website to ensure opportunities for patients to refuse using data.

13.3.8. Safety management responsibility system

JCOG Data Center establishes a privacy protection control manager and a privacy protection manager and takes various safety control measures to minimize the risks of information leakage when using personal information, etc.

13.3.9. Response to request of disclosure of patient information

In the event that JCOG is asked to disclose privacy-related information, etc., the responder shall, in principle, be a researcher (site investigator, subinvestigator) at the institution of the relevant patient.

13.3.10. Receipt of inquiries about JCOG

General inquiries and complaints about privacy policies will be received by either mail, e-mail, or fax below.

Inquiry liaison: JCOG Data Center Privacy Protective

Postal destination :〒104-0045 Tsukiji 5-1-1, Chuo-ku, Tokyo
Clinical Research Support Office, National Cancer Center Hospital
E-mail :JCOG_privacy@ml.JCOG.jp
FAX :03-3542-3374

13.4. Adherence to the protocol

Researchers participating in this study will adhere to this protocol unless they compromise patient safety and human rights.

13.5. Application to Certified Review Board and Notification of Implementation Plans

When conducting this study, the approval of Certified Review Board and the permission by the administrator of each institution to conduct the study using this protocol and written informed consent from patients must be obtained. Prior to the commencement of the study, the implementation plan ※1 shall be submitted to the Minister of Health, Labour and Welfare, and the test data shall be published to jRCT※2. Principal Investigator is responsible for applying to Certified Review Board, submitting Implementation Plans to the MHLW, and registration in jRCT, and JCOG Operations Office supports these application procedures.

※ 1 Form No.1 of the Ministerial Ordinance stipulated in Article 39, Paragraph 1 of the Ordinance for Clinical Trials Act Enforcement Regulations

※ 2 Databases (Japan Registry of Clinical Trials) prepared by MHLW as specified in Article 24, Paragraph 1 of Clinical Trials Act Enforcement Regulations https://jrct.niph.go.jp/

13.5.1. Procedures for new application

1) Procedures performed by Principal Investigator

<Procedures from initial submission to study initiation>

(i) Review and approval of the study protocol by JCOG Protocol Review Committee (ver.1.0.0).

(ii) Principal Investigator will prepare the following documents for this study and submit these documents to Certified Review Board for review through JCOG Operations Office.
- ➢ New Review Request Form (Unified Form No.2 for Clinical Research)
- ➢ Implementation Plan (Ministerial Ordinance Form No.1)
- ➢ Study plan (this protocol) (including response to "disease or the like")
- ➢ Informed consent form
- ➢ Documents describing summary of drugs, etc. (e.g., package inserts of drugs used as a part of protocol treatment)
- ➢ List of Subinvestigators (Unified Form No.1 for Clinical Research)
- ➢ Conflict of Interest Management Standard (Guidance Form A), Conflict of Interest Management Plan (Guidance Form E) (see 13.X.)
- ➢ Other documents to be submitted when prepared (review documents of JCOG Protocol Review Committee, sample Case Report Forms, and a draft agreement on funding with the marketing authorisation of drugs, etc. or its special associates)

(iii) Modifications such as protocols and informed consent forms are made to review opinions submitted by Certified Review Board as needed.

※ Response to the indications by Certified Review Board: Prepare modified versions with review and approval by the Director of Data Center if modification of the protocol or informed consent forms is needed (ver.1.0.1, ver.1.0.2, ver.1.0.3…). .

(iv) After Certified Review Board approval is obtained, JCOG Operations Office should have Certified Review Board approval date and the approved version number on the cover page of the protocol and informed consent form and appear on JCOG website's Protocol Download page.

(v) Principal Investigator will use Certified Review Board review results notification and the documents submitted in ② to obtain the approval of the administrator of the institution which he/she belongs to, send these documents to the site investigators of all participating institutions described in the Implementation Plan, and request that the administrators of each participating institution permit conduct the research.

(vi) JCOG Operations Office shall apply for jRCT registration under the supervision of Principal Investigator after obtaining the permission by the administrators of all participating institutions described in the Implementation Plan and confirming that the patient registration system is open. "Study progress" in the trial registration will be registered as "being recruited."

(vii) JCOG Operations Office will inform Principal Investigator that jRCT registration has been completed after submission of the registration application. Principal Investigator will output the Implementation Plan from jRCT and submit the Implementation Plan, informed consent form, and Certified Review Board review result notification to the MHLW (Local Health and Welfare Bureau, which is responsible for the location of Certified Review Board; the same below). After submission, Principal Investigator will promptly inform Certified Review Board described in the Implementation Plan. In addition, Principal Investigator shall promptly report the submission of the Implementation Plan to the administrator of the institution to which he/she belongs, and provide this information to the site investigators of all participating institutions listed in the Implementation Plan and JCOG Operations Office.

(viii) After confirming that the notification of the Implementation Plan, etc. to the Local Ministry of Health and Welfare has been accepted and that jRCT's status has been updated from "under registration application" to "registration open," Principal Investigator will inform JCOG Operations Office that it has become "registration open." JCOG Operations Office opens JCOG Data Center's patient registration system and provides trial initiation announcements to study group. The date of jRCT publication is the start date of the study. "】

JCOG Operations Office should be contacted for procedures involving the addition of participating institutions after the initial submission.

"16.X. Medical institutions" changes (adding or replacing participating institutions) correspond to changes in the content of the protocol, then the change procedure shall be performed in accordance with the "13.6.2.Procedures for

when a change in the protocol occurs after the start of the study"

2) Procedures performed by the investigators at each participating institution

Following Certified Review Board approval, the investigator at each participating institution will obtain permission to conduct the research by the administrator of the participating institution using a set of documents received from Principal Investigator (Certified Review Board review results notification and documents submitted to Certified Review Board). The site investigator shall promptly send a copy of the letter of permission of the relevant institution to the Data Center after obtaining the permission by the administrator to conduct the research.

If the study is Specified Clinical Trial under Clinical Trials Act, the site investigator who has been informed that the Implementation Plan has been submitted to the MHLW by Principal Investigator should promptly report that the Implementation Plan has been submitted to the administrator of the affiliated institution.

3) Permission to conduct research at participating institutions

The procedures for obtaining permission for conducting research from the administrator of the affiliated institution shall be in accordance with the regulations of each institution.

When a copy of the institutional approval form is sent to JCOG Data Center, either the site investigator or the site coordinator should send the copy. Original copies of the institutional approval form will be stored by the site coordinator, and copies will be stored by JCOG Data Center.

When a affiliated institution has restrictions on providing personal information, such as medical record numbers, when sending copies of the institutional approval form to JCOG Data Center, the site coordinator shall communicate that personal information cannot be provided, and also send documents, such as the corresponding table of the ID number for registration and the medical record number, which is specified in JCOG privacy policy.

In addition, the informed consent form for patients approved by Certified Review Board may not be modified except for the contact information of the institution or prespecified selection items. A common protocol will be used for all institutions, since no changes in the protocols will be permitted for each institution. If it is necessary to change the content of the protocol and informed consent form, consult with Principal Investigator and Study Coordinator if the administrator of the institution asks for modification of the protocol and the text of the informed consent form in order to make the change as the protocol and informed consent form used by all institutions.

13.5.2. Procedures for changing the study plan after the start of the study

1) Procedures performed by Principal Investigator

If any of the changes of (1), (2), or (3) below occurs in the conduct of this study from the <Procedures from the initial application to the commencement of the study> in "13.6.1. Procedures at the time of new application", Principal Investigator shall hear Certified Review Board's opinions by applying for a change to Certified Review Board.

The procedures for applying for changes to the Certified Review Board are in accordance with "13.6.1. Procedures at the time of new submission" in the procedures from initial submission to the start of the study in ②, ③, and ④, respectively. In the absence of a change in the Implementation Plan, notification to the MHLW is not necessary. In the event that a change in the Implementation Plan occurs, notification to the MHLW is required. If Principal Investigator becomes aware of the planned change in the Implementation Plan, he or she should promptly contact JCOG Operations Office. JCOG Operations Office will assist in the application procedure for change to Certified Review Board and registration in change to jRCT.

（1） When the content of the protocol or informed consent form is changed (when it is amendment or revision in 13.7.1.).

（2） When the protocol or informed consent form is not changed, but the Implementation Plan (registered in jRCT) is changed.

（3） When changing Conflict of Interest Management Standards or Conflict of Interest Management Plans

Because notification of changes in the Implementation Plan (registered in jRCT) to the MHLW except for changes in research progress must be done in advance, Principal Investigator should inform site investigators and JCOG Operations Office of the change including the replacement of the investigator or the subinvestigator at each

participating institution. In addition, inform other investigators as soon as there are any changes that need to be made known.

Principal Investigator shall promptly report to the administrator of the institution to which he/she belongs and provide information to other research investigators when informed by Certified Review Board.

Procedures when it is necessary to notify changes to the Certified Review Board and the MHLW before changes are made.

1. Changes to the Implementation Plan

If changes to any of the above (1), (2), or (3) are made to the Implementation Plan (registered in jRCT) after hearing Certified Review Board's opinions, JCOG Operations Office should enter changes to jRCT under the supervision of Principal Investigator. In addition, Principal Investigator submits the following notification to the MHLW. After the date of coming into force of the predefined changes, the study shall be conducted in accordance with the changes.

- Notification of Change in Implementation Plan (Form No.2 of the Ministerial Ordinance)
- Implementation Plan after the change (output of the change in jRCT)
- Certified Review Board Review Results Notification

※ On institution transitioning to JCOG Collaborating Institution by replacement of participating institution: For institutions where patients were not enrolled prior to transfer to collaborating institution, Principal Investigator should remove it from the participating institutions by notifying changes in the Implementation Plan.

Procedures when it is necessary to notify a change to the Certified Review Board and the MHLW after the change***

1. Change in progress

(2) Among the changes in the Implementation Plan (registered in jRCT), the change in "3. Issues related to checking the implementation of Specified Clinical Trials (2) Specified Clinical Trials progress" shall be made without delay after the change. Under the supervision of Principal Investigator, JCOG Operations Office shall change "Research Progress" in jRCT. Subsequently, Principal Investigator will promptly apply for a change to Certified Review Board. After obtaining approval from Certified Review Board, Principal Investigator will submit the following notification to the MHLW.

- Notification of Change in Implementation Plan (Form No.2 of the Ministerial Ordinance)
- Implementation Plan after the change (the content of the change in jRCT was outputted)
- Certified Review Board Review Results Notification].

2. Changes in Implementation Plan after registration of the first patient

At the time of the initial submission, the "date of registration of the first patient" in the Implementation Plan is provided in a blank space. The Implementation Plan should be changed without delay after registration in the first patient. In this instance, JCOG Operations Office enters and registers the "First Patient Registration Date" in jRCT under the supervision of Principal Investigator. Subsequently, Principal Investigator will promptly apply for a change to Certified Review Board.

- Notification of Change in Implementation Plan (Form No.2 of the Ministerial Ordinance)
- Implementation Plan after the change (output of the change in jRCT)
- Certified Review Board Review Results Notification]

3. Minor changes specified by MHLW ordinance (application for change to Certified Review Board is not required)

If the following minor changes are made to Implementation Planning and the registration of jRCT, Principal Investigator will not need to hear the opinions of Certified Review Board and will notify Certified Review Board of the changes within 10 days of the date of the change. In addition, a notification (Form No.3) shall be submitted to the MHLW.

Scope of minor changes to the Implementation Plan of Article 42 of Clinical Trials Act Enforcement Regulations

- A change in the name of a person engaged in Specified Clinical Trials that does not involve a replacement of the person engaged in Specified Clinical Trials.
- Changes due to a change in the name or address number of the area

2) Procedures performed by the investigators at each participating institution

If any changes occur in the part of the protocol that corresponds to the institution to which he/she belongs, (4) Items related to investigators in multi-institutional studies, inform Principal Investigator and JCOG Operations Office of the changes to be made prior to the changes. Depending on the content of the change, the investigator shall confirm the confirmation of institutional requirements, prepare documents for conflicts of interest and submits to Certified Review Board such as lists of subinvestigators, and inform Principal Investigator and JCOG Operations Office.

The content of the most recent Implementation Plan for the institution to which they belong (the same as the content of jRCT registration) should be checked on jRCT website (https://jrct.niph.go.jp/).

13.5.3. Review and approval of study progress and study continuation (periodic reports)

Principal Investigator will report periodic reports on the progress of the study, the occurrence of adverse events, and conflict of interest management (see 13.X.1.⑱) to the administrators of their institutions and report them to Certified Review Board. Within two months after the expiration of each year from the date of submission of the Implementation Plan to the Minister of Health, Labour and Welfare.

When the report is made to Certified Review Board, Principal Investigator shall promptly inform the investigators of other participating institutions of the fact. The investigator who receives the information shall promptly report the details of the information to the administrator of the institution to which he/she belongs.

If the study falls under Specified Clinical Trials above Clinical Trials Act, Principal Investigator will report to the MHLW regarding the implementation status of Specified Clinical Trials. Periodic reports to Certified Review Board shall be made within 1 month of the date on which the results on the appropriateness of continuation of the relevant Specified Clinical Trials are obtained. The report shall be made by submitting the attached Form 3※ to the Minister of Health, Labour and Welfare.

※ Enforcement of Clinical Trials Act Enforcement Regulations, etc. (Notification No. 0228-1 of the Sector of Economics, Ministry of Health, Labour and Welfare, Notification No. 0228-1 of the Notification No. 1 of the Evaluation and Development Division, Health Policy Bureau, Ministry of Health, Labour and Welfare, dated February 28, 2018)

13.6. Protocol revision/amendment

13.6.1. Categorization of protocol changes and procedures for changes

For a change in the protocol, the Protocol Revision Application must be submitted to Data and Safety Monitoring Committee Office prior to submission to Certified Review Board [in the case of Specified Clinical Trials, add the following: "and report to the MHLW"] (see 13.6.2).

JCOG deals with the changes in the content of the protocol after approval by the Protocol Review Committee, divided into amendment and revision. Data and Safety Monitoring Committee Office will distinguish between amendment and revision, so all of applications are submitted as revision. If it is classified as an amendment by the Secretary-General of Data and Safety Monitoring Committee, it is reviewed by Data and Safety Monitoring Committee. If it is classified as a revision, Secretary-General of Data and Safety Monitoring Committee will issue a verification form and will not be reviewed by Data and Safety Monitoring Committee. We also distinguish the addition of supplementary explanations that do not fall into a change in protocol content as "Memorandum". Definitions and handling are as follows:

When the protocol or informed consent form is revised or amended, Principal Investigator will promptly distribute the most recent protocol or informed consent form to those involved in the study. Individuals involved in the study should always conduct the study in accordance with the most recent protocol since the dates of entry into force of the amendment/revision.

1) Amendment

Partial protocol change which meets one or more of the followings: i) Potential to increase the risk of patients enrolled in the study, ii) Having substantial effects on primary endpoint of the study, iii) having essential effects on the study's implementation structure.

The amended version of the protocol and informed consent form version numbers are shown as in 2.0.0, 3.0.0, and 4.0.0….

Approval by the Group Chair and the Data Center Director must be obtained prior to submission to Data and Safety Monitoring Committee.

When classified as "amendment" by the Secretary-General of Data and Safety Monitoring Committee, review of changes by Data and Safety Monitoring Committee is performed prior to Certified Review Board review. After the protocol amendment has been approved by Data and Safety Monitoring Committee, an application for a change in the protocol will be submitted to Certified Review Board through JCOG Operations Office (at this time the version number is ver. 2.0.0, 3.0.0, 4.0.0…). When the protocol was changed based on the review opinions of Certified Review Board, the version number is ver. 2.0.1, ver.2.0.2… If the protocol is changed by review of Certified Review Board, the change will be reported to Data and Safety Monitoring Committee, but in principle, the change will not be reexamined by Data and Safety Monitoring Committee. When a protocol change is approved by Certified Review Board, the protocol cover page should include the date of approval by Data and Safety Monitoring Committee and Certified Review Board [If Specified Clinical Trials, the following is added: "and a notification of the change in the Implementation Plan to the Regional Bureau of Health and Welfare should be made"].

Following Certified Review Board approval, permission for the contents of the amendment by the administrator of each institution shall be obtained. If permission is obtained, the site coordinator of each institution will send copies of the permission notice by the administrator of each institution to the Data Center. After the permission of the administrator of all institutions is obtained, the protocol changes will come into effect (during this time, the patient registration will not be suspended unless there is a special need). The actual date of entry into force will be announced by JCOG Operations Office. Researchers in all participating institutions conduct the study according to revisions approved by Certified Review Board since the date of entry into force.

Treatment and assessment of enrolled patients will be performed according to the pre-change version protocol until entry into force. Protocol deviations to enhance patient safety during treatment will be permitted if pre-change protocols, such as inadequate treatment modification criteria, threaten patient safety. If protocol deviations occur, they should be listed in the monitoring report.

2) Revision

Protocol change which meets all of the followings: i) does not have an increased risk for patients enrolled in the study; ii) does not have a substantial effect on primary endpoint of the study; iii) does not have an inherent effect on the system in which the study is conducted. Includes changes in protocols due to mistakes or changes in institution-specific information, changes in institution-specific information without changes in protocols (changes in Implementation Plans and jRCT registrations), and changes in conflicts of interest at individual institutions. In principle, suspension of patient registration is not performed in case of revision.

The revised version of the Protocol and Informed Consent Form version numbers are shown as in 1.1.0, 1.2.0, and 1.3.0….

Approval by the Group Chair and the Data Center Director must be obtained prior to submission to Data and Safety Monitoring Committee.

If classified as "Revision" by the Secretary-General of Data and Safety Monitoring Committee, the Secretary-General of Data and Safety Monitoring Committee issued a verification form, Data and Safety Monitoring Committee did not review the changes. The Principal Investigator submit an application for a change in the protocol to Certified Review Board through JCOG Operations Office (at this time, the version number is ver.1.1.0, 1.2.0, 1.3.0…). If the protocol was changed based on the indication by the Certified Review Board, version number shall be as ver.1.1.1, ver.1.1.2… When a protocol change is approved by Certified Review Board, the date of approval by the Certified Review Board should be entered on the protocol cover page. [If Specified Clinical Trials, the followings are added: "and a notification of the change in the Implementation Plan to the Regional Bureau of Health

and Welfare should be made"]

The date of entry into force of the protocol change will be 2 weeks after Certified Review Board approval date unless otherwise specified. [in the case of a Specified Clinical Trials, the followings are added: "The effective date shall be the date after the notification of change of the Implementation Plan to the Regional Bureau of Health and Welfare"]

The actual date of entry into force will be announced by JCOG Operations Office. Researchers in all participating institutions conduct the study according to revisions approved by Certified Review Board since the date of entry into force.

At that time, the site investigator should obtain permission by the administrator of each institution after the date of approval of Certified Review Board and before the date of entry into force. In this case, the protocol revision may be permitted with a report to the administrator of the institution. However, the procedures for obtaining permission from the administrator shall be in accordance with the regulations of each institution. For protocol revisions, confirm with the institution prior to initiation of the study to ensure that permission is obtained within the aforementioned time periods, and contact JCOG Operations Office if this is difficult. Reports to the administrator and approval form by the administrator at each institution need not be sent to the Data Center, but the original copy will be retained by the site coordinator as it will be checked during site visit audits.

Treatment and assessment of enrolled patients will be performed according to the pre-change version protocol until entry into force. Protocol deviations to enhance patient safety during treatment will be permitted if pre-change protocols, such as inadequate treatment modification criteria, threaten patient safety. If protocol deviations occur, they should be listed in the monitoring report.

3) Memorandum

Supplementary description of the protocol distributed from Principal Investigator/Study Coordinator to study personnel in objective, such as reduction of interpretive variation in sentences, and special precautions, rather than change of protocol content. Any form is used.

Approval by Group Chair and the Director of the Data Center is needed prior to distribution. Reporting to Data and Safety Monitoring Committee before distribution or immediately after distribution is required.

It is not necessary to include the protocol on the cover page.

13.6.2. Patient explanation and re-consent at the time of protocol amendment/revision

In the event of a change in the content of the study, the investigator or the subinvestigator shall provide appropriate explanation to the enrolled patients (regarding protocols based on revision, treatment, follow-up, etc.). In addition, if Certified Review Board comments that re-consent of enrolled patients in writing is required, informed consent should be obtained in writing.

13.7. Conflicts of Interest (COIs) involved in this study

13.7.1. COI management involved in this study

The COIs involved in this study will be managed according to the "Guidance for Conflict of Interest Management in Clinical Trials Act (Notification No.1130-17 of the PMSB dated November 30, 2018) ※ (Guidance) of the Division, Research and Development, Ministry of Health, Labour and Welfare, in accordance with the following:

※ http://www.mhlw.go.jp/stf/seisakunitsuite/bunya/0000163417.html

The format used for COI control should be the latest version of the guidance.

- Conflict of Interest Management Standards: Form A
- Reports of related companies, etc.: Form B
- Investigator Conflict of Interest Self-Report Form: Form C
- Conflict of Interest Confirmation Report: Form D
- Conflict of Interest Management Plan; Form E

1) Procedures for New Application

Conflict of Interest Management Standards

(i) All JCOG trials according to Clinical Trials Act will employ Conflict of Interest Management Standards (Form A) in accordance with guidance.

Request for Confirmation of Conflicts of Interest

(ii) At the time of initiation of the primary review of the protocol, JCOG Operations Office shall identify companies, etc. related to this study that require conflict of interest management based on information on drugs and medical devices specified in the Protocol Treatment (see 6.1.), enter the relationship with the company in Form B, and ask Principal Investigator to confirm the accuracy of the entry.

(iii) After checking the entries in Form B received from JCOG Operations Office, Principal Investigator will send a format to the site investigator and site coordinator at each participating institution and ask to confirm the entries in Form B, create Form C, and create Form E. In addition, a form shall be sent from Principal Investigator to those who correspond to the "person responsible for statistical analysis" and the "administrator other than Principal Investigator and Investigators" of the Implementation Plan, and they shall be asked to prepare Forms C and E.

Confirmation of Conflicts of Interest (participating institutions)

(iv) The site investigator or site coordinator at each participating institution will register the investigators, subinvestigators, and research associates involved in the study in JCOG Web Entry System. Investigators and subinvestigators registered in JCOG Web Entry System will be the reporters of conflicts of interest in the study.

(v) The site coordinator will request the investigator of the study to confirm the description of Form B received from Principal Investigator, create Form C, and create Form E. The investigator will then summarize conflict of interest management within the study site.

(vi) The investigator checks the forms received from Principal Investigator for any relevant items in Q2 through Q5 of Form B and notifies Principal Investigator through JCOG Operations Office within a week if any.

(vii) In the column of Form C [Persons requiring a Form C self-declaration of conflict of interest (Form C)] ※, the site investigator inputs the information of the investigator and subinvestigator registered in JCOG Web Entry System, and asks subinvestigator to prepare Form C.

※ The information entered in Form C is automatically entered in Form E. This entry should be consistent with the physician's information contained in the "List of Subinvestigators" (Clinical Trials Act Uniform Form No.1) submitted to Certified Review Board with Form E, and physicians who do not agree may not be involved in this study. Therefore, when entering the information in the ※ column for [those who require a Conflict of Interest Self-Reporting Form (Form C)], the investigator or subinvestigator list should be downloaded in JCOG Web Entry System and the physician listed in the Study Subinvestigator list should be entered without missing the person who will be the investigator and the subinvestigator in the study. If the information in the downloaded "Study Subinvestigator List" is not consistent with the physician involved in the study, the registered information should be updated in JCOG Web Entry System, and the "Study Subinvestigator List" with the most recent information reflected should be downloaded and used.

(viii) The investigator and the subinvestigator shall complete the necessary information regarding the relationship with the company, etc. described in advance in Form C, and submit Form C to the Conflict of Interest Confirmation Department of the institution to which he/she belongs. In doing so, the investigator also submits Form A.

(ix) The investigator receives the results of confirmation of conflicts of interest (Form D) of the investigator and the study subinvestigator from the institution to which he/she belongs.

(x) The investigator will confirm the content of Form A, Form B, and Form D of the investigator and all study subinvestigators to create Form E. The investigator will notify Principal Investigator through JCOG Operations Office of Form E and the Study Subinvestigator List downloaded in Form 7.

Confirmation of Conflicts of Interest (outside participating institutions)

(xi) The person responsible for statistical analysis and the person overseeing a study other than Principal Investigator or investigator shall complete, in the form received from Principal Investigator, the necessary information regarding the relationship with the company, etc. described in advance in Form C, and submit Form A and Form C to the Conflict of Interest Confirmation Department of the Affiliated Institution.

(xii) The person responsible for statistical analysis and the person overseeing studies other than Principal Investigator or investigator shall receive confirmation results (Form D) from the institution.

(xiii) The person responsible for statistical analysis and the person overseeing studies other than Principal Investigator and investigator shall confirm the content of Form A and Form D, prepare Form E, and notify Form E to Principal Investigator through JCOG Operations Office.

Description of Conflicts of Interest in the protocol and informed consent form

(xiv) Principal Investigator and JCOG Operations Office will review Form A and Form E received from participating institutions and, as appropriate, will accurately describe the conflicts of interest (study COIs) between the study and the drug marketing authorisation holder, etc. in the protocol and informed consent form. Conflicts of interest (personal COI) between the reporters of conflicts of interest in this study and the marketing authorisation of drugs, etc. (personal COI) can change over time, so the personal COI is not described in the protocol and informed consent form, and the information is updated on JCOG website as needed.

Certified Review Board review

(xv) Principal Investigator compiles and submits the Form E and Study Subinvestigator List of all institutions to Certified Review Board for review.

2) Procedures for new involvement with companies after the start of the study

(xvi) When a new involvement with companies (research COI) occurs in the study after the start of the study

a. In the event of any change in the enterprises involved in this study, etc. to be described in Q1 of Form B, repeat the procedures ② to ⑭. However, the site investigator and site coordinator roles in ④, ⑤ shall be assumed by the investigator at each institution. If a new study COI needs to be added to the protocol and informed consent form, a protocol revision (see 13.X.X.) should be submitted to Certified Review Board for review.

b. If a change occurs from Q2 to Q5 in Form B, the investigator at the participating institution affected by the change will change the appropriate description in Form B and update Form E and send it to Principal Investigator and JCOG Operations Office. A Principal Investigator who has received a Form E will revise the protocol as needed (see 13.X.X.) and submitted to Certified Review Board for review.

(xvii) In the event that a conflict of interest reporter is newly involved with a company (personal COI) after the start of the study

Conflict of interest reporters repeat procedures from ⑧ to ⑩ or from ⑪ to ⑬. However, if there is no change in Form E, these procedures will be performed at each institution but will not be sent to Principal Investigator and JCOG Operations Office. Principal Investigator will submit Post-change Form E sent from the site investigator to Certified Review Board for review. Personal COIs are also disclosed on JCOG website.

3) Procedures for periodic reporting

(xviii) Principal Investigator checks for changes in study COI and personal COI annually at the timing of periodic reporting and reports to Certified Review Board.

13.7.2. COI with companies involved in this study(study COI)

There are no conflicts of interest to disclose about relationship between the company manufacturing and marketing drugs specified as a part of protocol treatments in this study (See 6.1).

13.7.3. COIs of JCOG Committee members and JCOG Data Center/Operations Office staff

COIs of the committee members of JCOG committees, the staffs of the office of committees, and JCOG Data Center/Operations Office staff involved in the study will be managed by JCOG Conflict of Interest Committee in accordance with Clinical Trials Act control standards.

13.8. Compensation

In order to comply with Clinical Trials Act, this study must take necessary measures such as sing up for insurance and ensuring a system to provide medical care in order to compensate for the health damage caused by the study

and provide medical care.

Therefore, regarding the health damage caused by participating in this study, appropriate treatment according to the condition is provided similarly to the usual insurance medical care. In addition, this study will be covered by clinical study insurance, and the following will be compensated based on insurance conditions, and this will be explained to patients and understood.

13.9. Intellectual property

The results, data, and intellectual property rights obtained from this study are attributed to the followings: National Cancer Center, Principal Investigator, Study Coordinator, and Group Chair. Specific procedures and allocation must be determined through consultation among four parties. Whether the intellectual property related to Principal Investigator, Study Coordinator, Group Chair will belong to the individuals or the affiliated institution will be determined according to the agreements of the affiliated institution.

13.10. Disclosure of information on this study

Summary, progress, and main results of this study will be published on JCOG website (www.jcog.jp) and on jRCT (https://jrct.niph.go.jp/).

14. Monitoring and audit

14.1. Periodic monitoring

In this study, monitoring is performed in order to ensure that the study is conducted safely and in accordance with this protocol and that data are collected accurately. Periodic monitoring should be performed twice a year in principle, using the entered data on the CRFs collected in the Data Center. Specific procedures for periodic monitoring are provided separately in the Monitoring Plan.

The Data Center submits a "Monitoring Report" summarizing the results of central monitoring to Principal Investigator, Study Coordinator and investigators. Together, they are submitted to Group Chair, Data and Safety Monitoring Committee, and JCOG Chair.

The Monitoring Report is a material for periodic reports to be made every year from the date of submission of the Implementation Plan.

14.1.1. Monitoring items

① Registration status: number of registration - cumulative/by month, by arm/site
② Eligibility: ineligibles/potentially ineligible cases: by arm/site
③ Pre-treatment baseline factors: by arm
④ On/off-treatment, reason for treatment termination: arm/site
⑤ Protocol deviation: arm/site
⑥ Serious Adverse Events: arm/site
⑦ Adverse reaction/event: arm
⑧ Overall survival, progression-free survival (or relapse-free survival, etc.): all registered patients
⑨ Other issues related to study progress and safety (studies in accordance with Clinical Trials Act: status of occurrence of non-compliance and subsequent response, number of subjects for compensation, number of disease or the like reports in accordance with Article 13 of Clinical Trials Act)

14.1.2. Eigibility (Eligible/Ineligible)

For all registered patients, eligibility will be classified according to the following definitions as either: In monitoring, Data Center lists potentially ineligible cases in the "Evaluation of Eligibility" section of the monitoring report, and after review by Study Coordinator, confirms them to be either 1), 2), 9), or 99) with Group Chair approval prior to performing primary analysis.

Only 1) eligible shall be "eligible case", 2) post hoc ineligible,9) de facto ineligible and 99) violation of registration shall be "ineligible case". This is a category established from the perspective of analysis set setting.

In the study in accordance with Clinical Trials Act, "99) violation of registration" will be regarded as "major non-compliance" in Clinical Trials Act, and Principal Investigator will promptly report the situation to Certified Review Board as soon as possible. See 14.3. for management of non-compliance.

9) de facto ineligibles corresponds to "non-compliance" on Clinical Trials Act and is reported to the administrator of participating medical organizations with the submission of the monitoring report on which they were described (twice a year).

2) Post hoc ineligibles is not treated as "non-compliance" on Clinical Trials Act because it does not correspond to non-compliance with the study protocol, as discussed below.

1) Eligible

All information generated prior to registration meets all o the Patients Selection Criteria according to the methods and criteria specified in the study protocol.

2) Post hoc ineligible

The information generated after registration does not meet either Patients Selection Criteria, or the information generated prior to registration does not meet either Patients Selection Criteria by methods or criteria other than those specified in the protocol.

Examples)

(i) In the study for Stage II-III, bone scintigraphy performed immediately after registration revealed bone

metastases, and the patient was diagnosed as Stage IV. The protocol treatment was terminated.

(ii) In the study for early gastric cancer, bloody stools is seen after registration, and colonoscopy revealed advanced colorectal cancer (synchronous double cancer). Colectomy was performed after termination of the protocol treatment.

(iii) In the study for gastric cancer (adenocarcinoma), the institution's pathological diagnosis was changed to malignant lymphoma after registration.

9) De facto ineligible

Information generated prior to registration according to protocol-specified methods (performed in all cases) and criteria does not meet either Patients Selection Criteria. This includes cases where it is determined after registration that the information that occurred before registration had been incorrect.

Example: When the supervising physician reviews the CT images performed before registration as specified, there is obvious liver metastasis (if it is a mistake by the attending physician and it is considered that there is no future).

99) Violation of registration

Deliberately (falsely) enroll while knowing that Patients Selection Criteria is not met. Corresponds to a misconduct and treats it as a serious problem.

14.1.3. Protocol Deviations/Violations

Protocol deviations are defined as those in which treatment, such as drug administration, radiotherapy, or surgical resection, as well as laboratory tests and evaluation of toxicity and efficacy, etc. were not performed according to the protocols.

In monitoring, deviations that exceed a certain acceptable range limit for each study decided by the Data Center and Principal Investigator/Study Coordinator prior to or after the initiation of the study are listed in the monitoring report as "possible deviations" and are classified into one of the following categories after consideration by Study Coordinator and study groups: Except for those described in the protocol and monitoring report, the acceptable deviations agreed between the Data Center and Principal Investigator/Study Coordinator may be changed through periodic monitoring during the study, so they should be described in the supplemental material rather than in the text of the Monitoring Plan, and the Monitoring Plan should be described as "defining the acceptable ranges separately".

1) Violation

Any deviation from the protocol that is clinically inappropriate and caused by the treating physician/institution and that meets two or more following criteria shall be classified as a violation. [When conducted in accordance with Clinical Trials Act, the following shall be added. [In the study according to Clinical Trials Act, the violation shall be treated as "major non-compliance" and Principal Investigator should report the situation to Certified Review Board as soon as possible].

① Have a substantial impact on the assessment of study endpoints

② Intentional or systematic

③ Dangerous or remarkable deviation

For "violations", in principle, the content of each violation should be described in a paper when publishing.

2) Deviation

Deviations that do not fall into 1) violation or 3) acceptable deviation. If same kind of deviations are frequent, they should preferably be included in the publication of the article. They are classified as either of the following at the time of monitoring report review:

Because deviations correspond to "non-compliance" in Clinical Trials Act, they are reported (biannually) to the administrators of the institution with the submission of the monitoring report. Not to be "major non-compliance".

(i) Deviations - Undesirable and to be reduced

(ii) Deviations (unavoidable) - things that are not proactively reducing (e.g. delay by the New Year period, equipment breakdown, etc.)

(iii) Deviations (clinically relevant) - Those in which the decision of the treating physician/institution are positively affirmed (if a similar situation again arises similar deviations are considered desirable).

※ Deviations do not always mean that the treating physician at the institution is problematic. Since patient safety is a primary priority in clinical trials, deviations should rather be made by the medical judgment of the treating physician if the condition of the individual patient are considered to be dangerous when following the protocols. If the deviation is judged to be clinically relevant for the safety of the patient, it is recorded as ③ Deviation (clinically relevant). Clinically relevant deviations in a small number of patients are not required to be particularly problematic; however, protocol revision should be considered when multiple deviations occur because protocol specification is likely to be inadequate. However, deviations that are not intended to be safety (e.g., increased doses of anticancer drugs in the hope of increasing efficacy, shortened treatment periods not specified in the protocol) are not considered clinically relevant deviations.

3) Acceptable deviation

Deviations from protocols within acceptable range agreed by the entire JCOG, study groups, or Study Chair/Study Coordinator and Data Center, pre- or post-study initiation, on a trial-by-trial basis. When conducted according to Clinical Trials Act, the following are added. Not considered to be "non-compliance" in Clinical Trials Act. Deviations within the pre-specified acceptable ranges are not included in the monitoring report.

14.2. Site visit audits

In this study, site visit audits will be conducted to ensure the reliability of clinical research and the reliability of data and information collected by clinical research from the perspective of protecting human subjects in clinical research.

Site visit audits are conducted by auditors appointed by Principal Investigator by visiting a medical institution to confirm the approval documents of the medical institution, check the list of subinvestigators in the research institution, confirm the informed consent documents, and verify CRF entry data with medical records (direct access to source documents). Specific procedures for site visit audits are provided separately in the operating procedures.

The auditor shall report the audit report summarizing the audit results to Principal Investigator/Study Coordinator, site investigators. Together, the report will be submitted to Group Chair, Director of JCOG Data Center, Director of JCOG Operations Office, and JCOG Chair. Reports should also be submitted to the site investigators of the relevant groups and JCOG Executive Committee as appropriate.

14.2.1. Items to be audited

In the site visit audits, the following items are checked by direct access to source documents:

<Confirmation Items by Study>

- Approval documents (including initial approval forms, revision approval forms, and annual report approvals/reports) from administrators of medical institutions
- Management status of the protocol
- Contents of explanatory documents and informed consent forms

<Confirmation Items by Patient>

- Patient consent (presence or absence of consent form, signature, and date of consent)
- Implementation of pre-registration mandatory tests, eligibility for registration (inclusion criteria and exclusion criteria)
- Accuracy of reported data
  - ➢ Pre-treatment evaluation, course of treatment (protocol treatment)
  - ➢ Various test results (including diagnostic imaging reports and pathology reports)
  - ➢ Accuracy of test date, response evaluation, adverse event, survival or death information, and other reported data

<Other items>

- Presence or absence of study misconduct (possibility of false reporting, fabrication, or falsification)
- Improvement status of the items pointed out in the previous audit

14.2.2. Reporting of major non-compliance found in audits to Certified Review Board

Principal Investigator/Study Coordinator should report to Certified Review Board immediately when finding possible "major non-compliance (See 14.3.2.)" as a result of site visit audits.

14.3. Management of non-compliance

14.3.1. Non-compliance.

Non-compliance in Clinical Trials Act refers to the condition in which clinical research is not compliant with Clinical Trials Act Enforcement Regulations or study protocols. In the Clinical Trials Act Enforcement Regulations, etc. (February 28, 2018), non-compliance with regulations, study protocols, operation procedures, etc., and fabrication of falsification of research data, etc. are listed as examples.

If the site investigator knows that there is non-compliance, the site investigator should report to the administrator of the medical institution and inform Principal Investigator/Study Coordinator.

If site investigator find non-compliance (regardless of major non-compliance or not) prior to the implementation of central monitoring or site visit audits, the site investigator will promptly report to Principal Investigator/Study Coordinator and JCOG Data Center.

"14.1.3.2) Deviations" correspond to "non-compliance" in Clinical Trials Act. As described in 14.1.3.2), these deviations are reported by submitting monitoring reports or their excerpts or summaries issued twice a year to the administrators of the medical institution.

Changes in protocols and implementation plans associated with investigator transfer require Certified Review Board review and notification of implementation plans to the MHLW, therefore, require a certain time to complete the sequence of procedures. In addition, it is often difficult to complete the change procedure before the transfer because the transfer may not be open until just before the transfer. Therefore, even if the site investigator is absent for a certain period of time due to the transfer, the study does not fall into "non-compliance" in this study if the research management system is maintained by the subinvestigator and the medical care system of the enrolled patients who are surviving is ensured.

14.3.2. Major non-compliance

"Major non-compliance" is that affect the human rights and safety of subjects of clinical research, the study progress and the reliability of study results. Examples of "major non-compliance" in JCOG study are provided in. If major non-compliance is likely, Principal Investigator/Study Coordinator will report the situation to Certified Review Board immediately.

1) Major non-compliance with respect to eligibility

Violation of registration

- Enrolled intentionally (falsely) while knowing that eligibility criteria was not met
- Patient enrollment was performed without necessary informed consent, and protocol treatment was performed
- The source documents for the determination of eligibility cannot be identified (including the loss of the consent form).

2) Protocol violation

Violations that affect the increased risk of enrolled patients or that affect the reliability of the study results

- Significant violation in inclusion criteria or exclusion criteria
- Violation threatening patient safety in off-treatment criteria
- Serious violation of prohibited concomitant drug, prohibited concomitant treatment, etc.
- e.g. intentional or systematic non-compliance with protocol regulations

3) Other major non-compliance

- Study was conducted prior to Certified Review Board approval or prior to approval of site administrator
- The study was continued without providing information to the enrolled patient that could affect the willingness to continue to participate the study.
- Those judged to be research misconduct (fabrication or falsification of data, etc.)

- Any leakage of personal information or violation of human rights that may have a significant impact on the enrolled patient.

15. Special Instructions

15.1 Central pathology diagnosis and related matters

15.1.1 Central pathological diagnosis

In this study, the pathological tissues of enrolled patients will be collected and the central pathological diagnosis will be determined after the fact. Since the central pathological diagnosis is not performed in real time for each individual patient enrollment, the main analysis target and the decision on treatment strategy will be based solely on the pathological diagnosis at the institution. The details of the central pathological diagnosis procedure will be specified separately in the Central Pathological Diagnosis Procedure Manual.

Timing: Once a year throughout the study period.

Subjects: All registered patients

Methods: Pathological specimens (or duplicates made from the same paraffin block) used for eligibility criteria determination at the registered facilities will be collected, and after necessary staining (chromogranin A, synaptophysin) is added, pathological eligibility will be re-determined by two or more pathology judges (16.8 Pathology judges) appointed by the group representative. (16.8 Central Pathological Review Comittie).

Management of the collected specimens: The Hepatobiliary and Pancreatic Group Study Office will be responsible for the management of the collected specimens.

Staining: If additional staining is required, it will be performed by the Central Pathology Coordinator (16.7).

Notification of the central judgment to each facility:

After the results of the central pathological diagnosis are fixed, the research secretariat will notify the results of the judgment to the registered facility (facility coordinator) of each patient. At that time, the rationale for the decision should be attached in writing. If there is a difference between the institutional diagnosis and the central diagnosis, the facility principal investigator/facility coordinator reports the decision results to the facility pathologist and discusses it with the facility pathologist, and carefully decides on the final pathological diagnosis at the facility (whether to change the diagnosis or not) and what to do if the patient is under treatment (whether to change the treatment or not).

15.1.2 Providing information to the institutional pathologist

In view of the high level of difficulty in NEC pathological diagnosis and the unique nature of this study, which covers multiple target organs, the following information will be provided to institutional pathologists.

(1) Pre-registration pathological diagnosis consultation

Since it is anticipated that there will be cases of confusion in diagnosis at facilities, pre-enrollment pathology consultation will be available as needed, with the pathology judgment committee members of this study serving as consultants. Details of the consultation operation are described in the Central Pathology Procedures.

(2) Holding of pathology-related meetings

In this study, the research secretariat and the pathology secretariat first held a pathology-related meeting (attended by pathologists from participating institutions) on February 11, 2014, during which a lecture was given to form a consensus on diagnostic criteria according to the WHO 2010 classification. The slides used at the meeting were revised as necessary based on the discussion at the meeting and posted on the JCOG website for reference by pathologists at each facility.

15.1.3 Response to cases in which pathological diagnosis was performed at a facility other than the home facility

In any of the following cases, be sure to ask a pathologist at your own institution to make the diagnosis, and confirm that the diagnosis at your institution is also NEC before enrolling in this study.

(1) When borrowing only stained tissue specimens from a previous physician for registration

Register after obtaining permission from the attending physician to the previous physician to submit the borrowed tissue specimen to JCOG 1213 for central diagnosis (loan again) and to borrow the specimen for a long period of time (up to about 1 year).

(2) If you have received virtual slides of "all tissue specimens for which a pathological diagnosis has been made" from

your previous doctor

The patient will be registered after obtaining permission from the attending physician of the previous physician to submit the virtual slides provided by the previous physician for the central diagnosis of this study.

(3) If you have received a "borrowed" pre-stained tissue specimen and an "offered" unstained preparative from your previous physician

The borrowed stained tissue specimens from the previous physician can be promptly reviewed at the patient's own institution, but the provided unstained preparations require time for pathological diagnosis after immunostaining at the patient's own institution, which may cause a time lag. There have been cases in which patients were enrolled in the study based only on the results of pathological review of borrowed tissue specimens at their own institutions, and later the results of staining and pathological diagnosis of undyed preparations at their own institutions overturned the pathological diagnosis (diagnosis of a different disease was made), resulting in post-hoc ineligible cases. Therefore, we will consider the timing of registration after sufficient consultation with pathologists at our own institution to avoid the occurrence of posterior ineligible cases as much as possible. However, if there is enough time, it is preferable to enroll unstained preparations into the study after staining and pathological diagnosis is obtained at your own institution.

15.2. JCOG BioBank Japan (BBJ) Biorepository

This study will participate in the banking of blood samples (DNA/plasma) in JCOG BBJ Biorepository based on a common protocol for all JCOG studies (hereafter referred to as common banking).

Subjects:

Among patients who agreed to participate in this study, patients whose consent to shared banking was obtained.

Sample:

1) Whole blood

Blood sampling is performed before the start of the protocol treatment in this study in principle. However, blood sampling after initiation of protocol treatment is allowed. Blood samples of 7 mL×2 (total 14 mL) of venous blood are collected using a blood collection tube (for blood counting) with a EDTA Na dedicated to the shared banking of JCOG-BBJ Biorepository, and stored at 4°C at the respective institutions until they are handed over to the sample transport/processing company (See "JCOG-Biobank Japan Biorepository protocol" for details).

2) Pathological specimens

Archived pathological tissues in daily clinical practice such as surgery, biopsy and laboratory tests can also be used in future translational researches, but the type of pathology specimen, preparation method and tissue quantity required by the studies are varied and there is no consensus that it is efficient to bank pathological tissues in a certain way prospectively. In addition, there is the opinion that the sample deteriorates (DNA fragmentation) when the thin-section sample from pathological tissue is stored for a long time.

Consent on the use of archival pathological tissue after medical care should therefore be obtained at the time of consent to banking, but actual collection should be initiated on an individual basis by creating a protocol and defining the most appropriate procedure for the study content in the protocol.

16. Organization

Changes to this chapter are considered to be revision rather than amendment.

16.1. Main study fund (funding source) of this study.

Practical Research for Innovative Cancer Control from Japan Agency for Medical Research and Development
"Establishment of standard treatments for neuroendocrine carcinoma of the digestive system" JP15ck0106138, JP16ck0106138, JP17ck0106355, JP18ck0106355, JP19ck0106355, JP20ck0106618
National Cancer Center Research and Development Fund (23-A-22, 26-A-4, 29-A-3, 2020-J-3)
"Scientific research on multi-institutional trials to establish new standard treatment of solid tumors in adults"

16.2. Japan Clinical Oncology Group (JCOG)

JCOG is a multi-institutional clinical research group consisting of research teams funded by public research grants

mainly on National Cancer Center Research and Development Fund and Japan Agency for Medical Research and Development research costs that receive direct support for research by the Clinical Research Support Office of the National Cancer Center Hospital in accordance with JCOG Policy (http://www.jcog.jp/).

This study is conducted using JCOG research organisations and in accordance with the regulations set out by JCOG Executive Committee.

16.3. JCOG Chair

Yuichiro Ohe National Cancer Center Hospital

16.4. Study group and Group Chair

JCOG Hepatobiliary and Pancreatic Oncology Group

Group Chair: Junji FURUSE
Department of Medical Oncology, Kyorin University Faculty of Medicine
〒181-8611 6-20-2, Shinkawa Mitaka-shi, Tokyo
TEL:+81-422-47-5511
FAX:+81-422-44-1858
E-mail: jfuruse@ks.kyorin-u.ac.jp
Group Secretary: Hirohi Ishii
Clinical Research Center, Chiba Cancer Center

〒260-8717, 666-2 Nitona, 260-8717, Chuo-Ku, Chiba
TEL:+81-43-264-5431
FAX:+81-43-264-8680
E-mail: hirishii@chiba-cc.jp

JCOG Stomach Cancer Study Group:

Group Chair: Masanori Terashima
Shizuoka Cancer Center
〒411-8777 1007 Shimonagakubo, Nagaizumi-cho, Sunto-gun, Shizuoka Prefecture
TEL:+81-55-989-5222
FAX:+81-55-989-5783
E-mail: m.terashima@scchr.jp
Group Secretary: Takaki Yoshikawa
National Cancer Center Hospital
〒104-0045 5-1-1 Tsukiji, Chuo-ku, Tokyo
TEL:+81-3-3542-2511
FAX:+81-3-3542-3815
E-mail: tayoshik@ncc.go.jp
Group Secretary: Narikazu Boku
National Cancer Center Hospital
〒104-0045 5-1-1 Tsukiji, Chuo-ku, Tokyo
TEL:+81-3-3542-2511
FAX:+81-3-3542-3815
E-mail: nboku@ncc.go.jp

JCOG Japan Esophageal Oncology Group

Group Chair: Yuko Kitagawa
Keio University Hospital
〒160-8582 35 Shinanomachi, Shinjuku-ku, Tokyo
TEL:+81-3-3353-1211
FAX:+81-3-3355-4707
E-mail: kitagawa@a3.keio.jp

Group Secretary: Ken Kato
National Cancer Center Hospital
〒104-0045 5-1-1 Tsukiji, Chuo-ku, Tokyo
TEL:+81-3-3542-2511
FAX:+81-3-3542-3815
E-mail: kenkato@ncc.go.jp

16.5. Study Chair (Principal Investigator)

JCOG Hepatobiliary and Pancreatic Oncology Group
Takuji Okusaka
National Cancer Center Hospital
〒104-0045 5-1-1 Tsukiji, Chuo-ku, Tokyo
TEL: +81-3-3542-2511
FAX: +81-3-3542-3815
E-mail: tokusaka@ncc.go.jp

JCOG Stomach Cancer Study Group:
Narikazu Boku
National Cancer Center Hospital
〒104-0045 5-1-1 Tsukiji, Chuo-ku, Tokyo
TEL: +81-3-3542-2511
FAX: +81-3-3542-3815
E-mail:nboku@ncc.go.jp

JCOG Japan Esophageal Oncology Group
Ken Kato
National Cancer Center Hospital
〒104-0045 5-1-1 Tsukiji, Chuo-ku, Tokyo
TEL: +81-3-3542-2511
FAX: +81-3-3542-3815
E-mail: kenkato@ncc.go.jp

16.6. Study Coordinator

JCOG Hepatobiliary and Pancreatic Oncology Group
Chigusa Morizane
National Cancer Center Hospital
〒104-0045 5-1-1 Tsukiji, Chuo-ku, Tokyo
TEL: +81-3-3542-2511
FAX: +81-3-3542-3815
E-mail: cmorizan@ncc.go.jp

JCOG Stomach Cancer Study Group:
Nozomu Machida
Kanagawa Cancer Center
〒241-8515, 2-3-2 Nakao, Asahi-Ku, Yokohama
TEL:+81-45-520-2222
FAX:+81-45-520-2202
E-mail:n-machida@kcch.jp

JCOG Japan Esophageal Oncology Group

Ken Kato

National Cancer Center Hospital

〒104-0045 5-1-1 Tsukiji, Chuo-ku, Tokyo

TEL: +81-3-3542-2511

FAX: +81-3-3542-3815

E-mail: kenkato@ncc.go.jp

Yoshitaka Honma

National Cancer Center Hospital

〒104-0045 5-1-1 Tsukiji, Chuo-ku, Tokyo

TEL: +81-3-3542-2511

FAX: +81-3-3542-3815

E-mail:yohonma@ncc.go.jp

16.7. Central Pathological Review Coordinator

Nobuyoshi Hiraoka

National Cancer Center Hospital

〒104-0045 5-1-1 Tsukiji, Chuo-ku, Tokyo

TEL: +81-3-3542-2511

FAX: +81-3-3542-3815

E-mail: nhiraoka@ncc.go.jp

Shigeki Sekine

National Cancer Center

〒104-0045 5-1-1 Tsukiji, Chuo-ku, Tokyo

TEL: +81-3-3542-2511

FAX: +81-3-3542-3815

E-mail: ssekine@ncc.go.jp

16.8. Central Pathological Review Coordinator

Nobuyoshi Hiraoka (National Cancer Center)
Noriyoshi Fukushima (Jichi Medical University)
Nobuyuki Ohike (Showa University Fujigaoka Hospital)
Ryoji Kushima (Shiga University of Medical Science)
Mitsuya Iwafuchi (Scool of Health Sciences Faculty of Medicine, Niigata University)
Tetsuo Ushiku (The University of Tokyo)

16.9. Participating sites (participating institutions)

HBP	SCSG	JEOG	Participating institutions
◯			Sapporo-Kosei General Hospital
◯		◯	Hokkaido University Hospital
	◯		Keiyukai Sapporo Hospital
	◯	◯	Iwate Medical University
		◯	Tohoku University Hospital
	◯		Miyagi Cancer Center
◯	◯	◯	Tochigi Cancer Center
◯			Jichi Medical University
◯	◯	◯	Saitama Cancer Center
◯	◯	◯	National Cancer Center Hospital East
◯		◯	Chiba Cancer Center
◯		◯	Chiba University, Graduate School of Medicine
◯	◯	◯	National Cancer Center Hospital
◯			Kyorin University Faculty of Medicine
◯			National Center for Global Health and Medicine (NCGM)
◯	◯	◯	Cancer Institute Hospital of Japanese Foundation for Cancer Research
	◯	◯	Toranomon Hospital
◯			Teikyo University School of Medicine
◯			St.Marianna University School of Medicine
◯	◯	◯	Kanagawa Cancer Center
◯	◯		Yokohama City University Medical Center
◯	◯		Niigata Cancer Center Hospital
◯			Toyama University Hospital
◯			Kanazawa University School of Medicine
	◯		Ishikawa Prefectural Central Hospital
	◯		Ogaki Municipal Hospital
◯	◯	◯	Shizuoka Cancer Center
◯	◯	◯	Aichi Cancer Center Hospital
		◯	Kyoto University Hospital
	◯	◯	Osaka University Graduate School of Medicine
◯	◯		Kindai University Hospital
◯	◯	◯	Osaka International Cancer Institute
◯	◯	◯	National Hospital Organization Osaka National Hospital
	◯	◯	Osaka General Medical Center
	◯	◯	Osaka Medical and Pharmaceutical University
	◯		Osaka Rosai Hospital
◯	◯	◯	Kobe University Graduate School of Medicine
	◯	◯	Kansai Rosai Hospital
◯	◯		Hyogo College of Medicine
◯	◯	◯	Hyogo Cancer Center
	◯		Itami City Hospital
	◯		Shimane University Faculty of Medicine
	◯	◯	Hiroshima University Hospital
	◯		Fukuyama City Hospital
	◯		Tokushima Red Cross Hospital
◯	◯	◯	National Hospital Organization Shikoku Cancer Center
◯		◯	Kochi Health Sciences Center
◯		◯	National Kyushu Cancer Center
◯		◯	Kyushu University Hospital

	◯	◯	Oita University Faculty of Medicine

HBP: Hepatobiliary and Pancreatic Oncology Group
SCSG: Stomach Cancer Study Group:
JEOG: Japan Esophageal Oncology Group

16.10. JCOG Protocol Review Committee

This protocol was approved by JCOG Protocol Review Committee prior to submit to Certified Review Board.
(For membership, see website http://www.jcog.jp/basic/org/committee/protocol.html)
Contact: Protocol Review Committee Office
JCOG Operations Office/Clinical Research Support Office, National Cancer Center Hospital
〒104-0045 5-1-1, Tsukiji, Chuo-ku, Tokyo
TEL: 03-3542-2511 (ext. 2302)
FAX:03-3542-7006
E-mail: jcogoffice@ml.jcog.jp

16.11. JCOG Data and Safety Monitoring Committee

During study period, the study will be monitored by Data and Safety Monitoring Committee (e.g., adverse event reports, interim analysis reviews, monitoring report reviews, protocol revision reviews). However, the committee members from the study group conducting this study do not participate directly in the review of this study.
(For membership, see website http://www.jcog.jp/basic/org/committee/jury.html)
Contact: JCOG Data and Safety Monitoring Committee Office
JCOG Operations Office/Clinical Research Support Office, National Cancer Center Hospital
〒104-0045 5-1-1, Tsukiji, Chuo-ku, Tokyo
TEL:03-3542-2511 (ext. 2403)
FAX:03-3542-7006
E-mail: jcogoffice@ml.jcog.jp

16.12. JCOG Audit Committee

Site-visit audits by Audit Committee will be conducted during study period.
(For membership, see website http://www.jcog.jp/basic/org/committee/audit.html)
Contact: JCOG Auditing Committee Office
JCOG Operations Office/Clinical Research Support Office, National Cancer Center Hospital
〒104-0045 5-1-1, Tsukiji, Chuo-ku, Tokyo
TEL:03-3542-2511 (ext. 2403)
FAX:03-3542-7006
E-mail: jcogoffice@ml.jcog.jp

16.13. JCOG Conflict of Interest Committee

During study period, JCOG investigators involved in this study will be managed by the Conflict of Interest Committee.
(For membership, see website http://www.jcog.jp/basic/org/committee/coi.html)
Contact: JCOG Conflict of Interest Committee Office
JCOG Operations Office/Clinical Research Support Office, National Cancer Center Hospital
〒104-0045 5-1-1, Tsukiji, Chuo-ku, Tokyo
TEL:03-3542-2511 (ext. 2404)
FAX:03-3547-1002
E-mail: jcogoffice@ml.jcog.jp

16.14. Data Center/Operations Office

JCOG Data Center
Director of Data Center: Haruhiko Fukuda
Clinical Research Support Office, National Cancer Center Hospital

〒104-0045 5-1-1, Tsukiji, Chuo-ku, Tokyo
TEL:03-3542-3373
FAX:03-3542-3374
E-mail: jcogdata@ml.jcog.jp

JCOG Operations Office
Director of Operations Office: Kenichi Nakamura
Clinical Research Support Office, National Cancer Center Hospital
〒104-0045 5-1-1, Tsukiji, Chuo-ku, Tokyo
TEL:03-3547-1002
FAX:03-3547-1002
E-mail: jcogoffice@ml.jcog.jp

Official website: http://www.jcog.jp/

16.14.1. Data management administrator

Data management organization		JCOG Data Center
Data management administrator	Name	Harumi Kaba
	e-Rad number	40543442
	Affiliation	Clinical Research Support Office, National Cancer Center Hospital
	Title	Head of Multi-institutional Data Management Section

16.14.2. Monitoring administrator

Monitoring organization		JCOG Data Center
Monitoring administrator	Name	Haruhiko Fukuda
	e-Rad number	70263390
	Affiliation	Clinical Research Support Office, National Cancer Center Hospital
	Title	Chief of Data Management Division

16.14.3. Site-visit audit administrator

Auditing organization		JCOG Operations Office
Site-visit audit administrator	Name	Kenichi Nakamura
	e-Rad number	40543533
	Affiliation	Clinical Research Support Office, National Cancer Center Hospital
	Title	Director of Research Management Division

16.14.4. Statistical analysis administrator

Statistical analysis organization		JCOG Data Center
Statistical analysis Administrator	Name	Junki Mizusawa
	e-Rad number	60706646
	Affiliation	Biostatistics Division, Center for Research Administration and Support, National Cancer Center/<br>Biostatistics Section, Research Management Division, Clinical Research Support Office, National Cancer Center Hospital
	Title	Biostatistics Section Head

16.14.5. Research and development plan support personnel

Organizations in charge of supporting research and development plans		JCOG Operations Office
Research and development plan support personnel	Name	Tomoko Kataoka
	e-Rad number	70569863
	Affiliation	Clinical Research Support Office, National Cancer Center Hospital
	Title	Medical officer

16.14.6. Coordination management practitioner

Organizations in charge of coordination and management		JCOG Operations Office	
Coordinating and managing practitioners	Name	Junko Eba	
	e-Rad number	80754085	
	Affiliation	Clinical Research Support Office, National Cancer Center Hospital	
	Title	Medical officer	

16.14.7. Personnel who oversees the study other than Principal Investigator and site investigators

Personnel who oversees the study other than Principal Investigator and site investigators	Name	Not applicable	
	e-Rad number		
	Affiliation		
	Title		
	Relevance of Secondary Sponsor	□ Applicable	□ Not applicable

16.14.8. Study group personnel

JCOG Data Center

Statistics Section　Gakuto OGAWA

Data Management Section　Kyoko HASEGAWA

JCOG Operations Office

Science Section　Tomoko KATAOKA/Tadayoshi HASHIMOTO

In addition, JCOG Data Center/Operations Office commissioned some of theire research support activities (such as support for the preparation of study protocols, data management, and site-visit audits) to other corporations. The commissioned duties are supervised by the National Cancer Center through routine work, as well as by receiving work reports from the institution and providing supervisory guidance. The current consignee is as follows

Clinical Oncology Research and Education, a specified non-profit organization

〒104-0061 DJ Ginza Building 7F, 8-18-3, Ginza, Chuo-ku, Tokyo

Official website　http://www.core.or.jp/

16.15. Developing a study protocol

Chigusa MORIZANE, National Cancer Center Hospital

Nozomu MACHIDA, Kanagawa Cancer Center

Yoshitaka HONMA/Ken KATO, National Cancer Center Hospital

Support for protocol development

JCOG Data Center

Statistics Section (in charge of study design)　Junki MIZUSAWA

Data Management Section (CRF preparation)　Harumi KABA

JCOG Operations Office

Protocol development　Hiroshi KATAYAMA/Kozo KATAOKA/Aya MIURA

Person in charge of IC documents　Aya KIMURA/Noriko TSUJI

17. Publication of the study results and completion of the study

17.1. Paper and conference presentations

Primary publication will be published in English journals.

Paper publication including review article and conference presentation of Introduction of the study, by Principal Investigator or Study Coordinator, which does not include the analytical results of the endpoint of the study, are allowed when Group Chair and JCOG Data Center Director agree to them. Publication of the distribution of baseline factors or the safety data after the end of accrual are also allowed. No publication other than primary analysis and final analysis will be performed unless previously approved by Data and Safety Monitoring Committee.

In principle, the authors of the main published papers on the results of the study (the first publication of the results of primary endpoint) shall be the first Study Coordinator (HBPOG), followed by Study Coordinator (JEOG or SCSG), Study Coordinator (JEOG or SCSG), Principal Investigator (HBPOG), Principal Investigator (JEOG or SCSG), Principal Investigator (JEOG or SCSG), the statistical staff of Data Center (one statistician in charge at the time of the analysis for publication), Centralized Pathological diagnosis Coordinator (HBP), Centralized Pathological diagnosis Coordinator (GI). In accordance with the limitations imposed by the article's posting provisions, researchers who contributed in descending order of their number of patients registrations were selected for each institution as co-authors, and the last author was Group Chair (or Study Chair). The inclusion of staffs of JCOG Operations Office as co-authors will be determined by Group Chair depending on their contributions. Authors of articles other than the primary publication (e.g., Secondary endpoints articles, secondary analysis articles) will be determined by Study Chair with Group Chair approval.

All co-authors will review the article contents prior to submission for publication and only those who agree to the article contents. If there is no consensus on the contents, principle investigator may not include the investigator in the co-authorship with the approval of Group Chair. If there is no consensus between groups and JCOG Data Center/Operations Office, ultimately follow JCOG Chair instructions.

17.2. Primary Endpoint Report and Clinical Summary Report

The procedures are specified from the preparation of the primary endpoint report and clinical summary report. If primary analysis is the final analysis, the primary endpoint report will not be prepared and the clinical summary report will be prepared.

17.2.1. Clinical Summary Report

Based on the final analysis report, Principal Investigator will prepare a "Abstract of the Clinical Summary Report" containing subjects background information (age, sex, etc.), study design and study progress, results of analyses for each endpoint, conclusions of the entire study, interpretations and discussion of the results, etc. within 6 months from the issue date of the final analysis report, submit it to the Data Center, and undergo review. In addition, the issue date of the final analysis report shall be "the date when the period for collecting data on all endpoints is completed" as specified in the Enforcement Regulations.

After obtaining approval from Group Chair and the Director of the Data Center, abstract of the clinical summary report will be submitted to Certified Review Board review as a "clinical summary report" with the final analysis report within 1 year of the issue date of the final analysis report.

Principal Investigator/Study Coordinator will disclose abstract of the clinical summary report (Notification of Completion of Form 1 of Article 24 of Regulation) to jRCT within 1 month after obtaining Certified Review Board approval (if the report is unpublished, abstract will not be released in jRCT and will be published immediately after publication).

The approved abstract of the clinical summary report will be submitted to the administrators of each participating institution through the site investigators at each institution and to JCOG Chair. Abstract of the clinical summary report is available on JCOG website (http://www.JCOG.jp/).

The timing of distribution of the final analysis report to participating institutions will be determined by Principal Investigator/Study Coordinator considering the timing of publication of the final analysis results, and the final analysis report will be distributed to the researchers at participating institutions by themselves or through the Data Center.

17.3. Completion of the study

On the date that abstract of the clinical summary report was released to iRCT, the study is completed.

In institutions where patients were not enrolled, the institution may be withdrawn from the institution list by submitting a request for change (Unified Form 3 for Clinical Research) and a Notification of Changes in Protocol (Form 2) stating that Principal Investigator withdraws the institution from the institution list in question to Certified Review Board, and after obtaining approval, notifying the MHLW (Local Health Bureau responsible for the location of Certified Review Board). When a protocol change notification is received by the Local Health Service, the investigator at the institution shall report the withdrawal to the Administrator of the institution.

18. References

omit

19. Appendix

- Informed consent form
- Body surface area table
- Toxicity Criteria (CTCAE v5.0-JCOG)
- CRF samples* (CRF draft attached for the first review submission)
- JCOG-Biobank Japan Biorepository Protocol
- JCOG-Biobank Japan Biorepository Informed Consent Form

Japan Clinical Oncology Group
Hepatobiliary and Pancreatic Oncology Group/Stomach Cancer Study Group/Japan Esophageal Oncology Group

Practical Research for Innovative Cancer Control from Japan Agency for Medical Research and Development
“Establishment of standard treatments for neuroendocrine carcinoma of the digestive system”
National Cancer Center Research and Development Fund 2020-J-3
“Scientific research on multi-institutional trials to establish new standard treatment of solid tumors in adults”

JCOG1213

Randomized phase III study of etoposide plus cisplatin combination therapy versus irinotecan plus cisplatin combination therapy in advanced neuroendocrine carcinoma of the digestive system. ver.1.7.0

TOPIC-NEC

HBPOG
Group Chair: Junji FURUSE
Department of Medical Oncology, Kyorin University Faculty of Medicine

Study Chair: Takuji OKUSAKA
Department of Hepatobiliary and Pancreatic Oncology, National Cancer Center Hospital 5-1-1 Tsukiji, Chuo-ku, Tokyo 104-0045 Japan
Tel: +81-3-3542-2511
E -mail : tokusaka@ncc.go.jp

Study Coordinator: Chigusa MORIZANE
Department of Hepatobiliary and Pancreatic Oncology, National Cancer Center Hospital 5-1-1 Tsukiji, Chuo-ku, Tokyo 104-0045 Japan
Tel: +81-3-3542-2511
E -mail : cmorizan@ncc.go.jp

SCSG
Group Chair: Masanori TERASHIMA
Division of Gastric Surgery, Shizuoka Cancer Center Hospital

Study Chair: Narikazu BOKU
Department of Gastrointestinal Oncology, National Cancer Center Hospital
5-1-1 Tsukiji, Chuo-ku, Tokyo 104-0045 Japan
Tel: +81-3-3542-2511
E -mail : nboku@ncc.go.jp

Study Coordinator: Nozomu MACHIDA
Department of Gastroenterology, Kanagawa Cancer Center (Gastrointestinal Tract)
TEL:+45-520-2222
FAX:+45-520-2202
E-mail:n-machida@kcch.jp

JEOG
Group Chair: Yuko KITAGAWA
General and Gastroenterological Surgery, Keio University Hospital

Study Chair: Ken KATO
Department of Head and Neck, Esphageal Medical Oncology, National Cancer Center Hospital 5-1-1 Tsukiji, Chuo-ku, Tokyo 104-0045 Japan
Tel: +81-3-3542-2511
E -mail : kenkato@ncc.go.jp

Study Coordinator: Yoshitaka HONMA/Ken KATO
Department of Head and Neck, Esphageal Medical Oncology, National Cancer Center Hospital 5-1-1 Tsukiji, Chuo-ku, Tokyo 104-0045 Japan
Tel: +81-3-3542-2511
E-mail:yohonma@ncc.go.jp
/kenkato@ncc.go.jp

2013/3/16	Protocol concept approved by JCOG Executive Committee (PC1213/1214/1215)
2014/6/27	Protocol approved byJCOG Protocol Review Committee
2017/5/23	Revision ver. 1.1 approved by JCOG Data and Safety Monitring Committee
2018/8/16	Revision ver. 1.2 approved by JCOG Data and Safety Monitring Committee
2018/10/11	Approved by Certified Review Board of National Cancer Center Hospital
2019/5/27	Revision ver. 1.3.0. approved by JCOG Data and Safety Monitring Committee
2019/6/27	Approved by Certified Review Board of National Cancer Center Hospital
2020/1/24	Revision ver. 1.4.0. approved by JCOG Data and Safety Monitring Committee
2020/2/27	Approved by Certified Review Board of National Cancer Center Hospital
2020/7/17	Revision ver. 1.5.0. approved by JCOG Data and Safety Monitring Committee
2020/9/24	Approved by Certified Review Board of National Cancer Center Hospital
2021/1/21	Revision ver. 1.6.0. approved by JCOG Data and Safety Monitring Committee
2021/2/25	Approved by Certified Review Board of National Cancer Center Hospital
2021/6/16	Revision ver. 1.7.0. approved by JCOG Data and Safety Monitring Committee
2020/7/29	Approved by Certified Review Board of National Cancer Center Hospital

0. Summary

This study is conducted as a "specified clinical trial" based on the Clinical Trials Act (Act No. 16 of April 14, 2017). In this protocol, the Principal Investigator is the Study Chair in the Hepatobiliary and Pancreatic Oncology Group of JCOG.

Name of study: "Randomized phase III study of etoposide plus cisplatin combination therapy versus irinotecan plus cisplatin combination therapy in advanced neuroendocrine carcinoma of the digestive system. (TOPIC-NEC) ".

Public study title: "Randomized phase III study of etoposide plus cisplatin combination therapy versus irinotecan plus cisplatin combination therapy in advanced neuroendocrine carcinoma of the digestive system. (TOPIC-NEC) "

0.1. Schema

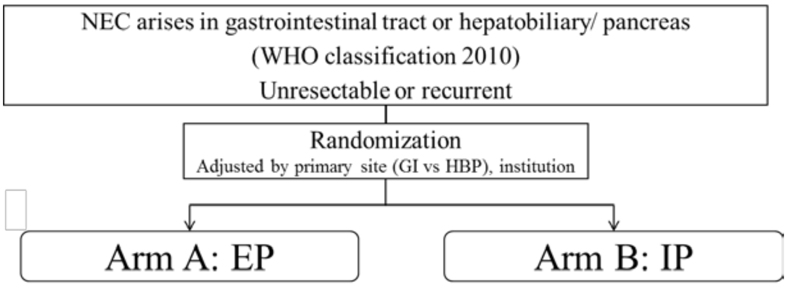

0.2. Objectives

A randomized phase III study was conducted to determine the better treatment option between etoposide/cisplatin combined therapy (EP therapy) or irinotecan/cisplatin combined therapy (IP therapy), both of which are standard treatments for non-resectable/recurrent neuroendocrine carcinoma (NEC as classified by WHO in 2010) with primary lesions in the gastrointestinal tract/hepatobiliary pancreatic organs.

Primary endpoint: Overall survival

Secondary endpoints: Response rate in case of measurable lesions, Progression-free survival (PFS), incidence rate of adverse events, dose intensity of Cisplatin, and incidence rate of serious adverse events.

0.3. Subjects

1) Any of the following is applicable based on pathological diagnosis taking findings of immunohistochemistry into consideration (see 3.1. to 3.3.).

[1] Pathologically diagnosed as neuroendocrine carcinoma (NEC*1) in the resected sample.

[2] Containing pathologically confirmed component of neuroendocrine carcinoma (NEC*1) in the biopsy sample.

1: Based on WHO 2010 classification

2) Any of the following is applicable

[1] NEC arise in esophagus, stomach, duodenum, intestine, appendix, colon, rectum, gallbladder, intrahepatic bile duct, extrahepatic bile duct, ampulla of Vater, pancreas,

[2] Liver NEC (primary liver or liver metastasis of unknown primary) *2.

*2: The tumor is only in one of the following sites after a thorough examination of the primary site by contrast CT (from the neck to pelvic) and upper/lower gastrointestinal endoscopy, FDG-PET scan, otolaryngology (head and neck) examination, urology examination (male patients only), and gynecology examination (female patients only).

a. Liver only

b. Bone, lymph nodes below the diaphragm, peritoneum, subcutaneous, muscle, spleen, and liver

3) Unresectable (see 3.6) or recurrent cancer (see 3.7). It is not essential for a pathological confirmation of the metastatic lesion or recurrent site. Cases of esophageal NEC is ineligible if corresponding to any of the following.

[1] cT4.

[2] No distant metastasis rather than supraclavicular lymph node

[3] Stenosis indicated for palliative radiotherapy

4) No previous chemotherapy or radiotherapy for NEC. Pre- or post-operative chemotherapy except irinotecan or

etoposide for NEC is allowed as long as it was completed at least 8 weeks prior to registration.

5) No previous chemotherapy using platinum agents for any malignancies.

6) No serious tumor-related complications.

Serious tumor-related complications include superior vena cava syndrome, inferior vena cava syndrome, pleural, ascites, or pericardial effusions that are large or uncontrollable (e.g., effusions that accumulate quickly after drainage or even after adhesive surgery), and brain metastases with neurological symptoms.

7) Aged 20 to 75 years old.

8) ECOG performance status of 0 or 1.

9) Sufficient oral intake

10) Measurable region is not required.

11) Adequate organ functions.

[1] WBC ≥3,000/ mm^3

[2] Neutrophils ≥ 1,500/ mm^3

[3] Hemoglobin ≥ 9.0 g/dL

[4] Platelets ≥ 10×10^4/ mm^3

[5] Total bilirubin≤ 1.5 mg/dL※4

[6] AST(sGOT)≦100 IU/L※4（for hepatic NEC and liver metastasis, ≦150 IU/L）

[7] ALT(sGPT)≦100 IU/L※4（for hepatic NEC and liver metastasis, ≦150 IU/L）

※4：Presence or absence of biliary drainage is not relevant

[8] Serum creatinine≦ 1.3 mg/dL

[9] Creatinine clearance※5≥ 60 mL/min

※5： Creatinine clearance must have been estimated using the Cockcroft-Gault formula, and must be 60 mL/min or more.

If the estimation is less than 60 mL/min, but the actual measurement is 60 mL/min or more, the patient can be deemed eligible.

Cockcroft-Gault formula

Male：Ccr ={(140－age) × body weight (kg)}/{72×serum creatinine (mg/dL)}

Female：Ccr = 0.85×{(140－age) × body weight (kg)}/{72×serum creatinine(mg/dL)}

12) Written informed consent.

0.4. Treatments

Arm A: etoposide plus cisplatin (EP) arm

The following chemotherapy is continued until the patient meets discontinuation criteria, with 3-weeks of treatments counting as one cycle.

Drug	Dosage (mg/m^2)	Dosing regimen/Dosing time	Dose day
Etoposide	100	IV/60-120 min	Day 1, 2, 3
Cisplatin	80	IV/60-120 min	Day 1

Arm B: Irinotecan plus Cisplatin (IP) arm

The following chemotherapy would be continued until the patient meets discontinuation criteria, with 4-weeks of treatments counting as one cycle.

Drug	Dose (mg/m^2)	Dosing regimen/Dosing time	Dose day
Irinotecan	60	IV/90 min	Day 1, 8, 15
Cisplatin	60	IV/60-120 min	Day 1

0.5. Planned sample size and study period

The planned sample size is 170.

Accrual period: 6 years.

Follow-up period: 2 years after accrual completion.

Analysis period: 1 year.

Total study duration 9 years.

Scheduled start date of the study Aug 8, 2014

Expected completion date of the study Aug 8, 2023

0.6. Contact information

Eligibility criteria, treatment modification, and other issues requiring clinical decisions: Study Coordinator (front cover and 16.6.)

Enrollment procedure protocol, case report form (CRF) entries, etc.: JCOG Data Center, (16.14.)

Adverse event reporting: JCOG Data and Safety Monitoring Committee (16.11.),

Contents

0. SUMMARY 2

0.1. SCHEMA 2
0.2. OBJECTIVES 2
0.3. SUBJECTS 2
0.4. TREATMENTS 3
0.5. PLANNED SAMPLE SIZE AND STUDY PERIOD 3
0.6. CONTACT INFORMATION 4

1. OBJECTIVES 8

2. BACKGROUND 8

2.1. TARGET 8
2.2. STANDARD TREATMENT FOR TARGET DISEASE 16
2.3. RATIONALE FOR ESTABLISHMENT OF TREATMENT PLAN 18
2.4. STUDY DESIGN 21
2.5. SUMMARY OF EXPECTED ADVANTAGES AND DISADVANTAGES ASSOCIATED WITH STUDY PARTICIPATION 22
2.6. SIGNIFICANCE OF THIS STUDY 23
2.7. ASSOCIATED RESEARCH (INCLUDING SAMPLE ANALYSIS RESEARCH) 23
2.8. JCOG-BIOBANK JAPAN (BBJ) COLLABORATING BIOBANK 23

3. CRITERIA/DEFINITIONS USED IN THIS STUDY 25

3.1. TISSUE CLASSIFICATION (WHO 2010 CLASSIFICATION) 25
3.2. GRADE CLASSIFICATION（ENETS [EUROPEAN NEUROENDOCRINE TUMOR SOCIETY] / WHO2010 CLASSIFICATION）..... 25
3.3. HISTOPATHOLOGICAL DIAGNOSIS 25
3.4. DISEASE STAGE CLASSIFICATION CRITERIA (UICC-TNM 7TH EDITION) 25
3.5. RESIDUAL TUMOR (R) CLASSIFICATION (UICC-TNM 7TH EDITION) 33
3.6. DEFINITION OF NON-RESECTABLE NEC 33
3.7. DEFINITION OF RECURRENT NEC 34
3.8. DEFINITION OF HEPATIC NEC (HEPATIC PRIMARY LESION OR LIVER METASTASIS FROM AN UNKNOWN PRIMARY LESION) 34

4. PATIENT SELECTION CRITERIA 34

4.1. INCLUSION CRITERIA (FOR ENROLLMENT) 34
4.2. EXCLUSION CRITERIA 35

5. REGISTRATION AND RANDOMIZATION 36

5.1. PROCEDURE OF REGISTRATION 36
5.2. RANDOMIZATION AND ALLOCATION ADJUSTMENT FACTOR 37

6. TREATMENT PLAN AND TREATMENT MODIFICATION CRITERIA 38

6.1. PROTOCOL TREATMENT 38
6.2. PROTOCOL TREATMENT TERMINATION/COMPLETION CRITERIA 41
6.3. TREATMENT MODIFICATION CRITERIA 42
6.4. CONCOMITANT TREATMENT AND SUPPORTIVE CARE 48
6.5. POST-STUDY TREATMENT 55

7. ANTICIPATED ADVERSE EVENTS 56

7.1. ANTICIPATED ADVERSE REACTIONS 56

7.2. ANTICIPATED ADVERSE EVENTS DUE TO PATHOGENESIS 59
7.3. EVALUATION OF ADVERSE EVENTS/REACTIONS 60

8. EXAMINATION AND EVALUATION 62

8.1. BASELINE EXAMINATION AND EVALUATION BEFORE REGISTRATION 62
8.2. EXAMINATION AND EVALUATION DURING TREATMENT 63
8.3. EXAMINATION AND EVALUATION AFTER COMPLETION OF TREATMENT 64
8.4. INFORMATION ON POST- STUDY TREATMENT 64
8.5. STUDY CALENDAR 65

9. DATA COLLECTION 69

9.1. CASE REPORT FORM (CRF) 69

10. REPORTING OF "DISEASE OR THE LIKE"(ADVERSE EVENTS) 70

10.1. SERIOUS ADVERSE EVENTS AND SUBJECTS OF EXPEDITED REPORTING. 70
10.2. INVESTIGATOR'S REPORTING REQUIREMENTS AND PROCEDURES 71
10.3. RESPONSIBILITIES OF PRINCIPAL INVESTIGATOR/STUDY COORDINATOR 73
10.4. RESPONSIBILITIES OF THE SITE INVESTIGATORS AT THE PARTICIPATING INSTITUTIONS (INCLUDING THE RELEVANT INSTITUTION) 75
10.5. RESPONSIBILITIES OF THE DATA AND SAFETY MONITORING COMMITTEE 75

11. RESPONSE EVALUATION AND ENDPOINT DEFINITION 76

11.1. RESPONSE ASSESSMENT (ONLY FOR PATIENTS WITH MEASURABLE DISEASE) 76
11.2. DEFINITIONS OF ANALYSES SET 82
11.3. DEFINITION OF ENDPOINTS 82

12. STATISTICAL CONSIDERATION 85

12.1. PRINCIPAL ANALYSIS AND DECISION CRITERIA 85
12.2. PLANNED ACCRUAL, ACCRUAL PERIOD, AND FOLLOW-UP PERIODS 86
12.3. INTERIM ANALYSIS AND EARLY TERMINATION OF THE STUDY 87
12.4. ANALYSIS OF SECONDARY ENDPOINTS 88
12.5. FINAL ANALYSIS 88
12.6. EXPLORATORY ANALYSIS 89
12.7. PREMATURE WITHDRAWAL FROM THE TRIAL 89
12.8. PROCEDURES AFTER EARLY TERMINATION OF THE STUDY 90

13. ETHICAL CONSIDERATIONS 92

13.1. PROTECTION OF HUMAN SUBJECTS 92
13.2. INFORMED CONSENT 92
13.3. PROTECTION OF PERSONAL INFORMATION AND PATIENT IDENTIFICATION 94
13.4. ADHERENCE TO THE PROTOCOL 96
13.5. APPLICATION TO CERTIFIED REVIEW BOARD AND NOTIFICATION OF IMPLEMENTATION PLANS 96
13.6. PROTOCOL REVISION/AMENDMENT 100
13.7. CONFLICTS OF INTEREST (COIS) INVOLVED IN THIS STUDY 102
13.8. COMPENSATION 104
13.9. INTELLECTUAL PROPERTY 105
13.10. DISCLOSURE OF INFORMATION ON THIS STUDY 105

14. MONITORING AND AUDIT 106

14.1. PERIODIC MONITORING 106
14.2. SITE VISIT AUDITS 108

14.3. MANAGEMENT OF NON-COMPLIANCE 109

15. SPECIAL INSTRUCTIONS 110

15.2. JCOG BIOBANK JAPAN (BBJ) BIOREPOSITORY 111

16. ORGANIZATION 111

16.1. MAIN STUDY FUND (FUNDING SOURCE) OF THIS STUDY. 111
16.2. JAPAN CLINICAL ONCOLOGY GROUP (JCOG) 111
16.3. JCOG CHAIR 112
16.4. STUDY GROUP AND GROUP CHAIR 112
16.5. STUDY CHAIR (PRINCIPAL INVESTIGATOR) 113
16.6. STUDY COORDINATOR 113
16.7. CENTRAL PATHOLOGICAL REVIEW COORDINATOR 114
16.8. CENTRAL PATHOLOGICAL REVIEW COORDINATOR 114
16.9. PARTICIPATING SITES (PARTICIPATING INSTITUTIONS) 115
16.10. JCOG PROTOCOL REVIEW COMMITTEE 116
16.11. JCOG DATA AND SAFETY MONITORING COMMITTEE 116
16.12. JCOG AUDIT COMMITTEE 116
16.13. JCOG CONFLICT OF INTEREST COMMITTEE 116
16.14. DATA CENTER/OPERATIONS OFFICE 116
16.15. DEVELOPING A STUDY PROTOCOL 118

17. PUBLICATION OF THE STUDY RESULTS AND COMPLETION OF THE STUDY 119

17.1. PAPER AND CONFERENCE PRESENTATIONS 119
17.2. PRIMARY ENDPOINT REPORT AND CLINICAL SUMMARY REPORT 119
17.3. COMPLETION OF THE STUDY 120

18. REFERENCES 121

19. APPENDIX 121

1. Objectives

A randomized phase III study was conducted to determine the better treatment option between etoposide/cisplatin combined therapy (EP therapy) or irinotecan/cisplatin combined therapy (IP therapy), both of which are standard treatments for non-resectable/recurrent neuroendocrine carcinoma (NEC as classified by WHO in 2010) with primary lesions in the gastrointestinal tract/hepatobiliary pancreatic organs.

Primary endpoint: Overall survival

Secondary endpoints: Response rate in case of measurable lesions
Progression-free survival (PFS), incidence rate of adverse events, dose intensity of Cisplatin, and incidence rate of serious adverse events

2. Background

2.1. Target

2.1.1. Epidemiology

Neuroendocrine tumors (NETs) and neuroendocrine carcinomas (NECs) can arise in various organs in the body. Of these, NECs with primary lesions occurring in the lungs, namely small cell lung cancer and large cell lung cancer, have been collectively referred to as high-grade NECs (HGNECs). However, terms such as extrapulmonary small cell carcinoma and extrapulmonary NEC have been used to refer collectively to NECs, with primary lesions outside the lungs.

The number of newly reported cases of neuroendocrine neoplasms (NENs) annually, estimated based on the cases registered in the U.S. SEER (Surveillance, Epidemiology, and End Results) database between 1973 to 2004 is 5 in 100,000 people[1]. In Japan, the Neuroendocrine Tumor Workshop Japan (NET Work Japan) carried out a nationwide survey of pancreatic and gastrointestinal NENs, and estimated the number of new cases in 2005 at approximately 1.01 people with pancreatic primary lesion per 100,000 people, and 2.10 people with gastrointestinal primary lesion per 100,000 people[2, 3].

The report by the PRONET Study Group in France concerning their prospective observational study is a good reference for the proportion of NENs that can be classified as NECs. Out of the 778 patients diagnosed with gastrointestinal primary lesion NENs in 80 facilities from August 2010 to July 2011, 104 patients (13.4%) had NEC[4].

The frequency of incidence of NECs according to the primary lesion organ has been shown in the tables below (Table 2.1.1a and Table 2.1.1b). Despite variations between reports, gastrointestinal primary lesion NECs account for approximately 20–68% of all extrapulmonary NEC cases. The breakdown of gastrointestinal primary lesion NEC cases with primary lesion in the gastrointestinal tract (esophagus, duodenum, small and large intestines) indicated that these cases accounted for 56–84% of the total cases, while hepatobiliary pancreatic cancer cases accounted for 15–35% of all cases.

Table 2.1.1a. Frequency of extrapulmonary NECs according to the organ of primary lesion

Reported year/Reporter	N	Gastrointestinal	Gynecologal organs	Urology	Head and Neck	Adrenal	Primary unknown	Other
2012/Terashima [5]	136	68%	12%	6%	NA	NA	8%	7%
2010/Brennan [6]	74	20%	35%	15%	19%	NA	9%	1%
2009/Wong [7]	1618	33%	NA	20%	11%	10%	4%	22%
2006/Haider [8]	101	20%	11%	18%	12%	9%	31%	NA
2007/Lee [9]	61	56%	20%	10%	8%	NA	6%	NA

Table 2.1.1b. Percentage breakdown of NECs as per the primary lesion organ of gastrointestinal NEC

Reported year/Reporter	N	Esophageal	Gastric	Duodenal/small intestine	Colorectal	Hepatobiliary	Pancreas	Other
2012/Machida [10]	258	33%	27%	2%	12%	12%	14%	NA
2012/Sorbye [11)]	205	6%	10%	NA	40%	NA	35%	10%
2004/Brenner [12]	544	53%	11%	0.2%	20%	11%	4%	NA

2.1.2. Clinical pathology

1) About the term of target disease

The target of this study has a complex disease concept, and since the method of classification has changed with time, various names have been assigned that could cause confusion. In this study, we use classification and nomenclature defined according to the WHO 2010 classification（WHO Classification of Tumors of the Digestive System 2010）[13].

All the tumors originating from neuroendocrine cells or differentiation into endocrine cells are called neuroendocrine neoplasms (NENs). Depending on the malignancy, NENs were classified as Grade 1 NETs (NET G1), Grade 2 NETs (NET G2), or NEC.

Furthermore, prior to the WHO 2010 classification, all disease concepts equivalent to NENs were called NETs, but in this study, we would have referred to all descriptions of the disease concept as NENs. Furthermore, according to the 2000 and 2004 WHO classifications, the term well-differentiated NEC (WDNEC) has been used for disease units equivalent to NET G2, but in this study NEC does not mean the same.

2) Classification and clinical pathology

NEN is a tumor that develops from neuroendocrine cells present in various tissues or shows a tendency of differentiation into neuroendocrine cells. Histologically these tumors express neuroendocrine markers, such as Chromogranin A, NSE (neuron-specific enolase), and synaptophysin. The WHO classifications in 2000 and 2004 combined the presence or absence of metastasis/local infiltration and cellular proliferative capacity (evaluated based on Ki-67 expression intensity and mitotic presentation), and thereby classified these tumors into well-differentiated (neuro)endocrine tumors without metastasis/local infiltration, well-differentiated (neuro)endocrine tumors with metastasis/local infiltration, and poorly differentiated (neuro)endocrine tumors with even greater cell proliferative capacity. On the other hand, European Neuroendocrine Tumor Society (ENETS) has proposed a method of classification that evaluates the malignancy as Grade 1, 2, or 3 (G1, G2, or G3), according to the cell proliferative capacity (Ki-67 index or number of mitotic presentations), which has proven to be most useful for classification of prognosis[14, 15]。

Against this backdrop, the WHO classification that targeted gastrointestinal diseases was published in 2010. The disease as a whole was considered “neuroendocrine neoplasms (NEN)”, and the disease malignancy was largely classified as NET G1 (neuroendocrine tumor Grade 1), NET G2 (Grade 2), NEC, and mixed adenoneuroendocrine carcinoma (MANEC). MANEC refers to the cancers wherein adenocarcinoma components account for more than 30% of the cancer, while those under 30% are classified as NECs. However, even though this method of classification is applicable while evaluating the entire tumor based on resected specimen, it is not possible to evaluate the proportion of each component in the entire tumor during a diagnosis based on biopsy sample.

Each of NET G1, NET G2, and NECs described in the WHO 2010 classification is equivalent to G1, G2, and G3 as per the ENETS classification. NETs are well-differentiated tumors, with relatively low atypicality and malignancy, and the clinical course is slow with a 5-year survival in the range of 62–85%[16, 17]. On the other hand, NECs are poorly differentiated tumors, where tumor cells with poor cellular constituents proliferate diffusely, and are pathologically similar to small cell lung cancers, since these show many mitotic presentations, involve necrotic lesions, and present with neuroendocrinological features during immunohistochemical staining[1]. The clinical presentations are also similar in terms of the rapid proliferation of NECs and relatively high sensitivity to anticancer drugs[18].

Similar to lung cancer, NECs also exist as small cell cancer type or large cell type (LCNEC：large cell NEC）[13]. Future challenges include studies on the frequency and differences in clinical presentation of each disease. Table 2.1.2 shows the WHO and Grade classification.

Table 2.1.2. Changes in WHO classification and grading; Shaded parts are targets of this study

WHO 2000 classification	WHO 2010 classification		ENETS Grade
Well-differentiated endocrine tumor（WDNET）<br>1.1‘Benign’ behavior<br>1.2 Uncertain behavior	Neuroendocrine tumors, NETs<br>• Well-differentiated<br>• Composed of cells similar to normal gastrointestinal	NET G1<br>（carcinoid）	G1<br>Same as 2010 WHO classification<br>Number of mitotic presentations < 2 per 10

	endocrine cells		high power fields（HPF） and/or Ki-67 index ≤2%
Well-differentiated endocrine carcinoma（WDEC）	• Expression of neuroendocrine markers<br>• Hormone production<br>• Mild to moderate nuclear atypia, low proliferative capacity（Grade：G1,G2）	NET G2	G2<br>Number of mitotic presentations 2–20 per 10 HPF and/or Ki-67 index 3-20%
Poorly differentiated endocrine carcinoma/small cell carcinoma（PDEC）	Neuroendocrine carcinoma：NEC（large cell or small cell type）:<br>• Poorly differentiated, highly malignant<br>• Include small cell to large cell type carcinoma<br>• Sometimes present tissue structure similar to NET<br>• Expression of neuroendocrine markers<br>• Significant nuclear atypia, multifocal necrosis, and high proliferative capacity (Grade: G3)		G3<br>Number of mitotic presentations >20 per 10 HPF and/or Ki-67 index > 20%
Mixed exocrine-endocrine carcinoma（MEEC）	Mixed adenoendocrine carcinoma（MANEC）		
Tumor-like lesions（TLL）	Hyperplastic and preneoplastic lesions		

2.1.3. Staging

Disease staging for NENs is represented by the TNM classification proposed by ENETS (hereafter, ENETS TNM[14, 15], AJCC 7th Edition TNM classification and UICC 7th Edition TNM classification published in 2009. The AJCC 7th Edition and UICC 7th Edition TNM classifications are unified, and in this study we would have used the UICC 7th Edition nomenclature).

In the UICC 7th edition, there are independent TNM classifications of NET G1 and NET G2 with primary lesions in the stomach, small intestine, appendix, and colon, but with respect to NEC, it describes "classification according to main tissue type in each organ of primary lesion (squamous cell carcinoma for esophagus, and adenocarcinoma in all other organs of primary lesion)." While there are TNM classifications for hepatocellular carcinoma and intrahepatic cholangiocarcinoma in the liver, there is no description for the classification of the tissue type for NECs with primary lesion in the liver. In this study, however, we would have used the TNM classification of intrahepatic cholangiocarcinoma, which shows similar clinical presentations. Details of each TNM classification would have been mentioned in "3.2 staging criteria".

2.1.4. Standard treatment according to disease staging and outline of prognosis

1) Standard treatment for resectable cases

Surgical resection is performed for resectable cases. The significance of postoperative adjuvant chemotherapy is not clear because a verification study has not yet been performed. Although the NCCN Guidelines[19] describe postoperative adjuvant therapy as the chemotherapy regimen for small cell lung cancers that is typically applied to advanced cases of NEC, it has relatively high toxicity and patient burden (see Table 2.2.2, 2.3.2); hence we cannot claim that it is widely used in routine practice. For NECs with gastrointestinal primary lesion with mixed adenocarcinoma, a chemotherapy regimen is suggested typically after surgery for adenocarcinoma; however, in practice, there is no consensus on postoperative adjuvant chemotherapy.

2) Standard treatment for locally advanced cases

While the NCCN Guidelines propose chemoradiotherapy for small cell lung cancer for locally advanced cases, the ENETS Guidelines provide no clear indication[20, 21] for the same. As there is no sufficient information related to radiation dose, efficacy, and safety for NECs with a multitude of primary lesion organs (scope of irradiation), radiation therapy is not widely used for pathologies other than NEC with esophageal primary lesion that is prone to stenosis, and treatment is often provided for distant metastasis.

For locally advanced cases of NEC with esophageal primary lesion, chemoradiotherapy is actively performed. Furthermore, supraclavicular lymph node metastasis in thoracic esophageal tumor is classified as Stage IV, and chemoradiotherapy is applied if metastatic lymph nodes are included in the field of irradiation.

3) Standard treatment for distal metastasis cases

Systemic chemotherapy is indicated for distal metastasis and recurrent cases regardless of the organ, and a regimen is chosen for the small cell lung cancer. With respect to NECs with esophageal primary lesion, even if there is organ metastasis, palliative chemoradiotherapy is prioritized for patients incapable of oral intake of drugs due to esophageal constriction.

The efficacy of everolimus and sunitinib has been demonstrated for NETs with pancreatic primary lesions. Furthermore, Octreotide and Lantreotide effectively inhibit proliferation of NETs that originate in the midgut and pancreatic/gastrointestinal NETs, respectively. However, these results are from clinical trials conducted in patient populations corresponding to NETs. Since the clinical presentations of NETs and NECs differ significantly, these treatments cannot be indicated for NECs during routine practice.

4) Indication of resection for distal metastasis

There is no consensus on the resection criteria specific to the NECs. Since the progression of NETs is slow, resection is actively considered even where there is distal metastasis to the liver, but as NEC progresses fast, surgical resection cannot be indicated for cases with distal metastasis[19, 21].

5) Prognosis

In a preceding study, Yamaguchi and Machida carried out a multicenter joint observational study of poorly

differentiated NEC (WHO 2010 NEC, including patients with clinical diagnosis of poor differentiation) in a total of 23 facilities, including facilities participating in JCOG Hepatobiliary and Pancreatic Oncology Group, former Gastroenterology Group, and observer facilities.

In the multicenter joint study by Yamaguchi and Machida, 89% of cases (229/258 patients) receiving systemic chemotherapy had distal metastasis, with a median survival time (MST) of 11.5 months for all patients, 11.2 months for distal metastasis cases, and 15.9 months for locally advanced cases. MST according to organ was 13.4 months for esophageal primary lesion (N = 85), 13.3 months for gastric primary lesion (N = 70), 29.7 months for small intestine/duodenal primary lesion (N = 6), 7.6 months for colonic primary lesion (N = 31), 8.5 months for pancreatic primary lesion (N = 35), and 7.9 months for hepatobiliary primary lesions (N = 31) (Table 2.1.4). While there were 9 cases of five-year survival among all the gastrointestinal cases, there were no five-year survival cases amongst those with primary lesions in hepatobiliary and pancreatic organs[10]. In a multicenter joint observational study for NECs conducted in four Northern European countries (hereafter referred to as NORDIC NEC Study), the MST for NECs with gastrointestinal primary lesion (albeit including ~30% of NECs with primary lesion sites unknown) was 11 months[11]. According to the U.S. SEER database, the MST of NEC was 10 months[3]. Based on the above information, despite differences in the primary lesion organ, the prognosis of the target population of this study is thought to have an overall MST of 10–11 months.

Table 2.1.4. Treatment results according to primary lesion organ for non-resectable/recurrent NECs that underwent systemic chemotherapy

	Esophageal	Gastric	Duodenal/small intestine	Colonic	**Gastrointestinal overall**	Pancreatic	Hepatobiliary	**Hepatobiliary and pancreatic organs overall**
N	85	70	6	31	**192**	35	31	**66**
Response rate（%）	58%	43%	50%	29%	**47%**	17%	16%	**17%**
Median PFS（month）	5.8	4.9	7.3	3.7	**5.1**	3.2	4.1	**3.7**
MST（month）	13.4	13.3	29.7	7.6	**13**	7.9	8.5	**7.9**

2.1.5. Tumor-related complications

We, here, report the complications that require attention during patient management, according to the organ of primary lesion. It is a rare disease and frequencies remain unknown.

1) Esophageal primary

Tumor bleeding/pain/esophageal stenosis/fistula formation due to primary lesion in the esophagus; hoarseness/difficulty swallowing/aspiration pneumonia caused by recurrent laryngeal nerve palsy due to lesion in lymph node metastasis; jaundice and liver failure associated with liver metastasis; respiratory failure and hemoptysis associated with lung metastasis, airway constriction, and pain due to lymph node metastasis; hypercalcemia, tracheal stenosis, tracheal obstruction, and suture failure due to disease progression.

2) Gastric primary

Chronic bleeding from primary lesion, accompanied by anemia, stomach pain, nausea, vomiting, gastric perforation, bloating, suture failure, fistula formation, cardiac/pyloric stenosis, ascites retention due to peritoneal metastasis, ileus, hydronephrosis due to ureteral stenosis, obstructive jaundice/liver failure due to bile duct stenosis, and obstructive jaundice/liver failure due to hepatic portal lymph node metastasis.

3) Small intestine/colonic primary

Tumor hemorrhage, ileus, fistula formation in the small intestine/bladder/vagina, intestinal obstruction, intestinal stenosis, intestinal perforation, and pelvic infection

4) Hepatobiliary and pancreatic primary

Cancer pain, rupture, obstructive jaundice associated with the growth of primary tumor, weight loss, fever, pancreatitis, cholangitis, liver abscess, cholecystitis, biliary hemorrhage, duodenal hemorrhage, duodenal stenosis, anastomotic ulcer/stenosis/leakage, symptoms associated with stenosis/obstruction of portal vein (liver

dysfunction/liver failure, esophageal varices and their rupture, gastritis, ascites retention, and hepatic encephalopathy/coma associated with portal hypertension).

5）Paraneoplastic syndrome

In addition to NECs, there are reports of paraneoplastic syndrome that is often associated with small cell lung carcinoma[22-24]. We report examples of paraneoplastic syndrome seen with small cell lung carcinoma below.

Hyponatremia due to inappropriate sodium secretion, psychological symptoms (changes in personality) due to ectopic ACTH syndrome, hypertension, hypokalemia, high blood sugar, Lambert-Eaton myasthenic syndrome, paraneoplastic cerebellar degeneration associated with autoantibody production (ataxia in the limbs, dysarthria, and nystagmus), paraneoplastic encephalomyelitis/sensory neuron disease (dementia, cranial nerve symptoms, dizziness, ataxia, autonomic imbalance, transverse paralysis, and sensory disorders).

Apart from these, there are thromboembolic events, aspiration (due to gastrointestinal stenosis), anemia, tumor pain, acute renal failure, myositis, and pulmonary fibrosis.

6）Complications due to metastatic lesions

Liver failure, pain, hepatobiliary infection, hemorrhage, bile duct stenosis, bile duct obstruction, respiratory failure, tracheal hemorrhage, respiratory tract infection, atelectasis, airway constriction, ascites, bloating, ileus, ureteral stenosis, ureteral obstruction, urinary retention, urinary tract infection, hiccups, pleural effusion, pleural hemorrhage, chest pain, pathological fracture, pain, hypercalcemia, meningeal carcinomatosis, ataxia, ischemia cerebrovascular, intracranial hemorrhage, nausea, vomiting, dizziness, consciousness disorder, cognitive disturbance, dysphasia, seizure, spasticity, edema limbs, intestinal obstruction/stenosis/perforation, pancreatitis, disseminated intravascular coagulation, thrombocytopenia, anorectal infection, anal hemorrhage, hoarseness, and superior vena cava syndrome

7）Others

General pain management including narcotic analgesics is performed for cancer pain.

Gastrointestinal stenting and bypass surgery are performed for esophageal stenosis, cardiac/pyloric stenosis, and duodenal stenosis.

Percutaneous transhepatic cholangial drainage (PTCD), percutaneous or endoscopic stenting, and bile duct jejunostomy is performed for obstructive jaundice. In addition, complications associated with biliary drainage and gastrointestinal stenting are listed below.

- PTCD, including internal and external fistula tube placement:
  Cholangitis, pancreatitis, cholecystitis, liver abscess, sepsis, biliary hemorrhage, PTCD tube obstruction/deviation, peritonitis, and pneumothorax/pleurisy
- Biliary stenting：
  Cholangitis, pancreatitis, cholecystitis, liver abscess, sepsis, biliary hemorrhage, peritonitis, pneumothorax/pleurisy (for percutaneous stenting), duodenal perforation, pneumonia (for endoscopic stenting), stent obstruction/deviation, duodenal ulcer, and duodenal hemorrhage
- Bile duct jejunostomy：
  Cholangitis, pancreatitis, cholecystitis, liver abscess, and sepsis
- Gastrointestinal stenting：
  Hemorrhage, perforation, pain, stent deviation, stent obstruction, foreign-body sensation, intestinal strangulation, ulcer formation, fever, sepsis, infection, diarrhea, constipation, tenesmus or uncontrollable urination/incontinence symptoms (colon), thyroid injury (esophagus), carotid artery injury (esophagus), and mediastinal abscess (esophagus).

2.1.6. Recurrent/progression

NEC is a very rare disease, and most reports of cases of surgery coincide with the case reports. According to the review by Arai et al., out of the 55 patients with NEC with gastric primary lesion who underwent surgical resection, only three patients survived for two years or more[25]. According to the report by Fischer et al., the MST of 13 patients with NEC with pancreatic primary lesion who underwent surgical resection was 11.7 months (seven of the patients showed distal metastasis)[26]. The form of recurrence among surgery cases remains unknown. The multicenter joint

study by Yamaguchi and Machida reported PFS among cases that underwent systemic chemotherapy with respect to the median as 5.8 months for esophageal primary lesions, 4.8 months for gastric primary lesions, 7.3 months for duodenal small intestine primary lesions, 3.7 months for colonic primary lesions, 3.2 months for pancreatic primary lesions, and 4.1 months for hepatic/biliary primary lesions[10]. In the NORDIC NEC study, the median PFS was 3 months for esophageal primary lesions, 5 months for gastric primary lesions, 3 months for colonic primary lesions, 4 months for rectal primary lesions, 5 months for pancreatic primary lesions, and 4 months for cases with unknown primary lesion site [11].

2.1.7. Prognostic/predictive factors

When prognostic factors were examined by multivariate analysis in the multicenter joint study by Yamaguchi and Machida, out of the various factors such as sex, age (younger or older than 60 years), PS (0 or 1 vs. 2 or more), primary lesion organ (gastrointestinal primary lesions vs. hepatobiliary or pancreatic primary lesions), LDH levels (below vs. above the upper limit of facility standard), presence or absence of liver metastasis, presence or absence of history of radical resection and treatment regimen (IP therapy vs. EP therapy); the independent prognostic factors identified were gastrointestinal primary lesion (vs. hepatobiliary or pancreatic primary lesion, hazard ratio (HR): 0.58), and LDH levels being below the upper limit of facility standard for LDH (vs. above the upper limit of facility standard for LDH, HR: 0.65).

Although IP therapy exhibited slightly better impact on overall survival as compared to EP therapy with an HR of 0.8, the *p*-value of 0.389 meant that there was no significant difference between the two therapies[10]. The NORIDIC NEC study reported poor PS, colonic primary lesions, high platelet count, and high LDH levels to be the main factors behind poor prognosis[11]. However, these prognostic/predictive factors were not obtained with a global consensus.

2.1.8. Rationale for selection of the target population

The objective of this study is to develop a primary chemotherapy regimen for non-resectable/recurrent NECs, and out of the non-resectable or postoperatively recurrent NECs with gastrointestinal primary lesions or hepatobiliary or pancreatic primary lesions (shaded parts in Table 2.1.2 of WHO 2010 classification of NEC), we established chemotherapy-naïve patients to be the target population.

In terms of whether or not to consider MANEC a target of this study, a discussion is needed from the viewpoint of standard treatment. MANEC is considered when 30% or more of the carcinoma consists of adenocarcinoma components, and it is treated as either adenocarcinoma or NEC by discretion of the attending physician, with no real consensus on standard treatment. MANEC was however, excluded from this study, as its disease concept is different from NEC in terms of the standard treatment, which has a consensus regarding the treatment by a “regimen according to small cell lung carcinoma”. In fact, a questionnaire was provided to the three groups participating in this JCOG study (response obtained from 52 facilities). The results showed that 79% of facilities considered the WHO 2010 classification of NEC as the appropriate tissue type for this study, while only 13% responded that consideration of NEC+MANEC would be appropriate. Therefore, majority of facilities considered “WHO 2010 classification of NEC as the appropriate target of this study”.

While diagnosis of NECs is generally carried out using tissue samples and biopsy samples, as mentioned in 2.1.3, there is no consensus methodology to strictly distinguish NEC and MANEC using biopsy samples. As a result of group discussions, a consensus was reached between the three groups that cases presenting with NEC components by biopsy sample-based diagnosis can be enrolled. Although the use of this method would mean that a certain percentage of patients with MANEC, who were not intended to participate in this study, would be enrolled however, currently there are no appropriate means to avoid this. Therefore, we decided to create a consensus for the future based on information obtained from this study.

1）**Reason for targeting gastrointestinal/hepatobiliary or pancreatic primary lesion NEC**

Various guidelines recommend treatment regimens suited for small cell lung carcinoma, regardless of the organ of primary lesion, and is the rationale showing the validity of the treatment development for NEC across many organs. Furthermore, taking into consideration the frequency of the disease, it is unrealistic to develop treatments according to each organ, and in practice the Minnie-Pearl Cancer Research Network Study (mentioned later in 2.2.2), which is a relatively large-scale clinical study of NEC, development of treatment was carried out in a cross-organ

manner[27].

On the other hand, if we take hypothesize that we do not sufficiently understand how the clinical presentations of the disease vary according to the organ, the fact that this is the first randomized study in Japan, and that the maintenance of foundation for clinical studies of this disease in Japan is inadequate at present time, the hurdle is too high to plan a study across all organs including the fields of gynecology and urology. Based on the above consideration, we have decided to target all gastrointestinal organs which have a relatively large number of common points between each other in terms of clinical presentation and types of complication.

According to the multicenter observatory study by Yamaguchi and Machida, the prognosis of NEC with hepatobiliary or pancreatic primary lesion was significantly poorer than that of NEC with gastrointestinal primary lesion (MST: 7.9 vs. 13.0 months, respectively), but we determined that it is possible to accurately evaluate the efficacy of the treatment regimen by randomizing the subjects using the primary lesion site (gastrointestinal vs. hepatobiliary or pancreatic) as an allocation adjustment factor. Therefore, we considered patients with both, gastrointestinal/hepatobiliary or pancreatic primary lesions as one target population of this study, and to examine differences between organs exploratively by subgroup analysis.

2) Reason for including hepatic NEC cases (hepatic primary lesion or liver metastasis with primary lesion site unknown)

Even among NECs, cases with hepatic primary lesions are particularly rare. There are no specific reports on the frequency of incidence and differences from the other organs and reports are limited only to case reports and their reviews. Moreover, since the liver is a major organ for metastasis of primary lesions into other organs, even when tumors have been clinically identified in the liver, in many cases the primary lesion tends to be in another organ. Therefore, liver should not be deemed the primary lesion site without sufficient examination for a primary lesion. On the other hand, although very rare, there have been reports of NEC with hepatic primary lesion[28]. Even if sufficient search for primary lesion leads to no indication of the same outside of the liver, currently it is not possible to distinguish whether it is a case of “NEC with hepatic primary lesion” or “lesion in the liver is a metastatic lesion, with the primary lesion unknown”. In this study, such situations wherein “liver alone has identifiable lesions” would be henceforth, referred to as “hepatic NEC (hepatic primary lesion or liver metastasis from an unknown primary lesion site) for convenience. As the NCCN Guidelines[19] and ENETS Guidelines[20, 21] indicate the usefulness of FDG-PET for detailed examination of the primary lesion, we would also examine the primary lesion using FDG-PET. Furthermore, by referring to the diagnostic procedures for cancers with unknown primary lesion site, detailed examinations into the primary lesion would be carried out by otolaryngological (head and neck) examination and urological examination for men only, and gynecological examination for women only).

Such hepatic NEC (hepatic primary lesion or liver metastasis from an unknown primary lesion site) is anticipated even from an anatomical viewpoint, since the tumor-related complications are the same as that for other gastrointestinal primary lesion NECs, and there is no issue with treating them the same as for gastrointestinal primary lesions.

3) Reason for orienting the study for non-resectable or recurrent cases

As mentioned in 2.1.4 “Standard treatment according to disease staging and outline of prognosis”, systemic chemotherapy is indicated for cases with distal metastasis or recurrent cases. Locally advanced cases are particularly prone to pancreatic or bile duct primary lesions situated in the vicinity of vital vessels, and while the NCCN Guidelines propose chemoradiotherapy according to treatment for small cell lung carcinoma[19], the ENETS Guidelines provide no clear indication[21]. In reality, for the treatment of NEC which has a variety of primary lesion organs (scope of irradiation) there is not enough information available for the chemotherapy regimen that should be combined in terms of its optimum dose, the radiation dose, efficacy, and safety. For this reason, chemoradiotherapy cannot be considered the standard treatment with consensus, and systemic chemotherapy for distal metastasis is used widely except for pathologies involving frequent stenotic symptoms when the primary lesion is in the esophagus. For this reason, this study would enroll non-resectable or recurrent cases that include locally advanced cases.

The section “3.6 Definition of non-resectable NEC” shows the definition of non-resectable cases summarized by referring to the JCOG protocol for clinical studies of systemic chemotherapy (esophagus: JCOG0807, stomach: JCOG1013 or JCOG1002, bile duct: JCOG0805, pancreas: JCOG1106). Furthermore, while radical resection has

been considered for cases of colonic primary lesion adenocarcinoma with liver or lung metastasis, as cases of NEC with distal metastasis are not indicated for resection, we defined non-resectable cases as those in "Stage IV", and followed this definition for duodenal primary lesions, small intestine primary lesions, and appendix primary lesions. Furthermore, with respect to NEC with esophageal primary lesion, chemoradiotherapy is well-indicated for cases with supraclavicular lymph node metastasis of thoracic esophageal tumor even in Stage IV, and hence were not included in this study.

4) Treatment of draft of new classification of NEC

In recent years, proposals have been made to further differentiate the WHO 2010 classification of NECs (G3 in ENETS) to "Grade 3 proliferative tumors showing the same morphological presentations as NET" and "Grade 3 tumors with strong morphological atypicality (previously classified as poorly differentiated NECs)"[29, 30], or by Ki-67 index of 20–50% and 50% or more[11, 19]. This is yet to be confirmed; however, in this study we would have used the WHO 2010 classification. However, we would have used samples collected for central pathological diagnosis to carry out studies related to classification which exploratively adds grade classification and morphological differentiation levels.

2.2. Standard treatment for target disease

Currently, there is no standard drug treatment for non-resectable/recurrent NECs with efficacy verified by a randomized controlled trial. However, given the similarity between pathological and clinical presentations, treatments according to small cell lung carcinoma have been attempted, and there have been reports of positive treatment outcome in small-scale clinical studies and observational studies.

2.2.1. Standard treatment for small cell lung carcinoma

Cisplatin-based multidrug combined therapy is the standard first-line treatment of extensive disease (ED) small cell lung carcinoma. While cyclophosphamide/doxorubicin/vincristine (CAV therapy) was established as a standard treatment in the 1970s in Europe and U.S., the etoposide/cisplatin combined therapy (EP therapy) was introduced in the late 1980s. A comparative study of EP therapy and CAV therapy did not show superiority of EP therapy over CAV therapy in terms of survival, but the response rate (CAV: 51% vs. EP: 61%) and MST (CAV: 8.3 months vs. EP: 8.6 months) were almost the same, and the lighter toxicity level (mucositis, interstitial pneumonia, hemotoxicity) meant that EP could be used as a standard treatment[31]. Thereafter, the JCOG Lung Cancer Group carried out the "comparison between EP therapy and Irinotecan/Cisplatin combined therapy (IP therapy) for Extensive-stage Small Cell Lung Cancer (JCOG9511)", and reported that the IP therapy provided a significantly better overall survival, with MST being 9.4 months vs. 12.8 months (p = 0.002) for IP therapy[32]. However, the two additional large-scale studies conducted primarily in U.S. did not show superiority of IP therapy over EP therapy, and for this reason IP therapy has not been not used as the standard treatment overseas[33, 34]. Furthermore, while the results of the "Randomized controlled trial to verify the non-inferiority of amrubicin/cisplatin combined therapy (AP therapy) over IP therapy (JCOG0509)" were reported in the 2012 conference of the American Society of Clinical Oncology (ASCO), but AP therapy was still considered significantly inferior to IP therapy[35]. Presently, the JCOG Lung Cancer Group considers IP therapy to be the standard treatment for extensive-disease small cell lung carcinoma.

2.2.2. Standard treatment for extrapulmonary NEC

While there are scattered reports relating to use of CAV therapy, EP therapy, IP therapy, and other multidrug combined therapies for extrapulmonary NEC, these reports are primarily from observational studies. Since these reports precede the unification of concept and classification methods of this disease, the nomenclature such as NEC (anaplastic type), NET (poorly differentiated type) and extrapulmonary small cell lung carcinoma have still been used. These reports, however, are presumed to be targeting almost the same disease group as NEC. There are no reports of prospective studies using EP therapy for extrapulmonary NEC. Observational studies have reported response rates between 42–67%, and MST between 15–19 months (Table 2.2.2a). On the other hand, IP therapy has reported results from observational studies and clinical studies, with response rates between 7–83% and MST of 10.1–22.6 months (Table 2.2.2b). As mentioned above, reports related to extrapulmonary NEC are limited to use of EP therapy and IP therapy in observational studies and small-scale clinical studies. The largest number of case enrollments in a clinical study of extrapulmonary NEC was seen in the Minnie-Pearl Cancer Research Network Study, which evaluated the efficacy of triple-drug combined therapy using carboplatin/etoposide/paclitaxel. The

response rate to this triple-drug combined therapy was 53%, while the MST was 14.5 months and adequate, there was intense toxicity involved, and the results were not significantly different from EP therapy, so this regimen was not considered the standard treatment[27].

The 2014 NCCN Guidelines recommend treating extrapulmonary NEC using a regimen according to small cell lung carcinoma, and the guidelines for treatment of small cell lung carcinoma mention both EP therapy and IP therapy as recommended regimens.

Therefore, the standard treatment for extrapulmonary NEC is taken as EP therapy or IP therapy.

Table 2.2.2.a. EP therapy for NEC (observational studies)

Reported year/Reporter	Target	N	Response rate	MST (in months)
1991/ Moertel[18]	Pancreatic/gastrointestinal NEC (anaplastic type)	18	67%	19
1999/ Mitry [36]	Pancreatic/gastrointestinal NEC	41	42%	15
2001/ Marie-Louise [37]	Pancreatic NET (well: 11, poorly: 4) Gastrointestinal cartinoid: 21	33	18%	19
1994/ Lo Re G [38]	Extrapulmonary SCLC	13	69%	NE
2010/ Iwasa [39]	Hepatobiliary or pancreatic NEC	21	14%	7.3
2012/ Yamaguchi [10]	Gastrointestinal NEC	12	75%	14
2012/ Yamaguchi [10]	Hepatobiliary or pancreatic NEC	34	12%	6.9
2012/ Sorbye [11]	Gastrointestinal primary lesion NEC (including primary lesion unknown)	129	31%	12

Table 2.2.2.b. IP therapy for NEC (shaded are clinical studies, others are observational studies)

Reported year/Reporter	Target	N	Response rate	MST (in months)
2003/ Hou [40]	NEC (gastrointestinal-80%)	18	43%	NE
2005/ Chin [41]	Esophageal NEC	12	83%	14
2011/ Okita [42]	Gastric NEC	12	75%	22.6
2012/ Yamaguchi [10]	Gastrointestinal NEC	142	51%	13.4
2012/ Yamaguchi [10]	Hepatobiliary or pancreatic NEC	18	39%	10.1
2006/ Kulke [43]	Pancreatic/gastrointestinal (including NET G1/G2)	15	7%	11.4
2008/ Mani [44]	Pancreatic/gastrointestinal NEC	20	58%	NE
2008/ Jin [45]	Extrapulmonary NEC	15	67%	11.4.

Table 2.2.2.c. Phase II study of carboplatin/etoposide/paclitaxel triple-drug combined therapy for NEC

Reported year/Reporter	Target (breakdown)	N	Response rate	MST (in months)
2006/ Hainsworth [27]	NEC (Colon：9, lung: 7, skin: 4, pancreas：3, gall bladder：1, thyroid:1, stomach: 1, esophagus: 1, endometrium:1, maxillary sinus: 1, prostate: 1, primary lesion unknown: 48)	78	53%	14.5

2.2.3. Standard treatment for gastrointestinal/hepatobiliary or pancreatic primary NEC

Treatments for extrapulmonary NEC have not been developed according to any specific organ, and the standard treatment for gastrointestinal/hepatobiliary or pancreatic NEC is also both, EP therapy and IP therapy. While both treatments are considered standard treatments, since this study considers them both to be study treatments, the anticipated effect and expected adverse reactions from EP therapy and IP therapy have been described in “2.3.2 Study treatment(s) of this study”.

Herewith, we describe the current status of the usage of each treatment in Japan and overseas. According to the multicenter joint study by Yamaguchi and Machida, 160 out of 258 patients (62%) who received systemic chemotherapy underwent IP therapy, the most common treatment, followed by EP therapy (46 patients, 18%). Although there is big deviation where 92% of gastrointestinal primary lesion NEC (142/154 patients) were given IP therapy, and 65% of hepatobiliary or pancreatic primary lesion NEC (34/52 patients) were given EP therapy, the present situation shows that treatments are being selected by discretion or preference of the facility/physician.

Furthermore, even with respect to the dosing schedule, the method of administration has not necessarily as per the treatment of small cell lung carcinoma. There is no unified consensus, as some facilities use methods used for treatment of gastric cancer (JCOG9912 regimen), based on the reasoning that they have familiarity with IP therapy. On the other hand, in the NORDIC NEC study the most common treatment used was the EP therapy, which was administered to 129 out of 252 patients (51%) who received systemic chemotherapy, followed by a combined therapy consisting of carboplatin and etoposide therapy (67 patients, 27%). Combined therapy of platinum and etoposide was used often, which is another choice of treatment in Japan[11].

2.3. Rationale for establishment of treatment plan

2.3.1. Drugs

1) Etoposide

Etoposide exerts an antitumor effect by inhibiting topoisomerase II, which catalyzes the untangling of supercoiled DNA strands. Main toxicities include myelosuppression, nausea/vomiting, alopecia, and stomatitis.

2) Cisplatin

It is a complex ion form of the heavy metal platinum which shows anti-tumor effect by cross-linking double-stranded DNA. Currently, it is considered one of the key drugs for NEC treatment, being a central medicine for the treatment of lung cancer due to its synergistic effect with radiation therapy and various drugs, and also due to its low bone marrow toxicity when used alone. Toxicities include nausea/vomiting, nephrotoxicity, and neurotoxicity. Sufficient infusion of cisplatin before and after drug administration is necessary to prevent nephrotoxicity.

3) Irinotecan

Irinotecan is a topoisomerase I inhibitor developed in Japan that inhibits DNA synthesis. The drug is directly converted into the active metabolite (SN-38) in human liver and various tissues by carboxyesterase. As it shows a potent antitumor effect against SCLC even when used on its own, this drug is used very often in routine clinical practice to treat NEC as well. Diarrhea and myelosuppression are observed as dose-dependent toxicities. Nausea/vomiting and interstitial pneumonia have also been noted in patients.

2.3.2. Study treatment(s) of this study

1) Etoposide/cisplatin combined therapy (EP therapy)

In the multicenter joint study performed by Yamaguchi and Machida, the response rate of gastrointestinal primary lesion NEC to EP therapy was 75% (9/12 patients), with an MST of 14 months. The response rate of hepatobiliary and pancreatic primary lesion NEC to EP therapy was 12% (4/34 patients), and the MST was 6.9 months. With respect to safety, the multicenter joint study by Yamaguchi and Machida showed no treatment-related deaths associated with EP therapy (N = 46) administered as first-line treatment for gastrointestinal/hepatobiliary and pancreatic primary lesion NEC, and toxicity-related study discontinuation was observed in 6.5% of cases. Details of toxicities remain unknown as they were not investigated. The observational study of EP therapy for hepatobiliary and pancreatic primary lesion NEC (N = 21) carried out by Iwasa et al. at the National Cancer Center Hospital showed major Grade 3/4 adverse events to be neutropenia (90%), nausea (33%), and anorexia (24%). Grade 3 febrile neutropenia was observed in 8 patients (38%)[39]. Table 2.3.2 shows the toxicity profile of EP therapy from the JCOG9511 study which treated small cell lung carcinoma (Grade 3 or higher as per JCOG toxicity criteria).

Table 2.3.2. Toxicity of EP therapy and IP therapy in studies treating small cell lung carcinoma※
※Report by Iwasa et al., JCOG0509：CTCAE v3.0 Grade 3 or higher, JCOG9511：JCOG toxicity criteria Grade 3 or higher

	EP therapy		IP therapy	
	Report by Iwasa et al.[34]	JCOG9511[27]	JCOG9511[27]	JCOG0509[30]
Neutropenia	90%	92.2%	65.3%	58.5%
Leukopenia	71%	51.9%	26.7%	22.5%
Decreased hemoglobin	29%	29.9%	26.7%	23.2%
Thrombocytopenia	24%	18.2%	5.3%	2.1%
Diarrhea	0%	0%	16%	7.7%

Nausea	33%	6.5%	13.3%	6.3%
Aspartate aminotransferase increased	19%	2.6%	0%	-
Alanine aminotransferase increased	24%	3.9%	4%	-
Blood bilirubin increased	19%	0%	0%	-
Creatinine increased	0%	0%	0%	-
Peripheral motor neuropathy	0%	0%	0%	-
Febrile neutropenia	38%	0%	0%	1.4%

There are several reports concerning the specific dosage schedule/administered dose for EP therapy, and four dosing methods have been described in the NCCN Guidelines for SCLC as well. However, it is not clear as to which of the dosing methods is the best, and the dosing method for NEC varies between reports. In Japan, the dosing for EP therapy according to JCOG9511 is also often used for NEC. For this reason, this study would also follow the same method of administration. In terms of the number of cycles, a comparison of 4 courses vs. 8 courses of combined chemotherapy including cyclophosphamide for SCLC showed poor efficacy even when the treatment continued for long-term[46], and treatment of 4 cycles has also been specified in JCOG9511. There have been no such comparative studies for gastrointestinal/hepatobiliary and pancreatic primary lesion NEC, rather, there is no evidence to suggest that first-line chemotherapy should be discontinued if the chemotherapy is efficacious and toxicity is within the permitted scope. Unlike SCLC, NEC does not respond well to chemotherapy and tends to re-exacerbation to the underlying disease soon after the end of chemotherapy. For this reason, continuation of chemotherapy is very likely to be beneficial for patients with NEC as long as the treatment is effective.

While peripheral motor neuropathy, hearing impairment, and renal disorder are known accumulation toxicities of cisplatin, if sufficiently safe, it is ideal for treatments to continue as much as possible, considering the very few treatment options available for NEC. Based on the above rationale, we decided to ensure safety by establishing strict protocol treatment discontinuation criteria and chose not to limit the total dose or prescribe the number of treatment cycles.

2）Irinotecan/Cisplatin combined therapy (IP therapy)

In the multicenter joint study by Yamaguchi and Machida, the response rate of gastrointestinal primary lesion NEC to IP therapy was 51% (73/142 patients), with an MST of 13.4 months. The response rate of hepatobiliary and pancreatic primary lesion NEC to IP therapy was 39% (7/18 patients), and the MST was 10.1 months. There were no treatment-related deaths among 160 patients, and discontinuation due to toxicity in first-line chemotherapy was seen in 11.2% of cases. Details about toxicity were not collected. As a reference, Table 2.3.2 shows the toxicity profile of IP therapy from the JCOG9511 and JCOG0509 studies which treated small cell lung carcinoma.

There are several reports concerning specific dosing methods for IP therapy, and two dosing methods have been described in the NCCN Guidelines for SCLC as well. It is not known which of the dosing methods is the best, and the dosing method has also been variable in the NEC. In Japan, the dosing method of IP therapy, according to JCOG9511 is considered the standard treatment for SCLC and the same method has also been used in this study.

The total dose and number of cycles would have not been prescribed for the same reason as EP therapy, and safety has been ensured by establishing strict protocol treatment discontinuation criteria.

3）Other study treatment candidates

Presently, there are no drugs or treatment methods, including molecular-targeted drugs, which surpass the EP and IP therapy. There are also no planned or ongoing large-scale clinical trials globally, related to first-line treatment of NEC.

As the second-line treatment, a phase II study of Everolimus is underway for pancreatic primary lesion NEC resistant or unresponsive to platinum preparations. It is a multicenter study being carried out in 31 facilities in Japan, led by the National Cancer Center East Hospital, through the cancer research and development fund (UMIN000012752）.

2.3.3. Summary of risk/benefit balance of the standard treatment and study treatment

In the multicenter joint study by Yamaguchi and Machida, IP therapy had better prognosis compared to EP therapy (MST: 13.0 months vs. 7.3 months; $p < 0.0001$). However, as shown in Table 2.3.3, since IP therapy was more frequently chosen for gastrointestinal primary lesions (142/154 patients) and EP therapy for hepatobiliary and

pancreatic primary lesions (34/52 patients), it has not been possible to determine whether this difference is due to difference in efficacy between the regimens, or due to differences in the primary lesion organ. Primary lesion organ remained a significant prognostic factor after multivariate analysis, and while IP therapy turned out to be a slightly better regimen than EP therapy with HR = 0.8 (95% CI. 0.48–1.33), $p = 0.389$ indicated that there was no significant difference between the two (see “2.1.5 Prognostic/predictive factors”). Taking into account the number of EP therapy cases (46 patients), the fact that it was an observational study, and including other unknown bias, the data interpretation is fairly limited. Therefore, based on these results we are unable to deem either of IP or EP therapies to be more efficacious than the other.

With regard to toxicity, while myelosuppression such as neutropenia is milder in IP therapy than EP therapy, frequencies of events such as diarrhea and nausea are high. While the two treatments have different toxicity profiles, it is difficult to conclude that one has clearly worse toxicity than the other. The risk of bile duct obstruction is high in biliary and pancreatic primary lesion NECs and the use of irinotecan (which undergoes biliary excretion) in patients exhibiting biliary excretion disorder runs the risk of increased toxicity, hence care must be taken while administering irinotecan. We believe that this risk can be avoided by appropriate pre-treatment drainage and proper monitoring during treatment. Based on the above, it is difficult to assign superiority to IP therapy or EP therapy over the other in terms of risk/benefit balance, and both the regimens can be considered suitable standard treatments.

Table 2.3.3. Summary of results from multicenter study by Yamaguchi and Machida

		IP	EP	*p*-value*
Total	N	160	46	
	Response rate（%）	50	27	＜0.001
	Median PFS (in months)	5.2	4.0	0.033
	Median OS (in months)	13.0	7.3	＜0.0001
Gastrointestinal primary lesion NEC	N	142	12	
	Response rate（%）	51	75	0.14
	Median PFS (in months)	5.4	4.9	0.585
	Median OS (in months)	13.4	14.0	0.976
Hepatobiliary and pancreatic primary lesion NEC	N	18	34	
	Response rate（%）	39%	12%	0.034
	Median PFS (in months)	4.4	3.7	0.056
	Median OS (in months)	10.1	6.9	0.05

*Response rate by chi-squared test, PFS and OS by log-rank test

2.3.4. Post-treatment(s)

In the multicenter joint study by Yamaguchi and Machida, 56% (116/206 patients) of patients with NEC who received IP therapy or EP therapy were administered chemotherapy as the second-line of treatment. The most common second-line chemotherapy after IP therapy was amurcibin (22/88 patients), while irinotecan was the most common second-line treatment after EP therapy (13/28 patients), and we expected a similar pattern for the second-line treatments in this study as well. The overall performance of second-line chemotherapy was poor with a response rate of 11%, and PFS of 2.1 months. As such, the significance of the second-line treatment has not been indicated, and hence, no standard treatment has been established.

2.4. Study design

2.4.1. Rationale for establishing endpoints

For NECs, an alternative endpoint for overall survival, such as PFS has not been established as an indicator for comparing the usefulness of the treatment regimens. Therefore, comparison of overall survival was considered appropriate when examining the therapeutic effects. For the same, overall survival was examined as the primary endpoint. The secondary endpoints to evaluate the efficacy and safety were the response rate (only for patients with a measurable lesion), progression-free survival, incidence rate of adverse events, and dose intensity of cisplatin.

2.4.2. Clinical hypothesis and rationale for setting the number of enrollments

This study compares the two standard treatments, both of which are difficult to deem superior than the other in terms of efficacy and safety. We have, therefore, adopted a study design using bilateral testing. The main clinical hypothesis of this study is that either IP therapy or EP therapy is likely to provide better overall survival than the other treatment group, and should this hypothesis be validated, the superior treatment with statistical significance would be deemed the better treatment, and would therefore be positioned as the standard treatment in future. If the study fails to validate this hypothesis, it would indicate that there was no clinically significant difference in overall survival between the two groups, and if there are no large differences in terms of toxicity, we would conclude that both treatments are viable options as the standard treatment. However, if unlike our initial hypothesis, one treatment shows clearly more toxicity than the other, we would reconsider the study design before carrying out the main analysis.

Furthermore, as mentioned in "section 2.1.1 Epidemiology", gastroenterological primary lesion NEC, which is the primary target of this study, is a very rare disease with an annual prevalence of around 3 in 100,000 people, hence, the significance level of testing has been placed at 10% bilaterally, instead of 5% bilaterally. In the multicenter joint study conducted by Yamaguchi and Machida, the MST of gastrointestinal/hepatobiliary or pancreatic primary lesion NEC was 11.5 months. To determine if one treatment is superior to the other, we expected a difference of four months in terms of MST. If we expect the more inferior treatment to have an MST of 8.0 months and the superior treatment to have an MST of 12.0 months, the number of enrollments are calculated using the aforementioned parameters and based on the discussion mentioned later (see "12.2 Expected number of enrollments/Enrollment period/Follow-up period"). This provided a research period of 7- years, consisting of 6-years of enrollment period and one year of follow-up period, and with $\alpha = 0.1$ bilaterally and detection power of 70% to detect differences between the two groups, 63 subjects were required per group. Taking into consideration some subjects who could be lost post follow-up, we aimed to enroll 70 subjects per group, and a total of 140 subjects between two groups.

If enrollment proceeds better than expected than prior to the start of the study (i.e. if the number of enrolled subjects reach 70 in less than 2.5 years from the start of enrollment), the number of enrollments is likely to be re-established by changing the detection power from 70% to 80% during the study, with the aim of obtaining more accurate results.

＜Addition to ver.1.1＞

The pace of enrollment after the start of enrollment period exceeded expectations, and the number of enrollments reached 70 subjects in October 2016, which was 2 years and 2 months after the start. As this exceeded the criteria of 70 subjects in less than 2.5 years, since the start as provided above, we obtained the approval of the Hepatobiliary and Pancreatic Oncology Group Meeting on October 29, 2016, Esophageal Cancer Group Meeting on November 19, 2016, and Gastric Cancer Group Meeting on January 7, 2017 to change the detection power to 80% according to provisions at the time of study planning. Consequently, the number of intended enrollments was changed to 170 subjects.

2.4.3. Expected patient enrollment

In the multicenter joint study by Yamaguchi and Machida, 258 cases of gastrointestinal/hepatobiliary or pancreatic primary lesion NECs were reported from 23 sites during the 11 years between 2000 and 2011, but between 2000 and 2006, information could not be obtained because of old cases. When limited to the most recent five years (2006–2011), when sufficient information was obtained from each facility, the number of enrollments was placed at162 subjects.

This is a joint study between three groups, namely the JCOG Hepatobiliary and Pancreatic Oncology Group,

JCOG Gastric Cancer Group, and the JCOG Esophageal Cancer Group. Therefore, the total number of facilities, excluding overlaps, amounted to 82 facilities. Compared to the multicenter joint study by Yamaguchi and Machida, we expect an increase in the number of patients enrolled, and simple calculations lead us to extrapolate 115 subjects to be enrolled annually. On the other hand, if we take into account the possibility of patient enrollments being biased to some high volume centers, it would be difficult to estimate the effect of increasing facility count to proceed. In addition, considering that the study is a randomized, we estimate the annual number of patients enrolled to be between 30–50 people. Taking into account ineligible cases, we provided 6-years for patient enrollment period.

2.4.4. Rationale for setting allocation adjustment factors

1) Facility

It is widely known that background, treatment, efficacy evaluation, and safety evaluation of enrolled patients vary depending on the facility, and JCOG standards have been used to make adjustments between facilities.

2) Primary lesion organ (gastrointestinal tract vs. hepatobiliary or pancreatic organ)

In the multicenter joint study by Yamaguchi and Machida, multivariate analysis using the Cox proportional hazard model was carried out with 183 subjects, which indicated primary lesion organ (gastrointestinal tract vs. hepatobiliary or pancreatic organ) to be a significant prognostic factor.

2.4.5. Centralized pathological diagnosis

In this study, a centralized pathological diagnosis would be performed for analysis of NEC, despite patients being diagnosed by a third party. The operation of the centralized pathological diagnosis is described in section 15.1, while details of the operation have been described in the Centralized Pathological Diagnosis Procedure Manual. Furthermore, an analysis of endpoints based on the results of centralized pathological diagnosis would be provided as a reference.

2.5. Summary of expected advantages and disadvantages associated with study participation

2.5.1. Expected advantages

Drugs used for both groups of this study are treatments used in routine medical practice. As mentioned in the next section, although it would be necessary to use some drugs for which insurance coverage does not apply, since insurance claims for these treatments are made in a similar manner to general medical care however, in practice they do not receive insurance assessments.

Furthermore, cases of off-label medication have been studied according to "18th Case Providing Review Information" at the Case Review Committee established by the Health Insurance Claims Review & Reimbursement Services. Based on the review information provided as on February 26, 2018;, The use of "irinotecan hydrochloride hydrate, etoposide, cisplatin, and carboplatin [injections]" is approved for treatment of "neuroendocrine carcinoma"], and the notices issued by the Ministry of Health, Labor and Welfare on February 26, 2016the results of this study have been considered valid by the ministry. For the same reason, essentially since February 26, 2018, there have been no concerns about claims of insurance assessments.

Moreover, since the medical fees of study participants during the study, which includes drug fees, are paid in principle by the patients themselves and their insurance coverage, so the patients would receive no special medical or financial benefits from participation in this study.

2.5.2. Expected risks and disadvantages

Both treatment arms A and B would receive chemotherapy regimen used in routine medical practice, so they would be unlikely to be exposed to special risks or disadvantages not observed in routine medical practice. The descriptions in "2.3.3 Risk/benefit balance of standard treatment and study treatments" outline the expected risks and disadvantages for each treatment arm.

To minimize the risk of adverse events and disadvantages, the " Patient selection criteria (Section 4)", " Criteria to change treatment (Section 6.3)" and "Concomitant/Supportive therapies (Section 6.4)" have been carefully considered for the three groups. Furthermore, the Institutional Review Board would monitor if the adverse events are within the scope of expectation, while any serious adverse events or unexpected adverse events would be carefully examined and reviewed according to the provisions related to "JCTN-Adverse Events Report Guideline"

and "JCOG Guidelines for Handling Clinical Safety Information", and a system has been provided to take any necessary countermeasures.

Precautions related to etoposide, irinotecan, and cisplatin

As of December 2013, etoposide therapy, which is the intended to be used in this study had not received insurance approval for the treatment of gastrointestinal or hepatobiliary and pancreatic primary lesion cancers. Irinotecan has also not received insurance approval for treatment of cancers with primary lesions in organs other than the stomach, colon, and rectum. Furthermore, as cisplatin is only indicated for esophageal, gastric cancer, and for biliary tract cancers when combined with gemcitabine (25 mg/m^2), the dosage and administration used in this study have not been approved previously.

As each facility in this study may carry out the insurance claims in the same manner as routine general medical practice, the treatment may receive insurance assessment after-the-fact. However, if a facility incurs losses, the loss must be borne by the relevant facility (medical institution), as there is no system of compensation arranged by the JCOG Research Organization. If actual losses occur, the continued participation in the study would then be carefully discussed between the facility supervisor and the principal investigator/clinical trial secretariat. The facility supervisor would be expected to gain approval from the facility IRB and the head of the medical institutions.

2.6. Significance of this study

While both, EP therapy and IP therapy are standard treatments, the efficiency of either treatment being better than the other is not yet clear, and currently in routine medical practice, the choice of treatment is being made on the discretion or preference of the facility/attending physician.

If this study is able to clearly indicate the efficiency of EP therapy or IP therapy over the other, we expect that the diminished use of the relatively inferior treatment would contribute to improvement in patient prognosis. If the study indicates no clinically meaningful differences between the two treatments, the results would provide evidence that there is no significant issue with the treatments being chosen by discretion or preference of the facility/attending physician, which has been done traditionally without any data that directly compare the two treatments, although that would not indicate that the effects of the two treatments are equivalent. We also expect that the establishment of a highly reliable standard treatment in this study which would provide a foundation for therapeutic development when promising treatment regimen in the future.

As NEC is a rare disease, and since each clinician is not likely to have adequate experience, we believe that work associated with multicenter studies such as confirmation of diagnosis by centralized diagnosis/results feedback and sharing results of treatments by unified treatment regimen would lead to improvement of quality of care for NEC in Japan.

2.7. Associated research (including sample analysis research)

No such studies have been planned or carried out at the time of preparation of the protocol.

2.8. JCOG-Biobank Japan (BBJ) collaborating biobank

This study would participate in the banking of blood samples (DNA/plasma) through the JCOG-BBJ collaboration biobank based on the protocol common to all JCOG studies (hereafter termed as "common banking").

Common banking by JCOG-BBJ collaborating biobank would collect and store the samples of patients enrolled in clinical studies conducted by JCOG regardless of presence/absence of pre-planned sample analysis research, would also provide samples for future analysis research and diagnostic information obtained through the main research.

The target of such biobanking are those patients who provided consent to participate in this study and gave consent to provide samples to the JCOG-BBJ collaborating biobank for their use in future sample analysis research (hereafter termed as consent for banking).

Samples collected during common banking include whole blood and preserved pathological tissue samples from routine clinical practice. Plasma and DNA separated/isolated from blood would be stored in the JCOG-BBJ collaborating biobank and would be provided for sample analysis research in the future. Preserved pathological tissue samples from routine medical practices such as surgery, and biopsy/clinical laboratory tests would also likely to be used for future sample analysis research. Although the type, sample preparation methods, and tissue quantity required would vary from study to study, there is no definite consensus that banking pathological tissue in a certain

method is more efficient than others. Furthermore, it has been suggested that long-term preservation of a sectioned specimen from preserved pathological tissue may lead to sample deterioration (DNA fragmentation). The discussions between the of JCOG and BBJ personnel about these problems, led to the conclusion that preserved pathological tissue taken after medical examination shall require only patients' consent for future use, and actual collection must be started after standardizing a separate protocol and specifying in it the procedures best suited for the details of the research.

The detailed procedures for sample collection, storage, and method of sample provision for future sample analysis research in common banking have been stipulated in the "JCOG-BioBank Japan Collaborating Biobank Protocols" that apply to all the JCOG studies. To participate in the common banking carried out by JCOG-BBJ collaborating biobanks, the subject matter must be reviewed and approved by the ethics committees of each participating facility.

Furthermore, to conduct sample analysis research in future using samples stored in the JCOG-BBJ collaborating biobank, it would be necessary to prepare a "Sample Analysis Research Protocol" and have it reviewed and approved by the JCOG Protocol Review Committee and the ethics committee of facilities involved in the sample analysis.

3. Criteria/definitions used in this study

Tissue classification would be performed according to WHO 2010 classification and ENETS（European Neuroendocrine Tumor Society） classification[14, 15], while disease staging would be done according to "UICC-TNM 7th Edition".

3.1. Tissue classification (WHO 2010 classification)

The shaded parts are the targets of this study

Neuroendocrine neoplasms

1) Neuroendocrine tumor：NET Grade 1（NETG1）
2) Neuroendocrine tumor：NET Grade 2（NETG2）
3) Neuroendocrine carcinoma：NEC（large cell or small cell type）
4) Mixed adenoendocrine carcinoma（MANEC）
5) Hyperplastic and preneoplastic lesions

3.2. Grade classification （ENETS [European Neuroendocrine Tumor Society] / WHO2010 classification）

Grade 1（G1）	Number of mitotic presentations < 2 per 10 high power fields（HPF）and/or Ki-67 index ≤2%＊
Grade 2（G2）	Number of mitotic presentations 2–20 per 10 HPF and/or Ki-67 index 3-20%
Grade 3（G3）	Number of mitotic presentations > 20 per 10 HPF and/or Ki-67 index >20%

＊The description of number of mitotic presentations in WHO 2010 classification is ≤2, but 2–3% are classified to G1[47].

3.3. Histopathological diagnosis

- Immunostaining (Chromogranin A and synaptophysin) is essential for a pathological diagnosis of NEC.
- Either1. or 2., or both are studied to determine proliferative activity. Number of mitotic presentations and Ki-67 index would adopt a high grade evaluation.
  1. Number of mitotic presentations (to evaluate 50HPF)
  2. Ki-67 index（500–2000 tumor cells*）

  *（Only in this study, if the number of tumor cells in the collected sample is less than 500, the number of tumor cells measured is also listed. A minimum of 100 tumor cells is required.）
- If sufficient amount of biopsy sample cannot be collected for a pathological diagnosis, a cell block prepared using material obtained from EUS-FNA, brushing or needle biopsy may be used. However, the use of a cell block prepared from ascites or pleural effusion is not permitted.
- During a histological diagnosis using a resected tumor sample, a slide with a representative section shall be used to evaluate the proportion of NEC components (differential diagnosis with mixed adenoendocrine carcinoma). For a comprehensive examination, the entire tumor must be evaluated.

For further details NEC Pathological Diagnosis Handbook would be referred (posted on JCOG website).

3.4. Disease stage classification criteria (UICC-TNM 7th Edition)

Special notice regarding disease stage classification: Although there is an independent TNM classification for NET G1 and NET G2 with primary lesions in the stomach, small intestines, and colon; since this study concerns NEC, the disease would be classified according to the classification method of major tissue type in each primary lesion organ (squamous cell carcinoma for esophagus, and adenocarcinoma for all other primary lesion organs). In this study, the classification of intrahepatic cholangiocarcinoma is used for hepatic NEC, due to their similar clinical presentation.

3.4.1. Esophagus

T –Primary tumor

TX： Primary tumor cannot be evaluated
T0： Primary tumor unidentified
Tis： Epithelial carcinoma/highly dysplastic
T1： Tumor invading mucosal lamina propria, muscularis mucosae, or submucosa
  T1a：Tumor invading the mucosal lamina propria or muscularis mucosae
  T1b： Tumor invading the submucosa

T2: Tumor invading the muscularis propia
T3: Tumor invading the adventitia
T4: Tumor invading the surrounding tissue
T4a: Tumor invading the pleura, pericardium, and diaphragm
T4b: Tumor invading other surrounding tissues such as the aorta, centrum, trachea

N –Regional lymph nodes
NX: Regional lymph node metastasis cannot be evaluated
N0: No regional lymph node metastasis
N1: 1–2 foci of regional lymph node metastases
N2: 3–6 regional foci of lymph node metastases
N3: 7 or more foci of regional lymph node metastases

M –Distal metastasis
M0: No distal metastasis
M1: Distal metastasis present

Stage	T	N	M
**0**	Tis	N0	M0
**IA**	T1	N0	M0
**IB**	T2	N0	M0
**IIA**	T3	N0	M0
**IIB**	T1, T2	N1	M0
**IIIA**	T4a	N0	M0
	T3	N1	M0
	T1, T2	N2	M0
**IIIB**	T3	N2	M0
**IIIC**	T4a	N1、N2	M0
	T4b	Unrelated to N	M0
	Any T	N3	M0
**IV**	Any T	Any N	M1

3.4.2. Stomach

T –Primary tumor
TX: Primary tumor cannot be evaluated
T0: Primary tumor unidentified
Tis: Epithelial carcinoma: Epithelial carcinoma/highly dysplastic carcinoma not invading the lamina propia mucosa
T1: Tumor invading mucosal lamina propria, muscularis mucosae, or submucosa
T1a: Tumor invading the mucosal lamina propria or muscularis mucosae
T1b: Tumor invading the submucosa
T2: Tumor invading the muscularis propia
T3: Tumor invading the subserosa
T4: Tumor perforating the serosa, or invading adjacent structures[1,2,3]
T4a: Tumor perforating the serosa
T4b: Tumor invading adjacent structures[1,2,3]

1. Adjacent organs of the stomach are spleen, transverse colon, liver, diaphragm, pancreas, abdominal wall, adrenal glands, kidneys, small intestine, and retroperitoneum.
2. If the invasion has spread from the stomach to the duodenum or esophagus, classification is made in terms of depth.
3. A tumor that advances into the gastrocolic ligament, into the hepatogastric ligament, or into the greater or lesser omentum, and is classified as T3, when there is no perforation of the visceral peritoneum.

N –Regional lymph nodes

NX: Regional lymph node metastasis cannot be evaluated

N0: No regional lymph node metastasis

N1: 1–2 regional foci of lymph node metastases

N2: 3–6 regional foci of lymph node metastases

N3: 7 or more regional foci of lymph node metastases

N3a: 7–15 regional foci of lymph node metastases

N3b: 16 or more regional foci of lymph node metastases

M –Distal metastasis

M0: No distal metastasis

M1: Distal metastasis present

Stage	T	N	M
**0**	Tis	N0	M0
**IA**	T1	N0	M0
**IB**	T2	N0	M0
	T1	N1	M0
**IIA**	T3	N0	M0
	T2	N1	M0
	T1	N2	M0
**IIB**	T4a	N0	M0
	T3	N1	M0
	T2	N2	M0
	T1	N3	M0
**IIIA**	T4a	N1	M0
	T3	N2	M0
	T2	N3	M0
**IIIB**	T4b	N0, N1	M0
	T4a	N2	M0
	T3	N3	M0
**IIIC**	T4a	N3	M0
	T4b	N2, N3	M0
**IV**	Any T	Any N	M1

3.4.3. Small intestines (including duodenum)

T –Primary tumor

TX: Primary tumor cannot be evaluated

T0: Primary tumor unidentified

Tis: Epithelial carcinoma

T1: Tumor invading mucosal lamina propria, muscularis mucosae, or submucosa

T1a: Tumor infiltrating the mucosal lamina propria or muscularis mucosae

T1b: Tumor infiltrating the submucosa

T2: Tumor infiltrating the muscularis propia

T3: Tumor invading the subserosa, or tumor invading within 2 cm of surrounding tissue of muscularis externa without peritoneal cover (mesenterium, retroperitoneum)*

*Surrounding tissue of muscularis externa without peritoneal cover refers to the mesenterium in the jejunum and ileum, and the retroperitoneum in the duodenum without serosa.

T4: Tumor penetrating the visceral peritoneum, or tumor directly invading another organ or tissue (invasion of other loops of the small intestine, invasion by 2 cm or more into the mesenterium and retroperitoneum, including invasion into the abdominal wall through the serosa; invasion to the pancreas only for duodenum)

N –Regional lymph nodes

NX: Regional lymph node metastasis cannot be evaluated

N0: No regional lymph node metastasis

N1 : 1–3 regional foci of lymph node metastases

N2 : 4 or more regional foci of lymph node metastases

M –Distal metastasis

M0 : No distal metastasis

M1 : Distal metastasis present

Stage	T	N	M
**0**	Tis	N0	M0
**I**	T1, T2	N0	M0
**IIA**	T3	N0	M0
**IIB**	T4	N0	M0
**IIIA**	Any T	N1	M0
**IIIB**	Any T	N2	M0
**IV**	Any T	Any N	M1

3.4.4. Appendix (partial revision of UICC-TNM 7th Edition)

T –Primary tumor

TX : Primary tumor cannot be evaluated

T0 : Primary tumor unidentified

Tis : Epithelial carcinoma: Tumor invading epithelium or lamina propia mucosae

T1 : Tumor invading the submucosa

T2 : Tumor invading the muscularis propia

T3 : Tumor invading the subserosa or mesoappendix

T4 : Tumor penetrating the visceral peritoneum, tumor including a peritoneal mucosal tumor in the lower right abdomen and/or tumor directly invading other organs or tissue

T4a : Tumor penetrating the visceral peritoneum, or peritoneal mucosal tumor in the lower right abdomen

T4b : Tumor directly invading other organs or tissues

N –Regional lymph nodes

NX : Regional lymph node metastasis cannot be evaluated

N0 : No regional lymph node metastasis

N1 : 1–3 regional foci of lymph node metastases

N2 : 4 or more foci of regional lymph node metastases

M –Distal metastasis

M0 : No distal metastasis

M1 : Distal metastasis present

M1a : Peritoneal metastasis beyond the lower right abdomen, including pseudomyxoma peritonei

M1b : Distal metastasis other than peritoneal metastasis

Stage	T	N	M
**0**	Tis	N0	M0
**I**	T1, T2	N0	M0
**IIA**	T3	N0	M0
**IIB**	T4a	N0	M0
**IIC**	T4b	N0	M0
**IIIA**	T1, T2	N1	M0
**IIIB**	T3, T4	N1	M0
**IIIC**	Any T	N2	M0
**IVA**	Any T	N0	M1a
**IVB**	Any T	N0	M1a
	Any T	N1, N2	M1a
**IVC**	Any T	Any N	M1b

3.4.5. Colon and rectum

T –Primary tumor

TX : Primary tumor cannot be evaluated

T0 : Primary tumor unidentified

Tis : Carcinoma in situ: Tumor invading epithelium or lamina propia mucosae

T1 : Tumor invading the submucosa

T2 : Tumor invading the muscularis propia

T3 : Tumor invading the subserosa, or tissue surrounding the colon or rectum without peritoneal covering

T4 : Tumor penetrating the visceral peritoneum, and/or directly invading another organ or tissues

T4a : Tumor penetrating the visceral peritoneum

T4b : Tumor directly invading another organ or tissues

N –Regional lymph nodes

NX : Regional lymph node metastasis cannot be evaluate

N0 : No regional lymph node metastasis

N1 : 1–3 regional foci of lymph node metastases

N1a : 1 regional lymph node metastasis

N1b : 2–3 regional foci of lymph node metastases

N1c : Presence of tumor deposits, that is, satellite nodes in the subserosa or in the soft tissue surrounding colon or rectum without peritoneal covering, but with no regional lymph node metastasis

N2 : 4 or more regional lymph node metastases

N2a : 4–6 regional foci of lymph node metastases

N2b : 7 or more regional foci of lymph node metastases

M –Distal metastasis

M0 : No distal metastasis

M1 : Distal metastasis present

M1a : Local metastasis to one organ (liver, lungs, ovaries, or lymph nodes other than regional lymph nodes)

M1b : 2 or more organs, or peritoneal metastasis

Stage	T	N	M
**0**	Tis	N0	M0
**I**	T1, T2	N0	M0
**II**	T3, T4	N0	M0
**IIA**	T3	N0	M0
**IIB**	T4a	N0	M0
**IIC**	T4b	N0	M0
**III**	Any T	N1, N2	M0
**IIIA**	T1, T2	N1	M0
	T1	N2a	M0
**IIIB**	T3, T4	N1	M0
	T2, T3	N2a	M0
	T1, T2	N2b	M0
**IIIC**	T4a	N2a	M0
	T3, T4a	N2b	M0
	T4b	N1, N2	M0
**IVA**	Any T	Any N	M1a
**IVB**	Any T	Any N	M1b

3.4.6. Applies to hepatic NEC (hepatic primary lesion or liver metastasis from unknown primary lesion)

T –Primary tumor

TX : Primary tumor cannot be evaluated

T0 : Primary tumor unidentified

Tis : Carcinoma *in situ*

T1 : Isolated tumor without vascular invasion

T2a : Isolated tumor with vascular invasion

T2b : Multifocal tumor regardless of vascular invasion

T3 : Tumor penetrating visceral peritoneum or directly invading adjacent extrahepatic structures

T4 : Tumor with bile duct invasion (bile duct proliferative type)

N –Regional lymph nodes

NX : Regional lymph node metastasis cannot be evaluated

N0 : No regional lymph node metastasis

N1 : Regional lymph node metastasis present

M –Distal metastasis

M0 : No distal metastasis

M1 : Distal metastasis present

Stage	T	N	M
**I**	T1	N0	M0
**II**	T2	N0	M0
**III**	T3	N0	M0
**IVA**	T4	N0	M0
	Any T	N1	M0
**IVB**	Any T	Any N	M1

3.4.7. Gall bladder

T –Primary tumor

TX : Primary tumor cannot be evaluated

T0 : Primary tumor unidentified

Tis : Carcinoma *in situ*

T1 : Tumor invading mucosal lamina propria or muscularis externa

T1a : Tumor invading mucosal lamina propria

T1b : Tumor invading muscularis externa

T2 : Tumor invading the connective tissue around the muscularis externa, but shows no progression beyond the serosa or to the liver

T3 : Tumor perforating the serosa (visceral peritoneum), tumor directly advancing to the liver and/or an adjacent organ that is not the liver (stomach, duodenum, colon, pancreas, omentum, or extrahepatic bile duct)

T4 : Tumor invading the main trunk of the portal vein or the hepatic artery, or tumor advancing to two or more adjacent organs which are not the liver

N –Regional lymph nodes

NX : Regional lymph node metastasis cannot be evaluated

N0 : No regional lymph node metastasis

N1 : Regional lymph node metastasis present (cystic duct, common bile duct, proper hepatic artery, including lymph nodes along the portal vein)

M –Distal metastasis

M0 : No distal metastasis

M1 : Distal metastasis present

Stage	T	N	M
**0**	Tis	N0	M0
**I**	T1	N0	M0
**II**	T2	N0	M0
**IIIA**	T3	N0	M0
**IIIB**	T1, T2, T3	N1	M0
**IVA**	T4	Any N	M0

**IVB**	Any T	Any N	M1

3.4.8. Extrahepatic bile duct-hepatic portal region

T –Primary tumor

TX : Primary tumor cannot be evaluated

T0 : Primary tumor unidentified

Tis : Carcinoma *in situ*

T1 : Tumor localized to the bile duct which advances until muscularis externa or fibrous tissue

T2a : Tumor invading beyond the bile duct wall and into the surrounding adipose tissue

T2b : Tumor invading the adjacent liver parenchyma

T3 : Tumor invading the branch of one side of portal vein or hepatic artery

T4 : Tumor invades the main trunk of portal vein, and branches on both sides of the portal vein, proper hepatic artery or the secondary branches of the bile duct on both left and right side, or to the secondary bile duct branches on one side and portal vein or hepatic artery on the other side

N –Regional lymph nodes

NX : Regional lymph node metastasis cannot be evaluated

N0 : No regional lymph node metastasis

N1 : Presence of regional lymph node metastasis in the cystic duct, common bile duct, proper hepatic artery, and lymph nodes along portal vein

M –Distal metastasis

M0 : No distal metastasis

M1 : Distal metastasis present

Stage	T	N	M
**0**	Tis	N0	M0
**I**	T1	N0	M0
**II**	T2a, T2b	N0	M0
**IIIA**	T3	N0	M0
**IIIB**	T1,T2, T3	N1	M0
**IVA**	T4	N0, N1	M0
**IVB**	Any T	Any N	M1

3.4.9. Extrahepatic bile duct-distal

T –Primary tumor

TX : Primary tumor cannot be evaluated

T0 : Primary tumor unidentified

Tis : Carcinoma *in situ*

T1 : Tumor localized in the bile duct wall

T2 : Tumor invading beyond the bile duct wall

T3 : Tumor invading the gallbladder, liver, pancreas, duodenum, or other adjacent organs

T4 : Tumor invading the celiac axis or superior mesenteric artery

N –Regional lymph nodes

NX : Regional lymph node metastasis cannot be evaluated

N0 : No regional lymph node metastasis

N1 : Regional lymph node metastasis present

M –Distal metastasis

M0 : No distal metastasis

M1 : Distal metastasis present

Stage	T	N	M
**0**	Tis	N0	M0
**IA**	T1	N0	M0
**IB**	T2	N0	M0

**IIA**	T3	N0	M0
**IIB**	T1, T2, T3	N1	M0
**III**	T4	Any N	M0
**IV**	Any T	Any N	M1

3.4.10. Ampulla of Vater

T –Primary tumor

TX: Primary tumor cannot be evaluated

T0: Primary tumor unidentified

Tis: Carcinoma *in situ*

T1: Tumor localized to the ampulla of Vater, or sphincter of Oddi

T2: Tumor invading the duodenal wall

T3: Tumor invading the pancreas

T4: Tumor invading the soft tissue around the pancreas or other adjacent organs

N –Regional lymph nodes

NX: Regional lymph node metastasis cannot be evaluated

N0: No regional lymph node metastasis

N1: Regional lymph node metastasis present

M –Distal metastasis

M0: No distal metastasis

M1: Distal metastasis present

Stage	T	N	M
**0**	Tis	N0	M0
**IA**	T1	N0	M0
**IB**	T2	N0	M0
**IIA**	T3	N0	M0
**IIB**	T1, T2, T3	N1	M0
**III**	T4	Any N	M0
**IV**	Any T	Any N	M1

3.4.11. Pancreas

T –Primary tumor

TX: Primary tumor cannot be evaluated

T0: Primary tumor unidentified

Tis: Carcinoma *in situ*

T1: Tumor localized within the pancreas, with a maximum size of < 2 cm

T2: Tumor localized within the pancreas, with a maximum size > cm

T3: Tumor advancing outside the pancreas, but with no invasion to the celiac axis or superior mesenteric artery

T4: Tumor invading the celiac axis or superior mesenteric artery

N –Regional lymph nodes

NX: Regional lymph node metastasis cannot be evaluated

N0: No regional lymph node metastasis

N1: Regional lymph node metastasis present

M –Distal metastasis

M0: No distal metastasis

M1: Distal metastasis present

Stage	T	N	M
**0**	Tis	N0	M0
**IA**	T1	N0	M0
**IB**	T2	N0	M0
**IIA**	T3	N0	M0

**IIB**	T1, T2, T3	N1	M0
**III**	T4	Any N	M0
**IV**	Any T	Any N	M1

3.5. Residual tumor (R) classification (UICC-TNM 7th Edition)

RX: Presence of residual tumor cannot be evaluated

R0: No residual tumor

R1: Presence of residual tumor by microscopic examination

R2: Presence of residual tumor by macroscopic examination

3.6. Definition of non-resectable NEC

Unoperated cases for which surgery as a curative process was not indicated based on clinical findings, including image-based diagnosis, or operated cases that underwent resection (including experimental laparotomy) but ultimately ended as R2 resection are considered non-resectable.

The details by organs are shown below. One or more criterion must be fulfilled for each organ.

<Esopghagus>

- Diagnose as Stage IV (UICC 7th edition) based on clinical findings.

<Stomach>

- Diagnose as Stage IV (UICC 7th edition) based on clinical or surgical findings. However, this does not include situations where Stage IV diagnosis is provided based only on peritoneal lavage cytology (CY1).
- Bulky lymph node* metastasis is identified.

  *Bulky lymph node: Two or more lymph nodes with a major axis of 1.5 cm or more, that are present in contact with each other around the celiac artery, the common hepatic artery, the splenic artery, and the proper hepatic artery, or on the front surface of the superior mesenteric vein, where single or multiple large and small lymph nodes have formed an aggregate with the overall major axis length of 3.0 cm or more.

<Duodenum (excluding the ampulla of Vater), small intestines, appendix, and colon>

- Diagnosed as Stage IV (UICC 7th edition) based on the clinical findings.

<Extrahepatic bile duct, ampulla of Vater, gallbladder>

- Distant metastasis is identified
- Para-aortic lymph node metastasis is identified
- Bulky metastasis identified in the hepatoduodenal mesentery and lymph nodes surrounding the head of the pancreas
- Invasion in the proper hepatic artery, common hepatic artery, celiac artery, or the superior mesenteric artery identified
- Invasion in the hepatic artery branches on both the left and right side identified
- Extensive invasion or occlusion of the main trunk of portal vein, or invasion in the portal vein branches on both the left and right side identified
- Invasion in the blood vessels on the lobe on one side of the liver (portal vein or artery) or atrophy of lobe on one side of liver identified, and advancement into the bile duct on the other side is identified up to the secondary branch level
- Advancement into the bile ducts on both sides identified up to the secondary branch level

<Pancreas>

- Diagnosed as Stage III or Stage IV (UICC 7th edition) based on clinical findings.

＜Hepatic NEC (hepatic primary lesion or liver metastasis from unknown primary lesion)>

- Distal metastasis* other than intrahepatic metastasis identified.

  *Limited to organs that cannot be the primary lesion site (bone, lymph nodes below the diaphragm,

peritoneum, subcutaneous, muscle, and spleen) (see 3.8)

- Para-aortic lymph node metastasis is identified
- Bulky metastasis identified in the hepatoduodenal mesentery and lymph nodes surrounding the head of the pancreas
- Multiple masses identified in the liver.
- Invasion in one of the proper hepatic artery, common hepatic artery, celiac artery, or the superior mesenteric artery identified.
- Invasion to hepatic artery branches on both the left and right side identified
- Extensive invasion or occlusion of the main trunk of portal vein, or invasion in portal vein branches on both the left and right side identified
- Invasion in blood vessels on lobe on one side of the liver (portal vein or artery) or atrophy of lobe on one side of liver identified, and advancement into the bile duct on the other side is identified up to the secondary branch level
- Advancement into the bile ducts on both sides identified up to the secondary branch level

3.7. Definition of recurrent NEC

If surgery (R0 resection or R1 resection) was performed for gastrointestinal/hepatobiliary or pancreatic primary lesion, malignant tumor and the condition was diagnosed as NEC based on pathological samples taken from surgery, and were deemed as a clinical relapse thereafter.

If there are past histological samples from the primary lesion, no histological biopsy of the relapse lesion is necessary.

3.8. Definition of hepatic NEC (hepatic primary lesion or liver metastasis from an unknown primary lesion)

After a detailed investigation into the primary lesion by cervical-pelvic contrast CT, gastrointestinal endoscopy, FDG-PET, otolaryngology (head and neck examination), urology examination (for men only) and gynecological examination (for women only), if the tumor is in one of the following sites, the case is defined as hepatic primary lesion (or primary lesion unknown).

- Tumor is present only in the liver
- Tumor is present in an organ that cannot be a primary lesion site (bone, lymph nodes below the diaphragm, peritoneum, subcutaneous, muscle, and spleen), and in the liver.

4. Patient selection criteria

Patients that fulfill all of the following inclusion criteria and do not correspond to any of the exclusion criteria are eligible for enrollment.

4.1. Inclusion criteria (for enrollment)

1) Any of the following is applicable based on pathological diagnosis taking findings of immunohistochemistry into consideration (see 3.1. to 3.3.).

[1] Pathologically diagnosed as neuroendocrine carcinoma (NEC*1) in the resected sample.

[2] Containing pathologically confirmed component of neuroendocrine carcinoma (NEC*1) in the biopsy sample.

1: Based on WHO 2010 classification

2) Any of the following is applicable

[1] NEC arise in esophagus, stomach, duodenum, intestine, appendix, colon, rectum, gallbladder, intrahepatic bile duct, extrahepatic bile duct, ampulla of Vater, pancreas,

[2] Liver NEC (primary liver or liver metastasis of unknown primary) *2.

*2: The tumor is only in one of the following sites after a thorough examination of the primary site by contrast CT (from the neck to pelvic) and upper/lower gastrointestinal endoscopy, FDG-PET scan, otolaryngology (head and neck) examination, urology examination (male patients only), and gynecology examination (female patients only).

a. Liver only

b. Bone, lymph nodes below the diaphragm, peritoneum, subcutaneous, muscle, spleen, and liver

3) Unresectable (see 3.6) or recurrent cancer (see 3.7). It is not essential for a pathological confirmation of the metastatic lesion or recurrent site. Cases of esophageal NEC is ineligible if corresponding to any of the following.

[1] cT4.

[2] No distant metastasis rather than supraclavicular lymph node

[3] Stenosis indicated for palliative radiotherapy

4) No previous chemotherapy or radiotherapy for NEC. Pre- or post-operative chemotherapy except irinotecan or etoposide for NEC is allowed as long as it was completed at least 8 weeks prior to registration.

5) No previous chemotherapy using platinum agents for any malignancies.

6) No serious tumor-related complications.

Serious tumor-related complications include superior vena cava syndrome, inferior vena cava syndrome, pleural, ascites, or pericardial effusions that are large or uncontrollable (e.g., effusions that accumulate quickly after drainage or even after adhesive surgery), and brain metastases with neurological symptoms.

7) Aged 20 to 75 years old.

8) ECOG performance status of 0 or 1.

9) Sufficient oral intake

10) Measurable region is not required.

11) Adequate organ functions.

[1] WBC ≥3,000/ mm^3

[2] Neutrophils ≥ 1,500/ mm^3

[3] Hemoglobin ≥ 9.0 g/dL

[4] Platelets ≥ 10×10^4/ mm^3

[5] Total bilirubin≤ 1.5 mg/dL※4

[6] AST(sGOT)≦100 IU/L※4（for hepatic NEC and liver metastasis, ≦150 IU/L）

[7] ALT(sGPT)≦100 IU/L※4（for hepatic NEC and liver metastasis, ≦150 IU/L）

※4：Presence or absence of biliary drainage is not relevant

[8] Serum creatinine≦ 1.3 mg/dL

[9] Creatinine clearance※5≥ 60 mL/min

※5： Creatinine clearance must have been estimated using the Cockcroft-Gault formula, and must be 60 mL/min or more. If the estimation is less than 60 mL/min, but the actual measurement is 60 mL/min or more, the patient can be deemed eligible.

Cockcroft-Gault formula

Male：Ccr ={(140－age) × body weight (kg)}/{72×serum creatinine (mg/dL)}

Female：Ccr = 0.85×{(140－age) × body weight (kg)}/{72×serum creatinine(mg/dL)}

12) Written informed consent.

4.2. Exclusion criteria

1) Synchronous or metachronous (within 5 years) malignancies except carcinoma in situ or intramucosal tumor curatively treated with local therapy.

2) Active infection requiring systemic therapy.

3) Fever of 38 degrees Celsius or higher.

4) Pregnant or lactating women, women of childbearing potential, or women within 28 days after delivery.

5) Psychiatric disease.

6) Patients requiring systemic steroids medication.

7) Interstitial pneumonia, pulmonary fibrosis.

8) Serious co-existing illness.

9) Unstable angina pectoris within 3 weeks, or with a history of myocardial infarction within 6 months.

10) Impossible to use both iodine and gadolinium due to being allergic to contrast agent.

11) Uncontrolled diabetes mellitus or routine administration of insulin.

5. Registration and randomization

5.1. Procedure of registration

Ensure that a patient to be registered meets all eligibility criteria and does not meet any of exclusion criteria and register the patient by using JCOG Web Entry System. JCOG Web System Personal Account and password are required for web registration. If unknown, contact JCOG Data Center.

Patient registration JCOG Web Entry System
URL:https://secure.jcog.jp/dc/
(Web registration can be used for 24 hours.)

Contact information for patient registration and JCOG Web Entry System
JCOG Data Center
TEL: 03-3542-3373
Weekdays 9:00-17:00 (not available in holidays, Saturdays and Sundays, New Year's holidays)
E-mail:JCOGdata@ml.jcog.jp

Contact information on Patients Selection Criteria
Study Coordinator Contact : Chigusa Morizane
Hepato-Biliary Pancreatic Group: Futomi Mori (Main Research Secretariat)
Department of Hepatobiliary and Pancreatic Medicine, National Cancer Center
TEL:03-3542-2511
FAX:03-3542-3815
E-mail:cmorizan@ncc.go.jp

Gastric Cancer Group: Yoshinori Machida
Department of Gastroenterology, Kanagawa Cancer Center (Gastrointestinal Tract)
TEL:045-520-2222
FAX:045-520-2202
E-mail:n-machida@kcch.jp

Esophageal Cancer Group: Ken Kato/Yushi Homma
Department of Head and Neck, Esphageal Medical Oncology, National Cancer Center
TEL: 03-3542-2511
FAX: 03-3542-3815
E-mail: kenkato@ncc.go.jp / yohonma@ncc.go.jp

5.1.1. Precautions for patient registration

① Registration after initiation of protocol treatment is unacceptable.
② Registration is performed by accessing the URL in '5.1. Procedure of registration'.
③ Eligibility checks are performed on the screen of Registration Form, so it is not necessary to send a Registration Form to Data Center by mail or fax.
④ If input data are insufficient, registration is not accepted until all are met.
⑤ The registration number is issued after the confirmation of eligibility on the registration screen, then the registration is completed.
⑥ Once registered, patients will not be retracted (retracted from the database) unless there is withdrawal of consent, including refusal to use the data for research. For duplicate registration, the information at the initial registration (registration number, allocated arm) are used in any case.
⑦ When misregistration or duplicate registration is found, contact Data Center immediately.
⑧ Body surface area and drug dose calculations are institutional responsibilities, and the body surface area and drug dose displayed on Web Entry System at registration are only for double-checking. Those should

always be calculated and checked at the institution. When the body surface area calculation formula adopted in the hospital information system of the institution differs from calculation formula adopted by JCOG (Dubois formula: Body surface area (m^2) = Body weight (kg) $^{0.425}$ x Height (cm) $^{0.725}$ 84 ÷ 10,000), there can be a difference in the dose by the hospital information system of the institution and the dose by the calculation formula adopted by JCOG, but in that case, either dosage used is decided by the site investigator.

5.2. Randomization and allocation adjustment factor

During enrollment, the treatment arm for a patient is allocated randomly by the data center.

Randomization would use a method of minimization using the 1) institutions, 2) Primary lesion organ (gastrointestinal tract [esophagus, stomach, duodenum, small intestines, appendix, colon, and rectum] vs. hepatobiliary and pancreatic organ [hepatic NEC, gallbladder, extrahepatic bile duct, ampulla of Vater, or pancreas]) as the adjustment factors, so that there are no large discrepancies between them. Researchers at participating facilities would not be informed of the detailed procedures of randomization.

6. Treatment Plan and Treatment Modification Criteria

Unless patient safety is threatened, treatment and treatment modifications is done in compliance with the specifications in this chapter.

If it is considered that the protocol specification may cause medically dangerous situation of the patient, treatment modifications should be made according to the medical judgment of the investigators/sub-investigators. Such protocol deviation is considered to be "clinically relevant deviation" if considered medically appropriate (see 14.1.4.Protocol deviation/violation). Deviations that occur with intentions other than safety, such as increasing efficacy, are not considered clinically relevant deviations.

6.1. Protocol treatment

Protocol treatment is initiated within 7 days of enrollment.

If treatment initiation occurs after 8 days from registration for any reason, the reason should be documented on the Treatment Course Form. If it is determined that treatment cannot be initiated, describe the details in the Off-treatment Form as Protocol Treatment Termination.

When laboratory parameters worsen and eligibility criteria are no longer met by the start of treatment after registration, the investigator/sub-investigator is allowed to decide whether initiate or terminate protocol treatment at their own discretion.

「6.3. The course initiation criteria is not applied at treatment changes.

Drugs used

- Etoposide ※
- Cisplatin ※
- Irinotecan ※
- Entecavir, tenofovir disoproxil fumarate, tenofovir alafenamide fumarate

The use of generic drugs is not restricted.

※ The company that manufactures or distributes these drugs, or intends to manufacture or sell these drugs, requires conflicts of interest control in the Clinical Trials Act as a company involved in this study (see 13.8.).

6.1.1. Arm A: Etoposide plus cisplatin (EP) therapy

The following chemotherapy courses will be given once a week for 3 weeks and treatment will be continued until the patient meets the discontinuation criteria.

Drug	Dosage(mg/m^2)	Dosing regimen/Dosing time	Dose day
Etoposide	100	IV/60-120 min	Day 1、2、3
Cisplatin	80	IV/60-120 min	Day 1

1) Calculation of the dosage

① Body surface area is calculated by determining drug dose to the third decimal point.

② For both etoposide and cisplatin, the calculated dose is determined by truncating the decimal point. For drug doses, ±10% is the acceptable range.

③ Dose recalculation due to body weight change after treatment initiation is not performed.

2) Administration of anticancer drugs

Etoposide and cisplatin are given in any order.

Examples of administration in the package insert are described below.

(i) Before administration: Before administration of anticancer drugs, 1,000-2,000 mL of hydration is done to achieve adequate diuresis.

(ii) Etoposide: Etoposide is mixed with infusion solutions such as isotonic sodium chloride solution of 250 mL or more, and infused intravenously in about 60-120 minutes. DEHP (2-ethylhexyl) phthalate: di-(2-ethylhexyl) phthalate), which is a plasticizer, elutes from polyvinyl chloride infusion sets and catheters. Avoid the use of polyvinyl chloride infusion sets and catheters.

(iii) Cisplatin administration: Cisplatin is mixed with stock solution or 500 mL of saline (or glucose-saline) and given intravenously in 60-120 min. During the administration, caution should be exercised in ensuring urine volume, and diuretics such as mannitol and furosemide should be administered as

necessary.

(iv) After administration: After administration of cisplatin, 1,000-2,000 mL of hydration should be done so that adequate diuresis can be achieved.

In addition, short hydration administration at the discretion of each institution is permitted. (see Table 6.1.1. for treatment cases).

Table 6.1.1. Example of short hydration

	Dose	Time
5HT3 antagonist<br>Dexamethasone<br>Physiological saline	9.9 mg<br>50 mL	15 min
Etoposide<br>Physiological saline	100 mg/m^2<br>250 mL	60 min
No. 1 solution<br>Potassium chloride<br>Magnesium sulfate	500 mL<br>10 mEq<br>8 mEq	60 min
20% mannitol	200 mL	30 min
Cisplatin<br>Physiological saline	80 mg/m^2<br>250 mL	60 min
No. 1 solution<br>Potassium chloride	500 mL<br>10 mEq	60 min

(Referred from Horinouchi H, et al., Japan Society of Clinical Oncology 2012

3) Precautions for treatment

JCOG9511, an upfront trial in small-cell lung cancer, suggested an association between treatment-related deaths and first-course neutropenia. Therefore, caution should be exercised when neutropenia is strongly observed from the first course.

6.1.2. Arm B: Irinotecan plus cisplatin (IP) therapy

One 4-week course of the following chemotherapy will be continued until the discontinuation criteria are met. However, if day 15 irinotecan is skipped, 3 weeks should be used as one course, and the next course should be started with day 22.

Drug	Dosage(mg/m^2)	Dosing regimen/Dosing time	Dose day
Irinotecan	60	IV/90 min	Day 1、8、15
Cisplatin	60	IV/60-120 min	Day 1

1) Calculation of the dosage

(i) Body surface area is determined by determining the dose of drug administered until the third decimal point.

(ii) The dose is calculated for both irinotecan and cisplatin by truncating the decimal point. For drug doses, ±10% is the acceptable range.

(iii) Dose recalculation due to body weight change after treatment initiation is not performed.

2) Administration of anticancer drugs

Irinotecan and cisplatin are given in any order.

Examples of administration in the package insert are described below.

(i) Before administration: Before administration of anticancer drugs, 1,000-2,000 mL of hydration is done to achieve adequate diuresis.

(ii) Irinotecan: Irinotecan is mixed with 500 mL or more of isotonic sodium chloride solution, glucose solution, or electrolyte maintenance solution and given by intravenous drip infusion at about 90 minutes.

(iii) Cisplatin administration: Cisplatin is mixed with stock solution or 500 mL of saline (or glucose-saline) and given intravenously in 60-120 min. During the administration, caution should be exercised in ensuring urine volume, and diuretics such as mannitol and furosemide should be administered as necessary.

(iv) After administration: After administration of cisplatin, 1,000-2,000 mL of hydration should be done so that adequate diuresis can be achieved.

In addition, short hydration administration at the discretion of each institution is permitted. (See Table 6.1.1.

3) Precautions for treatment

JCOG9511, an upfront trial in small-cell lung cancer, suggested an association between treatment-related deaths and first-course neutropenia. Therefore, caution should be exercised when neutropenia is strongly observed from the first course.

6.2. Protocol Treatment Termination/Completion Criteria

6.2.1. Definition of protocol treatment completion

Protocol treatment is continued in this study unless the protocol treatment discontinuation criteria are met, so no definition of protocol treatment completion is provided.

6.2.2. Criteria for termination of protocol treatment

Protocol treatment is terminated in any of the following cases:

1) Judged as protocol treatment is ineffective
   - When a definite tumor exacerbation is confirmed by imaging or clinically
     ※ Protocol treatment should not be discontinued if the clinical judgement of PD based on the assessment of response based on imaging indicates that continuation of protocol treatment is appropriate, and protocol treatment should be continued.
2) Protocol treatment cannot be continued due to adverse events
   ① If Grade 4 non-hematological toxicity is observed (with the exception of the following adverse events) 'hypernatremia', 'hyponatremia', 'hyperkalemia', 'hypokalemia', 'hyperglycemia', 'hypoglycemia', 'alkaline phosphatase increase', 'alanine aminotransferase increase', 'aspartate aminotransferase increase', 'blood bilirubin increase','serum amylase increase', 'lipase increase', 'GGT increase'.
   (*Adverse events other than "anaemia," "myeloid cytopenia," "lymphocyte count decreased," "neutrophil count decreased," "white blood cell count decreased," "platelet count decreased," "CD4 lymphopenia" in CTCAE v4 0)
   ② If the next course cannot be initiated beyond 21 days from the expected start date of the course due to an adverse event
   ③ When the criteria for terminating protocol treatment in the treatment modification criteria (6.3.) are met.
   ④ Adverse events other than the treatment modification criteria that the investigator/sub-investigator judges to require termination of protocol treatment
3) If the patient offers termination of protocol treatment for reasons not denied to be associated with the adverse event
   - This category should be used if an association with an adverse event cannot be ruled out.
4) When the patient offers termination of protocol treatment because of reasons for denial of association with adverse events
   - Patient refusal after enrollment and before initiation of protocol treatment
   - When the association with an adverse event can first be denied, such as the relocation of the person or household member during protocol treatment.
5) Death during protocol treatment
   - Death before deciding to terminate protocol treatment for other reasons
6) Palliative surgery for pathogenic adverse events or surgery for patients with complete response to chemotherapy (see Section 6.3.9).
7) In addition, exacerbations before the start of treatment after enrollment (protocol treatment could not be initiated due to rapid exacerbation), protocol violations were found, ineligibility was determined due to modifications in pathological diagnosis after enrollment, etc., treatment was changed, and it was judged that it was difficult to continue protocol treatment due to social reasons and safety management problems, etc.

The date of discontinuation of protocol treatment is defined as the date of death in 6.2.2.5), the date of surgery in 6.2.2.6), and otherwise, the date on which the treating physician judges that protocol treatment is discontinued.

In this study, non-NEC may be diagnosed by central pathological diagnosis during protocol treatment, because central pathological diagnosis is performed. If the patient is continuing on protocol treatment when the results of the central pathology diagnosis are reported to the registry, the attending physician and the institutional pathologist will review the results and take a clinically appropriate response. Protocol treatment is discontinued if discontinuation of protocol treatment is judged to be appropriate, and the reason for discontinuation is other.

6.3. Treatment modification criteria

The following terms shall be used for the treatment modification.

Delay: Delay the start of the course or administration of treatment from the planned date.

Termination: Discontinuation of a part of or all of the treatment without restarting.

Suspending: temporary interruptions or withdrawals that may be resumed if conditions are met

Skip: Do not administer one or more drugs and proceed to the next schedule.

Categories of infection (CTCAEv4.0) used in this study are as follows

Infection: CTCAEv4.0 infections and infestations

Bronchial infection; pulmonary infection; upper respiratory tract infection; mediastinal infection; pleural infection; catheter-related infection; Biliary tract infection; Gallbladder infection; urinary tract infection

6.3.1. Arm A (EP-therapy): Dose level

1) Etoposide

Drug	Dose level	Dosing schedule	Dose day
Etoposide	Level 0	100 mg/m^2 div	Day 1、2、3
	Level -1	80 mg/m^2 div	Day 1、2、3
	Level -2	60 mg/m^2 div	Day 1、2、3

2) Cisplatin

Drug	Dose level	Dosing schedule	Dose day
Cisplatin	Level 0	80 mg/m^2 div	Day 1
	Level -1	60 mg/m^2 div	Day 1
	Level -2	40 mg/m^2 div	Day 1

6.3.2. A arm (EP therapy): Course initiation criteria

- Initiate the course after confirming that all of the following "Course Initiation Criteria" are met on the scheduled start date of the course or the day before the plan start date of the course.
- If any one is not met, the initiation of the course is delayed.
- If the course cannot be initiated within 21 days of the expected start date of the course (if the start date of the previous course was day 1 and the course could not be initiated by day 42), discontinue the protocol treatment.
- The course initiation criteria is not applied at the start of the first course.

Course initiation criteria

① Neutrophil count Grade 0-1 (≧1500 per mm^3).

② Platelet count ≧10×10^4 /mm^3

③ AST≦100 IU/L (in the presence of hepatic metastases vs. hepatic NECs) was ≦200 IU/L).

④ ALT≦100 IU/L (≦200 IU/L for liver metastases vs liver NECs).

⑤ Total bilirubin <= 2.0 mg/dL

⑥ Serum creatinine <= 1.5 mg/dL

⑦ Fever Grade 0 (axillary temperature, no antipyretic)

⑧ Constipation, fatigue, phlebitis, oral mucositis, and infection ※1 are all Grade 2 or less.
Infected ※1: bronchial infection, pulmonary infection, upper respiratory tract infection, mediastinal infection, pleural infection, catheter-related infection, biliary tract infection, gallbladder infection, urinary tract infection

⑨ Anorexia, nausea, and emesis are all Grade 0-1.

⑩ Diarrhoeal Grade 0

6.3.3. Arm A (EP therapy): Dose reduction criteria

If any of the following toxicities are identified during the course, dose reduction should be performed in accordance with the dose reduction criteria (Table 6.3.3.) from the following course (no dose reduction in the course).

However, even if two or more items are met, the dose reduction for each drug is only one step. Re-escalation after dose reduction is not performed. Protocol treatment is terminated if the dose reduction criteria are met again after the dose reduction to Level-2.

Table 6.3.3. Arm A (EP therapy): Dose reduction criteria

Item	Etoposide	Cisplatin
Neutrophil count Grade 4 (<500 per mm^3).	Reduce the level by 1	No change
Platelet count Grade 4 (<$2.5 \times 10^4/mm^3$)	Reduce the level by 1	No change
1.5 <serum creatinine ≦2.0 mg/dL.	No change	Reduce the level by 1
Serum creatinine <= >2.0 mg/dL	Termination of protocol treatment	Termination of protocol treatment
Grade 3<br>Infected ※1	Reduce the level by 1	Reduce the level by 1
Grade 3<br>Assessment at onset of febrile.	Reduce the level by 1	Reduce the level by 1
Grade 2<br>Peripheral sensory neuropathy<br>Peripheral motor neuropathy,<br>Myalgia; arthralgia; tinnitus; hearing impairment	No change	Reduce the level by 1
Grade 3<br>Peripheral sensory neuropathy<br>Peripheral motor neuropathy,<br>Myalgia; arthralgia; tinnitus; hearing impairment	Termination of protocol treatment	Termination of protocol treatment
Non-hematologic toxicities of Grade 3 other than those listed above in ※2 that are causally related to EP-therapy (excluding hyponatremia, hypokalemia, hypomagnesemia, hypocalcemia, hyperglycemia, and weight loss)	Reduce the level by 1	Reduce the level by 1

※1:※1 of infection: bronchial infection, pulmonary infection, upper respiratory tract infection, mediastinal infection, pleural infection, catheter-related infection,
Biliary tract infection; Gallbladder infection; Urinary tract infection

※2:Causal relationship is judged as either of possible, probable, definite

6.3.4. Arm A (EP therapy): Within-course pause, skipping criteria

Following initiation of treatment with each course after the course initiation criteria are met, if any of the following adverse events are observed, day 2, day 3 etoposide will be suspended.

- Fever (axillary temperature) Grade 1-3
- Grade 3 of infection (bronchial infection, pulmonary infection, upper respiratory tract infection, mediastinal infection, pleural infection, catheter-related infection,
  Biliary tract infection, gallbladder infection, urinary tract infection)

Resting etoposide should be resumed after confirming that all initiation criteria are met until day 7. However, it should not be administered after day 8.

That is, if etoposide could not be administered by day 7, the remaining etoposide should be skipped.

If the above suspension and/or skip occur, the next course of etoposide should be started day 22 (after 3 weeks) counting from day 1 of the previous course if the ininiation criteria are met.

6.3.5. Arm B (IP therapy): Dose-level

1) Irinotecan

Drug	Dose level	Dosing schedule	Dose day
Irinotecan	Level 0	60 mg/m^2 div	Day 1、8、15
	Level -1	50 mg/m^2 div	Day 1、8、15
	Level -2	40 mg/m^2 div	Day 1、8、15

2) Cisplatin

Drug	Dose level	Dosing schedule	Dose day
Cisplatin	Level 0	60 mg/m^2 div	Day 1
	Level -1	50 mg/m^2 div	Day 1
	Level -2	40 mg/m^2 div	Day 1

6.3.6. Arm B (IP therapy): Course initiation criteria

- On the day of the initiation of the course or the day before the expected start of the course, start the course after confirming that all of the following "Course Initiation Criteria" are met.
- If any one is not met, the initiation of the course is delayed.
- If the course cannot be initiated within 21 days of the expected start date of the course (if the start date of the previous course was day 1 and the course could not be initiated by day 49), discontinue the protocol treatment.
- However, if day 15 irinotecan is skipped in the previous course, day 22 of the previous course is set as the scheduled start date of the next course regarded as one course per 3 weeks.
- The course initiation criteria is not applied at the start of the first course.

Course initiation criteria

① Neutrophil count Grade 0-1 (≧1500/mm^3).
② Platelet count ≧10×10^4 /mm^3
③ AST≦100 IU/L (≦200 IU/L for liver metastases versus liver NECs)).
④ ALT≦100 IU/L (≦200 IU/L for liver metastases vs liver NECs).
⑤ Total bilirubin <= 2.0 mg/dL
⑥ Serum creatinine <= 1.5 mg/dL
⑦ Fever Grade 0 (measured by axillary temperature, temperature <38°C without antipyretic use)
⑧ Constipation, fatigue, phlebitis, oral mucositis, and infection ※1 are all Grade 2 or less.
Infected ※1: bronchial infection, pulmonary infection, upper respiratory tract infection, mediastinal infection, pleural infection, catheter-related infection, biliary tract infection, gallbladder infection, urinary tract infection
⑨ Anorexia, nausea, and emesis are all Grade 0-1.
⑩ Diarrhoea Grade 0

6.3.7. Arm B (IP-therapy): Dosing criteria for day 8, day 15

After confirming that all of the following ①-③ are met, the second (day 8) or third (day 15) dose of irinotecan is administered. If day 8, day 15 dosing criteria are not met, skip day 8, day 15 dosing.

① All of the following are met with the most recent laboratory data on the scheduled day of administration or the day before the scheduled day of administration.
i) Neutrophil count Grade 0-2 (≧1000 per mm^3).
ii) Platelet count ≧10×10^4 /mm^3
iii) AST ≦ 100 IU/L (≦200 IU/L for liver metastases vs liver NECs).
iv) ALT ≦ 100 IU/L (≦200 IU/L in the presence of hepatic metastases versus hepatic NECs).
v) Total bilirubin <= 2.0 mg/dL
vi) Serum creatinine <= 2.0 mg/dL

② All of the following are met on the scheduled day of administration:
i) Fever Grade 0 (measured by axillary temperature, temperature <38°C without antipyretic use)

ii) Diarrhoeal Grade 0

③ Constipation, anorexia, nausea, emesis, fatigue, phlebitis, oral mucositis, and infection ※1 are all Grade 2 or less.

Infected ※1: bronchial infection, pulmonary infection, upper respiratory tract infection, mediastinal infection, pleural infection, catheter-related infection, biliary tract infection, gallbladder infection, urinary tract infection

6.3.8. Arm B (IP therapy): Dose reduction criteria

If any of the following toxicities are identified during the course, dose reduction should be performed in accordance with the dose reduction criteria (Table 6.3.8.) from the following course (no dose reduction in the course).

However, even if two or more items are met, the dose reduction for each drug is only one step. Re-escalation after dose reduction is not performed. Protocol treatment is terminated if the dose reduction criteria are met again after the dose reduction to Level-2.

Table 6.3.8. Arm B (IP therapy): Dose reduction criteria

Item	Irinotecan	Cisplatin
Neutrophil count Grade 4 (<500 per mm^3).	Reduce the level by 1	No change
Platelet count Grade 4 (< $2.5\times10^4/mm^3$)	Reduce the level by 1	No change
1.5 <serum creatinine ≦2.0 mg/dL.	No change	Reduce the level by 1
Serum creatinine <= >2.0 mg/dL	Termination of protocol treatment	Termination of protocol treatment
Grade 3<br>Infected ※1	Reduce the level by 1	Reduce the level by 1
Grade 3<br>Assessment at onset of febrile.	Reduce the level by 1	Reduce the level by 1
Grade2<br>Peripheral sensory neuropathy<br>Peripheral motor neuropathy,<br>Myalgia; arthralgia; tinnitus; hearing impairment	No change	Reduce the level by 1
Grade 3<br>Peripheral sensory neuropathy<br>Peripheral motor neuropathy,<br>Myalgia; arthralgia; tinnitus; hearing impairment	Termination of protocol treatment	Termination of protocol treatment
Non-hematologic toxicities of Grade 3 other than those listed above in ※2 that are causally related to IP-therapy (excluding hyponatremia, hypokalemia, hypomagnesemia, hypocalcemia, hyperglycemia, and weight loss)	Reduce the level by 1	Reduce the level by 1

※1:※1 of infection: bronchial infection, pulmonary infection, upper respiratory tract infection, mediastinal infection, pleural infection, catheter-related infection,

Biliary tract infection; Gallbladder infection; Urinary tract infection

※2:Causal relationship is judged as either of possible, probable, definite

6.3.9. Surgical of after end of chemotherapy

1) Surgery for Adverse Events Associated with Pathogenesis

Surgery may be performed if it is judged clinically desirable to perform surgery for newly appearing symptoms such as hemorrhage or stenosis. Protocol treatment is discontinued if surgery is performed, regardless of the content. In this case, the date of discontinuation of protocol treatment is the date of surgery.

2) Surgery for patients with complete response to chemotherapy

- When chemotherapy is highly effective, all metastases present at the time of enrollment on imaging studies disappear, and curative resection (R0 resection) is considered possible, resection including primary and metastatic disease may be performed.
- Surgical procedures are not specifically specified, but the Research Office will collect information on the details of the surgery performed individually in order to be reviewed by the research representative/research office.

- In all surgical cases, the group group conference shall confirm the validity of the judgment that surgery is indicated.
- When surgery is performed, protocol treatment is discontinued, regardless of whether curative resection was performed or not. In this case, the date of discontinuation of protocol treatment is the date of surgery.

6.3.10. Consultation on treatment modification

If there are any questions about treatment modification, contact "16.6. Study Coordinator".

Study Coordinator Contact : Chigusa Morizane

Hepato-Biliary Pancreatic Group: Futomi Mori (Main Research Secretariat)
Department of Hepatobiliary and Pancreatic Medicine, National Cancer Center
TEL:03-3542-2511
FAX:03-3542-3815
E-mail:cmorizan@ncc.go.jp

Gastric Cancer Group: Yoshinori Machida
Department of Gastroenterology, Kanagawa Cancer Center (Gastrointestinal Tract)
TEL:045-520-2222
FAX:045-520-2202
E-mail:n-machida@kcch.jp

Esophageal Cancer Group: Ken Kato/Yushi Homma
Department of Head and Neck, Esphageal Medical Oncology, National Cancer Center
TEL:03-3542-2511
FAX:03-3542-3815
E-mail: kenkato@ncc.go.jp / yohonma@ncc.go.jp

6.4. Concomitant treatment and supportive care

6.4.1. Required concomitant treatment/supportive care

1) Testing and Supportive Care for HBsAg-Positive Cases.

In HBsAg-positive cases, steroids and chemotherapy can lead to rapid hepatitis B virus (HBV) expansion (reactivation: reactivation) and potentially fatal severe hepatitis. Therefore, the following tests and supportive care are performed based on the "Guidelines for the Treatment of Hepatitis B, Third Edition (Japanese Society of Hepatology)." It is advisable to consult a hepatologist at the time prior to initiation of a nucleic acid analogue (entecavir, tenofovir disoproxil fumarate, and tenofovir alafenamide fumarate).

①Testing prior to initiation of chemotherapy: HBV-DNA quantitation

HBV-DNA quantitative analysis should be performed at least once prior to initiation of chemotherapy.

HBV-DNA assays are performed by real-time PCRs.

HBeAg and HBe antibodies should also be measured in accordance with the Guidelines for the Treatment of Hepatitis B, Third Edition (Japanese Society of Hepatology).

②Dosing schedule for supportive care (nucleic acid analogues prophylaxis)

- **Drugs used :**
  - **Entecavir (Bristol-Myers: Baraclude Tablets 0.5 mg)**
  - **Tenofovir disoproxil fumarate (GlaxoSmithKline: Tenozet Tablets 300 mg)**
  - **Tenofovir alafenamide fumarate (Giliado: Vemuridi Tablets 25 mg)**

The following dosage regimen should be followed, starting at least 1 week before the start of chemotherapy (as soon as possible), and continuing for at least 12 months after the end of chemotherapy. However, fulminant hepatitis has been reported in HBsAg-positive patients with high viral load, even during NA prophylaxis, and it is desirable to reduce the viral load before starting immunosuppression/chemotherapy. Nucleic acid analogues may be discontinued after 12 months after completion of chemotherapy if conditions *1 and 2 for discontinuation of NAs are met. However, if the administration of a nucleic acid analogue is discontinued, consultation with a hepatologist is always obtained, and the administration is discontinued only if the hepatologist deems it appropriate.

※1 Requirements for discontinuation of nucleic acid analogues (entecavir, tenofovir disoproxil fumarate, tenofovir alafenamide fumarate): all of the following

1. The patient has been on NA for more than 2 years.
2. HBV-DNA assays are not sensitive to detect
3. Be negative for HBeAg

※2 Patient background requirements: All of the following

1. Both the treating physician and the patient have sufficiently understood that the hepatitis exacerbation is frequently observed after the nucleic acid analog withdrawal, and that there is a danger of becoming serious in the time.
2. Follow-up is possible after treatment cessation, and appropriate treatment is possible even if hepatitis recurs
3. It is judged that the liver fibrillation is slight and the hepatic reserve is good, and it is difficult to become serious even if the hepatitis is exacerbated.

(Adapted from Guidelines for the Treatment of Hepatitis B, the 3rd edition (Japanese Society of Hepatology))

Entecavir

- **Dosage regimen: Take this medicine on an empty stomach (2 hours after meals and more than 2 hours before the next meal).**
- **Dosage :**

Creatinine clearance (mL/min).	Dosage
50 or more	0.5 mg once daily
30 Beyond 50	0.5 mg once every 2 days
10 Beyond 30	0.5 mg once every 3 days
10 Less than	0.5 mg once every 7 days

- **Adverse drug reactions (incidence of all grades): nucleoside analog-naïve patients**

Diarrhea (6.0%), nausea (4.5%), constipation (3.7%), upper abdominal pain (3.0%), malaise (1.5%), nasopharyngitis (3.0%), muscle stiffness (2.2%), headache (3.0%), rash (incidence unknown), laboratory tests: elevated AST (3.7%), increased blood bilirubin (6.0%), increased blood amylase (10.4%), and increased lipase

(10.4%).Blood glucose increased (6.0%), blood lactate increased (6.7%), urine occult blood positive (4.5%), white blood cell count decreased (8.2%), and eosinophil count increased (0.7%). [Significant adverse reactions (incidence unknown)] Hepatitis worsened after completion of treatment, anaphylactoid symptoms, lactic acidosis, and severe hepatomegaly due to fatty liver

Tenofovir disoproxil fumarate

- **Dosage and administration: 300 mg is orally administered once daily.**
- **Dosage :**

Creatinine clearance (mL/min).	Dosage
50 or more	300 mg once daily
30 Beyond 50	300 mg once every 2 days
10 Beyond 30	300 mg once every 3 to 4 days
Hemodialysis	[注)] of 300 mg once every 7 days<br>Or 300 mg after completion of cumulative approximately 12 hours of dialysis<br>NOTE) After hemodialysis was performed. The pharmacokinetics in patients with creatinine clearance < 10 mL/min and not on hemodialysis have not been investigated.

- **Dosing Precautions:**

In the long-term administration of tenofovir disoproxil fumarate, attention should be paid to renal dysfunction, hypophosphatemia (including Fanconi syndrome), and decrease in bone mineral density. It is recommended that renal function and serum phosphorus should be measured regularly during tenofovir disoproxil fumarate administration.

- **Adverse reactions (incidence of all grades):**

Abnormal liver function tests (AST, ALT and γ-GTP increased, etc.) in 7 patients (4.9%), increased creatinine in 4 patients (2.8%), increased amylase, increased lipase and nausea in 3 patients each (2.1%), abdominal pain in 2 patients (1.4%), [major adverse reactions (incidence unknown)] renal dysfunction, renal failure, acute renal failure, proximal renal tubular dysfunction, Fanconi syndrome, severe renal dysfunction such as acute renal tubular necrosis, nephrogenic diabetes insipidus or nephritis, severe hepatomegaly due to lactic acidosis and fatty deposition (steatohepatitis), pancreatitis

Tenofovir alafenamide fumarate

- **Dosage and administration: 25 mg is orally administered once daily.**
- **Dosage :**

Creatinine clearance (mL/min).	Dosage
15 or more	25 mg once daily
Less than 15	Consider discontinuation

- **Dosing Precautions:**

In the long-term administration of tenofovir alafenamide fumarate, attention should be paid to renal dysfunction, hypophosphatemia (including Fanconi syndrome), and decrease in bone density. It is recommended that renal function and serum phosphorus should be measured periodically during tenofovir alafenamide fumarate administration.

- **Adverse reactions (incidence of all grades):**

Nausea and abdominal distension, headache, fatigue (≧1%), dyspepsia and diarrhea, flatus, upper abdominal pain, constipation, ALT increased, arthralgia, dizziness, insomnia, pruritus, rash (≧0.5% to <1%), [significant adverse reactions (incidence unknown)] renal dysfunction, renal failure, acute renal failure, proximal renal tubular dysfunction, severe renal impairment such as Fanconi syndrome, acute renal tubular necrosis, renal diabetes insipidus or nephritis, severe hepatomegaly due to lactic acidosis and fatty deposits (fatty liver)

③Monitoring: Quantitative analysis of HBV-DNA (during and after administration of nucleic acid analogues)

During nucleic acid analogue administration:

They are monitored every 4 weeks by both HBV-DNA quantitative analysis and liver function (ASTs, ALTs). However, if HBV-DNA level is less than 20 IU/mL (1.3 log IU/mL) during administration of nucleic acid analogues, it is acceptable to perform tests every 4 to 12 weeks.

After discontinuation of nucleic acid analogue administration:

Bearing in mind that reactivation may occur even after discontinuation of administration of a nucleic acid analogues, the patient should be consulted with a hepatologist, and the patient should be monitored for HBV-DNA determination and hepatic function (AST/ALT) every 4 weeks for at least 1 year after discontinuation of administration of a nucleic acid analogues. Subsequent follow-up will be decided after consulting a hepatologist.

2) Laboratory Teste and Supportive Care for HBsAg-Negative and HBc Antibody-Positive and/or HBs Antibody-Positive Cases.

HBV-DNA quantitative analysis should be performed at least once prior to initiation of chemotherapy. HBV-DNA assays are performed by real-time PCRs.

i) HBV-DNA ≧ 20 IU/mL (1. 3 log IU/mL) prior to initiation of chemotherapy

It has been clarified that HBV-DNA replicates persist at low levels in the livers and peripheral blood mononuclear cells when HBc or HBs are positive, even if they are HBs-Ag negative. It has been reported that reactivation of HBV and development of severe hepatitis are caused by the use of potent immunosuppressive agents even in such patients with previous infections.

If HBV-DNA ≧ 20 IU/mL (1.3 log IU/mL), the risk of HBV reactivation is judged to be as high as in HBsAg-positive cases, and prophylactic administration of nucleic acid analogues (entecavir or tenofovir disoproxil fumarate, tenofovir alafenamide fumarate) is administered. The following laboratory tests and supportive care are performed in accordance with the "Guideline for the Treatment of Hepatitis B, 3rd edition (Japanese Society of Hepatology)" with reference to the following for examination, dosage, and monitoring of supportive care before the start of chemotherapy.

However, these are not applicable if the HBs antibody alone is positive and the HBV vaccination history is obvious.

①Dosing schedule for supportive care (nucleic acid analogues prophylaxis)

According to the dosage and administration of nucleic acid analogues (entecavir, tenofovir disoproxil fumarate, tenofovir alafenamide fumarate) in "1) Test and supportive care for HBsAg positive cases". Same conditions for NA discontinuation.

② Monitoring: Quantitative analysis of HBV-DNA (during and after administration of nucleic acid analogues)

The intervals for monitoring during and after discontinuation of NA are in accordance with the provision of "1) Testing and supportive care for HBsAg-positive patients".

ii) HBV-DNA less than 20 IU/mL (1.3 log IU/mL) prior to initiation of chemotherapy

HBV-DNA quantitative analysis and hepatic function (AST, ALT) will be monitored, and nucleic acid analogues (entecavir, tenofovir disoproxil fumarate, tenofovir alafenamide fumarate) will be started when ≧20 IU/mL (1.3 log IU/mL) is achieved.

The Guidelines for the Treatment of Hepatitis B, the 3rd edition (Japanese Society of Hepatology) recommends monitoring with HBV-DNA quantitative analysis or high-sensitivity HBs antibodies during and after chemotherapy, depending on the risks of revitalization.

①Monitor:HBV-DNA quantitative analysis

HBV-DNA quantitative analysis should be performed every 4-12 weeks from the start of chemotherapy until at least 12 months after the end of chemotherapy.

If HBV-DNA level is more than 20 IU/mL (1.3 log IU/mL), administration of nucleic acid analogues should be started immediately in accordance with the Guidelines for the Treatment of Hepatitis B, the 3rd edition (Japanese Society of Hepatology). If HBsAg monitoring is positive for < 1 IU/mL (low positive), nucleic acid analogues should be administered after additional HBV DNA determinations of ≧ 20 IU/mL (1.3 log IU/mL).

It is advisable to consult a hepatologist at a time prior to initiation of NAs.

②Supportive care in reactivation

Nucleic acid analogues should be administered according to the supportive care described in i) When HBV-DNA prior to the initiation of chemotherapy is 20 IU/mL (1.3 log IU/mL) or more in 6.4.1.2). Once administration of nucleic acid analogues is started, nucleic acid analogues should be discontinued only if appropriate by the hepatologist.

6.4.2. Recommended/not recommended concomitant treatment/supportive care

The following concomitant treatment and supportive care are recommended. Even if it is not carried out, it is not regarded as protocol deviation,

1) Addressing Febrile Neutropenia.

① Assessment at onset of febrile neutropenia (FN).

a) If the count is less than 500 per mm^3, or is less than 1000 per mm^3 and is expected to be less than 500 per mm^3 within 48 hours, and if the axillary temperature is 37.5°C or higher (mouth temperature is 38.0°C or higher), a severity-risk assessment should be performed promptly and anti-virus treatment initiated as appropriate.

b) Severity risk assessment is performed with reference to Multinational Association for Supportive Care in Cancer(MASCC) scoring system ※1.

c) For initial evaluation, complete blood count including differential WHITE BLOOD CELL and platelet count, renal function (BUN, creatinine), electrolytes, liver function (transaminases, total bilirubin, alkaline phosphatase) tests, ≧2 sets of venous blood cultures prior to initiation of antimicrobials, one set of cultures from the catheter lumen and one set from the peripheral vein if a central venous catheter is in place, cultures of suspected infection areas, and chest x-rays if respiratory symptoms and signs are present.

d) When febrile neutropenia (FN) develops in a patient with a central venous catheter, blood cultures from the catheter and peripheral blood are performed, and catheter-related infections are considered if there is a time difference of more than 120 minutes in the positivity of both. If appropriate antimicrobial therapy does not improve after more than 72 hours, catheter should be removed. For infections caused by Staphylococcus aureus, Pseudomonas aeruginosa, Bacillus, fungi, and acid-fast bacilli, the catheter should be removed and appropriate antimicrobial therapy based on culture results should be performed.

② Antibiotic use

a) In high-risk patients, β-lactams with anti-Pseudomonas aeruginosa activity are administered intravenously as a single agent. However, other antimicrobials (aminoglycosides, fluoroquinolones, and/or vancomycin) may be added to a single agent in the initial regimen in patients with unstable or complicated conditions or when drug-resistant organisms are strongly suspected. Low-risk patients may be treated with antibiotics orally or intravenously, hospitalized, or with adequate evaluation, if appropriate, as outpatients.

b) Re-evaluation will be performed 3-4 days after initiation of antibiotics to investigate the continuation or change of antibiotics. In principle, antibiotics will be continued until the neutrophil count recovers to 500 cells per mm^3 or more.

c) Empiric antifungal therapy is recommended in high-risk patients who do not respond to 4-7 days of broad-spectrum antibiotics.

d) Fluoroquinolone prophylaxis is recommended in high-risk individuals with an expected neutrophil count of 100 cells per mm^3 or less lasting >7 days.

③ Therapeutic administration of G-CSF

For therapeutic administration of G-CSF during the incidence of FNs, refer to "6.4.5. 3) Therapeutic administration of G-CSF".

※1 Multinational Association for Supportive Care in Cancer (MASCC) scoring system.
(Adapted in part from the Practice Guideline for Febrile Neutropenia (FN) [Japanese Society of Medical Oncology]. *2)

Item	Score
Clinical manifestations (select one of the following * mark 3 sections)<br>*No symptoms<br>*Mild symptoms<br>*Moderate symptoms	 5<br>5<br>3
No decrease in blood pressure	5
No chronic obstructive pulmonary disease	4
Solid tumors, or hematopoietic tumors without a history of fungal infection	4
No dehydration symptoms	3
Patients with fever during outpatient management	3
Age < 60	2

The total score is up to 26 points. Twenty-one points or more are considered low risk and 20 points or less are considered high risk

※2 Since patients aged 20 years or older are subjects in this study, we deleted "Not applicable to patients younger than 16 years old" from the original edition of the Practice Guideline for Febrile Neutropenia (FN) [Japanese Society of Medical Oncology].

2) Nausea and vomiting

Regarding nausea and vomiting, antiemetics are positively administered according to Clinical Practice Guidelines for Antiemesis in Oncology [48], and fluid and electrolyte repletion are performed when oral intake is severely reduced.

3) Anorexia

If oral intake drops markedly, fluid and electrolyte supplements should be given as needed. Especially, in the cases with diabetes mellitus, the abnormality of blood sugar level and electrolyte is noticed.

4) Anemia, thrombocytopenia

If anaemia (haemoglobin < 8.0 g/dL) or thrombocytopenia (platelet count < $2\times10^4/mm^3$) is observed, blood should be transfused as appropriate at the discretion of the treating physician.

5) Diarrhea

Severe diarrhoea occasionally occurs with arm B (IP therapy arm) and is extremely dangerous when complicated by febrile neutropenia. Patients should be fully informed about toxicities and their management, and should be instructed to measure body temperature, especially when neutrophils counts are most decreasing, and to contact a physician or nurse immediately during fever and diarrhea. Nonsteroidal anti-inflammatory drugs may not cause fever to become overt, so unnecessary anti-inflammatory drugs are not given.

If irinotecan-induced diarrhea occurs, the following supportive measures are recommended:

High-dose loperamide hydrochloride therapy

① Loperamide hydrochloride was started after signs of diarrhea were observed.
② Initial dose of 4 mg followed by 2 mg/2 hours (4 mg/4 hours at night)
③ It is administered until watery stool does not appear for more than 12 hours.
④ Doses should not be given for more than 48 hours.

6) Precautions on the day of cisplatin administration

Aminoglycoside antibiotics, vancomycin, and nonsteroidal anti-inflammatory drugs are not administered on the day of cisplatin administration or are used with caution.

6.4.3. Acceptable concomitant treatment and supportive care

The following concomitant treatment and supportive care may be used as needed.

Concomitant use of drugs for the treatment of complications such as hypertension and diabetes mellitus and symptomatic drugs such as morphine may be performed, but this drug should be administered with caution when furosemide, piretanide and phenytoin are used. Oral antibiotics for febrile neutropenia prophylaxis may be given at the discretion of the investigator/sub-investigator. Bisphosphonate denosumab may be used in combination for bone metastases.

6.4.4. Unacceptable concomitant treatment and supportive care

None of the following treatments will be given during protocol treatment:

① Administration of anticancer drugs other than protocol treatment

② Radiation therapy

6.4.5. Granulocyte colony-stimulating factor (granulocyte-colony stimulating factor:G-CSF

※This study permits the use of G-CSF biogenerics (biosimilars).

1)※ of primary prophylaxis with G-CSF

※Primary prophylaxis: G-CSF administration before developing febrile neutropenia or prolonged neutropenia to prevent them during anticancer therapy.

Primary prophylaxis with G-CSF was not recommended at the beginning of the study in this study. However, since febrile neutropenia in group A was 21.4% in the late 2016 periodic monitoring report and 15.8% in the early 2017 periodic monitoring report (18.4% when the time of occurrence was April 2017 and SAE reports not reflected in the early 2017 periodic monitoring report were included), it was decided that the patient would meet at least the recommended grade B or higher (almost A) according to G-CSF Appropriate Use Guideline 2013 and JSMO Febrile Neutropenia (FN) Practice Guideline. Primary prophylactic administration of G-CSF was recommended in group A. However, it is not considered a protocol deviation even if it is not administered. Since it is practical to administer pegfilgrastim from the viewpoint of convenience, when the primary preventive administration of G-CSF is carried out in the actual medical field, the administration example of pegfilgrastim is shown below.

(Administration cases)

Group A: 3.6 mg of pegfilgrastim (genetical recombination) will be injected subcutaneously (once per chemotherapy course) between day 4(day 3's completion of etoposide treatment and day 7 at least 24 hours after the completion of etoposide treatment.

Since the safety of pegfilgrastim administered 14 days prior to the initiation of cancer chemotherapy and within 24 hours after completion of administration has not been established, the primary prophylactic administration of pegfilgrastim is not performed in group B where day 8 is administered the drug.

Table 6.4.5. Primary prophylactic administration of G-CSF should be performed according to the approved dosage and administration shown in the table below.

Drug	･･ Pegfilgrastim (arm A only)<br>･･ Filgrastim<br>･･ Naltograstim<br>･･ Lenograstim
Time of initiation	Twenty-four hours after completion of chemotherapy
Dosage Dosing regimen	・Pegfilgrastim (genetical recombination) at a dose of 3.6 mg subcutaneously once per chemotherapy course (group A only)<br>・Filgrastim: 50 µg per m^2 SC once daily or 100 µg per m^2 IV once daily<br>・Naltograstim: 1 µg/kg SC once daily or 2µg/kg IV once daily<br>・Lenograstim: 2 µg/kg SC once daily or 5µg/kg IV once daily
Timing of discontinuation (other than pegfilgrastim)	・If the neutrophil count reaches 5000 per mm^3 or more after the course, administration should be discontinued.<br>・If the neutrophil count recovers to ≧2000 cells per mm^3, there are no suspected infections, and the patient's safety is judged to be ensured by the responsiveness to the drug used, the drug should be discontinued or reduced.

2)Secondary prophylactic※ of G-CSF

※ Secondary prophylaxis: G-CSF prophylactic administration after once occurrence of febrile neutropenia or prolonged neutropenia to prevent febrile neutropenia or prolonged neutropenia from occurring again during anticancer therapy.

If febrile neutropenia occurs in the previous course, secondary prophylaxis with G-CSF after the subsequent course is recommended, even if dose reduction or schedule modification or antimicrobial therapy is considered to be associated with a lower risk of febrile neutropenia (group A). However, it is not considered a protocol deviation even if it is not administered. As it is practical to administer pegfilgrastim in terms of convenience when secondary prophylactic administration of G-CSF is carried out in real medical practice, the following examples of administration of pegfilgrastim are shown.

(Administration cases)

Group A: 3.6 mg of pegfilgrastim (genetical recombination) is injected subcutaneously (once per course of chemotherapy) between the time of completion of etoposide treatment of day 4(day 3 and day 7 of at least 24 hours.

However, the safety of pegfilgrastim administered 14 days prior to the start of cancer chemotherapy and 24 hours after the end of treatment has not been established, so secondary prophylactic pegfilgrastim administration is not performed in group B, where day 8 is given the drug.

Secondary prophylactic administration of G-CSF should be performed according to the approved dosage and administration shown in the table below.

Drug	・・ Pegfilgrastim (arm A only)<br>・・ Filgrastim<br>・・ Naltograstim<br>・・ Lenograstim
Time of initiation	・・ Pegfilgrastim (arm A only)<br>Twenty-four hours after completion of chemotherapy<br>・・ Filgrastim, nartograstim, and lenograstim<br>When neutrophil counts <1000 per mm^3 are observed
Dosage Dosing regimen	・Pegfilgrastim (genetical recombination) at a dose of 3.6 mg subcutaneously once per chemotherapy course (group A only)<br>・Filgrastim: 50 μg per m^2 SC once daily or 100 μg per m^2 IV once daily<br>・Naltograstim: 1 μg/kg SC once daily or 2μg/kg IV once daily<br>・Lenograstim: 2 μg/kg SC once daily or 5μg/kg IV once daily
Timing of discontinuation (other than pegfilgrastim)	・If the neutrophil count reaches 5000 per mm^3 or more after the course, administration should be discontinued.<br>・If the neutrophil count recovers to ≧2000 cells per mm^3, there are no suspected infections, and the patient's safety is judged to be ensured by the responsiveness to the drug used, the drug should be discontinued or reduced.

3) Therapeutic administration of G-CSF

Therapeutic administration of G-CSF should be performed according to the approved dosage and administration shown in the table below.

Time of initiation	・When the neutrophil count is less than 1000 per mm^3 and fever (in principle, > 38.0°C) is observed<br>・When neutrophil counts <500 per mm^3 are observed
Dosage Dosing regimen	・Filgrastim: 50 μg per m^2 SC once daily or 100 μg per m^2 IV once daily<br>・Naltograstim: 1 μg/kg SC once daily or 2μg/kg IV once daily<br>・Lenograstim: 2 μg/kg SC once daily or 5μg/kg IV once daily
Timing of discontinuation	・If the neutrophil count reaches 5000 per mm^3 or more after the course, administration should be discontinued.<br>・If the neutrophil count recovers to ≧2000 cells per mm^3, there are no suspected infections, and the patient's safety is judged to be ensured by the responsiveness to the drug used, the drug should be discontinued or reduced.

6.5. Post-study treatment

Treatment after discontinuation of protocol treatment and treatment after progression or recurrence after completion are not specified.

Treatment (cross over) with drugs included in treatment regimens in the unassigned groups may be used, but if the total dose of cisplatin is greater than 500 mg/m^2, careful attention should be given to accumulating toxicities such as peripheral sensory/motor neuropathy, hearing loss, and renal impairment. Patients should be carefully monitored with adequate risk explanation only if the benefits are apparently outweighed by the risks, such as those with persistent sensitivity to cisplatin and mild cumulative toxicity.

If primary analysis or interim analysis reveals the primary conclusions of the trial, the results of the study will be explained to patients enrolled in this study as needed, and the best treatment will be provided, taking into account the course of treatment of individual patients.

In addition, if the protocol treatment discontinuation criteria apply but clinically "protocol treatment continuation" is judged to be appropriate, consult the research office through the institutional research director or institutional coordinator rather than at the physician level as a general rule (except when time is not allowed). In agreement between the Research Secretariat and the Investigator/Institution Coordinator, decide whether to treat as a → after discontinuation of protocol treatment or to deviate and continue protocol treatment. The details of the consultation

with the Research Secretariat and the decision-making process should be provided in the comment column for the patient's end-of-treatment report and progress record. If continuing the protocol treatment with protocol deviation occurs frequently, the Study Coordinator should consider revising protocol treatment termination criteria using group meetings and group mailing lists, because it is considered that the protocol treatment termination criteria is clinically inappropriate in such situation.

7. Anticipated Adverse Events

7.1. Anticipated adverse reactions

Anticipated adverse reactions in this study are as follows:

7.1.1. Anticipated Adverse Drug Reactions with Drugs

Adverse drug reactions anticipated with protocol treatments and drugs used in protocol-specified tests are referred to the latest version of the drug package insert.

7.1.2. Anticipated adverse reactions in the standard treatment arm (arm A)

Table 7.1.2. Adverse events in the EP-therapy group at JCOG9511 (excerpt from the final analysis report)

Examination Items	Grade 0	Grade 1	Grade 2	Grade 3	Grade 4	Grade 3,4(%)	Grade4(%)	Total
White blood cells	2	5	30	35	5	51.9%	6.5%	77
Neutrophils	1	0	5	21	50	92.2%	64.9%	77
Hemoglobin	2	9	43	23	-	29.9%	-	77
Platelet	31	19	13	14	0	18.2%	0%	77
Total bilirubin	57	-	20	0	0	0%	0%	77
GOT	49	24	2	1	1	2.6%	1.3%	77
GPT	40	28	6	2	1	3.9%	1.3%	77
Creatinine	56	16	5	0	0	0%	0%	77
Oxygen tension	13	27	9	2	1	5.8%	1.9%	52
Nausea and vomiting	13	36	23	5	-	6.5%	-	77
Diarrhea	64	8	5	0	0	0%	0%	77
Oral cavity (stomatitis)	68	6	2	1	0	1.3%	0%	77
Infection	42	23	9	1	2	3.9%	2.6%	77
Hair loss (hair)	9	46	19	-	-	-	-	74
Fever (uninfected)	45	14	16	2	0	2.6%	0%	77
Perception (peripheral nerve)	66	10	1	0	-	0%	-	77
Rash	74	2	1	0	0	0%	0%	77

※: Use JCOG Toxicity Criteria.

7.1.3. Anticipated adverse reactions in the study treatment arm (Arm B)

Table 7.1.3. Adverse Events in the IP-Therapy Group in a JCOG9511 (Extracted from the Final Analysis Report)

Examination Items	Grade 0	Grade 1	Grade 2	Grade 3	Grade 4	Grade 3,4(%)	Grade 4(%)	Total
White blood cells	1	16	38	17	3	26.7%	4.0%	75
Neutrophils	1	8	17	30	19	65.3%	25.3%	75
Hemoglobin	7	10	38	20	-	26.7%	-	75
Platelet	56	6	9	1	3	5.3%	4.0%	75
Total bilirubin	59	-	16	0	0	0%	0%	75
GOT	40	30	5	0	0	0%	0%	75
GPT	35	30	7	3	0	4.0%	0%	75
Creatinine	56	15	4	0	0	0%	0%	75
Oxygen tension	13	20	5	1	1	5.0%	2.5	40
Nausea and vomiting	11	26	28	10	-	13.3%	-	75
Diarrhea	23	19	21	8	4	16.0%	5.3%	75
Oral cavity (stomatitis)	66	9	0	0	0	0%	0%	75

Infection	45	17	9	3	1	5.3%	1.3%	75
Hair loss (hair)	17	44	13	-	-	-	-	74
Fever (uninfected)	45	12	17	1	0	1.3%	0%	75
Perception (peripheral nerve)	71	4	0	0	-	0%	-	75
Rash	70	5	0	0	0	0%	0%	75

※: Use JCOG Toxicity Criteria.

Table 7.1.3. Adverse events in the IP-therapy group in b JCOG0509 (abstracted from the main analysis report)

Examination Items	Grade 0	Grade 1	Grade 2	Grade 3	Grade 4	Grade 3,4(%)	Grade 4(%)	Total
White blood cells	16	36	58	29	3	22.5	2.1	142
Neutrophils	6	10	43	51	32	58.5	22.5	142
Hemoglobin	20	24	65	24	9	23.2	6.3	142
Platelet	125	6	8	2	1	2.1	0.7	142
Total bilirubin	116	15	10	1	0	0.7	0	142
GOT	98	37	5	2	0	1.4	0	142
GPT	80	56	4	2	0	1.4	0	142
Creatinine	97	37	8	0	0	0	0	142
Anorexia	22	67	33	19	1	14.1	0.7	142
Nausea	30	67	36	9	0	6.3	0	142
Vomiting	89	38	10	5	0	3.5	0	142
Diarrhea	52	51	28	11	0	7.7	0	142
Mucositis (examination findings)-oral cavity	126	15	0	0	1	0.7	0.7	142
Hair loss	64	66	12	-	-	-	-	142
Onset of febrile neutropenia	127	-	-	14	1	10.6	0.7	142
Infection with Grade3-4 neutropenia<br>-Bronchus	142	-	0	0	0	0	0	142
-Lung (pneumonia)	137	-	0	4	1	3.5	0.7	142
-Pharynx	142	-	0	0	0	0	0	142
-Upper respiratory tract-unclassifiable	141	-	1	0	0	0	0	142
-Bladder	142	-	0	0	0	0	0	142
-Kidney	142	-	0	0	0	0	0	142
-Urinary tract-subclassification impossible	142	-	0	0	0	0	0	142
Neuropathy: Sensory	127	13	2	0	0	0	0	142

Table 7.1.3.c Adverse events from a pilot trial of postoperative adjuvant chemotherapy with irinotecan plus cisplatin for high-grade neuroendocrine lung cancer (excerpt)

Examination Items	Grade 2	Grade 3	Grade 4	Grade 3,4(%)	Grade 4(%)	Total
White blood cells	17	7	0	17.5%	0%	40
Neutrophils	12	15	4	47.5%	10.0%	40
Hemoglobin	14	6	4	25.0%	10.0%	40
Platelet	2	0	0	0%	0%	40
Total bilirubin	0	0	0	0%	0%	40
GOT	0	0	0	0%	0%	40
GPT	1	0	0	0%	0%	40
Creatinine	0	0	0	0%	0%	40
Hyponatremia	0	5	0	12.5%	0%	40
Endotoxemia	3	1	0	2.5%	0%	40
Hypokalemia	0	4	0	10.0%	0%	40
Nausea	8	4	-	10.0%	-	40
Vomiting	4	2	0	5.0%	0%	40
Anorexia	2	0	-	0%	0%	40

Diarrhea	11	2	0	5.0%	0%	40
Fatigue	10	5	-	12.5%	0%	40
Infection	2	0	0	0%	0%	40

7.2. Anticipated Adverse Events Due to Pathogenesis

1) Esophageal primary

Esophageal bleeding; Esophageal pain; Esophageal stenosis; Esophageal obstruction; Esophageal perforation; Esophageal ulcer; Esophageal fistula; Hoarseness; Pharyngolaryngeal dysesthesia; Hypercalcemia of advanced disease; Tracheal stenosis; Tracheal obstruction; Esophageal anastomotic leakage; Recurrent laryngeal nerve palsy

2) Gastric primary

Gastric bleeding, upper gastrointestinal bleeding, gastric pain, gastrointestinal pain, back pain, nausea, vomiting, dyspepsia, gastroparesis, abdominal fullness, gastric stenosis, gastric obstruction, gastric perforation, duodenal bleeding, duodenal fistula, duodenal perforation, duodenal perforation, duodenal stenosis, gastrostomy, gastrointestinal fistula, gastrointestinal fistula, ileus, gastric anastomotic leak, gastrointestinal anastomotic leak

3) Small intestin/Colon and rectum

Bleeding from tumor; Ileus; Duodenal bleeding; Duodenal obstruction; Duodenal perforation; Duodenal stenosis; Small bowel obstruction; Small bowel stenosis; Small bowel ulcer; Small bowel perforation; Small bowel fistula; Vaginal fistula; Colonic fistula; Colonic stenosis; Colonic obstruction; Colonic perforation; Rectal stenosis; Rectal obstruction; Rectal fistula; Rectal perforation; Rectal fistula; Jejunal perforation; Jejunal perforation; Ileostomy; Ileovesical fistula; Intestinal fistula; Anal pain; Small bowel anastomotic leakage; Large bowel anastomotic leakage; Rectal anastomotic leakage; Pelvic infection

4) Pancreas, biliary tract, and liver

Body weight loss; Fever; Pancreatitis; Cholecystitis; Cholecystic obstruction; Gallbladder pain; Liver failure; Biliary tract infection; Hemorrhage from biliary tract; Duodenal hemorrhage; Duodenal fistula; Duodenal obstruction; Duodenal perforation; Duodenal stricture; Anastomotic ulcer; Biliary anastomosis leak; Pancreatic anastomosis leak; Anastomotic hemorrhage; Anastomotic stricture; Hemorrhage from tumor; Hemorrhage from portal vein stenosis/obstruction; Symptoms associated with portal vein stenosis/obstruction (Portal hypertension, Hepatic failure; Esophageal variceal hemorrhage; Gastritis; Ascites); Back pain; Abdominal pain

5) Anticipated Adverse Events Due to Metastasis

Liver failure, hepatic pain, fever, hepatic infection, respiratory failure, pulmonary infection, superior vena cava syndrome, abdominal distention, abdominal distention, ileus, esophageal obstruction, reverse smoking, gastric obstruction, duodenal obstruction, small bowel obstruction, colonic obstruction, rectal obstruction, rectal stenosis, anal bleeding, anorectal infection, nausea, diarrhea, constipation, ureteral obstruction, and urinary retention,urethral infection, urinary tract infection, bile duct obstruction, biliary obstruction, cholecystitis, biliary hemorrhage, gallbladder pain, pancreatitis, pain, narrowing of luminal organs near metastatic sites (tracheal/gastrointestinal tract, etc.), tracheal obstruction, bronchial stenosis, penetration with adjacent organs, perforation, hoarseness, pleural effusion, chest wall pain, pleural pain, atelectasis, hypercalcemia, disseminated intravascular coagulation, fracture, ataxia, cerebrovascular ischemia, intracranial hemorrhage,headache, dizziness, decreased level of consciousness, aphasia, seizures, spasticity

6) Paraneoplastic syndrome, etc.

(due to incompatible secretory syndrome) hyponatremia, (due to ectopic ACTH syndrome) personality changes, hypertension, hypokalemia, hyperglycemia, Lambert-Eaton myasthenic syndrome, subacute cerebellar degeneration associated with autoantibody production (including ataxia, dysarthria, and nystagmus of limbs), paraneoplastic encephalomyelitis, sensory neuropathy (including dementia, cranial nerve symptoms, dizziness, ataxia, autonomic ataxia, transverse paralysis, and sensory disturbance), thromboembolism, (due to gastrointestinal stenosis) aspiration, anaemia, tumor pain, acute renal failure, myositis, and pulmonary fibrosis

Complications associated with etiolation and gastrointestinal stent insertion are shown below.

- Percutaneous transhepatic cholangiodrainage (PTCD) procedures, including internal-external fistula tube placement:

  Pancreatitis, bile duct stricture, cholecystitis, gallbladder obstruction, gallbladder pain, liver failure, biliary

tract infection, liver infection, cholecystitis, septicemia, hemorrhage, PTCD tubing obstruction/deviation, peritoneal infection, pneumothorax, pleural pain, pleural effusion, intrapleural hemorrhage

- Biliary stenting:
  Pancreatitis, biliary stricture, cholecystitis, gallbladder obstruction, gallbladder pain, liver failure, biliary tract infection, liver infection, cholecystitis, sepsis, hemorrhage, peritoneum infection, pneumothorax, pleural pain, pleural effusion, intrapleural hemorrhage (in case of percutaneous placement), duodenal perforation, pneumonitis (in case of transendoscopic placement), stent obstruction/deviation, duodenal ulcer, and duodenal hemorrhage
- Choledochojejunostomy:
  Biliary tract infection; Pancreatitis; Cholecystitis; Liver infection; Sepsis
- Gastrointestinal stent insertion:
  Bleeding, perforation, pain, stent deviation, stent obstruction, ulceration, fever, sepsis, infection, diarrhea, constipation, urgency (colorectal), thyroid injury (esophagus), jugular arteriovenous injury (esophagus), and mediastinal abscess (esophagus).

7.3. Evaluation of Adverse Events/Reactions

The Common Terminology Criteria for Adverse Events v4.0 Japanese Translated JCOG Version (Japanese translation of NCI-Common Terminology Criteria for Adverse Events v4.0 (CTCAE v4.0)) (CTCAE v4.0-JCOG) will be used to assess adverse events/reactions. For CTCAE v4 0-JCOG in which Grade is defined by laboratory reference values at the institutional reference value, the "JCOG sharing reference range" will be used instead of the institutional reference value at each medical institution. For more information on JCOG sharing reference ranges, see JCOG website (see http://www.jcog.jp/doctor/tool/kijun.html)).

7.3.1. Grading of s adverse events

In grading of adverse events, each grading is closest to the definitions of Grade 0 4 (nearest match). Grading to a higher Grade when the definition of more than one Grade is comparable and when it is difficult to decide on either (highest grade).

Grading should also be given to Grade if specific actions are described, due to their clinical need. For example, patients may refuse oxygen inhalation or chest drainage, even when the patient's pleural effusion is increasing and oxygen inhalation or chest drainage is indicated. In such cases, grading is based on the medical judgment of what should have been done (what should be done) rather than on whether the treatment was actually given (what was actually done).

In the event of treatment-related deaths, original NCI-CTCAE states that the causative adverse event should be Grade 5, but the outcome of the serious adverse event is reported in the SAE report and reviewed in detail. Therefore, Grade 5 of the institutional physician's judgment is not likely to be changed, and whether or not the serious adverse event will result in death will be significantly affected by other factors than the event is not appropriate, so it is not appropriate to compare the frequency of the adverse event by Grade (%Grade 4 and%Grade 5, respectively) between treatment groups or between studies. Because of the poor significance of distinguishing between Grade 4 and Grade 5 in the tabulation, Grade 4 is not considered "Grade 5" in the recording form of this study. A discussion of the causal relationship between adverse events observed in treatment-related deaths and deaths should be included in the "Situation at Death" section of the treatment completion report form and follow-up form, and an urgent report should be made. For the adverse event items specified in "8.2. Testing and Assessment during Treatment" and "8.3. Testing and Endpoints after Treatment Completion" that are determined to be Grade 5 in the post-hoc review including the emergency report, Grade and the date of the first occurrence of the event should be included in the relevant record form (Treatment Course Record Form). If Grade 3 or greater is observed for any other adverse event, or if Grade 3/2/1 adverse event and treatment requires at least 24 hours of hospital stay or prolongation of hospital stay (see 10.1.1.3.)), the AE and Grade and the date of first occurrence should be included in the free form of the treatment course record.

Any Grade on the record form should be recorded in the medical record. Confirmed during site visit audit.

7.3.2. Determination of the causal relationship between adverse events and treatment

In determining the causal relationship between adverse events and treatment, patients are classified into 5 categories of "definite, probable, possible, unlikely, unrelated". Each "causality" is defined as "causality" when judged to be either "definite, probable, possible" and "no causality" when judged to be either "unlikely, unrelated" (see TABLE 7.3.2.

According to Grade of adverse events, serious adverse events requiring expedited reporting as specified in "10.1. Serious Adverse Events and Expedited Reporting" should be reported to the Research Secretariat in accordance with "10.2. Mandatory Reporting and Reporting Procedures of the Investigator".

Table 7.3.2. Criteria for a Causal Relationship Between Adverse Events and Treatment

	Determination	Approach to determination
Cause Fruit Concern Associate Ah Fistula	Definite	The AE is clearly related to the intervention<br>Adverse events are apparently caused/aggravated by protocol treatment and are unlikely to be due to exacerbation of the etiology or other factors (comorbidities, other medications/treatments, or incidents).
	Probable	The AE is likely related to the intervention<br>It is unlikely that the adverse event was caused/aggravated by progression of the underlying pathology or other factors (comorbidity, other drugs/treatments, incident) and is likely to be due to protocol treatment.
	Possible	The AE may be related to the intervention<br>It is plausible (plausible) that an adverse event is considered to have occurred/become more severe with protocol treatment, and unlikely to be due to exacerbation of the etiology or other factors (comorbidities, other medications/treatments, or incidents).
Cause Fruit Concern Associate In Pouch	Unlikely	The AE is doubtfully related to the intervention<br>It is considered plausible (plausible) that the adverse event is due to exacerbation of the pathogenic disease or other factors (comorbidity, other drugs/treatments, incident) rather than to the protocol treatment that it is caused/aggravated.
	Unrelated	The AE is clearly NOT related to the intervention<br>It is judged that the adverse event was caused/aggravated by aggravation of the pathogenic disease or other factors (comorbidity, other drugs/treatments, incident) and is unlikely to be caused by protocol treatment.

8. Examination and Evaluation

8.1. Baseline examination and evaluation before registration

8.1.1. Test conducted before registration (regardless of time before registration)

1） Histopathology (immunostaining requires chromogranin A and synaptophysin) (see Section 3.3)

2） HBs antigen; HBc antibody ※1; HBs antibody ※1, HBV-DNA※2

※1: For HBsAg positive, HBc and HBs antibodies are not required, and HBV-DNA, HBeAg, and HBe antibodies are measured.

※2: Positive results for at least one of the HBc and HBs antibodies also indicate HBV-DNA prior to initiation of therapy (see Section 6.4.1).

8.1.2. Test performed within 56 days before enrollment (liver primary (or unknown primary))

If the liver is primary (or of unknown primary), the following tests should be performed (all allowing for tests performed in other hospitals):

1）Upper gastrointestinal endoscope

2）Lower gastrointestinal endoscope

3）FDG-PET test

4）Otolaryngology (head and neck) examination

5）Urology consultation (male only)

6）Gynecologic exam (female only)

8.1.3. Tests performed within 28 days before enrollment

1) Contrast-enhanced CT※1 (slice thickness of 5 mm or less, if the patient is allergic to iodine), both contrast-enhanced MRI of the abdomen and plain computed tomography (CT) of the imaging range, which is considered to be indispensable below, are performed. All tests are not performed in other hospitals.

※The following areas are indispensable for each primary organ, and if there is another site suspected of metastasis, the radiographic extent is added accordingly.

Primary Organ	Essential radiographic area
Esophagus	Cervical, chest, or abdominal regions
Stomach, duodenum, small intestine, colon, appendix, rectum,<br>Extrahepatic bile duct, Vater ampulla, gallbladder, and pancreas	Chest, abdomen, and pelvis
Hepatic NEC<br>(liver primary or liver metastasis of unknown primary)	Neck, chest, abdomen, and pelvis

2) Endoscopic ※2

Primary Organ	Mandatory test ranges
Esophagus, stomach, duodenum, and ampulla of Vater	Upper gastrointestinal tract (no examination performed at another hospital)
Small intestine, extrahepatic bile ducts, gallbladder, and pancreas	Be not mandatory
Colon, appendix, and rectum	Lower gastrointestinal tract (no examination performed at another hospital)
Hepatic NEC<br>(liver primary or liver metastasis of unknown primary)	8.1.2. Refer to the test performed within 56 days before registration.

※2 Unnecessary if the primary lesion has been resected

3) 12-lead, resting electrocardiography

8.1.4. Tests performed within 14 days before enrollment

1) General condition: PS (ECOG), body weight

2) Physical findings

3) Peripheral blood count: white blood cell count, neutrophil count (ANC: rod + segmented karyocyte), hemoglobin, platelet count

4) Blood biochemistry: total protein, albumin, total bilirubin, AST (GOT), ALT (GPT), BUN, creatinine, LDH, ALP, sodium, potassium, calcium, magnesium, CRP, FBS (fasting blood glucose)
5) Creatinine clearance (CCr): CCr estimates by Cockcroft-Gault equation
   Cockcroft-Gault formula
   Male: Ccr = {(140-Age) × Body Weight (kg)}/{72 × Serum Creatinine Level (mg/dL)}
   Women: Ccr = 0.85 × {(140-Age) × Body Weight (kg)}/{72 × Serum Creatinine Level (mg/dL)}
6) Tumour markers: NSEs, ProGRP, CEAs, SCCs (esophagus primary only), CA19-9 (non-esophageal primary only)
7) Chest X-P (frontal) (substitutable if contrast-enhanced and plain chest CT is obtained)

8.2. Examination and evaluation during treatment

The following safety examination and evaluation are minimal in frequency: Performing examinations more frequently at the discretion of the treating physician is not prohibited.

However, the examination for efficacy evaluation should be performed at specified frequencies, unless progression is suspected, because dense frequency may lead to bias in the efficacy evaluation.

8.2.1. Safety endpoint assessed weekly (CTCAE v4 0 Japanese translation)

The following 1)-3) tests or evaluations should be performed at least weekly. In addition, all of the following items should be checked and evaluated on the scheduled date or the day before anticancer drug administration. However, at least weekly examinations or evaluations should be performed in the same manner until day 28 using the starting date of the last course as day 1, even when the treatment is completed or terminated.

1） Peripheral blood count: white blood cell count, neutrophil count (rod + segmented count), hemoglobin, and platelet count
2） Biochemical tests: albumin, total bilirubin, AST (GOT), ALT (GPT), creatinine, sodium, potassium, calcium, magnesium, CRP
3） Subjective and objective findings (described according to CTCAE v4.0JAPANESE TRANSLATION)
   - General disorders and administration site conditions: fever, fatigue
   - Ear and labyrinth disorders: Tinnitus, hearing impairment
   - Skin and subcutaneous tissue disorders: alopecia
   - Gastrointestinal disorders: Constipation, diarrhea, nausea, vomiting, oral mucositis
   - Metabolism and nutrition disorders: anorexia, dehydration
   - Nervous system disorders: dysgeusia, peripheral sensory neuropathy, peripheral motor neuropathy
   - Musculoskeletal and connective tissue disorders: myalgia, arthralgia
   - Infections and parasites: bronchial infection, pulmonary infection, upper respiratory tract infection, mediastinal infection, pleural infection, catheter-related infection, biliary tract infection, gallbladder infection, and urinary tract infection
   - Blood and lymphatic system disorders: Febrile Neutropenia
   - Vascular disorders: Phlebitis
   - Respiratory, thoracic and mediastinal disorders: dyspnea, hypoxia, pneumonitis

8.2.2. Safety examination and evaluation for each course

1) General condition: Body weight
2) Blood chemistry: LDH, ALP FBS (fasting glucose)

8.2.3. Safety examination and evaluation to be performed as necessary

1） When dyspnea is observed
   - Chest X-P, percutaneous oxygen saturation: SpO2, arterial blood gases: PaO_2
2） If an arrhythmia is observed
   - 12-lead, resting electrocardiography
3） When HBs antigen, HBs antibody, or HBc antibody is positive
   - HBV-DNA (see 6.4.1.).

8.2.4. Efficacy end point

The following tests will be performed every 6 weeks during protocol treatment (±1 week allowed: 6 ± 1 week, 12 ± 1 week, and 18 ± 1 week after the start date of protocol treatment). Tumor response will be assessed according to "11.1. Response Evaluation". Evaluation of the response will be performed using the same test conditions and test methods as the baseline evaluation.

If CT is allergic to contrast material, it is evaluated with plain CT and/or contrast-enhanced MRI. Allergy to contrast media on MRI is assessed by plain CT or plain MRI. When the use of CT contrast medium and MRI contrast medium becomes difficult due to renal dysfunction, it is evaluated by simple CT or simple MRI.

1） Tumour markers: NSEs, ProGRP, CEAs (≧all cases), SCCs (esophagus primary only), CA19-9 (non-esophageal primary only)

2） Contrast-enhanced CT: In principle, the extent of imaging is as follows. () Areas in can be omitted if there is no lesion

Primary Organ	Essential radiographic area
Esophagus	Cervical, chest, or abdominal regions
Stomach, duodenum, small intestine, colon, appendix, rectum,<br>Extrahepatic bile duct, Vater ampulla, gallbladder, and pancreas	(chest), abdomen, and pelvis
Liver NEC (liver primary or liver metastasis of unknown primary)	(neck), (chest), abdomen, and pelvis

8.3. Examination and evaluation after completion of treatment

8.3.1. Efficacy evaluation after completion of treatment

After completion of the protocol treatment, examination and evaluation are made at the following timing:
The following tests should be performed at least every 6 weeks until progressions are confirmed or death.

If progressions are observed, only observation of the outcome will be continued.

1） Tumour markers: NSEs, ProGRP, CEAs (≧all cases), SCCs (esophagus primary only), CA19-9 (non-esophageal primary only)

2） Contrast-enhanced CT: In principle, the extent of imaging is as follows. Areas in parentheses can be omitted if there is no lesion

Primary Organ	Essential radiographic area
Esophagus	Cervical, chest, or abdominal regions
Stomach, duodenum, small intestine, colon, appendix, rectum,<br>Extrahepatic bile duct, Vater ampulla, gallbladder, and pancreas	(chest), abdomen, and pelvis
Liver NEC (liver primary or liver metastasis of unknown primary)	(neck), (chest), abdomen, and pelvis

8.4. Information on post- study treatment

After completion/termination of protocol treatment, the following items will be recorded on the Follow-up Form at each follow-up survey:

1） Content of post-study treatment (if post-study treatment is performed)

2） After protocol treatment termination, the initiation date of the first post-study treatment ((if post-study treatment is performed)

3） PS at the start of post-study treatment

8.5. Study calendar

If the primary site is the esophagus, stomach, duodenum, small intestine, colon, appendix, rectum, extrahepatic bile duct, ampulla of Vater, gallbladder, or pancreas

	Before registratikon Record Pre	After end of chemotherapy		From the date of protocol treatment discontinuation Within 28 days	Until progression after completion of protocol treatment	
		Course Before initiation of the course	During the course		Only at the start of post-treatment	Every 6 weeks
Physical findings						
Body weight	$\circ^{14}$	○				
PS	$\circ^{14}$				■	
Physical findings	$\circ^{14}$	○			■	
Laboratory tests						
WBC, differential (neutrophil) Hb, platelets	$\circ^{14}$	○	●	●		
Alb, T-Bil, AST, ALT, Cr, Na, K, Ca, Mg, CRP	$\circ^{14}$	○	●	●		
LDH, ALP, FBS	$\circ^{14}$	○				
Total protein, BUN, Ca	$\circ^{14}$					
NSE, ProGRP、CEA	$\circ^{14}$		△			
SCC (esophagus primary only), CA19-9 (other than primary esophageal)	$\circ^{14}$		△			
HBs antigen, HBc antibody, and HBs antibody	○前					
Chest X-P (can be substituted if CT is taken)	$\circ^{14}$					
12-lead, resting electrocardiography	$\circ^{28}$					
Upper gastrointestinal endoscope (Primary: esophagus, stomach, duodenum)	$\circ^{28}$					
Lower gastrointestinal endoscope (Primary: colonic, appendix, rectum)	$\circ^{28}$					
Efficacy evaluation						
Contrast-Enhanced CT*	$\circ^{28}$		△			△
Toxicity evaluation						
Subjective symptom check		○	●	●		
Objective symptom check		○	●	●		
Submission of CRFs						
Pre-treatment Form		□				
Treatment Form			□			
Off-treatment Form				□		
Follow-up Form					2 times/year	

○前: Conduct before registration, $\circ^{28}$: Perform within 28 days before registration, $\circ^{14}$: Perform within 14 days before registration

○:Conduct, ♀: Implementation at least once a week

△: Every 6 weeks (see 8.2.4.), ∞: Only once at the beginning of aftertreatment, □: Submitted.

*See 8.1.3 for the shooting range. Contrast-induced allergy or renal dysfunction is assessed by plain CT or contrast-

enhanced MRI.

※Follow-up Forms will be sent up to 1 years after completion of accrual and will be submitted after 1 years of registration in the individual patient according to the closing date of registration.

For liver NEC (liver primary or liver metastasis of unknown primary)

	Before registratikon Record Pre	After end of chemotherapy		From the date of protocol treatment discontinuation Within 28 days	Until progression after completion of protocol treatment	
		Course Before initiation of the course	During the course		Only at the start of post-treatment	Every 6 weeks
Physical findings						
Body weight	○^{14}	○				
PS	○^{14}				■	
Physical findings	○^{14}	○			■	
Laboratory tests						
WBC, differential (neutrophil) Hb, platelets	○^{14}	○	●	●		
Alb, T-Bil, AST, ALT, Cr, Na,K, Ca, Mg, CRP	○^{14}	○	●	●		
LDH, ALP, FBS	○^{14}	○				
Total protein, BUN, Ca	○^{14}					
NSE, ProGRP、CEA、CA19-9	○^{14}		△			
HBs antigen, HBc antibody, and HBs antibody	○^{前}					
Chest X-P (can be substituted if CT is taken)	○^{14}					
12-lead, resting electrocardiography	○^{28}					
Upper gastrointestinal endoscope	○^{56}					
Lower gastrointestinal endoscope	○^{56}					
FDG-PET	○^{56}					
Otolaryngologic examination	○^{56}					
Urology consultation (male only)	○^{56}					
Gynecologic exam (female only)	○^{56}					
Efficacy evaluation						
Contrast-Enhanced CT*	○^{28}		△			△
Toxicity evaluation						
Subjective symptom check		○	●	●		
Objective symptom check		○	●	●		
Submission of CRFs						
Pre-treatment Form		□				
Treatment Form			□			
Off-treatment Form				□		
Follow-up Form					□ 2 times/year	

○^{前}: Conduct before registration, ○^{56}: Perform within 56 days before registration, ○^{28}: Perform within 28 days before registration,

○^{14}: Implemented within 14 days prior to enrollment

○:Conduct, ♀: Implementation at least once a week

△: Every 6 weeks (see 8.2.4.), ∞: Only once at the commencement of post-treatment, □: Submitted.

*See 8.1.3 for the shooting range. Contrast-induced allergy and renal dysfunction are evaluated by plain CT or contrast-enhanced MRI.

※Follow-up Forms will be sent up to 1 years after completion of accrual and will be submitted after 1 years of

registration in the individual patient according to the closing date of registration.

9. Data collection

9.1. Case Report Form (CRF)

9.1.1. Types of CRF and submission deadlines

The case report forms (CRF) used in this study and their submission deadlines are as follows:

1) Pre-treatment report (blue)- Less than 2 weeks after enrollment
2) Treatment course records- Every 2 cycles/less than 2 weeks after end of protocol treatment
   3）-1 Treatment (yellow)
   3）-2 Test (yellow)
   3）-3 Adverse events (yellow)
3) Tumor shrinkage report (green) - Less than 2 weeks after judgment of effect
4) End of treatment report (red) - Less than 2 weeks after discontinuation/end of protocol treatment
5) Follow-up investigation (white)- By the deadline indicated in the Follow-up Investigation Form

- For “1) Pre-treatment report to 4) End of treatment report”, CRFs with basic patient information (enrollment code, facility name) pre-printed on them would be sent by post from the data center. If the CRFs do not arrive within one week of enrollment, or if the CRFs have been lost/damaged, the data center should be contacted by telephone, and a request should be made for them to be re-issued.
- “5) Follow-up investigation” would be sent by post from the data center at the time of follow-up investigations that are conducted at the same time as monitoring and interim/final analyses at the data center.

9.1.2. Storage CRF

- Completed CRFs must all be archived at the facility as photocopies or in electronic form.
- Copies of CRFs should be kept archived until the final analysis report is issued for reference while filling other CRFs, or for review while retrieving information from the data center.

9.1.3. Method of sending CRF

- All CRF must be sent by post or handed over in person at the data center. They must not be sent by FAX.
- To avoid the risk of personal patient information being leaked, the patient enrollment code should be used, instead of using the patient medical chart number at the facility, when contacting the data center for request of CRF dispatch.

9.1.4. Correction of the contents of CRFs

If any data necessary for the CRF are found to be missing or there are inappropriate category classifications after the start of the study, the CRFs may be corrected with the agreement of the head of the data center and the clinical trial secretariat, in a manner not exceeding the scope of data collected as prescribed in “8. Evaluation item/Clinical laboratory tests/Evaluation schedule”, and within the scope deemed not to increase medical and financial burden on the enrolled patient from the CRF correction. Modification of CRFs that do not require the main body of the protocol to be revised is not considered a protocol revision by JCOG. Reports to the head of the medical institution related to CRF correction and the request of application for revision should follow the rules of the facility.

10. Reporting of "disease or the like"(adverse events)

Site investigator should report to Study Coordinator/Principal Investigator (Study Chair) if a serious adverse event ("disease or the like" on Clinical Trials Act) occurs in accordance with the regulations of Clinical Trials Act (Law No. 16, 2017), Enforcement Regulations of Clinical Trials Act (MHLW Notification No. 17, 2018) and the regulations in this chapter based on the relevant notifications.

The most recent version of the report is available on the MHLW website [1)] and on the JCOG website [2)]. Use the most recent version of the report.

Serious adverse events occurring after the initiation of protocol treatment (after the date of registration if death) by the date of final follow-up will be subjects.

1) http://www.mhlw.go.jp/stf/seisakunitsuite/bunya/0000163417.html
2) http://www.jcog.jp/doctor/todo/researcher/harmfulness.html
3) http://www.pmda.go.jp/safety/reports/hcp/pmd-act/0002.html

10.1. Serious Adverse Events and subjects of Expedited Reporting.

Serious adverse events are defined as any of the following:
(These are classified as "disease or the like" on Clinical Trials Act.)

1) Death
2) Diseases that may lead to death
3) "Disease or the like" requiring hospitalization or prolongation of hospital stay for treatment.
4) Disability
5) "Disease or the like" that may lead to disability
6) Serious "disease or the like" according to 1) to 5)
7) Congenital disease or abnormality in later generations

Of these, those that fall under any of the following categories shall be regarded as serious adverse events for which Expedited Reporting is mandatory.

1) Death

(i) All deaths that occur after registration and before the start of protocol treatment
(ii) All deaths (with or without causality to protocol treatment) that occur during protocol treatment or within 30 days of the last treatment day
(iii) Death that occur after 31 days from the last treatment date that are causally related to protocol treatment (definite, probable, possible)

2) "Disease or the like" that may lead to death

(i) Grade 4 adverse events that occur during protocol treatment or within 30 days of the last treatment day (excluding events in Table 10.1)
(ii) Grade 4 adverse events that occur after 31 days from the last treatment date (excluding events in Table 10.1) that are causally related to the protocol treatment (definite, probable, possible)

3) "Disease or the like" requiring hospitalization or prolongation of hospital stay[※1] for treatment

(i) Grade 3/2/1 adverse events that occur during or within 30 days of protocol treatment and requiring at least 24 hours of hospitalization or prolongation of hospital stay[※1] to treat the adverse event (excluding the event in Table 10.1).
(ii) Grade 3/2/1 adverse events that occur 31 days after the last treatment day and requires 24-hour or longer hospitalization or prolongation of hospital stay[※1] for treatment and causally related to protocol treatment (definite, probable, possible) (excluding the events in Table 10.1)

※ 1 "Hospitalization or prolongation of hospital stay" refers only to those for which hospitalization of at least 24 hours or prolongation of hospital stay is medically required for the treatment of an adverse event. The followings are not subjects for reporting:

- Hospitalization or prolongation of hospital stay performed for follow-up of adverse event that has disappeared or improved

- Hospitalization or prolongation of hospital stay for reducing patient burden, e.g. patients from distant areas.
- Hospitalization or prolongation of hospital stay for other medically unnecessary situation

※ 2 "Unexpected" refers to those not listed in "7. Expected Adverse Events"

4) Disability, 5) "Disease or the like" that may lead to disability

Permanent or marked disability/dysfunction (excluding myelodysplastic syndromes, secondary cancers, etc.) or possible medical situation

6) Serious "disease or the like" similar to 1) to 5)

7) Congenital disorders or abnormalities in later generations

Table 10.1. Adverse events excluded from the subjects of Expedited Reporting

SOC※(CTCAE ver4.0)	AE term
Blood and lymphocyte disorders	Anemia, bone marrow hypocellular
Gastrointestinal disorders	Constipation
General disorders and local symptoms	Fever
Infections and infestations	Viral hepatitis
Clinical laboratory test	ALP increased, CD4 lymphocytes decreased, high cholesterol, GGT increased, lipase increased, lymphocytes decreased, neutrophils decreased, platelet count decreased, serum amylase increased, WBC decreased, hyponatremia, hypokalemia, hyperglycemia, and hypoglycemia
Metabolism and nutritional disorders	Obesity, anorexia, hyperuricemia, and hypoalbuminemia
Musculoskeletal and connective tissue disorders	Fibrosis deep connective tissue and superficial soft tissue fibrosis
Renal and urinary disorders	Chronic kidney disease
Respiratory, thoracic, and mediastinal disorders	Sinus disorder and sleep apnea
Skin and subcutaneous tissue disorders	Hypohidrosis

※ SOC:System Organ Class

10.2. Investigator's reporting requirements and procedures

10.2.1. Expedited Reporting

In the event of a serious adverse event for which Expedited Reporting is mandatory, the Subinvestigator must promptly inform the Investigator. If the Investigator cannot be contacted, the Site Coordinator or Subinvestigator must take over the responsibility of the Investigator. The Investigator must report adverse events according to the following procedures.

Attention should be paid not to include the patient's name and medical record number when sent.

Serious adverse events that occur after the initiation of protocol treatment (after the date of registration if death) by the date of final follow-up are subjects of Expedited Reporting.

1) "Disease or the like" that may lead to death or death specified in 10.1 1) and 2).

Primary reporting:

The Subinvestigator who is aware of the occurrence of adverse events will promptly notify the Investigator. The Investigator who receives the notice should fill out JCOG Adverse Event Report Form(for institution) and the "Disease or the like" Report Form to Certified Review Board specified in Clinical Trials Act Enforcement Regulations as far as possible and contact Principal Investigator/Study Coordinator via e-mail within 72 hours of knowledge of the occurrence of the adverse event.

Secondary reporting:

The Investigator should add detailed information on adverse events to JCOG Adverse Event Report Form(for

institution) and the "Disease or the like" Report Form to Certified Review Board specified in Clinical Trials Act Enforcement Regulations within 7 days of knowledge of the occurrence of adverse events and send them to the Principal Investigator/Study Coordinator via e-mail. If necessary, attach copies of laboratory data, images, autopsy report, etc.

2) 10.1. 3)"Disease or the like" or other medically important condition requiring hospitalization or prolongation of hospital stay for treatment. Adverse events are judged to be either of 10.1. 4)-7)

The Subinvestigator who is aware of the occurrence of adverse events will promptly notify the Investigator. The Investigator who receives notice must fill out JCOG Adverse Event Report Form (for institution) and the "Disease or the like" Report Form to Certified Review Board specified in Clinical Trials Act Enforcement Regulations within 10 days of knowledge of the occurrence of an adverse event and send them to the Principal Investigator/Study Coordinator via e-mail. If necessary, attach copies of laboratory data, images, autopsy report, etc.

3) Additional reporting

If new information is obtained after conducting the above reporting, the Investigator must add information to JCOG Adverse Event Report Form (for institution) and the "Disease or the like" Report Form to Certified Review Board specified in Clinical Trials Act Enforcement Regulations and report it as needed.

Table 10.2.1. Summary of Adverse Events which are subjects for Expedited Reporting and the deadline of reporting to Principal Investigator/Study Coordinator

Causal relationship	The patient was hospitalized due to Grade 1-3 AE. Other medically important conditions		Grade 4		Death	
	Expected	Not expected	Expected	Not expected	Expected	Not expected
Present	No need of reporting	Primary reporting: within 10 days Additional reporting: as needed	Primary reporting: within 72 hours Secondary reporting: within 7 days Additional reporting: as needed			
None	<Only on-treatment or within 30 days of last protocol treatment day>					
	No need of reporting	Primary reporting: within 10 days Additional reporting: as needed	Primary reporting: within 72 hours Secondary reporting: within 7 days Additional reporting: as needed			

* 4) Disability, 5) "Disease or the like" that may lead to disability, and 6) "Disease or the like" that are serious similar to 1) to 5) in 10.1., 7) Congenital disorders or abnormalities in later generations

※ "Unexpected" refers to those not listed in "7. Expected Adverse Events"

Table 10.2.1. Summary of Adverse Events which are subjects for Expedited Reporting and the deadline of reporting to Principal Investigator/Study Coordinator

Causal relationship	The patient was hospitalized due to Grade 1-3 AE. *Other medically important conditions		Grade 4		Death	
	Expected	Not expected	Expected	Not expected	Expected	Not expected
Present	Primary reporting: within 10 days Additional reporting: as needed	Primary reporting: within 10 days Additional reporting: as needed	Primary reporting: within 72 hours Secondary reporting: within 7 days Additional reporting: as needed			
None	<Only on-treatment or within 30 days of last protocol treatment day>					
	Primary reporting: within 10 days Additional reporting: as needed	Primary reporting: within 10 days Additional reporting: as needed	Primary reporting: within 72 hours Secondary reporting: within 7 days Additional reporting: as needed			

* 4) Disability, 5) "Disease or the like" that may lead to disability, and 6) "Disease or the like" that are serious similar to 1) to 5) in 10.1., 7) Congenital disorders or abnormalities in later generations

※ "Unexpected" refers to those not listed in "7. Expected Adverse Events"

10.2.2. Reporting to the Administrator of participating medical organizations

If an Adverse Event which is subjects for Expedited Reporting occurs and is assessed as causal after reporting to Principal Investigator and reported to Certified Review Board, the Investigator must report it to the Administrator of the relevant medical institution in accordance with the requirements of the medical institution.

10.3. Responsibilities of Principal Investigator/Study Coordinator

10.3.1. Determination of necessity of suspension of registration and emergency notification to institutions

Principal Investigator/Study Coordinator who received the report from the Site Investigator should report to Group Chair and determine the urgency, significance, and impact of the report. If needed, take measures such as suspending registration (contacting JCOG Data Center and all participating institutions) and urgently communicating information to participating institutions. Telephone calls can be made to Data Center and institutions as urgent, but they should also be promptly contacted by document (e-mail).

10.3.2. Reporting to JCOG Operations Office and Certified Review Board and MHLW

1) Reporting from the Principal Investigator/Study Coordinator to JCOG Operations Office

Principal Investigator/Study Coordinator should consult with Group Chair and report to JCOG Operations Office (Safety Contact) by e-mail within 72 hours of knowledge of the occurrence of the adverse event, if reported AE is considered to meet the adverse events specified in 10.2.1. 1) and 2) In doing so, to the extent feasible, Principal Investigator/Study Coordinator should send "JCOG Adverse Event Report Form (for institution)" sent from the institution, "Disease or the like Report Form" addressed to Certified Review Board as stipulated in Clinical Trials Act Enforcement Regulations, and attach "JCOG Adverse Event Report (for Study Coordinator)" with Study Coordinator/Principal Investigator's view (including judgments of causality and expectation, and judgments of continuation/discontinuation of the study)". For the expected adverse events of 1) or 2) in 10.2.1., include a discussion not only of the individual patient's course but also of whether the frequency of appearance is within the expected range.

2) Reporting to Certified Review Board

JCOG Operations Office (Safety Contact) reviews the appropriateness of the judgement of causality and expectation of adverse events reported in the above procedures and can ask Principal Investigator/Study Coordinator to reconsider them if there is any doubt. Adverse events considered by Principal Investigator/Study Coordinator and JCOG Operations Office to be related to the protocol treatment and to be the subject of reporting in the following tables should reported to Certified Review Board through JCOG Operations Office.

If there is a disagreement between Principal Investigator/Study Coordinator and JCOG Operations Office, report it to Data and Safety Monitoring Committee and seek final judgment from the Chair of Data and Safety Monitoring Committee. However, if the reporting may exceed deadlines of reporting, the report can be tentatively reported as "causal" to Certified Review Board.

Reporting subjects and reporting deadline

Principal Investigator/Study Coordinator must report to Certified Review Board through JCOG Operations Office within the following time periods after knowledge of the occurrence of adverse events.

Causal relation ship	The patient was hospitalized due to Grade 1-3 AE. Other medically important condition		Grade 4		Death	
	Expected	Not expected	Expected	Not expected	Expected	Not expected
Present	No need of reporting	Within 15 days	Within 15 days	Within 7 days	Within 15 days	Within 7 days
None	No need of	No need of	No need of	No need of	No need of	No need of

	reporting	reporting	reporting	reporting	reporting	reporting

※ "Unexpected" refers to those not listed in "7. Expected Adverse Events"

Principal Investigator/Study Coordinator must report adverse events to Certified Review Board through JCOG Operations Office within the following deadlines after knowledge of the occurrence of adverse events.

Causal relation ship	The patient was hospitalized due to Grade 1-3 AE. Other medically important condition		Grade 4		Death	
	Expected	Not expected	Expected	Not expected	Expected	Not expected
Present	Within 30 days	Within 15 days	Within 30 days	Within 15 days	Within 15 days	Within 15 days
None	No need of reporting	No need of reporting	No need of reporting	No need of reporting	No need of reporting	No need of reporting

※ "Unexpected" refers to those not listed in "7. Expected Adverse Events"

3) Reporting to MHLW

Principal Investigator/Study Coordinator of the study with unapproved or off-label health care should report "Disease or the like" Report Form specified in Clinical Trials Act Enforcement Regulations to MHLW through JCOG Operations Office, if an adverse event is considered to be unexpected and to have a causal relationship to the protocol treatment (See 10.5. Responsibilities of the Data and Safety Monitoring Committee).

Reporting subjects and reporting deadlines

Principal Investigator/Study Coordinator should report to the MHLW through JCOG Operations Office within the following deadlines after knowledge of the occurrence of adverse events.

※ Pharmaceuticals and Medical Devices Agency Safety Division I (trk-shippeitouhokoku@pmda.go.jp)

Causal relation ship	The patient was hospitalized due to Grade 1-3 AE. Other medically important condition		Grade 4		Death	
	Expected	Not expected	Expected	Not expected	Expected	Not expected
Present	No need of reporting	Within 15 days	No need of reporting	Within 7 days	No need of reporting	Within 7 days
None	No need of reporting	No need of reporting	No need of reporting	No need of reporting	No need of reporting	No need of reporting

※ "Unexpected" refers to those not listed in "7. Expected Adverse Events"

4) Additional reporting

Following receipt of secondary or additional reports from the Investigator, Principal Investigator/Study Coordinator must add additional information from the primary report and their views to JCOG Adverse Event Report Form (for the institution) and the "Disease or the like" Report Form to Certified Review Board set out in Clinical Trials Act Enforcement Regulations, and promptly contact JCOG Operations Office (Safety Contact) by e-mail. If the report was sent to Certified Review Board and the MHLW in the primary reporting,, the secondary reporting and additional reporting must be made in the same manner.

10.3.3. Notification to the Site Investigators

When reported to Certified Review Board, Principal Investigator/Study Coordinator should inform the Investigators of all participating institutions of the review results and recommendations by documents (e-mail is allowed). Principal Investigator/Study Coordinator must inform the Investigators without waiting for Certified Review Board review if there is any urgent information to be disseminated. In the event that reported to the MHLW, Principal Investigator/Study Coordinator should notify the Investigators of all participating institutions.

In addition, even if no reporting is made to Certified Review Board, Principal Investigator/Study Coordinator must inform the Investigator of the reporting institution of the decision of Principal Investigator/Study Coordinator by documents (e-mail is allowed).

10.3.4. Assessment of Adverse Events in Periodic Monitoring

During Periodic Monitoring, Principal investigator/Study Coordinator should carefully review the adverse events in the Monitoring Reports issued by the Data Center and ensure that there are no missed reporting from the participating institutions. It should also be confirmed that all reported adverse events are listed in the Monitoring Reports. The presence or absence of a missed reporting should be indicated in the column of the results of Group review on the Periodic Monitoring Report.

10.4. Responsibilities of the Site Investigators at the participating institutions (including the relevant institution)

In accordance with the instructions of Principal Investigator/Study Coordinator, the Site Investigator at the participating institution should report to the administrator of the relevant institution if the adverse event is subjects of reporting of "disease or the like" to Certified Review Board in accordance with the regulations of the relevant institution.

10.5. Responsibilities of the Data and Safety Monitoring Committee

JCOG Operations Office (Safety Contact) should check the details of the adverse event reports received from the Principal Investigator/Study Coordinator according to the procedures described in 10.3.2. and should report them to Certified Review Board and the MHLW according to the procedures described below, with the presence or absence of causality or expectation.

In addition, Principal Investigator and JCOG Operations Office can hear the opinions of JCOG Data and Safety Monitoring Committee according to the reported adverse events. If a review request is issued, Data and Safety Monitoring Committee can review the appropriateness of the institutional response to adverse events and the propriety of continuation of the study in a consensus or written form.

In addition, the submitted information (JCOG Adverse Event Report (for institution), JCOG Adverse Event Report (for Study Coordinator), "Disease or the like" Report, etc.) will be stored semi-permanently in JCOG Operations Office.

Subjects, destination and deadlines for reporting after the knowledge by Investigator/Study Coordinator are as described in 10.3.2.

11. Response Evaluation and Endpoint Definition

11.1. Response assessment (only for patients with measurable disease)

Tumour response assessment will be performed according to the following steps according to the [49] of version 1. 1-Japanese translational JCOG version-Revised RECIST guideline (version 1. 1) Revised new guidelines for the assessment of treatment response in solid tumours (RECIST guidelines). RECISTv1. The 0 original article stipulates that "the use of this guideline for the purpose of determining continuation of treatment is not the subject of this guideline." Similar statements continue to be included in RECISTv1 1 as follows.

> "Many oncologists make decisions about whether to continue treatment based on both objective imaging criteria and symptom-based criteria for follow-up of patients with malignancies in their daily clinical practice, but these revised guidelines are not intended to be used to make decisions about whether or not to continue treatment in these individual patients, unless the treating oncologist determines that it is appropriate."

Therefore, the "overall effect" as determined by RECIST Guideline-based response assessment should be used to determine whether a drug or regimen shows encouraging results that merit continued developmental studies. In other words, judgment of whether or not to continue treatment in individual patients should not be based on CR/PR/SD/PD of overall efficacy, but rather on "clinical judgment" based on comprehensive consideration of symptoms, physical findings, and various laboratory data, in addition to imaging findings.

Therefore, it may be clinically appropriate to continue protocolized treatment, even when PD (Progressive Disease: progression) is judged as an overall response based on the assessment of response based on imaging. In this case, the pros and cons of continuing protocol treatment should be determined based on clinical judgment, regardless of response assessment, but the date of the event for progression-free survival, which is judged to be an overall effect of PD, should be used. This is due to three reasons: (i) it may be possible to decide whether protocol treatment should be continued for each group; (ii) RECIST is a criterion intended to standardize not only response rates but also progression-free survival; and (iii) the standard definition of US Cooperative Group is that PD is the event of progression-free survival for any reason if the overall response is PD.

On the other hand, if a physician judges "clinical progression" based on clinical and comprehensive judgment not based on diagnostic imaging, even if PD is not met by the response criteria based on diagnostic imaging, protocol treatment should be discontinued in accordance with "6.2.2. Criteria for discontinuation of protocol treatment". If "clinical exacerbation" is judged, even if "PD" is not judged by the response evaluation, the day of "clinical exacerbation" is considered as an event of progression-free survival. This is because imaging is often not performed as planned after a patient is judged to have a "clinical progression" and therefore the risk of overestimating progression-free survival is greater if "clinical progression" is not an event for progression-free survival. It is also statistically incorrect (informative censoring) to treat "clinical progression" as "censoring" progression-free survival, as it would censoring patients at increased risk of progression or death.

In RECISTv1 1, the original article described "definite progression (unequivocal progression") in the PD criteria for non-target lesions as "marked progression of non-target lesions that deserves discontinuation of treatment as an increase in total tumor burden" and therefore described "marked progression of non-target lesions" as "judgement of whether or not to continue treatment in individual patients" in some of the PD criteria for non-target lesions, which is confusing. It should be noted that this "unequivocal progression" is a criterion of judgment restricted to "PD of non-target lesions".

The relation between the events of 'PD', 'clinical progression', 'progression', and progression-free survival in JCOG is as in the lower panel.

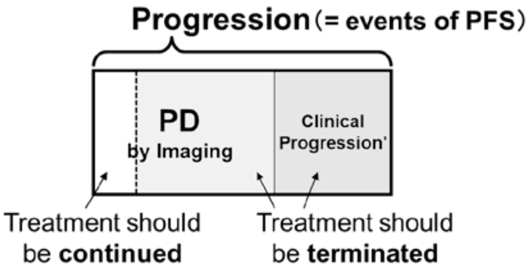

Figure 11.1. Relationship Between Exacerbations, PD on Imaging, and Clinical Exacerbations.

11.1.1. Baseline Evaluation

「8.1. According to "Pre-registration Evaluation Items" contrast enhanced-enhanced computed tomography (CT) with a range of indispensable for the primary organ is used to identify neoplastic lesions before enrollment, and each lesion is classified into "measurable lesions" and "unmeasurable lesions". If CT is allergic to contrast medium, both contrast-enhanced MRI and plain CT should be performed.

Tumor diameters are measured by CT or MRI in cross-sectional views, and sagittal and coronal measurements by three-dimensional reconstructed images are not used. Baseline assessment will be done using the latest imaging studies within 28 days prior to enrollment. If the imaging test is re-examined after enrollment and before the start of treatment, the latest imaging test with re-examination should be used.

In this study, the tumour markers NSEs and ProGRP are used to evaluate the response of non-target lesions.

11.1.2. Definition of measurable lesions

Lesions that fall under any of the following conditions are considered measurable lesions (measurable lesion):

1) Non-nodal disease (non-nodal disease) of 10 mm or greater in greatest dimension on CT or MRI with a slice thickness of 5 mm or less
2) CT or MRI of 5 mm or less slice thickness showing lymph node lesion of 15 mm or more in short diameter (Nodal lesions with short diameters between 10 mm and less than 15 mm are defined as non-target lesions, and those with short diameters less than 10 mm are not)

All other lesions will be non-measurable (non-measurable lesion).

Caution should be exercised because the following lesions are not measurable regardless of the examination method or the size of the lesion.

- Bone lesions (excluding osteolytic lesions with measurable soft tissue components)
- Cystic lesion
- Leptomeningeal lesions
- Ascites, pleural effusion, and pericardial effusion
- Lymphangiosis of the skin and lungs
- Palpable but not measurable abdominal mass or enlargement of abdominal organs
- Superficial skin lesions

11.1.3. Target Lesion Selection and Baseline Recording

Up to five measurable lesions, in descending order of diameter (non-lymph node lesions are long diameters and nodal lesions are short diameters), up to two lesions per organ are selected to be target lesions (target lesion) among measurable lesions at enrollment. Selection should take into account the universal inclusion of as many organs with measurable disease as possible and the reproducibility or ease of measurement (reproducible repeated measurement) of repeated measurements (avoiding lesions that are not measurable even if they are large in diameter).

For selected target lesions, in order from cranial to caudal, site (code), test method, test date, long diameter of non-lymph node target lesion, short diameter of nodal target lesion, and sum of all target lesion diameters (hereafter, sum of diameters) will be recorded in Pretreatment Report 3.

11.1.4. Baseline recording of non-target lesions

For lesions not selected as target lesions, all measurable or non-target lesions (non-target lesion) should be recorded as site of lesion (code), method of examination, and date of examination in Pretreatment Report 3. Multiple non-target lesions within the same organ may be recorded as a single lesion (e.g., multiple enlarged pelvic lymph nodes, multiple liver metastases).

11.1.5. Determining tumor response

Evaluation of target and non-target lesions will be performed every 6 weeks according to "8.2 Testing and Evaluation during Treatment" in the same manner as at enrollment. Target lesion diameter, non-target lesion disappearance or progression will be recorded in the "Tumor Reduction Effect Report".

11.1.6. Response Evaluation Criteria for Target Lesions

・CR(Complete Response): Complete response

When all non-lymph node target lesions disappear and all nodal target lesions have a short diameter of

less than 10 mm. If a nodal target lesion is selected at baseline, the effect of the target lesion may be CR even if the sum of diameters is not 0 mm.

・PR(Partial Response): Partial response

30% or more reduction in target diameter sum compared to baseline diameter sum

・PD(Progressive Disease): Progress

Compared to the minimum diameters (when the baseline is the minimum value during the passage, this is the minimum sum of diameters), the sum of the target disease increases by more than 20%, and the sum of diameters increases by more than 5 mm even in absolute value.

・SD(Stable Disease): Stability

There is no reduction corresponding to PR and no increase corresponding to PD compared to the smallest sum of diameters during the course

・Lack of study; Not all Evaluated

If the test cannot be performed for any reason or if neither CR, PR, PD, or SD can be determined

Pre-treatment sum of diameters-sum of diameters at study
Percentage of reduction of the diameter sum =---------------× x 100%
Pre-treatment diameter sum

Sum of diameters at study-minimum sum of diameters
Increasing Percentage of Diagram =---------------× x 100%
Minimum sum of diameters

※ Measured target lesion diameters are recorded whenever measurable (e.g., <5 mm). If the target lesion diameter is judged to be too small to be measured (too small to measure), the diameter should be 0 mm if the tumor lesion is judged not to be retained, and 5 mm if the tumor lesion is judged to be retained, regardless of the CT slice thickness.

※ PD is defined when the reduction ratio meets the condition of PR and the concomitant increase ratio meets the condition of PD.

※ When one lesion is separated during treatment, each diameter is added to the sum of diameters.

※ If more than one lesion fuses and the boundary cannot be distinguished during treatment, the diameter of the fused lesion is added to the sum of the diameters. The diameter of each lesion is added to the sum of diameters when the boundary of the lesion is identifiable, even if the lesion is in contact with each other.

11.1.7. Response Evaluation Criteria for Non-Target Lesions

・CR(Complete Response): Complete response

When all non-lymph node non-target lesions disappear, the short diameter of all nodal non-target lesions becomes less than 10 mm, and all tumour markers (NSEs, ProGRP※) are below the upper shared reference limits. When there are no (on the image) non-target lesions at baseline assessment and all tumor markers are below the shared reference range upper limit.

Because ※ProGRP is not included in the shared baseline range, the baseline range is 6.5-46.0 pg/mL.

・Non-CR/non-PD: non-CR/non-PD

Residual one or more non-target lesions (including residual nodal non-target lesions ≧10 mm in short diameter) and/or tumour markers (NSEs, ProGRP※) exceeding the shared upper reference limits. No non-target lesions (on images) at baseline assessment and any of the tumor markers exceeding the shared reference range upper limit.

・PD(Progressive Disease): Progress

'Apparent exacerbation' (including relapse) of pre-existing non-target lesions.

For measurable disease: A marked progression of a non-target lesion that deserves discontinuation of treatment as an increase in overall tumor burden must be observed if the effect of the target lesion is SD or PR but is judged to be "clear progression" based on the change in the non-target lesion. If the effect of the target lesion is SD or PR, then an increase in the tumor burden of the non-target lesion to a degree

that far exceeds the decrease in tumor burden is considered "obvious progression" and otherwise Non-CR/non-PD.

If only unmeasurable disease is present, the increase in non-target disease, as judged to clearly exceed the tumor burden corresponding to a 20% increase in diameter and a 73% increase in tumor volume, is considered "definite progression". Elevated tumor markers alone are not considered PD.

・NE(Not all Evaluated): Lack of study

If the test could not be done for any reason or if neither CR, Non-CR/non-PD nor PD could be determined.

11.1.8. Presence or absence of new lesions

If a lesion that was not present at baseline was observed after the start of treatment, it is considered "new lesion" to be present.

However, a "new lesion" requires that it is not an imaging change due to a difference in the imaging method from the baseline assessment or a change in the imaging modality, nor is it an imaging change due to a condition other than the tumor. For example, a cystic lesion arising within a lesion due to necrosis of a liver metastatic lesion is not a new lesion. New lesions will be defined as new lesions by examination of sites that were not mandatory at baseline (pre-enrollment study).

If a lesion disappears and later reappears, measurement is continued. However, the effect at the time the lesion reappears depends on the status of the other lesion. When the overall effect reappears after CR, the lesion is judged as PD at the time of reappearance. When the overall effect is PR or SD, on the other hand, once the disappeared lesion reappears, the diameter of the lesion will be added to the sum of the diameters of the remaining lesions to calculate the effect. That is, in the presence of many residual lesions, even if one lesion reappears after an apparent disappearance, it is not judged as PD by itself, and it is judged as PD when the sum of the diameters of all lesions meets the criteria for PD. This is because of the perception that the majority of lesions do not truly 'disappear' and are not only depicted by the limits of resolution of the imaging modalities used.

If there is a possibility of a new lesion but it cannot be determined, it should not be a new lesion, and imaging should be reexamined at a clinically relevant time. If a new lesion is confirmed by repeat imaging, the new lesion will appear based on the date of imaging at which the new lesion is confirmed.

11.1.9. Overall efficacy (Overall Response)

The overall response (Overall response) will be determined by combining the effects of target lesions, non-target lesions, and the presence or absence of new lesions every 6 weeks according to Table 11.1.9.a below. The overall effect in the absence of a non-target lesion at baseline will be determined by the effect of the target lesion and the presence or absence of a new lesion, and the overall effect in the absence of a target lesion at baseline will be determined according to the effect of a non-target lesion and the presence or absence of a new lesion according to Table 11.1.9.b.

Table 11.1.9.a. Overall efficacy at each time point for target lesions (with or without non-target lesions)

Target lesion	Nontarget lesions	New lesions	Overall effect
CR	CR	None	CR
CR	Non-CR/non-PD	None	PR
CR	Lack of study	None	PR
PR	Lack of Non-PD or study	None	PR
SD	Lack of Non-PD or study	None	SD
Lack of study	Non-PD	None	NE
PD (obvious progression)	Irrespective of	With or without	PD
Irrespective of	PD	With or without	PD
Irrespective of	Irrespective of	Present	PD

Table 11.1.9.b. Overall efficacy at each time point for patients with non-target lesions only

Nontarget lesions	New lesions	Overall effect
CR	None	CR

Non-CR/non-PD	None	Non-CR/non-PD
Lack of study	None	NE
PD (obvious progression)	With or without	PD
Irrespective of	Present	PD

11.1.10. Best overall effectiveness (Best Overall Response)

CR > PR > SD > PD > NE is considered good, and the best overall effect is the best overall effect throughout the entire course.

PD is defined when imaging cannot be determined due to exacerbation of obvious disease or death before the first response assessment. In addition, NE is defined if it cannot be determined by imaging due to discontinuation of toxicity before the first response assessment or patient refusal.

11.2. Definitions of analyses set

The analysis sets used in periodic central monitoring, interim analysis, and final analysis are defined as follows: The flow diagram below shows the analysis sets.

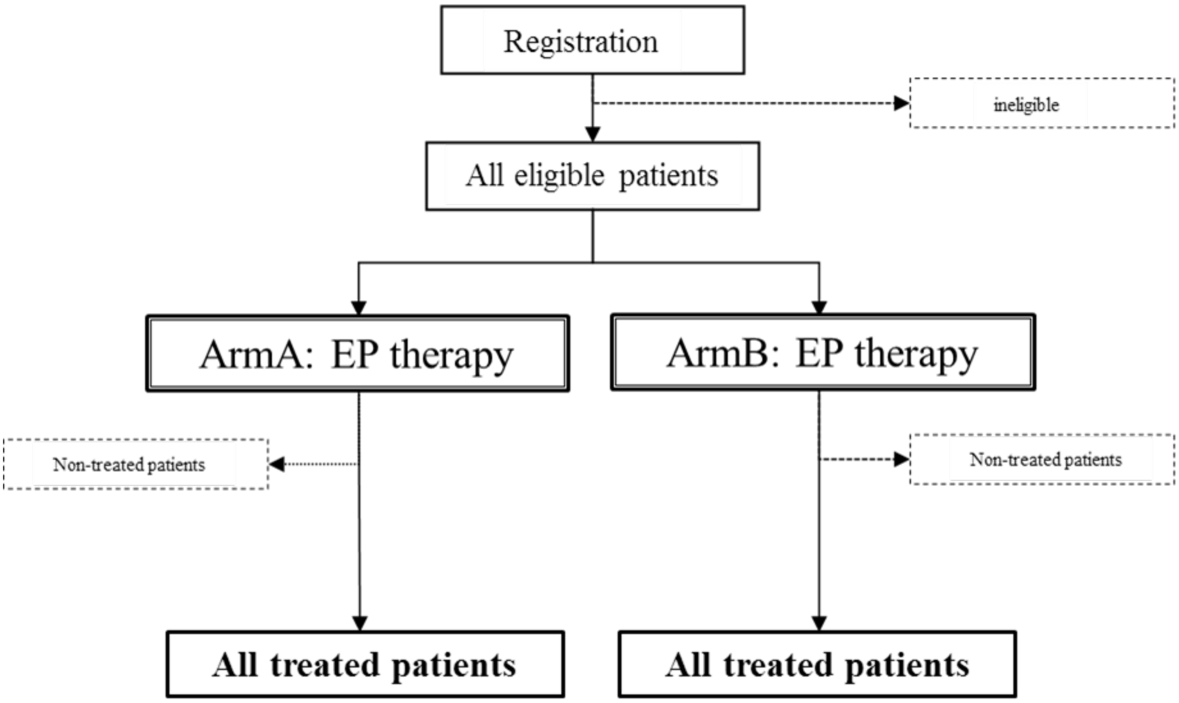

11.2.1. All registered patients

「5.1. Among the patients enrolled according to the Procedures for Enrollment, the population excluding duplicate or mis-enrollment is considered as "all enrolled cases".

11.2.2. All eligible patients

The group excluding "ineligible cases (post hoc ineligibles, de facto ineligibles, violation of registration)" determined by group review from all registered patients is regarded as all eligible patients. Ineligible cases as judged by the investigator or sub-investigator alone are included in all eligible patients. Only those judged not eligible by the central pathological diagnosis are ineligible and not included in all eligible patients.

11.2.3. All treated patients

Of all enrolled patients, all patients for whom part or all of the protocol treatment was performed will be defined as all treated patients.

The decision to treat "non-treated patients" for whom no protocol treatment has been given and whether it is excluded from the safety analysis can be determined by the data center with the consent of the Research Office. Ineligible patients will be excluded from all treated patients. However, if there are circumstances in which ineligible patients are included in the analysis, the nature of the ineligibility will be examined and determined by the Research Secretariat in consultation with JCOG Data Centre.

11.2.4. Patients eligible for central pathology diagnosis

Of all eligible patients, the population excluding ineligible patients with a central pathological diagnosis will be considered as eligible patients with a central pathological diagnosis.

11.3. Definition of endpoints

Endpoint	Events (whichever is earlier)		Censoring date
Overall survival time<br>Overall survival(OS)	All deaths	-	Date of final survival confirmation
Progression-free survival time<br>Progression-free survival(PFS)	All deaths	Progression/recurrence	Final date of clinically confirmed freedom from exacerbation

11.3.1. Overall survival

The duration from the date of registration to the date of death from any cause.

- Survivors are censored at the date of final survival confirmation (survival confirmation by telephone contact is also permitted, but the fact that survival confirmation was performed should be recorded in the medical record).
- Patients lost to follow-up are censored at the last date of survival confirmation before lost to follow-up.

11.3.2. Progression-free survival (PFS: Progression-free survival).

The duration from the date of registration to the date of the judgement of exacerbation or death from any cause, whichever comes first.

- The exacerbation (progression) includes both imaging-based PD (progression) and exacerbation of pathogenic disease (clinical exacerbation) that cannot be confirmed by imaging studies in Section 11.1.9. Global Effectiveness. If an exacerbation is judged based on diagnostic imaging, the exacerbation date is the test date on which the imaging was performed, and in the case of clinical exacerbation, the date of clinical judgment is the exacerbation date. PD may occur in accordance with the Response Evaluation Criteria, even if the tumor diameter is very small, but the disease is clinically judged to be "not obviously aggravated" in accordance with the Response Evaluation Criteria Criteria (in this case, clinical judgment should be prioritized for continued treatment with the protocol). In addition, even if PD is not observed according to the response evaluation criteria, the clinical judgment is prioritized for progression if it is judged to be clinically obvious exacerbation.
- In survivors who are not judged to have progression, clinically confirmed progression is censored (date of final progression-free survival confirmation) (confirmation of progression-free by imaging or specimen examination is not mandatory and clinical progression-free by outpatient examination etc. is not permitted. Telephone contact alone is not permitted. If information on progression or progression-free is obtained at a medical institution or referral center, receive and retain a medical information form describing the rationale for diagnosis. In this case, telephone contact alone is not permitted).
- Events and censoring are treated similarly, if chemotherapy is terminated for reasons such as toxicity or patient refusal, and if other therapies are added as post-study treatment. i.e., it is not censored at the time of treatment termination or at the date of initiation of post-study treatment.
- When the diagnosis of exacerbation is based on imaging, the relapse is not regarded as an event at the test date of imaging with "suspicious diagnosis of relapse", but regarded as an event at the later test date of imaging with "definite diagnosis of relapse"" If the event is judged to be clinically aggravated based on diagnostic imaging, the event is considered to be the day of the progression.
- If the definitive diagnosis of relapse or new lesion is based on biopsy pathology, the date of clinical diagnosis is defined as the date of clinical diagnosis when the diagnosis of recurrence or new lesion is made clinically, and the date of biopsy is defined as the date of event when the diagnosis of recurrence is made based on biopsy pathology diagnosis without clinical diagnosis of recurrence.
- The incidence of a second cancer (metachronous double cancer) is not censored or event, and progression-free survival is defined until other events are observed.

11.3.3. Response rate (response rate) Response proportion (Response rate)

Among all eligible patients with measurable disease, the response rate is defined as the proportion of patients with "11.1.10. Best Global Effect" that is either CR or PR.

11.3.4. Incidence of adverse events (adverse reactions)

Using all treated patients as the denominator, the frequency of the worst Grade during the entire course by CTCAE v4 0 Japanese translation JCOG version for each of the following adverse events (toxicities) will be determined by group:

- Laboratory tests: hemoglobin decreased, white blood cell decreased, neutrophil count decreased, platelet count decreased,

    Increased blood bilirubin, aspartate aminotransferase increased (AST or GOT),
    Alanine aminotransferase increased (ALT or GPT), creatinine increased,
    Hypernatremia, hyponatremia, hyperkalemia, hypokalemia, hypercalcemia, hypocalcemia,

hypermagnesemia, hypomagnesemia

- General disorders and administration site conditions: fever, fatigue
- Ear and labyrinth disorders: Tinnitus, hearing impairment
- Skin and subcutaneous tissue disorders: alopecia
- Gastrointestinal disorders: constipation, diarrhea, nausea, vomiting, oral mucositis
- Metabolism and nutrition disorders: anorexia, dehydration
- Nervous system disorders: dysgeusia, peripheral sensory neuropathy, peripheral motor neuropathy
- Musculoskeletal and connective tissue disorders: myalgia, arthralgia
- Infections and parasites: bronchial infection, pulmonary infection, upper respiratory tract infection, catheter-related infection, biliary tract infection,
  Gallbladder infection; Mediastinal infection; Pleural infection; Urinary tract infection
- Blood and lymphatic system disorders: Febrile Neutropenia
- Musculoskeletal and connective tissue disorders: arthralgia, Vascular disorders: phlebitis
- Respiratory, thoracic and mediastinal disorders: dyspnea, hypoxia, pneumonitis

In the other adverse event (toxicitiy) than the above, the proportion of occurrence are not calculated unless a large number of specific adverse events are observed, since only Grade 3 or more non-hematological toxicity ※ is reported in the Treatment Form.

※※ Non-hematological toxicity refers to adverse events other than those listed below in CTCAE v4.0-JCJCOG.
Anemia, decreased bone marrow cells, decreased lymphocyte count, decreased neutrophil count, decreased white blood cell count, and decreased platelet count.
CD4 lymphocytopenia

11.3.5. Dose intensity of cisplatin

Dose intensity of cisplatin per patient (DOOC.) will be calculated for all treated patients to assess treatment compliance with cisplatin. The summary statistics (minimum, 25% point, median, 75% point, maximum, mean, and standard deviation) will be calculated for each group.

- ・Active dose D.I. (mg/m2/week) = total drug dosebody surface areatreatment duration (weeks)
- Body surface area: BSA is calculated by height at enrollment and body weight calculated by Data Center.
- ・ Treatment period (weeks)
  Arm A = (start date of last course-start date of 1st course + 21)/7
  Arm B = (start date of last course-start date of 1st course + 28)/ 7

11.3.6. Incidence of serious adverse events (adverse reactions)

1) Grade 4 non-hematologic toxicities, early deaths, and treatment

Using all treatments as denominators, the percentage of patients with one or more Grade 4 non-hematologic toxic ※ that are considered to be related (either to definite, probable, possible) to the protocol treatment among the adverse events listed in the free text of the CRFs in addition to the stereotyped items in Section 11.3.4 is used as the numerator.

※※ Non-hematological toxicity refers to adverse events other than those listed below in CTCAE v4.0-JCJCOG.
Anemia, decreased bone marrow cells, decreased lymphocyte count, decreased neutrophil count, decreased white blood cell count, and decreased platelet count.
CD4 lymphocytopenia

2) Early mortality rate

Proportion of all deaths during the protocol treatment or within 30 days from the last protocol treatment day among all treated patients. Causes of death irrespective of causality with protocol treatment. However, if premature death occurs in patients excluded from all treated patients, the details are provided separately.

3) Proportion of treatment-related death (TRD incidence)

Proportion of all deaths judged as causally related (either definite, probable, possible) to the protocol treatment among all treated patients However, if TRD occurs in patients who have been removed from all treated patients, the details are presented separately.

12. Statistical consideration

Methods for statistical analysis are as follows: In addition, the details required for conducting specific analyses are specified in the statistical analysis plan prepared separately prior to the analysis, and in documents that clarify the endpoint definition, etc. If substantial changes occur in statistical analyses as described below, follow the policy set out in "13.6. Protocol Changes." Facilities are contacted by "14.1. Periodic Monitoring" for missing or abnormal results, and data collection or exclusion is determined by review by the Research Secretariat based on the results of inquiries to the institution in accordance with the policies stipulated in "14.1.2. eligible (eligibility/ineligibility)" and "14.1.3. Protocol Deviations/Violations". 「11. Handling of missing values and abnormal data that cannot be addressed by the definition and analysis method for each endpoint, as specified in "Definition of Response Evaluation and Endpoints" and "12. Statistical Items" below, is specified in the above statistical analysis plan.

12.1. Principal Analysis and Decision Criteria

The primary analysis of this study will be the final analysis.

The purpose analysis of this trial is to test whether one of the two community standard-of-care arm A (EP-therapy) and B (IP-therapy) outperforms primary endpoint overall survival. The null hypothesis of equal overall survival in the two groups in the main analysis will be tested by stratified log-rank test stratified by non-institutional allocation adjustment factors (primary organ [gastrointestinal vs. hepatobiliary-pancreatic]) in all patient patients. However, if it is assumed that a stratified log-rank test cannot be performed appropriately, such as when the number of subjects and events in each stratum is small, the allocation adjustment factors will be addressed in the analysis plan prepared without information related to the comparison between groups before performing a confirmatory analysis with comparison between groups. Sensitivity analysis will also be performed in all eligible patients and in patients eligible for central pathology diagnosis.

Due to interest in which treatment group is superior, the test will be two-tailed. The study-wise significance level for the study is set at both sides 10%. In the main analysis, two-sided 90% confidence intervals corresponding to a two-sided 10% significance level will be calculated, and in other analyses, two-sided 95% confidence intervals will be calculated for descriptive purposes.

We conclude that EP therapy is a more useful treatment when the two-sided p-value is less than 10% and the survival curve of EP therapy exceeds that of IP therapy. Meanwhile, we conclude that IP therapy is a more useful treatment when the two-sided p-value is less than 10% and the survival curve of IP therapy exceeds that of EP therapy. If the difference was not significant at a two-sided significance level of 10%, one of the two modalities cannot be judged to be useful. In this case, we conclude that both modalities continue to be the standard of care, as there is no evidence to actively recommend either one of them.

However, if there are major differences in the toxicity profiles of EP and IP therapies, contrary to prior assumptions, during the course of the study, and if the toxic therapy is not superior to other therapies, it is judged that it will not be of significance to be used in clinical practice, the following measures should be taken. In other words, the clinical hypothesis will be changed from two-sided to one-sided in an analysis plan with no information on group comparisons before a confirmatory analysis with group comparisons will be conducted, and the superiority of the toxicity treatment over other treatment modalities will be verified at a one-sided significance level of 5% (in this case, the policy of change for the interim analysis will be described in Section 12.3.2).

Estimates such as cumulative survival curves, median survival times, and annual survival rates are performed using Kaplan-Meier method. Brookmeyer and Crowley methods are used to obtain 95% confidence intervals for median survival. Greenwood's formula is used to obtain 95% confidence intervals for annual survival rates. Hazard ratios and their confidence intervals for treatment effects between groups are obtained using stratified Cox proportional hazards models with the same factors as the test of the primary analysis as estimates of treatment effects. Cox regression adjusted by imbalance background factors in addition to adjustment factors will be performed as needed.

The main analysis results will be summarized as the "Main Analysis Report" by the Data Center one year after the completion of enrollment and submitted to the Research Secretariat, Research Representatives, Group Representatives, Group Secretariats, Efficacy and Safety Assessment Committee, and JCOG Representatives.

The principal investigator/study coordinator summarizes the content of the main analysis report, prepares a

"Clinical Study Report" summarizing the conclusions, problems, interpretations and discussion of the results, and future policies of the entire study, and submits it to the Data and Safety Monitoring Committee and JCOG chair with approval from the Group chair and the Head of JCOG Data Center.

Approval of the clinical study report by the Data and Safety Monitoring Committee shall be considered as "end of the study."

12.2. Planned accrual, accrual period, and follow-up periods

「2.4.2. Based on the background presented in Clinical Hypothesis and Rationale for Number of Enrollment, we assume a median survival of 8 and 12 months (HR=0.67) for the inferior and superior treatment groups, respectively, among the two treatment groups. When a superiority trial design is used, 63 patients per group and 126 patients in both groups (114 events required) will be included in the required analysis using Schoenfeld & Richter methodological [50] at 6 years of enrollment, 1 year of follow-up, α = 10% (two-sided), and 70% power. The number of required Inferior treatment (number of required events) when the median survival time in A is different from the assumption as shown in Table 12.2.1 below.

Table 12.2.1. Number of Analyses Required (Number of Events Required)

	Power			
Median Survival (mo)	65%	70%	75%	80%
7.0 vs. 10.5	108(100)	124(114)	142(131)	162(150)
8.0 vs. 12.0	112(100)	126(114)	144(131)	166(150)
9.0 vs. 13.5	114(100)	130(114)	148(131)	170(150)

※※ Number of required events in parentheses

Based on these findings, the following will be established in view of some cases of loss to follow-up.

Planned enrollment: 70 patients in each group and 140 patients in both groups

Enrollment period: 6 years, follow-up period: 1 year after completion of enrollment

Consider redesigning the sample size if the prognosis is obviously better than assumed, or if it reaches 70 people within 2.5 years of enrollment initiation. Clinically meaningful differences will then be reviewed and redesigned in a blinded fashion prior to the conduct of the analysis.

<Additions in ver. 1.1>

The registration pace after the enrollment start was smooth and exceeded the plan, and 70 cases were reached in October, 2016, when 2 years and 2 months passed from the enrollment start. Since it exceeded the standard of 70 patients within 2.5 years from the initiation of enrollment specified above, acceptance was obtained at the meeting of the Hepatobiliary and Pancreatic Group on October 29, 2016, the group meeting of the Esophageal Cancer Group on November 19, 2016, and the group meeting of the Gastric Cancer Group on January 7, 2017 with respect to changing the power to 80% according to the rules at the time of the study plan. Therefore, the planned enrollment number was changed to 170. In addition, the "1-year analysis period" was added to the study period, and the following changes were made.

Planned enrollment: 85 patients in each group and 170 patients in both groups

Enrollment period: 6 years; Follow-up period: 1 year after completion of enrollment; Analysis period: 1 year;

Total study period: 8 years

<Additional information in ver. 1.5.0>

In the monitoring in the latter half of fiscal 2019, it was found that the number of events was less than expected. Follow-up will be extended by 1 year to 2 years after completion of enrollment because the required number of events may not be reached at 1 year after completion of enrollment for which final follow-up is planned. However, if the required number of events has been reached at 1 year after completion of enrollment, the final analysis will be performed at 1 year after completion of enrollment. Conversely, if the required number of events is not reached, follow-up will continue until the required number of events is reached to ensure power, and the final analysis will be performed without waiting for the end of the remaining follow-up period when the required number of events is found to be reached within the follow-up period.

Planned enrollment: 85 patients in each group and 170 patients in both groups

Enrollment period: 6 years; follow-up period: 2 years after completion of enrollment; analysis period: 1 year; total study period: 9 years

12.3. Interim Analysis and Early Termination of the Study

12.3.1. Purpose and Timing of the Interim Analysis

Once interim analyses will be conducted to determine if the primary objective of the study has been achieved during the study period. Interim analyses will be conducted to determine if it is reasonable to continue enrollment during enrollment. If the primary objective of the study is determined to be achieved, the study will be discontinued and the study results will be published promptly at the conference and in the article.

Interim analyses will be conducted using data from the initial periodic monitoring that will be queried after the time enrollment of half of the planned enrollment was obtained. Based on the information in the periodic monitoring report, the group will submit the presence or absence of changes in clinical hypotheses and, if any, details of changes to the Efficacy and Safety Assessment Committee by the time of the interim analysis.

During the first interim, patient accrual is continued in principle. If the progress of the study progressed as planned, the expected number of events at the time of the interim analysis under the assumptions presented in 12.2 is expected to be 44 if the interim analysis is conducted at 3 years after the start of enrollment.

12.3.2. Method of interim analysis

Interim analyses will be conducted by the JCOG Data Center. To keep the study-wise alpha errors at 10%, the multiplicity of the interim and final analyses is adjusted using Lan & DeMets's alpha spending functions, and statistical significance is examined for differences in primary endpoint between arms. As α spending functions, we use O'Brien & Fleming types.[51]

For details of the interim analysis, the statistical staff in charge of the group at the Data Center will prepare the statistical analysis plan by the time of the interim analysis. Actual interim analyses will be performed by statistical staff who are not in charge of the group and an interim analysis report will be prepared.

In the interim analysis, if the survival of one treatment group exceeds that of the period, and the p-value of the stratified log-rank test falls below the level specified by the above method, it is judged statistically significant and the trial is discontinued as a rule. The ineffective discontinuation is not planned at a stage where no statistically significant differences are observed unless prior assumptions regarding toxicity are changed. However, the clinical hypothesis may be changed for the reasons described in 12.1, and the primary analysis may be performed by one-sided rather than two-sided testing, which may result in deviations from prior assumptions. In such cases, the primary hypothesis change should be specified in the analysis plan to be prepared without information on group comparisons before a confirmatory analysis with group comparisons. If the overall survival curve in the highly toxic treatment group is below the other, the consideration of ineffective discontinuation should not be judged by a test and considered comprehensively.

12.3.3. Interim analysis Reporting and review of the results

The results of the interim analysis will be submitted to the Data and Safety Monitoring Committee by the Data Center as an Interim Analysis Report and reviewed for the acceptability of continuation of the study and for publication of the results. The Data and Safety Monitoring Committee considers whether to continue the study at the meeting and recommends whether to continue the study and whether to publish the results to principal physician or group chair based on the results of the review.

Members of the Data and Safety Monitoring Committee of the relevant group are not included in the review. Unless the results of the interim analysis make recommendations for discontinuation of the study from the Efficacy and Safety Assessment Committee, the research representative, research office, participating institution researchers, group representatives, and group secretaries of the study will not be able to know the results of the interim analysis until the final follow-up is completed.

When the Interim Analysis Report has been reviewed by the Data and Safety Monitoring Committee to recommend termination or change of all or part of the study, the principle investigator and group chair review the recommendations and decide whether to discontinue or change some of the study.

If the study is discontinued or part of the study is changed, the principal investigator and group chair shall submit

in written form a request to the Data and Safety Monitoring Committee for permission to discontinue the study or a request to revise the protocol. Following approval by the Data and Safety Monitoring Committee, the principal investigator may discontinue the study or change part of the study.

The Study Chair and Group Chair can disagree with the recommendations of the Data and Safety Monitoring Committee, but if they fail to coordinate their opinions with the Data and Safety Monitoring Committee, they will ultimately follow the instructions of JCOG Chair.

If the study is terminated, the subsequent follow-up period will be 1 years from last registration per study.

If the interim analysis resulted in study termination, the interim analysis will be the primary analysis of the study. The Data Center, in cooperation with the Research Representative Physicians and Research Secretariat, will conduct the analysis required to complement the incomplete data and publish the results, focusing on the results of the interim analysis, and promptly prepare the Major Analysis Report and submit it to the Group and the Efficacy and Safety Assessment Committee.

12.4. Analysis of Secondary endpoints

Secondary endpoints analyses will be conducted to provide a supplementary discussion of the primary analysis results of the study. Because the analysis of secondary endpoint is exploratory, no multiplicity adjustments are made. Comparisons between arms are made where appropriate, note that when the results of the group comparisons are not significant, they do not mean that there is no difference between the two arms.

12.4.1. Analysis of safety secondary endpoints

Among Secondary endpoints, the safety endpoints are the incidence of adverse events and the incidence of serious adverse events, which are in principle the items of periodic monitoring (14.1. Periodic monitoring).

The incidence rate of adverse events will be summarized as well as the incidence rate of Grade3 or higher. For adverse events other than laboratory data, the incidence of Grade 2 or higher is also calculated.. Non-hematological toxicity incidence, early mortality, and treatment-related mortality rates of Grade4, which are serious adverse events, are reported in periodic monitoring reports with registration numbers and details. The rates of non-hematologic toxicity, early mortality, and treatment-related mortality for Grade4 will be calculated at the time of the interim analysis and the main analysis. When interval estimation of proportions is performed, accurate confidence intervals based on binomial distributions are used. Comparisons between arms will be made using Fisher's exact test where appropriate.

12.4.2. Analysis of efficacy secondary endpoints

Among Secondary endpoints, efficacy endpoints are response rate, progression-free survival, which will only be analyzed in the interim and primary analyses.

Secondary endpoints analyses do not adjust for multiplicity.

Progression-free survival will be included in all enrolled patients, but a comparison of all eligible patients, excluding ineligible patients, as determined by group study, will also be performed as a sensitivity analysis.

The response rate will include all eligible patients with measurable disease, but a comparison of all enrolled patients will also be performed as a sensitivity analysis.

Fisher's exact test will be used to compare response rates between groups, and binomial distribution-based exact confidence intervals will be used for interval estimation. Estimates, including progression-free survival curves, median progression-free survival, and time-point progression-free survival, will be performed using Kaplan-Meier method, Brookmeyer and Crowley methods will be used to obtain confidence intervals for median progression-free survival, and Greenwood formulas will be used to obtain confidence intervals for progression-free survival. Log-rank test is used for comparison between arms. Hazard ratios and their 95% confidence intervals for treatment effects between arms will be calculated using Cox's proportional hazards model as an estimate of treatment effect. Cox regression adjusted by imbalance background factors in addition to adjustment factors will be performed as needed.

12.5. Final analysis

The primary analysis will be the final analysis unless this study is withdrawn from the interim analysis.

If the interim analysis is withdrawn from the study, then after the end of the 1-year follow-up period, the final analysis will be performed after the final survey confirms the data and then analyses will be performed for all

endpoints.

Except for the interim analyses and the final analysis, analyses with between-arms comparisons for the primary and secondary endpoints for efficacy are not performed unless approved by the Data and Safety Monitoring Committee.

If the final analysis is performed after the main analysis, the final analysis will be summarized by the Data Center as the "Final Analysis Report" and submitted to the Research Secretariat, Research Representatives, Group Representatives, Group Secretariats, Efficacy and Safety Assessment Committee, and JCOG Representatives.

The Study Representative Physician/Research Secretariat summarizes the content of the final analysis report, prepares the "Clinical Study Report" summarizing the conclusions, issues, interpretations and discussion of the results, future policies, etc. mainly from the clinical point of view (if the "Clinical Study Report" is prepared in the previous analysis report, it will be the "Clinical Study Report (Supplementary Version)) with additional updates), and submits it to the Study Representatives and JCOG Representatives with approval from the Group Representatives and the Head of JCOG Data Center.

Approval of the clinical study report by the Data and Safety Monitoring Committee shall be considered as "end of the study."

<Additional information in ver. 1.5.0>

If the required number of events has been reached at 1 year after completion of enrollment, the final analysis will be performed at 1 year after completion of enrollment. Conversely, if the required number of events is not reached, follow-up will continue until the required number of events is reached to ensure power, and the final analysis will be performed without waiting for the end of the remaining follow-up period when the required number of events is found to be reached within the follow-up period.

12.6. Exploratory analysis

To investigate the interaction between treatment effect and the subpopulation, subgroup analyses will be conducted exploratory with respect to the following factors: Because these analyses are not adequately powered and do not adjust for multiplicity, the results of each subgroup analysis should be interpreted as exploratory.

- PS0/1
- Age 65 years or older/<
- Gender (male/female)
- Primary organ (gastrointestinal tract/hepatobiliary pancreas)
- Organ of origin (esophagus/stomach/small intestine/large intestine/pancreas/biliary tract/liver NEC (liver primary or liver metastasis of unknown primary)
- Organ of origin (pancreas/non-pancreas)
- Extent of extension of the primary lesion (locally advanced/distant metastasis or recurrence)
- Extent of extension of the primary lesion (locally advanced/distant metastasis/recurrence)
- Prior radical resection of the primary lesion (none/present)
- Pathological diagnosis was biopsy/resection specimen
- Grade 3 tumour with morphologically similar features of NETs but Grade 3 proliferative activity/morphologically more atypical (previously classified as poorly differentiated endocrine carcinoma) on histopathology with central pathology
- Histopathological examination by central pathological diagnosis, including Small cell carcinoma/Large cell carcinoma /
- Histopathologically diagnosed by central pathology, Ki67 50% or higher/less than 50%

12.7. Premature withdrawal from the trial

In this study, early termination of the study may occur in the following cases:

1) Early termination due to discontinuation of interim analysis
2) Early termination due to adverse events
3) Early termination due to poor enrollment
4) Early termination due to other reasons

12.7.1. Early termination by interim analysis

In this study, based on the criteria described in 12.3, early termination recommendations may be made at the interim analysis review by the Efficacy and Safety Assessment Committee. If the Data and Safety Monitoring Committee provides recommendations for early termination of the study, the principle investigator and group chair will review the recommendations and decide whether to terminate the study early.

12.7.2. Early termination due to adverse events

In JCOG9511 for small-cell lung cancer, 1/77 (1.3%) treatment-related deaths were reported with EP therapy and 3/77 (3.9%) with IP therapy; in JCOG0509, a successor study for the same subject, 1/142 (0.7%) treatment-related deaths were reported with IP therapy and 2/142 (1.4%) with amrubicin plus cisplatin therapy. Using these as reference, we believe that the treatment-related mortality rate should not exceed 3% in this study. Since it is clear that the final point estimate will be at least 3% at the time of 3 treatment-related deaths in either group, immediate entry should be suspended to consider whether or not to withdraw from the study. At this point, the subsequent treatment of the patient being treated will be reviewed. At the time of 3 or fewer treatment-related deaths in each group, each patient will be reported to the Efficacy and Safety Assessment Committee for adjudication. Enrollment will be continued until the results are obtained in principle.

12.7.3. Early termination due to poor enrollment

If the patient enrollment pace is significantly worse than at the time of planning, early termination of the study may be advised by the Data and Safety Monitoring Committee. If early termination recommendations are issued by the Data and Safety Monitoring Committee due to poor enrollment, the principle investigator and group chair will review the recommendations and decide whether to terminate the study early.

12.7.4. Early termination due to other reasons

12.7.1.～12.7.3. If it is judged difficult to continue the study for other reasons, the research representative physician shall submit a request for early termination of the study to the Efficacy and Safety Assessment Committee. If the Data and Safety Monitoring Committee recommends early termination of the study based on the submitted data, the procedure for early termination of the study will be progressed.

12.8. Procedures after Early termination of the Study

If the Study Chair accepts the recommendations made by the Data and Safety Monitoring Committee based on Section 12.7, he/she will promptly submit a notification to the Data and Safety Monitoring Committee that early termination of the study will be performed.

The Study Chair will submit a termination notification to the Certified Review Board within 10 days of the date they decide to terminate the study early. If the study falls into a specified clinical trials under the Clinical Trials Act, the Study Chair shall submit a termination notification to the Certified Review Board within 10 days of the date on which the study was decided to be prematurely discontinued, as well as submit a specified clinical trials termination notification to the MHLW.

The Study Chair promptly informs the investigator of the decision to terminate the study early in writing, and the investigator who has received a report of early termination of the study will report in writing that the study was prematurely terminated to the institution's manager without delay.

If the study is terminated early, JCOG Data Center will promptly initiate the development of the primary analysis report or final analysis report. The subsequent follow-up period will be 1 year from the final enrollment.

13. Ethical Considerations

13.1. Protection of Human Subjects

All researchers involved in this study will conduct this trial in accordance with the "Helsinki Declaration" 1) and "Clinical Trials Act" (2017 Law No. 16) 2) "Clinical Trials Act Enforcement Regulations" (2018 Ministry of Health, Labour and Welfare Order No. 17) and related notices.

1） http://dl.med.or.jp/dl-med/wma/helsinki2013j.pdf
2） http://www.mhlw.go.jp/stf/seisakunitsuite/bunya/0000163417.html

Prior to commencing this study, the site investigator is required to obtain input from Certified Review Board※1 regarding the conduct of the study, and to obtain approval from the Administrator of the participating institution, and submit the Implementation Plan ※2 to the Ministry of Health, Labour and Welfare.

※ 1　JCOG study will be submitted to the following Certified Review Board.
National Cancer Center Hospital Certified Review Board (accreditation number CRB3180008)

※ 2　"Implementation Plan" means "Documented plan formatted according to the Form No.1 (Form No.1 of the Ministerial Ordinance) specified in Article 39 of the Clinical Trials Act Enforcement Regulations"

13.2. Informed consent

13.2.1. Explanation to the patient

Prior to patient registration, the investigator or subinvestigator will provide the patient with written informed consent form approved by Certified Review Board and explain the following details verbally.

Descriptions

1） Disease names, Stages, and expected prognosis
2） That this study is a clinical trial and is conducted by JCOG
Name of Certified Review Board and contact information for receiving complaints and inquiries to the committee
3） Design and rationale of the study
4） Protocol treatment content
5） Effects expected by protocol treatment
6） Expected adverse events, complications, and sequelae and how to deal with them
Explanation of the extent and frequency of expected adverse events, including complications, sequelae, and treatment-related deaths, and how to deal with them when they occur. In addition to these explanation, obtain the most recent version of the drug package insert and deliver it to patients (PMDA Prescription Pharmaceutical Information Search http ://www.pmda.go. jp/PmdaSearch/iyakuSearch/)
7） Post-study treatment after end of protocol treatment should also be performed appropriately
8） Cost burden and compensations
Explanation of the cost of treatment, compensation that can be received in the event of a health hazard (equivalent to measures taken in general practice, etc.)
9） Alternative treatment
Explanation of treatments that can be received if not participating in this study
10） Anticipated benefits and possible disadvantages
Explanation of anticipated benefits and possible disadvantages by participating in this study
11） Direct access to the medical records
Explanations on acceptance of site visit audits, such as "direct access to medical records etc. by healthcare professionals at other medical institutions for quality control with permission from the administrator of the participating institution."
12） Refusal of consent and withdrawal of consent
Refusal to consent prior to participation in the study is free, and withdrawal after having given consent is free, thereby not causing undue medical disadvantage.
13） Protecting human rights
Every effort should be made to ensure that personal information, such as names, is kept confidential.
14） Secondary use of data

The possibility of secondary use of data obtained from this study in Japan and overseas (ancillary studies, meta-analyses, etc.) only when approved by either committee in JCOG

15） Method of disclosure of information on the study

The study is registered and published in jRCT※. In addition, the results of clinical studies should also be published in jRCT (⁎ Databases (Japan Registry of Clinical Trials) https://jrct.niph.go.jp/ prepared by the MHLW as stipulated in Paragraph 1 in Article 24 of Clinical Trials Act Enforcement Regulations)

16） Freedom of questions

Explanation that investigators, written contact information for consultations on study details, on the Principal Investigator and the Study Coordinator, and freely asking questions about study and treatment

17） Explanation of the use and burden of medicinal products not covered by insurance

18） Central pathological review

19） Central image review for response

20） Sample collection for ancillary studies

13.2.2. Consent

Explain the study, give sufficient time to think, confirm that the patient understood the study well, and ask for participation in the study. If the patient agrees to participate in the study, the written informed consent form in the appendix will be used to obtain the patient's own signature. The site investigator or the subinvestigator confirms that the study consent form contains the name of the physician who provided the explanation and the date of explanation, the name of the patient who gave informed consent, and the date of informed consent.

In addition, when it is not possible to read the documents due to visual impairment, etc., but the details can be understood by verbal explanation, or the documents can not be signed due to limb disorders, etc., but the documents can be read and understood, signatures may be obtained from the proxy author under the consent by the patient. However, the signature of the proxy author should be based on this study's consent, and should be described as "Signing by the proxy author" and "Relationship with the patient" so that the person can be found to be the proxy author.

Two copies of the consent form will be provided, one will be handed over to the patient, and one will be stored by the site coordinator. Original copies will be stored in the medical records or in the archives specified by the participating institution.

13.2.3. Response to inquiries, consultations, etc. after consent

In principle, the investigator or subinvestigator of the relevant patient's participating institution responds to any consultation related to the study by the patient or his/her family after registration. If it is unclear how to respond, respond in consultation with Principal Investigator, Study Coordinator, the Group Secretariat, Group Chair, JCOG Data Center/Operations Office, etc. in accordance with the content of the consultation.

13.2.4. Withdrawal of consent

After obtaining informed consent for participation in the study, consent will be withdrawn if the patient expressed withdrawal of consent.

Withdrawal of consent refers to withdrawal of consent to participate in research and is distinguished from refusal to continue protocol treatment (below (i)). If the withdrawal of consent is expressed, clarify whether (ii) or (iii) below and promptly notify JCOG Data Center. If consent is withdrawn, record it in the medical record as well as (ii) or (iii).

Data Center discontinues subsequent follow-up requests according to the protocol in case of (ii) withdrawal of consent, In the case of (iii) full withdrawal of consent, the data of the patient are removed from the database when it is confirmed that the patient has full withdrawn of consent.

The procedures for discontinuation of the patient's follow-up request and removal of patient data will be specified separately in the procedural manual, and the completion of each task will be reported to Principal Investigator and Study Coordinator.

(i) Patient refusal: Refusal to continue subsequent protocol treatment (follow-up continues).

(ii) Withdrawal of consent: Withdrawal of consent to participate in the study and termination of all subsequent treatment and follow-up in accordance with the study protocol. Research use of data prior to withdrawal of consent is permitted.

(iii) Full withdrawal of consent: Withdrawal of consent to participate in the study and unavailability of all data from the time of patient registration, including information at registration.

In addition, some medical institutions may request that a "withdrawal of consent" form be prepared as a written document. However, in the event of withdrawal of consent, written expressions of willingness are required to increase the psychological barriers to withdrawal of consent (i.e., it is difficult to withdraw consent), and it is considered unwanted from the viewpoint of protecting human subjects. Therefore, in JCOG, written expressions of willingness are not mandatory for withdrawal of consent, verbal withdrawal of consent is valid, and the "withdrawal of consent" form is not prepared. If "withdrawal of consent" form is required by the participating institution, it should be prepared by the institution.

- This should be used in studies conducted in accordance with the Ethical Guidelines for Medical and Health Research Involving Human Subjects.

The procedures for discontinuation of the patient's follow-up request and removal of patient data will be specified separately in the procedural manual, and the completion of each task will be reported to Study Chair and Study Coordinator.

(i) Patient refusal: Refusal to continue subsequent protocol treatment (follow-up continues).

(ii) Withdrawal of consent: Withdrawal of consent to participate in the study and termination of all subsequent treatment and follow-up in accordance with the study protocol. Research use of data prior to withdrawal of consent is permitted.

(iii) Full withdrawal of consent: Withdrawal of consent to participate in the study and unavailability of all data from the time of patient registration, including information at registration.

In addition, some medical institutions may request that a "withdrawal of consent" form be prepared as a written document. However, in the event of withdrawal of consent, written expressions of willingness are required to increase the psychological barriers to withdrawal of consent (i.e., it is difficult to withdraw consent), and it is considered unwanted from the viewpoint of protecting human subjects. Therefore, in JCOG, written expressions of willingness are not mandatory for withdrawal of consent, verbal withdrawal of consent is valid, and the "withdrawal of consent" form is not prepared. If "withdrawal of consent" form is required by the participating institution, it should be prepared by the institution.

13.3. Protection of personal Information and patient identification

JCOG recognizes that information on privacy, such as personal information and medical information, should be protected and handled carefully, based on the principle of respecting the personality of individuals, and has formulated JCOG Privacy Policy, and will take all possible measures to protect privacy. For more information, see JCOG website (http://www.JCOG.jp/).

13.3.1. Policies, legislation, and norms followed by JCOG

In conducting JCOG study, JCOG follows, in principle, JCOG Privacy Policy as well as the following laws and norms depending on the content of the research. If other laws, norms, and policies are applicable, they should be followed.

- Clinical Trials Act (Law No. 16, 2017).
- Act on the Protection of Personal Information (Law No. 57, 2003, Final Amendment: Law No. 65, September 9, 2015).
- Helsinki Declaration (Translation by the Japan Medical Association)
- Ethical Guidelines for Medical and Health Research Involving Human Subjects (No.1 of Notice of the Ministry of Education, Culture, Sports, Science and Technology and the Ministry of Health, Labour and Welfare, 2017).

13.3.2. Use of personal information objective, items to be used, and methods of use

1) Objective of use

In accordance with the basic philosophy "Providing the best treatment to more patients," JCOG uses personal information, etc. of patients for objective of "Identifying patients and conducting surveys not only during treatment but also for a long period after end of treatment in order to obtain the correct results of clinical studies, and appropriately managing the acquired information."

2) Items to be used

Information to identify individuals who will be used by JCOG as minimally require for identification and inquiry of patients is as follows.

Medical record number, date of birth, initials, and registration number, Pathology specimen number

In other words, information that can identify individuals other than those listed above, such as the patient's name, is not informed to Data Center by participating institutions, and if they are falsely informed, they should be destroyed without using the recording medium or stored after performing appropriate processing, such as masking, which is incapable of reading.

3) Method of use

Personal information of patients used by JCOG will be collected by entering the CRFs etc. by researchers at participating institutions and submitting them to Data Center either by JCOG Web Entry System, mailing, or handover as a rule. However, telephone calls will be used only for patient registration where prompt contact is necessary.

In addition, in order to confirm the accuracy of the collected information, inquiries regarding various types of CRFs, including personal information, between Data Center and researchers at medical institutions are limited to either JCOG Web Entry System, mailing, or handover. Only the more anonymous registration number should be used when interacting with e-mail inquiries, and medical record numbers and initials should not be used.

13.3.3. Preparation of records for provision of samples and information, etc. at participating institutions

The investigator at each site will prepare records for this study of enrolled patients. The matters prescribed by the Ordinance of the Ministry of Health, Labour and Welfare (Article 53 of Clinical Trials Act Enforcement Regulations) are as follows.

- Identification of clinical-research subjects
- Items related to medical care and testing for subjects personnel in clinical studies
- Items related to participation in clinical research
- Other matters necessary to conduct clinical research

13.3.4. Source documents used in clinical studies (JCOG trials conducted under Clinical Trials Act)

The source documents for clinical studies used in this study refer to all records used for diagnosis and treatment, including medical records (including worksheets, etc.), laboratory test records, diagnostic images used for diagnosis, pathological diagnosis report, images used for response evaluation, and informed consent documents, of enrolled patients in this study. These should be available for direct access for monitoring (14.1.) and audit (14.2.) conducted by Data Center, Operations Office, Certified Review Board and regulatory persons

13.3.5. Storage of samples and information

Samples and information of enrolled patients on this study shall be stored in accordance with Article 53 of Clinical Trials Act Enforcement Regulations (MHLW ordinance No. 17, 2018). The retention time of records related to this study at participating institutions and the retention time of source documents will be 5 years from the date of completion of the study. It is recommended that the records be stored for as long as possible after the expiration date.

The retention time of data collected in JCOG Data Center will be semi-permanent in view of the possibility of long-term follow-up and secondary use for other studies. In addition, as records for the provision of samples and information, the study protocols and the model informed consent forms will be stored semi-permanently in JCOG Data Center.

13.3.6. Anonymization and control of response tables

In JCOG studies, information that can clearly identify individuals, such as patients' names, is not collected, and individuals are identified using registration numbers and medical record numbers (anonymized). Correspondence tables (not always in the form of a table) of information and registration numbers that can clearly identify individuals by themselves, such as patient names, are positioned as present at each participating institution and are appropriately managed according to the policy of participating institutions so that the identification of enrolled patients can be ensured.

13.3.7. Secondary use of data

Data from this study may be used in Japan or abroad for secondary use (e.g., meta-analysis) only if approved by the relevant committee of JCOG (e.g., Protocol Review Committee, Data and Safety Monitoring Committee). However, when providing data to external organization(e.g., a meta-analysis), data are provided so that it is not possible to identify individuals.

Secondary use of data should be disclosed on JCOG website to ensure opportunities for patients to refuse using data.

13.3.8. Safety management responsibility system

JCOG Data Center establishes a privacy protection control manager and a privacy protection manager and takes various safety control measures to minimize the risks of information leakage when using personal information, etc.

13.3.9. Response to request of disclosure of patient information

In the event that JCOG is asked to disclose privacy-related information, etc., the responder shall, in principle, be a researcher (site investigator, subinvestigator) at the institution of the relevant patient.

13.3.10. Receipt of inquiries about JCOG

General inquiries and complaints about privacy policies will be received by either mail, e-mail, or fax below.

Inquiry liaison: JCOG Data Center Privacy Protective

Postal destination :〒104-0045 Tsukiji 5-1-1, Chuo-ku, Tokyo

Clinical Research Support Office, National Cancer Center Hospital

E-mail :JCOG_privacy@ml.JCOG.jp

FAX :03-3542-3374

13.4. Adherence to the protocol

Researchers participating in this study will adhere to this protocol unless they compromise patient safety and human rights.

13.5. Application to Certified Review Board and Notification of Implementation Plans

When conducting this study, the approval of Certified Review Board and the permission by the administrator of each institution to conduct the study using this protocol and written informed consent from patients must be obtained. Prior to the commencement of the study, the implementation plan ※1 shall be submitted to the Minister of Health, Labour and Welfare, and the test data shall be published to jRCT※2. Principal Investigator is responsible for applying to Certified Review Board, submitting Implementation Plans to the MHLW, and registration in jRCT, and JCOG Operations Office supports these application procedures.

※ 1 Form No.1 of the Ministerial Ordinance stipulated in Article 39, Paragraph 1 of the Ordinance for Clinical Trials Act Enforcement Regulations

※ 2 Databases (Japan Registry of Clinical Trials) prepared by MHLW as specified in Article 24, Paragraph 1 of Clinical Trials Act Enforcement Regulations https://jrct.niph.go.jp/

13.5.1. Procedures for new application

<u>1) Procedures performed by Principal Investigator</u>

<Procedures from initial submission to study initiation>

(i) Review and approval of the study protocol by JCOG Protocol Review Committee (ver.1.0.0).

(ii) Principal Investigator will prepare the following documents for this study and submit these documents to Certified Review Board for review through JCOG Operations Office.

- ➢ New Review Request Form (Unified Form No.2 for Clinical Research)
- ➢ Implementation Plan (Ministerial Ordinance Form No.1)
- ➢ Study plan (this protocol) (including response to "disease or the like")
- ➢ Informed consent form
- ➢ Documents describing summary of drugs, etc. (e.g., package inserts of drugs used as a part of protocol treatment)
- ➢ List of Subinvestigators (Unified Form No.1 for Clinical Research)
- ➢ Conflict of Interest Management Standard (Guidance Form A), Conflict of Interest Management Plan (Guidance Form E) (see 13.X.)
- ➢ Other documents to be submitted when prepared (review documents of JCOG Protocol Review Committee, sample Case Report Forms, and a draft agreement on funding with the marketing authorisation of drugs, etc. or its special associates)

(iii) Modifications such as protocols and informed consent forms are made to review opinions submitted by Certified Review Board as needed.

※ Response to the indications by Certified Review Board: Prepare modified versions with review and approval by the Director of Data Center if modification of the protocol or informed consent forms is needed (ver.1.0.1, ver.1.0.2, ver.1.0.3…). .

(iv) After Certified Review Board approval is obtained, JCOG Operations Office should have Certified Review Board approval date and the approved version number on the cover page of the protocol and informed consent form and appear on JCOG website's Protocol Download page.

(v) Principal Investigator will use Certified Review Board review results notification and the documents submitted in ② to obtain the approval of the administrator of the institution which he/she belongs to, send these documents to the site investigators of all participating institutions described in the Implementation Plan, and request that the administrators of each participating institution permit conduct the research.

(vi) JCOG Operations Office shall apply for jRCT registration under the supervision of Principal Investigator after obtaining the permission by the administrators of all participating institutions described in the Implementation Plan and confirming that the patient registration system is open. "Study progress" in the trial registration will be registered as "being recruited."

(vii) JCOG Operations Office will inform Principal Investigator that jRCT registration has been completed after submission of the registration application. Principal Investigator will output the Implementation Plan from jRCT and submit the Implementation Plan, informed consent form, and Certified Review Board review result notification to the MHLW (Local Health and Welfare Bureau, which is responsible for the location of Certified Review Board; the same below). After submission, Principal Investigator will promptly inform Certified Review Board described in the Implementation Plan. In addition, Principal Investigator shall promptly report the submission of the Implementation Plan to the administrator of the institution to which he/she belongs, and provide this information to the site investigators of all participating institutions listed in the Implementation Plan and JCOG Operations Office.

(viii) After confirming that the notification of the Implementation Plan, etc. to the Local Ministry of Health and Welfare has been accepted and that jRCT's status has been updated from "under registration application" to "registration open," Principal Investigator will inform JCOG Operations Office that it has become "registration open." JCOG Operations Office opens JCOG Data Center's patient registration system and provides trial initiation announcements to study group. The date of jRCT publication is the start date of the study. "】

JCOG Operations Office should be contacted for procedures involving the addition of participating institutions after the initial submission.

"16.X. Medical institutions" changes (adding or replacing participating institutions) correspond to changes in the content of the protocol, then the change procedure shall be performed in accordance with the "13.6.2.Procedures for

when a change in the protocol occurs after the start of the study"

2) Procedures performed by the investigators at each participating institution

Following Certified Review Board approval, the investigator at each participating institution will obtain permission to conduct the research by the administrator of the participating institution using a set of documents received from Principal Investigator (Certified Review Board review results notification and documents submitted to Certified Review Board). The site investigator shall promptly send a copy of the letter of permission of the relevant institution to the Data Center after obtaining the permission by the administrator to conduct the research.

If the study is Specified Clinical Trial under Clinical Trials Act, the site investigator who has been informed that the Implementation Plan has been submitted to the MHLW by Principal Investigator should promptly report that the Implementation Plan has been submitted to the administrator of the affiliated institution.

3) Permission to conduct research at participating institutions

The procedures for obtaining permission for conducting research from the administrator of the affiliated institution shall be in accordance with the regulations of each institution.

When a copy of the institutional approval form is sent to JCOG Data Center, either the site investigator or the site coordinator should send the copy. Original copies of the institutional approval form will be stored by the site coordinator, and copies will be stored by JCOG Data Center.

When a affiliated institution has restrictions on providing personal information, such as medical record numbers, when sending copies of the institutional approval form to JCOG Data Center, the site coordinator shall communicate that personal information cannot be provided, and also send documents, such as the corresponding table of the ID number for registration and the medical record number, which is specified in JCOG privacy policy.

In addition, the informed consent form for patients approved by Certified Review Board may not be modified except for the contact information of the institution or prespecified selection items. A common protocol will be used for all institutions, since no changes in the protocols will be permitted for each institution. If it is necessary to change the content of the protocol and informed consent form, consult with Principal Investigator and Study Coordinator if the administrator of the institution asks for modification of the protocol and the text of the informed consent form in order to make the change as the protocol and informed consent form used by all institutions.

13.5.2. Procedures for changing the study plan after the start of the study

1) Procedures performed by Principal Investigator

If any of the changes of (1), (2), or (3) below occurs in the conduct of this study from the <Procedures from the initial application to the commencement of the study> in "13.6.1. Procedures at the time of new application", Principal Investigator shall hear Certified Review Board's opinions by applying for a change to Certified Review Board.

The procedures for applying for changes to the Certified Review Board are in accordance with "13.6.1. Procedures at the time of new submission" in the procedures from initial submission to the start of the study in ②, ③, and ④, respectively. In the absence of a change in the Implementation Plan, notification to the MHLW is not necessary. In the event that a change in the Implementation Plan occurs, notification to the MHLW is required. If Principal Investigator becomes aware of the planned change in the Implementation Plan, he or she should promptly contact JCOG Operations Office. JCOG Operations Office will assist in the application procedure for change to Certified Review Board and registration in change to jRCT.

(1) When the content of the protocol or informed consent form is changed (when it is amendment or revision in 13.7.1.).

(2) When the protocol or informed consent form is not changed, but the Implementation Plan (registered in jRCT) is changed.

(3) When changing Conflict of Interest Management Standards or Conflict of Interest Management Plans

Because notification of changes in the Implementation Plan (registered in jRCT) to the MHLW except for changes in research progress must be done in advance, Principal Investigator should inform site investigators and JCOG Operations Office of the change including the replacement of the investigator or the subinvestigator at each

participating institution. In addition, inform other investigators as soon as there are any changes that need to be made known.

Principal Investigator shall promptly report to the administrator of the institution to which he/she belongs and provide information to other research investigators when informed by Certified Review Board.

Procedures when it is necessary to notify changes to the Certified Review Board and the MHLW before changes are made.

1. Changes to the Implementation Plan

If changes to any of the above (1), (2), or (3) are made to the Implementation Plan (registered in jRCT) after hearing Certified Review Board's opinions, JCOG Operations Office should enter changes to jRCT under the supervision of Principal Investigator. In addition, Principal Investigator submits the following notification to the MHLW. After the date of coming into force of the predefined changes, the study shall be conducted in accordance with the changes.

- Notification of Change in Implementation Plan (Form No.2 of the Ministerial Ordinance)
- Implementation Plan after the change (output of the change in jRCT)
- Certified Review Board Review Results Notification

※ On institution transitioning to JCOG Collaborating Institution by replacement of participating institution: For institutions where patients were not enrolled prior to transfer to collaborating institution, Principal Investigator should remove it from the participating institutions by notifying changes in the Implementation Plan.

Procedures when it is necessary to notify a change to the Certified Review Board and the MHLW after the change***

1. Change in progress

(2) Among the changes in the Implementation Plan (registered in jRCT), the change in "3. Issues related to checking the implementation of Specified Clinical Trials (2) Specified Clinical Trials progress" shall be made without delay after the change. Under the supervision of Principal Investigator, JCOG Operations Office shall change "Research Progress" in jRCT. Subsequently, Principal Investigator will promptly apply for a change to Certified Review Board. After obtaining approval from Certified Review Board, Principal Investigator will submit the following notification to the MHLW.

- Notification of Change in Implementation Plan (Form No.2 of the Ministerial Ordinance)
- Implementation Plan after the change (the content of the change in jRCT was outputted)
- Certified Review Board Review Results Notification].

2. Changes in Implementation Plan after registration of the first patient

At the time of the initial submission, the "date of registration of the first patient" in the Implementation Plan is provided in a blank space. The Implementation Plan should be changed without delay after registration in the first patient. In this instance, JCOG Operations Office enters and registers the "First Patient Registration Date" in jRCT under the supervision of Principal Investigator. Subsequently, Principal Investigator will promptly apply for a change to Certified Review Board.

- Notification of Change in Implementation Plan (Form No.2 of the Ministerial Ordinance)
- Implementation Plan after the change (output of the change in jRCT)
- Certified Review Board Review Results Notification]

3. Minor changes specified by MHLW ordinance (application for change to Certified Review Board is not required)

If the following minor changes are made to Implementation Planning and the registration of jRCT, Principal Investigator will not need to hear the opinions of Certified Review Board and will notify Certified Review Board of the changes within 10 days of the date of the change. In addition, a notification (Form No.3) shall be submitted to the MHLW.

Scope of minor changes to the Implementation Plan of Article 42 of Clinical Trials Act Enforcement Regulations

- A change in the name of a person engaged in Specified Clinical Trials that does not involve a replacement of the person engaged in Specified Clinical Trials.
- Changes due to a change in the name or address number of the area

2) Procedures performed by the investigators at each participating institution

If any changes occur in the part of the protocol that corresponds to the institution to which he/she belongs, (4) Items related to investigators in multi-institutional studies, inform Principal Investigator and JCOG Operations Office of the changes to be made prior to the changes. Depending on the content of the change, the investigator shall confirm the confirmation of institutional requirements, prepare documents for conflicts of interest and submits to Certified Review Board such as lists of subinvestigators, and inform Principal Investigator and JCOG Operations Office.

The content of the most recent Implementation Plan for the institution to which they belong (the same as the content of jRCT registration) should be checked on jRCT website (https://jrct.niph.go.jp/).

13.5.3. Review and approval of study progress and study continuation (periodic reports)

Principal Investigator will report periodic reports on the progress of the study, the occurrence of adverse events, and conflict of interest management (see 13.X.1.⑱) to the administrators of their institutions and report them to Certified Review Board. Within two months after the expiration of each year from the date of submission of the Implementation Plan to the Minister of Health, Labour and Welfare.

When the report is made to Certified Review Board, Principal Investigator shall promptly inform the investigators of other participating institutions of the fact. The investigator who receives the information shall promptly report the details of the information to the administrator of the institution to which he/she belongs.

If the study falls under Specified Clinical Trials above Clinical Trials Act, Principal Investigator will report to the MHLW regarding the implementation status of Specified Clinical Trials. Periodic reports to Certified Review Board shall be made within 1 month of the date on which the results on the appropriateness of continuation of the relevant Specified Clinical Trials are obtained. The report shall be made by submitting the attached Form 3※ to the Minister of Health, Labour and Welfare.

※ Enforcement of Clinical Trials Act Enforcement Regulations, etc. (Notification No. 0228-1 of the Sector of Economics, Ministry of Health, Labour and Welfare, Notification No. 0228-1 of the Notification No. 1 of the Evaluation and Development Division, Health Policy Bureau, Ministry of Health, Labour and Welfare, dated February 28, 2018)

13.6. Protocol revision/amendment

13.6.1. Categorization of protocol changes and procedures for changes

For a change in the protocol, the Protocol Revision Application must be submitted to Data and Safety Monitoring Committee Office prior to submission to Certified Review Board [in the case of Specified Clinical Trials, add the following: "and report to the MHLW"] (see 13.6.2).

JCOG deals with the changes in the content of the protocol after approval by the Protocol Review Committee, divided into amendment and revision. Data and Safety Monitoring Committee Office will distinguish between amendment and revision, so all of applications are submitted as revision. If it is classified as an amendment by the Secretary-General of Data and Safety Monitoring Committee, it is reviewed by Data and Safety Monitoring Committee. If it is classified as a revision, Secretary-General of Data and Safety Monitoring Committee will issue a verification form and will not be reviewed by Data and Safety Monitoring Committee. We also distinguish the addition of supplementary explanations that do not fall into a change in protocol content as "Memorandum". Definitions and handling are as follows:

When the protocol or informed consent form is revised or amended, Principal Investigator will promptly distribute the most recent protocol or informed consent form to those involved in the study. Individuals involved in the study should always conduct the study in accordance with the most recent protocol since the dates of entry into force of the amendment/revision.

1) Amendment

Partial protocol change which meets one or more of the followings: i) Potential to increase the risk of patients enrolled in the study, ii) Having substantial effects on primary endpoint of the study, iii) having essential effects on the study's implementation structure.

The amended version of the protocol and informed consent form version numbers are shown as in 2.0.0, 3.0.0, and 4.0.0….

Approval by the Group Chair and the Data Center Director must be obtained prior to submission to Data and Safety Monitoring Committee.

When classified as "amendment" by the Secretary-General of Data and Safety Monitoring Committee, review of changes by Data and Safety Monitoring Committee is performed prior to Certified Review Board review. After the protocol amendment has been approved by Data and Safety Monitoring Committee, an application for a change in the protocol will be submitted to Certified Review Board through JCOG Operations Office (at this time the version number is ver. 2.0.0, 3.0.0, 4.0.0…). When the protocol was changed based on the review opinions of Certified Review Board, the version number is ver. 2.0.1, ver.2.0.2… If the protocol is changed by review of Certified Review Board, the change will be reported to Data and Safety Monitoring Committee, but in principle, the change will not be reexamined by Data and Safety Monitoring Committee. When a protocol change is approved by Certified Review Board, the protocol cover page should include the date of approval by Data and Safety Monitoring Committee and Certified Review Board [If Specified Clinical Trials, the following is added: "and a notification of the change in the Implementation Plan to the Regional Bureau of Health and Welfare should be made"].

Following Certified Review Board approval, permission for the contents of the amendment by the administrator of each institution shall be obtained. If permission is obtained, the site coordinator of each institution will send copies of the permission notice by the administrator of each institution to the Data Center. After the permission of the administrator of all institutions is obtained, the protocol changes will come into effect (during this time, the patient registration will not be suspended unless there is a special need). The actual date of entry into force will be announced by JCOG Operations Office. Researchers in all participating institutions conduct the study according to revisions approved by Certified Review Board since the date of entry into force.

Treatment and assessment of enrolled patients will be performed according to the pre-change version protocol until entry into force. Protocol deviations to enhance patient safety during treatment will be permitted if pre-change protocols, such as inadequate treatment modification criteria, threaten patient safety. If protocol deviations occur, they should be listed in the monitoring report.

2) Revision

Protocol change which meets all of the followings: i) does not have an increased risk for patients enrolled in the study; ii) does not have a substantial effect on primary endpoint of the study; iii) does not have an inherent effect on the system in which the study is conducted. Includes changes in protocols due to mistakes or changes in institution-specific information, changes in institution-specific information without changes in protocols (changes in Implementation Plans and jRCT registrations), and changes in conflicts of interest at individual institutions. In principle, suspension of patient registration is not performed in case of revision.

The revised version of the Protocol and Informed Consent Form version numbers are shown as in 1.1.0, 1.2.0, and 1.3.0….

Approval by the Group Chair and the Data Center Director must be obtained prior to submission to Data and Safety Monitoring Committee.

If classified as "Revision" by the Secretary-General of Data and Safety Monitoring Committee, the Secretary-General of Data and Safety Monitoring Committee issued a verification form, Data and Safety Monitoring Committee did not review the changes. The Principal Investigator submit an application for a change in the protocol to Certified Review Board through JCOG Operations Office (at this time, the version number is ver.1.1.0, 1.2.0, 1.3.0…). If the protocol was changed based on the indication by the Certified Review Board, version number shall be as ver.1.1.1, ver.1.1.2… When a protocol change is approved by Certified Review Board, the date of approval by the Certified Review Board should be entered on the protocol cover page. [If Specified Clinical Trials, the followings are added: "and a notification of the change in the Implementation Plan to the Regional Bureau of Health

and Welfare should be made"]

The date of entry into force of the protocol change will be 2 weeks after Certified Review Board approval date unless otherwise specified. [in the case of a Specified Clinical Trials, the followings are added: "The effective date shall be the date after the notification of change of the Implementation Plan to the Regional Bureau of Health and Welfare"]

The actual date of entry into force will be announced by JCOG Operations Office. Researchers in all participating institutions conduct the study according to revisions approved by Certified Review Board since the date of entry into force.

At that time, the site investigator should obtain permission by the administrator of each institution after the date of approval of Certified Review Board and before the date of entry into force. In this case, the protocol revision may be permitted with a report to the administrator of the institution. However, the procedures for obtaining permission from the administrator shall be in accordance with the regulations of each institution. For protocol revisions, confirm with the institution prior to initiation of the study to ensure that permission is obtained within the aforementioned time periods, and contact JCOG Operations Office if this is difficult. Reports to the administrator and approval form by the administrator at each institution need not be sent to the Data Center, but the original copy will be retained by the site coordinator as it will be checked during site visit audits.

Treatment and assessment of enrolled patients will be performed according to the pre-change version protocol until entry into force. Protocol deviations to enhance patient safety during treatment will be permitted if pre-change protocols, such as inadequate treatment modification criteria, threaten patient safety. If protocol deviations occur, they should be listed in the monitoring report.

3) Memorandum

Supplementary description of the protocol distributed from Principal Investigator/Study Coordinator to study personnel in objective, such as reduction of interpretive variation in sentences, and special precautions, rather than change of protocol content. Any form is used.

Approval by Group Chair and the Director of the Data Center is needed prior to distribution. Reporting to Data and Safety Monitoring Committee before distribution or immediately after distribution is required.

It is not necessary to include the protocol on the cover page.

13.6.2. Patient explanation and re-consent at the time of protocol amendment/revision

In the event of a change in the content of the study, the investigator or the subinvestigator shall provide appropriate explanation to the enrolled patients (regarding protocols based on revision, treatment, follow-up, etc.). In addition, if Certified Review Board comments that re-consent of enrolled patients in writing is required, informed consent should be obtained in writing.

13.7. Conflicts of Interest (COIs) involved in this study

13.7.1. COI management involved in this study

The COIs involved in this study will be managed according to the "Guidance for Conflict of Interest Management in Clinical Trials Act (Notification No.1130-17 of the PMSB dated November 30, 2018) ※ (Guidance) of the Division, Research and Development, Ministry of Health, Labour and Welfare, in accordance with the following:

※ http://www.mhlw.go.jp/stf/seisakunitsuite/bunya/0000163417.html

The format used for COI control should be the latest version of the guidance.

- Conflict of Interest Management Standards: Form A
- Reports of related companies, etc.: Form B
- Investigator Conflict of Interest Self-Report Form: Form C
- Conflict of Interest Confirmation Report: Form D
- Conflict of Interest Management Plan; Form E

1) Procedures for New Application

Conflict of Interest Management Standards

(i) All JCOG trials according to Clinical Trials Act will employ Conflict of Interest Management Standards (Form A) in accordance with guidance.

Request for Confirmation of Conflicts of Interest

(ii) At the time of initiation of the primary review of the protocol, JCOG Operations Office shall identify companies, etc. related to this study that require conflict of interest management based on information on drugs and medical devices specified in the Protocol Treatment (see 6.1.), enter the relationship with the company in Form B, and ask Principal Investigator to confirm the accuracy of the entry.

(iii) After checking the entries in Form B received from JCOG Operations Office, Principal Investigator will send a format to the site investigator and site coordinator at each participating institution and ask to confirm the entries in Form B, create Form C, and create Form E. In addition, a form shall be sent from Principal Investigator to those who correspond to the "person responsible for statistical analysis" and the "administrator other than Principal Investigator and Investigators" of the Implementation Plan, and they shall be asked to prepare Forms C and E.

Confirmation of Conflicts of Interest (participating institutions)

(iv) The site investigator or site coordinator at each participating institution will register the investigators, subinvestigators, and research associates involved in the study in JCOG Web Entry System. Investigators and subinvestigators registered in JCOG Web Entry System will be the reporters of conflicts of interest in the study.

(v) The site coordinator will request the investigator of the study to confirm the description of Form B received from Principal Investigator, create Form C, and create Form E. The investigator will then summarize conflict of interest management within the study site.

(vi) The investigator checks the forms received from Principal Investigator for any relevant items in Q2 through Q5 of Form B and notifies Principal Investigator through JCOG Operations Office within a week if any.

(vii) In the column of Form C [Persons requiring a Form C self-declaration of conflict of interest (Form C)] ※, the site investigator inputs the information of the investigator and subinvestigator registered in JCOG Web Entry System, and asks subinvestigator to prepare Form C.

※ The information entered in Form C is automatically entered in Form E. This entry should be consistent with the physician's information contained in the "List of Subinvestigators" (Clinical Trials Act Uniform Form No.1) submitted to Certified Review Board with Form E, and physicians who do not agree may not be involved in this study. Therefore, when entering the information in the ※ column for [those who require a Conflict of Interest Self-Reporting Form (Form C)], the investigator or subinvestigator list should be downloaded in JCOG Web Entry System and the physician listed in the Study Subinvestigator list should be entered without missing the person who will be the investigator and the subinvestigator in the study. If the information in the downloaded "Study Subinvestigator List" is not consistent with the physician involved in the study, the registered information should be updated in JCOG Web Entry System, and the "Study Subinvestigator List" with the most recent information reflected should be downloaded and used.

(viii) The investigator and the subinvestigator shall complete the necessary information regarding the relationship with the company, etc. described in advance in Form C, and submit Form C to the Conflict of Interest Confirmation Department of the institution to which he/she belongs. In doing so, the investigator also submits Form A.

(ix) The investigator receives the results of confirmation of conflicts of interest (Form D) of the investigator and the study subinvestigator from the institution to which he/she belongs.

(x) The investigator will confirm the content of Form A, Form B, and Form D of the investigator and all study subinvestigators to create Form E. The investigator will notify Principal Investigator through JCOG Operations Office of Form E and the Study Subinvestigator List downloaded in Form 7.

Confirmation of Conflicts of Interest (outside participating institutions)

(xi) The person responsible for statistical analysis and the person overseeing a study other than Principal Investigator or investigator shall complete, in the form received from Principal Investigator, the necessary information regarding the relationship with the company, etc. described in advance in Form C, and submit Form A and Form C to the Conflict of Interest Confirmation Department of the Affiliated Institution.

(xii) The person responsible for statistical analysis and the person overseeing studies other than Principal Investigator or investigator shall receive confirmation results (Form D) from the institution.

(xiii) The person responsible for statistical analysis and the person overseeing studies other than Principal Investigator and investigator shall confirm the content of Form A and Form D, prepare Form E, and notify Form E to Principal Investigator through JCOG Operations Office.

Description of Conflicts of Interest in the protocol and informed consent form

(xiv) Principal Investigator and JCOG Operations Office will review Form A and Form E received from participating institutions and, as appropriate, will accurately describe the conflicts of interest (study COIs) between the study and the drug marketing authorisation holder, etc. in the protocol and informed consent form. Conflicts of interest (personal COI) between the reporters of conflicts of interest in this study and the marketing authorisation of drugs, etc. (personal COI) can change over time, so the personal COI is not described in the protocol and informed consent form, and the information is updated on JCOG website as needed.

Certified Review Board review

(xv) Principal Investigator compiles and submits the Form E and Study Subinvestigator List of all institutions to Certified Review Board for review.

2) Procedures for new involvement with companies after the start of the study

(xvi) When a new involvement with companies (research COI) occurs in the study after the start of the study

a. In the event of any change in the enterprises involved in this study, etc. to be described in Q1 of Form B, repeat the procedures ② to ⑭. However, the site investigator and site coordinator roles in ④, ⑤ shall be assumed by the investigator at each institution. If a new study COI needs to be added to the protocol and informed consent form, a protocol revision (see 13.X.X.) should be submitted to Certified Review Board for review.

b. If a change occurs from Q2 to Q5 in Form B, the investigator at the participating institution affected by the change will change the appropriate description in Form B and update Form E and send it to Principal Investigator and JCOG Operations Office. A Principal Investigator who has received a Form E will revise the protocol as needed (see 13.X.X.) and submitted to Certified Review Board for review.

(xvii) In the event that a conflict of interest reporter is newly involved with a company (personal COI) after the start of the study

Conflict of interest reporters repeat procedures from ⑧ to ⑩ or from ⑪ to ⑬. However, if there is no change in Form E, these procedures will be performed at each institution but will not be sent to Principal Investigator and JCOG Operations Office. Principal Investigator will submit Post-change Form E sent from the site investigator to Certified Review Board for review. Personal COIs are also disclosed on JCOG website.

3) Procedures for periodic reporting

(xviii) Principal Investigator checks for changes in study COI and personal COI annually at the timing of periodic reporting and reports to Certified Review Board.

13.7.2. COI with companies involved in this study(study COI)

There are no conflicts of interest to disclose about relationship between the company manufacturing and marketing drugs specified as a part of protocol treatments in this study (See 6.1).

13.7.3. COIs of JCOG Committee members and JCOG Data Center/Operations Office staff

COIs of the committee members of JCOG committees, the staffs of the office of committees, and JCOG Data Center/Operations Office staff involved in the study will be managed by JCOG Conflict of Interest Committee in accordance with Clinical Trials Act control standards.

13.8. Compensation

In order to comply with Clinical Trials Act, this study must take necessary measures such as sing up for insurance and ensuring a system to provide medical care in order to compensate for the health damage caused by the study

and provide medical care.

Therefore, regarding the health damage caused by participating in this study, appropriate treatment according to the condition is provided similarly to the usual insurance medical care. In addition, this study will be covered by clinical study insurance, and the following will be compensated based on insurance conditions, and this will be explained to patients and understood.

13.9. Intellectual property

The results, data, and intellectual property rights obtained from this study are attributed to the followings: National Cancer Center, Principal Investigator, Study Coordinator, and Group Chair. Specific procedures and allocation must be determined through consultation among four parties. Whether the intellectual property related to Principal Investigator, Study Coordinator, Group Chair will belong to the individuals or the affiliated institution will be determined according to the agreements of the affiliated institution.

13.10. Disclosure of information on this study

Summary, progress, and main results of this study will be published on JCOG website (www.jcog.jp) and on jRCT (https://jrct.niph.go.jp/).

14. Monitoring and audit

14.1. Periodic monitoring

In this study, monitoring is performed in order to ensure that the study is conducted safely and in accordance with this protocol and that data are collected accurately. Periodic monitoring should be performed twice a year in principle, using the entered data on the CRFs collected in the Data Center. Specific procedures for periodic monitoring are provided separately in the Monitoring Plan.

The Data Center submits a "Monitoring Report" summarizing the results of central monitoring to Principal Investigator, Study Coordinator and investigators. Together, they are submitted to Group Chair, Data and Safety Monitoring Committee, and JCOG Chair.

The Monitoring Report is a material for periodic reports to be made every year from the date of submission of the Implementation Plan.

14.1.1. Monitoring items

① Registration status: number of registration - cumulative/by month, by arm/site
② Eligibility: ineligibles/potentially ineligible cases: by arm/site
③ Pre-treatment baseline factors: by arm
④ On/off-treatment, reason for treatment termination: arm/site
⑤ Protocol deviation: arm/site
⑥ Serious Adverse Events: arm/site
⑦ Adverse reaction/event: arm
⑧ Overall survival, progression-free survival (or relapse-free survival, etc.): all registered patients
⑨ Other issues related to study progress and safety (studies in accordance with Clinical Trials Act: status of occurrence of non-compliance and subsequent response, number of subjects for compensation, number of disease or the like reports in accordance with Article 13 of Clinical Trials Act)

14.1.2. Eigibility (Eligible/Ineligible)

For all registered patients, eligibility will be classified according to the following definitions as either: In monitoring, Data Center lists potentially ineligible cases in the "Evaluation of Eligibility" section of the monitoring report, and after review by Study Coordinator, confirms them to be either 1), 2), 9), or 99) with Group Chair approval prior to performing primary analysis.

Only 1) eligible shall be "eligible case", 2) post hoc ineligible,9) de facto ineligible and 99) violation of registration shall be "ineligible case". This is a category established from the perspective of analysis set setting.

In the study in accordance with Clinical Trials Act, "99) violation of registration" will be regarded as "major non-compliance" in Clinical Trials Act, and Principal Investigator will promptly report the situation to Certified Review Board as soon as possible. See 14.3. for management of non-compliance.

9) de facto ineligibles corresponds to "non-compliance" on Clinical Trials Act and is reported to the administrator of participating medical organizations with the submission of the monitoring report on which they were described (twice a year).

2) Post hoc ineligibles is not treated as "non-compliance" on Clinical Trials Act because it does not correspond to non-compliance with the study protocol, as discussed below.

1) Eligible

All information generated prior to registration meets all o the Patients Selection Criteria according to the methods and criteria specified in the study protocol.

2) Post hoc ineligible

The information generated after registration does not meet either Patients Selection Criteria, or the information generated prior to registration does not meet either Patients Selection Criteria by methods or criteria other than those specified in the protocol.

Examples)

(i) In the study for Stage II-III, bone scintigraphy performed immediately after registration revealed bone

metastases, and the patient was diagnosed as Stage IV. The protocol treatment was terminated.

(ii) In the study for early gastric cancer, bloody stools is seen after registration, and colonoscopy revealed advanced colorectal cancer (synchronous double cancer). Colectomy was performed after termination of the protocol treatment.

(iii) In the study for gastric cancer (adenocarcinoma), the institution's pathological diagnosis was changed to malignant lymphoma after registration.

9) De facto ineligible

Information generated prior to registration according to protocol-specified methods (performed in all cases) and criteria does not meet either Patients Selection Criteria. This includes cases where it is determined after registration that the information that occurred before registration had been incorrect.

Example: When the supervising physician reviews the CT images performed before registration as specified, there is obvious liver metastasis (if it is a mistake by the attending physician and it is considered that there is no future).

99) Violation of registration

Deliberately (falsely) enroll while knowing that Patients Selection Criteria is not met. Corresponds to a misconduct and treats it as a serious problem.

14.1.3. Protocol Deviations/Violations

Protocol deviations are defined as those in which treatment, such as drug administration, radiotherapy, or surgical resection, as well as laboratory tests and evaluation of toxicity and efficacy, etc. were not performed according to the protocols.

In monitoring, deviations that exceed a certain acceptable range limit for each study decided by the Data Center and Principal Investigator/Study Coordinator prior to or after the initiation of the study are listed in the monitoring report as "possible deviations" and are classified into one of the following categories after consideration by Study Coordinator and study groups: Except for those described in the protocol and monitoring report, the acceptable deviations agreed between the Data Center and Principal Investigator/Study Coordinator may be changed through periodic monitoring during the study, so they should be described in the supplemental material rather than in the text of the Monitoring Plan, and the Monitoring Plan should be described as "defining the acceptable ranges separately".

1) Violation

Any deviation from the protocol that is clinically inappropriate and caused by the treating physician/institution and that meets two or more following criteria shall be classified as a violation. [When conducted in accordance with Clinical Trials Act, the following shall be added. [In the study according to Clinical Trials Act, the violation shall be treated as "major non-compliance" and Principal Investigator should report the situation to Certified Review Board as soon as possible].

① Have a substantial impact on the assessment of study endpoints

② Intentional or systematic

③ Dangerous or remarkable deviation

For "violations", in principle, the content of each violation should be described in a paper when publishing.

2) Deviation

Deviations that do not fall into 1) violation or 3) acceptable deviation. If same kind of deviations are frequent, they should preferably be included in the publication of the article. They are classified as either of the following at the time of monitoring report review:

Because deviations correspond to "non-compliance" in Clinical Trials Act, they are reported (biannually) to the administrators of the institution with the submission of the monitoring report. Not to be "major non-compliance".

(i) Deviations - Undesirable and to be reduced

(ii) Deviations (unavoidable) - things that are not proactively reducing (e.g. delay by the New Year period, equipment breakdown, etc.)

(iii) Deviations (clinically relevant) - Those in which the decision of the treating physician/institution are positively affirmed (if a similar situation again arises similar deviations are considered desirable).

※ Deviations do not always mean that the treating physician at the institution is problematic. Since patient safety is a primary priority in clinical trials, deviations should rather be made by the medical judgment of the treating physician if the condition of the individual patient are considered to be dangerous when following the protocols. If the deviation is judged to be clinically relevant for the safety of the patient, it is recorded as ③ Deviation (clinically relevant). Clinically relevant deviations in a small number of patients are not required to be particularly problematic; however, protocol revision should be considered when multiple deviations occur because protocol specification is likely to be inadequate. However, deviations that are not intended to be safety (e.g., increased doses of anticancer drugs in the hope of increasing efficacy, shortened treatment periods not specified in the protocol) are not considered clinically relevant deviations.

3) Acceptable deviation

Deviations from protocols within acceptable range agreed by the entire JCOG, study groups, or Study Chair/Study Coordinator and Data Center, pre- or post-study initiation, on a trial-by-trial basis. When conducted according to Clinical Trials Act, the following are added. Not considered to be "non-compliance" in Clinical Trials Act. Deviations within the pre-specified acceptable ranges are not included in the monitoring report.

14.2. Site visit audits

In this study, site visit audits will be conducted to ensure the reliability of clinical research and the reliability of data and information collected by clinical research from the perspective of protecting human subjects in clinical research.

Site visit audits are conducted by auditors appointed by Principal Investigator by visiting a medical institution to confirm the approval documents of the medical institution, check the list of subinvestigators in the research institution, confirm the informed consent documents, and verify CRF entry data with medical records (direct access to source documents). Specific procedures for site visit audits are provided separately in the operating procedures.

The auditor shall report the audit report summarizing the audit results to Principal Investigator/Study Coordinator, site investigators. Together, the report will be submitted to Group Chair, Director of JCOG Data Center, Director of JCOG Operations Office, and JCOG Chair. Reports should also be submitted to the site investigators of the relevant groups and JCOG Executive Committee as appropriate.

14.2.1. Items to be audited

In the site visit audits, the following items are checked by direct access to source documents:

<Confirmation Items by Study>

- Approval documents (including initial approval forms, revision approval forms, and annual report approvals/reports) from administrators of medical institutions
- Management status of the protocol
- Contents of explanatory documents and informed consent forms

<Confirmation Items by Patient>

- Patient consent (presence or absence of consent form, signature, and date of consent)
- Implementation of pre-registration mandatory tests, eligibility for registration (inclusion criteria and exclusion criteria)
- Accuracy of reported data
  - Pre-treatment evaluation, course of treatment (protocol treatment)
  - Various test results (including diagnostic imaging reports and pathology reports)
  - Accuracy of test date, response evaluation, adverse event, survival or death information, and other reported data

<Other items>

- Presence or absence of study misconduct (possibility of false reporting, fabrication, or falsification)
- Improvement status of the items pointed out in the previous audit

14.2.2. Reporting of major non-compliance found in audits to Certified Review Board

Principal Investigator/Study Coordinator should report to Certified Review Board immediately when finding possible "major non-compliance (See 14.3.2.)" as a result of site visit audits.

14.3. Management of non-compliance

14.3.1. Non-compliance.

Non-compliance in Clinical Trials Act refers to the condition in which clinical research is not compliant with Clinical Trials Act Enforcement Regulations or study protocols. In the Clinical Trials Act Enforcement Regulations, etc. (February 28, 2018), non-compliance with regulations, study protocols, operation procedures, etc., and fabrication of falsification of research data, etc. are listed as examples.

If the site investigator knows that there is non-compliance, the site investigator should report to the administrator of the medical institution and inform Principal Investigator/Study Coordinator.

If site investigator find non-compliance (regardless of major non-compliance or not) prior to the implementation of central monitoring or site visit audits, the site investigator will promptly report to Principal Investigator/Study Coordinator and JCOG Data Center.

"14.1.3.2) Deviations" correspond to "non-compliance" in Clinical Trials Act. As described in 14.1.3.2), these deviations are reported by submitting monitoring reports or their excerpts or summaries issued twice a year to the administrators of the medical institution.

Changes in protocols and implementation plans associated with investigator transfer require Certified Review Board review and notification of implementation plans to the MHLW, therefore, require a certain time to complete the sequence of procedures. In addition, it is often difficult to complete the change procedure before the transfer because the transfer may not be open until just before the transfer. Therefore, even if the site investigator is absent for a certain period of time due to the transfer, the study does not fall into "non-compliance" in this study if the research management system is maintained by the subinvestigator and the medical care system of the enrolled patients who are surviving is ensured.

14.3.2. Major non-compliance

"Major non-compliance" is that affect the human rights and safety of subjects of clinical research, the study progress and the reliability of study results. Examples of "major non-compliance" in JCOG study are provided in. If major non-compliance is likely, Principal Investigator/Study Coordinator will report the situation to Certified Review Board immediately.

1) Major non-compliance with respect to eligibility

Violation of registration

- Enrolled intentionally (falsely) while knowing that eligibility criteria was not met
- Patient enrollment was performed without necessary informed consent, and protocol treatment was performed
- The source documents for the determination of eligibility cannot be identified (including the loss of the consent form).

2) Protocol violation

Violations that affect the increased risk of enrolled patients or that affect the reliability of the study results

- Significant violation in inclusion criteria or exclusion criteria
- Violation threatening patient safety in off-treatment criteria
- Serious violation of prohibited concomitant drug, prohibited concomitant treatment, etc.
- e.g. intentional or systematic non-compliance with protocol regulations

3) Other major non-compliance

- Study was conducted prior to Certified Review Board approval or prior to approval of site administrator
- The study was continued without providing information to the enrolled patient that could affect the willingness to continue to participate the study.
- Those judged to be research misconduct (fabrication or falsification of data, etc.)

- Any leakage of personal information or violation of human rights that may have a significant impact on the enrolled patient.

15. Special Instructions

15.1 Central pathology diagnosis and related matters

15.1.1 Central pathological diagnosis

In this study, the pathological tissues of enrolled patients will be collected and the central pathological diagnosis will be determined after the fact. Since the central pathological diagnosis is not performed in real time for each individual patient enrollment, the main analysis target and the decision on treatment strategy will be based solely on the pathological diagnosis at the institution. The details of the central pathological diagnosis procedure will be specified separately in the Central Pathological Diagnosis Procedure Manual.

Timing: Once a year throughout the study period.

Subjects: All registered patients

Methods: Pathological specimens (or duplicates made from the same paraffin block) used for eligibility criteria determination at the registered facilities will be collected, and after necessary staining (chromogranin A, synaptophysin) is added, pathological eligibility will be re-determined by two or more pathology judges (16.8 Pathology judges) appointed by the group representative. (16.8 Central Pathological Review Comittie).

Management of the collected specimens: The Hepatobiliary and Pancreatic Group Study Office will be responsible for the management of the collected specimens.

Staining: If additional staining is required, it will be performed by the Central Pathology Coordinator (16.7).

Notification of the central judgment to each facility:

After the results of the central pathological diagnosis are fixed, the research secretariat will notify the results of the judgment to the registered facility (facility coordinator) of each patient. At that time, the rationale for the decision should be attached in writing. If there is a difference between the institutional diagnosis and the central diagnosis, the facility principal investigator/facility coordinator reports the decision results to the facility pathologist and discusses it with the facility pathologist, and carefully decides on the final pathological diagnosis at the facility (whether to change the diagnosis or not) and what to do if the patient is under treatment (whether to change the treatment or not).

15.1.2 Providing information to the institutional pathologist

In view of the high level of difficulty in NEC pathological diagnosis and the unique nature of this study, which covers multiple target organs, the following information will be provided to institutional pathologists.

(1) Pre-registration pathological diagnosis consultation

Since it is anticipated that there will be cases of confusion in diagnosis at facilities, pre-enrollment pathology consultation will be available as needed, with the pathology judgment committee members of this study serving as consultants. Details of the consultation operation are described in the Central Pathology Procedures.

(2) Holding of pathology-related meetings

In this study, the research secretariat and the pathology secretariat first held a pathology-related meeting (attended by pathologists from participating institutions) on February 11, 2014, during which a lecture was given to form a consensus on diagnostic criteria according to the WHO 2010 classification. The slides used at the meeting were revised as necessary based on the discussion at the meeting and posted on the JCOG website for reference by pathologists at each facility.

15.1.3 Response to cases in which pathological diagnosis was performed at a facility other than the home facility

In any of the following cases, be sure to ask a pathologist at your own institution to make the diagnosis, and confirm that the diagnosis at your institution is also NEC before enrolling in this study.

(1) When borrowing only stained tissue specimens from a previous physician for registration

Register after obtaining permission from the attending physician to the previous physician to submit the borrowed tissue specimen to JCOG 1213 for central diagnosis (loan again) and to borrow the specimen for a long period of time (up to about 1 year).

(2) If you have received virtual slides of "all tissue specimens for which a pathological diagnosis has been made" from

your previous doctor

The patient will be registered after obtaining permission from the attending physician of the previous physician to submit the virtual slides provided by the previous physician for the central diagnosis of this study.

(3) If you have received a "borrowed" pre-stained tissue specimen and an "offered" unstained preparative from your previous physician

The borrowed stained tissue specimens from the previous physician can be promptly reviewed at the patient's own institution, but the provided unstained preparations require time for pathological diagnosis after immunostaining at the patient's own institution, which may cause a time lag. There have been cases in which patients were enrolled in the study based only on the results of pathological review of borrowed tissue specimens at their own institutions, and later the results of staining and pathological diagnosis of undyed preparations at their own institutions overturned the pathological diagnosis (diagnosis of a different disease was made), resulting in post-hoc ineligible cases. Therefore, we will consider the timing of registration after sufficient consultation with pathologists at our own institution to avoid the occurrence of posterior ineligible cases as much as possible. However, if there is enough time, it is preferable to enroll unstained preparations into the study after staining and pathological diagnosis is obtained at your own institution.

15.2. JCOG BioBank Japan (BBJ) Biorepository

This study will participate in the banking of blood samples (DNA/plasma) in JCOG BBJ Biorepository based on a common protocol for all JCOG studies (hereafter referred to as common banking).

Subjects:

Among patients who agreed to participate in this study, patients whose consent to shared banking was obtained.

Sample:

1) Whole blood

Blood sampling is performed before the start of the protocol treatment in this study in principle. However, blood sampling after initiation of protocol treatment is allowed. Blood samples of 7 mL×2 (total 14 mL) of venous blood are collected using a blood collection tube (for blood counting) with a EDTA Na dedicated to the shared banking of JCOG-BBJ Biorepository, and stored at 4°C at the respective institutions until they are handed over to the sample transport/processing company (See "JCOG-Biobank Japan Biorepository protocol" for details).

2) Pathological specimens

Archived pathological tissues in daily clinical practice such as surgery, biopsy and laboratory tests can also be used in future translational researches, but the type of pathology specimen, preparation method and tissue quantity required by the studies are varied and there is no consensus that it is efficient to bank pathological tissues in a certain way prospectively. In addition, there is the opinion that the sample deteriorates (DNA fragmentation) when the thin-section sample from pathological tissue is stored for a long time.

Consent on the use of archival pathological tissue after medical care should therefore be obtained at the time of consent to banking, but actual collection should be initiated on an individual basis by creating a protocol and defining the most appropriate procedure for the study content in the protocol.

16. Organization

Changes to this chapter are considered to be revision rather than amendment.

16.1. Main study fund (funding source) of this study.

Practical Research for Innovative Cancer Control from Japan Agency for Medical Research and Development “Establishment of standard treatments for neuroendocrine carcinoma of the digestive system” JP15ck0106138, JP16ck0106138, JP17ck0106355, JP18ck0106355, JP19ck0106355, JP20ck0106618

National Cancer Center Research and Development Fund (23-A-22, 26-A-4, 29-A-3, 2020-J-3)
“Scientific research on multi-institutional trials to establish new standard treatment of solid tumors in adults”

16.2. Japan Clinical Oncology Group (JCOG)

JCOG is a multi-institutional clinical research group consisting of research teams funded by public research grants

mainly on National Cancer Center Research and Development Fund and Japan Agency for Medical Research and Development research costs that receive direct support for research by the Clinical Research Support Office of the National Cancer Center Hospital in accordance with JCOG Policy (http://www.jcog.jp/).

This study is conducted using JCOG research organisations and in accordance with the regulations set out by JCOG Executive Committee.

16.3. JCOG Chair

Yuichiro Ohe National Cancer Center Hospital

16.4. Study group and Group Chair

JCOG Hepatobiliary and Pancreatic Oncology Group

Group Chair: Junji FURUSE

Department of Medical Oncology, Kyorin University Faculty of Medicine
〒181-8611 6-20-2, Shinkawa Mitaka-shi, Tokyo
TEL:+81-422-47-5511
FAX:+81-422-44-1858
E-mail: jfuruse@ks.kyorin-u.ac.jp

Group Secretary: Makoto Ueno

Division of Hepatobiliary and Pancreatic Medical Oncology, Kanagawa Cancer Center
〒241-8515, 2-3-2 Nakao, Asahi-Ku, Yokohama
TEL:+81-45-520-2222
FAX:+81-45-520-2202
E-mail: makoto23u@gmail.com

JCOG Stomach Cancer Study Group:

Group Chair: Masanori Terashima

Shizuoka Cancer Center
〒411-8777 1007 Shimonagakubo, Nagaizumi-cho, Sunto-gun, Shizuoka Prefecture
TEL:+81-55-989-5222
FAX:+81-55-989-5783
E-mail: m.terashima@scchr.jp

Group Secretary: Takaki Yoshikawa

National Cancer Center Hospital
〒104-0045 5-1-1 Tsukiji, Chuo-ku, Tokyo
TEL:+81-3-3542-2511
FAX:+81-3-3542-3815
E-mail: tayoshik@ncc.go.jp

Group Secretary: Narikazu Boku

National Cancer Center Hospital
〒104-0045 5-1-1 Tsukiji, Chuo-ku, Tokyo
TEL:+81-3-3542-2511
FAX:+81-3-3542-3815
E-mail: nboku@ncc.go.jp

JCOG Japan Esophageal Oncology Group

Group Chair: Yuko Kitagawa

Keio University Hospital
〒160-8582 35 Shinanomachi, Shinjuku-ku, Tokyo
TEL:+81-3-3353-1211
FAX:+81-3-3355-4707
E-mail: kitagawa@a3.keio.jp

Group Secretary: Ken Kato
National Cancer Center Hospital
〒104-0045 5-1-1 Tsukiji, Chuo-ku, Tokyo
TEL:+81-3-3542-2511
FAX:+81-3-3542-3815
E-mail: kenkato@ncc.go.jp

16.5. Study Chair (Principal Investigator)

JCOG Hepatobiliary and Pancreatic Oncology Group
Takuji Okusaka
National Cancer Center Hospital
〒104-0045 5-1-1 Tsukiji, Chuo-ku, Tokyo
TEL: +81-3-3542-2511
FAX: +81-3-3542-3815
E-mail: tokusaka@ncc.go.jp

JCOG Stomach Cancer Study Group:
Narikazu Boku
National Cancer Center Hospital
〒104-0045 5-1-1 Tsukiji, Chuo-ku, Tokyo
TEL: +81-3-3542-2511
FAX: +81-3-3542-3815
E-mail:nboku@ncc.go.jp

JCOG Japan Esophageal Oncology Group
Ken Kato
National Cancer Center Hospital
〒104-0045 5-1-1 Tsukiji, Chuo-ku, Tokyo
TEL: +81-3-3542-2511
FAX: +81-3-3542-3815
E-mail: kenkato@ncc.go.jp

16.6. Study Coordinator

JCOG Hepatobiliary and Pancreatic Oncology Group
Chigusa Morizane
National Cancer Center Hospital
〒104-0045 5-1-1 Tsukiji, Chuo-ku, Tokyo
TEL: +81-3-3542-2511
FAX: +81-3-3542-3815
E-mail: cmorizan@ncc.go.jp

JCOG Stomach Cancer Study Group:
Nozomu Machida
Kanagawa Cancer Center
〒241-8515, 2-3-2 Nakao, Asahi-Ku, Yokohama
TEL:+81-45-520-2222
FAX:+81-45-520-2202
E-mail:n-machida@kcch.jp

JCOG Japan Esophageal Oncology Group

Ken Kato
National Cancer Center Hospital
〒104-0045 5-1-1 Tsukiji, Chuo-ku, Tokyo
TEL: +81-3-3542-2511
FAX: +81-3-3542-3815
E-mail: kenkato@ncc.go.jp
Yoshitaka Honma
National Cancer Center Hospital
〒104-0045 5-1-1 Tsukiji, Chuo-ku, Tokyo
TEL: +81-3-3542-2511
FAX: +81-3-3542-3815
E-mail:yohonma@ncc.go.jp

16.7. Central Pathological Review Coordinator

Nobuyoshi Hiraoka
National Cancer Center Hospital
〒104-0045 5-1-1 Tsukiji, Chuo-ku, Tokyo
TEL: +81-3-3542-2511
FAX: +81-3-3542-3815
E-mail: nhiraoka@ncc.go.jp
Shigeki Sekine
National Cancer Center
〒104-0045 5-1-1 Tsukiji, Chuo-ku, Tokyo
TEL: +81-3-3542-2511
FAX: +81-3-3542-3815
E-mail: ssekine@ncc.go.jp

16.8. Central Pathological Review Coordinator

Nobuyoshi Hiraoka (National Cancer Center)
Noriyoshi Fukushima (Jichi Medical University)
Nobuyuki Ohike (Showa University Fujigaoka Hospital)
Ryoji Kushima (Shiga University of Medical Science)
Mitsuya Iwafuchi (Scool of Health Sciences Faculty of Medicine, Niigata University)
Tetsuo Ushiku (The University of Tokyo)

16.9. Participating sites (participating institutions)

HBP	SCSG	JEOG	Participating institutions
○			Sapporo-Kosei General Hospital
○		○	Hokkaido University Hospital
	○		Keiyukai Sapporo Hospital
	○	○	Iwate Medical University
		○	Tohoku University Hospital
	○		Miyagi Cancer Center
○	○	○	Tochigi Cancer Center
○			Jichi Medical University
○	○	○	Saitama Cancer Center
○	○	○	National Cancer Center Hospital East
○		○	Chiba Cancer Center
○		○	Chiba University, Graduate School of Medicine
○	○	○	National Cancer Center Hospital
○			Kyorin University Faculty of Medicine
○			National Center for Global Health and Medicine (NCGM)
○	○	○	Cancer Institute Hospital of Japanese Foundation for Cancer Research
	○	○	Toranomon Hospital
○			Teikyo University School of Medicine
○			St.Marianna University School of Medicine
○	○	○	Kanagawa Cancer Center
○	○		Yokohama City University Medical Center
○	○		Niigata Cancer Center Hospital
○			Toyama University Hospital
○			Kanazawa University School of Medicine
	○		Ishikawa Prefectural Central Hospital
	○		Ogaki Municipal Hospital
○	○	○	Shizuoka Cancer Center
○	○	○	Aichi Cancer Center Hospital
		○	Kyoto University Hospital
	○	○	Osaka University Graduate School of Medicine
○	○		Kindai University Hospital
○	○	○	Osaka International Cancer Institute
○	○	○	National Hospital Organization Osaka National Hospital
	○	○	Osaka General Medical Center
	○	○	Osaka Medical and Pharmaceutical University
	○		Osaka Rosai Hospital
○	○	○	Kobe University Graduate School of Medicine
	○	○	Kansai Rosai Hospital
○	○		Hyogo College of Medicine
○	○	○	Hyogo Cancer Center
	○		Itami City Hospital
	○		Shimane University Faculty of Medicine
	○	○	Hiroshima University Hospital
	○		Fukuyama City Hospital
	○		Tokushima Red Cross Hospital
○	○	○	National Hospital Organization Shikoku Cancer Center
○		○	Kochi Health Sciences Center
○		○	National Kyushu Cancer Center
○		○	Kyushu University Hospital

	○	○	Oita University Faculty of Medicine

HBP: Hepatobiliary and Pancreatic Oncology Group
SCSG: Stomach Cancer Study Group:
JEOG: Japan Esophageal Oncology Group

16.10. JCOG Protocol Review Committee

This protocol was approved by JCOG Protocol Review Committee prior to submit to Certified Review Board.
(For membership, see website http://www.jcog.jp/basic/org/committee/protocol.html)

Contact: Protocol Review Committee Office

JCOG Operations Office/Clinical Research Support Office, National Cancer Center Hospital
〒104-0045 5-1-1, Tsukiji, Chuo-ku, Tokyo
TEL: 03-3542-2511 (ext. 2302)
FAX:03-3542-7006
E-mail: jcogoffice@ml.jcog.jp

16.11. JCOG Data and Safety Monitoring Committee

During study period, the study will be monitored by Data and Safety Monitoring Committee (e.g., adverse event reports, interim analysis reviews, monitoring report reviews, protocol revision reviews). However, the committee members from the study group conducting this study do not participate directly in the review of this study.
(For membership, see website http://www.jcog.jp/basic/org/committee/jury.html)

Contact: JCOG Data and Safety Monitoring Committee Office

JCOG Operations Office/Clinical Research Support Office, National Cancer Center Hospital
〒104-0045 5-1-1, Tsukiji, Chuo-ku, Tokyo
TEL:03-3542-2511 (ext. 2403)
FAX:03-3542-7006
E-mail: jcogoffice@ml.jcog.jp

16.12. JCOG Audit Committee

Site-visit audits by Audit Committee will be conducted during study period.
(For membership, see website http://www.jcog.jp/basic/org/committee/audit.html)

Contact: JCOG Auditing Committee Office

JCOG Operations Office/Clinical Research Support Office, National Cancer Center Hospital
〒104-0045 5-1-1, Tsukiji, Chuo-ku, Tokyo
TEL:03-3542-2511 (ext. 2403)
FAX:03-3542-7006
E-mail: jcogoffice@ml.jcog.jp

16.13. JCOG Conflict of Interest Committee

During study period, JCOG investigators involved in this study will be managed by the Conflict of Interest Committee.
(For membership, see website http://www.jcog.jp/basic/org/committee/coi.html)

Contact: JCOG Conflict of Interest Committee Office

JCOG Operations Office/Clinical Research Support Office, National Cancer Center Hospital
〒104-0045 5-1-1, Tsukiji, Chuo-ku, Tokyo
TEL:03-3542-2511 (ext. 2404)
FAX:03-3547-1002
E-mail: jcogoffice@ml.jcog.jp

16.14. Data Center/Operations Office

JCOG Data Center

Director of Data Center: Haruhiko Fukuda
Clinical Research Support Office, National Cancer Center Hospital

〒104-0045 5-1-1, Tsukiji, Chuo-ku, Tokyo
TEL:03-3542-3373
FAX:03-3542-3374
E-mail: jcogdata@ml.jcog.jp

JCOG Operations Office
Director of Operations Office: Kenichi Nakamura
Clinical Research Support Office, National Cancer Center Hospital
〒104-0045 5-1-1, Tsukiji, Chuo-ku, Tokyo
TEL:03-3547-1002
FAX:03-3547-1002
E-mail: jcogoffice@ml.jcog.jp

Official website: http://www.jcog.jp/

16.14.1. Data management administrator

Data management organization		JCOG Data Center
Data management administrator	Name	Harumi Kaba
	e-Rad number	40543442
	Affiliation	Clinical Research Support Office, National Cancer Center Hospital
	Title	Head of Multi-institutional Data Management Section

16.14.2. Monitoring administrator

Monitoring organization		JCOG Data Center
Monitoring administrator	Name	Haruhiko Fukuda
	e-Rad number	70263390
	Affiliation	Clinical Research Support Office, National Cancer Center Hospital
	Title	Chief of Data Management Division

16.14.3. Site-visit audit administrator

Auditing organization		JCOG Operations Office
Site-visit audit administrator	Name	Kenichi Nakamura
	e-Rad number	40543533
	Affiliation	Clinical Research Support Office, National Cancer Center Hospital
	Title	Director of Research Management Division

16.14.4. Statistical analysis administrator

Statistical analysis organization		JCOG Data Center
Statistical analysis Administrator	Name	Junki Mizusawa
	e-Rad number	60706646
	Affiliation	Biostatistics Division, Center for Research Administration and Support, National Cancer Center/<br>Biostatistics Section, Research Management Division, Clinical Research Support Office, National Cancer Center Hospital
	Title	Biostatistics Section Head

16.14.5. Research and development plan support personnel

Organizations in charge of supporting research and development plans		JCOG Operations Office
Research and development plan support personnel	Name	Tomoko Kataoka
	e-Rad number	70569863
	Affiliation	Clinical Research Support Office, National Cancer Center Hospital
	Title	Medical officer

16.14.6. Coordination management practitioner

Organizations in charge of coordination and management		JCOG Operations Office
Coordinating and managing practitioners	Name	Junko Eba
	e-Rad number	80754085
	Affiliation	Clinical Research Support Office, National Cancer Center Hospital
	Title	Medical officer

16.14.7. Personnel who oversees the study other than Principal Investigator and site investigators

Personnel who oversees the study other than Principal Investigator and site investigators	Name	Not applicable	
	e-Rad number		
	Affiliation		
	Title		
	Relevance of Secondary Sponsor	□ Applicable	□ Not applicable

16.14.8. Study group personnel

JCOG Data Center

Statistics Section Gakuto OGAWA

Data Management Section Kyoko HASEGAWA

JCOG Operations Office

Science Section Tomoko KATAOKA/Tadayoshi HASHIMOTO

In addition, JCOG Data Center/Operations Office commissioned some of theire research support activities (such as support for the preparation of study protocols, data management, and site-visit audits) to other corporations. The commissioned duties are supervised by the National Cancer Center through routine work, as well as by receiving work reports from the institution and providing supervisory guidance. The current consignee is as follows

Clinical Oncology Research and Education, a specified non-profit organization

〒104-0061 DJ Ginza Building 7F, 8-18-3, Ginza, Chuo-ku, Tokyo

Official website http://www.core.or.jp/

16.15. Developing a study protocol

Chigusa MORIZANE, National Cancer Center Hospital

Nozomu MACHIDA, Kanagawa Cancer Center

Yoshitaka HONMA/Ken KATO, National Cancer Center Hospital

Support for protocol development

JCOG Data Center

Statistics Section (in charge of study design) Junki MIZUSAWA

Data Management Section (CRF preparation) Harumi KABA

JCOG Operations Office

Protocol development Hiroshi KATAYAMA/Kozo KATAOKA/Aya MIURA

Person in charge of IC documents Aya KIMURA/Noriko TSUJI

17. Publication of the study results and completion of the study

17.1. Paper and conference presentations

Primary publication will be published in English journals.

Paper publication including review article and conference presentation of Introduction of the study, by Principal Investigator or Study Coordinator, which does not include the analytical results of the endpoint of the study, are allowed when Group Chair and JCOG Data Center Director agree to them. Publication of the distribution of baseline factors or the safety data after the end of accrual are also allowed. No publication other than primary analysis and final analysis will be performed unless previously approved by Data and Safety Monitoring Committee.

In principle, the authors of the main published papers on the results of the study (the first publication of the results of primary endpoint) shall be the first Study Coordinator (HBPOG), followed by Study Coordinator (JEOG or SCSG), Study Coordinator (JEOG or SCSG), Principal Investigator (HBPOG), Principal Investigator (JEOG or SCSG), Principal Investigator (JEOG or SCSG), the statistical staff of Data Center (one statistician in charge at the time of the analysis for publication), Centralized Pathological diagnosis Coordinator (HBP), Centralized Pathological diagnosis Coordinator (GI). In accordance with the limitations imposed by the article's posting provisions, researchers who contributed in descending order of their number of patients registrations were selected for each institution as co-authors, and the last author was Group Chair (or Study Chair). The inclusion of staffs of JCOG Operations Office as co-authors will be determined by Group Chair depending on their contributions. Authors of articles other than the primary publication (e.g., Secondary endpoints articles, secondary analysis articles) will be determined by Study Chair with Group Chair approval.

All co-authors will review the article contents prior to submission for publication and only those who agree to the article contents. If there is no consensus on the contents, principle investigator may not include the investigator in the co-authorship with the approval of Group Chair. If there is no consensus between groups and JCOG Data Center/Operations Office, ultimately follow JCOG Chair instructions.

17.2. Primary Endpoint Report and Clinical Summary Report

The procedures are specified from the preparation of the primary endpoint report and clinical summary report. If primary analysis is the final analysis, the primary endpoint report will not be prepared and the clinical summary report will be prepared.

17.2.1. Clinical Summary Report

Based on the final analysis report, Principal Investigator will prepare a "Abstract of the Clinical Summary Report" containing subjects background information (age, sex, etc.), study design and study progress, results of analyses for each endpoint, conclusions of the entire study, interpretations and discussion of the results, etc. within 6 months from the issue date of the final analysis report, submit it to the Data Center, and undergo review. In addition, the issue date of the final analysis report shall be "the date when the period for collecting data on all endpoints is completed" as specified in the Enforcement Regulations.

After obtaining approval from Group Chair and the Director of the Data Center, abstract of the clinical summary report will be submitted to Certified Review Board review as a "clinical summary report" with the final analysis report within 1 year of the issue date of the final analysis report.

Principal Investigator/Study Coordinator will disclose abstract of the clinical summary report (Notification of Completion of Form 1 of Article 24 of Regulation) to jRCT within 1 month after obtaining Certified Review Board approval (if the report is unpublished, abstract will not be released in jRCT and will be published immediately after publication).

The approved abstract of the clinical summary report will be submitted to the administrators of each participating institution through the site investigators at each institution and to JCOG Chair. Abstract of the clinical summary report is available on JCOG website (http://www.JCOG.jp/).

The timing of distribution of the final analysis report to participating institutions will be determined by Principal Investigator/Study Coordinator considering the timing of publication of the final analysis results, and the final analysis report will be distributed to the researchers at participating institutions by themselves or through the Data Center.

17.3. Completion of the study

On the date that abstract of the clinical summary report was released to iRCT, the study is completed.

In institutions where patients were not enrolled, the institution may be withdrawn from the institution list by submitting a request for change (Unified Form 3 for Clinical Research) and a Notification of Changes in Protocol (Form 2) stating that Principal Investigator withdraws the institution from the institution list in question to Certified Review Board, and after obtaining approval, notifying the MHLW (Local Health Bureau responsible for the location of Certified Review Board). When a protocol change notification is received by the Local Health Service, the investigator at the institution shall report the withdrawal to the Administrator of the institution.

18. References

omit

19. Appendix

- Informed consent form
- Body surface area table
- Toxicity Criteria (CTCAE v5.0-JCOG)
- CRF samples* (CRF draft attached for the first review submission)
- JCOG-Biobank Japan Biorepository Protocol
- JCOG-Biobank Japan Biorepository Informed Consent Form
