# Supplementary material for: Effectiveness of Etoposide and Cisplatin vs Irinotecan and Cisplatin Therapy for Patients With Advanced Neuroendocrine Carcinoma of the Digestive System: The TOPIC-NEC Phase 3 Randomized Clinical Trial
Source: JAMA Oncol. 2022 Aug 18;8(10):1447–55. doi: 10.1001/jamaoncol.2022.3395 (PMC9389440; doi:10.1001/jamaoncol.2022.3395)
Supplement: Supplement 3. — Statistical Analysis Plan [file jamaoncol-e223395-s003.pdf]

This is an abridged translation of the original document which was written in Japanese.

JCOG1213 Statistical Analysis Plan

Version	Date	Author	Protocol version	Revised content
1.0	2017/8/23	Junki MIZUSAWA	Ver 1.1	–
2.0	2021/6/10	Shogo NOMURA	Ver 1.6.0	Add descriptions about the final analysis

Approval

YYYY/MM/DD Haruhiko FUKUDA, Director of JCOG Data Center

Signature ____

YYYY/MM/DD Taro SHIBATA, Section head, statistics section, JCOG Data Center

Signature ____

YYYY/MM/DD Shogo NOMURA, Trial statistician of JCOG1213 (Trial statistician), JCOG Data Center

Signature ____

YYYY/MM/DD Ryo SADACHI, Trial statistician of JCOG1213 (Statistical analyst), JCOG Data Center

Signature ____

YYYY/MM/DD Kanako FUYAMA, Trial statistician of JCOG1213 (Statistical analyst), JCOG Data Center

Signature ____

Contents

1. KEY DECISION CRITERIA AND MULTIPLICITY ADJUSTMENT ... 3
1.1. Key decision criteria for study results ... 3
1.2. Multiplicity Adjustment ... 3
2. EFFICACY ANALYSIS ... 3
2.1. Analyses of primary endpoint ... 3
2.1.1. Primary analysis methods ... 3
2.1.2. Analysis methods using statistical models ... 3
2.1.3. Method of supplementary analysis ... 4
2.1.4. Handling of missing data ... 4
2.1.5. Summary of follow-up status ... 4
2.2. Secondary endpoint (Progression-free Survival) analysis ... 4
2.2.1. Primary analysis methods ... 4
2.2.2. Analysis methods using statistical models. ... 4
2.2.3. Method of subgroup analysis ... 4
2.2.4. Method of supplementary analysis ... 5
2.2.5. Handling of missing data ... 5
2.3. Secondary endpoint (response rate) analysis ... 5
2.3.1. Primary analysis methods ... 5
2.3.2. Analysis methods using statistical models ... 5
2.3.3. Method of subgroup analysis ... 5
2.3.4. Method of supplementary analysis ... 6
2.3.5. Handling of missing data ... 6
3. SAFETY ANALYSIS ... 6
3.1. Proportion of patients with adverse events ... 6
3.2. Proportion of patients with serious adverse events ... 6
4. OTHER ANALYSES ... 6
4.1. Dose intensity of cisplatin ... 6
5. INTERIM ANALYSIS ... 7
5.1. Purpose of the interim analysis ... 7
5.2. Timing for interim analysis ... 7
5.3. Multiplicity adjustment methods in interim analyses ... 7
5.4. Decision Criteria in the interim analysis ... 7
5.5. Reporting and review of the results ... 8
6. FINAL ANALYSIS ... 8
6.1. The timing ... 8
6.2. Analysis methods ... 8
7. PLANNED ACCRUAL, ACCRUAL PERIOD, AND FOLLOW-UP PERIODS ... 9
8. SOFTWARE ... 9
9. REFERENCE ... 9
SUPPLEMENTAL NOTE FOR THE FIRST INTERIM ANALYSIS (2017/9) ... 10
SUPPLEMENTAL NOTE FOR THE FINAL ANALYSIS (2021/6) ... 12

1. Key decision criteria and multiplicity adjustment

1.1. Key decision criteria for study results

The purpose is to test whether one of the two deemed standard-of-care arms A (EP-therapy) and B (IP-therapy) outperforms the rest regarding the primary endpoint, overall survival.

We conclude that EP therapy is a more useful treatment when the two-sided p-value is less than 10% and the survival curve of EP therapy exceeds that of IP therapy. Meanwhile, we conclude that IP therapy is a more useful treatment when the two-sided p-value is less than 10% and the survival curve of IP therapy exceeds that of EP therapy. If the difference was not significant at a two-sided significance level of 10%, one of the two deemed standard treatment cannot be judged to be more useful. In this case, we conclude that both regimen continue to be the standard of care, as there is no evidence to actively recommend either one of them.

1.2. Multiplicity Adjustment

For a reason that our primary interest is whether either of two arms is superior to the other, two-sided test is used. The study-wise significance level is set at two-sided 10%. In the primary analysis, two-sided 90% confidence intervals corresponding to a two-sided 10% significance level will be calculated, and in the other analyses, two-sided 95% confidence intervals will be calculated for descriptive purposes.

Significance levels and corresponding confidence coefficients used to test the primary hypotheses are based on the multiplicity adjustment associated with the interim analysis.

2. Efficacy analysis

2.1. Analyses of primary endpoint

2.1.1. Primary analysis methods

The primary analysis will be the final analysis unless this study is withdrawn from the interim analysis. In the primary analysis, the null hypothesis of equality of the overall survival among the two groups will be tested by stratified log-rank test. The stratification factors include randomization adjustment factors except institution (i.e., primary organ [gastrointestinal vs. hepatobiliary-pancreatic]) in all enrolled patients. However, if it is assumed that a stratified log-rank test cannot be performed appropriately, such as when the number of subjects and events in each stratum is small, handling of randomization adjustment factors will be addressed in the statistical analysis plan which will be finalized without information related to the comparison between groups before performing a confirmatory analysis.

Cumulative survival curves, median survival times, and annual survival rates are estimated using Kaplan-Meier method. Brookmeyer and Crowley methods are used to obtain 95% confidence intervals for median survival. Greenwood's formula is used to obtain 95% confidence intervals for annual survival rates. If the required number of events has been reached at 1 year after completion of enrollment, the final analysis will be performed at 1 year after completion of enrollment. Conversely, if the required number of events is not reached, follow-up will continue until the required number of events is reached to ensure power, and the final analysis will be performed without waiting for the end of the remaining follow-up period when the required number of events is found to be reached within the follow-up period. The final analysis will be summarized by the Data Center as the "Final Analysis Report" and submitted to the Research Secretariat, Research Representatives, Group Representatives, Group Secretariats, Data and Safety Monitoring Committee, and JCOG Representatives.

2.1.2. Analysis methods using statistical models

As a treatment effect measure, hazard ratios and their confidence intervals for treatment effects between groups are estimated using stratified Cox proportional hazards models with the same factors as the stratified log-rank test for the primary analysis.

To investigate the interaction between treatment effect and the subpopulation, hazard ratios and their 95% confidence intervals for treatment effects will be calculated using unstratified Cox's proportional hazards model. These will be done in an exploratory manner with respect to the following factors. Because these analyses are not adequately powered and do not adjust for multiplicity, the results of each subgroup analysis should be interpreted as exploratory.

Factors planned for subgroup analysis:

- PS0/1
- Age 65 years or older/<
- Gender (male/female)
- Primary organ (gastrointestinal tract/hepatobiliary pancreas)
- Organ of origin (esophagus/stomach/small intestine/large intestine/pancreas/biliary tract/liver NEC (liver primary or liver metastasis of unknown primary)
- Organ of origin (pancreas/non-pancreas)
- Extent of extension of the primary lesion (locally advanced/distant metastasis or recurrence)
- Extent of extension of the primary lesion (locally advanced/distant metastasis/recurrence)
- Prior radical resection of the primary lesion (none/present)
- Pathological diagnosis was biopsy/resection specimen
- Grade 3 tumour with morphologically similar features of NETs but Grade 3 proliferative activity/morphologically more atypical (previously classified as poorly differentiated endocrine carcinoma) on histopathology with central pathology
- Histopathological examination by central pathological diagnosis, including Small cell carcinoma/Large cell carcinoma /
- Histopathologically diagnosed by central pathology, Ki67 50% or higher/less than 50%

2.1.3. Method of supplementary analysis

To evaluate the robustness of the primary analysis results, the following sensitivity analyses will be performed if necessary.

- Hazard ratios and their confidence intervals for treatment effects between arms using a unstratified Cox proportional hazards model for all enrolled patients.
- Hazard ratios and their confidence intervals for treatment effects between arms using stratified Cox proportional hazards models with the same factors as the test of the primary analysis, with background factors biased between arms as covariates, for all enrolled patients.
- Hazard ratios and their confidence intervals for treatment effects between arms using the same stratified Cox proportional hazards model as in the primary analysis (* analysis population is all eligible patients).
- Hazard ratios and their confidence intervals for treatment effects between arms using the same stratified Cox proportional hazards model as in the primary analysis (* analysis population is patients eligible for central pathology review).

2.1.4. Handling of missing data

No imputations will be made for missing data.

2.1.5. Summary of follow-up status

For all enrolled patients, the follow-up period is calculated for each patient according to the following definitions, and the summary statistics (minimum, 25th percentile; median, 75th percentile; maximum) are calculated.

Follow-up time (days) = (date of death (died patient) or date of last survival confirmation (survivors))-date of registration + 1

2.2. Secondary endpoint (Progression-free Survival) analysis

2.2.1. Primary analysis methods

Estimates, including progression-free survival curves, median progression-free survival, and time-point progression-free survival, will be performed using Kaplan-Meier method. Greenwood formulas will be used to obtain 95% confidence intervals. Log-rank test is used for comparison between arms.

2.2.2. Analysis methods using statistical models.

Hazard ratios and their 95% confidence intervals for treatment effects are estimated using unstratified Cox proportional hazards models.

2.2.3. Method of subgroup analysis

To investigate the interaction between treatment effect and the subpopulation, subgroup analyses will be conducted in an exploratory manner with respect to the following factors. Because these analyses are not adequately powered and do not adjust for multiplicity, the results of each subgroup analysis should be interpreted as exploratory.

Factors planned for subgroup analysis:
- PS0/1
- Age 65 years or older/<
- Gender (male/female)
- Primary organ (gastrointestinal tract/hepatobiliary pancreas)
- Organ of origin (esophagus/stomach/small intestine/large intestine/pancreas/biliary tract/liver NEC (liver primary or liver metastasis of unknown primary)
- Organ of origin (pancreas/non-pancreas)
- Extent of extension of the primary lesion (locally advanced/distant metastasis or recurrence)
- Extent of extension of the primary lesion (locally advanced/distant metastasis/recurrence)
- Prior radical resection of the primary lesion (none/present)
- Pathological diagnosis was biopsy/resection specimen
- Grade 3 tumour with morphologically similar features of NETs but Grade 3 proliferative activity/morphologically more atypical (previously classified as poorly differentiated endocrine carcinoma) on histopathology with central pathology
- Histopathological examination by central pathological diagnosis, including Small cell carcinoma/Large cell carcinoma /
- Histopathologically diagnosed by central pathology, Ki67 50% or higher/less than 50%

2.2.4. Method of supplementary analysis

To evaluate the robustness of the analysis results for PFS, the following sensitivity analyses will be performed if necessary.

- Hazard ratios and their confidence intervals for treatment effects between arms using a stratified Cox proportional hazards model for all enrolled patients.
- Hazard ratios and their confidence intervals for treatment effects between arms using a unstratified Cox proportional hazards models including clinical background factors differentially distributed between arms as covariates (* analysis population is all enrolled patients).
- Hazard ratios and their confidence intervals for treatment effects between arms using unstratified Cox proportional hazards model for all eligible patients.
- Hazard ratios and their confidence intervals for treatment effects between arms using the same stratified Cox proportional hazards model as in the primary analysis (* analysis population is patients eligible for central pathology review).

2.2.5. Handling of missing data

No imputations will be made for missing data. We censor patients who did not progress following criteria in Section 11.3.2 in the protocol. Note that we describe how to address a problem when there exists missing data which may seriously affect the analysis results.

2.3. Secondary endpoint (response rate) analysis

2.3.1. Primary analysis methods

Response rate is a proportion of patients among all eligible patients with measurable disease who are categorized into CR or PR according to the criteria in the Section 11.1.10 in the protocol. Fisher's exact test will be used to compare response rates between groups, and the exact confidence intervals will be constructed using Clopper and Pearson method.

2.3.2. Analysis methods using statistical models

Odds ratios and their confidence intervals will be constructed using logistic regression models if necessary.

2.3.3. Method of subgroup analysis

To investigate the interaction between treatment effect and the subpopulation, subgroup analyses will be conducted in an exploratory manner with respect to the following factors. Because these analyses are not adequately powered and do not adjust for multiplicity, the results of each subgroup analysis should be interpreted as exploratory.

Factors planned for subgroup analysis
- PS0/1
- Age 65 years or older/<

- Gender (male/female)
- Primary organ (gastrointestinal tract/hepatobiliary pancreas)
- Organ of origin (esophagus/stomach/small intestine/large intestine/pancreas/biliary tract/liver NEC (liver primary or liver metastasis of unknown primary)
- Organ of origin (pancreas/non-pancreas)
- Extent of extension of the primary lesion (locally advanced/distant metastasis or recurrence)
- Extent of extension of the primary lesion (locally advanced/distant metastasis/recurrence)
- Prior radical resection of the primary lesion (none/present)
- Pathological diagnosis was biopsy/resection specimen
- Grade 3 tumour with morphologically similar features of NETs but Grade 3 proliferative activity/morphologically more atypical (previously classified as poorly differentiated endocrine carcinoma) on histopathology with central pathology
- Histopathological examination by central pathological diagnosis, including Small cell carcinoma/Large cell carcinoma /
- Histopathologically diagnosed by central pathology, Ki67 50% or higher/less than 50%

2.3.4. Method of supplementary analysis

To confirm the robustness of the primary analysis results, the following sensitivity analyses will be performed if necessary.

- ORR and the 95% confidence interval among all enrolled patients with measurable disease.
- ORR and the 95% confidence interval among all patients who are eligible for centrally pathological review and with measurable disease.

2.3.5. Handling of missing data

No imputations will be made for missing data. Patients whose best response are NE will be categorized as non-responders and will not be excluded from the analysis.

3. Safety analysis

Among secondary endpoints, the safety endpoints are the incidence of adverse events and the incidence of serious adverse events, which are in principle the items of periodical monitoring (see Section 14.1 in the protocol)

3.1. Proportion of patients with adverse events

The proportion is calculated to evaluate differences in toxicity among the two groups. Frequency of adverse events marking the worst grade in CTCAE v4.0-JCOG that occured during the entire course of therapy will be calculated (by group) using the number of all treated patients as a denominator. If necessary, exact confidence intervals will be calculated and Fisher’s exact test will be used for group comparisons.

3.2. Proportion of patients with serious adverse events

Grade 4 non-hematologic adverse events, early deaths, and treatment-related deaths are defined as serious adverse events which are reported in semi-annual Monitoring Report with patient registry numbers and details.

In addition, the incidence of Grade 4 non-hematologic adverse events, early deaths, and treatment-related deaths will be calculated for all treated patients when comparing efficacy analyses between arms.

Exact confidence intervals for the proportion is calculated.

Neither of these is judged based on statistical tests, but comparisons are made between arms using Fisher's exact test, as appropriate.

4. Other analyses

4.1. Dose intensity of cisplatin

Summary statistics (minimum, percentiles [25%/50%/75%], maximum, mean, and SD) of dose intensity (D.I.) regarding cisplatin will be calculated. Analysis population is all treated patients. The aim is to evaluate the treatment compliance of cisplatin.

- Actual D.I.（mg/m^2/week）= (total amount of cisplatin) /BSA/ (treatment duration [weeks])
  - ➢ BSA = weight $^{0.425}$ x height $^{0.725}$ x 0.007184
    （For weight and height, the values calculated in data center at the timing of enrollment are used）
  - ➢ Treatment duration (weeks)
    - ✧ For arm A, it is defined as a duration from the initiation date of the first treatment course to the date of 21 days from the initiation date of the last treatment course.
    - ✧ For arm B, it is defined as a duration from the initiation date of the first treatment course to the date of 28 days from the initiation date of the last treatment course.

5. Interim analysis

5.1. Purpose of the interim analysis

One interim analysis will be conducted to determine if the primary objective of the study has been achieved during the study period. Interim analysis will be conducted to determine if it is reasonable to continue enrollment during enrollment. If the primary objective of the study is determined to be achieved, the study will be discontinued and the study results will be published promptly at the conference and in the article.

5.2. Timing for interim analysis

Interim analysis will be conducted using data from the initial periodic monitoring that will be queried after the time enrollment of half of the planned enrollment was obtained. Based on the information in the periodic monitoring report, the group will submit the presence or absence of changes in clinical hypotheses and, if any, details of changes to the Data and Safety Monitoring Committee by the time of the interim analysis.

During interim analysis, patient accrual will not be halted in principle. If the study progressed as expected, the expected number of events at the time of the interim analysis is expected to be 44 if the interim analysis is conducted at 3 years after the start of enrollment.

5.3. Multiplicity adjustment methods in interim analyses

Interim analysis will be conducted by the JCOG Data Center. To control the study-wise type-I error rate at 10%, the multiplicity of the interim and final analyses is adjusted using Lan & DeMets's alpha spending functions, and the calculated statistical significance level is used for testing primary endpoint between arms. As alpha spending functions, we use O'Brien & Fleming type.

5.4. Decision Criteria in the interim analysis

Decision criteria based on the results of the interim analysis is following.

- If superiority of arm B over arm A (or vice versa) is demonstrated based on a criterion that the two-sided p-value calculated from stratified log-rank test is below the significance level calculated from the method detailed in Section 5.3, then the study is to be discontinued due to an efficacy.
- Otherwise, the study is to be discontinued.

Futility stop is also considered from various aspects. As a reference, the following is calculated.

- Predictive probability for primary endpoint (based on Spiegelhalter et al. (1992)) that will be calculated based on a Bayesian predictive distribution of hazard ration on the final analysis. To calculate the following predictive probabilities, log-rank score statistic calculated from the stratified log-rank test for overall survival (in the primary analysis) is used.
  - ➢ Predictive probability that the superiority of arm B over arm A (or vice versa) will be demonstrated on the final analysis (at the time of information time=1).
  - ➢ Predictive probability that point estimate of hazard ratio for arm A will be greater than one on the final analysis (at the time of information time=1).
  - ➢ Predictive probability that point estimate of hazard ratio for arm B will be greater than one on the final analysis (at the time of information time=1).

- Conditional power for primary endpoint (based on Halperin et al. (1982)). To calculate the following conditional power, log-rank score statistic calculated from the stratified log-rank test for overall survival (in the primary analysis) is used.

- Conditional power given the observed interim analysis data that the trial would reject the null hypothesis on the final analysis assuming the true hazard ratio for arm A after 1st interim analysis would be one (the null hypothesis for the primary analysis)
- Conditional power given the observed interim analysis data that the trial would reject the null hypothesis on the final analysis assuming the true hazard ratio for arm B after 1st interim analysis would be one (the null hypothesis for the primary analysis)
- Conditional power given the observed interim analysis data that the trial would reject the null hypothesis on the final analysis assuming the true hazard ratio for arm A after 1st interim analysis would be 0.67 (the expected hazard ratio for overall survival)
- Conditional power given the observed interim analysis data that the trial would reject the null hypothesis on the final analysis assuming the true hazard ratio for arm B after 1st interim analysis would be 0.67 (the expected hazard ratio for overall survival)

5.5. Reporting and review of the results

The results of the interim analysis will be submitted to the Data and Safety Monitoring Committee by the Data Center as an Interim Analysis Report and reviewed for the acceptability of continuation of the study and for publication of the results. The Data and Safety Monitoring Committee considers whether to continue the study at the meeting and recommends whether to continue the study and whether to inform the results to principal physician or group chair based on the results of the review.

Members of the Data and Safety Monitoring Committee of the relevant groups are not included in the review. Unless the results of the interim analysis make recommendations for discontinuation of the study from the Data and Safety Monitoring Committee, the research representative, research office, participating institution researchers, group representatives, and group secretaries of the study will not be able to know the results of the interim analysis until the final follow-up is completed.

When the Interim Analysis Report has been reviewed by the Data and Safety Monitoring Committee to recommend termination or change of all or part of the study, the principle investigator and group chair review the recommendations and decide whether to discontinue or change some of the study.

If the study is discontinued or part of the study is changed, the principal investigator and group chair shall submit in written form a request to the Data and Safety Monitoring Committee for permission to discontinue the study or a request to revise the protocol. Following approval by the Data and Safety Monitoring Committee, the principal investigator may discontinue the study or change part of the study.

The Study Chair and Group Chair can disagree with the recommendations of the Data and Safety Monitoring Committee, but if they fail to coordinate their opinions with the Data and Safety Monitoring Committee, they will ultimately follow the instructions of JCOG Chair.

If the study is terminated, the subsequent follow-up period will be 1 years from the date of last registration.

If the interim analysis resulted in study termination, the interim analysis will be the primary analysis of the study. The Data Center, in cooperation with the Research Representative Physicians and Research Secretariat, will conduct the analysis required to complement the incomplete data and publish the results, focusing on the results of the interim analysis, and promptly prepare the Major Analysis Report and submit it to the Group and the Data and Safety Monitoring Committee.

6. Final analysis

6.1. The timing

The final analysis will be performed one year after accrual completion.

6.2. Analysis methods

The same as those detailed in Section 2 to 4 in the SAP.

7. Planned accrual, accrual period, and follow-up periods

As described in ”Section 2.4.2. in the protocol. Based on the background presented in Clinical Hypothesis and Rationale for Number of Enrollment”, we assume a median survival of 8 and 12 months (HR=0.67) for inferior and superior treatment groups. Total of 63 patients per group and 126 patients in both groups (114 events required) will be required using the method of Schoenfeld and Richter[50]. The above calculation assumes 6 years of enrollment, 1 year of follow-up, α = 10% (two-sided), and 70% power. The required number of patients and events are listed in table below when median survival time of arm A is different to the expectation.

Table: Number of Analyses Required (Number of Events Required)

	Power			
Median Survival (mo)	65%	70%	75%	80%
7.0 vs. 10.5	108(100)	124(114)	142(131)	162(150)
8.0 vs. 12.0	112(100)	126(114)	144(131)	166(150)
9.0 vs. 13.5	114(100)	130(114)	148(131)	170(150)

※ Number of required events in parentheses

Based on these, the following will be established in view of some cases of loss to follow-up.

Planned enrollment: 70 patients in each group and 140 patients in both groups
Enrollment period: 6 years, follow-up period: 1 year after completion of enrollment

Consider sample size recalculation (SSR) if the prognosis is obviously better than assumed, or if 70 or more patients are enrolled within first 2.5 years. The SSR needs reconsideration of clinically meaningful differences in a blinded fashion and needs to be performed prior to the conduct of the primary analysis.

<Additions in protocol ver. 1.1>

The registration pace after the enrollment start was smooth and exceeded the plan, and 70 cases were enrolled in October, 2016, when 2 years and 2 months passed from the enrollment start, which met the above-mentioned criteria for SSR. SSR with an aim of raising power to 80% was accepted at the meeting of the Hepatobiliary and Pancreatic Group on October 29, 2016, the group meeting of the Esophageal Cancer Group on November 19, 2016, and the group meeting of the Gastric Cancer Group on January 7, 2017. Therefore, the required number of enrollment was changed to 170. In addition, the "1-year analysis period" was added to the study period, and the following changes were made.

Planned enrollment: 85 patients in each group and 170 patients in both groups
Enrollment period: 6 years;
Follow-up period: 1 year after completion of enrollment;
Analysis period: 1 year;
Total study period: 8 years

8. Software

SAS 9.4

9. Reference

1. Lan KKG, DeMets DL. Discrete sequential boundaries for clinical trials. Biometrika 1983;70(3):659-663.
2. Spiegelhalter DJ, Freedman LS, Parmar MKB. Applying Bayesian Ideas in Drug Development and Clinical-Trials. Statistics in medicine. 1993;12(15-16):1501-11.
3. Halperin M, Lan KKG, Ware JH, Johnson NJ, DeMets DL. An Aid to Data Monitoring in Long-Term Clinical-Trials. Controlled Clinical Trials. 1982;3(4):311-23.
4. Schoenfeld DA, Richter JR. Nomograms for calculating the number of patients needed for a clinical trial with survival as an endpoint. Biometrics 1982;38(1):163-170.

Supplemental note for the first interim analysis (2017/9)

<Data sets for the analysis (see Section 5.2 in the SAP)>
Data sets used for the first half year of 2017 fiscal year semi-annual monitoring report is used.

<Population for primary analysis (see Section 2 in the SAP)>
OS and PFS are analyzed for all the 86 patients enrolled until March 31, 2017. For response rate, only 46 patients are analyzed among 79 patients with measurable disease. This is because some patients are under treatment and thus CRF regarding response evaluation is not submitted. Note that CRFs of the 46 patients have been submitted and the associated queries have been resolved.

<Population for safety analyses (see Section 3 in the SAP)>
Of the 86 patients enrolled until March 31, 2017, safety analyses are performed on 72 patients whose CRF for treatment are collected by March 31, 2017. In the interim analysis, the results of safety analysis are presented using the first half year of 2017 fiscal year semi-annual monitoring report.

<Population for the other analysis (see Section 4 in the SAP>
Dose intensity of cisplatin is not analyzed at the timing of interim analysis.

<Information time>
Among the 86 patients for the efficacy population, the number of observed events is 55 in total. Thus, the information time at the 1st interim analysis is 55/150 (0.3666666666666600). The calculated significance level and boundary are as follows.

- Two-sided significance level: α' = 0.0024177822594063
- Boundary for log-rank test statistic = ±3.0334456905510900

<Items to be reported in the interim analysis report>

- Primary endpoint：OS
  - Interim analysis methods and decision rules
    - Summary of decision rules in the interim analysis and the significance level.
  - Kaplan-Meier curves for OS
    - Group comparisons
    - Kaplan-Meier curves in the subgroups of randomization adjustment factors.
  - Summary statistics
    - Sample size, number of events, median OS and the 95% confidence interval, 1-year OS and the 95% confidence interval
    - The above summary statistics in the pooled survival curve
    - The above summary statistics in the survival curves of subgroups regarding randomization adjustment factors
  - The primary analysis results for evaluating a stop for efficacy
    - Results of stratified log-rank test for superiority hypothesis
    - Results of unstratified log-rank test for superiority hypothesis
    - Hazard ratio and the (1-2α')-% confidence interval constructed from stratified Cox regression model
    - Hazard ratio and the (1-2α')-% confidence interval constructed from unstratified Cox regression model
  - Supporting information to evaluate a stop for inefficacy
    - Predictive probabilities defined in the Section 5.4 in the SAP.
    - Conditional power defined in the Section 5.4 in the SAP.

- Secondary endpoint： PFS
  - Kaplan-Meier curves for PFS

- Summary statistics
  - Sample size, number of PFS events, median PFS and the 95% confidence interval, 1-year PFS and the 95% confidence interval
  - The above summary statistics in the pooled survival curve
- The results of unstratified log-rank test
- Hazard ratio and the 95% confidence interval constructed from unstratified Cox regression model

- Secondary endpoint：response rate
  - Best responses in the two groups
  - Response rates in the two groups with corresponding exact 95% confidence intervals
    - Two-sided p-value is also calculated using Fisher’s exact test.

Supplemental note for the final analysis (2021/6)

<Data sets for the analysis (see Section 5.2 in the SAP)>
Data sets based on a follow-up survey on March, 2021.

<Population for primary analysis (see Section 2 in the SAP)>
OS and PFS are analyzed for all enrolled 170 patients. As supportive analysis for OS and PFS, two analysis populations (all eligible 166 patients and 152 patients eligible for centrally pathological review) are also analyzed. Response rate is analyzed for 157 eligible patients with measurable disease.

<Population for safety analyses (see Section 3 in the SAP)>
Safety analyses are performed on 164 eligible and treated patients.

<Information time>
Among the 170 patients for the efficacy population, the total number of observed events was 55 at the timing of interim analysis and that is 151 in the final analysis. Thus, the information time, the calculated significance level, and boundary in the interim and final analyses are as follows.

- The interim analysis
  - Information time: 55/150 (0.3666666666666600)
  - Two-sided significance level: α’ = 0.0024177822594063
  - Boundary for log-rank test statistic = ±3.0334456905510900
- The final analysis
  - Information time: 1.00 (all the remaining alpha level is spent)
  - Two-sided significance level: α’ = 0.0992019861996200
  - Boundary for log-rank test statistic = ±1.6487347573298200

<Adjustment factors>
The primary analysis (stratified log-rank test) for overall survival is planned to use a randomization adjustment factor of primary organ (gastrointestinal vs. hepatobiliary-pancreatic). For a reason that the number of deaths is enough for each stratum in the adjustment factor, the stratified log-rank test uses the adjustment factor as planned.
