# Supplementary material for: Effectiveness of Etoposide and Cisplatin vs Irinotecan and Cisplatin Therapy for Patients With Advanced Neuroendocrine Carcinoma of the Digestive System: The TOPIC-NEC Phase 3 Randomized Clinical Trial
Source: JAMA Oncol. 2022 Aug 18;8(10):1447–55. doi: 10.1001/jamaoncol.2022.3395 (PMC9389440; doi:10.1001/jamaoncol.2022.3395)
Supplement: Supplement 4. — Nonauthor Collaborators. Japan Clinical Oncology Group (JCOG) nonauthor collaborators [file jamaoncol-e223395-s004.pdf]

Supplemental Online Content: Nonauthor Collaborators

*Indicates required information. Only first name, last name, and suffix will appear in PubMed.

*Group Name(s): Japan Clinical Oncology Group (JCOG)							
***First Name and Middle Initial(s)**	***Last Name**	***Suffix (eg, Jr, III)**	Academic Degrees	Institution	Location (city, state/province, country)	Role or Contribution, eg, chair, principal investigator	Group (if more than 1 Group listed in the byline) and/or Subgroup (eg, Steering Committee)
Yusuke	Sano		M.D.	Japan Clinical Oncology Group Data Center/Operations Office, National Cancer Center Hospital	Tokyo, Japan	Preparing the manuscript	
Kyoko	Hasegawa		-	Japan Clinical Oncology Group Data Center/Operations Office, National Cancer Center Hospital	Tokyo, Japan	Data management	
Ryo	Sadachi		MS	Japan Clinical Oncology Group Data Center/Operations Office, National Cancer Center Hospital	Tokyo, Japan	Statistical analysis supports	
Kenichi	Nakamura		M.D., Ph.D.	Japan Clinical Oncology Group Data Center/Operations Office, National Cancer Center Hospital	Tokyo, Japan	Director, JCOG Data Center	
Haruhiko	Fukuda		M.D.	Japan Clinical Oncology Group Data Center/Operations Office, National Cancer Center Hospital	Tokyo, Japan	Director, JCOG Operations Office	
Mitsuya	Iwafuchi		Professor	Laboratory of Pathology, Medical Labotatory Science, Niigata University Graduate School of Health Sciences	Niigata, Japan	Central pathological review	Central pathological review (CPR) panel
Ryoji	Kushima		M.D., Ph.D.	Department of Pathology, Shiga University of Medical Science	Otsu, Shiga, Japan	Central pathological review	Central pathological review (CPR) panel
Tetsuo	Ushiku		M.D., Ph.D.	Department of Pathology, The University of Tokyo	Tokyo, Japan	Central pathological review	Central pathological review (CPR) panel
Noriyoshi	Fukushima		M.D., Ph.D.	Jichi Medical University	Shimotsuke, Tochigi, Japan	Central pathological review	Central pathological review (CPR) panel
Nobuyuki	Ohike		Professor	Department of Pathology,St.Marianna University School of Medicine	Kawasaki, Kanagawa, Japan	Central pathological review	Central pathological review (CPR) panel
Yuki	Katsuta			Department of Hepatobiliary and Pancreatic Oncology, National Cancer Center Hospital	Tokyo, Japan	Management of Central pathological review	
Keiya	Okamura		M.D.	Sapporo-Kosei General Hospital		Patient Recruitment, Enrollment, and Retention	Hepatobiliary and Pancreatic Oncology Group:HBPOG
Yasyuki	Kawamoto		M.D., Ph.D.	Hokkaido University Hospital	Sapporo,Hokkaido,japan	Patient Recruitment, Enrollment, and Retention	Hepatobiliary and Pancreatic Oncology Group:HBPOG
Hirofumi	Shirakawa		M.D., Ph.D.	Tochigi Cancer Center		Patient Recruitment, Enrollment, and Retention	Hepatobiliary and Pancreatic Oncology Group:HBPOG
Hironori	Yamaguchi		M.D., Ph.D.	Jichi Medical University	Shimotsuke, Tochigi, Japan	Patient Recruitment, Enrollment, and Retention	Hepatobiliary and Pancreatic Oncology Group:HBPOG
Satoshi	Shimizu		M.D.	Saitama Cancer Center	Ina, Saitama, Japan	Patient Recruitment, Enrollment, and Retention	Hepatobiliary and Pancreatic Oncology Group:HBPOG
Hisahiro	Matsubara		M.D., Ph.D.	Chiba University, Graduate School of Medicine	Chiba, Chiba, Japan	Patient Recruitment, Enrollment, and Retention	Hepatobiliary and Pancreatic Oncology Group:HBPOG

*Indicates required information. Only first name, last name, and suffix will appear in PubMed.

***First Name and Middle Initial(s)**	***Last Name**	***Suffix (eg, Jr, III)**	Academic Degrees	Institution	Location (city, state/province, country)	Role or Contribution, eg, chair, principal investigator	Group (if more than 1 Group listed in the byline) and/or Subgroup (eg, Steering Committee)
Yasushi	Kojima		M.D., Ph.D.	National Center for Global Health and Medicine (NCGM)	Shinjuku, Tokyo, Japan	Patient Recruitment, Enrollment, and Retention	Hepatobiliary and Pancreatic Oncology Group:HBPOG
Keiji	Sano		M.D., Ph.D.	Teikyo University School of Medicine	Itabashi-ku, Tokyo, Japan	Patient Recruitment, Enrollment, and Retention	Hepatobiliary and Pancreatic Oncology Group:HBPOG
Kumiko	Umemoto		M.D., Ph.D.	St.Marianna University School of Medicine	Kawasaki, Kanagawa, Japan	Patient Recruitment, Enrollment, and Retention	Hepatobiliary and Pancreatic Oncology Group:HBPOG
Rika	Sakai		M.D., Ph.D.	Kanagawa Cancer Center	Yokohama, Kanagawa, Japan	Patient Recruitment, Enrollment, and Retention	Hepatobiliary and Pancreatic Oncology Group:HBPOG
Haruo	Miwa		M.D., Ph.D.	Yokohama City University Medical Center	Yokohama, Kanagawa, Japan	Patient Recruitment, Enrollment, and Retention	Hepatobiliary and Pancreatic Oncology Group:HBPOG
Kazuhiko	Shioji		M.D.	Niigata Cancer Center Hospital	Niigata, Niigata, Japan	Patient Recruitment, Enrollment, and Retention	Hepatobiliary and Pancreatic Oncology Group:HBPOG
Shinya	Kajiura		M.D., Ph.D.	Toyama University Hospital	Toyama, Toyama, Japan	Patient Recruitment, Enrollment, and Retention	Hepatobiliary and Pancreatic Oncology Group:HBPOG
Takeshi	Terashima		M.D., Ph.D.	Kanazawa University Hospital	Kanazawa, Ishikawa, Japan	Patient Recruitment, Enrollment, and Retention	Hepatobiliary and Pancreatic Oncology Group:HBPOG
Kazuyoshi	Ohkawa		M.D.	Osaka International Cancer Institute		Patient Recruitment, Enrollment, and Retention	Hepatobiliary and Pancreatic Oncology Group:HBPOG
Masahiro	Tsuda		M.D., Ph.D.	Hyogo Cancer Center	Akashi, Hyogo, Japan	Patient Recruitment, Enrollment, and Retention	Hepatobiliary and Pancreatic Oncology Group:HBPOG
Akinori	Asagi		M.D., Ph.D.	National Hospital Organization Shikoku Cancer Center	Matsuyama, Ehime, Japan	Patient Recruitment, Enrollment, and Retention	Hepatobiliary and Pancreatic Oncology Group:HBPOG
Toshiyuki	Suzuki		M.D.	National Hospital Organization Kyushu Cancer Center	Fukuoka, Fukuoka, Japan	Patient Recruitment, Enrollment, and Retention	Hepatobiliary and Pancreatic Oncology Group:HBPOG
Nao	Fujimori		M.D., Ph.D.	Kyushu University Hospital	Fukuoka, Fukuoka, Japan	Patient Recruitment, Enrollment, and Retention	Hepatobiliary and Pancreatic Oncology Group:HBPOG
Kentaro	Kawakami		M.D., Ph.D.	Keiyukai Sapporo Hospital	Sapporo, Hokkaido, Japan	Patient Recruitment, Enrollment, and Retention	Stomach Cancer Study Group:SCSG
Yuji	Akiyama		M.D., Ph.D.	Iwate Medical University	Shiwa-gun, Iwate, Japan	Patient Recruitment, Enrollment, and Retention	Stomach Cancer Study Group:SCSG
Yasuko	Murakawa		M.D., Ph.D.	Miyagi Cancer Center	Natrori, Miyagi, Japan	Patient Recruitment, Enrollment, and Retention	Stomach Cancer Study Group:SCSG
Akihito	Kawazoe		M.D., Ph.D.	National Cancer Center Hospital East	Kashiwa, Chiba, Japan	Patient Recruitment, Enrollment, and Retention	Stomach Cancer Study Group:SCSG
Chihiro	Kondoh		M.D.	Toranomon Hospital	Minato-ku, Tokyo, Japan	Patient Recruitment, Enrollment, and Retention	Stomach Cancer Study Group:SCSG
Hiroshi	Yabusaki		M.D., Ph.D.	Niigata Cancer Center Hospital	Niigata, Niigata, Japan	Patient Recruitment, Enrollment, and Retention	Stomach Cancer Study Group:SCSG
Kunihiro	Tsuji		M.D.	Ishikawa Prefectural Central Hospital	Kanazawa, Ishikwa, Japan	Patient Recruitment, Enrollment, and Retention	Stomach Cancer Study Group:SCSG
Atsuyuki	Maeda		M.D., Ph.D.	Ogaki Municipal Hospital	Ogaki, Gifu, Japan	Patient Recruitment, Enrollment, and Retention	Stomach Cancer Study Group:SCSG

*Indicates required information. Only first name, last name, and suffix will appear in PubMed.

***First Name and Middle Initial(s)**	***Last Name**	***Suffix (eg, Jr, III)**	Academic Degrees	Institution	Location (city, state/province, country)	Role or Contribution, eg, chair, principal investigator	Group (if more than 1 Group listed in the byline) and/or Subgroup (eg, Steering Committee)
Takushi	Yasuda		M.D.	Kindai University Hospital		Patient Recruitment, Enrollment, and Retention	Stomach Cancer Study Group:SCSG
Takuya	Hamakawa		M.D., Ph.D.	National Hospital Organization Osaka National Hospital	Osaka city, Osaka, Japan	Patient Recruitment, Enrollment, and Retention	Stomach Cancer Study Group:SCSG
Kazumasa	Fujitani		M.D., Ph.D.	Osaka General Medical Center	Osaka, Japan	Patient Recruitment, Enrollment, and Retention	Stomach Cancer Study Group:SCSG
Masahiro	Goto		M.D., Ph.D.	Osaka Medical and Pharmaceutical University	Takatsuki,Osaka,Japan	Patient Recruitment, Enrollment, and Retention	Stomach Cancer Study Group:SCSG
Ryouhei	Kawabata		M.D., Ph.D.	Osaka Rosai Hospital	Sakai, Osaka, Japan	Patient Recruitment, Enrollment, and Retention	Stomach Cancer Study Group:SCSG
Yoshihiro	Kakeji		M.D.	Kobe University Graduate School of Medicine		Patient Recruitment, Enrollment, and Retention	Stomach Cancer Study Group:SCSG
Takashi	Ohta		M.D., Ph.D.	Kansai Rosai Hospital	Amagasaki, Hyogo, Japan	Patient Recruitment, Enrollment, and Retention	Stomach Cancer Study Group:SCSG
Hisashi	Shinohara		M.D.	Hyogo College of Medicine		Patient Recruitment, Enrollment, and Retention	Stomach Cancer Study Group:SCSG
Masahiro	Tsuda		M.D., Ph.D.	Hyogo Cancer Center	Akashi, Hyogo, Japan	Patient Recruitment, Enrollment, and Retention	Stomach Cancer Study Group:SCSG
Hiroki	Fukunaga		M.D.	Itami City Hospital	Itami, Hyogo, Japan	Patient Recruitment, Enrollment, and Retention	Stomach Cancer Study Group:SCSG
Noriyuki	Hirahara		M.D., Ph.D.	Shimane University Faculty of Medicine	Izumo, Shimane, Japan	Patient Recruitment, Enrollment, and Retention	Stomach Cancer Study Group:SCSG
Kazuaki	Tanabe		M.D.	Hiroshima University Hospital		Patient Recruitment, Enrollment, and Retention	Stomach Cancer Study Group:SCSG
Satoshi	Oono		M.D.	Fukuyama City Hospital		Patient Recruitment, Enrollment, and Retention	Stomach Cancer Study Group:SCSG
Yasuhiro	Yuasa		M.D.	Tokushima Red Cross Hospital		Patient Recruitment, Enrollment, and Retention	Stomach Cancer Study Group:SCSG
Tsuyoshi	Etoh		M.D., Ph.D.	Oita University Hospital	Yufu, Oita, Japan	Patient Recruitment, Enrollment, and Retention	Stomach Cancer Study Group:SCSG
Masanobu	Takahashi		M.D., Ph.D.	Tohoku University Hospital	Sendai, Miyagi, Japan	Patient Recruitment, Enrollment, and Retention	Japan Esophageal Oncology Group:JEOG
Yusuke	Amanuma		M.D., Ph.D.	Chiba Cancer Center	Chiba, Chiba, Japan	Patient Recruitment, Enrollment, and Retention	Japan Esophageal Oncology Group:JEOG
Motoo	Nomura		M.D., Ph.D.	Kyoto University Hospital	Kyoto, Kyoto, Japan	Patient Recruitment, Enrollment, and Retention	Japan Esophageal Oncology Group:JEOG
Yuichiro	Doki		M.D.	Osaka University Graduate School of Medicine		Patient Recruitment, Enrollment, and Retention	Japan Esophageal Oncology Group:JEOG
Masahiro	Goto		M.D., Ph.D.	Osaka Medical and Pharmaceutical University	Takatsuki,Osaka,Japan	Patient Recruitment, Enrollment, and Retention	Japan Esophageal Oncology Group:JEOG
Yoshiaki	Nagatani		M.D., Ph.D.	Kobe University Graduate School of Medicine	Kobe, Hyogo, Japan	Patient Recruitment, Enrollment, and Retention	Japan Esophageal Oncology Group:JEOG

Supplemental Online Content: Nonauthor Collaborators

*Indicates required information. Only first name, last name, and suffix will appear in PubMed.

***First Name and Middle Initial(s)**	***Last Name**	***Suffix (eg, Jr, III)**	Academic Degrees	Institution	Location (city, state/province, country)	Role or Contribution, eg, chair, principal investigator	Group (if more than 1 Group listed in the byline) and/or Subgroup (eg, Steering Committee)
Masahiro	Tsuda		M.D., Ph.D.	Hyogo Cancer Center	Akashi, Hyogo, Japan	Patient Recruitment, Enrollment, and Retention	Japan Esophageal Oncology Group:JEOG
Hiroshi	Ariyama		M.D., Ph.D.	Kyushu University Hospital	Fukuoka, Fukuoka, Japan	Patient Recruitment, Enrollment, and Retention	Japan Esophageal Oncology Group:JEOG
