# Supplementary material for: Effectiveness of Etoposide and Cisplatin vs Irinotecan and Cisplatin Therapy for Patients With Advanced Neuroendocrine Carcinoma of the Digestive System: The TOPIC-NEC Phase 3 Randomized Clinical Trial
Source: JAMA Oncol. 2022 Aug 18;8(10):1447–55. doi: 10.1001/jamaoncol.2022.3395 (PMC9389440; doi:10.1001/jamaoncol.2022.3395)
Supplement: Supplement 5. — Data Sharing Statement [file jamaoncol-e223395-s005.pdf]

Data Sharing Statement

Morizane. Effectiveness of Etoposide and Cisplatin vs Irinotecan and Cisplatin Therapy for Patients With Advanced Neuroendocrine Carcinoma of the Digestive System. *JAMA Oncol*. Published August 18, 2022. doi:10.1001/jamaoncol.2022.3395

Data
Data available: Yes
Data types: Deidentified participant data, Data dictionary
How to access data: Individual participant data that underlie the results reported in this article, after deidentification will be shared if investigators whose proposed use of the data has been approved by the investigators from JCOG Hepatobiliary and Pancreatic Oncology Group, Stomach Cancer Study Group and Japan Esophageal Oncology Group identified for this purpose. Proposals should be directed to tokusaka@ncc.go.jp
When available: With publication

Supporting Documents
Document types: None

Additional Information
Who can access the data: researchers whose proposed use of the data
Types of analyses: Academic Use, Application for Approval
Mechanisms of data availability: With/without investigator support (it depends on case), After approval of a proposal, or with a signed data access agreement
